# Recent extreme heatwaves are no match for future events

**DOI:** 10.1038/s41467-026-76809-5

**Published:** 2026-11-05

**Authors:** Philipp Aglas-Leitner, Sarah E. Perkins-Kirkpatrick, Lukas Brunner, Sabine Undorf, Florentina Aglas-Leitner, Erich Fischer, Dáithí A. Stone

**Affiliations:** 1 Department of Physics, Freie Universität Berlin, Berlin, Germany; 2 Department of Mathematics and Computer Science, Freie Universität Berlin, Berlin, Germany; 3 Climate Change Research Centre, University of New South Wales, Sydney, NSW Australia; 4 Fenner School of Environment and Society, Australian National University, Canberra, ACT Australia; 5 Department for Climate Resilience, Potsdam Institute for Climate Impact Research, Potsdam, Germany; 6 ARC Centre of Excellence for Climate Extremes, Canberra, Australia; 7 ARC Centre of Excellence for the Weather of the 21st Century, Canberra, Australia; 8 Research Unit Sustainability and Climate Risk, Earth and Society Research Hub (ESRAH), University of Hamburg, Hamburg, Germany; 9 Department of Infectious Diseases, Respiratory Medicine, and Critical Care, Charité-Universitätsmedizin Berlin, Berlin, Germany; 10 Institute for Atmospheric and Climate Science, ETH Zurich, Zurich, Switzerland; 11 Earth Sciences New Zealand, Wellington, New Zealand

**Keywords:** Projection and prediction, Natural hazards

## Abstract

In recent years, record-breaking heatwaves have occurred over many regions of the Earth causing serious impacts to human and natural systems. Here we show that recently observed exceptional heatwaves are no match to potential future 21^st^ century events based on multiple metrics. For several observed heatwaves, this holds even under low- and medium-emissions scenarios. In a high- to very high-emissions future, many regions are projected to experience heatwaves that generate several times more cumulative heat and are many times longer (up to several months) than the observed heatwaves. Should the Nationally Determined Contributions under the Paris Agreement not be implemented, in most regions a concerningly large proportion of projected heatwaves exceeds all analyzed heatwave characteristics at once. Our results underscore the crucial need for substantial emissions reduction and have important implications for adaptation measures, as a substantial proportion of projected heatwaves is well outside the recent lived experience.

## Abstract (web summary)

This paper shows that current temperature and emission-reduction targets provide only limited protection against future heatwaves, which are found to surpass recent exceptional events across multiple regions of the world.

## Introduction

During recent decades, the world has seen a large number of exceptional heatwaves^1–4^, associated with severe impacts on human health^5–7^ and mortality^8–10^, ecosystems^11,12^, electricity availability and usage^13,14^, and with fire weather^15–17^. Such impacts may be avoided if their scale is anticipated^18^. It therefore seems beneficial to put projected future heatwaves in the context of recently observed heatwaves. This contextualization of future heatwaves may, in particular, help inform adequate adaptation, motivate mitigation measures, and facilitate communication of climate risks by helping people grasp the magnitude of projected future heatwaves^19–21^.

In this study, we assess projected heatwaves in the context of recent exceptional events. To cover a broad range of temperature thresholds, we analyzed heatwaves based on four separately applied definitions, where the daily maximum temperature (${T}_{\max }$) exceeds one of the four calendar-day-based temperature thresholds – 90^th^ (Tx90p), 95^th^ (Tx95p), 97.5^th^ (Tx97.5p), and 99^th^ percentile (Tx99p) – for at least three consecutive days^22^. Taking into account differing statistical robustness due to different sample sizes originating from temperature threshold choice, we mainly focus on Tx90p-based results in the text and discuss results for higher percentiles where appropriate. Hence, if not explicitly mentioned, results presented are based on Tx90p. Future heatwaves are assessed based on global climate model simulations from the ScenarioMIP project of the Coupled Model Intercomparison Project Phase 6 (CMIP6)^23^ for a range of Shared Socioeconomic Pathways (SSPs)^24^. We additionally employ a CMIP5 ensemble^25^ to determine whether the heatwave projections differ between the two CMIP generations, and the CanESM5 and ACCESS-ESM1.5 single model initial-condition large ensembles (SMILEs)^26,27^ to assess the role of internal climate variability. This study provides a pragmatic approach in assessing heatwave projections by placing projected future heatwaves in the context of recent extreme events observed over various regions of the earth, and assessing multiple combined and individual heatwave characteristics across several emission scenarios and global warming levels. This approach provides more detailed projections of individual events than previously applied methods^28–33^, and establishes a consistent in-depth framework across regions and time periods.

Here we show that recently observed exceptional heatwaves are outmatched by a substantial proportion of projected events and that current political temperature- and emissions-reduction-targets only provide limited protection against heatwaves surpassing exceptional events in the recent lived experience.

## Results

We analyzed 22 observed terrestrial heatwaves (2010-2023) and projected future heatwaves (2020-2100) in their respective regions and focus here on a subset of nine heatwaves observed between 2015 and 2023 (for entire set of results, see Supplements). The heatwave characteristics analyzed in this study include duration^22^, cumulative heat^34^ (accumulated temperature anomaly above respective heatwave threshold), median and maximum event severity^35,36^ (extreme heat index relative to difference between climatology and respective heatwave threshold), and median and maximum spatial extent of locally heat-affected area (heatwave area). To facilitate contextualization of projected events, we ranked the characteristics of the observed heatwaves relative to the respective reference data set, i.e. Berkeley Earth and ERA5 (Table 2, Methods). We furthermore, analyze exceedance likelihoods and return periods of the six heatwave characteristics for each region.

### Combined heatwave characteristics of individual heatwaves based on CMIP scenarios

Our results show that a considerable percentage of future heatwaves are projected to become longer, larger, more severe, and to generate more cumulative heat than most exceptionally high observed reference heatwave characteristics under low- (SSP1-2.6, RCP2.6), intermediate (SSP2-4.5, RCP4.5), high- (SSP3-7.0), and very high-emissions scenarios (SSP5-8.5, RCP8.5) in CMIP6 and CMIP5 multi-model ensembles (MME) (Figs. 1–2, and Supplementary Figs. 19–40). In many cases during and particularly towards the end of the 21^st^ century, recent observed events are outmatched by a substantial margin, even under ambitious emissions reduction scenarios (SSP1-2.6, RCP2.6) (Fig. 1 & Supplementary Figs. 41–82). These trends can also be observed in our Tx95p-, Tx97.5p-, and Tx99p-based analyses in CMIP6 (Supplementary Figs. 83–202).

**Fig. 1:**
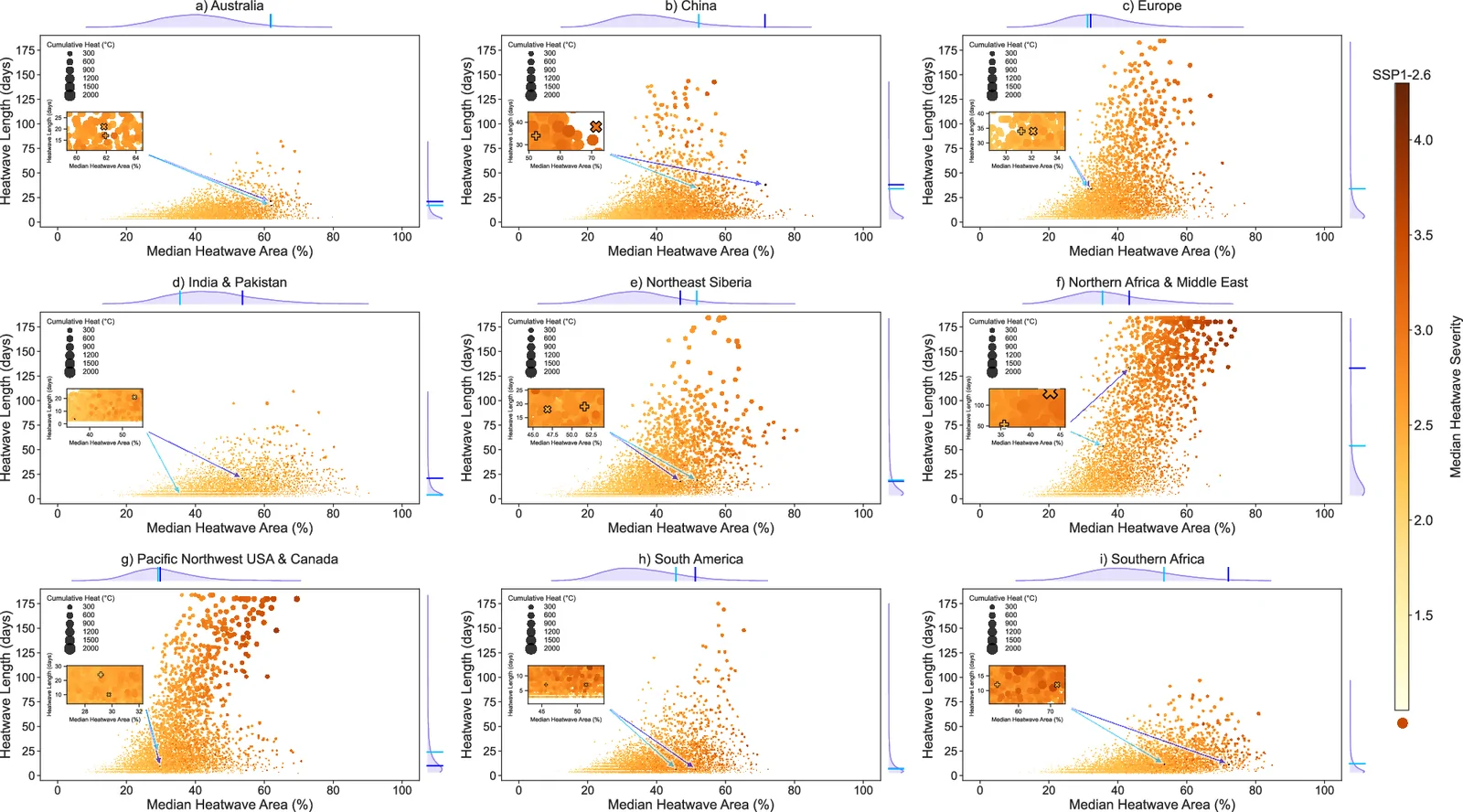
Recently observed heatwaves and future heatwave projections according to SSP1-2.6. Each panel shows regional CMIP6 SSP1-2.6 multi-model ensemble (MME) projections of four combined heatwave characteristics for each event in the ensemble between 2020 and 2100. Each dot represents an individual heatwave in the projections, characterized by median heatwave area (*x*-axis), i.e. latitude-weighted percentage of grid boxes affected by extreme heat, heatwave length (*y*-axis) in days, median heatwave severity (color), and cumulative heat (dot size). The characteristics of the observed event according to the Berkeley Earth observational data (x symbol) and the ERA5 reanalysis data (+ symbol), respectively, are pointed to by an arrow, and a Figure inset shows a zoom-in on this Figure section. The Figure inset shows the observed heatwave in the two reference data sets and projections in the indicated section of the respective sub-plot. It facilitates readability of color and size of the reference values and comparison to each other and the neighboring projections. Note that the zoom-in causes the dots to overlap more in the inset than in the main sub-plot. The univariate kernel density estimates along the *x*-axis (heatwave area) and *y*-axis (duration) are on the top and right side of the subplots, respectively, with vertical lines in dark blue (Berkeley Earth) and light blue (ERA5) indicating the observed event. For additional contextualization relative to the models' historical period, see Supplementary Figs. 1–18.

**Fig. 2:**
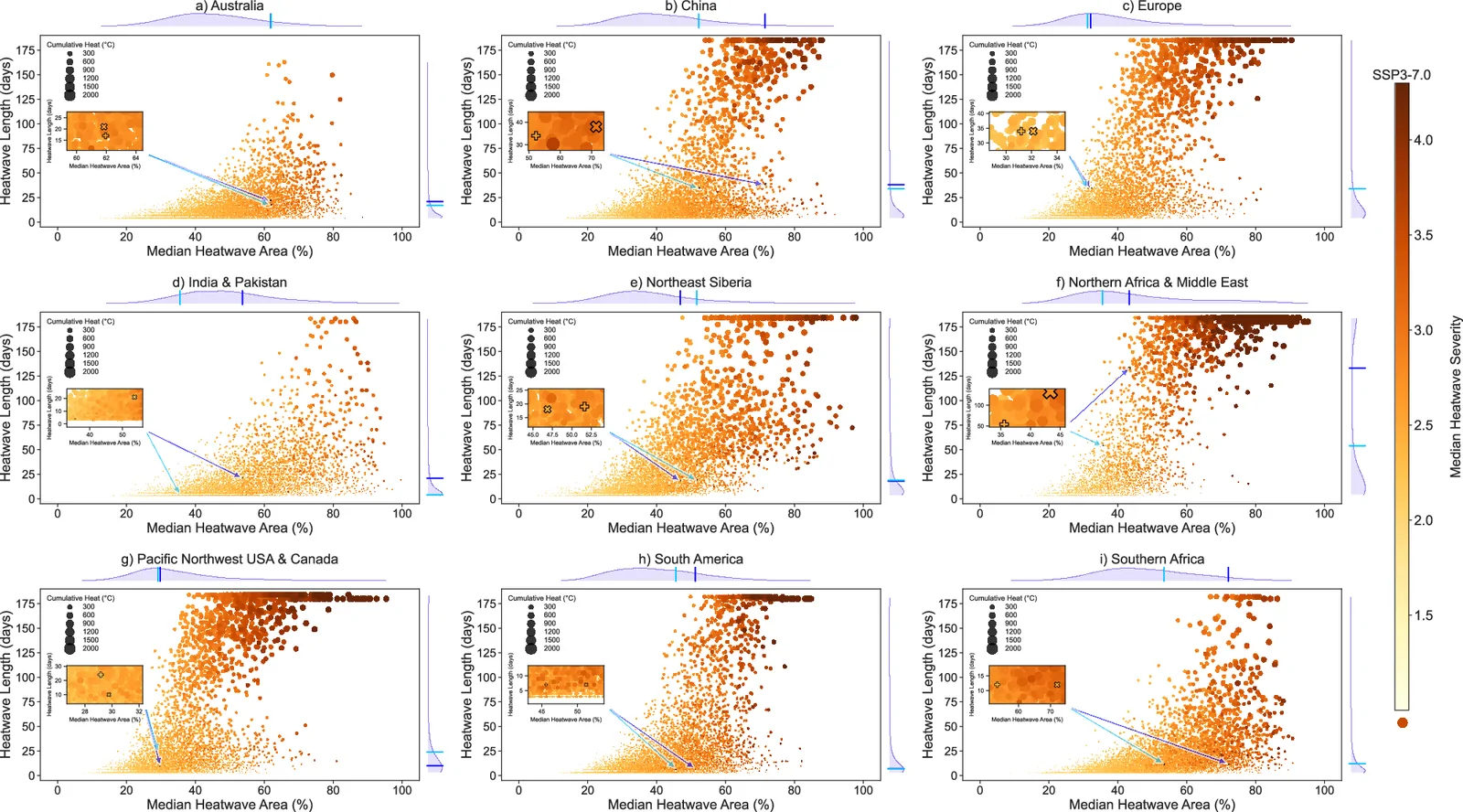
Recently observed heatwaves and future heatwave projections according to SSP3-7.0. Each panel shows regional CMIP6 SSP3-7.0 multi-model ensemble (MME) projections of four combined heatwave characteristics for each event in the ensemble between 2020 and 2100. Each dot represents an individual heatwave in the projections, characterized by median heatwave area (*x*-axis), i.e. latitude-weighted percentage of grid boxes affected by extreme heat, heatwave length (*y*-axis) in days, median heatwave severity (color), and cumulative heat (dot size). The characteristics of the observed event according to the Berkeley Earth observational data (x symbol) and the ERA5 reanalysis data (+ symbol), respectively, are pointed to by an arrow, and a Figure inset shows a zoom-in on this Figure section. The Figure inset shows the observed heatwave in the two reference data sets and projections in the indicated section of the respective sub-plot. It facilitates readability of color and size of the reference values and comparison to each other and the neighboring projections. Note that the zoom-in causes the dots to overlap more in the inset than in the main sub-plot. The univariate kernel density estimates along the *x*-axis (heatwave area) and *y*-axis (duration) are on the top and right side of the subplots, respectively, with vertical lines in dark blue (Berkeley Earth) and light blue (ERA5) indicating the observed event. For additional contextualization relative to the models' historical period, see Supplementary Figs. 1–18.

Hereafter, we use the term exceptional as a statistical descriptor (definition in Methods and Table 2). Hence, the term does not constitute a qualitative assessment of a heatwave characteristic. We contextualize heatwave projections relative to two independent reference data sets (i.e. Berkeley Earth and ERA5). However, for the sake of readability, the exceedance percentage results discussed below are relative to Berkeley Earth, if not explicitly stated otherwise (see Methods, and Supplements for ERA5-based results). Regions with reference heatwaves reaching exceptionally high median severity values (Northern Africa & Middle East, South America, and Southern Africa; see Table 2 and methods), have a projected exceedance likelihood of approximately 1-in-8 to 1-in-6 heatwaves under SSP1-2.6 (Supplementary Data 9), which increases to approximately 27% in South America and Southern Africa and 36% in Northern Africa & Middle East under SSP3-7.0 (Supplementary Data 10). Australia and China, which both experienced peak-median-severity heatwaves in 2019 and 2022 (Table 2), respectively, are projected to exceed the median severity of the observed event in  < 3% and  < 0.5% of future heatwaves under SSP1-2.6 (Supplementary Data 9). These relatively low exceedance percentages highlight the exceptional median severity of these events. However, under high-emissions conditions (SSP3-7.0), the chances of exceeding the median severity of the two observed heatwaves increases to approximately 12% in Australia and 3% in China (Supplementary Data 10). This means that the chances of surpassing the reference heatwave are nearly 5-times and 8-times as high, respectively, under high-emissions conditions compared to a low-emissions future. Under SSP1-2.6, most regions (China, Europe, Northern Africa & Middle East, Pacific Northwestern USA & Canada, and South America) show a  > 10% chance to reach a median heatwave severity of at least 2 (Supplementary Data 11), which means that the average heatwave-day reaches temperatures where the difference to the climatological mean is twice as large as the difference between climatology and extreme heat threshold (Tx90p). Under SSP3-7.0, these chances more than double. Furthermore, under these conditions Australia and India & Pakistan show projected chances of  > 10% to reach median heatwave severity values of  > 2 (Supplementary Data 12). Heatwaves with exceptionally high median severity relative to Tx99p occurred in Australia, China, and Northern Africa & Middle East (Supplementary Data 8). Whereas the exceedance percentage in China is limited to  < 2% in a low-emissions future (SSP1-2.6), the chances are approximately 1-in-5 heatwaves in Australia and Northern Africa & Middle East (Supplementary Data 13). Under SSP3-7.0, these chances increase substantially: approximately 8% of projected heatwaves in China and close to 40% in Australia and Northern Africa & Middle East exceed the exceptionally high median severity of the observed reference heatwave relative to the respective region’s 99^th^ percentile (Supplementary Data 14).

Furthermore, our analysis of the spatial extent of heatwaves shows that from 2020 to 2100 between  < 1% and 2% of heatwaves in Australia, China, and Southern Africa exceed the exceptionally large median spatial extent (62–72% of the regions’ area) of the respective reference heatwave under SSP1-2.6 (Supplementary Data 15). In South America and Northern Africa & Middle East (43% and 51% of the region’s area affected, respectively) this is the case in 6% and 23% of heatwaves (Supplementary Data 15). The exceedance likelihood is substantially higher under high-emissions conditions (SSP3-7.0): Australia and Southern Africa have a projected exceedance likelihood of 1-in-10 and 1-in-20, respectively, whereas in South America and Northern Africa & Middle East 1-in-5 and 2-in-5 heatwaves are projected to exceed the exceptional median spatial extent (Supplementary Data 16). For China, the chances of exceeding the peak-spatial-extent are still  < 2% under SSP3-7.0 (Supplementary Data 16), again highlighting the exceptionality of the event. This, nevertheless, represents a  > 14-times increase in exceedance likelihood compared to low-emissions conditions. Under low-emissions conditions (SSP1-2.6), in five of the nine regions (Australia, China, India & Pakistan, Northern Africa & Middle East, and Southern Africa) at least 1-in-10 heatwaves reaches a median heatwave area of  > 50% (Supplementary Data 17). This means that those heatwaves on average cover more than half of the region with extreme local heat. These chances more than double under SSP3-7.0 (Supplementary Data 18). For four of those regions, the chances even more than triple (Supplementary Data 18).

Even under a strong mitigation scenario (SSP1-2.6), projections show heatwave lengths up to several months, with five out of nine regions (Europe, Northeast Siberia, Northern Africa & Middle East, Pacific Northwestern USA & Canada, and South America) showing heatwaves approaching or even hitting the theoretical maximum length of six consecutive months (see Methods). Moreover, exceptionally long observed heatwaves in Australia, China, Europe, India & Pakistan, Northeast Siberia, and Northern Africa & Middle East (Table 2) are outmatched by projected events, even when strong emissions mitigation occurs. While exceedance percentages in Australia and China are limited to  < 4%, between 6% and 11% of projected heatwaves in Europe, India & Pakistan, and Northern Africa & Middle East and approximately 1-in-4 projected heatwaves in Northeast Siberia exceed the exceptionally long reference events (Supplementary Data 19). These proportions increase substantially under SSP3-7.0 (Supplementary Data 20). In Australia and China, this results in an exceedance likelihood of approximately 1-in-10. Furthermore, close to 1-in-5 heatwaves in Europe and India & Pakistan, 1-in-4 in Northern Africa & Middle East and 1-in-3 in Northeast Siberia are projected to last longer than the exceptionally long reference events (Supplementary Data 20). In Australia, China, and Northern Africa & Middle East, this would mean chances of exceeding the reference events ~3- to 4-times as high as under SSP1-2.6. Under high-emission scenarios (Fig. 2), Australia is the only region with a possible maximum heatwave length of less than six months (Australia only reaches the six-month limit under CMIP6 SSP5-8.5 and CMIP5 RCP8.5, see Supplementary Figs. 22 & 37). This means that without significant emissions reduction, the other regions might experience sustained heatwave conditions for an entire half of the year. Albeit with a markedly reduced likelihood, this holds true even for Tx95p, Tx97.5p, and, with the exception of India & Pakistan, for Tx99p (Supplementary Figs. 110–112).

Observed heatwaves that generated exceptionally large amounts of cumulative heat show projected exceedance likelihoods of 9–13% in Europe, India & Pakistan, and Northern Africa & Middle East under SSP1-2.6 (Supplementary Data 21). In South America and Southern Africa the respective cumulative heat is exceeded by ~1-in-5 and 1-in-4 projected heatwaves, respectively. This is the case in only 1-in-50 heatwaves in Australia and China (Supplementary Data 21), highlighting the record-nature of these two events. These chances, however, substantially increase under high emissions (SSP3-7.0), where approximately 1-in-8 and 1-in-13 projected heatwaves in Australia and China, respectively, generate more cumulative heat than the observed event (Supplementary Data 22). In Europe, India & Pakistan, and Northern Africa & Middle East, projected heatwaves show chances of 20–30% to exceed the exceptionally high cumulative heat. In South America and Southern Africa, this is the case in 30–40% of projected heatwaves (Supplementary Data 22). Except for Australia and China, most of the regions that experienced heatwaves with exceptionally high cumulative heat have a chance of approximately 1-in-10 to 1-in-5 heatwaves generating more than twice the respective reference event’s cumulative heat under SSP1-2.6 (Supplementary Data 23). Under SSP3-7.0, this increases to approximately 1-in-8 to 1-in-4 projected heatwaves in these regions, whereas Australia and China show a chance of approximately 1-in-5 projected heatwaves (Supplementary Data 24). Moreover, under these conditions most of these regions have a chance of approximately 1-in-10 to 1-in-5 heatwaves generating more than five-times the reference event’s cumulative heat (Supplementary Data 24).

Due to a greater and faster increase in global average temperature, exceptionally long, severe, and spatially extensive heatwaves and events that generate large amounts of cumulative heat are more likely to be projected by climate models with high climate sensitivity. However, we find that the occurrence of such exceptionally high values of projected heatwave characteristics are not limited to this sub-set of models^37^ and the projected spread of each characteristic is comparable across most models in MME-CMIP6 (Supplementary Figs. 203–290). A more detailed analysis, however, would be beyond the scope of this study.

Each region shows a distinct pattern in coupling between heatwave characteristics. In particular in China, Europe, Northern Africa & Middle East and Pacific Northwestern USA & Canada, there is a tendency of events with very large median heatwave area to persist for several months (Fig. 2). Regions like Australia, India & Pakistan, Northeast Siberia, Southern Africa, and South America, show a greater spread in event duration among spatially large heatwaves (Fig. 2). Across all regions, spatially small heatwaves (median and maximum area) tend to be on the very low end of the duration- and severity-distributions, whereas short-lived events tend to have a considerable range of severity and spatial extent (Fig. 2 & Supplementary Fig. 25). This implies that short-lived heatwaves are not to be underestimated, as they, for example, can still exceed the spatial extent of the observed reference events in many regions by a substantial margin (Fig. 2 & Supplementary Fig. 25). Results based on Tx95p, Tx97.5p, and Tx99p, show the same tendencies regarding the coupling of heatwave characteristics (Supplementary Figs. 92–94 & 107–109).

The seemingly large discrepancies between some heatwave characteristics in our two reference data sets in some regions, for instance the heatwave length in Northern Africa & Middle East, can in part be explained by relatively small differences between the data sets themselves. However, while this further highlights the necessity in using multiple reference products when assessing extreme events, the overall broad implications of our results are not critically impacted by these discrepancies (see Methods).

For most regions, our assessment of combined heatwave characteristics does not differ much between CMIP5 and CMIP6. As the ensemble spread, the percentage of heatwaves exceeding the observed event, and the coupling patterns of the combined characteristics are similar in either result, there is no clear evidence that one MME generation would provide systematically more or less reliable projections over the other (see Annex).

### Heatwave characteristics relative to global warming

In addition to model-scenario-based analysis of the regional heatwave characteristics, we analyze the same characteristics for global warming levels (GWLs) from 0°C to 4.5°C (see Methods). Here, we focus on maximum severity, maximum heatwave area, and an analysis of all six heatwave characteristics combined, while results for all 22 regions and the remaining heatwave characteristics based on CMIP5- and CMIP6-MME are presented in Supplementary Figs. 291–302. Among other things, GWL-based analysis quantifies changes in heatwave characteristics and their respective exceedance percentages per degree of global warming and thereby directly maps on political global temperature targets. This facilitates communication^38^ of the projected changes and the exceedance likelihood. Scenario-based analysis, on the other hand, has the advantage that it retains socio-economic context and scenario-specific emissions pathways, which is particularly relevant for scenarios with a peak warming period and a subsequent reduction of global average temperatures^39^, that can be relevant for political decision-making. As each analysis type has a different focus and different advantages, combining the two approaches in this study provides deeper understanding of how projected heatwaves compare to recently observed extreme events under different circumstances, and increases the applicability of our results to risk assessments and adaptation planning compared to only using one of the two approaches.

**Fig. 3:**
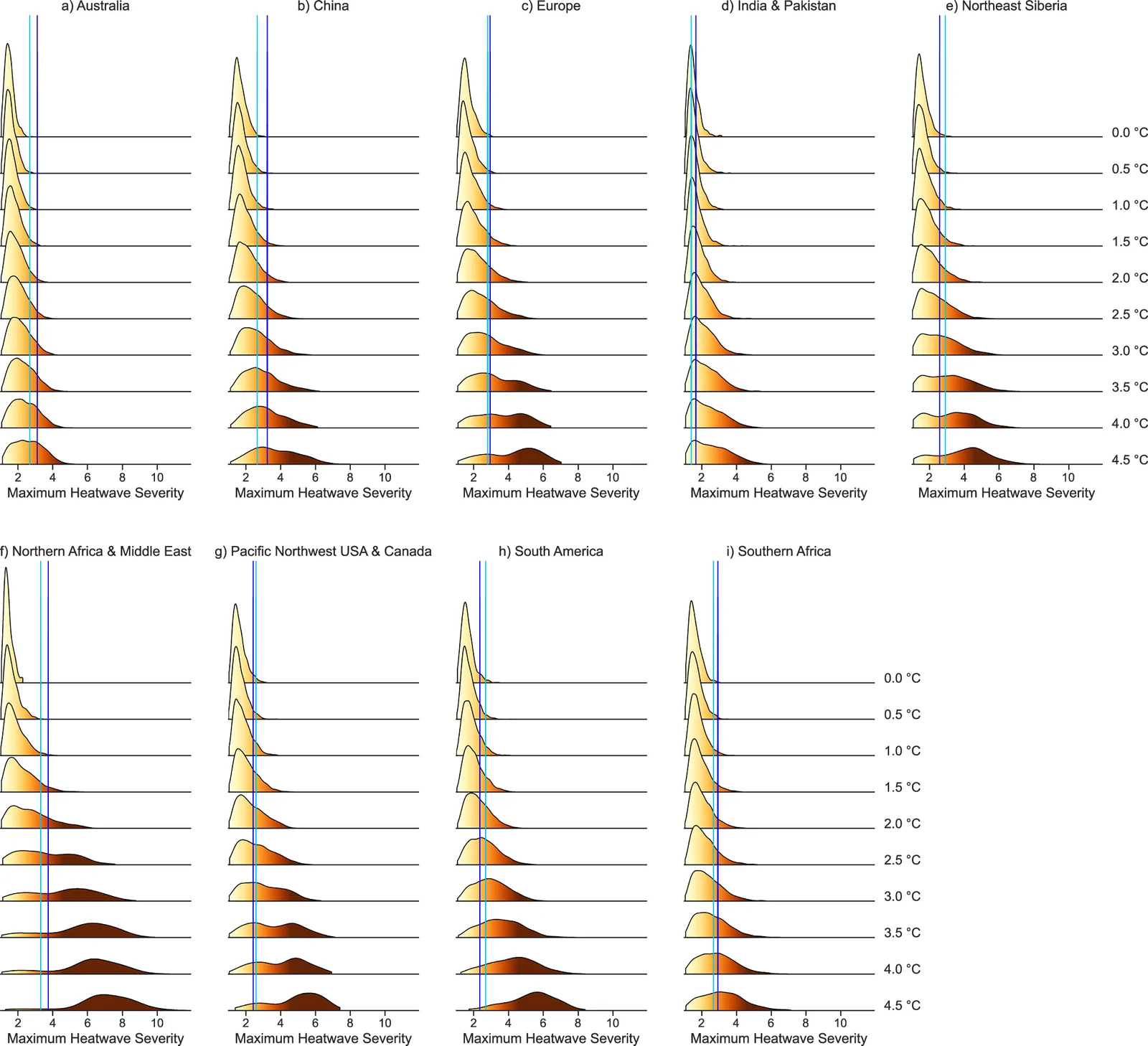
Distributions of Maximum Severity across Global Warming Levels (GWLs). Kernel density estimates of maximum heatwave severity for different global warming levels (GWLs) in CMIP6-MME. Area under each curve is normalized to 1. Maximum heatwave severity of the observed event is indicated by vertical lines in dark blue (Berkeley Earth) and light blue (ERA5).

Distributions of regional maximum heatwave severity at different global warming levels (GWLs) show substantial changes under enhanced climate change for all regions (Fig. 3, Supplementary Figs. 292 & 298). The exceptionally high maximum severity in Australia, China, Europe, Northern Africa & Middle East, and Southern Africa is nearly impossible without global warming (exceedance chances of  < 0.3%) and also extremely unlikely under limited global warming (up to 0.5°C) with exceedance likelihoods of  < 0.8% (Supplementary Data 25). Other exceptionally severe heatwaves in Northeast Siberia and Pacific Northwestern USA & Canada also show limited chances (< 5%) of exceedance under limited global warming (Supplementary Data 25). In the case of the 2021-heatwave in Pacific Northwestern USA & Canada, which is among the approximately top 3.5% of heatwaves in the Berkeley Earth observational record and among the top 2.5% in the ERA5 reanalysis data for regional (i.e. area-weighted-mean ${T}_{\max }$) severity (Supplementary Data 5), the most extreme absolute temperatures recorded were very localized to a small geographical region^40,41^. Based on the spatio-temporal nature of the heatwave (see Table 1, Supplementary Figs. 303–304, and discussion), we, however, analyzed a significantly larger geographical region. Hence, the limited re-occurrence likelihood of maximum severity discussed above highlights the exceptional nature of the event, which shows that it was not only exceptional at the very localized level but also for a much larger geographical region (Supplementary Figs. 303–304).

**Table 1:** Regional Coordinates of Observed Reference Heatwaves

	Region	Regional Coordinates
a)	Australia	112.5E–153.5E, 43.5S–10.5S
b)	China	89.5E–122.5E, 20.5N–40.5N
c)	Europe	10.5W–39.5E, 34.5N–71.5N
d)	India & Pakistan	66.5E–88.5E, 6.5N–32.5N
e)	Northeast Siberia	80.5E–190.5E, 59.5N–80.5N
f)	Northern Africa & Middle East	17.5W–68.5E, 12.5N–36.5N
g)	Pacific Northwest USA & Canada	140.5W–85.5W, 31.5N–73.5N
h)	South America	78.5W–50.5W, 55.5S–5.5S
i)	Southern Africa	14.5E–35.5E, 34.5S–14.5S

Regional boundaries were determined by the spatio-temporal evolution of each observed heatwave, and consequently used for model data analysis. For more information on reference heatwave selection and region definition, see Methods.

Increasing temperatures to GWL 2.0°C, substantially increases chances of exceeding maximum severity projected to be nearly impossible without global warming (Australia, China, Europe, Northern Africa & Middle East, and Southern Africa). In China and Southern Africa, chances of reaching higher heatwave severity are approximately 6–8%, whereas the chances in Europe and Northern Africa & Middle East increase to nearly 1-in-5 (Supplementary Data 25). Only Australia is limited to a chance of  < 2% under these conditions (Supplementary Data 25), which highlights the unprecedented nature of the 2019-heatwave in Australia. For GWLs beyond 2.0°C, distributions begin to flatten out, with maximum severity of seven of the nine regions even reaching values of 6.0 or higher.

Transitioning from a warming level of 2.5°C to 3.0°C, the maximum severity in both China and Southern Africa increases from approximately 13% (GWL 2.5°C) to 22% (GWL 3.0°C). In Europe and Northeast Siberia the exceedance percentage increases from 27% and 33% (GWL 2.5°C) to 37% and 47% (GWL 3.0°C), respectively (Supplementary Data 25). Moreover, the maximum severity of the reference heatwaves in Northern Africa & Middle East, Pacific Northwestern USA & Canada, and South America is exceeded by approximately two to three fifths (GWL 2.5°C) and 63–71% (GWL 3.0°C) (Supplementary Data 25). Compared to other regions, chances of exceeding the maximum severity of the 2019 Australian heatwave, with the highest maximum severity in the region’s observational period (Table 2), are limited to approximately 1-in-20 to 1-in-10 heatwaves under these global warming conditions (Supplementary Data 25), highlighting the exceptionally high temperatures of this observed heatwave. Nevertheless, chances of exceeding the observed maximum severity are approximately 40- (GWL 2.5°C) to 80-times (GWL 3.0°C) as high as under GWL 1.0°C (Supplementary Data 25). Except for Australia, the selected regions show an average increase in exceedance percentage of 5–12 percentage points per 0.5°C of global warming for maximum severity. Most of these regions are projected to see their strongest shifts in the temperature interval between GWLs 2.0°C and 3.5°C (Supplementary Data 26–27).

**Table 2:** Heatwave characteristics of observed reference heatwaves based on calendar-day 90^th^ percentile of daily maximum temperature (Tx90p)

	Region	Start Date	End Date	Reference Dataset	Median Heatwave Severity	Maximum Heatwave Severity	Median Heatwave Area (%)	Maximum Heatwave Area (%)	Cumulative Heat (°C)	Heatwave Length (days)
a)	Australia	2019-12-14	2020-01-03	Berkeley Earth	2.08‘+**	3.10‘+**	61.85‘+	84.78‘+**	26.23‘+**	21‘+**
b)	China	2022-07-21	2022-08-27	Berkeley Earth	2.65‘+**	3.23‘+	71.43‘+**	89.45‘+	73.87‘+**	38‘+*
c)	Europe	2018-07-10	2018-08-12	Berkeley Earth	1.89	2.95‘+*	32.13	60.49‘	37.39‘+*	34‘+
d)	India & Pakistan	2019-05-27	2019-06-16	Berkeley Earth	1.32	1.68	53.68	73.61	12.94‘+	21+
e)	Northeast Siberia	2020-06-14	2020-07-01	Berkeley Earth	1.80	2.61+	46.86	72.90‘+	31.40	18‘+
f)	Northern Africa & Middle East	2023-06-21	2023-10-30	Berkeley Earth	2.23‘+	3.74‘+**	43.33‘+	74.12‘+**	120.17‘+**	133‘+**
g)	Pacific Northwest USA & Canada	2021-06-25	2021-07-04	Berkeley Earth	1.71	2.42	29.77	43.57	9.79	10
h)	South America	2022-11-24	2022-11-30	Berkeley Earth	2.04‘+	2.35‘+	51.21‘+	62.85	7.97‘	7
i)	Southern Africa	2015-12-29	2016-01-09	Berkeley Earth	1.90‘	2.95‘+**	72.09‘+	94.59‘+	18.73‘	12
a)	Australia	2019-12-14	2019-12-30	ERA5	2.10‘+**	2.67‘+**	61.96‘+**	81.39‘+**	23.37‘+**	17‘+
b)	China	2022-07-24	2022-08-26	ERA5	2.08‘+	2.65‘+	52.25‘+	66.58‘+	41.50‘+**	34‘+**
c)	Europe	2018-07-10	2018-08-12	ERA5	1.83	2.81‘+	31.19	56.60‘	32.51‘+*	34‘+
d)	India & Pakistan	2019-06-09	2019-06-12	ERA5	1.33	1.42	35.51	37.18	1.29	4
e)	Northeast Siberia	2020-06-13	2020-07-01	ERA5	2.09	2.93‘+	51.63‘+	71.50‘+	43.59‘+	19
f)	Northern Africa & Middle East	2023-08-18	2023-10-30	ERA5	1.96‘+	3.30‘+	35.56	60.58‘+	42.61‘+*	54‘+
g)	Pacific Northwest USA & Canada	2021-06-22	2021-07-15	ERA5	1.45	2.59‘+	29.17	42.18	18.60‘+	24‘+
h)	South America	2022-11-24	2022-11-30	ERA5	2.10‘+	2.69‘	45.59	54.54	6.27	7
i)	Southern Africa	2015-12-31	2016-01-11	ERA5	1.74	2.69‘+**	53.40	79.92‘+	18.15‘	12

Values based on Berkeley Earth (top) and ERA5 (bottom) reference data ^56,58^ listing for one observed heatwave between 2015 and 2023 in each region: start and end date, and six heatwave metrics, namely median/maximum heatwave severity, median/maximum heatwave area (i.e., latitude-weighted percentage of grid boxes affected by extreme heat), cumulative heat, and heatwave length in days. Symbols indicating characteristics among the top 3.0% from 1961 to date of reference heatwave (‘), top 3.0% in entire reference dataset from 1961 to 2023 (+), peak value from 1961 to date of reference heatwave (*) or peak value in entire reference dataset from 1961 to 2023 (**). For more information on reference heatwave selection and region definition, definitions of heatwave metrics, and discussion on difference between Berkeley Earth and ERA5, see Methods. For information on all 22 observed terrestrial heatwaves, see Supplementary Data 1–5, and for information on calendar-day 95^th^- (Tx95p), 97.5^th^- (Tx97.5p), and 99^th^- (Tx99p) percentile-based characteristics see Supplementary Data 6–8, respectively.

Should global average temperatures rise beyond 3.0°C, 13–29% of projected heatwaves in Australia exceed the peak-maximum-severity of the 2019-heatwave under GWLs 3.5–4.5°C (Supplementary Data 25). Under the same conditions, approximately 1-in-3 to close to 60% of heatwaves in China and Southern Africa exceed the respective observed maximum severity (Supplementary Data 25). Furthermore, between approximately half and 75% of heatwaves in Europe and Northeast Siberia and roughly 80–98% in Northern Africa & Middle East and South America surpass the maximum severity of the respective reference events (Supplementary Data 25). Results indicate that in the latter two regions there is a chance of approximately 1-in-20 that a projected heatwave exceeds the observed maximum severity on every single day of the event under GWL 4.5°C (Supplementary Data 28). Moreover, under GWL 3.5°C Australia and India & Pakistan have approximately a chance of 1-in-6 heatwaves to reach a maximum severity of 3 (Supplementary Data 29). In China, Europe, Northeast Siberia, and Southern Africa this is the case for approximately 1-in-3 to 1-in-2, in Pacific Northwestern USA & Canada and South America for approximately two thirds and in Northern Africa & Middle East in 85% of projected heatwaves (Supplementary Data 29).

**Fig. 4:**
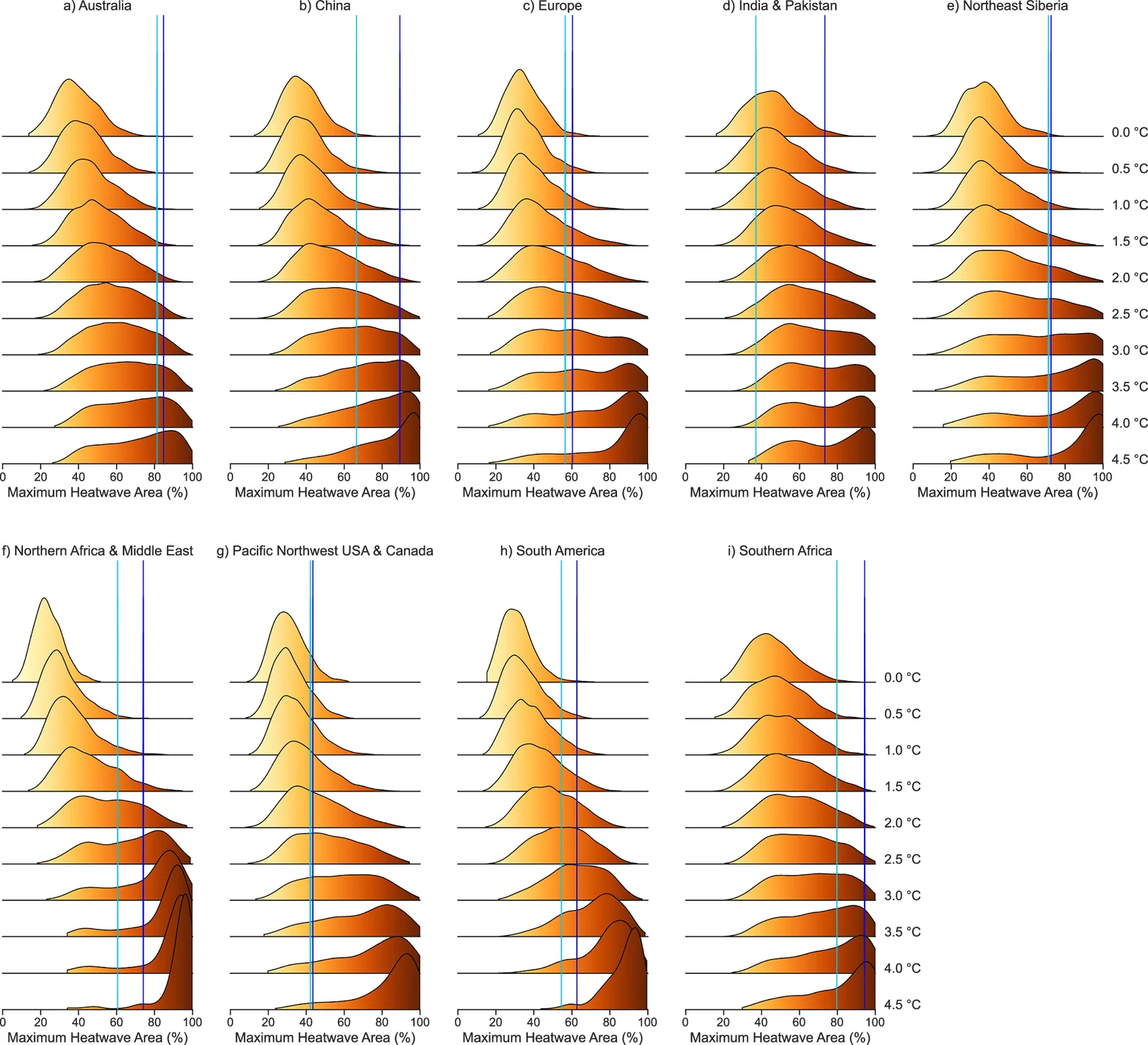
Distributions of Maximum Heatwave Area across Global Warming Levels (GWLs). Kernel density estimates of maximum heatwave area for different global warming levels (GWLs) in CMIP6-MME. Area under each curve is normalized to 1. Maximum heatwave area of the observed event is indicated by vertical lines in dark blue (Berkeley Earth) and light blue (ERA5).

Similar to heatwave severity, there is a substantial GWL-dependent shift in the percentage of heatwave-affected area (Fig. 4, Supplementary Figs. 291 & 297). Without global warming or under limited warming (up to 0.5°C), for five of the six reference heatwaves with exceptionally high or peak-maximum-affected-area (covering up to 72–95% of the respective region’s area, see Table 2) only 0–1% of projected heatwaves reach the observed maximum area (Fig. 4 & Supplementary Data 30), rendering such large affected areas nearly impossible without stronger global warming. In four of these regions (Australia, China, Northern Africa & Middle East, and Southern Africa) this continues to higher global temperatures (GWLs 1.0–2.0°C) (Fig. 4 & Supplementary Data 30), highlighting the exceptionality of these heatwaves. Of the six reference heatwaves with exceptionally high or peak-maximum-affected-area, only Europe, where the reference event covered up to 60% of the region’s area, is projected a slightly higher exceedance likelihood of 1-2.5% (GWLs 0–0.5°C) (Supplementary Data 30). GWL 2.0°C would increase the chances of exceeding the maximum affected area in Northeast Siberia and Northern Africa & Middle East to approximately 1-in-7 heatwaves and to 1-in-4 heatwaves in Europe (Supplementary Data 30). Under GWL 2.5–3.0°C, three of these regions show particularly strong shifts in exceedance percentage: While in Europe and Northeast Siberia the exceedance percentage increases by 11–15 percentage points per 0.5°C, the exceedance likelihood in Northern Africa & Middle East increases by approximately 25 percentage points (Supplementary Data 31–32). Under GWL 4.0°C, our Tx99p-based analysis shows that all nine regions display a considerable proportion of projected heatwaves affecting more than two thirds of the region with local extreme heat for at least one day (Supplementary Data 33). This means that between approximately 1-in-10 and 1-in-6 projected heatwaves in Australia, China, Europe, and South America affect more than two thirds of the region’s area with extreme heat beyond the local 99^th^ temperature percentile (Supplementary Data 33). In India & Pakistan, Northeast Siberia, and Southern Africa this is the case in between 1-in-5 and 1-in-3 projected heatwaves (Supplementary Data 33).

For global warming levels beyond 3.0°C, several regions affected by observed heatwaves with peak and/or exceptionally high heatwave characteristics show a considerable number of projected events exceeding all six characteristics at once (Supplementary Data 34). In Australia, China, and Southern Africa, for instance, between 1-in-20 and 1-in-5 projected heatwaves under GWL 3.5–4.5°C exceed all six heatwave characteristics at once (Supplementary Data 34). This includes the 2019-heatwave in Australia which has five peak-characteristics and an exceptionally high median heatwave area (Supplementary Data 34). Furthermore, 36–64% of projected heatwaves in Europe and 75–90% in Northern Africa & Middle East exceed all six heatwave characteristics (Supplementary Data 34). In other words, in Northern Africa & Middle East there is only a relatively small proportion of projected events that does not exceed all heatwave characteristics, including four peak-characteristics, a median heatwave area in the top 2.5% and a median severity in the top 1% of the observational data (Supplementary Data 34).

### Historical and Future return periods of observed heatwaves

**Fig. 5:**
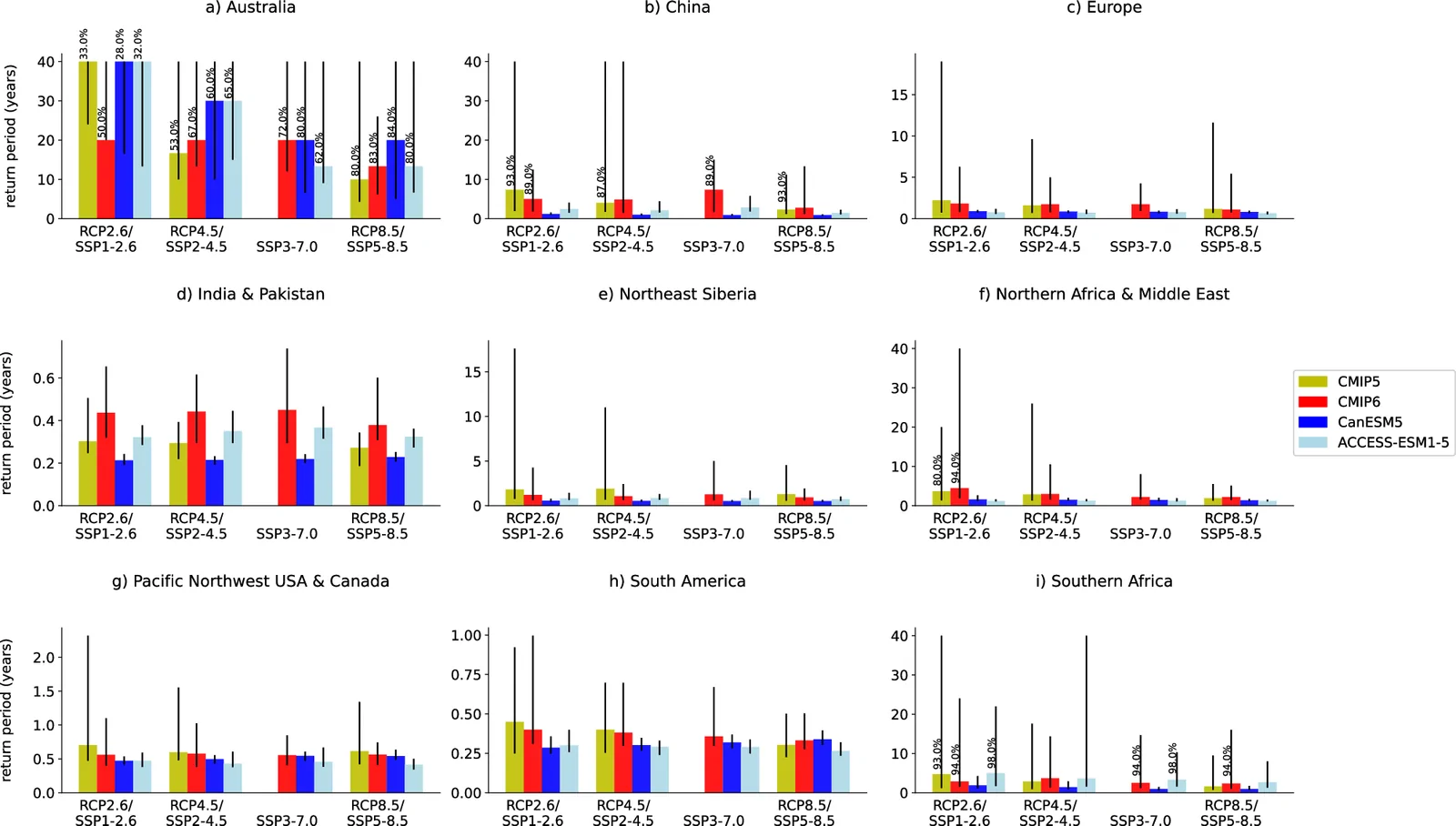
Return time (in years) between 2015 and 2054 for maximum heatwave severity of the respective observed event. For each region (**a**-**i**), values are shown for the different scenarios (increasing in emissions from left to right for the CMIP5 (yellow), CMIP6 (red), CanESM5 (dark blue), and ACCESS-ESM1.5 (light blue) ensembles. The error bars indicate the 5^th^ and 95^th^ percentile of the respective sample. Where the respective ensemble contains models not able to simulate the severity of the observed event, the proportion of models that are able to simulate an event at least as severe as the observed is indicated by the percentage values above the individual bars. The observed reference values are calculated using observations from Berkeley Earth. For results of all 22 regions, see Supplementary Figs. 317–328.

**Fig. 6:**
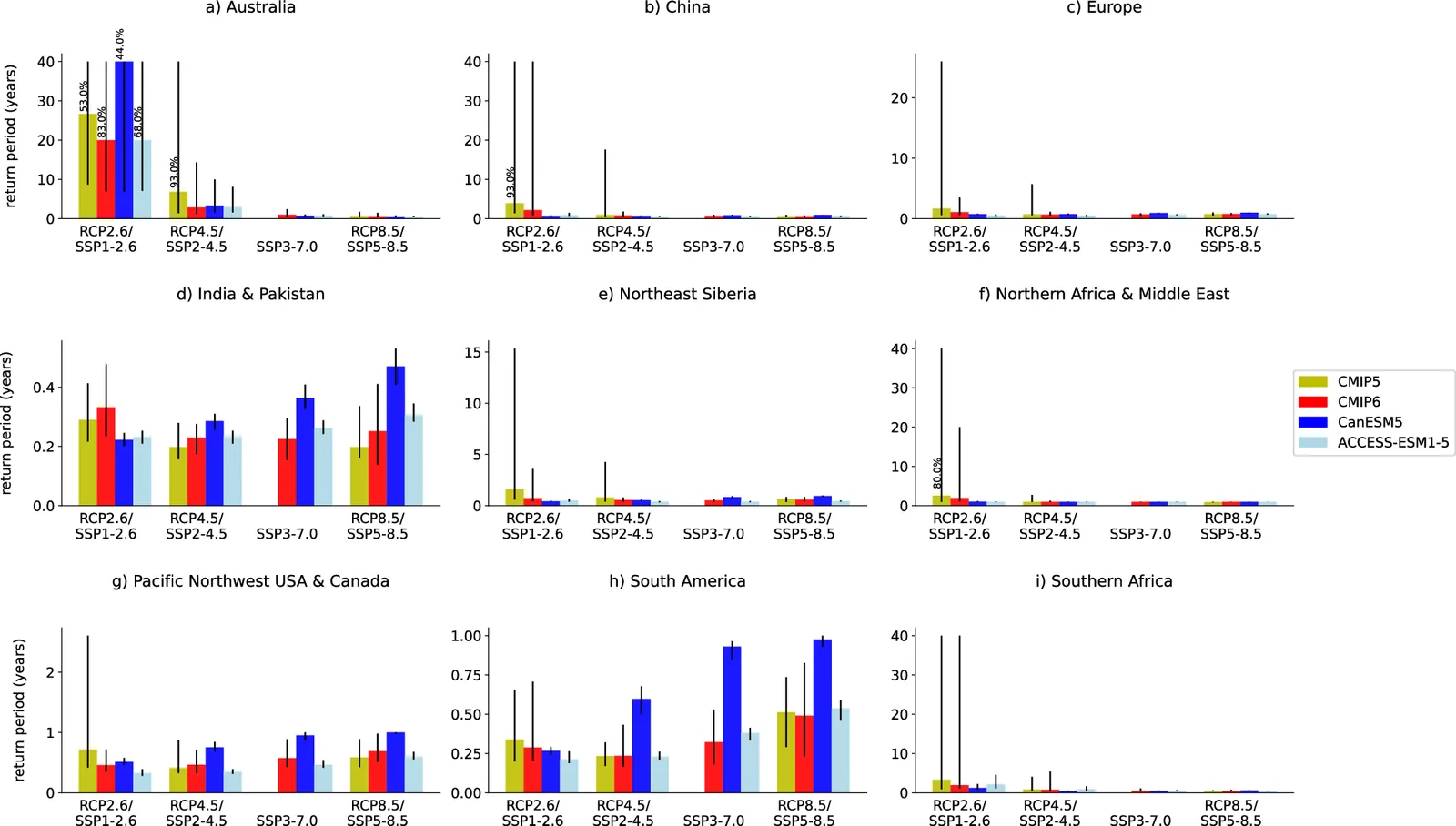
Return time (in years) between 2060 and 2099 for maximum heatwave severity of the respective observed event. For each region (**a**-**i**), values are shown for the different scenarios (increasing in emissions from left to right for the CMIP5 (yellow), CMIP6 (red), CanESM5 (dark blue), and ACCESS-ESM1.5 (light blue) ensembles. The error bars indicate the 5^th^ and 95^th^ percentile of the respective sample. Where the respective ensemble contains models not able to simulate the severity of the observed event, the proportion of models that are able to simulate an event at least as severe as the observed is indicated by the percentage values above the individual bars. The observed reference values are calculated using observations from Berkeley Earth. For results of all 22 regions, see Supplementary Figs. 329–340.

Finally, we assessed return periods for the six heatwave characteristics of the observed heatwaves during three different time windows in the model data: (1) a historical 55-year period from 1951 to 2005, (2) a present-to-near-future 40-year window from 2015 to 2054, and (3) an end-of-century 40-year window from 2060 to 2099. Figures 5 and 6 show the median return time for maximum severity of each ensemble for different scenarios during the present-to-near-future (Fig. 5) and the end-of-century period (Fig. 6). If not explicitly stated otherwise, results discussed below are relative to both Berkeley Earth and ERA5. Across all six heatwave characteristics, most exceptionally high characteristics of observed heatwaves are estimated to have a median return period of 55 years in the historical period (Supplementary Figs. 305–316). In the vast majority of model ensembles, a considerable proportion of models is not able to simulate the level of the respective heatwave characteristics at all during the historical time window. This implies that these models would project return periods beyond the 55-year-mark (see methods for description of return time calculations). In some cases, not a single ensemble member is able to simulate the exceptionally high heatwave characteristic. During the present-to-near-future 40-years period, in most regions exceptionally high heatwave characteristics show clear decreases in median return time (to a number of years in the low single digits), compared to the historical time window, irrespective of emission scenario and ensemble (Supplementary Figs. 317–328). This suggests that recent exceptional events will be far more commonplace than during the second half of the 20^th^ and early 21^st^ century. A notable exception is the maximum severity in Australia (Fig. 5), which shows substantially longer return periods than in other regions, with a considerable number of models not simulating the 2019-heatwave even under high- and very high-emission scenarios.

During the end-of-century period, the majority of ensembles project approximately one or more heatwaves per year for most observed exceptional heatwave characteristics, irrespective of emissions pathway (Figs. 6, Supplementary Figs. 329–340). One exception is, again, the maximum severity during the 2019-heatwave in Australia, where low-emission scenarios (RCP2.6, SSP1-2.6) project return periods of ~20–40 years, depending on the ensemble (Fig. 6).

Tx95p-, Tx97.5p-, and Tx99p-based return times for the six characteristics in MME-CMIP6 show the same tendency as the results discussed above (Supplementary Figs. 341–448).

While there are varying degrees of uncertainty in the range of projected return periods, results indicate considerable agreement among the four ensembles across all three time windows. One exception is maximum severity in Australia under RCP2.6/SSP1-2.6, where for example between 2060 and 2099 (Fig. 6) both MMEs and ACCESS-ESM1.5 SMILE, on the one hand, show a median return time of 20-25 years, with most models in each sample able to simulate the event severity. On the other hand, CanESM5 SMILE projects a median return time of 40 years with most of the 50 model runs not able to simulate the event. This disagreement does, of course, limit the interpretability of the low-emission results in this particular case. It nevertheless hints at a considerable decrease in return period compared to both previous time windows. Our results indicate that much of the uncertainty in future heatwave return times depends on the emissions trajectory followed^42^. The vast majority of SMILEs 5^th^-95^th^ percentile ranges are a fraction of the size of the corresponding MME ranges for the respective region/scenario combination (Figs. 5– 6, Supplementary Figs. 317–340), indicating little influence from internal variability.

## Discussion

We have demonstrated that recently observed exceptional heatwaves are no match for numerous projected future 21^st^ century events. This is clearly evident when multiple heatwave characteristics are assessed in conjunction with one another, as well as when regional changes in distributions and return periods of the analyzed characteristics are examined individually. Our results show that there is a concerningly high probability for recent extreme events being surpassed by projected heatwaves, in many instances even by a large margin, in an intermediate to very high-emissions future. Furthermore, surpassing exceptionally high observed heatwave characteristics within the 21^st^ century is still very plausible over most regions analyzed despite ambitious mitigation under low-emissions scenarios. Ambitious emissions reduction efforts, however, remain just as relevant. Nationally Determined Contributions (NDCs) under the Paris Agreement are projected to limit global warming to approximately 2.6°C (conditional NDCs) or 2.8°C (unconditional NDCs) above pre-industrial levels^43^, corresponding to a GWL of approximately 2.5°C and 3.0°C, respectively. As shown here, limiting temperatures in accordance to a Paris-Agreement-conform 2°C target or NDC-associated temperature levels only provides limited protection and already leads the world into a dangerous future where a concerningly high percentage of projected heatwaves are more severe than exceptionally severe observed events in most regions. However, our results also show that not implementing the current NDCs leads to an even higher likelihood of surpassing observed regional heatwave characteristics. In this case, recently experienced heatwaves resulting in exceptionally high or even peak-characteristics are exceeded by a concerningly high proportion of projected events for all six heatwave characteristics at once.

The usage of regional heatwave thresholds and climatology implicitly assumes that the human population (including infrastructure) and the environment in a particular region is adapted to that specific climate^44^. The population and the environment may partly adapt to some of the future changes in heatwaves described in this study. But there are, for instance, physiological limitations in the human heat tolerance^45^, restricting acclimatization capacity to increased global temperatures^46^, and adaptation efforts may lag behind if future changes in heatwaves are as fast and strong as reflected by our results.

Importantly, we chose to analyze the entire geographical extent of the observed events, encompassing the spatio-temporal evolution of the heatwaves, whereas previous reports on, for instance, India & Pakistan (Supplementary Data 1) and Pacific Northwestern USA & Canada^40,41,47^ focus on considerably smaller regions, emphasizing unprecedented absolute temperatures on grid-box level and from individual station locations^41^, or area-averaged absolute temperatures of a markedly smaller region^40,41,47^. Even though we analyzed a larger geographical region (which does not necessarily capture the more localized effects of the excessive heat, but provides a clearer picture of the entire heatwave, in particular its spatial extent), and applied a different method (severity as opposed to absolute temperature), the shape of the area-weighted-mean-based severity-curve in our study shows remarkable similarities to the area-averaged temperature curves presented in Thompson et al. (2021)^40^ and White et al. (2023)^41^. It speaks to the record-nature of the 2021-heatwave in the North American Pacific region that not only the more localized temperatures, analyzed in previous studies, are outside of the temperature range in the ERA5 data^40^ but that the area-weighted-mean-based maximum severity of the significantly larger region analyzed in the current study is among the top heatwaves in both Berkeley Earth and ERA5.

Acknowledging that observational uncertainty can be an issue in some regions and to compensate for different limitations of certain data products (e.g. spatial resolution), we did not focus our analysis on a single reference data set but chose to present data relative to two independent data sets, representing different types of data products, i.e. Berkeley Earth land-surface observational and ERA5 reanalysis data.

When interpreting projected results in the context of recently observed heatwaves, it has to be taken into consideration that, even though a particular projected heatwave might surpass the observed characteristics, this does not necessarily mean that the projected event will have similar or worse socio-economic impacts as the recently observed reference event. Factors influencing potential differences in socio-economic impacts, for example, are the level and speed of adaptation to future heatwave conditions. Furthermore, the time-window-of-year can have a significant influence regarding socio-economic impacts. A heatwave breaking a severity record in a specific region may have significantly different socio-economic impacts when it occurs in early or late summer compared to the same severity level in mid-summer. Note that this does not imply a seasonal ranking regarding impacts. Even though the same severity level might mean higher absolute temperatures during a specific time-window-of-the-year compared to another, record severity resulting in lower absolute temperatures outside the mid-summer season might have significantly different, yet not necessarily weaker, impacts on society and/or the environment^44,48,49^. Hence, caution needs to be taken when trying to link specific recorded impacts of the observed reference events to projected heatwaves in this study.

Even though we do not quantify and extrapolate specific impacts or impact types into the future – a study focus worthy of future applications of our framework, however, beyond the scope of this study – which might limit the direct applicability of our results for questions about specific impacts, we think that our study still provides valuable impact-relevant information and a useful framework for future impact-specific studies attempting to extrapolate specific recorded impacts into the future. Such an extrapolation of impacts would, for instance, necessitate the focus on a specific set of impacts. In addition, it would require a significantly sized set of additional assumptions and probably a focus on much smaller geographical scales (sub-regional or grid box level only), as the additional assumptions, depending on the selected impact, might not be valid across larger regions^48^. As a result, this could, for instance, limit inter-regional comparability of a large number of regions across the globe, which is one of the strengths of the current study. Furthermore, our research provides critical information for appropriate adaptation to, and mitigation of future changes in heatwaves. Knowledge on how projections of future individual heatwaves directly compare to some of the most extreme observed heatwaves over the recent lived experience provides a line of sight not previously attained, including patterns in the coupling between characteristics, and how the combinations of characteristics compare in their spread across regions. This may facilitate comprehensive risk assessments on how the current coping range of systems will be affected by projected heatwaves over an extensive timeline, ensuring that support systems are sufficiently bolstered, since recent events have already challenged human and natural systems^3,8,11,12,50–52^. In addition, examining heatwave projections in the context of now-observed events may assist in communication of climate risks by helping people understand the significance of potential future 21^st^ century events.

In summary, this study has demonstrated the importance of placing future heatwaves in the perspective of observed events. We demonstrate that the worst heatwaves are yet to come, even in a low-emissions future. However, present-day adaption and mitigation measures to extremes are generally tailored to events occurring during the lifetime of current generations^53–55^. Acknowledging the array of factors influencing the relationship between extreme heat and its impacts^4^, we suggest that our results critically highlight the urgency in including heatwaves well outside the lived experience in the planning of future adaptation and mitigation strategies, given the exceptional nature of observed heatwave characteristics examined in this study. Without this consideration, the gap by which strategies fall short will continue to widen.

## Methods

### Reference heatwaves

Twenty-two observed terrestrial heatwaves (2010-2023) were considered due to their reported high impacts and/or record-breaking nature during recent times (see Supplementary Data 1 for source reports). Calculations of the respective heatwave characteristics draw on daily maximum temperature (${T}_{\max }$) data from two independent data sets: (1) Observational data from the Berkeley Earth gridded (1° x 1°) global land surface temperature dataset^56,57^ (version Feb 10, 2024), and (2) the fifth generation of the European Centre for Medium-Range Weather Forecasts (ECMWF) gridded (0.25° × 0.25°) atmospheric reanalysis (ERA5)^58^. For the purpose of readability we decided to present exceedance percentage results relative to one of the two reference datasets in the main paper, i.e. Berkeley Earth. We present the exceedance percentage relative to ERA5 in Supplementary Data 37–492 and additionally discuss ERA5-based results in the main text where appropriate. Acknowledging the potential advantages of ERA5 (e.g. higher resolution), for the purpose of this study we think that Berkeley Earth is better suited for the exceedance percentage results in the main paper as it interpolates weather station thermometer data to any location on Earth, while concomitantly maximizing the usage of short and discontinuous temperature records^32,57^. In contrast, reanalysis products like ERA5 are produced by data assimilation, i.e. integrating various observational sources with numerical models^59,60^. In addition, Berkeley Earth has previously been used to study regional heatwave trends around the globe^32^. Hence, if not explicitly stated otherwise, the exceedance probability values referred to in the results section are relative to Berkeley Earth data. ERA5 was chosen over other reanalysis products as it is a widely used data set with a high resolution and its performance in relevant regions has been ranked among the best for extreme temperatures^59^. For exceedance probability values relative to ERA5, see Supplements. Observational uncertainty can be an issue in some regions, hence why we employ two independent data products. As our results show, the extremity of the corresponding indices of the observed heatwaves shows relatively large discrepancies in, for example, China, Northern Africa & Middle East, India & Pakistan, and Southern Africa. Overall, the shapes of daily area-weighted-mean-based severity curves in these cases are similar between the reference data sets. In Northern Africa & Middle East, the clear difference between the two heatwave length reference values is simply a result of two consecutive days for which the ERA5 value is marginally below the heatwave threshold ( < 0.02 in heatwave severity), whereas the Berkeley Earth value is not. Apart from these two consecutive days, splitting the ERA5 heatwave into two shorter events, heatwave conditions cover approximately the same time window as in Berkeley Earth. Of the two resulting ERA5-based heatwaves, we chose the one that included the dates mentioned in the source reports (Supplementary Data 1). In India & Pakistan, ERA5 shows consistently lower daily area-weighted-mean-based severity values compared to Berkeley Earth, though the majority of differences in daily severity is relatively small, during the time window of the observed heatwave. As a result, during what are relatively mild heatwave-days in the Berkeley Earth data, ERA5 goes slightly below the heatwave threshold, splitting a period of sustained high temperatures into several different heat events. As in the case of Northern Africa & Middle East, the heatwave including the reported dates was chosen for further analysis. The differences in heatwave length in these two regions directly influence the respective differences in cumulative heat (see definition of cumulative heat below). The differences in spatial extent of the observed events in China and Southern Africa may in part be influenced by the different interpolation methods of Berkeley Earth (distance-based correlation function^56^) and ERA5 (data assimilation^58^), and by certain temperature biases in ERA5^61^. However, a more detailed analysis of the differences in heatwave characteristics between ERA5 and Berkeley Earth, and the potential factors influencing those differences, is beyond the scope of this study.

Observed heatwaves were chosen due to a combination of several factors, including their occurrence during the respective region’s climatologically warmest six months of the year, the magnitude of multiple heatwave characteristics (see Heatwave Characteristics and Exceedance Percentage below), the reported impacts of the events and/or their reported record-breaking nature (see Supplementary Data 1 for a short description and sources of reporting), as well as their geographical distribution across the world and size of the exposed human population. Regional boundaries were determined by the spatio-temporal evolution of each observed heatwave using daily gridded data. A single longitude-latitude box was defined around the affected grids for the entire event and subsequently used for model data analysis (see Model Data). This approach removes complications that can occur when pre-defined regional definitions are used^62^. As the spatio-temporal evolution of heatwaves does not stop at pre-defined regions, i.e. politically defined regions or regions defined in previous peer-reviewed studies, using such pre-defined regions could lead to neglecting relevant parts of the reference heatwaves.

The characteristics of the observed reference heatwaves were ranked based on four categories: (1) heatwave characteristic among the top 3.0% from 1961 to date of reference heatwave, (2) top 3.0% in entire reference dataset from 1961 to 2023, (3) peak value from 1961 to date of reference heatwave, and (4) peak value in entire reference dataset from 1961 to 2023. We find that this provides an important means for interpreting the results in the main text and the basis for defining our statistical descriptor, i.e. exceptional heatwave characteristics. Note that the term exceptional is not used a qualitative statement about a heatwave characteristic but as an intuitive descriptor of heatwave characteristics that reach very rare levels.

The region titles used in our study are supposed to roughly capture the affected region in an intuitive way. The title will, of course, not perfectly capture all parts of a specific region. Hence, in addition to the list of regional coordinates in Table 1, we provide Figures showing the spatio-temporal evolution of the reference events on a geographical map (see Supplementary Figs. 449–492).

### Model data

Heatwave projections were computed for the respective region’s warmest six months (approx. 180 days, depending on the climate model calendar and the region’s climatological cycle) across four different global climate model ensembles. The model analysis draws on the Scenario Model Intercomparison Project (ScenarioMIP)^24^, as part of the Coupled Model Intercomparison Project Phase 6 (CMIP6)^23^, as well as future projections from the Coupled Model Intercomparison Project Phase 5 (CMIP5)^25^. To ensure model-sampling consistency and direct comparability across individual ensemble experiments, analyzed CMIP models had to have daily ${T}_{\max }$ data for historical simulations as well as the following emission scenarios: SSP1-2.6, SSP2-4.5, SSP3-7.0 and SSP5-8.5 for CMIP6^24^, and RCP2.6, RCP4.5 and RCP8.5 for CMIP5^25^. We constructed two multi-model ensembles (MMEs), one for CMIP6 and CMIP5 each. For the MMEs we selected the first available model realization for each CMIP model in the sample (Supplementary Data 35–36). In addition, we utilized two large initial-condition ensembles (SMILEs), participating in CMIP6^23^, of the Canadian Earth System Model version 5 (CanESM5)^26^ and the Australian Earth System Model version 1.5 (ACCESS-ESM1.5)^27^, with 50 and 40 model runs, respectively. The SMILEs were employed to estimate the influence of internal variability on heatwave projections. Because of the large number of individual members, these model ensembles provide a means to investigate how internal climate variability may influence the range in scenario-dependent heatwave trends.

### Model evaluation

Our model evaluation is inspired by Angelil et al. (2017)^63^ and Perkins-Kirkpatrick (2025)^64^. The following steps describing our model evaluation approach, which is inspired by quantile-mapping-based evaluation methods, were performed on each available CMIP model relative to Berkeley Earth and ERA5 separately. Based on the area-weighted mean ${T}_{\max }$, we (1) computed the occurrence percentage of the individual temperature values (Temp%) during the climatological base period (1961-1990) for the climate model and the reference data set. The main focus of the evaluation was to determine the model performance for extreme daily ${T}_{\max }$. At the same time, we wanted to ensure a large enough sample for the performance test. Hence, our evaluation focused on the temperature window above the 80^th^ percentile. We then (2) estimated the temperature variability for each available Temp%. As we only selected the first available realization of each model in the MME, we utilized the climate variability of CanESM5-SMILE to get an estimate of the 1^st^-99^th^ percentile confidence interval range of each available Temp%. We also considered ACCESS-ESM1.5-SMILE for constructing an estimate of the confidence intervals. However, as this did not produce major differences in confidence intervals, we selected CanESM5-SMILE climate variability for the model evaluation due to its larger sample size. (3) Similar to Angelil et al. (2017)^63^ and Perkins-Kirkpatrick et al. (2025)^64^, we aimed to reduce the systematic mean temperature bias in each model by bias correcting the temperature distribution by the difference in the mean temperature above the 80^th^ percentile between the model and the reference data. This results in a simple vertical shift of the entire temperature curve without influencing its shape. In other words, we calculated the mean of the daily area-weighted ${T}_{\max }$ above the 80^th^ percentile for both the model under evaluation and the reference data set. Then we computed the difference between these two values and added this constant to the temperature curve of the reference data set. This means that we shifted the reference data to the models and not the other way around. As the choice of which of the two data sets is shifted vertically does not affect the evaluation of the model performance, this approach was chosen, similar to Angelil et. al. (2017)^63^ and Perkins-Kirkpatrick et. al. (2025)^64^, due to its relative computational simplicity. We then (4) applied the confidence intervals computed based on CanESM5-SMILE (see point (2) above) to the temperature curve of the model via linear interpolation. In other words, we linearly interpolated the model’s temperature values for the available Temp% of the confidence intervals and subsequently centered each confidence interval on the respective interpolated temperature value of the model. We (5) also linearly interpolated the vertically shifted reference temperature data to the available Temp% of the confidence intervals. In the next step (6), each of these interpolated reference data values was compared to the upper and lower bound of the respective confidence interval to calculate how many of the temperature values fall within their respective confidence interval. We computed the percentage of reference temperature data values falling within the confidence intervals of the model for Berkeley Earth and ERA5 individually and (7) ranked the models in accordance to the mean of the two percentage values provided by the comparison to the reference data sets. Subsequently, we (8) selected the best performing 75% of each MME for further analysis and rejected the remaining models, resulting in a sample size of 15 and 18 models for MME-CMIP5 and 6, respectively. As a consequence of this evaluation approach, models with a fairly constant bias in the tail of the temperature curve relative to the reference data will reach a high performance index in the aforementioned ranking. In contrast, climate models with a clearly different tail behavior will perform poorly in our model evaluation. We find that this evaluation approach provides an effective means to reject the models with clearly poor performance in the tail of the temperature distribution. Assuming model performance varies for different regions, we assessed the performance of the MME-CMIP5 and 6 members for each region separately. As a result, the model-sampling slightly differs for each region.

### Heatwave definition

Extreme heat conditions (Tx90p, Tx95p, Tx97.5p, Tx99p) were calculated both on regional and grid box scales. As mentioned in the introduction, taking into account differing statistical robustness due to different sample sizes originating from temperature threshold choice, we mainly focus on the results based on Tx90p in the text and discuss results for higher percentiles where appropriate. As mentioned above (Reference Heatwaves), temperatures above Tx90p on the grid box level were used to establish the geographical boundaries of each regionally observed event, which where consequently used for the model data analysis, whereas the regional scale was used as an indicator to determine if a specific day is part of a heatwave. For an event to be considered a heatwave, (1) regionally area-weighted mean daily ${T}_{\max }$ had to exceed the calendar-day temperature threshold (Tx90p, Tx95p, Tx97.5p, Tx99p) for (2) at least three consecutive days^22,32,65^. In view of the substantial computational cost and as the results discussed in this paper are mainly based on MME-CMIP6, Tx95p-, Tx97.5p-, and Tx99p-based results are calculated for MME-CMIP6 only. To increase the sample size for the day-of-year temperature threshold calculations, we used a 15-day^36,66^ moving window^36^ over the base period 1961-1990^35,65^. For model simulations, the temperature threshold was computed from the corresponding historical simulations over the same time window. A second moving window (31 days) was applied to generate a smoothly varying climatology and heatwave temperature threshold^36^.

Recent research by Brunner & Voigt (2024)^66^ has established that using running windows of 15 days or longer to increase the sample size for percentile-based temperature thresholds can lead to significant biases, as moving windows of this length will artificially mix the mean seasonal cycle into the heatwave temperature threshold. In accordance with their proposed bias correction^66^, we therefore removed the mean seasonal cycle before computing the temperature threshold with a 15-day moving window, and subsequently added the mean seasonal cycle back onto the preliminary temperature threshold. Next, we used a second, longer moving window of 31 days after the percentile threshold calculation to generate a smoothly varying temperature threshold, which does not significantly influence the effect of the bias correction and the threshold calculation (Supplementary Fig. 493). The relative heatwave definition uses a fixed historical base period (1961-1990). We chose this approach, as (1) humans are physiologically limited in their heat tolerance^45^, restricting their capacity for acclimatization as global temperatures continue to rise^46^. And (2) a fixed base period allows for the assessment of how heatwaves have changed due to the presence of an external forcing (i.e. anthropogenic climate change), whereas the application of a moving base period would smooth out any such changes by constantly shifting the climate against which heatwaves are measured towards increasingly warmer background conditions.

### Heatwave characteristics and exceedance percentage

Our heatwave analysis framework is based on six characteristics, which are used to put projected heatwaves in the context of recent extreme events. These characteristics are calculated for each of the spatial domains (Tables 1 & Supplementary Data 1) prescribed by the selected observed heatwaves. Most characteristics have previously been established in the literature^22,32,35,65,67^ although not combined to consider a spectrum of individual events.

1. Heatwave severity^36,65^, an extreme heat index, characterizes temperature values relative to the underlying regional climatology. This heatwave characteristic has previously been defined for marine heatwaves^36,65^, however is directly transferable to atmospheric events. The severity value of a specific day (Sev_day_) is the difference between that day’s temperature value (T_day_) and the calendar-day climatology (${{{\rm{Clim}}}}_{{{\rm{CalDay}}}}$), normalized by the difference between the day-of-year temperature threshold (Txpct, i.e. Tx90p, Tx95p, Tx97.5p, Tx99p) and the day-of-year climatology (${{{\rm{Clim}}}}_{{{\rm{CalDay}}}}$):
  1 $${{{\rm{Sev}}}}_{{{\rm{day}}}}=\dfrac{{{{\rm{T}}}}_{{{\rm{day}}}}-{{{\rm{Clim}}}}_{{{\rm{CalDay}}}}}{{{\rm{Txpct}}}-{{{\rm{Clim}}}}_{{{\rm{CalDay}}}}}$$
  For example, a severity value of 2 indicates that the difference between the calendar-day climatology (${{{\rm{Clim}}}}_{{{\rm{CalDay}}}}$) and the temperature of a specific day (T_day_) during a heatwave is twice as large as the difference between climatology and the heatwave threshold value. For each heatwave we calculated the median and maximum severity.
2. Cumulative Heat (heat_cum_)^34^ describes the accumulated temperature anomaly, in °C, above the temperature threshold (Txpct, i.e. Tx90p, Tx95p, Tx97.5p, Tx99p) during the course of an individual heatwave:
  2 $${{{\rm{heat}}}}_{{{\rm{cum}}}}=\sum ({{{\rm{T}}}}_{{{\rm{day}}}}-{{\rm{Txpct}}})$$
3. Heatwave duration^22^ shows the overall length of the event, measured in days.
4. Spatial extent of locally heat-affected area ("heatwave area”) describes the latitude-weighted (*w*_Lat_) percentage of grid boxes affected by extreme heat (gb_aff_), i.e. grid boxes (gb) exceeding the aforementioned temperature threshold:
  3 $${{\rm{Area}}}=\dfrac{\sum {w}_{{{\rm{Lat}}}}\,{{{\rm{gb}}}}_{{{\rm{aff}}}}}{\sum {w}_{{{\rm{Lat}}}}\,{{\rm{gb}}}}$$
  For each heatwave we calculated the median and maximum locally heat-affected area.

The percentage of heatwaves exceeding the respective heatwave characteristics (exceedance percentage) used in the main text to put projected heatwaves in the context of recent extreme events represents the relative likelihood of a heatwave in the respective ensemble sample to surpass the analyzed heatwave characteristic of the observed event. In other words, if e.g. a specific region shows an exceedance percentage of approximately 10% in SSP1-2.6 for heatwave length, this means that approximately 10% of projected heatwaves in the SSP1-2.6 sample of this region are longer than the observed reference event. The same applies to the respective GWL samples (GWLs 0–4.5°C).

### Grid resolution

All grid box-level heatwave calculations were performed on the native grid of each dataset. We did consider regridding for the purpose of area calculations. Conservative regridding, for example, would have had the advantage that area-weighted-mean results remain the same, compared to using the native grid (at least to numerical precision) while providing a standard grid resolution across all models, with potential advantages when comparing spatial extent of heatwaves across different models. However, regridding can have significant disadvantages compared to using the native grid, like averaging across coastal grid-points’ ocean fraction at high resolution and interpolating to a grid/grid-resolution that does not align with the effective resolved orography and physics of the model. Thus, we designed a sensitivity test to estimate if different resolutions of the models might have a significant effect on the distribution of the computed heatwave areas, i.e. if the apparent differences in the spread in heatwave areas among different models can be explained by the different grid resolutions. In other words we test if the apparent differences are only statistical artifacts rather than differences in model physics. Our sensitivity test works as follows:

1. Selection of three different regular lon-lat grids (r320 × 160, r256 × 128, r128 × 64) available for the Climate Data Operators (CDO) (MPI for Meteorology, Hamburg, Germany)^68^.
2. Re-gridding of entire set of CMIP6 models using conservative re-gridding (CDO remapcon) for SSP1-2.6, SSP3-7.0, and historical model runs.
3. Calculation of local severity and, subsequently, heatwave area for each heatwave in the respective sample.
4. Kernel Density Estimates (KDEs) (Supplementary Figs. 494–497) of the entire distribution of heatwave area values per model, region, model run, and grid resolution.

The results of our sensitivity test show that the distributions of the computed heatwave areas for the different grid resolutions do not display substantial differences (Supplementary Figs. 494–497). Hence, our test indicates that differing grid resolutions, if anything, only have a minor effect on model-specific differences regarding distributions of heatwave areas. These results are robust across model scenarios (SSP1-2.6 and SSP3-7.0) for using both a finer as well as a coarser target grid for regridding (Supplementary Figs. 494–497).

### Global warming levels (GWLs)

Future changes in heatwave characteristics mentioned above were assessed relative to GWLs. Centered 20-year rolling windows are used to determine which time window corresponds to a specific GWL^69^. The sample period for a particular GWL-value is the 20-year period centered around the first year in which the 20-year rolling mean reaches the specified value^69^. In our study, we present GWLs up to 4.5°C of warming in 0.5°C increments. These GWLs are reached by at least six models in MME-CMIP5 ( > 40%), nine models in MME-CMIP6 (50%) and 100% of the model realizations in both SMILEs. The warming levels are computed from area-weighted yearly global mean temperature of each model realization, relative to its global mean temperature between 1850 and 1900. Due to a greater number of available climate models reaching higher GWLs ( > 3.0°C), and the similarity to the distributions under lower emission scenarios, we only present the results where the GWL is computed using the highest emission scenarios (SSP5-8.5/RCP8.5). Note that historical simulations for some CMIP5 models start after 1850. Thus, to maximize the sample size for the GWL-based distributions we also included models with a start year of up to 1861 in the CMIP5-MME.

### Return periods

We estimated changes in return periods for each regional reference event characteristic across all four model ensembles and corresponding emission scenarios for three different time windows in the model data: (1) a historical 55-year period from 1951 to 2005, (2) a present-to-near-future 40-year window from 2015 to 2054, and (3) an end-of-century 40-year window from 2060 to 2099. We chose these specific time windows (1) to compare the recent 21^st^ century period to an end-of-century period, where the emission scenarios start to diverge^24,25,70^, and to results of a historical period, that is covered by both Berkeley Earth and ERA5, (2) to provide a sufficiently sized data sample for computing return periods, and (3) to avoid using an artificially shortened summer season for southern hemisphere regions in the year 2100, as a significant part of this particular season extends to the year 2101. We applied an empirical approach and estimated the return periods by counting the number of heatwaves, where heatwave characteristics are greater or equal to the reference values for each model in the ensemble, during the respective time window, and dividing it by the length of the time window. While acknowledging the caveats of using an empirical return period method, such as the limitation of the maximum return period to the length of the analyzed time window, we think that, for the purpose of our study, applying an empirical return period approach has significant advantages compared to e.g. fitting a GEV distribution. In addition to only using minimal assumptions (essentially non-parametric), our empirical return period approach does not only use block maxima, as in the case of fitting a GEV distribution, but uses the entire sample of events exceeding the reference heatwave characteristic. Hence, apart from avoiding the reduction in sample size as a result of fitting a GEV distribution, taking the entire sample of exceeding events is relevant in our case, as our analysis shows that there is a considerable number of heatwaves with a heatwave characteristic value greater than those of the observed event but less than the respective block maximum. Hence, by only taking block maxima into account, our analysis would lose a considerable number of relevant projected events. Our analysis indicates that in many of the selected regions, future heatwaves exceeding the respective reference heatwaves are projected to happen more often than once per year. Thus, our empirical return period approach provides a higher precision compared to using block maxima. If anything, our results are conservative in the sense that we compare the return periods in the present-to-near-future and the end-of-century time windows to a historical 55-year period, which shows return periods limited by the length of the time window. However, as indicated in the results section, the relatively large percentage of models not able to simulate the respective heatwave characteristic of the observed event indicates that the return period would likely be considerably longer than 55-years. Nevertheless, the return times markedly decrease in our analysis in the present-to-near-future and the end-of-century time windows compared to the historical 55-year period. As an alternative to GEV, we did consider the use of a Generalized Pareto Distribution (GPD) within a Peaks-Over-Threshold (POT) framework, as this could alleviate the issue related to block-maxima discussed above. However, GPD is not applicable to a substantial part of our analysis framework. To the best of our knowledge, there is no theoretical basis for assuming that a sum such as cumulative heat or summary statistics like median severity and median heatwave area inherit the generalized Pareto distribution of their daily threshold exceedances. Hence, the application of GPD would risk fundamental misspecification.

## Supplementary information

Supplementary Information

Description of Additional Supplementary Files

Transparent Peer Review file

## Source data

Supplementary Data

## Data Availability

All raw data used in this study are freely available for research applications. Berkeley Earth daily gridded land-surface data: https://berkeleyearth.org/data; ERA5 hourly 2 m temperature: https://cds.climate.copernicus.eu/datasets/reanalysis-era5-single-levels?Tab=download; CMIP5 and CMIP6 daily maximum 2 m surface air temperature: https://esgf-node.ornl.gov/search. All data needed to evaluate the conclusions of the paper are present in the paper and/or Supplementary Data 1–36. The data necessary for reproducing the Figures presented in the paper cannot be made publicly available upon publication of the paper due to its size, and is, hence, available upon request from the first author. Response to data requests can be expected within a few days. Source data are provided with this paper.

## Code availability

All codes used for analyses of the simulation data are available from the first author upon request. Demo versions of central calculation scripts are available on Zenodo under https://doi.org/10.5281/zenodo.21599194.
