# Supplementary material for: Recent extreme heatwaves are no match for future events
Source: Nat Commun. 2026 Nov 5;17:11456. doi: 10.1038/s41467-026-76809-5 (PMC13740290; doi:10.1038/s41467-026-76809-5)
Supplement: Supplementary file 1 [file 41467_2026_76809_MOESM1_ESM.pdf]

Supplementary Information: 'Recent extreme heatwaves are no match for future events'

Philipp Aglas-Leitner[1,2,3,4,5,6*], Sarah E. Perkins-Kirkpatrick[4,6,7], Lukas Brunner[8], Sabine Undorf[5], Florentina Aglas-Leitner[9], Erich Fischer[10], Dáithí A. Stone[11]

[1]Department of Physics, Freie Universität Berlin, Berlin, Germany.
[2]Department of Mathematics and Computer Science, Freie Universität Berlin, Berlin, Germany.
[3]Climate Change Research Centre, University of New South Wales, Sydney, Australia.
[4]Fenner School of Environment and Society, Australian National University, Canberra, Australia.
[5]Department for Climate Resilience, Potsdam Institute for Climate Impact Research, Potsdam,Germany.
[6]ARC Centre of Excellence for Climate Extremes, Canberra, Australia.
[7]ARC Centre of Excellence for the Weather of the 21st Century, Canberra, Australia.
[8]Research Unit Sustainability and Climate Risk, Earth and Society Research Hub (ESRAH), University of Hamburg, Hamburg, Germany.
[9]Department of Infectious Diseases, Respiratory Medicine, and Critical Care, Charité-Universitätsmedizin Berlin, Berlin, Germany.
[10]Institute for Atmospheric and Climate Science, ETH Zurich, Zurich, Switzerland.
[11]Earth Sciences New Zealand, Wellington, New Zealand.

*Corresponding author(s). E-mail(s): philipp.aglas@fu-berlin.de;

Description

The Supplementary Information contains all Supplementary Figures referenced in the paper. The information in this PDF is structured in blocks of two pages. The links

to each such block is provided in the PDF bookmarks. The first page of each such block contains the figure caption consisting of the figure title (see bookmarks) and a description of what information the supplementary figure is presenting. The second page of each block presents the supplementary figure.

The PDF bookmarks provide a detailed table of content where each figure referenced in the paper can easily be accessed. In addition, we provide a summary table of content below for the reader to have an overview of the different types of figures presented in this PDF.

Table of Content

Supplementary Figs 1–18: Multi-HW-characteristic figures and Kernel Density Estimates (KDE) based on historical Multi-Model-Ensemble simulations

Supplementary Figs 19–40: Multi-HW-characteristic figures based on Multi-Model-Ensemble future simulations (CMIP6 and CMIP5) (Tx90p)

Supplementary Figs 41–82: Kernel Density Estimates (KDE) across Decades based on Multi-Model-Ensemble future simulations (CMIP6 and CMIP5) (Tx90p)

Supplementary Figs 83–112: Multi-HW-characteristic figures based on Multi-Model-Ensemble simulations (CMIP6) (Tx95p-Tx99p)

Supplementary Figs 113–202: Kernel Density Estimates (KDE) across Decades based on Multi-Model-Ensemble simulations (CMIP6) (Tx95p-Tx99p)

Supplementary Figs 203–290: Multi-HW-characteristic figures per individual model based on Multi-Model-Ensemble future simulations (CMIP6)

Supplementary Figs 291–302: Kernel Density Estimates (KDE) across Global Warming Levels (GWLs) based on Multi-Model-Ensemble future simulations (CMIP6 and CMIP5) (Tx90p)

Supplementary Figs 303–304: Reference Event Mapping for Pacific Northwestern USA & Canada

Supplementary Figs 304–340: Return Times based on Tx90p

Supplementary Figs 341–448: Return Times based on Tx95p–Tx99p

Supplementary Figs 449–492: Reference Event Mapping

Supplementary Figs 493–497: Sensitivity Tests

Supplementary Figure 1: Multi-HW-characteristic figure according to Historical CMIP6 multi-model ensemble (MME) (Tx90p) - Each panel shows regional projections according to Historical model runs in the CMIP6 multi-model ensemble (MME) of four combined heatwave characteristics for each projected heatwave in the ensemble between 2020 and 2100. Each dot represents an individual heatwave in the projections, characterized by Median Heatwave Area (%) (x-axis) as latitude-weighted percentage of grid boxes affected by extreme heat, Heatwave Length (days) (y-axis) in days, Median Heatwave Severity (color), and Cumulative Heat (°C) (dot size). The characteristics of the observed event according to the Berkeley Earth observational data (x symbol) and the ERA5 reanalysis data (+ symbol), respectively, are pointed to by an arrow and a figure inset shows a zoom-in on this figure section. The figure inset shows the observed heatwave in the two reference data sets and projections in the indicated section of the respective sub-plot. It facilitates readability of color and size of the reference values and comparison to each other and the neighboring projections. Note that the zoom-in causes the dots to overlap more in the inset than in the main sub-plot. The univariate kernel density estimates along the x- (Median Heatwave Area (%)) and y-axis (Heatwave Length (days)) are on the top and right side of the subplots, respectively, with vertical lines in dark blue (Berkeley Earth) and light blue (ERA5) indicating the observed event.

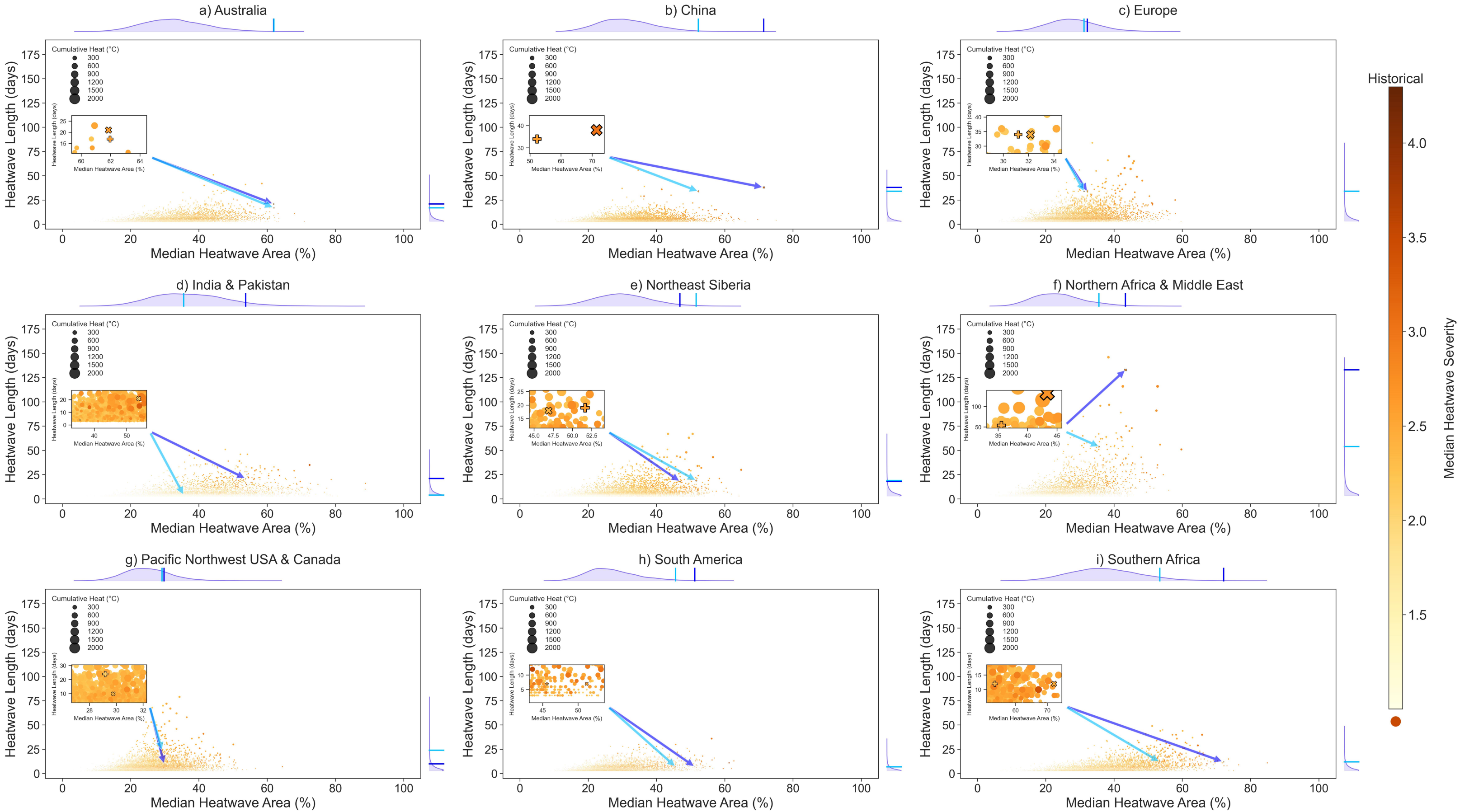
a) Australia
b) China
c) Europe
d) India & Pakistan
e) Northeast Siberia
f) Northern Africa & Middle East
g) Pacific Northwest USA & Canada
h) South America
i) Southern Africa
Heatwave Length (days)
Median Heatwave Area (%)
Cumulative Heat (°C)
300
600
900
1200
1500
2000
Historical
Median Heatwave Severity

Supplementary Figure 2: Multi-HW-characteristic figure according to Historical CMIP6 multi-model ensemble (MME) (Tx90p) - Each panel shows regional projections according to Historical model runs in the CMIP6 multi-model ensemble (MME) of four combined heatwave characteristics for each projected heatwave in the ensemble between 2020 and 2100. Each dot represents an individual heatwave in the projections, characterized by Maximum Heatwave Area (%) (x-axis) as latitude-weighted percentage of grid boxes affected by extreme heat, Heatwave Length (days) (y-axis) in days, Maximum Heatwave Severity (color), and Cumulative Heat (°C) (dot size). The characteristics of the observed event according to the Berkeley Earth observational data (x symbol) and the ERA5 reanalysis data (+ symbol), respectively, are pointed to by an arrow and a figure inset shows a zoom-in on this figure section. The figure inset shows the observed heatwave in the two reference data sets and projections in the indicated section of the respective sub-plot. It facilitates readability of color and size of the reference values and comparison to each other and the neighboring projections. Note that the zoom-in causes the dots to overlap more in the inset than in the main sub-plot. The univariate kernel density estimates along the x- (Maximum Heatwave Area (%)) and y-axis (Heatwave Length (days)) are on the top and right side of the subplots, respectively, with vertical lines in dark blue (Berkeley Earth) and light blue (ERA5) indicating the observed event.

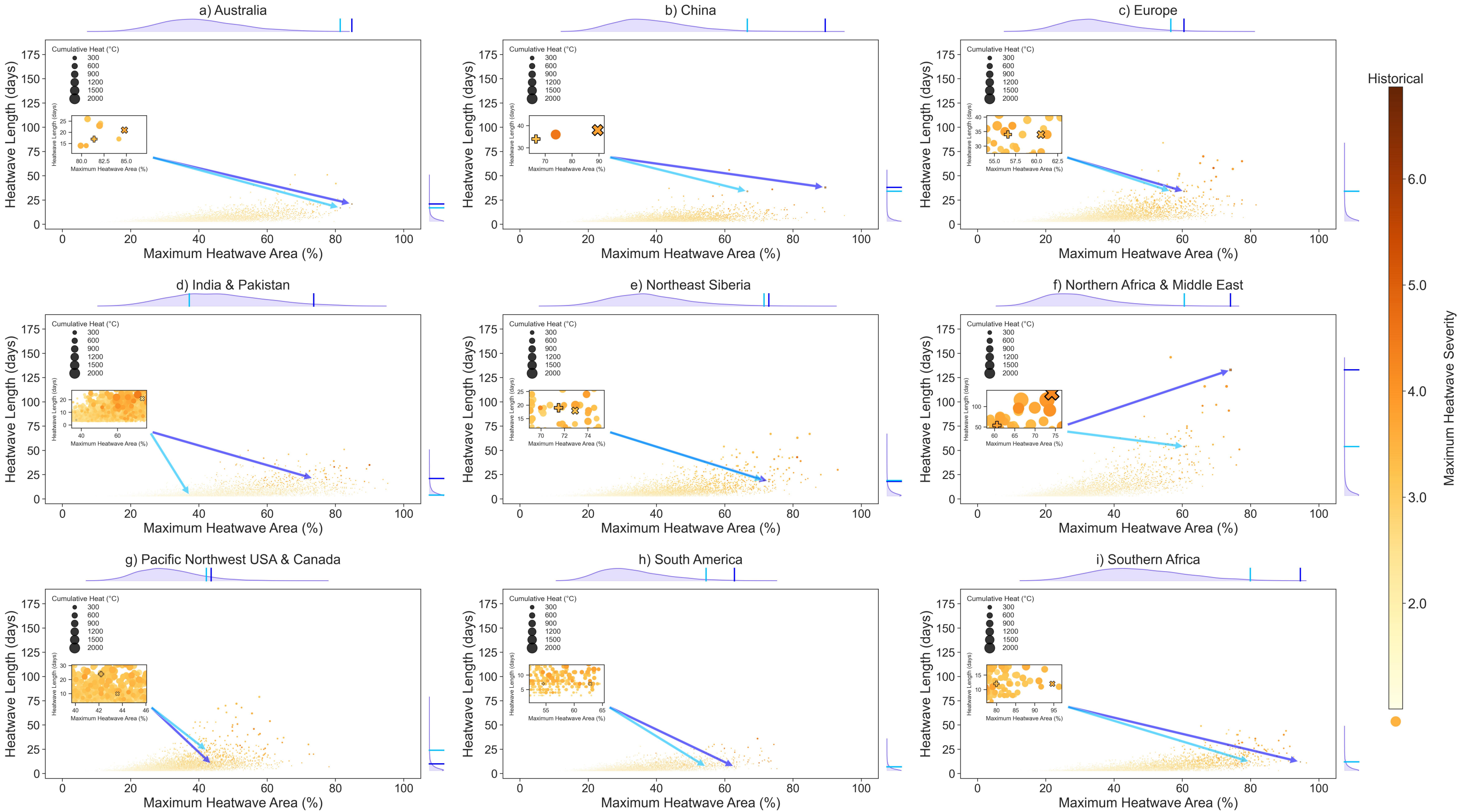

a) Australia
b) China
c) Europe
d) India & Pakistan
e) Northeast Siberia
f) Northern Africa & Middle East
g) Pacific Northwest USA & Canada
h) South America
i) Southern Africa
Heatwave Length (days)
Maximum Heatwave Area (%)
Cumulative Heat (°C)
300
600
900
1200
1500
2000
Historical
Maximum Heatwave Severity

Supplementary Figure 3: Multi-HW-characteristic figure according to Historical CMIP6 multi-model ensemble (MME) (Tx90p) - Each panel shows regional projections according to Historical model runs in the CMIP6 multi-model ensemble (MME) of four combined heatwave characteristics for each projected heatwave in the ensemble between 2020 and 2100. Each dot represents an individual heatwave in the projections, characterized by Median Heatwave Area (%) (x-axis) as latitude-weighted percentage of grid boxes affected by extreme heat, Heatwave Length (days) (y-axis) in days, Median Heatwave Severity (color), and Cumulative Heat (°C) (dot size). The characteristics of the observed event according to the Berkeley Earth observational data (x symbol) and the ERA5 reanalysis data (+ symbol), respectively, are pointed to by an arrow and a figure inset shows a zoom-in on this figure section. The figure inset shows the observed heatwave in the two reference data sets and projections in the indicated section of the respective sub-plot. It facilitates readability of color and size of the reference values and comparison to each other and the neighboring projections. Note that the zoom-in causes the dots to overlap more in the inset than in the main sub-plot. The univariate kernel density estimates along the x- (Median Heatwave Area (%)) and y-axis (Heatwave Length (days)) are on the top and right side of the subplots, respectively, with vertical lines in dark blue (Berkeley Earth) and light blue (ERA5) indicating the observed event.

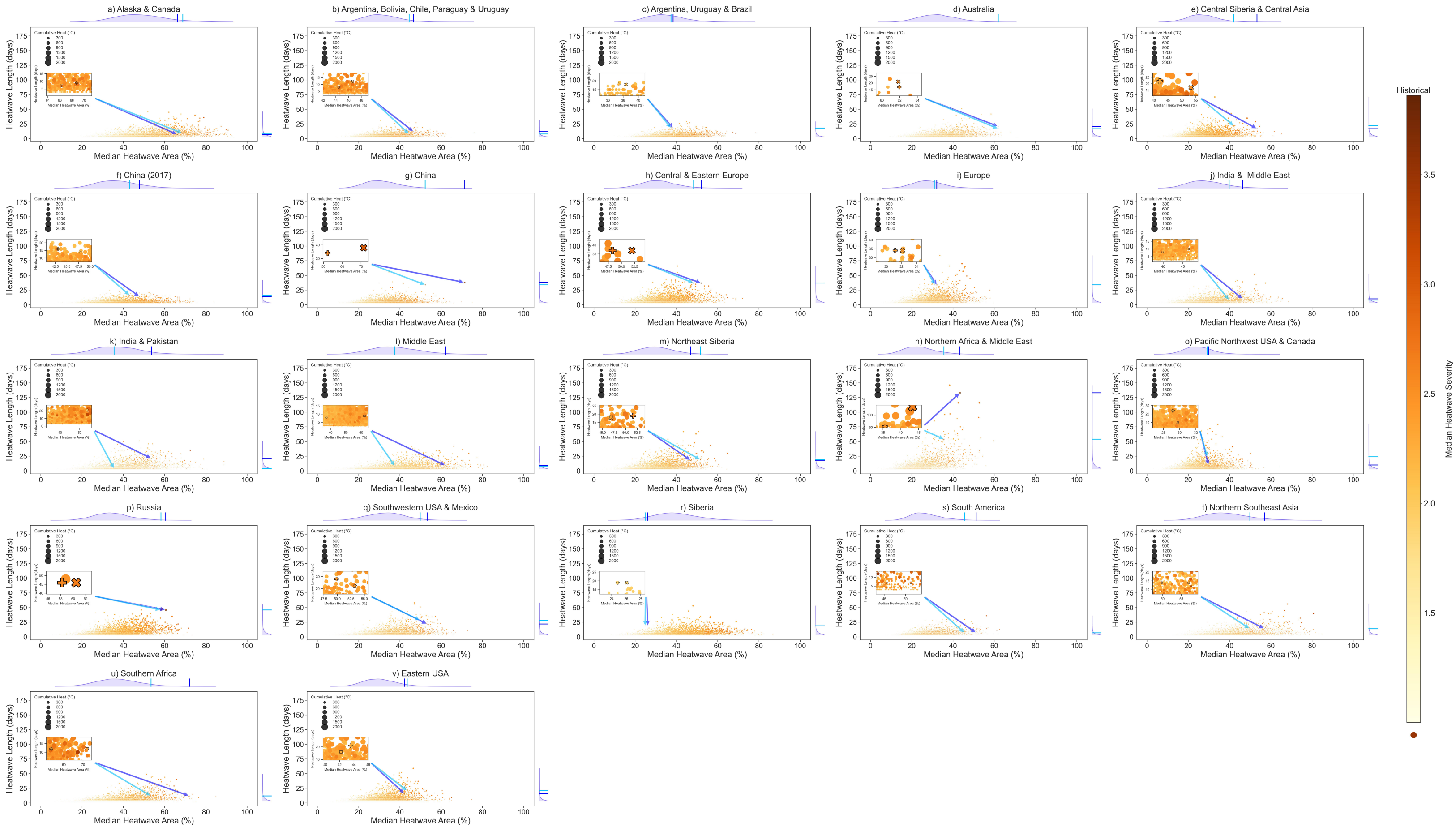

a) Alaska & Canada
b) Argentina, Bolivia, Chile, Paraguay & Uruguay
c) Argentina, Uruguay & Brazil
d) Australia
e) Central Siberia & Central Asia
f) China (2017)
g) China
h) Central & Eastern Europe
i) Europe
j) India & Middle East
k) India & Pakistan
l) Middle East
m) Northeast Siberia
n) Northern Africa & Middle East
o) Pacific Northwest USA & Canada
p) Russia
q) Southwestern USA & Mexico
r) Siberia
s) South America
t) Northern Southeast Asia
u) Southern Africa
v) Eastern USA
Heatwave Length (days)
Median Heatwave Area (%)
Cumulative Heat (°C)
300
600
900
1200
1500
2000
Historical
Median Heatwave Severity

Supplementary Figure 4: Multi-HW-characteristic figure according to Historical CMIP6 multi-model ensemble (MME) (Tx90p) - Each panel shows regional projections according to Historical model runs in the CMIP6 multi-model ensemble (MME) of four combined heatwave characteristics for each projected heatwave in the ensemble between 2020 and 2100. Each dot represents an individual heatwave in the projections, characterized by Maximum Heatwave Area (%) (x-axis) as latitude-weighted percentage of grid boxes affected by extreme heat, Heatwave Length (days) (y-axis) in days, Maximum Heatwave Severity (color), and Cumulative Heat (°C) (dot size). The characteristics of the observed event according to the Berkeley Earth observational data (x symbol) and the ERA5 reanalysis data (+ symbol), respectively, are pointed to by an arrow and a figure inset shows a zoom-in on this figure section. The figure inset shows the observed heatwave in the two reference data sets and projections in the indicated section of the respective sub-plot. It facilitates readability of color and size of the reference values and comparison to each other and the neighboring projections. Note that the zoom-in causes the dots to overlap more in the inset than in the main sub-plot. The univariate kernel density estimates along the x- (Maximum Heatwave Area (%)) and y-axis (Heatwave Length (days)) are on the top and right side of the subplots, respectively, with vertical lines in dark blue (Berkeley Earth) and light blue (ERA5) indicating the observed event.

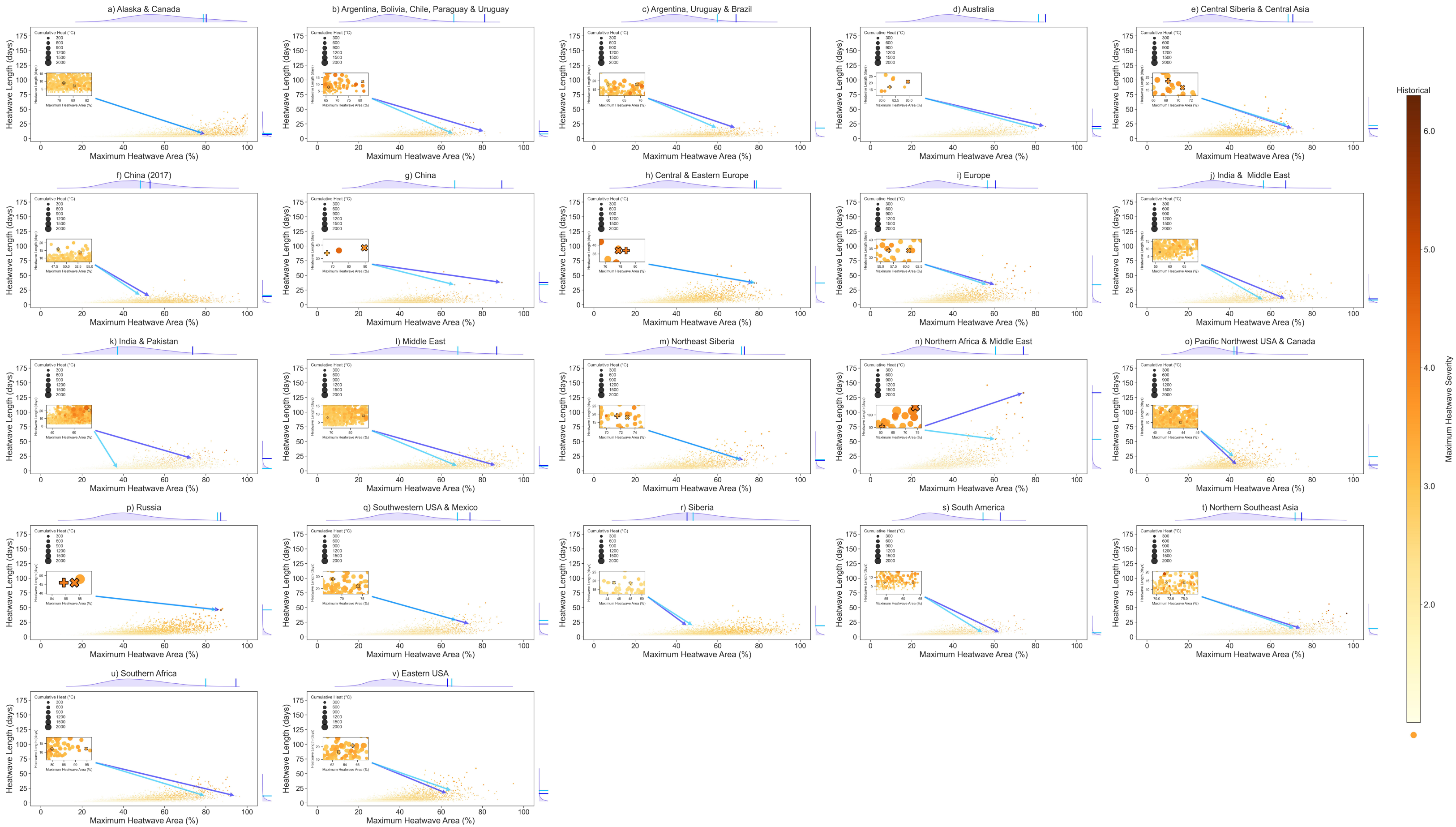

a) Alaska & Canada
b) Argentina, Bolivia, Chile, Paraguay & Uruguay
c) Argentina, Uruguay & Brazil
d) Australia
e) Central Siberia & Central Asia
f) China (2017)
g) China
h) Central & Eastern Europe
i) Europe
j) India & Middle East
k) India & Pakistan
l) Middle East
m) Northeast Siberia
n) Northern Africa & Middle East
o) Pacific Northwest USA & Canada
p) Russia
q) Southwestern USA & Mexico
r) Siberia
s) South America
t) Northern Southeast Asia
u) Southern Africa
v) Eastern USA
Heatwave Length (days)
Maximum Heatwave Area (%)
Cumulative Heat (°C)
300
600
900
1200
1500
2000
Historical
Maximum Heatwave Severity
2.0
3.0
4.0
5.0
6.0

Supplementary Figure 5: Multi-HW-characteristic figure according to Historical CMIP5 multi-model ensemble (MME) (Tx90p) - Each panel shows regional projections according to Historical model runs in the CMIP5 multi-model ensemble (MME) of four combined heatwave characteristics for each projected heatwave in the ensemble between 2020 and 2100. Each dot represents an individual heatwave in the projections, characterized by Median Heatwave Area (%) (x-axis) as latitude-weighted percentage of grid boxes affected by extreme heat, Heatwave Length (days) (y-axis) in days, Median Heatwave Severity (color), and Cumulative Heat (°C) (dot size). The characteristics of the observed event according to the Berkeley Earth observational data (x symbol) and the ERA5 reanalysis data (+ symbol), respectively, are pointed to by an arrow and a figure inset shows a zoom-in on this figure section. The figure inset shows the observed heatwave in the two reference data sets and projections in the indicated section of the respective sub-plot. It facilitates readability of color and size of the reference values and comparison to each other and the neighboring projections. Note that the zoom-in causes the dots to overlap more in the inset than in the main sub-plot. The univariate kernel density estimates along the x- (Median Heatwave Area (%)) and y-axis (Heatwave Length (days)) are on the top and right side of the subplots, respectively, with vertical lines in dark blue (Berkeley Earth) and light blue (ERA5) indicating the observed event.

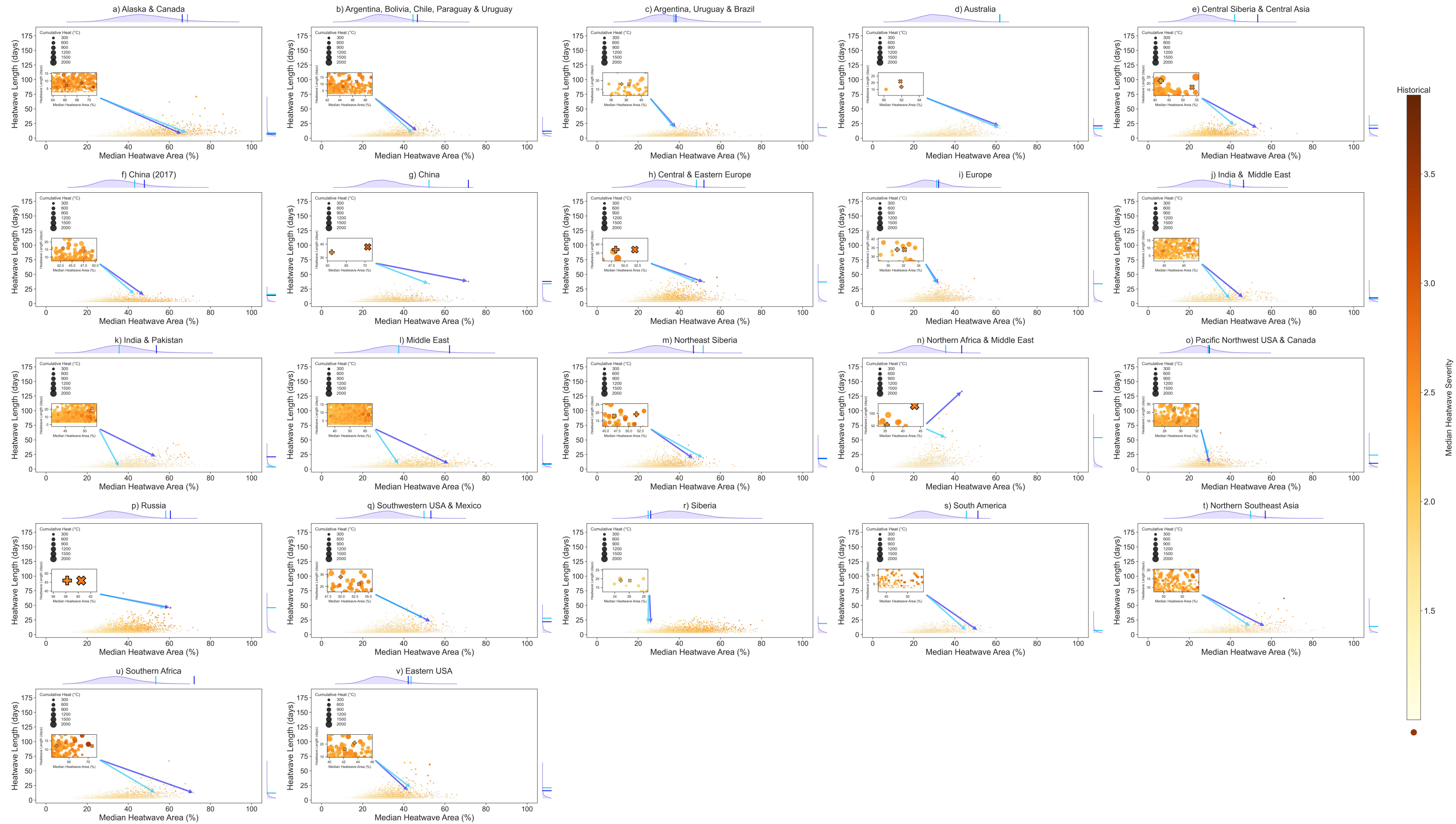
a) Alaska & Canada
b) Argentina, Bolivia, Chile, Paraguay & Uruguay
c) Argentina, Uruguay & Brazil
d) Australia
e) Central Siberia & Central Asia
f) China (2017)
g) China
h) Central & Eastern Europe
i) Europe
j) India & Middle East
k) India & Pakistan
l) Middle East
m) Northeast Siberia
n) Northern Africa & Middle East
o) Pacific Northwest USA & Canada
p) Russia
q) Southwestern USA & Mexico
r) Siberia
s) South America
t) Northern Southeast Asia
u) Southern Africa
v) Eastern USA
Heatwave Length (days)
Median Heatwave Area (%)
Cumulative Heat (°C)
300
600
900
1200
1500
2000
Historical
Median Heatwave Severity
1.5
2.0
2.5
3.0
3.5

Supplementary Figure 6: Multi-HW-characteristic figure according to Historical CMIP5 multi-model ensemble (MME) (Tx90p) - Each panel shows regional projections according to Historical model runs in the CMIP5 multi-model ensemble (MME) of four combined heatwave characteristics for each projected heatwave in the ensemble between 2020 and 2100. Each dot represents an individual heatwave in the projections, characterized by Maximum Heatwave Area (%) (x-axis) as latitude-weighted percentage of grid boxes affected by extreme heat, Heatwave Length (days) (y-axis) in days, Maximum Heatwave Severity (color), and Cumulative Heat (°C) (dot size). The characteristics of the observed event according to the Berkeley Earth observational data (x symbol) and the ERA5 reanalysis data (+ symbol), respectively, are pointed to by an arrow and a figure inset shows a zoom-in on this figure section. The figure inset shows the observed heatwave in the two reference data sets and projections in the indicated section of the respective sub-plot. It facilitates readability of color and size of the reference values and comparison to each other and the neighboring projections. Note that the zoom-in causes the dots to overlap more in the inset than in the main sub-plot. The univariate kernel density estimates along the x- (Maximum Heatwave Area (%)) and y-axis (Heatwave Length (days)) are on the top and right side of the subplots, respectively, with vertical lines in dark blue (Berkeley Earth) and light blue (ERA5) indicating the observed event.

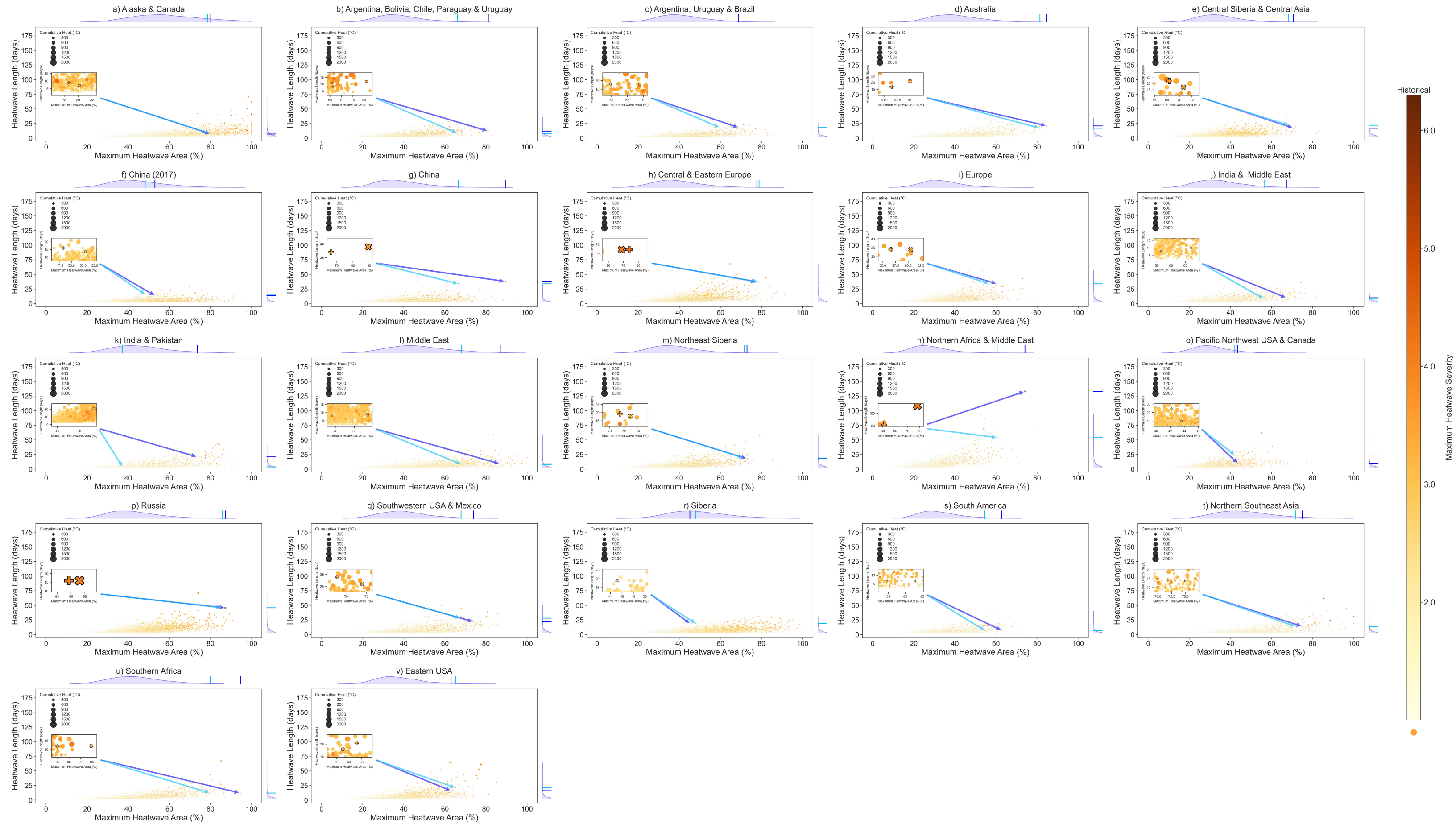
a) Alaska & Canada
b) Argentina, Bolivia, Chile, Paraguay & Uruguay
c) Argentina, Uruguay & Brazil
d) Australia
e) Central Siberia & Central Asia
f) China (2017)
g) China
h) Central & Eastern Europe
i) Europe
j) India & Middle East
k) India & Pakistan
l) Middle East
m) Northeast Siberia
n) Northern Africa & Middle East
o) Pacific Northwest USA & Canada
p) Russia
q) Southwestern USA & Mexico
r) Siberia
s) South America
t) Northern Southeast Asia
u) Southern Africa
v) Eastern USA
Heatwave Length (days)
Maximum Heatwave Area (%)
Cumulative Heat (°C)
300
600
900
1200
1500
2000
Historical
Maximum Heatwave Severity

Supplementary Figure 7: Kernel Density Estimates (KDE) across Decades for Cumulative Heat (°C) in Historical CMIP6 multi-model ensemble (MME) (Tx90p) - Kernel Density Estimates (KDE) of Cumulative Heat (°C) for different Decades in the CMIP6 multi-model ensemble (MME). Area under each curve is normalized to 1. Cumulative Heat (°C) of the observed event is indicated by vertical lines in dark blue (Berkeley Earth) and light blue (ERA5).

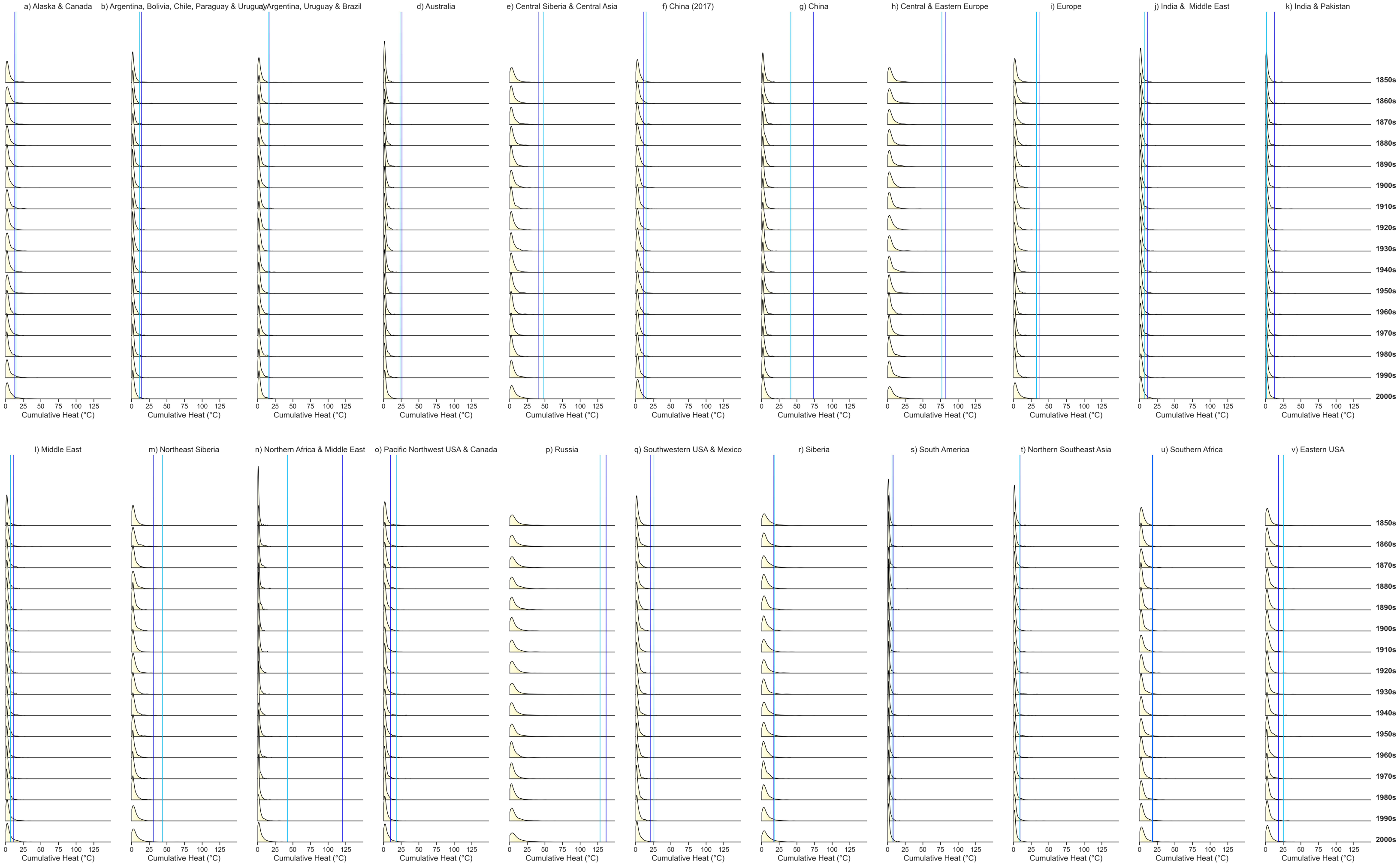
a) Alaska & Canada
b) Argentina, Bolivia, Chile, Paraguay & Uruguay
c) Argentina, Uruguay & Brazil
d) Australia
e) Central Siberia & Central Asia
f) China (2017)
g) China
h) Central & Eastern Europe
i) Europe
j) India & Middle East
k) India & Pakistan
l) Middle East
m) Northeast Siberia
n) Northern Africa & Middle East
o) Pacific Northwest USA & Canada
p) Russia
q) Southwestern USA & Mexico
r) Siberia
s) South America
t) Northern Southeast Asia
u) Southern Africa
v) Eastern USA
1850s
1860s
1870s
1880s
1890s
1900s
1910s
1920s
1930s
1940s
1950s
1960s
1970s
1980s
1990s
2000s
0 25 50 75 100 125
Cumulative Heat (°C)

Supplementary Figure 8: Kernel Density Estimates (KDE) across Decades for Heatwave Length (days) in Historical CMIP6 multi-model ensemble (MME) (Tx90p) - Kernel Density Estimates (KDE) of Heatwave Length (days) for different Decades in the CMIP6 multi-model ensemble (MME). Area under each curve is normalized to 1. Heatwave Length (days) of the observed event is indicated by vertical lines in dark blue (Berkeley Earth) and light blue (ERA5).

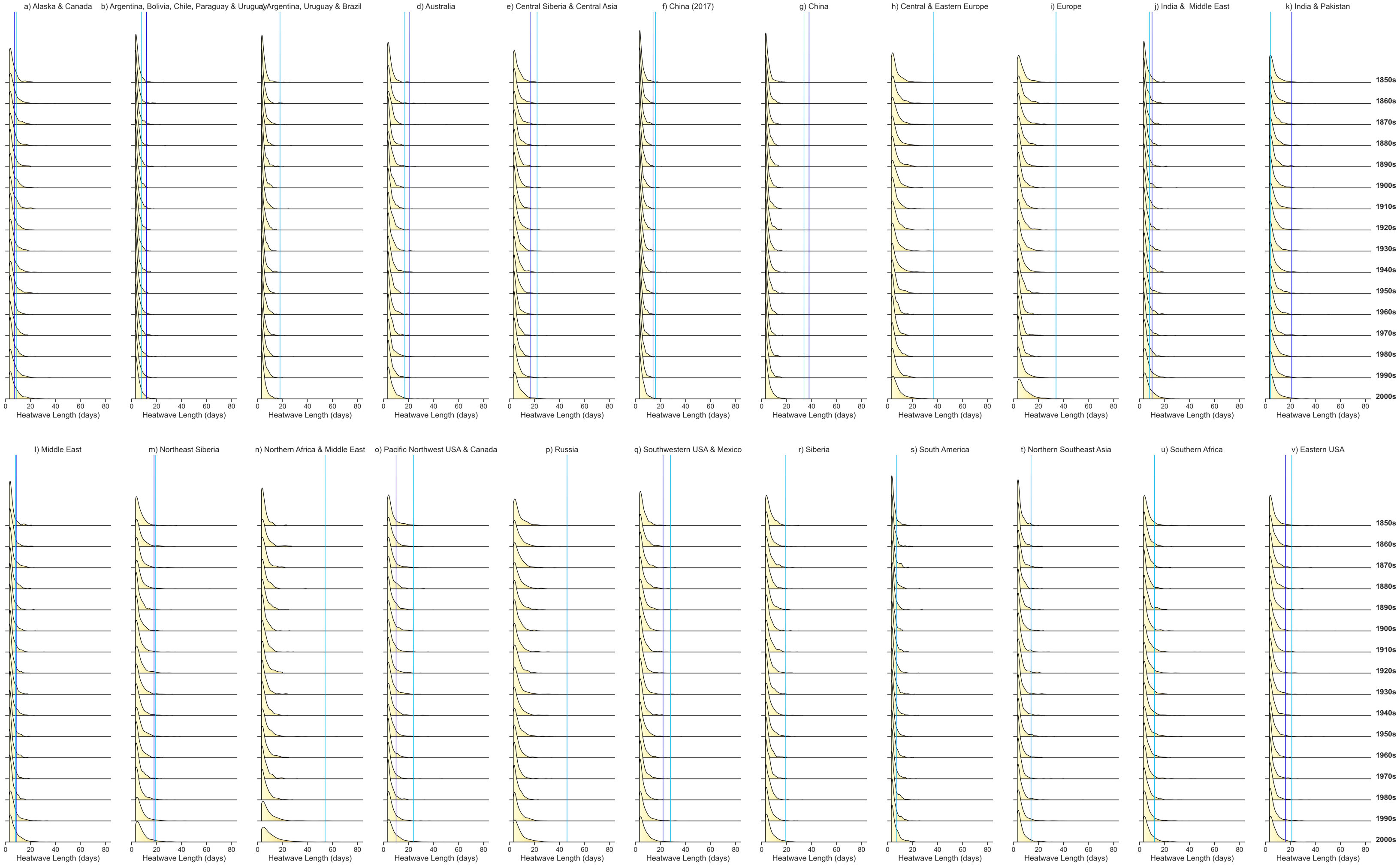

a) Alaska & Canada
b) Argentina, Bolivia, Chile, Paraguay & Urugu
c) Argentina, Uruguay & Brazil
d) Australia
e) Central Siberia & Central Asia
f) China (2017)
g) China
h) Central & Eastern Europe
i) Europe
j) India & Middle East
k) India & Pakistan
l) Middle East
m) Northeast Siberia
n) Northern Africa & Middle East
o) Pacific Northwest USA & Canada
p) Russia
q) Southwestern USA & Mexico
r) Siberia
s) South America
t) Northern Southeast Asia
u) Southern Africa
v) Eastern USA
1850s
1860s
1870s
1880s
1890s
1900s
1910s
1920s
1930s
1940s
1950s
1960s
1970s
1980s
1990s
2000s
0
20
40
60
80
Heatwave Length (days)

Supplementary Figure 9: Kernel Density Estimates (KDE) across Decades for Median Heatwave Severity in Historical CMIP6 multi-model ensemble (MME) (Tx90p) - Kernel Density Estimates (KDE) of Median Heatwave Severity for different Decades in the CMIP6 multi-model ensemble (MME). Area under each curve is normalized to 1. Median Heatwave Severity of the observed event is indicated by vertical lines in dark blue (Berkeley Earth) and light blue (ERA5).

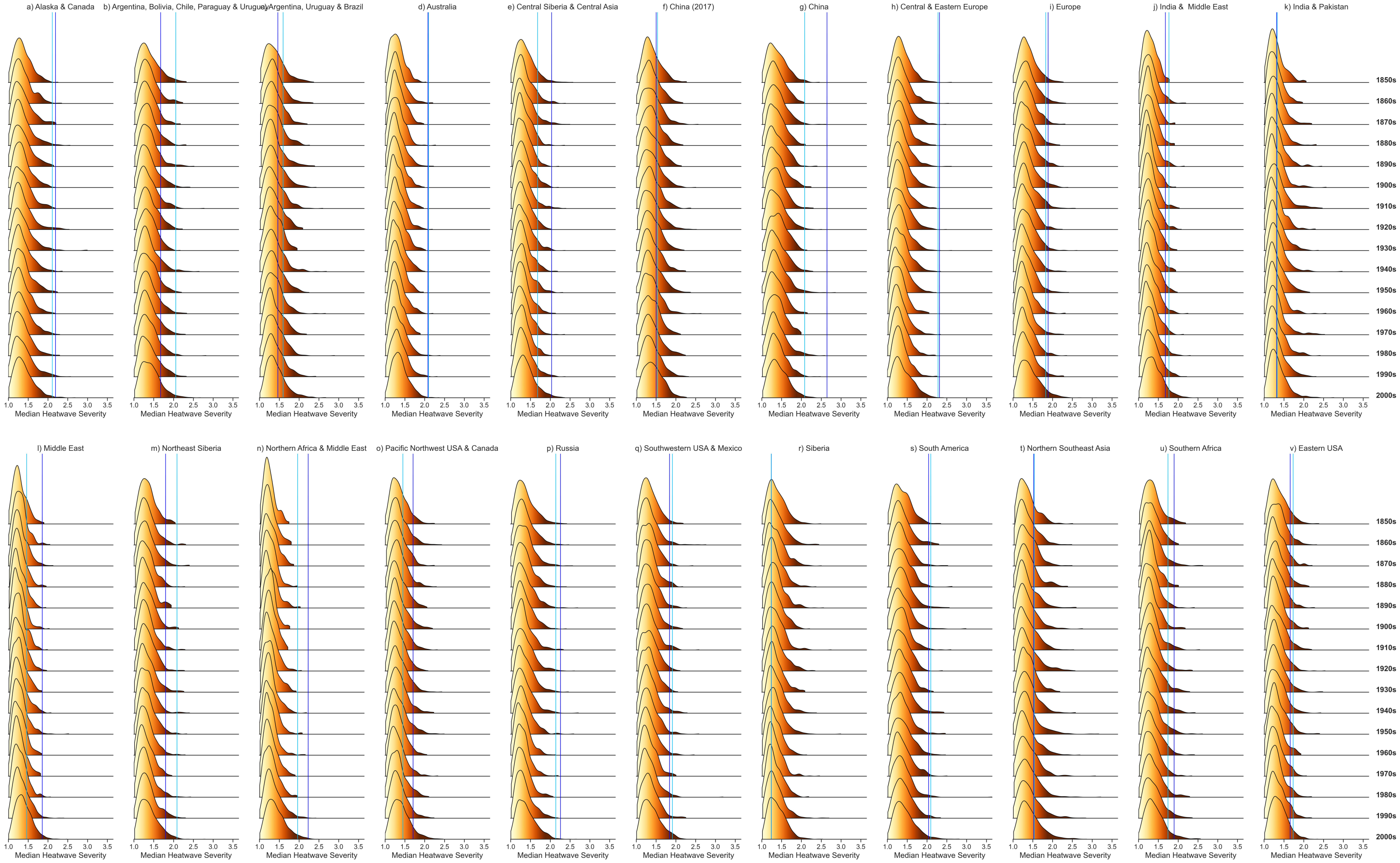
a) Alaska & Canada
b) Argentina, Bolivia, Chile, Paraguay & Uruguay
c) Argentina, Uruguay & Brazil
d) Australia
e) Central Siberia & Central Asia
f) China (2017)
g) China
h) Central & Eastern Europe
i) Europe
j) India & Middle East
k) India & Pakistan
l) Middle East
m) Northeast Siberia
n) Northern Africa & Middle East
o) Pacific Northwest USA & Canada
p) Russia
q) Southwestern USA & Mexico
r) Siberia
s) South America
t) Northern Southeast Asia
u) Southern Africa
v) Eastern USA
1850s
1860s
1870s
1880s
1890s
1900s
1910s
1920s
1930s
1940s
1950s
1960s
1970s
1980s
1990s
2000s
1.0 1.5 2.0 2.5 3.0 3.5
Median Heatwave Severity

Supplementary Figure 10: Kernel Density Estimates (KDE) across Decades for Maximum Heatwave Severity in Historical CMIP6 multi-model ensemble (MME) (Tx90p) - Kernel Density Estimates (KDE) of Maximum Heatwave Severity for different Decades in the CMIP6 multi-model ensemble (MME). Area under each curve is normalized to 1. Maximum Heatwave Severity of the observed event is indicated by vertical lines in dark blue (Berkeley Earth) and light blue (ERA5).

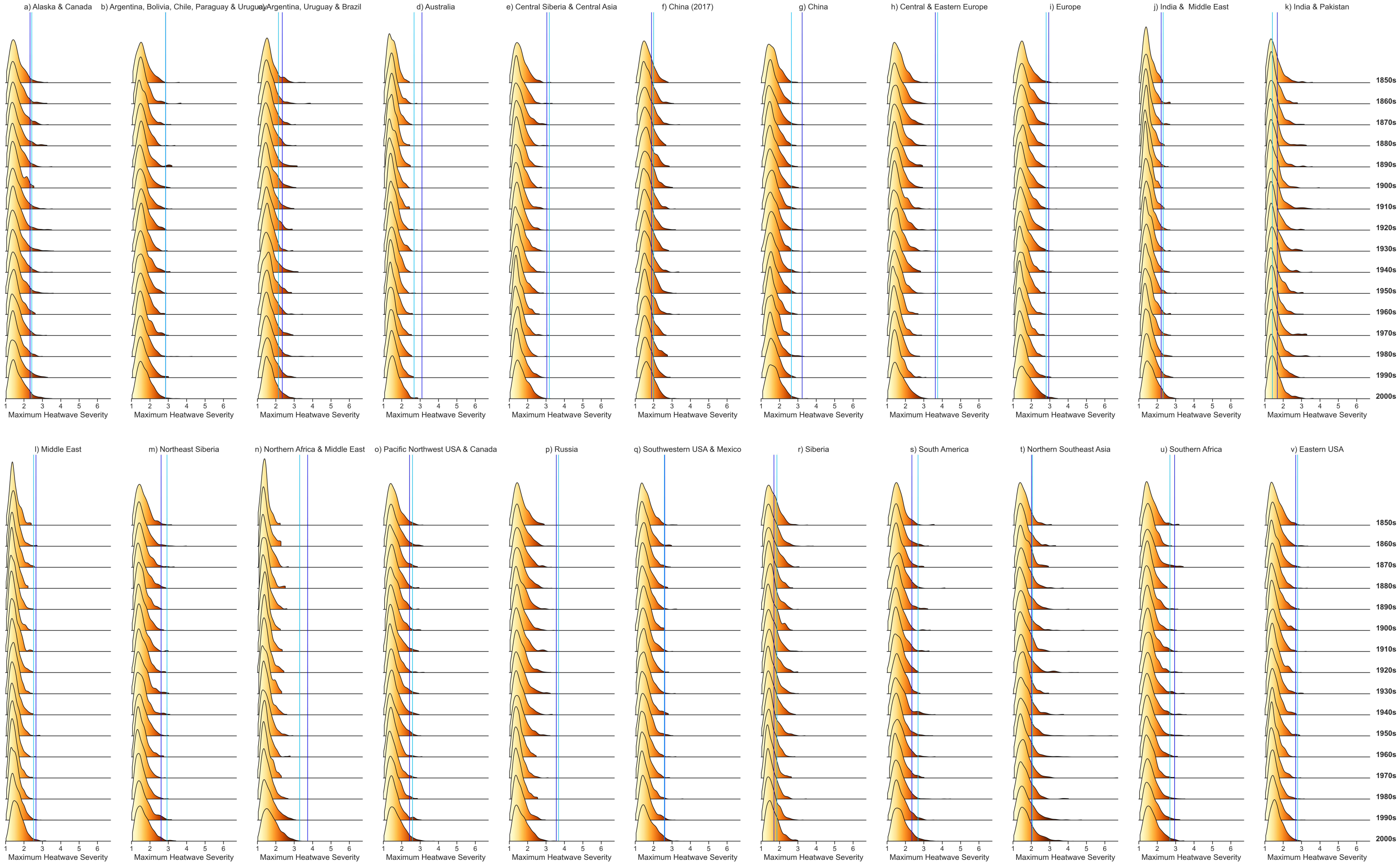

a) Alaska & Canada
b) Argentina, Bolivia, Chile, Paraguay & Uruguay
c) Argentina, Uruguay & Brazil
d) Australia
e) Central Siberia & Central Asia
f) China (2017)
g) China
h) Central & Eastern Europe
i) Europe
j) India & Middle East
k) India & Pakistan
l) Middle East
m) Northeast Siberia
n) Northern Africa & Middle East
o) Pacific Northwest USA & Canada
p) Russia
q) Southwestern USA & Mexico
r) Siberia
s) South America
t) Northern Southeast Asia
u) Southern Africa
v) Eastern USA
1850s
1860s
1870s
1880s
1890s
1900s
1910s
1920s
1930s
1940s
1950s
1960s
1970s
1980s
1990s
2000s
1 2 3 4 5 6
Maximum Heatwave Severity

Supplementary Figure 11: Kernel Density Estimates (KDE) across Decades for Median Heatwave Area (%) in Historical CMIP6 multi-model ensemble (MME) (Tx90p) - Kernel Density Estimates (KDE) of Median Heatwave Area (%) for different Decades in the CMIP6 multi-model ensemble (MME). Area under each curve is normalized to 1. Median Heatwave Area (%) of the observed event is indicated by vertical lines in dark blue (Berkeley Earth) and light blue (ERA5).

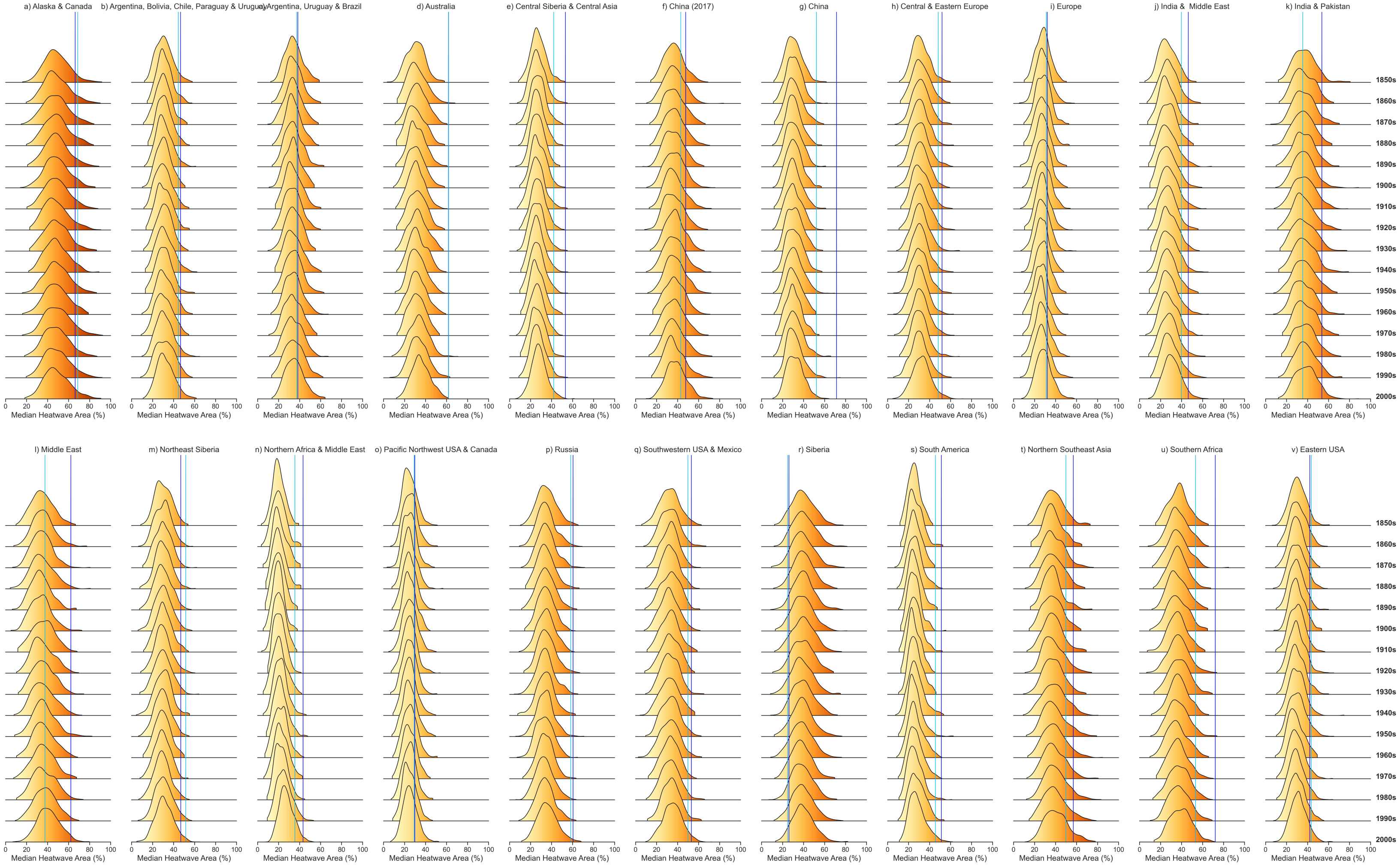

a) Alaska & Canada
b) Argentina, Bolivia, Chile, Paraguay & Uruguay
c) Argentina, Uruguay & Brazil
d) Australia
e) Central Siberia & Central Asia
f) China (2017)
g) China
h) Central & Eastern Europe
i) Europe
j) India & Middle East
k) India & Pakistan
l) Middle East
m) Northeast Siberia
n) Northern Africa & Middle East
o) Pacific Northwest USA & Canada
p) Russia
q) Southwestern USA & Mexico
r) Siberia
s) South America
t) Northern Southeast Asia
u) Southern Africa
v) Eastern USA
1850s
1860s
1870s
1880s
1890s
1900s
1910s
1920s
1930s
1940s
1950s
1960s
1970s
1980s
1990s
2000s
0 20 40 60 80 100
Median Heatwave Area (%)

Supplementary Figure 12: Kernel Density Estimates (KDE) across Decades for Maximum Heatwave Area (%) in Historical CMIP6 multi-model ensemble (MME) (Tx90p) - Kernel Density Estimates (KDE) of Maximum Heatwave Area (%) for different Decades in the CMIP6 multi-model ensemble (MME). Area under each curve is normalized to 1. Maximum Heatwave Area (%) of the observed event is indicated by vertical lines in dark blue (Berkeley Earth) and light blue (ERA5).

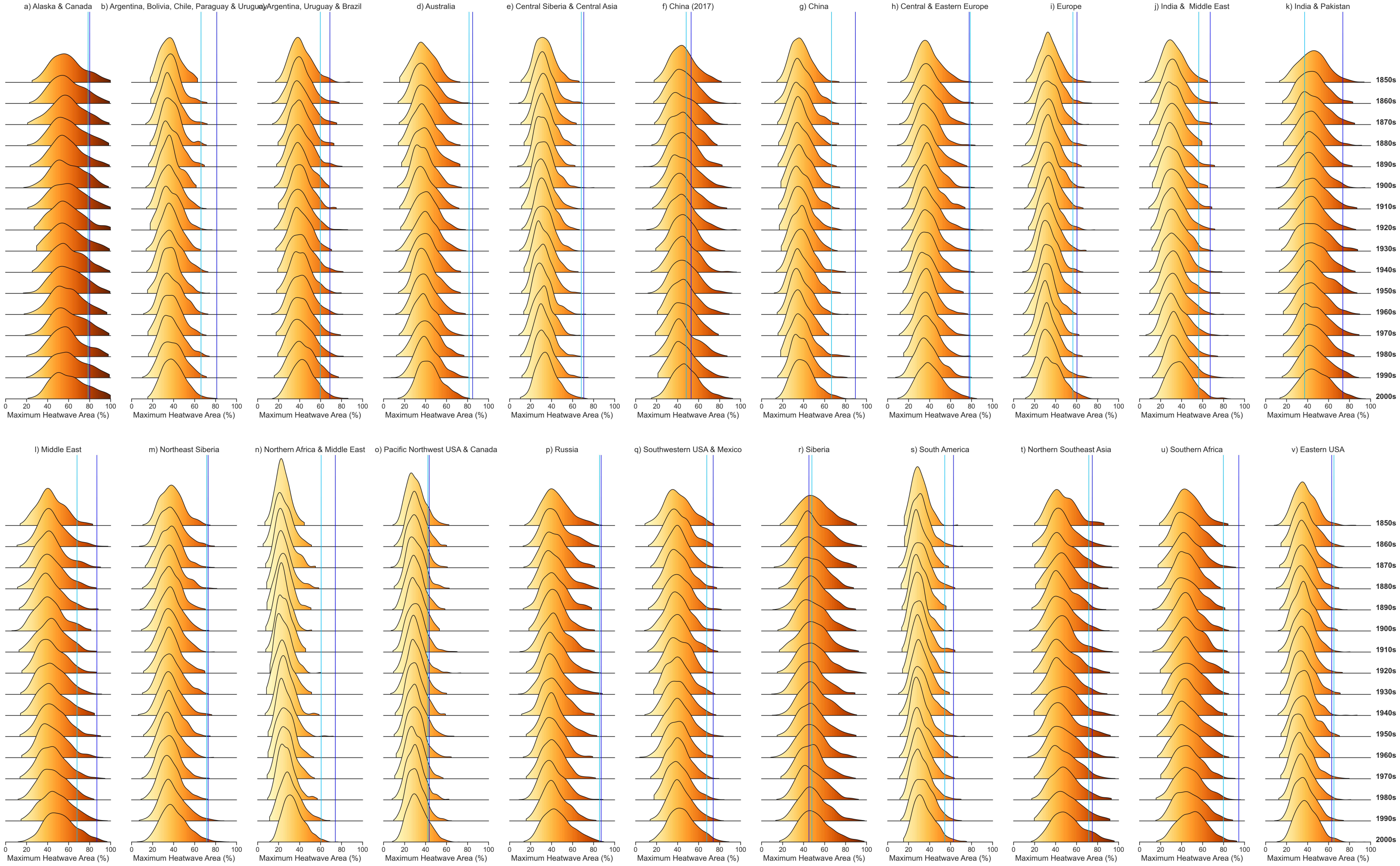

a) Alaska & Canada
b) Argentina, Bolivia, Chile, Paraguay & Uruguay
c) Argentina, Uruguay & Brazil
d) Australia
e) Central Siberia & Central Asia
f) China (2017)
g) China
h) Central & Eastern Europe
i) Europe
j) India & Middle East
k) India & Pakistan
l) Middle East
m) Northeast Siberia
n) Northern Africa & Middle East
o) Pacific Northwest USA & Canada
p) Russia
q) Southwestern USA & Mexico
r) Siberia
s) South America
t) Northern Southeast Asia
u) Southern Africa
v) Eastern USA
1850s
1860s
1870s
1880s
1890s
1900s
1910s
1920s
1930s
1940s
1950s
1960s
1970s
1980s
1990s
2000s
0 20 40 60 80 100
Maximum Heatwave Area (%)

Supplementary Figure 13: Kernel Density Estimates (KDE) across Decades for Cumulative Heat (°C) in Historical CMIP5 multi-model ensemble (MME) (Tx90p) - Kernel Density Estimates (KDE) of Cumulative Heat (°C) for different Decades in the CMIP5 multi-model ensemble (MME). Area under each curve is normalized to 1. Cumulative Heat (°C) of the observed event is indicated by vertical lines in dark blue (Berkeley Earth) and light blue (ERA5).

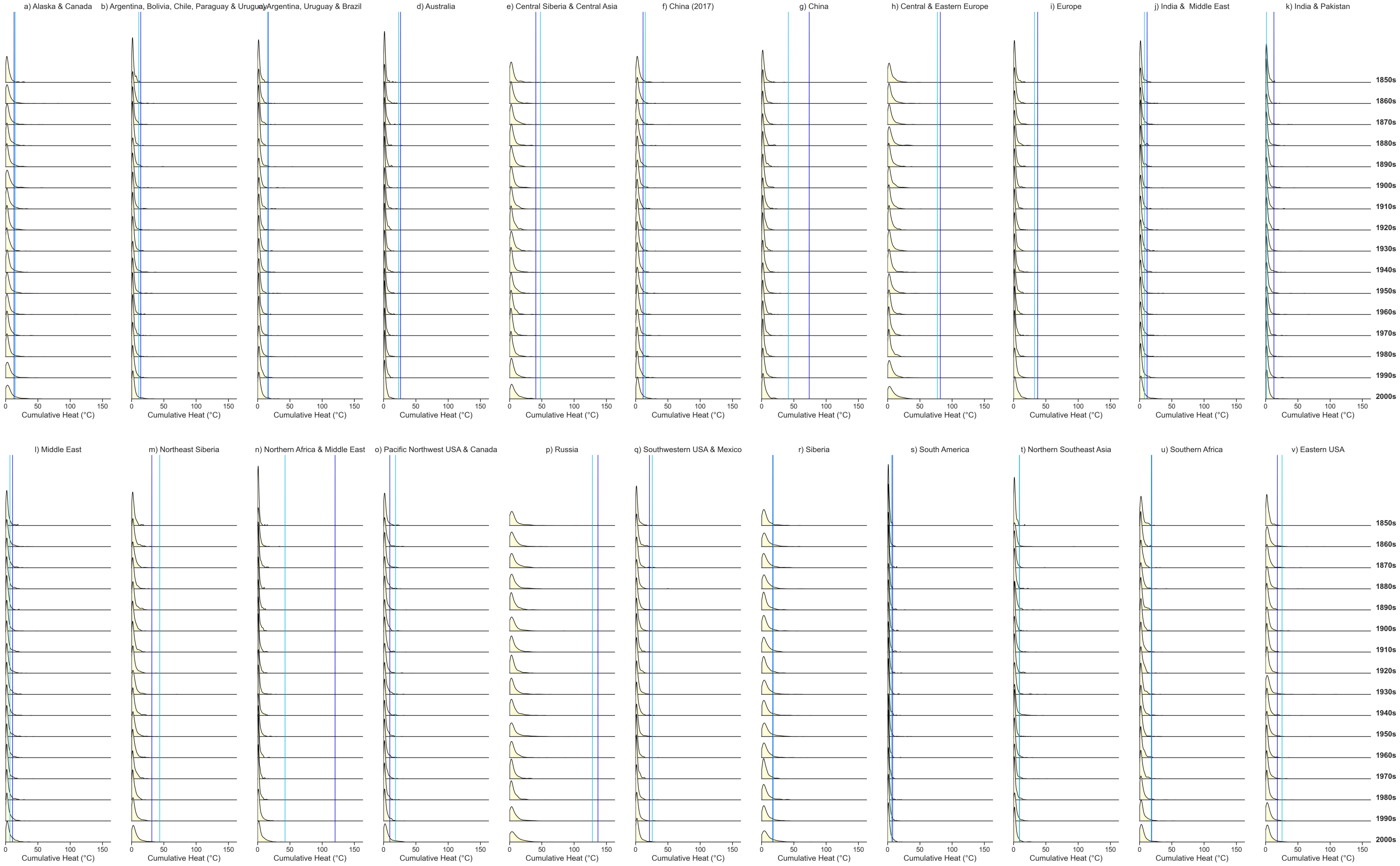

a) Alaska & Canada
b) Argentina, Bolivia, Chile, Paraguay & Uruguay
c) Argentina, Uruguay & Brazil
d) Australia
e) Central Siberia & Central Asia
f) China (2017)
g) China
h) Central & Eastern Europe
i) Europe
j) India & Middle East
k) India & Pakistan
l) Middle East
m) Northeast Siberia
n) Northern Africa & Middle East
o) Pacific Northwest USA & Canada
p) Russia
q) Southwestern USA & Mexico
r) Siberia
s) South America
t) Northern Southeast Asia
u) Southern Africa
v) Eastern USA
1850s
1860s
1870s
1880s
1890s
1900s
1910s
1920s
1930s
1940s
1950s
1960s
1970s
1980s
1990s
2000s
0
50
100
150
Cumulative Heat (°C)

Supplementary Figure 14: Kernel Density Estimates (KDE) across Decades for Heatwave Length (days) in Historical CMIP5 multi-model ensemble (MME) (Tx90p) - Kernel Density Estimates (KDE) of Heatwave Length (days) for different Decades in the CMIP5 multi-model ensemble (MME). Area under each curve is normalized to 1. Heatwave Length (days) of the observed event is indicated by vertical lines in dark blue (Berkeley Earth) and light blue (ERA5).

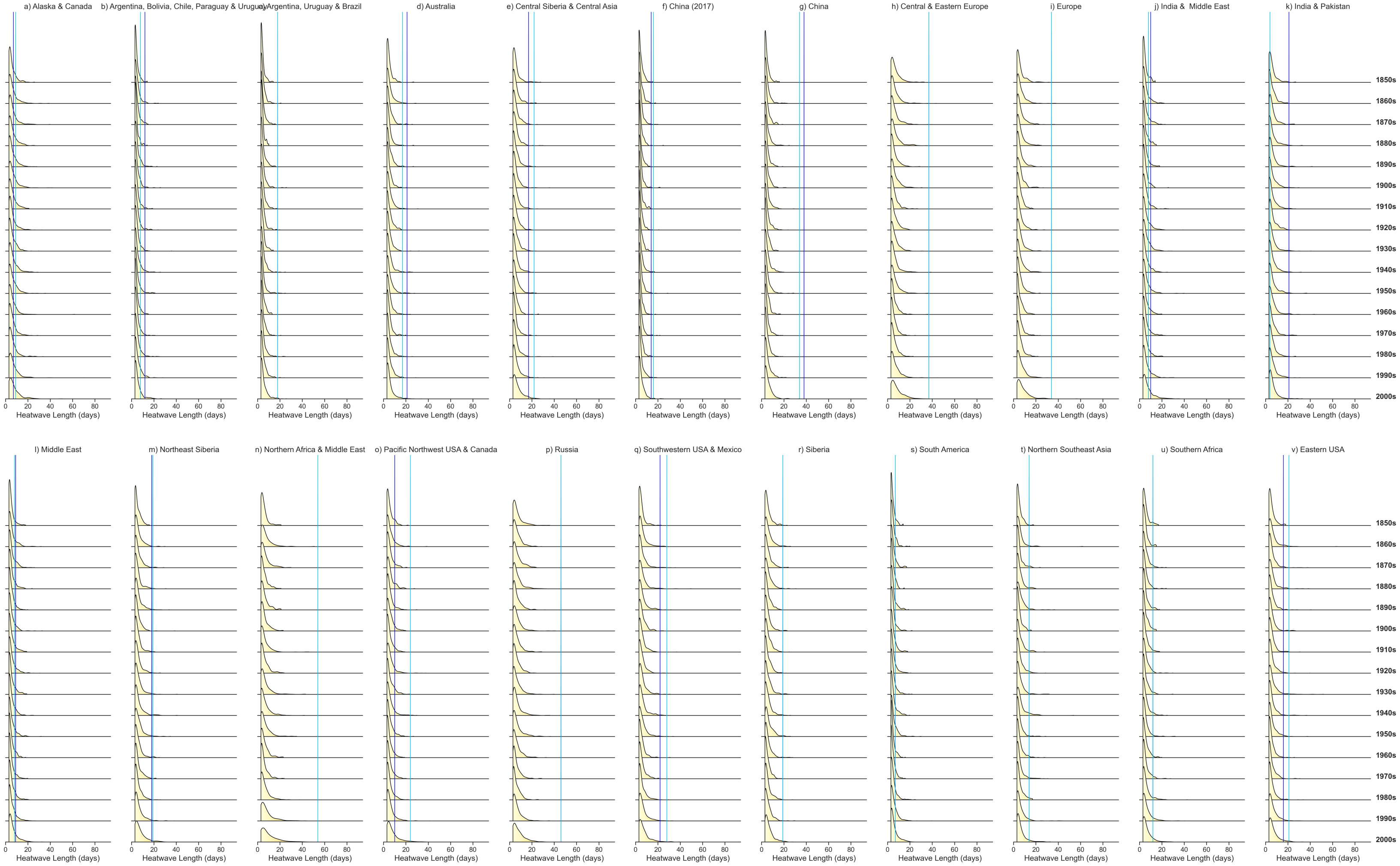

a) Alaska & Canada
b) Argentina, Bolivia, Chile, Paraguay & Uruguay
c) Argentina, Uruguay & Brazil
d) Australia
e) Central Siberia & Central Asia
f) China (2017)
g) China
h) Central & Eastern Europe
i) Europe
j) India & Middle East
k) India & Pakistan
l) Middle East
m) Northeast Siberia
n) Northern Africa & Middle East
o) Pacific Northwest USA & Canada
p) Russia
q) Southwestern USA & Mexico
r) Siberia
s) South America
t) Northern Southeast Asia
u) Southern Africa
v) Eastern USA
1850s
1860s
1870s
1880s
1890s
1900s
1910s
1920s
1930s
1940s
1950s
1960s
1970s
1980s
1990s
2000s
0 20 40 60 80
Heatwave Length (days)

Supplementary Figure 15: Kernel Density Estimates (KDE) across Decades for Median Heatwave Severity in Historical CMIP5 multi-model ensemble (MME) (Tx90p) - Kernel Density Estimates (KDE) of Median Heatwave Severity for different Decades in the CMIP5 multi-model ensemble (MME). Area under each curve is normalized to 1. Median Heatwave Severity of the observed event is indicated by vertical lines in dark blue (Berkeley Earth) and light blue (ERA5).

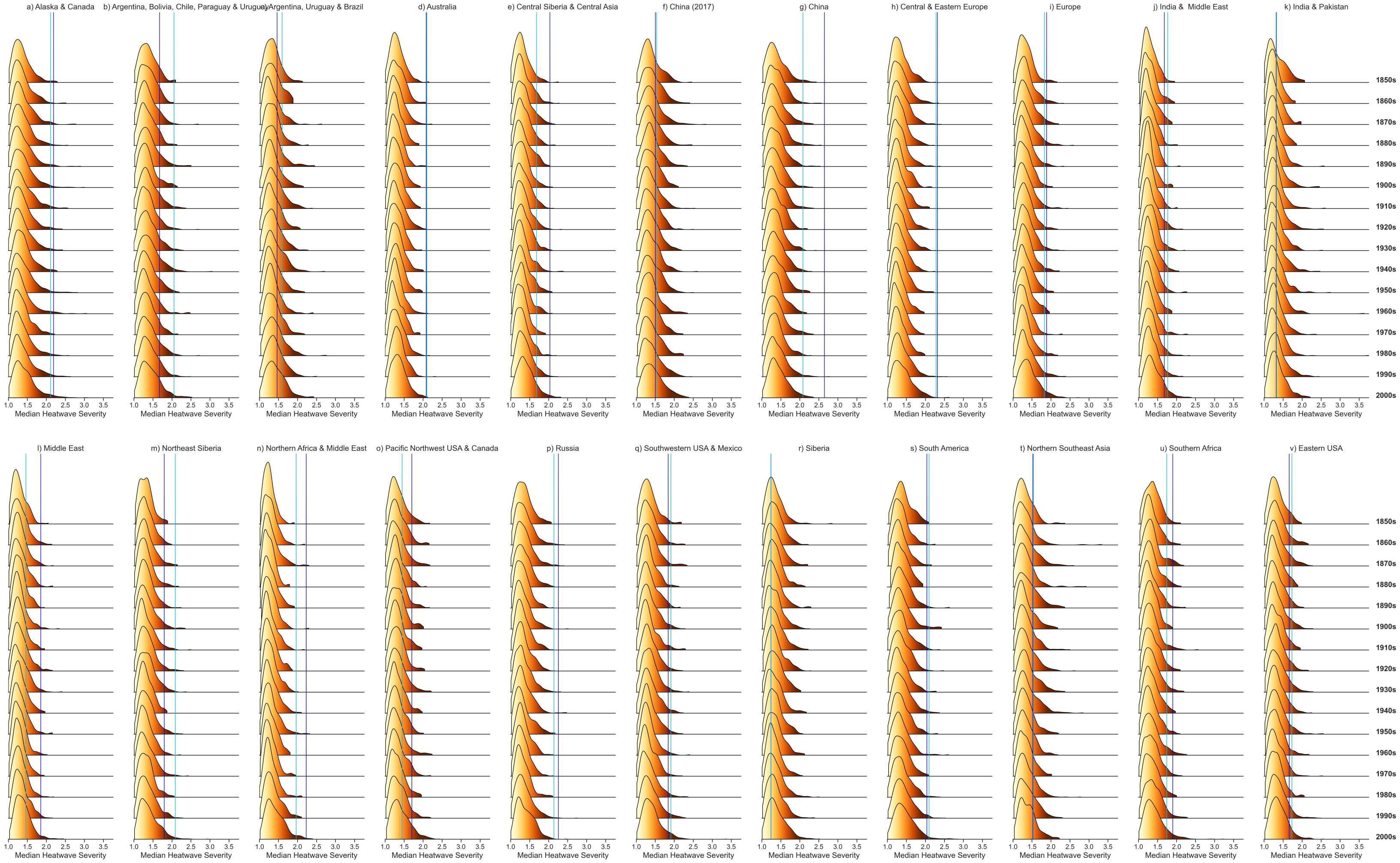

a) Alaska & Canada
b) Argentina, Bolivia, Chile, Paraguay & Uruguay
c) Argentina, Uruguay & Brazil
d) Australia
e) Central Siberia & Central Asia
f) China (2017)
g) China
h) Central & Eastern Europe
i) Europe
j) India & Middle East
k) India & Pakistan
l) Middle East
m) Northeast Siberia
n) Northern Africa & Middle East
o) Pacific Northwest USA & Canada
p) Russia
q) Southwestern USA & Mexico
r) Siberia
s) South America
t) Northern Southeast Asia
u) Southern Africa
v) Eastern USA
1850s
1860s
1870s
1880s
1890s
1900s
1910s
1920s
1930s
1940s
1950s
1960s
1970s
1980s
1990s
2000s
1.0 1.5 2.0 2.5 3.0 3.5
Median Heatwave Severity

Supplementary Figure 16: Kernel Density Estimates (KDE) across Decades for Maximum Heatwave Severity in Historical CMIP5 multi-model ensemble (MME) (Tx90p) - Kernel Density Estimates (KDE) of Maximum Heatwave Severity for different Decades in the CMIP5 multi-model ensemble (MME). Area under each curve is normalized to 1. Maximum Heatwave Severity of the observed event is indicated by vertical lines in dark blue (Berkeley Earth) and light blue (ERA5).

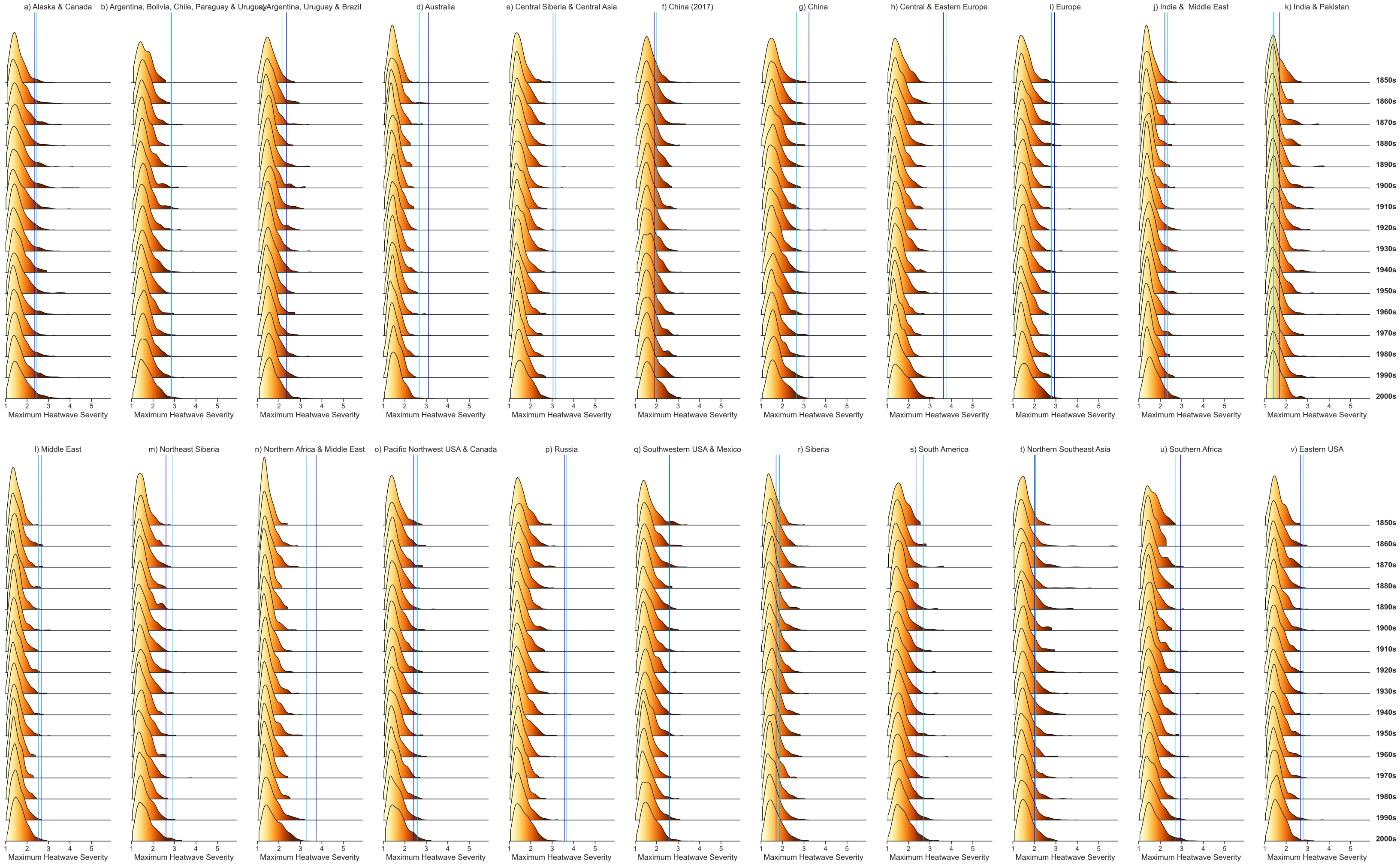
a) Alaska & Canada
b) Argentina, Bolivia, Chile, Paraguay & Uruguay
c) Argentina, Uruguay & Brazil
d) Australia
e) Central Siberia & Central Asia
f) China (2017)
g) China
h) Central & Eastern Europe
i) Europe
j) India & Middle East
k) India & Pakistan
l) Middle East
m) Northeast Siberia
n) Northern Africa & Middle East
o) Pacific Northwest USA & Canada
p) Russia
q) Southwestern USA & Mexico
r) Siberia
s) South America
t) Northern Southeast Asia
u) Southern Africa
v) Eastern USA
1850s
1860s
1870s
1880s
1890s
1900s
1910s
1920s
1930s
1940s
1950s
1960s
1970s
1980s
1990s
2000s
1 2 3 4 5
Maximum Heatwave Severity

Supplementary Figure 17: Kernel Density Estimates (KDE) across Decades for Median Heatwave Area (%) in Historical CMIP5 multi-model ensemble (MME) (Tx90p) - Kernel Density Estimates (KDE) of Median Heatwave Area (%) for different Decades in the CMIP5 multi-model ensemble (MME). Area under each curve is normalized to 1. Median Heatwave Area (%) of the observed event is indicated by vertical lines in dark blue (Berkeley Earth) and light blue (ERA5).

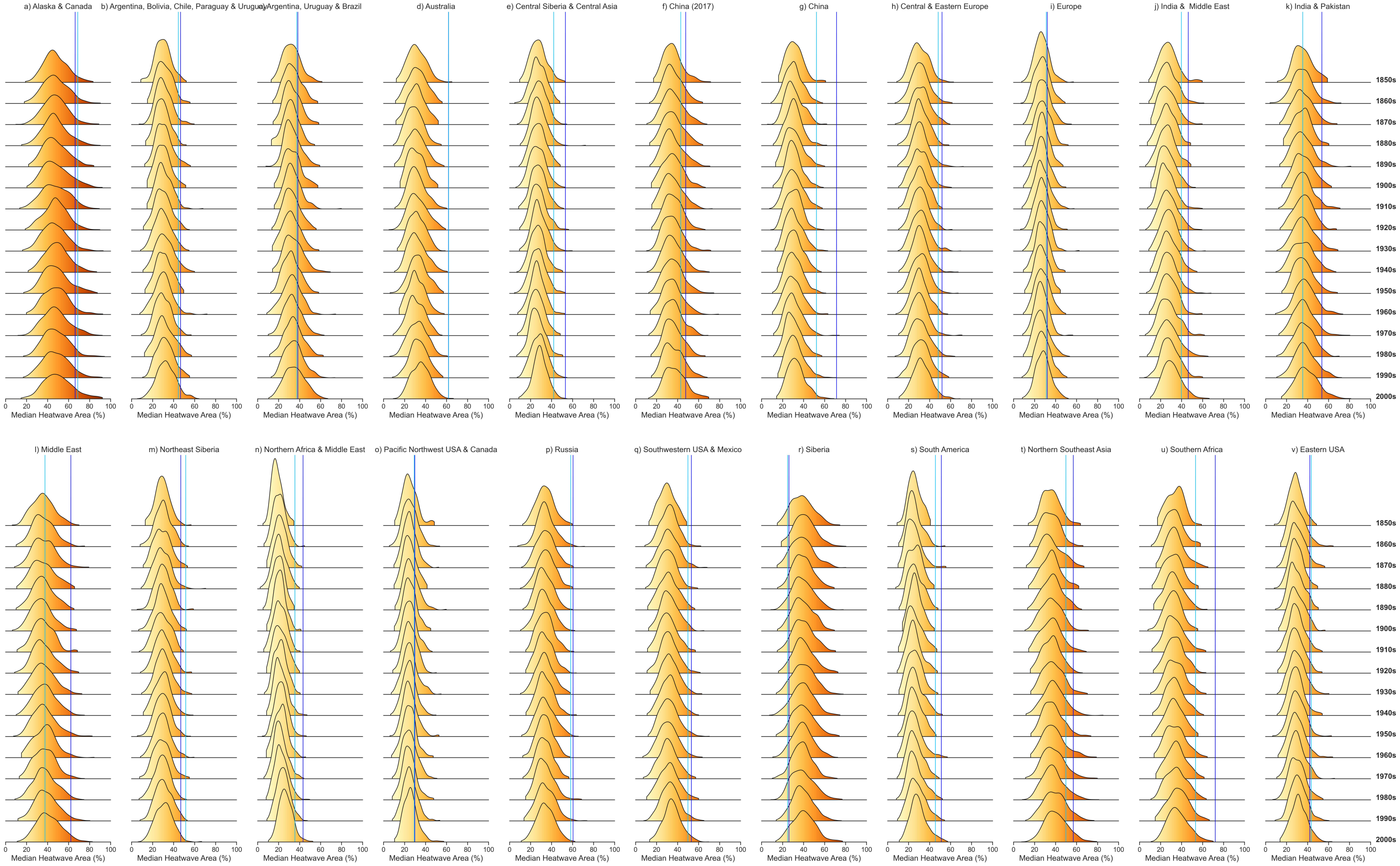

a) Alaska & Canada
b) Argentina, Bolivia, Chile, Paraguay & Uruguay
c) Argentina, Uruguay & Brazil
d) Australia
e) Central Siberia & Central Asia
f) China (2017)
g) China
h) Central & Eastern Europe
i) Europe
j) India & Middle East
k) India & Pakistan
l) Middle East
m) Northeast Siberia
n) Northern Africa & Middle East
o) Pacific Northwest USA & Canada
p) Russia
q) Southwestern USA & Mexico
r) Siberia
s) South America
t) Northern Southeast Asia
u) Southern Africa
v) Eastern USA
1850s
1860s
1870s
1880s
1890s
1900s
1910s
1920s
1930s
1940s
1950s
1960s
1970s
1980s
1990s
2000s
0 20 40 60 80 100
Median Heatwave Area (%)

Supplementary Figure 18: Kernel Density Estimates (KDE) across Decades for Maximum Heatwave Area (%) in Historical CMIP5 multi-model ensemble (MME) (Tx90p) - Kernel Density Estimates (KDE) of Maximum Heatwave Area (%) for different Decades in the CMIP5 multi-model ensemble (MME). Area under each curve is normalized to 1. Maximum Heatwave Area (%) of the observed event is indicated by vertical lines in dark blue (Berkeley Earth) and light blue (ERA5).

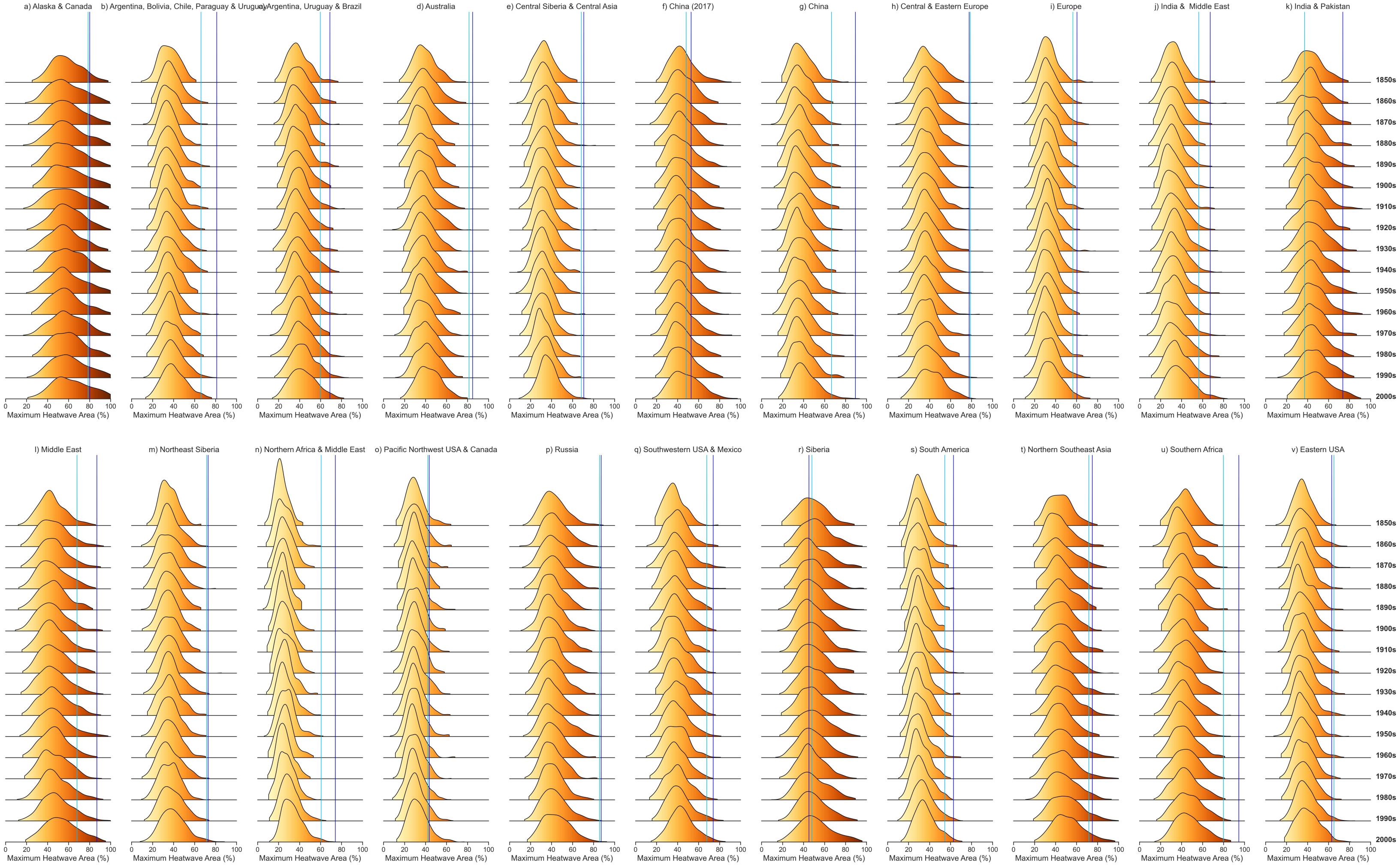
a) Alaska & Canada
b) Argentina, Bolivia, Chile, Paraguay & Uruguay
c) Argentina, Uruguay & Brazil
d) Australia
e) Central Siberia & Central Asia
f) China (2017)
g) China
h) Central & Eastern Europe
i) Europe
j) India & Middle East
k) India & Pakistan
l) Middle East
m) Northeast Siberia
n) Northern Africa & Middle East
o) Pacific Northwest USA & Canada
p) Russia
q) Southwestern USA & Mexico
r) Siberia
s) South America
t) Northern Southeast Asia
u) Southern Africa
v) Eastern USA
1850s
1860s
1870s
1880s
1890s
1900s
1910s
1920s
1930s
1940s
1950s
1960s
1970s
1980s
1990s
2000s
0 20 40 60 80 100
Maximum Heatwave Area (%)

Supplementary Figure 19: Multi-HW-characteristic figure according to SSP1-2.6 CMIP6 multi-model ensemble (MME) (Tx90p) - Each panel shows regional projections according to SSP1-2.6 model runs in the CMIP6 multi-model ensemble (MME) of four combined heatwave characteristics for each projected heatwave in the ensemble between 2020 and 2100. Each dot represents an individual heatwave in the projections, characterized by Median Heatwave Area (%) (x-axis) as latitude-weighted percentage of grid boxes affected by extreme heat, Heatwave Length (days) (y-axis) in days, Median Heatwave Severity (color), and Cumulative Heat (°C) (dot size). The characteristics of the observed event according to the Berkeley Earth observational data (x symbol) and the ERA5 reanalysis data (+ symbol), respectively, are pointed to by an arrow and a figure inset shows a zoom-in on this figure section. The figure inset shows the observed heatwave in the two reference data sets and projections in the indicated section of the respective sub-plot. It facilitates readability of color and size of the reference values and comparison to each other and the neighboring projections. Note that the zoom-in causes the dots to overlap more in the inset than in the main sub-plot. The univariate kernel density estimates along the x- (Median Heatwave Area (%)) and y-axis (Heatwave Length (days)) are on the top and right side of the subplots, respectively, with vertical lines in dark blue (Berkeley Earth) and light blue (ERA5) indicating the observed event.

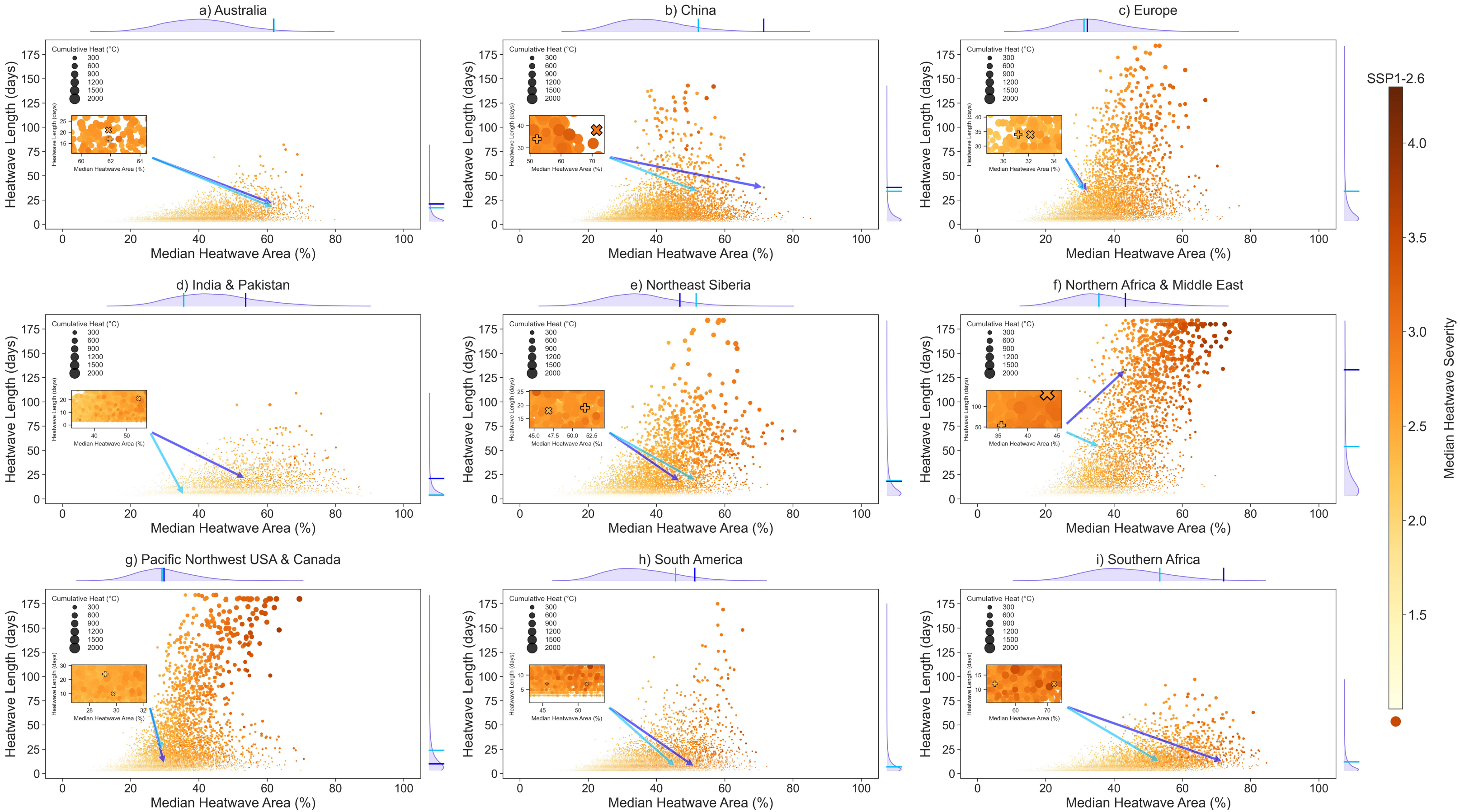

a) Australia
b) China
c) Europe
d) India & Pakistan
e) Northeast Siberia
f) Northern Africa & Middle East
g) Pacific Northwest USA & Canada
h) South America
i) Southern Africa
Cumulative Heat (°C)
300
600
900
1200
1500
2000
Heatwave Length (days)
Median Heatwave Area (%)
SSP1-2.6
Median Heatwave Severity

Supplementary Figure 20: Multi-HW-characteristic figure according to SSP2-4.5 CMIP6 multi-model ensemble (MME) (Tx90p) - Each panel shows regional projections according to SSP2-4.5 model runs in the CMIP6 multi-model ensemble (MME) of four combined heatwave characteristics for each projected heatwave in the ensemble between 2020 and 2100. Each dot represents an individual heatwave in the projections, characterized by Median Heatwave Area (%) (x-axis) as latitude-weighted percentage of grid boxes affected by extreme heat, Heatwave Length (days) (y-axis) in days, Median Heatwave Severity (color), and Cumulative Heat (°C) (dot size). The characteristics of the observed event according to the Berkeley Earth observational data (x symbol) and the ERA5 reanalysis data (+ symbol), respectively, are pointed to by an arrow and a figure inset shows a zoom-in on this figure section. The figure inset shows the observed heatwave in the two reference data sets and projections in the indicated section of the respective sub-plot. It facilitates readability of color and size of the reference values and comparison to each other and the neighboring projections. Note that the zoom-in causes the dots to overlap more in the inset than in the main sub-plot. The univariate kernel density estimates along the x- (Median Heatwave Area (%)) and y-axis (Heatwave Length (days)) are on the top and right side of the subplots, respectively, with vertical lines in dark blue (Berkeley Earth) and light blue (ERA5) indicating the observed event.

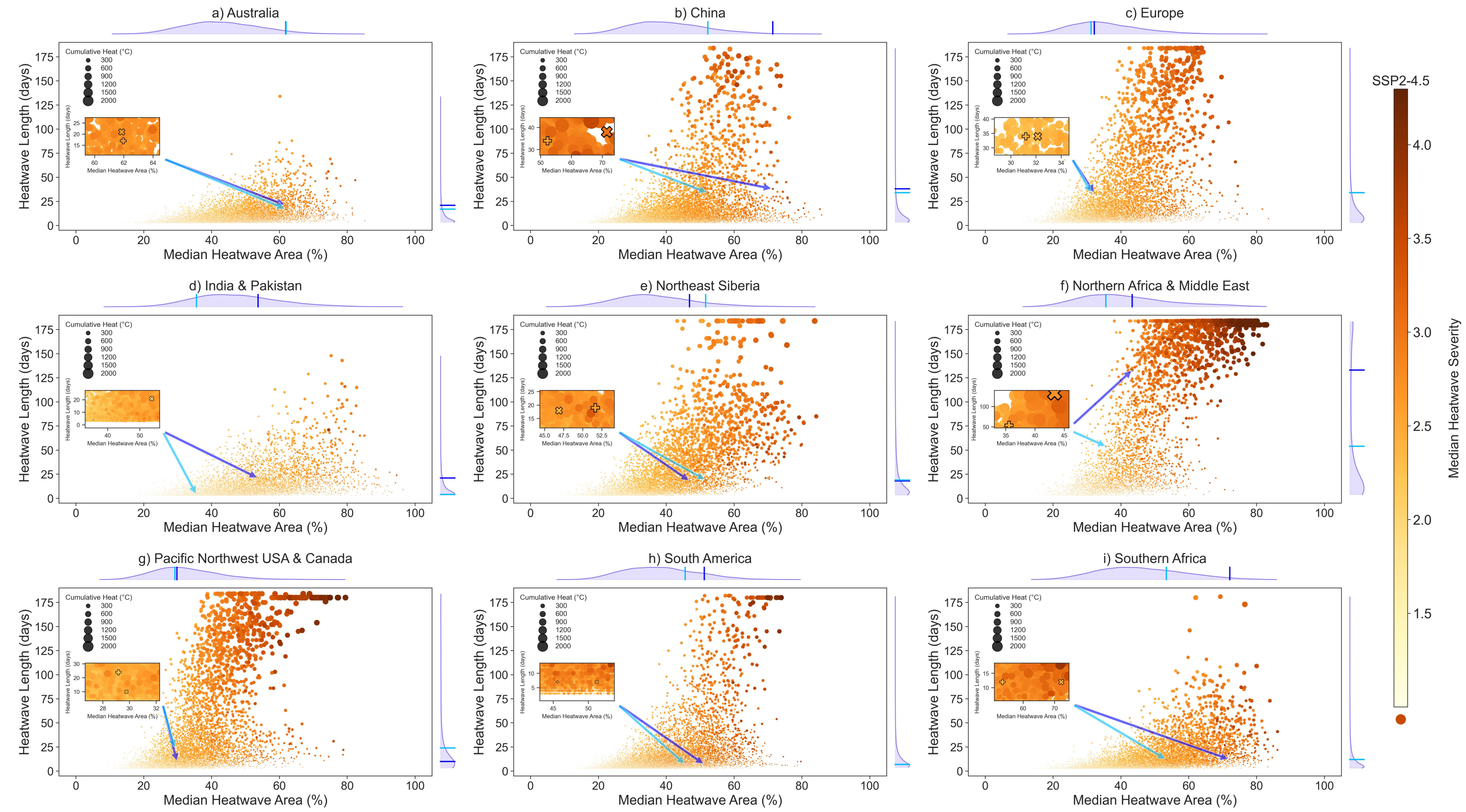

a) Australia
b) China
c) Europe
d) India & Pakistan
e) Northeast Siberia
f) Northern Africa & Middle East
g) Pacific Northwest USA & Canada
h) South America
i) Southern Africa
Heatwave Length (days)
Median Heatwave Area (%)
Cumulative Heat (°C)
300
600
900
1200
1500
2000
SSP2-4.5
Median Heatwave Severity

Supplementary Figure 21: Multi-HW-characteristic figure according to SSP3-7.0 CMIP6 multi-model ensemble (MME) (Tx90p) - Each panel shows regional projections according to SSP3-7.0 model runs in the CMIP6 multi-model ensemble (MME) of four combined heatwave characteristics for each projected heatwave in the ensemble between 2020 and 2100. Each dot represents an individual heatwave in the projections, characterized by Median Heatwave Area (%) (x-axis) as latitude-weighted percentage of grid boxes affected by extreme heat, Heatwave Length (days) (y-axis) in days, Median Heatwave Severity (color), and Cumulative Heat (°C) (dot size). The characteristics of the observed event according to the Berkeley Earth observational data (x symbol) and the ERA5 reanalysis data (+ symbol), respectively, are pointed to by an arrow and a figure inset shows a zoom-in on this figure section. The figure inset shows the observed heatwave in the two reference data sets and projections in the indicated section of the respective sub-plot. It facilitates readability of color and size of the reference values and comparison to each other and the neighboring projections. Note that the zoom-in causes the dots to overlap more in the inset than in the main sub-plot. The univariate kernel density estimates along the x- (Median Heatwave Area (%)) and y-axis (Heatwave Length (days)) are on the top and right side of the subplots, respectively, with vertical lines in dark blue (Berkeley Earth) and light blue (ERA5) indicating the observed event.

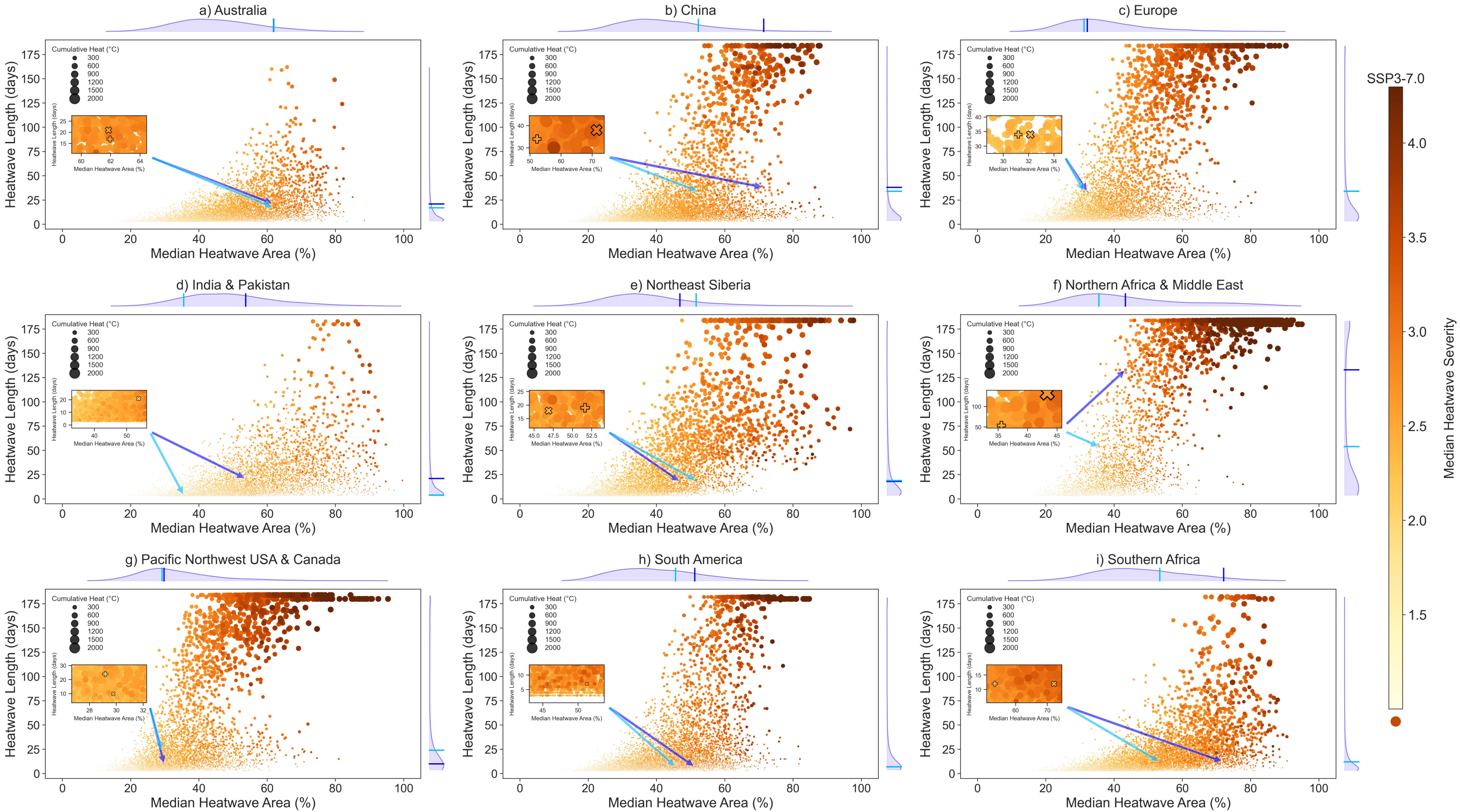

a) Australia
b) China
c) Europe
d) India & Pakistan
e) Northeast Siberia
f) Northern Africa & Middle East
g) Pacific Northwest USA & Canada
h) South America
i) Southern Africa
Cumulative Heat (°C)
300
600
900
1200
1500
2000
Heatwave Length (days)
Median Heatwave Area (%)
SSP3-7.0
Median Heatwave Severity

Supplementary Figure 22: Multi-HW-characteristic figure according to SSP5-8.5 CMIP6 multi-model ensemble (MME) (Tx90p) - Each panel shows regional projections according to SSP5-8.5 model runs in the CMIP6 multi-model ensemble (MME) of four combined heatwave characteristics for each projected heatwave in the ensemble between 2020 and 2100. Each dot represents an individual heatwave in the projections, characterized by Median Heatwave Area (%) (x-axis) as latitude-weighted percentage of grid boxes affected by extreme heat, Heatwave Length (days) (y-axis) in days, Median Heatwave Severity (color), and Cumulative Heat (°C) (dot size). The characteristics of the observed event according to the Berkeley Earth observational data (x symbol) and the ERA5 reanalysis data (+ symbol), respectively, are pointed to by an arrow and a figure inset shows a zoom-in on this figure section. The figure inset shows the observed heatwave in the two reference data sets and projections in the indicated section of the respective sub-plot. It facilitates readability of color and size of the reference values and comparison to each other and the neighboring projections. Note that the zoom-in causes the dots to overlap more in the inset than in the main sub-plot. The univariate kernel density estimates along the x- (Median Heatwave Area (%)) and y-axis (Heatwave Length (days)) are on the top and right side of the subplots, respectively, with vertical lines in dark blue (Berkeley Earth) and light blue (ERA5) indicating the observed event.

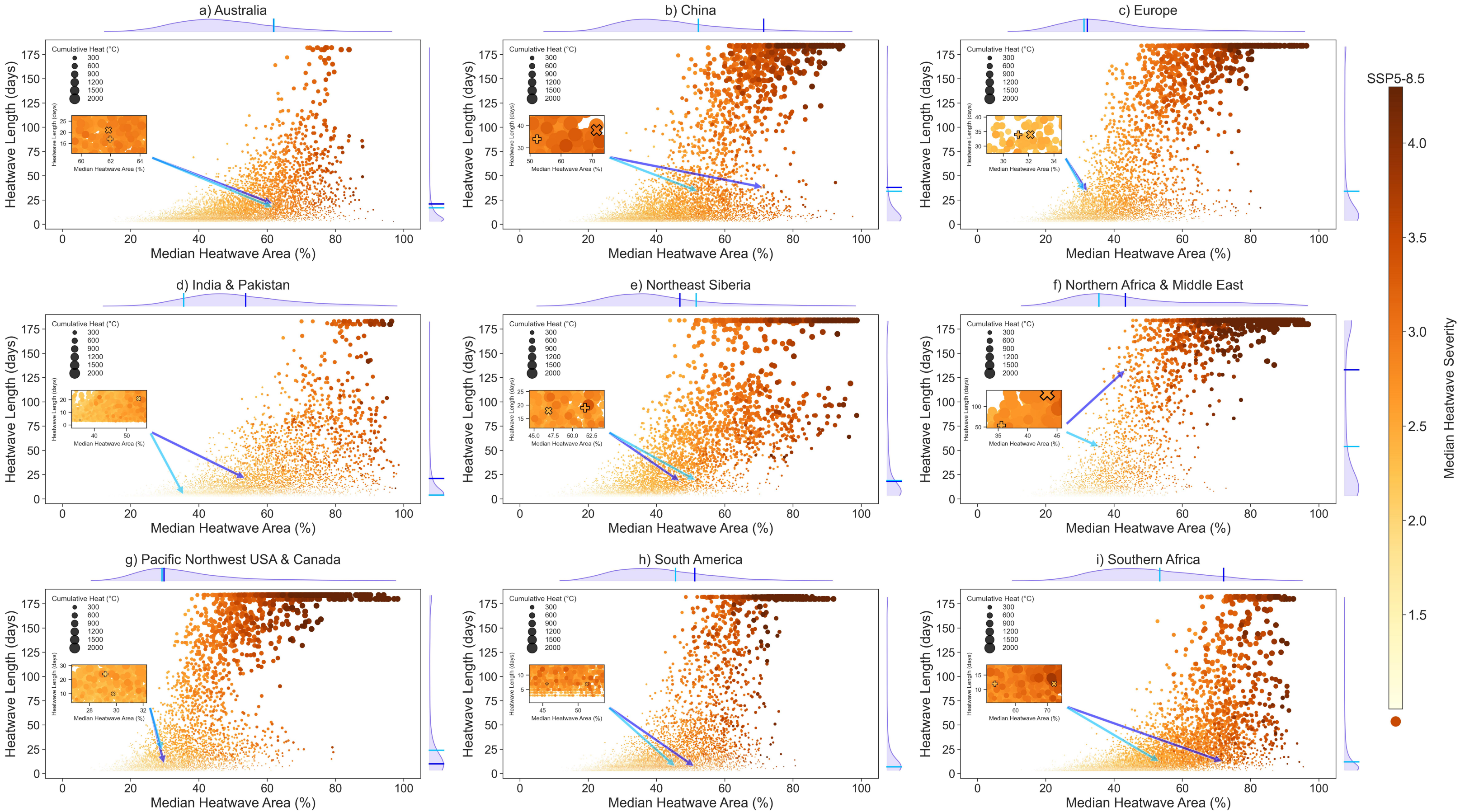
a) Australia
b) China
c) Europe
d) India & Pakistan
e) Northeast Siberia
f) Northern Africa & Middle East
g) Pacific Northwest USA & Canada
h) South America
i) Southern Africa
Heatwave Length (days)
Median Heatwave Area (%)
Cumulative Heat (°C)
300
600
900
1200
1500
2000
SSP5-8.5
Median Heatwave Severity
1.5
2.0
2.5
3.0
3.5
4.0

Supplementary Figure 23: Multi-HW-characteristic figure according to SSP1-2.6 CMIP6 multi-model ensemble (MME) (Tx90p) - Each panel shows regional projections according to SSP1-2.6 model runs in the CMIP6 multi-model ensemble (MME) of four combined heatwave characteristics for each projected heatwave in the ensemble between 2020 and 2100. Each dot represents an individual heatwave in the projections, characterized by Maximum Heatwave Area (%) (x-axis) as latitude-weighted percentage of grid boxes affected by extreme heat, Heatwave Length (days) (y-axis) in days, Maximum Heatwave Severity (color), and Cumulative Heat (°C) (dot size). The characteristics of the observed event according to the Berkeley Earth observational data (x symbol) and the ERA5 reanalysis data (+ symbol), respectively, are pointed to by an arrow and a figure inset shows a zoom-in on this figure section. The figure inset shows the observed heatwave in the two reference data sets and projections in the indicated section of the respective sub-plot. It facilitates readability of color and size of the reference values and comparison to each other and the neighboring projections. Note that the zoom-in causes the dots to overlap more in the inset than in the main sub-plot. The univariate kernel density estimates along the x- (Maximum Heatwave Area (%)) and y-axis (Heatwave Length (days)) are on the top and right side of the subplots, respectively, with vertical lines in dark blue (Berkeley Earth) and light blue (ERA5) indicating the observed event.

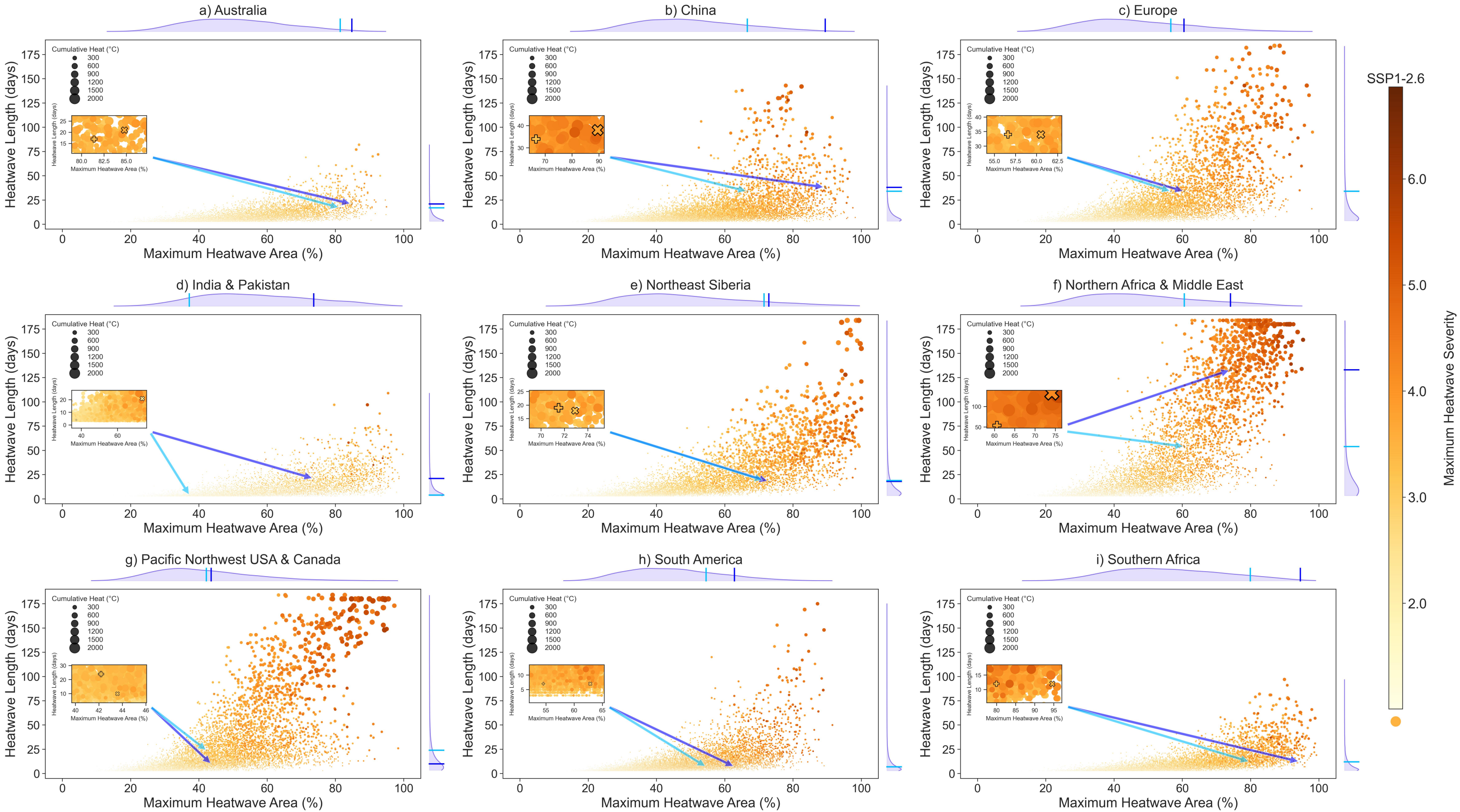

a) Australia
b) China
c) Europe
d) India & Pakistan
e) Northeast Siberia
f) Northern Africa & Middle East
g) Pacific Northwest USA & Canada
h) South America
i) Southern Africa
Heatwave Length (days)
Maximum Heatwave Area (%)
Cumulative Heat (°C)
300
600
900
1200
1500
2000
SSP1-2.6
Maximum Heatwave Severity

Supplementary Figure 24: Multi-HW-characteristic figure according to SSP2-4.5 CMIP6 multi-model ensemble (MME) (Tx90p) - Each panel shows regional projections according to SSP2-4.5 model runs in the CMIP6 multi-model ensemble (MME) of four combined heatwave characteristics for each projected heatwave in the ensemble between 2020 and 2100. Each dot represents an individual heatwave in the projections, characterized by Maximum Heatwave Area (%) (x-axis) as latitude-weighted percentage of grid boxes affected by extreme heat, Heatwave Length (days) (y-axis) in days, Maximum Heatwave Severity (color), and Cumulative Heat (°C) (dot size). The characteristics of the observed event according to the Berkeley Earth observational data (x symbol) and the ERA5 reanalysis data (+ symbol), respectively, are pointed to by an arrow and a figure inset shows a zoom-in on this figure section. The figure inset shows the observed heatwave in the two reference data sets and projections in the indicated section of the respective sub-plot. It facilitates readability of color and size of the reference values and comparison to each other and the neighboring projections. Note that the zoom-in causes the dots to overlap more in the inset than in the main sub-plot. The univariate kernel density estimates along the x- (Maximum Heatwave Area (%)) and y-axis (Heatwave Length (days)) are on the top and right side of the subplots, respectively, with vertical lines in dark blue (Berkeley Earth) and light blue (ERA5) indicating the observed event.

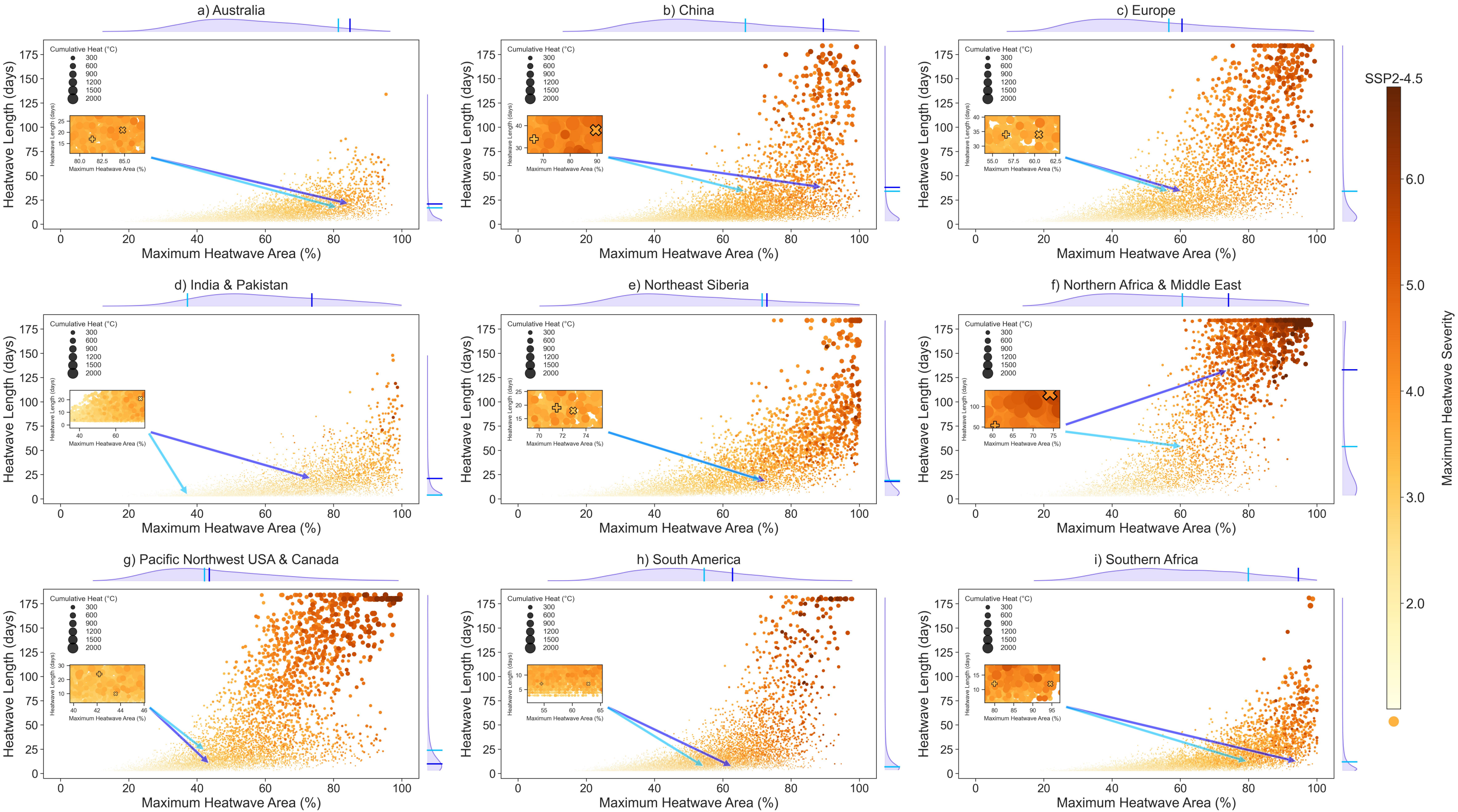

a) Australia
b) China
c) Europe
d) India & Pakistan
e) Northeast Siberia
f) Northern Africa & Middle East
g) Pacific Northwest USA & Canada
h) South America
i) Southern Africa
Heatwave Length (days)
Maximum Heatwave Area (%)
Cumulative Heat (°C)
300
600
900
1200
1500
2000
SSP2-4.5
Maximum Heatwave Severity

Supplementary Figure 25: Multi-HW-characteristic figure according to SSP3-7.0 CMIP6 multi-model ensemble (MME) (Tx90p) - Each panel shows regional projections according to SSP3-7.0 model runs in the CMIP6 multi-model ensemble (MME) of four combined heatwave characteristics for each projected heatwave in the ensemble between 2020 and 2100. Each dot represents an individual heatwave in the projections, characterized by Maximum Heatwave Area (%) (x-axis) as latitude-weighted percentage of grid boxes affected by extreme heat, Heatwave Length (days) (y-axis) in days, Maximum Heatwave Severity (color), and Cumulative Heat (°C) (dot size). The characteristics of the observed event according to the Berkeley Earth observational data (x symbol) and the ERA5 reanalysis data (+ symbol), respectively, are pointed to by an arrow and a figure inset shows a zoom-in on this figure section. The figure inset shows the observed heatwave in the two reference data sets and projections in the indicated section of the respective sub-plot. It facilitates readability of color and size of the reference values and comparison to each other and the neighboring projections. Note that the zoom-in causes the dots to overlap more in the inset than in the main sub-plot. The univariate kernel density estimates along the x- (Maximum Heatwave Area (%)) and y-axis (Heatwave Length (days)) are on the top and right side of the subplots, respectively, with vertical lines in dark blue (Berkeley Earth) and light blue (ERA5) indicating the observed event.

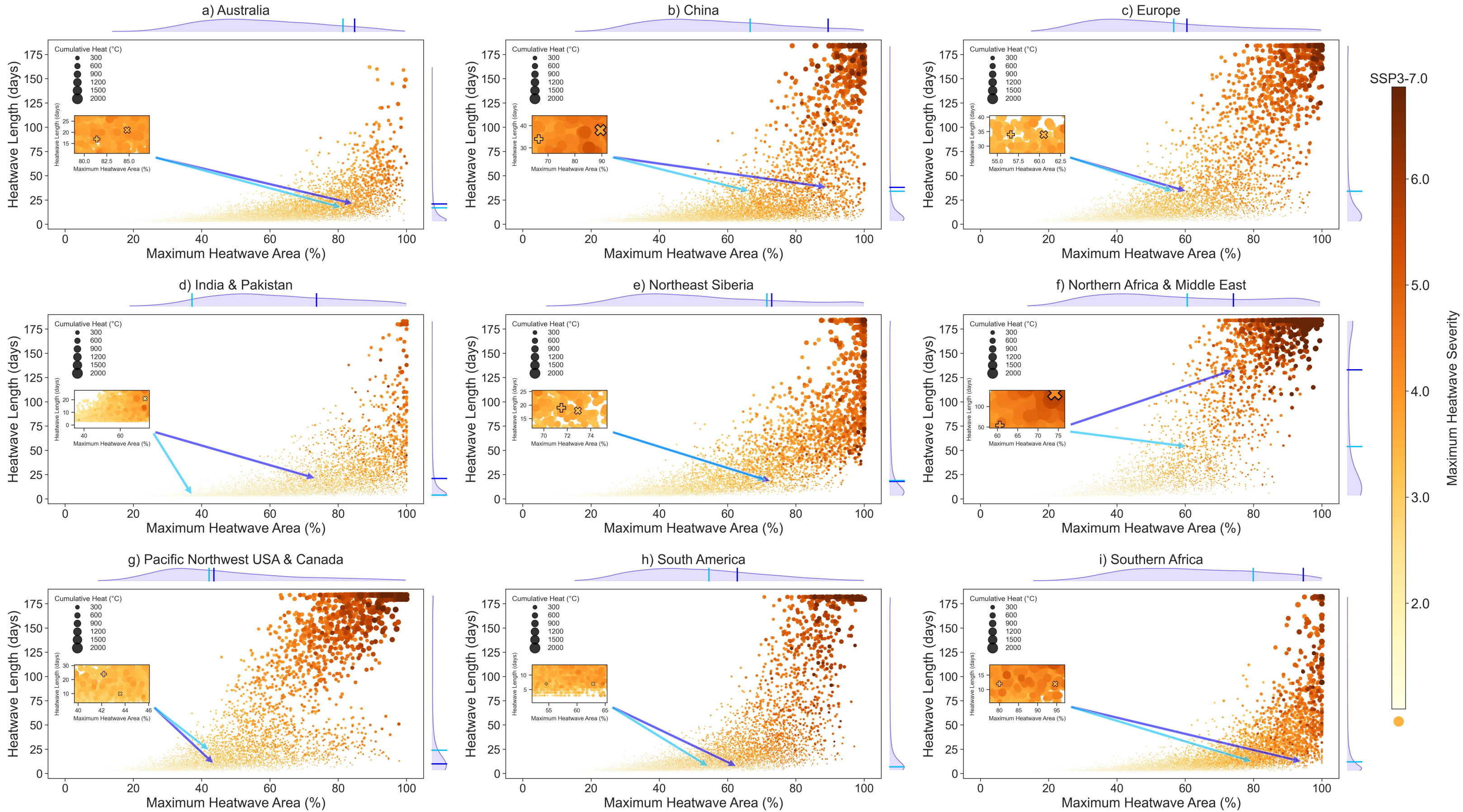
a) Australia
b) China
c) Europe
d) India & Pakistan
e) Northeast Siberia
f) Northern Africa & Middle East
g) Pacific Northwest USA & Canada
h) South America
i) Southern Africa
Heatwave Length (days)
Maximum Heatwave Area (%)
Cumulative Heat (°C)
300
600
900
1200
1500
2000
SSP3-7.0
Maximum Heatwave Severity

Supplementary Figure 26: Multi-HW-characteristic figure according to SSP5-8.5 CMIP6 multi-model ensemble (MME) (Tx90p) - Each panel shows regional projections according to SSP5-8.5 model runs in the CMIP6 multi-model ensemble (MME) of four combined heatwave characteristics for each projected heatwave in the ensemble between 2020 and 2100. Each dot represents an individual heatwave in the projections, characterized by Maximum Heatwave Area (%) (x-axis) as latitude-weighted percentage of grid boxes affected by extreme heat, Heatwave Length (days) (y-axis) in days, Maximum Heatwave Severity (color), and Cumulative Heat (°C) (dot size). The characteristics of the observed event according to the Berkeley Earth observational data (x symbol) and the ERA5 reanalysis data (+ symbol), respectively, are pointed to by an arrow and a figure inset shows a zoom-in on this figure section. The figure inset shows the observed heatwave in the two reference data sets and projections in the indicated section of the respective sub-plot. It facilitates readability of color and size of the reference values and comparison to each other and the neighboring projections. Note that the zoom-in causes the dots to overlap more in the inset than in the main sub-plot. The univariate kernel density estimates along the x- (Maximum Heatwave Area (%)) and y-axis (Heatwave Length (days)) are on the top and right side of the subplots, respectively, with vertical lines in dark blue (Berkeley Earth) and light blue (ERA5) indicating the observed event.

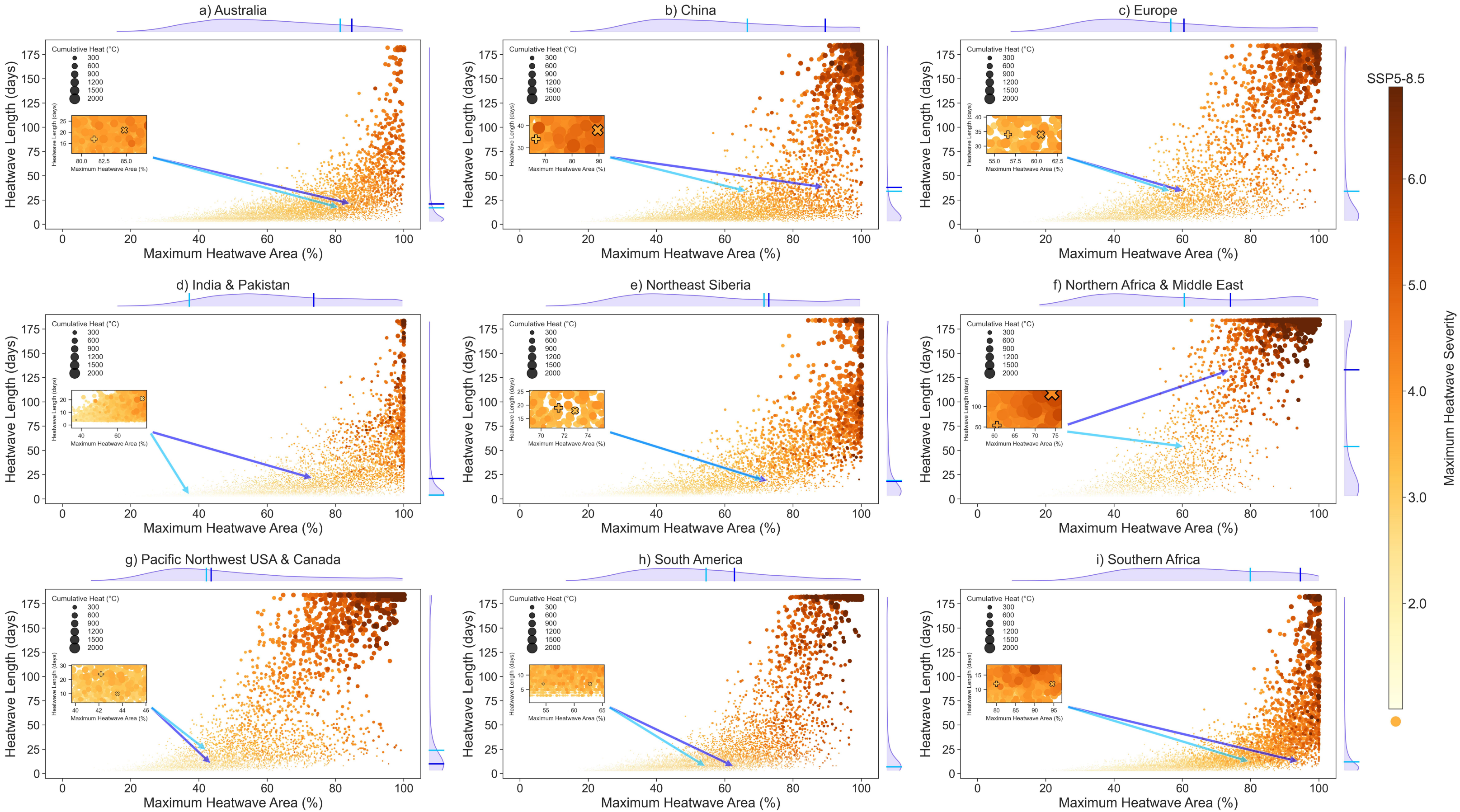
a) Australia
b) China
c) Europe
d) India & Pakistan
e) Northeast Siberia
f) Northern Africa & Middle East
g) Pacific Northwest USA & Canada
h) South America
i) Southern Africa
Cumulative Heat (°C)
300
600
900
1200
1500
2000
Heatwave Length (days)
Maximum Heatwave Area (%)
SSP5-8.5
Maximum Heatwave Severity

Supplementary Figure 27: Multi-HW-characteristic figure according to SSP1-2.6 CMIP6 multi-model ensemble (MME) (Tx90p) - Each panel shows regional projections according to SSP1-2.6 model runs in the CMIP6 multi-model ensemble (MME) of four combined heatwave characteristics for each projected heatwave in the ensemble between 2020 and 2100. Each dot represents an individual heatwave in the projections, characterized by Median Heatwave Area (%) (x-axis) as latitude-weighted percentage of grid boxes affected by extreme heat, Heatwave Length (days) (y-axis) in days, Median Heatwave Severity (color), and Cumulative Heat (°C) (dot size). The characteristics of the observed event according to the Berkeley Earth observational data (x symbol) and the ERA5 reanalysis data (+ symbol), respectively, are pointed to by an arrow and a figure inset shows a zoom-in on this figure section. The figure inset shows the observed heatwave in the two reference data sets and projections in the indicated section of the respective sub-plot. It facilitates readability of color and size of the reference values and comparison to each other and the neighboring projections. Note that the zoom-in causes the dots to overlap more in the inset than in the main sub-plot. The univariate kernel density estimates along the x- (Median Heatwave Area (%)) and y-axis (Heatwave Length (days)) are on the top and right side of the subplots, respectively, with vertical lines in dark blue (Berkeley Earth) and light blue (ERA5) indicating the observed event.

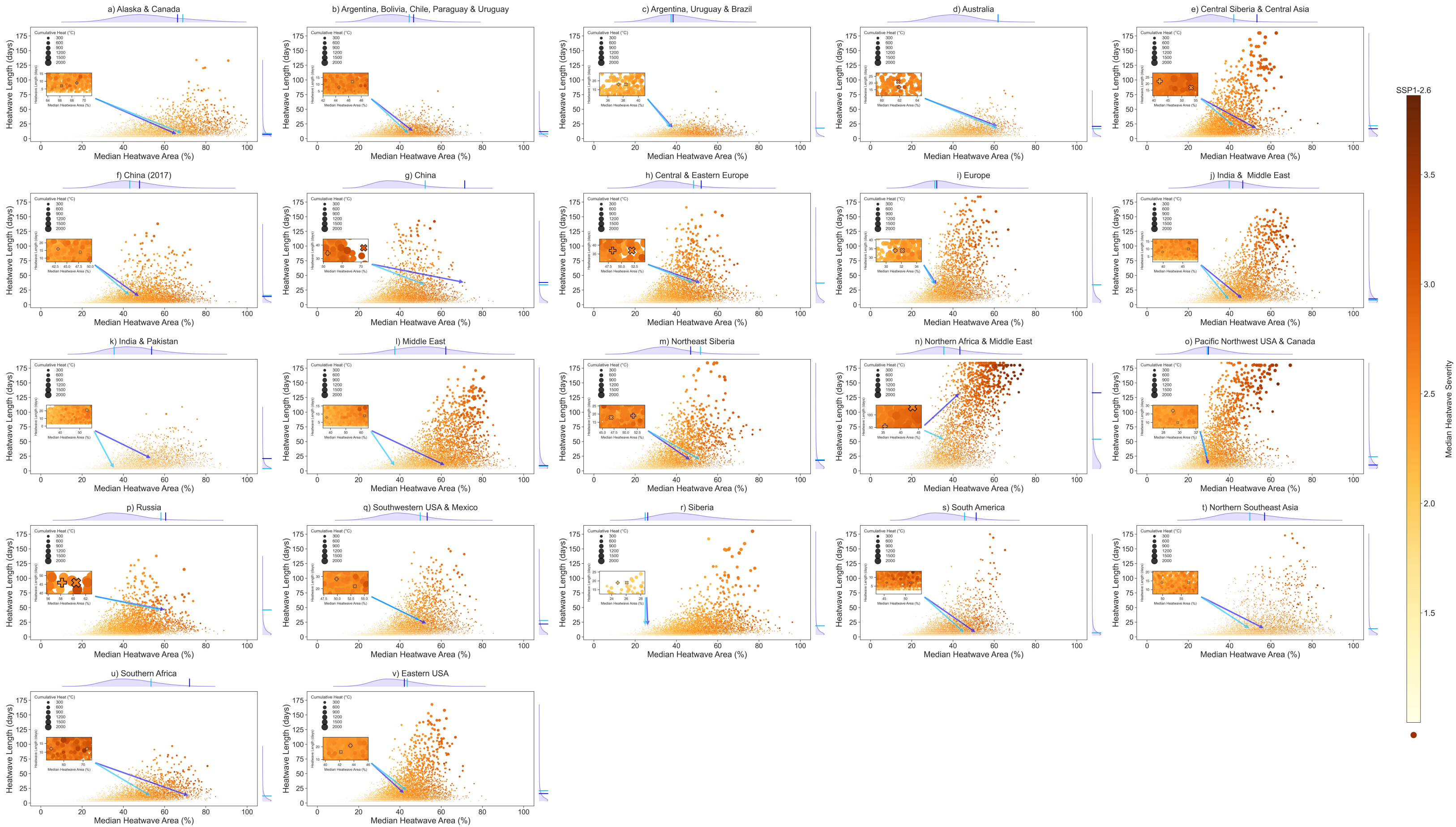
a) Alaska & Canada
b) Argentina, Bolivia, Chile, Paraguay & Uruguay
c) Argentina, Uruguay & Brazil
d) Australia
e) Central Siberia & Central Asia
f) China (2017)
g) China
h) Central & Eastern Europe
i) Europe
j) India & Middle East
k) India & Pakistan
l) Middle East
m) Northeast Siberia
n) Northern Africa & Middle East
o) Pacific Northwest USA & Canada
p) Russia
q) Southwestern USA & Mexico
r) Siberia
s) South America
t) Northern Southeast Asia
u) Southern Africa
v) Eastern USA
Heatwave Length (days)
Median Heatwave Area (%)
Cumulative Heat (°C)
300
600
900
1200
1500
2000
SSP1-2.6
Median Heatwave Severity
1.5
2.0
2.5
3.0
3.5

Supplementary Figure 28: Multi-HW-characteristic figure according to SSP2-4.5 CMIP6 multi-model ensemble (MME) (Tx90p) - Each panel shows regional projections according to SSP2-4.5 model runs in the CMIP6 multi-model ensemble (MME) of four combined heatwave characteristics for each projected heatwave in the ensemble between 2020 and 2100. Each dot represents an individual heatwave in the projections, characterized by Median Heatwave Area (%) (x-axis) as latitude-weighted percentage of grid boxes affected by extreme heat, Heatwave Length (days) (y-axis) in days, Median Heatwave Severity (color), and Cumulative Heat (°C) (dot size). The characteristics of the observed event according to the Berkeley Earth observational data (x symbol) and the ERA5 reanalysis data (+ symbol), respectively, are pointed to by an arrow and a figure inset shows a zoom-in on this figure section. The figure inset shows the observed heatwave in the two reference data sets and projections in the indicated section of the respective sub-plot. It facilitates readability of color and size of the reference values and comparison to each other and the neighboring projections. Note that the zoom-in causes the dots to overlap more in the inset than in the main sub-plot. The univariate kernel density estimates along the x- (Median Heatwave Area (%)) and y-axis (Heatwave Length (days)) are on the top and right side of the subplots, respectively, with vertical lines in dark blue (Berkeley Earth) and light blue (ERA5) indicating the observed event.

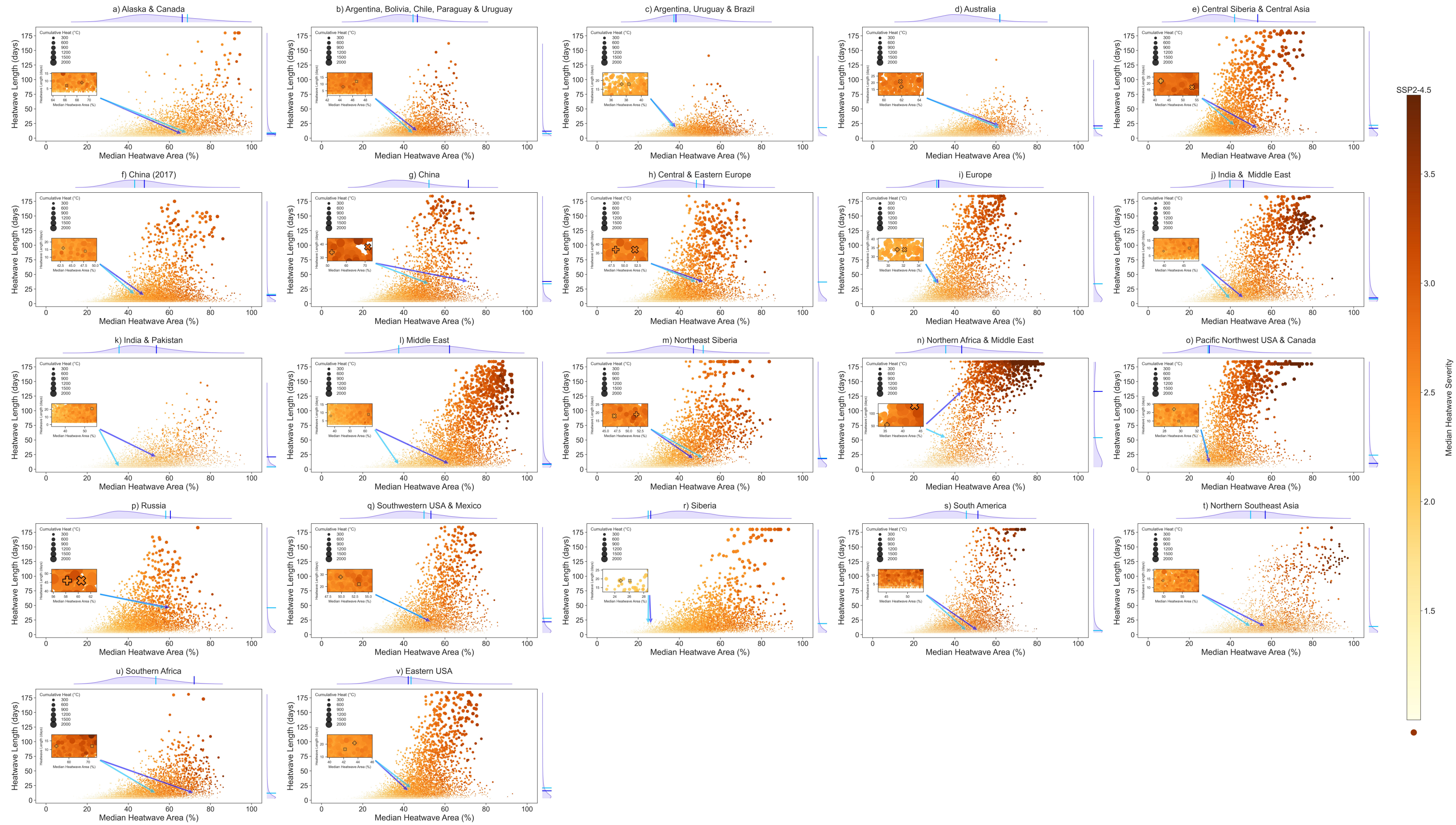

a) Alaska & Canada
b) Argentina, Bolivia, Chile, Paraguay & Uruguay
c) Argentina, Uruguay & Brazil
d) Australia
e) Central Siberia & Central Asia
f) China (2017)
g) China
h) Central & Eastern Europe
i) Europe
j) India & Middle East
k) India & Pakistan
l) Middle East
m) Northeast Siberia
n) Northern Africa & Middle East
o) Pacific Northwest USA & Canada
p) Russia
q) Southwestern USA & Mexico
r) Siberia
s) South America
t) Northern Southeast Asia
u) Southern Africa
v) Eastern USA
Heatwave Length (days)
Median Heatwave Area (%)
Cumulative Heat (°C)
300
600
900
1200
1500
2000
SSP2-4.5
Median Heatwave Severity

Supplementary Figure 29: Multi-HW-characteristic figure according to SSP3-7.0 CMIP6 multi-model ensemble (MME) (Tx90p) - Each panel shows regional projections according to SSP3-7.0 model runs in the CMIP6 multi-model ensemble (MME) of four combined heatwave characteristics for each projected heatwave in the ensemble between 2020 and 2100. Each dot represents an individual heatwave in the projections, characterized by Median Heatwave Area (%) (x-axis) as latitude-weighted percentage of grid boxes affected by extreme heat, Heatwave Length (days) (y-axis) in days, Median Heatwave Severity (color), and Cumulative Heat (°C) (dot size). The characteristics of the observed event according to the Berkeley Earth observational data (x symbol) and the ERA5 reanalysis data (+ symbol), respectively, are pointed to by an arrow and a figure inset shows a zoom-in on this figure section. The figure inset shows the observed heatwave in the two reference data sets and projections in the indicated section of the respective sub-plot. It facilitates readability of color and size of the reference values and comparison to each other and the neighboring projections. Note that the zoom-in causes the dots to overlap more in the inset than in the main sub-plot. The univariate kernel density estimates along the x- (Median Heatwave Area (%)) and y-axis (Heatwave Length (days)) are on the top and right side of the subplots, respectively, with vertical lines in dark blue (Berkeley Earth) and light blue (ERA5) indicating the observed event.

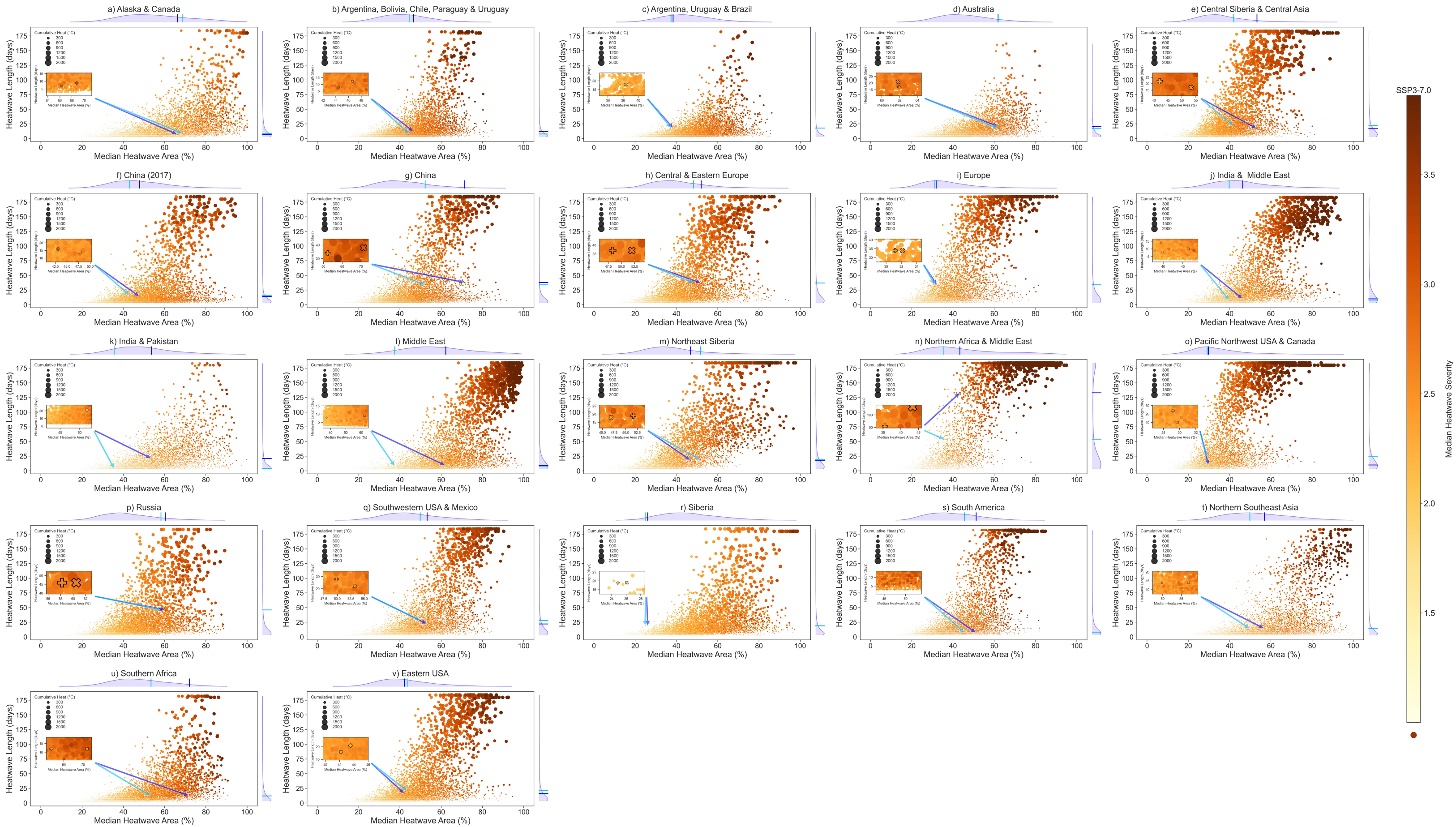
a) Alaska & Canada
b) Argentina, Bolivia, Chile, Paraguay & Uruguay
c) Argentina, Uruguay & Brazil
d) Australia
e) Central Siberia & Central Asia
f) China (2017)
g) China
h) Central & Eastern Europe
i) Europe
j) India & Middle East
k) India & Pakistan
l) Middle East
m) Northeast Siberia
n) Northern Africa & Middle East
o) Pacific Northwest USA & Canada
p) Russia
q) Southwestern USA & Mexico
r) Siberia
s) South America
t) Northern Southeast Asia
u) Southern Africa
v) Eastern USA
Heatwave Length (days)
Median Heatwave Area (%)
Cumulative Heat (°C)
300
600
900
1200
1500
2000
SSP3-7.0
Median Heatwave Severity

Supplementary Figure 30: Multi-HW-characteristic figure according to SSP5-8.5 CMIP6 multi-model ensemble (MME) (Tx90p) - Each panel shows regional projections according to SSP5-8.5 model runs in the CMIP6 multi-model ensemble (MME) of four combined heatwave characteristics for each projected heatwave in the ensemble between 2020 and 2100. Each dot represents an individual heatwave in the projections, characterized by Median Heatwave Area (%) (x-axis) as latitude-weighted percentage of grid boxes affected by extreme heat, Heatwave Length (days) (y-axis) in days, Median Heatwave Severity (color), and Cumulative Heat (°C) (dot size). The characteristics of the observed event according to the Berkeley Earth observational data (x symbol) and the ERA5 reanalysis data (+ symbol), respectively, are pointed to by an arrow and a figure inset shows a zoom-in on this figure section. The figure inset shows the observed heatwave in the two reference data sets and projections in the indicated section of the respective sub-plot. It facilitates readability of color and size of the reference values and comparison to each other and the neighboring projections. Note that the zoom-in causes the dots to overlap more in the inset than in the main sub-plot. The univariate kernel density estimates along the x- (Median Heatwave Area (%)) and y-axis (Heatwave Length (days)) are on the top and right side of the subplots, respectively, with vertical lines in dark blue (Berkeley Earth) and light blue (ERA5) indicating the observed event.

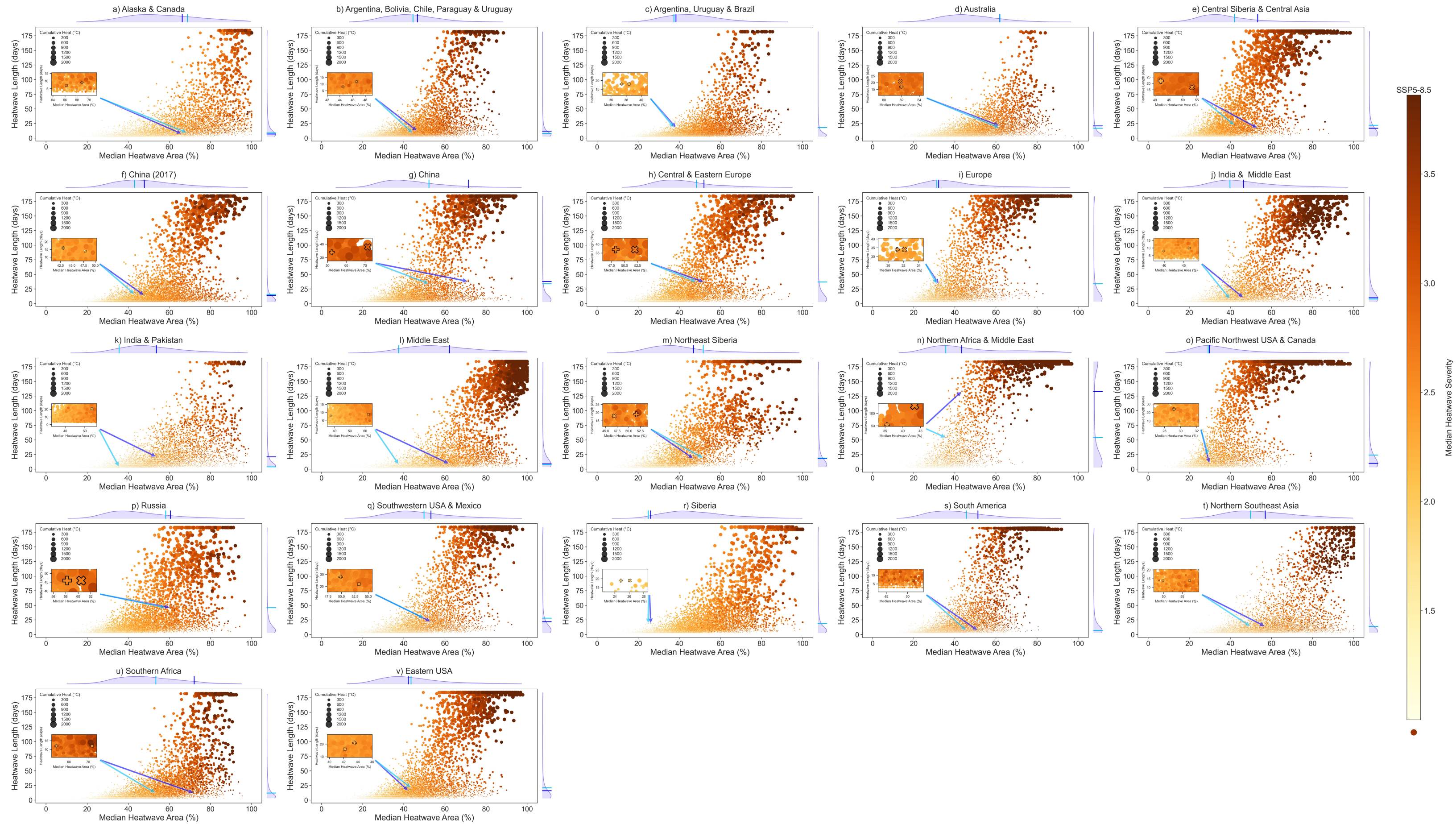
a) Alaska & Canada
b) Argentina, Bolivia, Chile, Paraguay & Uruguay
c) Argentina, Uruguay & Brazil
d) Australia
e) Central Siberia & Central Asia
f) China (2017)
g) China
h) Central & Eastern Europe
i) Europe
j) India & Middle East
k) India & Pakistan
l) Middle East
m) Northeast Siberia
n) Northern Africa & Middle East
o) Pacific Northwest USA & Canada
p) Russia
q) Southwestern USA & Mexico
r) Siberia
s) South America
t) Northern Southeast Asia
u) Southern Africa
v) Eastern USA
Heatwave Length (days)
Median Heatwave Area (%)
Cumulative Heat (°C)
300
600
900
1200
1500
2000
SSP5-8.5
Median Heatwave Severity
3.5
3.0
2.5
2.0
1.5

Supplementary Figure 31: Multi-HW-characteristic figure according to SSP1-2.6 CMIP6 multi-model ensemble (MME) (Tx90p) - Each panel shows regional projections according to SSP1-2.6 model runs in the CMIP6 multi-model ensemble (MME) of four combined heatwave characteristics for each projected heatwave in the ensemble between 2020 and 2100. Each dot represents an individual heatwave in the projections, characterized by Maximum Heatwave Area (%) (x-axis) as latitude-weighted percentage of grid boxes affected by extreme heat, Heatwave Length (days) (y-axis) in days, Maximum Heatwave Severity (color), and Cumulative Heat (°C) (dot size). The characteristics of the observed event according to the Berkeley Earth observational data (x symbol) and the ERA5 reanalysis data (+ symbol), respectively, are pointed to by an arrow and a figure inset shows a zoom-in on this figure section. The figure inset shows the observed heatwave in the two reference data sets and projections in the indicated section of the respective sub-plot. It facilitates readability of color and size of the reference values and comparison to each other and the neighboring projections. Note that the zoom-in causes the dots to overlap more in the inset than in the main sub-plot. The univariate kernel density estimates along the x- (Maximum Heatwave Area (%)) and y-axis (Heatwave Length (days)) are on the top and right side of the subplots, respectively, with vertical lines in dark blue (Berkeley Earth) and light blue (ERA5) indicating the observed event.

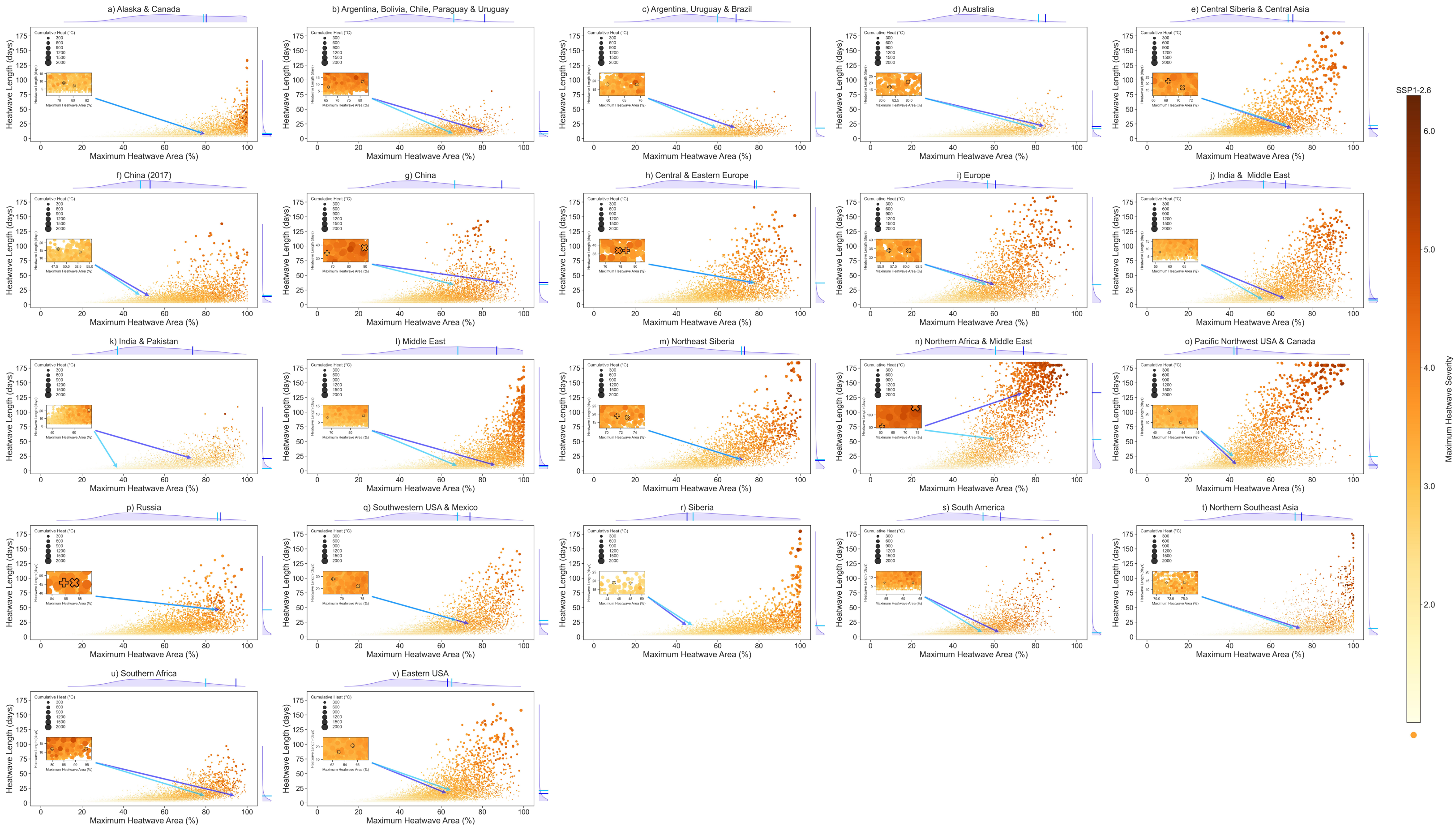

a) Alaska & Canada
b) Argentina, Bolivia, Chile, Paraguay & Uruguay
c) Argentina, Uruguay & Brazil
d) Australia
e) Central Siberia & Central Asia
f) China (2017)
g) China
h) Central & Eastern Europe
i) Europe
j) India & Middle East
k) India & Pakistan
l) Middle East
m) Northeast Siberia
n) Northern Africa & Middle East
o) Pacific Northwest USA & Canada
p) Russia
q) Southwestern USA & Mexico
r) Siberia
s) South America
t) Northern Southeast Asia
u) Southern Africa
v) Eastern USA
Heatwave Length (days)
Maximum Heatwave Area (%)
Cumulative Heat (°C)
300
600
900
1200
1500
2000
SSP1-2.6
Maximum Heatwave Severity

Supplementary Figure 32: Multi-HW-characteristic figure according to SSP2-4.5 CMIP6 multi-model ensemble (MME) (Tx90p) - Each panel shows regional projections according to SSP2-4.5 model runs in the CMIP6 multi-model ensemble (MME) of four combined heatwave characteristics for each projected heatwave in the ensemble between 2020 and 2100. Each dot represents an individual heatwave in the projections, characterized by Maximum Heatwave Area (%) (x-axis) as latitude-weighted percentage of grid boxes affected by extreme heat, Heatwave Length (days) (y-axis) in days, Maximum Heatwave Severity (color), and Cumulative Heat (°C) (dot size). The characteristics of the observed event according to the Berkeley Earth observational data (x symbol) and the ERA5 reanalysis data (+ symbol), respectively, are pointed to by an arrow and a figure inset shows a zoom-in on this figure section. The figure inset shows the observed heatwave in the two reference data sets and projections in the indicated section of the respective sub-plot. It facilitates readability of color and size of the reference values and comparison to each other and the neighboring projections. Note that the zoom-in causes the dots to overlap more in the inset than in the main sub-plot. The univariate kernel density estimates along the x- (Maximum Heatwave Area (%)) and y-axis (Heatwave Length (days)) are on the top and right side of the subplots, respectively, with vertical lines in dark blue (Berkeley Earth) and light blue (ERA5) indicating the observed event.

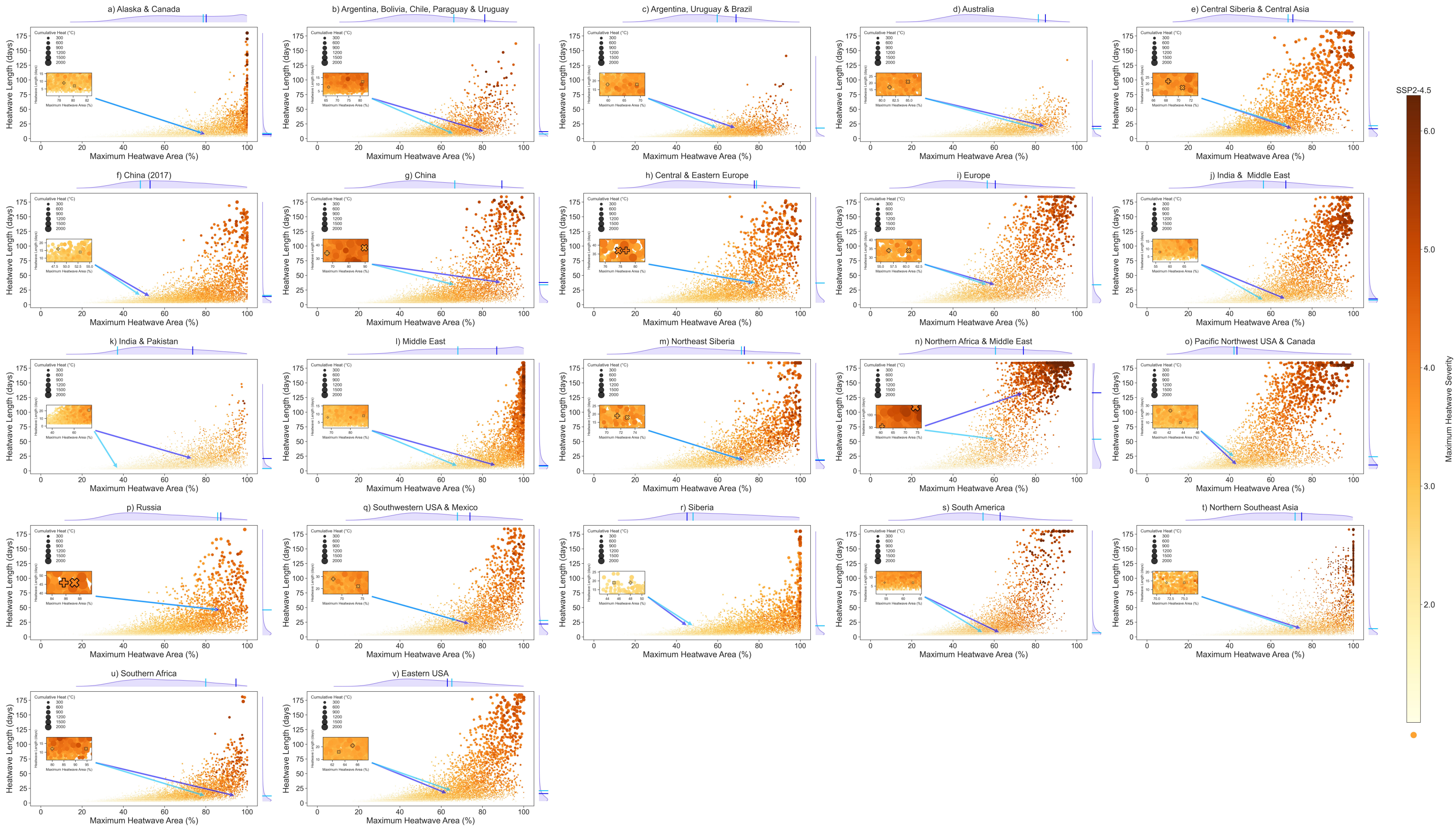
a) Alaska & Canada
b) Argentina, Bolivia, Chile, Paraguay & Uruguay
c) Argentina, Uruguay & Brazil
d) Australia
e) Central Siberia & Central Asia
f) China (2017)
g) China
h) Central & Eastern Europe
i) Europe
j) India & Middle East
k) India & Pakistan
l) Middle East
m) Northeast Siberia
n) Northern Africa & Middle East
o) Pacific Northwest USA & Canada
p) Russia
q) Southwestern USA & Mexico
r) Siberia
s) South America
t) Northern Southeast Asia
u) Southern Africa
v) Eastern USA
Heatwave Length (days)
Maximum Heatwave Area (%)
Cumulative Heat (°C)
300
600
900
1200
1500
2000
SSP2-4.5
Maximum Heatwave Severity

Supplementary Figure 33: Multi-HW-characteristic figure according to SSP3-7.0 CMIP6 multi-model ensemble (MME) (Tx90p) - Each panel shows regional projections according to SSP3-7.0 model runs in the CMIP6 multi-model ensemble (MME) of four combined heatwave characteristics for each projected heatwave in the ensemble between 2020 and 2100. Each dot represents an individual heatwave in the projections, characterized by Maximum Heatwave Area (%) (x-axis) as latitude-weighted percentage of grid boxes affected by extreme heat, Heatwave Length (days) (y-axis) in days, Maximum Heatwave Severity (color), and Cumulative Heat (°C) (dot size). The characteristics of the observed event according to the Berkeley Earth observational data (x symbol) and the ERA5 reanalysis data (+ symbol), respectively, are pointed to by an arrow and a figure inset shows a zoom-in on this figure section. The figure inset shows the observed heatwave in the two reference data sets and projections in the indicated section of the respective sub-plot. It facilitates readability of color and size of the reference values and comparison to each other and the neighboring projections. Note that the zoom-in causes the dots to overlap more in the inset than in the main sub-plot. The univariate kernel density estimates along the x- (Maximum Heatwave Area (%)) and y-axis (Heatwave Length (days)) are on the top and right side of the subplots, respectively, with vertical lines in dark blue (Berkeley Earth) and light blue (ERA5) indicating the observed event.

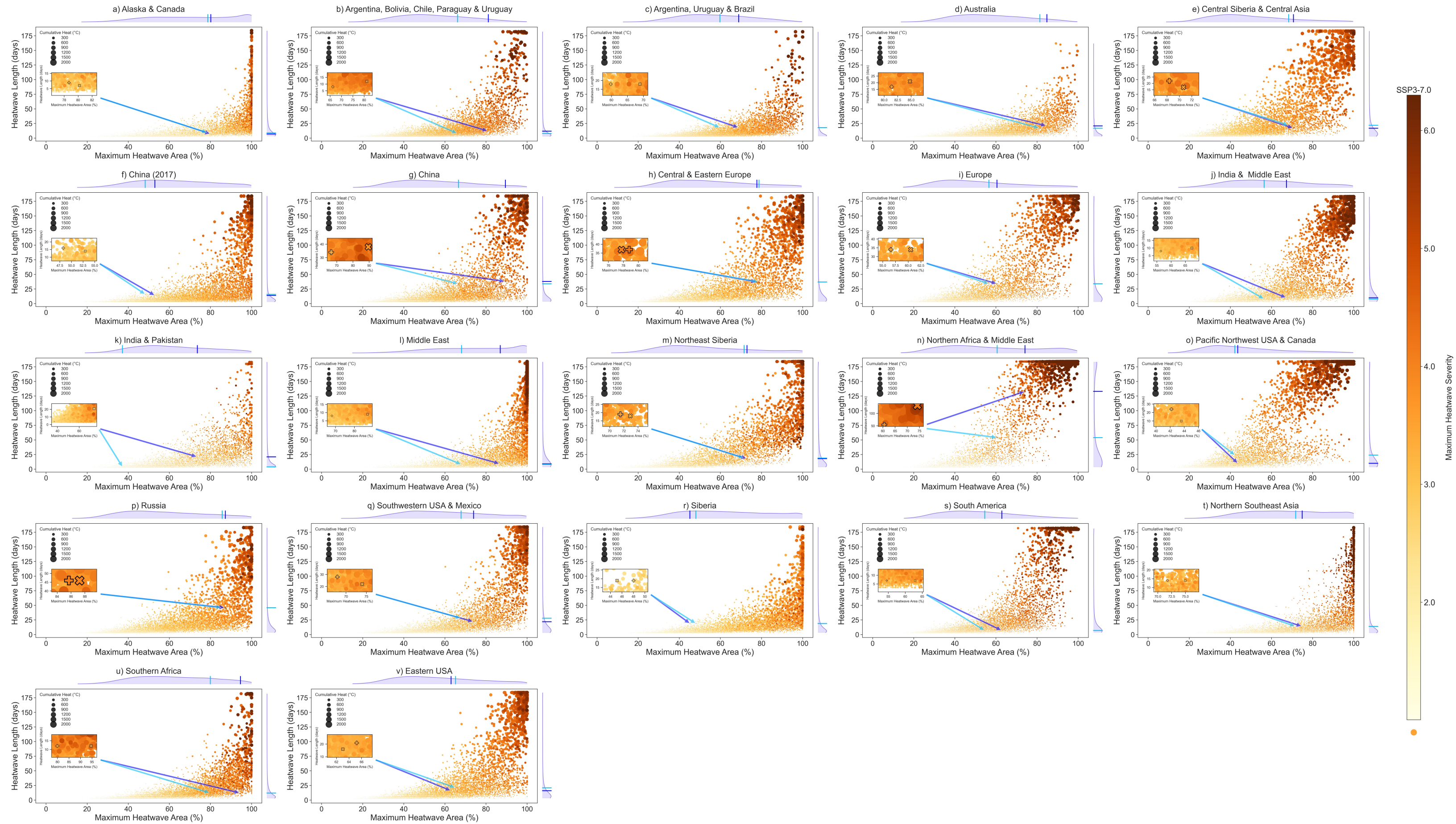

a) Alaska & Canada
b) Argentina, Bolivia, Chile, Paraguay & Uruguay
c) Argentina, Uruguay & Brazil
d) Australia
e) Central Siberia & Central Asia
f) China (2017)
g) China
h) Central & Eastern Europe
i) Europe
j) India & Middle East
k) India & Pakistan
l) Middle East
m) Northeast Siberia
n) Northern Africa & Middle East
o) Pacific Northwest USA & Canada
p) Russia
q) Southwestern USA & Mexico
r) Siberia
s) South America
t) Northern Southeast Asia
u) Southern Africa
v) Eastern USA
Heatwave Length (days)
Maximum Heatwave Area (%)
Cumulative Heat (°C)
300
600
900
1200
1500
2000
SSP3-7.0
Maximum Heatwave Severity
6.0
5.0
4.0
3.0
2.0

Supplementary Figure 34: Multi-HW-characteristic figure according to SSP5-8.5 CMIP6 multi-model ensemble (MME) (Tx90p) - Each panel shows regional projections according to SSP5-8.5 model runs in the CMIP6 multi-model ensemble (MME) of four combined heatwave characteristics for each projected heatwave in the ensemble between 2020 and 2100. Each dot represents an individual heatwave in the projections, characterized by Maximum Heatwave Area (%) (x-axis) as latitude-weighted percentage of grid boxes affected by extreme heat, Heatwave Length (days) (y-axis) in days, Maximum Heatwave Severity (color), and Cumulative Heat (°C) (dot size). The characteristics of the observed event according to the Berkeley Earth observational data (x symbol) and the ERA5 reanalysis data (+ symbol), respectively, are pointed to by an arrow and a figure inset shows a zoom-in on this figure section. The figure inset shows the observed heatwave in the two reference data sets and projections in the indicated section of the respective sub-plot. It facilitates readability of color and size of the reference values and comparison to each other and the neighboring projections. Note that the zoom-in causes the dots to overlap more in the inset than in the main sub-plot. The univariate kernel density estimates along the x- (Maximum Heatwave Area (%)) and y-axis (Heatwave Length (days)) are on the top and right side of the subplots, respectively, with vertical lines in dark blue (Berkeley Earth) and light blue (ERA5) indicating the observed event.

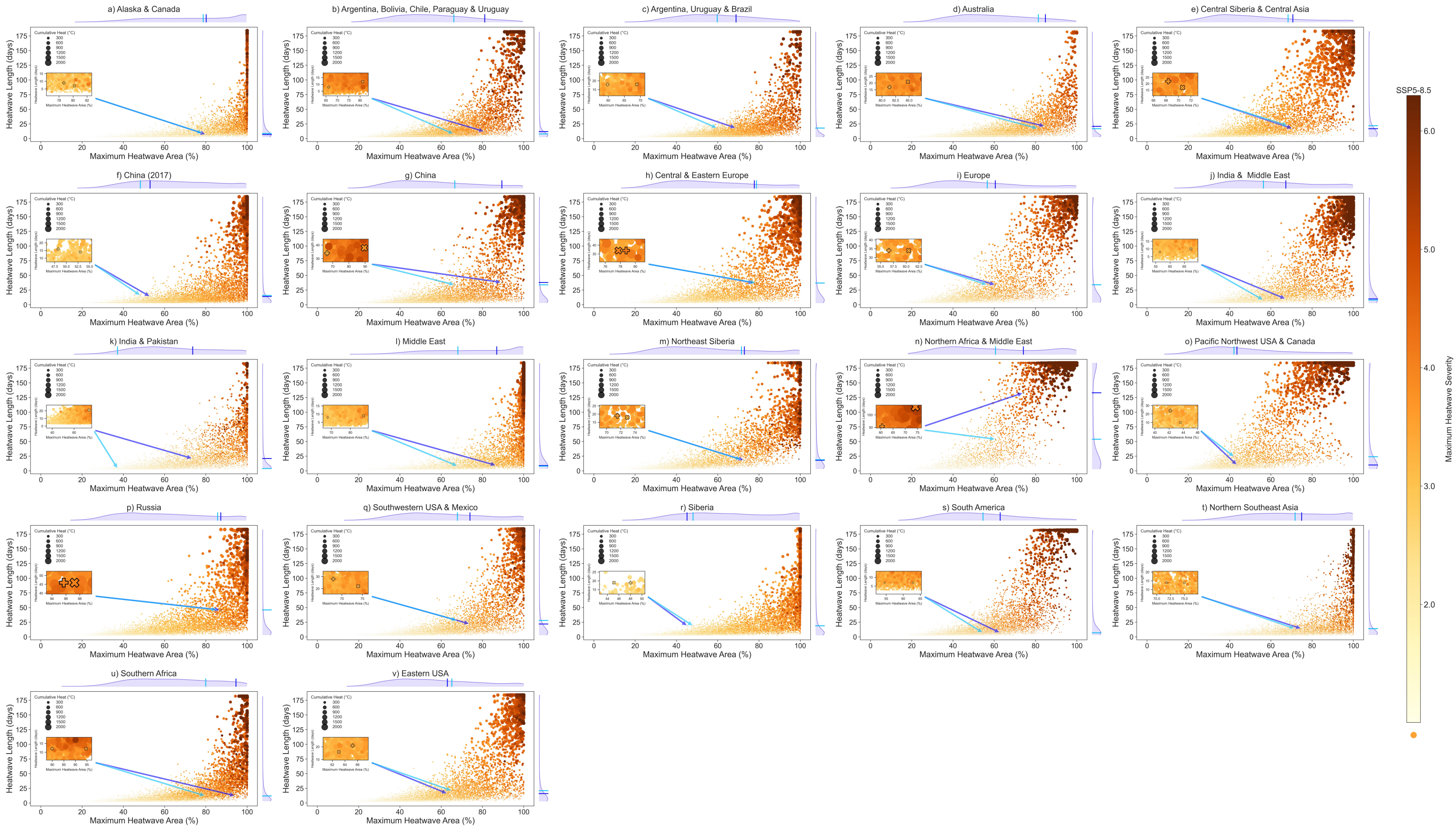
a) Alaska & Canada
b) Argentina, Bolivia, Chile, Paraguay & Uruguay
c) Argentina, Uruguay & Brazil
d) Australia
e) Central Siberia & Central Asia
f) China (2017)
g) China
h) Central & Eastern Europe
i) Europe
j) India & Middle East
k) India & Pakistan
l) Middle East
m) Northeast Siberia
n) Northern Africa & Middle East
o) Pacific Northwest USA & Canada
p) Russia
q) Southwestern USA & Mexico
r) Siberia
s) South America
t) Northern Southeast Asia
u) Southern Africa
v) Eastern USA
Heatwave Length (days)
Maximum Heatwave Area (%)
Cumulative Heat (°C)
300
600
900
1200
1500
2000
SSP5-8.5
Maximum Heatwave Severity
2.0
3.0
4.0
5.0
6.0

Supplementary Figure 35: Multi-HW-characteristic figure according to RCP 2.6 CMIP5 multi-model ensemble (MME) (Tx90p) - Each panel shows regional projections according to RCP 2.6 model runs in the CMIP5 multi-model ensemble (MME) of four combined heatwave characteristics for each projected heatwave in the ensemble between 2020 and 2100. Each dot represents an individual heatwave in the projections, characterized by Median Heatwave Area (%) (x-axis) as latitude-weighted percentage of grid boxes affected by extreme heat, Heatwave Length (days) (y-axis) in days, Median Heatwave Severity (color), and Cumulative Heat (°C) (dot size). The characteristics of the observed event according to the Berkeley Earth observational data (x symbol) and the ERA5 reanalysis data (+ symbol), respectively, are pointed to by an arrow and a figure inset shows a zoom-in on this figure section. The figure inset shows the observed heatwave in the two reference data sets and projections in the indicated section of the respective sub-plot. It facilitates readability of color and size of the reference values and comparison to each other and the neighboring projections. Note that the zoom-in causes the dots to overlap more in the inset than in the main sub-plot. The univariate kernel density estimates along the x- (Median Heatwave Area (%)) and y-axis (Heatwave Length (days)) are on the top and right side of the subplots, respectively, with vertical lines in dark blue (Berkeley Earth) and light blue (ERA5) indicating the observed event.

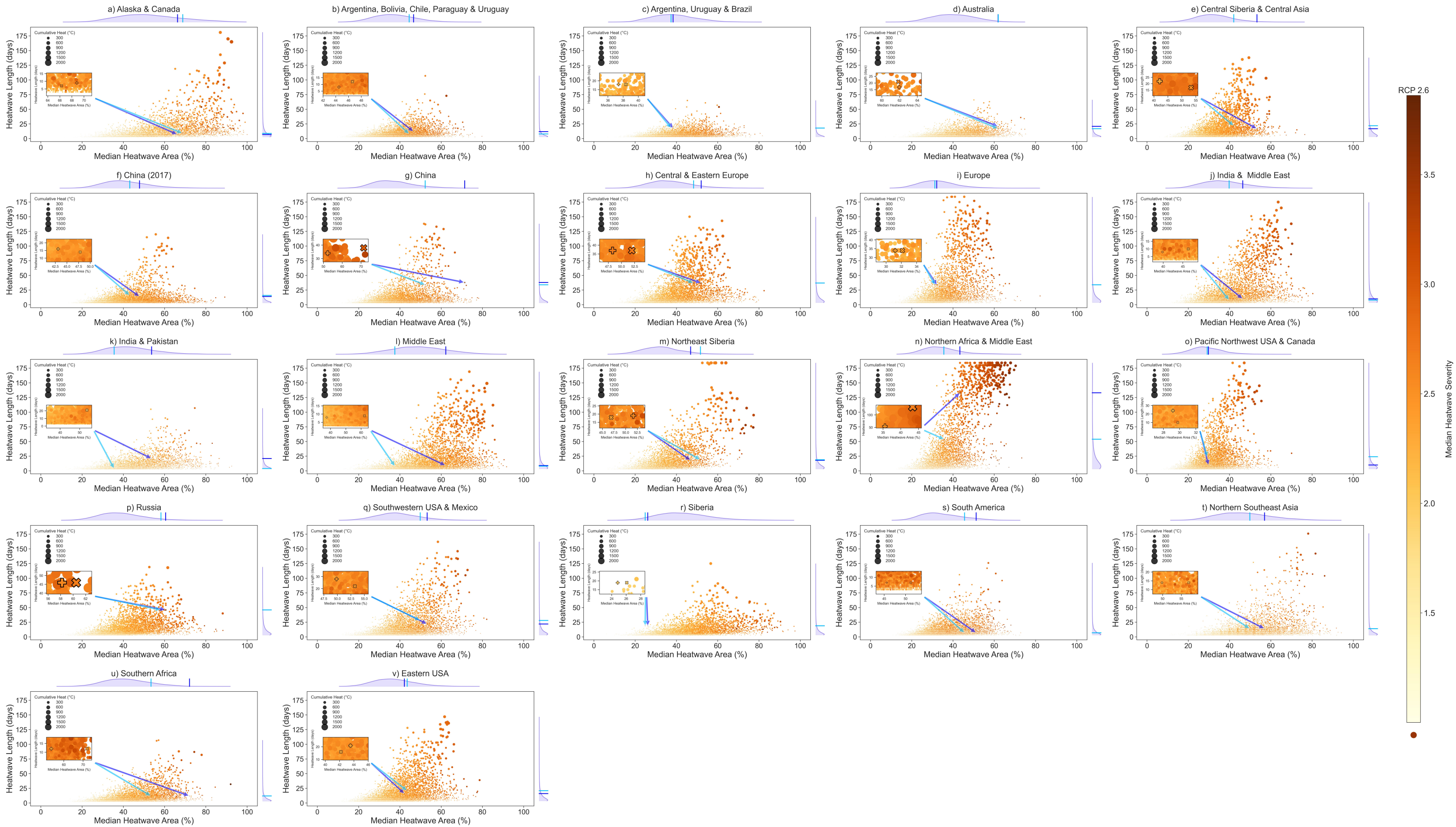

a) Alaska & Canada
b) Argentina, Bolivia, Chile, Paraguay & Uruguay
c) Argentina, Uruguay & Brazil
d) Australia
e) Central Siberia & Central Asia
f) China (2017)
g) China
h) Central & Eastern Europe
i) Europe
j) India & Middle East
k) India & Pakistan
l) Middle East
m) Northeast Siberia
n) Northern Africa & Middle East
o) Pacific Northwest USA & Canada
p) Russia
q) Southwestern USA & Mexico
r) Siberia
s) South America
t) Northern Southeast Asia
u) Southern Africa
v) Eastern USA
Heatwave Length (days)
Median Heatwave Area (%)
Cumulative Heat (°C)
300
600
900
1200
1500
2000
RCP 2.6
Median Heatwave Severity

Supplementary Figure 36: Multi-HW-characteristic figure according to RCP 4.5 CMIP5 multi-model ensemble (MME) (Tx90p) - Each panel shows regional projections according to RCP 4.5 model runs in the CMIP5 multi-model ensemble (MME) of four combined heatwave characteristics for each projected heatwave in the ensemble between 2020 and 2100. Each dot represents an individual heatwave in the projections, characterized by Median Heatwave Area (%) (x-axis) as latitude-weighted percentage of grid boxes affected by extreme heat, Heatwave Length (days) (y-axis) in days, Median Heatwave Severity (color), and Cumulative Heat (°C) (dot size). The characteristics of the observed event according to the Berkeley Earth observational data (x symbol) and the ERA5 reanalysis data (+ symbol), respectively, are pointed to by an arrow and a figure inset shows a zoom-in on this figure section. The figure inset shows the observed heatwave in the two reference data sets and projections in the indicated section of the respective sub-plot. It facilitates readability of color and size of the reference values and comparison to each other and the neighboring projections. Note that the zoom-in causes the dots to overlap more in the inset than in the main sub-plot. The univariate kernel density estimates along the x- (Median Heatwave Area (%)) and y-axis (Heatwave Length (days)) are on the top and right side of the subplots, respectively, with vertical lines in dark blue (Berkeley Earth) and light blue (ERA5) indicating the observed event.

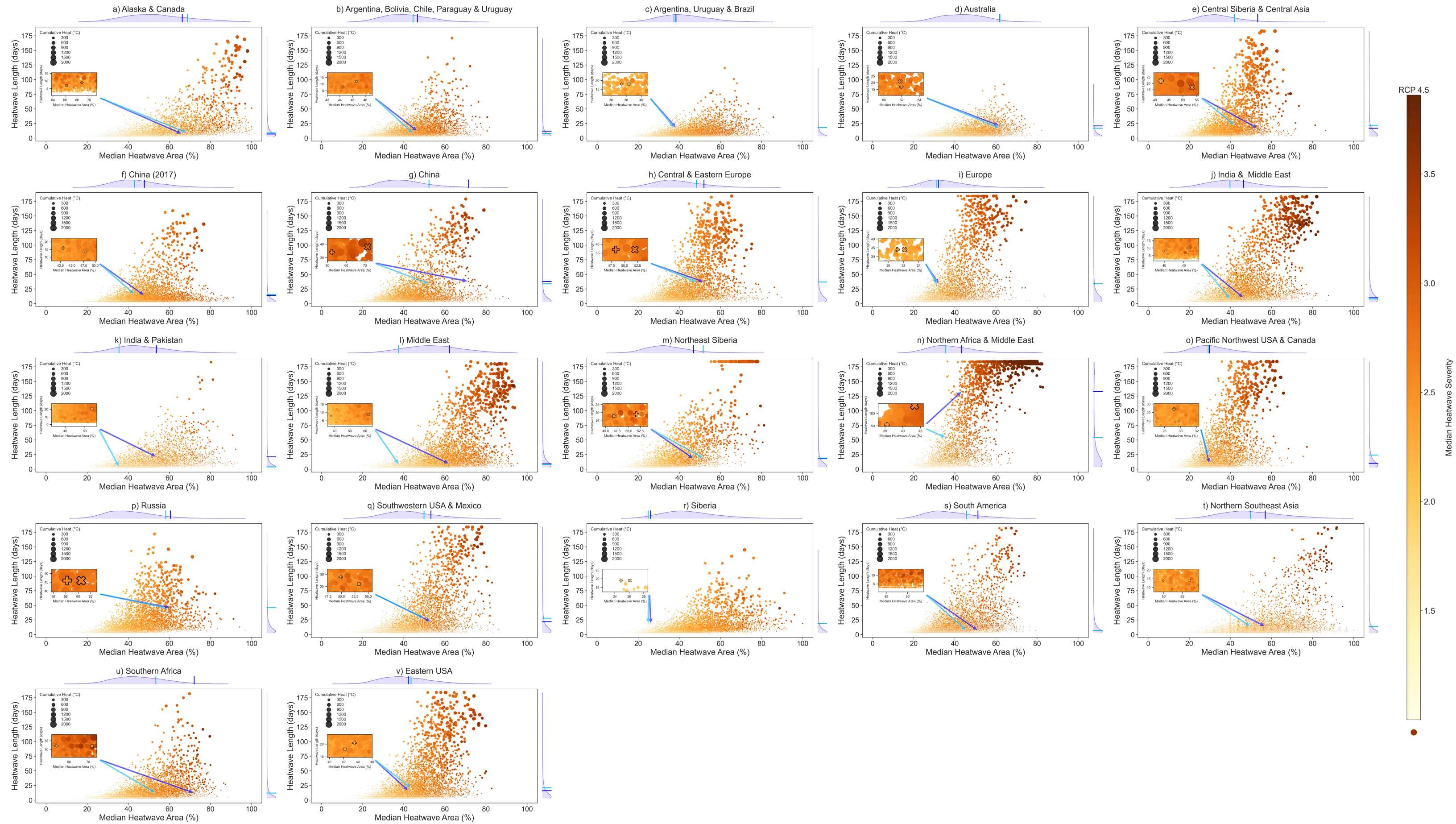

a) Alaska & Canada
b) Argentina, Bolivia, Chile, Paraguay & Uruguay
c) Argentina, Uruguay & Brazil
d) Australia
e) Central Siberia & Central Asia
f) China (2017)
g) China
h) Central & Eastern Europe
i) Europe
j) India & Middle East
k) India & Pakistan
l) Middle East
m) Northeast Siberia
n) Northern Africa & Middle East
o) Pacific Northwest USA & Canada
p) Russia
q) Southwestern USA & Mexico
r) Siberia
s) South America
t) Northern Southeast Asia
u) Southern Africa
v) Eastern USA
Heatwave Length (days)
Median Heatwave Area (%)
Cumulative Heat (°C)
300
600
900
1200
1500
2000
RCP 4.5
Median Heatwave Severity

Supplementary Figure 37: Multi-HW-characteristic figure according to RCP 8.5 CMIP5 multi-model ensemble (MME) (Tx90p) - Each panel shows regional projections according to RCP 8.5 model runs in the CMIP5 multi-model ensemble (MME) of four combined heatwave characteristics for each projected heatwave in the ensemble between 2020 and 2100. Each dot represents an individual heatwave in the projections, characterized by Median Heatwave Area (%) (x-axis) as latitude-weighted percentage of grid boxes affected by extreme heat, Heatwave Length (days) (y-axis) in days, Median Heatwave Severity (color), and Cumulative Heat (°C) (dot size). The characteristics of the observed event according to the Berkeley Earth observational data (x symbol) and the ERA5 reanalysis data (+ symbol), respectively, are pointed to by an arrow and a figure inset shows a zoom-in on this figure section. The figure inset shows the observed heatwave in the two reference data sets and projections in the indicated section of the respective sub-plot. It facilitates readability of color and size of the reference values and comparison to each other and the neighboring projections. Note that the zoom-in causes the dots to overlap more in the inset than in the main sub-plot. The univariate kernel density estimates along the x- (Median Heatwave Area (%)) and y-axis (Heatwave Length (days)) are on the top and right side of the subplots, respectively, with vertical lines in dark blue (Berkeley Earth) and light blue (ERA5) indicating the observed event.

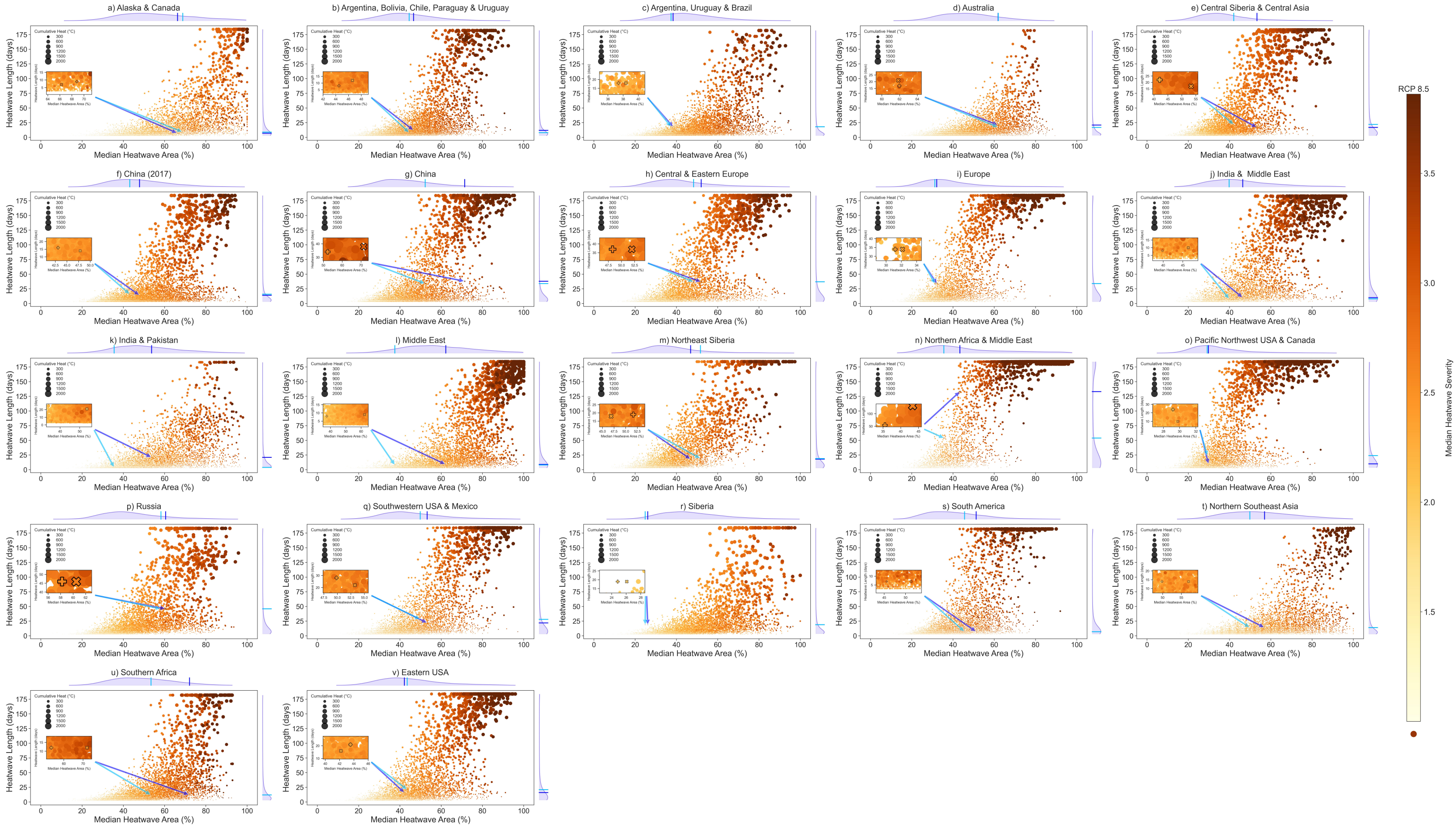
a) Alaska & Canada
b) Argentina, Bolivia, Chile, Paraguay & Uruguay
c) Argentina, Uruguay & Brazil
d) Australia
e) Central Siberia & Central Asia
f) China (2017)
g) China
h) Central & Eastern Europe
i) Europe
j) India & Middle East
k) India & Pakistan
l) Middle East
m) Northeast Siberia
n) Northern Africa & Middle East
o) Pacific Northwest USA & Canada
p) Russia
q) Southwestern USA & Mexico
r) Siberia
s) South America
t) Northern Southeast Asia
u) Southern Africa
v) Eastern USA
Cumulative Heat (°C)
300
600
900
1200
1500
2000
Heatwave Length (days)
Median Heatwave Area (%)
RCP 8.5
Median Heatwave Severity

Supplementary Figure 38: Multi-HW-characteristic figure according to RCP 2.6 CMIP5 multi-model ensemble (MME) (Tx90p) - Each panel shows regional projections according to RCP 2.6 model runs in the CMIP5 multi-model ensemble (MME) of four combined heatwave characteristics for each projected heatwave in the ensemble between 2020 and 2100. Each dot represents an individual heatwave in the projections, characterized by Maximum Heatwave Area (%) (x-axis) as latitude-weighted percentage of grid boxes affected by extreme heat, Heatwave Length (days) (y-axis) in days, Maximum Heatwave Severity (color), and Cumulative Heat (°C) (dot size). The characteristics of the observed event according to the Berkeley Earth observational data (x symbol) and the ERA5 reanalysis data (+ symbol), respectively, are pointed to by an arrow and a figure inset shows a zoom-in on this figure section. The figure inset shows the observed heatwave in the two reference data sets and projections in the indicated section of the respective sub-plot. It facilitates readability of color and size of the reference values and comparison to each other and the neighboring projections. Note that the zoom-in causes the dots to overlap more in the inset than in the main sub-plot. The univariate kernel density estimates along the x- (Maximum Heatwave Area (%)) and y-axis (Heatwave Length (days)) are on the top and right side of the subplots, respectively, with vertical lines in dark blue (Berkeley Earth) and light blue (ERA5) indicating the observed event.

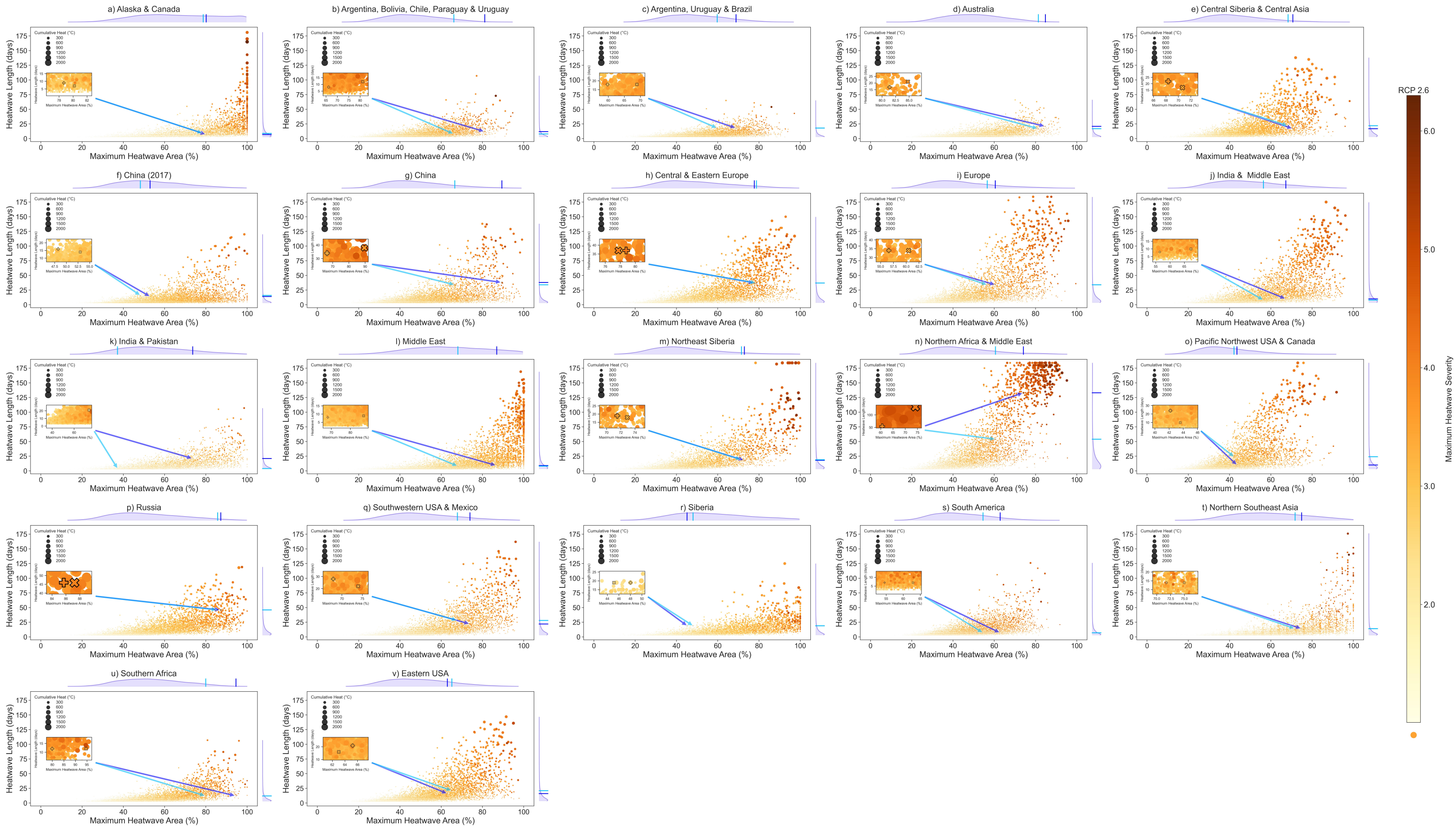

a) Alaska & Canada
b) Argentina, Bolivia, Chile, Paraguay & Uruguay
c) Argentina, Uruguay & Brazil
d) Australia
e) Central Siberia & Central Asia
f) China (2017)
g) China
h) Central & Eastern Europe
i) Europe
j) India & Middle East
k) India & Pakistan
l) Middle East
m) Northeast Siberia
n) Northern Africa & Middle East
o) Pacific Northwest USA & Canada
p) Russia
q) Southwestern USA & Mexico
r) Siberia
s) South America
t) Northern Southeast Asia
u) Southern Africa
v) Eastern USA
Heatwave Length (days)
Maximum Heatwave Area (%)
Cumulative Heat (°C)
300
600
900
1200
1500
2000
RCP 2.6
Maximum Heatwave Severity

Supplementary Figure 39: Multi-HW-characteristic figure according to RCP 4.5 CMIP5 multi-model ensemble (MME) (Tx90p) - Each panel shows regional projections according to RCP 4.5 model runs in the CMIP5 multi-model ensemble (MME) of four combined heatwave characteristics for each projected heatwave in the ensemble between 2020 and 2100. Each dot represents an individual heatwave in the projections, characterized by Maximum Heatwave Area (%) (x-axis) as latitude-weighted percentage of grid boxes affected by extreme heat, Heatwave Length (days) (y-axis) in days, Maximum Heatwave Severity (color), and Cumulative Heat (°C) (dot size). The characteristics of the observed event according to the Berkeley Earth observational data (x symbol) and the ERA5 reanalysis data (+ symbol), respectively, are pointed to by an arrow and a figure inset shows a zoom-in on this figure section. The figure inset shows the observed heatwave in the two reference data sets and projections in the indicated section of the respective sub-plot. It facilitates readability of color and size of the reference values and comparison to each other and the neighboring projections. Note that the zoom-in causes the dots to overlap more in the inset than in the main sub-plot. The univariate kernel density estimates along the x- (Maximum Heatwave Area (%)) and y-axis (Heatwave Length (days)) are on the top and right side of the subplots, respectively, with vertical lines in dark blue (Berkeley Earth) and light blue (ERA5) indicating the observed event.

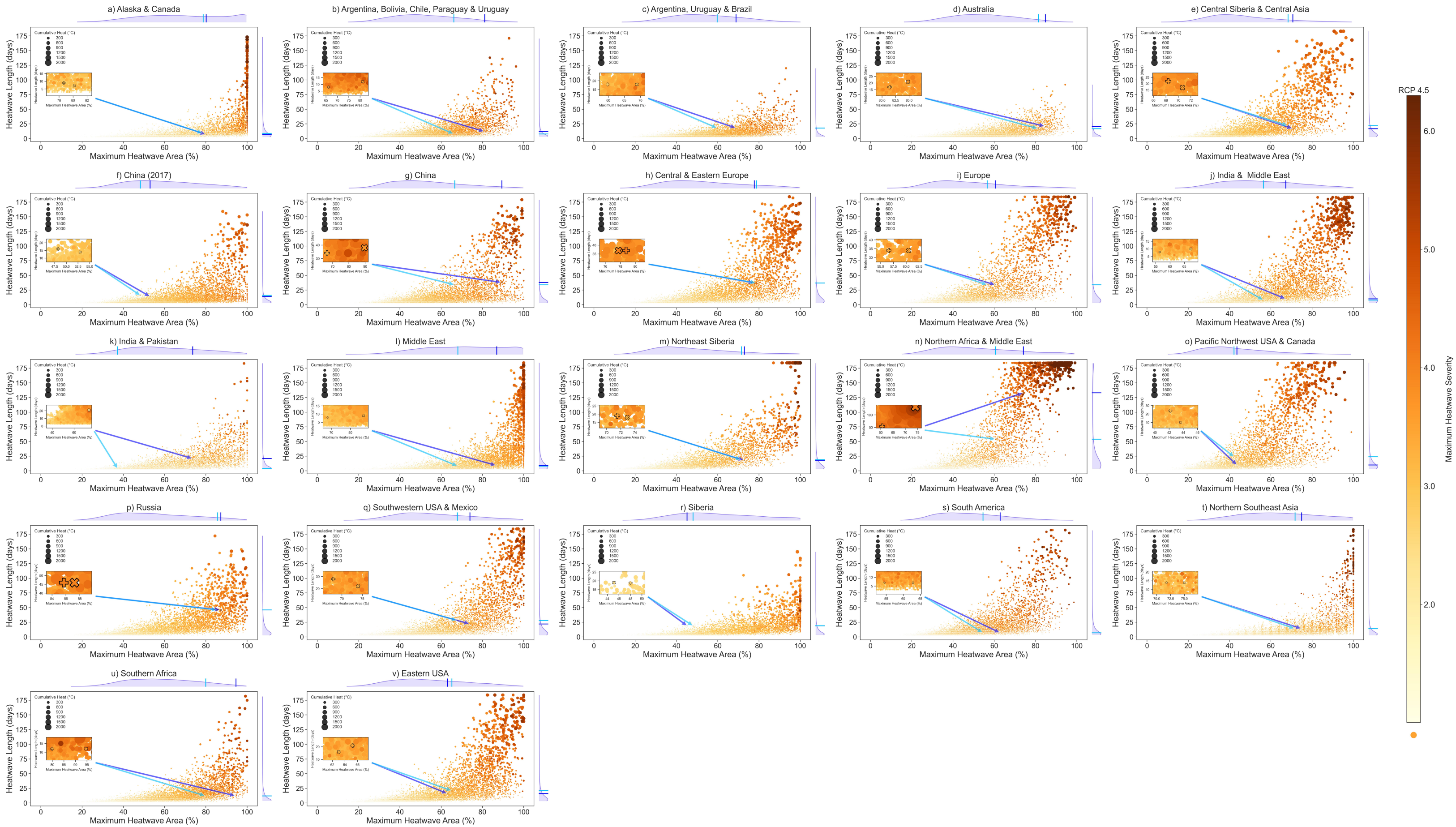
a) Alaska & Canada
b) Argentina, Bolivia, Chile, Paraguay & Uruguay
c) Argentina, Uruguay & Brazil
d) Australia
e) Central Siberia & Central Asia
f) China (2017)
g) China
h) Central & Eastern Europe
i) Europe
j) India & Middle East
k) India & Pakistan
l) Middle East
m) Northeast Siberia
n) Northern Africa & Middle East
o) Pacific Northwest USA & Canada
p) Russia
q) Southwestern USA & Mexico
r) Siberia
s) South America
t) Northern Southeast Asia
u) Southern Africa
v) Eastern USA
Heatwave Length (days)
Maximum Heatwave Area (%)
Cumulative Heat (°C)
300
600
900
1200
1500
2000
RCP 4.5
Maximum Heatwave Severity

Supplementary Figure 40: Multi-HW-characteristic figure according to RCP 8.5 CMIP5 multi-model ensemble (MME) (Tx90p) - Each panel shows regional projections according to RCP 8.5 model runs in the CMIP5 multi-model ensemble (MME) of four combined heatwave characteristics for each projected heatwave in the ensemble between 2020 and 2100. Each dot represents an individual heatwave in the projections, characterized by Maximum Heatwave Area (%) (x-axis) as latitude-weighted percentage of grid boxes affected by extreme heat, Heatwave Length (days) (y-axis) in days, Maximum Heatwave Severity (color), and Cumulative Heat (°C) (dot size). The characteristics of the observed event according to the Berkeley Earth observational data (x symbol) and the ERA5 reanalysis data (+ symbol), respectively, are pointed to by an arrow and a figure inset shows a zoom-in on this figure section. The figure inset shows the observed heatwave in the two reference data sets and projections in the indicated section of the respective sub-plot. It facilitates readability of color and size of the reference values and comparison to each other and the neighboring projections. Note that the zoom-in causes the dots to overlap more in the inset than in the main sub-plot. The univariate kernel density estimates along the x- (Maximum Heatwave Area (%)) and y-axis (Heatwave Length (days)) are on the top and right side of the subplots, respectively, with vertical lines in dark blue (Berkeley Earth) and light blue (ERA5) indicating the observed event.

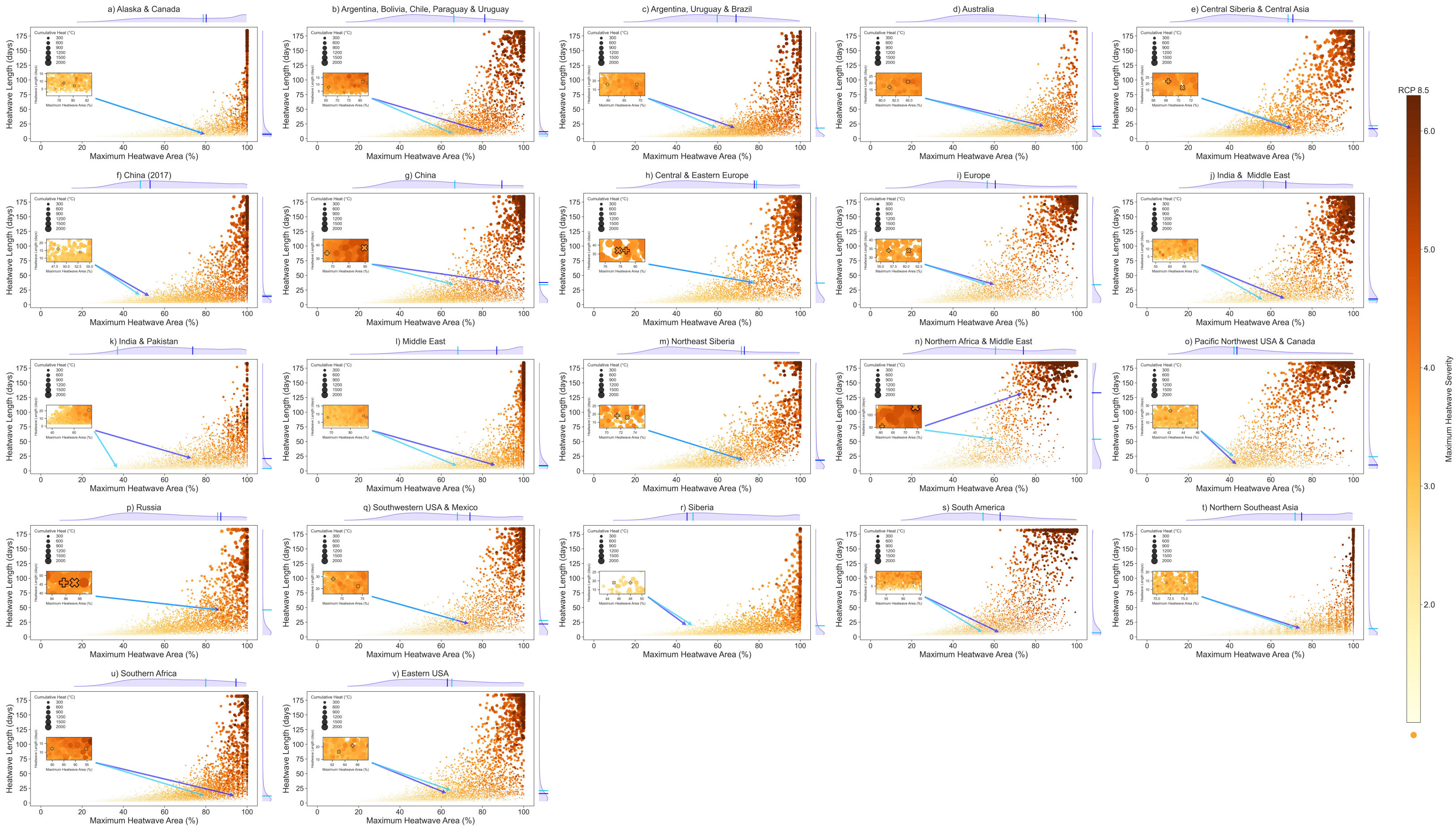
a) Alaska & Canada
b) Argentina, Bolivia, Chile, Paraguay & Uruguay
c) Argentina, Uruguay & Brazil
d) Australia
e) Central Siberia & Central Asia
f) China (2017)
g) China
h) Central & Eastern Europe
i) Europe
j) India & Middle East
k) India & Pakistan
l) Middle East
m) Northeast Siberia
n) Northern Africa & Middle East
o) Pacific Northwest USA & Canada
p) Russia
q) Southwestern USA & Mexico
r) Siberia
s) South America
t) Northern Southeast Asia
u) Southern Africa
v) Eastern USA
Heatwave Length (days)
Maximum Heatwave Area (%)
Cumulative Heat (°C)
300
600
900
1200
1500
2000
RCP 8.5
Maximum Heatwave Severity

Supplementary Figure 41: Kernel Density Estimates (KDE) across Decades for Cumulative Heat (°C) in SSP1-2.6 CMIP6 multi-model ensemble (MME) (Tx90p) - Kernel Density Estimates (KDE) of Cumulative Heat (°C) for different Decades in the CMIP6 multi-model ensemble (MME). Area under each curve is normalized to 1. Cumulative Heat (°C) of the observed event is indicated by vertical lines in dark blue (Berkeley Earth) and light blue (ERA5).

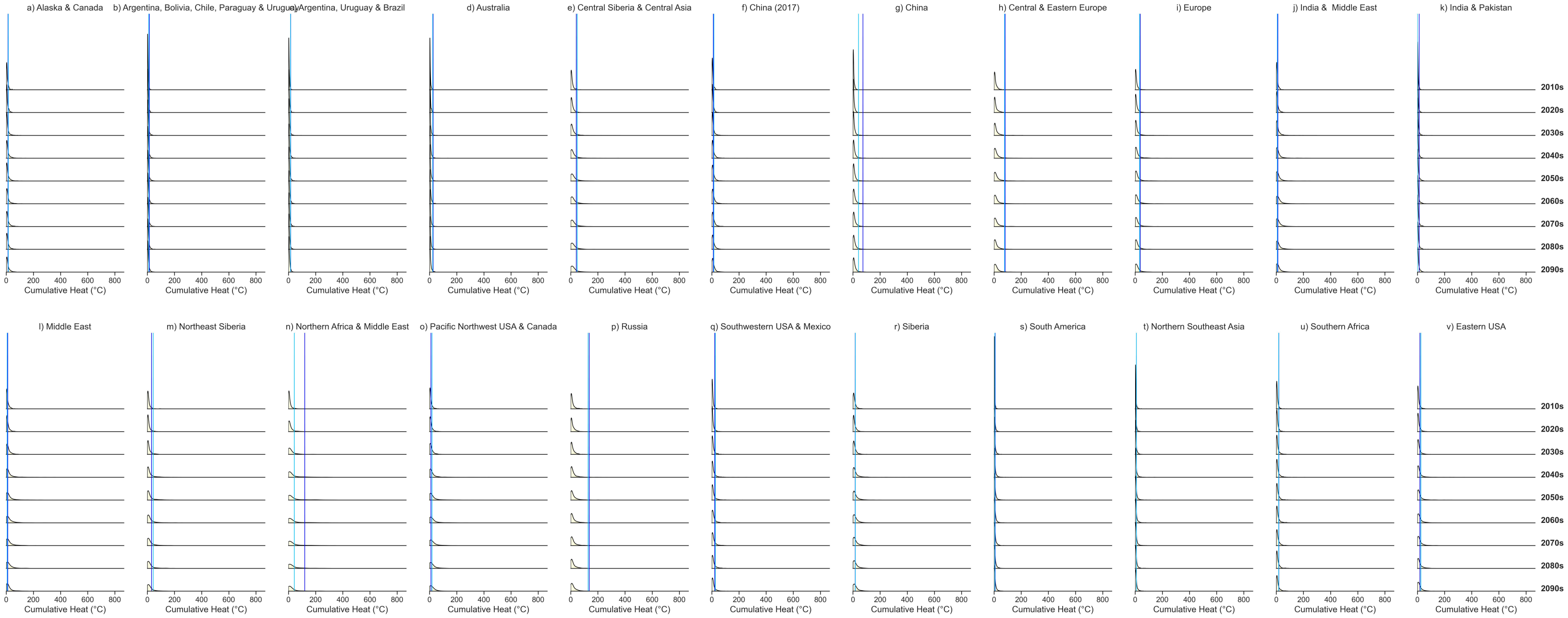
a) Alaska & Canada
b) Argentina, Bolivia, Chile, Paraguay & Uruguay
c) Argentina, Uruguay & Brazil
d) Australia
e) Central Siberia & Central Asia
f) China (2017)
g) China
h) Central & Eastern Europe
i) Europe
j) India & Middle East
k) India & Pakistan
l) Middle East
m) Northeast Siberia
n) Northern Africa & Middle East
o) Pacific Northwest USA & Canada
p) Russia
q) Southwestern USA & Mexico
r) Siberia
s) South America
t) Northern Southeast Asia
u) Southern Africa
v) Eastern USA
2010s
2020s
2030s
2040s
2050s
2060s
2070s
2080s
2090s
0
200
400
600
800
Cumulative Heat (°C)

Supplementary Figure 42: Kernel Density Estimates (KDE) across Decades for Cumulative Heat (°C) in SSP2-4.5 CMIP6 multi-model ensemble (MME) (Tx90p) - Kernel Density Estimates (KDE) of Cumulative Heat (°C) for different Decades in the CMIP6 multi-model ensemble (MME). Area under each curve is normalized to 1. Cumulative Heat (°C) of the observed event is indicated by vertical lines in dark blue (Berkeley Earth) and light blue (ERA5).

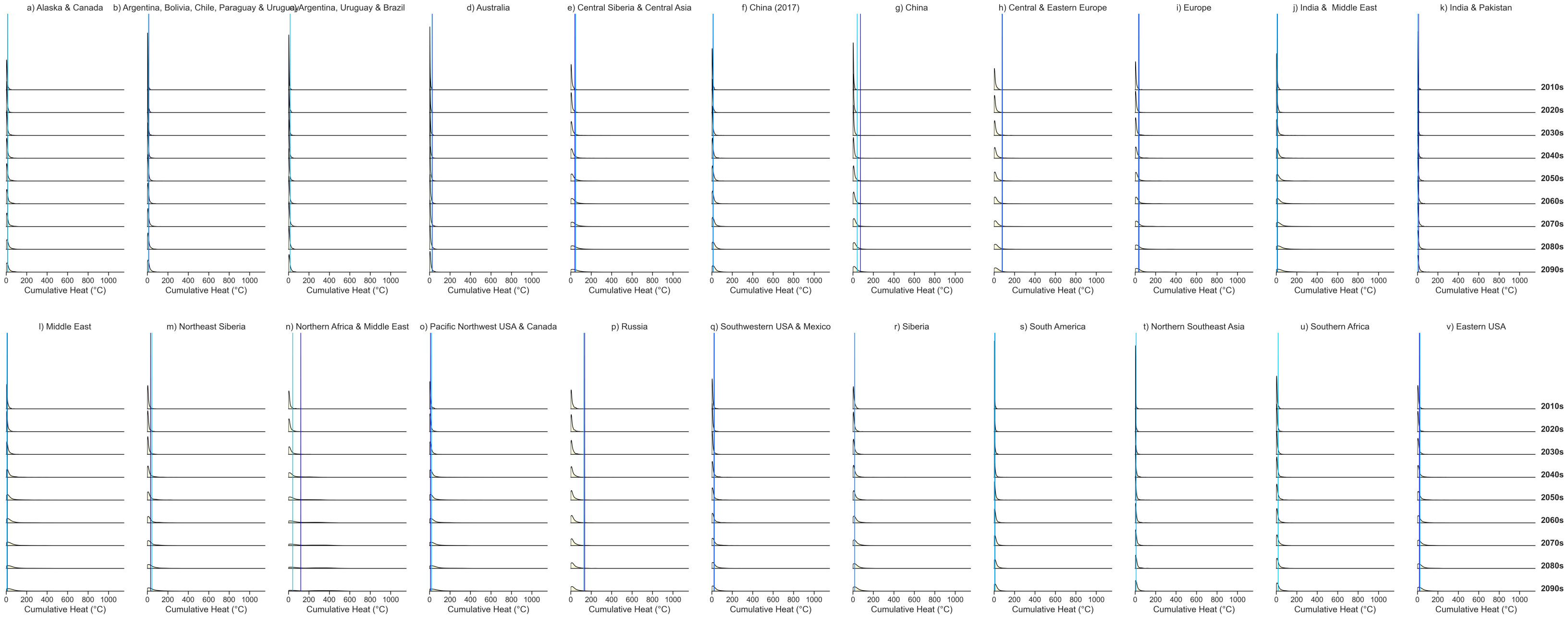
a) Alaska & Canada
b) Argentina, Bolivia, Chile, Paraguay & Uruguay
c) Argentina, Uruguay & Brazil
d) Australia
e) Central Siberia & Central Asia
f) China (2017)
g) China
h) Central & Eastern Europe
i) Europe
j) India & Middle East
k) India & Pakistan
l) Middle East
m) Northeast Siberia
n) Northern Africa & Middle East
o) Pacific Northwest USA & Canada
p) Russia
q) Southwestern USA & Mexico
r) Siberia
s) South America
t) Northern Southeast Asia
u) Southern Africa
v) Eastern USA
2010s
2020s
2030s
2040s
2050s
2060s
2070s
2080s
2090s
0 200 400 600 800 1000
Cumulative Heat (°C)

Supplementary Figure 43: Kernel Density Estimates (KDE) across Decades for Cumulative Heat (°C) in SSP3-7.0 CMIP6 multi-model ensemble (MME) (Tx90p) - Kernel Density Estimates (KDE) of Cumulative Heat (°C) for different Decades in the CMIP6 multi-model ensemble (MME). Area under each curve is normalized to 1. Cumulative Heat (°C) of the observed event is indicated by vertical lines in dark blue (Berkeley Earth) and light blue (ERA5).

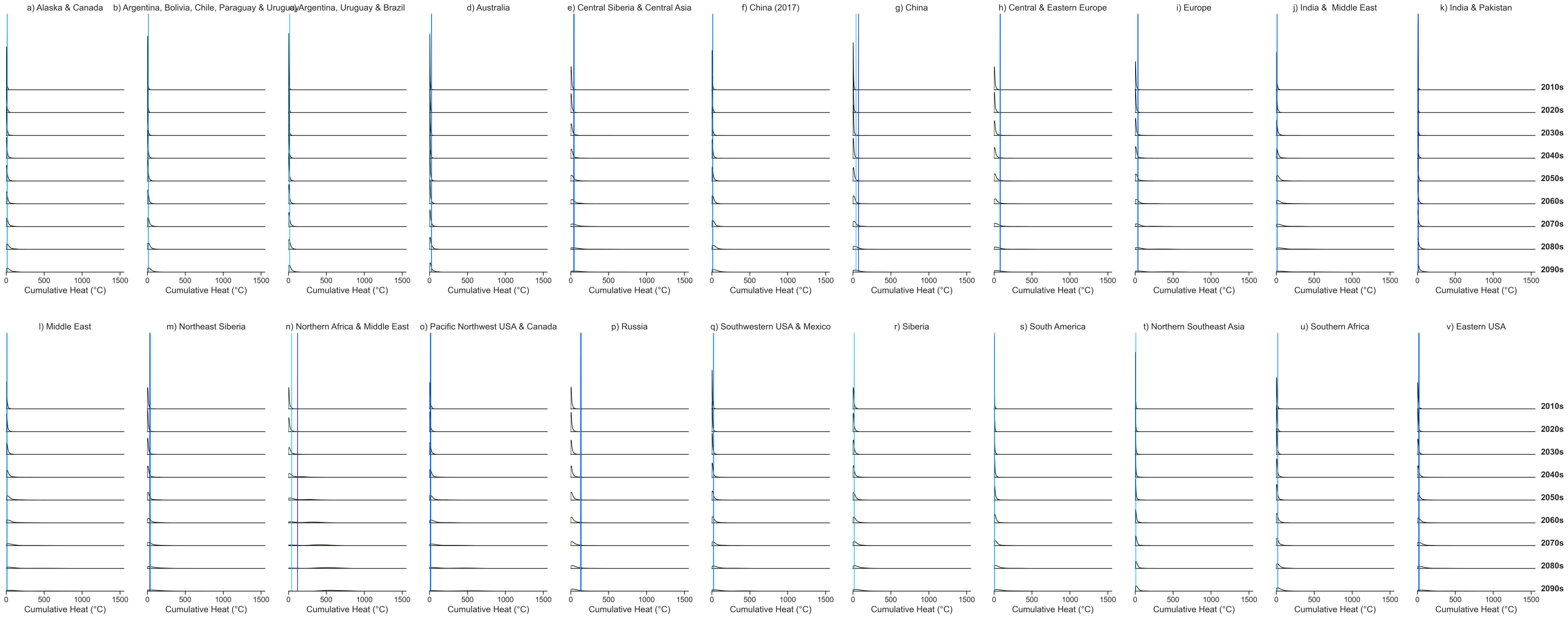

a) Alaska & Canada
b) Argentina, Bolivia, Chile, Paraguay & Uruguay
c) Argentina, Uruguay & Brazil
d) Australia
e) Central Siberia & Central Asia
f) China (2017)
g) China
h) Central & Eastern Europe
i) Europe
j) India & Middle East
k) India & Pakistan
l) Middle East
m) Northeast Siberia
n) Northern Africa & Middle East
o) Pacific Northwest USA & Canada
p) Russia
q) Southwestern USA & Mexico
r) Siberia
s) South America
t) Northern Southeast Asia
u) Southern Africa
v) Eastern USA
2010s
2020s
2030s
2040s
2050s
2060s
2070s
2080s
2090s
0
500
1000
1500
Cumulative Heat (°C)

Supplementary Figure 44: Kernel Density Estimates (KDE) across Decades for Cumulative Heat (°C) in SSP5-8.5 CMIP6 multi-model ensemble (MME) (Tx90p) - Kernel Density Estimates (KDE) of Cumulative Heat (°C) for different Decades in the CMIP6 multi-model ensemble (MME). Area under each curve is normalized to 1. Cumulative Heat (°C) of the observed event is indicated by vertical lines in dark blue (Berkeley Earth) and light blue (ERA5).

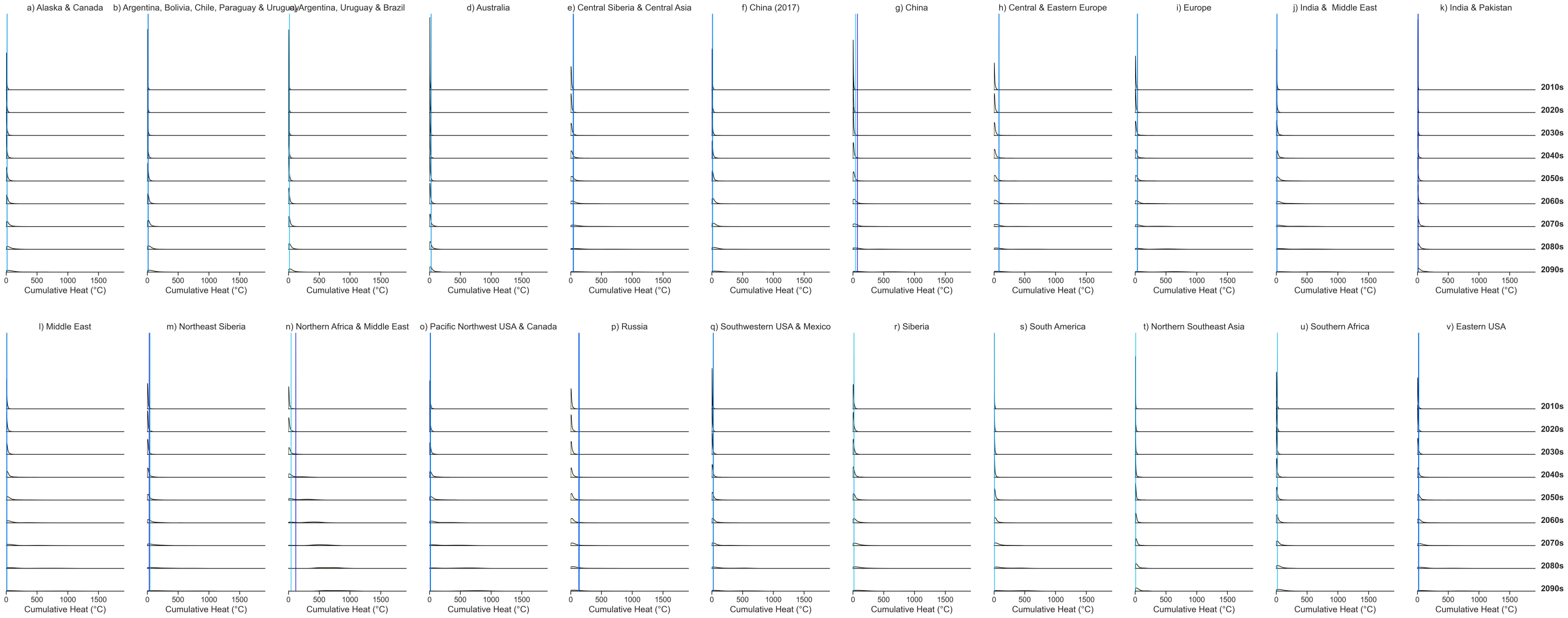
a) Alaska & Canada
b) Argentina, Bolivia, Chile, Paraguay & Uruguay
c) Argentina, Uruguay & Brazil
d) Australia
e) Central Siberia & Central Asia
f) China (2017)
g) China
h) Central & Eastern Europe
i) Europe
j) India & Middle East
k) India & Pakistan
l) Middle East
m) Northeast Siberia
n) Northern Africa & Middle East
o) Pacific Northwest USA & Canada
p) Russia
q) Southwestern USA & Mexico
r) Siberia
s) South America
t) Northern Southeast Asia
u) Southern Africa
v) Eastern USA
2010s
2020s
2030s
2040s
2050s
2060s
2070s
2080s
2090s
0
500
1000
1500
Cumulative Heat (°C)

Supplementary Figure 45: Kernel Density Estimates (KDE) across Decades for Heatwave Length (days) in SSP1-2.6 CMIP6 multi-model ensemble (MME) (Tx90p) - Kernel Density Estimates (KDE) of Heatwave Length (days) for different Decades in the CMIP6 multi-model ensemble (MME). Area under each curve is normalized to 1. Heatwave Length (days) of the observed event is indicated by vertical lines in dark blue (Berkeley Earth) and light blue (ERA5).

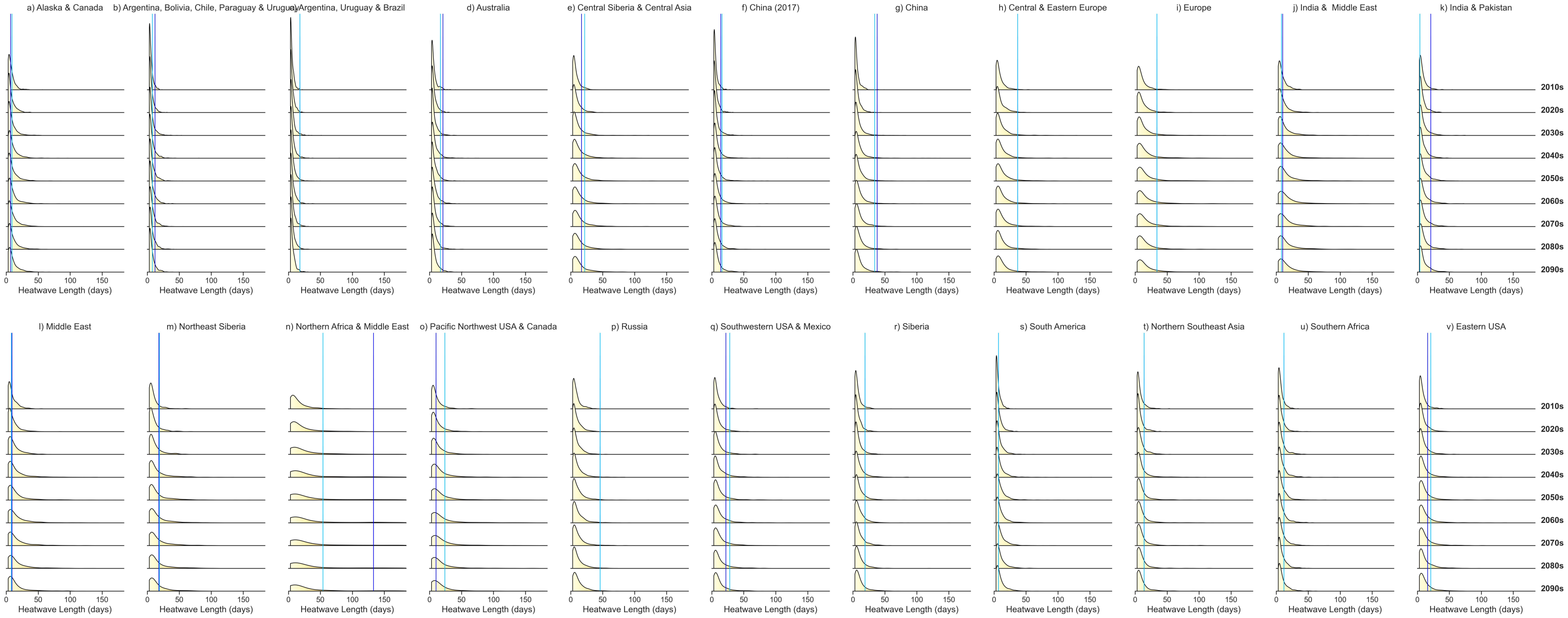

a) Alaska & Canada
b) Argentina, Bolivia, Chile, Paraguay & Uruguay
c) Argentina, Uruguay & Brazil
d) Australia
e) Central Siberia & Central Asia
f) China (2017)
g) China
h) Central & Eastern Europe
i) Europe
j) India & Middle East
k) India & Pakistan
l) Middle East
m) Northeast Siberia
n) Northern Africa & Middle East
o) Pacific Northwest USA & Canada
p) Russia
q) Southwestern USA & Mexico
r) Siberia
s) South America
t) Northern Southeast Asia
u) Southern Africa
v) Eastern USA
2010s
2020s
2030s
2040s
2050s
2060s
2070s
2080s
2090s
0
50
100
150
Heatwave Length (days)

Supplementary Figure 46: Kernel Density Estimates (KDE) across Decades for Heatwave Length (days) in SSP2-4.5 CMIP6 multi-model ensemble (MME) (Tx90p) - Kernel Density Estimates (KDE) of Heatwave Length (days) for different Decades in the CMIP6 multi-model ensemble (MME). Area under each curve is normalized to 1. Heatwave Length (days) of the observed event is indicated by vertical lines in dark blue (Berkeley Earth) and light blue (ERA5).

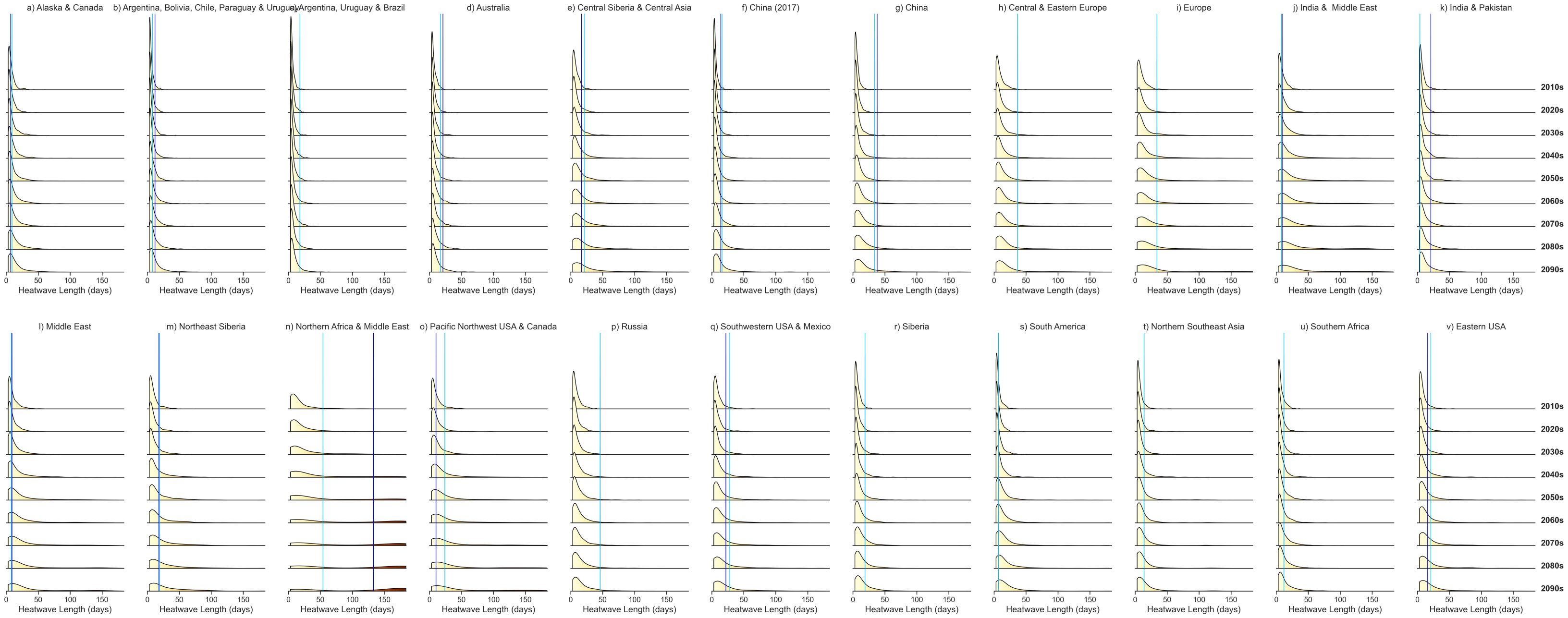

a) Alaska & Canada
b) Argentina, Bolivia, Chile, Paraguay & Uruguay
c) Argentina, Uruguay & Brazil
d) Australia
e) Central Siberia & Central Asia
f) China (2017)
g) China
h) Central & Eastern Europe
i) Europe
j) India & Middle East
k) India & Pakistan
l) Middle East
m) Northeast Siberia
n) Northern Africa & Middle East
o) Pacific Northwest USA & Canada
p) Russia
q) Southwestern USA & Mexico
r) Siberia
s) South America
t) Northern Southeast Asia
u) Southern Africa
v) Eastern USA
2010s
2020s
2030s
2040s
2050s
2060s
2070s
2080s
2090s
0
50
100
150
Heatwave Length (days)

Supplementary Figure 47: Kernel Density Estimates (KDE) across Decades for Heatwave Length (days) in SSP3-7.0 CMIP6 multi-model ensemble (MME) (Tx90p) - Kernel Density Estimates (KDE) of Heatwave Length (days) for different Decades in the CMIP6 multi-model ensemble (MME). Area under each curve is normalized to 1. Heatwave Length (days) of the observed event is indicated by vertical lines in dark blue (Berkeley Earth) and light blue (ERA5).

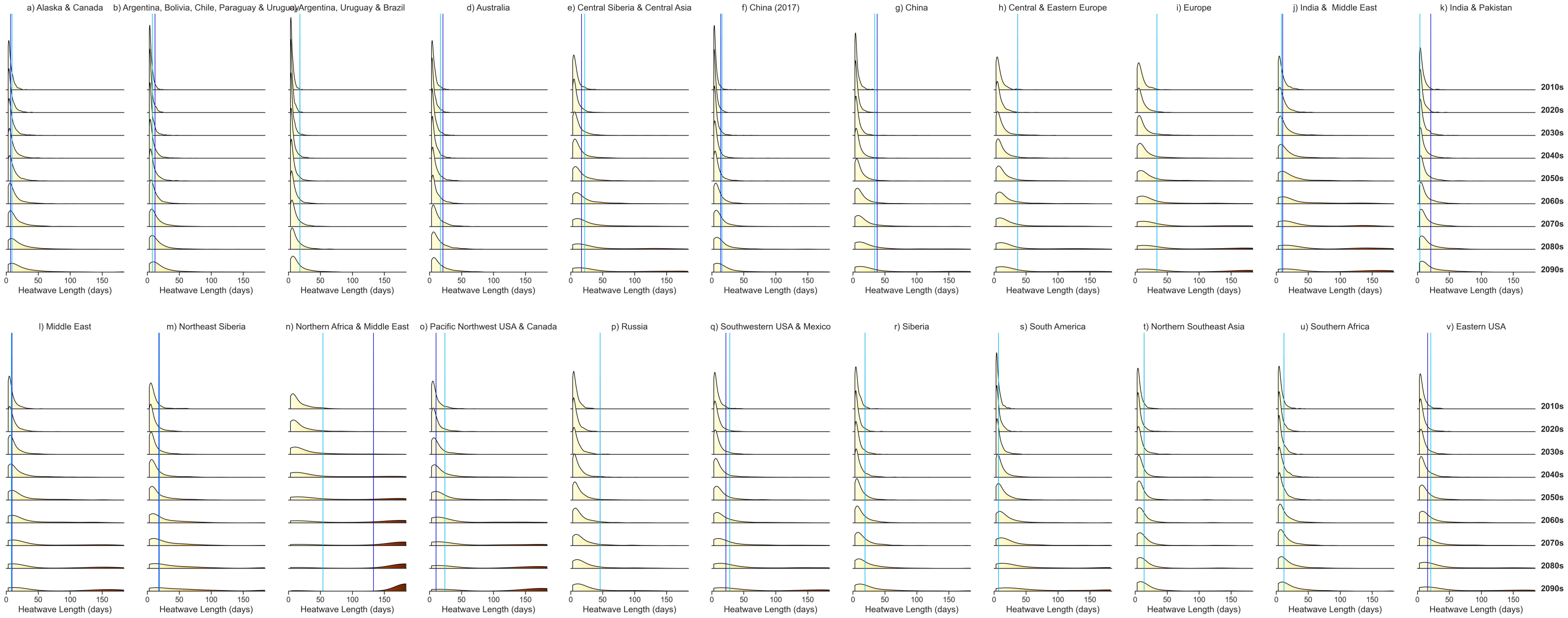
a) Alaska & Canada
b) Argentina, Bolivia, Chile, Paraguay & Uruguay
c) Argentina, Uruguay & Brazil
d) Australia
e) Central Siberia & Central Asia
f) China (2017)
g) China
h) Central & Eastern Europe
i) Europe
j) India & Middle East
k) India & Pakistan
l) Middle East
m) Northeast Siberia
n) Northern Africa & Middle East
o) Pacific Northwest USA & Canada
p) Russia
q) Southwestern USA & Mexico
r) Siberia
s) South America
t) Northern Southeast Asia
u) Southern Africa
v) Eastern USA
2010s
2020s
2030s
2040s
2050s
2060s
2070s
2080s
2090s
0
50
100
150
Heatwave Length (days)

Supplementary Figure 48: Kernel Density Estimates (KDE) across Decades for Heatwave Length (days) in SSP5-8.5 CMIP6 multi-model ensemble (MME) (Tx90p) - Kernel Density Estimates (KDE) of Heatwave Length (days) for different Decades in the CMIP6 multi-model ensemble (MME). Area under each curve is normalized to 1. Heatwave Length (days) of the observed event is indicated by vertical lines in dark blue (Berkeley Earth) and light blue (ERA5).

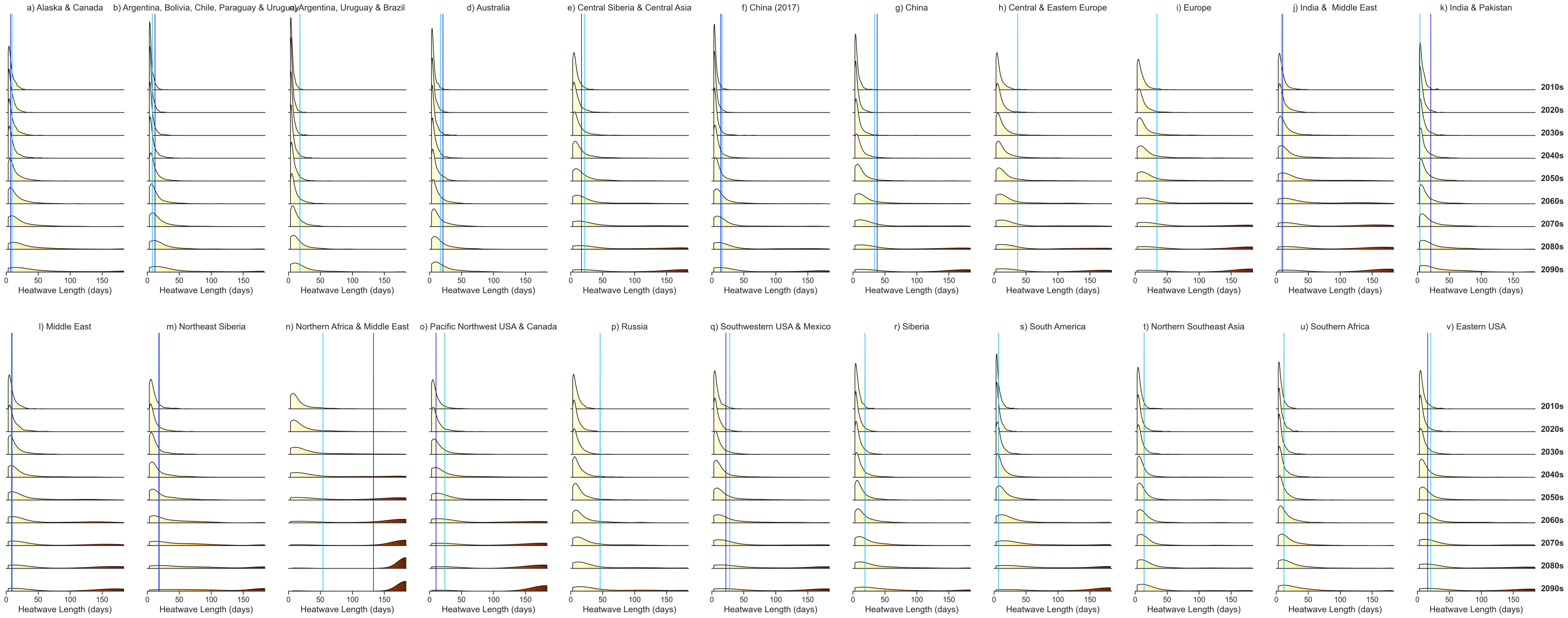

a) Alaska & Canada
b) Argentina, Bolivia, Chile, Paraguay & Uruguay
c) Argentina, Uruguay & Brazil
d) Australia
e) Central Siberia & Central Asia
f) China (2017)
g) China
h) Central & Eastern Europe
i) Europe
j) India & Middle East
k) India & Pakistan
l) Middle East
m) Northeast Siberia
n) Northern Africa & Middle East
o) Pacific Northwest USA & Canada
p) Russia
q) Southwestern USA & Mexico
r) Siberia
s) South America
t) Northern Southeast Asia
u) Southern Africa
v) Eastern USA
2010s
2020s
2030s
2040s
2050s
2060s
2070s
2080s
2090s
0 50 100 150
Heatwave Length (days)

Supplementary Figure 49: Kernel Density Estimates (KDE) across Decades for Median Heatwave Severity in SSP1-2.6 CMIP6 multi-model ensemble (MME) (Tx90p) - Kernel Density Estimates (KDE) of Median Heatwave Severity for different Decades in the CMIP6 multi-model ensemble (MME). Area under each curve is normalized to 1. Median Heatwave Severity of the observed event is indicated by vertical lines in dark blue (Berkeley Earth) and light blue (ERA5).

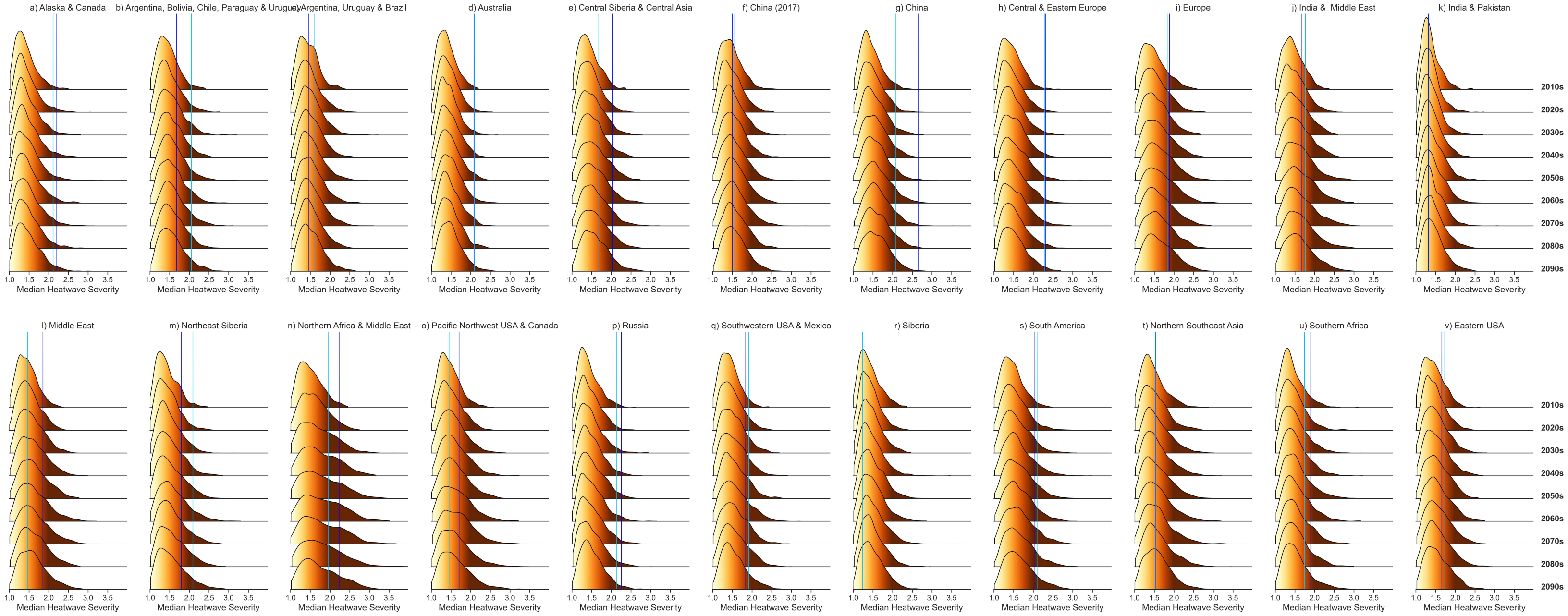
a) Alaska & Canada
b) Argentina, Bolivia, Chile, Paraguay & Uruguay
c) Argentina, Uruguay & Brazil
d) Australia
e) Central Siberia & Central Asia
f) China (2017)
g) China
h) Central & Eastern Europe
i) Europe
j) India & Middle East
k) India & Pakistan
l) Middle East
m) Northeast Siberia
n) Northern Africa & Middle East
o) Pacific Northwest USA & Canada
p) Russia
q) Southwestern USA & Mexico
r) Siberia
s) South America
t) Northern Southeast Asia
u) Southern Africa
v) Eastern USA
2010s
2020s
2030s
2040s
2050s
2060s
2070s
2080s
2090s
1.0 1.5 2.0 2.5 3.0 3.5
Median Heatwave Severity

Supplementary Figure 50: Kernel Density Estimates (KDE) across Decades for Median Heatwave Severity in SSP2-4.5 CMIP6 multi-model ensemble (MME) (Tx90p) - Kernel Density Estimates (KDE) of Median Heatwave Severity for different Decades in the CMIP6 multi-model ensemble (MME). Area under each curve is normalized to 1. Median Heatwave Severity of the observed event is indicated by vertical lines in dark blue (Berkeley Earth) and light blue (ERA5).

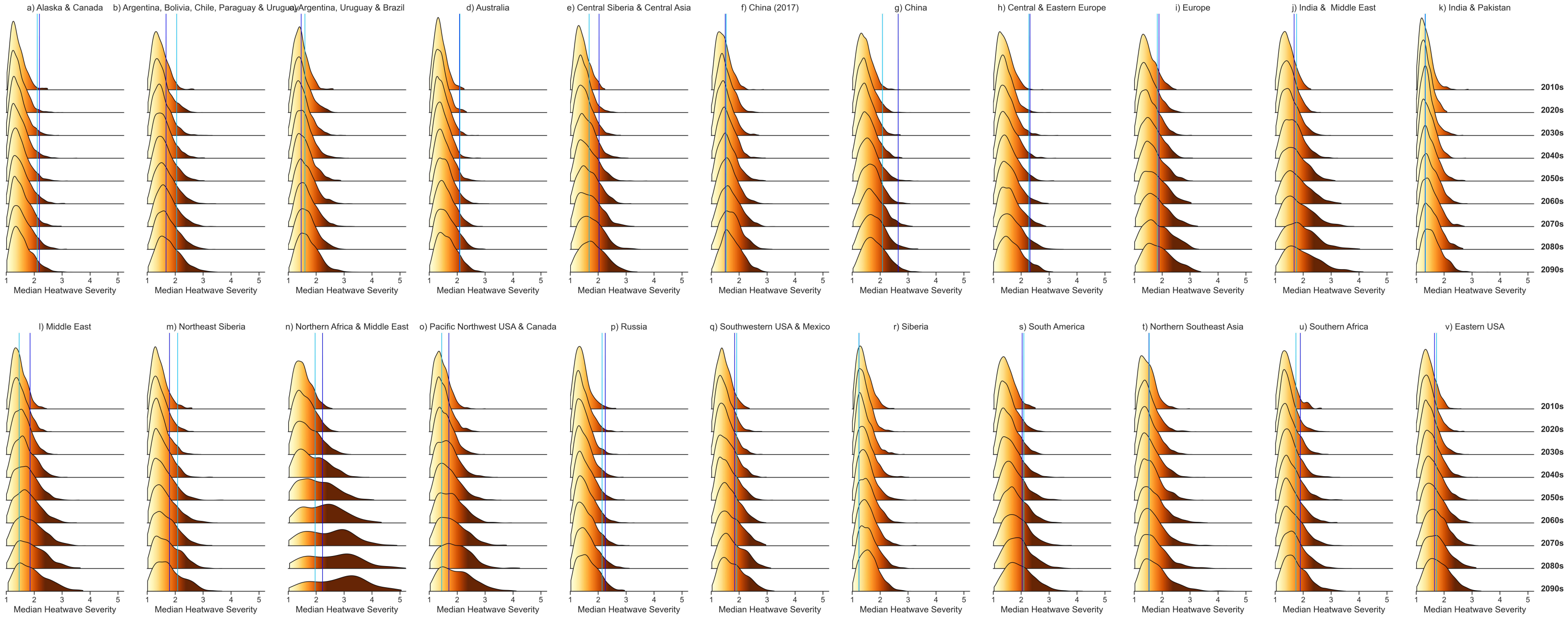

a) Alaska & Canada
b) Argentina, Bolivia, Chile, Paraguay & Uruguay
c) Argentina, Uruguay & Brazil
d) Australia
e) Central Siberia & Central Asia
f) China (2017)
g) China
h) Central & Eastern Europe
i) Europe
j) India & Middle East
k) India & Pakistan
l) Middle East
m) Northeast Siberia
n) Northern Africa & Middle East
o) Pacific Northwest USA & Canada
p) Russia
q) Southwestern USA & Mexico
r) Siberia
s) South America
t) Northern Southeast Asia
u) Southern Africa
v) Eastern USA
2010s
2020s
2030s
2040s
2050s
2060s
2070s
2080s
2090s
Median Heatwave Severity
1 2 3 4 5

Supplementary Figure 51: Kernel Density Estimates (KDE) across Decades for Median Heatwave Severity in SSP3-7.0 CMIP6 multi-model ensemble (MME) (Tx90p) - Kernel Density Estimates (KDE) of Median Heatwave Severity for different Decades in the CMIP6 multi-model ensemble (MME). Area under each curve is normalized to 1. Median Heatwave Severity of the observed event is indicated by vertical lines in dark blue (Berkeley Earth) and light blue (ERA5).

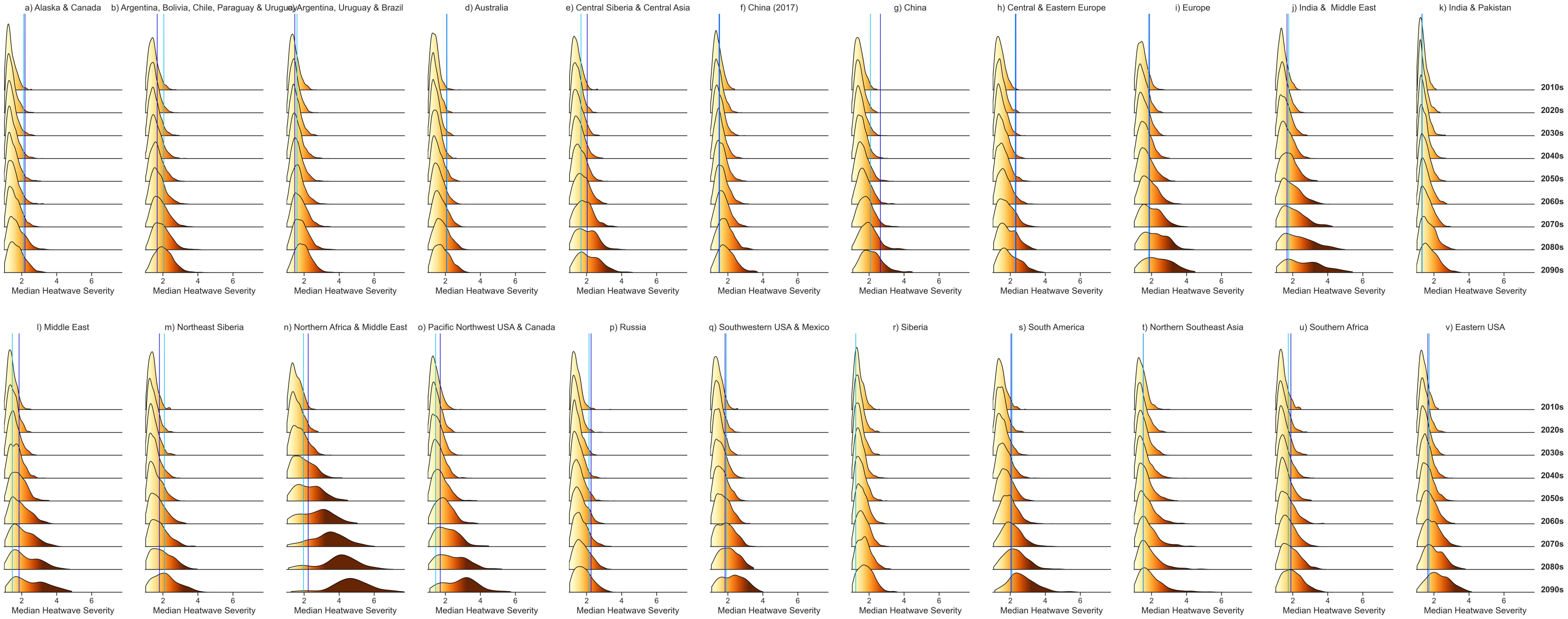

a) Alaska & Canada
b) Argentina, Bolivia, Chile, Paraguay & Uruguay
c) Argentina, Uruguay & Brazil
d) Australia
e) Central Siberia & Central Asia
f) China (2017)
g) China
h) Central & Eastern Europe
i) Europe
j) India & Middle East
k) India & Pakistan
l) Middle East
m) Northeast Siberia
n) Northern Africa & Middle East
o) Pacific Northwest USA & Canada
p) Russia
q) Southwestern USA & Mexico
r) Siberia
s) South America
t) Northern Southeast Asia
u) Southern Africa
v) Eastern USA
2010s
2020s
2030s
2040s
2050s
2060s
2070s
2080s
2090s
2
4
6
Median Heatwave Severity

Supplementary Figure 52: Kernel Density Estimates (KDE) across Decades for Median Heatwave Severity in SSP5-8.5 CMIP6 multi-model ensemble (MME) (Tx90p) - Kernel Density Estimates (KDE) of Median Heatwave Severity for different Decades in the CMIP6 multi-model ensemble (MME). Area under each curve is normalized to 1. Median Heatwave Severity of the observed event is indicated by vertical lines in dark blue (Berkeley Earth) and light blue (ERA5).

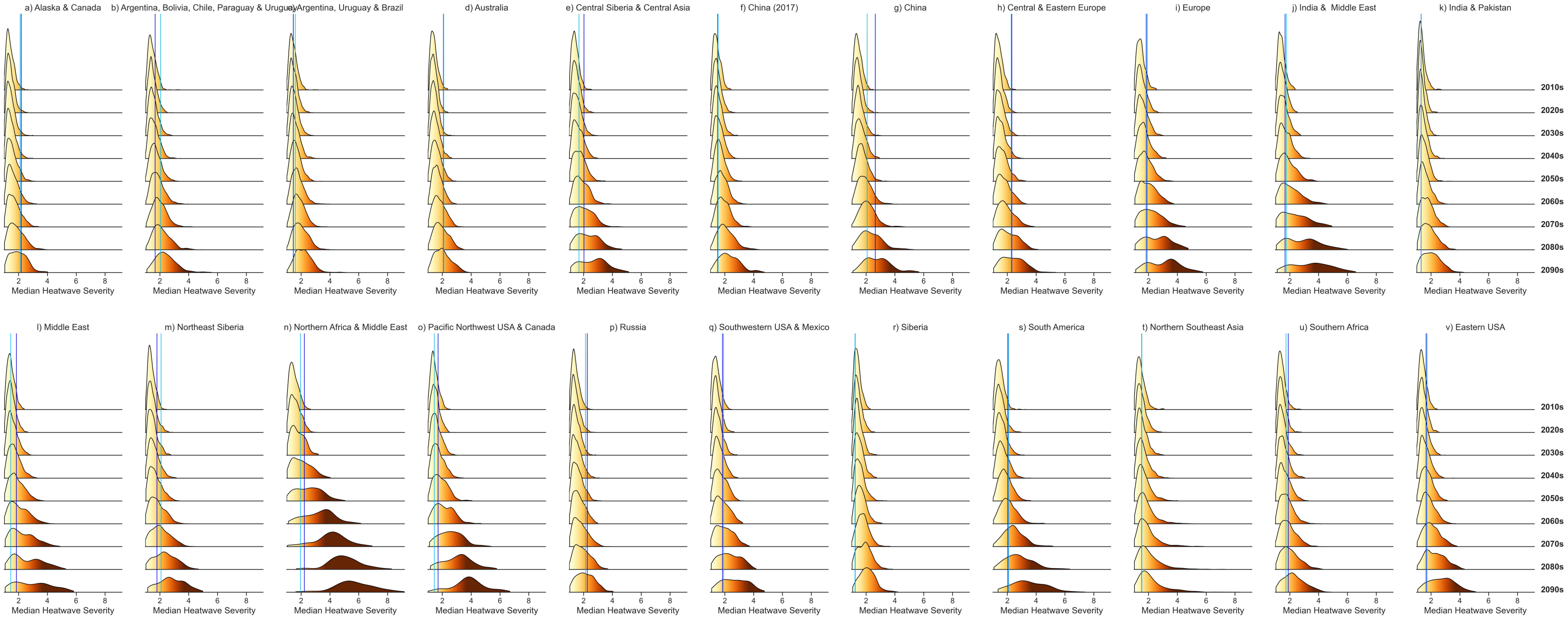

a) Alaska & Canada
b) Argentina, Bolivia, Chile, Paraguay & Uruguay
c) Argentina, Uruguay & Brazil
d) Australia
e) Central Siberia & Central Asia
f) China (2017)
g) China
h) Central & Eastern Europe
i) Europe
j) India & Middle East
k) India & Pakistan
l) Middle East
m) Northeast Siberia
n) Northern Africa & Middle East
o) Pacific Northwest USA & Canada
p) Russia
q) Southwestern USA & Mexico
r) Siberia
s) South America
t) Northern Southeast Asia
u) Southern Africa
v) Eastern USA
2010s
2020s
2030s
2040s
2050s
2060s
2070s
2080s
2090s
2 4 6 8
Median Heatwave Severity

Supplementary Figure 53: Kernel Density Estimates (KDE) across Decades for Maximum Heatwave Severity in SSP1-2.6 CMIP6 multi-model ensemble (MME) (Tx90p) - Kernel Density Estimates (KDE) of Maximum Heatwave Severity for different Decades in the CMIP6 multi-model ensemble (MME). Area under each curve is normalized to 1. Maximum Heatwave Severity of the observed event is indicated by vertical lines in dark blue (Berkeley Earth) and light blue (ERA5).

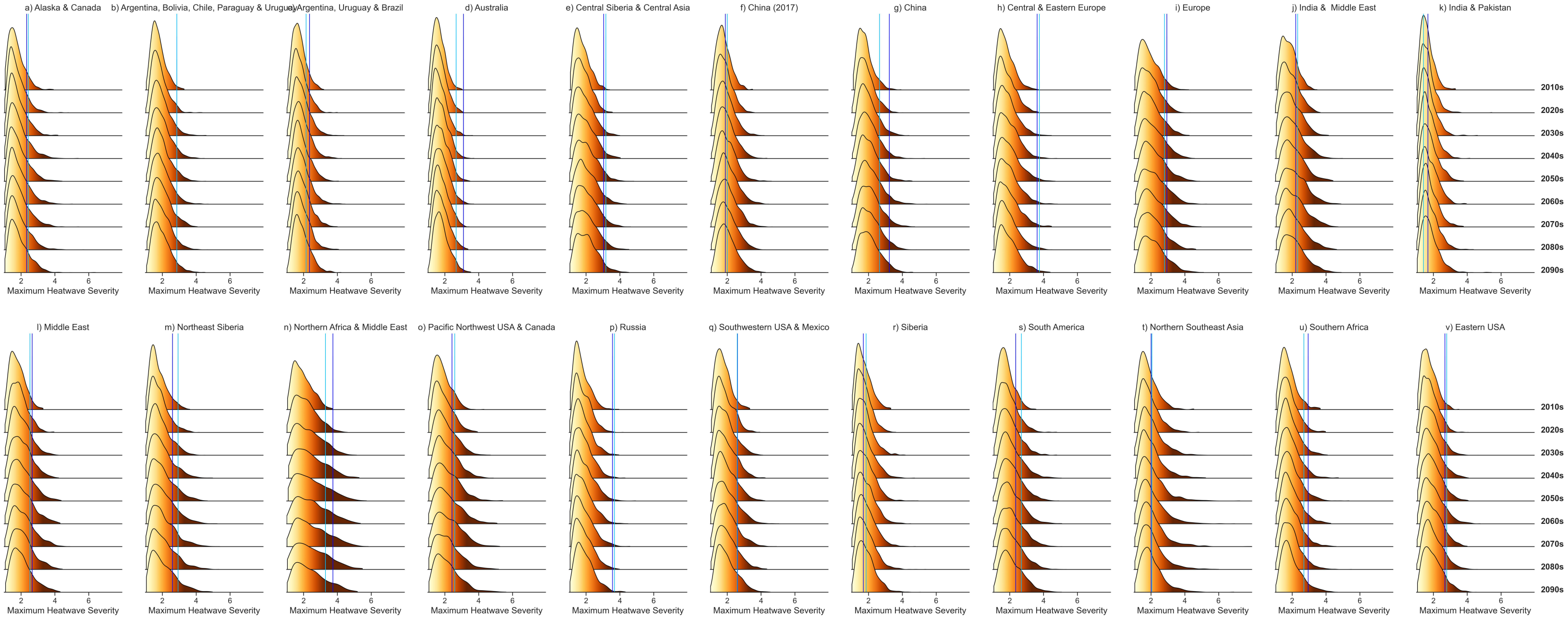

a) Alaska & Canada
b) Argentina, Bolivia, Chile, Paraguay & Uruguay
c) Argentina, Uruguay & Brazil
d) Australia
e) Central Siberia & Central Asia
f) China (2017)
g) China
h) Central & Eastern Europe
i) Europe
j) India & Middle East
k) India & Pakistan
l) Middle East
m) Northeast Siberia
n) Northern Africa & Middle East
o) Pacific Northwest USA & Canada
p) Russia
q) Southwestern USA & Mexico
r) Siberia
s) South America
t) Northern Southeast Asia
u) Southern Africa
v) Eastern USA
2010s
2020s
2030s
2040s
2050s
2060s
2070s
2080s
2090s
2
4
6
Maximum Heatwave Severity

Supplementary Figure 54: Kernel Density Estimates (KDE) across Decades for Maximum Heatwave Severity in SSP2-4.5 CMIP6 multi-model ensemble (MME) (Tx90p) - Kernel Density Estimates (KDE) of Maximum Heatwave Severity for different Decades in the CMIP6 multi-model ensemble (MME). Area under each curve is normalized to 1. Maximum Heatwave Severity of the observed event is indicated by vertical lines in dark blue (Berkeley Earth) and light blue (ERA5).

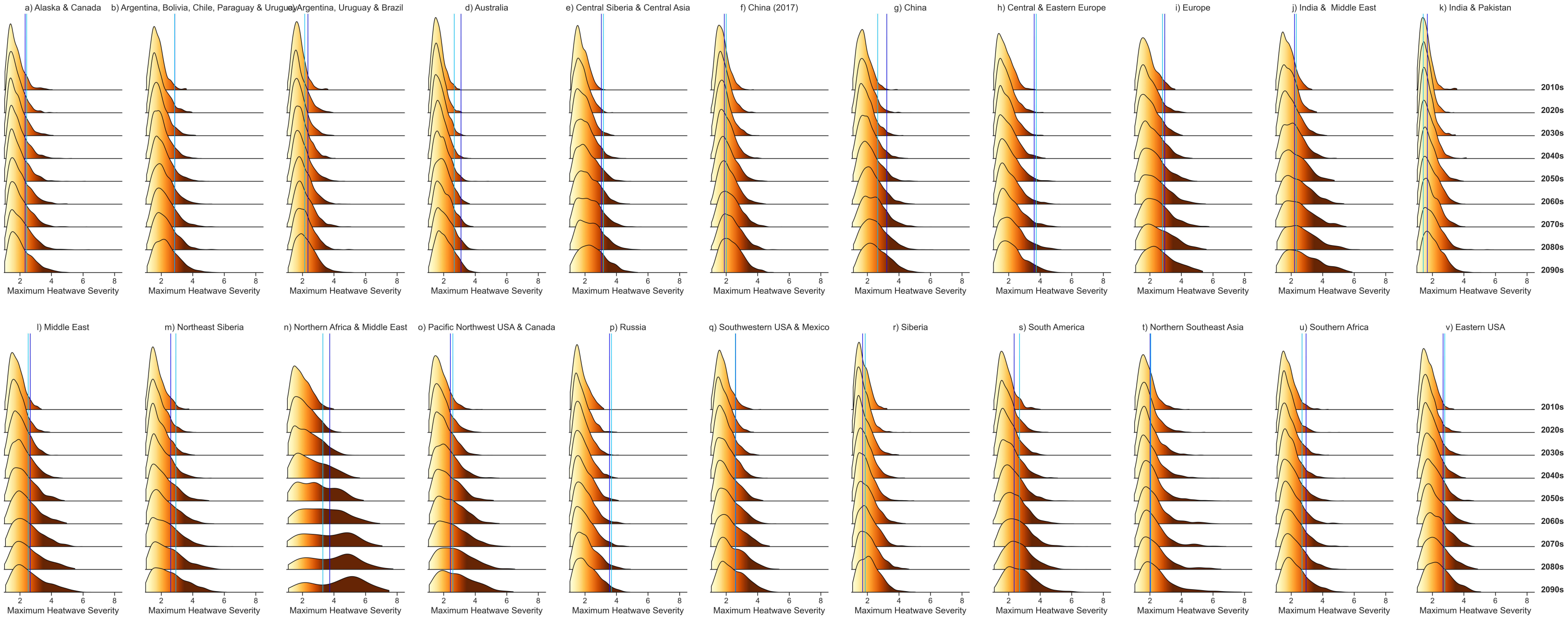

a) Alaska & Canada
b) Argentina, Bolivia, Chile, Paraguay & Uruguay
c) Argentina, Uruguay & Brazil
d) Australia
e) Central Siberia & Central Asia
f) China (2017)
g) China
h) Central & Eastern Europe
i) Europe
j) India & Middle East
k) India & Pakistan
l) Middle East
m) Northeast Siberia
n) Northern Africa & Middle East
o) Pacific Northwest USA & Canada
p) Russia
q) Southwestern USA & Mexico
r) Siberia
s) South America
t) Northern Southeast Asia
u) Southern Africa
v) Eastern USA
2010s
2020s
2030s
2040s
2050s
2060s
2070s
2080s
2090s
Maximum Heatwave Severity
2
4
6
8

Supplementary Figure 55: Kernel Density Estimates (KDE) across Decades for Maximum Heatwave Severity in SSP3-7.0 CMIP6 multi-model ensemble (MME) (Tx90p) - Kernel Density Estimates (KDE) of Maximum Heatwave Severity for different Decades in the CMIP6 multi-model ensemble (MME). Area under each curve is normalized to 1. Maximum Heatwave Severity of the observed event is indicated by vertical lines in dark blue (Berkeley Earth) and light blue (ERA5).

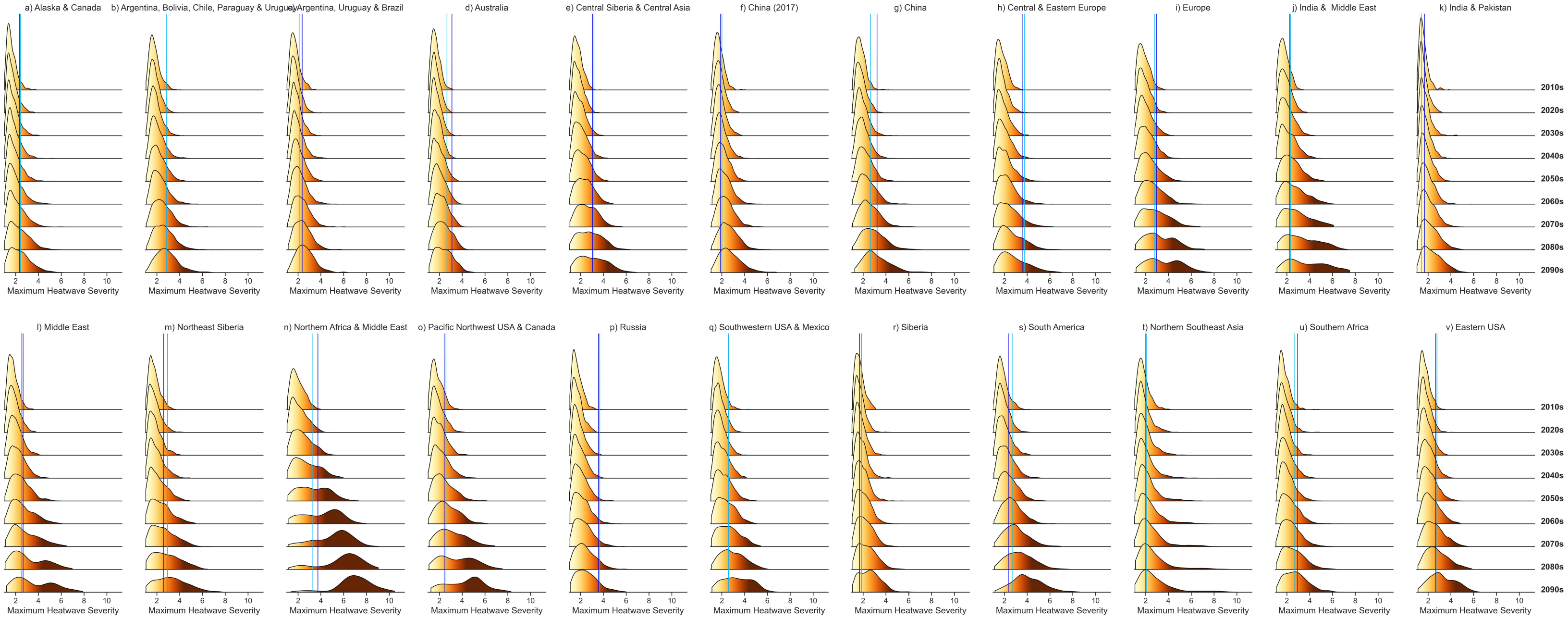
a) Alaska & Canada
b) Argentina, Bolivia, Chile, Paraguay & Uruguay
c) Argentina, Uruguay & Brazil
d) Australia
e) Central Siberia & Central Asia
f) China (2017)
g) China
h) Central & Eastern Europe
i) Europe
j) India & Middle East
k) India & Pakistan
l) Middle East
m) Northeast Siberia
n) Northern Africa & Middle East
o) Pacific Northwest USA & Canada
p) Russia
q) Southwestern USA & Mexico
r) Siberia
s) South America
t) Northern Southeast Asia
u) Southern Africa
v) Eastern USA
2010s
2020s
2030s
2040s
2050s
2060s
2070s
2080s
2090s
Maximum Heatwave Severity
2
4
6
8
10

Supplementary Figure 56: Kernel Density Estimates (KDE) across Decades for Maximum Heatwave Severity in SSP5-8.5 CMIP6 multi-model ensemble (MME) (Tx90p) - Kernel Density Estimates (KDE) of Maximum Heatwave Severity for different Decades in the CMIP6 multi-model ensemble (MME). Area under each curve is normalized to 1. Maximum Heatwave Severity of the observed event is indicated by vertical lines in dark blue (Berkeley Earth) and light blue (ERA5).

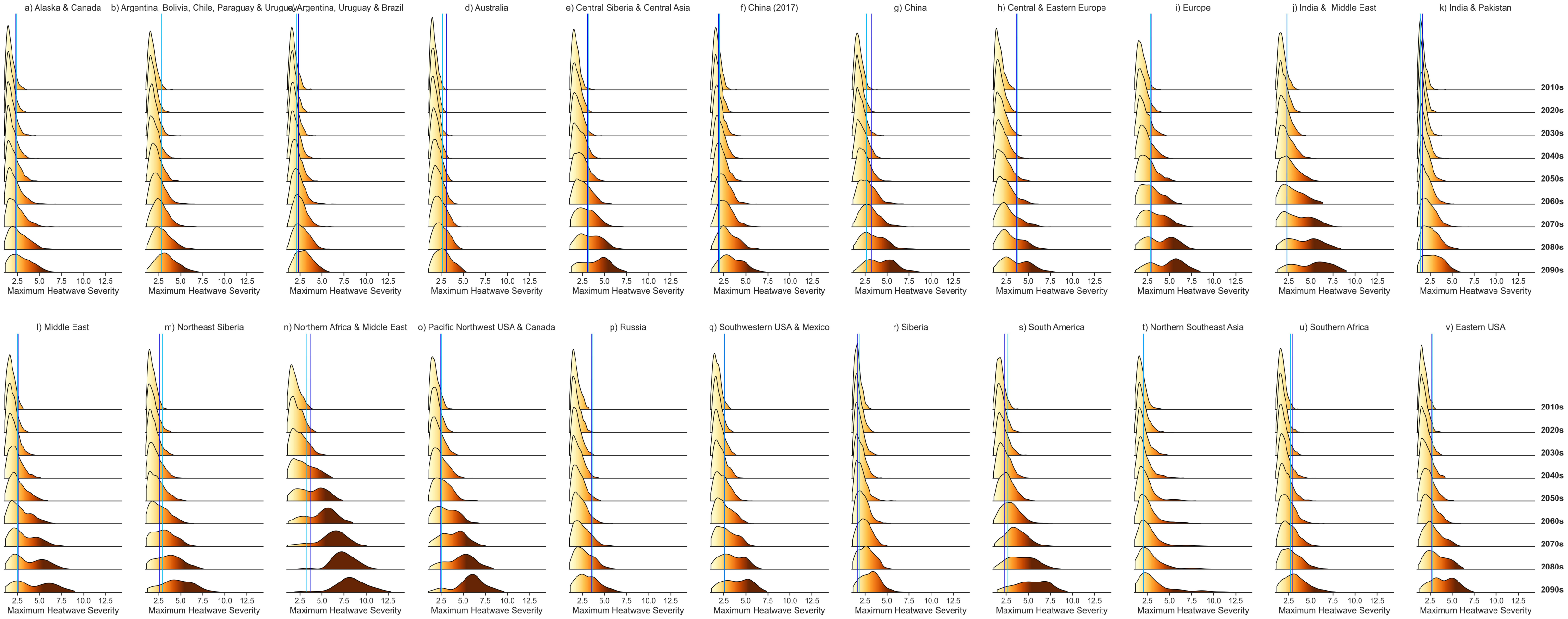

a) Alaska & Canada
b) Argentina, Bolivia, Chile, Paraguay & Uruguay
c) Argentina, Uruguay & Brazil
d) Australia
e) Central Siberia & Central Asia
f) China (2017)
g) China
h) Central & Eastern Europe
i) Europe
j) India & Middle East
k) India & Pakistan
l) Middle East
m) Northeast Siberia
n) Northern Africa & Middle East
o) Pacific Northwest USA & Canada
p) Russia
q) Southwestern USA & Mexico
r) Siberia
s) South America
t) Northern Southeast Asia
u) Southern Africa
v) Eastern USA
2010s
2020s
2030s
2040s
2050s
2060s
2070s
2080s
2090s
2.5 5.0 7.5 10.0 12.5
Maximum Heatwave Severity

Supplementary Figure 57: Kernel Density Estimates (KDE) across Decades for Median Heatwave Area (%) in SSP1-2.6 CMIP6 multi-model ensemble (MME) (Tx90p) - Kernel Density Estimates (KDE) of Median Heatwave Area (%) for different Decades in the CMIP6 multi-model ensemble (MME). Area under each curve is normalized to 1. Median Heatwave Area (%) of the observed event is indicated by vertical lines in dark blue (Berkeley Earth) and light blue (ERA5).

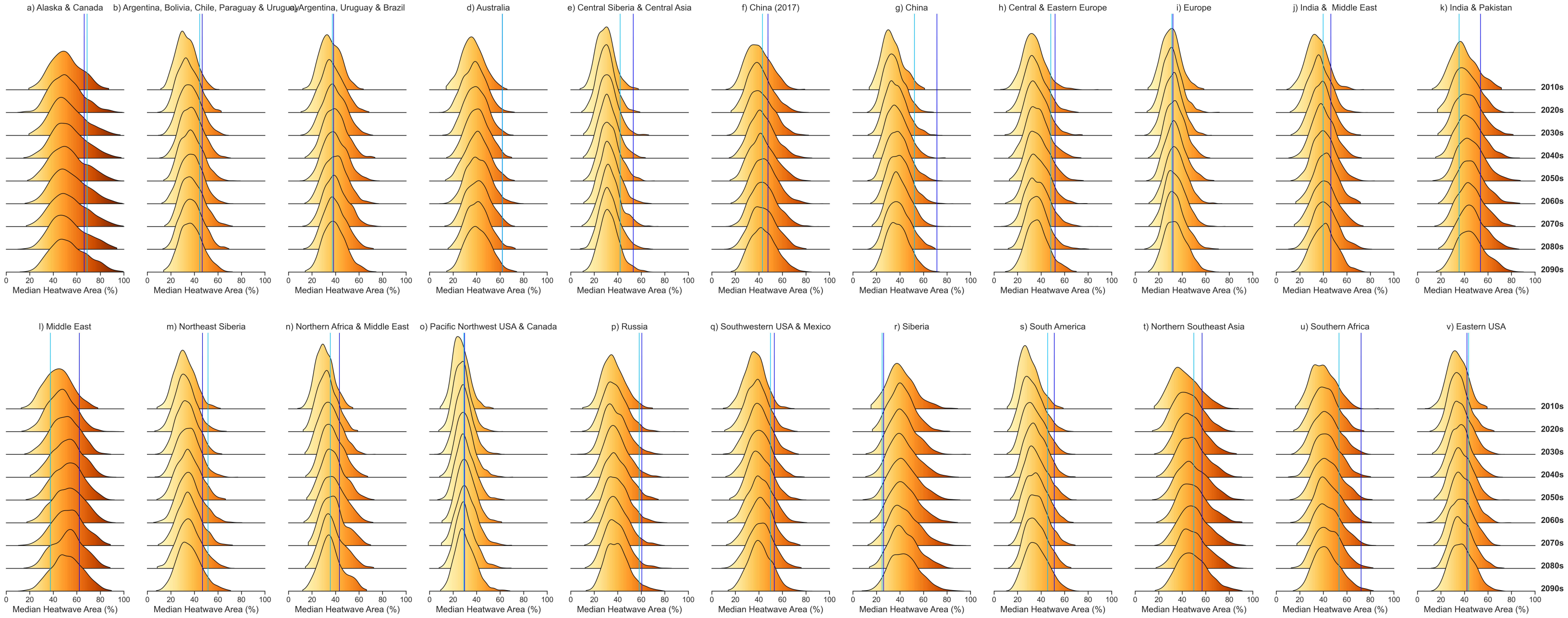

a) Alaska & Canada
b) Argentina, Bolivia, Chile, Paraguay & Uruguay
c) Argentina, Uruguay & Brazil
d) Australia
e) Central Siberia & Central Asia
f) China (2017)
g) China
h) Central & Eastern Europe
i) Europe
j) India & Middle East
k) India & Pakistan
l) Middle East
m) Northeast Siberia
n) Northern Africa & Middle East
o) Pacific Northwest USA & Canada
p) Russia
q) Southwestern USA & Mexico
r) Siberia
s) South America
t) Northern Southeast Asia
u) Southern Africa
v) Eastern USA
2010s
2020s
2030s
2040s
2050s
2060s
2070s
2080s
2090s
Median Heatwave Area (%)
0
20
40
60
80
100

Supplementary Figure 58: Kernel Density Estimates (KDE) across Decades for Median Heatwave Area (%) in SSP2-4.5 CMIP6 multi-model ensemble (MME) (Tx90p) - Kernel Density Estimates (KDE) of Median Heatwave Area (%) for different Decades in the CMIP6 multi-model ensemble (MME). Area under each curve is normalized to 1. Median Heatwave Area (%) of the observed event is indicated by vertical lines in dark blue (Berkeley Earth) and light blue (ERA5).

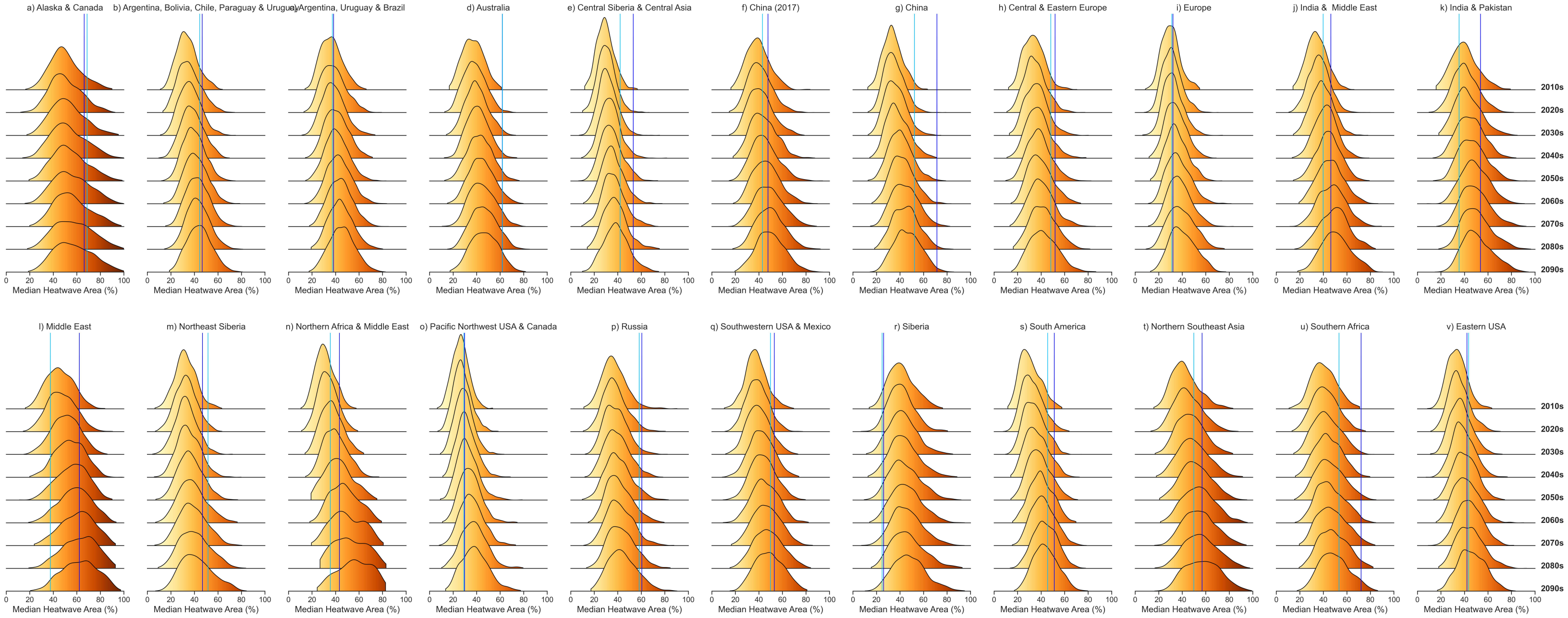

a) Alaska & Canada
b) Argentina, Bolivia, Chile, Paraguay & Uruguay
c) Argentina, Uruguay & Brazil
d) Australia
e) Central Siberia & Central Asia
f) China (2017)
g) China
h) Central & Eastern Europe
i) Europe
j) India & Middle East
k) India & Pakistan
l) Middle East
m) Northeast Siberia
n) Northern Africa & Middle East
o) Pacific Northwest USA & Canada
p) Russia
q) Southwestern USA & Mexico
r) Siberia
s) South America
t) Northern Southeast Asia
u) Southern Africa
v) Eastern USA
2010s
2020s
2030s
2040s
2050s
2060s
2070s
2080s
2090s
0 20 40 60 80 100
Median Heatwave Area (%)

Supplementary Figure 59: Kernel Density Estimates (KDE) across Decades for Median Heatwave Area (%) in SSP3-7.0 CMIP6 multi-model ensemble (MME) (Tx90p) - Kernel Density Estimates (KDE) of Median Heatwave Area (%) for different Decades in the CMIP6 multi-model ensemble (MME). Area under each curve is normalized to 1. Median Heatwave Area (%) of the observed event is indicated by vertical lines in dark blue (Berkeley Earth) and light blue (ERA5).

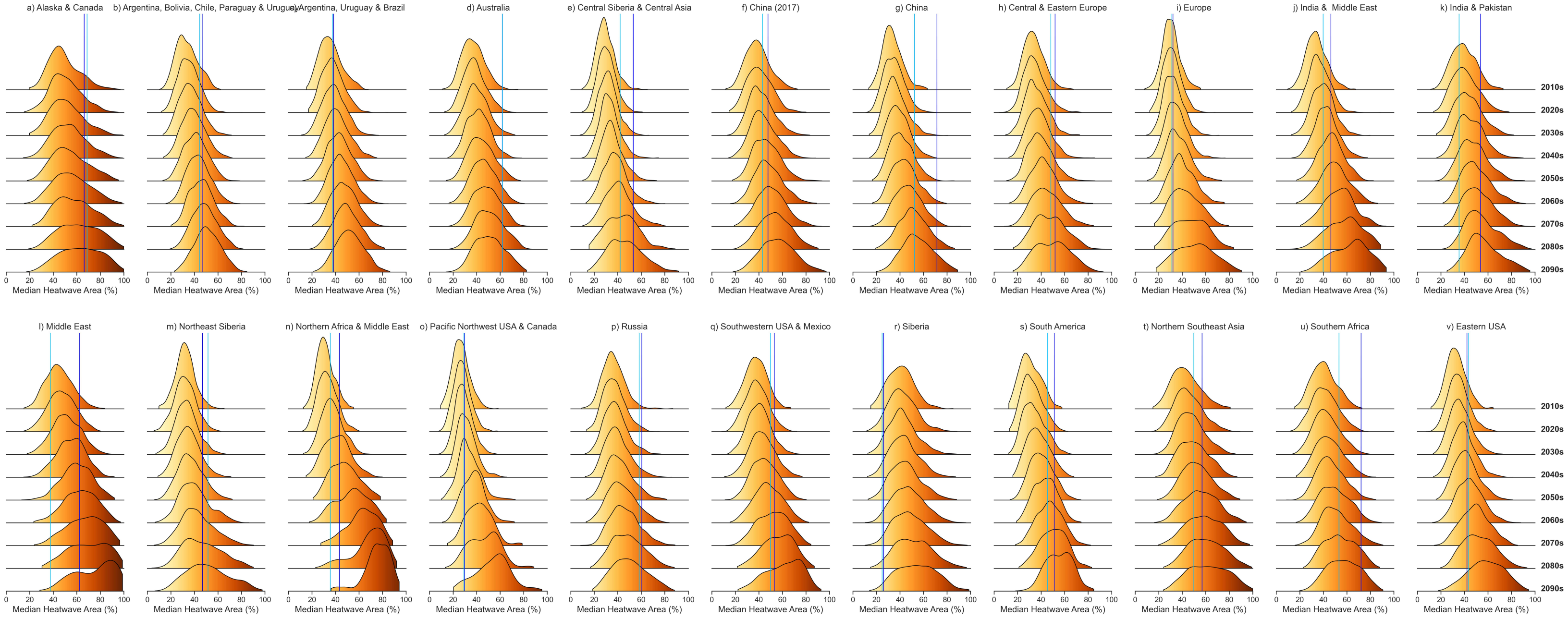
a) Alaska & Canada
b) Argentina, Bolivia, Chile, Paraguay & Uruguay
c) Argentina, Uruguay & Brazil
d) Australia
e) Central Siberia & Central Asia
f) China (2017)
g) China
h) Central & Eastern Europe
i) Europe
j) India & Middle East
k) India & Pakistan
l) Middle East
m) Northeast Siberia
n) Northern Africa & Middle East
o) Pacific Northwest USA & Canada
p) Russia
q) Southwestern USA & Mexico
r) Siberia
s) South America
t) Northern Southeast Asia
u) Southern Africa
v) Eastern USA
2010s
2020s
2030s
2040s
2050s
2060s
2070s
2080s
2090s
0 20 40 60 80 100
Median Heatwave Area (%)

Supplementary Figure 60: Kernel Density Estimates (KDE) across Decades for Median Heatwave Area (%) in SSP5-8.5 CMIP6 multi-model ensemble (MME) (Tx90p) - Kernel Density Estimates (KDE) of Median Heatwave Area (%) for different Decades in the CMIP6 multi-model ensemble (MME). Area under each curve is normalized to 1. Median Heatwave Area (%) of the observed event is indicated by vertical lines in dark blue (Berkeley Earth) and light blue (ERA5).

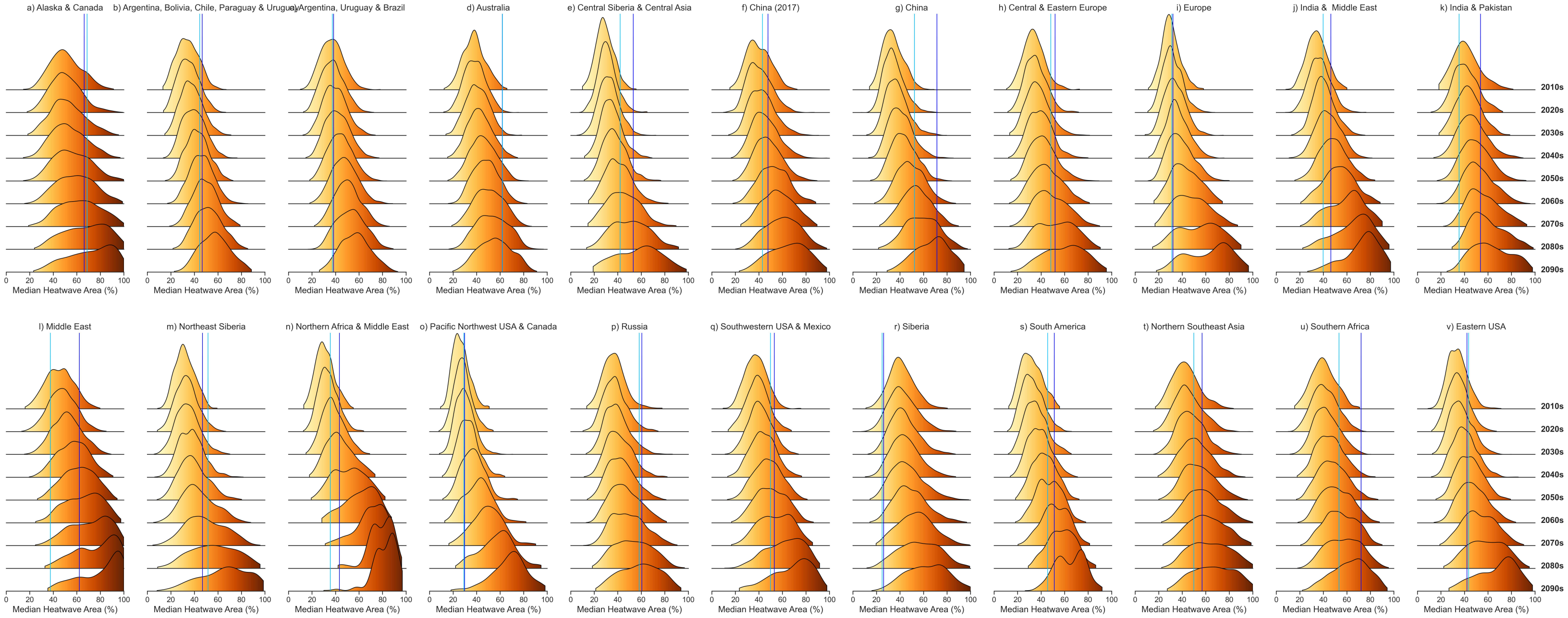

a) Alaska & Canada
b) Argentina, Bolivia, Chile, Paraguay & Uruguay
c) Argentina, Uruguay & Brazil
d) Australia
e) Central Siberia & Central Asia
f) China (2017)
g) China
h) Central & Eastern Europe
i) Europe
j) India & Middle East
k) India & Pakistan
l) Middle East
m) Northeast Siberia
n) Northern Africa & Middle East
o) Pacific Northwest USA & Canada
p) Russia
q) Southwestern USA & Mexico
r) Siberia
s) South America
t) Northern Southeast Asia
u) Southern Africa
v) Eastern USA
2010s
2020s
2030s
2040s
2050s
2060s
2070s
2080s
2090s
0
20
40
60
80
100
Median Heatwave Area (%)

Supplementary Figure 61: Kernel Density Estimates (KDE) across Decades for Maximum Heatwave Area (%) in SSP1-2.6 CMIP6 multi-model ensemble (MME) (Tx90p) - Kernel Density Estimates (KDE) of Maximum Heatwave Area (%) for different Decades in the CMIP6 multi-model ensemble (MME). Area under each curve is normalized to 1. Maximum Heatwave Area (%) of the observed event is indicated by vertical lines in dark blue (Berkeley Earth) and light blue (ERA5).

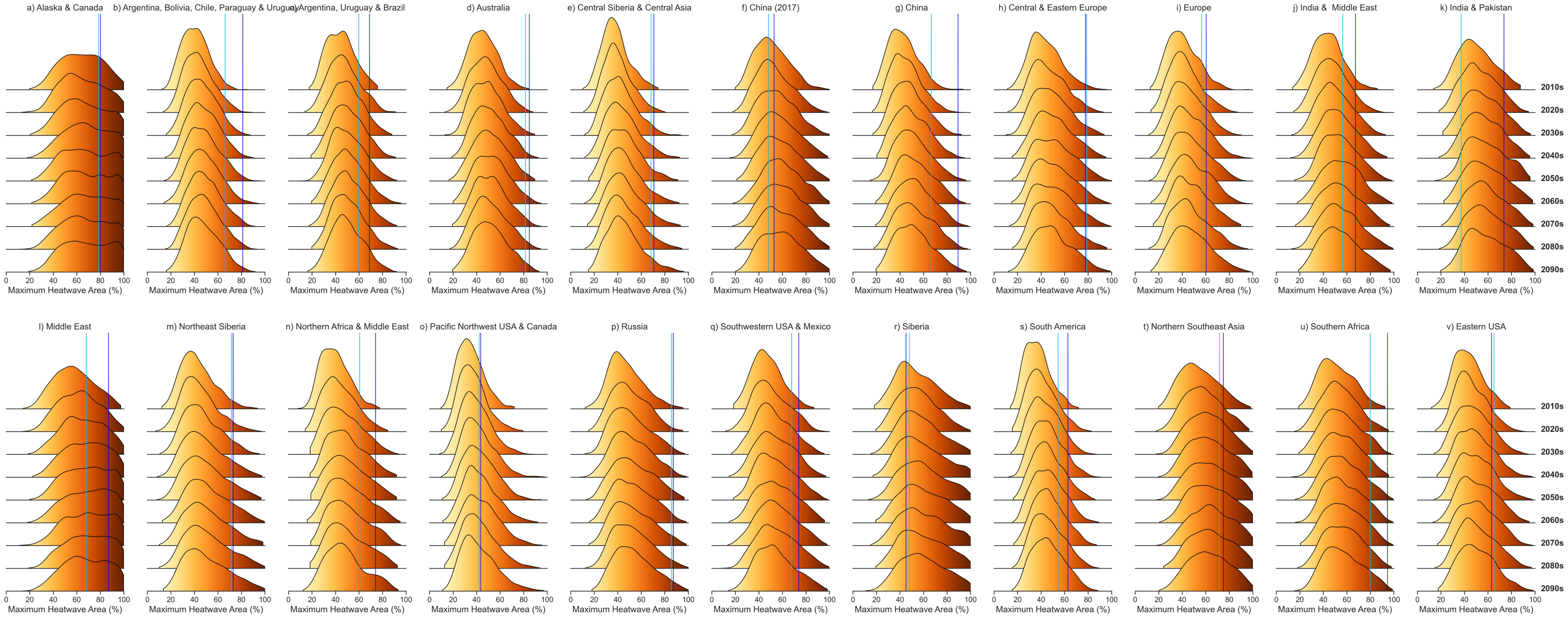

a) Alaska & Canada
b) Argentina, Bolivia, Chile, Paraguay & Uruguay
c) Argentina, Uruguay & Brazil
d) Australia
e) Central Siberia & Central Asia
f) China (2017)
g) China
h) Central & Eastern Europe
i) Europe
j) India & Middle East
k) India & Pakistan
l) Middle East
m) Northeast Siberia
n) Northern Africa & Middle East
o) Pacific Northwest USA & Canada
p) Russia
q) Southwestern USA & Mexico
r) Siberia
s) South America
t) Northern Southeast Asia
u) Southern Africa
v) Eastern USA
2010s
2020s
2030s
2040s
2050s
2060s
2070s
2080s
2090s
0
20
40
60
80
100
Maximum Heatwave Area (%)

Supplementary Figure 62: Kernel Density Estimates (KDE) across Decades for Maximum Heatwave Area (%) in SSP2-4.5 CMIP6 multi-model ensemble (MME) (Tx90p) - Kernel Density Estimates (KDE) of Maximum Heatwave Area (%) for different Decades in the CMIP6 multi-model ensemble (MME). Area under each curve is normalized to 1. Maximum Heatwave Area (%) of the observed event is indicated by vertical lines in dark blue (Berkeley Earth) and light blue (ERA5).

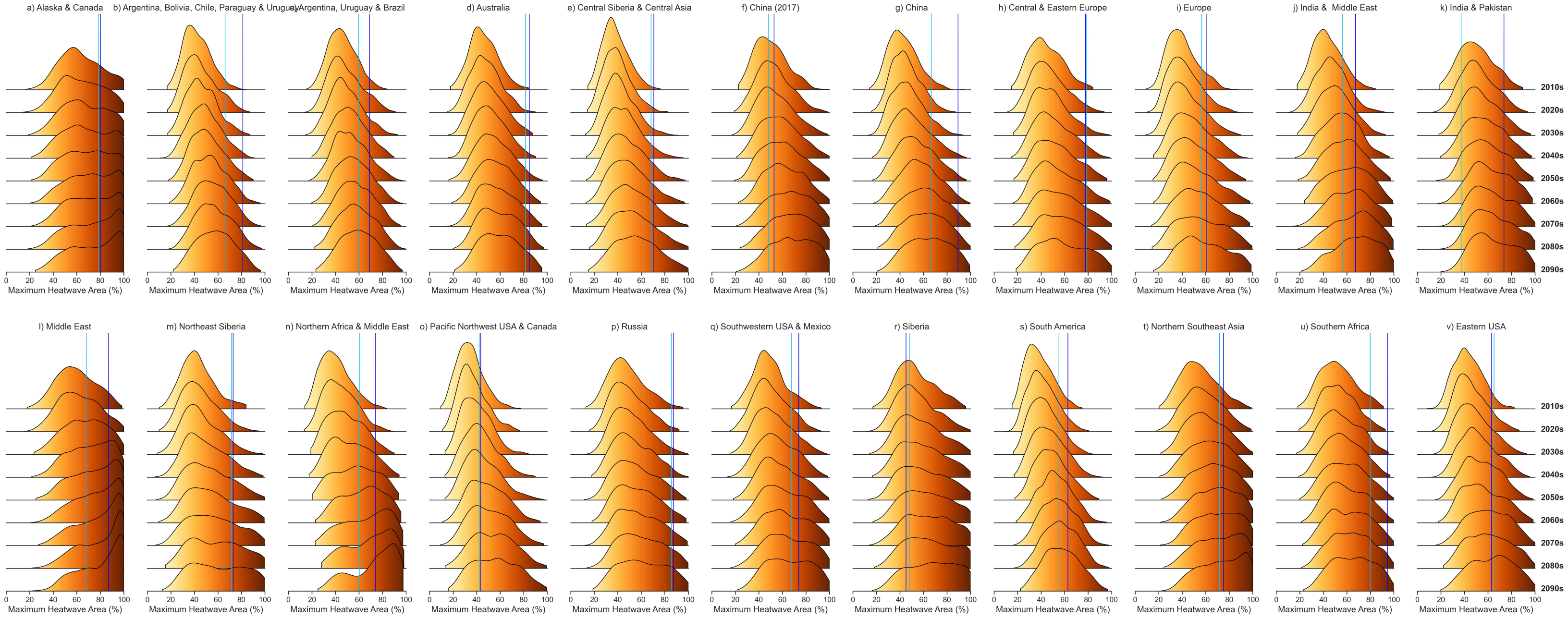

a) Alaska & Canada
b) Argentina, Bolivia, Chile, Paraguay & Uruguay
c) Argentina, Uruguay & Brazil
d) Australia
e) Central Siberia & Central Asia
f) China (2017)
g) China
h) Central & Eastern Europe
i) Europe
j) India & Middle East
k) India & Pakistan
l) Middle East
m) Northeast Siberia
n) Northern Africa & Middle East
o) Pacific Northwest USA & Canada
p) Russia
q) Southwestern USA & Mexico
r) Siberia
s) South America
t) Northern Southeast Asia
u) Southern Africa
v) Eastern USA
2010s
2020s
2030s
2040s
2050s
2060s
2070s
2080s
2090s
0 20 40 60 80 100
Maximum Heatwave Area (%)

Supplementary Figure 63: Kernel Density Estimates (KDE) across Decades for Maximum Heatwave Area (%) in SSP3-7.0 CMIP6 multi-model ensemble (MME) (Tx90p) - Kernel Density Estimates (KDE) of Maximum Heatwave Area (%) for different Decades in the CMIP6 multi-model ensemble (MME). Area under each curve is normalized to 1. Maximum Heatwave Area (%) of the observed event is indicated by vertical lines in dark blue (Berkeley Earth) and light blue (ERA5).

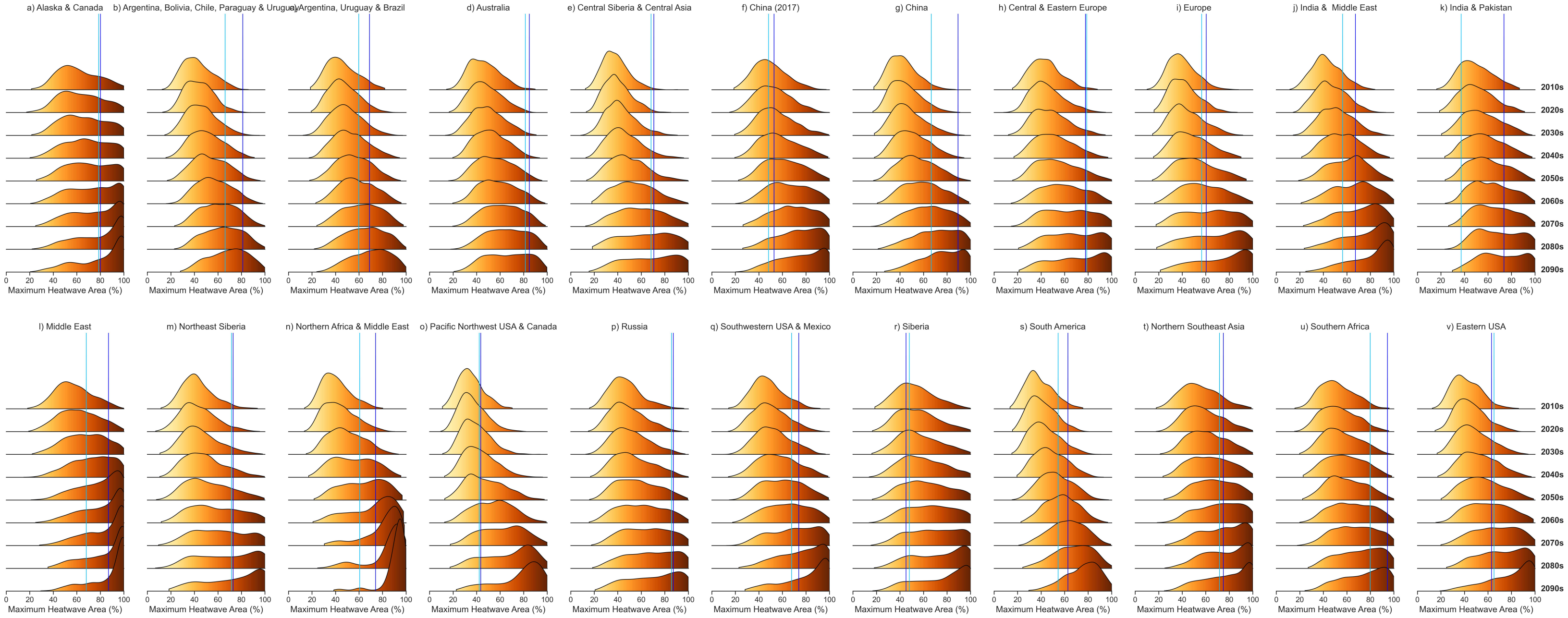
a) Alaska & Canada
b) Argentina, Bolivia, Chile, Paraguay & Uruguay
c) Argentina, Uruguay & Brazil
d) Australia
e) Central Siberia & Central Asia
f) China (2017)
g) China
h) Central & Eastern Europe
i) Europe
j) India & Middle East
k) India & Pakistan
l) Middle East
m) Northeast Siberia
n) Northern Africa & Middle East
o) Pacific Northwest USA & Canada
p) Russia
q) Southwestern USA & Mexico
r) Siberia
s) South America
t) Northern Southeast Asia
u) Southern Africa
v) Eastern USA
2010s
2020s
2030s
2040s
2050s
2060s
2070s
2080s
2090s
Maximum Heatwave Area (%)

Supplementary Figure 64: Kernel Density Estimates (KDE) across Decades for Maximum Heatwave Area (%) in SSP5-8.5 CMIP6 multi-model ensemble (MME) (Tx90p) - Kernel Density Estimates (KDE) of Maximum Heatwave Area (%) for different Decades in the CMIP6 multi-model ensemble (MME). Area under each curve is normalized to 1. Maximum Heatwave Area (%) of the observed event is indicated by vertical lines in dark blue (Berkeley Earth) and light blue (ERA5).

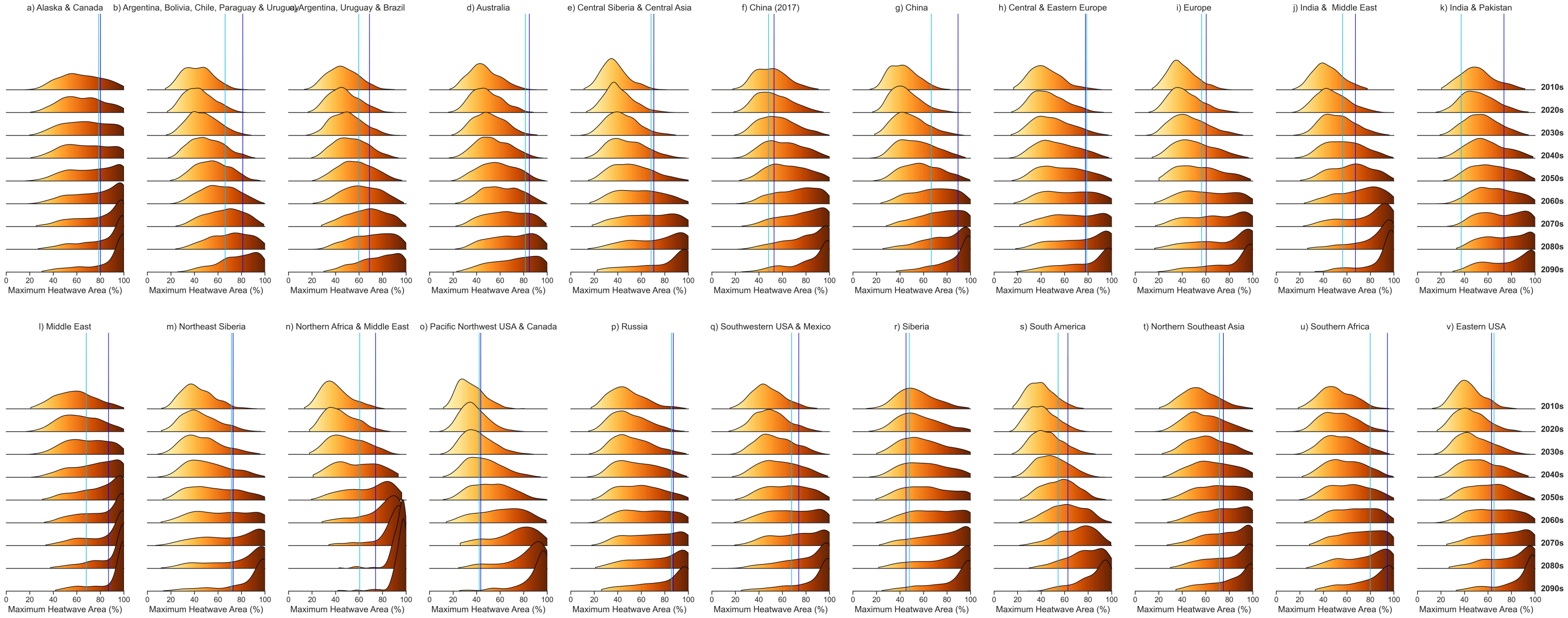

a) Alaska & Canada
b) Argentina, Bolivia, Chile, Paraguay & Uruguay
c) Argentina, Uruguay & Brazil
d) Australia
e) Central Siberia & Central Asia
f) China (2017)
g) China
h) Central & Eastern Europe
i) Europe
j) India & Middle East
k) India & Pakistan
l) Middle East
m) Northeast Siberia
n) Northern Africa & Middle East
o) Pacific Northwest USA & Canada
p) Russia
q) Southwestern USA & Mexico
r) Siberia
s) South America
t) Northern Southeast Asia
u) Southern Africa
v) Eastern USA
2010s
2020s
2030s
2040s
2050s
2060s
2070s
2080s
2090s
0 20 40 60 80 100
Maximum Heatwave Area (%)

Supplementary Figure 65: Kernel Density Estimates (KDE) across Decades for Cumulative Heat (°C) in RCP 2.6 CMIP5 multi-model ensemble (MME) (Tx90p) - Kernel Density Estimates (KDE) of Cumulative Heat (°C) for different Decades in the CMIP5 multi-model ensemble (MME). Area under each curve is normalized to 1. Cumulative Heat (°C) of the observed event is indicated by vertical lines in dark blue (Berkeley Earth) and light blue (ERA5).

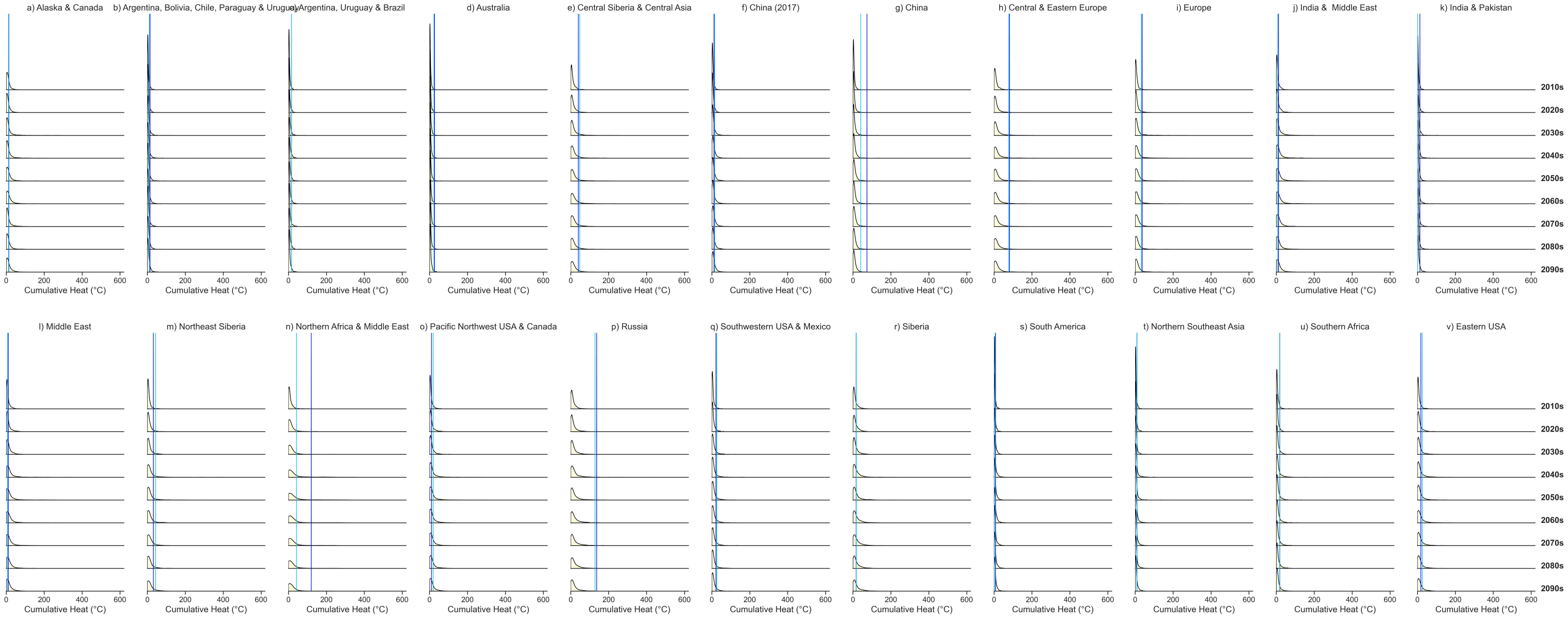
a) Alaska & Canada
b) Argentina, Bolivia, Chile, Paraguay & Uruguay
c) Argentina, Uruguay & Brazil
d) Australia
e) Central Siberia & Central Asia
f) China (2017)
g) China
h) Central & Eastern Europe
i) Europe
j) India & Middle East
k) India & Pakistan
l) Middle East
m) Northeast Siberia
n) Northern Africa & Middle East
o) Pacific Northwest USA & Canada
p) Russia
q) Southwestern USA & Mexico
r) Siberia
s) South America
t) Northern Southeast Asia
u) Southern Africa
v) Eastern USA
2010s
2020s
2030s
2040s
2050s
2060s
2070s
2080s
2090s
0
200
400
600
Cumulative Heat (°C)

Supplementary Figure 66: Kernel Density Estimates (KDE) across Decades for Cumulative Heat (°C) in RCP 4.5 CMIP5 multi-model ensemble (MME) (Tx90p) - Kernel Density Estimates (KDE) of Cumulative Heat (°C) for different Decades in the CMIP5 multi-model ensemble (MME). Area under each curve is normalized to 1. Cumulative Heat (°C) of the observed event is indicated by vertical lines in dark blue (Berkeley Earth) and light blue (ERA5).

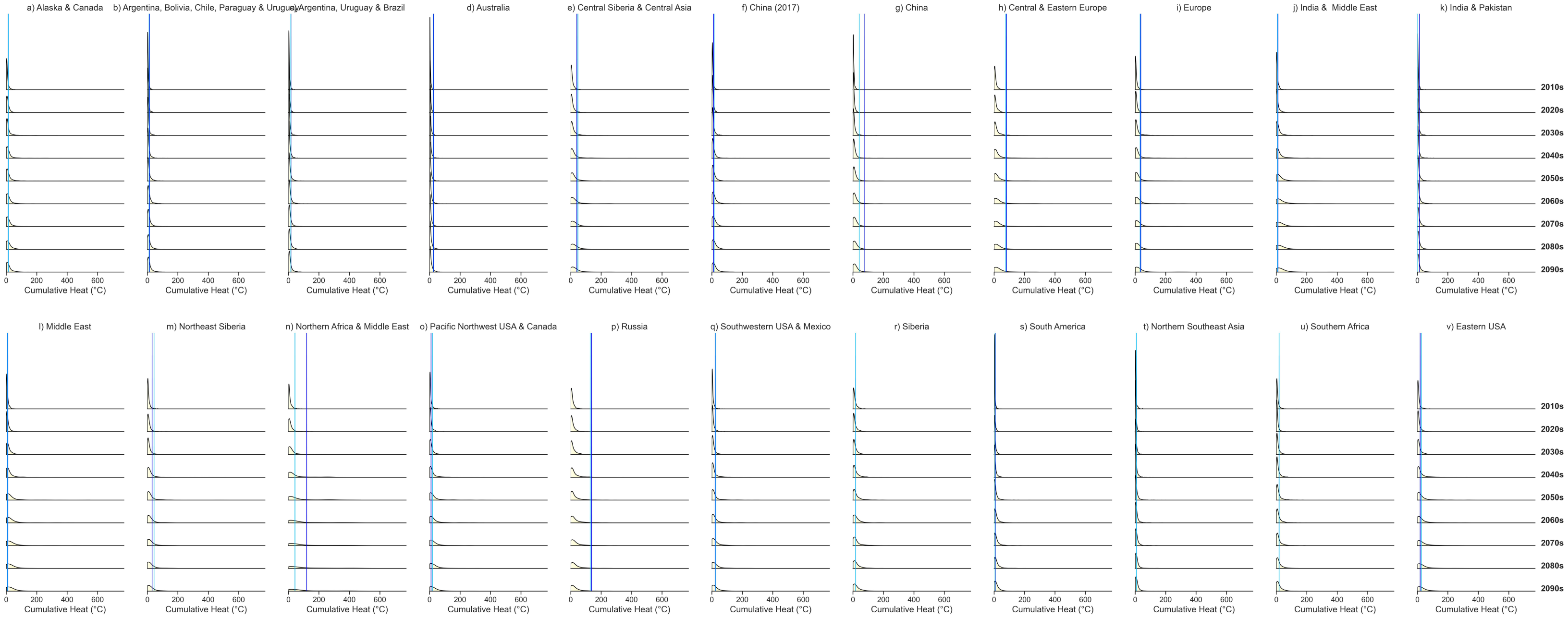

a) Alaska & Canada
b) Argentina, Bolivia, Chile, Paraguay & Uruguay
c) Argentina, Uruguay & Brazil
d) Australia
e) Central Siberia & Central Asia
f) China (2017)
g) China
h) Central & Eastern Europe
i) Europe
j) India & Middle East
k) India & Pakistan
l) Middle East
m) Northeast Siberia
n) Northern Africa & Middle East
o) Pacific Northwest USA & Canada
p) Russia
q) Southwestern USA & Mexico
r) Siberia
s) South America
t) Northern Southeast Asia
u) Southern Africa
v) Eastern USA
2010s
2020s
2030s
2040s
2050s
2060s
2070s
2080s
2090s
0
200
400
600
Cumulative Heat (°C)

Supplementary Figure 67: Kernel Density Estimates (KDE) across Decades for Cumulative Heat (°C) in RCP 8.5 CMIP5 multi-model ensemble (MME) (Tx90p) - Kernel Density Estimates (KDE) of Cumulative Heat (°C) for different Decades in the CMIP5 multi-model ensemble (MME). Area under each curve is normalized to 1. Cumulative Heat (°C) of the observed event is indicated by vertical lines in dark blue (Berkeley Earth) and light blue (ERA5).

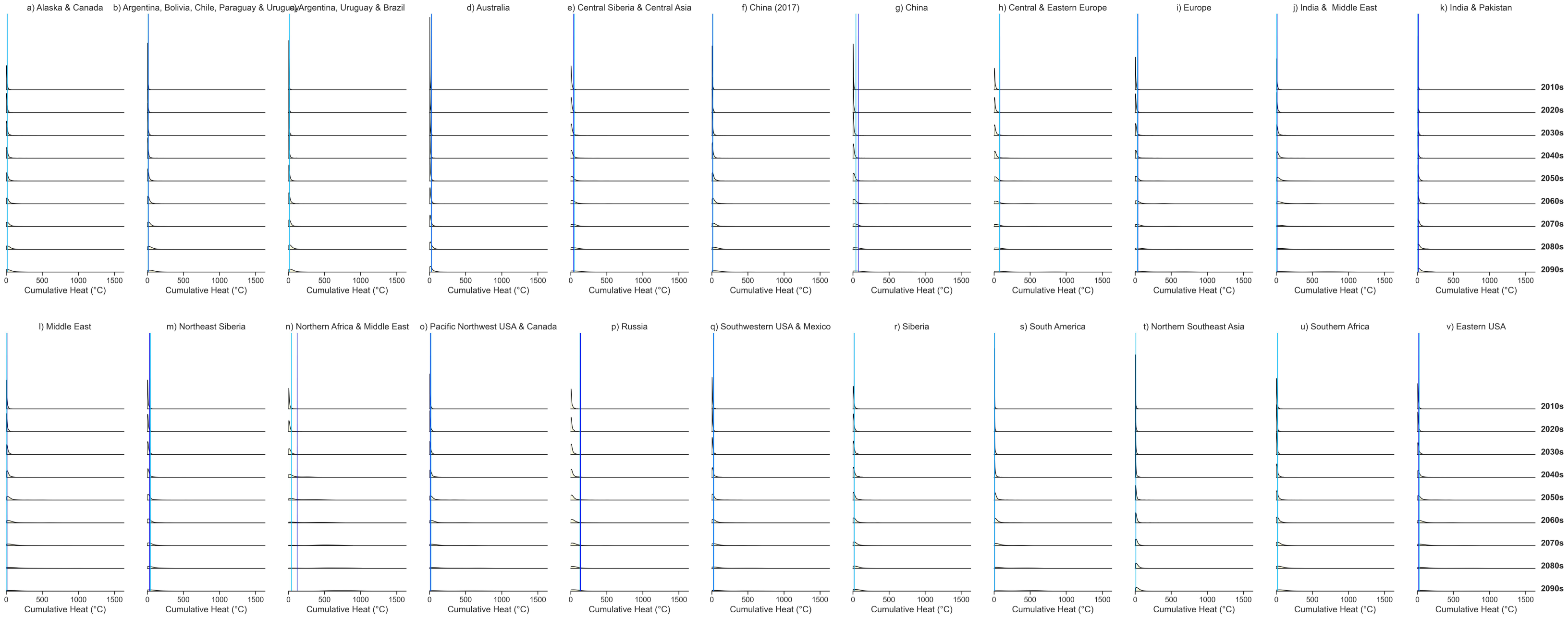
a) Alaska & Canada
b) Argentina, Bolivia, Chile, Paraguay & Uruguay
c) Argentina, Uruguay & Brazil
d) Australia
e) Central Siberia & Central Asia
f) China (2017)
g) China
h) Central & Eastern Europe
i) Europe
j) India & Middle East
k) India & Pakistan
l) Middle East
m) Northeast Siberia
n) Northern Africa & Middle East
o) Pacific Northwest USA & Canada
p) Russia
q) Southwestern USA & Mexico
r) Siberia
s) South America
t) Northern Southeast Asia
u) Southern Africa
v) Eastern USA
2010s
2020s
2030s
2040s
2050s
2060s
2070s
2080s
2090s
0
500
1000
1500
Cumulative Heat (°C)

Supplementary Figure 68: Kernel Density Estimates (KDE) across Decades for Heatwave Length (days) in RCP 2.6 CMIP5 multi-model ensemble (MME) (Tx90p) - Kernel Density Estimates (KDE) of Heatwave Length (days) for different Decades in the CMIP5 multi-model ensemble (MME). Area under each curve is normalized to 1. Heatwave Length (days) of the observed event is indicated by vertical lines in dark blue (Berkeley Earth) and light blue (ERA5).

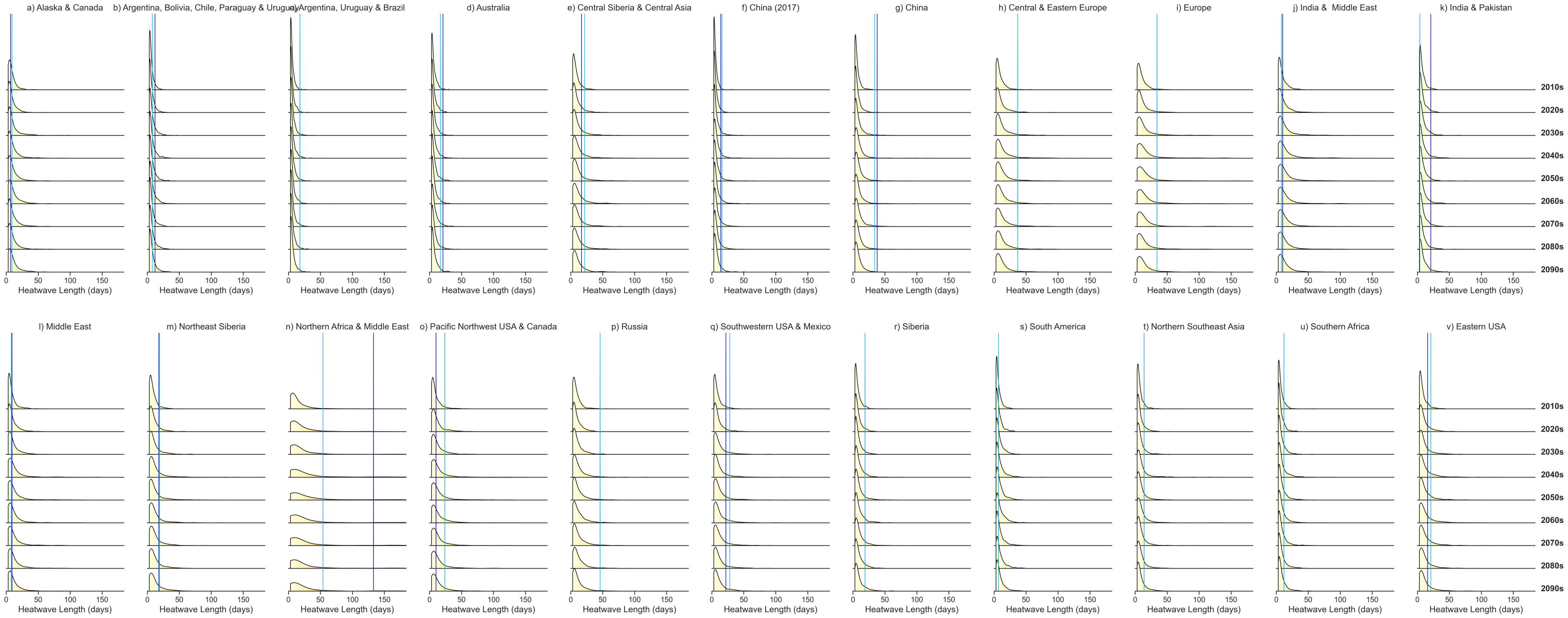

a) Alaska & Canada
b) Argentina, Bolivia, Chile, Paraguay & Uruguay
c) Argentina, Uruguay & Brazil
d) Australia
e) Central Siberia & Central Asia
f) China (2017)
g) China
h) Central & Eastern Europe
i) Europe
j) India & Middle East
k) India & Pakistan
l) Middle East
m) Northeast Siberia
n) Northern Africa & Middle East
o) Pacific Northwest USA & Canada
p) Russia
q) Southwestern USA & Mexico
r) Siberia
s) South America
t) Northern Southeast Asia
u) Southern Africa
v) Eastern USA
2010s
2020s
2030s
2040s
2050s
2060s
2070s
2080s
2090s
0
50
100
150
Heatwave Length (days)

Supplementary Figure 69: Kernel Density Estimates (KDE) across Decades for Heatwave Length (days) in RCP 4.5 CMIP5 multi-model ensemble (MME) (Tx90p) - Kernel Density Estimates (KDE) of Heatwave Length (days) for different Decades in the CMIP5 multi-model ensemble (MME). Area under each curve is normalized to 1. Heatwave Length (days) of the observed event is indicated by vertical lines in dark blue (Berkeley Earth) and light blue (ERA5).

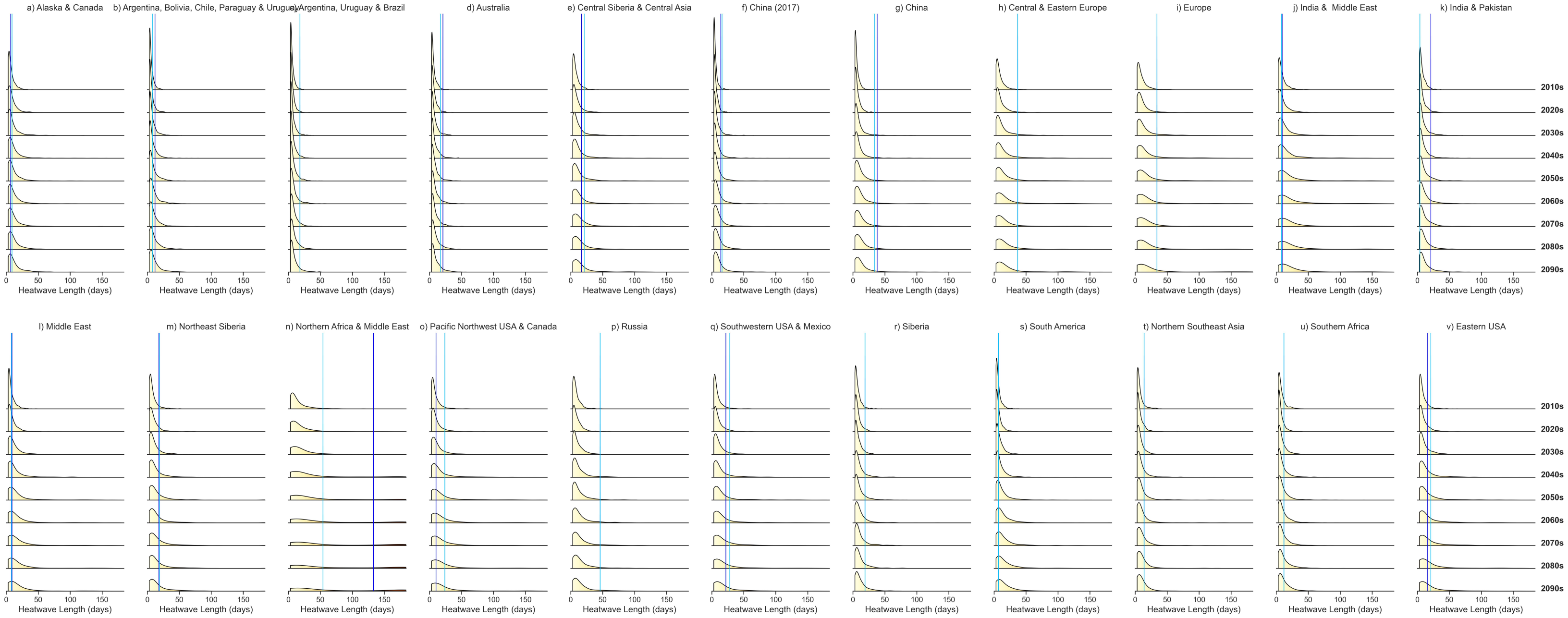

a) Alaska & Canada
b) Argentina, Bolivia, Chile, Paraguay & Uruguay
c) Argentina, Uruguay & Brazil
d) Australia
e) Central Siberia & Central Asia
f) China (2017)
g) China
h) Central & Eastern Europe
i) Europe
j) India & Middle East
k) India & Pakistan
l) Middle East
m) Northeast Siberia
n) Northern Africa & Middle East
o) Pacific Northwest USA & Canada
p) Russia
q) Southwestern USA & Mexico
r) Siberia
s) South America
t) Northern Southeast Asia
u) Southern Africa
v) Eastern USA
2010s
2020s
2030s
2040s
2050s
2060s
2070s
2080s
2090s
0
50
100
150
Heatwave Length (days)

Supplementary Figure 70: Kernel Density Estimates (KDE) across Decades for Heatwave Length (days) in RCP 8.5 CMIP5 multi-model ensemble (MME) (Tx90p) - Kernel Density Estimates (KDE) of Heatwave Length (days) for different Decades in the CMIP5 multi-model ensemble (MME). Area under each curve is normalized to 1. Heatwave Length (days) of the observed event is indicated by vertical lines in dark blue (Berkeley Earth) and light blue (ERA5).

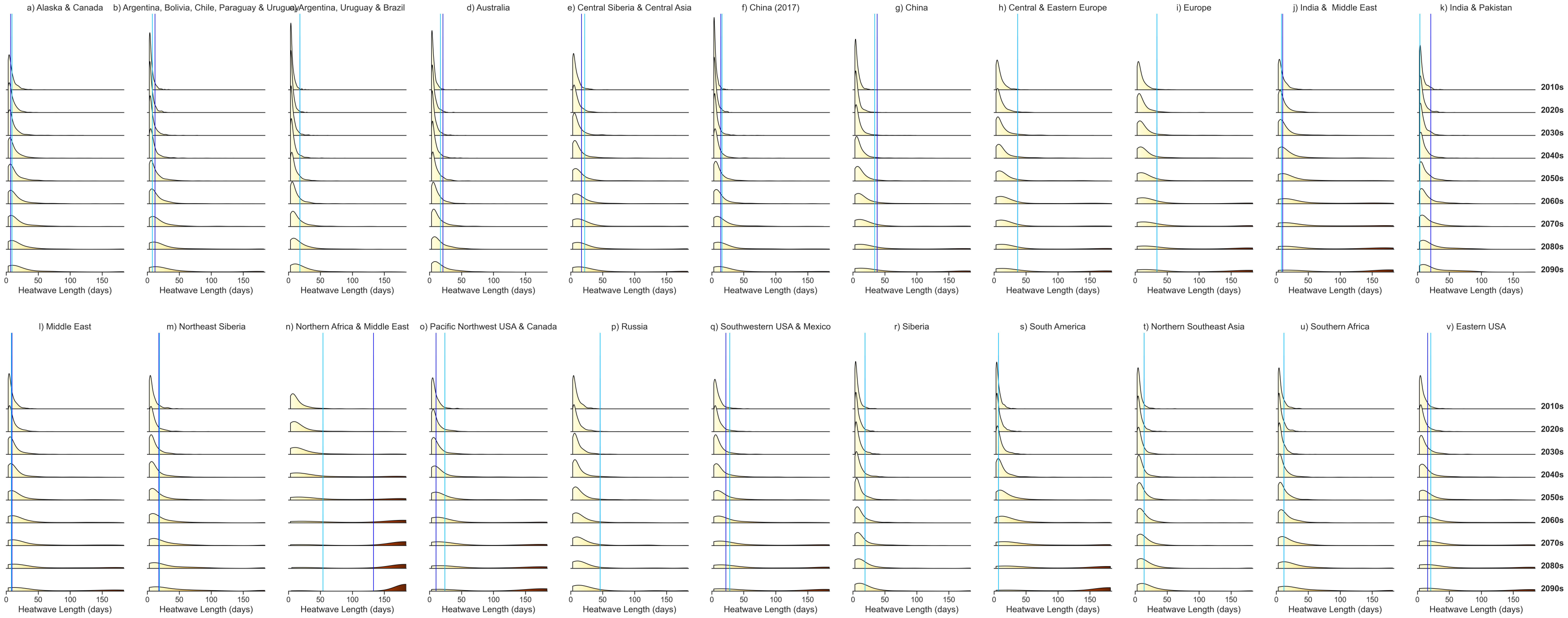
a) Alaska & Canada
b) Argentina, Bolivia, Chile, Paraguay & Uruguay
c) Argentina, Uruguay & Brazil
d) Australia
e) Central Siberia & Central Asia
f) China (2017)
g) China
h) Central & Eastern Europe
i) Europe
j) India & Middle East
k) India & Pakistan
l) Middle East
m) Northeast Siberia
n) Northern Africa & Middle East
o) Pacific Northwest USA & Canada
p) Russia
q) Southwestern USA & Mexico
r) Siberia
s) South America
t) Northern Southeast Asia
u) Southern Africa
v) Eastern USA
2010s
2020s
2030s
2040s
2050s
2060s
2070s
2080s
2090s
0
50
100
150
Heatwave Length (days)

Supplementary Figure 71: Kernel Density Estimates (KDE) across Decades for Median Heatwave Severity in RCP 2.6 CMIP5 multi-model ensemble (MME) (Tx90p) - Kernel Density Estimates (KDE) of Median Heatwave Severity for different Decades in the CMIP5 multi-model ensemble (MME). Area under each curve is normalized to 1. Median Heatwave Severity of the observed event is indicated by vertical lines in dark blue (Berkeley Earth) and light blue (ERA5).

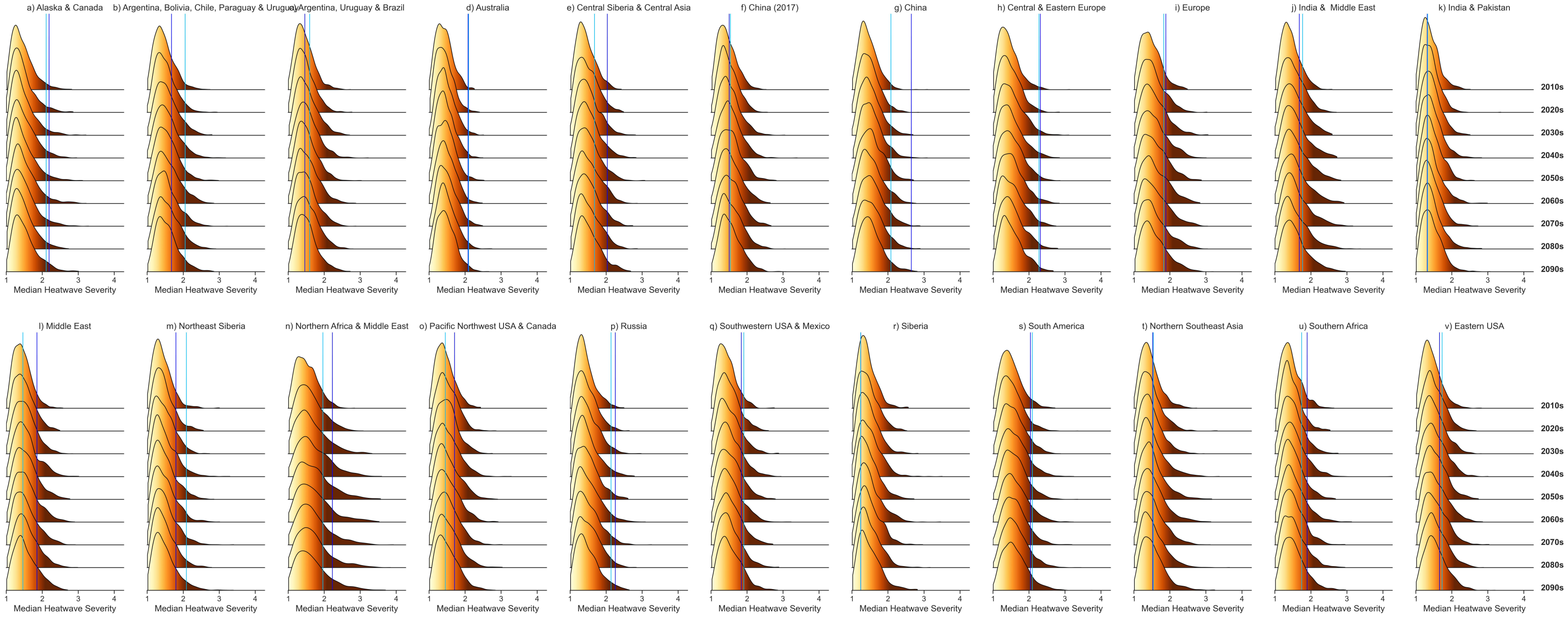

a) Alaska & Canada
b) Argentina, Bolivia, Chile, Paraguay & Uruguay
c) Argentina, Uruguay & Brazil
d) Australia
e) Central Siberia & Central Asia
f) China (2017)
g) China
h) Central & Eastern Europe
i) Europe
j) India & Middle East
k) India & Pakistan
l) Middle East
m) Northeast Siberia
n) Northern Africa & Middle East
o) Pacific Northwest USA & Canada
p) Russia
q) Southwestern USA & Mexico
r) Siberia
s) South America
t) Northern Southeast Asia
u) Southern Africa
v) Eastern USA
2010s
2020s
2030s
2040s
2050s
2060s
2070s
2080s
2090s
Median Heatwave Severity
1
2
3
4

Supplementary Figure 72: Kernel Density Estimates (KDE) across Decades for Median Heatwave Severity in RCP 4.5 CMIP5 multi-model ensemble (MME) (Tx90p) - Kernel Density Estimates (KDE) of Median Heatwave Severity for different Decades in the CMIP5 multi-model ensemble (MME). Area under each curve is normalized to 1. Median Heatwave Severity of the observed event is indicated by vertical lines in dark blue (Berkeley Earth) and light blue (ERA5).

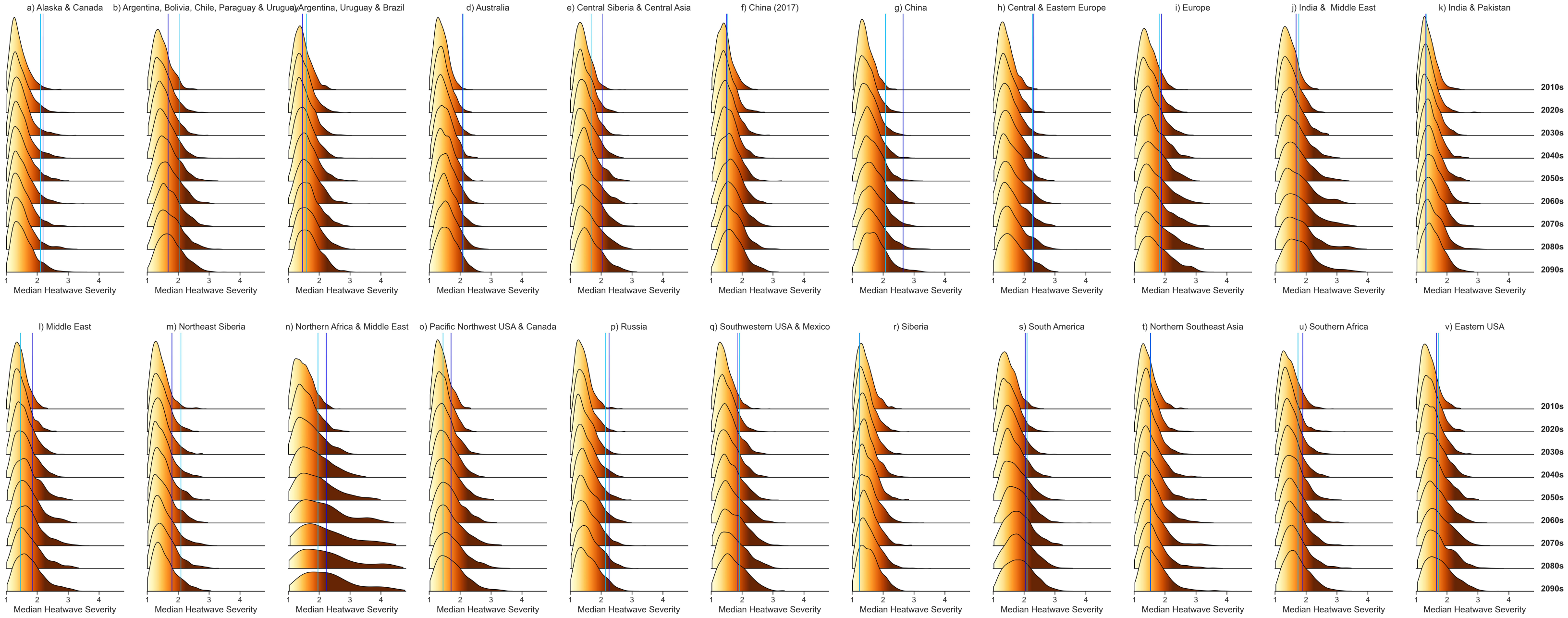

a) Alaska & Canada
b) Argentina, Bolivia, Chile, Paraguay & Uruguay
c) Argentina, Uruguay & Brazil
d) Australia
e) Central Siberia & Central Asia
f) China (2017)
g) China
h) Central & Eastern Europe
i) Europe
j) India & Middle East
k) India & Pakistan
l) Middle East
m) Northeast Siberia
n) Northern Africa & Middle East
o) Pacific Northwest USA & Canada
p) Russia
q) Southwestern USA & Mexico
r) Siberia
s) South America
t) Northern Southeast Asia
u) Southern Africa
v) Eastern USA
2010s
2020s
2030s
2040s
2050s
2060s
2070s
2080s
2090s
Median Heatwave Severity
1
2
3
4

Supplementary Figure 73: Kernel Density Estimates (KDE) across Decades for Median Heatwave Severity in RCP 8.5 CMIP5 multi-model ensemble (MME) (Tx90p) - Kernel Density Estimates (KDE) of Median Heatwave Severity for different Decades in the CMIP5 multi-model ensemble (MME). Area under each curve is normalized to 1. Median Heatwave Severity of the observed event is indicated by vertical lines in dark blue (Berkeley Earth) and light blue (ERA5).

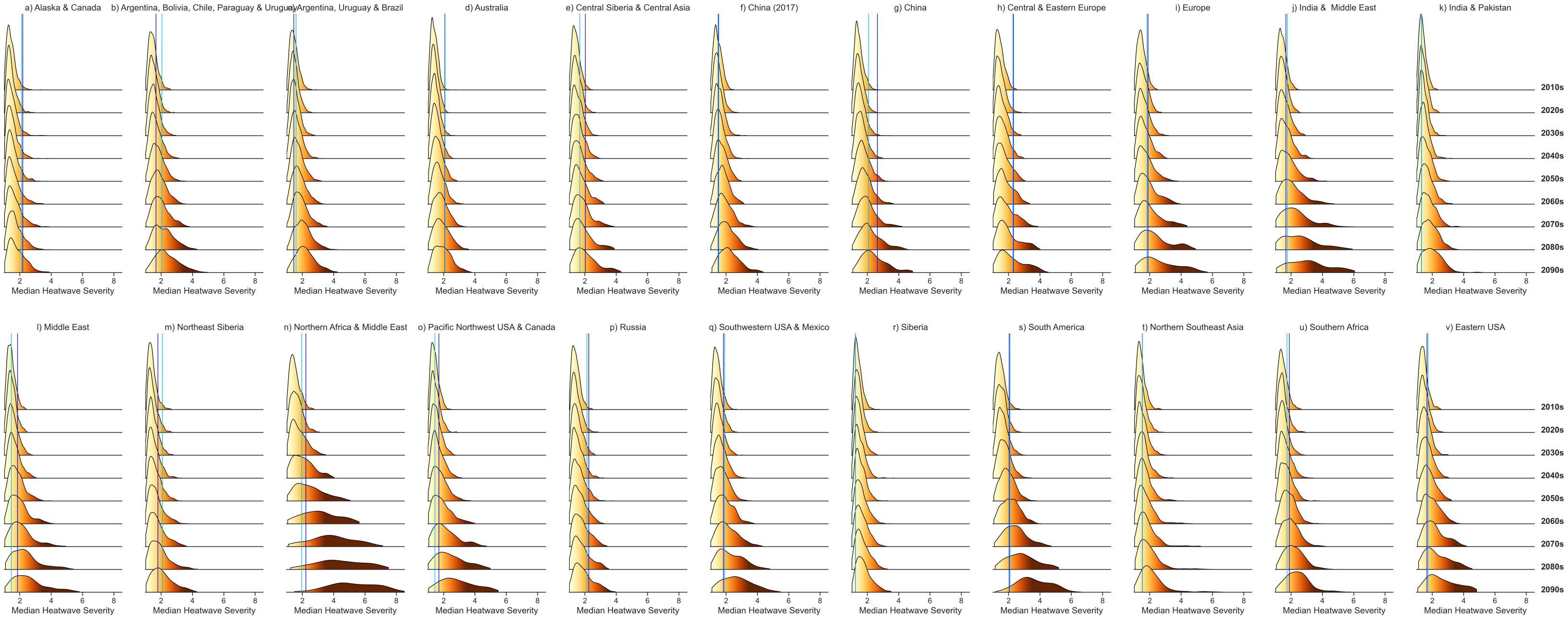

a) Alaska & Canada
b) Argentina, Bolivia, Chile, Paraguay & Uruguay
c) Argentina, Uruguay & Brazil
d) Australia
e) Central Siberia & Central Asia
f) China (2017)
g) China
h) Central & Eastern Europe
i) Europe
j) India & Middle East
k) India & Pakistan
l) Middle East
m) Northeast Siberia
n) Northern Africa & Middle East
o) Pacific Northwest USA & Canada
p) Russia
q) Southwestern USA & Mexico
r) Siberia
s) South America
t) Northern Southeast Asia
u) Southern Africa
v) Eastern USA
2010s
2020s
2030s
2040s
2050s
2060s
2070s
2080s
2090s
2
4
6
8
Median Heatwave Severity

Supplementary Figure 74: Kernel Density Estimates (KDE) across Decades for Maximum Heatwave Severity in RCP 2.6 CMIP5 multi-model ensemble (MME) (Tx90p) - Kernel Density Estimates (KDE) of Maximum Heatwave Severity for different Decades in the CMIP5 multi-model ensemble (MME). Area under each curve is normalized to 1. Maximum Heatwave Severity of the observed event is indicated by vertical lines in dark blue (Berkeley Earth) and light blue (ERA5).

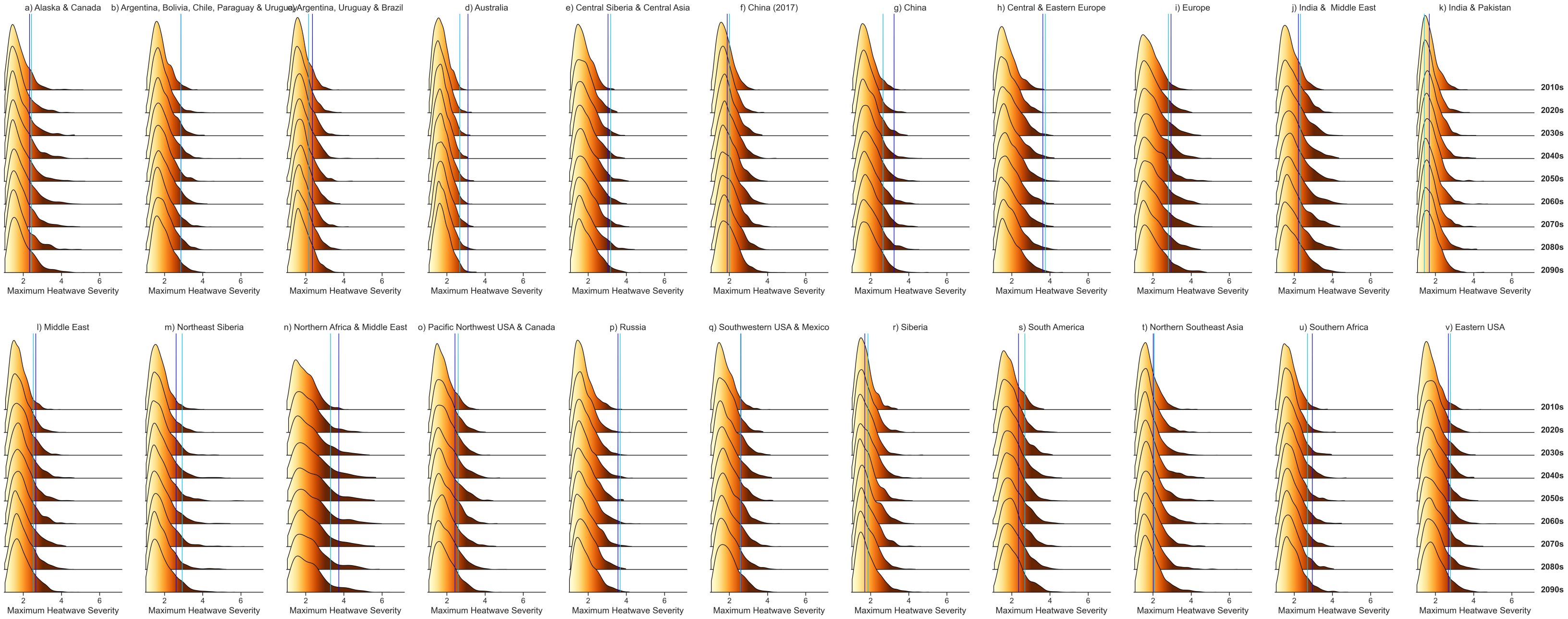
a) Alaska & Canada
b) Argentina, Bolivia, Chile, Paraguay & Uruguay
c) Argentina, Uruguay & Brazil
d) Australia
e) Central Siberia & Central Asia
f) China (2017)
g) China
h) Central & Eastern Europe
i) Europe
j) India & Middle East
k) India & Pakistan
l) Middle East
m) Northeast Siberia
n) Northern Africa & Middle East
o) Pacific Northwest USA & Canada
p) Russia
q) Southwestern USA & Mexico
r) Siberia
s) South America
t) Northern Southeast Asia
u) Southern Africa
v) Eastern USA
2010s
2020s
2030s
2040s
2050s
2060s
2070s
2080s
2090s
2
4
6
Maximum Heatwave Severity

Supplementary Figure 75: Kernel Density Estimates (KDE) across Decades for Maximum Heatwave Severity in RCP 4.5 CMIP5 multi-model ensemble (MME) (Tx90p) - Kernel Density Estimates (KDE) of Maximum Heatwave Severity for different Decades in the CMIP5 multi-model ensemble (MME). Area under each curve is normalized to 1. Maximum Heatwave Severity of the observed event is indicated by vertical lines in dark blue (Berkeley Earth) and light blue (ERA5).

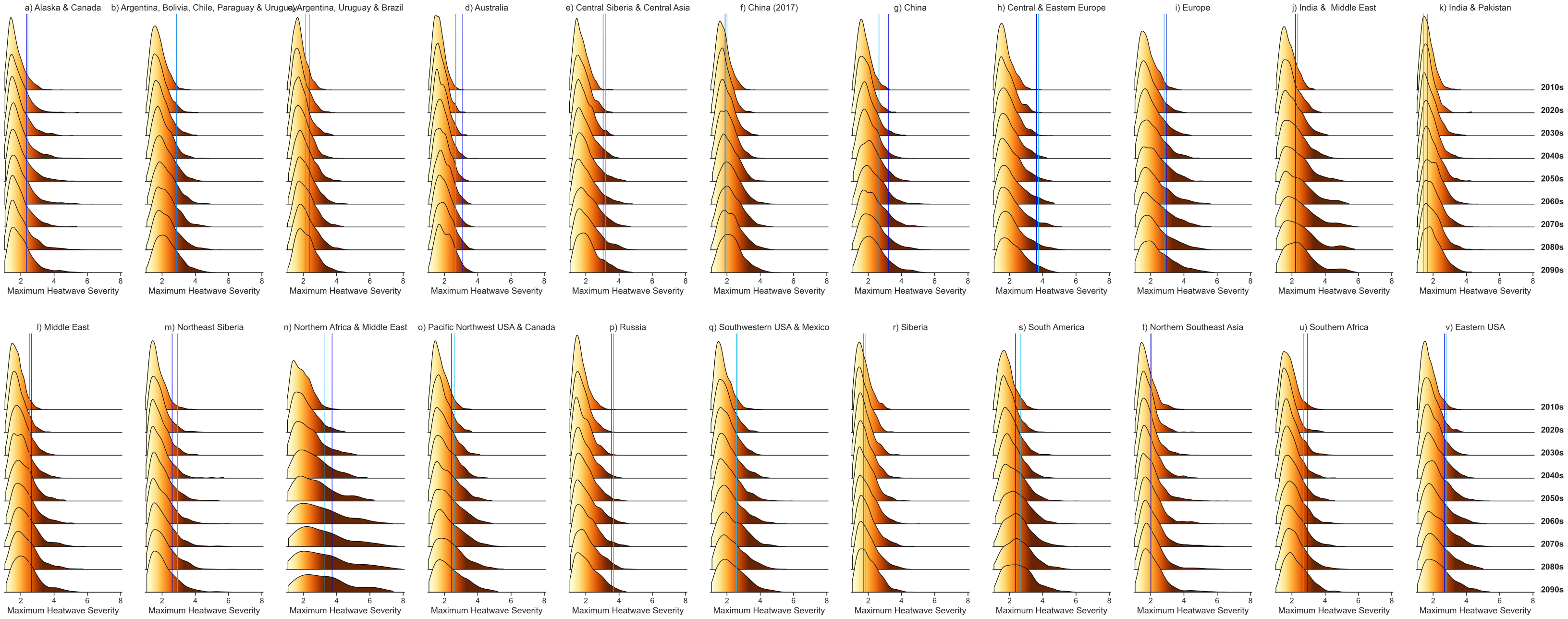

a) Alaska & Canada
b) Argentina, Bolivia, Chile, Paraguay & Uruguay
c) Argentina, Uruguay & Brazil
d) Australia
e) Central Siberia & Central Asia
f) China (2017)
g) China
h) Central & Eastern Europe
i) Europe
j) India & Middle East
k) India & Pakistan
l) Middle East
m) Northeast Siberia
n) Northern Africa & Middle East
o) Pacific Northwest USA & Canada
p) Russia
q) Southwestern USA & Mexico
r) Siberia
s) South America
t) Northern Southeast Asia
u) Southern Africa
v) Eastern USA
2010s
2020s
2030s
2040s
2050s
2060s
2070s
2080s
2090s
2
4
6
8
Maximum Heatwave Severity

Supplementary Figure 76: Kernel Density Estimates (KDE) across Decades for Maximum Heatwave Severity in RCP 8.5 CMIP5 multi-model ensemble (MME) (Tx90p) - Kernel Density Estimates (KDE) of Maximum Heatwave Severity for different Decades in the CMIP5 multi-model ensemble (MME). Area under each curve is normalized to 1. Maximum Heatwave Severity of the observed event is indicated by vertical lines in dark blue (Berkeley Earth) and light blue (ERA5).

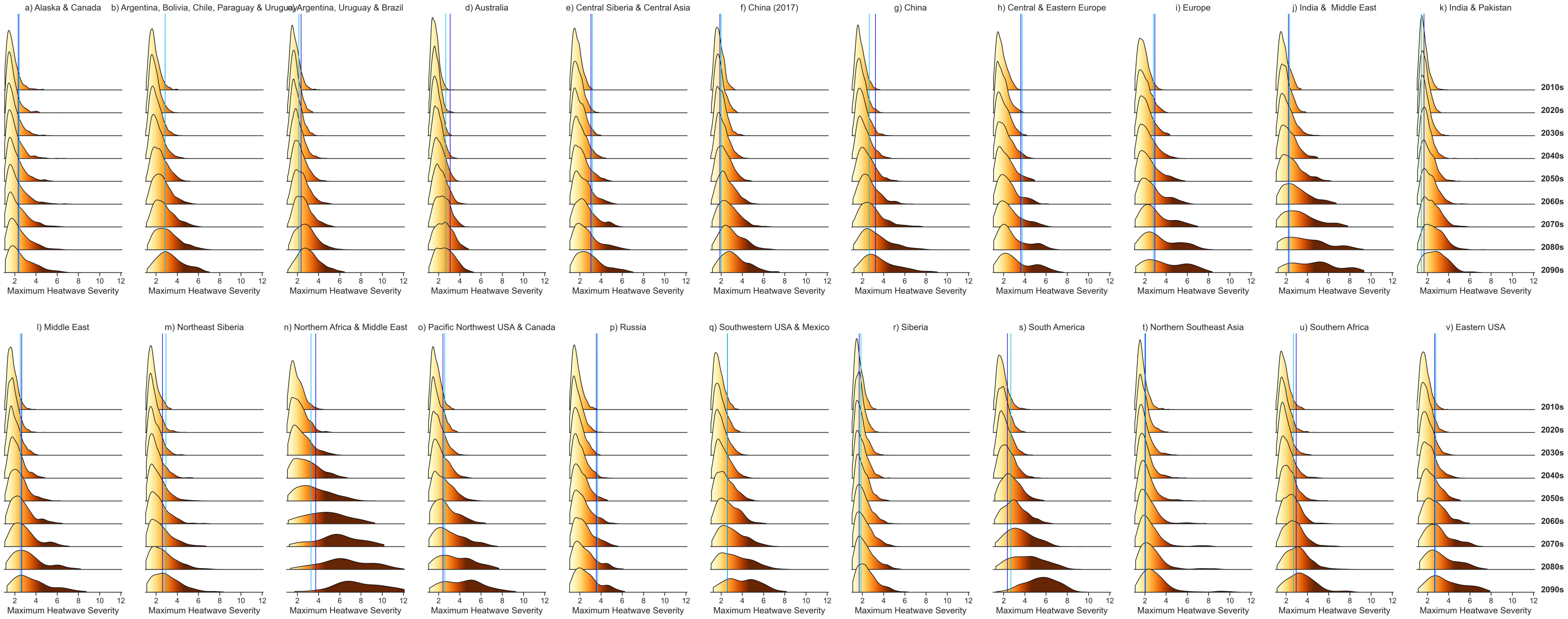
a) Alaska & Canada
b) Argentina, Bolivia, Chile, Paraguay & Uruguay
c) Argentina, Uruguay & Brazil
d) Australia
e) Central Siberia & Central Asia
f) China (2017)
g) China
h) Central & Eastern Europe
i) Europe
j) India & Middle East
k) India & Pakistan
l) Middle East
m) Northeast Siberia
n) Northern Africa & Middle East
o) Pacific Northwest USA & Canada
p) Russia
q) Southwestern USA & Mexico
r) Siberia
s) South America
t) Northern Southeast Asia
u) Southern Africa
v) Eastern USA
2010s
2020s
2030s
2040s
2050s
2060s
2070s
2080s
2090s
2 4 6 8 10 12
Maximum Heatwave Severity

Supplementary Figure 77: Kernel Density Estimates (KDE) across Decades for Median Heatwave Area (%) in RCP 2.6 CMIP5 multi-model ensemble (MME) (Tx90p) - Kernel Density Estimates (KDE) of Median Heatwave Area (%) for different Decades in the CMIP5 multi-model ensemble (MME). Area under each curve is normalized to 1. Median Heatwave Area (%) of the observed event is indicated by vertical lines in dark blue (Berkeley Earth) and light blue (ERA5).

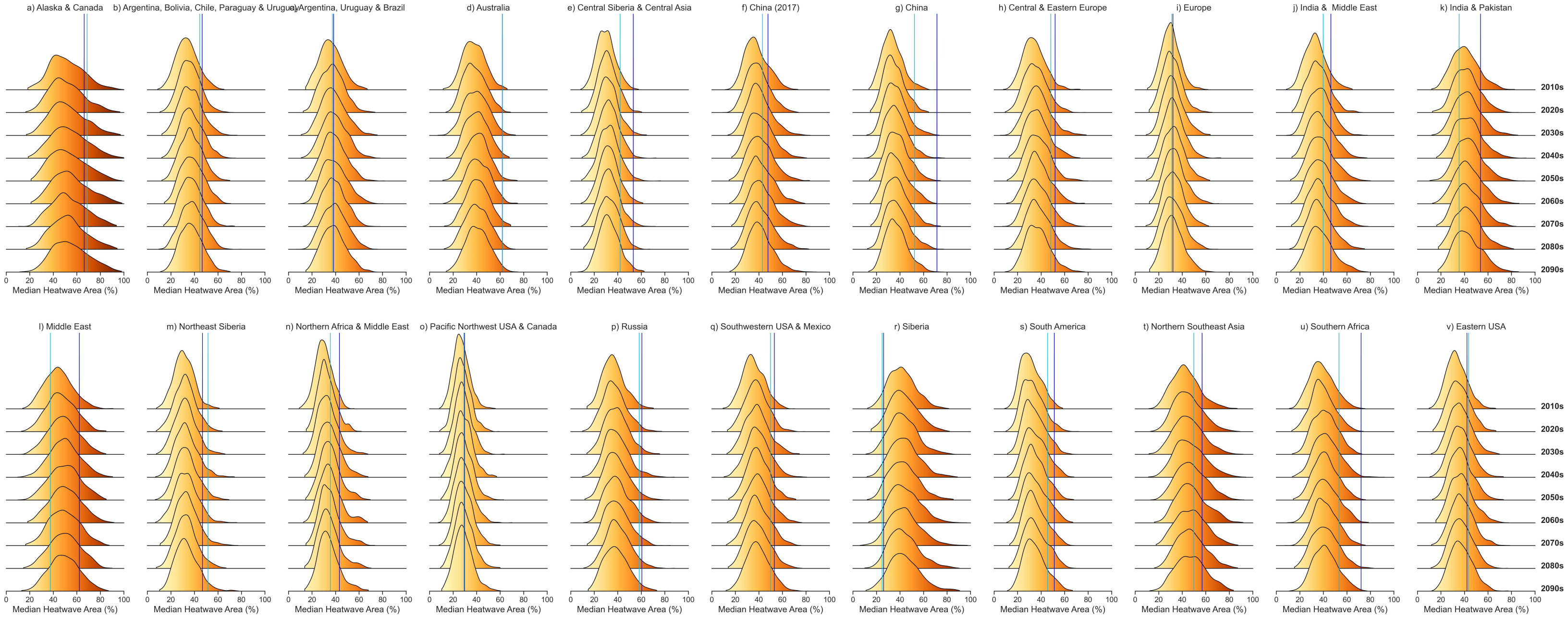
a) Alaska & Canada
b) Argentina, Bolivia, Chile, Paraguay & Uruguay
c) Argentina, Uruguay & Brazil
d) Australia
e) Central Siberia & Central Asia
f) China (2017)
g) China
h) Central & Eastern Europe
i) Europe
j) India & Middle East
k) India & Pakistan
l) Middle East
m) Northeast Siberia
n) Northern Africa & Middle East
o) Pacific Northwest USA & Canada
p) Russia
q) Southwestern USA & Mexico
r) Siberia
s) South America
t) Northern Southeast Asia
u) Southern Africa
v) Eastern USA
2010s
2020s
2030s
2040s
2050s
2060s
2070s
2080s
2090s
Median Heatwave Area (%)
0 20 40 60 80 100

Supplementary Figure 78: Kernel Density Estimates (KDE) across Decades for Median Heatwave Area (%) in RCP 4.5 CMIP5 multi-model ensemble (MME) (Tx90p) - Kernel Density Estimates (KDE) of Median Heatwave Area (%) for different Decades in the CMIP5 multi-model ensemble (MME). Area under each curve is normalized to 1. Median Heatwave Area (%) of the observed event is indicated by vertical lines in dark blue (Berkeley Earth) and light blue (ERA5).

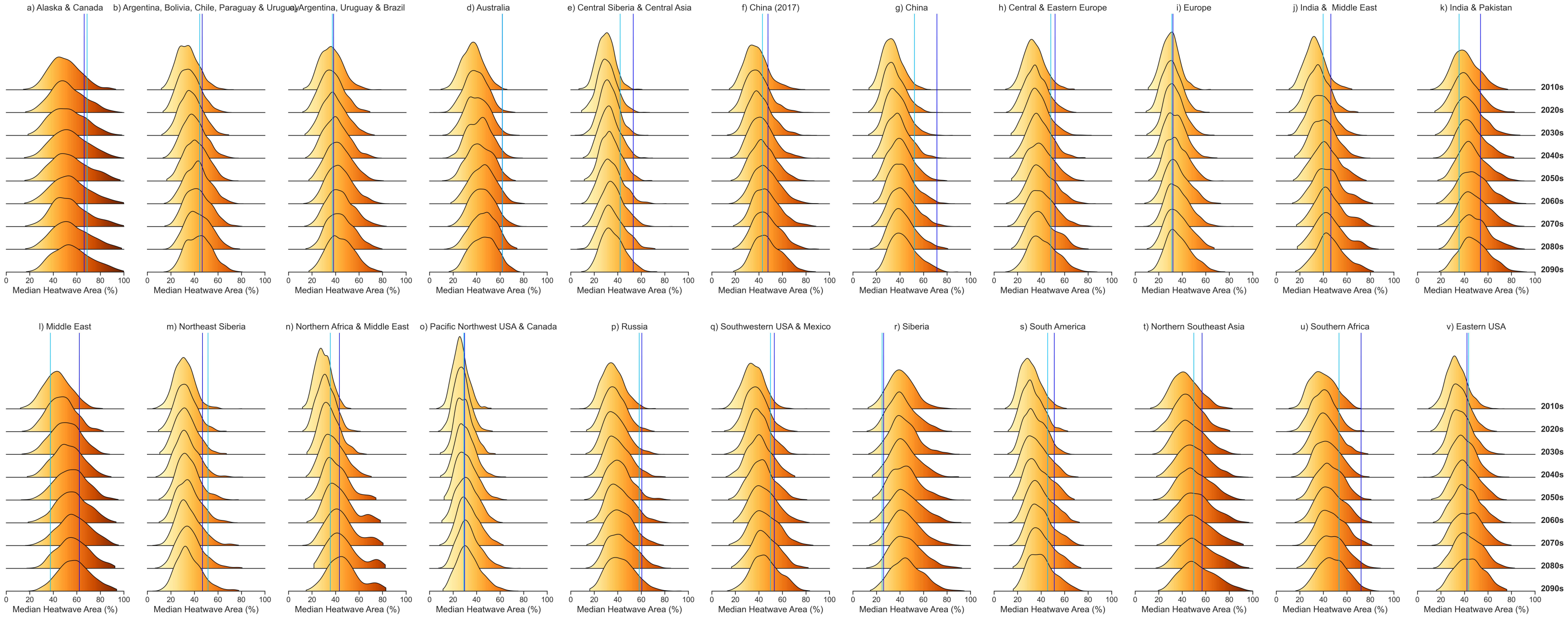
a) Alaska & Canada
b) Argentina, Bolivia, Chile, Paraguay & Uruguay
c) Argentina, Uruguay & Brazil
d) Australia
e) Central Siberia & Central Asia
f) China (2017)
g) China
h) Central & Eastern Europe
i) Europe
j) India & Middle East
k) India & Pakistan
l) Middle East
m) Northeast Siberia
n) Northern Africa & Middle East
o) Pacific Northwest USA & Canada
p) Russia
q) Southwestern USA & Mexico
r) Siberia
s) South America
t) Northern Southeast Asia
u) Southern Africa
v) Eastern USA
2010s
2020s
2030s
2040s
2050s
2060s
2070s
2080s
2090s
Median Heatwave Area (%)
0 20 40 60 80 100

Supplementary Figure 79: Kernel Density Estimates (KDE) across Decades for Median Heatwave Area (%) in RCP 8.5 CMIP5 multi-model ensemble (MME) (Tx90p) - Kernel Density Estimates (KDE) of Median Heatwave Area (%) for different Decades in the CMIP5 multi-model ensemble (MME). Area under each curve is normalized to 1. Median Heatwave Area (%) of the observed event is indicated by vertical lines in dark blue (Berkeley Earth) and light blue (ERA5).

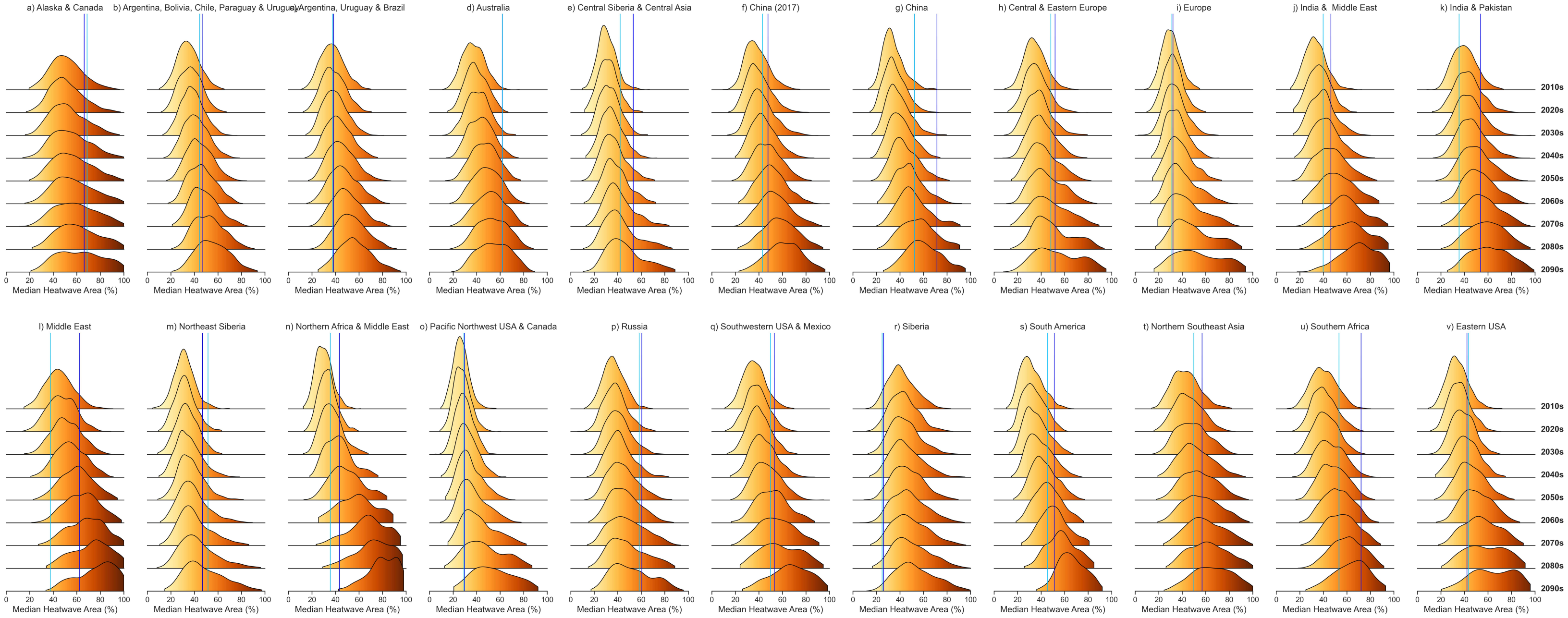
a) Alaska & Canada
b) Argentina, Bolivia, Chile, Paraguay & Uruguay
c) Argentina, Uruguay & Brazil
d) Australia
e) Central Siberia & Central Asia
f) China (2017)
g) China
h) Central & Eastern Europe
i) Europe
j) India & Middle East
k) India & Pakistan
l) Middle East
m) Northeast Siberia
n) Northern Africa & Middle East
o) Pacific Northwest USA & Canada
p) Russia
q) Southwestern USA & Mexico
r) Siberia
s) South America
t) Northern Southeast Asia
u) Southern Africa
v) Eastern USA
2010s
2020s
2030s
2040s
2050s
2060s
2070s
2080s
2090s
0
20
40
60
80
100
Median Heatwave Area (%)

Supplementary Figure 80: Kernel Density Estimates (KDE) across Decades for Maximum Heatwave Area (%) in RCP 2.6 CMIP5 multi-model ensemble (MME) (Tx90p) - Kernel Density Estimates (KDE) of Maximum Heatwave Area (%) for different Decades in the CMIP5 multi-model ensemble (MME). Area under each curve is normalized to 1. Maximum Heatwave Area (%) of the observed event is indicated by vertical lines in dark blue (Berkeley Earth) and light blue (ERA5).

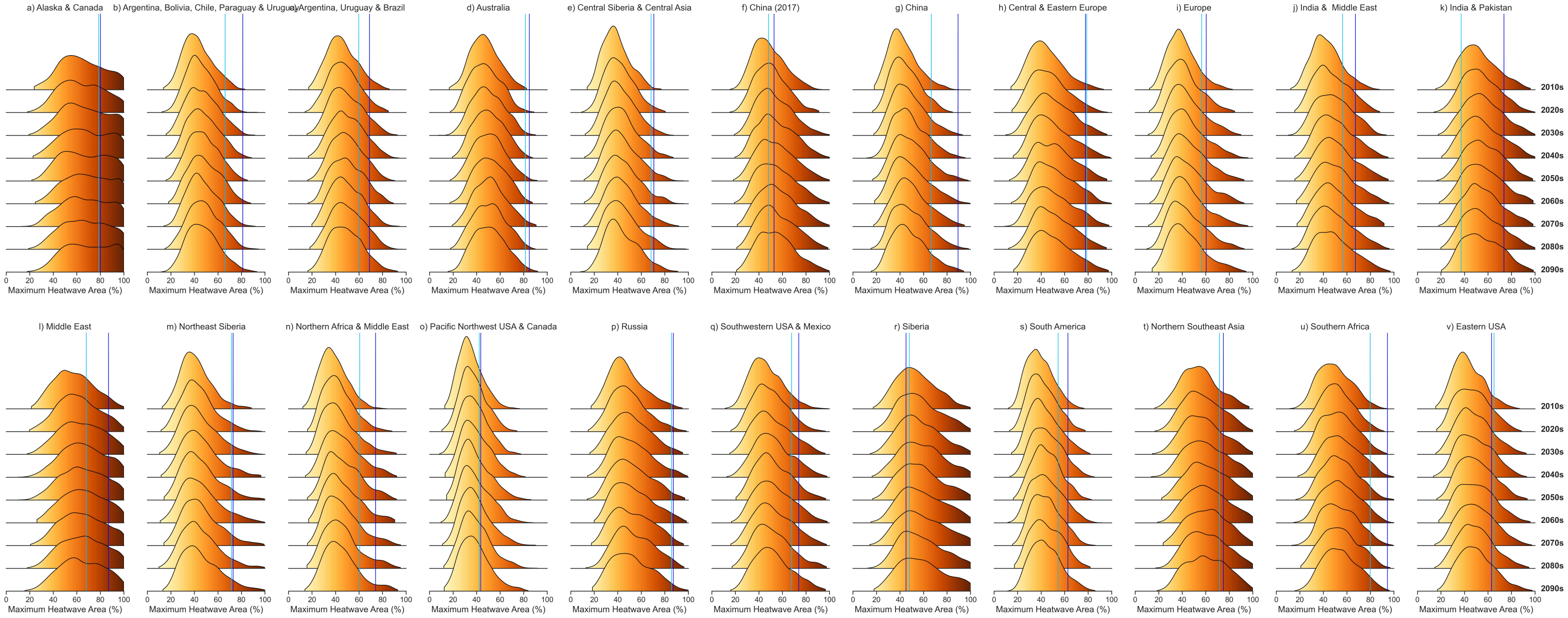
a) Alaska & Canada
b) Argentina, Bolivia, Chile, Paraguay & Uruguay
c) Argentina, Uruguay & Brazil
d) Australia
e) Central Siberia & Central Asia
f) China (2017)
g) China
h) Central & Eastern Europe
i) Europe
j) India & Middle East
k) India & Pakistan
l) Middle East
m) Northeast Siberia
n) Northern Africa & Middle East
o) Pacific Northwest USA & Canada
p) Russia
q) Southwestern USA & Mexico
r) Siberia
s) South America
t) Northern Southeast Asia
u) Southern Africa
v) Eastern USA
2010s
2020s
2030s
2040s
2050s
2060s
2070s
2080s
2090s
0 20 40 60 80 100
Maximum Heatwave Area (%)

Supplementary Figure 81: Kernel Density Estimates (KDE) across Decades for Maximum Heatwave Area (%) in RCP 4.5 CMIP5 multi-model ensemble (MME) (Tx90p) - Kernel Density Estimates (KDE) of Maximum Heatwave Area (%) for different Decades in the CMIP5 multi-model ensemble (MME). Area under each curve is normalized to 1. Maximum Heatwave Area (%) of the observed event is indicated by vertical lines in dark blue (Berkeley Earth) and light blue (ERA5).

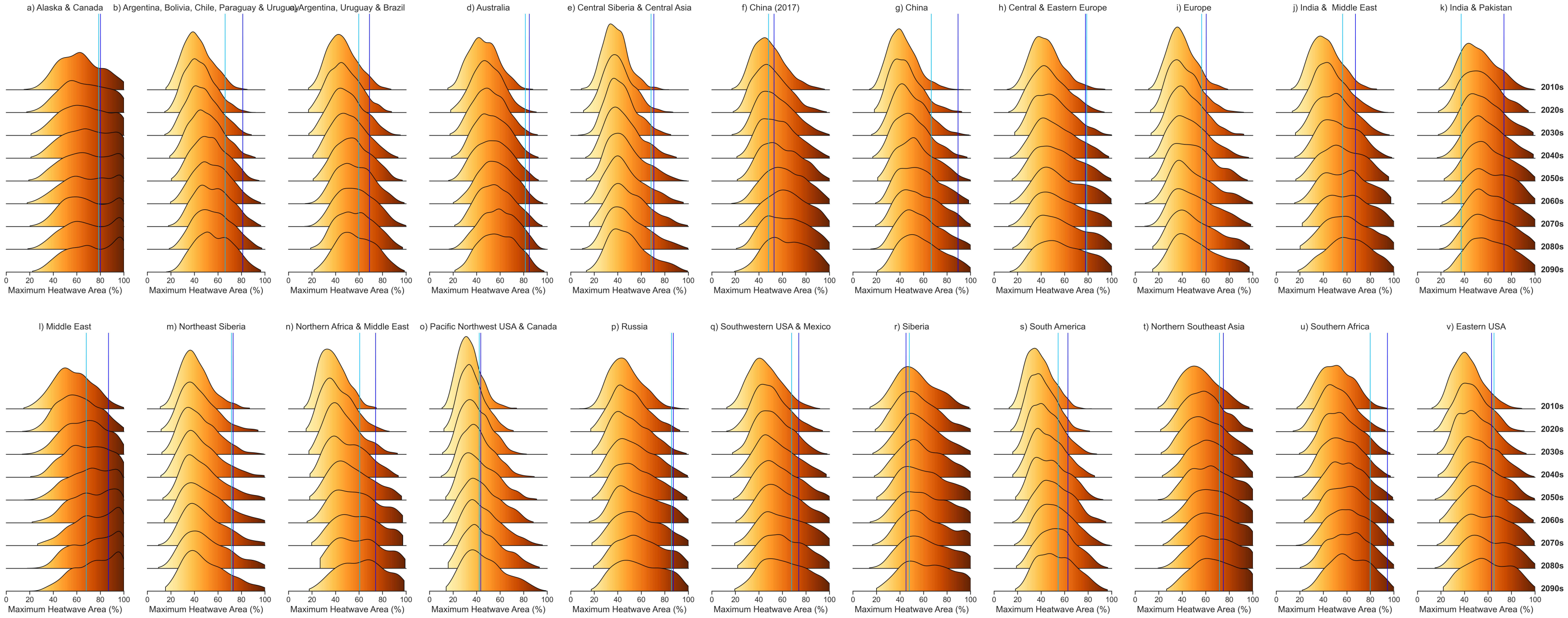

a) Alaska & Canada
b) Argentina, Bolivia, Chile, Paraguay & Uruguay
c) Argentina, Uruguay & Brazil
d) Australia
e) Central Siberia & Central Asia
f) China (2017)
g) China
h) Central & Eastern Europe
i) Europe
j) India & Middle East
k) India & Pakistan
l) Middle East
m) Northeast Siberia
n) Northern Africa & Middle East
o) Pacific Northwest USA & Canada
p) Russia
q) Southwestern USA & Mexico
r) Siberia
s) South America
t) Northern Southeast Asia
u) Southern Africa
v) Eastern USA
2010s
2020s
2030s
2040s
2050s
2060s
2070s
2080s
2090s
0 20 40 60 80 100
Maximum Heatwave Area (%)

Supplementary Figure 82: Kernel Density Estimates (KDE) across Decades for Maximum Heatwave Area (%) in RCP 8.5 CMIP5 multi-model ensemble (MME) (Tx90p) - Kernel Density Estimates (KDE) of Maximum Heatwave Area (%) for different Decades in the CMIP5 multi-model ensemble (MME). Area under each curve is normalized to 1. Maximum Heatwave Area (%) of the observed event is indicated by vertical lines in dark blue (Berkeley Earth) and light blue (ERA5).

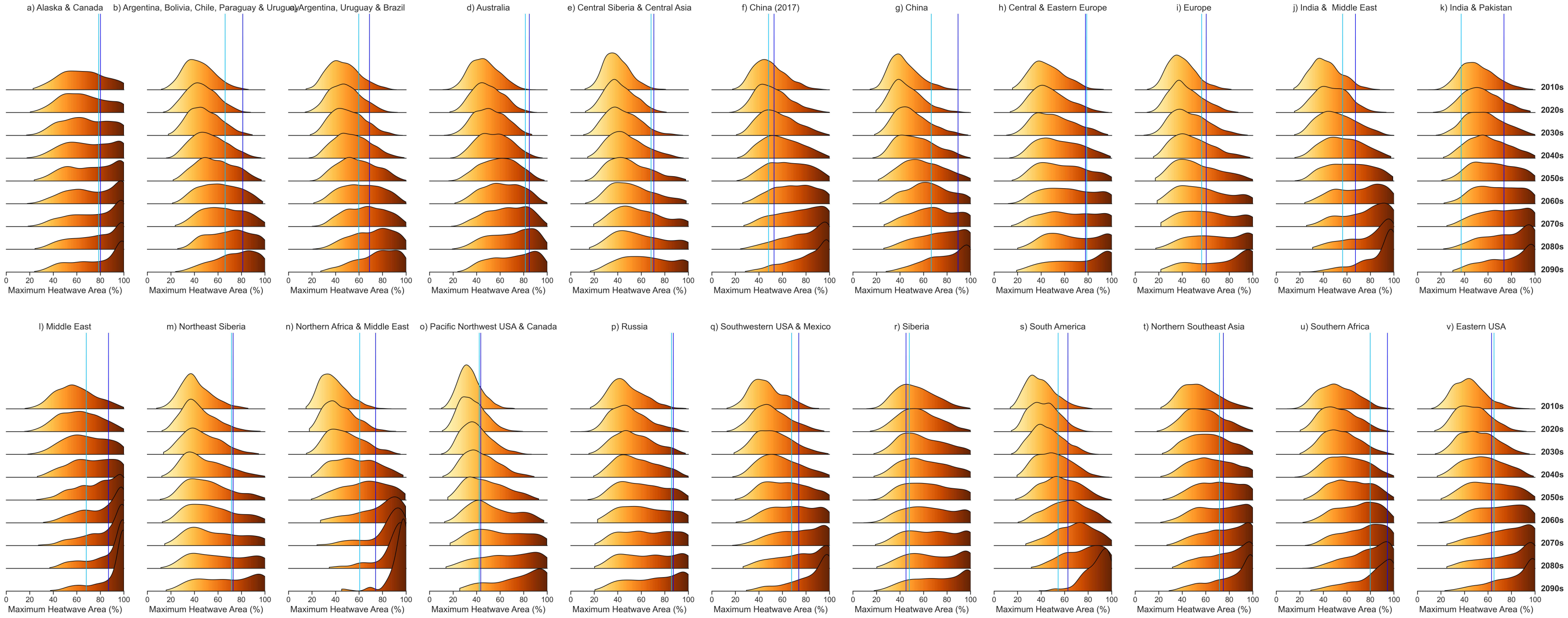

a) Alaska & Canada
b) Argentina, Bolivia, Chile, Paraguay & Uruguay
c) Argentina, Uruguay & Brazil
d) Australia
e) Central Siberia & Central Asia
f) China (2017)
g) China
h) Central & Eastern Europe
i) Europe
j) India & Middle East
k) India & Pakistan
l) Middle East
m) Northeast Siberia
n) Northern Africa & Middle East
o) Pacific Northwest USA & Canada
p) Russia
q) Southwestern USA & Mexico
r) Siberia
s) South America
t) Northern Southeast Asia
u) Southern Africa
v) Eastern USA
2010s
2020s
2030s
2040s
2050s
2060s
2070s
2080s
2090s
0
20
40
60
80
100
Maximum Heatwave Area (%)

Supplementary Figure 83: Multi-HW-characteristic figure according to Historical CMIP6 multi-model ensemble (MME) (Tx95.0p) - Each panel shows regional projections according to Historical model runs in the CMIP6 multi-model ensemble (MME) of four combined heatwave characteristics for each projected heatwave in the ensemble between 2020 and 2100. Each dot represents an individual heatwave in the projections, characterized by Maximum Heatwave Area (%) (x-axis) as latitude-weighted percentage of grid boxes affected by extreme heat, Heatwave Length (days) (y-axis) in days, Maximum Heatwave Severity (color), and Cumulative Heat (°C) (dot size). The characteristics of the observed event according to the Berkeley Earth observational data (x symbol) and the ERA5 reanalysis data (+ symbol), respectively, are pointed to by an arrow and a figure inset shows a zoom-in on this figure section. The figure inset shows the observed heatwave in the two reference data sets and projections in the indicated section of the respective sub-plot. It facilitates readability of color and size of the reference values and comparison to each other and the neighboring projections. Note that the zoom-in causes the dots to overlap more in the inset than in the main sub-plot. The univariate kernel density estimates along the x- (Maximum Heatwave Area (%)) and y-axis (Heatwave Length (days)) are on the top and right side of the subplots, respectively, with vertical lines in dark blue (Berkeley Earth) and light blue (ERA5) indicating the observed event.

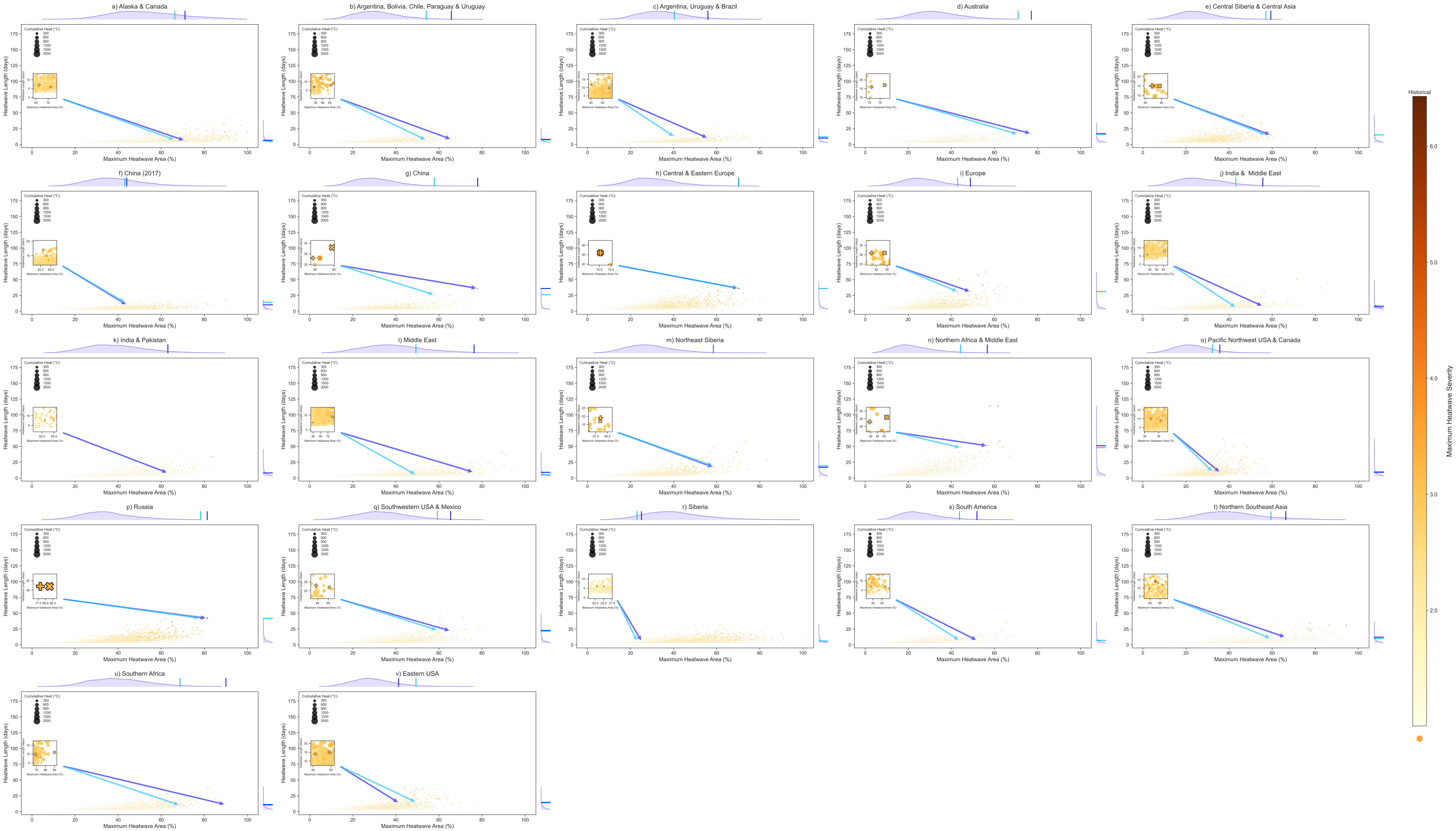

a) Alaska & Canada
b) Argentina, Bolivia, Chile, Paraguay & Uruguay
c) Argentina, Uruguay & Brazil
d) Australia
e) Central Siberia & Central Asia
f) China (2017)
g) China
h) Central & Eastern Europe
i) Europe
j) India & Middle East
k) India & Pakistan
l) Middle East
m) Northeast Siberia
n) Northern Africa & Middle East
o) Pacific Northwest USA & Canada
p) Russia
q) Southwestern USA & Mexico
r) Siberia
s) South America
t) Northern Southeast Asia
u) Southern Africa
v) Eastern USA
Heatwave Length (days)
Maximum Heatwave Area (%)
Cumulative Heat (°C)
300
600
900
1200
1500
2000
Historical
Maximum Heatwave Severity

Supplementary Figure 84: Multi-HW-characteristic figure according to Historical CMIP6 multi-model ensemble (MME) (Tx97.5p) - Each panel shows regional projections according to Historical model runs in the CMIP6 multi-model ensemble (MME) of four combined heatwave characteristics for each projected heatwave in the ensemble between 2020 and 2100. Each dot represents an individual heatwave in the projections, characterized by Maximum Heatwave Area (%) (x-axis) as latitude-weighted percentage of grid boxes affected by extreme heat, Heatwave Length (days) (y-axis) in days, Maximum Heatwave Severity (color), and Cumulative Heat (°C) (dot size). The characteristics of the observed event according to the Berkeley Earth observational data (x symbol) and the ERA5 reanalysis data (+ symbol), respectively, are pointed to by an arrow and a figure inset shows a zoom-in on this figure section. The figure inset shows the observed heatwave in the two reference data sets and projections in the indicated section of the respective sub-plot. It facilitates readability of color and size of the reference values and comparison to each other and the neighboring projections. Note that the zoom-in causes the dots to overlap more in the inset than in the main sub-plot. The univariate kernel density estimates along the x- (Maximum Heatwave Area (%)) and y-axis (Heatwave Length (days)) are on the top and right side of the subplots, respectively, with vertical lines in dark blue (Berkeley Earth) and light blue (ERA5) indicating the observed event.

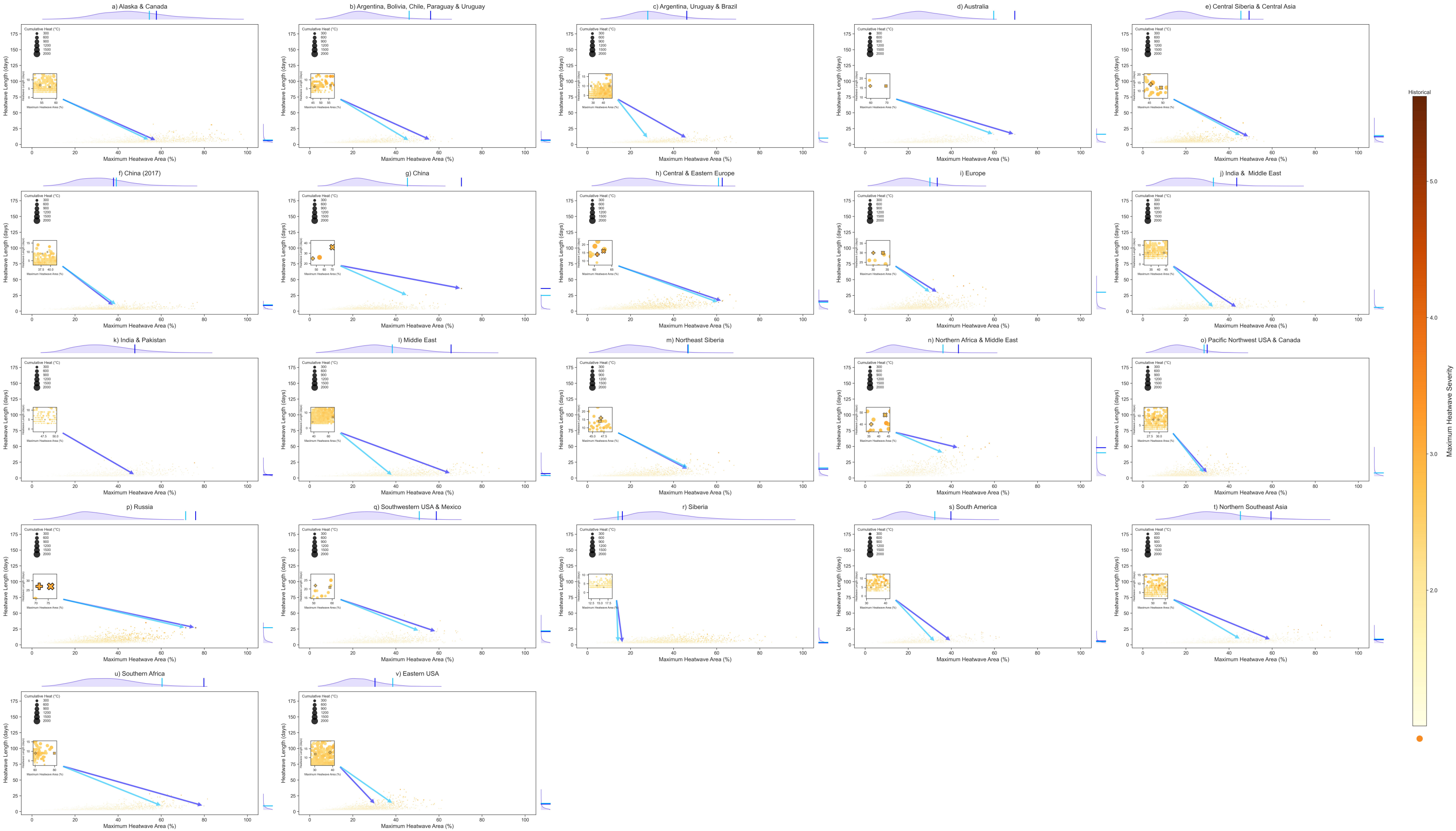

a) Alaska & Canada
b) Argentina, Bolivia, Chile, Paraguay & Uruguay
c) Argentina, Uruguay & Brazil
d) Australia
e) Central Siberia & Central Asia
f) China (2017)
g) China
h) Central & Eastern Europe
i) Europe
j) India & Middle East
k) India & Pakistan
l) Middle East
m) Northeast Siberia
n) Northern Africa & Middle East
o) Pacific Northwest USA & Canada
p) Russia
q) Southwestern USA & Mexico
r) Siberia
s) South America
t) Northern Southeast Asia
u) Southern Africa
v) Eastern USA
Cumulative Heat (°C)
300
600
900
1200
1500
2000
Heatwave Length (days)
Maximum Heatwave Area (%)
Historical
Maximum Heatwave Severity
2.0
3.0
4.0
5.0

Supplementary Figure 85: Multi-HW-characteristic figure according to Historical CMIP6 multi-model ensemble (MME) (Tx99.0p) - Each panel shows regional projections according to Historical model runs in the CMIP6 multi-model ensemble (MME) of four combined heatwave characteristics for each projected heatwave in the ensemble between 2020 and 2100. Each dot represents an individual heatwave in the projections, characterized by Maximum Heatwave Area (%) (x-axis) as latitude-weighted percentage of grid boxes affected by extreme heat, Heatwave Length (days) (y-axis) in days, Maximum Heatwave Severity (color), and Cumulative Heat (°C) (dot size). The characteristics of the observed event according to the Berkeley Earth observational data (x symbol) and the ERA5 reanalysis data (+ symbol), respectively, are pointed to by an arrow and a figure inset shows a zoom-in on this figure section. The figure inset shows the observed heatwave in the two reference data sets and projections in the indicated section of the respective sub-plot. It facilitates readability of color and size of the reference values and comparison to each other and the neighboring projections. Note that the zoom-in causes the dots to overlap more in the inset than in the main sub-plot. The univariate kernel density estimates along the x- (Maximum Heatwave Area (%)) and y-axis (Heatwave Length (days)) are on the top and right side of the subplots, respectively, with vertical lines in dark blue (Berkeley Earth) and light blue (ERA5) indicating the observed event.

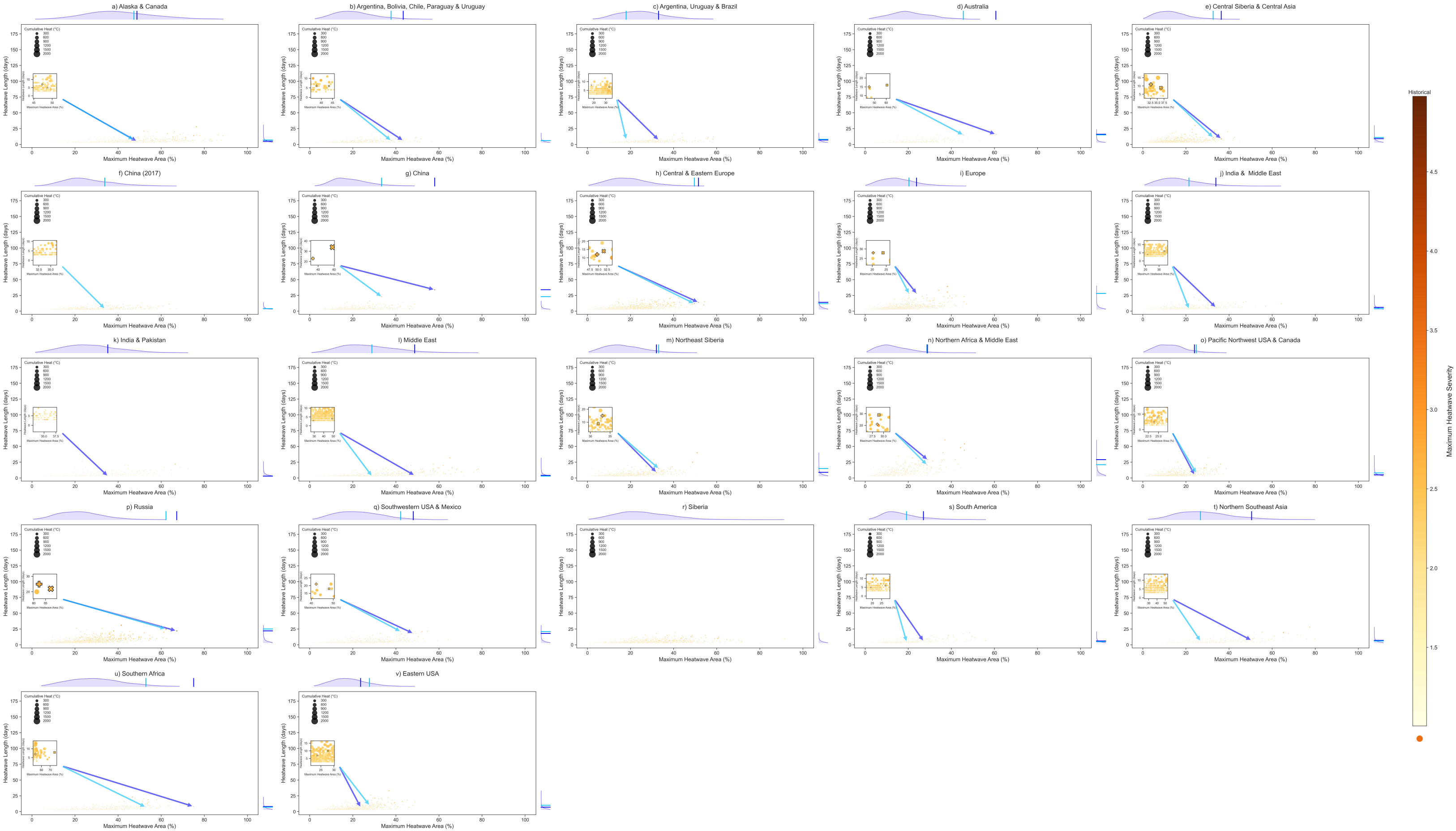

a) Alaska & Canada
b) Argentina, Bolivia, Chile, Paraguay & Uruguay
c) Argentina, Uruguay & Brazil
d) Australia
e) Central Siberia & Central Asia
f) China (2017)
g) China
h) Central & Eastern Europe
i) Europe
j) India & Middle East
k) India & Pakistan
l) Middle East
m) Northeast Siberia
n) Northern Africa & Middle East
o) Pacific Northwest USA & Canada
p) Russia
q) Southwestern USA & Mexico
r) Siberia
s) South America
t) Northern Southeast Asia
u) Southern Africa
v) Eastern USA
Cumulative Heat (°C)
300
600
900
1200
1500
2000
Heatwave Length (days)
Maximum Heatwave Area (%)
Historical
Maximum Heatwave Severity

Supplementary Figure 86: Multi-HW-characteristic figure according to SSP1-2.6 CMIP6 multi-model ensemble (MME) (Tx95.0p) - Each panel shows regional projections according to SSP1-2.6 model runs in the CMIP6 multi-model ensemble (MME) of four combined heatwave characteristics for each projected heatwave in the ensemble between 2020 and 2100. Each dot represents an individual heatwave in the projections, characterized by Maximum Heatwave Area (%) (x-axis) as latitude-weighted percentage of grid boxes affected by extreme heat, Heatwave Length (days) (y-axis) in days, Maximum Heatwave Severity (color), and Cumulative Heat (°C) (dot size). The characteristics of the observed event according to the Berkeley Earth observational data (x symbol) and the ERA5 reanalysis data (+ symbol), respectively, are pointed to by an arrow and a figure inset shows a zoom-in on this figure section. The figure inset shows the observed heatwave in the two reference data sets and projections in the indicated section of the respective sub-plot. It facilitates readability of color and size of the reference values and comparison to each other and the neighboring projections. Note that the zoom-in causes the dots to overlap more in the inset than in the main sub-plot. The univariate kernel density estimates along the x- (Maximum Heatwave Area (%)) and y-axis (Heatwave Length (days)) are on the top and right side of the subplots, respectively, with vertical lines in dark blue (Berkeley Earth) and light blue (ERA5) indicating the observed event.

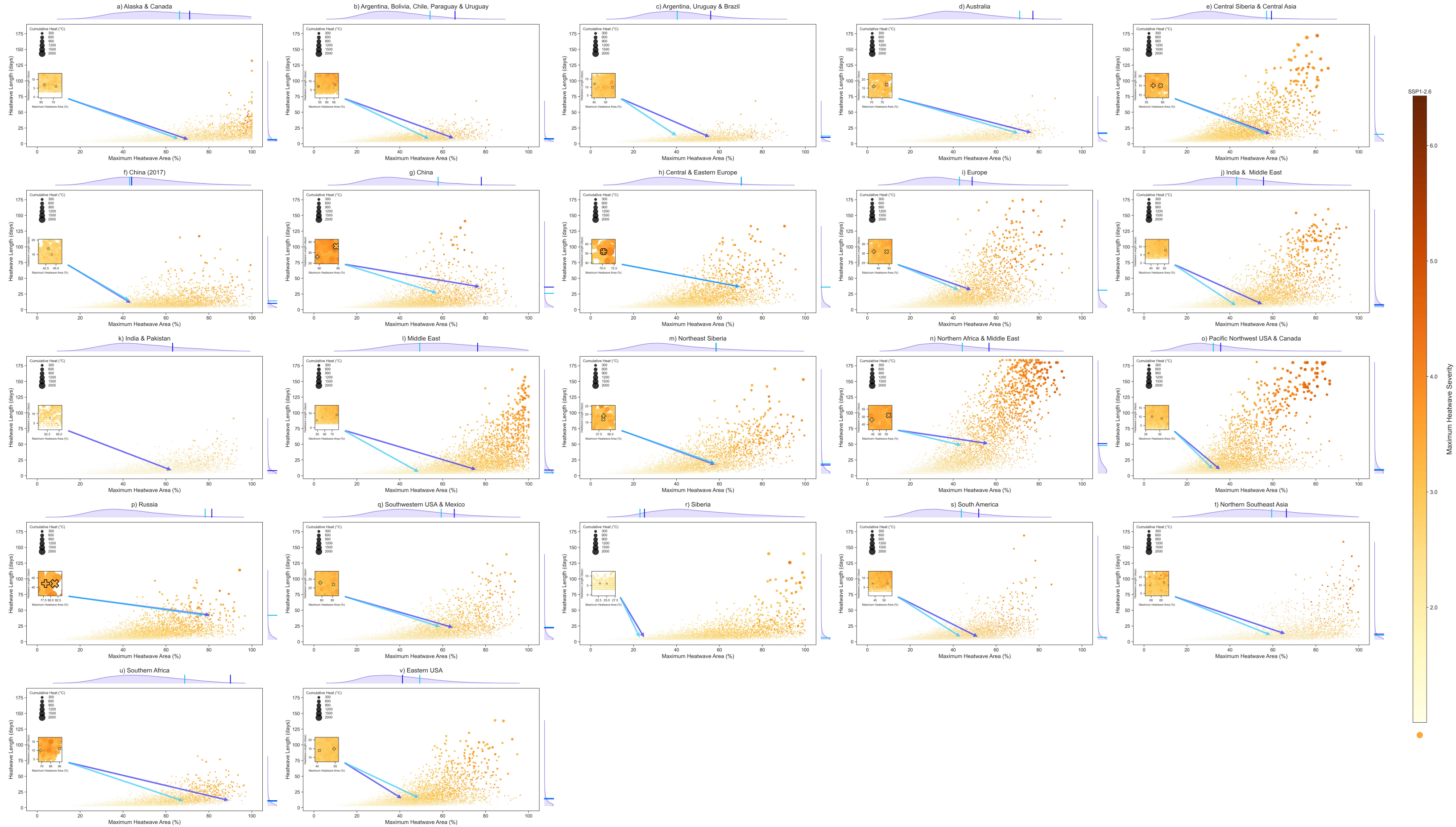

a) Alaska & Canada
b) Argentina, Bolivia, Chile, Paraguay & Uruguay
c) Argentina, Uruguay & Brazil
d) Australia
e) Central Siberia & Central Asia
f) China (2017)
g) China
h) Central & Eastern Europe
i) Europe
j) India & Middle East
k) India & Pakistan
l) Middle East
m) Northeast Siberia
n) Northern Africa & Middle East
o) Pacific Northwest USA & Canada
p) Russia
q) Southwestern USA & Mexico
r) Siberia
s) South America
t) Northern Southeast Asia
u) Southern Africa
v) Eastern USA
Cumulative Heat (°C)
300
600
900
1200
1500
2000
Heatwave Length (days)
Maximum Heatwave Area (%)
SSP1-2.6
Maximum Heatwave Severity

Supplementary Figure 87: Multi-HW-characteristic figure according to SSP1-2.6 CMIP6 multi-model ensemble (MME) (Tx97.5p) - Each panel shows regional projections according to SSP1-2.6 model runs in the CMIP6 multi-model ensemble (MME) of four combined heatwave characteristics for each projected heatwave in the ensemble between 2020 and 2100. Each dot represents an individual heatwave in the projections, characterized by Maximum Heatwave Area (%) (x-axis) as latitude-weighted percentage of grid boxes affected by extreme heat, Heatwave Length (days) (y-axis) in days, Maximum Heatwave Severity (color), and Cumulative Heat (°C) (dot size). The characteristics of the observed event according to the Berkeley Earth observational data (x symbol) and the ERA5 reanalysis data (+ symbol), respectively, are pointed to by an arrow and a figure inset shows a zoom-in on this figure section. The figure inset shows the observed heatwave in the two reference data sets and projections in the indicated section of the respective sub-plot. It facilitates readability of color and size of the reference values and comparison to each other and the neighboring projections. Note that the zoom-in causes the dots to overlap more in the inset than in the main sub-plot. The univariate kernel density estimates along the x- (Maximum Heatwave Area (%)) and y-axis (Heatwave Length (days)) are on the top and right side of the subplots, respectively, with vertical lines in dark blue (Berkeley Earth) and light blue (ERA5) indicating the observed event.

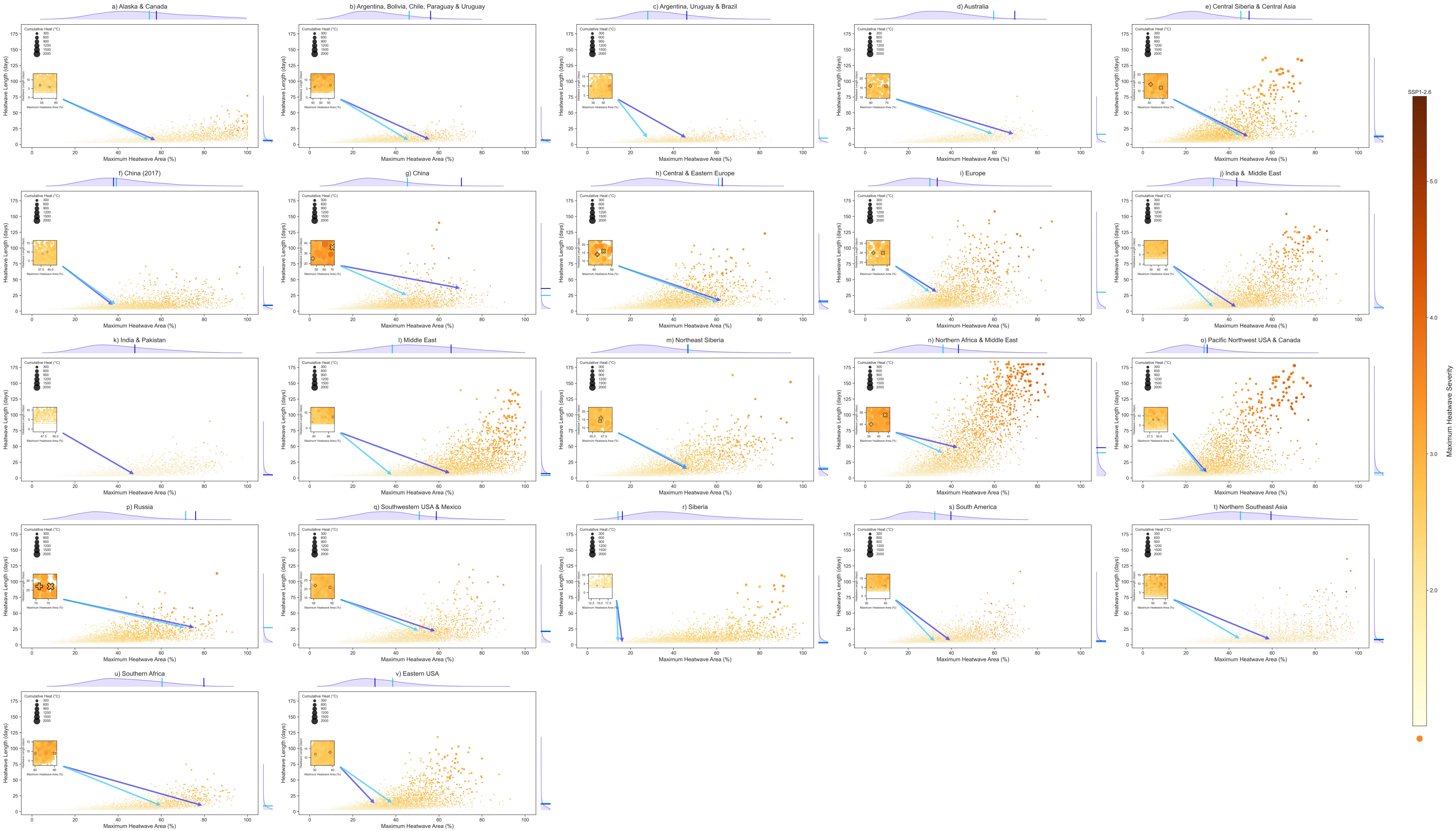

a) Alaska & Canada
b) Argentina, Bolivia, Chile, Paraguay & Uruguay
c) Argentina, Uruguay & Brazil
d) Australia
e) Central Siberia & Central Asia
f) China (2017)
g) China
h) Central & Eastern Europe
i) Europe
j) India & Middle East
k) India & Pakistan
l) Middle East
m) Northeast Siberia
n) Northern Africa & Middle East
o) Pacific Northwest USA & Canada
p) Russia
q) Southwestern USA & Mexico
r) Siberia
s) South America
t) Northern Southeast Asia
u) Southern Africa
v) Eastern USA
Heatwave Length (days)
Maximum Heatwave Area (%)
Cumulative Heat (°C)
300
600
900
1200
1500
2000
SSP1-2.6
Maximum Heatwave Severity

Supplementary Figure 88: Multi-HW-characteristic figure according to SSP1-2.6 CMIP6 multi-model ensemble (MME) (Tx99.0p) - Each panel shows regional projections according to SSP1-2.6 model runs in the CMIP6 multi-model ensemble (MME) of four combined heatwave characteristics for each projected heatwave in the ensemble between 2020 and 2100. Each dot represents an individual heatwave in the projections, characterized by Maximum Heatwave Area (%) (x-axis) as latitude-weighted percentage of grid boxes affected by extreme heat, Heatwave Length (days) (y-axis) in days, Maximum Heatwave Severity (color), and Cumulative Heat (°C) (dot size). The characteristics of the observed event according to the Berkeley Earth observational data (x symbol) and the ERA5 reanalysis data (+ symbol), respectively, are pointed to by an arrow and a figure inset shows a zoom-in on this figure section. The figure inset shows the observed heatwave in the two reference data sets and projections in the indicated section of the respective sub-plot. It facilitates readability of color and size of the reference values and comparison to each other and the neighboring projections. Note that the zoom-in causes the dots to overlap more in the inset than in the main sub-plot. The univariate kernel density estimates along the x- (Maximum Heatwave Area (%)) and y-axis (Heatwave Length (days)) are on the top and right side of the subplots, respectively, with vertical lines in dark blue (Berkeley Earth) and light blue (ERA5) indicating the observed event.

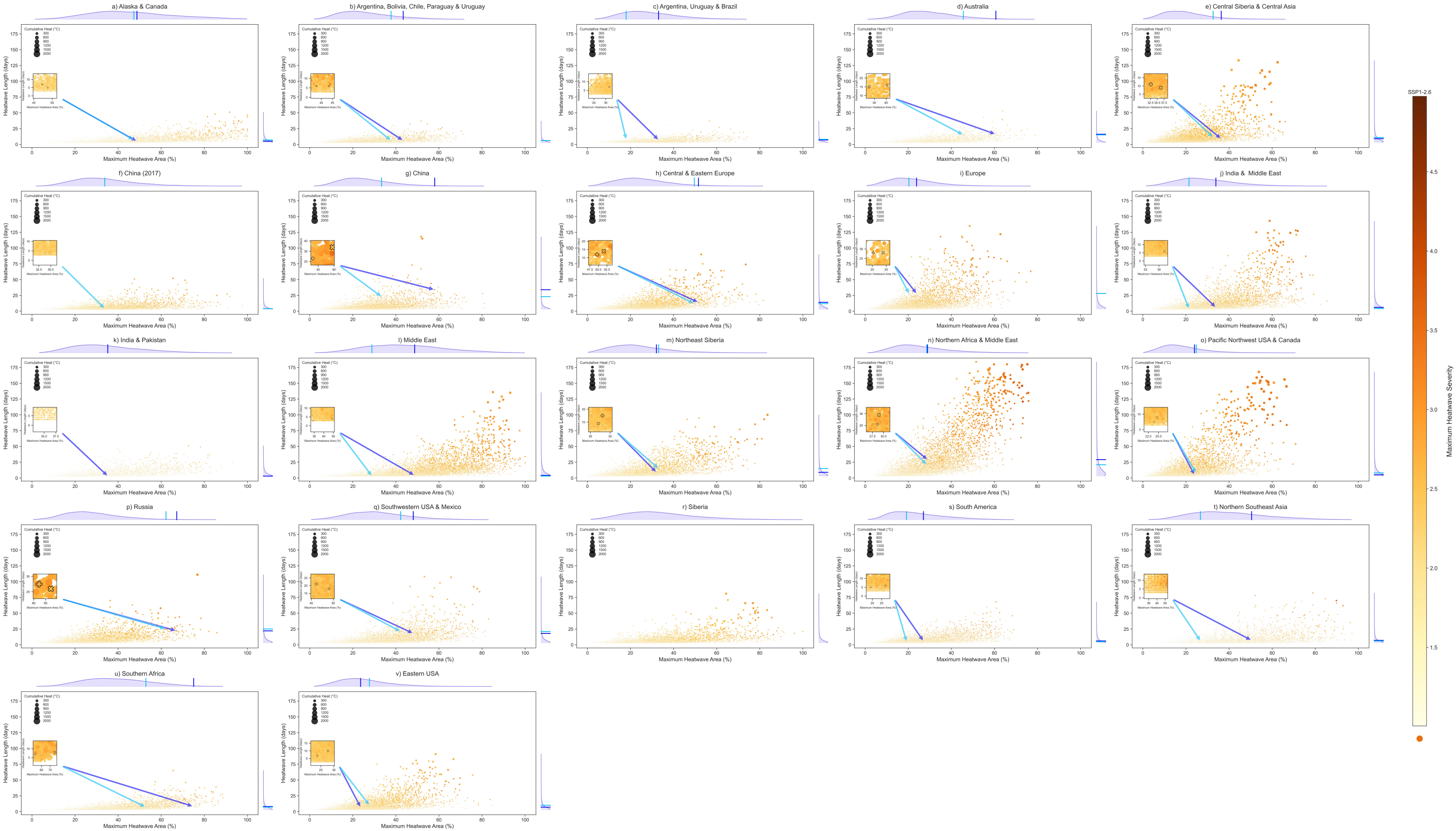

a) Alaska & Canada
b) Argentina, Bolivia, Chile, Paraguay & Uruguay
c) Argentina, Uruguay & Brazil
d) Australia
e) Central Siberia & Central Asia
f) China (2017)
g) China
h) Central & Eastern Europe
i) Europe
j) India & Middle East
k) India & Pakistan
l) Middle East
m) Northeast Siberia
n) Northern Africa & Middle East
o) Pacific Northwest USA & Canada
p) Russia
q) Southwestern USA & Mexico
r) Siberia
s) South America
t) Northern Southeast Asia
u) Southern Africa
v) Eastern USA
Cumulative Heat (°C)
300
600
900
1200
1500
2000
Heatwave Length (days)
Maximum Heatwave Area (%)
SSP1-2.6
Maximum Heatwave Severity

Supplementary Figure 89: Multi-HW-characteristic figure according to SSP2-4.5 CMIP6 multi-model ensemble (MME) (Tx95.0p) - Each panel shows regional projections according to SSP2-4.5 model runs in the CMIP6 multi-model ensemble (MME) of four combined heatwave characteristics for each projected heatwave in the ensemble between 2020 and 2100. Each dot represents an individual heatwave in the projections, characterized by Maximum Heatwave Area (%) (x-axis) as latitude-weighted percentage of grid boxes affected by extreme heat, Heatwave Length (days) (y-axis) in days, Maximum Heatwave Severity (color), and Cumulative Heat (°C) (dot size). The characteristics of the observed event according to the Berkeley Earth observational data (x symbol) and the ERA5 reanalysis data (+ symbol), respectively, are pointed to by an arrow and a figure inset shows a zoom-in on this figure section. The figure inset shows the observed heatwave in the two reference data sets and projections in the indicated section of the respective sub-plot. It facilitates readability of color and size of the reference values and comparison to each other and the neighboring projections. Note that the zoom-in causes the dots to overlap more in the inset than in the main sub-plot. The univariate kernel density estimates along the x- (Maximum Heatwave Area (%)) and y-axis (Heatwave Length (days)) are on the top and right side of the subplots, respectively, with vertical lines in dark blue (Berkeley Earth) and light blue (ERA5) indicating the observed event.

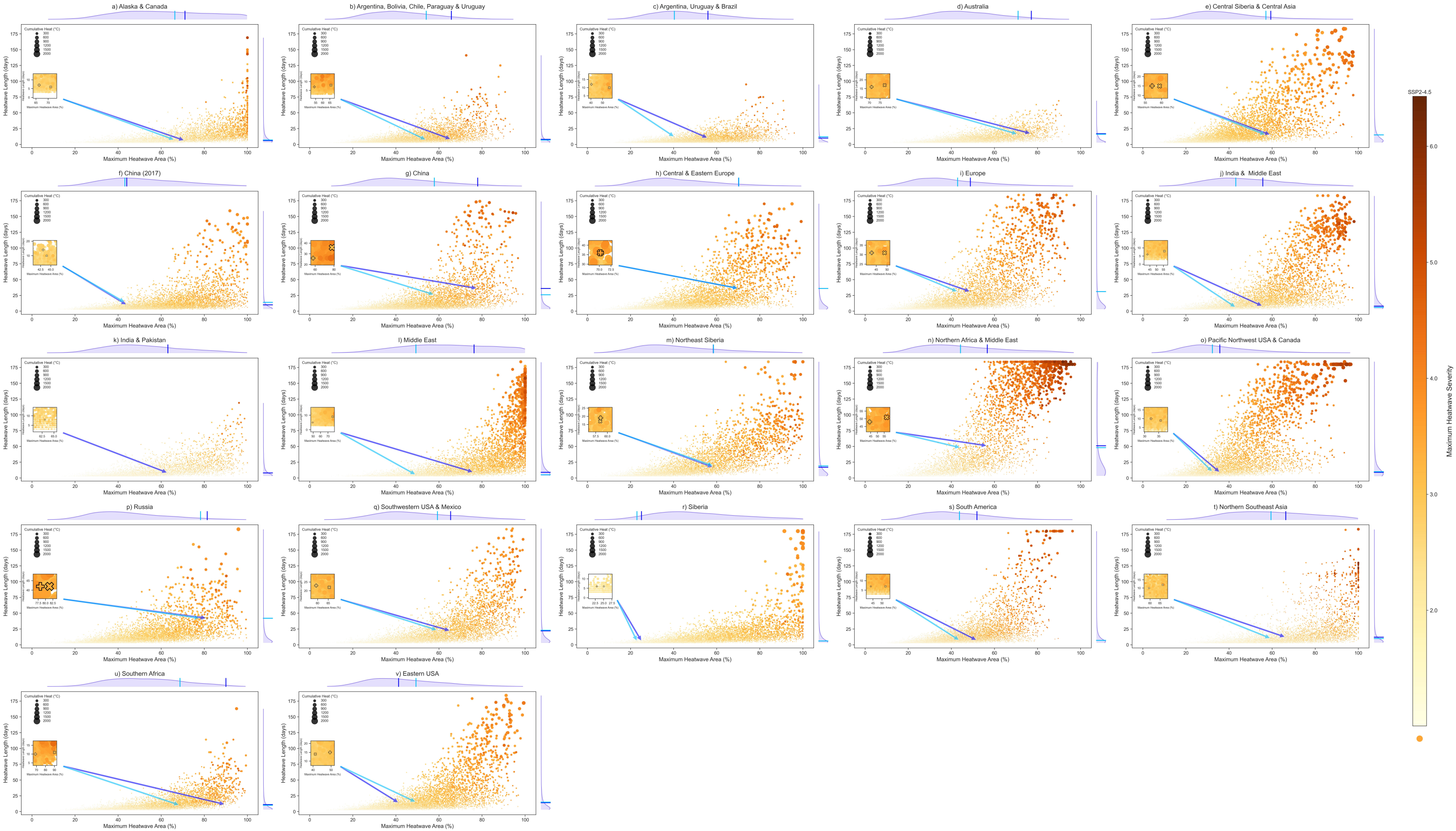

a) Alaska & Canada
b) Argentina, Bolivia, Chile, Paraguay & Uruguay
c) Argentina, Uruguay & Brazil
d) Australia
e) Central Siberia & Central Asia
f) China (2017)
g) China
h) Central & Eastern Europe
i) Europe
j) India & Middle East
k) India & Pakistan
l) Middle East
m) Northeast Siberia
n) Northern Africa & Middle East
o) Pacific Northwest USA & Canada
p) Russia
q) Southwestern USA & Mexico
r) Siberia
s) South America
t) Northern Southeast Asia
u) Southern Africa
v) Eastern USA
Heatwave Length (days)
Maximum Heatwave Area (%)
Cumulative Heat (°C)
300
600
900
1200
1500
2000
SSP2-4.5
Maximum Heatwave Severity
2.0
3.0
4.0
5.0
6.0

Supplementary Figure 90: Multi-HW-characteristic figure according to SSP2-4.5 CMIP6 multi-model ensemble (MME) (Tx97.5p) - Each panel shows regional projections according to SSP2-4.5 model runs in the CMIP6 multi-model ensemble (MME) of four combined heatwave characteristics for each projected heatwave in the ensemble between 2020 and 2100. Each dot represents an individual heatwave in the projections, characterized by Maximum Heatwave Area (%) (x-axis) as latitude-weighted percentage of grid boxes affected by extreme heat, Heatwave Length (days) (y-axis) in days, Maximum Heatwave Severity (color), and Cumulative Heat (°C) (dot size). The characteristics of the observed event according to the Berkeley Earth observational data (x symbol) and the ERA5 reanalysis data (+ symbol), respectively, are pointed to by an arrow and a figure inset shows a zoom-in on this figure section. The figure inset shows the observed heatwave in the two reference data sets and projections in the indicated section of the respective sub-plot. It facilitates readability of color and size of the reference values and comparison to each other and the neighboring projections. Note that the zoom-in causes the dots to overlap more in the inset than in the main sub-plot. The univariate kernel density estimates along the x- (Maximum Heatwave Area (%)) and y-axis (Heatwave Length (days)) are on the top and right side of the subplots, respectively, with vertical lines in dark blue (Berkeley Earth) and light blue (ERA5) indicating the observed event.

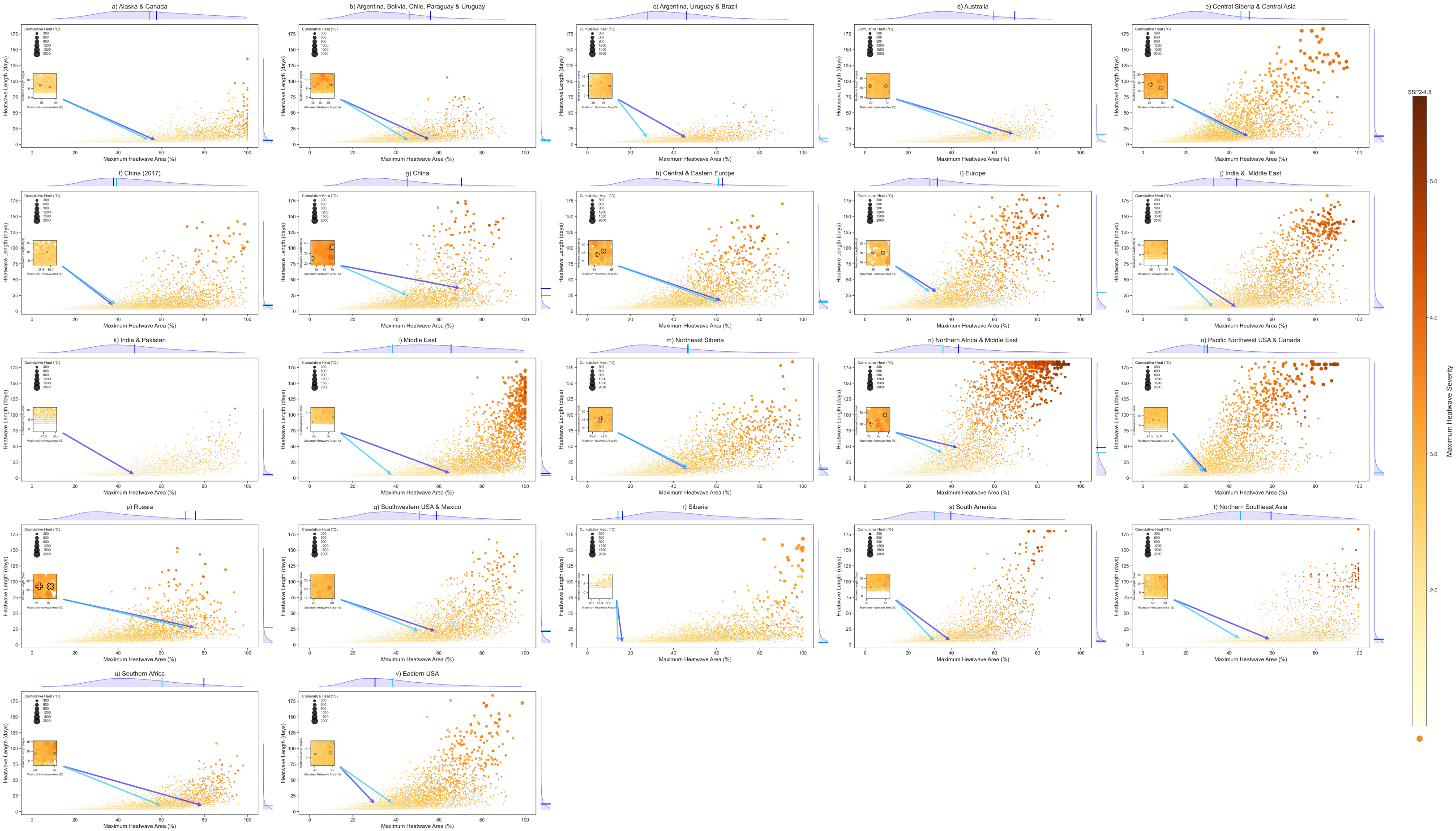

a) Alaska & Canada
b) Argentina, Bolivia, Chile, Paraguay & Uruguay
c) Argentina, Uruguay & Brazil
d) Australia
e) Central Siberia & Central Asia
f) China (2017)
g) China
h) Central & Eastern Europe
i) Europe
j) India & Middle East
k) India & Pakistan
l) Middle East
m) Northeast Siberia
n) Northern Africa & Middle East
o) Pacific Northwest USA & Canada
p) Russia
q) Southwestern USA & Mexico
r) Siberia
s) South America
t) Northern Southeast Asia
u) Southern Africa
v) Eastern USA
Cumulative Heat (°C)
300
600
900
1200
1500
2000
Heatwave Length (days)
Maximum Heatwave Area (%)
SSP2-4.5
Maximum Heatwave Severity

Supplementary Figure 91: Multi-HW-characteristic figure according to SSP2-4.5 CMIP6 multi-model ensemble (MME) (Tx99.0p) - Each panel shows regional projections according to SSP2-4.5 model runs in the CMIP6 multi-model ensemble (MME) of four combined heatwave characteristics for each projected heatwave in the ensemble between 2020 and 2100. Each dot represents an individual heatwave in the projections, characterized by Maximum Heatwave Area (%) (x-axis) as latitude-weighted percentage of grid boxes affected by extreme heat, Heatwave Length (days) (y-axis) in days, Maximum Heatwave Severity (color), and Cumulative Heat (°C) (dot size). The characteristics of the observed event according to the Berkeley Earth observational data (x symbol) and the ERA5 reanalysis data (+ symbol), respectively, are pointed to by an arrow and a figure inset shows a zoom-in on this figure section. The figure inset shows the observed heatwave in the two reference data sets and projections in the indicated section of the respective sub-plot. It facilitates readability of color and size of the reference values and comparison to each other and the neighboring projections. Note that the zoom-in causes the dots to overlap more in the inset than in the main sub-plot. The univariate kernel density estimates along the x- (Maximum Heatwave Area (%)) and y-axis (Heatwave Length (days)) are on the top and right side of the subplots, respectively, with vertical lines in dark blue (Berkeley Earth) and light blue (ERA5) indicating the observed event.

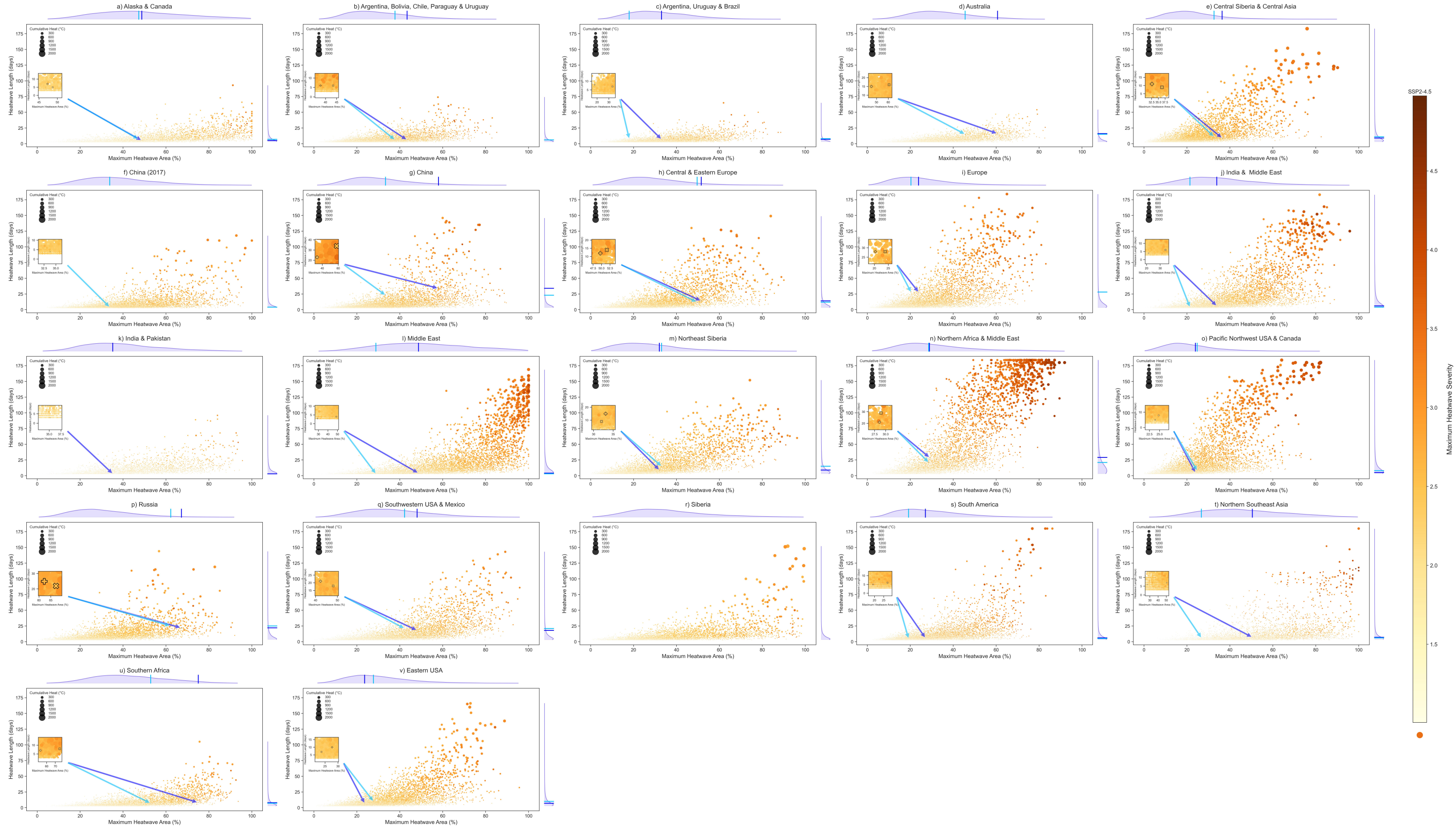
a) Alaska & Canada
b) Argentina, Bolivia, Chile, Paraguay & Uruguay
c) Argentina, Uruguay & Brazil
d) Australia
e) Central Siberia & Central Asia
f) China (2017)
g) China
h) Central & Eastern Europe
i) Europe
j) India & Middle East
k) India & Pakistan
l) Middle East
m) Northeast Siberia
n) Northern Africa & Middle East
o) Pacific Northwest USA & Canada
p) Russia
q) Southwestern USA & Mexico
r) Siberia
s) South America
t) Northern Southeast Asia
u) Southern Africa
v) Eastern USA
Cumulative Heat (°C)
300
600
900
1200
1500
2000
Heatwave Length (days)
Maximum Heatwave Area (%)
SSP2-4.5
Maximum Heatwave Severity

Supplementary Figure 92: Multi-HW-characteristic figure according to SSP3-7.0 CMIP6 multi-model ensemble (MME) (Tx95.0p) - Each panel shows regional projections according to SSP3-7.0 model runs in the CMIP6 multi-model ensemble (MME) of four combined heatwave characteristics for each projected heatwave in the ensemble between 2020 and 2100. Each dot represents an individual heatwave in the projections, characterized by Maximum Heatwave Area (%) (x-axis) as latitude-weighted percentage of grid boxes affected by extreme heat, Heatwave Length (days) (y-axis) in days, Maximum Heatwave Severity (color), and Cumulative Heat (°C) (dot size). The characteristics of the observed event according to the Berkeley Earth observational data (x symbol) and the ERA5 reanalysis data (+ symbol), respectively, are pointed to by an arrow and a figure inset shows a zoom-in on this figure section. The figure inset shows the observed heatwave in the two reference data sets and projections in the indicated section of the respective sub-plot. It facilitates readability of color and size of the reference values and comparison to each other and the neighboring projections. Note that the zoom-in causes the dots to overlap more in the inset than in the main sub-plot. The univariate kernel density estimates along the x- (Maximum Heatwave Area (%)) and y-axis (Heatwave Length (days)) are on the top and right side of the subplots, respectively, with vertical lines in dark blue (Berkeley Earth) and light blue (ERA5) indicating the observed event.

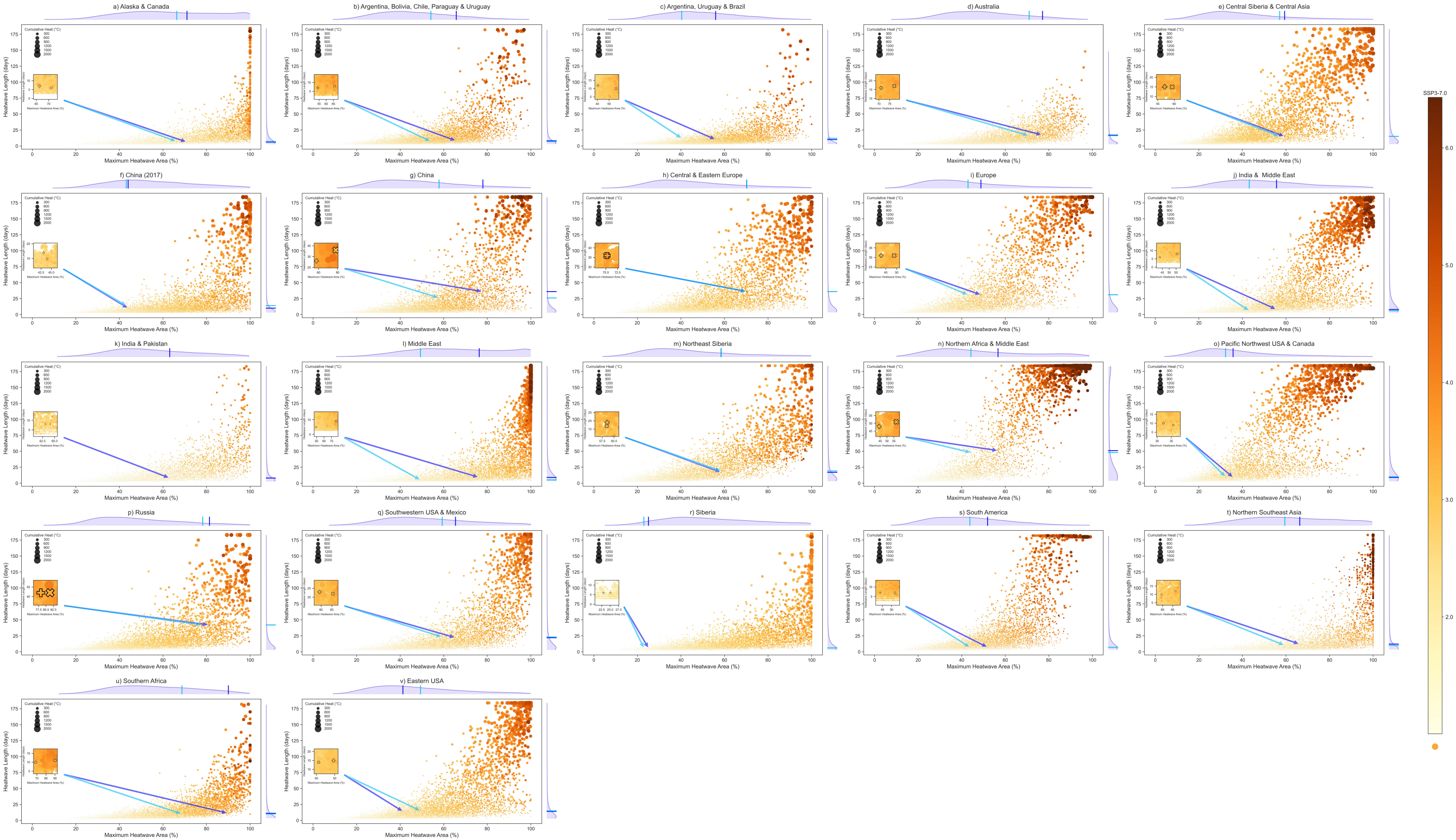
a) Alaska & Canada
b) Argentina, Bolivia, Chile, Paraguay & Uruguay
c) Argentina, Uruguay & Brazil
d) Australia
e) Central Siberia & Central Asia
f) China (2017)
g) China
h) Central & Eastern Europe
i) Europe
j) India & Middle East
k) India & Pakistan
l) Middle East
m) Northeast Siberia
n) Northern Africa & Middle East
o) Pacific Northwest USA & Canada
p) Russia
q) Southwestern USA & Mexico
r) Siberia
s) South America
t) Northern Southeast Asia
u) Southern Africa
v) Eastern USA
Cumulative Heat (°C)
300
600
900
1200
1500
2000
Heatwave Length (days)
Maximum Heatwave Area (%)
SSP3-7.0
Maximum Heatwave Severity
2.0
3.0
4.0
5.0
6.0

Supplementary Figure 93: Multi-HW-characteristic figure according to SSP3-7.0 CMIP6 multi-model ensemble (MME) (Tx97.5p) - Each panel shows regional projections according to SSP3-7.0 model runs in the CMIP6 multi-model ensemble (MME) of four combined heatwave characteristics for each projected heatwave in the ensemble between 2020 and 2100. Each dot represents an individual heatwave in the projections, characterized by Maximum Heatwave Area (%) (x-axis) as latitude-weighted percentage of grid boxes affected by extreme heat, Heatwave Length (days) (y-axis) in days, Maximum Heatwave Severity (color), and Cumulative Heat (°C) (dot size). The characteristics of the observed event according to the Berkeley Earth observational data (x symbol) and the ERA5 reanalysis data (+ symbol), respectively, are pointed to by an arrow and a figure inset shows a zoom-in on this figure section. The figure inset shows the observed heatwave in the two reference data sets and projections in the indicated section of the respective sub-plot. It facilitates readability of color and size of the reference values and comparison to each other and the neighboring projections. Note that the zoom-in causes the dots to overlap more in the inset than in the main sub-plot. The univariate kernel density estimates along the x- (Maximum Heatwave Area (%)) and y-axis (Heatwave Length (days)) are on the top and right side of the subplots, respectively, with vertical lines in dark blue (Berkeley Earth) and light blue (ERA5) indicating the observed event.

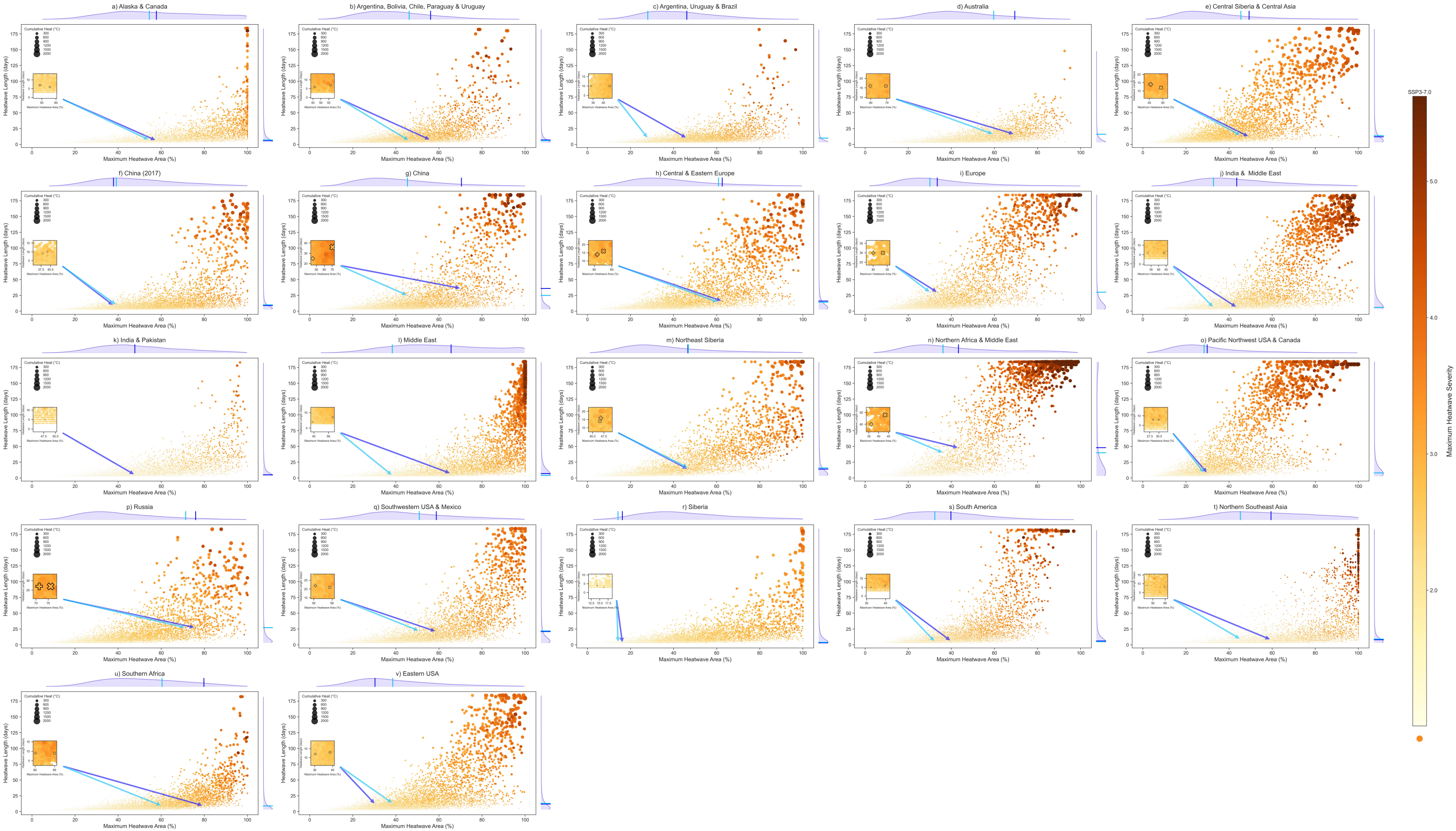
a) Alaska & Canada
b) Argentina, Bolivia, Chile, Paraguay & Uruguay
c) Argentina, Uruguay & Brazil
d) Australia
e) Central Siberia & Central Asia
f) China (2017)
g) China
h) Central & Eastern Europe
i) Europe
j) India & Middle East
k) India & Pakistan
l) Middle East
m) Northeast Siberia
n) Northern Africa & Middle East
o) Pacific Northwest USA & Canada
p) Russia
q) Southwestern USA & Mexico
r) Siberia
s) South America
t) Northern Southeast Asia
u) Southern Africa
v) Eastern USA
Cumulative Heat (°C)
300
600
900
1200
1500
2000
Heatwave Length (days)
Maximum Heatwave Area (%)
SSP3-7.0
Maximum Heatwave Severity

Supplementary Figure 94: Multi-HW-characteristic figure according to SSP3-7.0 CMIP6 multi-model ensemble (MME) (Tx99.0p) - Each panel shows regional projections according to SSP3-7.0 model runs in the CMIP6 multi-model ensemble (MME) of four combined heatwave characteristics for each projected heatwave in the ensemble between 2020 and 2100. Each dot represents an individual heatwave in the projections, characterized by Maximum Heatwave Area (%) (x-axis) as latitude-weighted percentage of grid boxes affected by extreme heat, Heatwave Length (days) (y-axis) in days, Maximum Heatwave Severity (color), and Cumulative Heat (°C) (dot size). The characteristics of the observed event according to the Berkeley Earth observational data (x symbol) and the ERA5 reanalysis data (+ symbol), respectively, are pointed to by an arrow and a figure inset shows a zoom-in on this figure section. The figure inset shows the observed heatwave in the two reference data sets and projections in the indicated section of the respective sub-plot. It facilitates readability of color and size of the reference values and comparison to each other and the neighboring projections. Note that the zoom-in causes the dots to overlap more in the inset than in the main sub-plot. The univariate kernel density estimates along the x- (Maximum Heatwave Area (%)) and y-axis (Heatwave Length (days)) are on the top and right side of the subplots, respectively, with vertical lines in dark blue (Berkeley Earth) and light blue (ERA5) indicating the observed event.

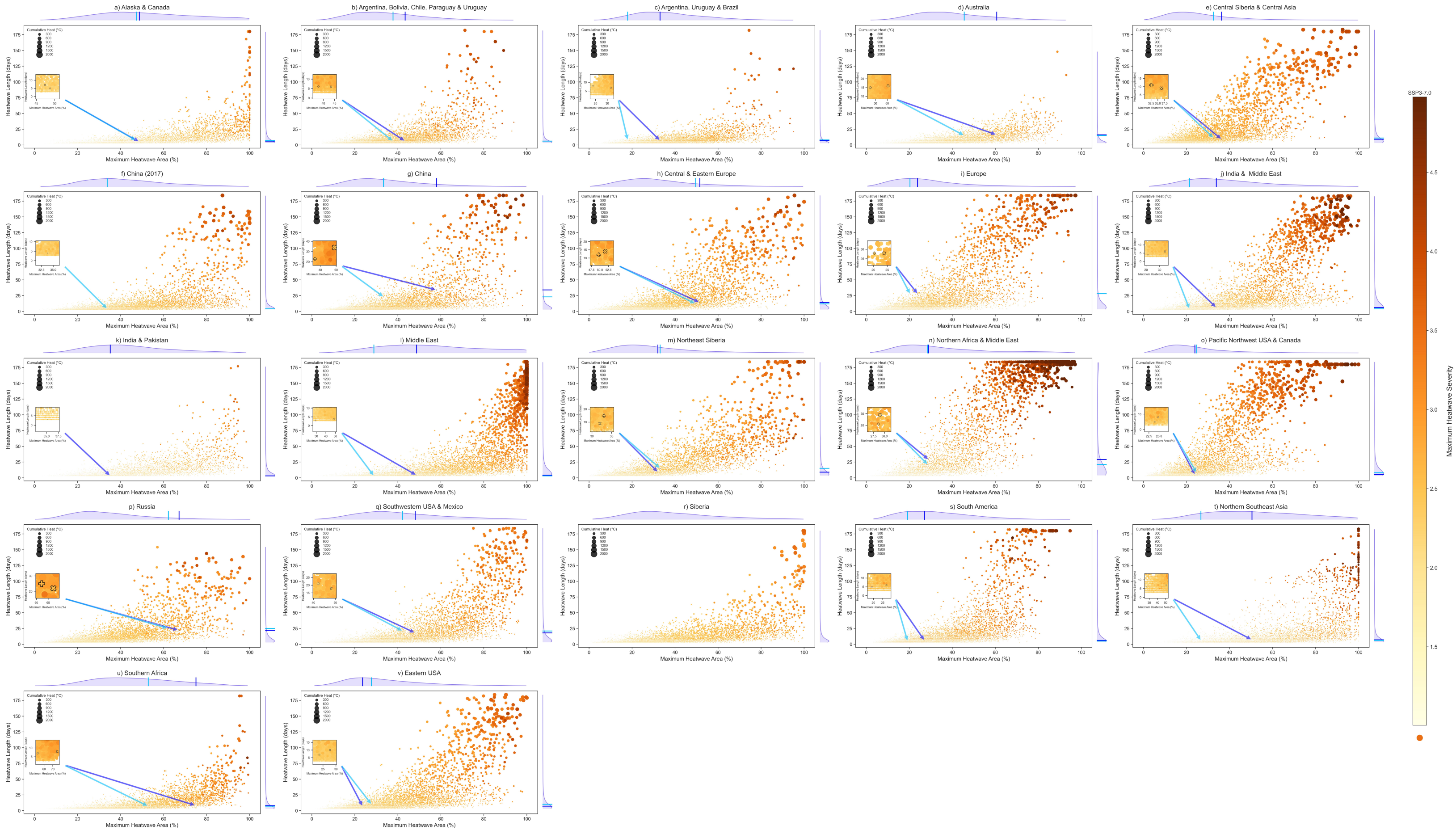
a) Alaska & Canada
b) Argentina, Bolivia, Chile, Paraguay & Uruguay
c) Argentina, Uruguay & Brazil
d) Australia
e) Central Siberia & Central Asia
f) China (2017)
g) China
h) Central & Eastern Europe
i) Europe
j) India & Middle East
k) India & Pakistan
l) Middle East
m) Northeast Siberia
n) Northern Africa & Middle East
o) Pacific Northwest USA & Canada
p) Russia
q) Southwestern USA & Mexico
r) Siberia
s) South America
t) Northern Southeast Asia
u) Southern Africa
v) Eastern USA
Cumulative Heat (°C)
300
600
900
1200
1500
2000
Heatwave Length (days)
Maximum Heatwave Area (%)
SSP3-7.0
Maximum Heatwave Severity
1.5
2.0
2.5
3.0
3.5
4.0
4.5

Supplementary Figure 95: Multi-HW-characteristic figure according to SSP5-8.5 CMIP6 multi-model ensemble (MME) (Tx95.0p) - Each panel shows regional projections according to SSP5-8.5 model runs in the CMIP6 multi-model ensemble (MME) of four combined heatwave characteristics for each projected heatwave in the ensemble between 2020 and 2100. Each dot represents an individual heatwave in the projections, characterized by Maximum Heatwave Area (%) (x-axis) as latitude-weighted percentage of grid boxes affected by extreme heat, Heatwave Length (days) (y-axis) in days, Maximum Heatwave Severity (color), and Cumulative Heat (°C) (dot size). The characteristics of the observed event according to the Berkeley Earth observational data (x symbol) and the ERA5 reanalysis data (+ symbol), respectively, are pointed to by an arrow and a figure inset shows a zoom-in on this figure section. The figure inset shows the observed heatwave in the two reference data sets and projections in the indicated section of the respective sub-plot. It facilitates readability of color and size of the reference values and comparison to each other and the neighboring projections. Note that the zoom-in causes the dots to overlap more in the inset than in the main sub-plot. The univariate kernel density estimates along the x- (Maximum Heatwave Area (%)) and y-axis (Heatwave Length (days)) are on the top and right side of the subplots, respectively, with vertical lines in dark blue (Berkeley Earth) and light blue (ERA5) indicating the observed event.

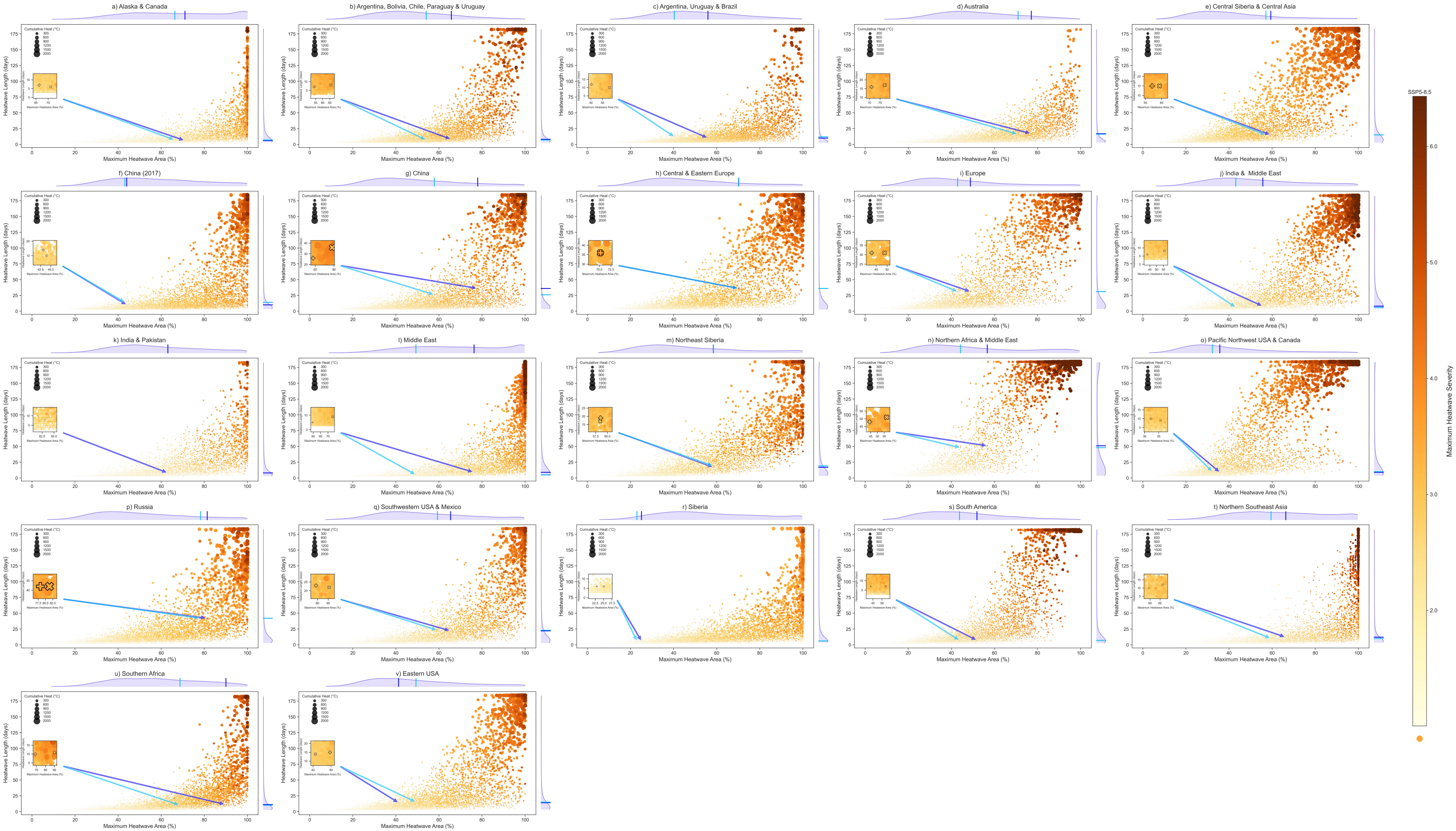

a) Alaska & Canada
b) Argentina, Bolivia, Chile, Paraguay & Uruguay
c) Argentina, Uruguay & Brazil
d) Australia
e) Central Siberia & Central Asia
f) China (2017)
g) China
h) Central & Eastern Europe
i) Europe
j) India & Middle East
k) India & Pakistan
l) Middle East
m) Northeast Siberia
n) Northern Africa & Middle East
o) Pacific Northwest USA & Canada
p) Russia
q) Southwestern USA & Mexico
r) Siberia
s) South America
t) Northern Southeast Asia
u) Southern Africa
v) Eastern USA
Cumulative Heat (°C)
300
600
900
1200
1500
2000
Heatwave Length (days)
Maximum Heatwave Area (%)
SSP5-8.5
Maximum Heatwave Severity

Supplementary Figure 96: Multi-HW-characteristic figure according to SSP5-8.5 CMIP6 multi-model ensemble (MME) (Tx97.5p) - Each panel shows regional projections according to SSP5-8.5 model runs in the CMIP6 multi-model ensemble (MME) of four combined heatwave characteristics for each projected heatwave in the ensemble between 2020 and 2100. Each dot represents an individual heatwave in the projections, characterized by Maximum Heatwave Area (%) (x-axis) as latitude-weighted percentage of grid boxes affected by extreme heat, Heatwave Length (days) (y-axis) in days, Maximum Heatwave Severity (color), and Cumulative Heat (°C) (dot size). The characteristics of the observed event according to the Berkeley Earth observational data (x symbol) and the ERA5 reanalysis data (+ symbol), respectively, are pointed to by an arrow and a figure inset shows a zoom-in on this figure section. The figure inset shows the observed heatwave in the two reference data sets and projections in the indicated section of the respective sub-plot. It facilitates readability of color and size of the reference values and comparison to each other and the neighboring projections. Note that the zoom-in causes the dots to overlap more in the inset than in the main sub-plot. The univariate kernel density estimates along the x- (Maximum Heatwave Area (%)) and y-axis (Heatwave Length (days)) are on the top and right side of the subplots, respectively, with vertical lines in dark blue (Berkeley Earth) and light blue (ERA5) indicating the observed event.

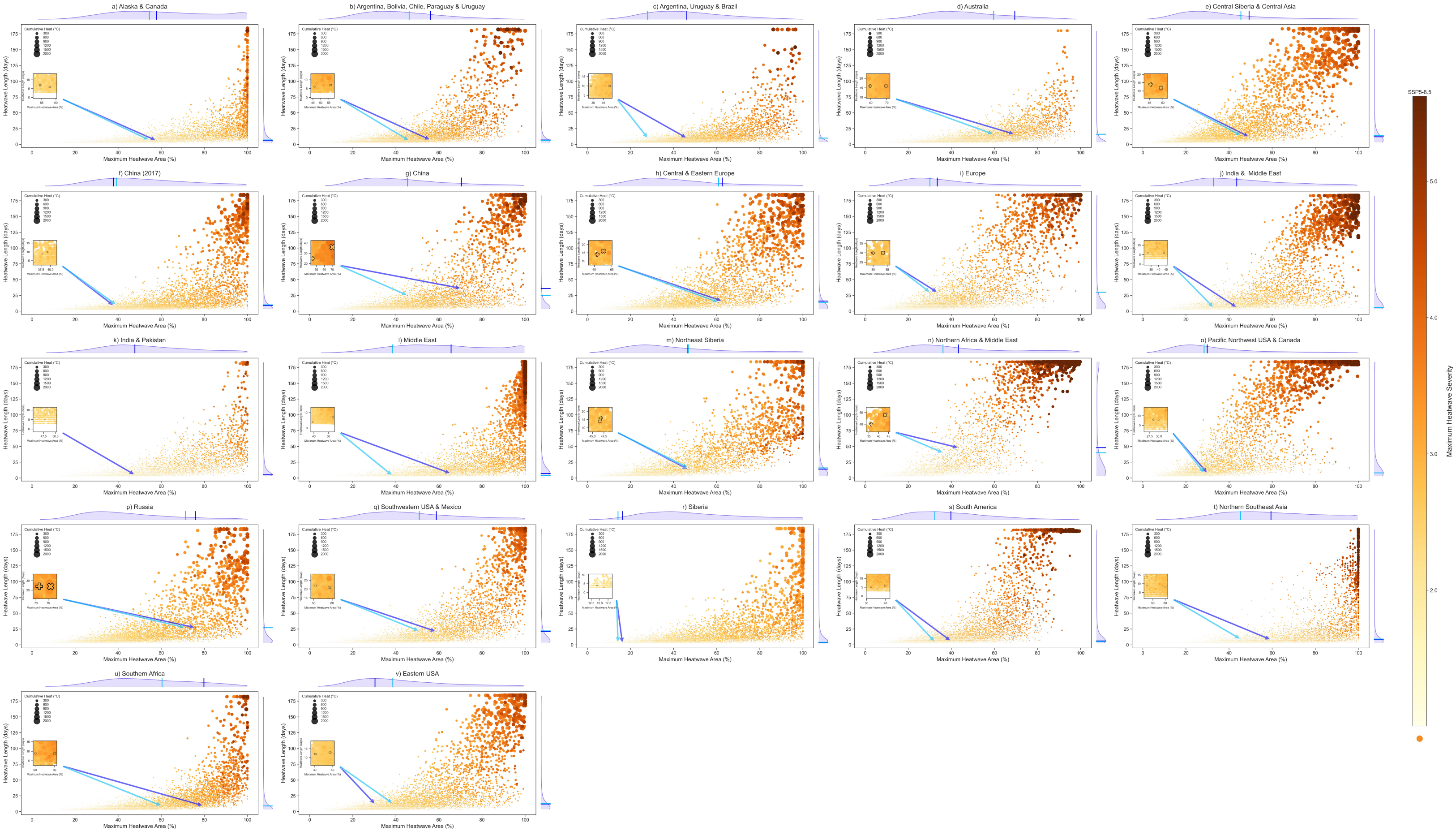

a) Alaska & Canada
b) Argentina, Bolivia, Chile, Paraguay & Uruguay
c) Argentina, Uruguay & Brazil
d) Australia
e) Central Siberia & Central Asia
f) China (2017)
g) China
h) Central & Eastern Europe
i) Europe
j) India & Middle East
k) India & Pakistan
l) Middle East
m) Northeast Siberia
n) Northern Africa & Middle East
o) Pacific Northwest USA & Canada
p) Russia
q) Southwestern USA & Mexico
r) Siberia
s) South America
t) Northern Southeast Asia
u) Southern Africa
v) Eastern USA
Heatwave Length (days)
Maximum Heatwave Area (%)
Cumulative Heat (°C)
300
600
900
1200
1500
2000
SSP5-8.5
Maximum Heatwave Severity
2.0
3.0
4.0
5.0

Supplementary Figure 97: Multi-HW-characteristic figure according to SSP5-8.5 CMIP6 multi-model ensemble (MME) (Tx99.0p) - Each panel shows regional projections according to SSP5-8.5 model runs in the CMIP6 multi-model ensemble (MME) of four combined heatwave characteristics for each projected heatwave in the ensemble between 2020 and 2100. Each dot represents an individual heatwave in the projections, characterized by Maximum Heatwave Area (%) (x-axis) as latitude-weighted percentage of grid boxes affected by extreme heat, Heatwave Length (days) (y-axis) in days, Maximum Heatwave Severity (color), and Cumulative Heat (°C) (dot size). The characteristics of the observed event according to the Berkeley Earth observational data (x symbol) and the ERA5 reanalysis data (+ symbol), respectively, are pointed to by an arrow and a figure inset shows a zoom-in on this figure section. The figure inset shows the observed heatwave in the two reference data sets and projections in the indicated section of the respective sub-plot. It facilitates readability of color and size of the reference values and comparison to each other and the neighboring projections. Note that the zoom-in causes the dots to overlap more in the inset than in the main sub-plot. The univariate kernel density estimates along the x- (Maximum Heatwave Area (%)) and y-axis (Heatwave Length (days)) are on the top and right side of the subplots, respectively, with vertical lines in dark blue (Berkeley Earth) and light blue (ERA5) indicating the observed event.

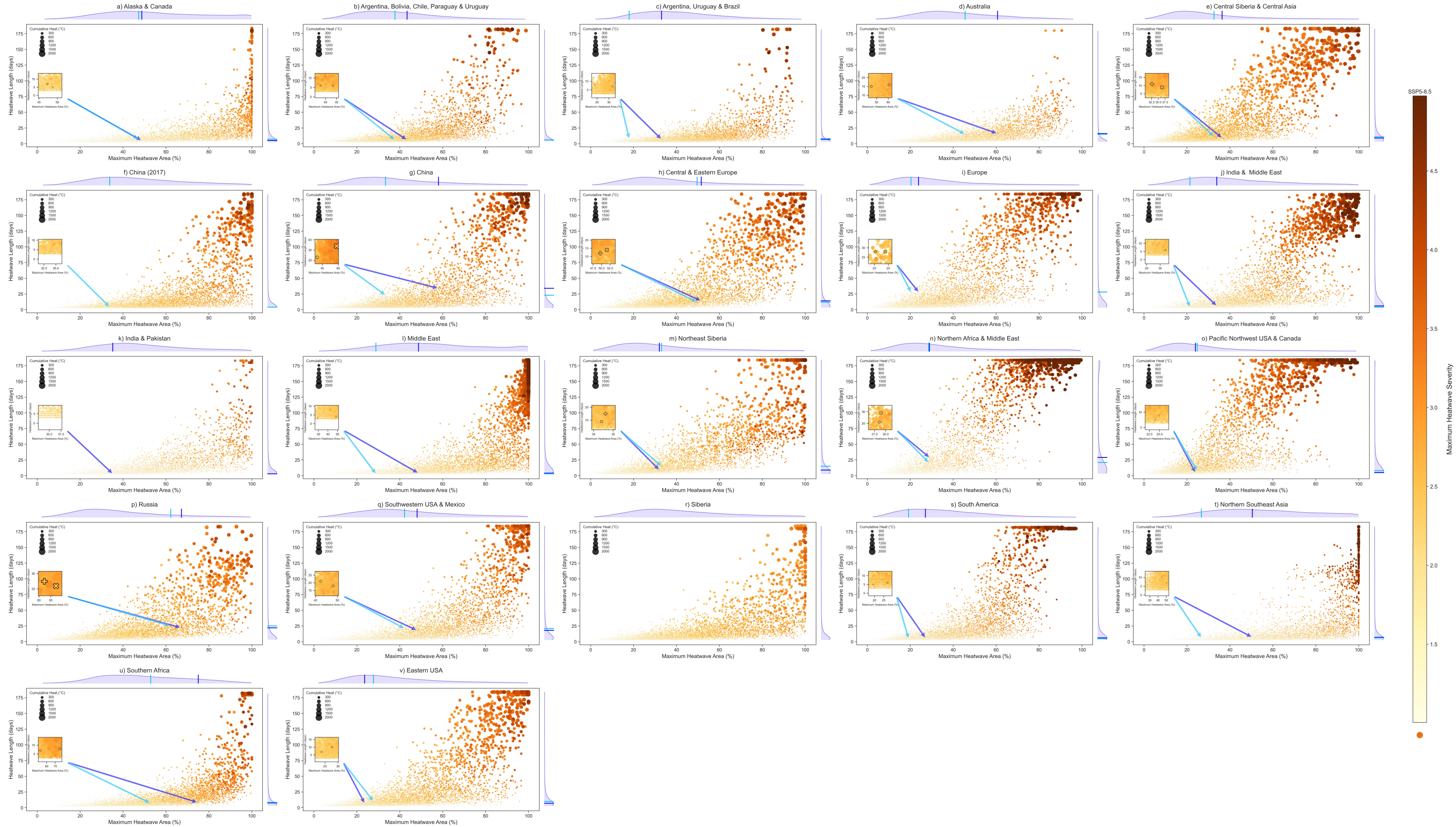
a) Alaska & Canada
b) Argentina, Bolivia, Chile, Paraguay & Uruguay
c) Argentina, Uruguay & Brazil
d) Australia
e) Central Siberia & Central Asia
f) China (2017)
g) China
h) Central & Eastern Europe
i) Europe
j) India & Middle East
k) India & Pakistan
l) Middle East
m) Northeast Siberia
n) Northern Africa & Middle East
o) Pacific Northwest USA & Canada
p) Russia
q) Southwestern USA & Mexico
r) Siberia
s) South America
t) Northern Southeast Asia
u) Southern Africa
v) Eastern USA
Heatwave Length (days)
Maximum Heatwave Area (%)
Cumulative Heat (°C)
300
600
900
1200
1500
2000
SSP5-8.5
Maximum Heatwave Severity

Supplementary Figure 98: Multi-HW-characteristic figure according to Historical CMIP6 multi-model ensemble (MME) (Tx95.0p) - Each panel shows regional projections according to Historical model runs in the CMIP6 multi-model ensemble (MME) of four combined heatwave characteristics for each projected heatwave in the ensemble between 2020 and 2100. Each dot represents an individual heatwave in the projections, characterized by Median Heatwave Area (%) (x-axis) as latitude-weighted percentage of grid boxes affected by extreme heat, Heatwave Length (days) (y-axis) in days, Median Heatwave Severity (color), and Cumulative Heat (°C) (dot size). The characteristics of the observed event according to the Berkeley Earth observational data (x symbol) and the ERA5 reanalysis data (+ symbol), respectively, are pointed to by an arrow and a figure inset shows a zoom-in on this figure section. The figure inset shows the observed heatwave in the two reference data sets and projections in the indicated section of the respective sub-plot. It facilitates readability of color and size of the reference values and comparison to each other and the neighboring projections. Note that the zoom-in causes the dots to overlap more in the inset than in the main sub-plot. The univariate kernel density estimates along the x- (Median Heatwave Area (%)) and y-axis (Heatwave Length (days)) are on the top and right side of the subplots, respectively, with vertical lines in dark blue (Berkeley Earth) and light blue (ERA5) indicating the observed event.

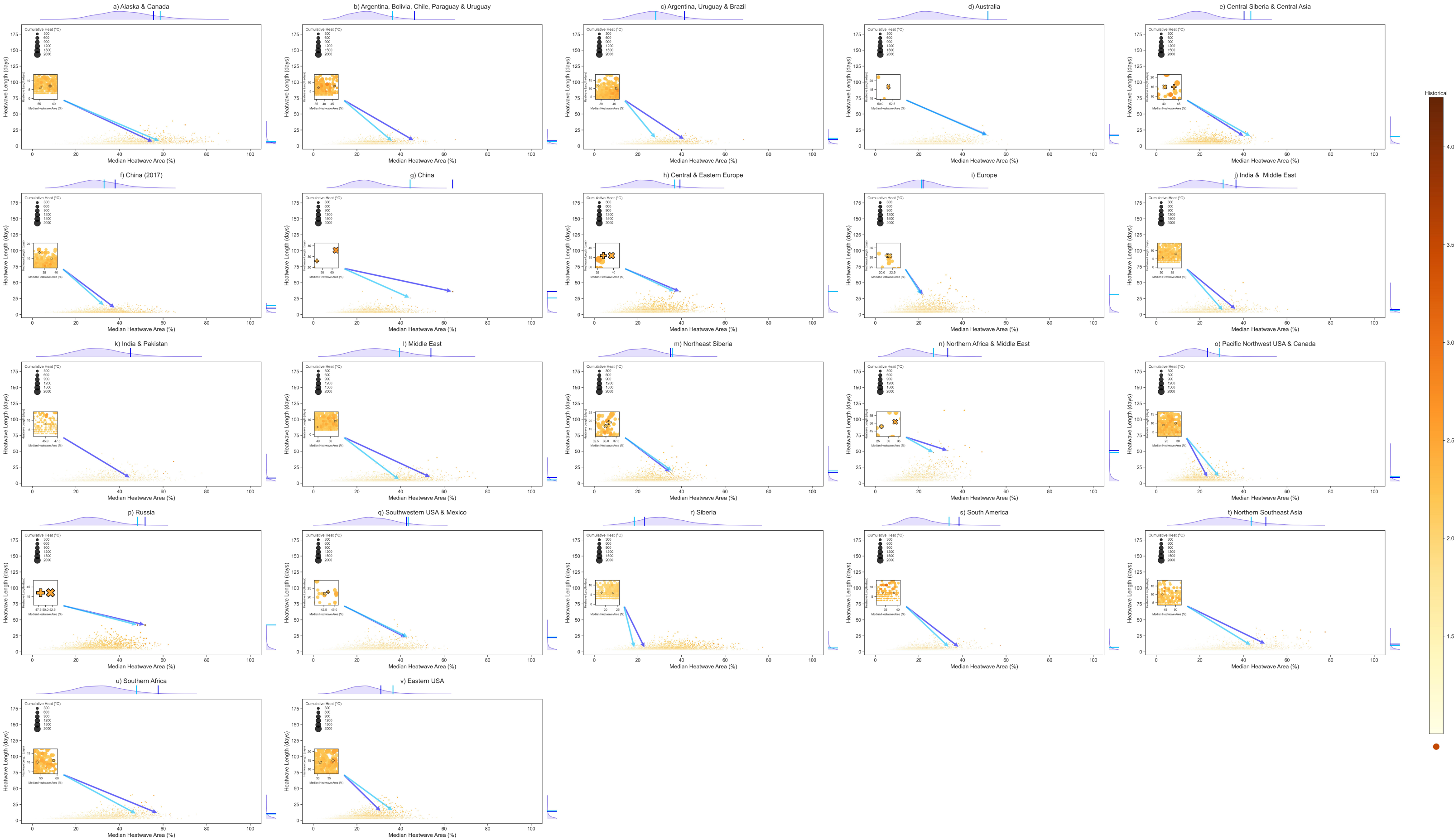
a) Alaska & Canada
b) Argentina, Bolivia, Chile, Paraguay & Uruguay
c) Argentina, Uruguay & Brazil
d) Australia
e) Central Siberia & Central Asia
f) China (2017)
g) China
h) Central & Eastern Europe
i) Europe
j) India & Middle East
k) India & Pakistan
l) Middle East
m) Northeast Siberia
n) Northern Africa & Middle East
o) Pacific Northwest USA & Canada
p) Russia
q) Southwestern USA & Mexico
r) Siberia
s) South America
t) Northern Southeast Asia
u) Southern Africa
v) Eastern USA
Heatwave Length (days)
Median Heatwave Area (%)
Cumulative Heat (°C)
300
600
900
1200
1500
2000
Historical
Median Heatwave Severity
1.5
2.0
2.5
3.0
3.5
4.0

Supplementary Figure 99: Multi-HW-characteristic figure according to Historical CMIP6 multi-model ensemble (MME) (Tx97.5p) - Each panel shows regional projections according to Historical model runs in the CMIP6 multi-model ensemble (MME) of four combined heatwave characteristics for each projected heatwave in the ensemble between 2020 and 2100. Each dot represents an individual heatwave in the projections, characterized by Median Heatwave Area (%) (x-axis) as latitude-weighted percentage of grid boxes affected by extreme heat, Heatwave Length (days) (y-axis) in days, Median Heatwave Severity (color), and Cumulative Heat (°C) (dot size). The characteristics of the observed event according to the Berkeley Earth observational data (x symbol) and the ERA5 reanalysis data (+ symbol), respectively, are pointed to by an arrow and a figure inset shows a zoom-in on this figure section. The figure inset shows the observed heatwave in the two reference data sets and projections in the indicated section of the respective sub-plot. It facilitates readability of color and size of the reference values and comparison to each other and the neighboring projections. Note that the zoom-in causes the dots to overlap more in the inset than in the main sub-plot. The univariate kernel density estimates along the x- (Median Heatwave Area (%)) and y-axis (Heatwave Length (days)) are on the top and right side of the subplots, respectively, with vertical lines in dark blue (Berkeley Earth) and light blue (ERA5) indicating the observed event.

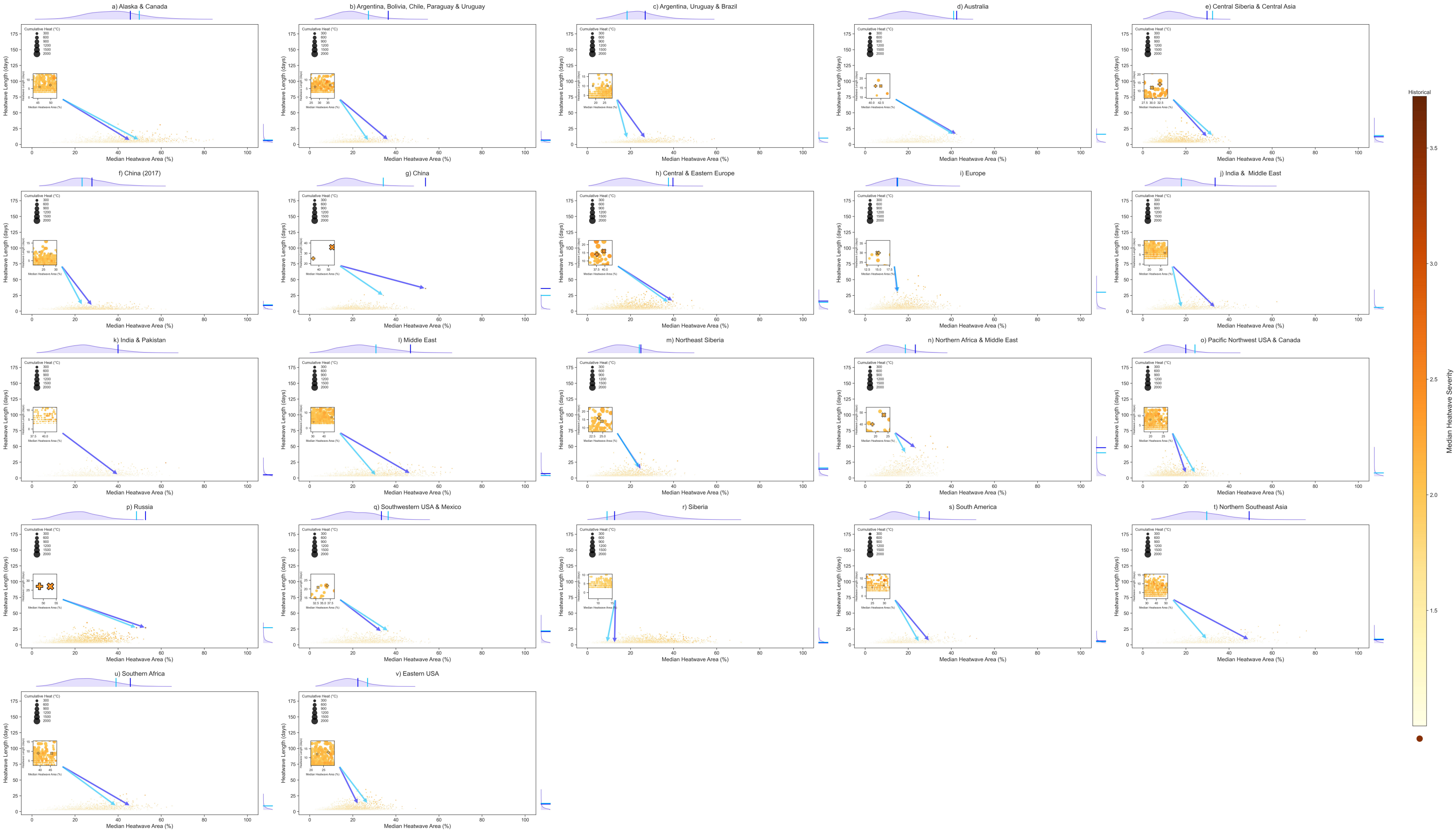

a) Alaska & Canada
b) Argentina, Bolivia, Chile, Paraguay & Uruguay
c) Argentina, Uruguay & Brazil
d) Australia
e) Central Siberia & Central Asia
f) China (2017)
g) China
h) Central & Eastern Europe
i) Europe
j) India & Middle East
k) India & Pakistan
l) Middle East
m) Northeast Siberia
n) Northern Africa & Middle East
o) Pacific Northwest USA & Canada
p) Russia
q) Southwestern USA & Mexico
r) Siberia
s) South America
t) Northern Southeast Asia
u) Southern Africa
v) Eastern USA
Heatwave Length (days)
Median Heatwave Area (%)
Cumulative Heat (°C)
300
600
900
1200
1500
2000
Historical
Median Heatwave Severity

Supplementary Figure 100: Multi-HW-characteristic figure according to Historical CMIP6 multi-model ensemble (MME) (Tx99.0p) - Each panel shows regional projections according to Historical model runs in the CMIP6 multi-model ensemble (MME) of four combined heatwave characteristics for each projected heatwave in the ensemble between 2020 and 2100. Each dot represents an individual heatwave in the projections, characterized by Median Heatwave Area (%) (x-axis) as latitude-weighted percentage of grid boxes affected by extreme heat, Heatwave Length (days) (y-axis) in days, Median Heatwave Severity (color), and Cumulative Heat (°C) (dot size). The characteristics of the observed event according to the Berkeley Earth observational data (x symbol) and the ERA5 reanalysis data (+ symbol), respectively, are pointed to by an arrow and a figure inset shows a zoom-in on this figure section. The figure inset shows the observed heatwave in the two reference data sets and projections in the indicated section of the respective sub-plot. It facilitates readability of color and size of the reference values and comparison to each other and the neighboring projections. Note that the zoom-in causes the dots to overlap more in the inset than in the main sub-plot. The univariate kernel density estimates along the x- (Median Heatwave Area (%)) and y-axis (Heatwave Length (days)) are on the top and right side of the subplots, respectively, with vertical lines in dark blue (Berkeley Earth) and light blue (ERA5) indicating the observed event.

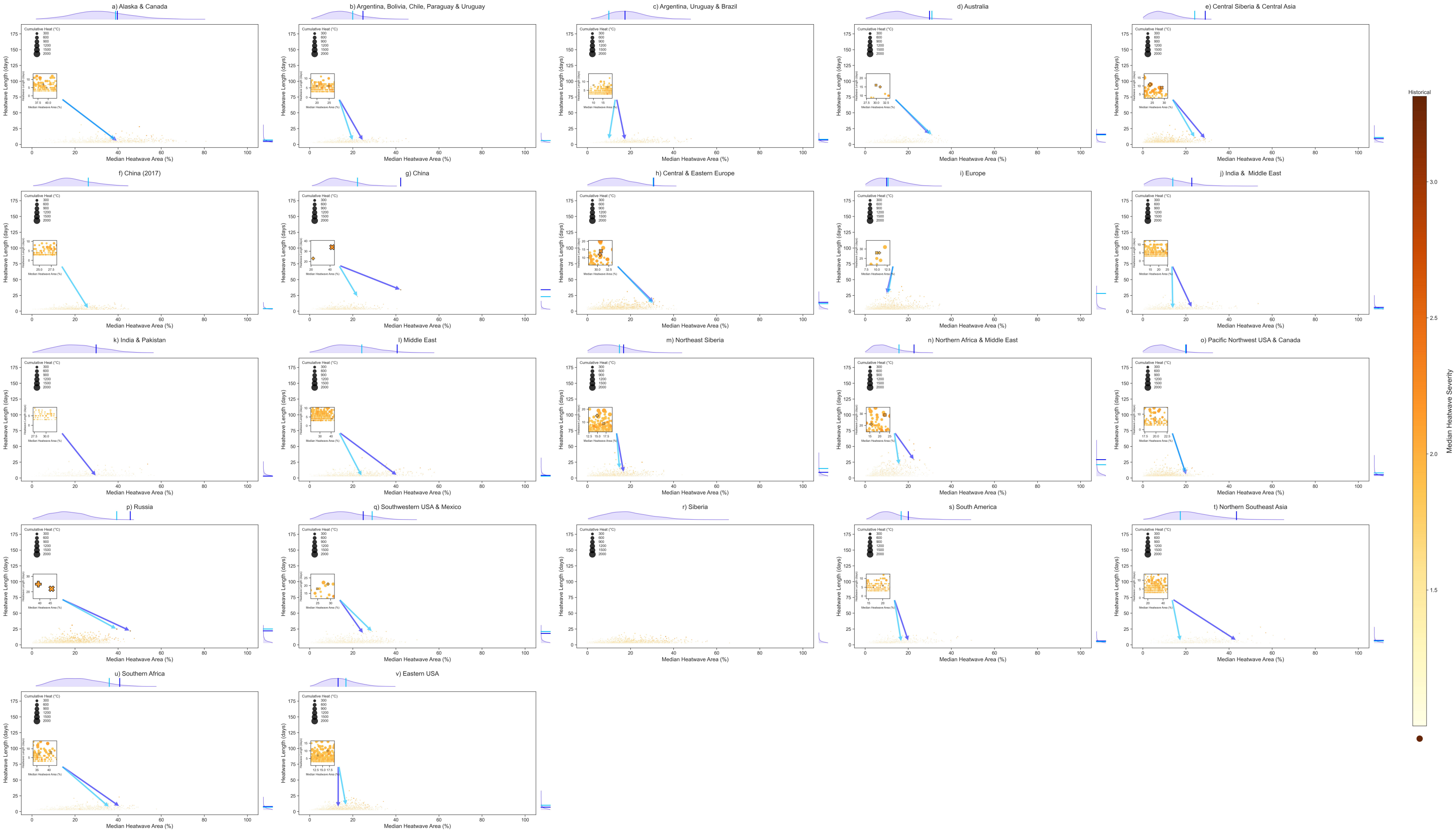
a) Alaska & Canada
b) Argentina, Bolivia, Chile, Paraguay & Uruguay
c) Argentina, Uruguay & Brazil
d) Australia
e) Central Siberia & Central Asia
f) China (2017)
g) China
h) Central & Eastern Europe
i) Europe
j) India & Middle East
k) India & Pakistan
l) Middle East
m) Northeast Siberia
n) Northern Africa & Middle East
o) Pacific Northwest USA & Canada
p) Russia
q) Southwestern USA & Mexico
r) Siberia
s) South America
t) Northern Southeast Asia
u) Southern Africa
v) Eastern USA
Cumulative Heat (°C)
300
600
900
1200
1500
2000
Heatwave Length (days)
Median Heatwave Area (%)
Historical
Median Heatwave Severity

Supplementary Figure 101: Multi-HW-characteristic figure according to SSP1-2.6 CMIP6 multi-model ensemble (MME) (Tx95.0p) - Each panel shows regional projections according to SSP1-2.6 model runs in the CMIP6 multi-model ensemble (MME) of four combined heatwave characteristics for each projected heatwave in the ensemble between 2020 and 2100. Each dot represents an individual heatwave in the projections, characterized by Median Heatwave Area (%) (x-axis) as latitude-weighted percentage of grid boxes affected by extreme heat, Heatwave Length (days) (y-axis) in days, Median Heatwave Severity (color), and Cumulative Heat (°C) (dot size). The characteristics of the observed event according to the Berkeley Earth observational data (x symbol) and the ERA5 reanalysis data (+ symbol), respectively, are pointed to by an arrow and a figure inset shows a zoom-in on this figure section. The figure inset shows the observed heatwave in the two reference data sets and projections in the indicated section of the respective sub-plot. It facilitates readability of color and size of the reference values and comparison to each other and the neighboring projections. Note that the zoom-in causes the dots to overlap more in the inset than in the main sub-plot. The univariate kernel density estimates along the x- (Median Heatwave Area (%)) and y-axis (Heatwave Length (days)) are on the top and right side of the subplots, respectively, with vertical lines in dark blue (Berkeley Earth) and light blue (ERA5) indicating the observed event.

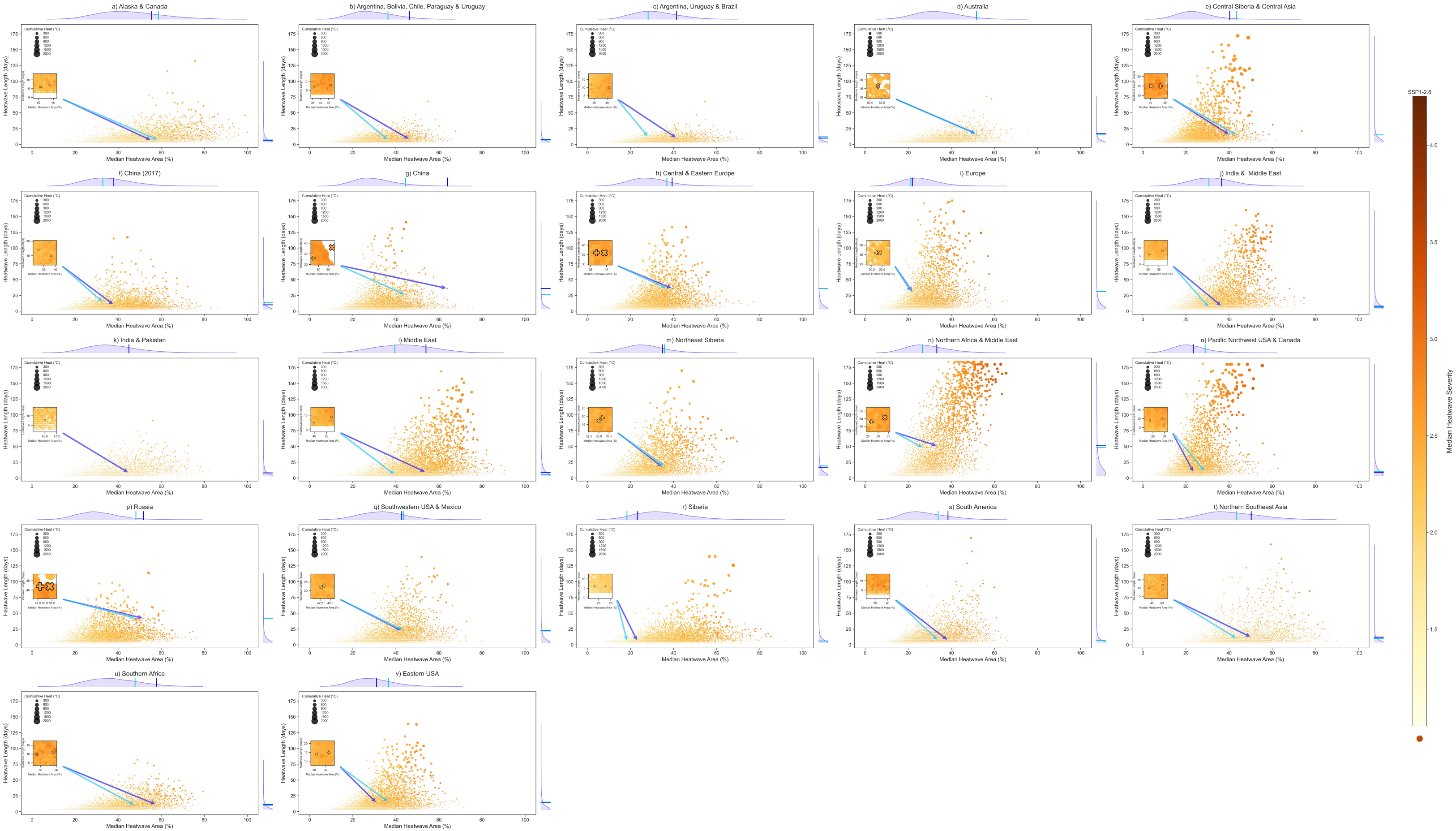
a) Alaska & Canada
b) Argentina, Bolivia, Chile, Paraguay & Uruguay
c) Argentina, Uruguay & Brazil
d) Australia
e) Central Siberia & Central Asia
f) China (2017)
g) China
h) Central & Eastern Europe
i) Europe
j) India & Middle East
k) India & Pakistan
l) Middle East
m) Northeast Siberia
n) Northern Africa & Middle East
o) Pacific Northwest USA & Canada
p) Russia
q) Southwestern USA & Mexico
r) Siberia
s) South America
t) Northern Southeast Asia
u) Southern Africa
v) Eastern USA
Heatwave Length (days)
Median Heatwave Area (%)
Cumulative Heat (°C)
300
600
900
1200
1500
2000
SSP1-2.6
Median Heatwave Severity

Supplementary Figure 102: Multi-HW-characteristic figure according to SSP1-2.6 CMIP6 multi-model ensemble (MME) (Tx97.5p) - Each panel shows regional projections according to SSP1-2.6 model runs in the CMIP6 multi-model ensemble (MME) of four combined heatwave characteristics for each projected heatwave in the ensemble between 2020 and 2100. Each dot represents an individual heatwave in the projections, characterized by Median Heatwave Area (%) (x-axis) as latitude-weighted percentage of grid boxes affected by extreme heat, Heatwave Length (days) (y-axis) in days, Median Heatwave Severity (color), and Cumulative Heat (°C) (dot size). The characteristics of the observed event according to the Berkeley Earth observational data (x symbol) and the ERA5 reanalysis data (+ symbol), respectively, are pointed to by an arrow and a figure inset shows a zoom-in on this figure section. The figure inset shows the observed heatwave in the two reference data sets and projections in the indicated section of the respective sub-plot. It facilitates readability of color and size of the reference values and comparison to each other and the neighboring projections. Note that the zoom-in causes the dots to overlap more in the inset than in the main sub-plot. The univariate kernel density estimates along the x- (Median Heatwave Area (%)) and y-axis (Heatwave Length (days)) are on the top and right side of the subplots, respectively, with vertical lines in dark blue (Berkeley Earth) and light blue (ERA5) indicating the observed event.

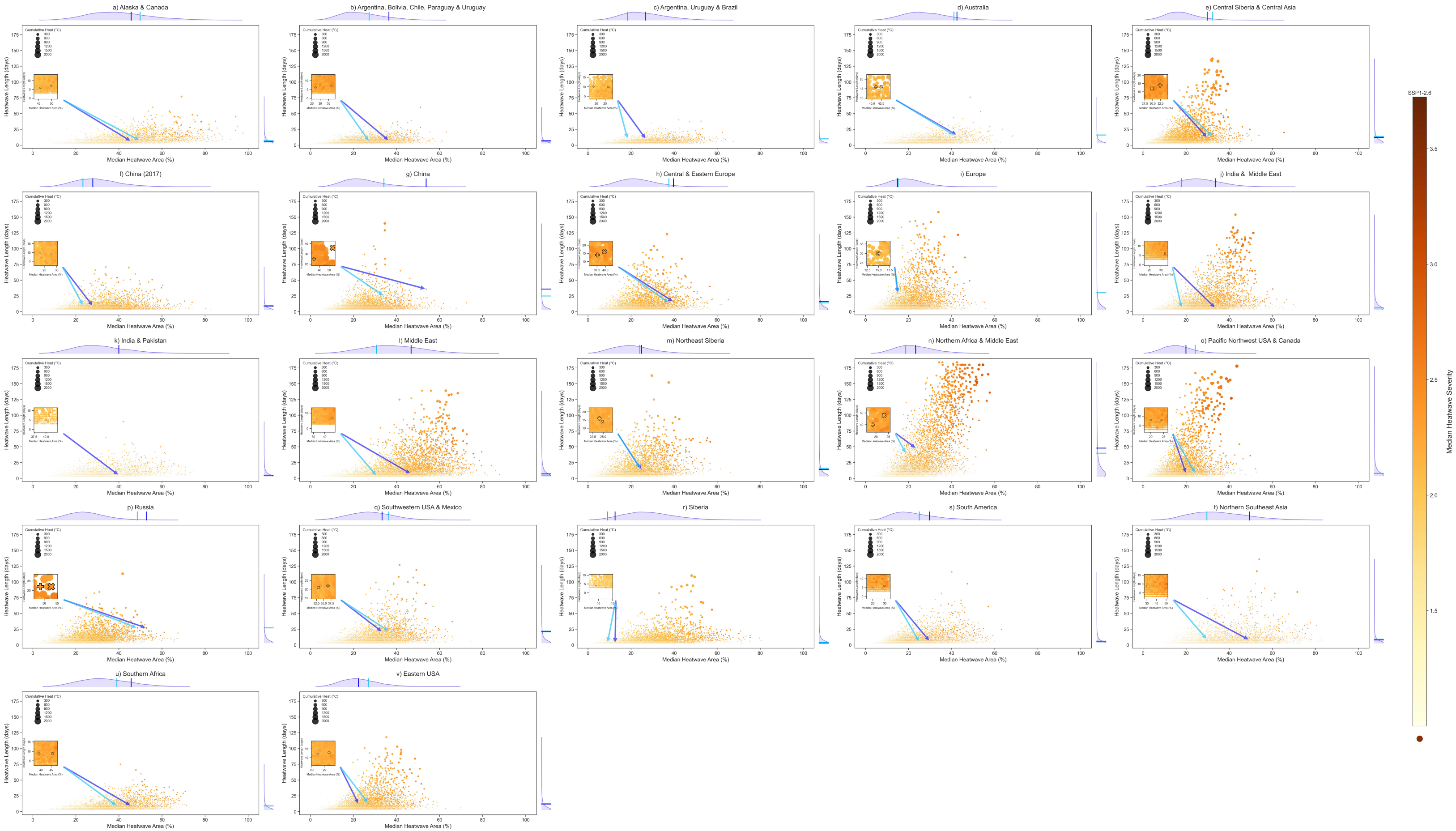
a) Alaska & Canada
b) Argentina, Bolivia, Chile, Paraguay & Uruguay
c) Argentina, Uruguay & Brazil
d) Australia
e) Central Siberia & Central Asia
f) China (2017)
g) China
h) Central & Eastern Europe
i) Europe
j) India & Middle East
k) India & Pakistan
l) Middle East
m) Northeast Siberia
n) Northern Africa & Middle East
o) Pacific Northwest USA & Canada
p) Russia
q) Southwestern USA & Mexico
r) Siberia
s) South America
t) Northern Southeast Asia
u) Southern Africa
v) Eastern USA
Cumulative Heat (°C)
300
600
900
1200
1500
2000
Heatwave Length (days)
Median Heatwave Area (%)
SSP1-2.6
Median Heatwave Severity

Supplementary Figure 103: Multi-HW-characteristic figure according to SSP1-2.6 CMIP6 multi-model ensemble (MME) (Tx99.0p) - Each panel shows regional projections according to SSP1-2.6 model runs in the CMIP6 multi-model ensemble (MME) of four combined heatwave characteristics for each projected heatwave in the ensemble between 2020 and 2100. Each dot represents an individual heatwave in the projections, characterized by Median Heatwave Area (%) (x-axis) as latitude-weighted percentage of grid boxes affected by extreme heat, Heatwave Length (days) (y-axis) in days, Median Heatwave Severity (color), and Cumulative Heat (°C) (dot size). The characteristics of the observed event according to the Berkeley Earth observational data (x symbol) and the ERA5 reanalysis data (+ symbol), respectively, are pointed to by an arrow and a figure inset shows a zoom-in on this figure section. The figure inset shows the observed heatwave in the two reference data sets and projections in the indicated section of the respective sub-plot. It facilitates readability of color and size of the reference values and comparison to each other and the neighboring projections. Note that the zoom-in causes the dots to overlap more in the inset than in the main sub-plot. The univariate kernel density estimates along the x- (Median Heatwave Area (%)) and y-axis (Heatwave Length (days)) are on the top and right side of the subplots, respectively, with vertical lines in dark blue (Berkeley Earth) and light blue (ERA5) indicating the observed event.

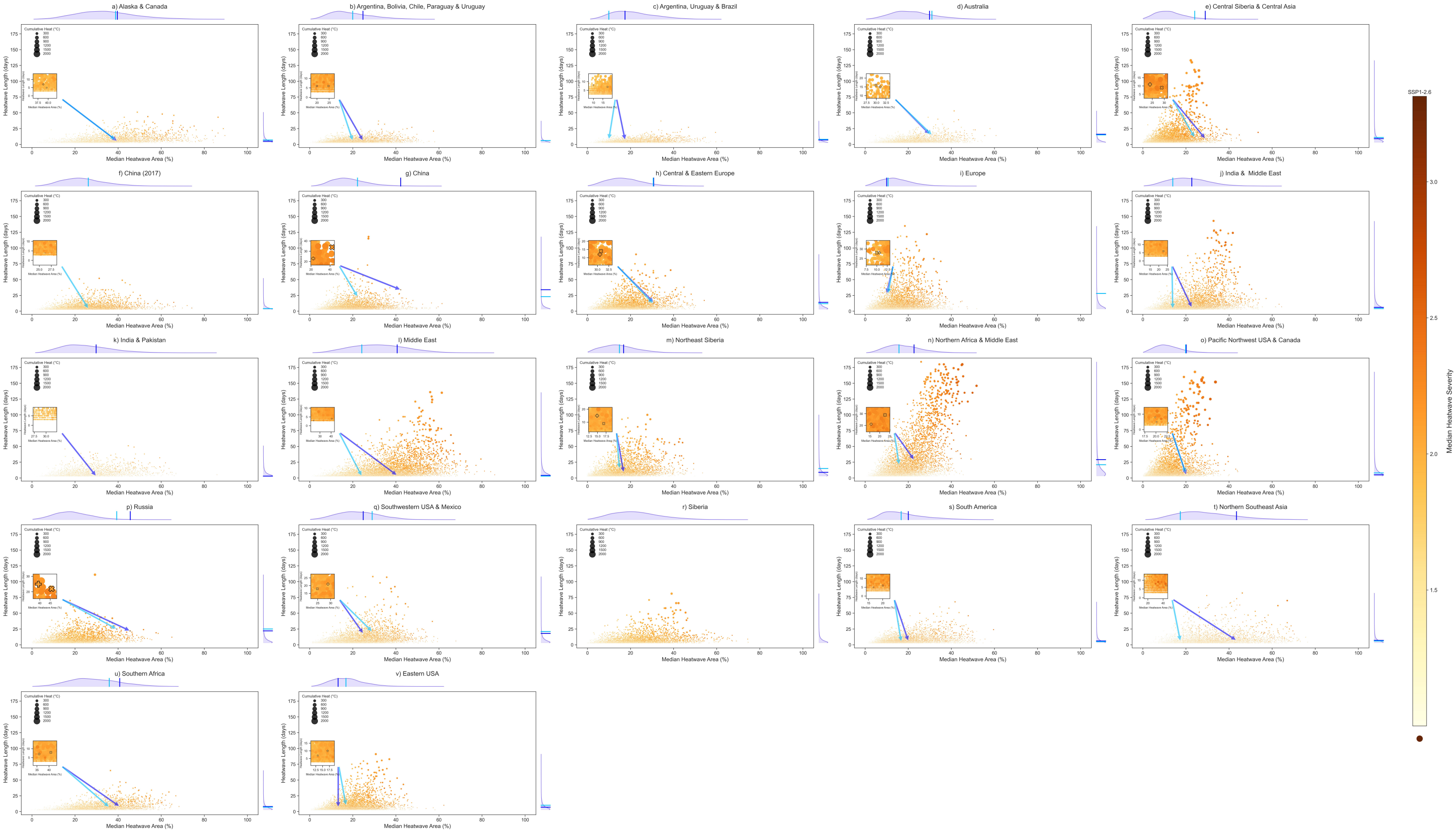
a) Alaska & Canada
b) Argentina, Bolivia, Chile, Paraguay & Uruguay
c) Argentina, Uruguay & Brazil
d) Australia
e) Central Siberia & Central Asia
f) China (2017)
g) China
h) Central & Eastern Europe
i) Europe
j) India & Middle East
k) India & Pakistan
l) Middle East
m) Northeast Siberia
n) Northern Africa & Middle East
o) Pacific Northwest USA & Canada
p) Russia
q) Southwestern USA & Mexico
r) Siberia
s) South America
t) Northern Southeast Asia
u) Southern Africa
v) Eastern USA
Cumulative Heat (°C)
300
600
900
1200
1500
2000
Heatwave Length (days)
Median Heatwave Area (%)
SSP1-2.6
Median Heatwave Severity
1.5
2.0
2.5
3.0

Supplementary Figure 104: Multi-HW-characteristic figure according to SSP2-4.5 CMIP6 multi-model ensemble (MME) (Tx95.0p) - Each panel shows regional projections according to SSP2-4.5 model runs in the CMIP6 multi-model ensemble (MME) of four combined heatwave characteristics for each projected heatwave in the ensemble between 2020 and 2100. Each dot represents an individual heatwave in the projections, characterized by Median Heatwave Area (%) (x-axis) as latitude-weighted percentage of grid boxes affected by extreme heat, Heatwave Length (days) (y-axis) in days, Median Heatwave Severity (color), and Cumulative Heat (°C) (dot size). The characteristics of the observed event according to the Berkeley Earth observational data (x symbol) and the ERA5 reanalysis data (+ symbol), respectively, are pointed to by an arrow and a figure inset shows a zoom-in on this figure section. The figure inset shows the observed heatwave in the two reference data sets and projections in the indicated section of the respective sub-plot. It facilitates readability of color and size of the reference values and comparison to each other and the neighboring projections. Note that the zoom-in causes the dots to overlap more in the inset than in the main sub-plot. The univariate kernel density estimates along the x- (Median Heatwave Area (%)) and y-axis (Heatwave Length (days)) are on the top and right side of the subplots, respectively, with vertical lines in dark blue (Berkeley Earth) and light blue (ERA5) indicating the observed event.

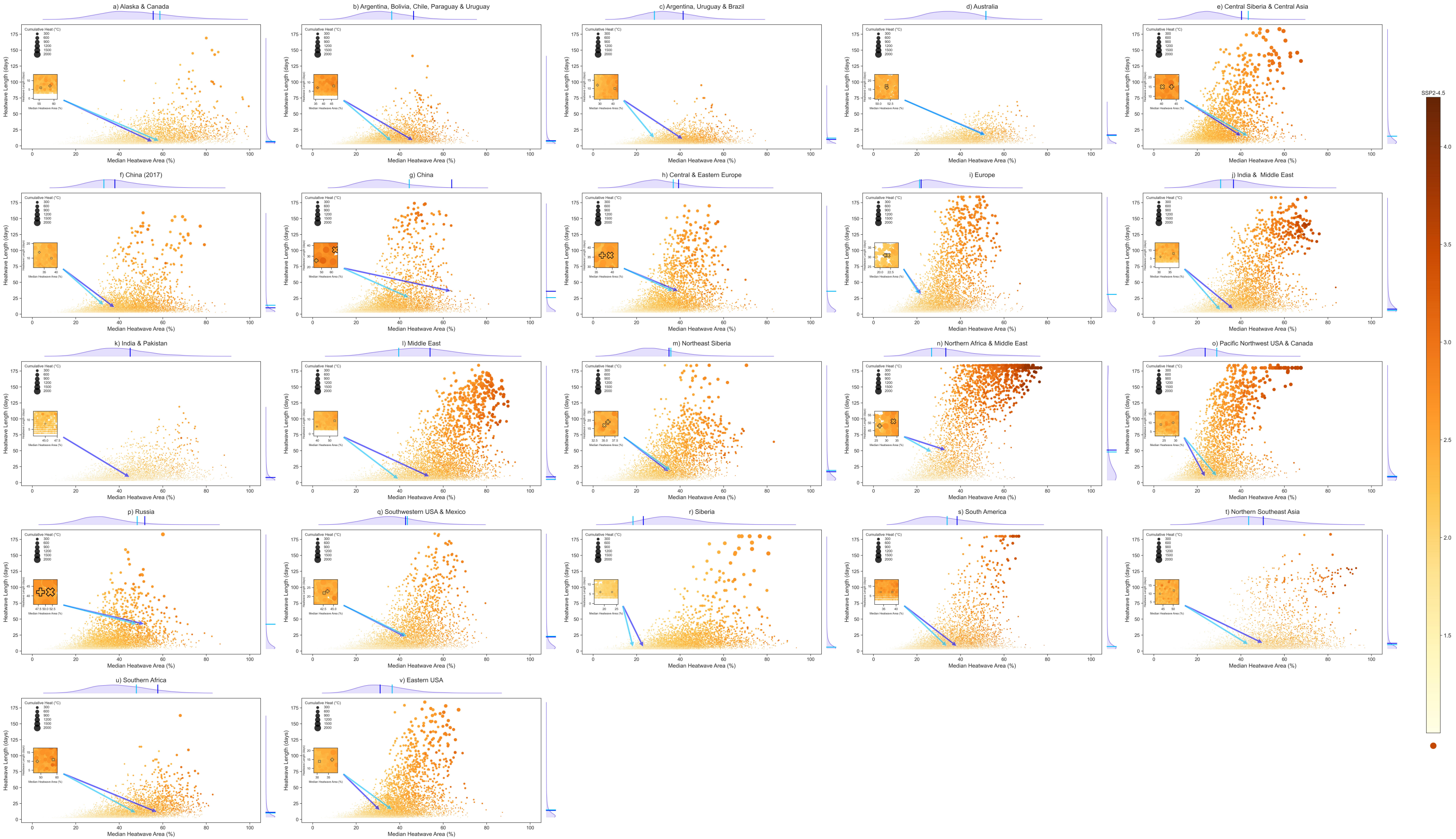
a) Alaska & Canada
b) Argentina, Bolivia, Chile, Paraguay & Uruguay
c) Argentina, Uruguay & Brazil
d) Australia
e) Central Siberia & Central Asia
f) China (2017)
g) China
h) Central & Eastern Europe
i) Europe
j) India & Middle East
k) India & Pakistan
l) Middle East
m) Northeast Siberia
n) Northern Africa & Middle East
o) Pacific Northwest USA & Canada
p) Russia
q) Southwestern USA & Mexico
r) Siberia
s) South America
t) Northern Southeast Asia
u) Southern Africa
v) Eastern USA
Cumulative Heat (°C)
300
600
900
1200
1500
2000
Heatwave Length (days)
Median Heatwave Area (%)
SSP2-4.5
Median Heatwave Severity
1.5
2.0
2.5
3.0
3.5
4.0

Supplementary Figure 105: Multi-HW-characteristic figure according to SSP2-4.5 CMIP6 multi-model ensemble (MME) (Tx97.5p) - Each panel shows regional projections according to SSP2-4.5 model runs in the CMIP6 multi-model ensemble (MME) of four combined heatwave characteristics for each projected heatwave in the ensemble between 2020 and 2100. Each dot represents an individual heatwave in the projections, characterized by Median Heatwave Area (%) (x-axis) as latitude-weighted percentage of grid boxes affected by extreme heat, Heatwave Length (days) (y-axis) in days, Median Heatwave Severity (color), and Cumulative Heat (°C) (dot size). The characteristics of the observed event according to the Berkeley Earth observational data (x symbol) and the ERA5 reanalysis data (+ symbol), respectively, are pointed to by an arrow and a figure inset shows a zoom-in on this figure section. The figure inset shows the observed heatwave in the two reference data sets and projections in the indicated section of the respective sub-plot. It facilitates readability of color and size of the reference values and comparison to each other and the neighboring projections. Note that the zoom-in causes the dots to overlap more in the inset than in the main sub-plot. The univariate kernel density estimates along the x- (Median Heatwave Area (%)) and y-axis (Heatwave Length (days)) are on the top and right side of the subplots, respectively, with vertical lines in dark blue (Berkeley Earth) and light blue (ERA5) indicating the observed event.

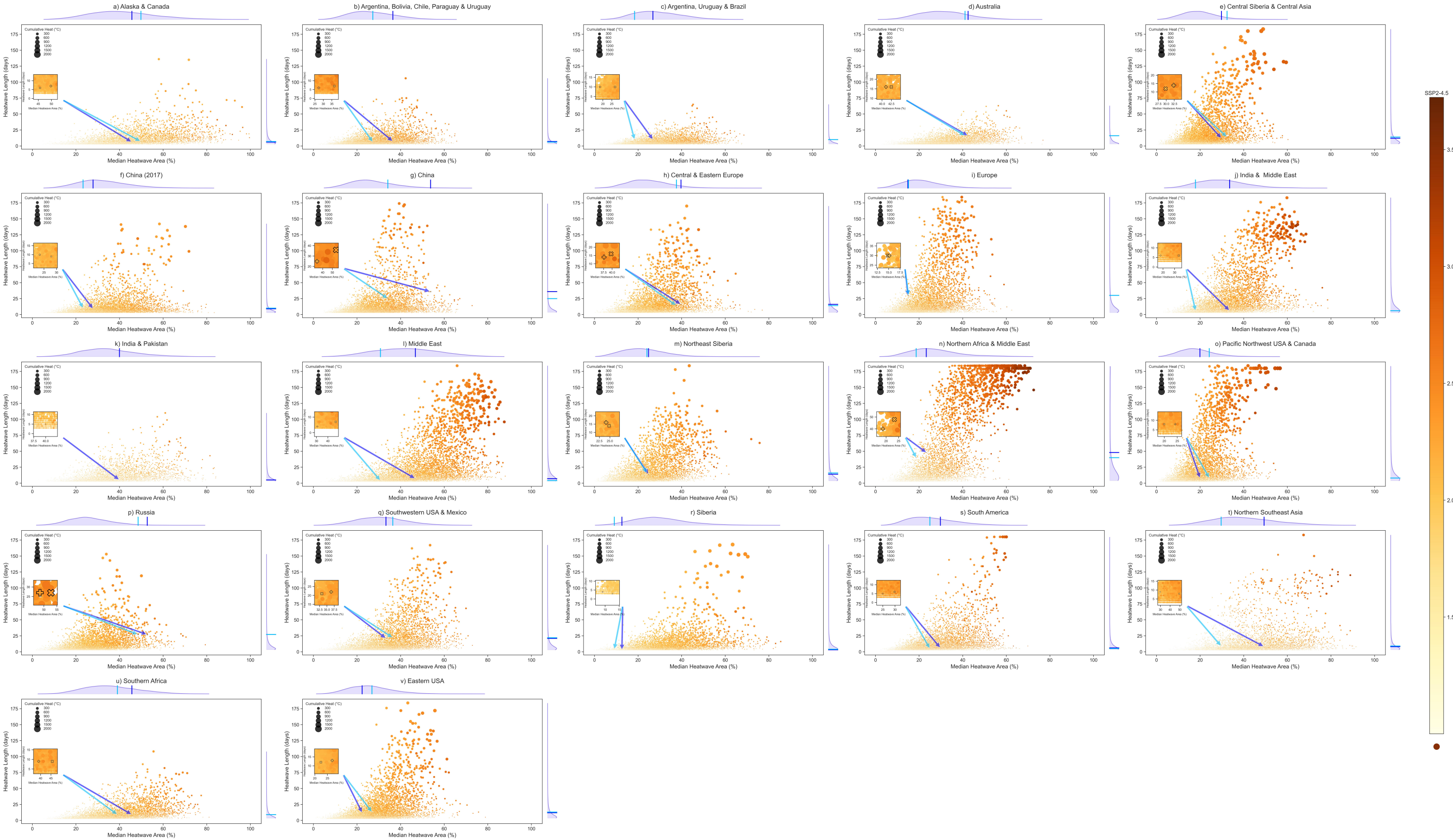

a) Alaska & Canada
b) Argentina, Bolivia, Chile, Paraguay & Uruguay
c) Argentina, Uruguay & Brazil
d) Australia
e) Central Siberia & Central Asia
f) China (2017)
g) China
h) Central & Eastern Europe
i) Europe
j) India & Middle East
k) India & Pakistan
l) Middle East
m) Northeast Siberia
n) Northern Africa & Middle East
o) Pacific Northwest USA & Canada
p) Russia
q) Southwestern USA & Mexico
r) Siberia
s) South America
t) Northern Southeast Asia
u) Southern Africa
v) Eastern USA
Cumulative Heat (°C)
300
600
900
1200
1500
2000
Heatwave Length (days)
Median Heatwave Area (%)
0
20
40
60
80
100
25
50
75
125
150
175
SSP2-4.5
Median Heatwave Severity
1.5
2.0
2.5
3.0
3.5

Supplementary Figure 106: Multi-HW-characteristic figure according to SSP2-4.5 CMIP6 multi-model ensemble (MME) (Tx99.0p) - Each panel shows regional projections according to SSP2-4.5 model runs in the CMIP6 multi-model ensemble (MME) of four combined heatwave characteristics for each projected heatwave in the ensemble between 2020 and 2100. Each dot represents an individual heatwave in the projections, characterized by Median Heatwave Area (%) (x-axis) as latitude-weighted percentage of grid boxes affected by extreme heat, Heatwave Length (days) (y-axis) in days, Median Heatwave Severity (color), and Cumulative Heat (°C) (dot size). The characteristics of the observed event according to the Berkeley Earth observational data (x symbol) and the ERA5 reanalysis data (+ symbol), respectively, are pointed to by an arrow and a figure inset shows a zoom-in on this figure section. The figure inset shows the observed heatwave in the two reference data sets and projections in the indicated section of the respective sub-plot. It facilitates readability of color and size of the reference values and comparison to each other and the neighboring projections. Note that the zoom-in causes the dots to overlap more in the inset than in the main sub-plot. The univariate kernel density estimates along the x- (Median Heatwave Area (%)) and y-axis (Heatwave Length (days)) are on the top and right side of the subplots, respectively, with vertical lines in dark blue (Berkeley Earth) and light blue (ERA5) indicating the observed event.

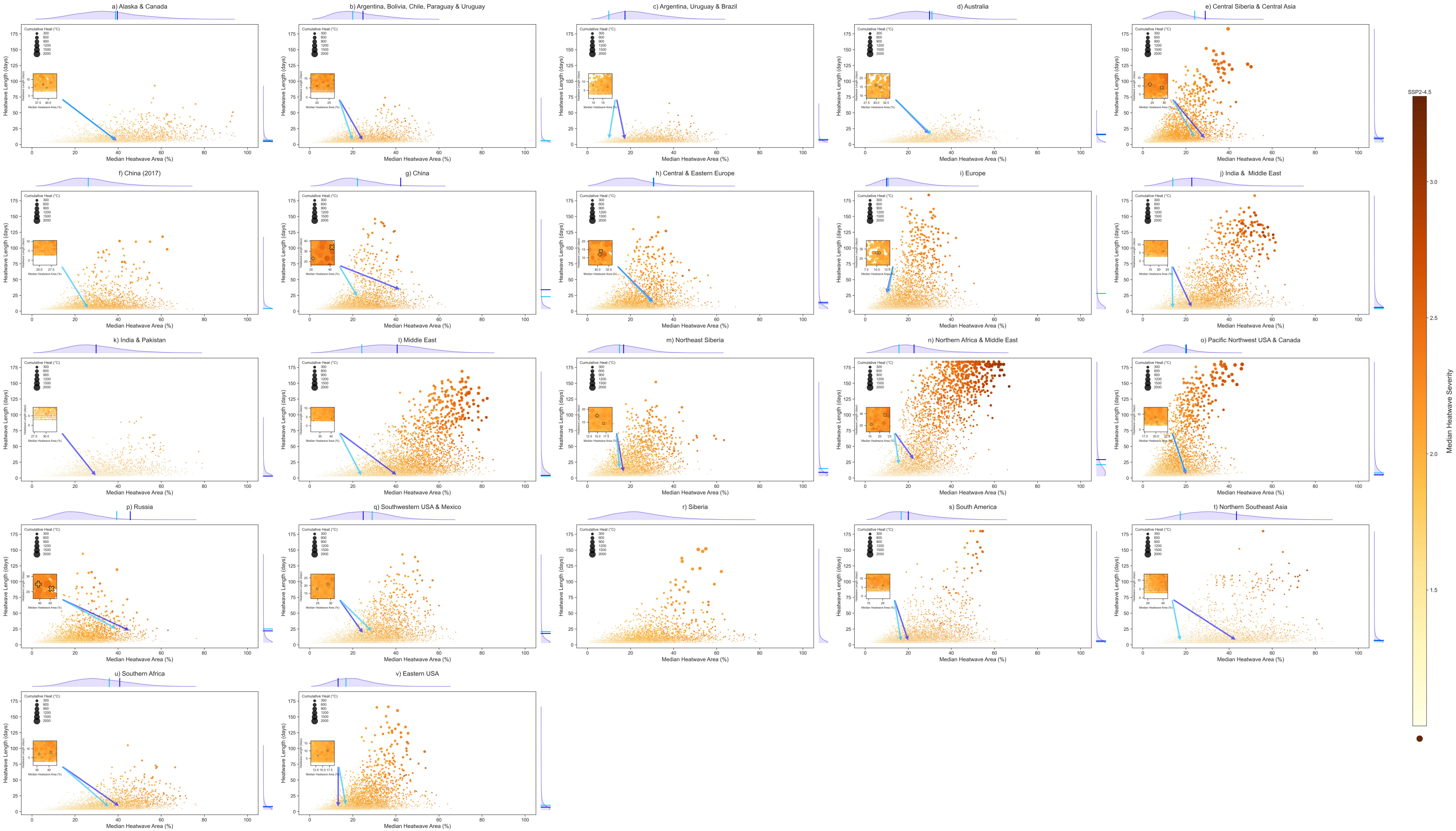

a) Alaska & Canada
b) Argentina, Bolivia, Chile, Paraguay & Uruguay
c) Argentina, Uruguay & Brazil
d) Australia
e) Central Siberia & Central Asia
f) China (2017)
g) China
h) Central & Eastern Europe
i) Europe
j) India & Middle East
k) India & Pakistan
l) Middle East
m) Northeast Siberia
n) Northern Africa & Middle East
o) Pacific Northwest USA & Canada
p) Russia
q) Southwestern USA & Mexico
r) Siberia
s) South America
t) Northern Southeast Asia
u) Southern Africa
v) Eastern USA
Cumulative Heat (°C)
300
600
900
1200
1500
2000
Heatwave Length (days)
Median Heatwave Area (%)
SSP2-4.5
Median Heatwave Severity
1.5
2.0
2.5
3.0

Supplementary Figure 107: Multi-HW-characteristic figure according to SSP3-7.0 CMIP6 multi-model ensemble (MME) (Tx95.0p) - Each panel shows regional projections according to SSP3-7.0 model runs in the CMIP6 multi-model ensemble (MME) of four combined heatwave characteristics for each projected heatwave in the ensemble between 2020 and 2100. Each dot represents an individual heatwave in the projections, characterized by Median Heatwave Area (%) (x-axis) as latitude-weighted percentage of grid boxes affected by extreme heat, Heatwave Length (days) (y-axis) in days, Median Heatwave Severity (color), and Cumulative Heat (°C) (dot size). The characteristics of the observed event according to the Berkeley Earth observational data (x symbol) and the ERA5 reanalysis data (+ symbol), respectively, are pointed to by an arrow and a figure inset shows a zoom-in on this figure section. The figure inset shows the observed heatwave in the two reference data sets and projections in the indicated section of the respective sub-plot. It facilitates readability of color and size of the reference values and comparison to each other and the neighboring projections. Note that the zoom-in causes the dots to overlap more in the inset than in the main sub-plot. The univariate kernel density estimates along the x- (Median Heatwave Area (%)) and y-axis (Heatwave Length (days)) are on the top and right side of the subplots, respectively, with vertical lines in dark blue (Berkeley Earth) and light blue (ERA5) indicating the observed event.

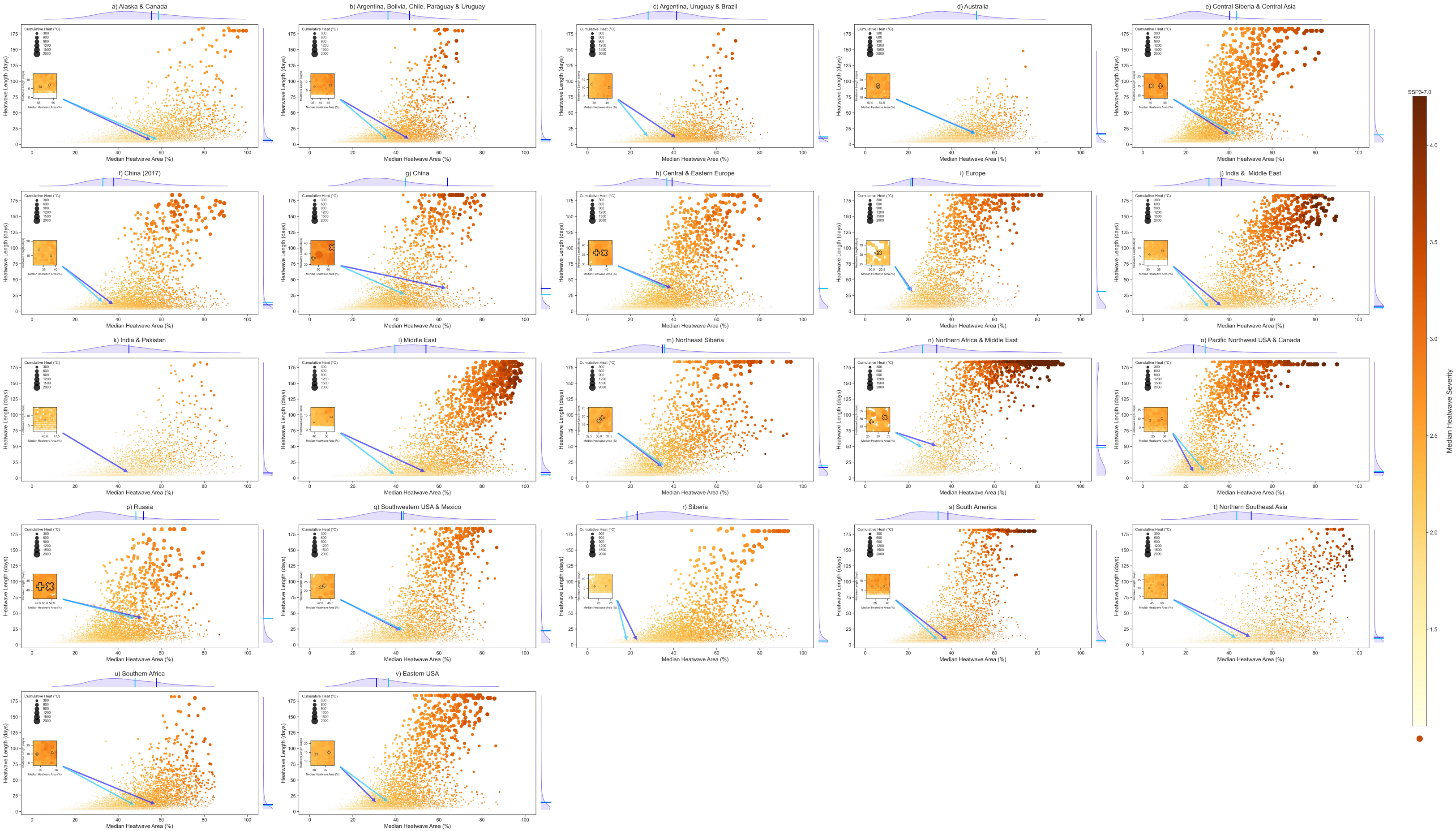

a) Alaska & Canada
b) Argentina, Bolivia, Chile, Paraguay & Uruguay
c) Argentina, Uruguay & Brazil
d) Australia
e) Central Siberia & Central Asia
f) China (2017)
g) China
h) Central & Eastern Europe
i) Europe
j) India & Middle East
k) India & Pakistan
l) Middle East
m) Northeast Siberia
n) Northern Africa & Middle East
o) Pacific Northwest USA & Canada
p) Russia
q) Southwestern USA & Mexico
r) Siberia
s) South America
t) Northern Southeast Asia
u) Southern Africa
v) Eastern USA
Cumulative Heat (°C)
300
600
900
1200
1500
2000
Heatwave Length (days)
Median Heatwave Area (%)
SSP3-7.0
Median Heatwave Severity

Supplementary Figure 108: Multi-HW-characteristic figure according to SSP3-7.0 CMIP6 multi-model ensemble (MME) (Tx97.5p) - Each panel shows regional projections according to SSP3-7.0 model runs in the CMIP6 multi-model ensemble (MME) of four combined heatwave characteristics for each projected heatwave in the ensemble between 2020 and 2100. Each dot represents an individual heatwave in the projections, characterized by Median Heatwave Area (%) (x-axis) as latitude-weighted percentage of grid boxes affected by extreme heat, Heatwave Length (days) (y-axis) in days, Median Heatwave Severity (color), and Cumulative Heat (°C) (dot size). The characteristics of the observed event according to the Berkeley Earth observational data (x symbol) and the ERA5 reanalysis data (+ symbol), respectively, are pointed to by an arrow and a figure inset shows a zoom-in on this figure section. The figure inset shows the observed heatwave in the two reference data sets and projections in the indicated section of the respective sub-plot. It facilitates readability of color and size of the reference values and comparison to each other and the neighboring projections. Note that the zoom-in causes the dots to overlap more in the inset than in the main sub-plot. The univariate kernel density estimates along the x- (Median Heatwave Area (%)) and y-axis (Heatwave Length (days)) are on the top and right side of the subplots, respectively, with vertical lines in dark blue (Berkeley Earth) and light blue (ERA5) indicating the observed event.

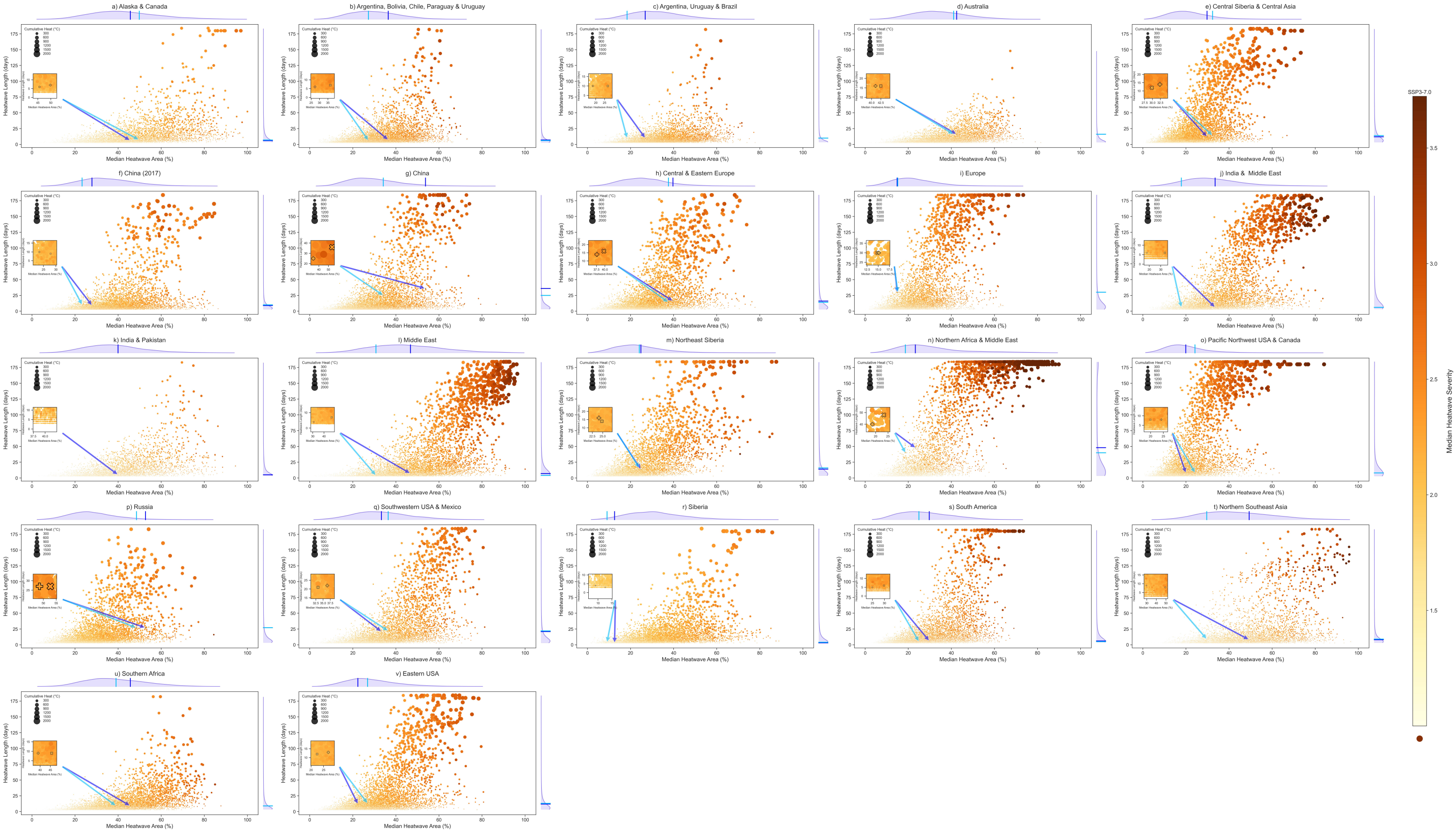

a) Alaska & Canada
b) Argentina, Bolivia, Chile, Paraguay & Uruguay
c) Argentina, Uruguay & Brazil
d) Australia
e) Central Siberia & Central Asia
f) China (2017)
g) China
h) Central & Eastern Europe
i) Europe
j) India & Middle East
k) India & Pakistan
l) Middle East
m) Northeast Siberia
n) Northern Africa & Middle East
o) Pacific Northwest USA & Canada
p) Russia
q) Southwestern USA & Mexico
r) Siberia
s) South America
t) Northern Southeast Asia
u) Southern Africa
v) Eastern USA
Cumulative Heat (°C)
300
600
900
1200
1500
2000
Heatwave Length (days)
Median Heatwave Area (%)
SSP3-7.0
Median Heatwave Severity

Supplementary Figure 109: Multi-HW-characteristic figure according to SSP3-7.0 CMIP6 multi-model ensemble (MME) (Tx99.0p) - Each panel shows regional projections according to SSP3-7.0 model runs in the CMIP6 multi-model ensemble (MME) of four combined heatwave characteristics for each projected heatwave in the ensemble between 2020 and 2100. Each dot represents an individual heatwave in the projections, characterized by Median Heatwave Area (%) (x-axis) as latitude-weighted percentage of grid boxes affected by extreme heat, Heatwave Length (days) (y-axis) in days, Median Heatwave Severity (color), and Cumulative Heat (°C) (dot size). The characteristics of the observed event according to the Berkeley Earth observational data (x symbol) and the ERA5 reanalysis data (+ symbol), respectively, are pointed to by an arrow and a figure inset shows a zoom-in on this figure section. The figure inset shows the observed heatwave in the two reference data sets and projections in the indicated section of the respective sub-plot. It facilitates readability of color and size of the reference values and comparison to each other and the neighboring projections. Note that the zoom-in causes the dots to overlap more in the inset than in the main sub-plot. The univariate kernel density estimates along the x- (Median Heatwave Area (%)) and y-axis (Heatwave Length (days)) are on the top and right side of the subplots, respectively, with vertical lines in dark blue (Berkeley Earth) and light blue (ERA5) indicating the observed event.

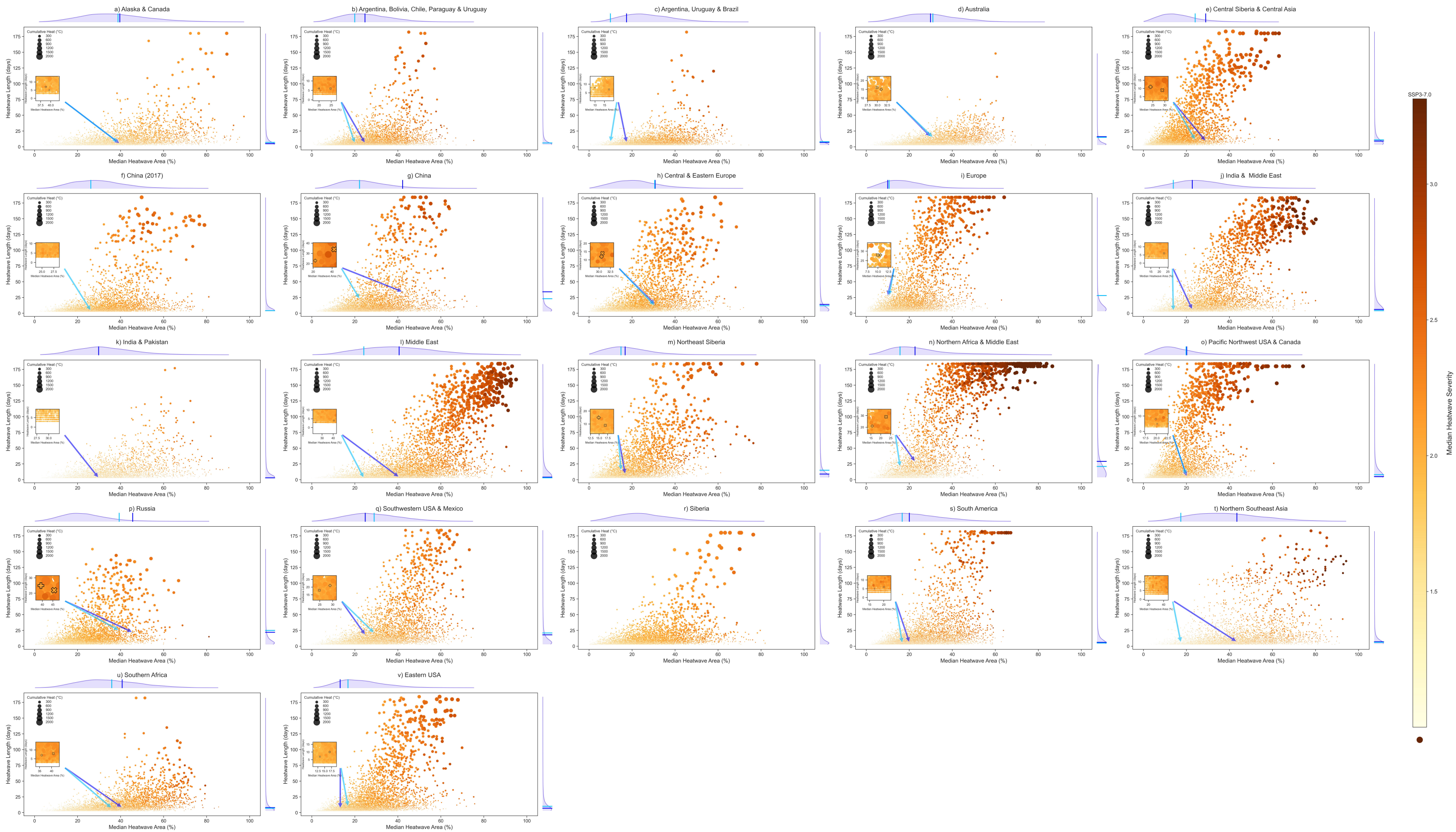

a) Alaska & Canada
b) Argentina, Bolivia, Chile, Paraguay & Uruguay
c) Argentina, Uruguay & Brazil
d) Australia
e) Central Siberia & Central Asia
f) China (2017)
g) China
h) Central & Eastern Europe
i) Europe
j) India & Middle East
k) India & Pakistan
l) Middle East
m) Northeast Siberia
n) Northern Africa & Middle East
o) Pacific Northwest USA & Canada
p) Russia
q) Southwestern USA & Mexico
r) Siberia
s) South America
t) Northern Southeast Asia
u) Southern Africa
v) Eastern USA
Cumulative Heat (°C)
300
600
900
1200
1500
2000
Heatwave Length (days)
Median Heatwave Area (%)
SSP3-7.0
Median Heatwave Severity

Supplementary Figure 110: Multi-HW-characteristic figure according to SSP5-8.5 CMIP6 multi-model ensemble (MME) (Tx95.0p) - Each panel shows regional projections according to SSP5-8.5 model runs in the CMIP6 multi-model ensemble (MME) of four combined heatwave characteristics for each projected heatwave in the ensemble between 2020 and 2100. Each dot represents an individual heatwave in the projections, characterized by Median Heatwave Area (%) (x-axis) as latitude-weighted percentage of grid boxes affected by extreme heat, Heatwave Length (days) (y-axis) in days, Median Heatwave Severity (color), and Cumulative Heat (°C) (dot size). The characteristics of the observed event according to the Berkeley Earth observational data (x symbol) and the ERA5 reanalysis data (+ symbol), respectively, are pointed to by an arrow and a figure inset shows a zoom-in on this figure section. The figure inset shows the observed heatwave in the two reference data sets and projections in the indicated section of the respective sub-plot. It facilitates readability of color and size of the reference values and comparison to each other and the neighboring projections. Note that the zoom-in causes the dots to overlap more in the inset than in the main sub-plot. The univariate kernel density estimates along the x- (Median Heatwave Area (%)) and y-axis (Heatwave Length (days)) are on the top and right side of the subplots, respectively, with vertical lines in dark blue (Berkeley Earth) and light blue (ERA5) indicating the observed event.

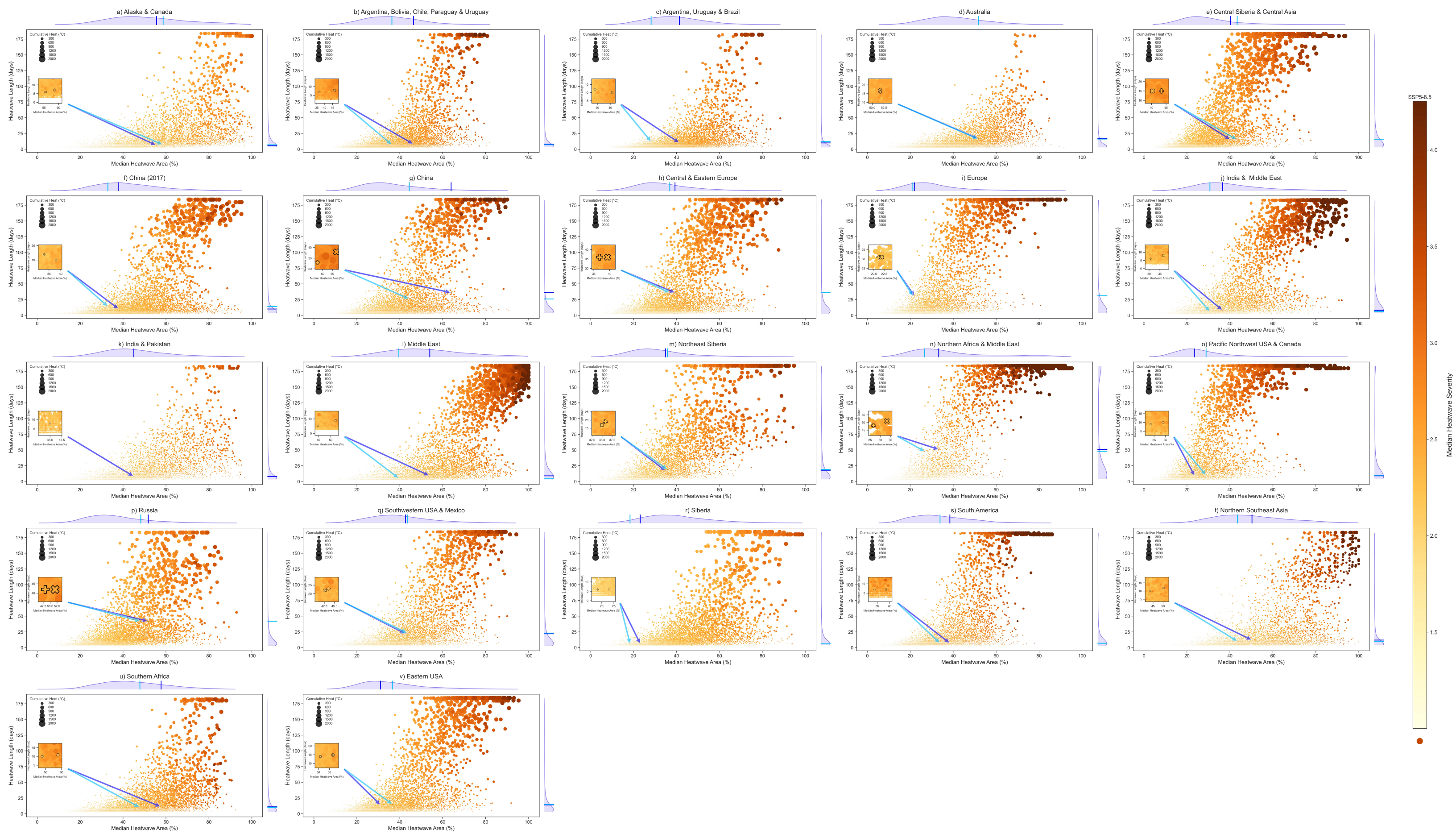
a) Alaska & Canada
b) Argentina, Bolivia, Chile, Paraguay & Uruguay
c) Argentina, Uruguay & Brazil
d) Australia
e) Central Siberia & Central Asia
f) China (2017)
g) China
h) Central & Eastern Europe
i) Europe
j) India & Middle East
k) India & Pakistan
l) Middle East
m) Northeast Siberia
n) Northern Africa & Middle East
o) Pacific Northwest USA & Canada
p) Russia
q) Southwestern USA & Mexico
r) Siberia
s) South America
t) Northern Southeast Asia
u) Southern Africa
v) Eastern USA
Cumulative Heat (°C)
300
600
900
1200
1500
2000
Heatwave Length (days)
Median Heatwave Area (%)
SSP5-8.5
Median Heatwave Severity

Supplementary Figure 111: Multi-HW-characteristic figure according to SSP5-8.5 CMIP6 multi-model ensemble (MME) (Tx97.5p) - Each panel shows regional projections according to SSP5-8.5 model runs in the CMIP6 multi-model ensemble (MME) of four combined heatwave characteristics for each projected heatwave in the ensemble between 2020 and 2100. Each dot represents an individual heatwave in the projections, characterized by Median Heatwave Area (%) (x-axis) as latitude-weighted percentage of grid boxes affected by extreme heat, Heatwave Length (days) (y-axis) in days, Median Heatwave Severity (color), and Cumulative Heat (°C) (dot size). The characteristics of the observed event according to the Berkeley Earth observational data (x symbol) and the ERA5 reanalysis data (+ symbol), respectively, are pointed to by an arrow and a figure inset shows a zoom-in on this figure section. The figure inset shows the observed heatwave in the two reference data sets and projections in the indicated section of the respective sub-plot. It facilitates readability of color and size of the reference values and comparison to each other and the neighboring projections. Note that the zoom-in causes the dots to overlap more in the inset than in the main sub-plot. The univariate kernel density estimates along the x- (Median Heatwave Area (%)) and y-axis (Heatwave Length (days)) are on the top and right side of the subplots, respectively, with vertical lines in dark blue (Berkeley Earth) and light blue (ERA5) indicating the observed event.

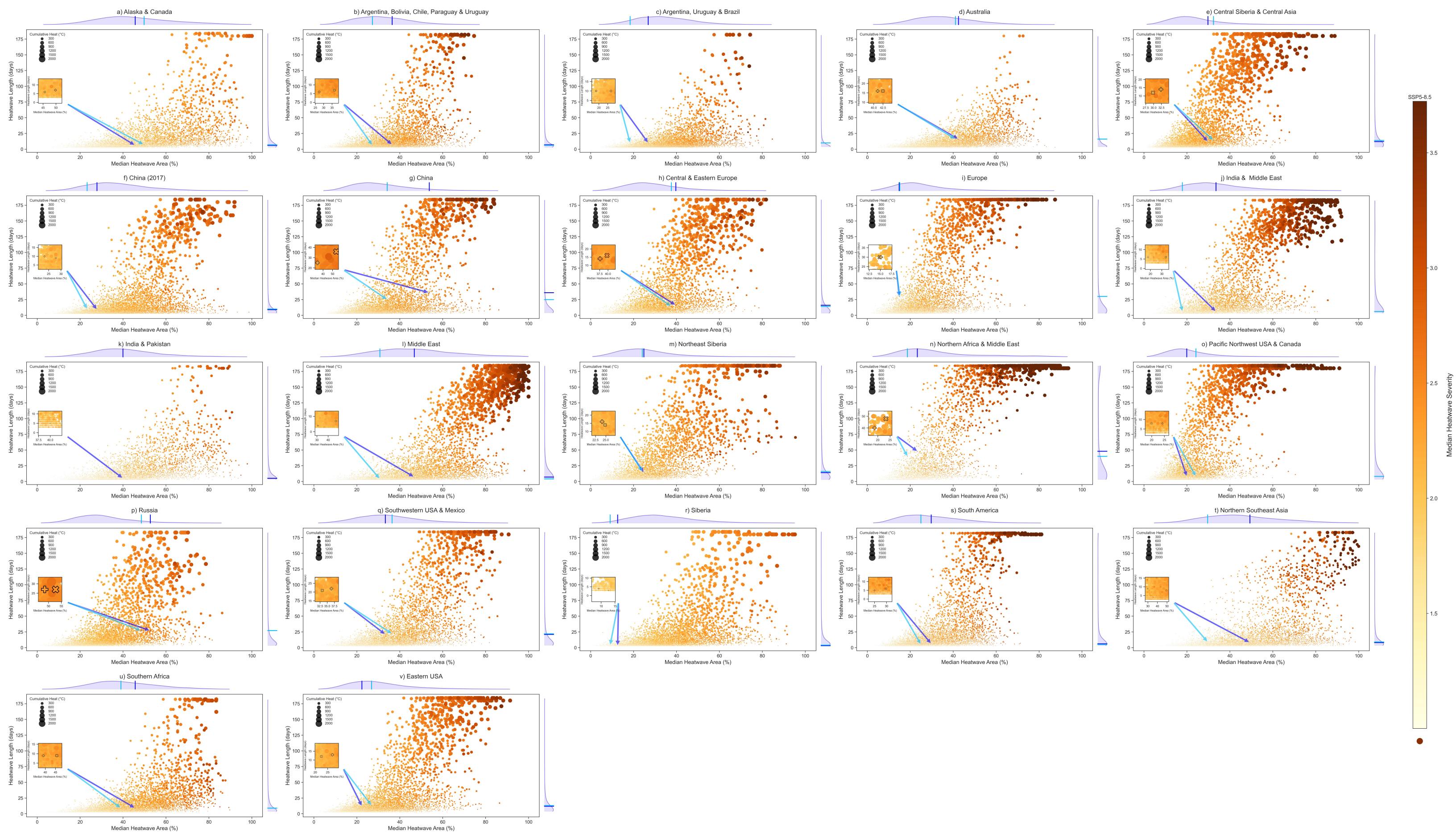
a) Alaska & Canada
b) Argentina, Bolivia, Chile, Paraguay & Uruguay
c) Argentina, Uruguay & Brazil
d) Australia
e) Central Siberia & Central Asia
f) China (2017)
g) China
h) Central & Eastern Europe
i) Europe
j) India & Middle East
k) India & Pakistan
l) Middle East
m) Northeast Siberia
n) Northern Africa & Middle East
o) Pacific Northwest USA & Canada
p) Russia
q) Southwestern USA & Mexico
r) Siberia
s) South America
t) Northern Southeast Asia
u) Southern Africa
v) Eastern USA
Cumulative Heat (°C)
300
600
900
1200
1500
2000
Heatwave Length (days)
Median Heatwave Area (%)
SSP5-8.5
Median Heatwave Severity

Supplementary Figure 112: Multi-HW-characteristic figure according to SSP5-8.5 CMIP6 multi-model ensemble (MME) (Tx99.0p) - Each panel shows regional projections according to SSP5-8.5 model runs in the CMIP6 multi-model ensemble (MME) of four combined heatwave characteristics for each projected heatwave in the ensemble between 2020 and 2100. Each dot represents an individual heatwave in the projections, characterized by Median Heatwave Area (%) (x-axis) as latitude-weighted percentage of grid boxes affected by extreme heat, Heatwave Length (days) (y-axis) in days, Median Heatwave Severity (color), and Cumulative Heat (°C) (dot size). The characteristics of the observed event according to the Berkeley Earth observational data (x symbol) and the ERA5 reanalysis data (+ symbol), respectively, are pointed to by an arrow and a figure inset shows a zoom-in on this figure section. The figure inset shows the observed heatwave in the two reference data sets and projections in the indicated section of the respective sub-plot. It facilitates readability of color and size of the reference values and comparison to each other and the neighboring projections. Note that the zoom-in causes the dots to overlap more in the inset than in the main sub-plot. The univariate kernel density estimates along the x- (Median Heatwave Area (%)) and y-axis (Heatwave Length (days)) are on the top and right side of the subplots, respectively, with vertical lines in dark blue (Berkeley Earth) and light blue (ERA5) indicating the observed event.

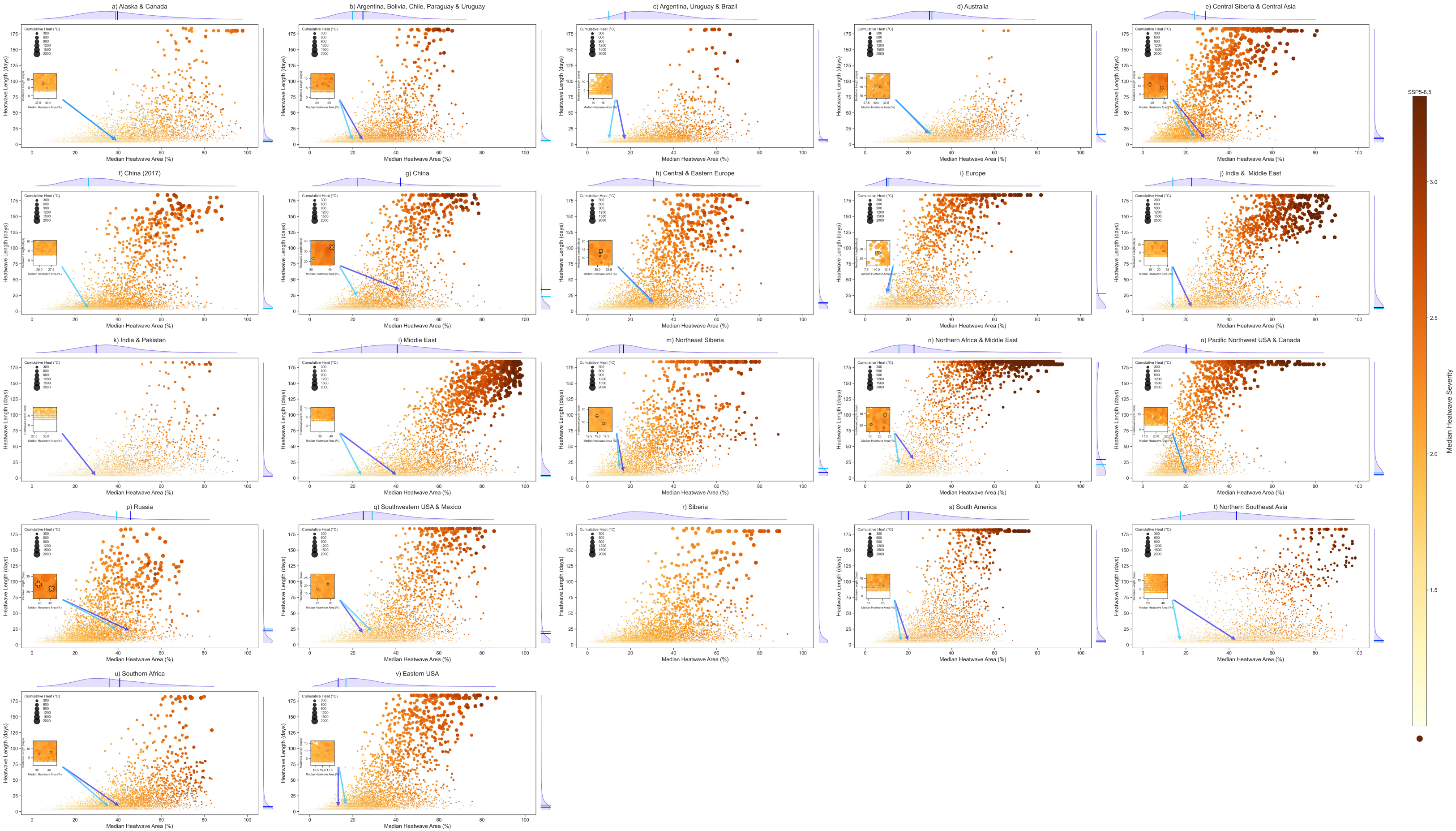

a) Alaska & Canada
b) Argentina, Bolivia, Chile, Paraguay & Uruguay
c) Argentina, Uruguay & Brazil
d) Australia
e) Central Siberia & Central Asia
f) China (2017)
g) China
h) Central & Eastern Europe
i) Europe
j) India & Middle East
k) India & Pakistan
l) Middle East
m) Northeast Siberia
n) Northern Africa & Middle East
o) Pacific Northwest USA & Canada
p) Russia
q) Southwestern USA & Mexico
r) Siberia
s) South America
t) Northern Southeast Asia
u) Southern Africa
v) Eastern USA
Cumulative Heat (°C)
300
600
900
1200
1500
2000
Heatwave Length (days)
0
25
50
75
100
125
150
175
Median Heatwave Area (%)
0
20
40
60
80
100
SSP5-8.5
Median Heatwave Severity
1.5
2.0
2.5
3.0

Supplementary Figure 113: Kernel Density Estimates (KDE) across Decades for Cumulative Heat (°C) in Historical CMIP6 multi-model ensemble (MME) (Tx95.0p) - Kernel Density Estimates (KDE) of Cumulative Heat (°C) for different Decades in the CMIP6 multi-model ensemble (MME). Area under each curve is normalized to 1. Cumulative Heat (°C) of the observed event is indicated by vertical lines in dark blue (Berkeley Earth) and light blue (ERA5).

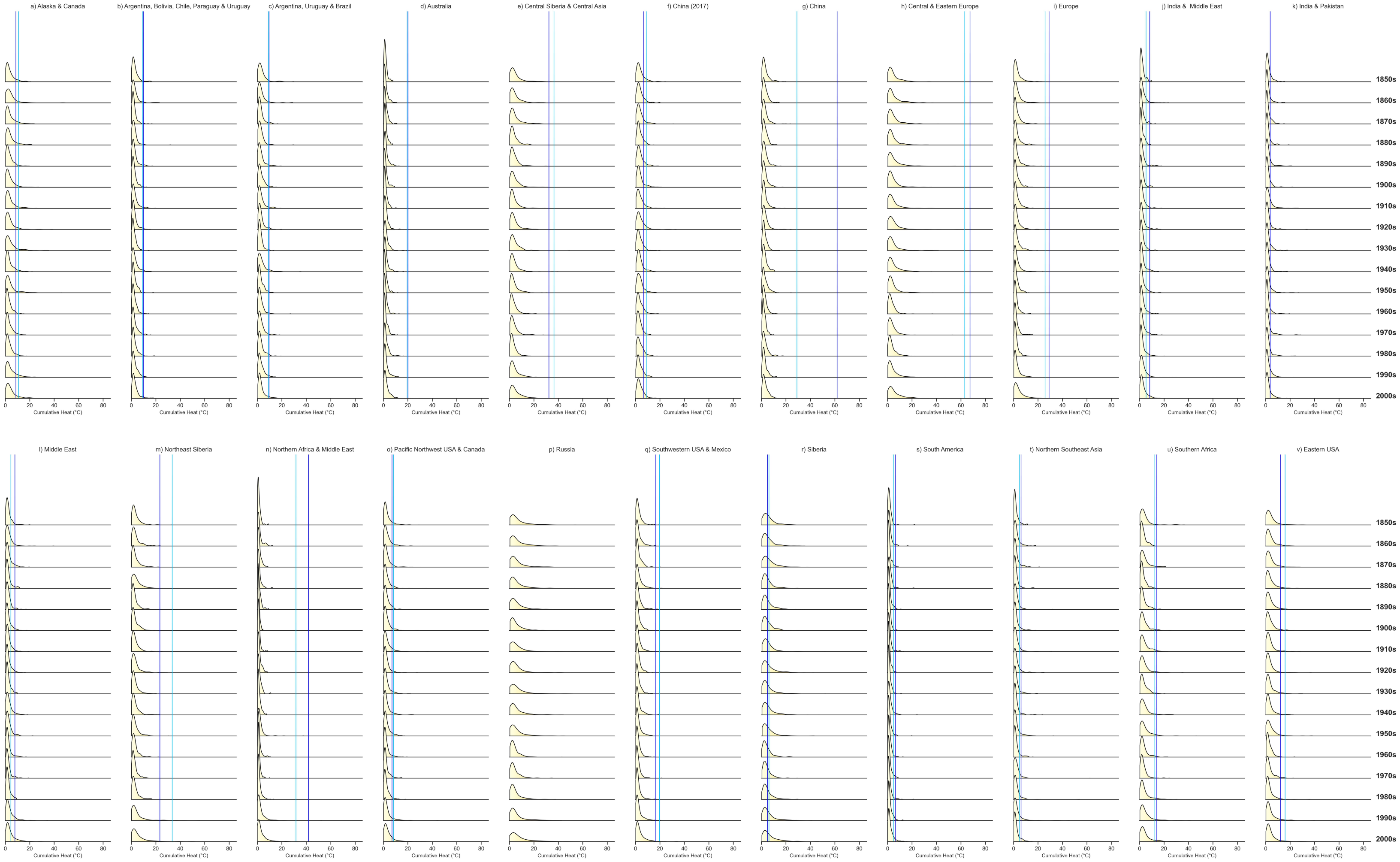

a) Alaska & Canada
b) Argentina, Bolivia, Chile, Paraguay & Uruguay
c) Argentina, Uruguay & Brazil
d) Australia
e) Central Siberia & Central Asia
f) China (2017)
g) China
h) Central & Eastern Europe
i) Europe
j) India & Middle East
k) India & Pakistan
l) Middle East
m) Northeast Siberia
n) Northern Africa & Middle East
o) Pacific Northwest USA & Canada
p) Russia
q) Southwestern USA & Mexico
r) Siberia
s) South America
t) Northern Southeast Asia
u) Southern Africa
v) Eastern USA
1850s
1860s
1870s
1880s
1890s
1900s
1910s
1920s
1930s
1940s
1950s
1960s
1970s
1980s
1990s
2000s
0
20
40
60
80
Cumulative Heat (°C)

Supplementary Figure 114: Kernel Density Estimates (KDE) across Decades for Cumulative Heat (°C) in Historical CMIP6 multi-model ensemble (MME) (Tx97.5p) - Kernel Density Estimates (KDE) of Cumulative Heat (°C) for different Decades in the CMIP6 multi-model ensemble (MME). Area under each curve is normalized to 1. Cumulative Heat (°C) of the observed event is indicated by vertical lines in dark blue (Berkeley Earth) and light blue (ERA5).

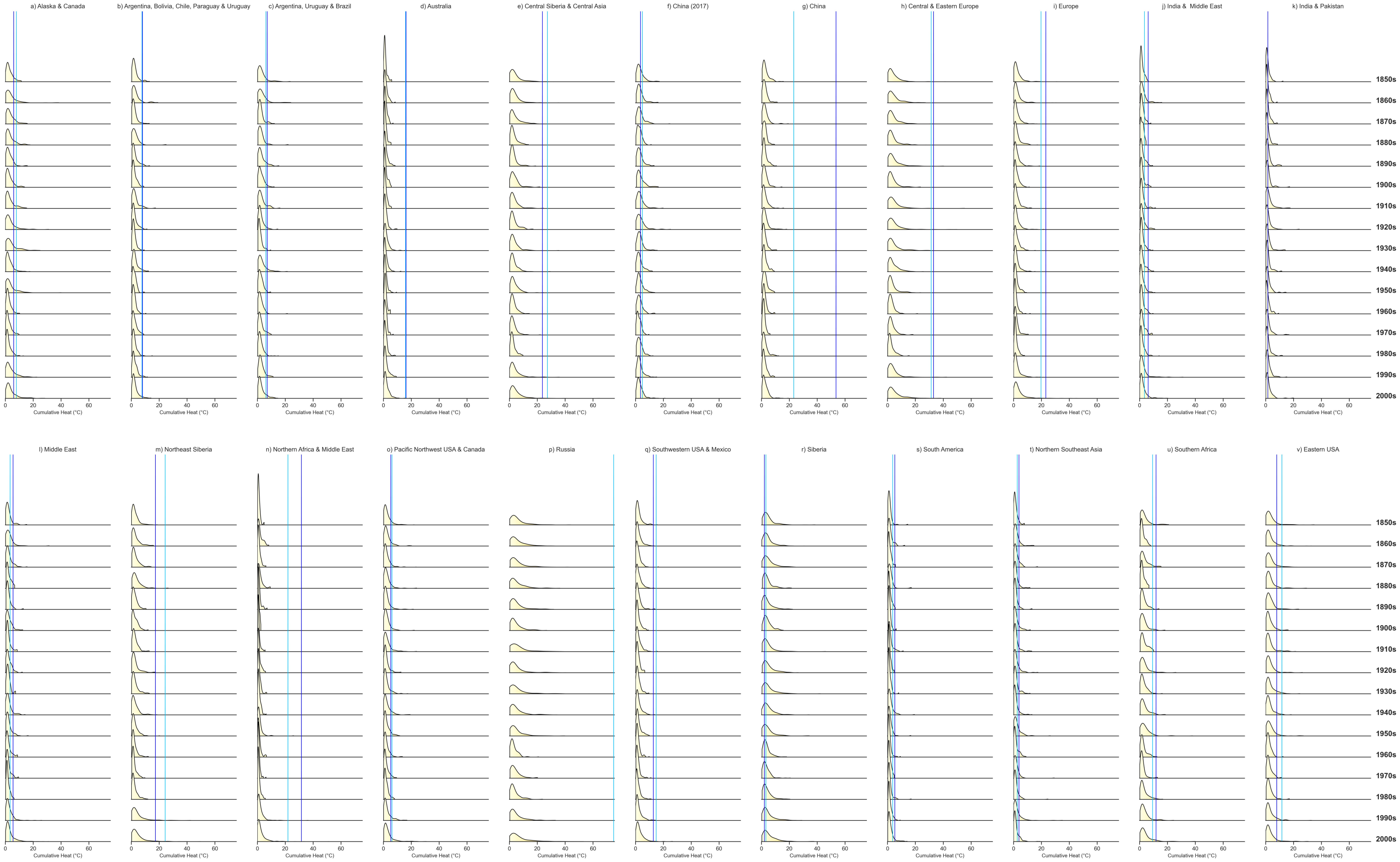
a) Alaska & Canada
b) Argentina, Bolivia, Chile, Paraguay & Uruguay
c) Argentina, Uruguay & Brazil
d) Australia
e) Central Siberia & Central Asia
f) China (2017)
g) China
h) Central & Eastern Europe
i) Europe
j) India & Middle East
k) India & Pakistan
l) Middle East
m) Northeast Siberia
n) Northern Africa & Middle East
o) Pacific Northwest USA & Canada
p) Russia
q) Southwestern USA & Mexico
r) Siberia
s) South America
t) Northern Southeast Asia
u) Southern Africa
v) Eastern USA
1850s
1860s
1870s
1880s
1890s
1900s
1910s
1920s
1930s
1940s
1950s
1960s
1970s
1980s
1990s
2000s
0
20
40
60
Cumulative Heat (°C)

Supplementary Figure 115: Kernel Density Estimates (KDE) across Decades for Cumulative Heat (°C) in Historical CMIP6 multi-model ensemble (MME) (Tx99.0p) - Kernel Density Estimates (KDE) of Cumulative Heat (°C) for different Decades in the CMIP6 multi-model ensemble (MME). Area under each curve is normalized to 1. Cumulative Heat (°C) of the observed event is indicated by vertical lines in dark blue (Berkeley Earth) and light blue (ERA5).

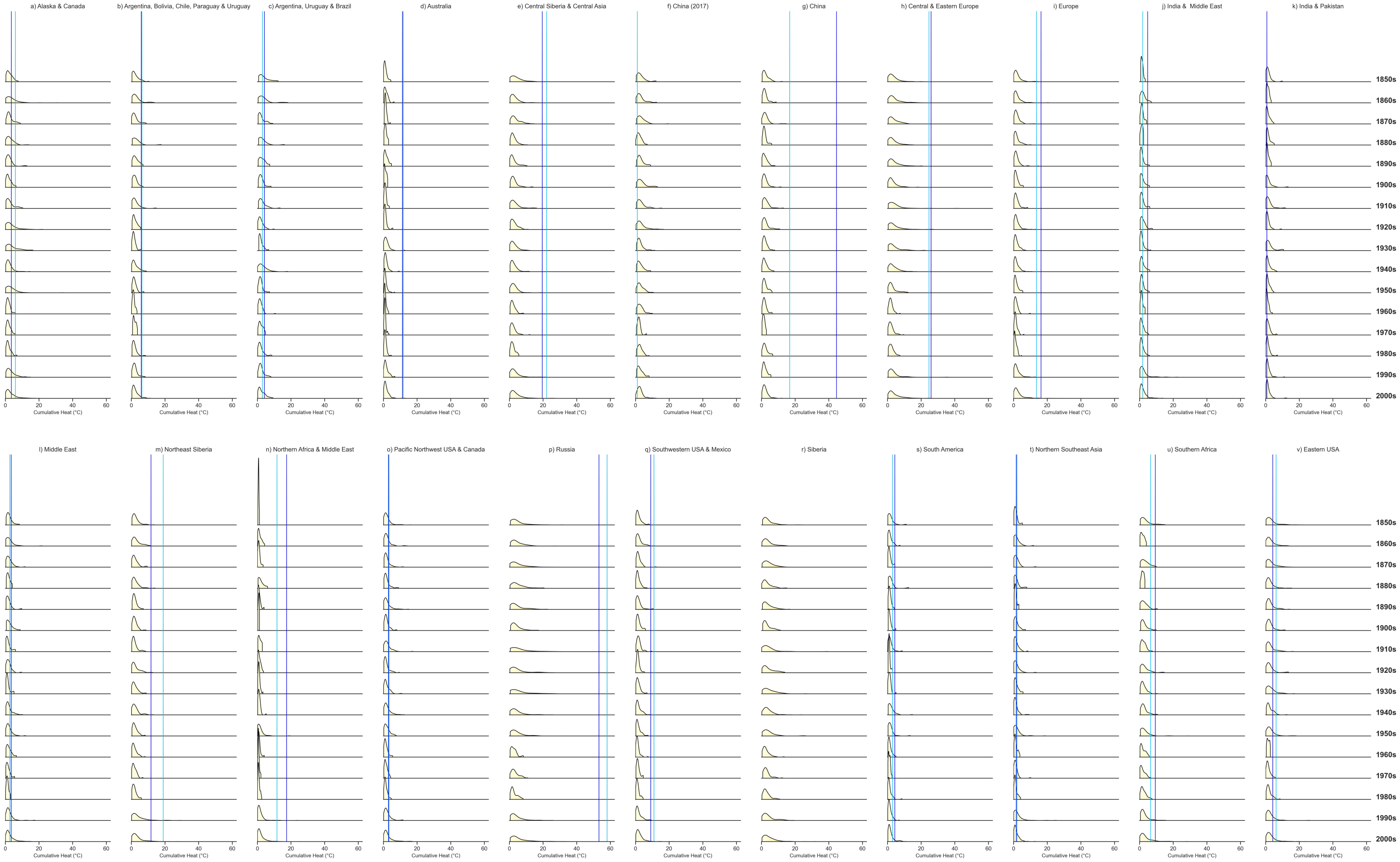
a) Alaska & Canada
b) Argentina, Bolivia, Chile, Paraguay & Uruguay
c) Argentina, Uruguay & Brazil
d) Australia
e) Central Siberia & Central Asia
f) China (2017)
g) China
h) Central & Eastern Europe
i) Europe
j) India & Middle East
k) India & Pakistan
l) Middle East
m) Northeast Siberia
n) Northern Africa & Middle East
o) Pacific Northwest USA & Canada
p) Russia
q) Southwestern USA & Mexico
r) Siberia
s) South America
t) Northern Southeast Asia
u) Southern Africa
v) Eastern USA
1850s
1860s
1870s
1880s
1890s
1900s
1910s
1920s
1930s
1940s
1950s
1960s
1970s
1980s
1990s
2000s
0
20
40
60
Cumulative Heat (°C)

Supplementary Figure 116: Kernel Density Estimates (KDE) across Decades for Cumulative Heat (°C) in SSP1-2.6 CMIP6 multi-model ensemble (MME) (Tx95.0p) - Kernel Density Estimates (KDE) of Cumulative Heat (°C) for different Decades in the CMIP6 multi-model ensemble (MME). Area under each curve is normalized to 1. Cumulative Heat (°C) of the observed event is indicated by vertical lines in dark blue (Berkeley Earth) and light blue (ERA5).

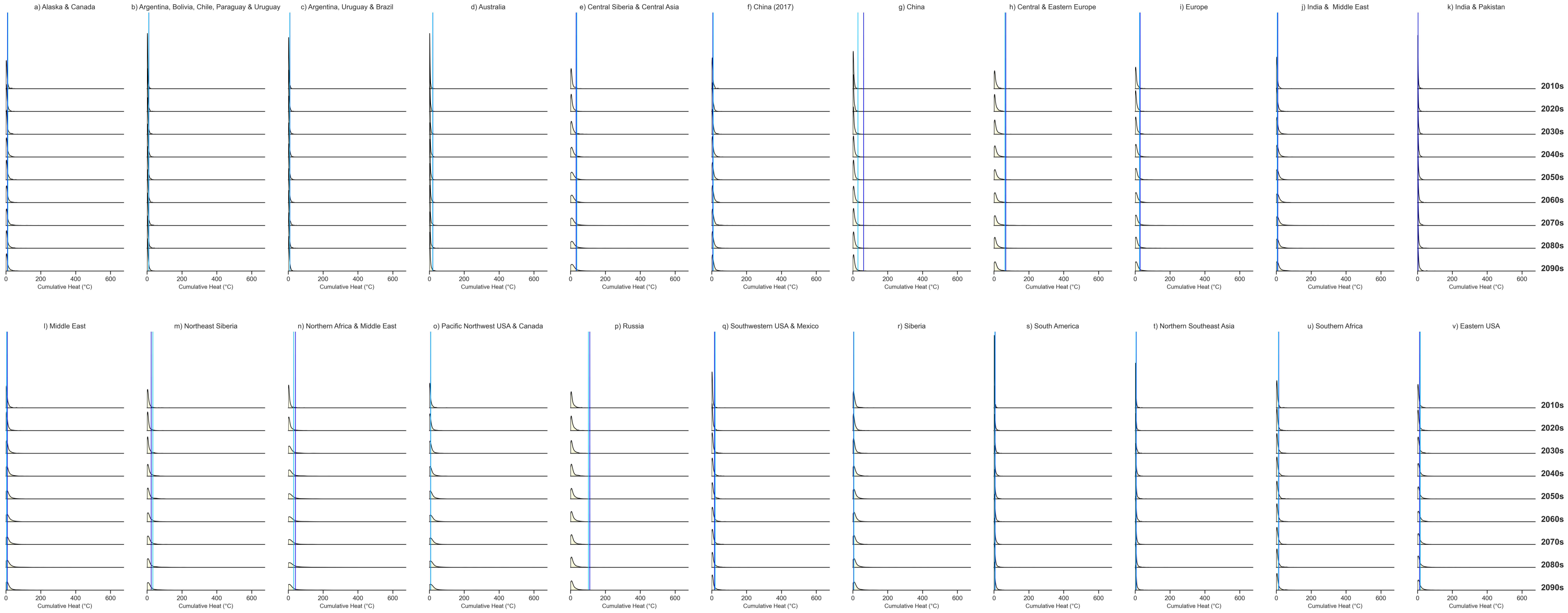

a) Alaska & Canada
b) Argentina, Bolivia, Chile, Paraguay & Uruguay
c) Argentina, Uruguay & Brazil
d) Australia
e) Central Siberia & Central Asia
f) China (2017)
g) China
h) Central & Eastern Europe
i) Europe
j) India & Middle East
k) India & Pakistan
l) Middle East
m) Northeast Siberia
n) Northern Africa & Middle East
o) Pacific Northwest USA & Canada
p) Russia
q) Southwestern USA & Mexico
r) Siberia
s) South America
t) Northern Southeast Asia
u) Southern Africa
v) Eastern USA
2010s
2020s
2030s
2040s
2050s
2060s
2070s
2080s
2090s
0
200
400
600
Cumulative Heat (°C)

Supplementary Figure 117: Kernel Density Estimates (KDE) across Decades for Cumulative Heat (°C) in SSP1-2.6 CMIP6 multi-model ensemble (MME) (Tx97.5p) - Kernel Density Estimates (KDE) of Cumulative Heat (°C) for different Decades in the CMIP6 multi-model ensemble (MME). Area under each curve is normalized to 1. Cumulative Heat (°C) of the observed event is indicated by vertical lines in dark blue (Berkeley Earth) and light blue (ERA5).

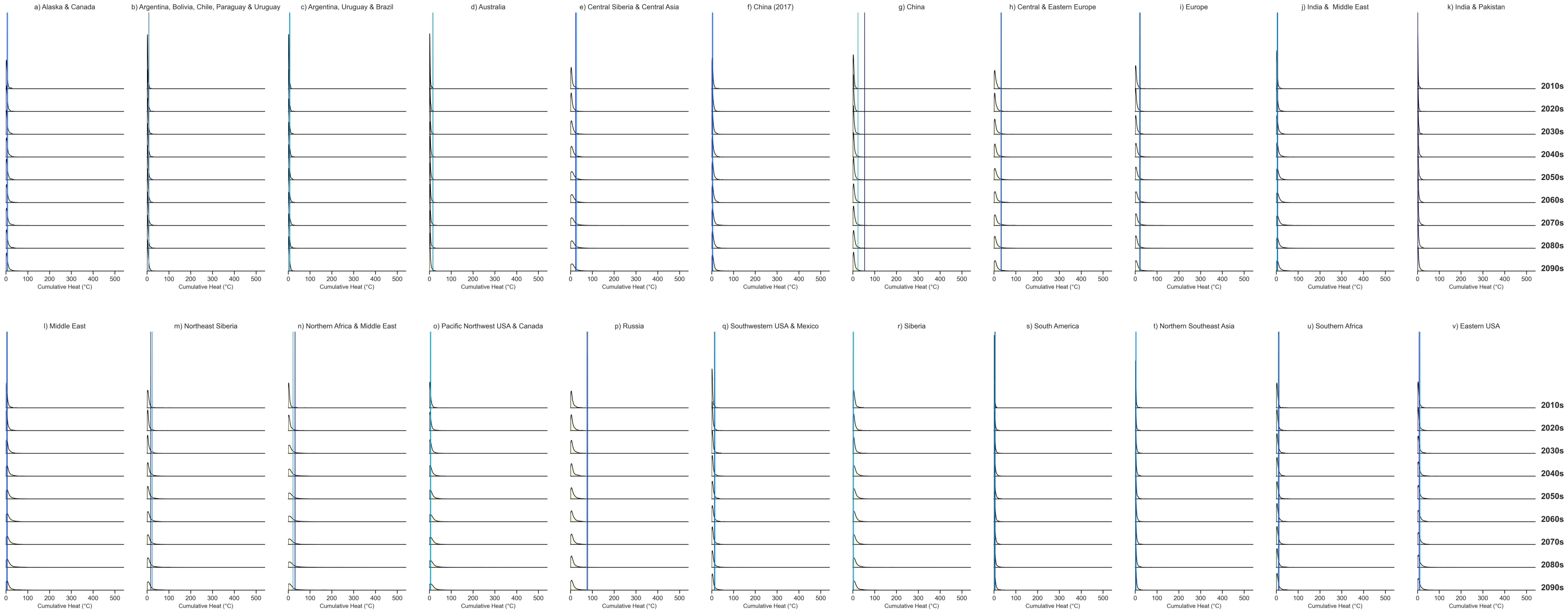

a) Alaska & Canada
b) Argentina, Bolivia, Chile, Paraguay & Uruguay
c) Argentina, Uruguay & Brazil
d) Australia
e) Central Siberia & Central Asia
f) China (2017)
g) China
h) Central & Eastern Europe
i) Europe
j) India & Middle East
k) India & Pakistan
l) Middle East
m) Northeast Siberia
n) Northern Africa & Middle East
o) Pacific Northwest USA & Canada
p) Russia
q) Southwestern USA & Mexico
r) Siberia
s) South America
t) Northern Southeast Asia
u) Southern Africa
v) Eastern USA
2010s
2020s
2030s
2040s
2050s
2060s
2070s
2080s
2090s
0 100 200 300 400 500
Cumulative Heat (°C)

Supplementary Figure 118: Kernel Density Estimates (KDE) across Decades for Cumulative Heat (°C) in SSP1-2.6 CMIP6 multi-model ensemble (MME) (Tx99.0p) - Kernel Density Estimates (KDE) of Cumulative Heat (°C) for different Decades in the CMIP6 multi-model ensemble (MME). Area under each curve is normalized to 1. Cumulative Heat (°C) of the observed event is indicated by vertical lines in dark blue (Berkeley Earth) and light blue (ERA5).

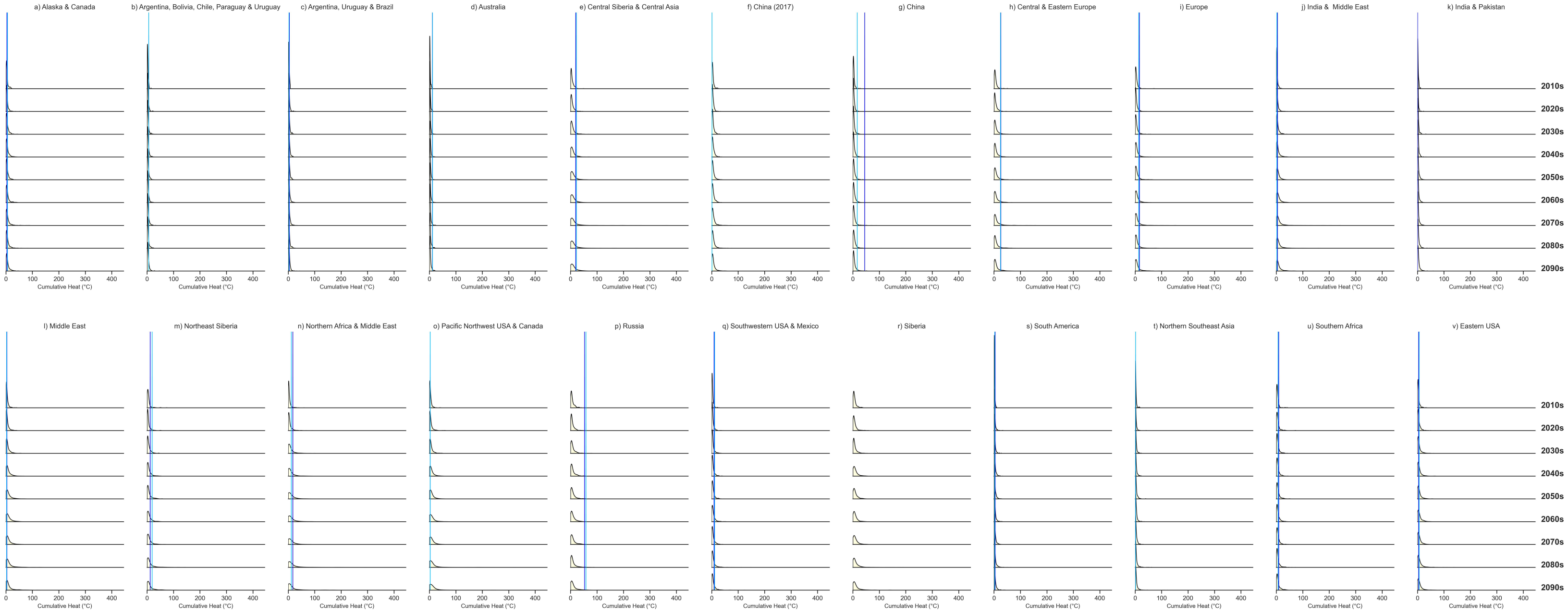
a) Alaska & Canada
b) Argentina, Bolivia, Chile, Paraguay & Uruguay
c) Argentina, Uruguay & Brazil
d) Australia
e) Central Siberia & Central Asia
f) China (2017)
g) China
h) Central & Eastern Europe
i) Europe
j) India & Middle East
k) India & Pakistan
l) Middle East
m) Northeast Siberia
n) Northern Africa & Middle East
o) Pacific Northwest USA & Canada
p) Russia
q) Southwestern USA & Mexico
r) Siberia
s) South America
t) Northern Southeast Asia
u) Southern Africa
v) Eastern USA
2010s
2020s
2030s
2040s
2050s
2060s
2070s
2080s
2090s
0
100
200
300
400
Cumulative Heat (°C)

Supplementary Figure 119: Kernel Density Estimates (KDE) across Decades for Cumulative Heat (°C) in SSP2-4.5 CMIP6 multi-model ensemble (MME) (Tx95.0p) - Kernel Density Estimates (KDE) of Cumulative Heat (°C) for different Decades in the CMIP6 multi-model ensemble (MME). Area under each curve is normalized to 1. Cumulative Heat (°C) of the observed event is indicated by vertical lines in dark blue (Berkeley Earth) and light blue (ERA5).

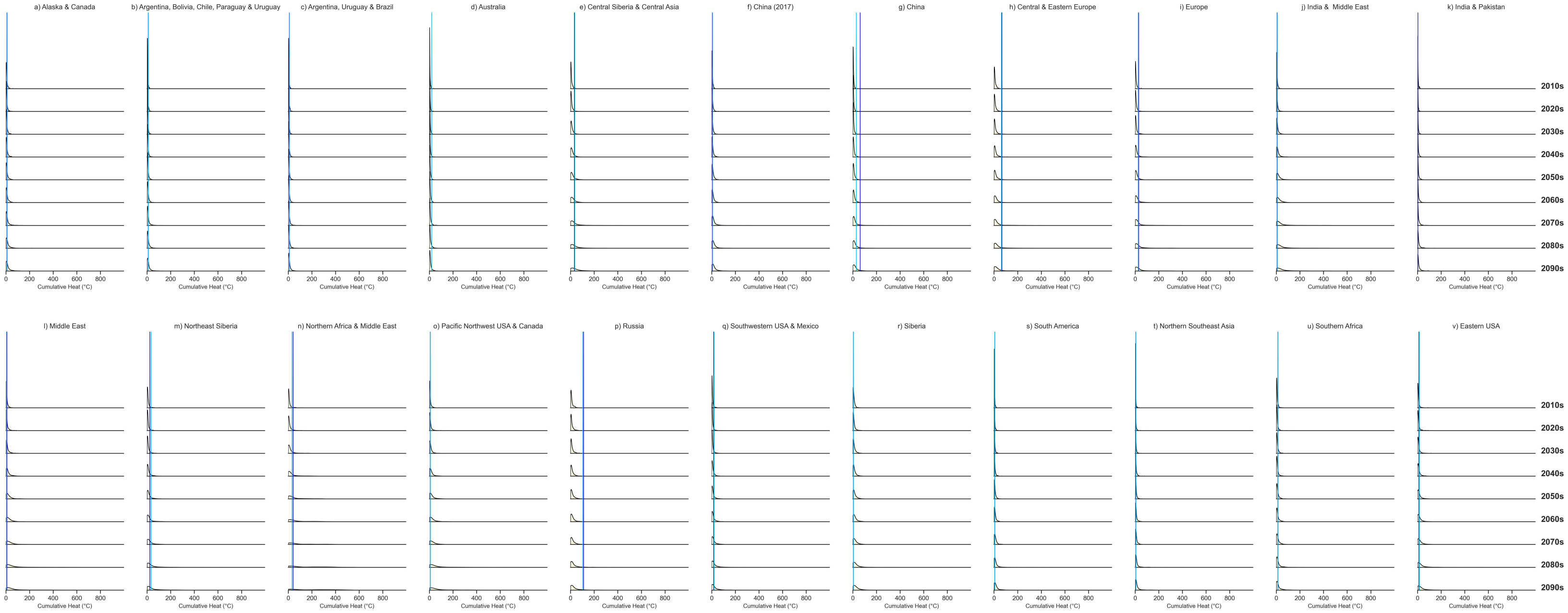

a) Alaska & Canada
b) Argentina, Bolivia, Chile, Paraguay & Uruguay
c) Argentina, Uruguay & Brazil
d) Australia
e) Central Siberia & Central Asia
f) China (2017)
g) China
h) Central & Eastern Europe
i) Europe
j) India & Middle East
k) India & Pakistan
l) Middle East
m) Northeast Siberia
n) Northern Africa & Middle East
o) Pacific Northwest USA & Canada
p) Russia
q) Southwestern USA & Mexico
r) Siberia
s) South America
t) Northern Southeast Asia
u) Southern Africa
v) Eastern USA
2010s
2020s
2030s
2040s
2050s
2060s
2070s
2080s
2090s
0
200
400
600
800
Cumulative Heat (°C)

Supplementary Figure 120: Kernel Density Estimates (KDE) across Decades for Cumulative Heat (°C) in SSP2-4.5 CMIP6 multi-model ensemble (MME) (Tx97.5p) - Kernel Density Estimates (KDE) of Cumulative Heat (°C) for different Decades in the CMIP6 multi-model ensemble (MME). Area under each curve is normalized to 1. Cumulative Heat (°C) of the observed event is indicated by vertical lines in dark blue (Berkeley Earth) and light blue (ERA5).

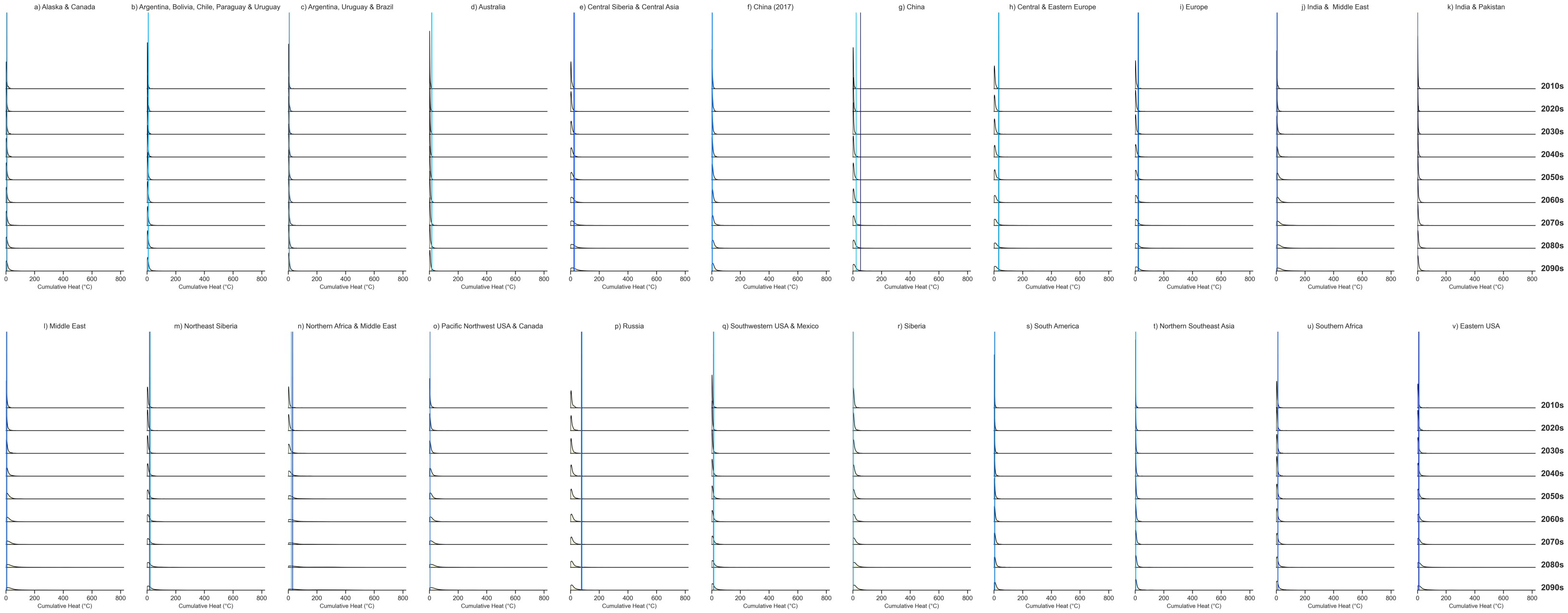

a) Alaska & Canada
b) Argentina, Bolivia, Chile, Paraguay & Uruguay
c) Argentina, Uruguay & Brazil
d) Australia
e) Central Siberia & Central Asia
f) China (2017)
g) China
h) Central & Eastern Europe
i) Europe
j) India & Middle East
k) India & Pakistan
l) Middle East
m) Northeast Siberia
n) Northern Africa & Middle East
o) Pacific Northwest USA & Canada
p) Russia
q) Southwestern USA & Mexico
r) Siberia
s) South America
t) Northern Southeast Asia
u) Southern Africa
v) Eastern USA
2010s
2020s
2030s
2040s
2050s
2060s
2070s
2080s
2090s
0
200
400
600
800
Cumulative Heat (°C)

Supplementary Figure 121: Kernel Density Estimates (KDE) across Decades for Cumulative Heat (°C) in SSP2-4.5 CMIP6 multi-model ensemble (MME) (Tx99.0p) - Kernel Density Estimates (KDE) of Cumulative Heat (°C) for different Decades in the CMIP6 multi-model ensemble (MME). Area under each curve is normalized to 1. Cumulative Heat (°C) of the observed event is indicated by vertical lines in dark blue (Berkeley Earth) and light blue (ERA5).

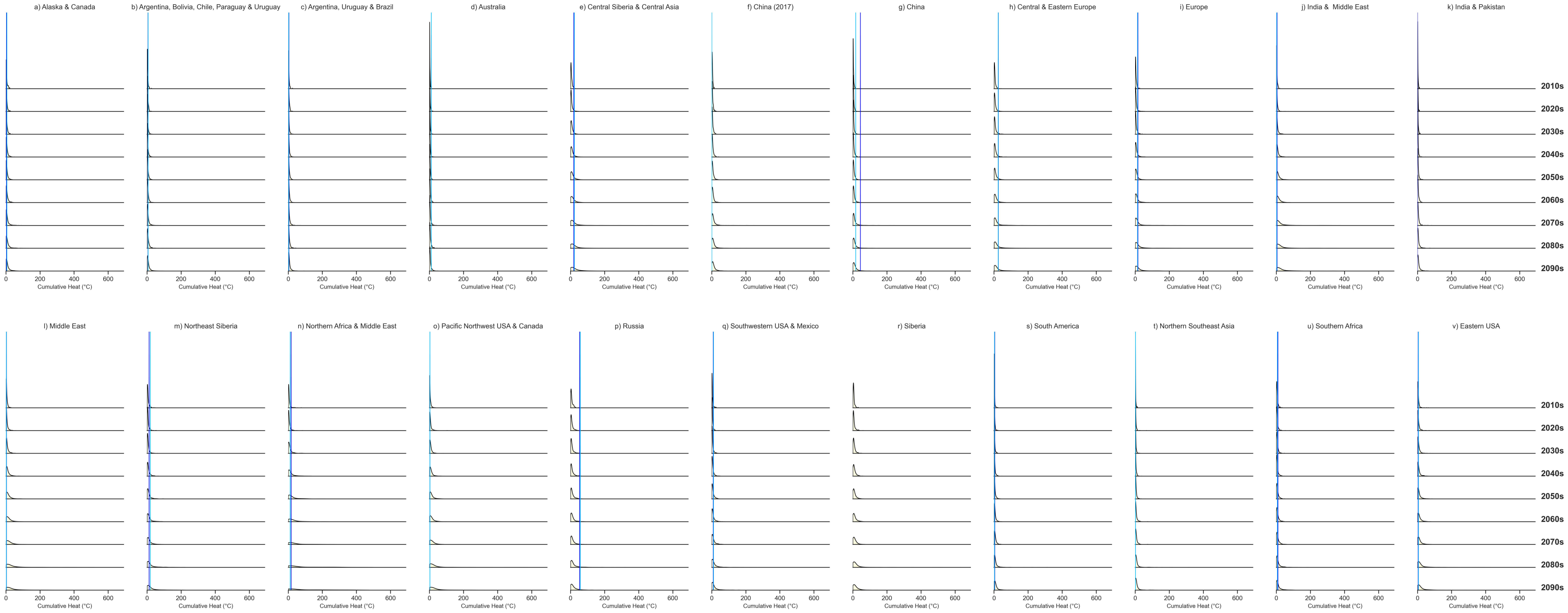

a) Alaska & Canada
b) Argentina, Bolivia, Chile, Paraguay & Uruguay
c) Argentina, Uruguay & Brazil
d) Australia
e) Central Siberia & Central Asia
f) China (2017)
g) China
h) Central & Eastern Europe
i) Europe
j) India & Middle East
k) India & Pakistan
l) Middle East
m) Northeast Siberia
n) Northern Africa & Middle East
o) Pacific Northwest USA & Canada
p) Russia
q) Southwestern USA & Mexico
r) Siberia
s) South America
t) Northern Southeast Asia
u) Southern Africa
v) Eastern USA
2010s
2020s
2030s
2040s
2050s
2060s
2070s
2080s
2090s
0
200
400
600
Cumulative Heat (°C)

Supplementary Figure 122: Kernel Density Estimates (KDE) across Decades for Cumulative Heat (°C) in SSP3-7.0 CMIP6 multi-model ensemble (MME) (Tx95.0p) - Kernel Density Estimates (KDE) of Cumulative Heat (°C) for different Decades in the CMIP6 multi-model ensemble (MME). Area under each curve is normalized to 1. Cumulative Heat (°C) of the observed event is indicated by vertical lines in dark blue (Berkeley Earth) and light blue (ERA5).

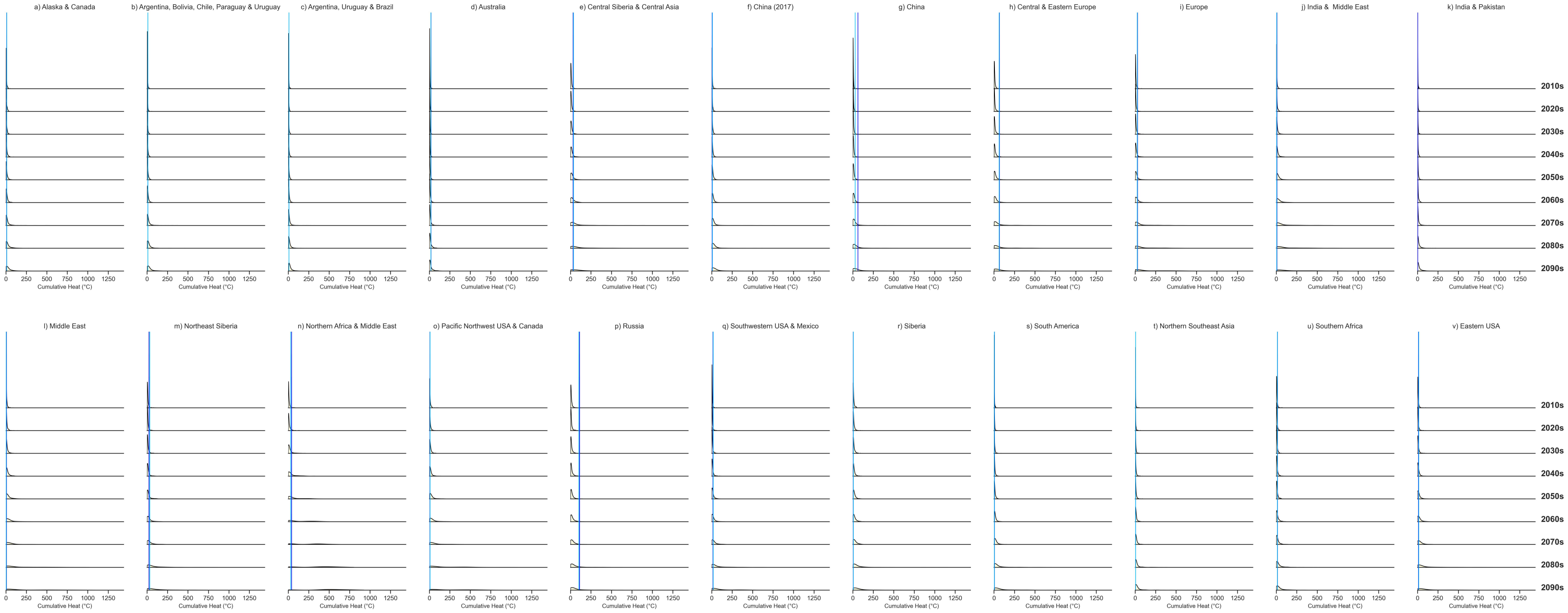

a) Alaska & Canada
b) Argentina, Bolivia, Chile, Paraguay & Uruguay
c) Argentina, Uruguay & Brazil
d) Australia
e) Central Siberia & Central Asia
f) China (2017)
g) China
h) Central & Eastern Europe
i) Europe
j) India & Middle East
k) India & Pakistan
l) Middle East
m) Northeast Siberia
n) Northern Africa & Middle East
o) Pacific Northwest USA & Canada
p) Russia
q) Southwestern USA & Mexico
r) Siberia
s) South America
t) Northern Southeast Asia
u) Southern Africa
v) Eastern USA
2010s
2020s
2030s
2040s
2050s
2060s
2070s
2080s
2090s
0 250 500 750 1000 1250
Cumulative Heat (°C)

Supplementary Figure 123: Kernel Density Estimates (KDE) across Decades for Cumulative Heat (°C) in SSP3-7.0 CMIP6 multi-model ensemble (MME) (Tx97.5p) - Kernel Density Estimates (KDE) of Cumulative Heat (°C) for different Decades in the CMIP6 multi-model ensemble (MME). Area under each curve is normalized to 1. Cumulative Heat (°C) of the observed event is indicated by vertical lines in dark blue (Berkeley Earth) and light blue (ERA5).

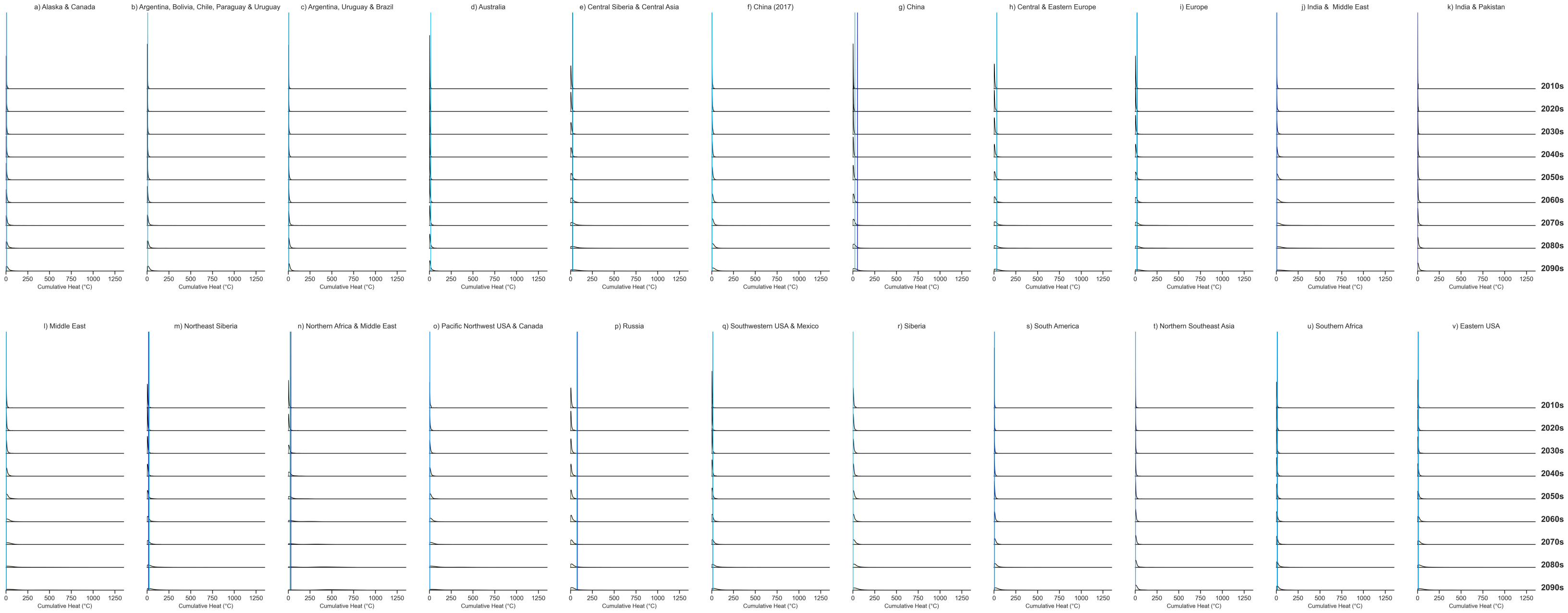

a) Alaska & Canada
b) Argentina, Bolivia, Chile, Paraguay & Uruguay
c) Argentina, Uruguay & Brazil
d) Australia
e) Central Siberia & Central Asia
f) China (2017)
g) China
h) Central & Eastern Europe
i) Europe
j) India & Middle East
k) India & Pakistan
l) Middle East
m) Northeast Siberia
n) Northern Africa & Middle East
o) Pacific Northwest USA & Canada
p) Russia
q) Southwestern USA & Mexico
r) Siberia
s) South America
t) Northern Southeast Asia
u) Southern Africa
v) Eastern USA
2010s
2020s
2030s
2040s
2050s
2060s
2070s
2080s
2090s
0 250 500 750 1000 1250
Cumulative Heat (°C)

Supplementary Figure 124: Kernel Density Estimates (KDE) across Decades for Cumulative Heat (°C) in SSP3-7.0 CMIP6 multi-model ensemble (MME) (Tx99.0p) - Kernel Density Estimates (KDE) of Cumulative Heat (°C) for different Decades in the CMIP6 multi-model ensemble (MME). Area under each curve is normalized to 1. Cumulative Heat (°C) of the observed event is indicated by vertical lines in dark blue (Berkeley Earth) and light blue (ERA5).

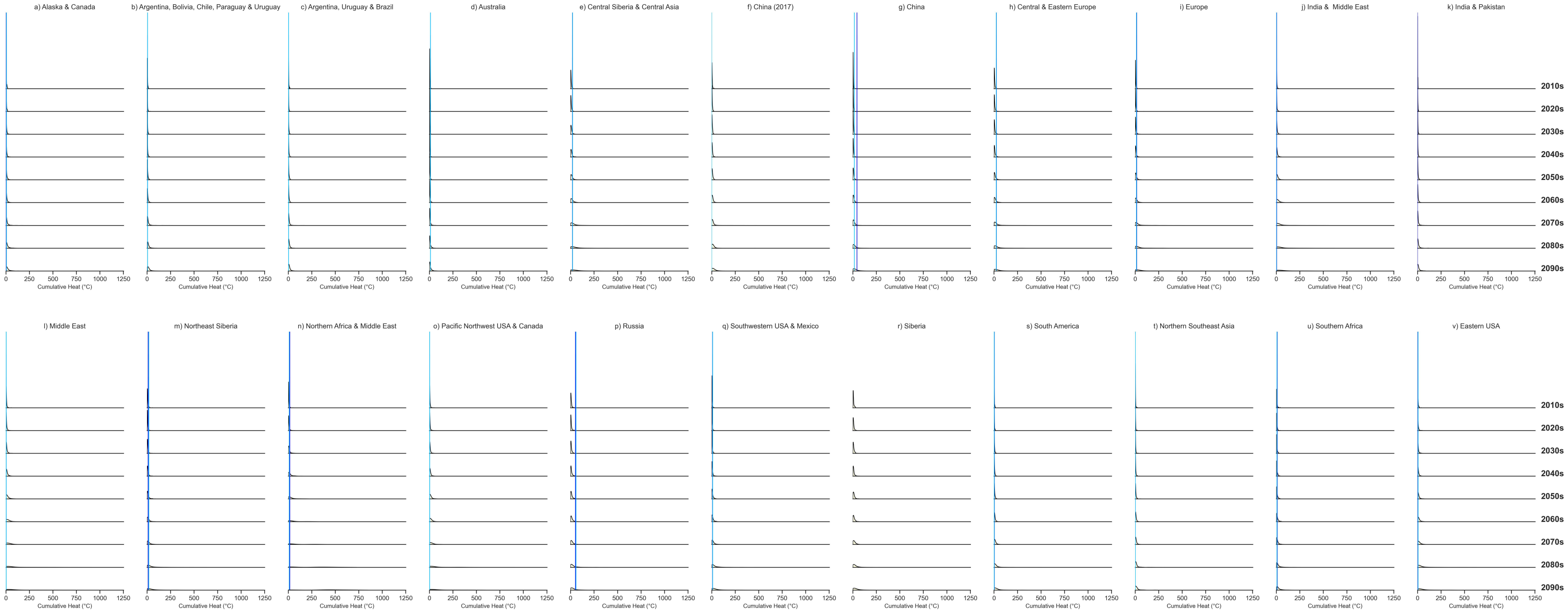
a) Alaska & Canada
b) Argentina, Bolivia, Chile, Paraguay & Uruguay
c) Argentina, Uruguay & Brazil
d) Australia
e) Central Siberia & Central Asia
f) China (2017)
g) China
h) Central & Eastern Europe
i) Europe
j) India & Middle East
k) India & Pakistan
l) Middle East
m) Northeast Siberia
n) Northern Africa & Middle East
o) Pacific Northwest USA & Canada
p) Russia
q) Southwestern USA & Mexico
r) Siberia
s) South America
t) Northern Southeast Asia
u) Southern Africa
v) Eastern USA
2010s
2020s
2030s
2040s
2050s
2060s
2070s
2080s
2090s
0
250
500
750
1000
1250
Cumulative Heat (°C)

Supplementary Figure 125: Kernel Density Estimates (KDE) across Decades for Cumulative Heat (°C) in SSP5-8.5 CMIP6 multi-model ensemble (MME) (Tx95.0p) - Kernel Density Estimates (KDE) of Cumulative Heat (°C) for different Decades in the CMIP6 multi-model ensemble (MME). Area under each curve is normalized to 1. Cumulative Heat (°C) of the observed event is indicated by vertical lines in dark blue (Berkeley Earth) and light blue (ERA5).

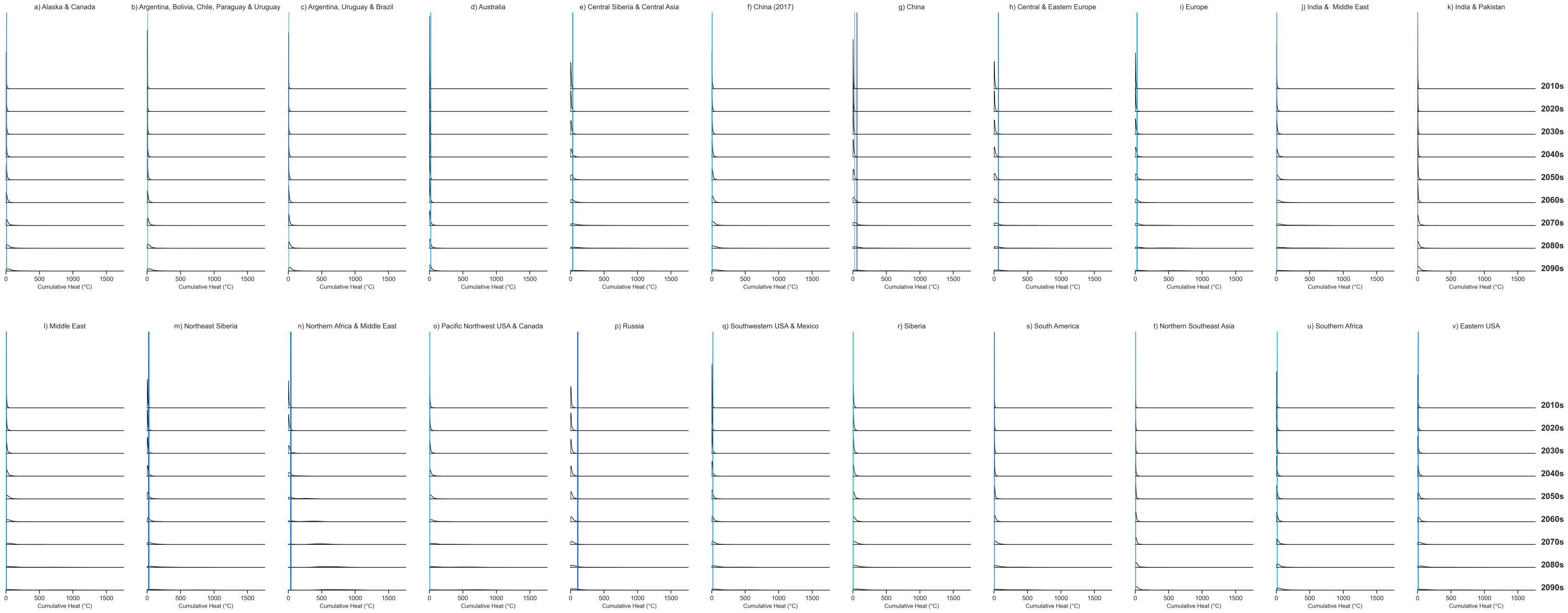
a) Alaska & Canada
b) Argentina, Bolivia, Chile, Paraguay & Uruguay
c) Argentina, Uruguay & Brazil
d) Australia
e) Central Siberia & Central Asia
f) China (2017)
g) China
h) Central & Eastern Europe
i) Europe
j) India & Middle East
k) India & Pakistan
l) Middle East
m) Northeast Siberia
n) Northern Africa & Middle East
o) Pacific Northwest USA & Canada
p) Russia
q) Southwestern USA & Mexico
r) Siberia
s) South America
t) Northern Southeast Asia
u) Southern Africa
v) Eastern USA
2010s
2020s
2030s
2040s
2050s
2060s
2070s
2080s
2090s
0
500
1000
1500
Cumulative Heat (°C)

Supplementary Figure 126: Kernel Density Estimates (KDE) across Decades for Cumulative Heat (°C) in SSP5-8.5 CMIP6 multi-model ensemble (MME) (Tx97.5p) - Kernel Density Estimates (KDE) of Cumulative Heat (°C) for different Decades in the CMIP6 multi-model ensemble (MME). Area under each curve is normalized to 1. Cumulative Heat (°C) of the observed event is indicated by vertical lines in dark blue (Berkeley Earth) and light blue (ERA5).

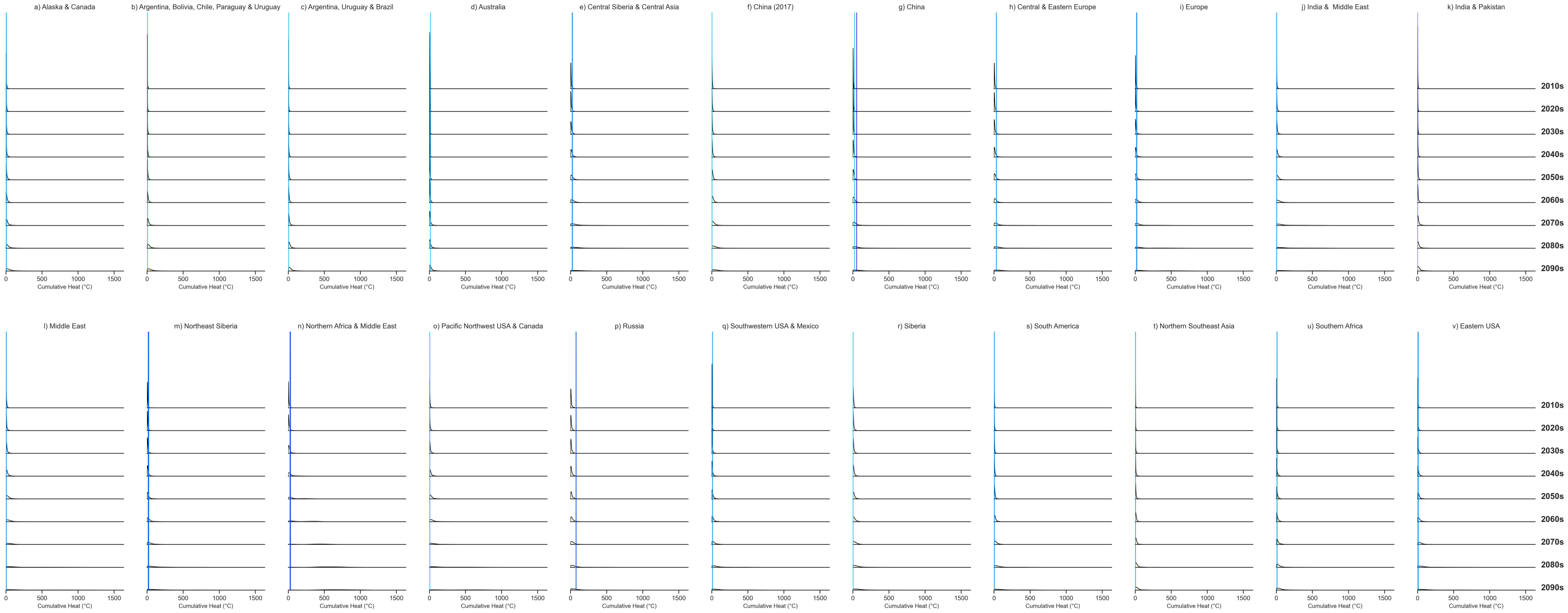

a) Alaska & Canada
b) Argentina, Bolivia, Chile, Paraguay & Uruguay
c) Argentina, Uruguay & Brazil
d) Australia
e) Central Siberia & Central Asia
f) China (2017)
g) China
h) Central & Eastern Europe
i) Europe
j) India & Middle East
k) India & Pakistan
l) Middle East
m) Northeast Siberia
n) Northern Africa & Middle East
o) Pacific Northwest USA & Canada
p) Russia
q) Southwestern USA & Mexico
r) Siberia
s) South America
t) Northern Southeast Asia
u) Southern Africa
v) Eastern USA
2010s
2020s
2030s
2040s
2050s
2060s
2070s
2080s
2090s
0
500
1000
1500
Cumulative Heat (°C)

Supplementary Figure 127: Kernel Density Estimates (KDE) across Decades for Cumulative Heat (°C) in SSP5-8.5 CMIP6 multi-model ensemble (MME) (Tx99.0p) - Kernel Density Estimates (KDE) of Cumulative Heat (°C) for different Decades in the CMIP6 multi-model ensemble (MME). Area under each curve is normalized to 1. Cumulative Heat (°C) of the observed event is indicated by vertical lines in dark blue (Berkeley Earth) and light blue (ERA5).

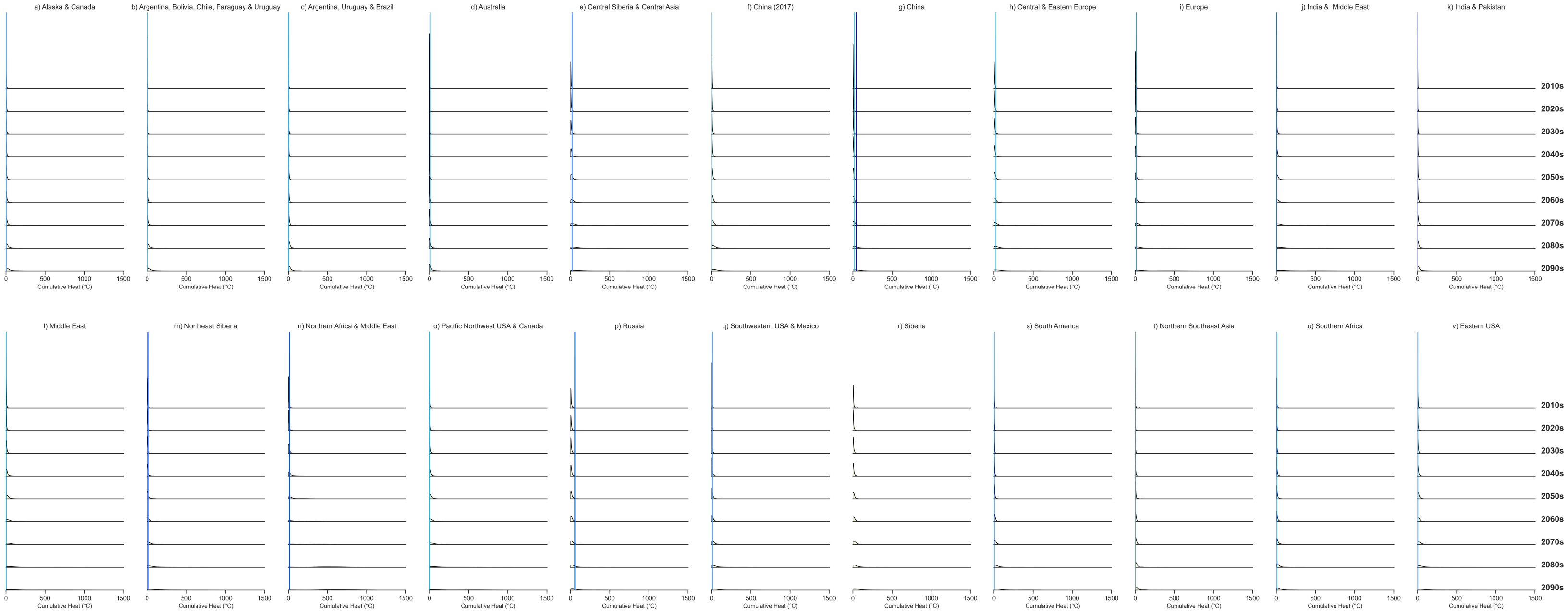

a) Alaska & Canada
b) Argentina, Bolivia, Chile, Paraguay & Uruguay
c) Argentina, Uruguay & Brazil
d) Australia
e) Central Siberia & Central Asia
f) China (2017)
g) China
h) Central & Eastern Europe
i) Europe
j) India & Middle East
k) India & Pakistan
l) Middle East
m) Northeast Siberia
n) Northern Africa & Middle East
o) Pacific Northwest USA & Canada
p) Russia
q) Southwestern USA & Mexico
r) Siberia
s) South America
t) Northern Southeast Asia
u) Southern Africa
v) Eastern USA
2010s
2020s
2030s
2040s
2050s
2060s
2070s
2080s
2090s
0
500
1000
1500
Cumulative Heat (°C)

Supplementary Figure 128: Kernel Density Estimates (KDE) across Decades for Heatwave Length (days) in Historical CMIP6 multi-model ensemble (MME) (Tx95.0p) - Kernel Density Estimates (KDE) of Heatwave Length (days) for different Decades in the CMIP6 multi-model ensemble (MME). Area under each curve is normalized to 1. Heatwave Length (days) of the observed event is indicated by vertical lines in dark blue (Berkeley Earth) and light blue (ERA5).

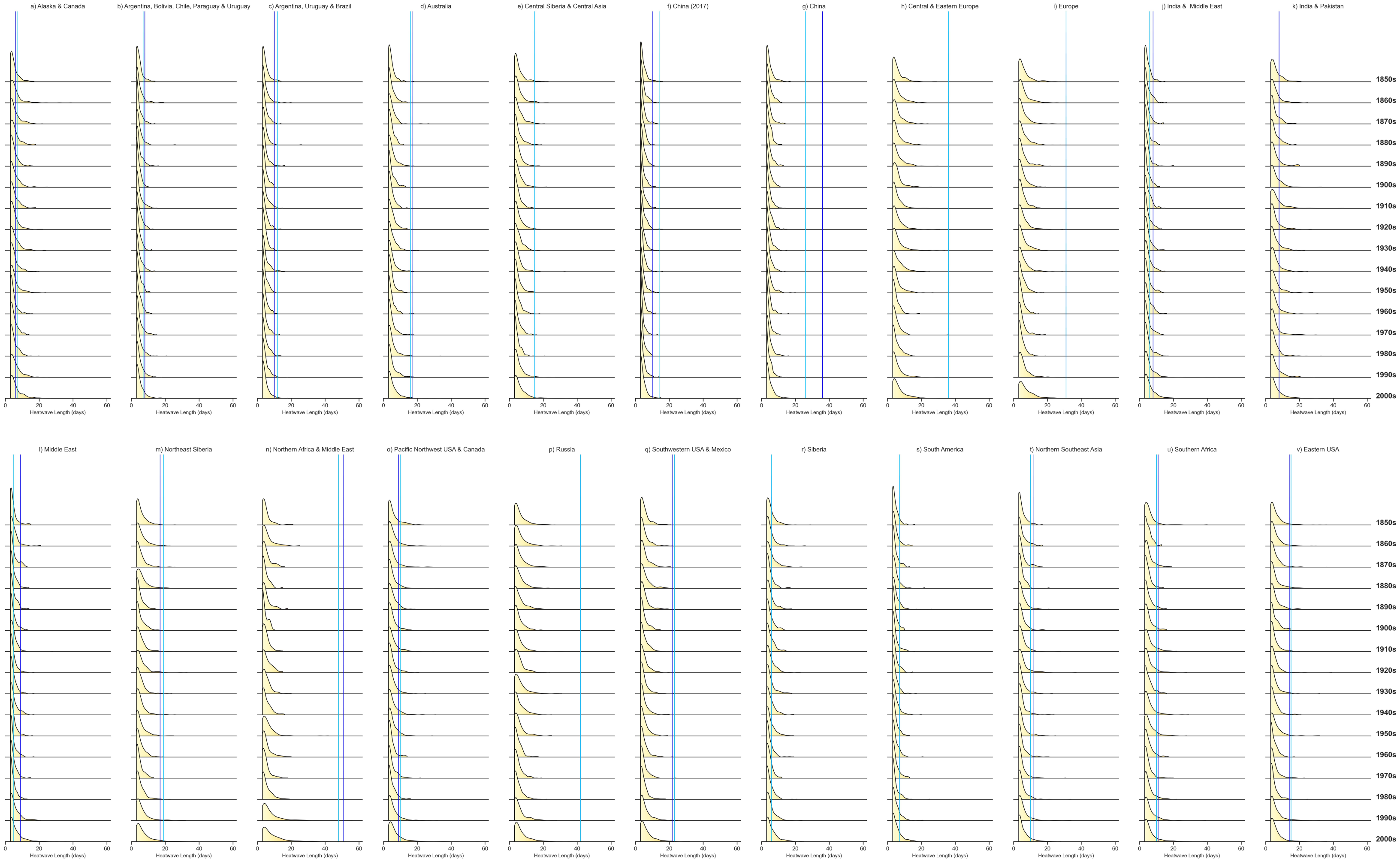
a) Alaska & Canada
b) Argentina, Bolivia, Chile, Paraguay & Uruguay
c) Argentina, Uruguay & Brazil
d) Australia
e) Central Siberia & Central Asia
f) China (2017)
g) China
h) Central & Eastern Europe
i) Europe
j) India & Middle East
k) India & Pakistan
l) Middle East
m) Northeast Siberia
n) Northern Africa & Middle East
o) Pacific Northwest USA & Canada
p) Russia
q) Southwestern USA & Mexico
r) Siberia
s) South America
t) Northern Southeast Asia
u) Southern Africa
v) Eastern USA
1850s
1860s
1870s
1880s
1890s
1900s
1910s
1920s
1930s
1940s
1950s
1960s
1970s
1980s
1990s
2000s
0
20
40
60
Heatwave Length (days)

Supplementary Figure 129: Kernel Density Estimates (KDE) across Decades for Heatwave Length (days) in Historical CMIP6 multi-model ensemble (MME) (Tx97.5p) - Kernel Density Estimates (KDE) of Heatwave Length (days) for different Decades in the CMIP6 multi-model ensemble (MME). Area under each curve is normalized to 1. Heatwave Length (days) of the observed event is indicated by vertical lines in dark blue (Berkeley Earth) and light blue (ERA5).

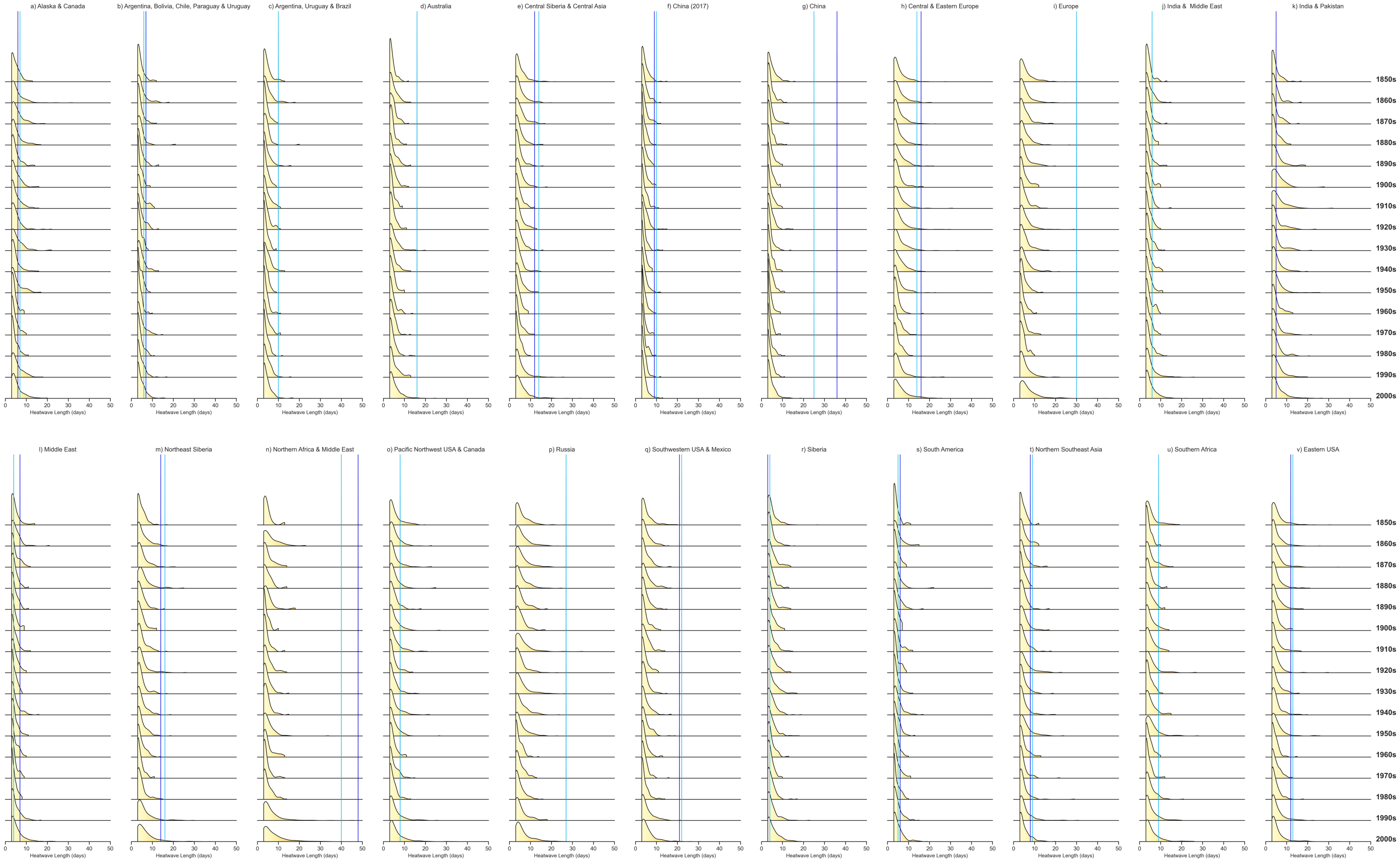
a) Alaska & Canada
b) Argentina, Bolivia, Chile, Paraguay & Uruguay
c) Argentina, Uruguay & Brazil
d) Australia
e) Central Siberia & Central Asia
f) China (2017)
g) China
h) Central & Eastern Europe
i) Europe
j) India & Middle East
k) India & Pakistan
l) Middle East
m) Northeast Siberia
n) Northern Africa & Middle East
o) Pacific Northwest USA & Canada
p) Russia
q) Southwestern USA & Mexico
r) Siberia
s) South America
t) Northern Southeast Asia
u) Southern Africa
v) Eastern USA
1850s
1860s
1870s
1880s
1890s
1900s
1910s
1920s
1930s
1940s
1950s
1960s
1970s
1980s
1990s
2000s
0 10 20 30 40 50
Heatwave Length (days)

Supplementary Figure 130: Kernel Density Estimates (KDE) across Decades for Heatwave Length (days) in Historical CMIP6 multi-model ensemble (MME) (Tx99.0p) - Kernel Density Estimates (KDE) of Heatwave Length (days) for different Decades in the CMIP6 multi-model ensemble (MME). Area under each curve is normalized to 1. Heatwave Length (days) of the observed event is indicated by vertical lines in dark blue (Berkeley Earth) and light blue (ERA5).

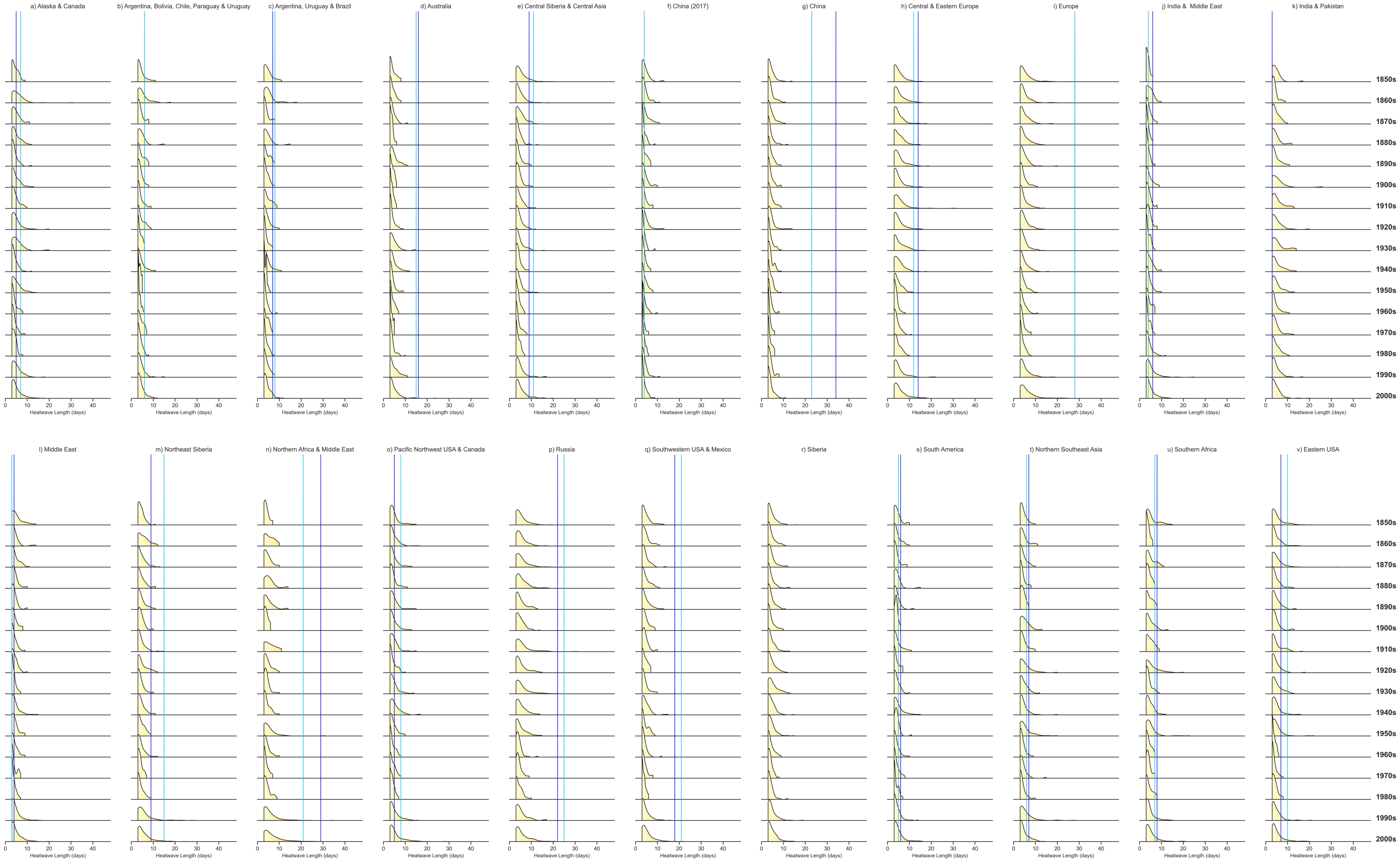

a) Alaska & Canada
b) Argentina, Bolivia, Chile, Paraguay & Uruguay
c) Argentina, Uruguay & Brazil
d) Australia
e) Central Siberia & Central Asia
f) China (2017)
g) China
h) Central & Eastern Europe
i) Europe
j) India & Middle East
k) India & Pakistan
l) Middle East
m) Northeast Siberia
n) Northern Africa & Middle East
o) Pacific Northwest USA & Canada
p) Russia
q) Southwestern USA & Mexico
r) Siberia
s) South America
t) Northern Southeast Asia
u) Southern Africa
v) Eastern USA
1850s
1860s
1870s
1880s
1890s
1900s
1910s
1920s
1930s
1940s
1950s
1960s
1970s
1980s
1990s
2000s
0 10 20 30 40
Heatwave Length (days)

Supplementary Figure 131: Kernel Density Estimates (KDE) across Decades for Heatwave Length (days) in SSP1-2.6 CMIP6 multi-model ensemble (MME) (Tx95.0p) - Kernel Density Estimates (KDE) of Heatwave Length (days) for different Decades in the CMIP6 multi-model ensemble (MME). Area under each curve is normalized to 1. Heatwave Length (days) of the observed event is indicated by vertical lines in dark blue (Berkeley Earth) and light blue (ERA5).

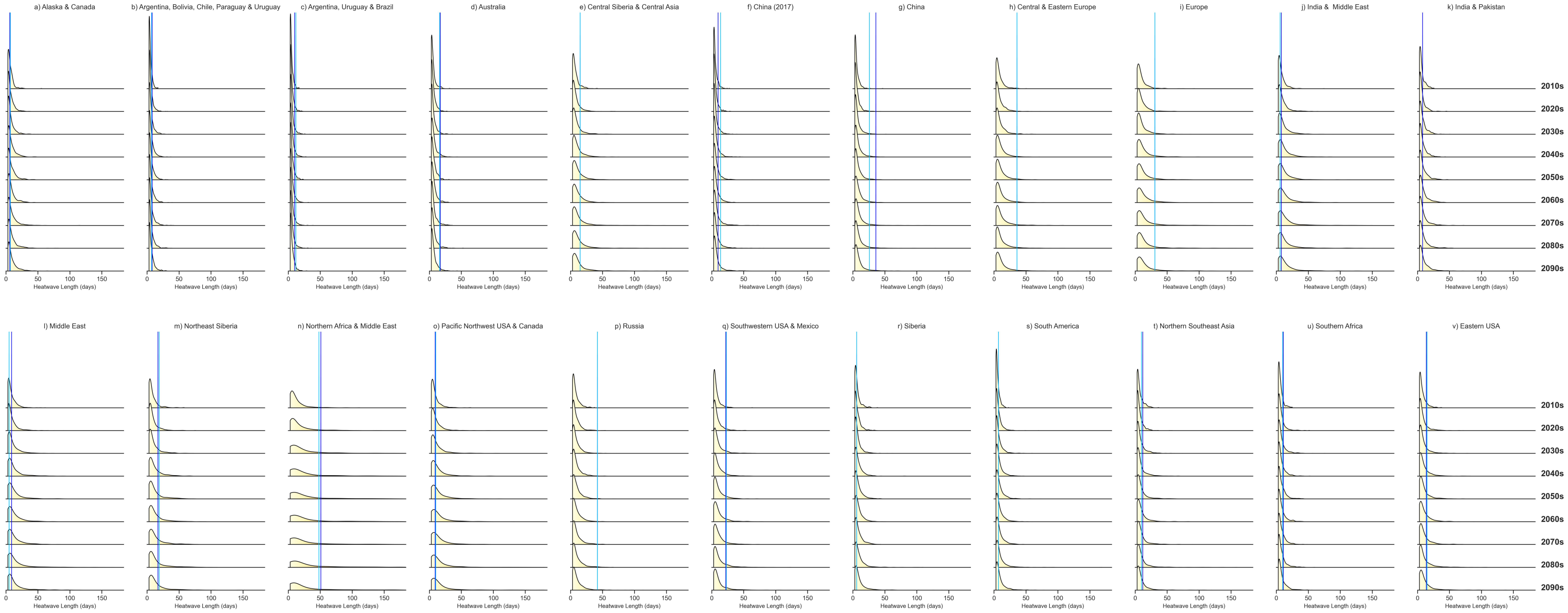

a) Alaska & Canada
b) Argentina, Bolivia, Chile, Paraguay & Uruguay
c) Argentina, Uruguay & Brazil
d) Australia
e) Central Siberia & Central Asia
f) China (2017)
g) China
h) Central & Eastern Europe
i) Europe
j) India & Middle East
k) India & Pakistan
l) Middle East
m) Northeast Siberia
n) Northern Africa & Middle East
o) Pacific Northwest USA & Canada
p) Russia
q) Southwestern USA & Mexico
r) Siberia
s) South America
t) Northern Southeast Asia
u) Southern Africa
v) Eastern USA
2010s
2020s
2030s
2040s
2050s
2060s
2070s
2080s
2090s
0
50
100
150
Heatwave Length (days)

Supplementary Figure 132: Kernel Density Estimates (KDE) across Decades for Heatwave Length (days) in SSP1-2.6 CMIP6 multi-model ensemble (MME) (Tx97.5p) - Kernel Density Estimates (KDE) of Heatwave Length (days) for different Decades in the CMIP6 multi-model ensemble (MME). Area under each curve is normalized to 1. Heatwave Length (days) of the observed event is indicated by vertical lines in dark blue (Berkeley Earth) and light blue (ERA5).

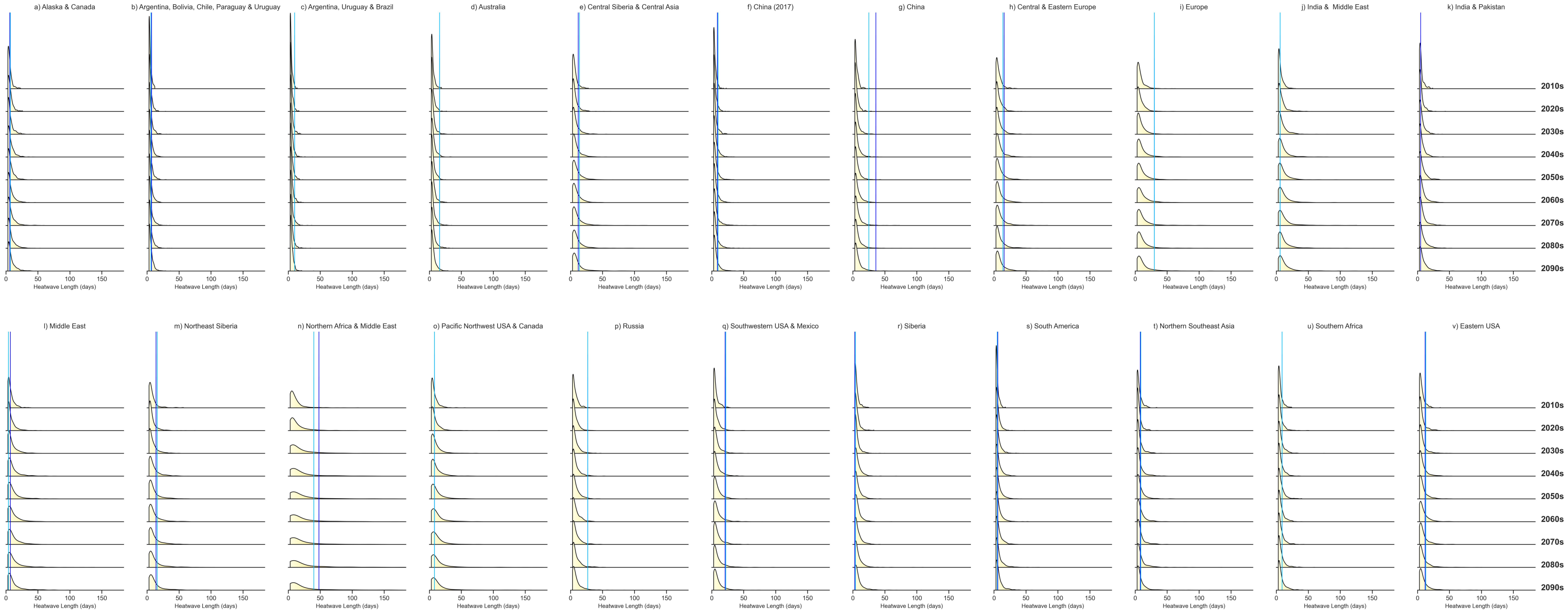
a) Alaska & Canada
b) Argentina, Bolivia, Chile, Paraguay & Uruguay
c) Argentina, Uruguay & Brazil
d) Australia
e) Central Siberia & Central Asia
f) China (2017)
g) China
h) Central & Eastern Europe
i) Europe
j) India & Middle East
k) India & Pakistan
l) Middle East
m) Northeast Siberia
n) Northern Africa & Middle East
o) Pacific Northwest USA & Canada
p) Russia
q) Southwestern USA & Mexico
r) Siberia
s) South America
t) Northern Southeast Asia
u) Southern Africa
v) Eastern USA
2010s
2020s
2030s
2040s
2050s
2060s
2070s
2080s
2090s
0
50
100
150
Heatwave Length (days)

Supplementary Figure 133: Kernel Density Estimates (KDE) across Decades for Heatwave Length (days) in SSP1-2.6 CMIP6 multi-model ensemble (MME) (Tx99.0p) - Kernel Density Estimates (KDE) of Heatwave Length (days) for different Decades in the CMIP6 multi-model ensemble (MME). Area under each curve is normalized to 1. Heatwave Length (days) of the observed event is indicated by vertical lines in dark blue (Berkeley Earth) and light blue (ERA5).

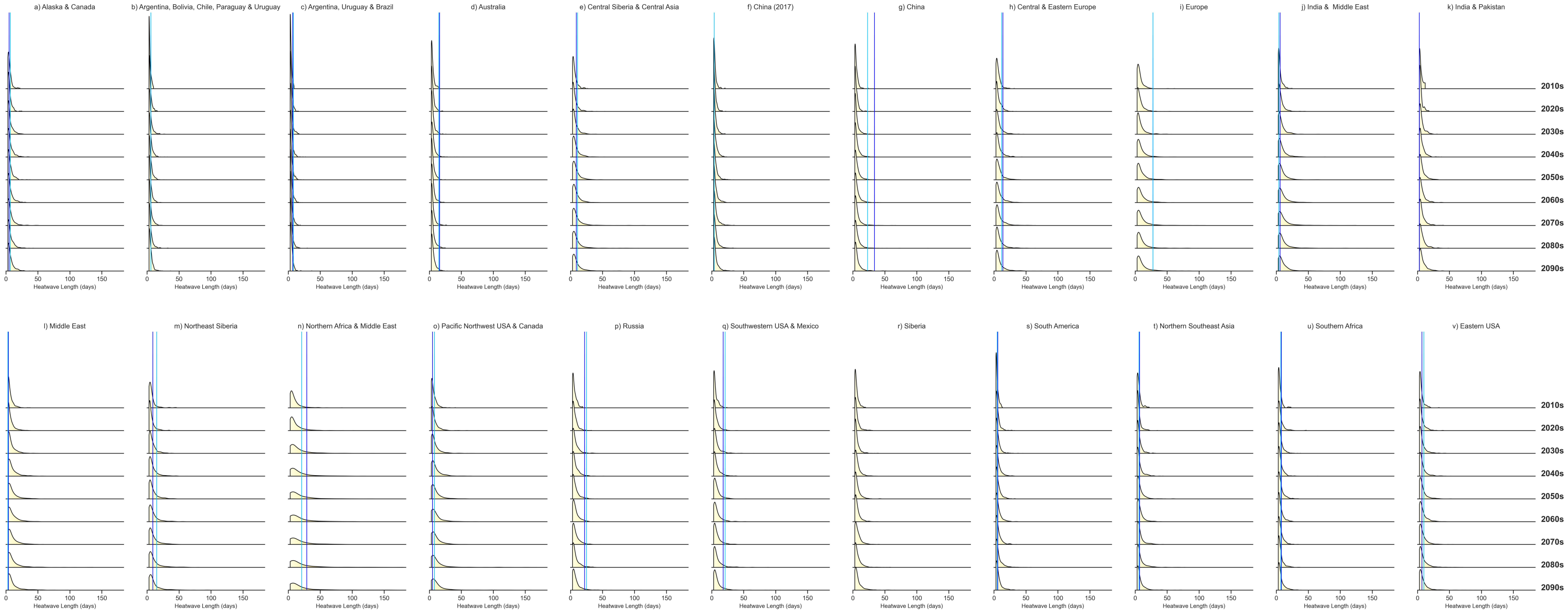
a) Alaska & Canada
b) Argentina, Bolivia, Chile, Paraguay & Uruguay
c) Argentina, Uruguay & Brazil
d) Australia
e) Central Siberia & Central Asia
f) China (2017)
g) China
h) Central & Eastern Europe
i) Europe
j) India & Middle East
k) India & Pakistan
l) Middle East
m) Northeast Siberia
n) Northern Africa & Middle East
o) Pacific Northwest USA & Canada
p) Russia
q) Southwestern USA & Mexico
r) Siberia
s) South America
t) Northern Southeast Asia
u) Southern Africa
v) Eastern USA
2010s
2020s
2030s
2040s
2050s
2060s
2070s
2080s
2090s
0
50
100
150
Heatwave Length (days)

Supplementary Figure 134: Kernel Density Estimates (KDE) across Decades for Heatwave Length (days) in SSP2-4.5 CMIP6 multi-model ensemble (MME) (Tx95.0p) - Kernel Density Estimates (KDE) of Heatwave Length (days) for different Decades in the CMIP6 multi-model ensemble (MME). Area under each curve is normalized to 1. Heatwave Length (days) of the observed event is indicated by vertical lines in dark blue (Berkeley Earth) and light blue (ERA5).

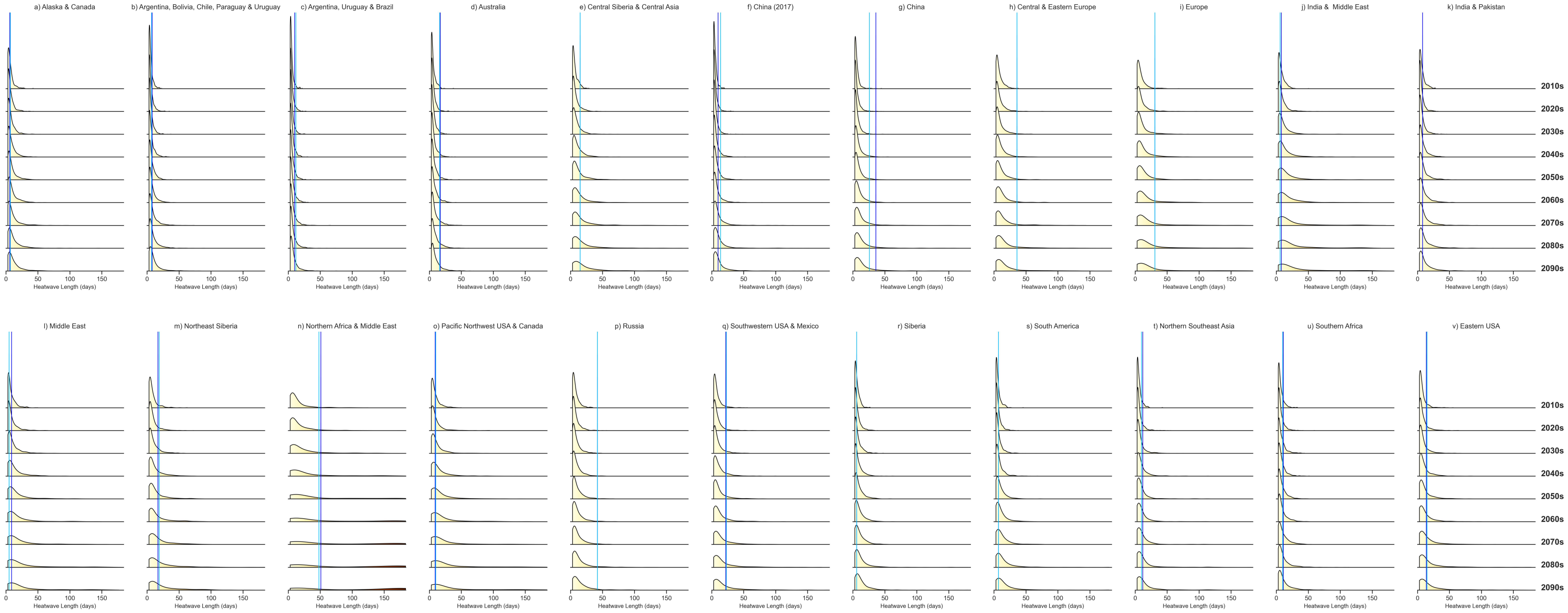

a) Alaska & Canada
b) Argentina, Bolivia, Chile, Paraguay & Uruguay
c) Argentina, Uruguay & Brazil
d) Australia
e) Central Siberia & Central Asia
f) China (2017)
g) China
h) Central & Eastern Europe
i) Europe
j) India & Middle East
k) India & Pakistan
l) Middle East
m) Northeast Siberia
n) Northern Africa & Middle East
o) Pacific Northwest USA & Canada
p) Russia
q) Southwestern USA & Mexico
r) Siberia
s) South America
t) Northern Southeast Asia
u) Southern Africa
v) Eastern USA
2010s
2020s
2030s
2040s
2050s
2060s
2070s
2080s
2090s
0
50
100
150
Heatwave Length (days)

Supplementary Figure 135: Kernel Density Estimates (KDE) across Decades for Heatwave Length (days) in SSP2-4.5 CMIP6 multi-model ensemble (MME) (Tx97.5p) - Kernel Density Estimates (KDE) of Heatwave Length (days) for different Decades in the CMIP6 multi-model ensemble (MME). Area under each curve is normalized to 1. Heatwave Length (days) of the observed event is indicated by vertical lines in dark blue (Berkeley Earth) and light blue (ERA5).

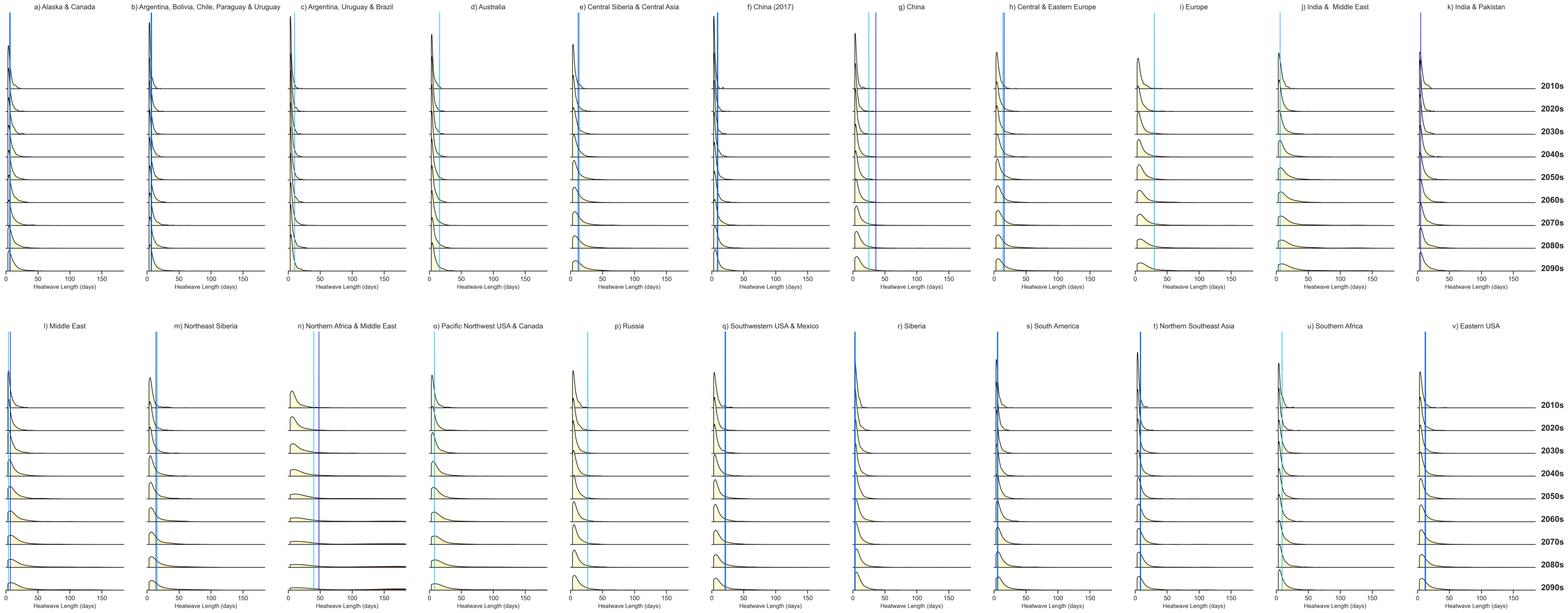
a) Alaska & Canada
b) Argentina, Bolivia, Chile, Paraguay & Uruguay
c) Argentina, Uruguay & Brazil
d) Australia
e) Central Siberia & Central Asia
f) China (2017)
g) China
h) Central & Eastern Europe
i) Europe
j) India & Middle East
k) India & Pakistan
l) Middle East
m) Northeast Siberia
n) Northern Africa & Middle East
o) Pacific Northwest USA & Canada
p) Russia
q) Southwestern USA & Mexico
r) Siberia
s) South America
t) Northern Southeast Asia
u) Southern Africa
v) Eastern USA
2010s
2020s
2030s
2040s
2050s
2060s
2070s
2080s
2090s
0
50
100
150
Heatwave Length (days)

Supplementary Figure 136: Kernel Density Estimates (KDE) across Decades for Heatwave Length (days) in SSP2-4.5 CMIP6 multi-model ensemble (MME) (Tx99.0p) - Kernel Density Estimates (KDE) of Heatwave Length (days) for different Decades in the CMIP6 multi-model ensemble (MME). Area under each curve is normalized to 1. Heatwave Length (days) of the observed event is indicated by vertical lines in dark blue (Berkeley Earth) and light blue (ERA5).

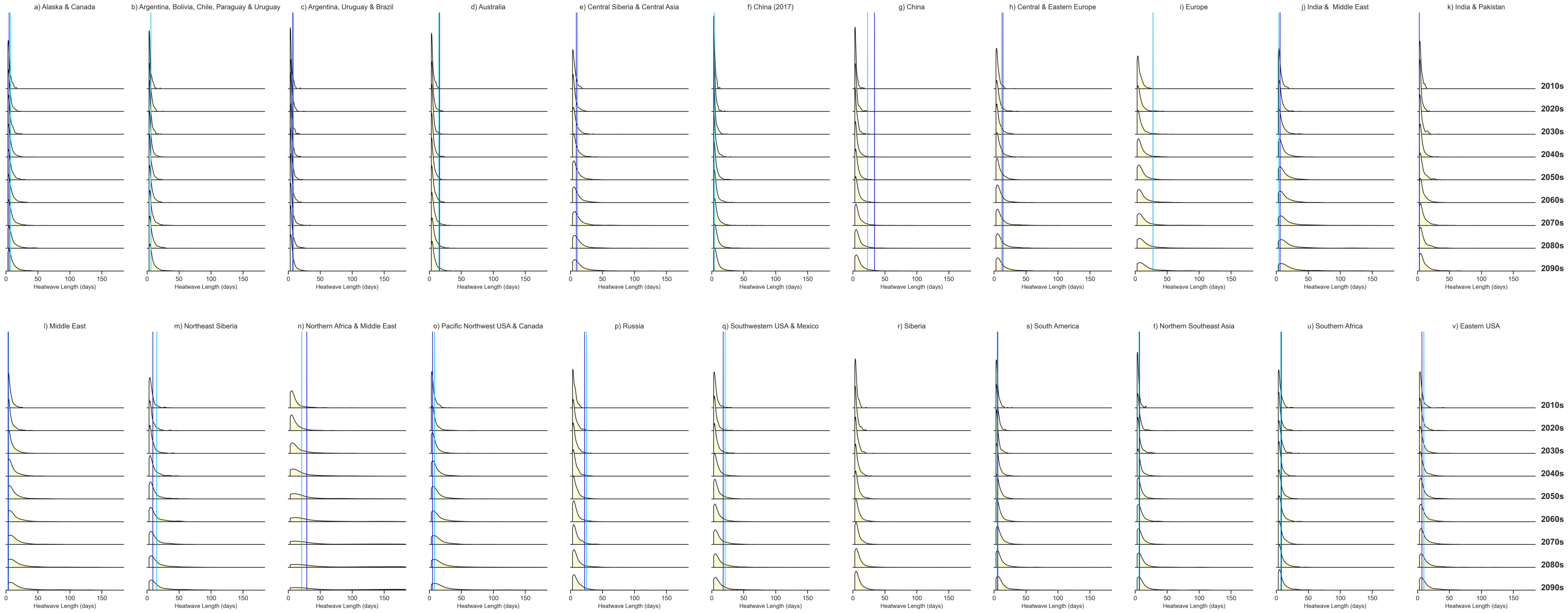

a) Alaska & Canada
b) Argentina, Bolivia, Chile, Paraguay & Uruguay
c) Argentina, Uruguay & Brazil
d) Australia
e) Central Siberia & Central Asia
f) China (2017)
g) China
h) Central & Eastern Europe
i) Europe
j) India & Middle East
k) India & Pakistan
l) Middle East
m) Northeast Siberia
n) Northern Africa & Middle East
o) Pacific Northwest USA & Canada
p) Russia
q) Southwestern USA & Mexico
r) Siberia
s) South America
t) Northern Southeast Asia
u) Southern Africa
v) Eastern USA
2010s
2020s
2030s
2040s
2050s
2060s
2070s
2080s
2090s
0
50
100
150
Heatwave Length (days)

Supplementary Figure 137: Kernel Density Estimates (KDE) across Decades for Heatwave Length (days) in SSP3-7.0 CMIP6 multi-model ensemble (MME) (Tx95.0p) - Kernel Density Estimates (KDE) of Heatwave Length (days) for different Decades in the CMIP6 multi-model ensemble (MME). Area under each curve is normalized to 1. Heatwave Length (days) of the observed event is indicated by vertical lines in dark blue (Berkeley Earth) and light blue (ERA5).

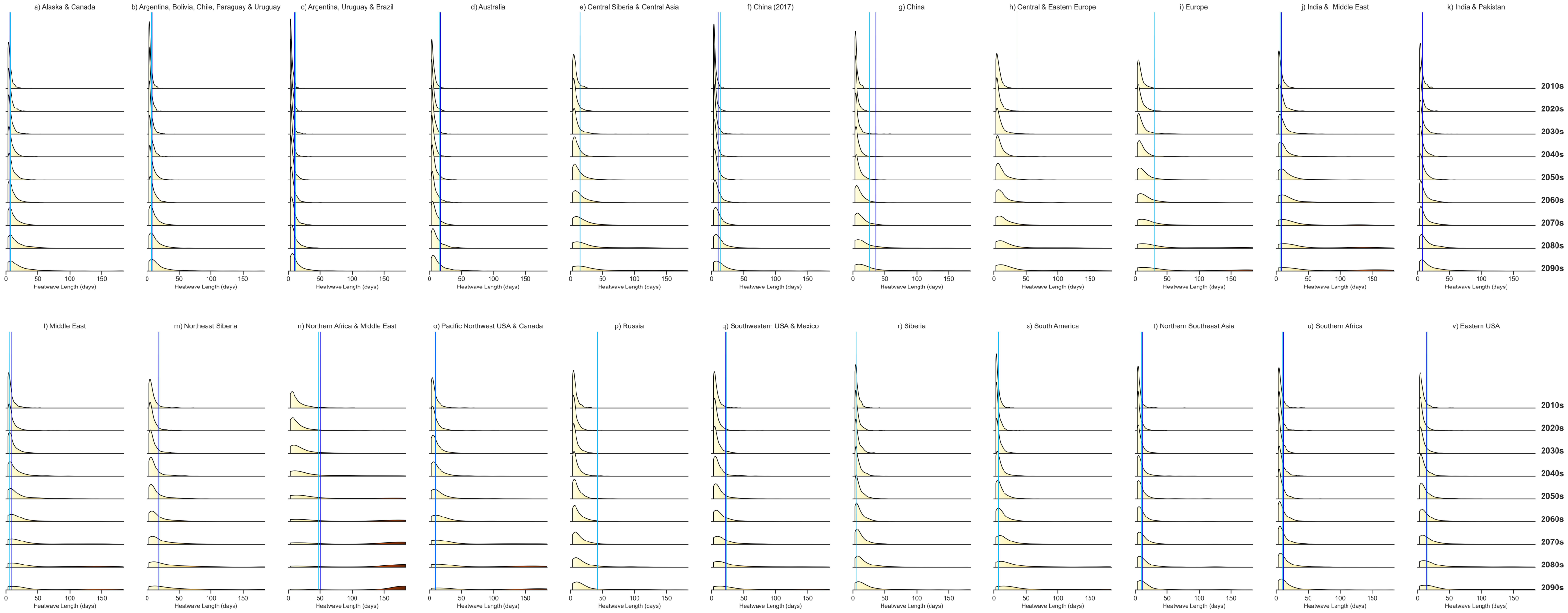

a) Alaska & Canada
b) Argentina, Bolivia, Chile, Paraguay & Uruguay
c) Argentina, Uruguay & Brazil
d) Australia
e) Central Siberia & Central Asia
f) China (2017)
g) China
h) Central & Eastern Europe
i) Europe
j) India & Middle East
k) India & Pakistan
l) Middle East
m) Northeast Siberia
n) Northern Africa & Middle East
o) Pacific Northwest USA & Canada
p) Russia
q) Southwestern USA & Mexico
r) Siberia
s) South America
t) Northern Southeast Asia
u) Southern Africa
v) Eastern USA
2010s
2020s
2030s
2040s
2050s
2060s
2070s
2080s
2090s
0
50
100
150
Heatwave Length (days)

Supplementary Figure 138: Kernel Density Estimates (KDE) across Decades for Heatwave Length (days) in SSP3-7.0 CMIP6 multi-model ensemble (MME) (Tx97.5p) - Kernel Density Estimates (KDE) of Heatwave Length (days) for different Decades in the CMIP6 multi-model ensemble (MME). Area under each curve is normalized to 1. Heatwave Length (days) of the observed event is indicated by vertical lines in dark blue (Berkeley Earth) and light blue (ERA5).

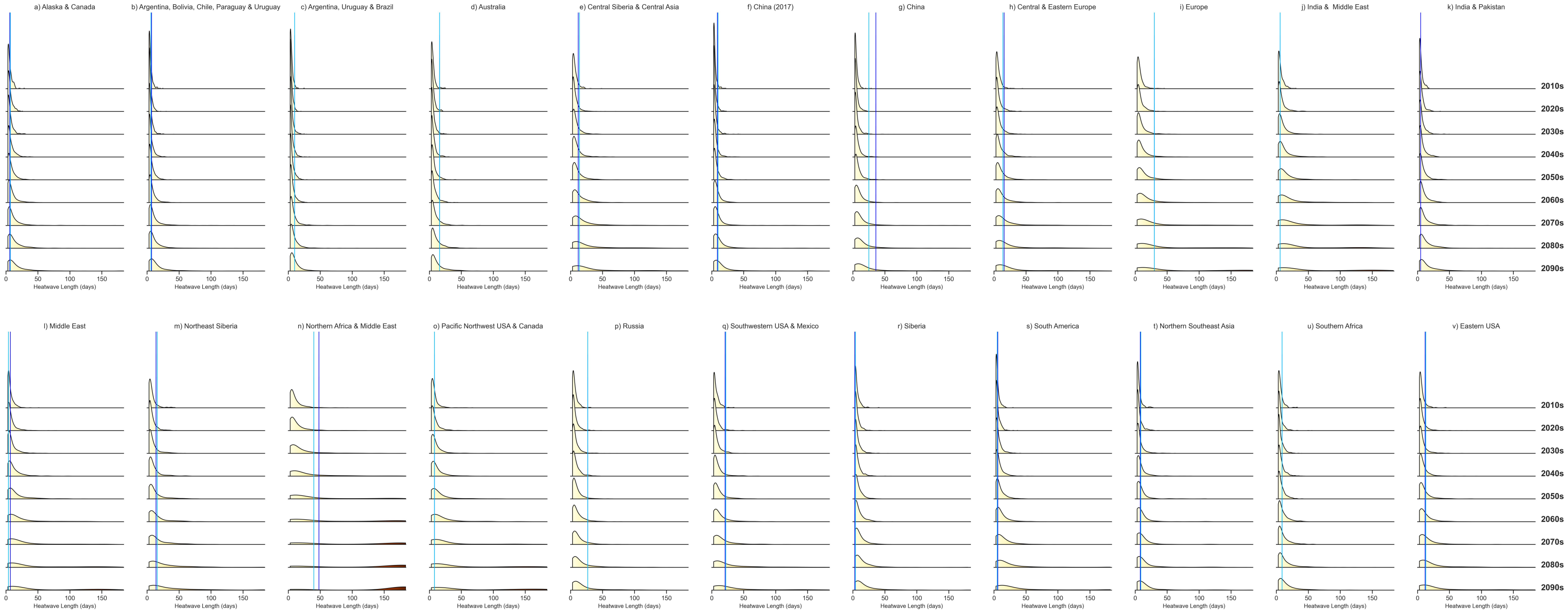

a) Alaska & Canada
b) Argentina, Bolivia, Chile, Paraguay & Uruguay
c) Argentina, Uruguay & Brazil
d) Australia
e) Central Siberia & Central Asia
f) China (2017)
g) China
h) Central & Eastern Europe
i) Europe
j) India & Middle East
k) India & Pakistan
l) Middle East
m) Northeast Siberia
n) Northern Africa & Middle East
o) Pacific Northwest USA & Canada
p) Russia
q) Southwestern USA & Mexico
r) Siberia
s) South America
t) Northern Southeast Asia
u) Southern Africa
v) Eastern USA
2010s
2020s
2030s
2040s
2050s
2060s
2070s
2080s
2090s
0
50
100
150
Heatwave Length (days)

Supplementary Figure 139: Kernel Density Estimates (KDE) across Decades for Heatwave Length (days) in SSP3-7.0 CMIP6 multi-model ensemble (MME) (Tx99.0p) - Kernel Density Estimates (KDE) of Heatwave Length (days) for different Decades in the CMIP6 multi-model ensemble (MME). Area under each curve is normalized to 1. Heatwave Length (days) of the observed event is indicated by vertical lines in dark blue (Berkeley Earth) and light blue (ERA5).

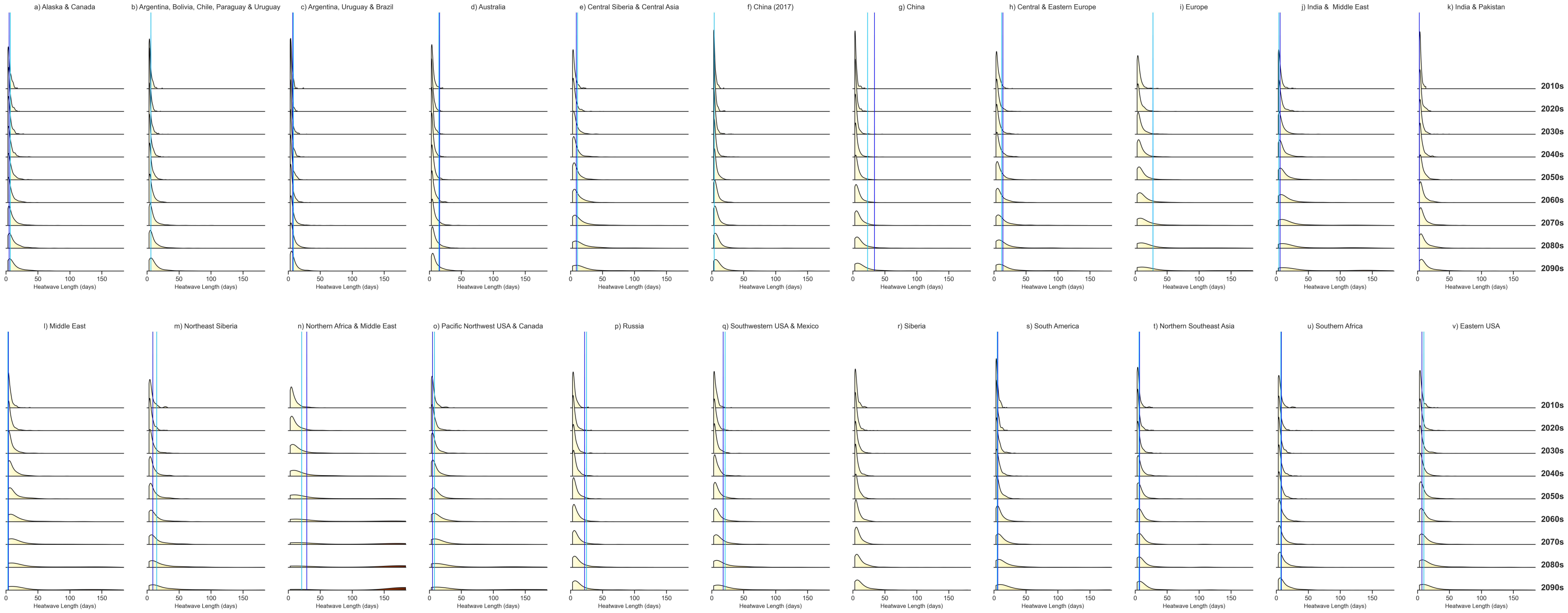

a) Alaska & Canada
b) Argentina, Bolivia, Chile, Paraguay & Uruguay
c) Argentina, Uruguay & Brazil
d) Australia
e) Central Siberia & Central Asia
f) China (2017)
g) China
h) Central & Eastern Europe
i) Europe
j) India & Middle East
k) India & Pakistan
l) Middle East
m) Northeast Siberia
n) Northern Africa & Middle East
o) Pacific Northwest USA & Canada
p) Russia
q) Southwestern USA & Mexico
r) Siberia
s) South America
t) Northern Southeast Asia
u) Southern Africa
v) Eastern USA
2010s
2020s
2030s
2040s
2050s
2060s
2070s
2080s
2090s
Heatwave Length (days)
0
50
100
150

Supplementary Figure 140: Kernel Density Estimates (KDE) across Decades for Heatwave Length (days) in SSP5-8.5 CMIP6 multi-model ensemble (MME) (Tx95.0p) - Kernel Density Estimates (KDE) of Heatwave Length (days) for different Decades in the CMIP6 multi-model ensemble (MME). Area under each curve is normalized to 1. Heatwave Length (days) of the observed event is indicated by vertical lines in dark blue (Berkeley Earth) and light blue (ERA5).

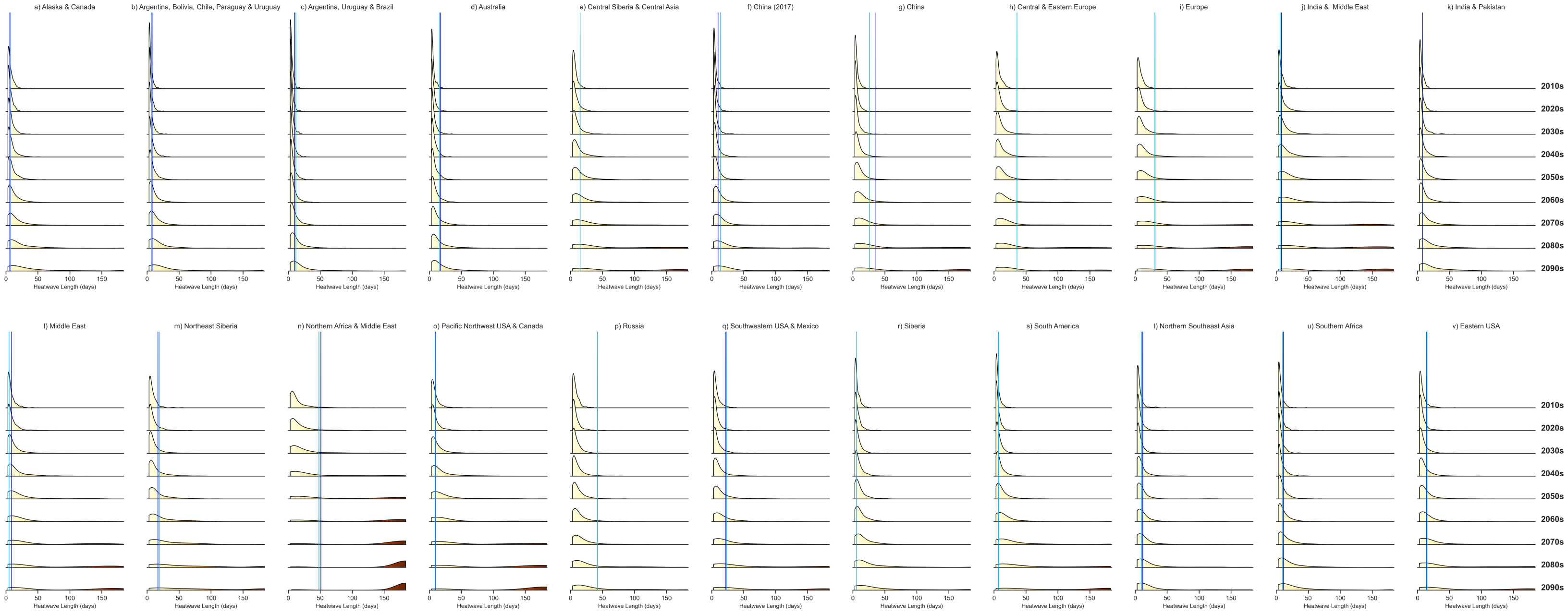

a) Alaska & Canada
b) Argentina, Bolivia, Chile, Paraguay & Uruguay
c) Argentina, Uruguay & Brazil
d) Australia
e) Central Siberia & Central Asia
f) China (2017)
g) China
h) Central & Eastern Europe
i) Europe
j) India & Middle East
k) India & Pakistan
l) Middle East
m) Northeast Siberia
n) Northern Africa & Middle East
o) Pacific Northwest USA & Canada
p) Russia
q) Southwestern USA & Mexico
r) Siberia
s) South America
t) Northern Southeast Asia
u) Southern Africa
v) Eastern USA
2010s
2020s
2030s
2040s
2050s
2060s
2070s
2080s
2090s
0
50
100
150
Heatwave Length (days)

Supplementary Figure 141: Kernel Density Estimates (KDE) across Decades for Heatwave Length (days) in SSP5-8.5 CMIP6 multi-model ensemble (MME) (Tx97.5p) - Kernel Density Estimates (KDE) of Heatwave Length (days) for different Decades in the CMIP6 multi-model ensemble (MME). Area under each curve is normalized to 1. Heatwave Length (days) of the observed event is indicated by vertical lines in dark blue (Berkeley Earth) and light blue (ERA5).

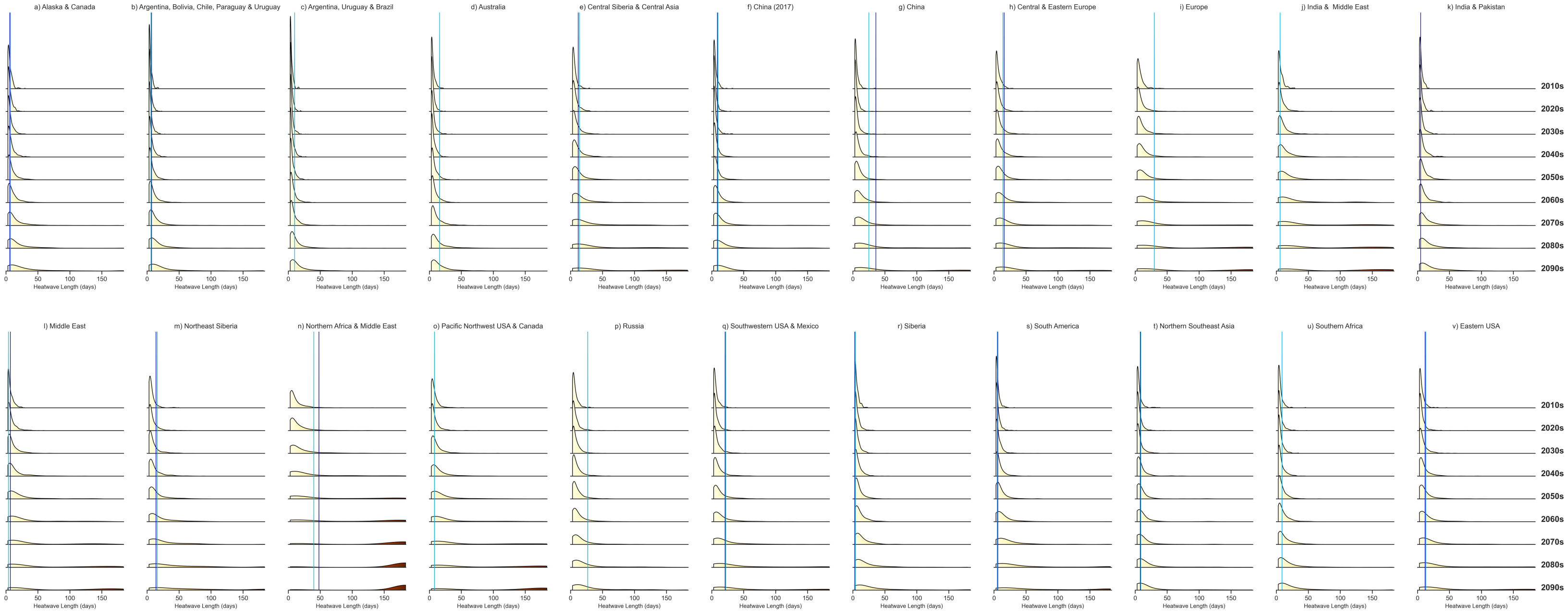

a) Alaska & Canada
b) Argentina, Bolivia, Chile, Paraguay & Uruguay
c) Argentina, Uruguay & Brazil
d) Australia
e) Central Siberia & Central Asia
f) China (2017)
g) China
h) Central & Eastern Europe
i) Europe
j) India & Middle East
k) India & Pakistan
l) Middle East
m) Northeast Siberia
n) Northern Africa & Middle East
o) Pacific Northwest USA & Canada
p) Russia
q) Southwestern USA & Mexico
r) Siberia
s) South America
t) Northern Southeast Asia
u) Southern Africa
v) Eastern USA
2010s
2020s
2030s
2040s
2050s
2060s
2070s
2080s
2090s
0
50
100
150
Heatwave Length (days)

Supplementary Figure 142: Kernel Density Estimates (KDE) across Decades for Heatwave Length (days) in SSP5-8.5 CMIP6 multi-model ensemble (MME) (Tx99.0p) - Kernel Density Estimates (KDE) of Heatwave Length (days) for different Decades in the CMIP6 multi-model ensemble (MME). Area under each curve is normalized to 1. Heatwave Length (days) of the observed event is indicated by vertical lines in dark blue (Berkeley Earth) and light blue (ERA5).

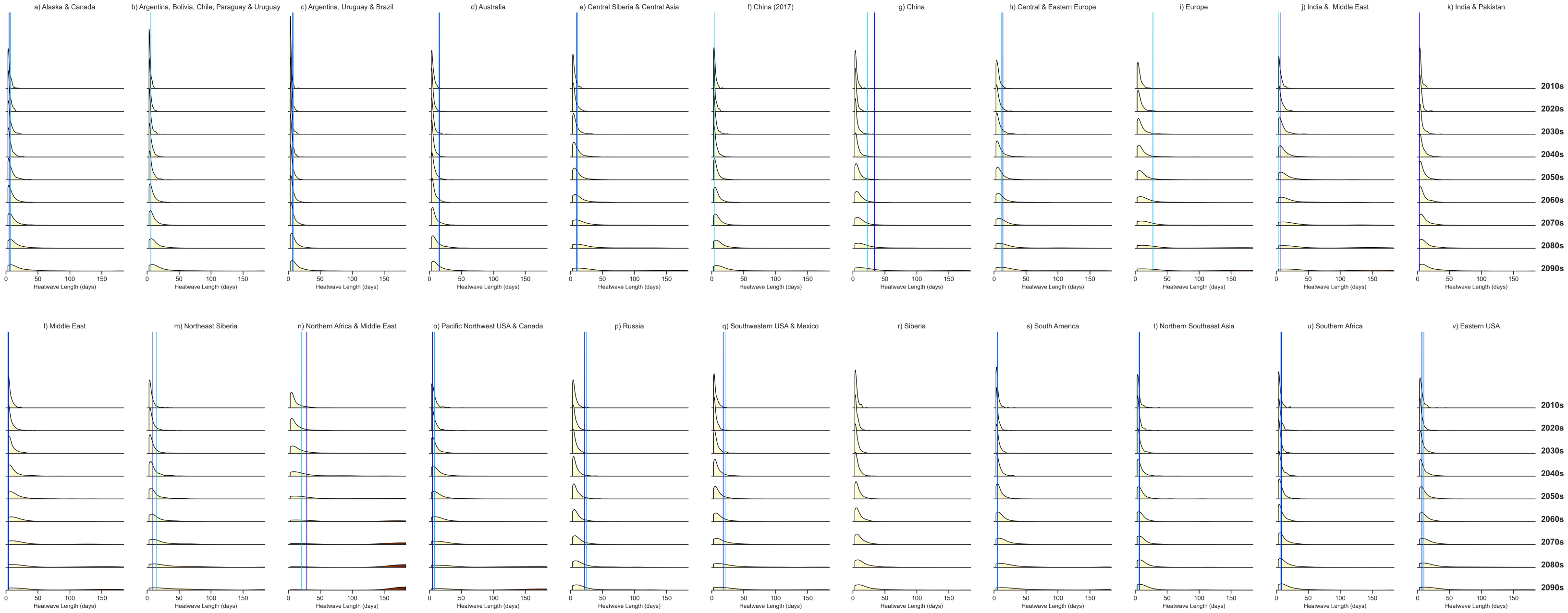
a) Alaska & Canada
b) Argentina, Bolivia, Chile, Paraguay & Uruguay
c) Argentina, Uruguay & Brazil
d) Australia
e) Central Siberia & Central Asia
f) China (2017)
g) China
h) Central & Eastern Europe
i) Europe
j) India & Middle East
k) India & Pakistan
l) Middle East
m) Northeast Siberia
n) Northern Africa & Middle East
o) Pacific Northwest USA & Canada
p) Russia
q) Southwestern USA & Mexico
r) Siberia
s) South America
t) Northern Southeast Asia
u) Southern Africa
v) Eastern USA
2010s
2020s
2030s
2040s
2050s
2060s
2070s
2080s
2090s
0
50
100
150
Heatwave Length (days)

Supplementary Figure 143: Kernel Density Estimates (KDE) across Decades for Median Heatwave Severity in Historical CMIP6 multi-model ensemble (MME) (Tx95.0p) - Kernel Density Estimates (KDE) of Median Heatwave Severity for different Decades in the CMIP6 multi-model ensemble (MME). Area under each curve is normalized to 1. Median Heatwave Severity of the observed event is indicated by vertical lines in dark blue (Berkeley Earth) and light blue (ERA5).

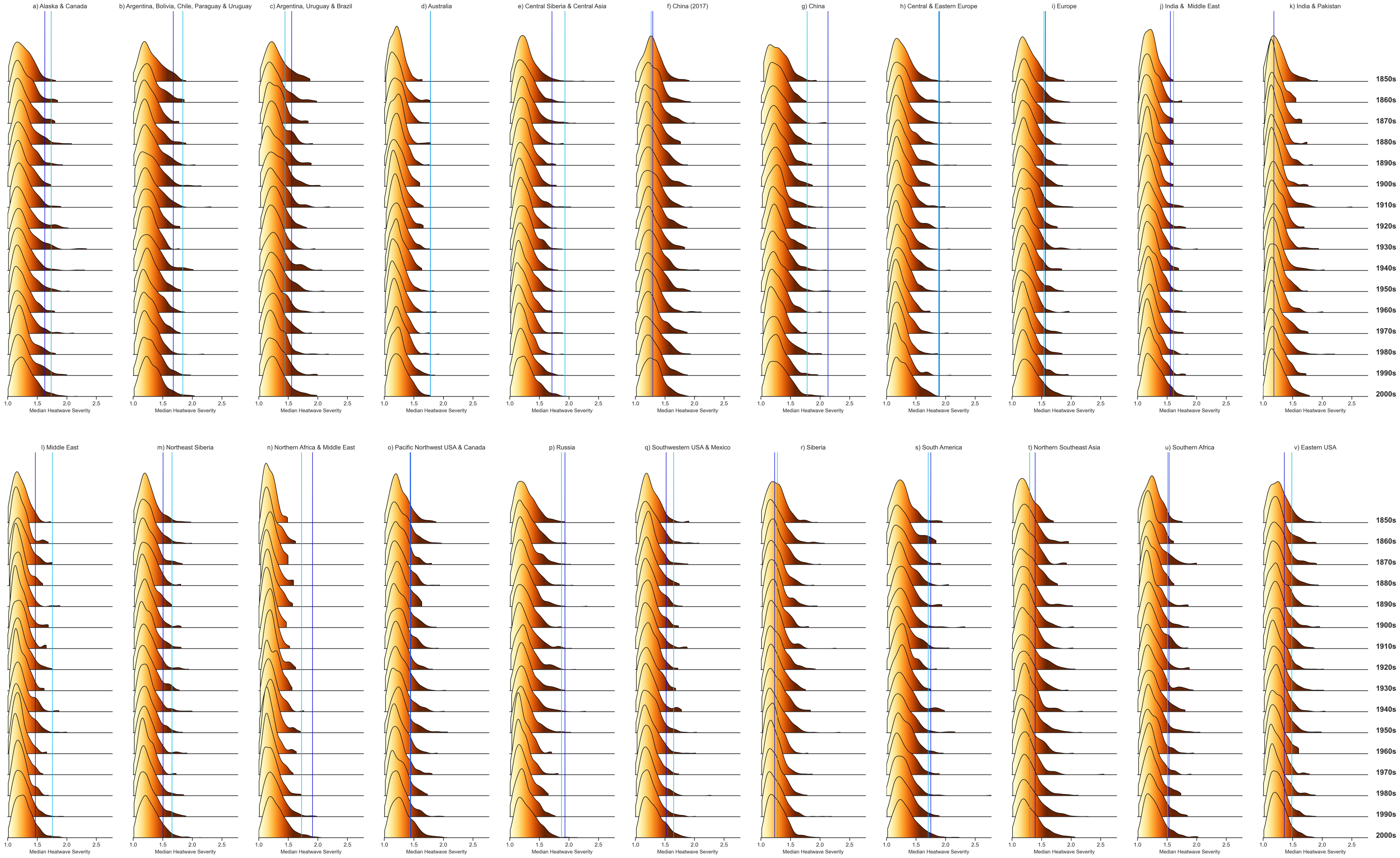

a) Alaska & Canada
b) Argentina, Bolivia, Chile, Paraguay & Uruguay
c) Argentina, Uruguay & Brazil
d) Australia
e) Central Siberia & Central Asia
f) China (2017)
g) China
h) Central & Eastern Europe
i) Europe
j) India & Middle East
k) India & Pakistan
l) Middle East
m) Northeast Siberia
n) Northern Africa & Middle East
o) Pacific Northwest USA & Canada
p) Russia
q) Southwestern USA & Mexico
r) Siberia
s) South America
t) Northern Southeast Asia
u) Southern Africa
v) Eastern USA
1850s
1860s
1870s
1880s
1890s
1900s
1910s
1920s
1930s
1940s
1950s
1960s
1970s
1980s
1990s
2000s
1.0 1.5 2.0 2.5
Median Heatwave Severity

Supplementary Figure 144: Kernel Density Estimates (KDE) across Decades for Median Heatwave Severity in Historical CMIP6 multi-model ensemble (MME) (Tx97.5p) - Kernel Density Estimates (KDE) of Median Heatwave Severity for different Decades in the CMIP6 multi-model ensemble (MME). Area under each curve is normalized to 1. Median Heatwave Severity of the observed event is indicated by vertical lines in dark blue (Berkeley Earth) and light blue (ERA5).

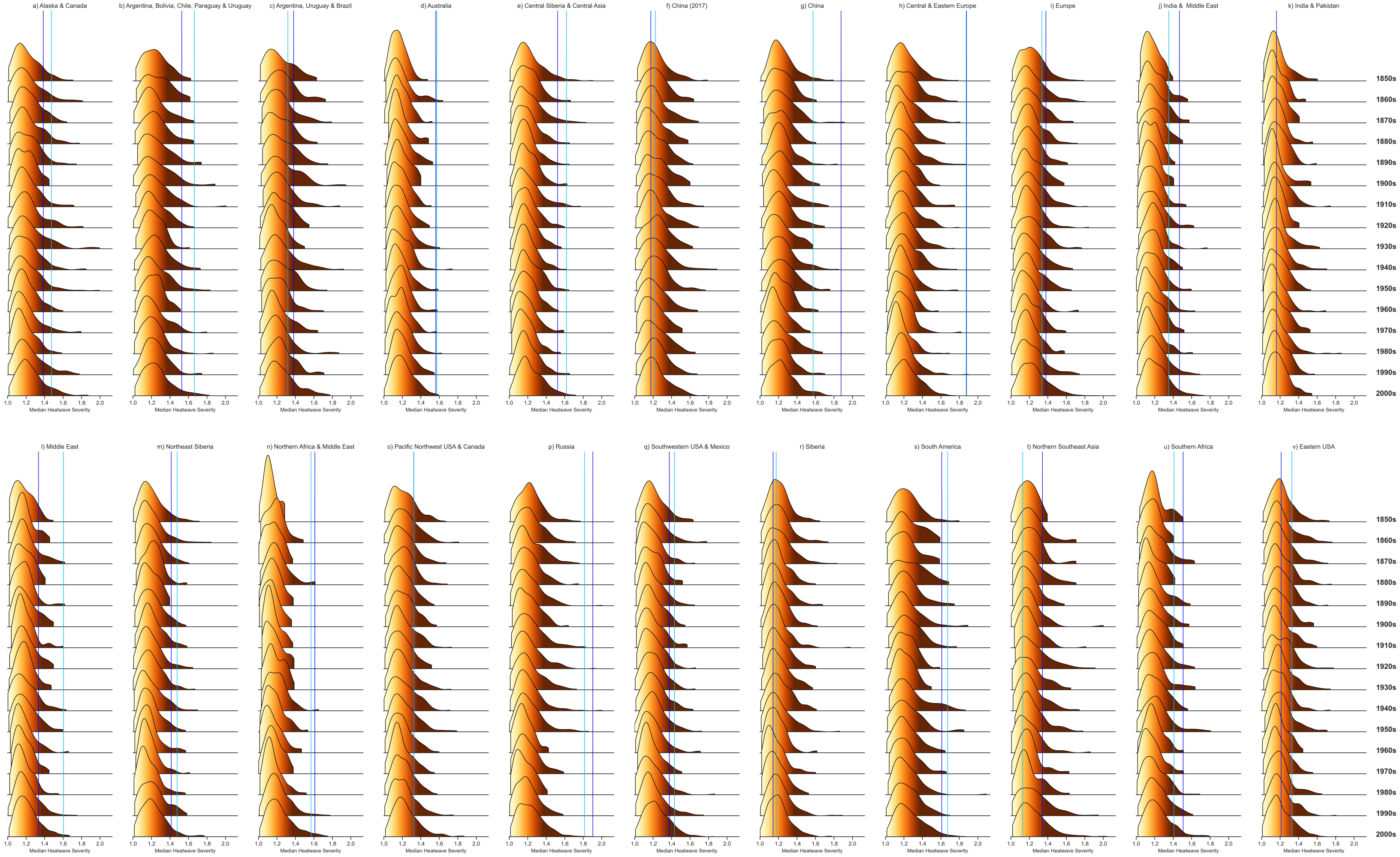

a) Alaska & Canada
b) Argentina, Bolivia, Chile, Paraguay & Uruguay
c) Argentina, Uruguay & Brazil
d) Australia
e) Central Siberia & Central Asia
f) China (2017)
g) China
h) Central & Eastern Europe
i) Europe
j) India & Middle East
k) India & Pakistan
l) Middle East
m) Northeast Siberia
n) Northern Africa & Middle East
o) Pacific Northwest USA & Canada
p) Russia
q) Southwestern USA & Mexico
r) Siberia
s) South America
t) Northern Southeast Asia
u) Southern Africa
v) Eastern USA
1850s
1860s
1870s
1880s
1890s
1900s
1910s
1920s
1930s
1940s
1950s
1960s
1970s
1980s
1990s
2000s
1.0 1.2 1.4 1.6 1.8 2.0
Median Heatwave Severity

Supplementary Figure 145: Kernel Density Estimates (KDE) across Decades for Median Heatwave Severity in Historical CMIP6 multi-model ensemble (MME) (Tx99.0p) - Kernel Density Estimates (KDE) of Median Heatwave Severity for different Decades in the CMIP6 multi-model ensemble (MME). Area under each curve is normalized to 1. Median Heatwave Severity of the observed event is indicated by vertical lines in dark blue (Berkeley Earth) and light blue (ERA5).

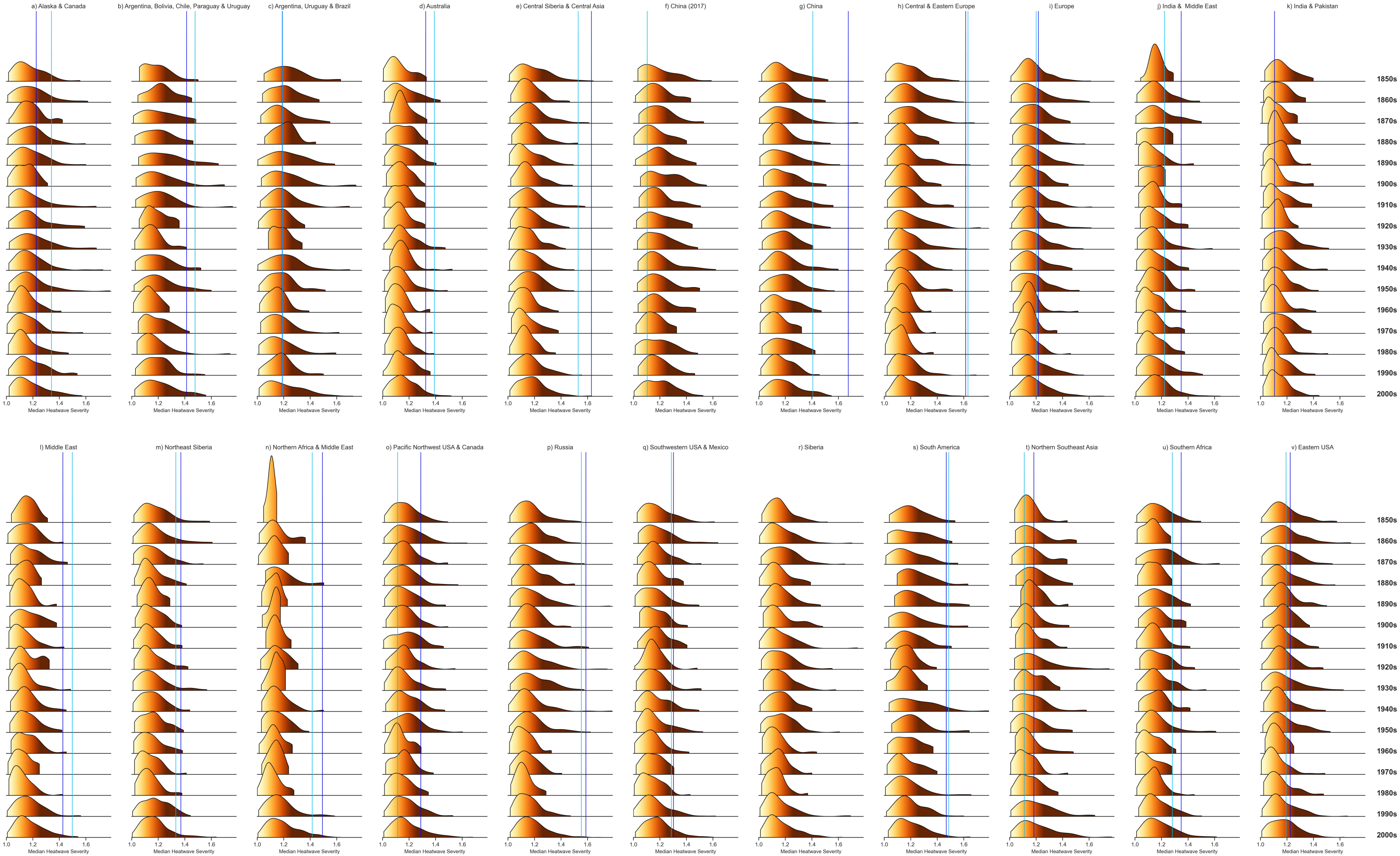
a) Alaska & Canada
b) Argentina, Bolivia, Chile, Paraguay & Uruguay
c) Argentina, Uruguay & Brazil
d) Australia
e) Central Siberia & Central Asia
f) China (2017)
g) China
h) Central & Eastern Europe
i) Europe
j) India & Middle East
k) India & Pakistan
l) Middle East
m) Northeast Siberia
n) Northern Africa & Middle East
o) Pacific Northwest USA & Canada
p) Russia
q) Southwestern USA & Mexico
r) Siberia
s) South America
t) Northern Southeast Asia
u) Southern Africa
v) Eastern USA
Median Heatwave Severity
1.0
1.2
1.4
1.6
1850s
1860s
1870s
1880s
1890s
1900s
1910s
1920s
1930s
1940s
1950s
1960s
1970s
1980s
1990s
2000s

Supplementary Figure 146: Kernel Density Estimates (KDE) across Decades for Median Heatwave Severity in SSP1-2.6 CMIP6 multi-model ensemble (MME) (Tx95.0p) - Kernel Density Estimates (KDE) of Median Heatwave Severity for different Decades in the CMIP6 multi-model ensemble (MME). Area under each curve is normalized to 1. Median Heatwave Severity of the observed event is indicated by vertical lines in dark blue (Berkeley Earth) and light blue (ERA5).

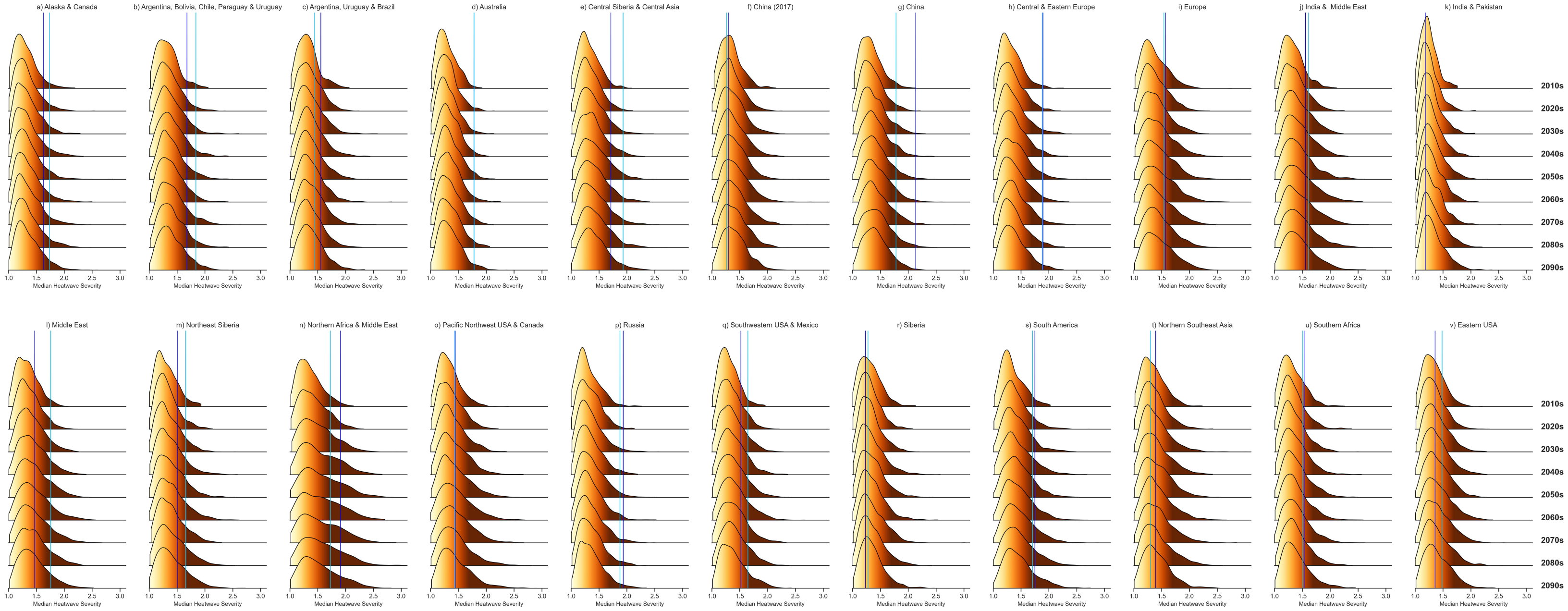
a) Alaska & Canada
b) Argentina, Bolivia, Chile, Paraguay & Uruguay
c) Argentina, Uruguay & Brazil
d) Australia
e) Central Siberia & Central Asia
f) China (2017)
g) China
h) Central & Eastern Europe
i) Europe
j) India & Middle East
k) India & Pakistan
l) Middle East
m) Northeast Siberia
n) Northern Africa & Middle East
o) Pacific Northwest USA & Canada
p) Russia
q) Southwestern USA & Mexico
r) Siberia
s) South America
t) Northern Southeast Asia
u) Southern Africa
v) Eastern USA
2010s
2020s
2030s
2040s
2050s
2060s
2070s
2080s
2090s
1.0
1.5
2.0
2.5
3.0
Median Heatwave Severity

Supplementary Figure 147: Kernel Density Estimates (KDE) across Decades for Median Heatwave Severity in SSP1-2.6 CMIP6 multi-model ensemble (MME) (Tx97.5p) - Kernel Density Estimates (KDE) of Median Heatwave Severity for different Decades in the CMIP6 multi-model ensemble (MME). Area under each curve is normalized to 1. Median Heatwave Severity of the observed event is indicated by vertical lines in dark blue (Berkeley Earth) and light blue (ERA5).

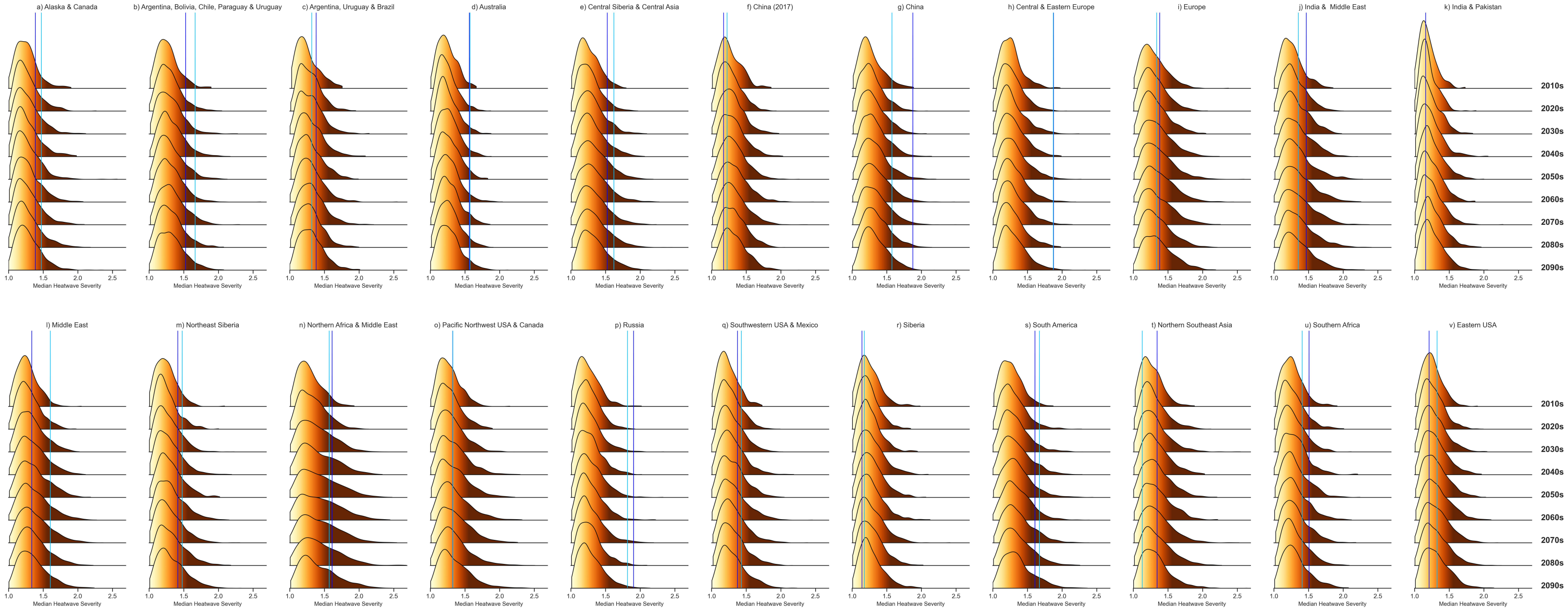

a) Alaska & Canada
b) Argentina, Bolivia, Chile, Paraguay & Uruguay
c) Argentina, Uruguay & Brazil
d) Australia
e) Central Siberia & Central Asia
f) China (2017)
g) China
h) Central & Eastern Europe
i) Europe
j) India & Middle East
k) India & Pakistan
l) Middle East
m) Northeast Siberia
n) Northern Africa & Middle East
o) Pacific Northwest USA & Canada
p) Russia
q) Southwestern USA & Mexico
r) Siberia
s) South America
t) Northern Southeast Asia
u) Southern Africa
v) Eastern USA
2010s
2020s
2030s
2040s
2050s
2060s
2070s
2080s
2090s
1.0
1.5
2.0
2.5
Median Heatwave Severity

Supplementary Figure 148: Kernel Density Estimates (KDE) across Decades for Median Heatwave Severity in SSP1-2.6 CMIP6 multi-model ensemble (MME) (Tx99.0p) - Kernel Density Estimates (KDE) of Median Heatwave Severity for different Decades in the CMIP6 multi-model ensemble (MME). Area under each curve is normalized to 1. Median Heatwave Severity of the observed event is indicated by vertical lines in dark blue (Berkeley Earth) and light blue (ERA5).

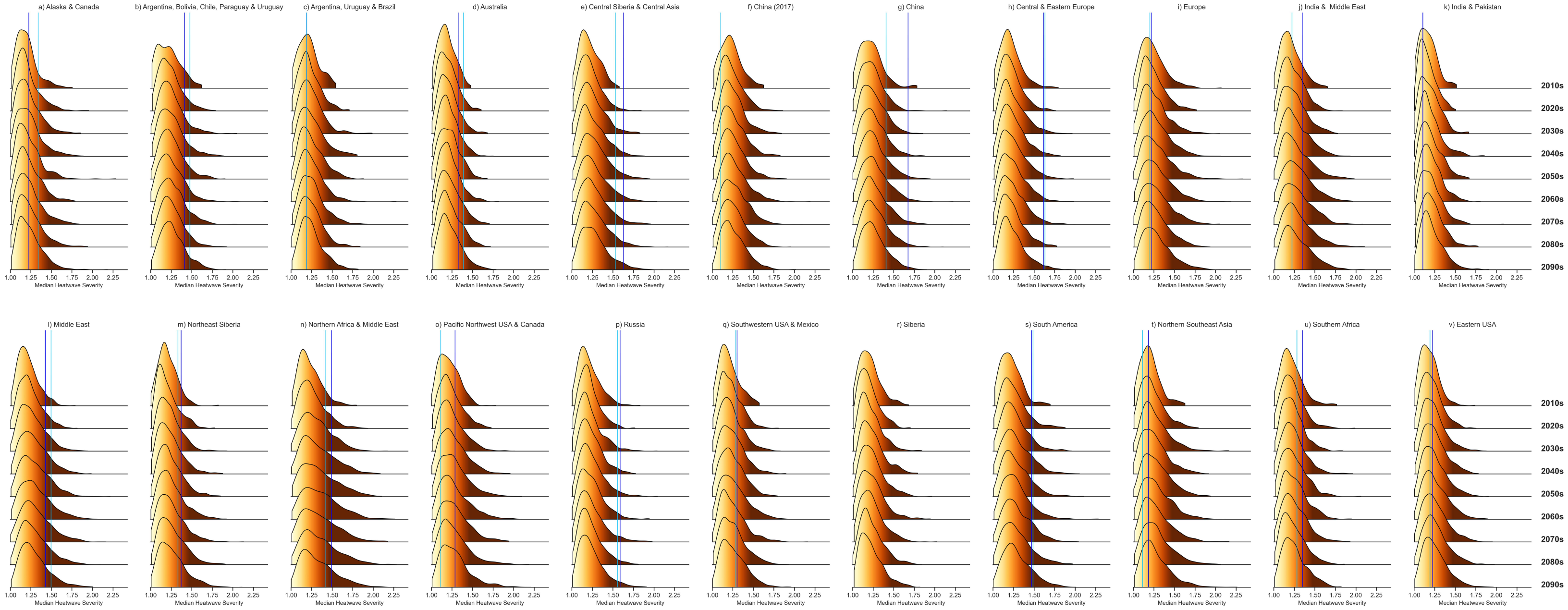

a) Alaska & Canada
b) Argentina, Bolivia, Chile, Paraguay & Uruguay
c) Argentina, Uruguay & Brazil
d) Australia
e) Central Siberia & Central Asia
f) China (2017)
g) China
h) Central & Eastern Europe
i) Europe
j) India & Middle East
k) India & Pakistan
l) Middle East
m) Northeast Siberia
n) Northern Africa & Middle East
o) Pacific Northwest USA & Canada
p) Russia
q) Southwestern USA & Mexico
r) Siberia
s) South America
t) Northern Southeast Asia
u) Southern Africa
v) Eastern USA
2010s
2020s
2030s
2040s
2050s
2060s
2070s
2080s
2090s
1.00
1.25
1.50
1.75
2.00
2.25
Median Heatwave Severity

Supplementary Figure 149: Kernel Density Estimates (KDE) across Decades for Median Heatwave Severity in SSP2-4.5 CMIP6 multi-model ensemble (MME) (Tx95.0p) - Kernel Density Estimates (KDE) of Median Heatwave Severity for different Decades in the CMIP6 multi-model ensemble (MME). Area under each curve is normalized to 1. Median Heatwave Severity of the observed event is indicated by vertical lines in dark blue (Berkeley Earth) and light blue (ERA5).

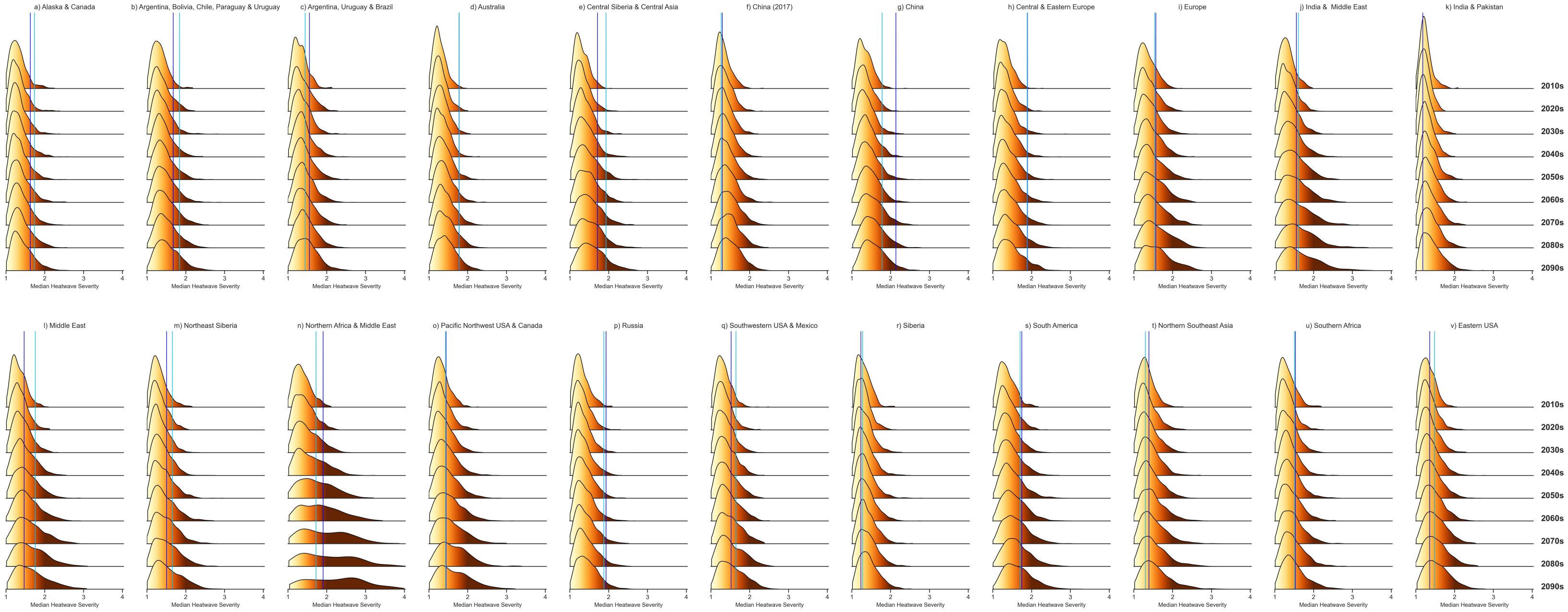

a) Alaska & Canada
b) Argentina, Bolivia, Chile, Paraguay & Uruguay
c) Argentina, Uruguay & Brazil
d) Australia
e) Central Siberia & Central Asia
f) China (2017)
g) China
h) Central & Eastern Europe
i) Europe
j) India & Middle East
k) India & Pakistan
l) Middle East
m) Northeast Siberia
n) Northern Africa & Middle East
o) Pacific Northwest USA & Canada
p) Russia
q) Southwestern USA & Mexico
r) Siberia
s) South America
t) Northern Southeast Asia
u) Southern Africa
v) Eastern USA
2010s
2020s
2030s
2040s
2050s
2060s
2070s
2080s
2090s
Median Heatwave Severity
1
2
3
4

Supplementary Figure 150: Kernel Density Estimates (KDE) across Decades for Median Heatwave Severity in SSP2-4.5 CMIP6 multi-model ensemble (MME) (Tx97.5p) - Kernel Density Estimates (KDE) of Median Heatwave Severity for different Decades in the CMIP6 multi-model ensemble (MME). Area under each curve is normalized to 1. Median Heatwave Severity of the observed event is indicated by vertical lines in dark blue (Berkeley Earth) and light blue (ERA5).

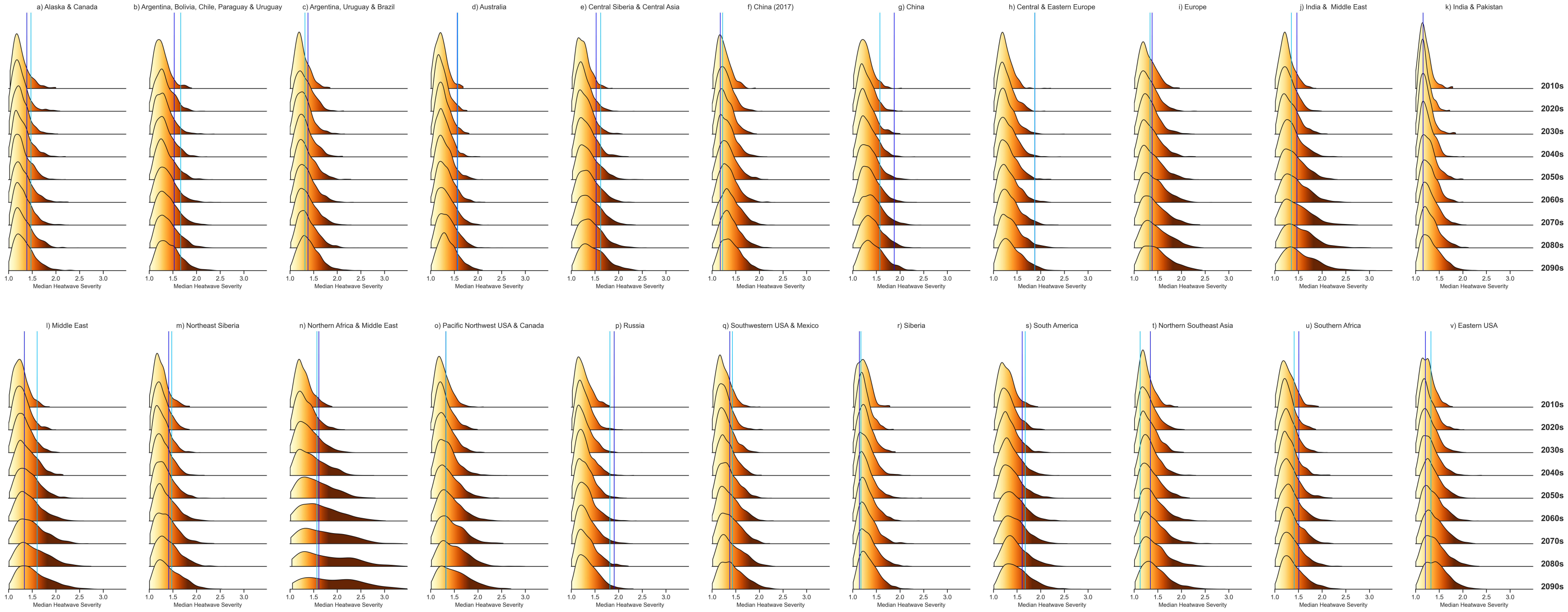

a) Alaska & Canada
b) Argentina, Bolivia, Chile, Paraguay & Uruguay
c) Argentina, Uruguay & Brazil
d) Australia
e) Central Siberia & Central Asia
f) China (2017)
g) China
h) Central & Eastern Europe
i) Europe
j) India & Middle East
k) India & Pakistan
l) Middle East
m) Northeast Siberia
n) Northern Africa & Middle East
o) Pacific Northwest USA & Canada
p) Russia
q) Southwestern USA & Mexico
r) Siberia
s) South America
t) Northern Southeast Asia
u) Southern Africa
v) Eastern USA
2010s
2020s
2030s
2040s
2050s
2060s
2070s
2080s
2090s
1.0
1.5
2.0
2.5
3.0
Median Heatwave Severity

Supplementary Figure 151: Kernel Density Estimates (KDE) across Decades for Median Heatwave Severity in SSP2-4.5 CMIP6 multi-model ensemble (MME) (Tx99.0p) - Kernel Density Estimates (KDE) of Median Heatwave Severity for different Decades in the CMIP6 multi-model ensemble (MME). Area under each curve is normalized to 1. Median Heatwave Severity of the observed event is indicated by vertical lines in dark blue (Berkeley Earth) and light blue (ERA5).

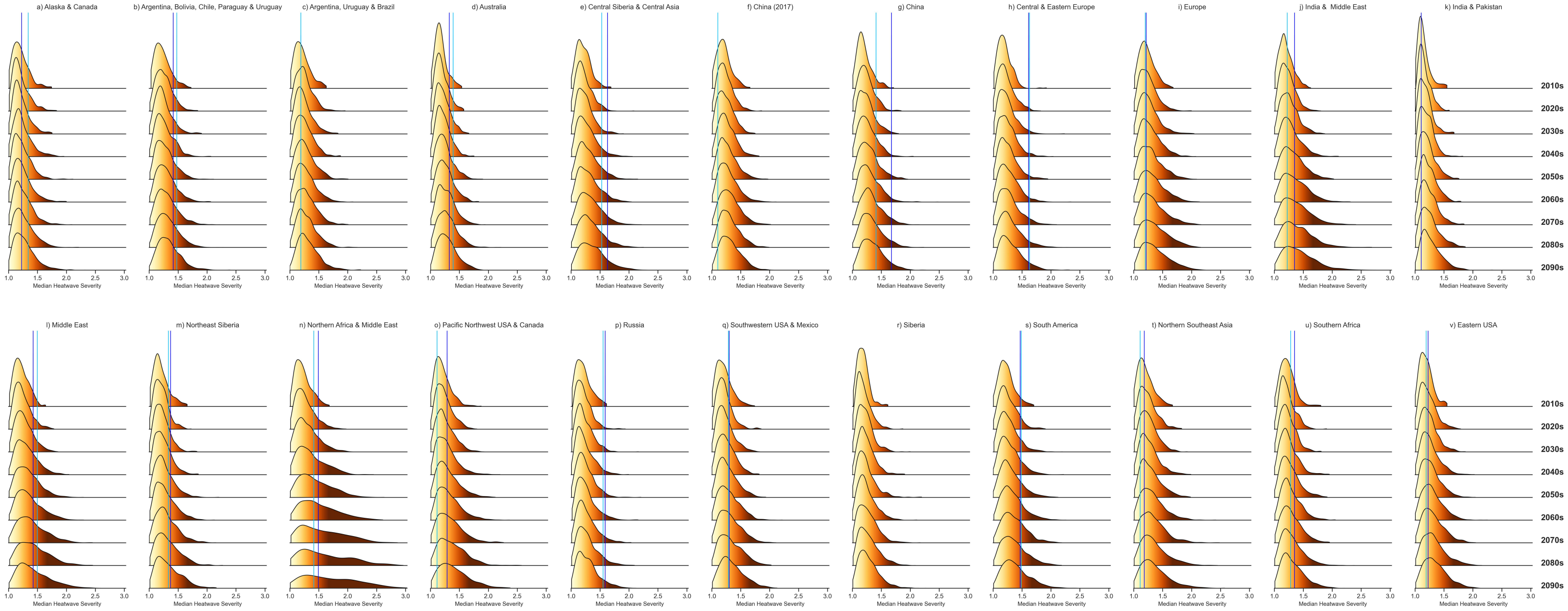

a) Alaska & Canada
b) Argentina, Bolivia, Chile, Paraguay & Uruguay
c) Argentina, Uruguay & Brazil
d) Australia
e) Central Siberia & Central Asia
f) China (2017)
g) China
h) Central & Eastern Europe
i) Europe
j) India & Middle East
k) India & Pakistan
l) Middle East
m) Northeast Siberia
n) Northern Africa & Middle East
o) Pacific Northwest USA & Canada
p) Russia
q) Southwestern USA & Mexico
r) Siberia
s) South America
t) Northern Southeast Asia
u) Southern Africa
v) Eastern USA
2010s
2020s
2030s
2040s
2050s
2060s
2070s
2080s
2090s
1.0
1.5
2.0
2.5
3.0
Median Heatwave Severity

Supplementary Figure 152: Kernel Density Estimates (KDE) across Decades for Median Heatwave Severity in SSP3-7.0 CMIP6 multi-model ensemble (MME) (Tx95.0p) - Kernel Density Estimates (KDE) of Median Heatwave Severity for different Decades in the CMIP6 multi-model ensemble (MME). Area under each curve is normalized to 1. Median Heatwave Severity of the observed event is indicated by vertical lines in dark blue (Berkeley Earth) and light blue (ERA5).

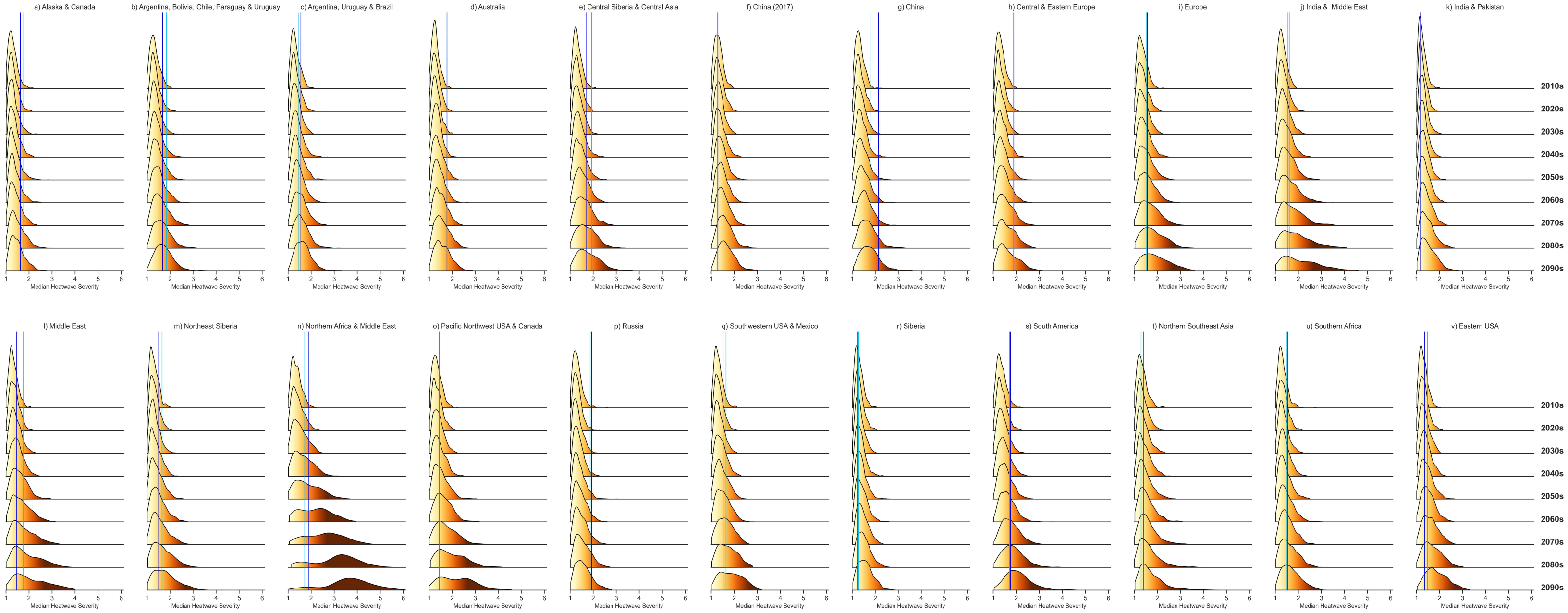

a) Alaska & Canada
b) Argentina, Bolivia, Chile, Paraguay & Uruguay
c) Argentina, Uruguay & Brazil
d) Australia
e) Central Siberia & Central Asia
f) China (2017)
g) China
h) Central & Eastern Europe
i) Europe
j) India & Middle East
k) India & Pakistan
l) Middle East
m) Northeast Siberia
n) Northern Africa & Middle East
o) Pacific Northwest USA & Canada
p) Russia
q) Southwestern USA & Mexico
r) Siberia
s) South America
t) Northern Southeast Asia
u) Southern Africa
v) Eastern USA
2010s
2020s
2030s
2040s
2050s
2060s
2070s
2080s
2090s
Median Heatwave Severity
1 2 3 4 5 6

Supplementary Figure 153: Kernel Density Estimates (KDE) across Decades for Median Heatwave Severity in SSP3-7.0 CMIP6 multi-model ensemble (MME) (Tx97.5p) - Kernel Density Estimates (KDE) of Median Heatwave Severity for different Decades in the CMIP6 multi-model ensemble (MME). Area under each curve is normalized to 1. Median Heatwave Severity of the observed event is indicated by vertical lines in dark blue (Berkeley Earth) and light blue (ERA5).

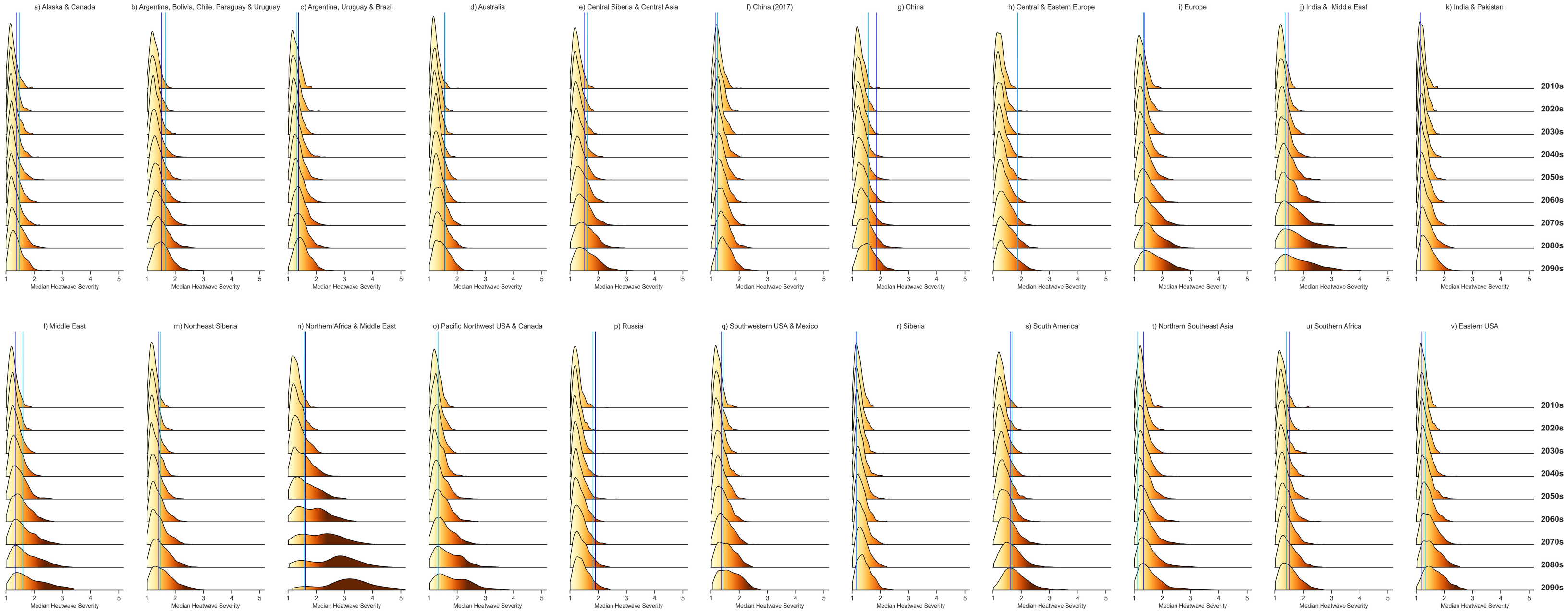

a) Alaska & Canada
b) Argentina, Bolivia, Chile, Paraguay & Uruguay
c) Argentina, Uruguay & Brazil
d) Australia
e) Central Siberia & Central Asia
f) China (2017)
g) China
h) Central & Eastern Europe
i) Europe
j) India & Middle East
k) India & Pakistan
l) Middle East
m) Northeast Siberia
n) Northern Africa & Middle East
o) Pacific Northwest USA & Canada
p) Russia
q) Southwestern USA & Mexico
r) Siberia
s) South America
t) Northern Southeast Asia
u) Southern Africa
v) Eastern USA
2010s
2020s
2030s
2040s
2050s
2060s
2070s
2080s
2090s
Median Heatwave Severity
1
2
3
4
5

Supplementary Figure 154: Kernel Density Estimates (KDE) across Decades for Median Heatwave Severity in SSP3-7.0 CMIP6 multi-model ensemble (MME) (Tx99.0p) - Kernel Density Estimates (KDE) of Median Heatwave Severity for different Decades in the CMIP6 multi-model ensemble (MME). Area under each curve is normalized to 1. Median Heatwave Severity of the observed event is indicated by vertical lines in dark blue (Berkeley Earth) and light blue (ERA5).

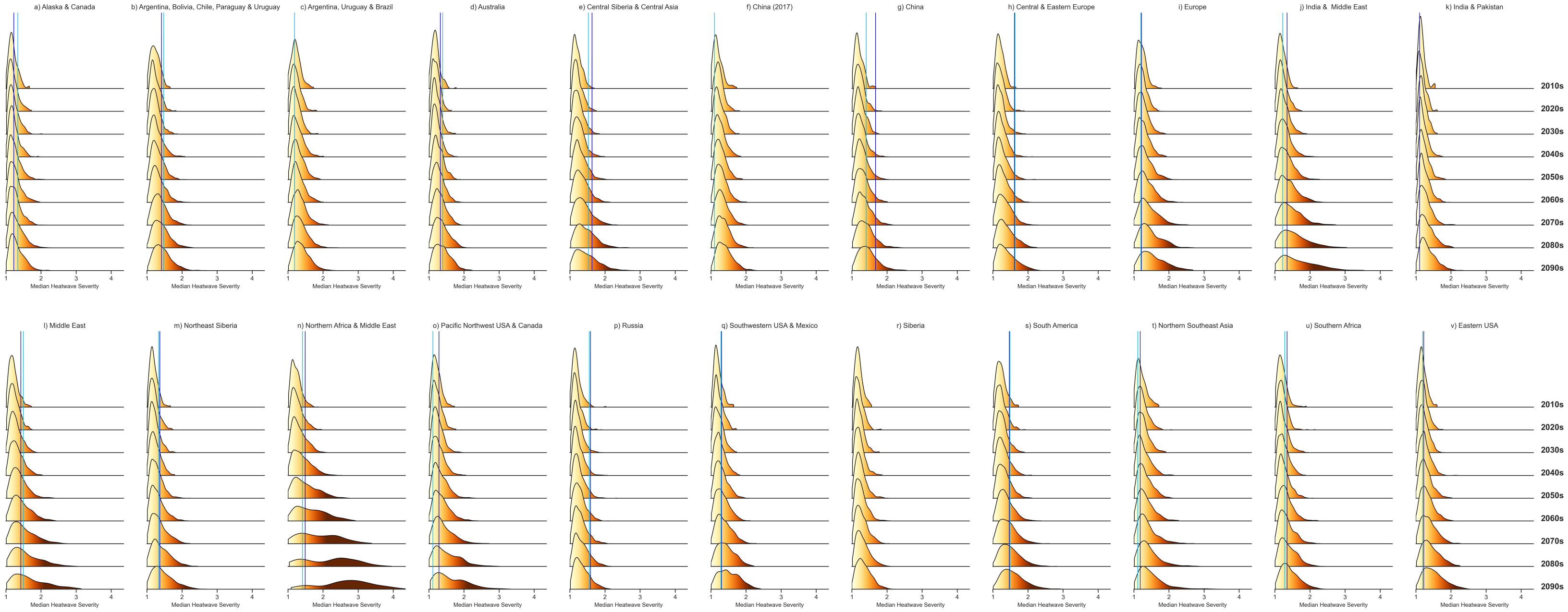

a) Alaska & Canada
b) Argentina, Bolivia, Chile, Paraguay & Uruguay
c) Argentina, Uruguay & Brazil
d) Australia
e) Central Siberia & Central Asia
f) China (2017)
g) China
h) Central & Eastern Europe
i) Europe
j) India & Middle East
k) India & Pakistan
l) Middle East
m) Northeast Siberia
n) Northern Africa & Middle East
o) Pacific Northwest USA & Canada
p) Russia
q) Southwestern USA & Mexico
r) Siberia
s) South America
t) Northern Southeast Asia
u) Southern Africa
v) Eastern USA
2010s
2020s
2030s
2040s
2050s
2060s
2070s
2080s
2090s
1
2
3
4
Median Heatwave Severity

Supplementary Figure 155: Kernel Density Estimates (KDE) across Decades for Median Heatwave Severity in SSP5-8.5 CMIP6 multi-model ensemble (MME) (Tx95.0p) - Kernel Density Estimates (KDE) of Median Heatwave Severity for different Decades in the CMIP6 multi-model ensemble (MME). Area under each curve is normalized to 1. Median Heatwave Severity of the observed event is indicated by vertical lines in dark blue (Berkeley Earth) and light blue (ERA5).

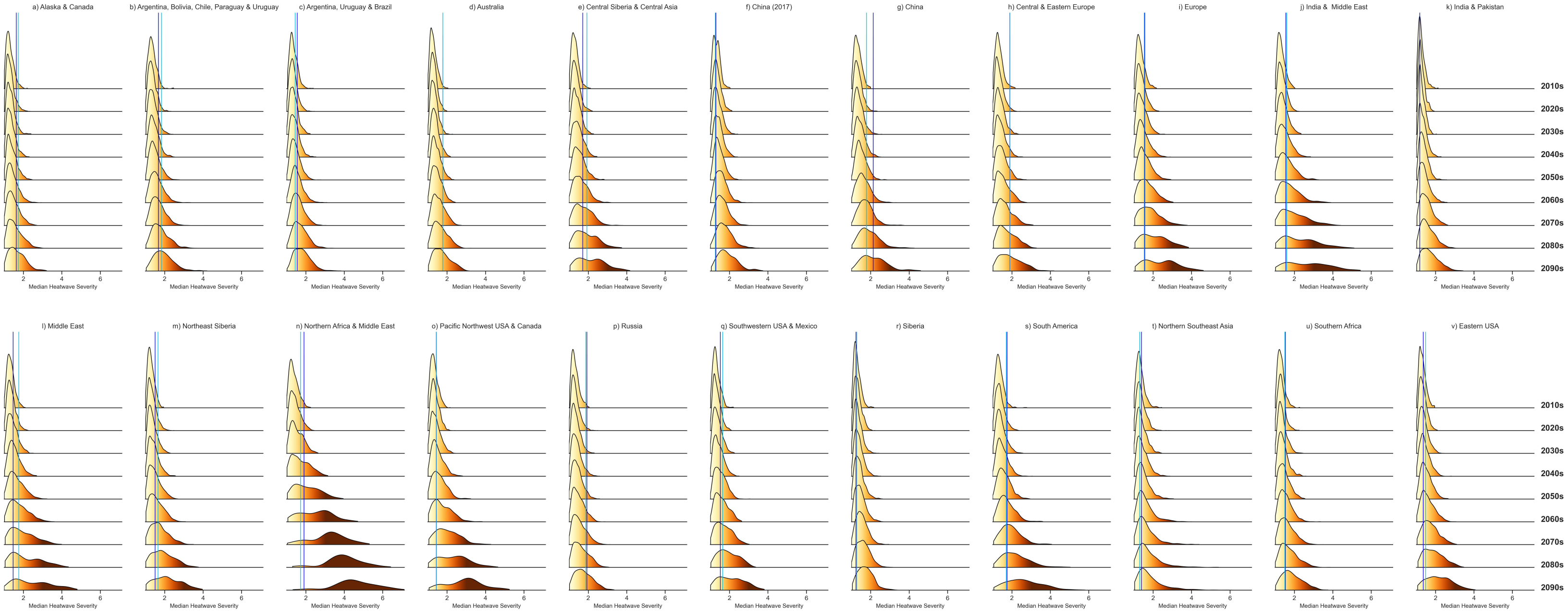

a) Alaska & Canada
b) Argentina, Bolivia, Chile, Paraguay & Uruguay
c) Argentina, Uruguay & Brazil
d) Australia
e) Central Siberia & Central Asia
f) China (2017)
g) China
h) Central & Eastern Europe
i) Europe
j) India & Middle East
k) India & Pakistan
l) Middle East
m) Northeast Siberia
n) Northern Africa & Middle East
o) Pacific Northwest USA & Canada
p) Russia
q) Southwestern USA & Mexico
r) Siberia
s) South America
t) Northern Southeast Asia
u) Southern Africa
v) Eastern USA
2010s
2020s
2030s
2040s
2050s
2060s
2070s
2080s
2090s
2
4
6
Median Heatwave Severity

Supplementary Figure 156: Kernel Density Estimates (KDE) across Decades for Median Heatwave Severity in SSP5-8.5 CMIP6 multi-model ensemble (MME) (Tx97.5p) - Kernel Density Estimates (KDE) of Median Heatwave Severity for different Decades in the CMIP6 multi-model ensemble (MME). Area under each curve is normalized to 1. Median Heatwave Severity of the observed event is indicated by vertical lines in dark blue (Berkeley Earth) and light blue (ERA5).

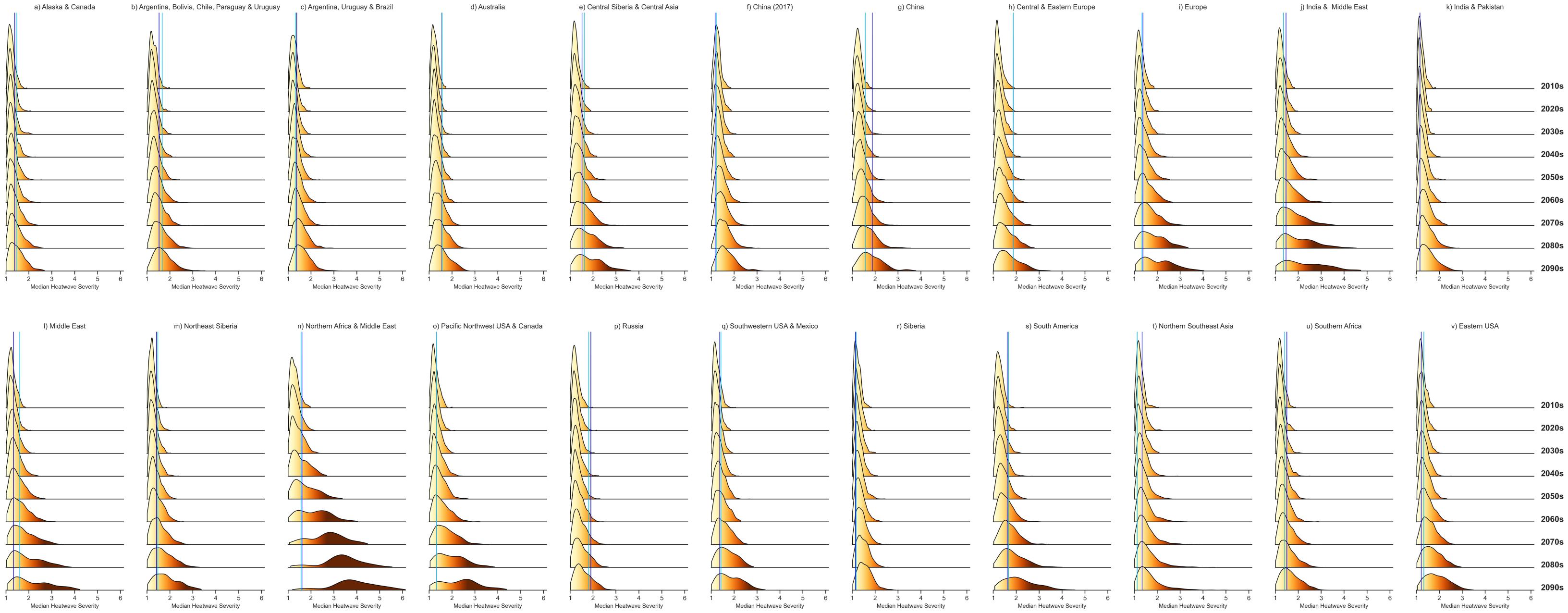

a) Alaska & Canada
b) Argentina, Bolivia, Chile, Paraguay & Uruguay
c) Argentina, Uruguay & Brazil
d) Australia
e) Central Siberia & Central Asia
f) China (2017)
g) China
h) Central & Eastern Europe
i) Europe
j) India & Middle East
k) India & Pakistan
l) Middle East
m) Northeast Siberia
n) Northern Africa & Middle East
o) Pacific Northwest USA & Canada
p) Russia
q) Southwestern USA & Mexico
r) Siberia
s) South America
t) Northern Southeast Asia
u) Southern Africa
v) Eastern USA
2010s
2020s
2030s
2040s
2050s
2060s
2070s
2080s
2090s
Median Heatwave Severity
1 2 3 4 5 6

Supplementary Figure 157: Kernel Density Estimates (KDE) across Decades for Median Heatwave Severity in SSP5-8.5 CMIP6 multi-model ensemble (MME) (Tx99.0p) - Kernel Density Estimates (KDE) of Median Heatwave Severity for different Decades in the CMIP6 multi-model ensemble (MME). Area under each curve is normalized to 1. Median Heatwave Severity of the observed event is indicated by vertical lines in dark blue (Berkeley Earth) and light blue (ERA5).

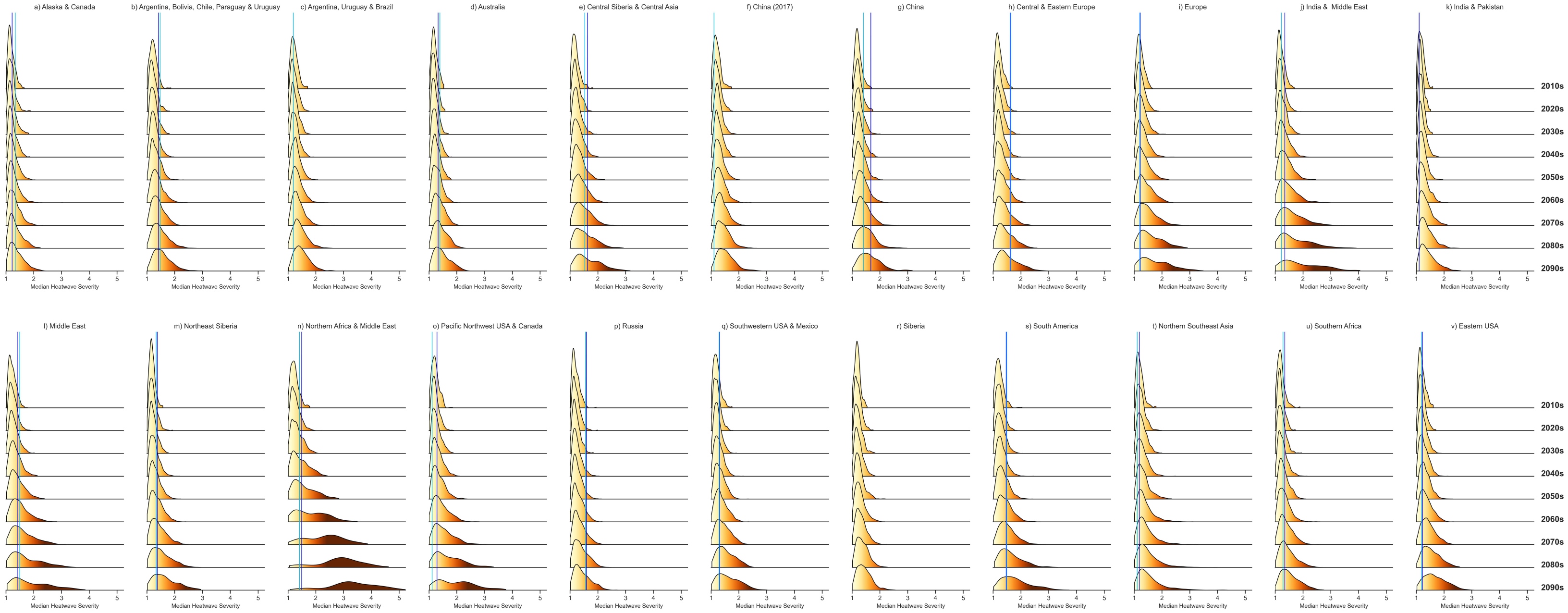

a) Alaska & Canada
b) Argentina, Bolivia, Chile, Paraguay & Uruguay
c) Argentina, Uruguay & Brazil
d) Australia
e) Central Siberia & Central Asia
f) China (2017)
g) China
h) Central & Eastern Europe
i) Europe
j) India & Middle East
k) India & Pakistan
l) Middle East
m) Northeast Siberia
n) Northern Africa & Middle East
o) Pacific Northwest USA & Canada
p) Russia
q) Southwestern USA & Mexico
r) Siberia
s) South America
t) Northern Southeast Asia
u) Southern Africa
v) Eastern USA
2010s
2020s
2030s
2040s
2050s
2060s
2070s
2080s
2090s
Median Heatwave Severity

Supplementary Figure 158: Kernel Density Estimates (KDE) across Decades for Maximum Heatwave Severity in Historical CMIP6 multi-model ensemble (MME) (Tx95.0p) - Kernel Density Estimates (KDE) of Maximum Heatwave Severity for different Decades in the CMIP6 multi-model ensemble (MME). Area under each curve is normalized to 1. Maximum Heatwave Severity of the observed event is indicated by vertical lines in dark blue (Berkeley Earth) and light blue (ERA5).

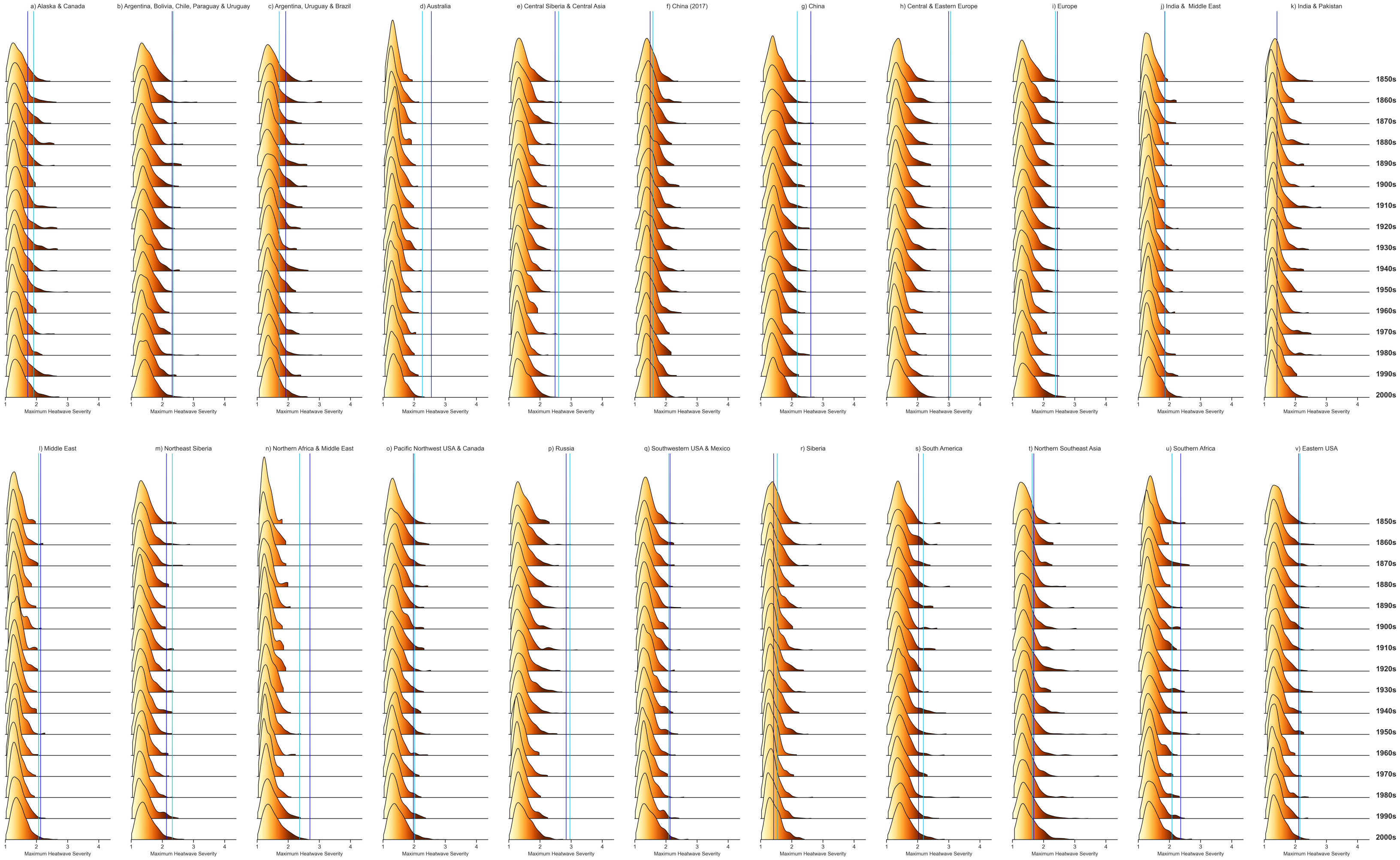

a) Alaska & Canada
b) Argentina, Bolivia, Chile, Paraguay & Uruguay
c) Argentina, Uruguay & Brazil
d) Australia
e) Central Siberia & Central Asia
f) China (2017)
g) China
h) Central & Eastern Europe
i) Europe
j) India & Middle East
k) India & Pakistan
l) Middle East
m) Northeast Siberia
n) Northern Africa & Middle East
o) Pacific Northwest USA & Canada
p) Russia
q) Southwestern USA & Mexico
r) Siberia
s) South America
t) Northern Southeast Asia
u) Southern Africa
v) Eastern USA
1850s
1860s
1870s
1880s
1890s
1900s
1910s
1920s
1930s
1940s
1950s
1960s
1970s
1980s
1990s
2000s
1 2 3 4
Maximum Heatwave Severity

Supplementary Figure 159: Kernel Density Estimates (KDE) across Decades for Maximum Heatwave Severity in Historical CMIP6 multi-model ensemble (MME) (Tx97.5p) - Kernel Density Estimates (KDE) of Maximum Heatwave Severity for different Decades in the CMIP6 multi-model ensemble (MME). Area under each curve is normalized to 1. Maximum Heatwave Severity of the observed event is indicated by vertical lines in dark blue (Berkeley Earth) and light blue (ERA5).

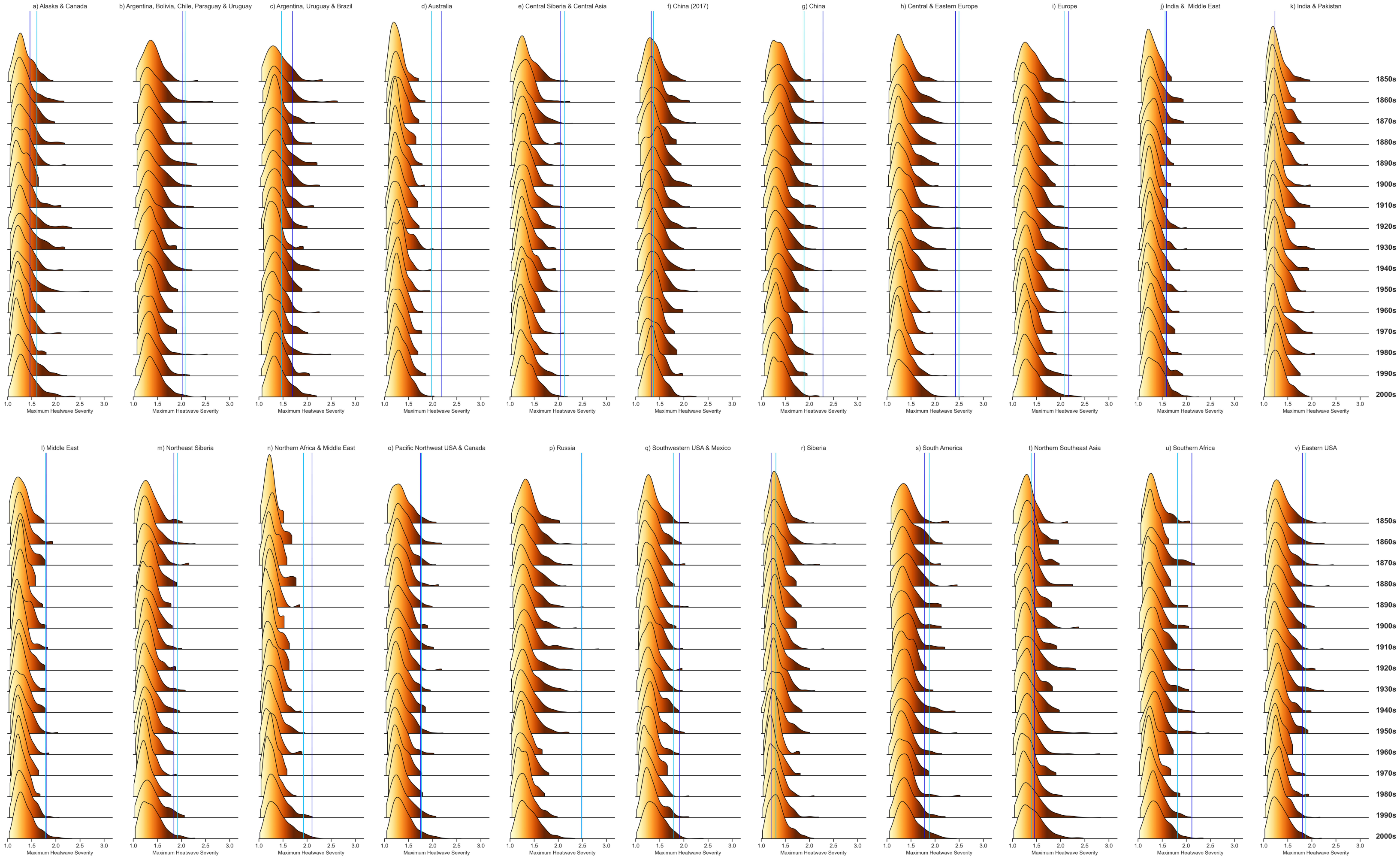
a) Alaska & Canada
b) Argentina, Bolivia, Chile, Paraguay & Uruguay
c) Argentina, Uruguay & Brazil
d) Australia
e) Central Siberia & Central Asia
f) China (2017)
g) China
h) Central & Eastern Europe
i) Europe
j) India & Middle East
k) India & Pakistan
l) Middle East
m) Northeast Siberia
n) Northern Africa & Middle East
o) Pacific Northwest USA & Canada
p) Russia
q) Southwestern USA & Mexico
r) Siberia
s) South America
t) Northern Southeast Asia
u) Southern Africa
v) Eastern USA
1850s
1860s
1870s
1880s
1890s
1900s
1910s
1920s
1930s
1940s
1950s
1960s
1970s
1980s
1990s
2000s
1.0 1.5 2.0 2.5 3.0
Maximum Heatwave Severity

Supplementary Figure 160: Kernel Density Estimates (KDE) across Decades for Maximum Heatwave Severity in Historical CMIP6 multi-model ensemble (MME) (Tx99.0p) - Kernel Density Estimates (KDE) of Maximum Heatwave Severity for different Decades in the CMIP6 multi-model ensemble (MME). Area under each curve is normalized to 1. Maximum Heatwave Severity of the observed event is indicated by vertical lines in dark blue (Berkeley Earth) and light blue (ERA5).

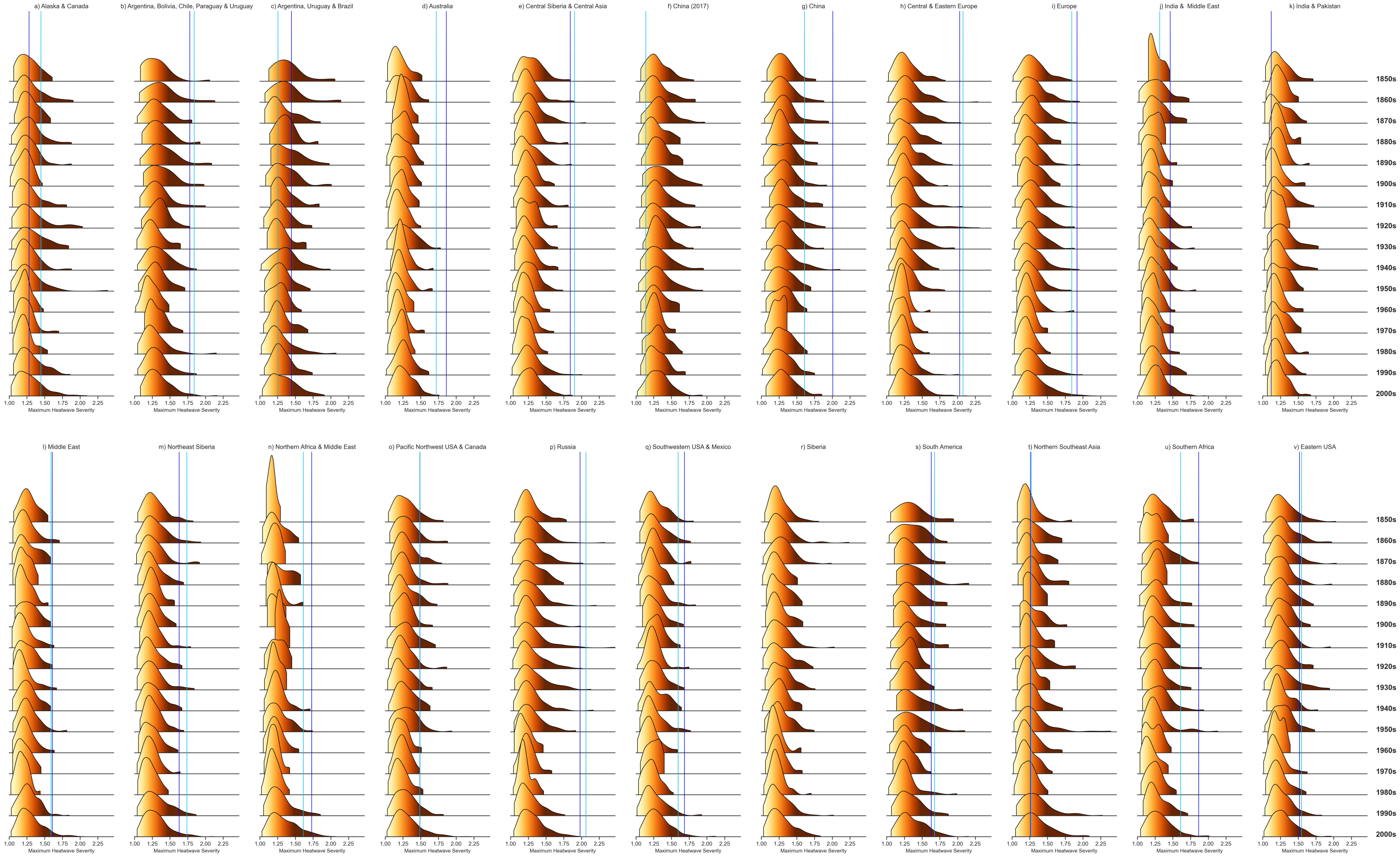
a) Alaska & Canada
b) Argentina, Bolivia, Chile, Paraguay & Uruguay
c) Argentina, Uruguay & Brazil
d) Australia
e) Central Siberia & Central Asia
f) China (2017)
g) China
h) Central & Eastern Europe
i) Europe
j) India & Middle East
k) India & Pakistan
l) Middle East
m) Northeast Siberia
n) Northern Africa & Middle East
o) Pacific Northwest USA & Canada
p) Russia
q) Southwestern USA & Mexico
r) Siberia
s) South America
t) Northern Southeast Asia
u) Southern Africa
v) Eastern USA
1850s
1860s
1870s
1880s
1890s
1900s
1910s
1920s
1930s
1940s
1950s
1960s
1970s
1980s
1990s
2000s
1.00
1.25
1.50
1.75
2.00
2.25
Maximum Heatwave Severity

Supplementary Figure 161: Kernel Density Estimates (KDE) across Decades for Maximum Heatwave Severity in SSP1-2.6 CMIP6 multi-model ensemble (MME) (Tx95.0p) - Kernel Density Estimates (KDE) of Maximum Heatwave Severity for different Decades in the CMIP6 multi-model ensemble (MME). Area under each curve is normalized to 1. Maximum Heatwave Severity of the observed event is indicated by vertical lines in dark blue (Berkeley Earth) and light blue (ERA5).

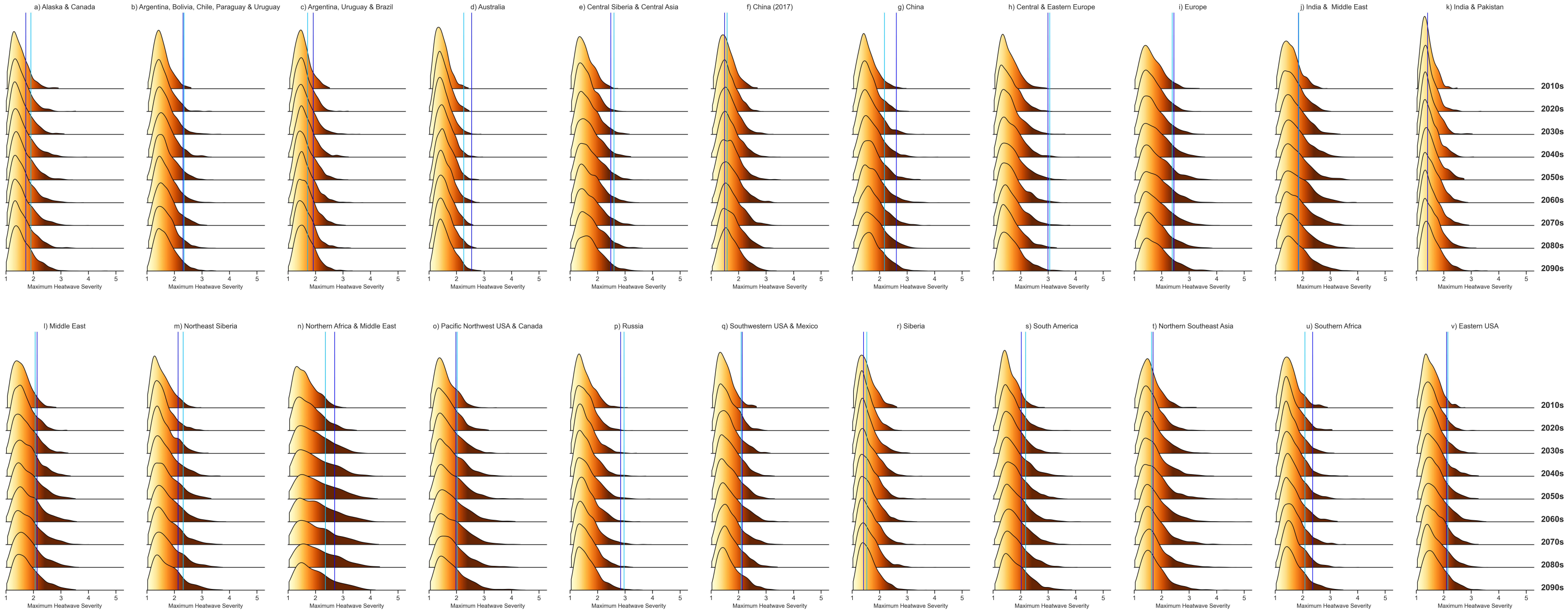

a) Alaska & Canada
b) Argentina, Bolivia, Chile, Paraguay & Uruguay
c) Argentina, Uruguay & Brazil
d) Australia
e) Central Siberia & Central Asia
f) China (2017)
g) China
h) Central & Eastern Europe
i) Europe
j) India & Middle East
k) India & Pakistan
l) Middle East
m) Northeast Siberia
n) Northern Africa & Middle East
o) Pacific Northwest USA & Canada
p) Russia
q) Southwestern USA & Mexico
r) Siberia
s) South America
t) Northern Southeast Asia
u) Southern Africa
v) Eastern USA
2010s
2020s
2030s
2040s
2050s
2060s
2070s
2080s
2090s
1
2
3
4
5
Maximum Heatwave Severity

Supplementary Figure 162: Kernel Density Estimates (KDE) across Decades for Maximum Heatwave Severity in SSP1-2.6 CMIP6 multi-model ensemble (MME) (Tx97.5p) - Kernel Density Estimates (KDE) of Maximum Heatwave Severity for different Decades in the CMIP6 multi-model ensemble (MME). Area under each curve is normalized to 1. Maximum Heatwave Severity of the observed event is indicated by vertical lines in dark blue (Berkeley Earth) and light blue (ERA5).

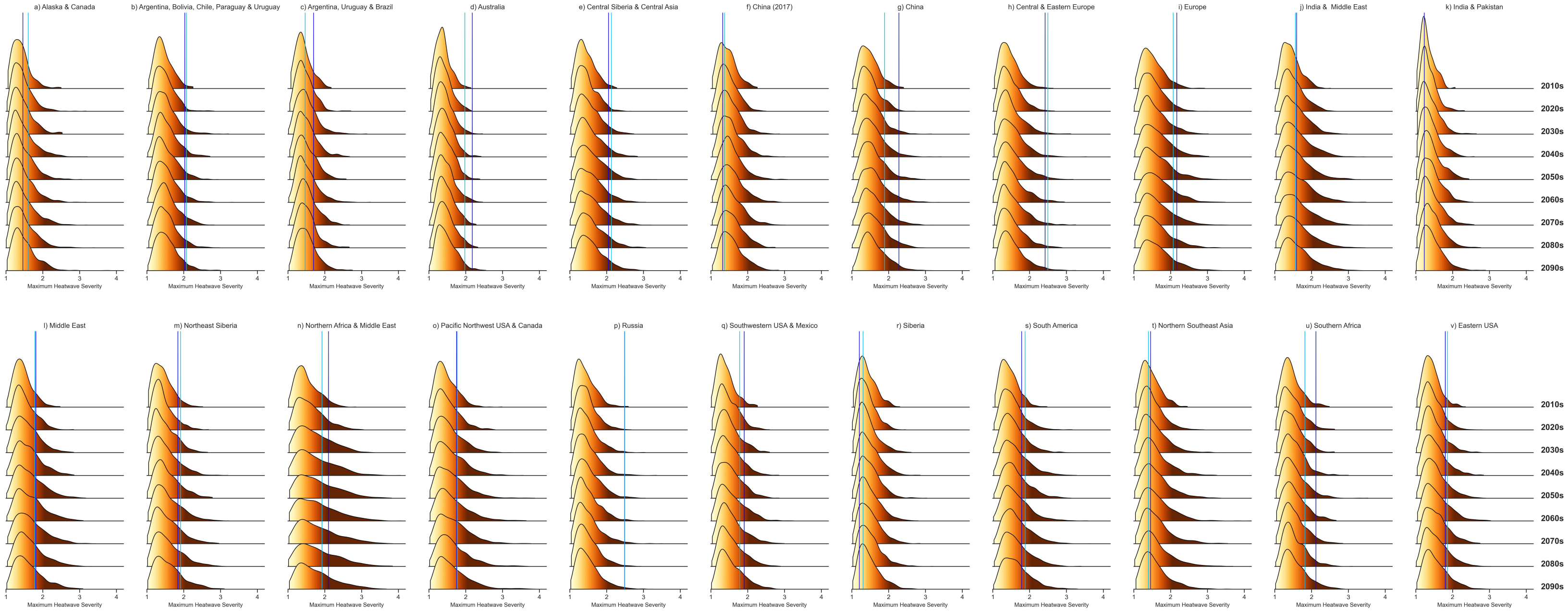

a) Alaska & Canada
b) Argentina, Bolivia, Chile, Paraguay & Uruguay
c) Argentina, Uruguay & Brazil
d) Australia
e) Central Siberia & Central Asia
f) China (2017)
g) China
h) Central & Eastern Europe
i) Europe
j) India & Middle East
k) India & Pakistan
l) Middle East
m) Northeast Siberia
n) Northern Africa & Middle East
o) Pacific Northwest USA & Canada
p) Russia
q) Southwestern USA & Mexico
r) Siberia
s) South America
t) Northern Southeast Asia
u) Southern Africa
v) Eastern USA
2010s
2020s
2030s
2040s
2050s
2060s
2070s
2080s
2090s
Maximum Heatwave Severity
1
2
3
4

Supplementary Figure 163: Kernel Density Estimates (KDE) across Decades for Maximum Heatwave Severity in SSP1-2.6 CMIP6 multi-model ensemble (MME) (Tx99.0p) - Kernel Density Estimates (KDE) of Maximum Heatwave Severity for different Decades in the CMIP6 multi-model ensemble (MME). Area under each curve is normalized to 1. Maximum Heatwave Severity of the observed event is indicated by vertical lines in dark blue (Berkeley Earth) and light blue (ERA5).

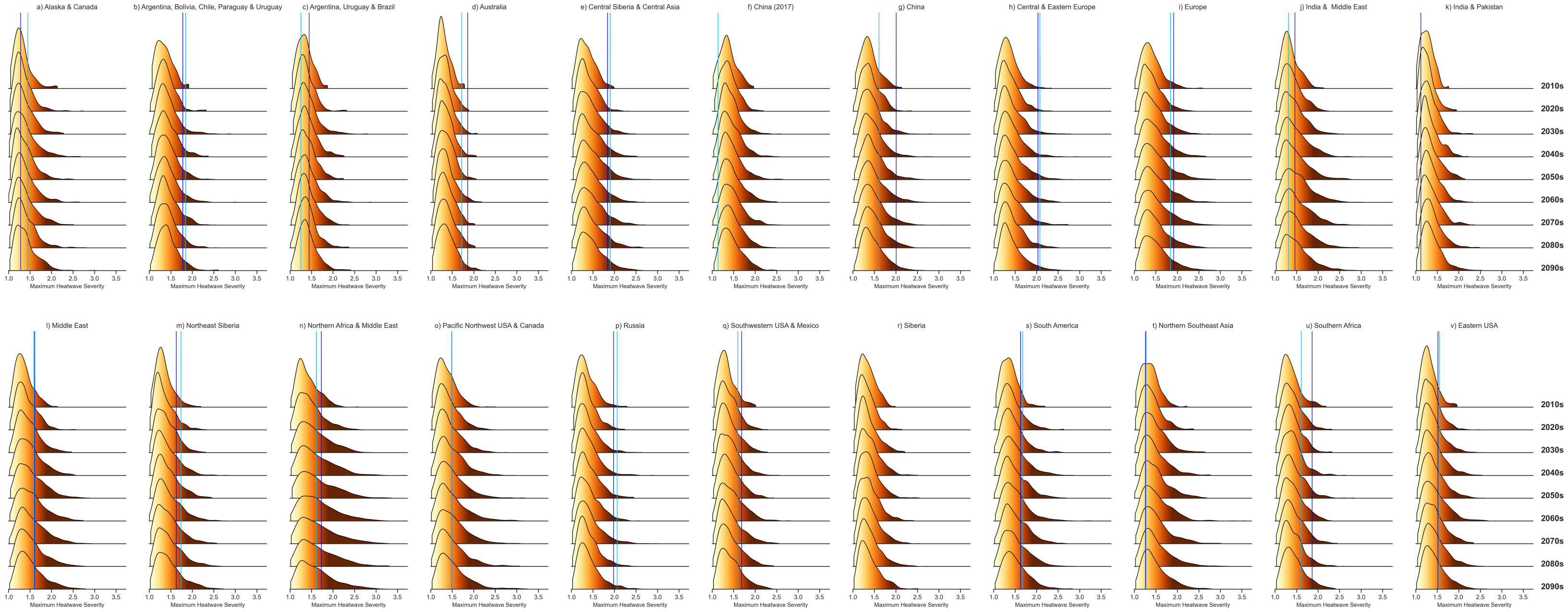
a) Alaska & Canada
b) Argentina, Bolivia, Chile, Paraguay & Uruguay
c) Argentina, Uruguay & Brazil
d) Australia
e) Central Siberia & Central Asia
f) China (2017)
g) China
h) Central & Eastern Europe
i) Europe
j) India & Middle East
k) India & Pakistan
l) Middle East
m) Northeast Siberia
n) Northern Africa & Middle East
o) Pacific Northwest USA & Canada
p) Russia
q) Southwestern USA & Mexico
r) Siberia
s) South America
t) Northern Southeast Asia
u) Southern Africa
v) Eastern USA
2010s
2020s
2030s
2040s
2050s
2060s
2070s
2080s
2090s
1.0 1.5 2.0 2.5 3.0 3.5
Maximum Heatwave Severity

Supplementary Figure 164: Kernel Density Estimates (KDE) across Decades for Maximum Heatwave Severity in SSP2-4.5 CMIP6 multi-model ensemble (MME) (Tx95.0p) - Kernel Density Estimates (KDE) of Maximum Heatwave Severity for different Decades in the CMIP6 multi-model ensemble (MME). Area under each curve is normalized to 1. Maximum Heatwave Severity of the observed event is indicated by vertical lines in dark blue (Berkeley Earth) and light blue (ERA5).

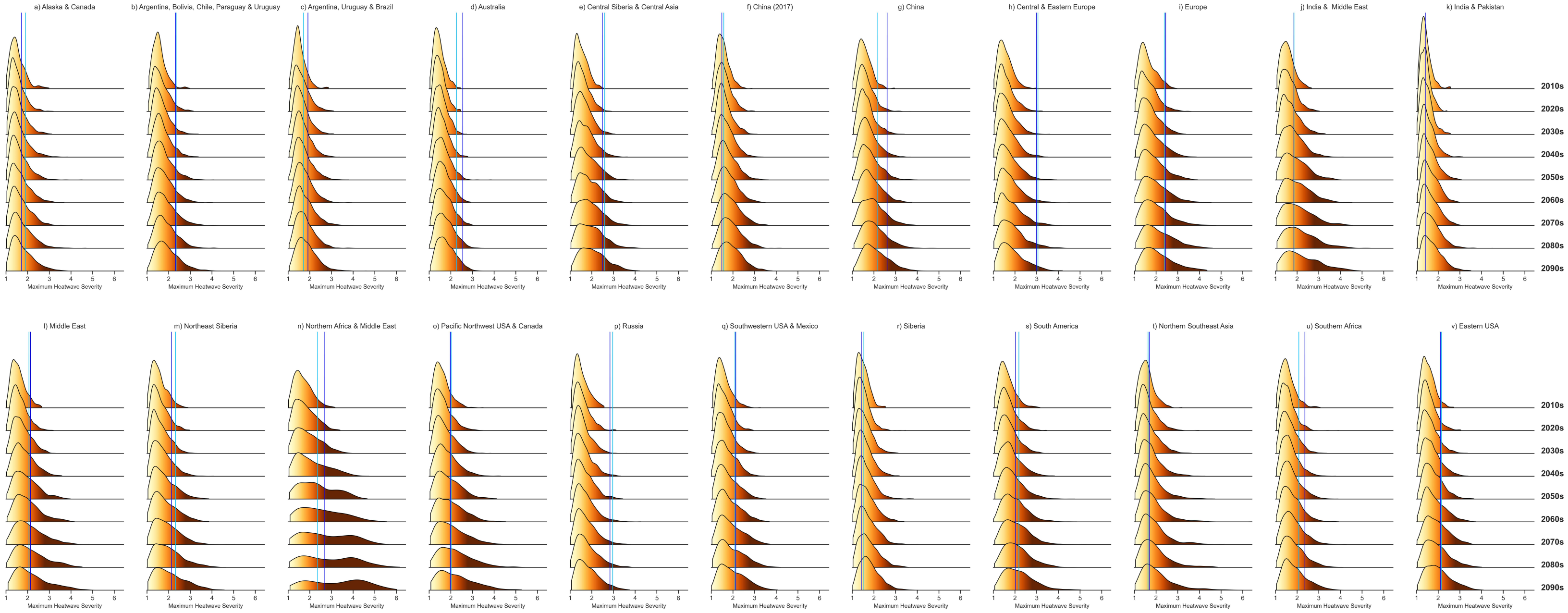

a) Alaska & Canada
b) Argentina, Bolivia, Chile, Paraguay & Uruguay
c) Argentina, Uruguay & Brazil
d) Australia
e) Central Siberia & Central Asia
f) China (2017)
g) China
h) Central & Eastern Europe
i) Europe
j) India & Middle East
k) India & Pakistan
l) Middle East
m) Northeast Siberia
n) Northern Africa & Middle East
o) Pacific Northwest USA & Canada
p) Russia
q) Southwestern USA & Mexico
r) Siberia
s) South America
t) Northern Southeast Asia
u) Southern Africa
v) Eastern USA
2010s
2020s
2030s
2040s
2050s
2060s
2070s
2080s
2090s
Maximum Heatwave Severity
1 2 3 4 5 6

Supplementary Figure 165: Kernel Density Estimates (KDE) across Decades for Maximum Heatwave Severity in SSP2-4.5 CMIP6 multi-model ensemble (MME) (Tx97.5p) - Kernel Density Estimates (KDE) of Maximum Heatwave Severity for different Decades in the CMIP6 multi-model ensemble (MME). Area under each curve is normalized to 1. Maximum Heatwave Severity of the observed event is indicated by vertical lines in dark blue (Berkeley Earth) and light blue (ERA5).

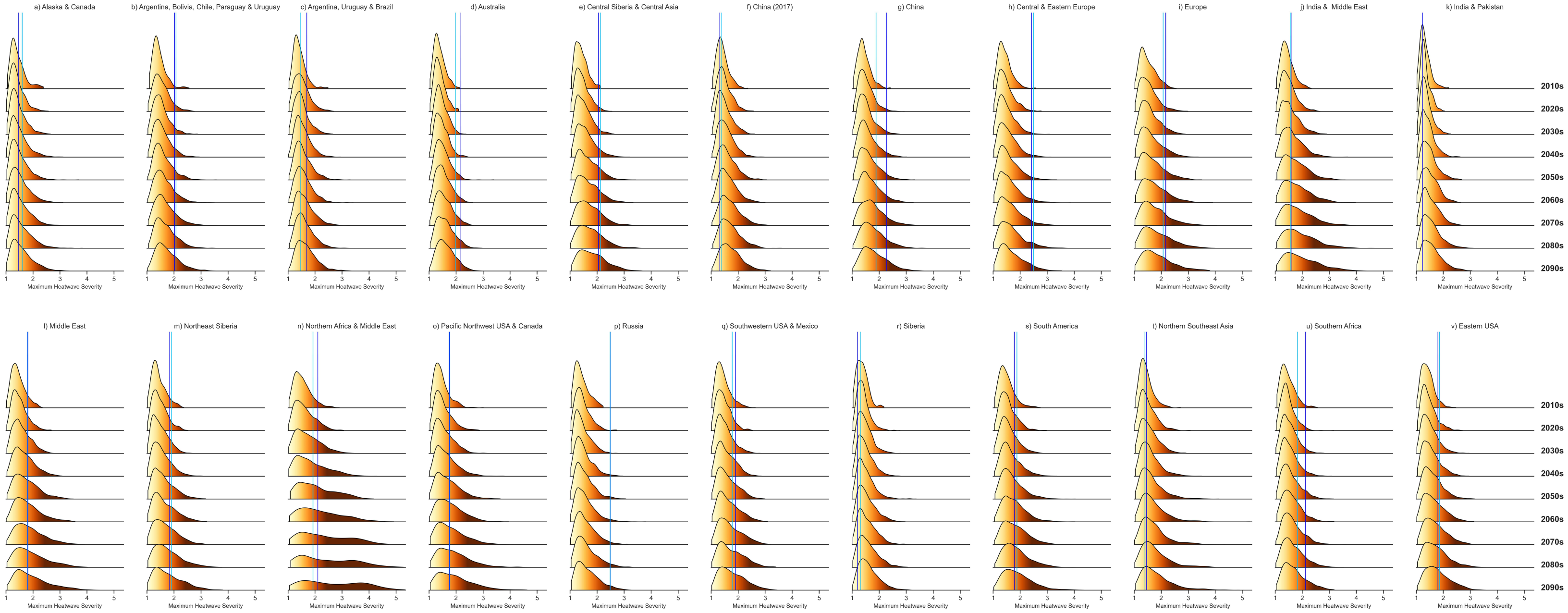

a) Alaska & Canada
b) Argentina, Bolivia, Chile, Paraguay & Uruguay
c) Argentina, Uruguay & Brazil
d) Australia
e) Central Siberia & Central Asia
f) China (2017)
g) China
h) Central & Eastern Europe
i) Europe
j) India & Middle East
k) India & Pakistan
l) Middle East
m) Northeast Siberia
n) Northern Africa & Middle East
o) Pacific Northwest USA & Canada
p) Russia
q) Southwestern USA & Mexico
r) Siberia
s) South America
t) Northern Southeast Asia
u) Southern Africa
v) Eastern USA
2010s
2020s
2030s
2040s
2050s
2060s
2070s
2080s
2090s
1 2 3 4 5
Maximum Heatwave Severity

Supplementary Figure 166: Kernel Density Estimates (KDE) across Decades for Maximum Heatwave Severity in SSP2-4.5 CMIP6 multi-model ensemble (MME) (Tx99.0p) - Kernel Density Estimates (KDE) of Maximum Heatwave Severity for different Decades in the CMIP6 multi-model ensemble (MME). Area under each curve is normalized to 1. Maximum Heatwave Severity of the observed event is indicated by vertical lines in dark blue (Berkeley Earth) and light blue (ERA5).

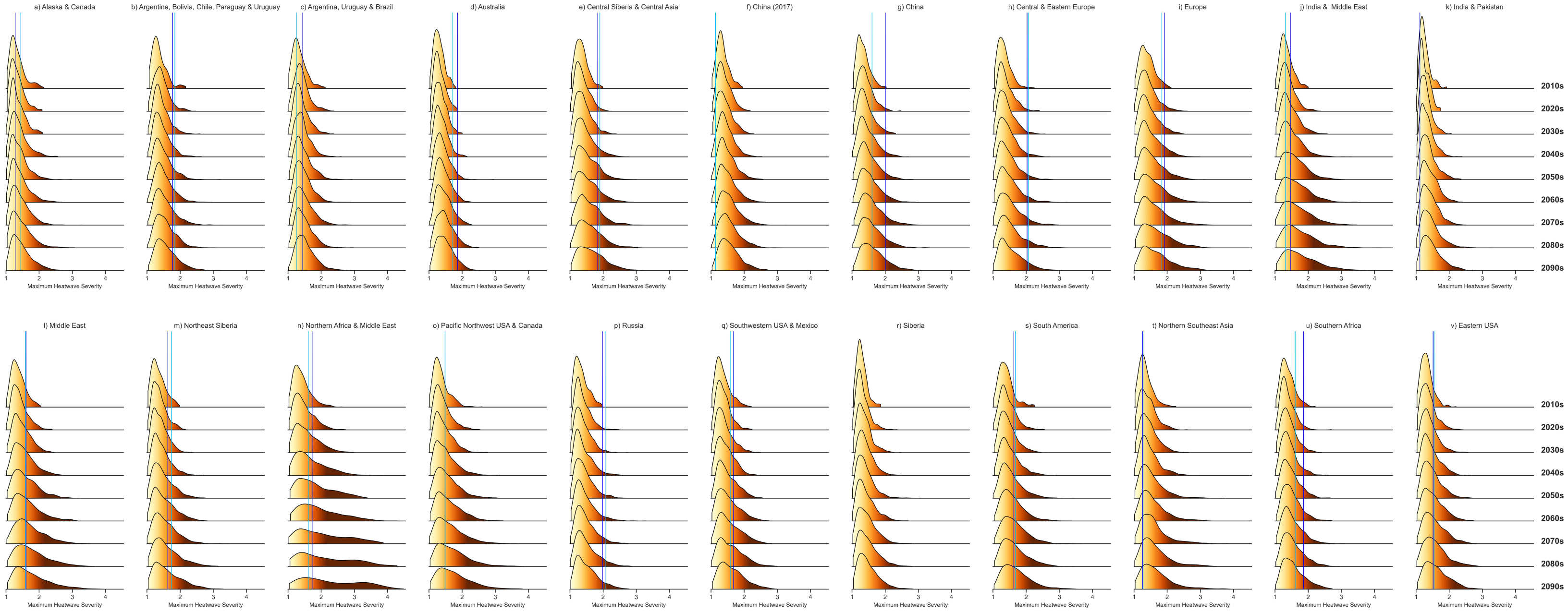

a) Alaska & Canada
b) Argentina, Bolivia, Chile, Paraguay & Uruguay
c) Argentina, Uruguay & Brazil
d) Australia
e) Central Siberia & Central Asia
f) China (2017)
g) China
h) Central & Eastern Europe
i) Europe
j) India & Middle East
k) India & Pakistan
l) Middle East
m) Northeast Siberia
n) Northern Africa & Middle East
o) Pacific Northwest USA & Canada
p) Russia
q) Southwestern USA & Mexico
r) Siberia
s) South America
t) Northern Southeast Asia
u) Southern Africa
v) Eastern USA
2010s
2020s
2030s
2040s
2050s
2060s
2070s
2080s
2090s
Maximum Heatwave Severity
1
2
3
4

Supplementary Figure 167: Kernel Density Estimates (KDE) across Decades for Maximum Heatwave Severity in SSP3-7.0 CMIP6 multi-model ensemble (MME) (Tx95.0p) - Kernel Density Estimates (KDE) of Maximum Heatwave Severity for different Decades in the CMIP6 multi-model ensemble (MME). Area under each curve is normalized to 1. Maximum Heatwave Severity of the observed event is indicated by vertical lines in dark blue (Berkeley Earth) and light blue (ERA5).

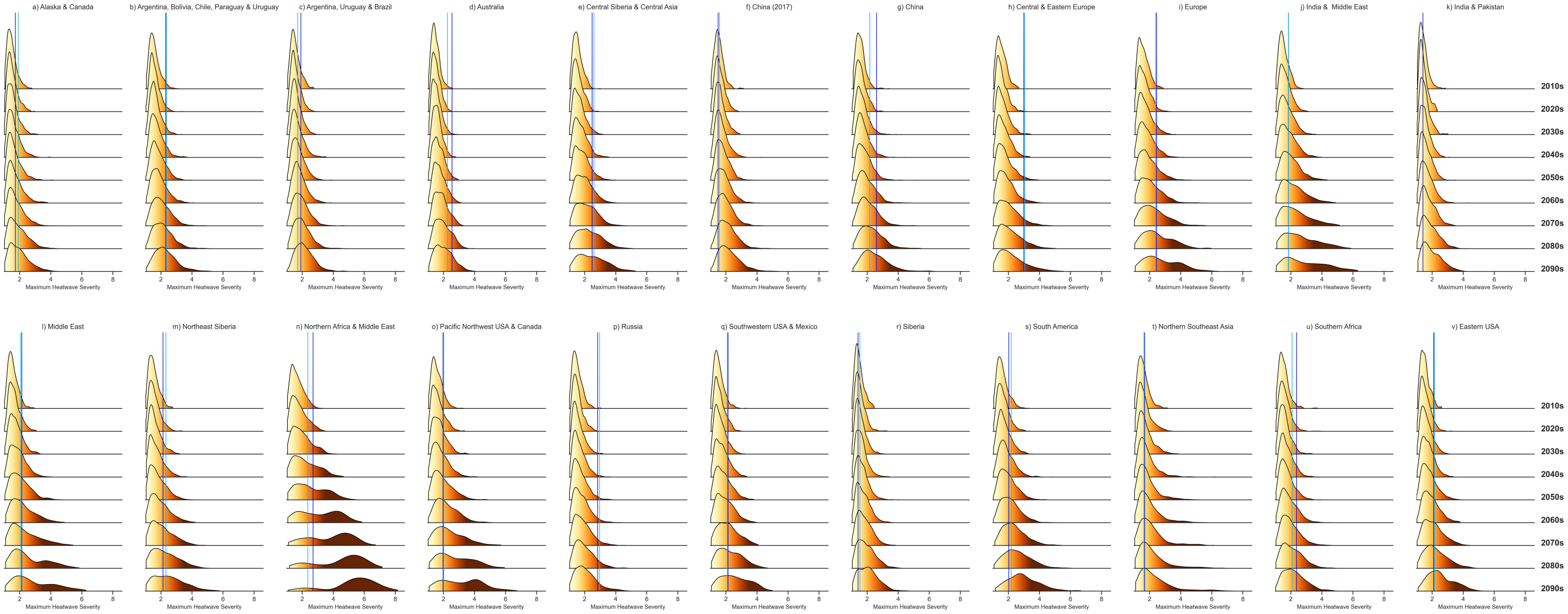
a) Alaska & Canada
b) Argentina, Bolivia, Chile, Paraguay & Uruguay
c) Argentina, Uruguay & Brazil
d) Australia
e) Central Siberia & Central Asia
f) China (2017)
g) China
h) Central & Eastern Europe
i) Europe
j) India & Middle East
k) India & Pakistan
l) Middle East
m) Northeast Siberia
n) Northern Africa & Middle East
o) Pacific Northwest USA & Canada
p) Russia
q) Southwestern USA & Mexico
r) Siberia
s) South America
t) Northern Southeast Asia
u) Southern Africa
v) Eastern USA
2010s
2020s
2030s
2040s
2050s
2060s
2070s
2080s
2090s
2
4
6
8
Maximum Heatwave Severity

Supplementary Figure 168: Kernel Density Estimates (KDE) across Decades for Maximum Heatwave Severity in SSP3-7.0 CMIP6 multi-model ensemble (MME) (Tx97.5p) - Kernel Density Estimates (KDE) of Maximum Heatwave Severity for different Decades in the CMIP6 multi-model ensemble (MME). Area under each curve is normalized to 1. Maximum Heatwave Severity of the observed event is indicated by vertical lines in dark blue (Berkeley Earth) and light blue (ERA5).

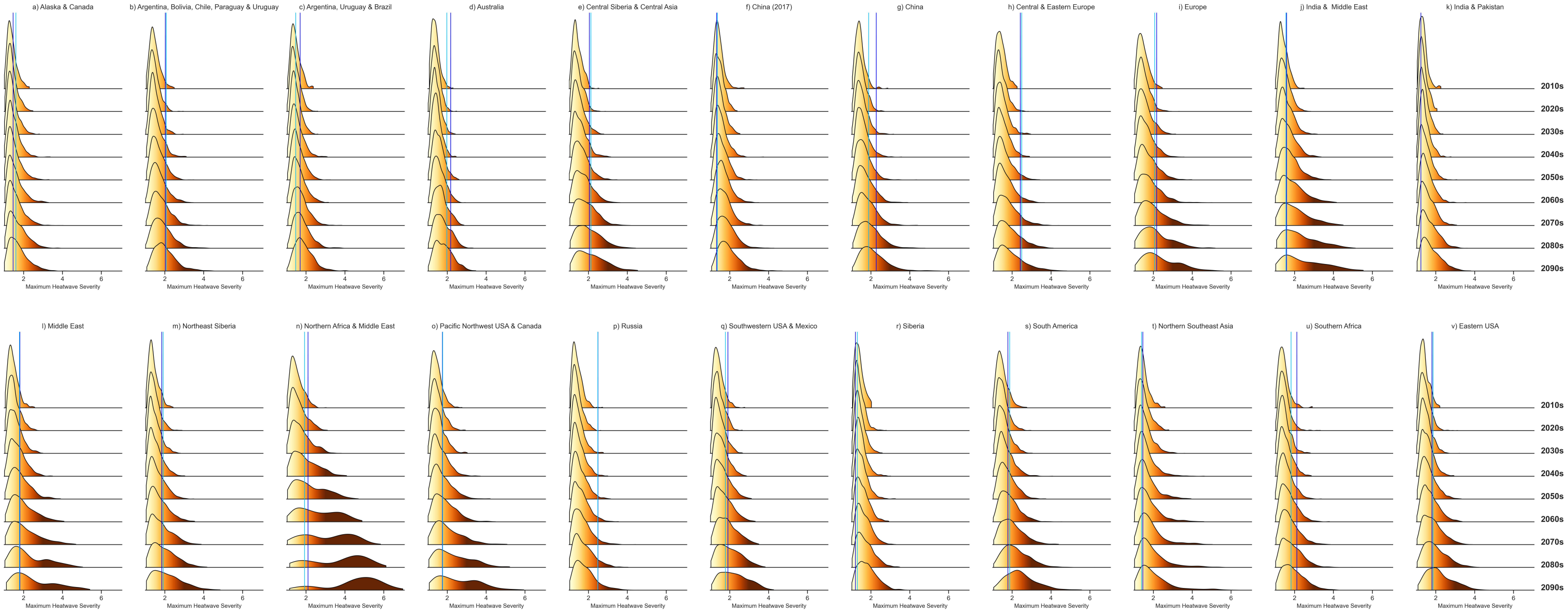

a) Alaska & Canada
b) Argentina, Bolivia, Chile, Paraguay & Uruguay
c) Argentina, Uruguay & Brazil
d) Australia
e) Central Siberia & Central Asia
f) China (2017)
g) China
h) Central & Eastern Europe
i) Europe
j) India & Middle East
k) India & Pakistan
l) Middle East
m) Northeast Siberia
n) Northern Africa & Middle East
o) Pacific Northwest USA & Canada
p) Russia
q) Southwestern USA & Mexico
r) Siberia
s) South America
t) Northern Southeast Asia
u) Southern Africa
v) Eastern USA
2010s
2020s
2030s
2040s
2050s
2060s
2070s
2080s
2090s
2
4
6
Maximum Heatwave Severity

Supplementary Figure 169: Kernel Density Estimates (KDE) across Decades for Maximum Heatwave Severity in SSP3-7.0 CMIP6 multi-model ensemble (MME) (Tx99.0p) - Kernel Density Estimates (KDE) of Maximum Heatwave Severity for different Decades in the CMIP6 multi-model ensemble (MME). Area under each curve is normalized to 1. Maximum Heatwave Severity of the observed event is indicated by vertical lines in dark blue (Berkeley Earth) and light blue (ERA5).

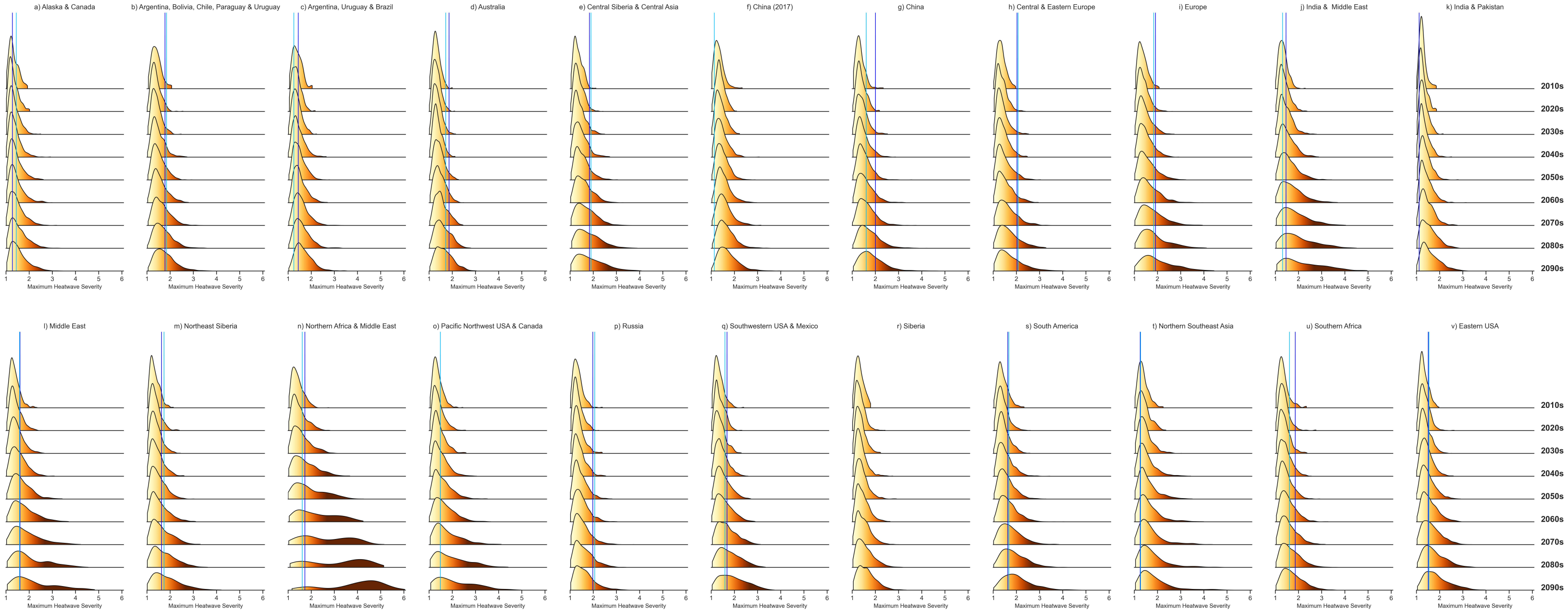
a) Alaska & Canada
b) Argentina, Bolivia, Chile, Paraguay & Uruguay
c) Argentina, Uruguay & Brazil
d) Australia
e) Central Siberia & Central Asia
f) China (2017)
g) China
h) Central & Eastern Europe
i) Europe
j) India & Middle East
k) India & Pakistan
l) Middle East
m) Northeast Siberia
n) Northern Africa & Middle East
o) Pacific Northwest USA & Canada
p) Russia
q) Southwestern USA & Mexico
r) Siberia
s) South America
t) Northern Southeast Asia
u) Southern Africa
v) Eastern USA
2010s
2020s
2030s
2040s
2050s
2060s
2070s
2080s
2090s
Maximum Heatwave Severity
1 2 3 4 5 6

Supplementary Figure 170: Kernel Density Estimates (KDE) across Decades for Maximum Heatwave Severity in SSP5-8.5 CMIP6 multi-model ensemble (MME) (Tx95.0p) - Kernel Density Estimates (KDE) of Maximum Heatwave Severity for different Decades in the CMIP6 multi-model ensemble (MME). Area under each curve is normalized to 1. Maximum Heatwave Severity of the observed event is indicated by vertical lines in dark blue (Berkeley Earth) and light blue (ERA5).

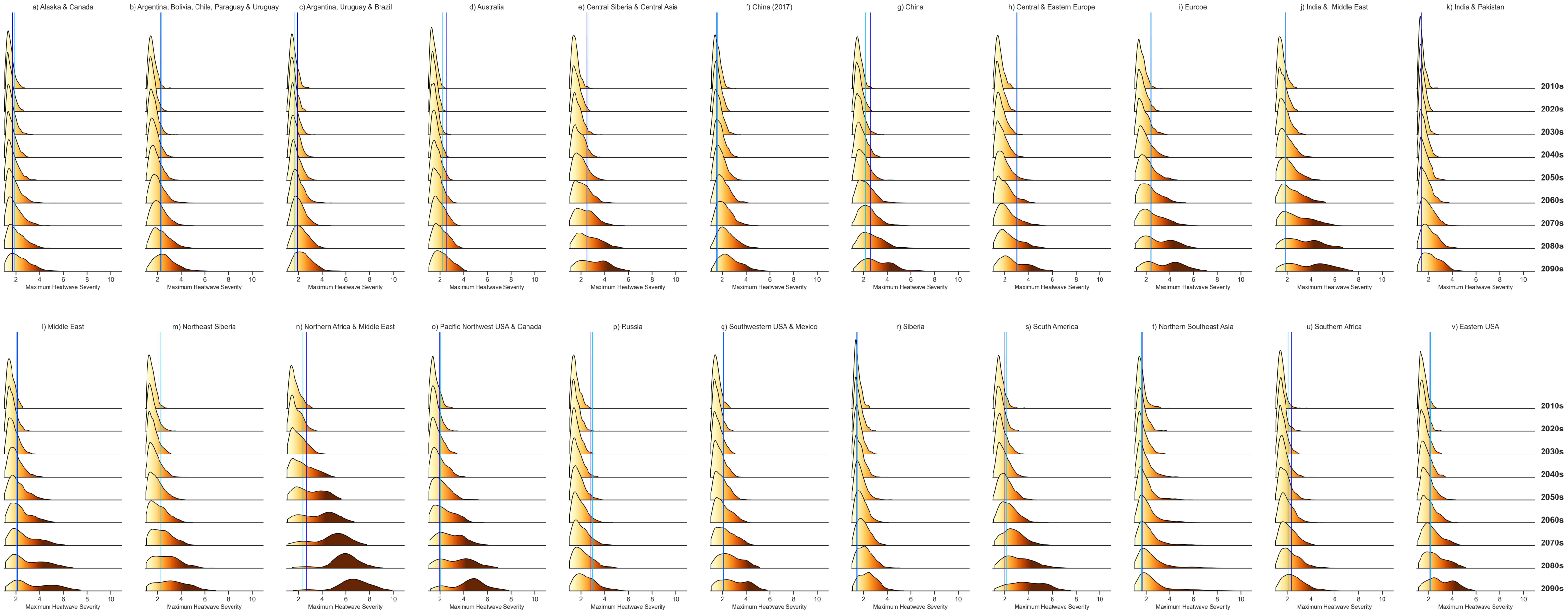

a) Alaska & Canada
b) Argentina, Bolivia, Chile, Paraguay & Uruguay
c) Argentina, Uruguay & Brazil
d) Australia
e) Central Siberia & Central Asia
f) China (2017)
g) China
h) Central & Eastern Europe
i) Europe
j) India & Middle East
k) India & Pakistan
l) Middle East
m) Northeast Siberia
n) Northern Africa & Middle East
o) Pacific Northwest USA & Canada
p) Russia
q) Southwestern USA & Mexico
r) Siberia
s) South America
t) Northern Southeast Asia
u) Southern Africa
v) Eastern USA
2010s
2020s
2030s
2040s
2050s
2060s
2070s
2080s
2090s
Maximum Heatwave Severity
2 4 6 8 10

Supplementary Figure 171: Kernel Density Estimates (KDE) across Decades for Maximum Heatwave Severity in SSP5-8.5 CMIP6 multi-model ensemble (MME) (Tx97.5p) - Kernel Density Estimates (KDE) of Maximum Heatwave Severity for different Decades in the CMIP6 multi-model ensemble (MME). Area under each curve is normalized to 1. Maximum Heatwave Severity of the observed event is indicated by vertical lines in dark blue (Berkeley Earth) and light blue (ERA5).

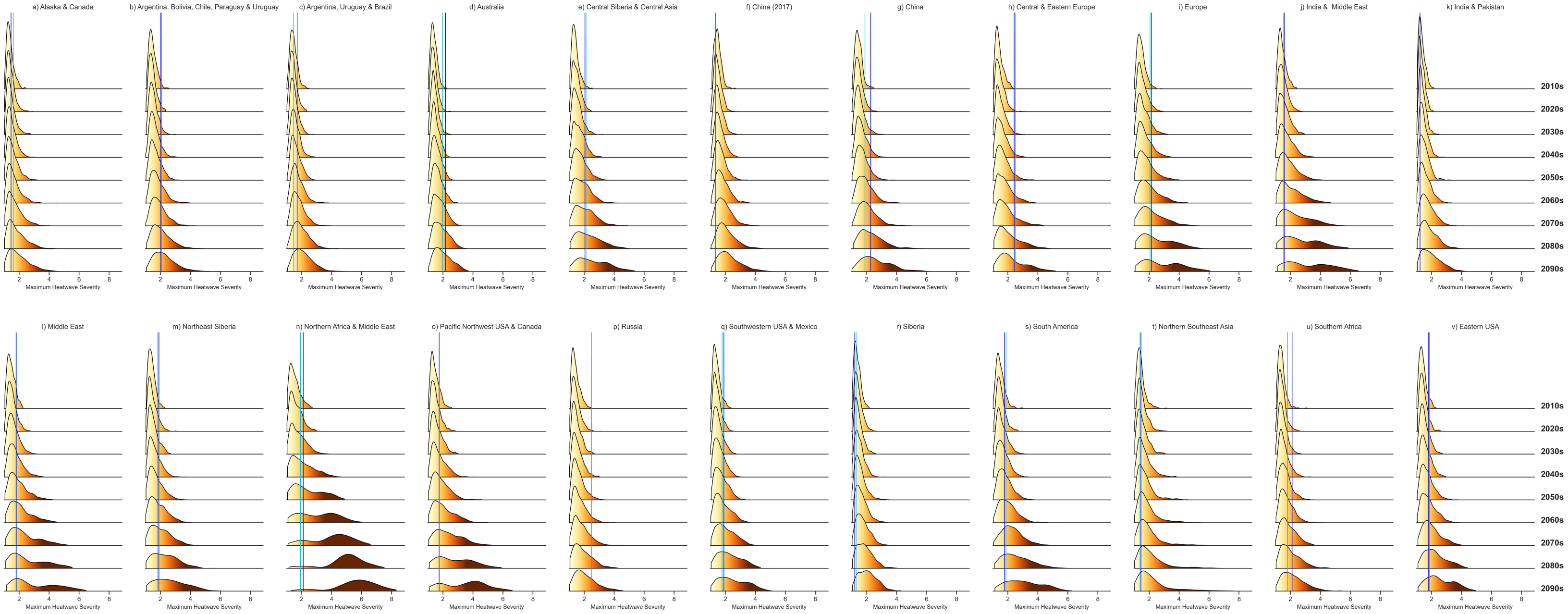

a) Alaska & Canada
b) Argentina, Bolivia, Chile, Paraguay & Uruguay
c) Argentina, Uruguay & Brazil
d) Australia
e) Central Siberia & Central Asia
f) China (2017)
g) China
h) Central & Eastern Europe
i) Europe
j) India & Middle East
k) India & Pakistan
l) Middle East
m) Northeast Siberia
n) Northern Africa & Middle East
o) Pacific Northwest USA & Canada
p) Russia
q) Southwestern USA & Mexico
r) Siberia
s) South America
t) Northern Southeast Asia
u) Southern Africa
v) Eastern USA
2010s
2020s
2030s
2040s
2050s
2060s
2070s
2080s
2090s
2
4
6
8
Maximum Heatwave Severity

Supplementary Figure 172: Kernel Density Estimates (KDE) across Decades for Maximum Heatwave Severity in SSP5-8.5 CMIP6 multi-model ensemble (MME) (Tx99.0p) - Kernel Density Estimates (KDE) of Maximum Heatwave Severity for different Decades in the CMIP6 multi-model ensemble (MME). Area under each curve is normalized to 1. Maximum Heatwave Severity of the observed event is indicated by vertical lines in dark blue (Berkeley Earth) and light blue (ERA5).

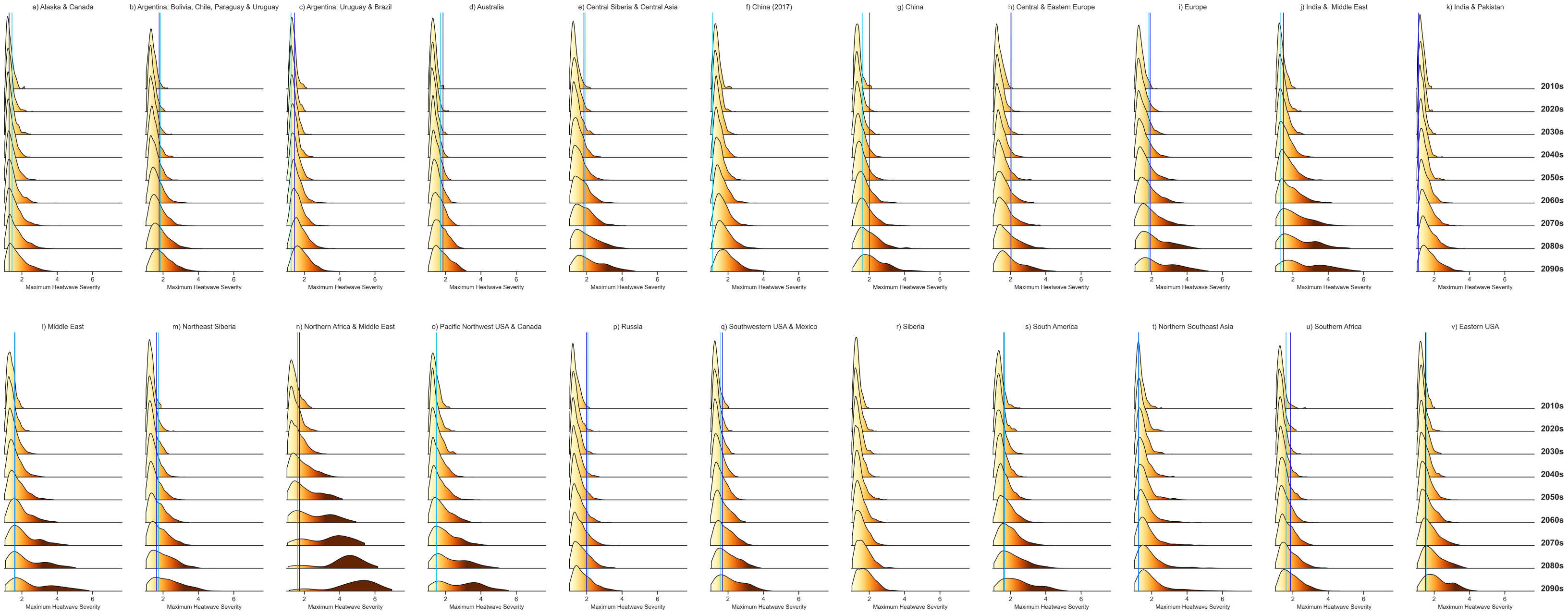
a) Alaska & Canada
b) Argentina, Bolivia, Chile, Paraguay & Uruguay
c) Argentina, Uruguay & Brazil
d) Australia
e) Central Siberia & Central Asia
f) China (2017)
g) China
h) Central & Eastern Europe
i) Europe
j) India & Middle East
k) India & Pakistan
l) Middle East
m) Northeast Siberia
n) Northern Africa & Middle East
o) Pacific Northwest USA & Canada
p) Russia
q) Southwestern USA & Mexico
r) Siberia
s) South America
t) Northern Southeast Asia
u) Southern Africa
v) Eastern USA
2010s
2020s
2030s
2040s
2050s
2060s
2070s
2080s
2090s
Maximum Heatwave Severity
2
4
6

Supplementary Figure 173: Kernel Density Estimates (KDE) across Decades for Median Heatwave Area (%) in Historical CMIP6 multi-model ensemble (MME) (Tx95.0p) - Kernel Density Estimates (KDE) of Median Heatwave Area (%) for different Decades in the CMIP6 multi-model ensemble (MME). Area under each curve is normalized to 1. Median Heatwave Area (%) of the observed event is indicated by vertical lines in dark blue (Berkeley Earth) and light blue (ERA5).

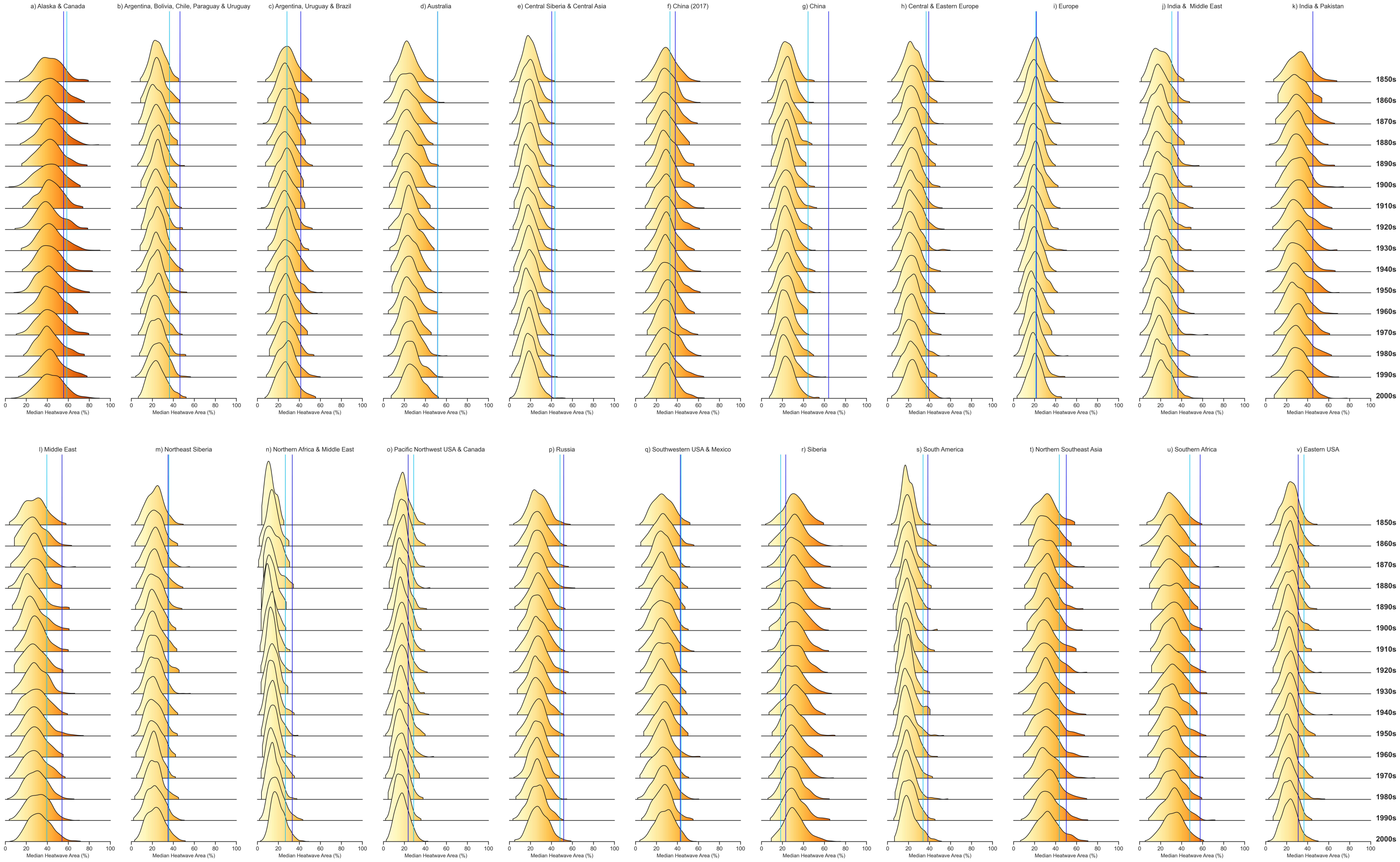
a) Alaska & Canada
b) Argentina, Bolivia, Chile, Paraguay & Uruguay
c) Argentina, Uruguay & Brazil
d) Australia
e) Central Siberia & Central Asia
f) China (2017)
g) China
h) Central & Eastern Europe
i) Europe
j) India & Middle East
k) India & Pakistan
l) Middle East
m) Northeast Siberia
n) Northern Africa & Middle East
o) Pacific Northwest USA & Canada
p) Russia
q) Southwestern USA & Mexico
r) Siberia
s) South America
t) Northern Southeast Asia
u) Southern Africa
v) Eastern USA
1850s
1860s
1870s
1880s
1890s
1900s
1910s
1920s
1930s
1940s
1950s
1960s
1970s
1980s
1990s
2000s
0 20 40 60 80 100
Median Heatwave Area (%)

Supplementary Figure 174: Kernel Density Estimates (KDE) across Decades for Median Heatwave Area (%) in Historical CMIP6 multi-model ensemble (MME) (Tx97.5p) - Kernel Density Estimates (KDE) of Median Heatwave Area (%) for different Decades in the CMIP6 multi-model ensemble (MME). Area under each curve is normalized to 1. Median Heatwave Area (%) of the observed event is indicated by vertical lines in dark blue (Berkeley Earth) and light blue (ERA5).

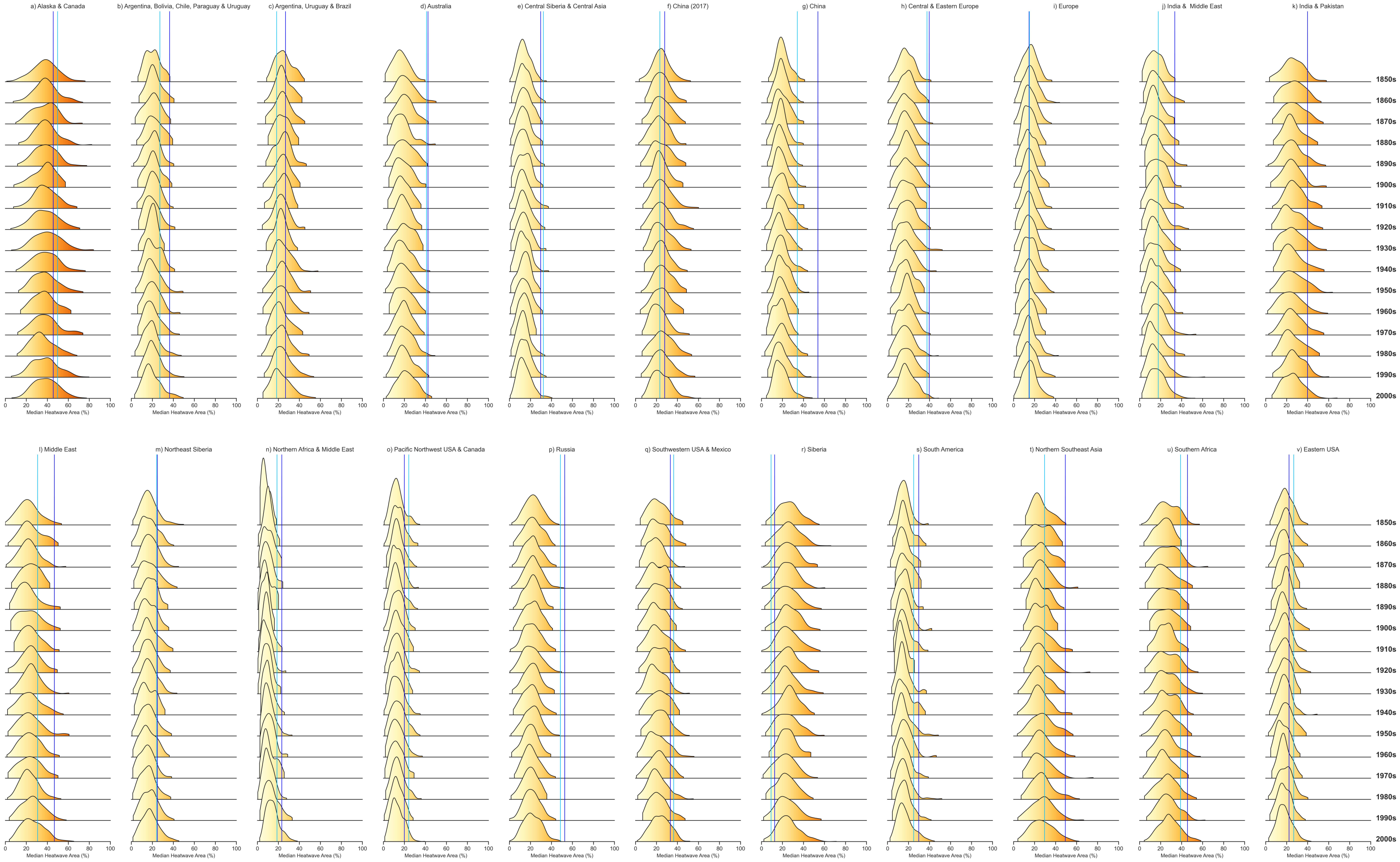

a) Alaska & Canada
b) Argentina, Bolivia, Chile, Paraguay & Uruguay
c) Argentina, Uruguay & Brazil
d) Australia
e) Central Siberia & Central Asia
f) China (2017)
g) China
h) Central & Eastern Europe
i) Europe
j) India & Middle East
k) India & Pakistan
l) Middle East
m) Northeast Siberia
n) Northern Africa & Middle East
o) Pacific Northwest USA & Canada
p) Russia
q) Southwestern USA & Mexico
r) Siberia
s) South America
t) Northern Southeast Asia
u) Southern Africa
v) Eastern USA
1850s
1860s
1870s
1880s
1890s
1900s
1910s
1920s
1930s
1940s
1950s
1960s
1970s
1980s
1990s
2000s
0 20 40 60 80 100
Median Heatwave Area (%)

Supplementary Figure 175: Kernel Density Estimates (KDE) across Decades for Median Heatwave Area (%) in Historical CMIP6 multi-model ensemble (MME) (Tx99.0p) - Kernel Density Estimates (KDE) of Median Heatwave Area (%) for different Decades in the CMIP6 multi-model ensemble (MME). Area under each curve is normalized to 1. Median Heatwave Area (%) of the observed event is indicated by vertical lines in dark blue (Berkeley Earth) and light blue (ERA5).

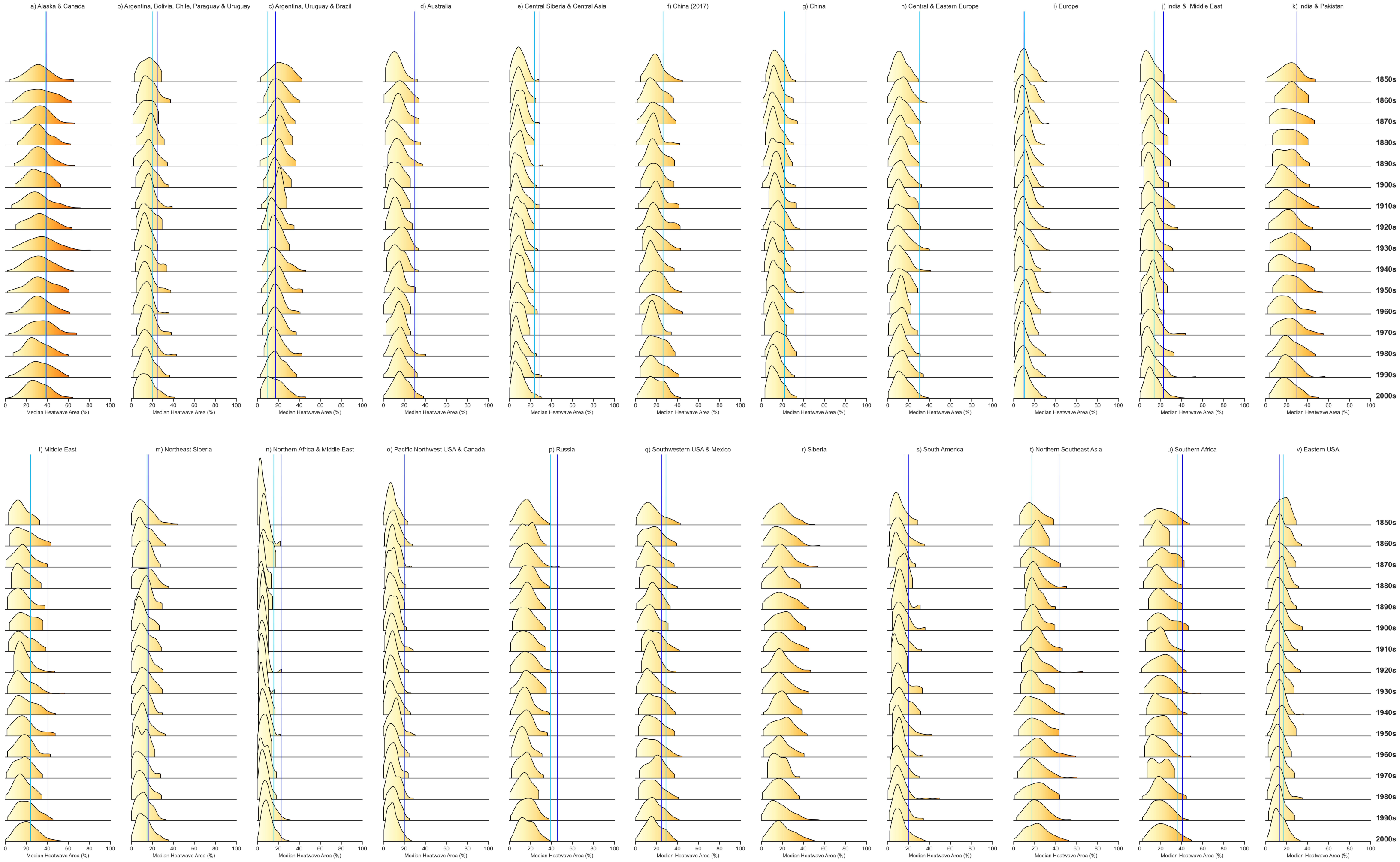
a) Alaska & Canada
b) Argentina, Bolivia, Chile, Paraguay & Uruguay
c) Argentina, Uruguay & Brazil
d) Australia
e) Central Siberia & Central Asia
f) China (2017)
g) China
h) Central & Eastern Europe
i) Europe
j) India & Middle East
k) India & Pakistan
l) Middle East
m) Northeast Siberia
n) Northern Africa & Middle East
o) Pacific Northwest USA & Canada
p) Russia
q) Southwestern USA & Mexico
r) Siberia
s) South America
t) Northern Southeast Asia
u) Southern Africa
v) Eastern USA
1850s
1860s
1870s
1880s
1890s
1900s
1910s
1920s
1930s
1940s
1950s
1960s
1970s
1980s
1990s
2000s
0
20
40
60
80
100
Median Heatwave Area (%)

Supplementary Figure 176: Kernel Density Estimates (KDE) across Decades for Median Heatwave Area (%) in SSP1-2.6 CMIP6 multi-model ensemble (MME) (Tx95.0p) - Kernel Density Estimates (KDE) of Median Heatwave Area (%) for different Decades in the CMIP6 multi-model ensemble (MME). Area under each curve is normalized to 1. Median Heatwave Area (%) of the observed event is indicated by vertical lines in dark blue (Berkeley Earth) and light blue (ERA5).

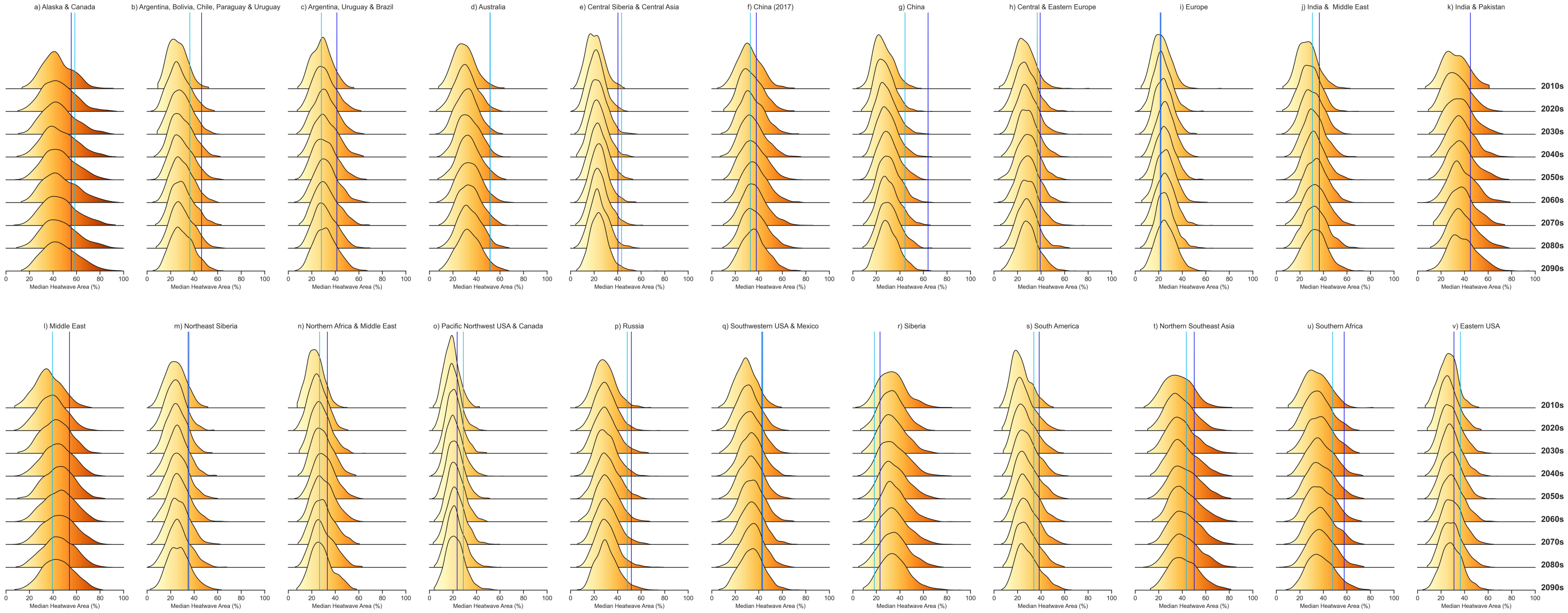

a) Alaska & Canada
b) Argentina, Bolivia, Chile, Paraguay & Uruguay
c) Argentina, Uruguay & Brazil
d) Australia
e) Central Siberia & Central Asia
f) China (2017)
g) China
h) Central & Eastern Europe
i) Europe
j) India & Middle East
k) India & Pakistan
l) Middle East
m) Northeast Siberia
n) Northern Africa & Middle East
o) Pacific Northwest USA & Canada
p) Russia
q) Southwestern USA & Mexico
r) Siberia
s) South America
t) Northern Southeast Asia
u) Southern Africa
v) Eastern USA
2010s
2020s
2030s
2040s
2050s
2060s
2070s
2080s
2090s
Median Heatwave Area (%)
0
20
40
60
80
100

Supplementary Figure 177: Kernel Density Estimates (KDE) across Decades for Median Heatwave Area (%) in SSP1-2.6 CMIP6 multi-model ensemble (MME) (Tx97.5p) - Kernel Density Estimates (KDE) of Median Heatwave Area (%) for different Decades in the CMIP6 multi-model ensemble (MME). Area under each curve is normalized to 1. Median Heatwave Area (%) of the observed event is indicated by vertical lines in dark blue (Berkeley Earth) and light blue (ERA5).

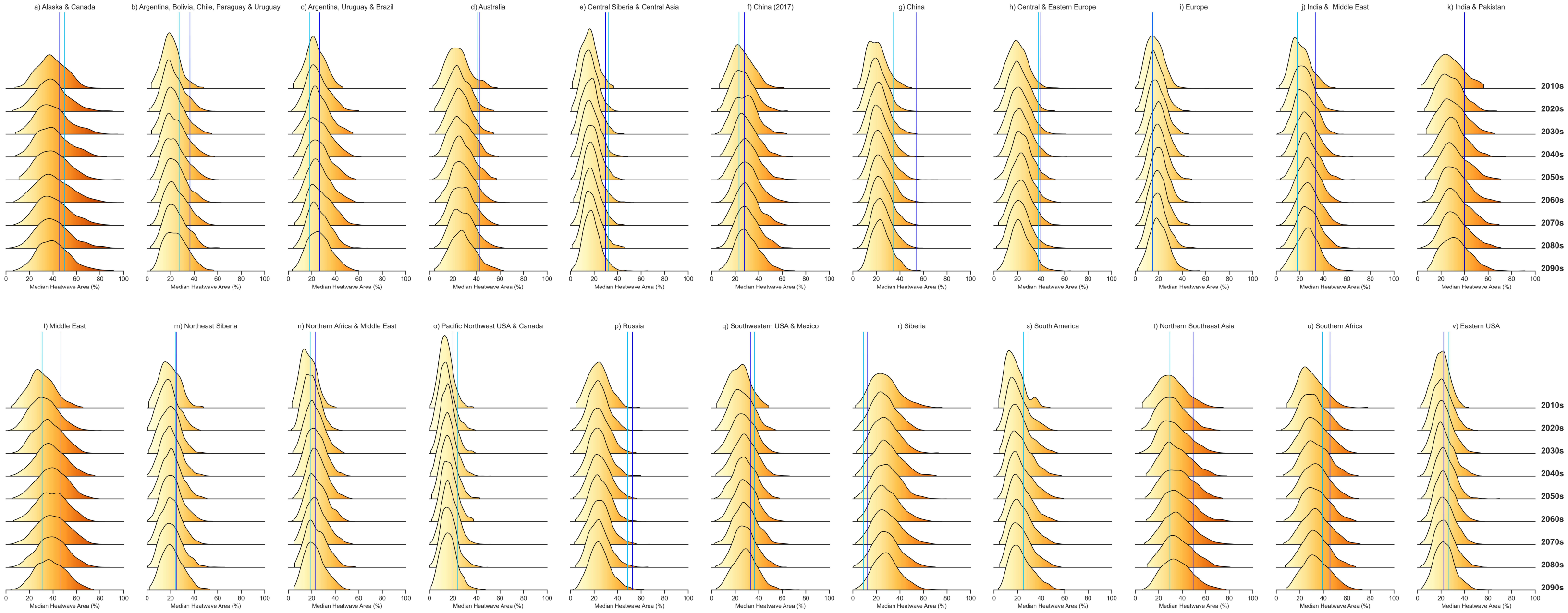
a) Alaska & Canada
b) Argentina, Bolivia, Chile, Paraguay & Uruguay
c) Argentina, Uruguay & Brazil
d) Australia
e) Central Siberia & Central Asia
f) China (2017)
g) China
h) Central & Eastern Europe
i) Europe
j) India & Middle East
k) India & Pakistan
l) Middle East
m) Northeast Siberia
n) Northern Africa & Middle East
o) Pacific Northwest USA & Canada
p) Russia
q) Southwestern USA & Mexico
r) Siberia
s) South America
t) Northern Southeast Asia
u) Southern Africa
v) Eastern USA
2010s
2020s
2030s
2040s
2050s
2060s
2070s
2080s
2090s
Median Heatwave Area (%)
0 20 40 60 80 100

Supplementary Figure 178: Kernel Density Estimates (KDE) across Decades for Median Heatwave Area (%) in SSP1-2.6 CMIP6 multi-model ensemble (MME) (Tx99.0p) - Kernel Density Estimates (KDE) of Median Heatwave Area (%) for different Decades in the CMIP6 multi-model ensemble (MME). Area under each curve is normalized to 1. Median Heatwave Area (%) of the observed event is indicated by vertical lines in dark blue (Berkeley Earth) and light blue (ERA5).

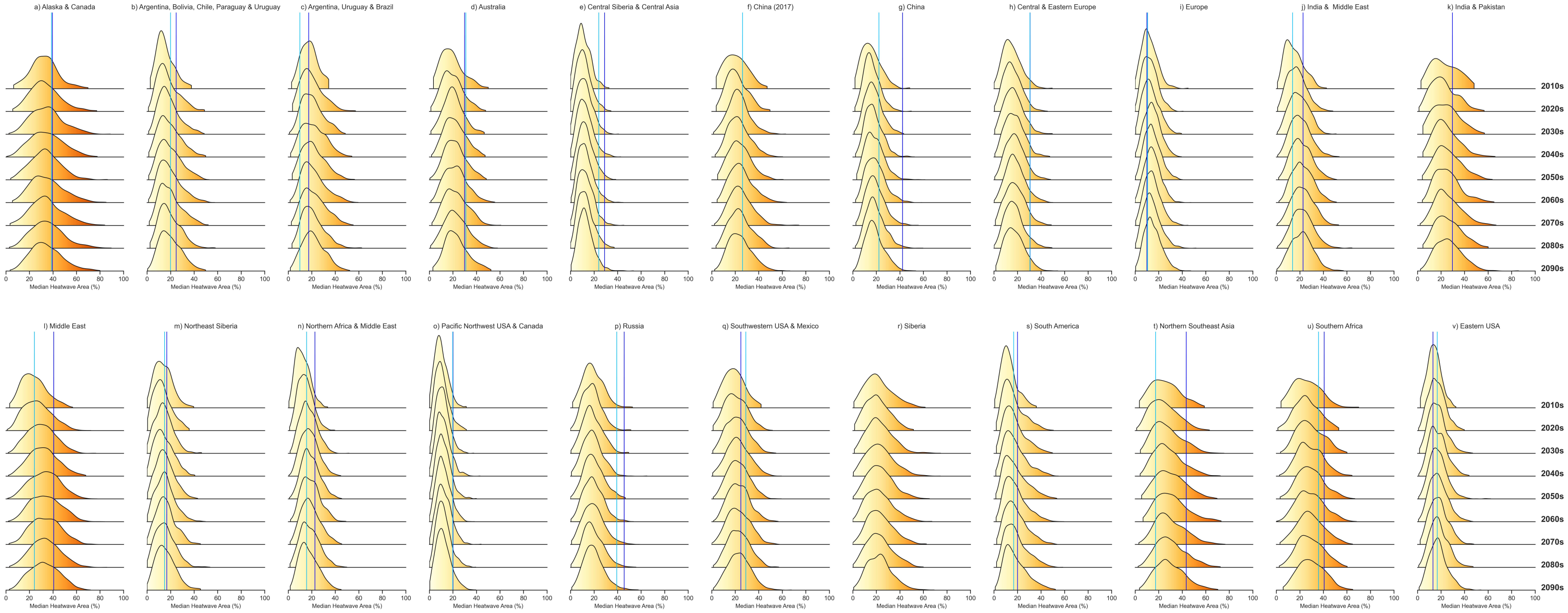

a) Alaska & Canada
b) Argentina, Bolivia, Chile, Paraguay & Uruguay
c) Argentina, Uruguay & Brazil
d) Australia
e) Central Siberia & Central Asia
f) China (2017)
g) China
h) Central & Eastern Europe
i) Europe
j) India & Middle East
k) India & Pakistan
l) Middle East
m) Northeast Siberia
n) Northern Africa & Middle East
o) Pacific Northwest USA & Canada
p) Russia
q) Southwestern USA & Mexico
r) Siberia
s) South America
t) Northern Southeast Asia
u) Southern Africa
v) Eastern USA
2010s
2020s
2030s
2040s
2050s
2060s
2070s
2080s
2090s
0 20 40 60 80 100
Median Heatwave Area (%)

Supplementary Figure 179: Kernel Density Estimates (KDE) across Decades for Median Heatwave Area (%) in SSP2-4.5 CMIP6 multi-model ensemble (MME) (Tx95.0p) - Kernel Density Estimates (KDE) of Median Heatwave Area (%) for different Decades in the CMIP6 multi-model ensemble (MME). Area under each curve is normalized to 1. Median Heatwave Area (%) of the observed event is indicated by vertical lines in dark blue (Berkeley Earth) and light blue (ERA5).

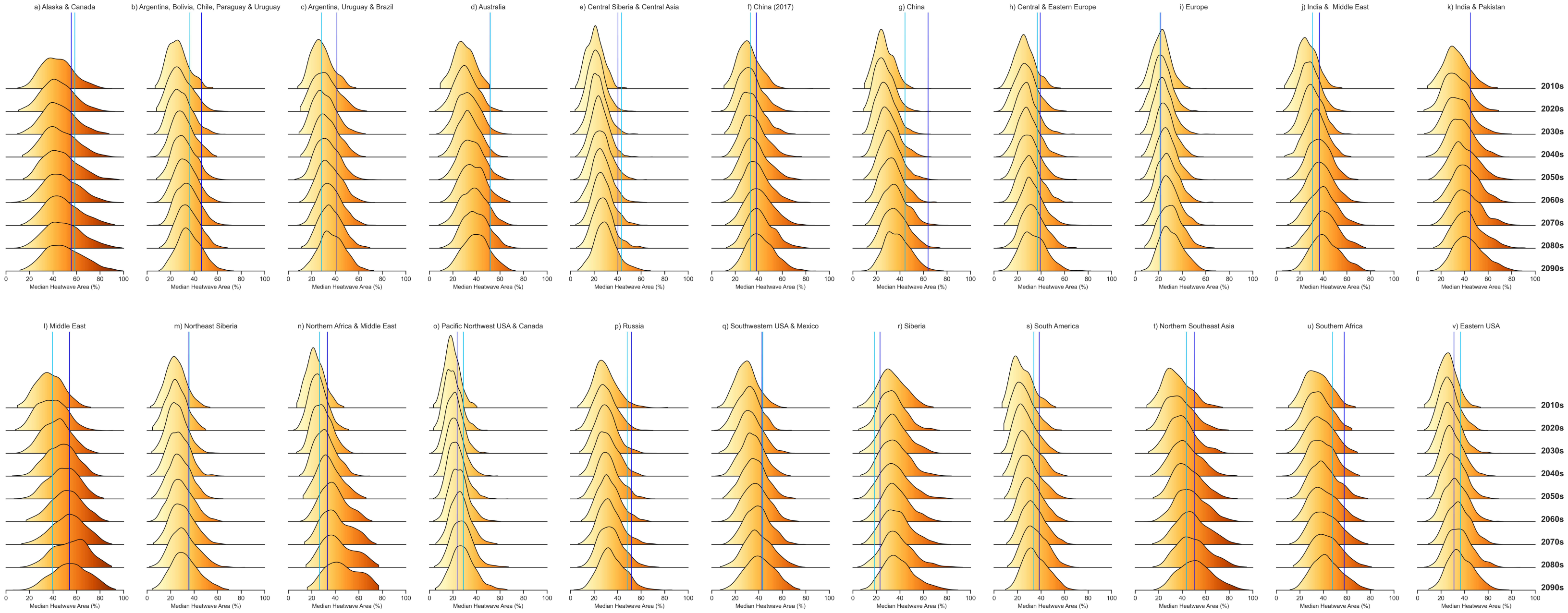

a) Alaska & Canada
b) Argentina, Bolivia, Chile, Paraguay & Uruguay
c) Argentina, Uruguay & Brazil
d) Australia
e) Central Siberia & Central Asia
f) China (2017)
g) China
h) Central & Eastern Europe
i) Europe
j) India & Middle East
k) India & Pakistan
l) Middle East
m) Northeast Siberia
n) Northern Africa & Middle East
o) Pacific Northwest USA & Canada
p) Russia
q) Southwestern USA & Mexico
r) Siberia
s) South America
t) Northern Southeast Asia
u) Southern Africa
v) Eastern USA
2010s
2020s
2030s
2040s
2050s
2060s
2070s
2080s
2090s
Median Heatwave Area (%)
0
20
40
60
80
100

Supplementary Figure 180: Kernel Density Estimates (KDE) across Decades for Median Heatwave Area (%) in SSP2-4.5 CMIP6 multi-model ensemble (MME) (Tx97.5p) - Kernel Density Estimates (KDE) of Median Heatwave Area (%) for different Decades in the CMIP6 multi-model ensemble (MME). Area under each curve is normalized to 1. Median Heatwave Area (%) of the observed event is indicated by vertical lines in dark blue (Berkeley Earth) and light blue (ERA5).

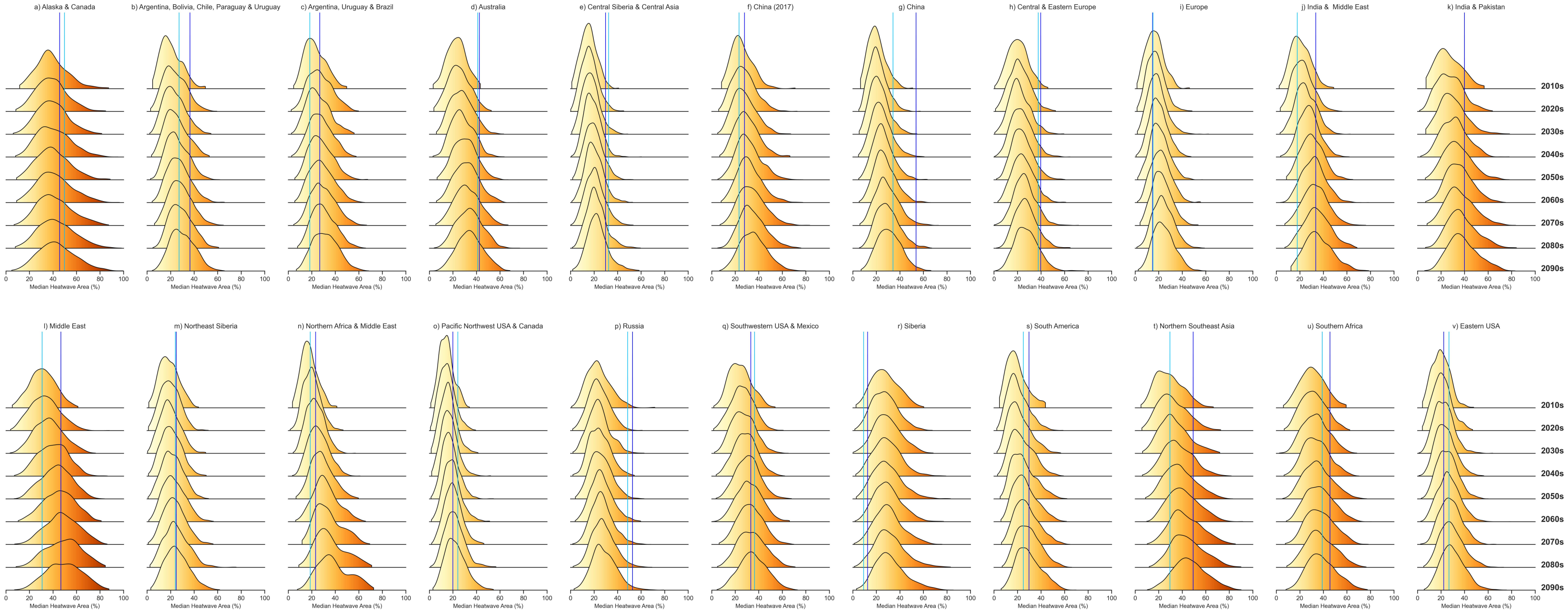

a) Alaska & Canada
b) Argentina, Bolivia, Chile, Paraguay & Uruguay
c) Argentina, Uruguay & Brazil
d) Australia
e) Central Siberia & Central Asia
f) China (2017)
g) China
h) Central & Eastern Europe
i) Europe
j) India & Middle East
k) India & Pakistan
l) Middle East
m) Northeast Siberia
n) Northern Africa & Middle East
o) Pacific Northwest USA & Canada
p) Russia
q) Southwestern USA & Mexico
r) Siberia
s) South America
t) Northern Southeast Asia
u) Southern Africa
v) Eastern USA
2010s
2020s
2030s
2040s
2050s
2060s
2070s
2080s
2090s
0 20 40 60 80 100
Median Heatwave Area (%)

Supplementary Figure 181: Kernel Density Estimates (KDE) across Decades for Median Heatwave Area (%) in SSP2-4.5 CMIP6 multi-model ensemble (MME) (Tx99.0p) - Kernel Density Estimates (KDE) of Median Heatwave Area (%) for different Decades in the CMIP6 multi-model ensemble (MME). Area under each curve is normalized to 1. Median Heatwave Area (%) of the observed event is indicated by vertical lines in dark blue (Berkeley Earth) and light blue (ERA5).

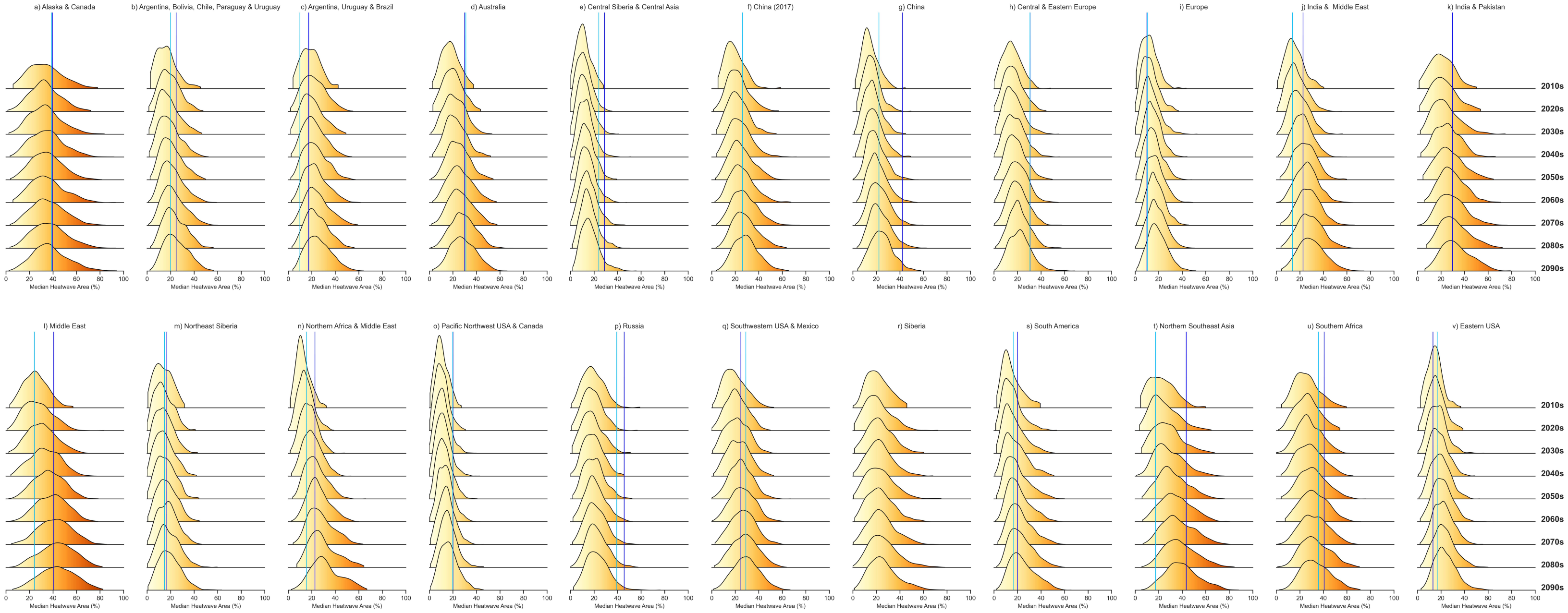

a) Alaska & Canada
b) Argentina, Bolivia, Chile, Paraguay & Uruguay
c) Argentina, Uruguay & Brazil
d) Australia
e) Central Siberia & Central Asia
f) China (2017)
g) China
h) Central & Eastern Europe
i) Europe
j) India & Middle East
k) India & Pakistan
l) Middle East
m) Northeast Siberia
n) Northern Africa & Middle East
o) Pacific Northwest USA & Canada
p) Russia
q) Southwestern USA & Mexico
r) Siberia
s) South America
t) Northern Southeast Asia
u) Southern Africa
v) Eastern USA
2010s
2020s
2030s
2040s
2050s
2060s
2070s
2080s
2090s
0 20 40 60 80 100
Median Heatwave Area (%)

Supplementary Figure 182: Kernel Density Estimates (KDE) across Decades for Median Heatwave Area (%) in SSP3-7.0 CMIP6 multi-model ensemble (MME) (Tx95.0p) - Kernel Density Estimates (KDE) of Median Heatwave Area (%) for different Decades in the CMIP6 multi-model ensemble (MME). Area under each curve is normalized to 1. Median Heatwave Area (%) of the observed event is indicated by vertical lines in dark blue (Berkeley Earth) and light blue (ERA5).

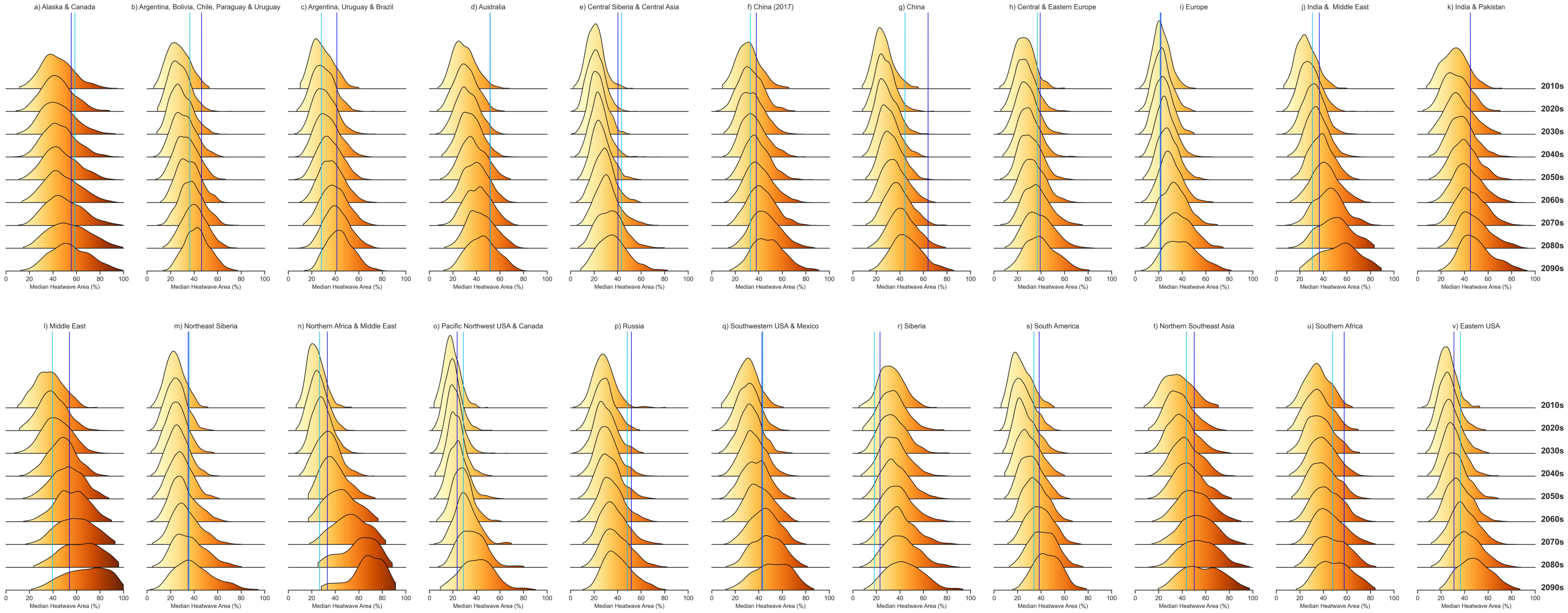
a) Alaska & Canada
b) Argentina, Bolivia, Chile, Paraguay & Uruguay
c) Argentina, Uruguay & Brazil
d) Australia
e) Central Siberia & Central Asia
f) China (2017)
g) China
h) Central & Eastern Europe
i) Europe
j) India & Middle East
k) India & Pakistan
l) Middle East
m) Northeast Siberia
n) Northern Africa & Middle East
o) Pacific Northwest USA & Canada
p) Russia
q) Southwestern USA & Mexico
r) Siberia
s) South America
t) Northern Southeast Asia
u) Southern Africa
v) Eastern USA
2010s
2020s
2030s
2040s
2050s
2060s
2070s
2080s
2090s
0
20
40
60
80
100
Median Heatwave Area (%)

Supplementary Figure 183: Kernel Density Estimates (KDE) across Decades for Median Heatwave Area (%) in SSP3-7.0 CMIP6 multi-model ensemble (MME) (Tx97.5p) - Kernel Density Estimates (KDE) of Median Heatwave Area (%) for different Decades in the CMIP6 multi-model ensemble (MME). Area under each curve is normalized to 1. Median Heatwave Area (%) of the observed event is indicated by vertical lines in dark blue (Berkeley Earth) and light blue (ERA5).

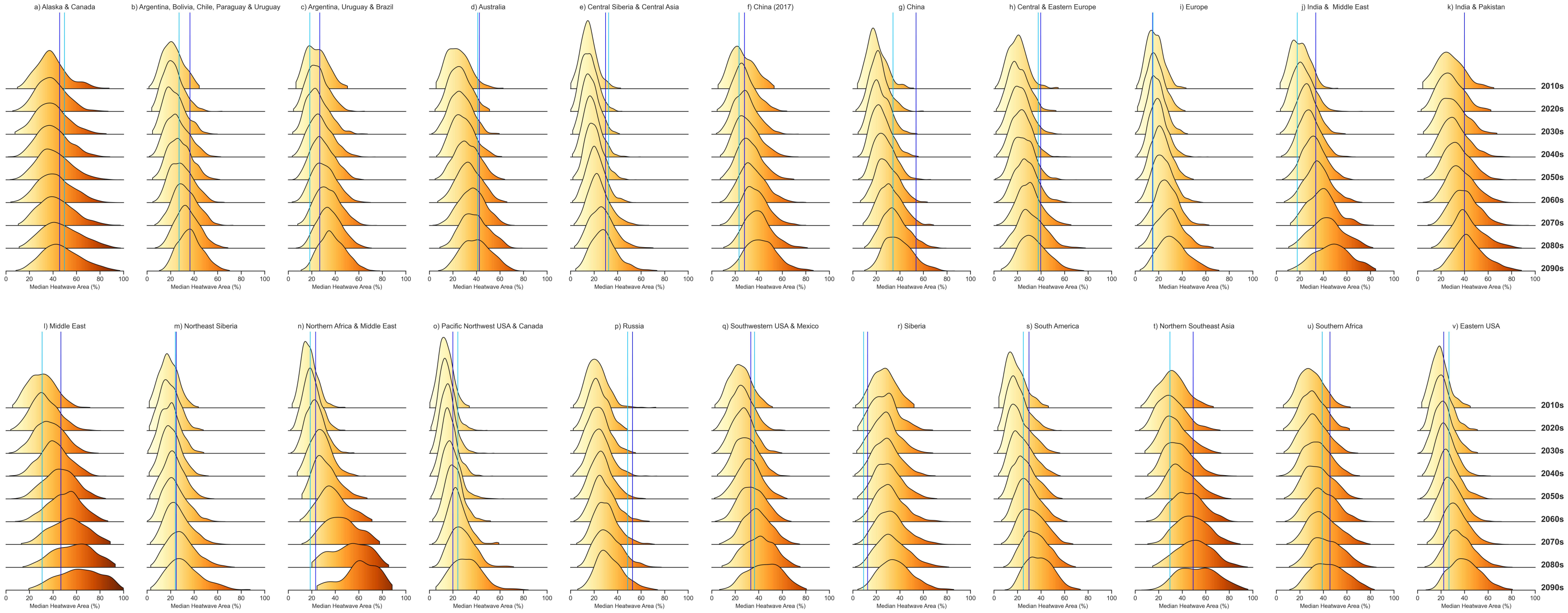
a) Alaska & Canada
b) Argentina, Bolivia, Chile, Paraguay & Uruguay
c) Argentina, Uruguay & Brazil
d) Australia
e) Central Siberia & Central Asia
f) China (2017)
g) China
h) Central & Eastern Europe
i) Europe
j) India & Middle East
k) India & Pakistan
l) Middle East
m) Northeast Siberia
n) Northern Africa & Middle East
o) Pacific Northwest USA & Canada
p) Russia
q) Southwestern USA & Mexico
r) Siberia
s) South America
t) Northern Southeast Asia
u) Southern Africa
v) Eastern USA
2010s
2020s
2030s
2040s
2050s
2060s
2070s
2080s
2090s
0 20 40 60 80 100
Median Heatwave Area (%)

Supplementary Figure 184: Kernel Density Estimates (KDE) across Decades for Median Heatwave Area (%) in SSP3-7.0 CMIP6 multi-model ensemble (MME) (Tx99.0p) - Kernel Density Estimates (KDE) of Median Heatwave Area (%) for different Decades in the CMIP6 multi-model ensemble (MME). Area under each curve is normalized to 1. Median Heatwave Area (%) of the observed event is indicated by vertical lines in dark blue (Berkeley Earth) and light blue (ERA5).

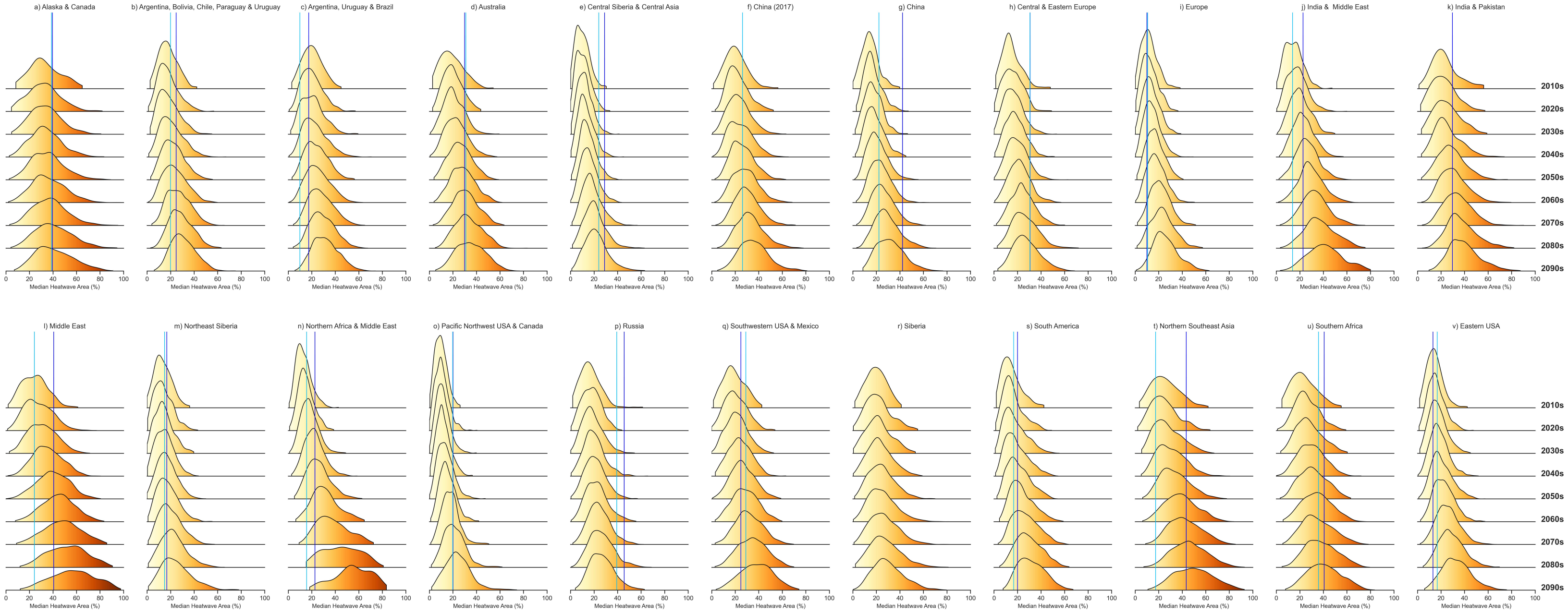

a) Alaska & Canada
b) Argentina, Bolivia, Chile, Paraguay & Uruguay
c) Argentina, Uruguay & Brazil
d) Australia
e) Central Siberia & Central Asia
f) China (2017)
g) China
h) Central & Eastern Europe
i) Europe
j) India & Middle East
k) India & Pakistan
l) Middle East
m) Northeast Siberia
n) Northern Africa & Middle East
o) Pacific Northwest USA & Canada
p) Russia
q) Southwestern USA & Mexico
r) Siberia
s) South America
t) Northern Southeast Asia
u) Southern Africa
v) Eastern USA
2010s
2020s
2030s
2040s
2050s
2060s
2070s
2080s
2090s
Median Heatwave Area (%)
0 20 40 60 80 100

Supplementary Figure 185: Kernel Density Estimates (KDE) across Decades for Median Heatwave Area (%) in SSP5-8.5 CMIP6 multi-model ensemble (MME) (Tx95.0p) - Kernel Density Estimates (KDE) of Median Heatwave Area (%) for different Decades in the CMIP6 multi-model ensemble (MME). Area under each curve is normalized to 1. Median Heatwave Area (%) of the observed event is indicated by vertical lines in dark blue (Berkeley Earth) and light blue (ERA5).

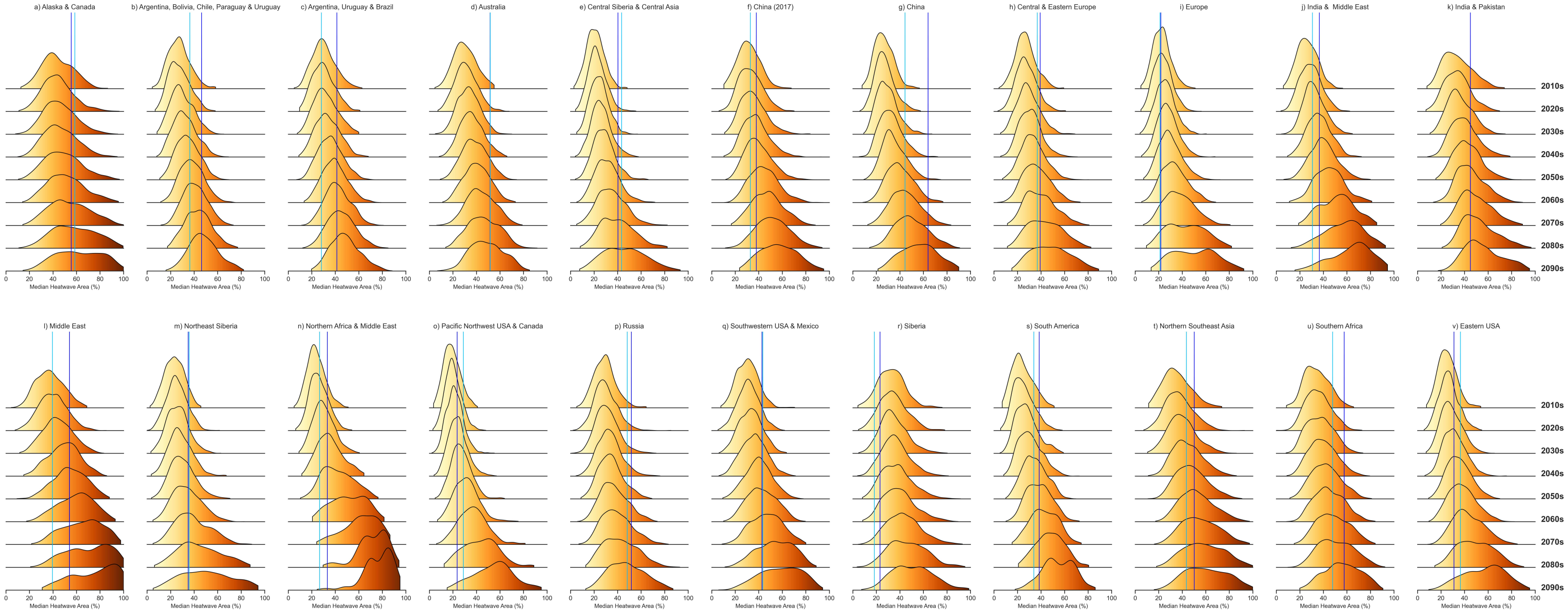

a) Alaska & Canada
b) Argentina, Bolivia, Chile, Paraguay & Uruguay
c) Argentina, Uruguay & Brazil
d) Australia
e) Central Siberia & Central Asia
f) China (2017)
g) China
h) Central & Eastern Europe
i) Europe
j) India & Middle East
k) India & Pakistan
l) Middle East
m) Northeast Siberia
n) Northern Africa & Middle East
o) Pacific Northwest USA & Canada
p) Russia
q) Southwestern USA & Mexico
r) Siberia
s) South America
t) Northern Southeast Asia
u) Southern Africa
v) Eastern USA
2010s
2020s
2030s
2040s
2050s
2060s
2070s
2080s
2090s
0 20 40 60 80 100
Median Heatwave Area (%)

Supplementary Figure 186: Kernel Density Estimates (KDE) across Decades for Median Heatwave Area (%) in SSP5-8.5 CMIP6 multi-model ensemble (MME) (Tx97.5p) - Kernel Density Estimates (KDE) of Median Heatwave Area (%) for different Decades in the CMIP6 multi-model ensemble (MME). Area under each curve is normalized to 1. Median Heatwave Area (%) of the observed event is indicated by vertical lines in dark blue (Berkeley Earth) and light blue (ERA5).

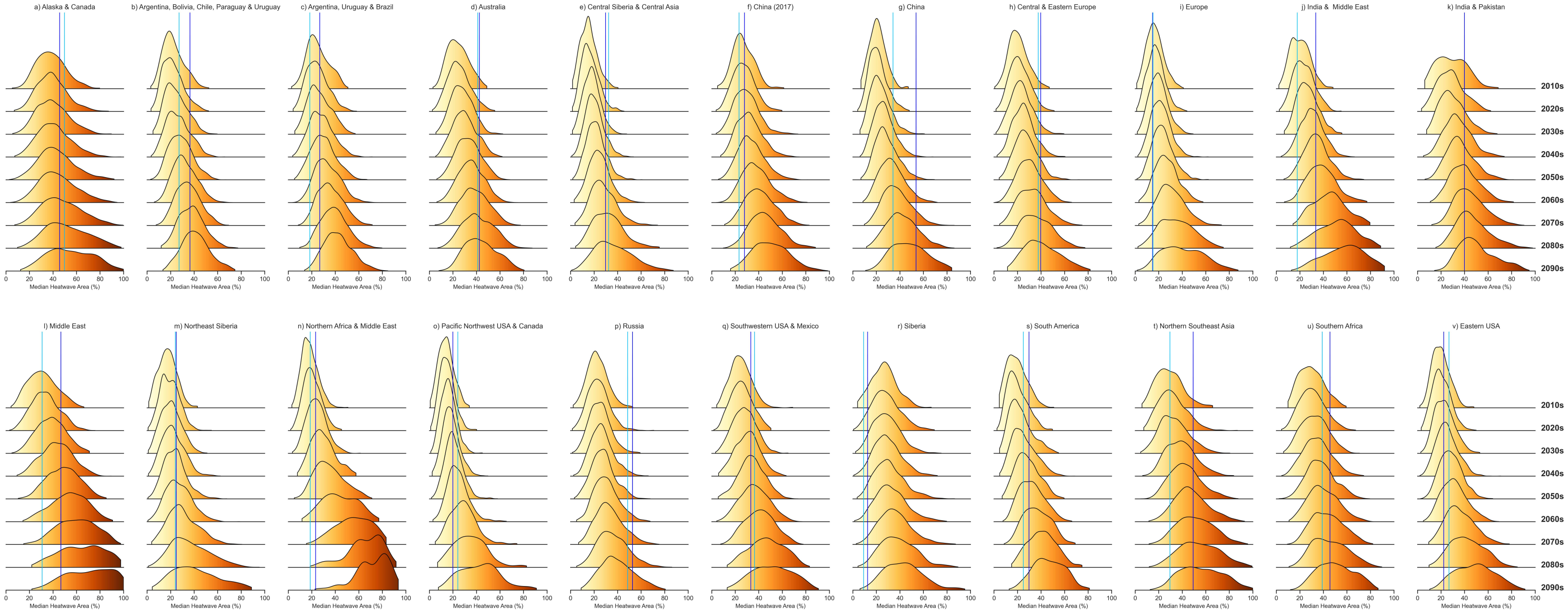
a) Alaska & Canada
b) Argentina, Bolivia, Chile, Paraguay & Uruguay
c) Argentina, Uruguay & Brazil
d) Australia
e) Central Siberia & Central Asia
f) China (2017)
g) China
h) Central & Eastern Europe
i) Europe
j) India & Middle East
k) India & Pakistan
l) Middle East
m) Northeast Siberia
n) Northern Africa & Middle East
o) Pacific Northwest USA & Canada
p) Russia
q) Southwestern USA & Mexico
r) Siberia
s) South America
t) Northern Southeast Asia
u) Southern Africa
v) Eastern USA
2010s
2020s
2030s
2040s
2050s
2060s
2070s
2080s
2090s
Median Heatwave Area (%)
0
20
40
60
80
100

Supplementary Figure 187: Kernel Density Estimates (KDE) across Decades for Median Heatwave Area (%) in SSP5-8.5 CMIP6 multi-model ensemble (MME) (Tx99.0p) - Kernel Density Estimates (KDE) of Median Heatwave Area (%) for different Decades in the CMIP6 multi-model ensemble (MME). Area under each curve is normalized to 1. Median Heatwave Area (%) of the observed event is indicated by vertical lines in dark blue (Berkeley Earth) and light blue (ERA5).

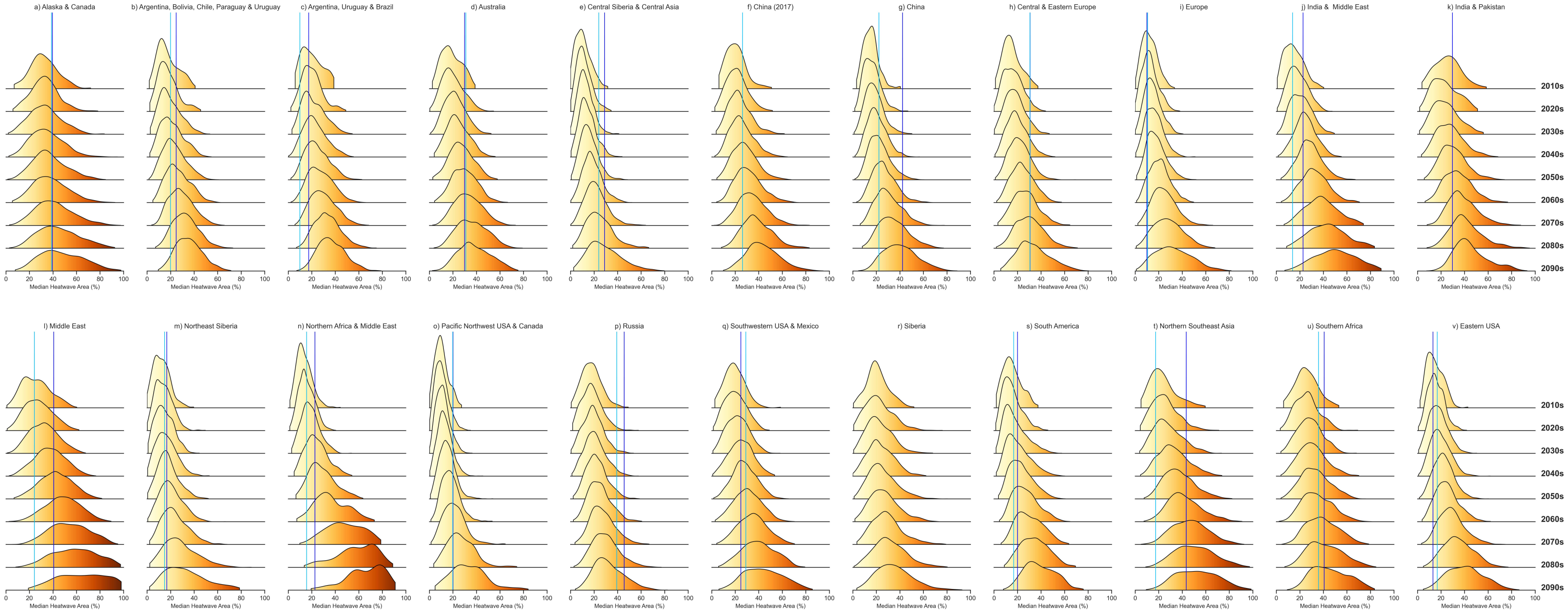

a) Alaska & Canada
b) Argentina, Bolivia, Chile, Paraguay & Uruguay
c) Argentina, Uruguay & Brazil
d) Australia
e) Central Siberia & Central Asia
f) China (2017)
g) China
h) Central & Eastern Europe
i) Europe
j) India & Middle East
k) India & Pakistan
l) Middle East
m) Northeast Siberia
n) Northern Africa & Middle East
o) Pacific Northwest USA & Canada
p) Russia
q) Southwestern USA & Mexico
r) Siberia
s) South America
t) Northern Southeast Asia
u) Southern Africa
v) Eastern USA
2010s
2020s
2030s
2040s
2050s
2060s
2070s
2080s
2090s
0
20
40
60
80
100
Median Heatwave Area (%)

Supplementary Figure 188: Kernel Density Estimates (KDE) across Decades for Maximum Heatwave Area (%) in Historical CMIP6 multi-model ensemble (MME) (Tx95.0p) - Kernel Density Estimates (KDE) of Maximum Heatwave Area (%) for different Decades in the CMIP6 multi-model ensemble (MME). Area under each curve is normalized to 1. Maximum Heatwave Area (%) of the observed event is indicated by vertical lines in dark blue (Berkeley Earth) and light blue (ERA5).

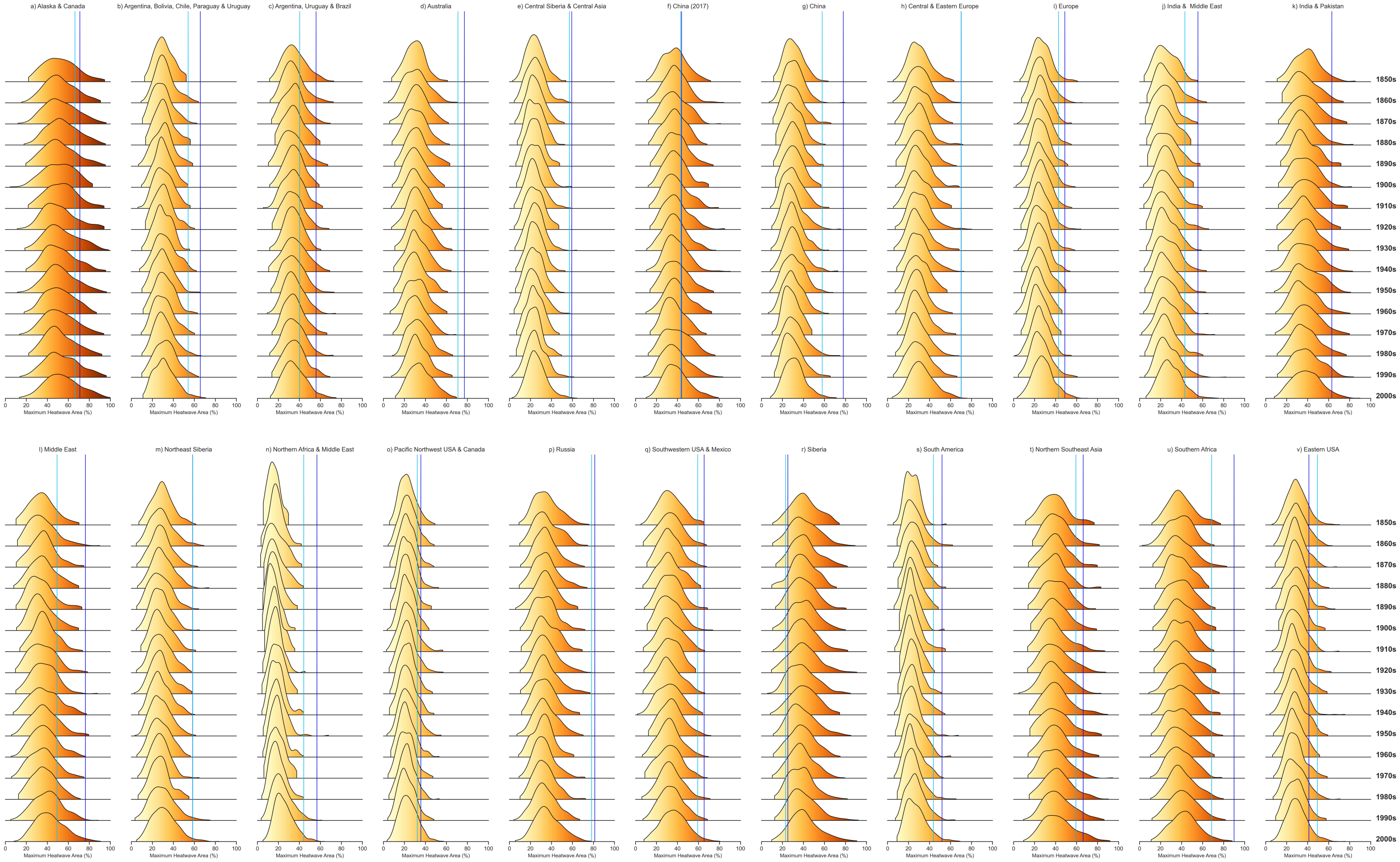

a) Alaska & Canada
b) Argentina, Bolivia, Chile, Paraguay & Uruguay
c) Argentina, Uruguay & Brazil
d) Australia
e) Central Siberia & Central Asia
f) China (2017)
g) China
h) Central & Eastern Europe
i) Europe
j) India & Middle East
k) India & Pakistan
l) Middle East
m) Northeast Siberia
n) Northern Africa & Middle East
o) Pacific Northwest USA & Canada
p) Russia
q) Southwestern USA & Mexico
r) Siberia
s) South America
t) Northern Southeast Asia
u) Southern Africa
v) Eastern USA
1850s
1860s
1870s
1880s
1890s
1900s
1910s
1920s
1930s
1940s
1950s
1960s
1970s
1980s
1990s
2000s
0 20 40 60 80 100
Maximum Heatwave Area (%)

Supplementary Figure 189: Kernel Density Estimates (KDE) across Decades for Maximum Heatwave Area (%) in Historical CMIP6 multi-model ensemble (MME) (Tx97.5p) - Kernel Density Estimates (KDE) of Maximum Heatwave Area (%) for different Decades in the CMIP6 multi-model ensemble (MME). Area under each curve is normalized to 1. Maximum Heatwave Area (%) of the observed event is indicated by vertical lines in dark blue (Berkeley Earth) and light blue (ERA5).

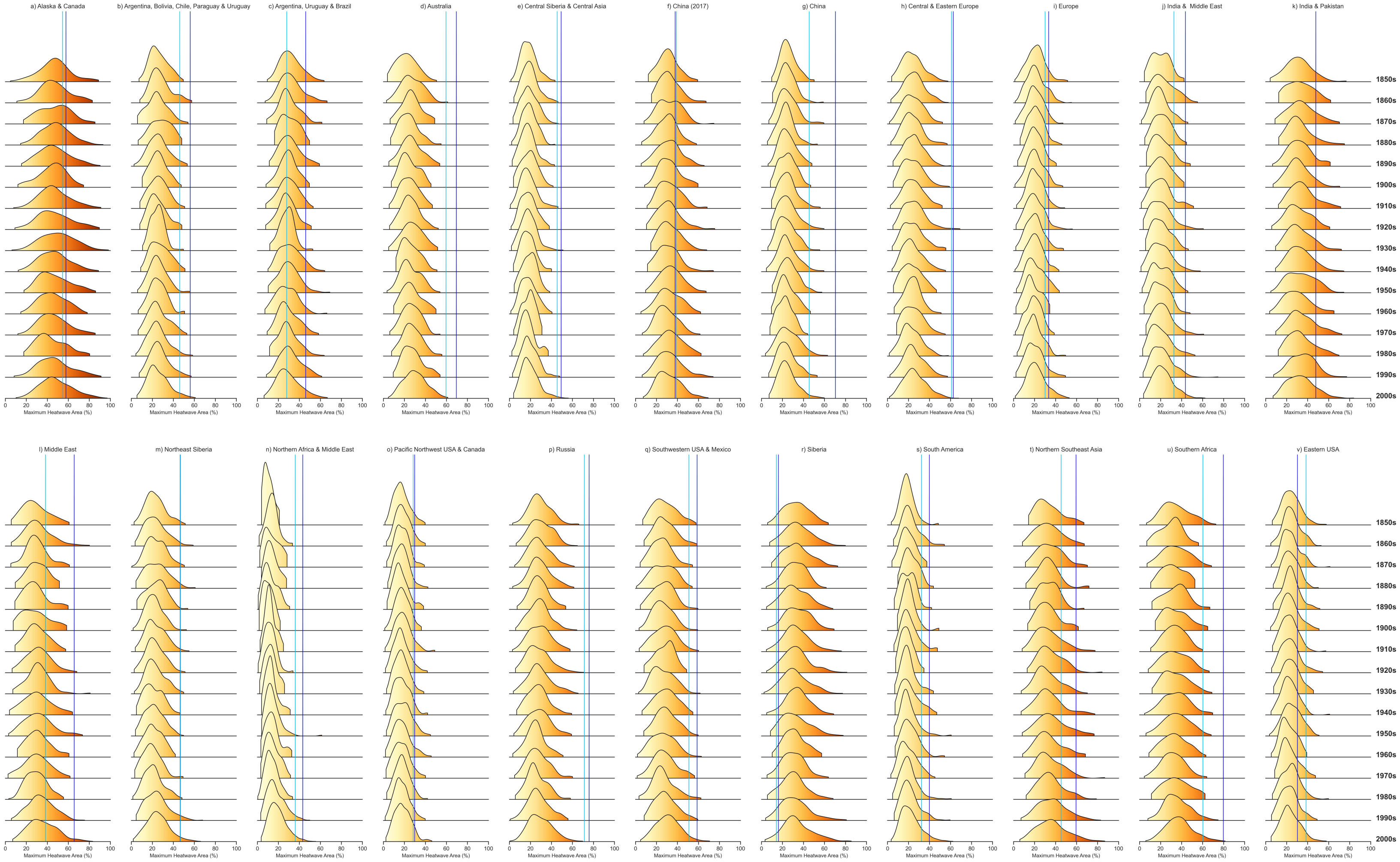
a) Alaska & Canada
b) Argentina, Bolivia, Chile, Paraguay & Uruguay
c) Argentina, Uruguay & Brazil
d) Australia
e) Central Siberia & Central Asia
f) China (2017)
g) China
h) Central & Eastern Europe
i) Europe
j) India & Middle East
k) India & Pakistan
l) Middle East
m) Northeast Siberia
n) Northern Africa & Middle East
o) Pacific Northwest USA & Canada
p) Russia
q) Southwestern USA & Mexico
r) Siberia
s) South America
t) Northern Southeast Asia
u) Southern Africa
v) Eastern USA
1850s
1860s
1870s
1880s
1890s
1900s
1910s
1920s
1930s
1940s
1950s
1960s
1970s
1980s
1990s
2000s
0 20 40 60 80 100
Maximum Heatwave Area (%)

Supplementary Figure 190: Kernel Density Estimates (KDE) across Decades for Maximum Heatwave Area (%) in Historical CMIP6 multi-model ensemble (MME) (Tx99.0p) - Kernel Density Estimates (KDE) of Maximum Heatwave Area (%) for different Decades in the CMIP6 multi-model ensemble (MME). Area under each curve is normalized to 1. Maximum Heatwave Area (%) of the observed event is indicated by vertical lines in dark blue (Berkeley Earth) and light blue (ERA5).

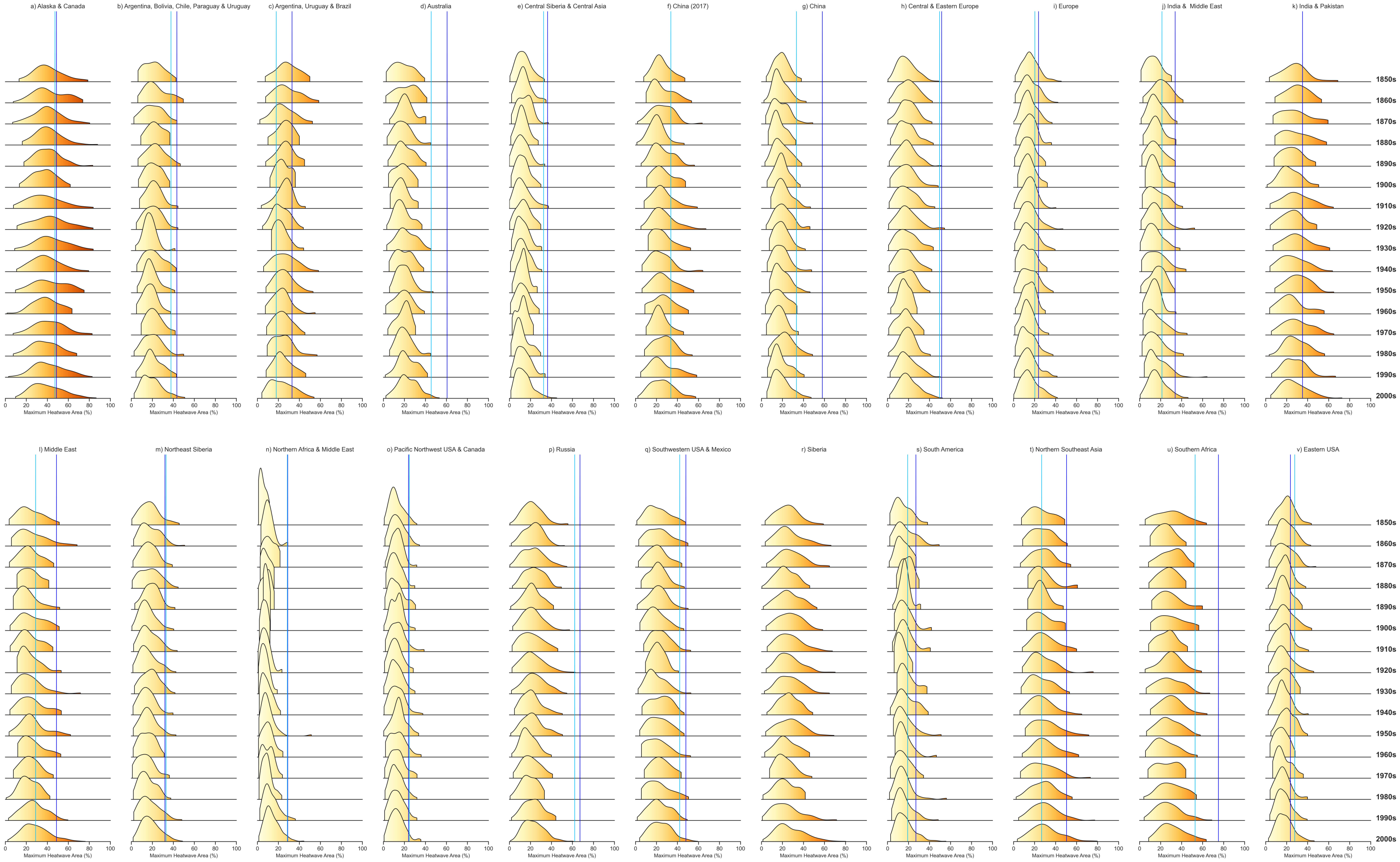

a) Alaska & Canada
b) Argentina, Bolivia, Chile, Paraguay & Uruguay
c) Argentina, Uruguay & Brazil
d) Australia
e) Central Siberia & Central Asia
f) China (2017)
g) China
h) Central & Eastern Europe
i) Europe
j) India & Middle East
k) India & Pakistan
l) Middle East
m) Northeast Siberia
n) Northern Africa & Middle East
o) Pacific Northwest USA & Canada
p) Russia
q) Southwestern USA & Mexico
r) Siberia
s) South America
t) Northern Southeast Asia
u) Southern Africa
v) Eastern USA
1850s
1860s
1870s
1880s
1890s
1900s
1910s
1920s
1930s
1940s
1950s
1960s
1970s
1980s
1990s
2000s
0 20 40 60 80 100
Maximum Heatwave Area (%)

Supplementary Figure 191: Kernel Density Estimates (KDE) across Decades for Maximum Heatwave Area (%) in SSP1-2.6 CMIP6 multi-model ensemble (MME) (Tx95.0p) - Kernel Density Estimates (KDE) of Maximum Heatwave Area (%) for different Decades in the CMIP6 multi-model ensemble (MME). Area under each curve is normalized to 1. Maximum Heatwave Area (%) of the observed event is indicated by vertical lines in dark blue (Berkeley Earth) and light blue (ERA5).

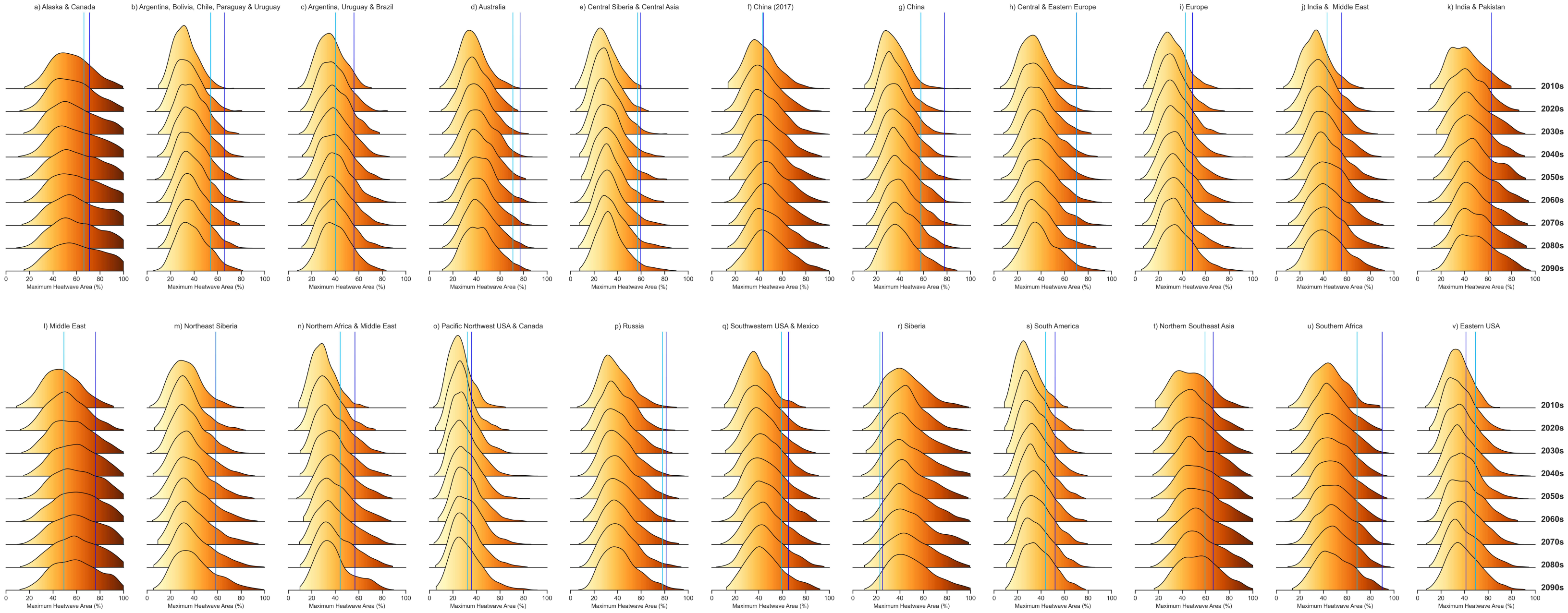

a) Alaska & Canada
b) Argentina, Bolivia, Chile, Paraguay & Uruguay
c) Argentina, Uruguay & Brazil
d) Australia
e) Central Siberia & Central Asia
f) China (2017)
g) China
h) Central & Eastern Europe
i) Europe
j) India & Middle East
k) India & Pakistan
l) Middle East
m) Northeast Siberia
n) Northern Africa & Middle East
o) Pacific Northwest USA & Canada
p) Russia
q) Southwestern USA & Mexico
r) Siberia
s) South America
t) Northern Southeast Asia
u) Southern Africa
v) Eastern USA
2010s
2020s
2030s
2040s
2050s
2060s
2070s
2080s
2090s
0 20 40 60 80 100
Maximum Heatwave Area (%)

Supplementary Figure 192: Kernel Density Estimates (KDE) across Decades for Maximum Heatwave Area (%) in SSP1-2.6 CMIP6 multi-model ensemble (MME) (Tx97.5p) - Kernel Density Estimates (KDE) of Maximum Heatwave Area (%) for different Decades in the CMIP6 multi-model ensemble (MME). Area under each curve is normalized to 1. Maximum Heatwave Area (%) of the observed event is indicated by vertical lines in dark blue (Berkeley Earth) and light blue (ERA5).

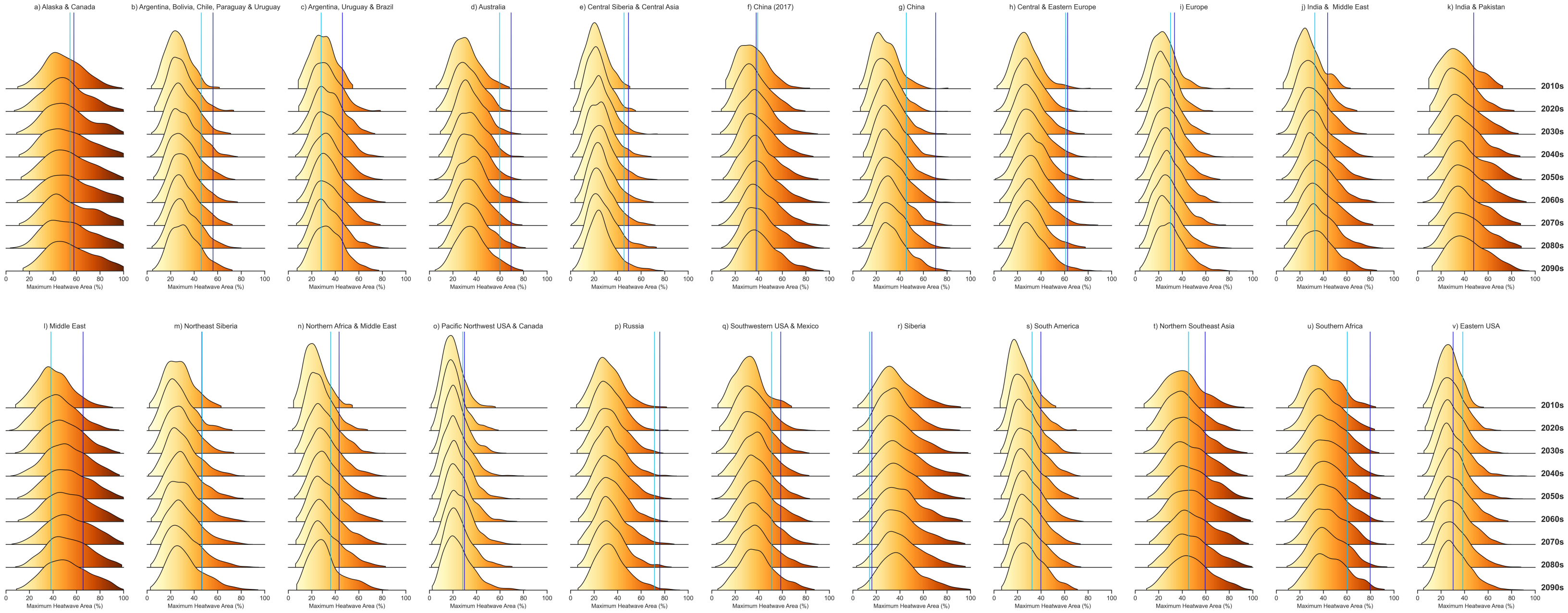
a) Alaska & Canada
b) Argentina, Bolivia, Chile, Paraguay & Uruguay
c) Argentina, Uruguay & Brazil
d) Australia
e) Central Siberia & Central Asia
f) China (2017)
g) China
h) Central & Eastern Europe
i) Europe
j) India & Middle East
k) India & Pakistan
l) Middle East
m) Northeast Siberia
n) Northern Africa & Middle East
o) Pacific Northwest USA & Canada
p) Russia
q) Southwestern USA & Mexico
r) Siberia
s) South America
t) Northern Southeast Asia
u) Southern Africa
v) Eastern USA
2010s
2020s
2030s
2040s
2050s
2060s
2070s
2080s
2090s
0
20
40
60
80
100
Maximum Heatwave Area (%)

Supplementary Figure 193: Kernel Density Estimates (KDE) across Decades for Maximum Heatwave Area (%) in SSP1-2.6 CMIP6 multi-model ensemble (MME) (Tx99.0p) - Kernel Density Estimates (KDE) of Maximum Heatwave Area (%) for different Decades in the CMIP6 multi-model ensemble (MME). Area under each curve is normalized to 1. Maximum Heatwave Area (%) of the observed event is indicated by vertical lines in dark blue (Berkeley Earth) and light blue (ERA5).

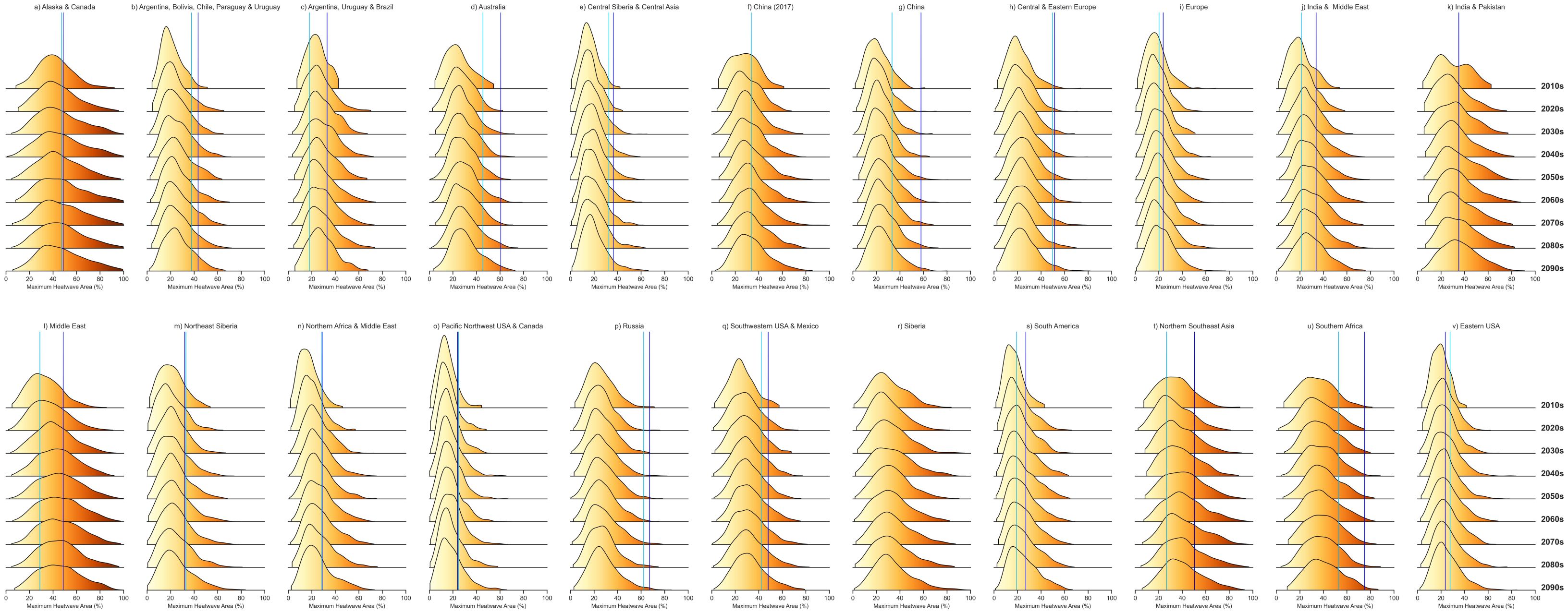
a) Alaska & Canada
b) Argentina, Bolivia, Chile, Paraguay & Uruguay
c) Argentina, Uruguay & Brazil
d) Australia
e) Central Siberia & Central Asia
f) China (2017)
g) China
h) Central & Eastern Europe
i) Europe
j) India & Middle East
k) India & Pakistan
l) Middle East
m) Northeast Siberia
n) Northern Africa & Middle East
o) Pacific Northwest USA & Canada
p) Russia
q) Southwestern USA & Mexico
r) Siberia
s) South America
t) Northern Southeast Asia
u) Southern Africa
v) Eastern USA
2010s
2020s
2030s
2040s
2050s
2060s
2070s
2080s
2090s
0 20 40 60 80 100
Maximum Heatwave Area (%)

Supplementary Figure 194: Kernel Density Estimates (KDE) across Decades for Maximum Heatwave Area (%) in SSP2-4.5 CMIP6 multi-model ensemble (MME) (Tx95.0p) - Kernel Density Estimates (KDE) of Maximum Heatwave Area (%) for different Decades in the CMIP6 multi-model ensemble (MME). Area under each curve is normalized to 1. Maximum Heatwave Area (%) of the observed event is indicated by vertical lines in dark blue (Berkeley Earth) and light blue (ERA5).

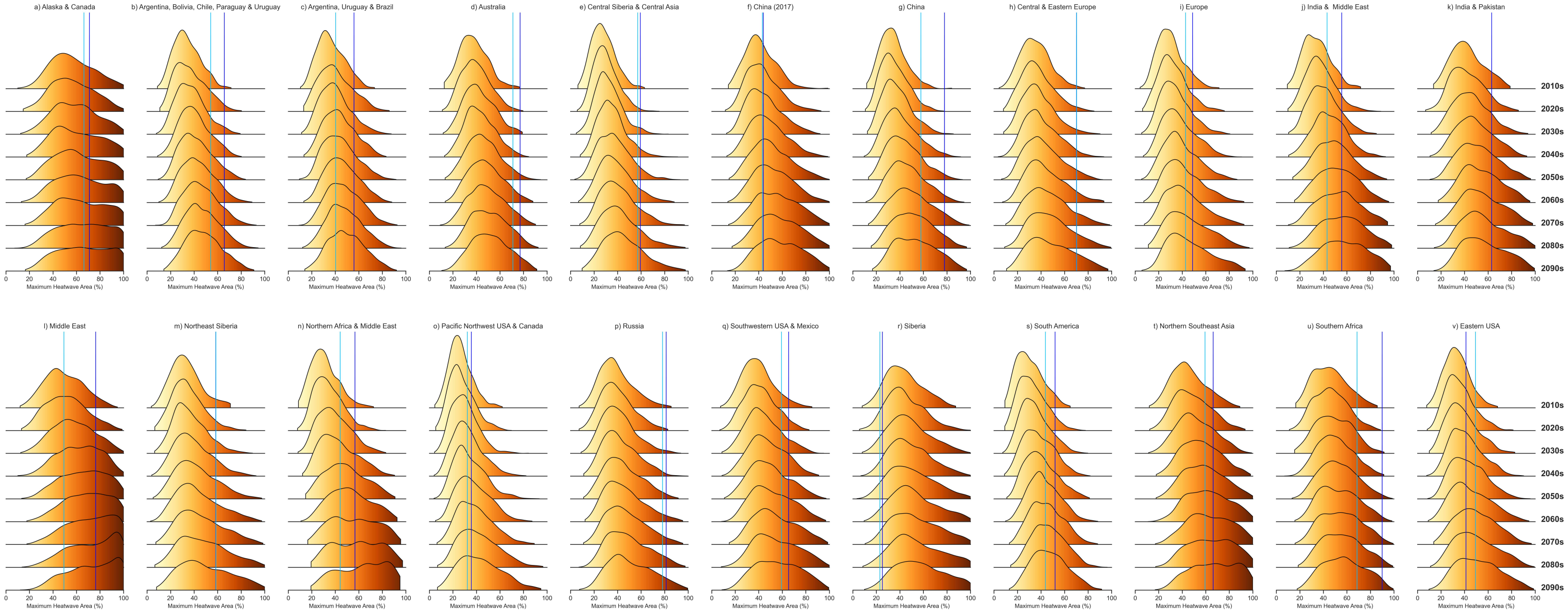
a) Alaska & Canada
b) Argentina, Bolivia, Chile, Paraguay & Uruguay
c) Argentina, Uruguay & Brazil
d) Australia
e) Central Siberia & Central Asia
f) China (2017)
g) China
h) Central & Eastern Europe
i) Europe
j) India & Middle East
k) India & Pakistan
l) Middle East
m) Northeast Siberia
n) Northern Africa & Middle East
o) Pacific Northwest USA & Canada
p) Russia
q) Southwestern USA & Mexico
r) Siberia
s) South America
t) Northern Southeast Asia
u) Southern Africa
v) Eastern USA
2010s
2020s
2030s
2040s
2050s
2060s
2070s
2080s
2090s
Maximum Heatwave Area (%)
0
20
40
60
80
100

Supplementary Figure 195: Kernel Density Estimates (KDE) across Decades for Maximum Heatwave Area (%) in SSP2-4.5 CMIP6 multi-model ensemble (MME) (Tx97.5p) - Kernel Density Estimates (KDE) of Maximum Heatwave Area (%) for different Decades in the CMIP6 multi-model ensemble (MME). Area under each curve is normalized to 1. Maximum Heatwave Area (%) of the observed event is indicated by vertical lines in dark blue (Berkeley Earth) and light blue (ERA5).

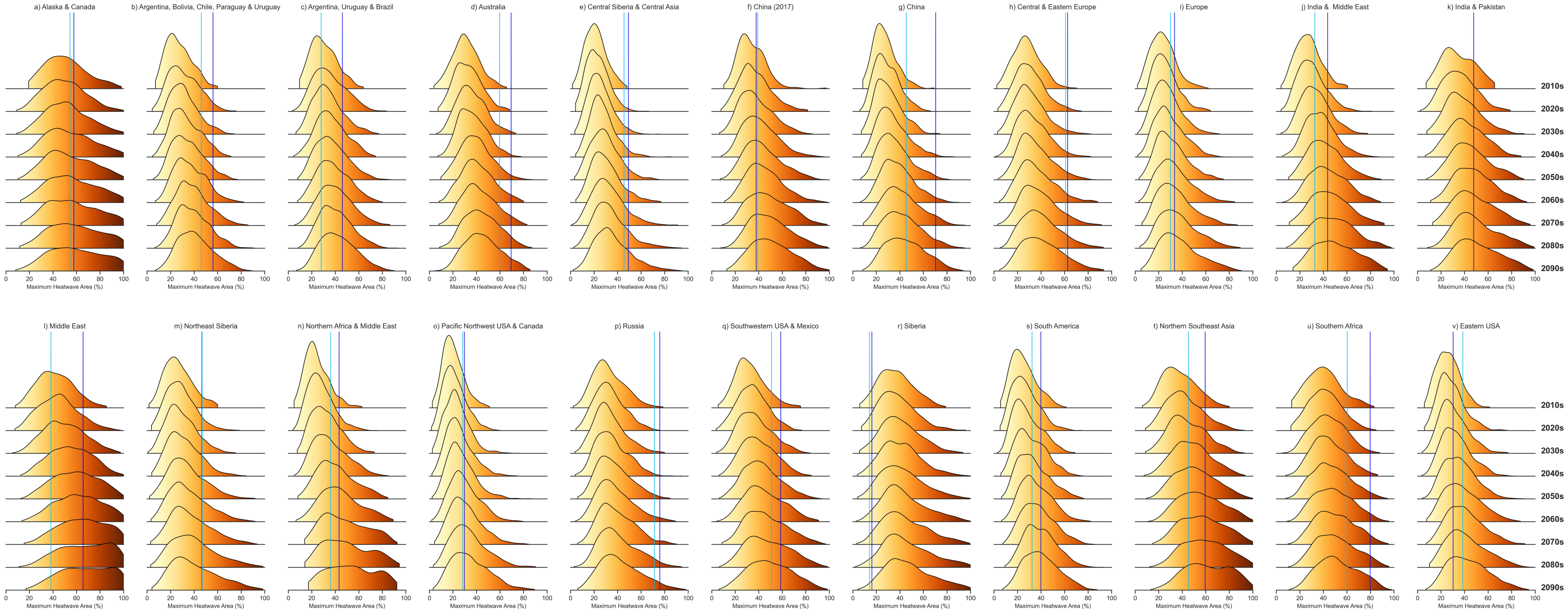
a) Alaska & Canada
b) Argentina, Bolivia, Chile, Paraguay & Uruguay
c) Argentina, Uruguay & Brazil
d) Australia
e) Central Siberia & Central Asia
f) China (2017)
g) China
h) Central & Eastern Europe
i) Europe
j) India & Middle East
k) India & Pakistan
l) Middle East
m) Northeast Siberia
n) Northern Africa & Middle East
o) Pacific Northwest USA & Canada
p) Russia
q) Southwestern USA & Mexico
r) Siberia
s) South America
t) Northern Southeast Asia
u) Southern Africa
v) Eastern USA
2010s
2020s
2030s
2040s
2050s
2060s
2070s
2080s
2090s
0
20
40
60
80
100
Maximum Heatwave Area (%)

Supplementary Figure 196: Kernel Density Estimates (KDE) across Decades for Maximum Heatwave Area (%) in SSP2-4.5 CMIP6 multi-model ensemble (MME) (Tx99.0p) - Kernel Density Estimates (KDE) of Maximum Heatwave Area (%) for different Decades in the CMIP6 multi-model ensemble (MME). Area under each curve is normalized to 1. Maximum Heatwave Area (%) of the observed event is indicated by vertical lines in dark blue (Berkeley Earth) and light blue (ERA5).

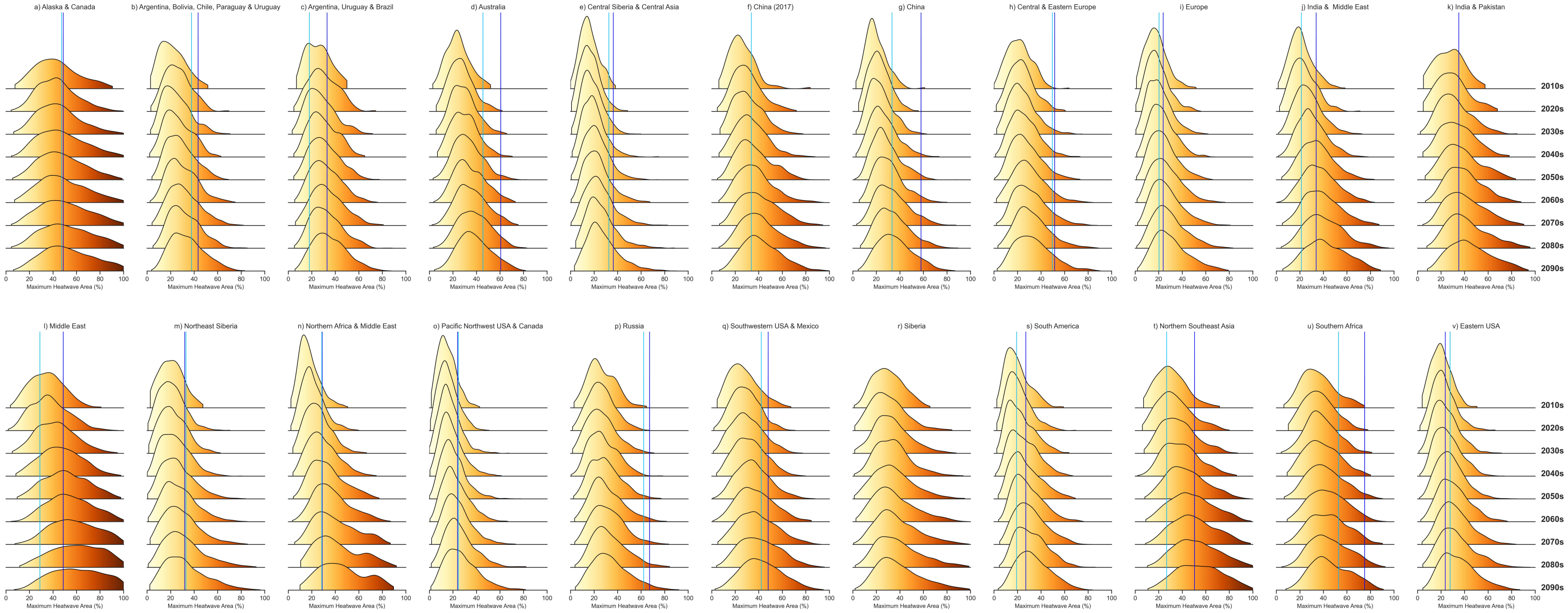

a) Alaska & Canada
b) Argentina, Bolivia, Chile, Paraguay & Uruguay
c) Argentina, Uruguay & Brazil
d) Australia
e) Central Siberia & Central Asia
f) China (2017)
g) China
h) Central & Eastern Europe
i) Europe
j) India & Middle East
k) India & Pakistan
l) Middle East
m) Northeast Siberia
n) Northern Africa & Middle East
o) Pacific Northwest USA & Canada
p) Russia
q) Southwestern USA & Mexico
r) Siberia
s) South America
t) Northern Southeast Asia
u) Southern Africa
v) Eastern USA
2010s
2020s
2030s
2040s
2050s
2060s
2070s
2080s
2090s
0 20 40 60 80 100
Maximum Heatwave Area (%)

Supplementary Figure 197: Kernel Density Estimates (KDE) across Decades for Maximum Heatwave Area (%) in SSP3-7.0 CMIP6 multi-model ensemble (MME) (Tx95.0p) - Kernel Density Estimates (KDE) of Maximum Heatwave Area (%) for different Decades in the CMIP6 multi-model ensemble (MME). Area under each curve is normalized to 1. Maximum Heatwave Area (%) of the observed event is indicated by vertical lines in dark blue (Berkeley Earth) and light blue (ERA5).

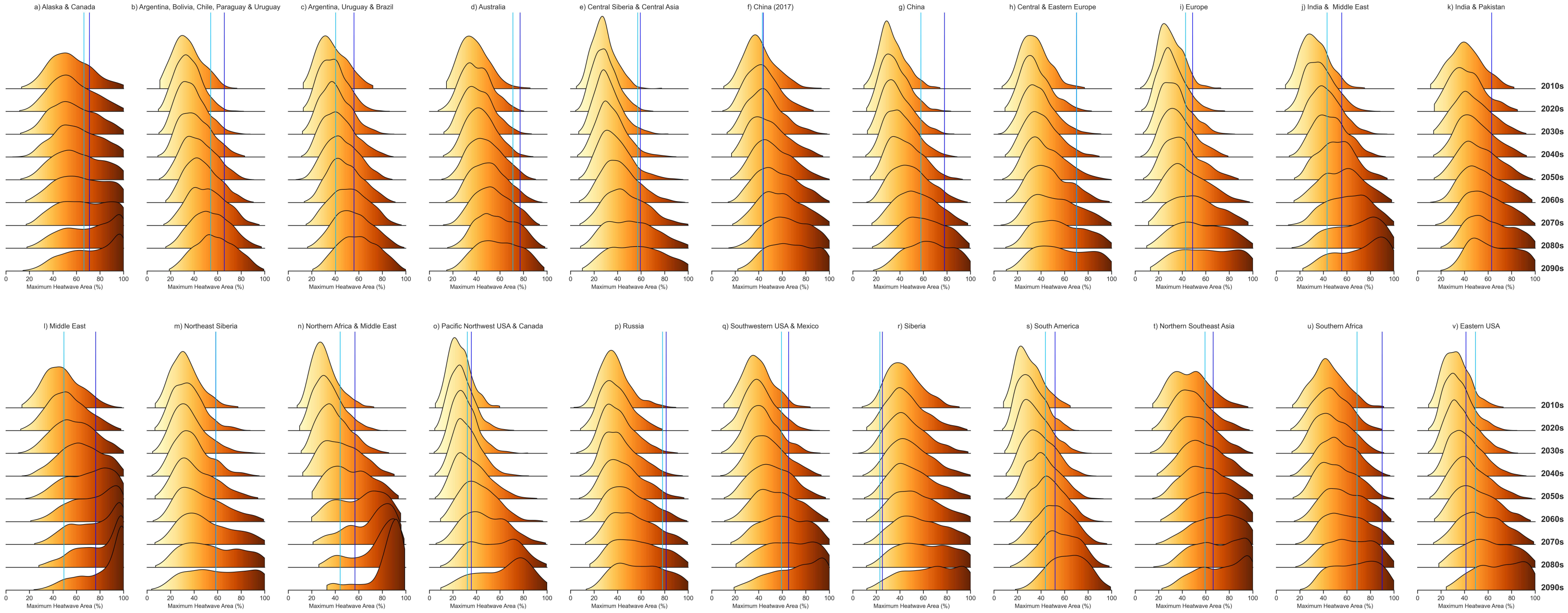

a) Alaska & Canada
b) Argentina, Bolivia, Chile, Paraguay & Uruguay
c) Argentina, Uruguay & Brazil
d) Australia
e) Central Siberia & Central Asia
f) China (2017)
g) China
h) Central & Eastern Europe
i) Europe
j) India & Middle East
k) India & Pakistan
l) Middle East
m) Northeast Siberia
n) Northern Africa & Middle East
o) Pacific Northwest USA & Canada
p) Russia
q) Southwestern USA & Mexico
r) Siberia
s) South America
t) Northern Southeast Asia
u) Southern Africa
v) Eastern USA
2010s
2020s
2030s
2040s
2050s
2060s
2070s
2080s
2090s
Maximum Heatwave Area (%)
0
20
40
60
80
100

Supplementary Figure 198: Kernel Density Estimates (KDE) across Decades for Maximum Heatwave Area (%) in SSP3-7.0 CMIP6 multi-model ensemble (MME) (Tx97.5p) - Kernel Density Estimates (KDE) of Maximum Heatwave Area (%) for different Decades in the CMIP6 multi-model ensemble (MME). Area under each curve is normalized to 1. Maximum Heatwave Area (%) of the observed event is indicated by vertical lines in dark blue (Berkeley Earth) and light blue (ERA5).

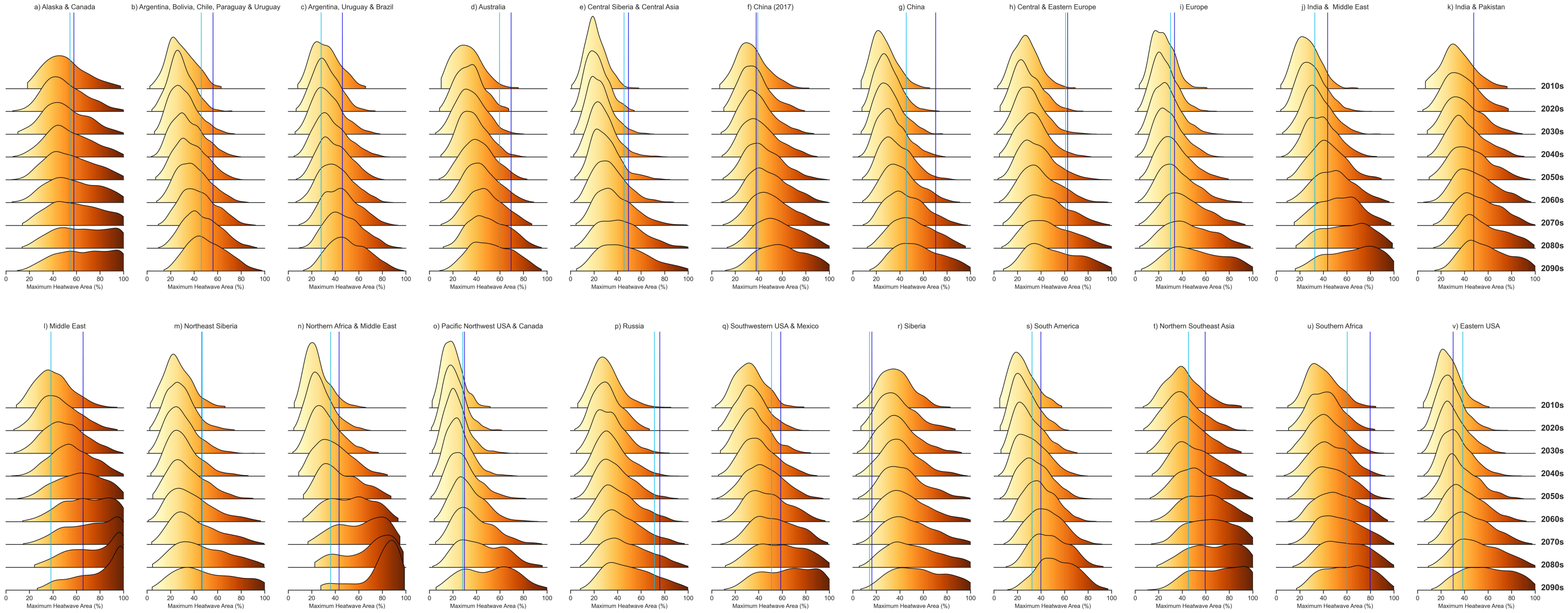

a) Alaska & Canada
b) Argentina, Bolivia, Chile, Paraguay & Uruguay
c) Argentina, Uruguay & Brazil
d) Australia
e) Central Siberia & Central Asia
f) China (2017)
g) China
h) Central & Eastern Europe
i) Europe
j) India & Middle East
k) India & Pakistan
l) Middle East
m) Northeast Siberia
n) Northern Africa & Middle East
o) Pacific Northwest USA & Canada
p) Russia
q) Southwestern USA & Mexico
r) Siberia
s) South America
t) Northern Southeast Asia
u) Southern Africa
v) Eastern USA
2010s
2020s
2030s
2040s
2050s
2060s
2070s
2080s
2090s
0 20 40 60 80 100
Maximum Heatwave Area (%)

Supplementary Figure 199: Kernel Density Estimates (KDE) across Decades for Maximum Heatwave Area (%) in SSP3-7.0 CMIP6 multi-model ensemble (MME) (Tx99.0p) - Kernel Density Estimates (KDE) of Maximum Heatwave Area (%) for different Decades in the CMIP6 multi-model ensemble (MME). Area under each curve is normalized to 1. Maximum Heatwave Area (%) of the observed event is indicated by vertical lines in dark blue (Berkeley Earth) and light blue (ERA5).

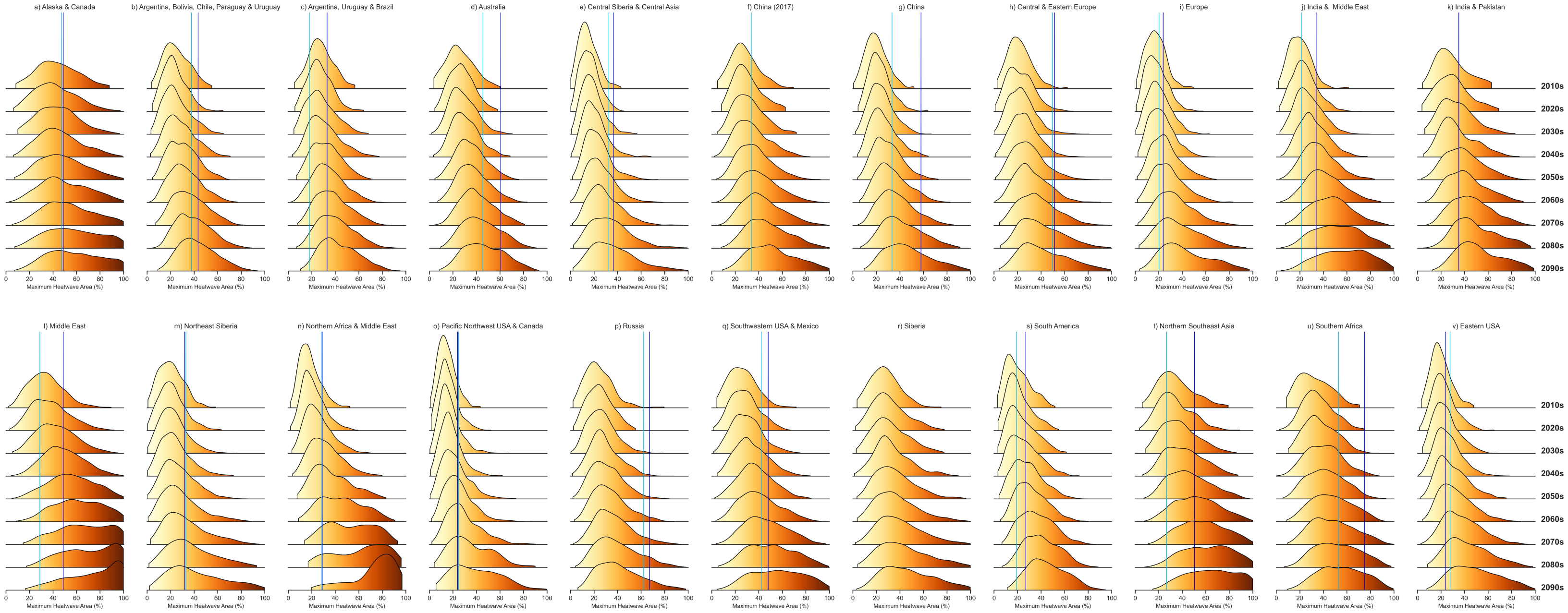

a) Alaska & Canada
b) Argentina, Bolivia, Chile, Paraguay & Uruguay
c) Argentina, Uruguay & Brazil
d) Australia
e) Central Siberia & Central Asia
f) China (2017)
g) China
h) Central & Eastern Europe
i) Europe
j) India & Middle East
k) India & Pakistan
l) Middle East
m) Northeast Siberia
n) Northern Africa & Middle East
o) Pacific Northwest USA & Canada
p) Russia
q) Southwestern USA & Mexico
r) Siberia
s) South America
t) Northern Southeast Asia
u) Southern Africa
v) Eastern USA
2010s
2020s
2030s
2040s
2050s
2060s
2070s
2080s
2090s
0 20 40 60 80 100
Maximum Heatwave Area (%)

Supplementary Figure 200: Kernel Density Estimates (KDE) across Decades for Maximum Heatwave Area (%) in SSP5-8.5 CMIP6 multi-model ensemble (MME) (Tx95.0p) - Kernel Density Estimates (KDE) of Maximum Heatwave Area (%) for different Decades in the CMIP6 multi-model ensemble (MME). Area under each curve is normalized to 1. Maximum Heatwave Area (%) of the observed event is indicated by vertical lines in dark blue (Berkeley Earth) and light blue (ERA5).

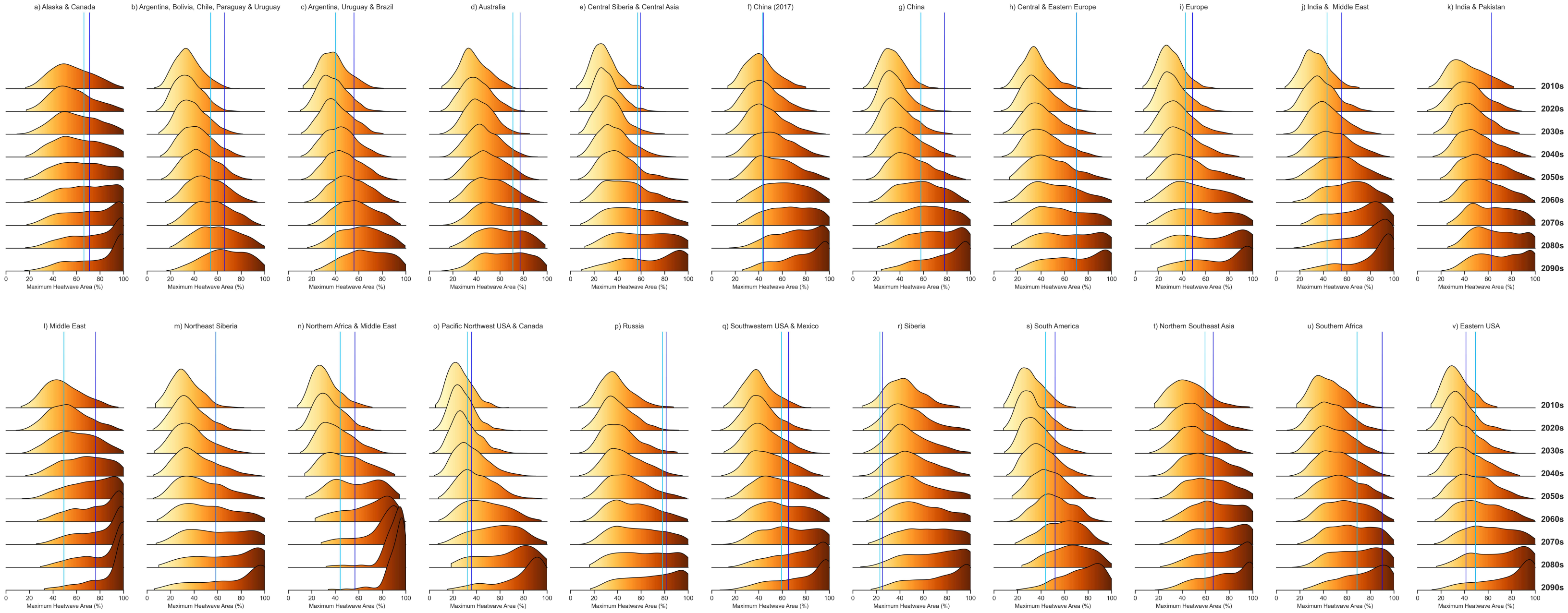
a) Alaska & Canada
b) Argentina, Bolivia, Chile, Paraguay & Uruguay
c) Argentina, Uruguay & Brazil
d) Australia
e) Central Siberia & Central Asia
f) China (2017)
g) China
h) Central & Eastern Europe
i) Europe
j) India & Middle East
k) India & Pakistan
l) Middle East
m) Northeast Siberia
n) Northern Africa & Middle East
o) Pacific Northwest USA & Canada
p) Russia
q) Southwestern USA & Mexico
r) Siberia
s) South America
t) Northern Southeast Asia
u) Southern Africa
v) Eastern USA
2010s
2020s
2030s
2040s
2050s
2060s
2070s
2080s
2090s
0
20
40
60
80
100
Maximum Heatwave Area (%)

Supplementary Figure 201: Kernel Density Estimates (KDE) across Decades for Maximum Heatwave Area (%) in SSP5-8.5 CMIP6 multi-model ensemble (MME) (Tx97.5p) - Kernel Density Estimates (KDE) of Maximum Heatwave Area (%) for different Decades in the CMIP6 multi-model ensemble (MME). Area under each curve is normalized to 1. Maximum Heatwave Area (%) of the observed event is indicated by vertical lines in dark blue (Berkeley Earth) and light blue (ERA5).

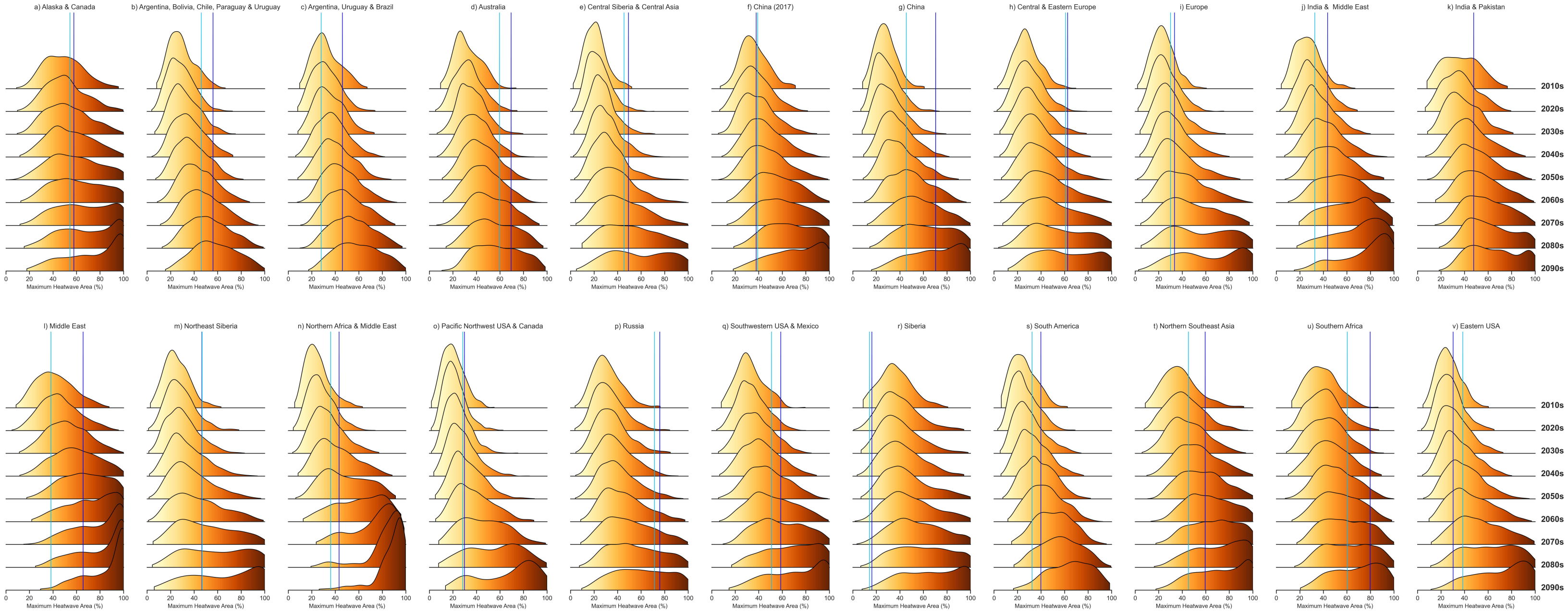
a) Alaska & Canada
b) Argentina, Bolivia, Chile, Paraguay & Uruguay
c) Argentina, Uruguay & Brazil
d) Australia
e) Central Siberia & Central Asia
f) China (2017)
g) China
h) Central & Eastern Europe
i) Europe
j) India & Middle East
k) India & Pakistan
l) Middle East
m) Northeast Siberia
n) Northern Africa & Middle East
o) Pacific Northwest USA & Canada
p) Russia
q) Southwestern USA & Mexico
r) Siberia
s) South America
t) Northern Southeast Asia
u) Southern Africa
v) Eastern USA
2010s
2020s
2030s
2040s
2050s
2060s
2070s
2080s
2090s
0
20
40
60
80
100
Maximum Heatwave Area (%)

Supplementary Figure 202: Kernel Density Estimates (KDE) across Decades for Maximum Heatwave Area (%) in SSP5-8.5 CMIP6 multi-model ensemble (MME) (Tx99.0p) - Kernel Density Estimates (KDE) of Maximum Heatwave Area (%) for different Decades in the CMIP6 multi-model ensemble (MME). Area under each curve is normalized to 1. Maximum Heatwave Area (%) of the observed event is indicated by vertical lines in dark blue (Berkeley Earth) and light blue (ERA5).

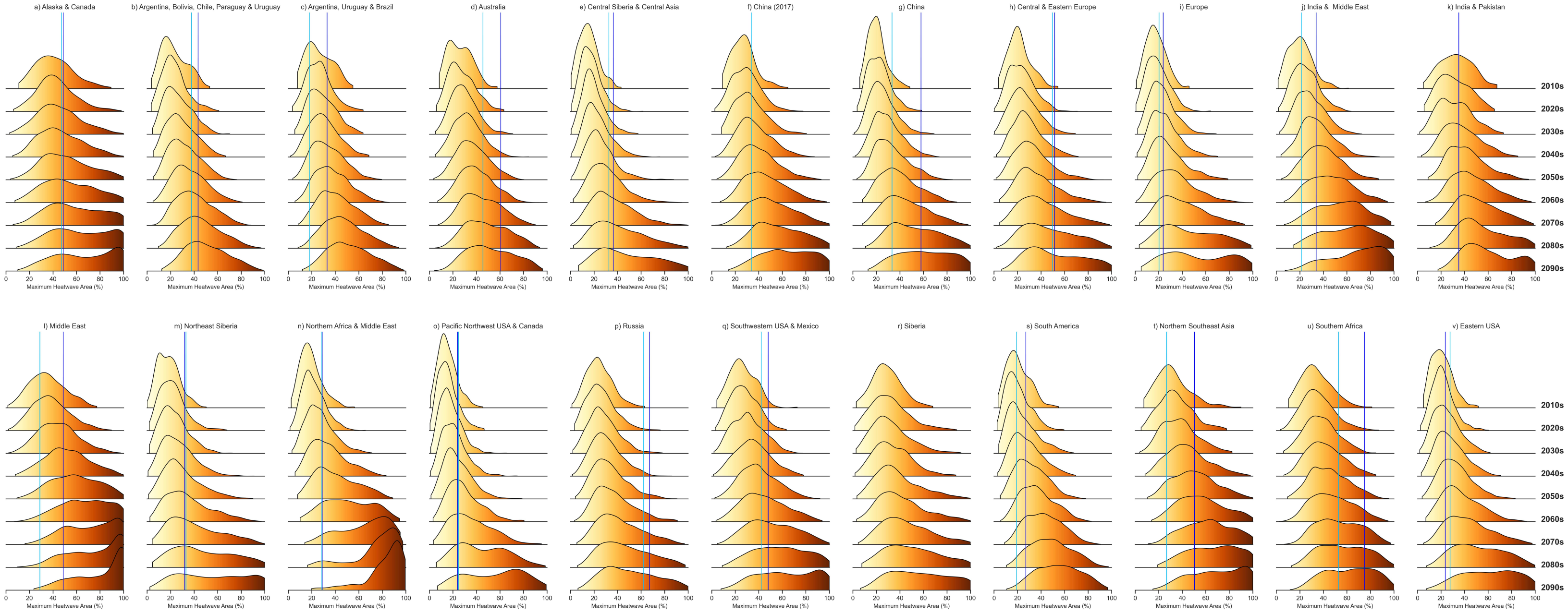
a) Alaska & Canada
b) Argentina, Bolivia, Chile, Paraguay & Uruguay
c) Argentina, Uruguay & Brazil
d) Australia
e) Central Siberia & Central Asia
f) China (2017)
g) China
h) Central & Eastern Europe
i) Europe
j) India & Middle East
k) India & Pakistan
l) Middle East
m) Northeast Siberia
n) Northern Africa & Middle East
o) Pacific Northwest USA & Canada
p) Russia
q) Southwestern USA & Mexico
r) Siberia
s) South America
t) Northern Southeast Asia
u) Southern Africa
v) Eastern USA
2010s
2020s
2030s
2040s
2050s
2060s
2070s
2080s
2090s
0
20
40
60
80
100
Maximum Heatwave Area (%)

Supplementary Figure 203: Multi-HW-characteristic figure according to SSP1-2.6 CMIP6 multi-model ensemble (MME) per model for Alaska & Canada (Tx90p) - Each panel shows regional projections according to SSP1-2.6 model runs in the CMIP6 multi-model ensemble (MME) of four combined heatwave characteristics for each projected heatwave in the ensemble between 2020 and 2100. Each dot represents an individual heatwave in the projections, characterized by Median Heatwave Area (%) (x-axis) as latitude-weighted percentage of grid boxes affected by extreme heat, Heatwave Length (days) (y-axis) in days, Median Heatwave Severity (color), and Cumulative Heat (°C) (dot size). The characteristics of the observed event according to the Berkeley Earth observational data (x symbol) and the ERA5 reanalysis data (+ symbol), respectively, are pointed to by an arrow and a figure inset shows a zoom-in on this figure section. The figure inset shows the observed heatwave in the two reference data sets and projections in the indicated section of the respective sub-plot. It facilitates readability of color and size of the reference values and comparison to each other and the neighboring projections. Note that the zoom-in causes the dots to overlap more in the inset than in the main sub-plot. The univariate kernel density estimates along the x- (Median Heatwave Area (%)) and y-axis (Heatwave Length (days)) are on the top and right side of the subplots, respectively, with vertical lines in dark blue (Berkeley Earth) and light blue (ERA5) indicating the observed event. The respective region is indicated above the color bar.

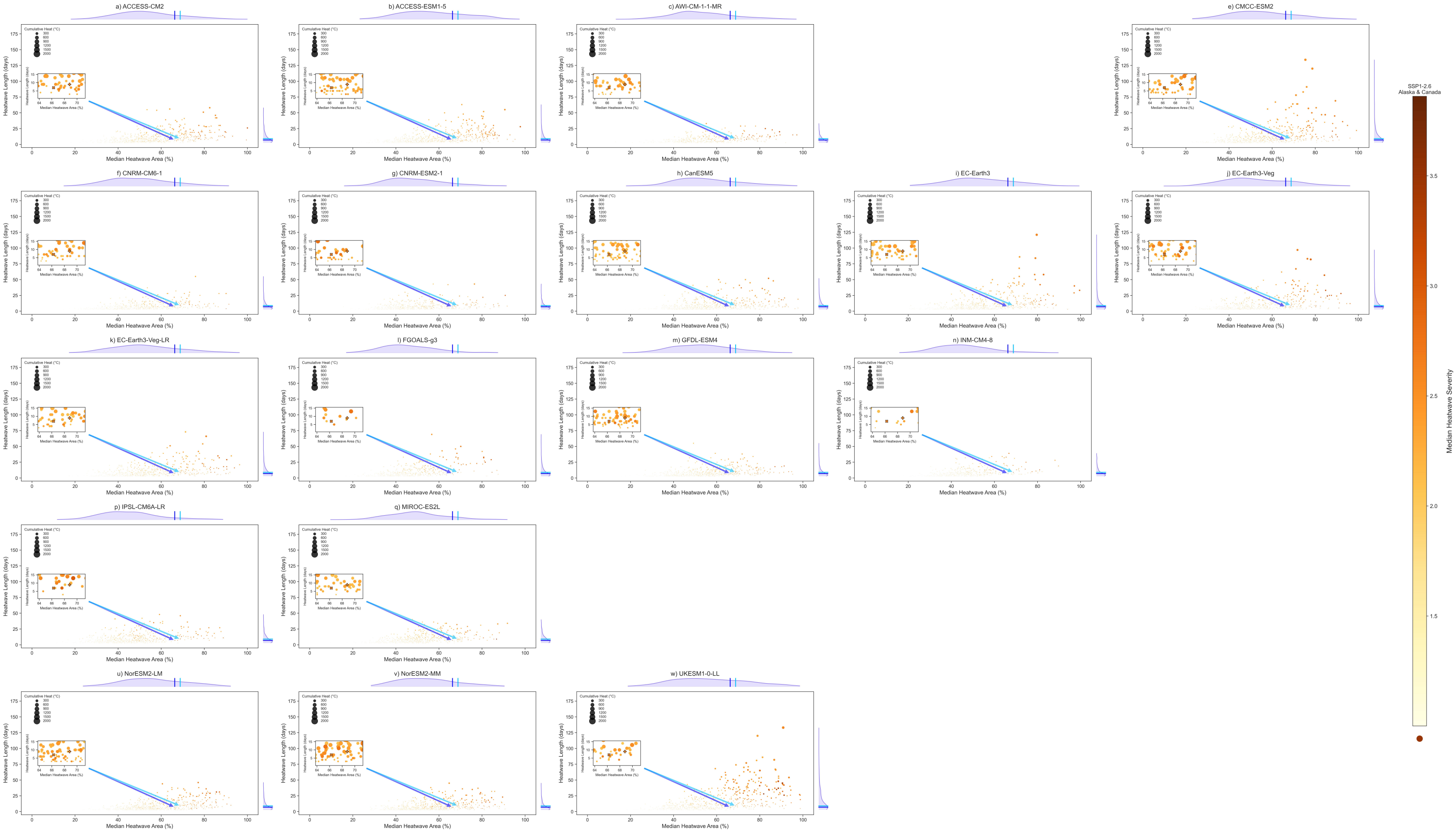

a) ACCESS-CM2
b) ACCESS-ESM1-5
c) AWI-CM-1-1-MR
e) CMCC-ESM2
f) CNRM-CM6-1
g) CNRM-ESM2-1
h) CanESM5
i) EC-Earth3
j) EC-Earth3-Veg
k) EC-Earth3-Veg-LR
l) FGOALS-g3
m) GFDL-ESM4
n) INM-CM4-8
p) IPSL-CM6A-LR
q) MIROC-ES2L
u) NorESM2-LM
v) NorESM2-MM
w) UKESM1-0-LL
Heatwave Length (days)
Median Heatwave Area (%)
Cumulative Heat (°C)
300
600
900
1200
1500
2000
SSP1-2.6
Alaska & Canada
Median Heatwave Severity

Supplementary Figure 204: Multi-HW-characteristic figure according to SSP1-2.6 CMIP6 multi-model ensemble (MME) per model for Argentina, Bolivia, Chile, Paraguay & Uruguay (Tx90p) - Each panel shows regional projections according to SSP1-2.6 model runs in the CMIP6 multi-model ensemble (MME) of four combined heatwave characteristics for each projected heatwave in the ensemble between 2020 and 2100. Each dot represents an individual heatwave in the projections, characterized by Median Heatwave Area (%) (x-axis) as latitude-weighted percentage of grid boxes affected by extreme heat, Heatwave Length (days) (y-axis) in days, Median Heatwave Severity (color), and Cumulative Heat (°C) (dot size). The characteristics of the observed event according to the Berkeley Earth observational data (x symbol) and the ERA5 reanalysis data (+ symbol), respectively, are pointed to by an arrow and a figure inset shows a zoom-in on this figure section. The figure inset shows the observed heatwave in the two reference data sets and projections in the indicated section of the respective sub-plot. It facilitates readability of color and size of the reference values and comparison to each other and the neighboring projections. Note that the zoom-in causes the dots to overlap more in the inset than in the main sub-plot. The univariate kernel density estimates along the x- (Median Heatwave Area (%)) and y-axis (Heatwave Length (days)) are on the top and right side of the subplots, respectively, with vertical lines in dark blue (Berkeley Earth) and light blue (ERA5) indicating the observed event. The respective region is indicated above the color bar.

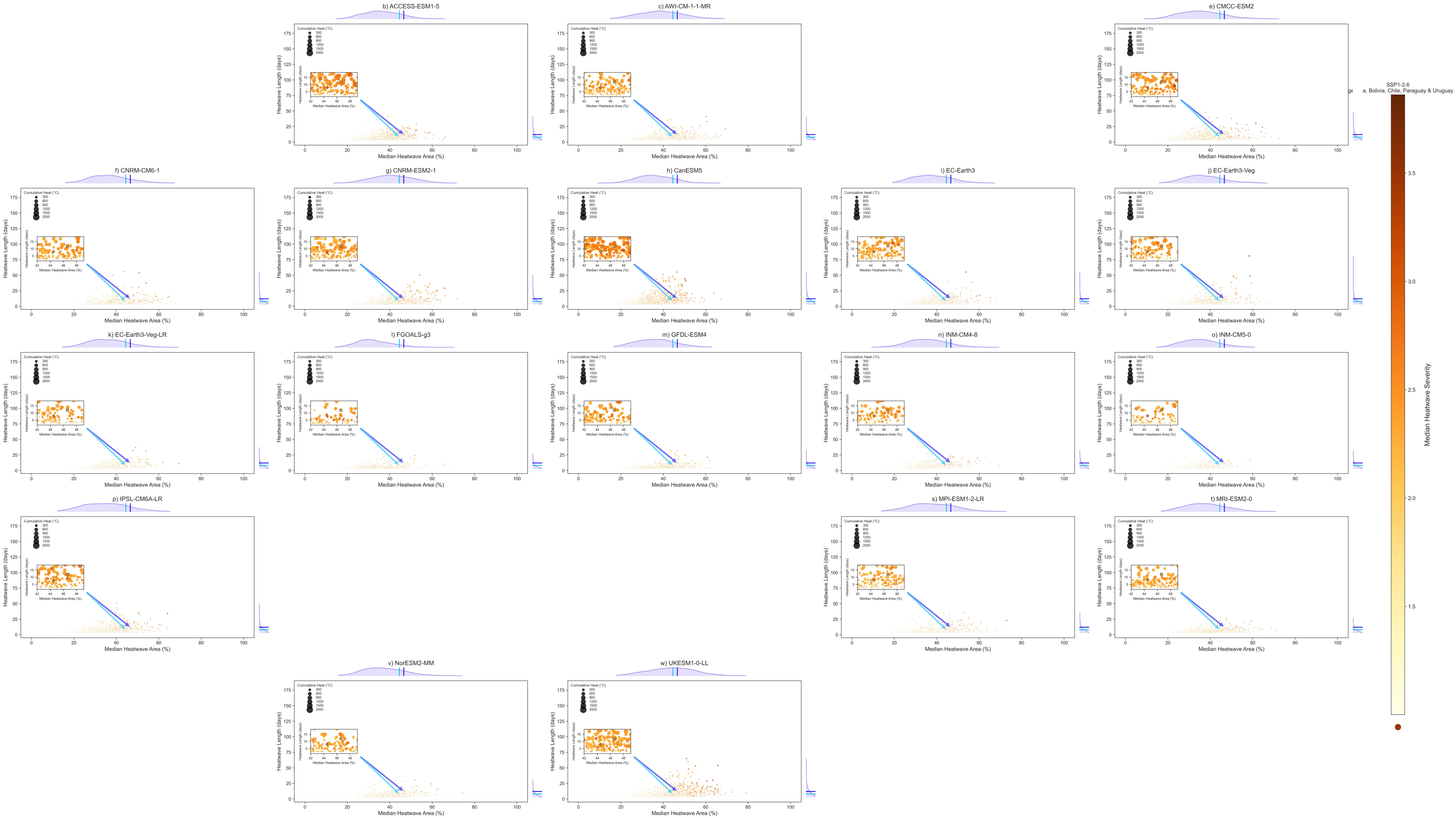
b) ACCESS-ESM1-5
c) AWI-CM-1-1-MR
e) CMCC-ESM2
f) CNRM-CM6-1
g) CNRM-ESM2-1
h) CanESM5
i) EC-Earth3
j) EC-Earth3-Veg
k) EC-Earth3-Veg-LR
l) FGOALS-g3
m) GFDL-ESM4
n) INM-CM4-8
o) INM-CM5-0
p) IPSL-CM6A-LR
s) MPI-ESM1-2-LR
t) MRI-ESM2-0
v) NorESM2-MM
w) UKESM1-0-LL
Cumulative Heat (°C)
300
600
900
1200
1500
2000
Heatwave Length (days)
Median Heatwave Area (%)
SSP1-2.6
a, Bolivia, Chile, Paraguay & Uruguay
Median Heatwave Severity

Supplementary Figure 205: Multi-HW-characteristic figure according to SSP1-2.6 CMIP6 multi-model ensemble (MME) per model for Argentina, Uruguay & Brazil (Tx90p) - Each panel shows regional projections according to SSP1-2.6 model runs in the CMIP6 multi-model ensemble (MME) of four combined heatwave characteristics for each projected heatwave in the ensemble between 2020 and 2100. Each dot represents an individual heatwave in the projections, characterized by Median Heatwave Area (%) (x-axis) as latitude-weighted percentage of grid boxes affected by extreme heat, Heatwave Length (days) (y-axis) in days, Median Heatwave Severity (color), and Cumulative Heat (°C) (dot size). The characteristics of the observed event according to the Berkeley Earth observational data (x symbol) and the ERA5 reanalysis data (+ symbol), respectively, are pointed to by an arrow and a figure inset shows a zoom-in on this figure section. The figure inset shows the observed heatwave in the two reference data sets and projections in the indicated section of the respective sub-plot. It facilitates readability of color and size of the reference values and comparison to each other and the neighboring projections. Note that the zoom-in causes the dots to overlap more in the inset than in the main sub-plot. The univariate kernel density estimates along the x- (Median Heatwave Area (%)) and y-axis (Heatwave Length (days)) are on the top and right side of the subplots, respectively, with vertical lines in dark blue (Berkeley Earth) and light blue (ERA5) indicating the observed event. The respective region is indicated above the color bar.

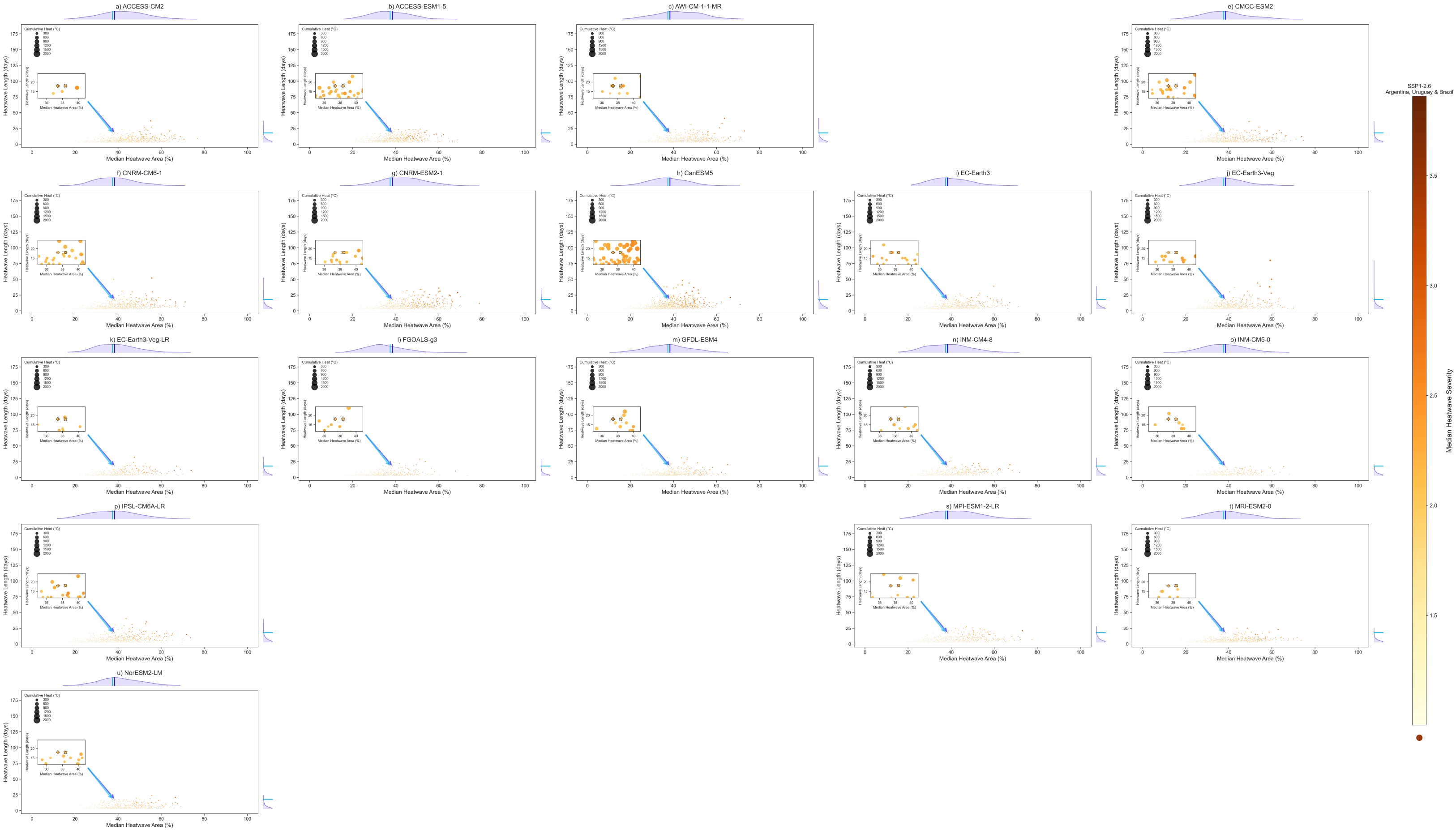
a) ACCESS-CM2
b) ACCESS-ESM1-5
c) AWI-CM-1-1-MR
e) CMCC-ESM2
f) CNRM-CM6-1
g) CNRM-ESM2-1
h) CanESM5
i) EC-Earth3
j) EC-Earth3-Veg
k) EC-Earth3-Veg-LR
l) FGOALS-g3
m) GFDL-ESM4
n) INM-CM4-8
o) INM-CM5-0
p) IPSL-CM6A-LR
s) MPI-ESM1-2-LR
t) MRI-ESM2-0
u) NorESM2-LM
Cumulative Heat (°C)
300
600
900
1200
1500
2000
Heatwave Length (days)
Median Heatwave Area (%)
SSP1-2.6
Argentina, Uruguay & Brazil
Median Heatwave Severity

Supplementary Figure 206: Multi-HW-characteristic figure according to SSP1-2.6 CMIP6 multi-model ensemble (MME) per model for Australia (Tx90p) - Each panel shows regional projections according to SSP1-2.6 model runs in the CMIP6 multi-model ensemble (MME) of four combined heatwave characteristics for each projected heatwave in the ensemble between 2020 and 2100. Each dot represents an individual heatwave in the projections, characterized by Median Heatwave Area (%) (x-axis) as latitude-weighted percentage of grid boxes affected by extreme heat, Heatwave Length (days) (y-axis) in days, Median Heatwave Severity (color), and Cumulative Heat (°C) (dot size). The characteristics of the observed event according to the Berkeley Earth observational data (x symbol) and the ERA5 reanalysis data (+ symbol), respectively, are pointed to by an arrow and a figure inset shows a zoom-in on this figure section. The figure inset shows the observed heatwave in the two reference data sets and projections in the indicated section of the respective sub-plot. It facilitates readability of color and size of the reference values and comparison to each other and the neighboring projections. Note that the zoom-in causes the dots to overlap more in the inset than in the main sub-plot. The univariate kernel density estimates along the x- (Median Heatwave Area (%)) and y-axis (Heatwave Length (days)) are on the top and right side of the subplots, respectively, with vertical lines in dark blue (Berkeley Earth) and light blue (ERA5) indicating the observed event. The respective region is indicated above the color bar.

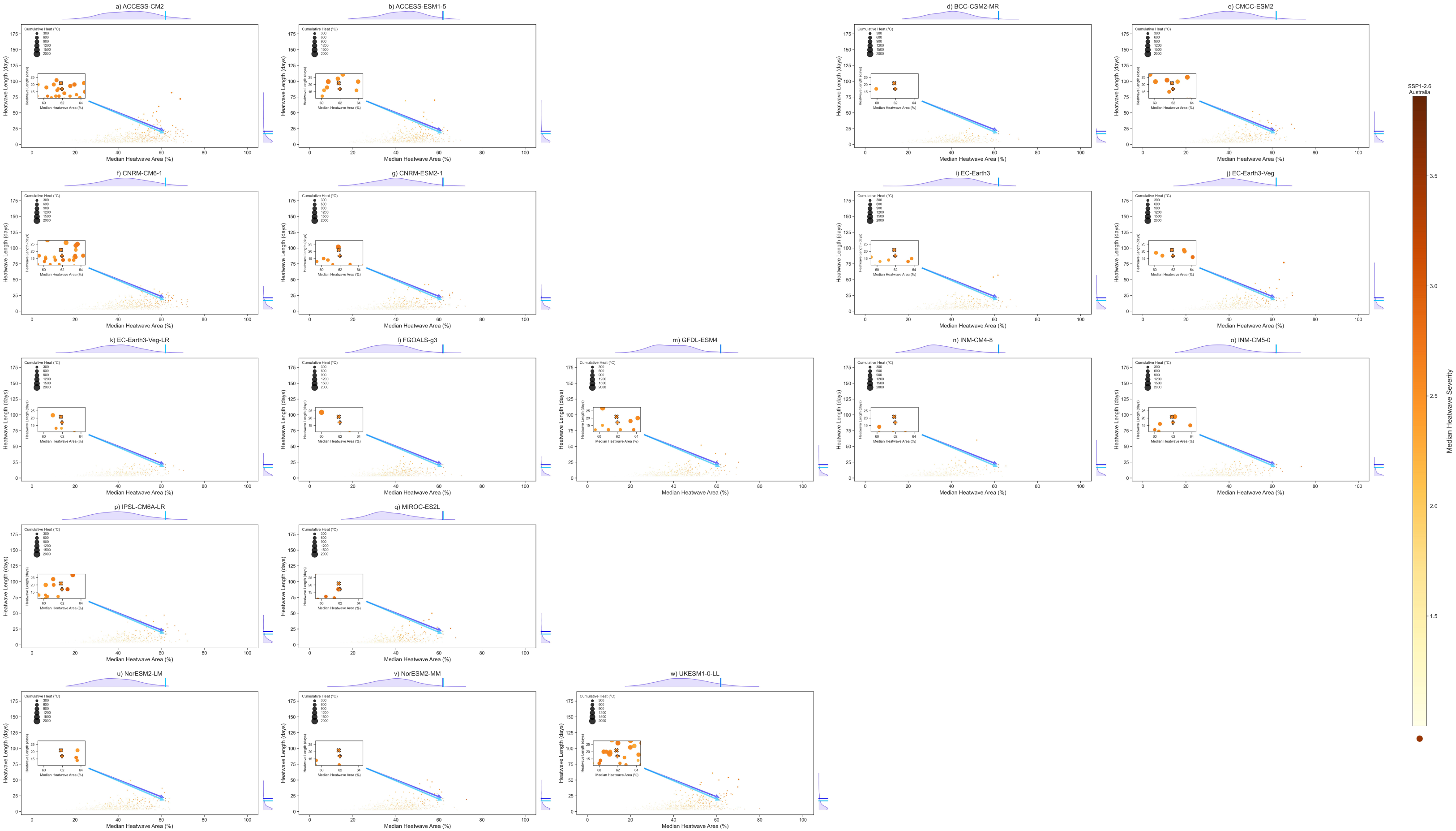

a) ACCESS-CM2
b) ACCESS-ESM1-5
d) BCC-CSM2-MR
e) CMCC-ESM2
f) CNRM-CM6-1
g) CNRM-ESM2-1
i) EC-Earth3
j) EC-Earth3-Veg
k) EC-Earth3-Veg-LR
l) FGOALS-g3
m) GFDL-ESM4
n) INM-CM4-8
o) INM-CM5-0
p) IPSL-CM6A-LR
q) MIROC-ES2L
u) NorESM2-LM
v) NorESM2-MM
w) UKESM1-0-LL
Cumulative Heat (°C)
300
600
900
1200
1500
2000
Heatwave Length (days)
Median Heatwave Area (%)
SSP1-2.6
Australia
Median Heatwave Severity

Supplementary Figure 207: Multi-HW-characteristic figure according to SSP1-2.6 CMIP6 multi-model ensemble (MME) per model for Central Siberia & Central Asia (Tx90p) - Each panel shows regional projections according to SSP1-2.6 model runs in the CMIP6 multi-model ensemble (MME) of four combined heatwave characteristics for each projected heatwave in the ensemble between 2020 and 2100. Each dot represents an individual heatwave in the projections, characterized by Median Heatwave Area (%) (x-axis) as latitude-weighted percentage of grid boxes affected by extreme heat, Heatwave Length (days) (y-axis) in days, Median Heatwave Severity (color), and Cumulative Heat (°C) (dot size). The characteristics of the observed event according to the Berkeley Earth observational data (x symbol) and the ERA5 reanalysis data (+ symbol), respectively, are pointed to by an arrow and a figure inset shows a zoom-in on this figure section. The figure inset shows the observed heatwave in the two reference data sets and projections in the indicated section of the respective sub-plot. It facilitates readability of color and size of the reference values and comparison to each other and the neighboring projections. Note that the zoom-in causes the dots to overlap more in the inset than in the main sub-plot. The univariate kernel density estimates along the x- (Median Heatwave Area (%)) and y-axis (Heatwave Length (days)) are on the top and right side of the subplots, respectively, with vertical lines in dark blue (Berkeley Earth) and light blue (ERA5) indicating the observed event. The respective region is indicated above the color bar.

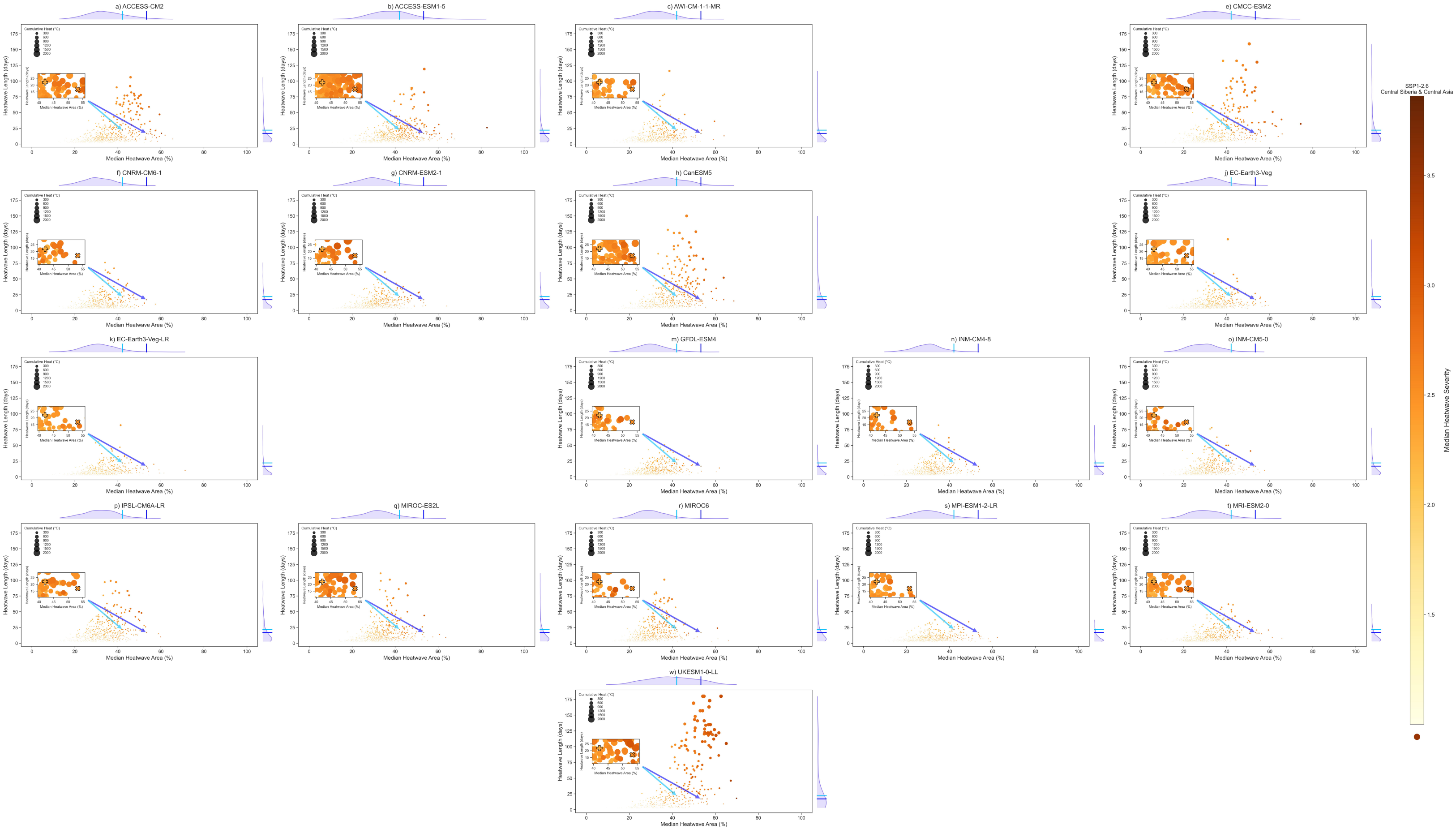

a) ACCESS-CM2
b) ACCESS-ESM1-5
c) AWI-CM-1-1-MR
e) CMCC-ESM2
f) CNRM-CM6-1
g) CNRM-ESM2-1
h) CanESM5
j) EC-Earth3-Veg
k) EC-Earth3-Veg-LR
m) GFDL-ESM4
n) INM-CM4-8
o) INM-CM5-0
p) IPSL-CM6A-LR
q) MIROC-ES2L
r) MIROC6
s) MPI-ESM1-2-LR
t) MRI-ESM2-0
w) UKESM1-0-LL
Cumulative Heat (°C)
300
600
900
1200
1500
2000
Heatwave Length (days)
Median Heatwave Area (%)
SSP1-2.6
Central Siberia & Central Asia
Median Heatwave Severity

Supplementary Figure 208: Multi-HW-characteristic figure according to SSP1-2.6 CMIP6 multi-model ensemble (MME) per model for China (2017) (Tx90p) - Each panel shows regional projections according to SSP1-2.6 model runs in the CMIP6 multi-model ensemble (MME) of four combined heatwave characteristics for each projected heatwave in the ensemble between 2020 and 2100. Each dot represents an individual heatwave in the projections, characterized by Median Heatwave Area (%) (x-axis) as latitude-weighted percentage of grid boxes affected by extreme heat, Heatwave Length (days) (y-axis) in days, Median Heatwave Severity (color), and Cumulative Heat (°C) (dot size). The characteristics of the observed event according to the Berkeley Earth observational data (x symbol) and the ERA5 reanalysis data (+ symbol), respectively, are pointed to by an arrow and a figure inset shows a zoom-in on this figure section. The figure inset shows the observed heatwave in the two reference data sets and projections in the indicated section of the respective sub-plot. It facilitates readability of color and size of the reference values and comparison to each other and the neighboring projections. Note that the zoom-in causes the dots to overlap more in the inset than in the main sub-plot. The univariate kernel density estimates along the x- (Median Heatwave Area (%)) and y-axis (Heatwave Length (days)) are on the top and right side of the subplots, respectively, with vertical lines in dark blue (Berkeley Earth) and light blue (ERA5) indicating the observed event. The respective region is indicated above the color bar.

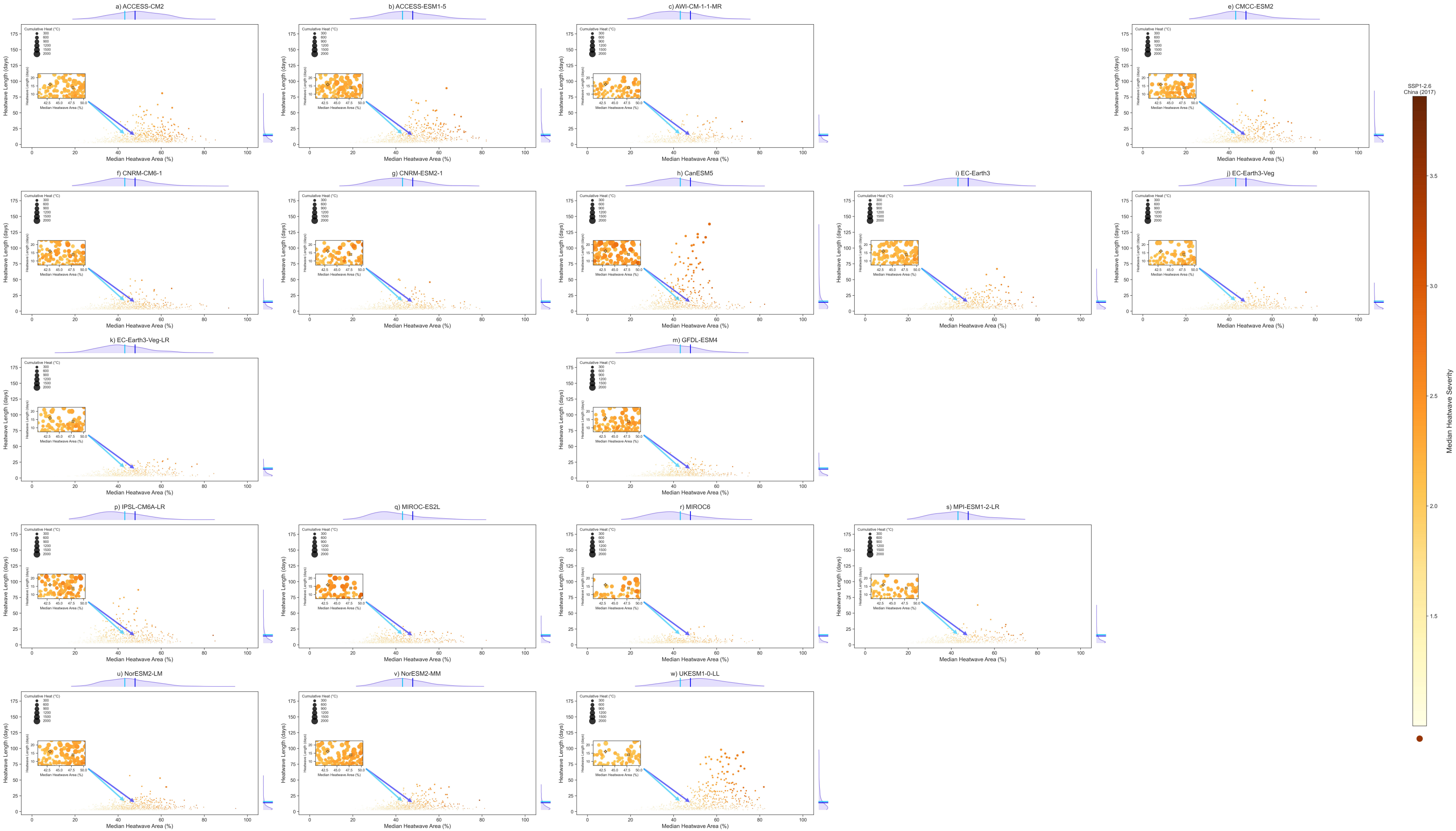

a) ACCESS-CM2
b) ACCESS-ESM1-5
c) AWI-CM-1-1-MR
e) CMCC-ESM2
f) CNRM-CM6-1
g) CNRM-ESM2-1
h) CanESM5
i) EC-Earth3
j) EC-Earth3-Veg
k) EC-Earth3-Veg-LR
m) GFDL-ESM4
p) IPSL-CM6A-LR
q) MIROC-ES2L
r) MIROC6
s) MPI-ESM1-2-LR
u) NorESM2-LM
v) NorESM2-MM
w) UKESM1-0-LL
Cumulative Heat (°C)
300
600
900
1200
1500
2000
Heatwave Length (days)
Median Heatwave Area (%)
SSP1-2.6
China (2017)
Median Heatwave Severity
1.5
2.0
2.5
3.0
3.5

Supplementary Figure 209: Multi-HW-characteristic figure according to SSP1-2.6 CMIP6 multi-model ensemble (MME) per model for China (Tx90p) - Each panel shows regional projections according to SSP1-2.6 model runs in the CMIP6 multi-model ensemble (MME) of four combined heatwave characteristics for each projected heatwave in the ensemble between 2020 and 2100. Each dot represents an individual heatwave in the projections, characterized by Median Heatwave Area (%) (x-axis) as latitude-weighted percentage of grid boxes affected by extreme heat, Heatwave Length (days) (y-axis) in days, Median Heatwave Severity (color), and Cumulative Heat (°C) (dot size). The characteristics of the observed event according to the Berkeley Earth observational data (x symbol) and the ERA5 reanalysis data (+ symbol), respectively, are pointed to by an arrow and a figure inset shows a zoom-in on this figure section. The figure inset shows the observed heatwave in the two reference data sets and projections in the indicated section of the respective sub-plot. It facilitates readability of color and size of the reference values and comparison to each other and the neighboring projections. Note that the zoom-in causes the dots to overlap more in the inset than in the main sub-plot. The univariate kernel density estimates along the x- (Median Heatwave Area (%)) and y-axis (Heatwave Length (days)) are on the top and right side of the subplots, respectively, with vertical lines in dark blue (Berkeley Earth) and light blue (ERA5) indicating the observed event. The respective region is indicated above the color bar.

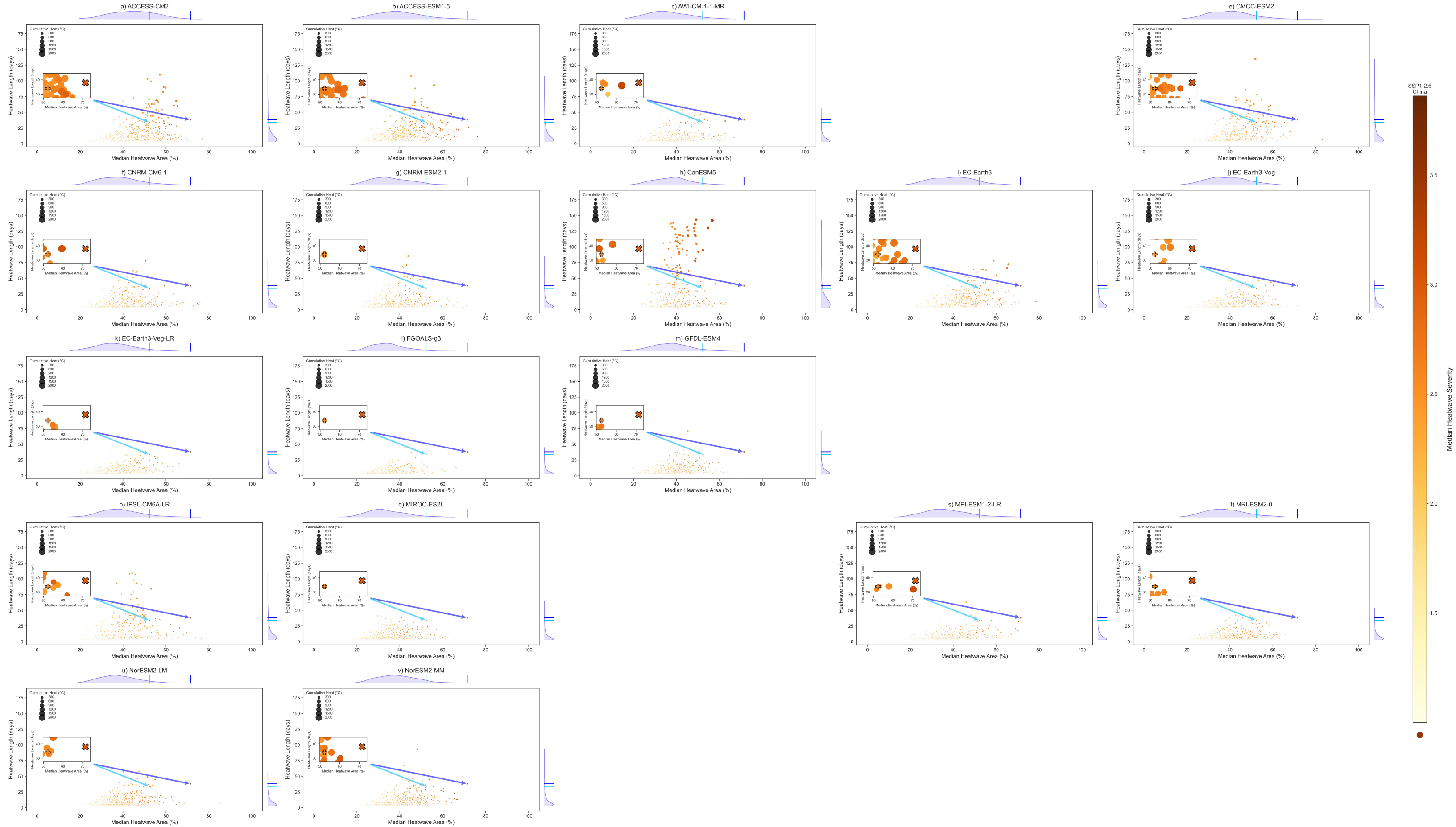
a) ACCESS-CM2
b) ACCESS-ESM1-5
c) AWI-CM-1-1-MR
e) CMCC-ESM2
f) CNRM-CM6-1
g) CNRM-ESM2-1
h) CanESM5
i) EC-Earth3
j) EC-Earth3-Veg
k) EC-Earth3-Veg-LR
l) FGOALS-g3
m) GFDL-ESM4
p) IPSL-CM6A-LR
q) MIROC-ES2L
s) MPI-ESM1-2-LR
t) MRI-ESM2-0
u) NorESM2-LM
v) NorESM2-MM
Cumulative Heat (°C)
300
600
900
1200
1500
2000
Heatwave Length (days)
Median Heatwave Area (%)
SSP1-2.6
China
Median Heatwave Severity

Supplementary Figure 210: Multi-HW-characteristic figure according to SSP1-2.6 CMIP6 multi-model ensemble (MME) per model for Central & Eastern Europe (Tx90p) - Each panel shows regional projections according to SSP1-2.6 model runs in the CMIP6 multi-model ensemble (MME) of four combined heatwave characteristics for each projected heatwave in the ensemble between 2020 and 2100. Each dot represents an individual heatwave in the projections, characterized by Median Heatwave Area (%) (x-axis) as latitude-weighted percentage of grid boxes affected by extreme heat, Heatwave Length (days) (y-axis) in days, Median Heatwave Severity (color), and Cumulative Heat (°C) (dot size). The characteristics of the observed event according to the Berkeley Earth observational data (x symbol) and the ERA5 reanalysis data (+ symbol), respectively, are pointed to by an arrow and a figure inset shows a zoom-in on this figure section. The figure inset shows the observed heatwave in the two reference data sets and projections in the indicated section of the respective sub-plot. It facilitates readability of color and size of the reference values and comparison to each other and the neighboring projections. Note that the zoom-in causes the dots to overlap more in the inset than in the main sub-plot. The univariate kernel density estimates along the x- (Median Heatwave Area (%)) and y-axis (Heatwave Length (days)) are on the top and right side of the subplots, respectively, with vertical lines in dark blue (Berkeley Earth) and light blue (ERA5) indicating the observed event. The respective region is indicated above the color bar.

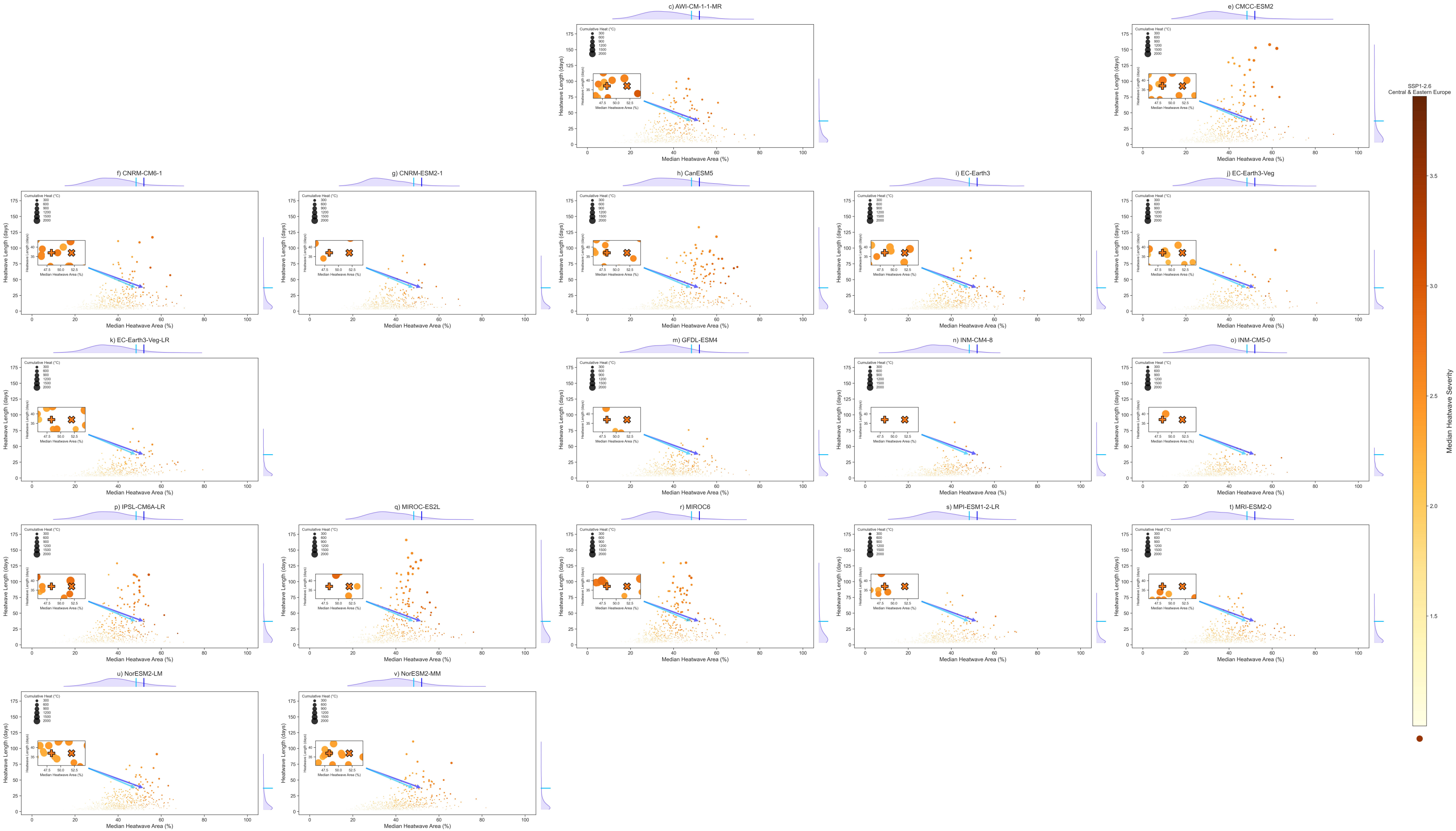

c) AWI-CM-1-1-MR
e) CMCC-ESM2
f) CNRM-CM6-1
g) CNRM-ESM2-1
h) CanESM5
i) EC-Earth3
j) EC-Earth3-Veg
k) EC-Earth3-Veg-LR
m) GFDL-ESM4
n) INM-CM4-8
o) INM-CM5-0
p) IPSL-CM6A-LR
q) MIROC-ES2L
r) MIROC6
s) MPI-ESM1-2-LR
t) MRI-ESM2-0
u) NorESM2-LM
v) NorESM2-MM
Heatwave Length (days)
Median Heatwave Area (%)
Cumulative Heat (°C)
300
600
900
1200
1500
2000
SSP1-2.6
Central & Eastern Europe
Median Heatwave Severity

Supplementary Figure 211: Multi-HW-characteristic figure according to SSP1-2.6 CMIP6 multi-model ensemble (MME) per model for Europe (Tx90p) - Each panel shows regional projections according to SSP1-2.6 model runs in the CMIP6 multi-model ensemble (MME) of four combined heatwave characteristics for each projected heatwave in the ensemble between 2020 and 2100. Each dot represents an individual heatwave in the projections, characterized by Median Heatwave Area (%) (x-axis) as latitude-weighted percentage of grid boxes affected by extreme heat, Heatwave Length (days) (y-axis) in days, Median Heatwave Severity (color), and Cumulative Heat (°C) (dot size). The characteristics of the observed event according to the Berkeley Earth observational data (x symbol) and the ERA5 reanalysis data (+ symbol), respectively, are pointed to by an arrow and a figure inset shows a zoom-in on this figure section. The figure inset shows the observed heatwave in the two reference data sets and projections in the indicated section of the respective sub-plot. It facilitates readability of color and size of the reference values and comparison to each other and the neighboring projections. Note that the zoom-in causes the dots to overlap more in the inset than in the main sub-plot. The univariate kernel density estimates along the x- (Median Heatwave Area (%)) and y-axis (Heatwave Length (days)) are on the top and right side of the subplots, respectively, with vertical lines in dark blue (Berkeley Earth) and light blue (ERA5) indicating the observed event. The respective region is indicated above the color bar.

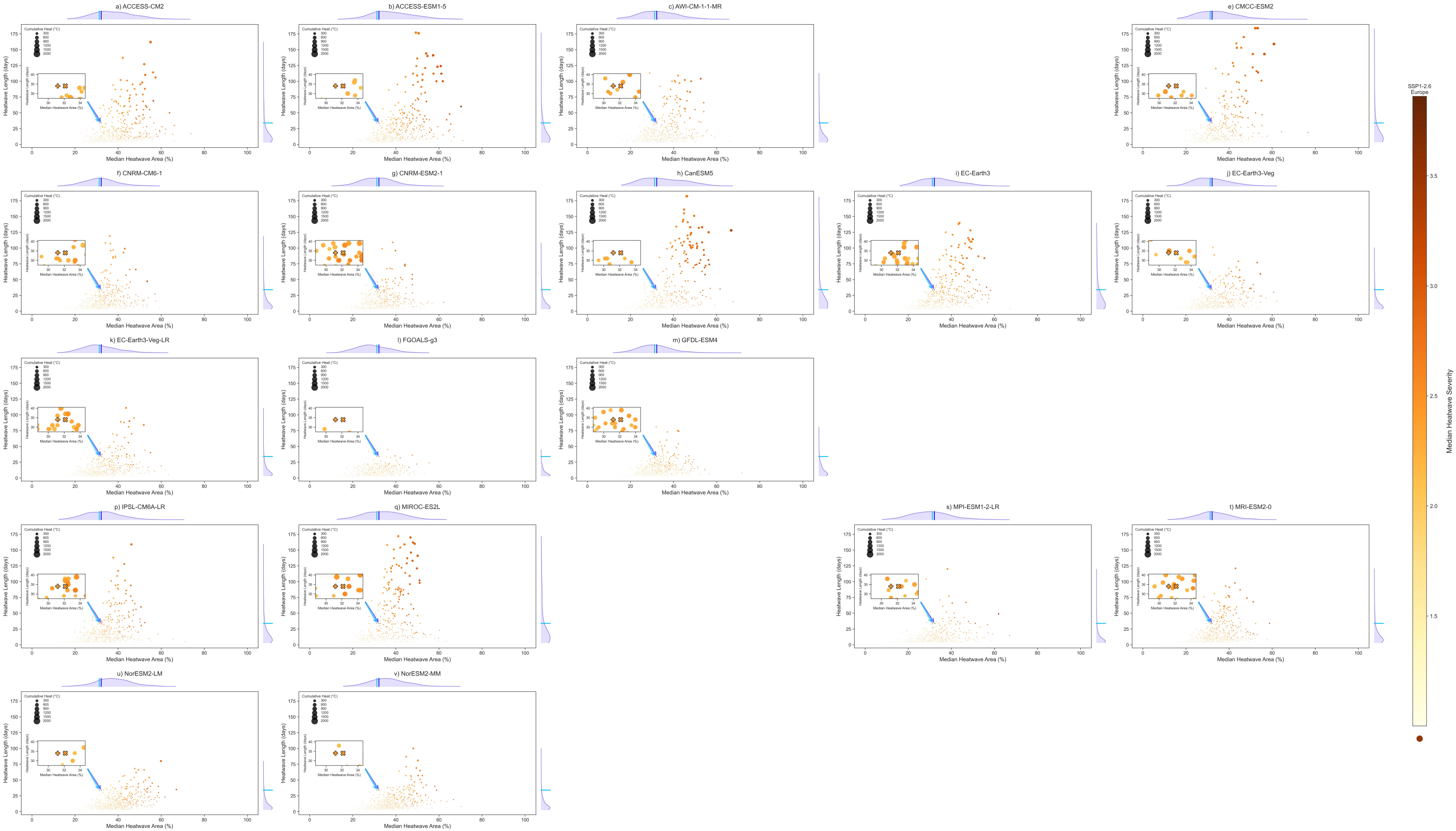
a) ACCESS-CM2
b) ACCESS-ESM1-5
c) AWI-CM-1-1-MR
e) CMCC-ESM2
f) CNRM-CM6-1
g) CNRM-ESM2-1
h) CanESM5
i) EC-Earth3
j) EC-Earth3-Veg
k) EC-Earth3-Veg-LR
l) FGOALS-g3
m) GFDL-ESM4
p) IPSL-CM6A-LR
q) MIROC-ES2L
s) MPI-ESM1-2-LR
t) MRI-ESM2-0
u) NorESM2-LM
v) NorESM2-MM
Cumulative Heat (°C)
300
600
900
1200
1500
2000
Heatwave Length (days)
Median Heatwave Area (%)
SSP1-2.6
Europe
Median Heatwave Severity

Supplementary Figure 212: Multi-HW-characteristic figure according to SSP1-2.6 CMIP6 multi-model ensemble (MME) per model for India & Middle East (Tx90p) - Each panel shows regional projections according to SSP1-2.6 model runs in the CMIP6 multi-model ensemble (MME) of four combined heatwave characteristics for each projected heatwave in the ensemble between 2020 and 2100. Each dot represents an individual heatwave in the projections, characterized by Median Heatwave Area (%) (x-axis) as latitude-weighted percentage of grid boxes affected by extreme heat, Heatwave Length (days) (y-axis) in days, Median Heatwave Severity (color), and Cumulative Heat (°C) (dot size). The characteristics of the observed event according to the Berkeley Earth observational data (x symbol) and the ERA5 reanalysis data (+ symbol), respectively, are pointed to by an arrow and a figure inset shows a zoom-in on this figure section. The figure inset shows the observed heatwave in the two reference data sets and projections in the indicated section of the respective sub-plot. It facilitates readability of color and size of the reference values and comparison to each other and the neighboring projections. Note that the zoom-in causes the dots to overlap more in the inset than in the main sub-plot. The univariate kernel density estimates along the x- (Median Heatwave Area (%)) and y-axis (Heatwave Length (days)) are on the top and right side of the subplots, respectively, with vertical lines in dark blue (Berkeley Earth) and light blue (ERA5) indicating the observed event. The respective region is indicated above the color bar.

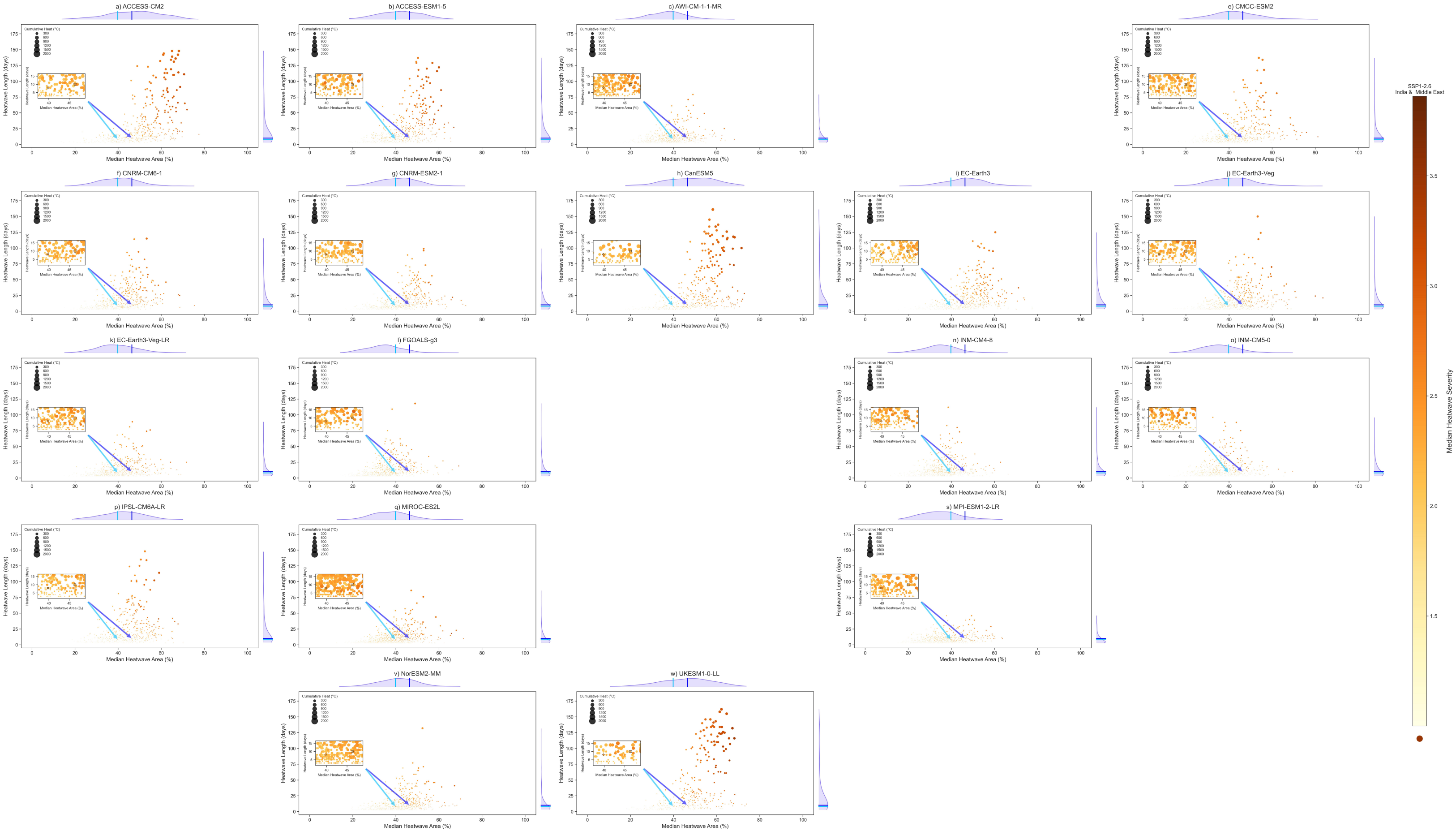

a) ACCESS-CM2
b) ACCESS-ESM1-5
c) AWI-CM-1-1-MR
e) CMCC-ESM2
f) CNRM-CM6-1
g) CNRM-ESM2-1
h) CanESM5
i) EC-Earth3
j) EC-Earth3-Veg
k) EC-Earth3-Veg-LR
l) FGOALS-g3
n) INM-CM4-8
o) INM-CM5-0
p) IPSL-CM6A-LR
q) MIROC-ES2L
s) MPI-ESM1-2-LR
v) NorESM2-MM
w) UKESM1-0-LL
Cumulative Heat (°C)
300
600
900
1200
1500
2000
Heatwave Length (days)
Median Heatwave Area (%)
SSP1-2.6
India & Middle East
Median Heatwave Severity

Supplementary Figure 213: Multi-HW-characteristic figure according to SSP1-2.6 CMIP6 multi-model ensemble (MME) per model for India & Pakistan (Tx90p) - Each panel shows regional projections according to SSP1-2.6 model runs in the CMIP6 multi-model ensemble (MME) of four combined heatwave characteristics for each projected heatwave in the ensemble between 2020 and 2100. Each dot represents an individual heatwave in the projections, characterized by Median Heatwave Area (%) (x-axis) as latitude-weighted percentage of grid boxes affected by extreme heat, Heatwave Length (days) (y-axis) in days, Median Heatwave Severity (color), and Cumulative Heat (°C) (dot size). The characteristics of the observed event according to the Berkeley Earth observational data (x symbol) and the ERA5 reanalysis data (+ symbol), respectively, are pointed to by an arrow and a figure inset shows a zoom-in on this figure section. The figure inset shows the observed heatwave in the two reference data sets and projections in the indicated section of the respective sub-plot. It facilitates readability of color and size of the reference values and comparison to each other and the neighboring projections. Note that the zoom-in causes the dots to overlap more in the inset than in the main sub-plot. The univariate kernel density estimates along the x-(Median Heatwave Area (%)) and y-axis (Heatwave Length (days)) are on the top and right side of the subplots, respectively, with vertical lines in dark blue (Berkeley Earth) and light blue (ERA5) indicating the observed event. The respective region is indicated above the color bar.

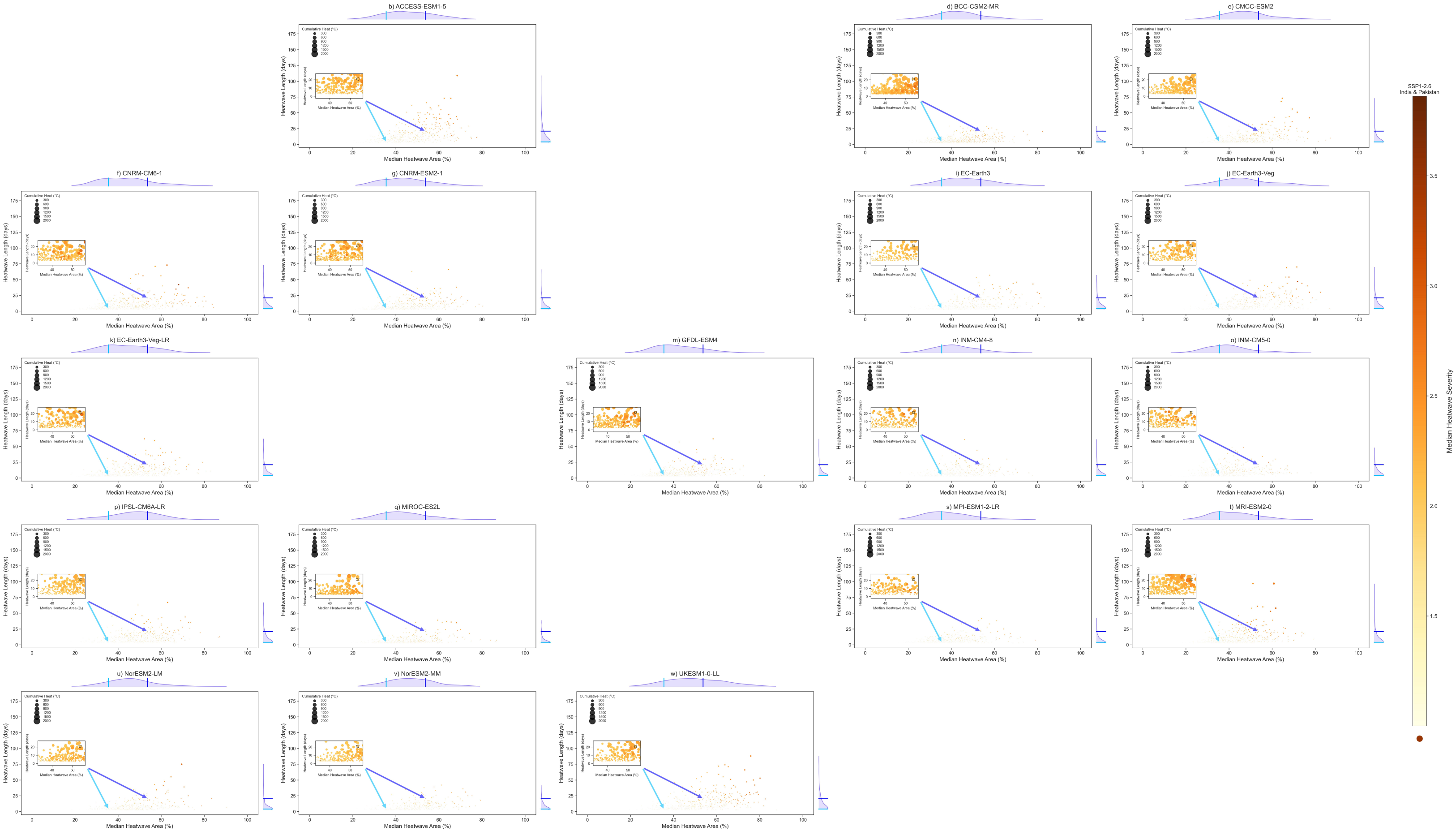
b) ACCESS-ESM1-5
d) BCC-CSM2-MR
e) CMCC-ESM2
f) CNRM-CM6-1
g) CNRM-ESM2-1
i) EC-Earth3
j) EC-Earth3-Veg
k) EC-Earth3-Veg-LR
m) GFDL-ESM4
n) INM-CM4-8
o) INM-CM5-0
p) IPSL-CM6A-LR
q) MIROC-ES2L
s) MPI-ESM1-2-LR
t) MRI-ESM2-0
u) NorESM2-LM
v) NorESM2-MM
w) UKESM1-0-LL
Cumulative Heat (°C)
300
600
900
1200
1500
2000
Heatwave Length (days)
Median Heatwave Area (%)
SSP1-2.6
India & Pakistan
Median Heatwave Severity

Supplementary Figure 214: Multi-HW-characteristic figure according to SSP1-2.6 CMIP6 multi-model ensemble (MME) per model for Middle East (Tx90p) - Each panel shows regional projections according to SSP1-2.6 model runs in the CMIP6 multi-model ensemble (MME) of four combined heatwave characteristics for each projected heatwave in the ensemble between 2020 and 2100. Each dot represents an individual heatwave in the projections, characterized by Median Heatwave Area (%) (x-axis) as latitude-weighted percentage of grid boxes affected by extreme heat, Heatwave Length (days) (y-axis) in days, Median Heatwave Severity (color), and Cumulative Heat (°C) (dot size). The characteristics of the observed event according to the Berkeley Earth observational data (x symbol) and the ERA5 reanalysis data (+ symbol), respectively, are pointed to by an arrow and a figure inset shows a zoom-in on this figure section. The figure inset shows the observed heatwave in the two reference data sets and projections in the indicated section of the respective sub-plot. It facilitates readability of color and size of the reference values and comparison to each other and the neighboring projections. Note that the zoom-in causes the dots to overlap more in the inset than in the main sub-plot. The univariate kernel density estimates along the x- (Median Heatwave Area (%)) and y-axis (Heatwave Length (days)) are on the top and right side of the subplots, respectively, with vertical lines in dark blue (Berkeley Earth) and light blue (ERA5) indicating the observed event. The respective region is indicated above the color bar.

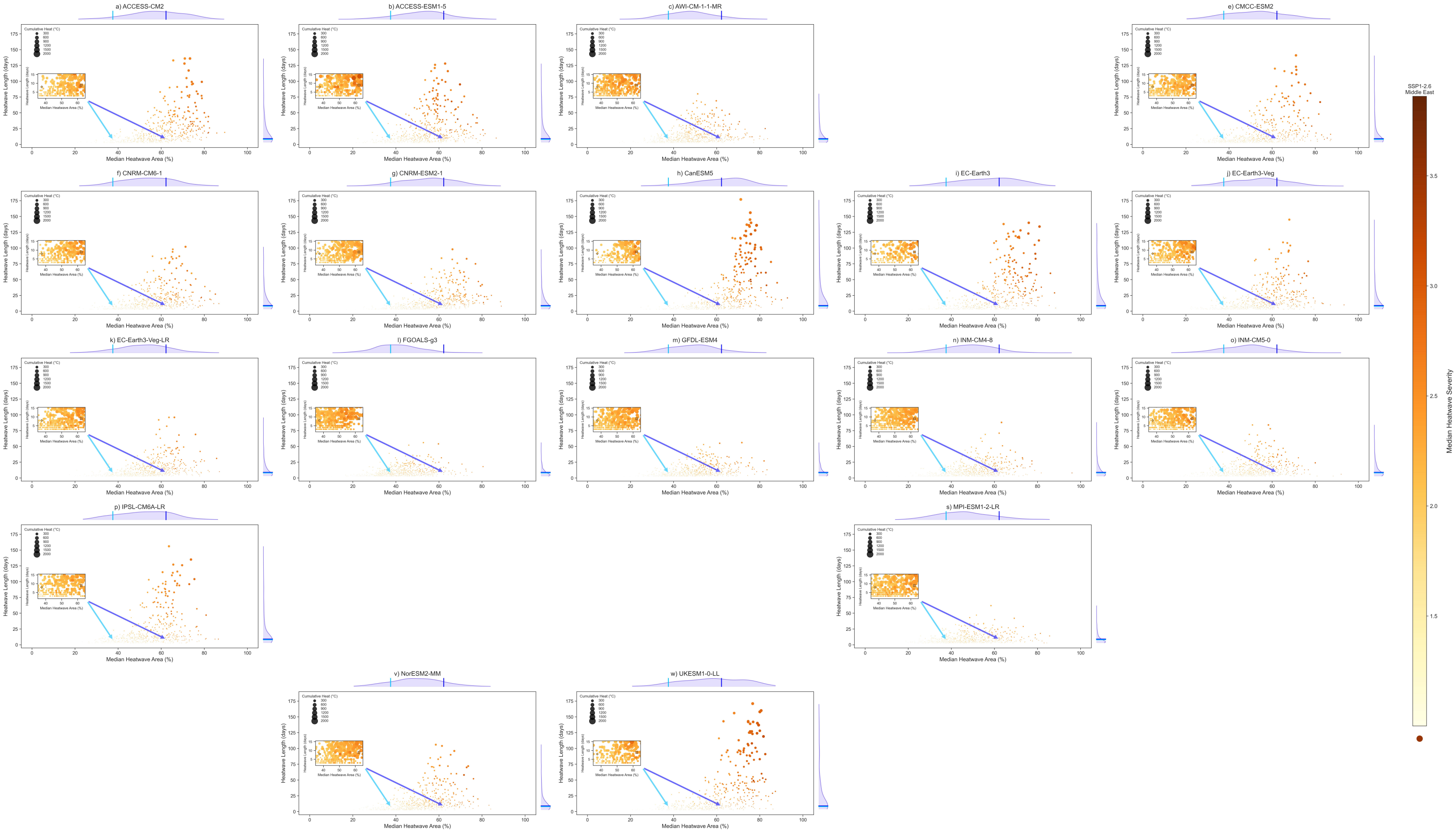
a) ACCESS-CM2
b) ACCESS-ESM1-5
c) AWI-CM-1-1-MR
e) CMCC-ESM2
f) CNRM-CM6-1
g) CNRM-ESM2-1
h) CanESM5
i) EC-Earth3
j) EC-Earth3-Veg
k) EC-Earth3-Veg-LR
l) FGOALS-g3
m) GFDL-ESM4
n) INM-CM4-8
o) INM-CM5-0
p) IPSL-CM6A-LR
s) MPI-ESM1-2-LR
v) NorESM2-MM
w) UKESM1-0-LL
Cumulative Heat (°C)
300
600
900
1200
1500
2000
Heatwave Length (days)
Median Heatwave Area (%)
SSP1-2.6
Middle East
Median Heatwave Severity

Supplementary Figure 215: Multi-HW-characteristic figure according to SSP1-2.6 CMIP6 multi-model ensemble (MME) per model for Northeast Siberia (Tx90p) - Each panel shows regional projections according to SSP1-2.6 model runs in the CMIP6 multi-model ensemble (MME) of four combined heatwave characteristics for each projected heatwave in the ensemble between 2020 and 2100. Each dot represents an individual heatwave in the projections, characterized by Median Heatwave Area (%) (x-axis) as latitude-weighted percentage of grid boxes affected by extreme heat, Heatwave Length (days) (y-axis) in days, Median Heatwave Severity (color), and Cumulative Heat (°C) (dot size). The characteristics of the observed event according to the Berkeley Earth observational data (x symbol) and the ERA5 reanalysis data (+ symbol), respectively, are pointed to by an arrow and a figure inset shows a zoom-in on this figure section. The figure inset shows the observed heatwave in the two reference data sets and projections in the indicated section of the respective sub-plot. It facilitates readability of color and size of the reference values and comparison to each other and the neighboring projections. Note that the zoom-in causes the dots to overlap more in the inset than in the main sub-plot. The univariate kernel density estimates along the x- (Median Heatwave Area (%)) and y-axis (Heatwave Length (days)) are on the top and right side of the subplots, respectively, with vertical lines in dark blue (Berkeley Earth) and light blue (ERA5) indicating the observed event. The respective region is indicated above the color bar.

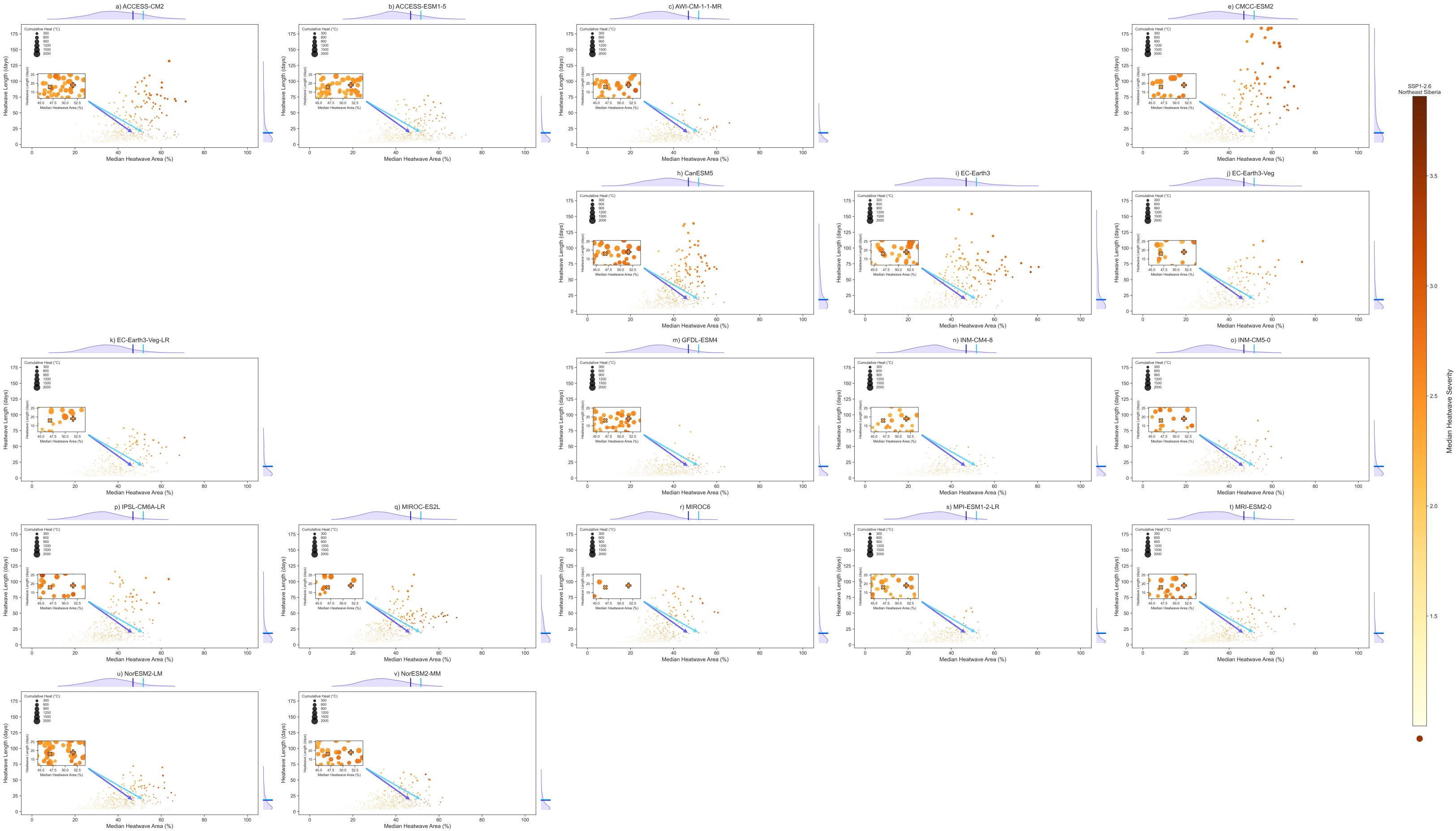
a) ACCESS-CM2
b) ACCESS-ESM1-5
c) AWI-CM-1-1-MR
e) CMCC-ESM2
h) CanESM5
i) EC-Earth3
j) EC-Earth3-Veg
k) EC-Earth3-Veg-LR
m) GFDL-ESM4
n) INM-CM4-8
o) INM-CM5-0
p) IPSL-CM6A-LR
q) MIROC-ES2L
r) MIROC6
s) MPI-ESM1-2-LR
t) MRI-ESM2-0
u) NorESM2-LM
v) NorESM2-MM
Heatwave Length (days)
Median Heatwave Area (%)
Cumulative Heat (°C)
300
600
900
1200
1500
2000
SSP1-2.6
Northeast Siberia
Median Heatwave Severity
1.5
2.0
2.5
3.0
3.5

Supplementary Figure 216: Multi-HW-characteristic figure according to SSP1-2.6 CMIP6 multi-model ensemble (MME) per model for Northern Africa & Middle East (Tx90p) - Each panel shows regional projections according to SSP1-2.6 model runs in the CMIP6 multi-model ensemble (MME) of four combined heatwave characteristics for each projected heatwave in the ensemble between 2020 and 2100. Each dot represents an individual heatwave in the projections, characterized by Median Heatwave Area (%) (x-axis) as latitude-weighted percentage of grid boxes affected by extreme heat, Heatwave Length (days) (y-axis) in days, Median Heatwave Severity (color), and Cumulative Heat (°C) (dot size). The characteristics of the observed event according to the Berkeley Earth observational data (x symbol) and the ERA5 reanalysis data (+ symbol), respectively, are pointed to by an arrow and a figure inset shows a zoom-in on this figure section. The figure inset shows the observed heatwave in the two reference data sets and projections in the indicated section of the respective sub-plot. It facilitates readability of color and size of the reference values and comparison to each other and the neighboring projections. Note that the zoom-in causes the dots to overlap more in the inset than in the main sub-plot. The univariate kernel density estimates along the x- (Median Heatwave Area (%)) and y-axis (Heatwave Length (days)) are on the top and right side of the subplots, respectively, with vertical lines in dark blue (Berkeley Earth) and light blue (ERA5) indicating the observed event. The respective region is indicated above the color bar.

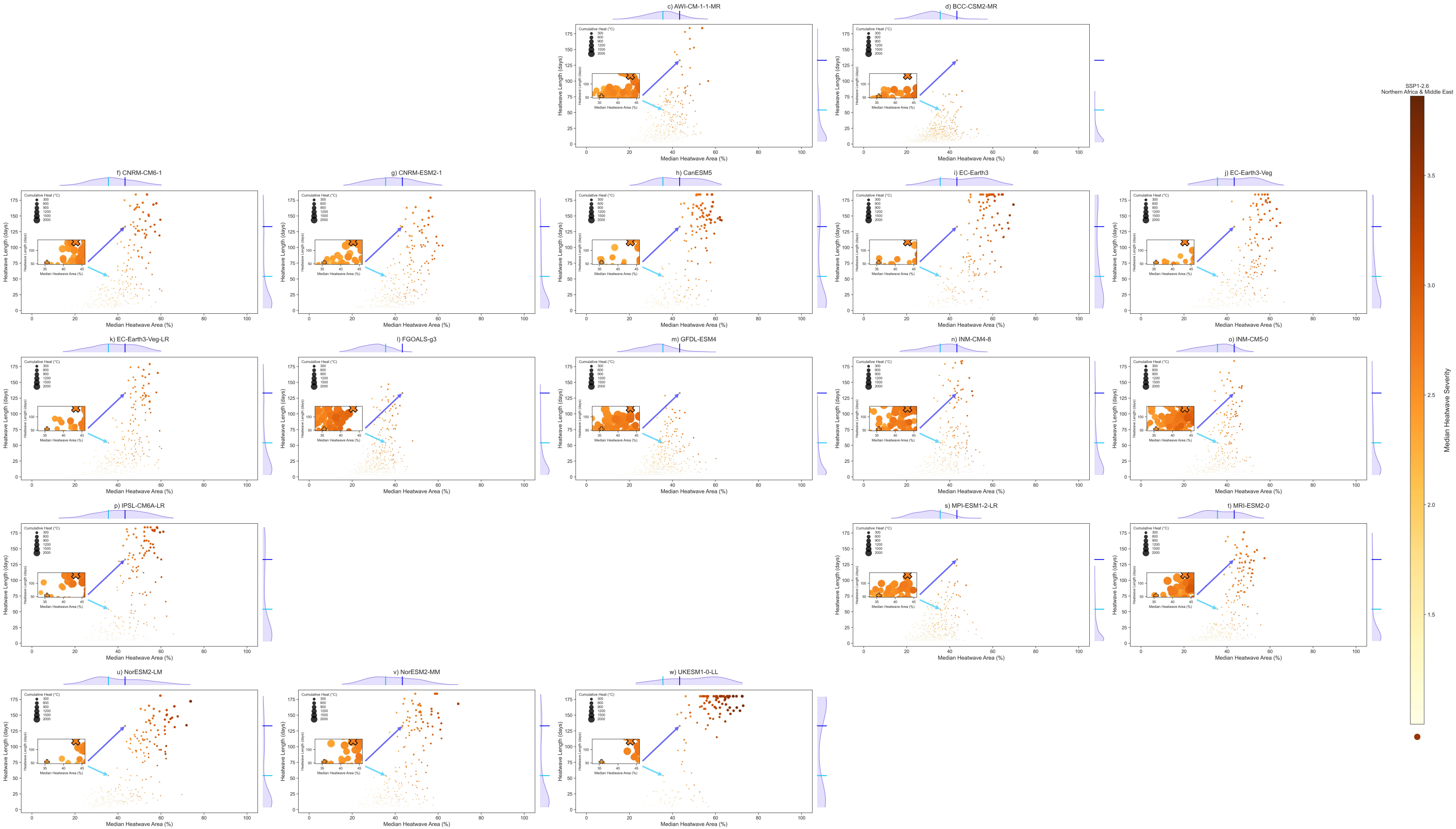
c) AWI-CM-1-1-MR
d) BCC-CSM2-MR
f) CNRM-CM6-1
g) CNRM-ESM2-1
h) CanESM5
i) EC-Earth3
j) EC-Earth3-Veg
k) EC-Earth3-Veg-LR
l) FGOALS-g3
m) GFDL-ESM4
n) INM-CM4-8
o) INM-CM5-0
p) IPSL-CM6A-LR
s) MPI-ESM1-2-LR
t) MRI-ESM2-0
u) NorESM2-LM
v) NorESM2-MM
w) UKESM1-0-LL
Heatwave Length (days)
Median Heatwave Area (%)
Cumulative Heat (°C)
SSP1-2.6
Northern Africa & Middle East
Median Heatwave Severity

Supplementary Figure 217: Multi-HW-characteristic figure according to SSP1-2.6 CMIP6 multi-model ensemble (MME) per model for Pacific Northwest USA & Canada (Tx90p) - Each panel shows regional projections according to SSP1-2.6 model runs in the CMIP6 multi-model ensemble (MME) of four combined heatwave characteristics for each projected heatwave in the ensemble between 2020 and 2100. Each dot represents an individual heatwave in the projections, characterized by Median Heatwave Area (%) (x-axis) as latitude-weighted percentage of grid boxes affected by extreme heat, Heatwave Length (days) (y-axis) in days, Median Heatwave Severity (color), and Cumulative Heat (°C) (dot size). The characteristics of the observed event according to the Berkeley Earth observational data (x symbol) and the ERA5 reanalysis data (+ symbol), respectively, are pointed to by an arrow and a figure inset shows a zoom-in on this figure section. The figure inset shows the observed heatwave in the two reference data sets and projections in the indicated section of the respective sub-plot. It facilitates readability of color and size of the reference values and comparison to each other and the neighboring projections. Note that the zoom-in causes the dots to overlap more in the inset than in the main sub-plot. The univariate kernel density estimates along the x- (Median Heatwave Area (%)) and y-axis (Heatwave Length (days)) are on the top and right side of the subplots, respectively, with vertical lines in dark blue (Berkeley Earth) and light blue (ERA5) indicating the observed event. The respective region is indicated above the color bar.

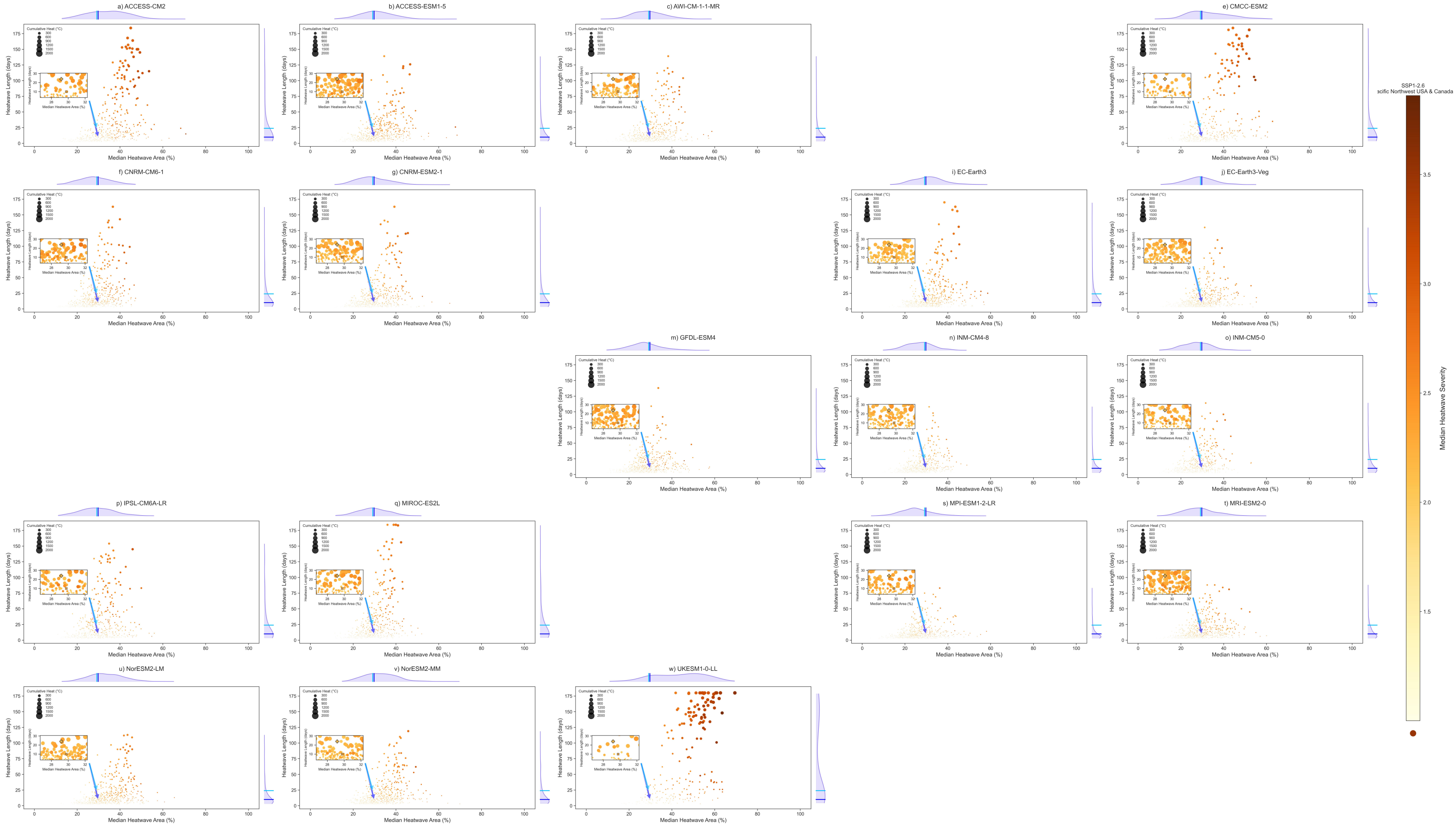

a) ACCESS-CM2
b) ACCESS-ESM1-5
c) AWI-CM-1-1-MR
e) CMCC-ESM2
f) CNRM-CM6-1
g) CNRM-ESM2-1
i) EC-Earth3
j) EC-Earth3-Veg
m) GFDL-ESM4
n) INM-CM4-8
o) INM-CM5-0
p) IPSL-CM6A-LR
q) MIROC-ES2L
s) MPI-ESM1-2-LR
t) MRI-ESM2-0
u) NorESM2-LM
v) NorESM2-MM
w) UKESM1-0-LL
Cumulative Heat (°C)
300
600
900
1200
1500
2000
Heatwave Length (days)
Median Heatwave Area (%)
SSP1-2.6
acific Northwest USA & Canada
Median Heatwave Severity

Supplementary Figure 218: Multi-HW-characteristic figure according to SSP1-2.6 CMIP6 multi-model ensemble (MME) per model for Russia (Tx90p) - Each panel shows regional projections according to SSP1-2.6 model runs in the CMIP6 multi-model ensemble (MME) of four combined heatwave characteristics for each projected heatwave in the ensemble between 2020 and 2100. Each dot represents an individual heatwave in the projections, characterized by Median Heatwave Area (%) (x-axis) as latitude-weighted percentage of grid boxes affected by extreme heat, Heatwave Length (days) (y-axis) in days, Median Heatwave Severity (color), and Cumulative Heat (°C) (dot size). The characteristics of the observed event according to the Berkeley Earth observational data (x symbol) and the ERA5 reanalysis data (+ symbol), respectively, are pointed to by an arrow and a figure inset shows a zoom-in on this figure section. The figure inset shows the observed heatwave in the two reference data sets and projections in the indicated section of the respective sub-plot. It facilitates readability of color and size of the reference values and comparison to each other and the neighboring projections. Note that the zoom-in causes the dots to overlap more in the inset than in the main sub-plot. The univariate kernel density estimates along the x- (Median Heatwave Area (%)) and y-axis (Heatwave Length (days)) are on the top and right side of the subplots, respectively, with vertical lines in dark blue (Berkeley Earth) and light blue (ERA5) indicating the observed event. The respective region is indicated above the color bar.

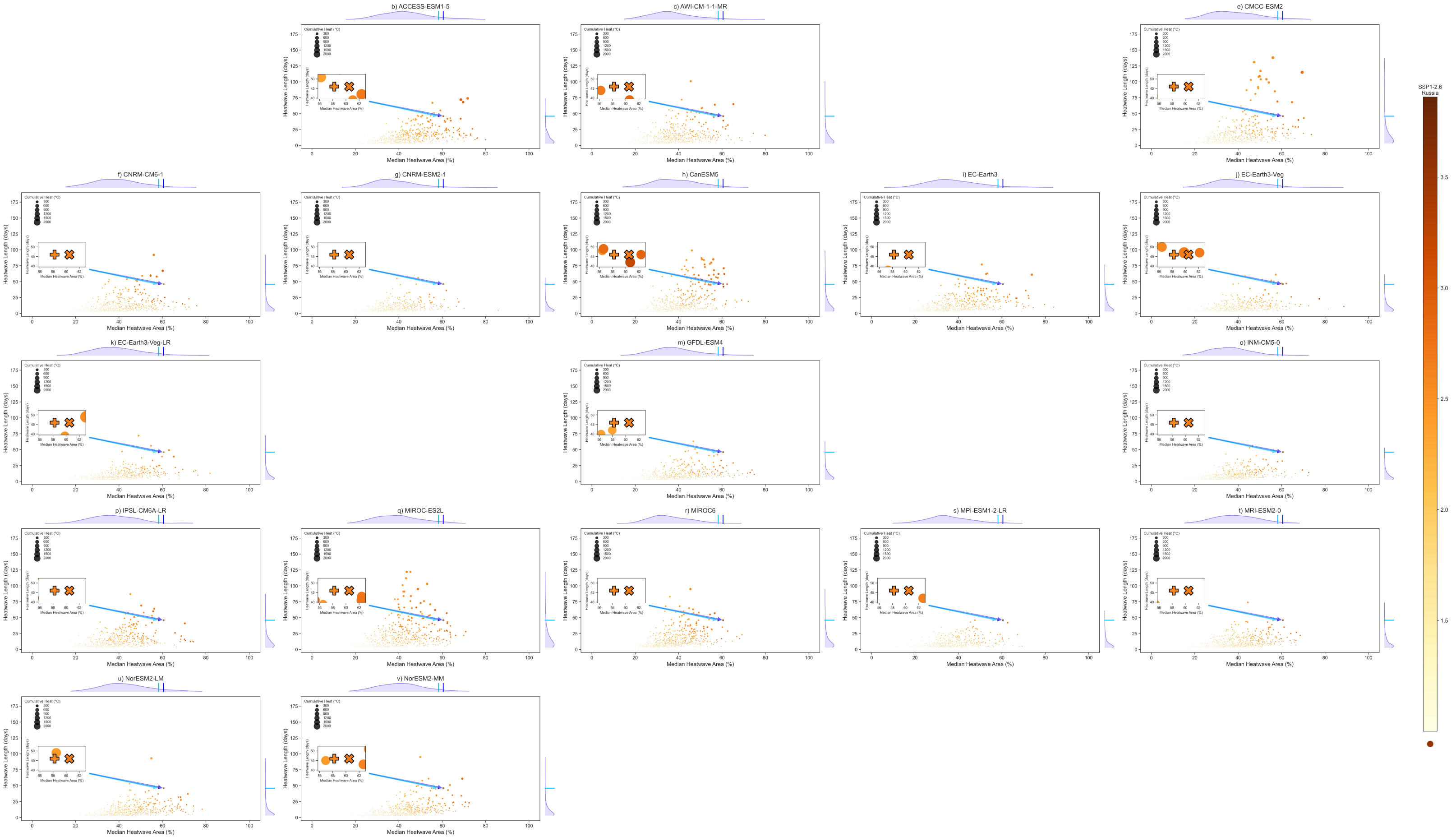

b) ACCESS-ESM1-5
c) AWI-CM-1-1-MR
e) CMCC-ESM2
f) CNRM-CM6-1
g) CNRM-ESM2-1
h) CanESM5
i) EC-Earth3
j) EC-Earth3-Veg
k) EC-Earth3-Veg-LR
m) GFDL-ESM4
o) INM-CM5-0
p) IPSL-CM6A-LR
q) MIROC-ES2L
r) MIROC6
s) MPI-ESM1-2-LR
t) MRI-ESM2-0
u) NorESM2-LM
v) NorESM2-MM
Heatwave Length (days)
Median Heatwave Area (%)
Cumulative Heat (°C)
SSP1-2.6
Russia
Median Heatwave Severity

Supplementary Figure 219: Multi-HW-characteristic figure according to SSP1-2.6 CMIP6 multi-model ensemble (MME) per model for Southwestern USA & Mexico (Tx90p) - Each panel shows regional projections according to SSP1-2.6 model runs in the CMIP6 multi-model ensemble (MME) of four combined heatwave characteristics for each projected heatwave in the ensemble between 2020 and 2100. Each dot represents an individual heatwave in the projections, characterized by Median Heatwave Area (%) (x-axis) as latitude-weighted percentage of grid boxes affected by extreme heat, Heatwave Length (days) (y-axis) in days, Median Heatwave Severity (color), and Cumulative Heat (°C) (dot size). The characteristics of the observed event according to the Berkeley Earth observational data (x symbol) and the ERA5 reanalysis data (+ symbol), respectively, are pointed to by an arrow and a figure inset shows a zoom-in on this figure section. The figure inset shows the observed heatwave in the two reference data sets and projections in the indicated section of the respective sub-plot. It facilitates readability of color and size of the reference values and comparison to each other and the neighboring projections. Note that the zoom-in causes the dots to overlap more in the inset than in the main sub-plot. The univariate kernel density estimates along the x- (Median Heatwave Area (%)) and y-axis (Heatwave Length (days)) are on the top and right side of the subplots, respectively, with vertical lines in dark blue (Berkeley Earth) and light blue (ERA5) indicating the observed event. The respective region is indicated above the color bar.

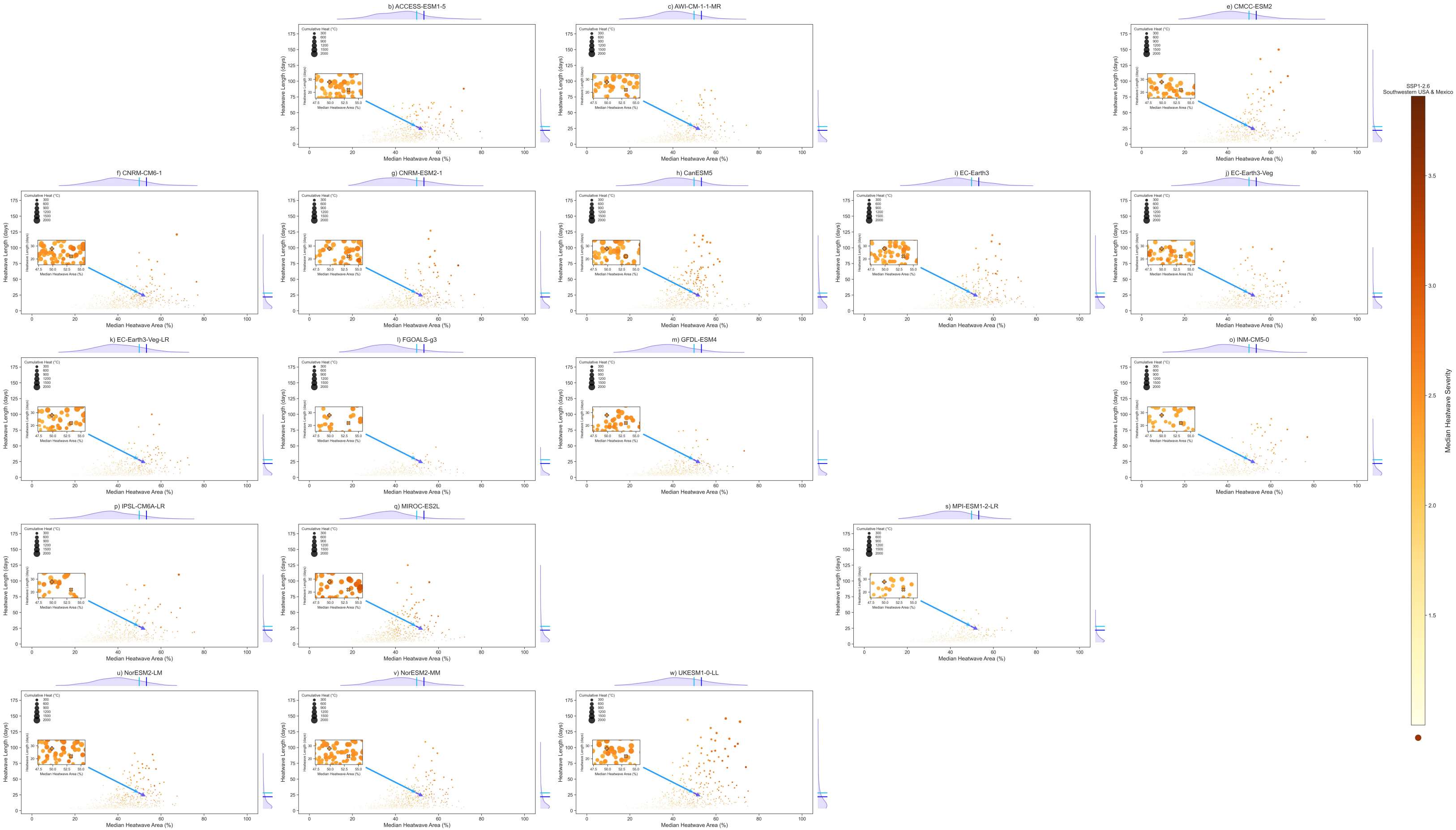

b) ACCESS-ESM1-5
c) AWI-CM-1-1-MR
e) CMCC-ESM2
f) CNRM-CM6-1
g) CNRM-ESM2-1
h) CanESM5
i) EC-Earth3
j) EC-Earth3-Veg
k) EC-Earth3-Veg-LR
l) FGOALS-g3
m) GFDL-ESM4
o) INM-CM5-0
p) IPSL-CM6A-LR
q) MIROC-ES2L
s) MPI-ESM1-2-LR
u) NorESM2-LM
v) NorESM2-MM
w) UKESM1-0-LL
Cumulative Heat (°C)
300
600
900
1200
1500
2000
Heatwave Length (days)
Median Heatwave Area (%)
SSP1-2.6
Southwestern USA & Mexico
Median Heatwave Severity

Supplementary Figure 220: Multi-HW-characteristic figure according to SSP1-2.6 CMIP6 multi-model ensemble (MME) per model for Siberia (Tx90p) - Each panel shows regional projections according to SSP1-2.6 model runs in the CMIP6 multi-model ensemble (MME) of four combined heatwave characteristics for each projected heatwave in the ensemble between 2020 and 2100. Each dot represents an individual heatwave in the projections, characterized by Median Heatwave Area (%) (x-axis) as latitude-weighted percentage of grid boxes affected by extreme heat, Heatwave Length (days) (y-axis) in days, Median Heatwave Severity (color), and Cumulative Heat (°C) (dot size). The characteristics of the observed event according to the Berkeley Earth observational data (x symbol) and the ERA5 reanalysis data (+ symbol), respectively, are pointed to by an arrow and a figure inset shows a zoom-in on this figure section. The figure inset shows the observed heatwave in the two reference data sets and projections in the indicated section of the respective sub-plot. It facilitates readability of color and size of the reference values and comparison to each other and the neighboring projections. Note that the zoom-in causes the dots to overlap more in the inset than in the main sub-plot. The univariate kernel density estimates along the x- (Median Heatwave Area (%)) and y-axis (Heatwave Length (days)) are on the top and right side of the subplots, respectively, with vertical lines in dark blue (Berkeley Earth) and light blue (ERA5) indicating the observed event. The respective region is indicated above the color bar.

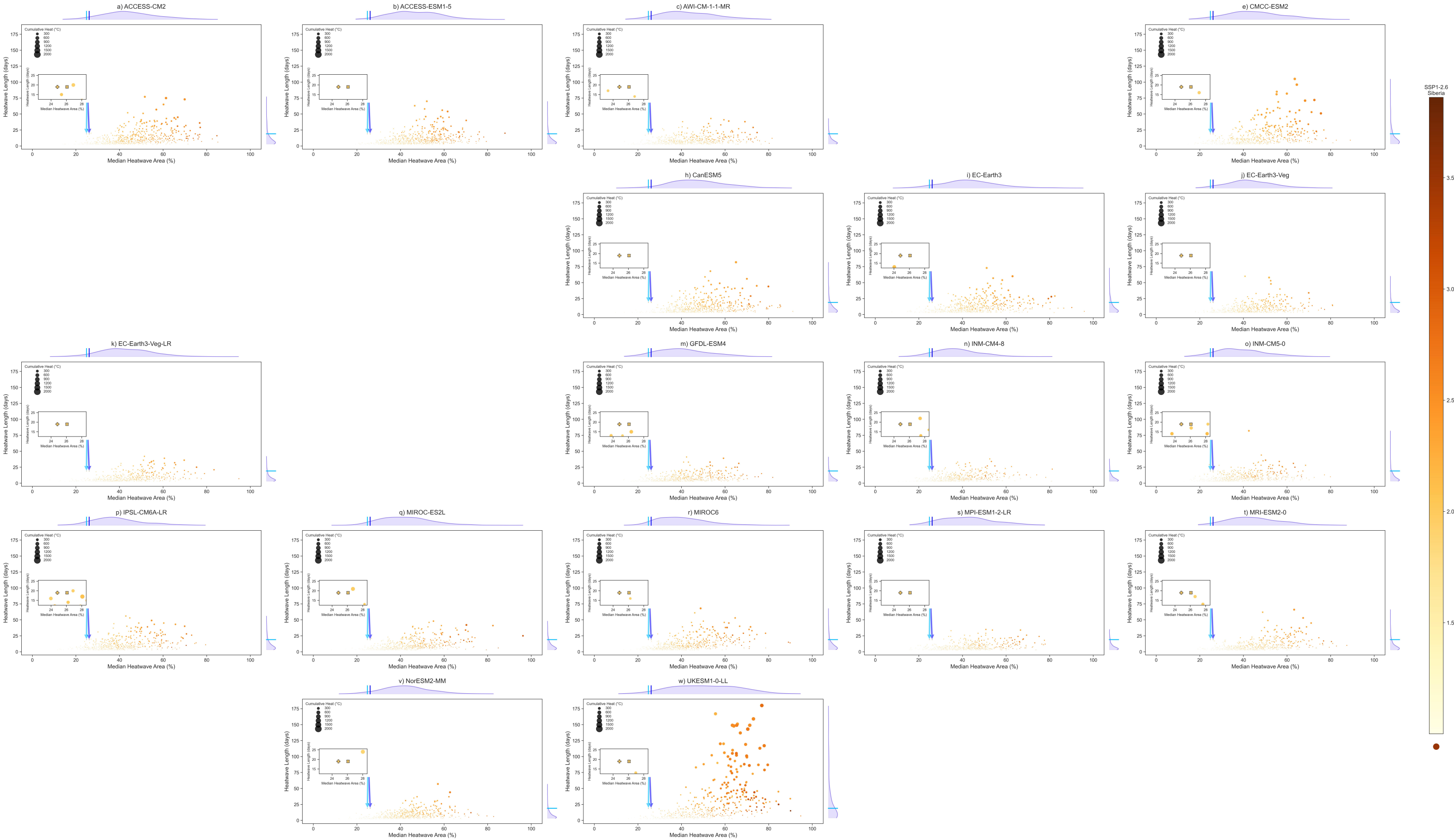

a) ACCESS-CM2
b) ACCESS-ESM1-5
c) AWI-CM-1-1-MR
e) CMCC-ESM2
h) CanESM5
i) EC-Earth3
j) EC-Earth3-Veg
k) EC-Earth3-Veg-LR
m) GFDL-ESM4
n) INM-CM4-8
o) INM-CM5-0
p) IPSL-CM6A-LR
q) MIROC-ES2L
r) MIROC6
s) MPI-ESM1-2-LR
t) MRI-ESM2-0
v) NorESM2-MM
w) UKESM1-0-LL
Cumulative Heat (°C)
300
600
900
1200
1500
2000
Heatwave Length (days)
Median Heatwave Area (%)
SSP1-2.6
Siberia
Median Heatwave Severity

Supplementary Figure 221: Multi-HW-characteristic figure according to SSP1-2.6 CMIP6 multi-model ensemble (MME) per model for South America (Tx90p) - Each panel shows regional projections according to SSP1-2.6 model runs in the CMIP6 multi-model ensemble (MME) of four combined heatwave characteristics for each projected heatwave in the ensemble between 2020 and 2100. Each dot represents an individual heatwave in the projections, characterized by Median Heatwave Area (%) (x-axis) as latitude-weighted percentage of grid boxes affected by extreme heat, Heatwave Length (days) (y-axis) in days, Median Heatwave Severity (color), and Cumulative Heat (°C) (dot size). The characteristics of the observed event according to the Berkeley Earth observational data (x symbol) and the ERA5 reanalysis data (+ symbol), respectively, are pointed to by an arrow and a figure inset shows a zoom-in on this figure section. The figure inset shows the observed heatwave in the two reference data sets and projections in the indicated section of the respective sub-plot. It facilitates readability of color and size of the reference values and comparison to each other and the neighboring projections. Note that the zoom-in causes the dots to overlap more in the inset than in the main sub-plot. The univariate kernel density estimates along the x- (Median Heatwave Area (%)) and y-axis (Heatwave Length (days)) are on the top and right side of the subplots, respectively, with vertical lines in dark blue (Berkeley Earth) and light blue (ERA5) indicating the observed event. The respective region is indicated above the color bar.

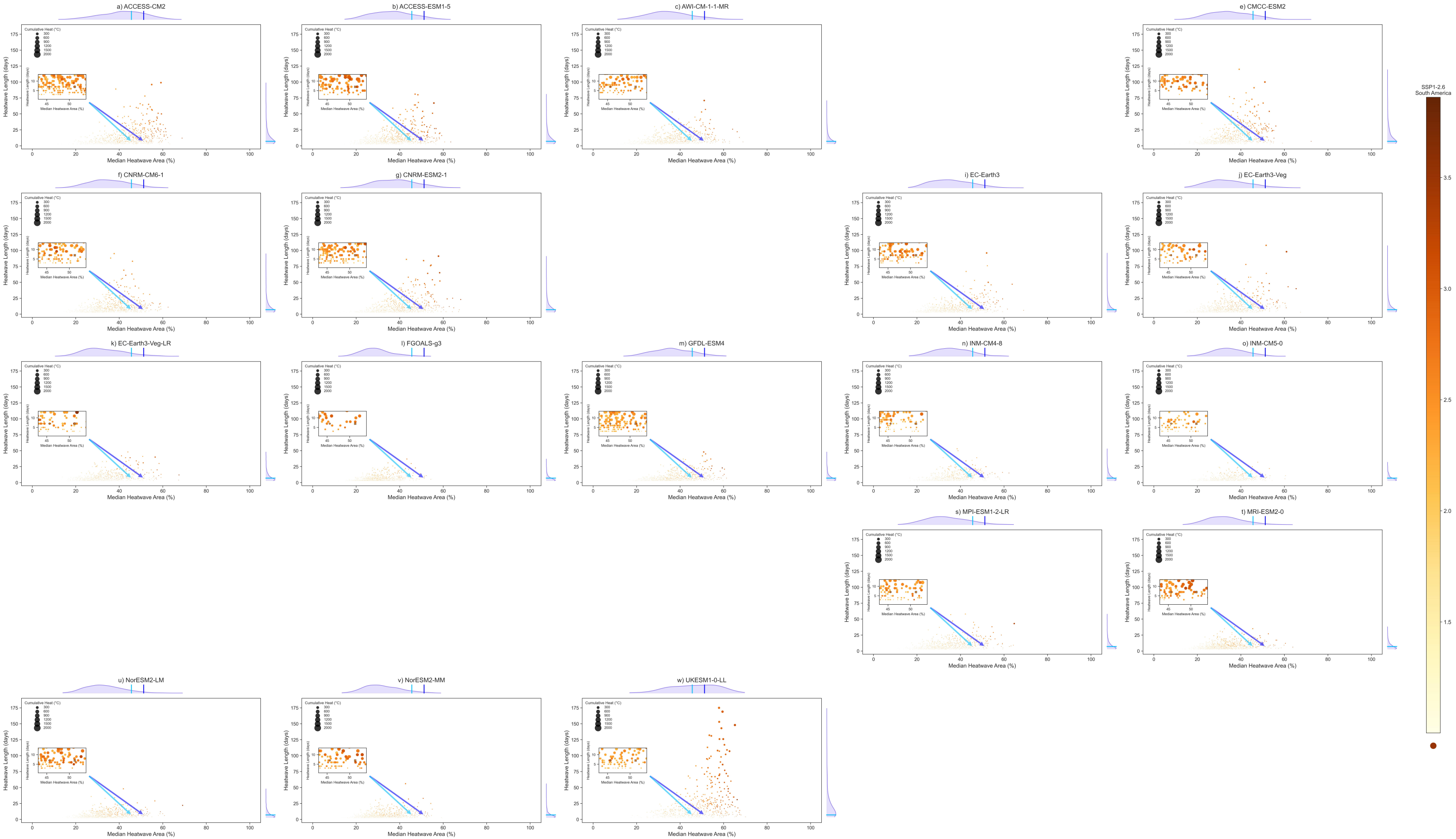
a) ACCESS-CM2
b) ACCESS-ESM1-5
c) AWI-CM-1-1-MR
e) CMCC-ESM2
f) CNRM-CM6-1
g) CNRM-ESM2-1
i) EC-Earth3
j) EC-Earth3-Veg
k) EC-Earth3-Veg-LR
l) FGOALS-g3
m) GFDL-ESM4
n) INM-CM4-8
o) INM-CM5-0
s) MPI-ESM1-2-LR
t) MRI-ESM2-0
u) NorESM2-LM
v) NorESM2-MM
w) UKESM1-0-LL
Cumulative Heat (°C)
300
600
900
1200
1500
2000
Heatwave Length (days)
Median Heatwave Area (%)
SSP1-2.6
South America
Median Heatwave Severity

Supplementary Figure 222: Multi-HW-characteristic figure according to SSP1-2.6 CMIP6 multi-model ensemble (MME) per model for Northern Southeast Asia (Tx90p) - Each panel shows regional projections according to SSP1-2.6 model runs in the CMIP6 multi-model ensemble (MME) of four combined heatwave characteristics for each projected heatwave in the ensemble between 2020 and 2100. Each dot represents an individual heatwave in the projections, characterized by Median Heatwave Area (%) (x-axis) as latitude-weighted percentage of grid boxes affected by extreme heat, Heatwave Length (days) (y-axis) in days, Median Heatwave Severity (color), and Cumulative Heat (°C) (dot size). The characteristics of the observed event according to the Berkeley Earth observational data (x symbol) and the ERA5 reanalysis data (+ symbol), respectively, are pointed to by an arrow and a figure inset shows a zoom-in on this figure section. The figure inset shows the observed heatwave in the two reference data sets and projections in the indicated section of the respective sub-plot. It facilitates readability of color and size of the reference values and comparison to each other and the neighboring projections. Note that the zoom-in causes the dots to overlap more in the inset than in the main sub-plot. The univariate kernel density estimates along the x- (Median Heatwave Area (%)) and y-axis (Heatwave Length (days)) are on the top and right side of the subplots, respectively, with vertical lines in dark blue (Berkeley Earth) and light blue (ERA5) indicating the observed event. The respective region is indicated above the color bar.

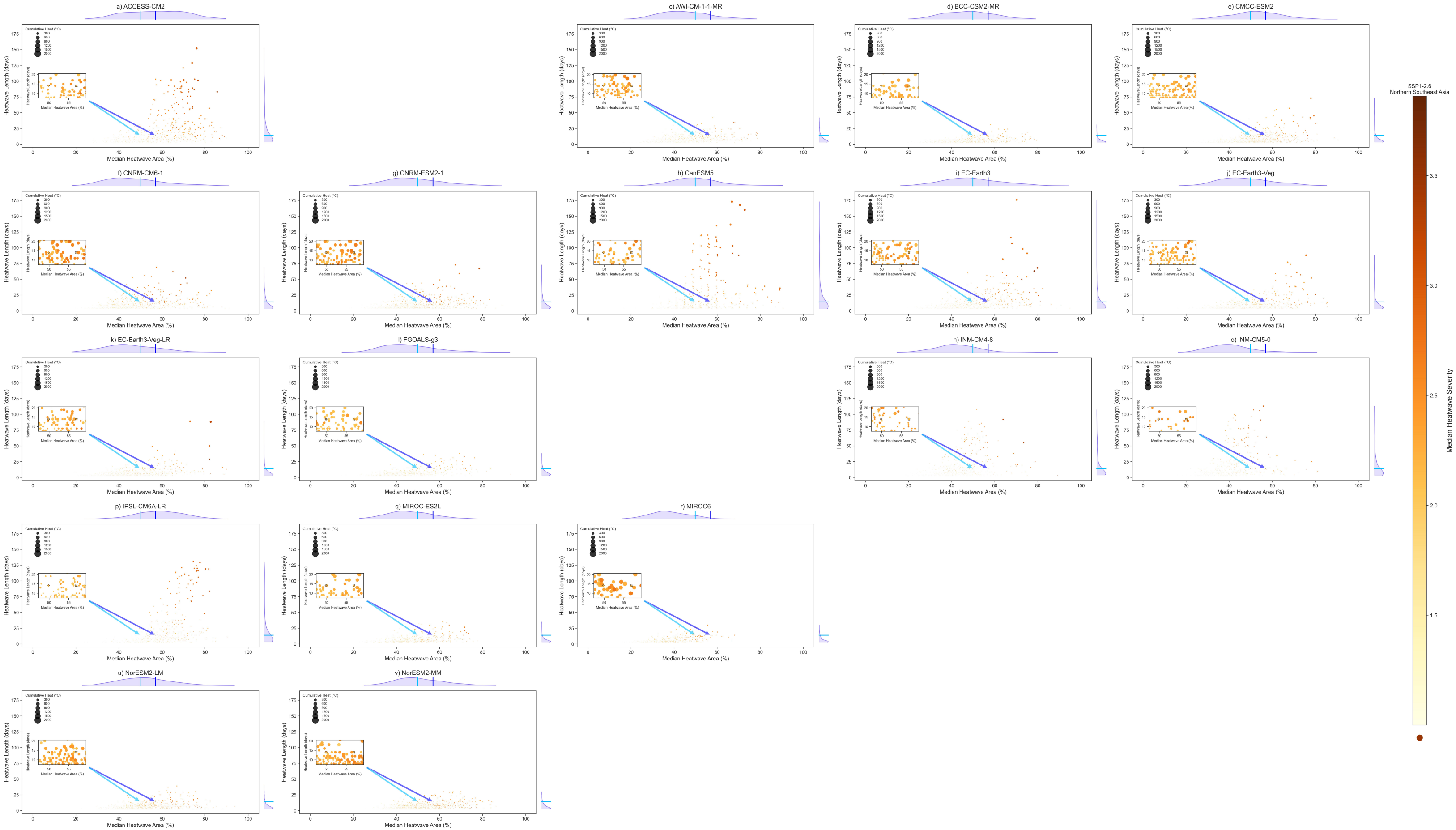

a) ACCESS-CM2
c) AWI-CM-1-1-MR
d) BCC-CSM2-MR
e) CMCC-ESM2
f) CNRM-CM6-1
g) CNRM-ESM2-1
h) CanESM5
i) EC-Earth3
j) EC-Earth3-Veg
k) EC-Earth3-Veg-LR
l) FGOALS-g3
n) INM-CM4-8
o) INM-CM5-0
p) IPSL-CM6A-LR
q) MIROC-ES2L
r) MIROC6
u) NorESM2-LM
v) NorESM2-MM
Heatwave Length (days)
Median Heatwave Area (%)
Cumulative Heat (°C)
300
600
900
1200
1500
2000
SSP1-2.6
Northern Southeast Asia
Median Heatwave Severity

Supplementary Figure 223: Multi-HW-characteristic figure according to SSP1-2.6 CMIP6 multi-model ensemble (MME) per model for Southern Africa (Tx90p) - Each panel shows regional projections according to SSP1-2.6 model runs in the CMIP6 multi-model ensemble (MME) of four combined heatwave characteristics for each projected heatwave in the ensemble between 2020 and 2100. Each dot represents an individual heatwave in the projections, characterized by Median Heatwave Area (%) (x-axis) as latitude-weighted percentage of grid boxes affected by extreme heat, Heatwave Length (days) (y-axis) in days, Median Heatwave Severity (color), and Cumulative Heat (°C) (dot size). The characteristics of the observed event according to the Berkeley Earth observational data (x symbol) and the ERA5 reanalysis data (+ symbol), respectively, are pointed to by an arrow and a figure inset shows a zoom-in on this figure section. The figure inset shows the observed heatwave in the two reference data sets and projections in the indicated section of the respective sub-plot. It facilitates readability of color and size of the reference values and comparison to each other and the neighboring projections. Note that the zoom-in causes the dots to overlap more in the inset than in the main sub-plot. The univariate kernel density estimates along the x- (Median Heatwave Area (%)) and y-axis (Heatwave Length (days)) are on the top and right side of the subplots, respectively, with vertical lines in dark blue (Berkeley Earth) and light blue (ERA5) indicating the observed event. The respective region is indicated above the color bar.

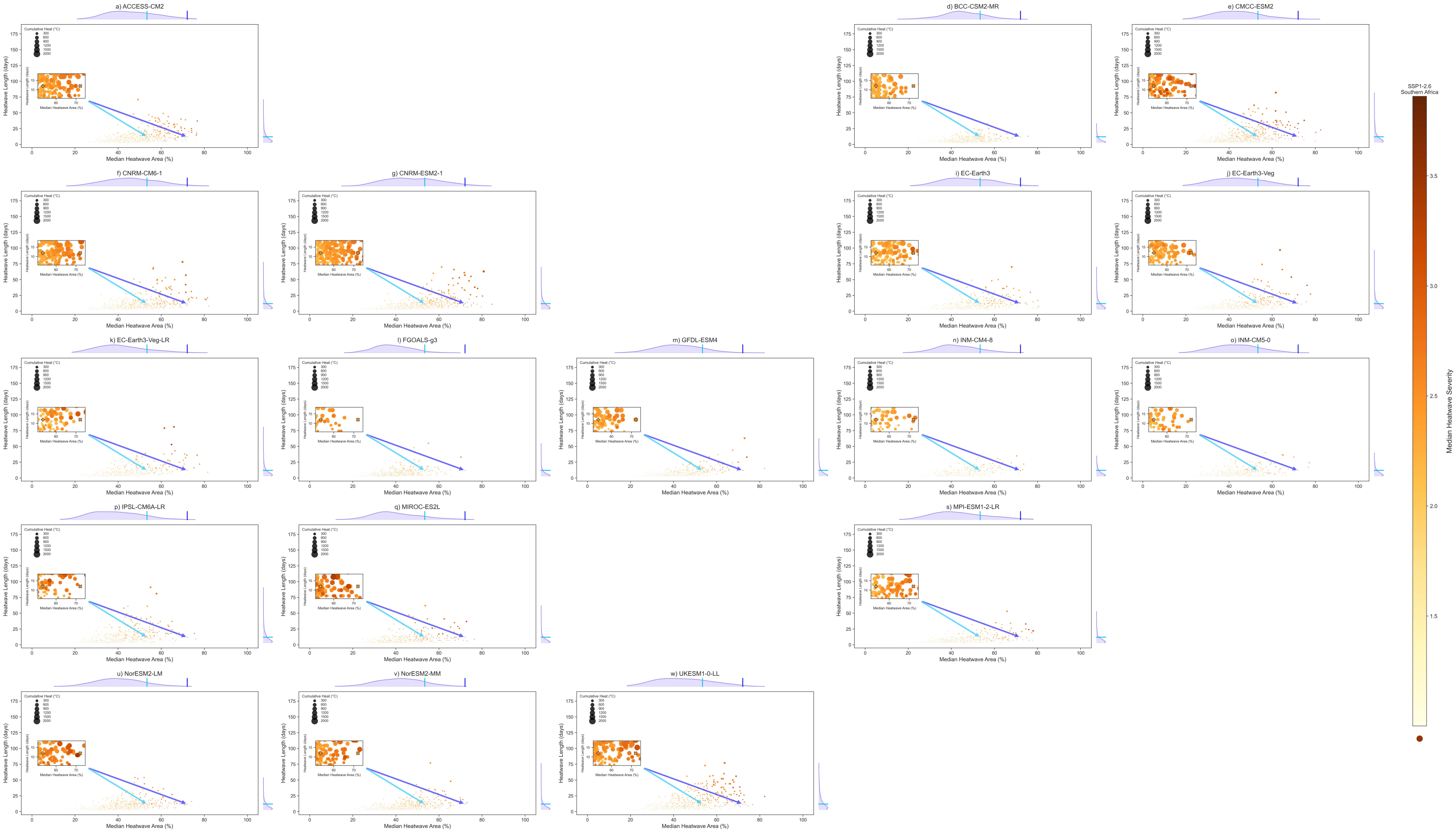

a) ACCESS-CM2
d) BCC-CSM2-MR
e) CMCC-ESM2
f) CNRM-CM6-1
g) CNRM-ESM2-1
i) EC-Earth3
j) EC-Earth3-Veg
k) EC-Earth3-Veg-LR
l) FGOALS-g3
m) GFDL-ESM4
n) INM-CM4-8
o) INM-CM5-0
p) IPSL-CM6A-LR
q) MIROC-ES2L
s) MPI-ESM1-2-LR
u) NorESM2-LM
v) NorESM2-MM
w) UKESM1-0-LL
Cumulative Heat (°C)
300
600
900
1200
1500
2000
Heatwave Length (days)
Median Heatwave Area (%)
SSP1-2.6
Southern Africa
Median Heatwave Severity

Supplementary Figure 224: Multi-HW-characteristic figure according to SSP1-2.6 CMIP6 multi-model ensemble (MME) per model for Eastern USA (Tx90p) - Each panel shows regional projections according to SSP1-2.6 model runs in the CMIP6 multi-model ensemble (MME) of four combined heatwave characteristics for each projected heatwave in the ensemble between 2020 and 2100. Each dot represents an individual heatwave in the projections, characterized by Median Heatwave Area (%) (x-axis) as latitude-weighted percentage of grid boxes affected by extreme heat, Heatwave Length (days) (y-axis) in days, Median Heatwave Severity (color), and Cumulative Heat (°C) (dot size). The characteristics of the observed event according to the Berkeley Earth observational data (x symbol) and the ERA5 reanalysis data (+ symbol), respectively, are pointed to by an arrow and a figure inset shows a zoom-in on this figure section. The figure inset shows the observed heatwave in the two reference data sets and projections in the indicated section of the respective sub-plot. It facilitates readability of color and size of the reference values and comparison to each other and the neighboring projections. Note that the zoom-in causes the dots to overlap more in the inset than in the main sub-plot. The univariate kernel density estimates along the x- (Median Heatwave Area (%)) and y-axis (Heatwave Length (days)) are on the top and right side of the subplots, respectively, with vertical lines in dark blue (Berkeley Earth) and light blue (ERA5) indicating the observed event. The respective region is indicated above the color bar.

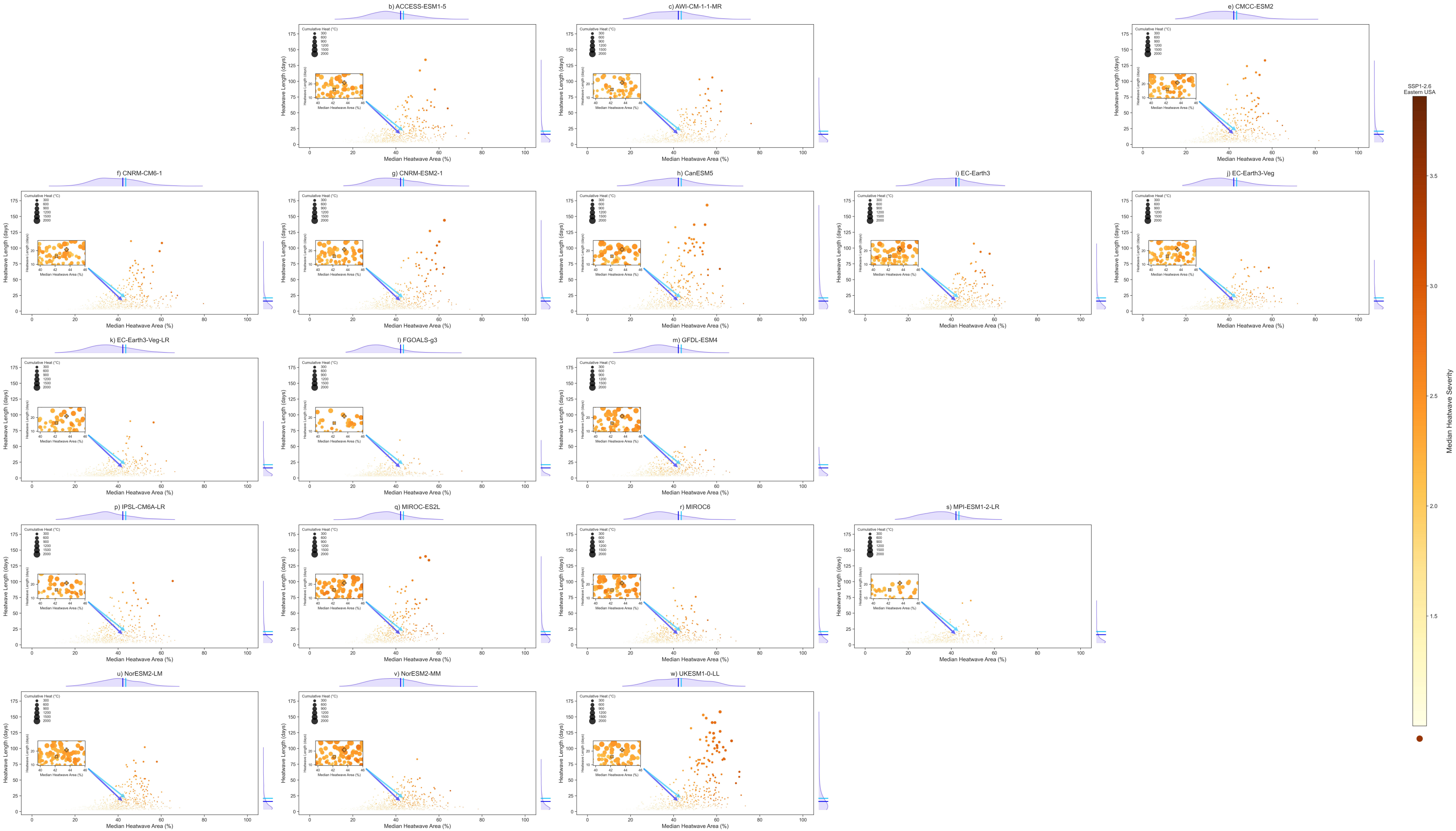

b) ACCESS-ESM1-5
c) AWI-CM-1-1-MR
e) CMCC-ESM2
f) CNRM-CM6-1
g) CNRM-ESM2-1
h) CanESM5
i) EC-Earth3
j) EC-Earth3-Veg
k) EC-Earth3-Veg-LR
l) FGOALS-g3
m) GFDL-ESM4
p) IPSL-CM6A-LR
q) MIROC-ES2L
r) MIROC6
s) MPI-ESM1-2-LR
u) NorESM2-LM
v) NorESM2-MM
w) UKESM1-0-LL
Cumulative Heat (°C)
300
600
900
1200
1500
2000
Heatwave Length (days)
Median Heatwave Area (%)
SSP1-2.6
Eastern USA
Median Heatwave Severity

Supplementary Figure 225: Multi-HW-characteristic figure according to SSP2-4.5 CMIP6 multi-model ensemble (MME) per model for Alaska & Canada (Tx90p) - Each panel shows regional projections according to SSP2-4.5 model runs in the CMIP6 multi-model ensemble (MME) of four combined heatwave characteristics for each projected heatwave in the ensemble between 2020 and 2100. Each dot represents an individual heatwave in the projections, characterized by Median Heatwave Area (%) (x-axis) as latitude-weighted percentage of grid boxes affected by extreme heat, Heatwave Length (days) (y-axis) in days, Median Heatwave Severity (color), and Cumulative Heat (°C) (dot size). The characteristics of the observed event according to the Berkeley Earth observational data (x symbol) and the ERA5 reanalysis data (+ symbol), respectively, are pointed to by an arrow and a figure inset shows a zoom-in on this figure section. The figure inset shows the observed heatwave in the two reference data sets and projections in the indicated section of the respective sub-plot. It facilitates readability of color and size of the reference values and comparison to each other and the neighboring projections. Note that the zoom-in causes the dots to overlap more in the inset than in the main sub-plot. The univariate kernel density estimates along the x- (Median Heatwave Area (%)) and y-axis (Heatwave Length (days)) are on the top and right side of the subplots, respectively, with vertical lines in dark blue (Berkeley Earth) and light blue (ERA5) indicating the observed event. The respective region is indicated above the color bar.

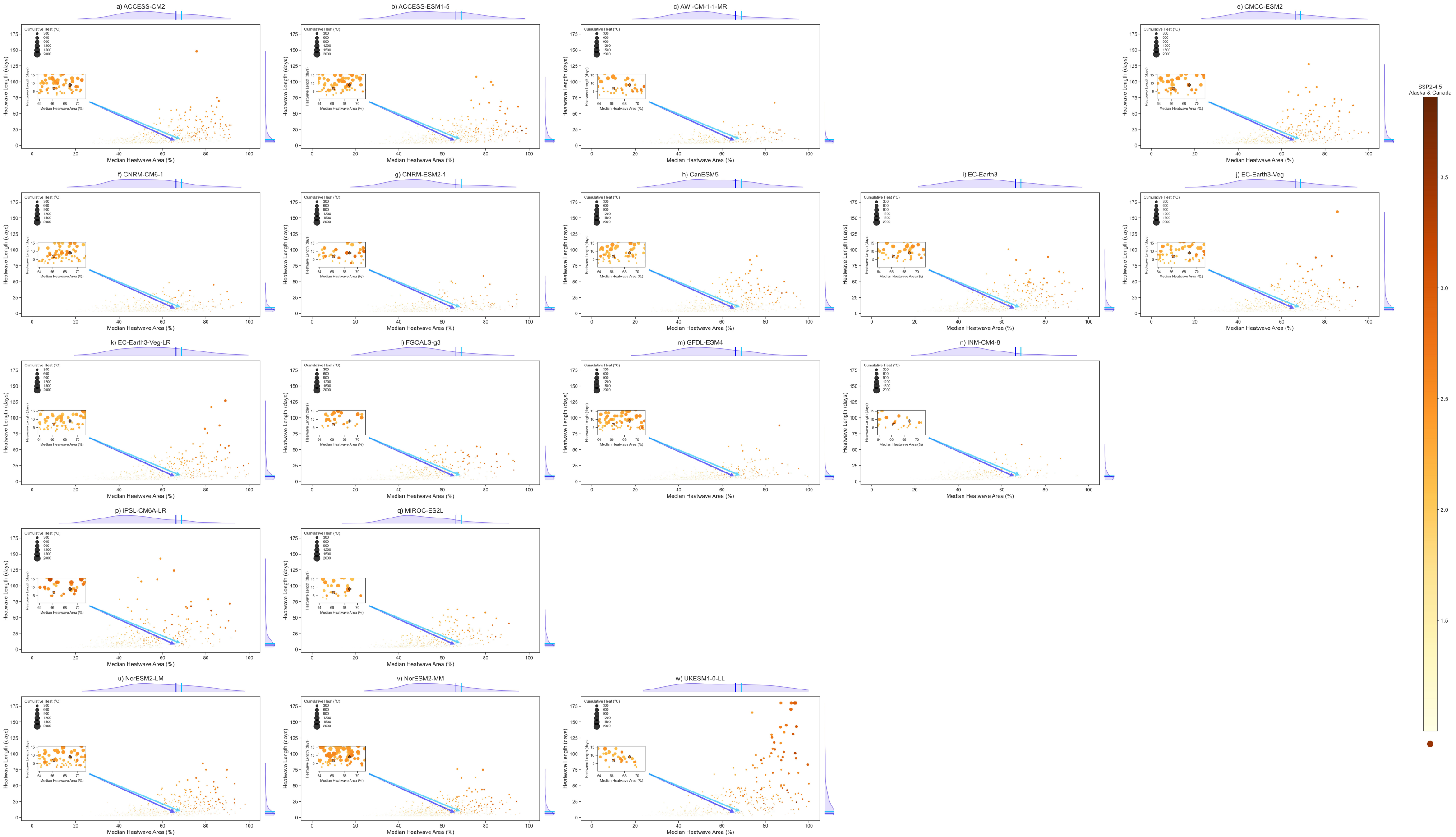

a) ACCESS-CM2
b) ACCESS-ESM1-5
c) AWI-CM-1-1-MR
e) CMCC-ESM2
f) CNRM-CM6-1
g) CNRM-ESM2-1
h) CanESM5
i) EC-Earth3
j) EC-Earth3-Veg
k) EC-Earth3-Veg-LR
l) FGOALS-g3
m) GFDL-ESM4
n) INM-CM4-8
p) IPSL-CM6A-LR
q) MIROC-ES2L
u) NorESM2-LM
v) NorESM2-MM
w) UKESM1-0-LL
Cumulative Heat (°C)
300
600
900
1200
1500
2000
Heatwave Length (days)
Median Heatwave Area (%)
SSP2-4.5
Alaska & Canada
Median Heatwave Severity

Supplementary Figure 226: Multi-HW-characteristic figure according to SSP2-4.5 CMIP6 multi-model ensemble (MME) per model for Argentina, Bolivia, Chile, Paraguay & Uruguay (Tx90p) - Each panel shows regional projections according to SSP2-4.5 model runs in the CMIP6 multi-model ensemble (MME) of four combined heatwave characteristics for each projected heatwave in the ensemble between 2020 and 2100. Each dot represents an individual heatwave in the projections, characterized by Median Heatwave Area (%) (x-axis) as latitude-weighted percentage of grid boxes affected by extreme heat, Heatwave Length (days) (y-axis) in days, Median Heatwave Severity (color), and Cumulative Heat (°C) (dot size). The characteristics of the observed event according to the Berkeley Earth observational data (x symbol) and the ERA5 reanalysis data (+ symbol), respectively, are pointed to by an arrow and a figure inset shows a zoom-in on this figure section. The figure inset shows the observed heatwave in the two reference data sets and projections in the indicated section of the respective sub-plot. It facilitates readability of color and size of the reference values and comparison to each other and the neighboring projections. Note that the zoom-in causes the dots to overlap more in the inset than in the main sub-plot. The univariate kernel density estimates along the x- (Median Heatwave Area (%)) and y-axis (Heatwave Length (days)) are on the top and right side of the subplots, respectively, with vertical lines in dark blue (Berkeley Earth) and light blue (ERA5) indicating the observed event. The respective region is indicated above the color bar.

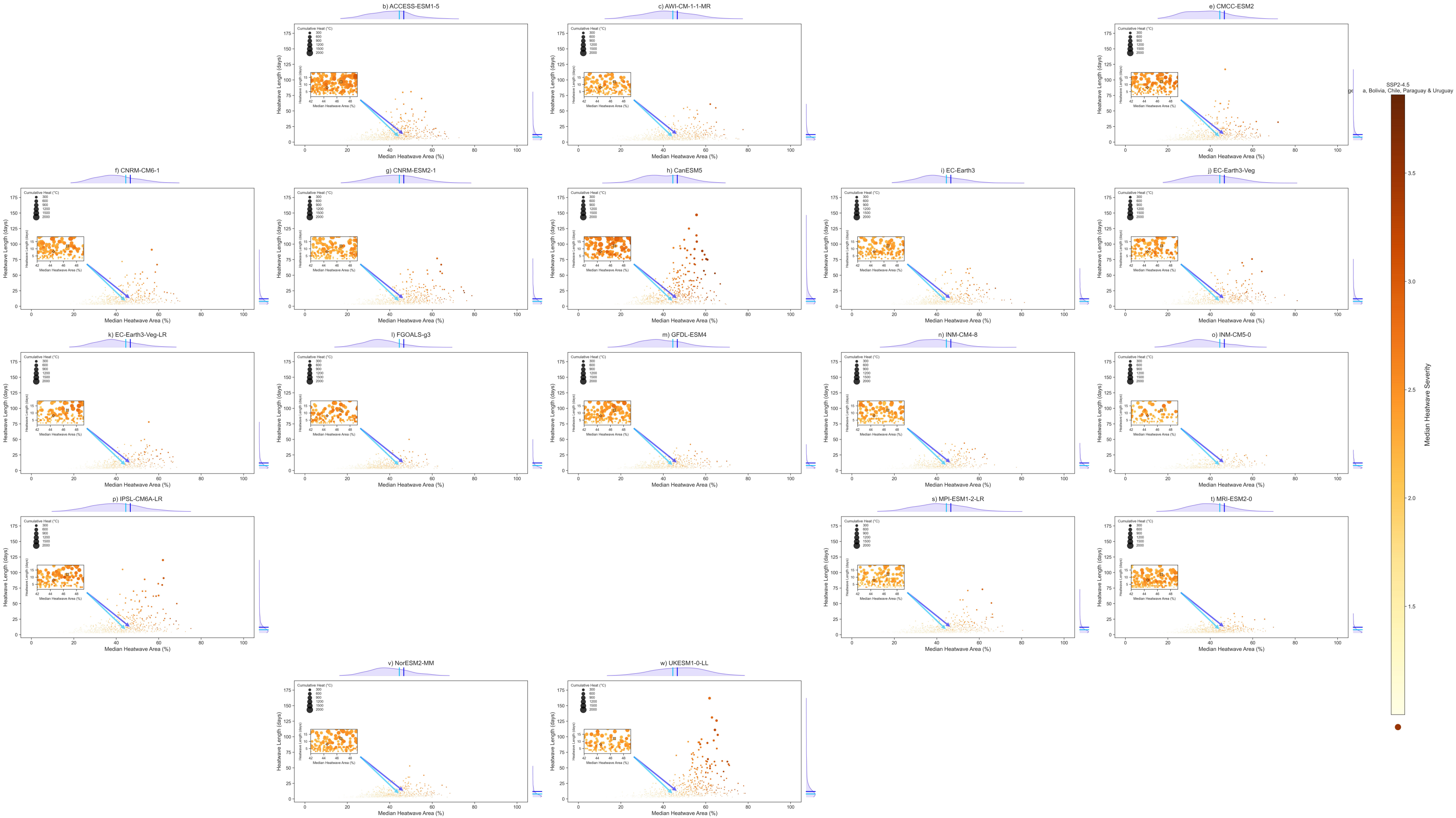
b) ACCESS-ESM1-5
c) AWI-CM-1-1-MR
e) CMCC-ESM2
f) CNRM-CM6-1
g) CNRM-ESM2-1
h) CanESM5
i) EC-Earth3
j) EC-Earth3-Veg
k) EC-Earth3-Veg-LR
l) FGOALS-g3
m) GFDL-ESM4
n) INM-CM4-8
o) INM-CM5-0
p) IPSL-CM6A-LR
s) MPI-ESM1-2-LR
t) MRI-ESM2-0
v) NorESM2-MM
w) UKESM1-0-LL
Cumulative Heat (°C)
300
600
900
1200
1500
2000
Heatwave Length (days)
Median Heatwave Area (%)
SSP2-4.5
a, Bolivia, Chile, Paraguay & Uruguay
Median Heatwave Severity

Supplementary Figure 227: Multi-HW-characteristic figure according to SSP2-4.5 CMIP6 multi-model ensemble (MME) per model for Argentina, Uruguay & Brazil (Tx90p) - Each panel shows regional projections according to SSP2-4.5 model runs in the CMIP6 multi-model ensemble (MME) of four combined heatwave characteristics for each projected heatwave in the ensemble between 2020 and 2100. Each dot represents an individual heatwave in the projections, characterized by Median Heatwave Area (%) (x-axis) as latitude-weighted percentage of grid boxes affected by extreme heat, Heatwave Length (days) (y-axis) in days, Median Heatwave Severity (color), and Cumulative Heat (°C) (dot size). The characteristics of the observed event according to the Berkeley Earth observational data (x symbol) and the ERA5 reanalysis data (+ symbol), respectively, are pointed to by an arrow and a figure inset shows a zoom-in on this figure section. The figure inset shows the observed heatwave in the two reference data sets and projections in the indicated section of the respective sub-plot. It facilitates readability of color and size of the reference values and comparison to each other and the neighboring projections. Note that the zoom-in causes the dots to overlap more in the inset than in the main sub-plot. The univariate kernel density estimates along the x- (Median Heatwave Area (%)) and y-axis (Heatwave Length (days)) are on the top and right side of the subplots, respectively, with vertical lines in dark blue (Berkeley Earth) and light blue (ERA5) indicating the observed event. The respective region is indicated above the color bar.

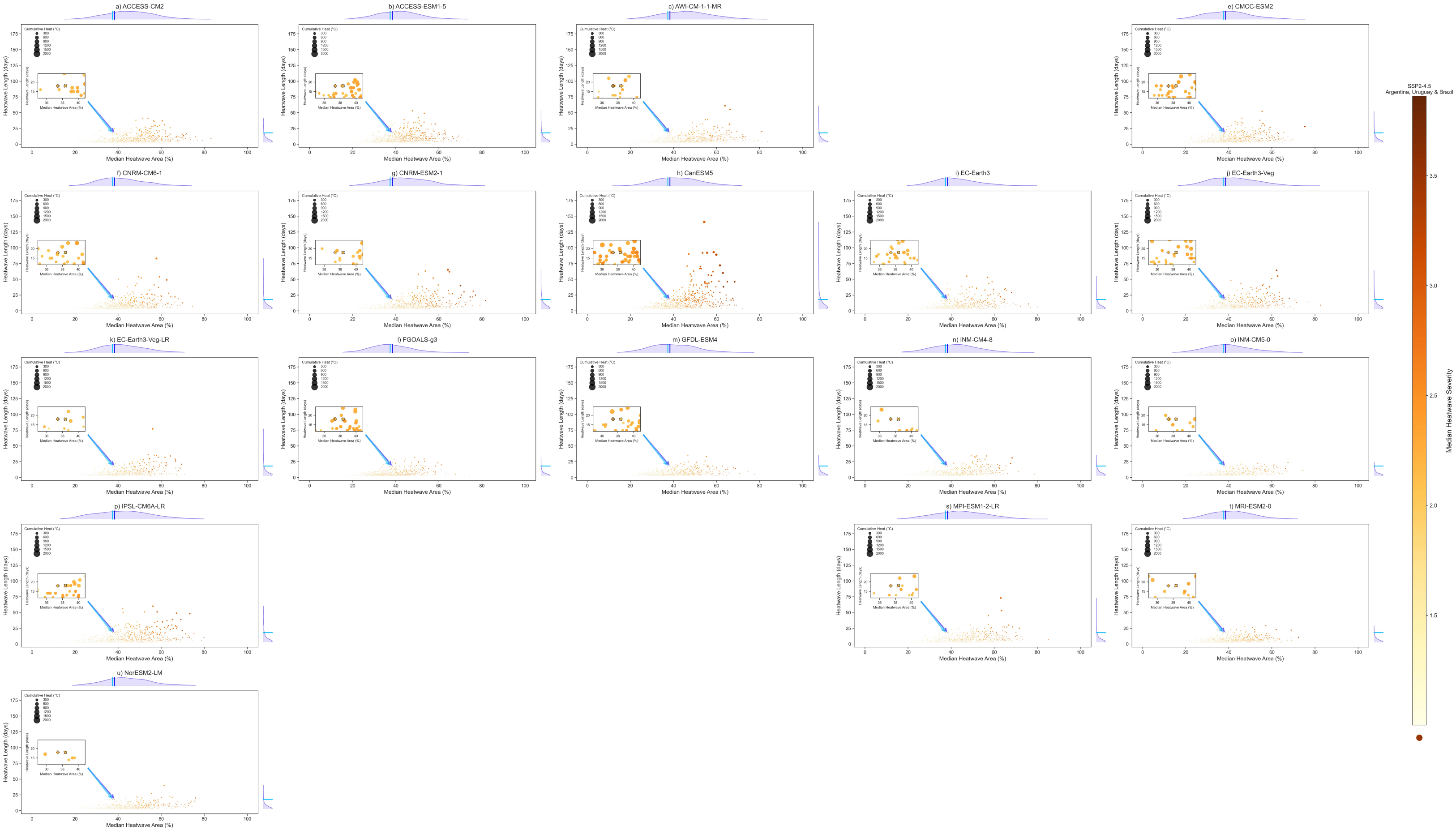
a) ACCESS-CM2
b) ACCESS-ESM1-5
c) AWI-CM-1-1-MR
e) CMCC-ESM2
f) CNRM-CM6-1
g) CNRM-ESM2-1
h) CanESM5
i) EC-Earth3
j) EC-Earth3-Veg
k) EC-Earth3-Veg-LR
l) FGOALS-g3
m) GFDL-ESM4
n) INM-CM4-8
o) INM-CM5-0
p) IPSL-CM6A-LR
s) MPI-ESM1-2-LR
t) MRI-ESM2-0
u) NorESM2-LM
Cumulative Heat (°C)
300
600
900
1200
1500
2000
Heatwave Length (days)
Median Heatwave Area (%)
SSP2-4.5
Argentina, Uruguay & Brazil
Median Heatwave Severity

Supplementary Figure 228: Multi-HW-characteristic figure according to SSP2-4.5 CMIP6 multi-model ensemble (MME) per model for Australia (Tx90p) - Each panel shows regional projections according to SSP2-4.5 model runs in the CMIP6 multi-model ensemble (MME) of four combined heatwave characteristics for each projected heatwave in the ensemble between 2020 and 2100. Each dot represents an individual heatwave in the projections, characterized by Median Heatwave Area (%) (x-axis) as latitude-weighted percentage of grid boxes affected by extreme heat, Heatwave Length (days) (y-axis) in days, Median Heatwave Severity (color), and Cumulative Heat (°C) (dot size). The characteristics of the observed event according to the Berkeley Earth observational data (x symbol) and the ERA5 reanalysis data (+ symbol), respectively, are pointed to by an arrow and a figure inset shows a zoom-in on this figure section. The figure inset shows the observed heatwave in the two reference data sets and projections in the indicated section of the respective sub-plot. It facilitates readability of color and size of the reference values and comparison to each other and the neighboring projections. Note that the zoom-in causes the dots to overlap more in the inset than in the main sub-plot. The univariate kernel density estimates along the x- (Median Heatwave Area (%)) and y-axis (Heatwave Length (days)) are on the top and right side of the subplots, respectively, with vertical lines in dark blue (Berkeley Earth) and light blue (ERA5) indicating the observed event. The respective region is indicated above the color bar.

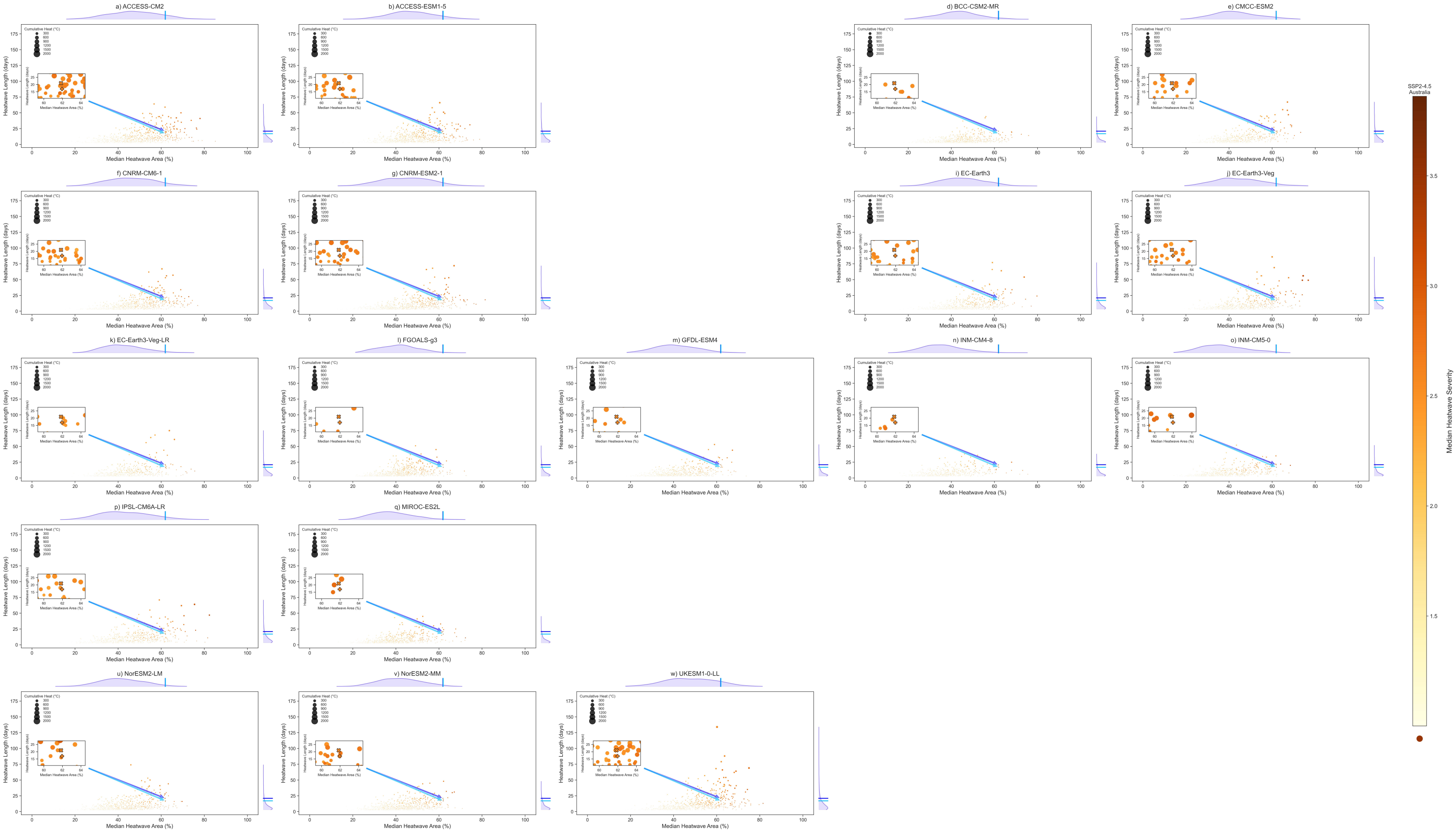

a) ACCESS-CM2
b) ACCESS-ESM1-5
d) BCC-CSM2-MR
e) CMCC-ESM2
f) CNRM-CM6-1
g) CNRM-ESM2-1
i) EC-Earth3
j) EC-Earth3-Veg
k) EC-Earth3-Veg-LR
l) FGOALS-g3
m) GFDL-ESM4
n) INM-CM4-8
o) INM-CM5-0
p) IPSL-CM6A-LR
q) MIROC-ES2L
u) NorESM2-LM
v) NorESM2-MM
w) UKESM1-0-LL
Cumulative Heat (°C)
300
600
900
1200
1500
2000
Heatwave Length (days)
Median Heatwave Area (%)
SSP2-4.5
Australia
Median Heatwave Severity

Supplementary Figure 229: Multi-HW-characteristic figure according to SSP2-4.5 CMIP6 multi-model ensemble (MME) per model for Central Siberia & Central Asia (Tx90p) - Each panel shows regional projections according to SSP2-4.5 model runs in the CMIP6 multi-model ensemble (MME) of four combined heatwave characteristics for each projected heatwave in the ensemble between 2020 and 2100. Each dot represents an individual heatwave in the projections, characterized by Median Heatwave Area (%) (x-axis) as latitude-weighted percentage of grid boxes affected by extreme heat, Heatwave Length (days) (y-axis) in days, Median Heatwave Severity (color), and Cumulative Heat (°C) (dot size). The characteristics of the observed event according to the Berkeley Earth observational data (x symbol) and the ERA5 reanalysis data (+ symbol), respectively, are pointed to by an arrow and a figure inset shows a zoom-in on this figure section. The figure inset shows the observed heatwave in the two reference data sets and projections in the indicated section of the respective sub-plot. It facilitates readability of color and size of the reference values and comparison to each other and the neighboring projections. Note that the zoom-in causes the dots to overlap more in the inset than in the main sub-plot. The univariate kernel density estimates along the x- (Median Heatwave Area (%)) and y-axis (Heatwave Length (days)) are on the top and right side of the subplots, respectively, with vertical lines in dark blue (Berkeley Earth) and light blue (ERA5) indicating the observed event. The respective region is indicated above the color bar.

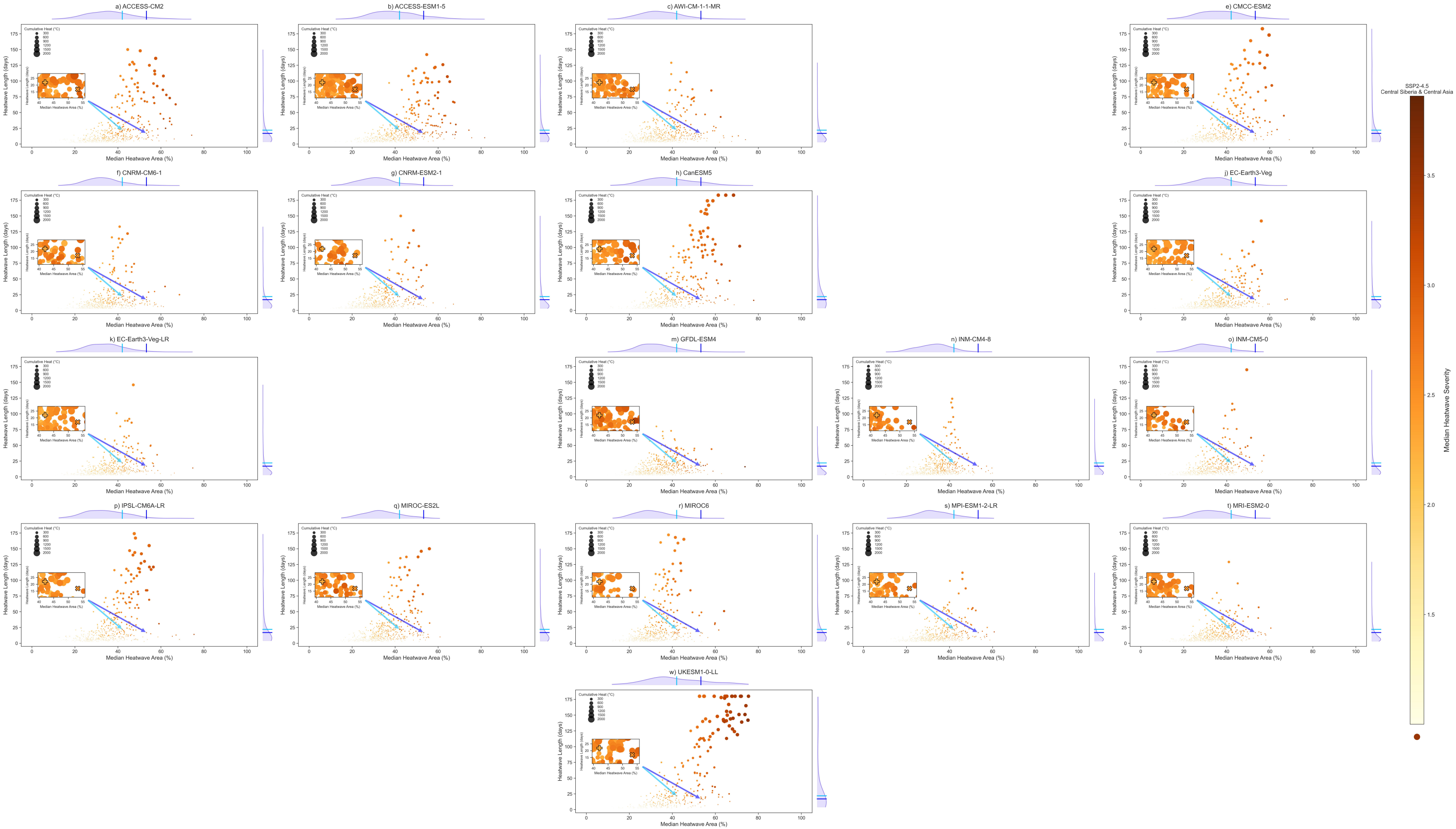

a) ACCESS-CM2
b) ACCESS-ESM1-5
c) AWI-CM-1-1-MR
e) CMCC-ESM2
f) CNRM-CM6-1
g) CNRM-ESM2-1
h) CanESM5
j) EC-Earth3-Veg
k) EC-Earth3-Veg-LR
m) GFDL-ESM4
n) INM-CM4-8
o) INM-CM5-0
p) IPSL-CM6A-LR
q) MIROC-ES2L
r) MIROC6
s) MPI-ESM1-2-LR
t) MRI-ESM2-0
w) UKESM1-0-LL
Cumulative Heat (°C)
300
600
900
1200
1500
2000
Heatwave Length (days)
Median Heatwave Area (%)
SSP2-4.5
Central Siberia & Central Asia
Median Heatwave Severity

Supplementary Figure 230: Multi-HW-characteristic figure according to SSP2-4.5 CMIP6 multi-model ensemble (MME) per model for China (2017) (Tx90p) - Each panel shows regional projections according to SSP2-4.5 model runs in the CMIP6 multi-model ensemble (MME) of four combined heatwave characteristics for each projected heatwave in the ensemble between 2020 and 2100. Each dot represents an individual heatwave in the projections, characterized by Median Heatwave Area (%) (x-axis) as latitude-weighted percentage of grid boxes affected by extreme heat, Heatwave Length (days) (y-axis) in days, Median Heatwave Severity (color), and Cumulative Heat (°C) (dot size). The characteristics of the observed event according to the Berkeley Earth observational data (x symbol) and the ERA5 reanalysis data (+ symbol), respectively, are pointed to by an arrow and a figure inset shows a zoom-in on this figure section. The figure inset shows the observed heatwave in the two reference data sets and projections in the indicated section of the respective sub-plot. It facilitates readability of color and size of the reference values and comparison to each other and the neighboring projections. Note that the zoom-in causes the dots to overlap more in the inset than in the main sub-plot. The univariate kernel density estimates along the x- (Median Heatwave Area (%)) and y-axis (Heatwave Length (days)) are on the top and right side of the subplots, respectively, with vertical lines in dark blue (Berkeley Earth) and light blue (ERA5) indicating the observed event. The respective region is indicated above the color bar.

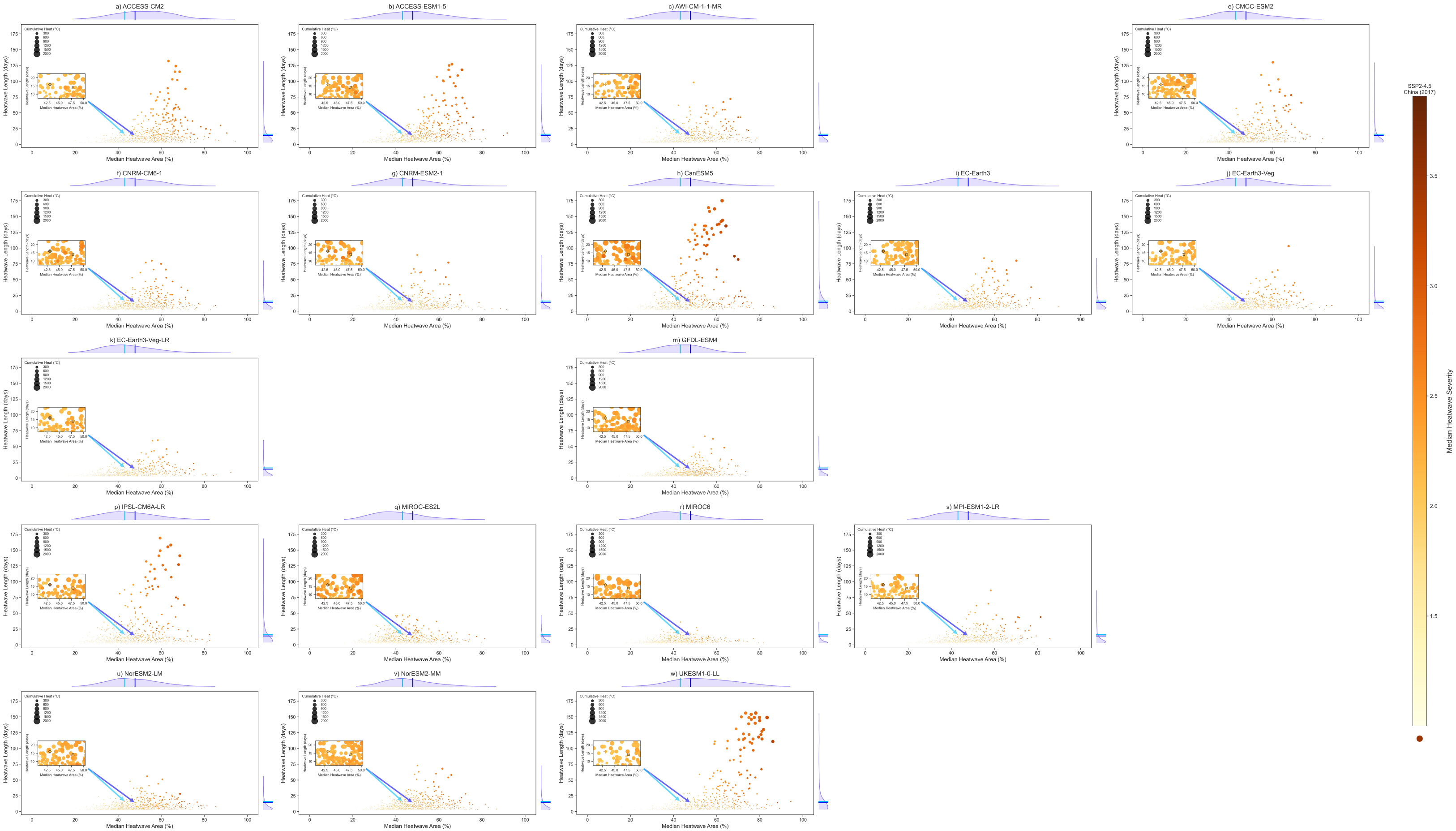

a) ACCESS-CM2
b) ACCESS-ESM1-5
c) AWI-CM-1-1-MR
e) CMCC-ESM2
f) CNRM-CM6-1
g) CNRM-ESM2-1
h) CanESM5
i) EC-Earth3
j) EC-Earth3-Veg
k) EC-Earth3-Veg-LR
m) GFDL-ESM4
p) IPSL-CM6A-LR
q) MIROC-ES2L
r) MIROC6
s) MPI-ESM1-2-LR
u) NorESM2-LM
v) NorESM2-MM
w) UKESM1-0-LL
Cumulative Heat (°C)
300
600
900
1200
1500
2000
Heatwave Length (days)
Median Heatwave Area (%)
SSP2-4.5
China (2017)
Median Heatwave Severity

Supplementary Figure 231: Multi-HW-characteristic figure according to SSP2-4.5 CMIP6 multi-model ensemble (MME) per model for China (Tx90p) - Each panel shows regional projections according to SSP2-4.5 model runs in the CMIP6 multi-model ensemble (MME) of four combined heatwave characteristics for each projected heatwave in the ensemble between 2020 and 2100. Each dot represents an individual heatwave in the projections, characterized by Median Heatwave Area (%) (x-axis) as latitude-weighted percentage of grid boxes affected by extreme heat, Heatwave Length (days) (y-axis) in days, Median Heatwave Severity (color), and Cumulative Heat (°C) (dot size). The characteristics of the observed event according to the Berkeley Earth observational data (x symbol) and the ERA5 reanalysis data (+ symbol), respectively, are pointed to by an arrow and a figure inset shows a zoom-in on this figure section. The figure inset shows the observed heatwave in the two reference data sets and projections in the indicated section of the respective sub-plot. It facilitates readability of color and size of the reference values and comparison to each other and the neighboring projections. Note that the zoom-in causes the dots to overlap more in the inset than in the main sub-plot. The univariate kernel density estimates along the x- (Median Heatwave Area (%)) and y-axis (Heatwave Length (days)) are on the top and right side of the subplots, respectively, with vertical lines in dark blue (Berkeley Earth) and light blue (ERA5) indicating the observed event. The respective region is indicated above the color bar.

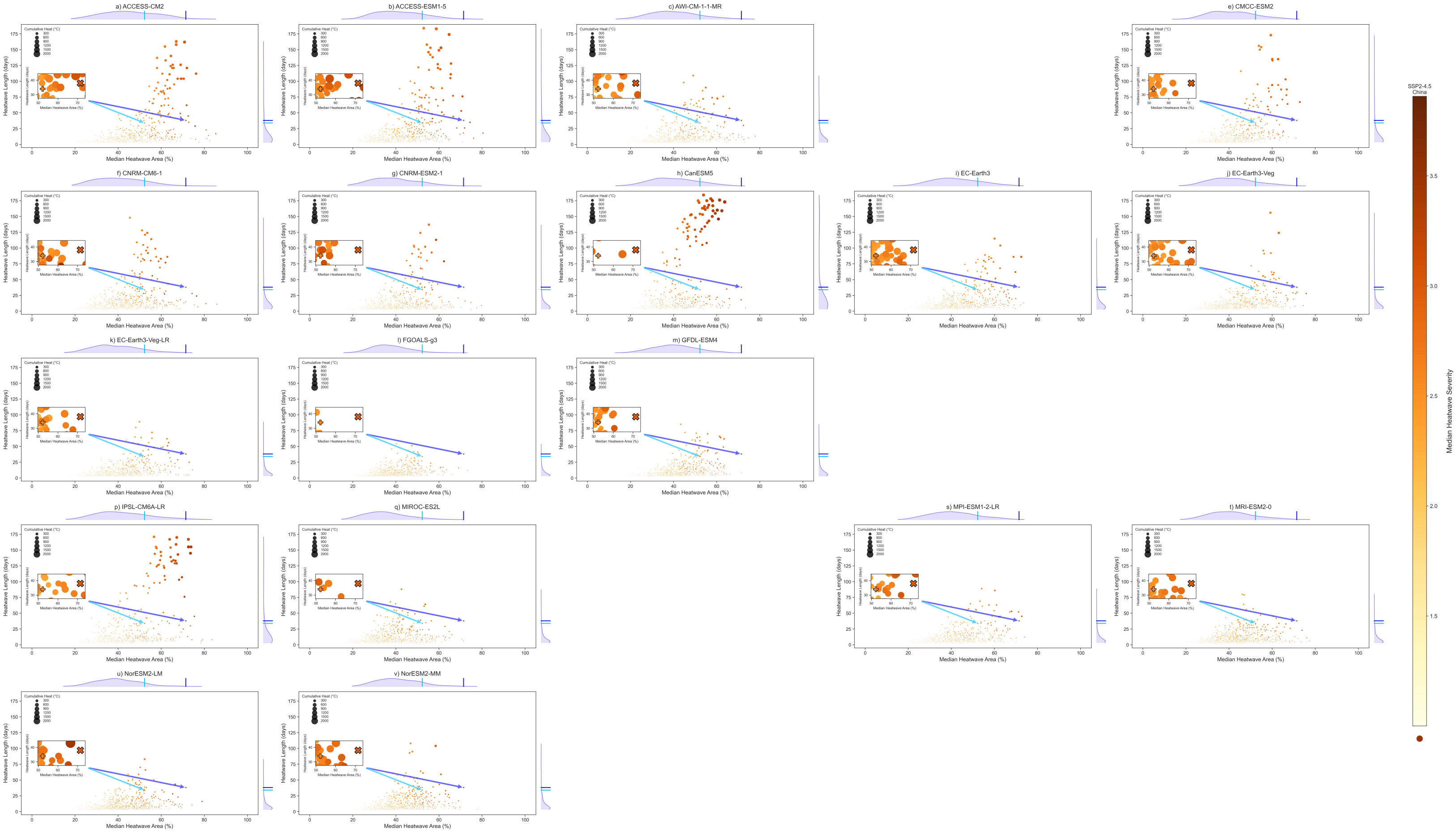
a) ACCESS-CM2
b) ACCESS-ESM1-5
c) AWI-CM-1-1-MR
e) CMCC-ESM2
f) CNRM-CM6-1
g) CNRM-ESM2-1
h) CanESM5
i) EC-Earth3
j) EC-Earth3-Veg
k) EC-Earth3-Veg-LR
l) FGOALS-g3
m) GFDL-ESM4
p) IPSL-CM6A-LR
q) MIROC-ES2L
s) MPI-ESM1-2-LR
t) MRI-ESM2-0
u) NorESM2-LM
v) NorESM2-MM
Cumulative Heat (°C)
Heatwave Length (days)
Median Heatwave Area (%)
SSP2-4.5
China
Median Heatwave Severity

Supplementary Figure 232: Multi-HW-characteristic figure according to SSP2-4.5 CMIP6 multi-model ensemble (MME) per model for Central & Eastern Europe (Tx90p) - Each panel shows regional projections according to SSP2-4.5 model runs in the CMIP6 multi-model ensemble (MME) of four combined heatwave characteristics for each projected heatwave in the ensemble between 2020 and 2100. Each dot represents an individual heatwave in the projections, characterized by Median Heatwave Area (%) (x-axis) as latitude-weighted percentage of grid boxes affected by extreme heat, Heatwave Length (days) (y-axis) in days, Median Heatwave Severity (color), and Cumulative Heat (°C) (dot size). The characteristics of the observed event according to the Berkeley Earth observational data (x symbol) and the ERA5 reanalysis data (+ symbol), respectively, are pointed to by an arrow and a figure inset shows a zoom-in on this figure section. The figure inset shows the observed heatwave in the two reference data sets and projections in the indicated section of the respective sub-plot. It facilitates readability of color and size of the reference values and comparison to each other and the neighboring projections. Note that the zoom-in causes the dots to overlap more in the inset than in the main sub-plot. The univariate kernel density estimates along the x- (Median Heatwave Area (%)) and y-axis (Heatwave Length (days)) are on the top and right side of the subplots, respectively, with vertical lines in dark blue (Berkeley Earth) and light blue (ERA5) indicating the observed event. The respective region is indicated above the color bar.

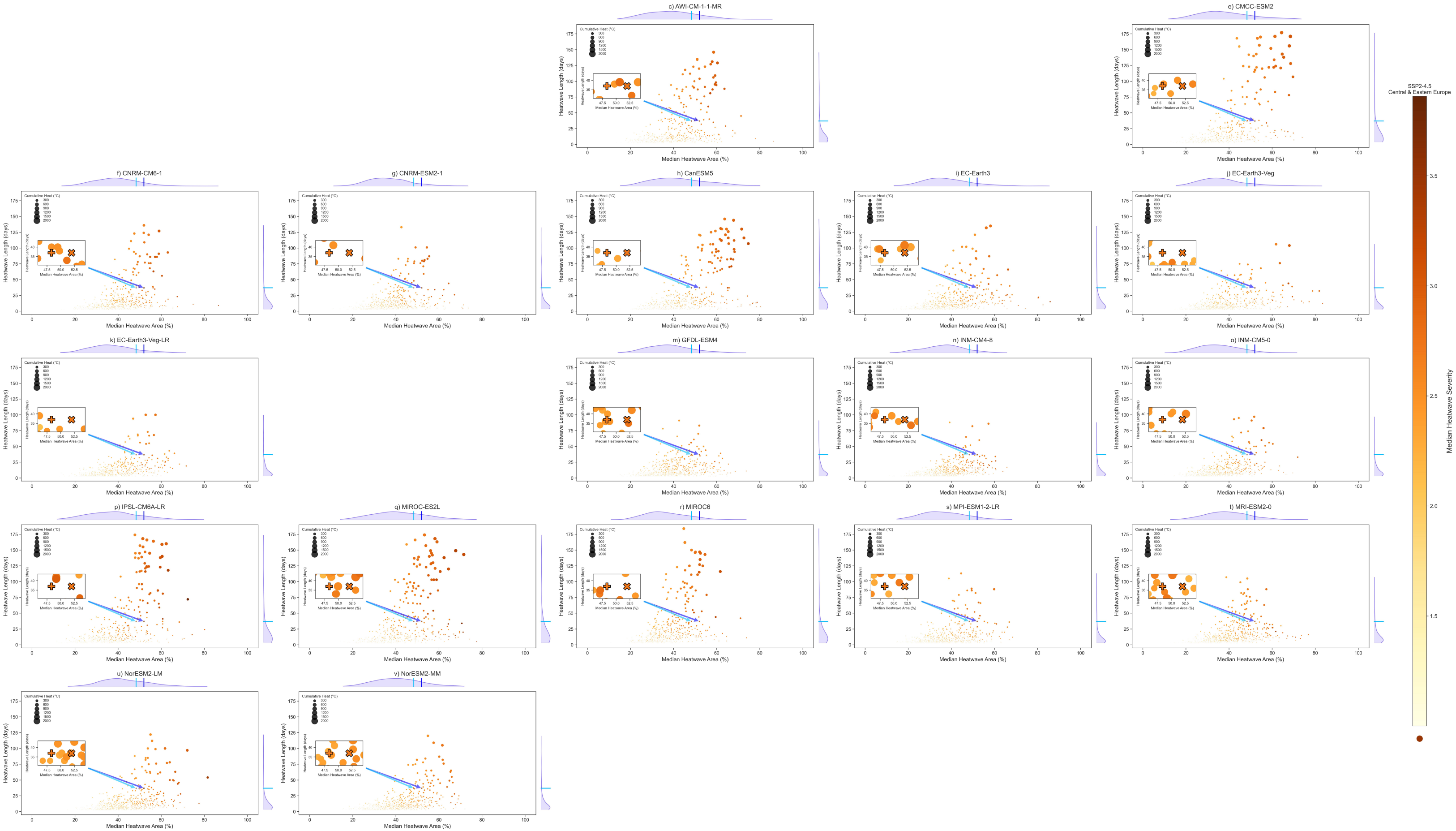

c) AWI-CM-1-1-MR
e) CMCC-ESM2
f) CNRM-CM6-1
g) CNRM-ESM2-1
h) CanESM5
i) EC-Earth3
j) EC-Earth3-Veg
k) EC-Earth3-Veg-LR
m) GFDL-ESM4
n) INM-CM4-8
o) INM-CM5-0
p) IPSL-CM6A-LR
q) MIROC-ES2L
r) MIROC6
s) MPI-ESM1-2-LR
t) MRI-ESM2-0
u) NorESM2-LM
v) NorESM2-MM
Cumulative Heat (°C)
300
600
900
1200
1500
2000
Heatwave Length (days)
Median Heatwave Area (%)
SSP2-4.5
Central & Eastern Europe
Median Heatwave Severity

Supplementary Figure 233: Multi-HW-characteristic figure according to SSP2-4.5 CMIP6 multi-model ensemble (MME) per model for Europe (Tx90p) - Each panel shows regional projections according to SSP2-4.5 model runs in the CMIP6 multi-model ensemble (MME) of four combined heatwave characteristics for each projected heatwave in the ensemble between 2020 and 2100. Each dot represents an individual heatwave in the projections, characterized by Median Heatwave Area (%) (x-axis) as latitude-weighted percentage of grid boxes affected by extreme heat, Heatwave Length (days) (y-axis) in days, Median Heatwave Severity (color), and Cumulative Heat (°C) (dot size). The characteristics of the observed event according to the Berkeley Earth observational data (x symbol) and the ERA5 reanalysis data (+ symbol), respectively, are pointed to by an arrow and a figure inset shows a zoom-in on this figure section. The figure inset shows the observed heatwave in the two reference data sets and projections in the indicated section of the respective sub-plot. It facilitates readability of color and size of the reference values and comparison to each other and the neighboring projections. Note that the zoom-in causes the dots to overlap more in the inset than in the main sub-plot. The univariate kernel density estimates along the x- (Median Heatwave Area (%)) and y-axis (Heatwave Length (days)) are on the top and right side of the subplots, respectively, with vertical lines in dark blue (Berkeley Earth) and light blue (ERA5) indicating the observed event. The respective region is indicated above the color bar.

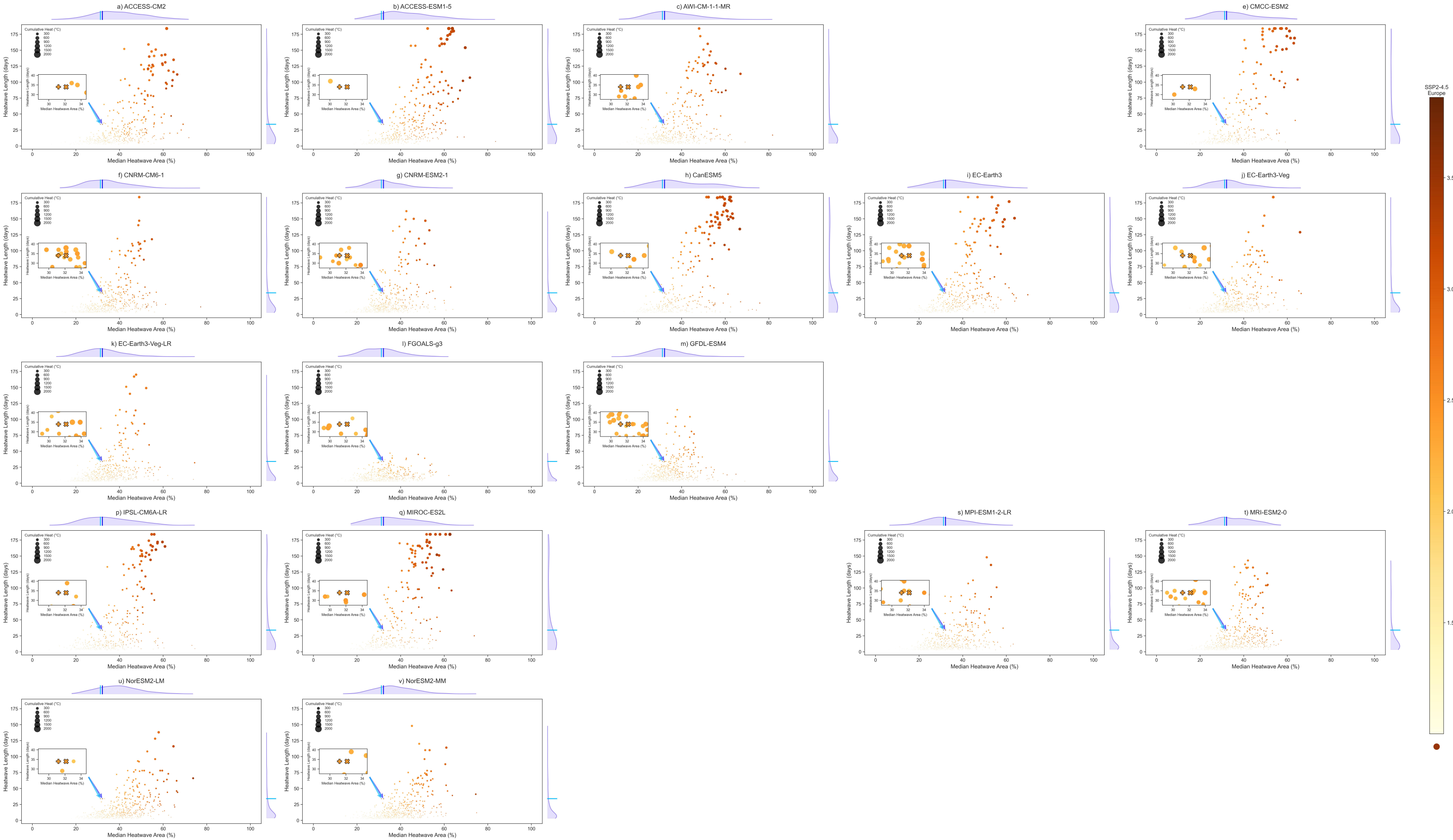

a) ACCESS-CM2
b) ACCESS-ESM1-5
c) AWI-CM-1-1-MR
e) CMCC-ESM2
f) CNRM-CM6-1
g) CNRM-ESM2-1
h) CanESM5
i) EC-Earth3
j) EC-Earth3-Veg
k) EC-Earth3-Veg-LR
l) FGOALS-g3
m) GFDL-ESM4
p) IPSL-CM6A-LR
q) MIROC-ES2L
s) MPI-ESM1-2-LR
t) MRI-ESM2-0
u) NorESM2-LM
v) NorESM2-MM
Cumulative Heat (°C)
300
600
900
1200
1500
2000
Heatwave Length (days)
Median Heatwave Area (%)
SSP2-4.5
Europe
Median Heatwave Severity

Supplementary Figure 234: Multi-HW-characteristic figure according to SSP2-4.5 CMIP6 multi-model ensemble (MME) per model for India & Middle East (Tx90p) - Each panel shows regional projections according to SSP2-4.5 model runs in the CMIP6 multi-model ensemble (MME) of four combined heatwave characteristics for each projected heatwave in the ensemble between 2020 and 2100. Each dot represents an individual heatwave in the projections, characterized by Median Heatwave Area (%) (x-axis) as latitude-weighted percentage of grid boxes affected by extreme heat, Heatwave Length (days) (y-axis) in days, Median Heatwave Severity (color), and Cumulative Heat (°C) (dot size). The characteristics of the observed event according to the Berkeley Earth observational data (x symbol) and the ERA5 reanalysis data (+ symbol), respectively, are pointed to by an arrow and a figure inset shows a zoom-in on this figure section. The figure inset shows the observed heatwave in the two reference data sets and projections in the indicated section of the respective sub-plot. It facilitates readability of color and size of the reference values and comparison to each other and the neighboring projections. Note that the zoom-in causes the dots to overlap more in the inset than in the main sub-plot. The univariate kernel density estimates along the x- (Median Heatwave Area (%)) and y-axis (Heatwave Length (days)) are on the top and right side of the subplots, respectively, with vertical lines in dark blue (Berkeley Earth) and light blue (ERA5) indicating the observed event. The respective region is indicated above the color bar.

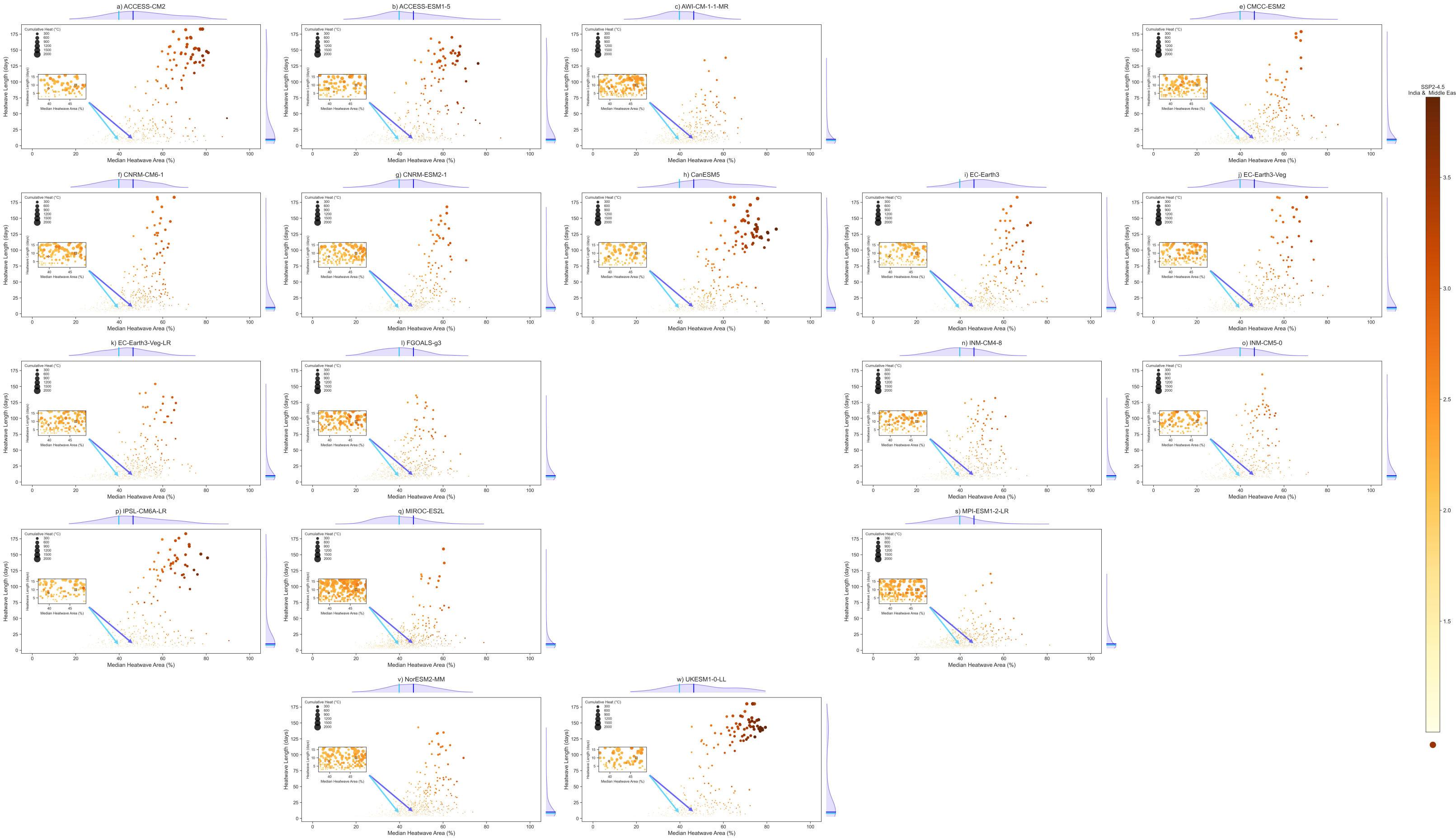

a) ACCESS-CM2
b) ACCESS-ESM1-5
c) AWI-CM-1-1-MR
e) CMCC-ESM2
f) CNRM-CM6-1
g) CNRM-ESM2-1
h) CanESM5
i) EC-Earth3
j) EC-Earth3-Veg
k) EC-Earth3-Veg-LR
l) FGOALS-g3
n) INM-CM4-8
o) INM-CM5-0
p) IPSL-CM6A-LR
q) MIROC-ES2L
s) MPI-ESM1-2-LR
v) NorESM2-MM
w) UKESM1-0-LL
Cumulative Heat (°C)
300
600
900
1200
1500
2000
Heatwave Length (days)
Median Heatwave Area (%)
SSP2-4.5
India & Middle East
Median Heatwave Severity

Supplementary Figure 235: Multi-HW-characteristic figure according to SSP2-4.5 CMIP6 multi-model ensemble (MME) per model for India & Pakistan (Tx90p) - Each panel shows regional projections according to SSP2-4.5 model runs in the CMIP6 multi-model ensemble (MME) of four combined heatwave characteristics for each projected heatwave in the ensemble between 2020 and 2100. Each dot represents an individual heatwave in the projections, characterized by Median Heatwave Area (%) (x-axis) as latitude-weighted percentage of grid boxes affected by extreme heat, Heatwave Length (days) (y-axis) in days, Median Heatwave Severity (color), and Cumulative Heat (°C) (dot size). The characteristics of the observed event according to the Berkeley Earth observational data (x symbol) and the ERA5 reanalysis data (+ symbol), respectively, are pointed to by an arrow and a figure inset shows a zoom-in on this figure section. The figure inset shows the observed heatwave in the two reference data sets and projections in the indicated section of the respective sub-plot. It facilitates readability of color and size of the reference values and comparison to each other and the neighboring projections. Note that the zoom-in causes the dots to overlap more in the inset than in the main sub-plot. The univariate kernel density estimates along the x- (Median Heatwave Area (%)) and y-axis (Heatwave Length (days)) are on the top and right side of the subplots, respectively, with vertical lines in dark blue (Berkeley Earth) and light blue (ERA5) indicating the observed event. The respective region is indicated above the color bar.

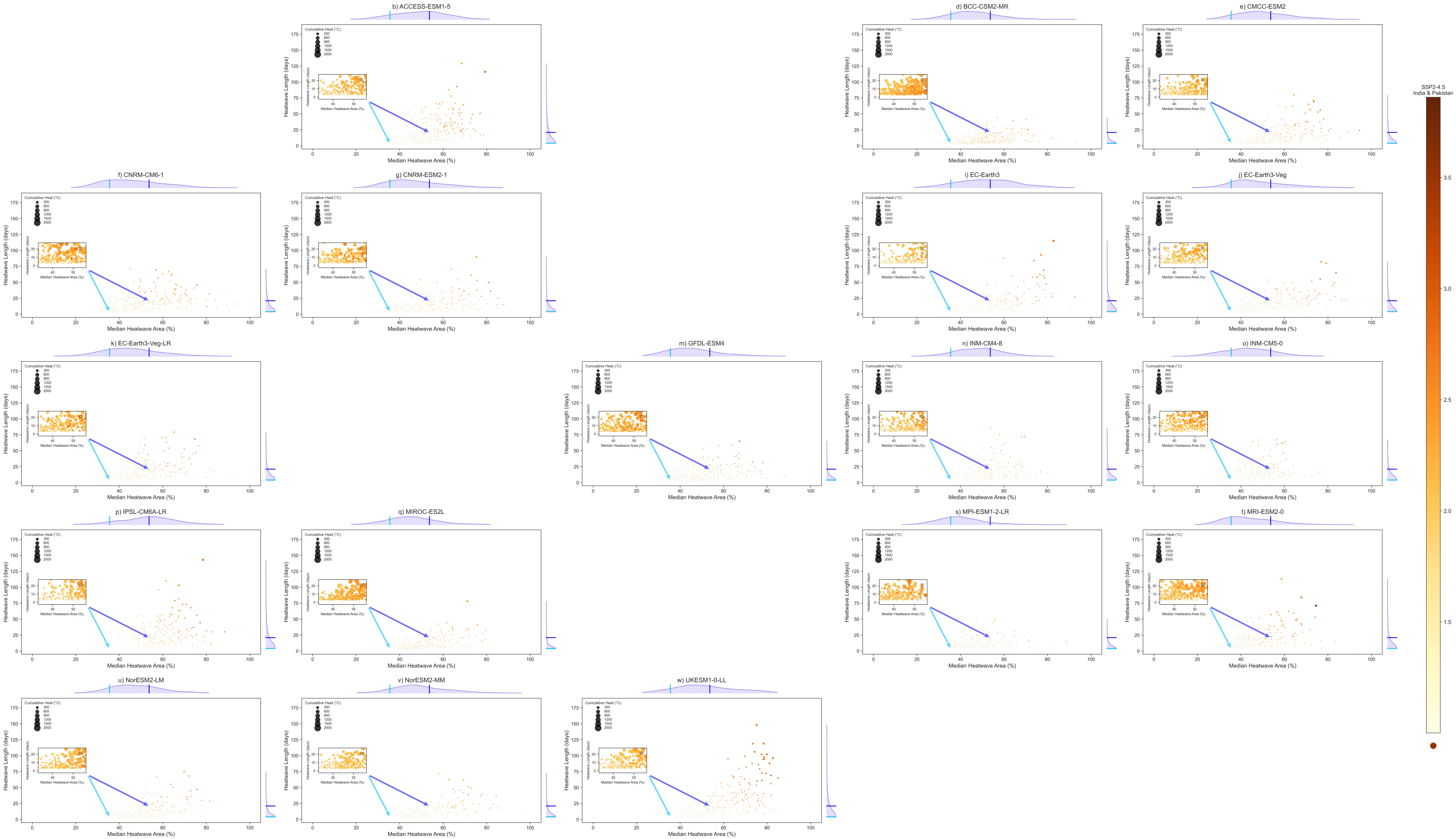
b) ACCESS-ESM1-5
d) BCC-CSM2-MR
e) CMCC-ESM2
f) CNRM-CM6-1
g) CNRM-ESM2-1
i) EC-Earth3
j) EC-Earth3-Veg
k) EC-Earth3-Veg-LR
m) GFDL-ESM4
n) INM-CM4-8
o) INM-CM5-0
p) IPSL-CM6A-LR
q) MIROC-ES2L
s) MPI-ESM1-2-LR
t) MRI-ESM2-0
u) NorESM2-LM
v) NorESM2-MM
w) UKESM1-0-LL
Cumulative Heat (°C)
300
600
900
1200
1500
2000
Heatwave Length (days)
Median Heatwave Area (%)
SSP2-4.5
India & Pakistan
Median Heatwave Severity

Supplementary Figure 236: Multi-HW-characteristic figure according to SSP2-4.5 CMIP6 multi-model ensemble (MME) per model for Middle East (Tx90p) - Each panel shows regional projections according to SSP2-4.5 model runs in the CMIP6 multi-model ensemble (MME) of four combined heatwave characteristics for each projected heatwave in the ensemble between 2020 and 2100. Each dot represents an individual heatwave in the projections, characterized by Median Heatwave Area (%) (x-axis) as latitude-weighted percentage of grid boxes affected by extreme heat, Heatwave Length (days) (y-axis) in days, Median Heatwave Severity (color), and Cumulative Heat (°C) (dot size). The characteristics of the observed event according to the Berkeley Earth observational data (x symbol) and the ERA5 reanalysis data (+ symbol), respectively, are pointed to by an arrow and a figure inset shows a zoom-in on this figure section. The figure inset shows the observed heatwave in the two reference data sets and projections in the indicated section of the respective sub-plot. It facilitates readability of color and size of the reference values and comparison to each other and the neighboring projections. Note that the zoom-in causes the dots to overlap more in the inset than in the main sub-plot. The univariate kernel density estimates along the x- (Median Heatwave Area (%)) and y-axis (Heatwave Length (days)) are on the top and right side of the subplots, respectively, with vertical lines in dark blue (Berkeley Earth) and light blue (ERA5) indicating the observed event. The respective region is indicated above the color bar.

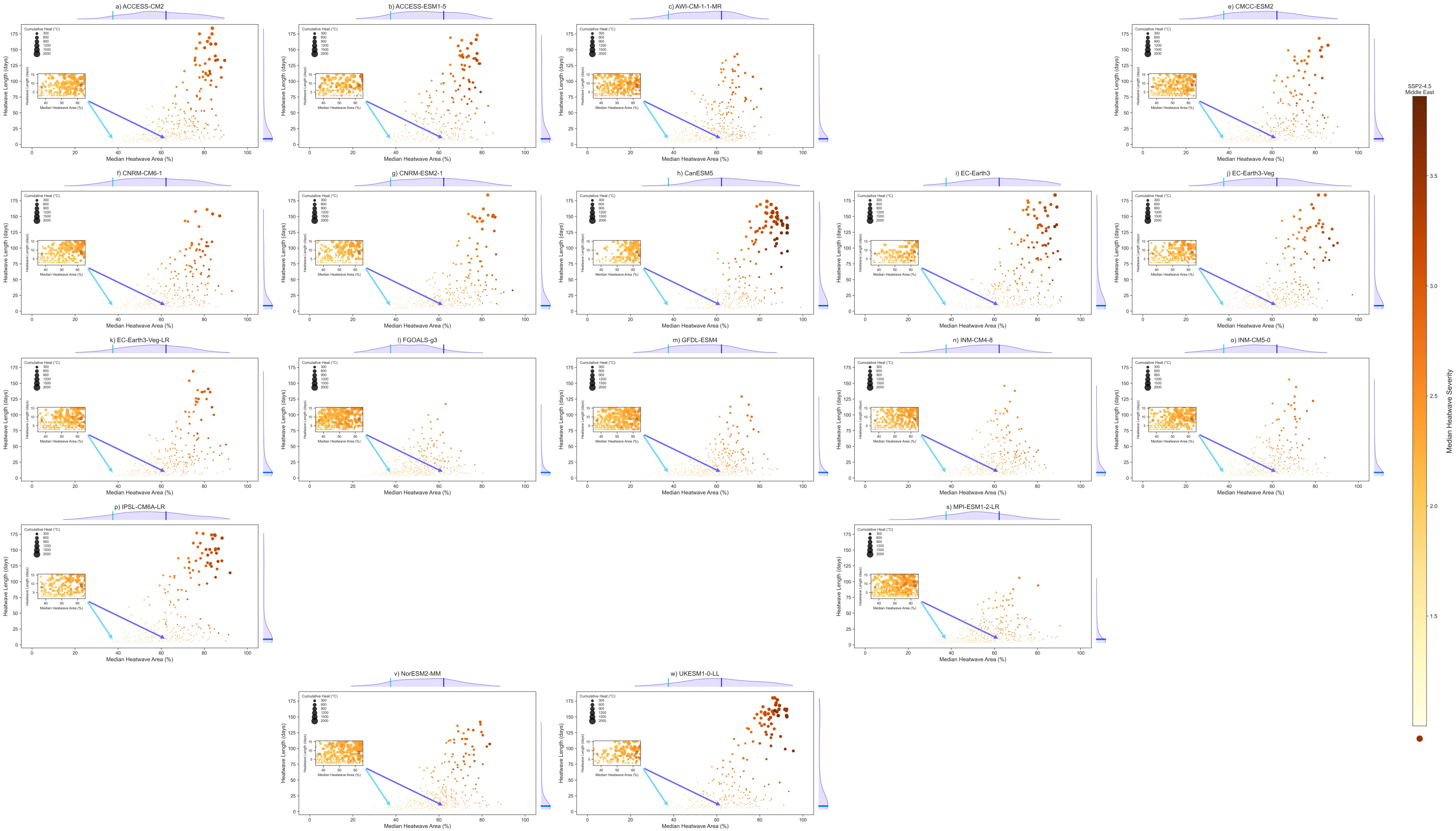

a) ACCESS-CM2
b) ACCESS-ESM1-5
c) AWI-CM-1-1-MR
e) CMCC-ESM2
f) CNRM-CM6-1
g) CNRM-ESM2-1
h) CanESM5
i) EC-Earth3
j) EC-Earth3-Veg
k) EC-Earth3-Veg-LR
l) FGOALS-g3
m) GFDL-ESM4
n) INM-CM4-8
o) INM-CM5-0
p) IPSL-CM6A-LR
s) MPI-ESM1-2-LR
v) NorESM2-MM
w) UKESM1-0-LL
Heatwave Length (days)
Median Heatwave Area (%)
Cumulative Heat (°C)
300
600
900
1200
1500
2000
SSP2-4.5
Middle East
Median Heatwave Severity

Supplementary Figure 237: Multi-HW-characteristic figure according to SSP2-4.5 CMIP6 multi-model ensemble (MME) per model for Northeast Siberia (Tx90p) - Each panel shows regional projections according to SSP2-4.5 model runs in the CMIP6 multi-model ensemble (MME) of four combined heatwave characteristics for each projected heatwave in the ensemble between 2020 and 2100. Each dot represents an individual heatwave in the projections, characterized by Median Heatwave Area (%) (x-axis) as latitude-weighted percentage of grid boxes affected by extreme heat, Heatwave Length (days) (y-axis) in days, Median Heatwave Severity (color), and Cumulative Heat (°C) (dot size). The characteristics of the observed event according to the Berkeley Earth observational data (x symbol) and the ERA5 reanalysis data (+ symbol), respectively, are pointed to by an arrow and a figure inset shows a zoom-in on this figure section. The figure inset shows the observed heatwave in the two reference data sets and projections in the indicated section of the respective sub-plot. It facilitates readability of color and size of the reference values and comparison to each other and the neighboring projections. Note that the zoom-in causes the dots to overlap more in the inset than in the main sub-plot. The univariate kernel density estimates along the x- (Median Heatwave Area (%)) and y-axis (Heatwave Length (days)) are on the top and right side of the subplots, respectively, with vertical lines in dark blue (Berkeley Earth) and light blue (ERA5) indicating the observed event. The respective region is indicated above the color bar.

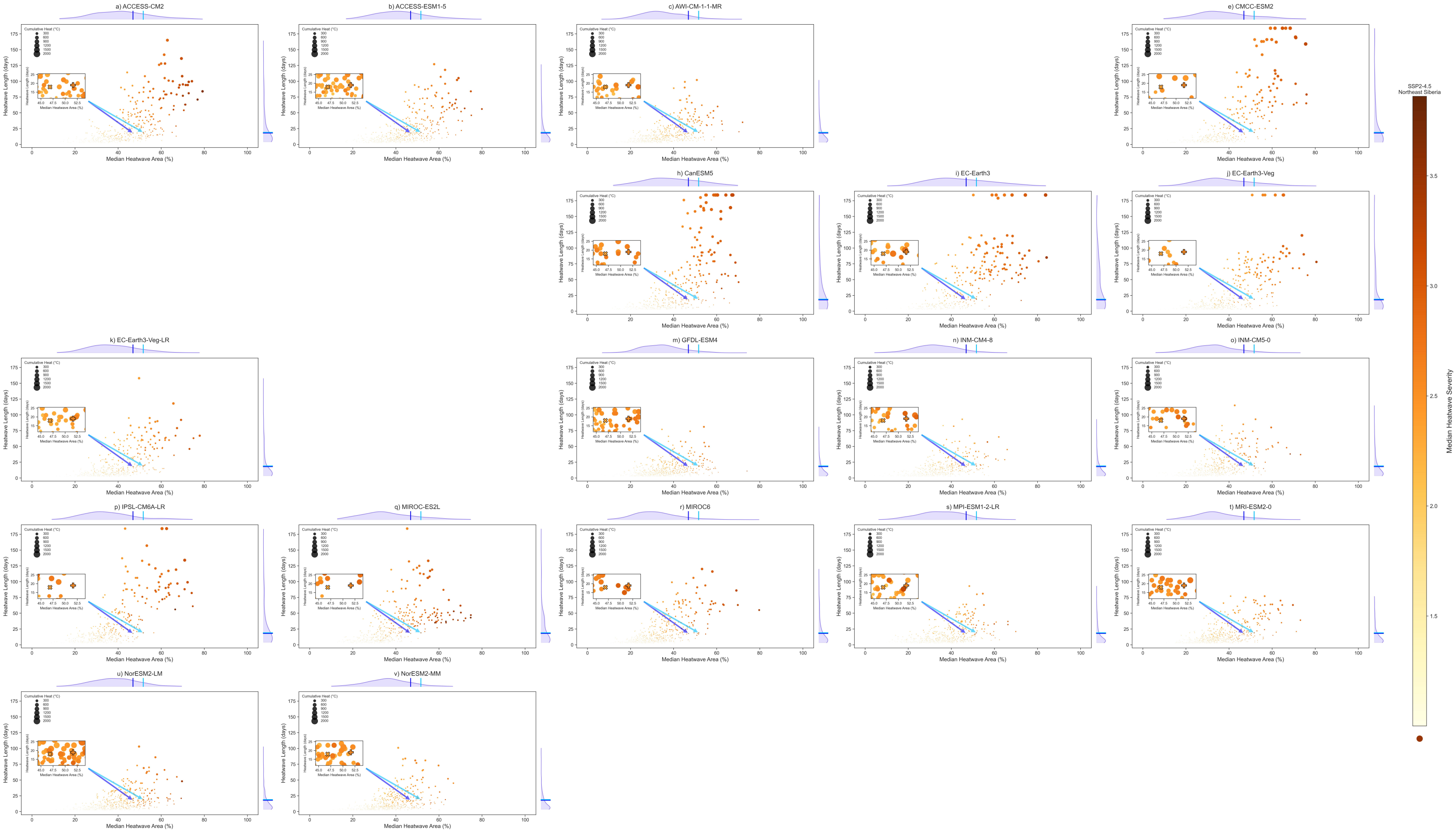
a) ACCESS-CM2
b) ACCESS-ESM1-5
c) AWI-CM-1-1-MR
e) CMCC-ESM2
h) CanESM5
i) EC-Earth3
j) EC-Earth3-Veg
k) EC-Earth3-Veg-LR
m) GFDL-ESM4
n) INM-CM4-8
o) INM-CM5-0
p) IPSL-CM6A-LR
q) MIROC-ES2L
r) MIROC6
s) MPI-ESM1-2-LR
t) MRI-ESM2-0
u) NorESM2-LM
v) NorESM2-MM
Cumulative Heat (°C)
300
600
900
1200
1500
2000
Heatwave Length (days)
Median Heatwave Area (%)
SSP2-4.5
Northeast Siberia
Median Heatwave Severity

Supplementary Figure 238: Multi-HW-characteristic figure according to SSP2-4.5 CMIP6 multi-model ensemble (MME) per model for Northern Africa & Middle East (Tx90p) - Each panel shows regional projections according to SSP2-4.5 model runs in the CMIP6 multi-model ensemble (MME) of four combined heatwave characteristics for each projected heatwave in the ensemble between 2020 and 2100. Each dot represents an individual heatwave in the projections, characterized by Median Heatwave Area (%) (x-axis) as latitude-weighted percentage of grid boxes affected by extreme heat, Heatwave Length (days) (y-axis) in days, Median Heatwave Severity (color), and Cumulative Heat (°C) (dot size). The characteristics of the observed event according to the Berkeley Earth observational data (x symbol) and the ERA5 reanalysis data (+ symbol), respectively, are pointed to by an arrow and a figure inset shows a zoom-in on this figure section. The figure inset shows the observed heatwave in the two reference data sets and projections in the indicated section of the respective sub-plot. It facilitates readability of color and size of the reference values and comparison to each other and the neighboring projections. Note that the zoom-in causes the dots to overlap more in the inset than in the main sub-plot. The univariate kernel density estimates along the x- (Median Heatwave Area (%)) and y-axis (Heatwave Length (days)) are on the top and right side of the subplots, respectively, with vertical lines in dark blue (Berkeley Earth) and light blue (ERA5) indicating the observed event. The respective region is indicated above the color bar.

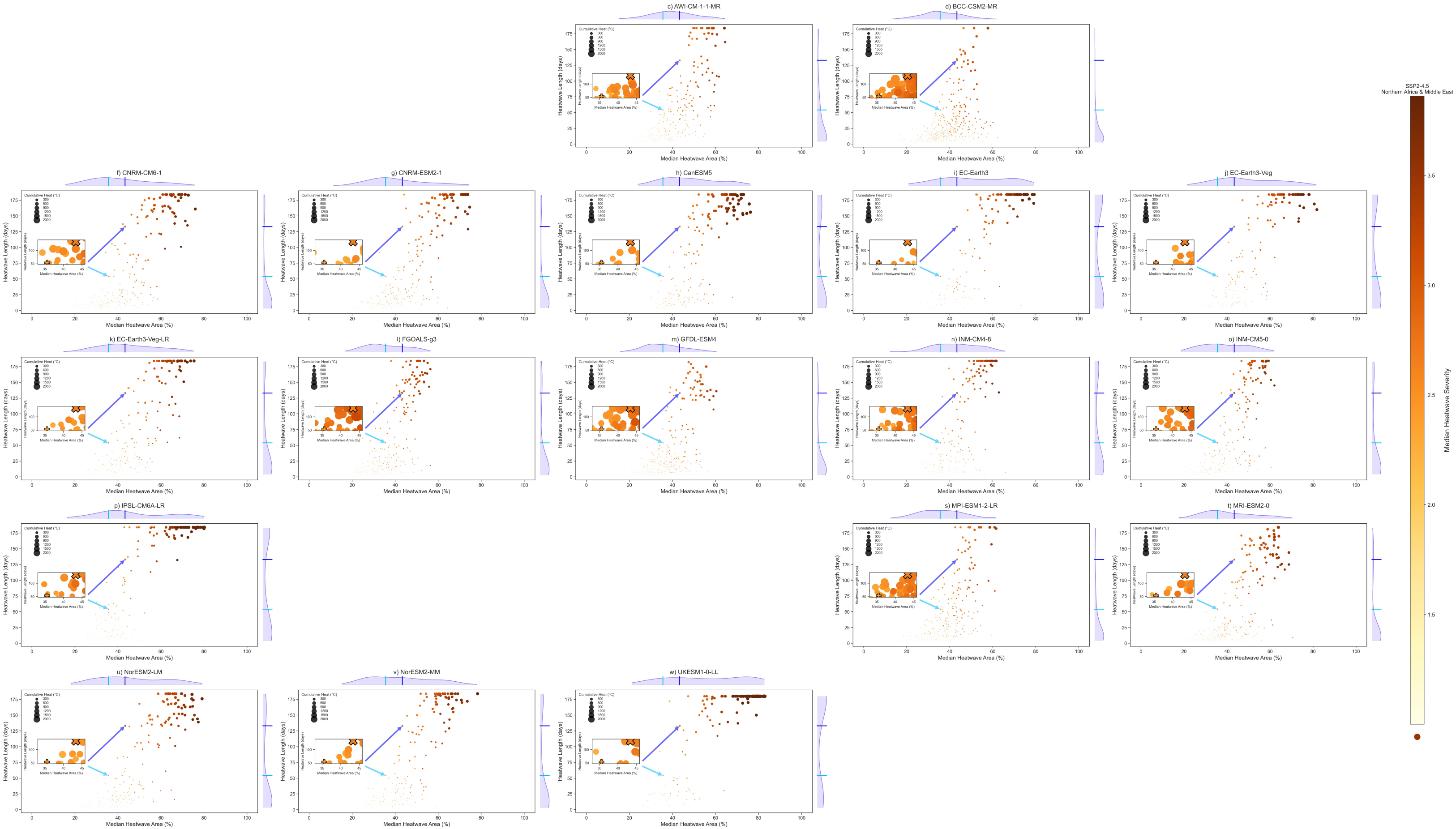
c) AWI-CM-1-1-MR
d) BCC-CSM2-MR
f) CNRM-CM6-1
g) CNRM-ESM2-1
h) CanESM5
i) EC-Earth3
j) EC-Earth3-Veg
k) EC-Earth3-Veg-LR
l) FGOALS-g3
m) GFDL-ESM4
n) INM-CM4-8
o) INM-CM5-0
p) IPSL-CM6A-LR
s) MPI-ESM1-2-LR
t) MRI-ESM2-0
u) NorESM2-LM
v) NorESM2-MM
w) UKESM1-0-LL
Cumulative Heat (°C)
300
600
900
1200
1500
2000
Heatwave Length (days)
Median Heatwave Area (%)
SSP2-4.5
Northern Africa & Middle East
Median Heatwave Severity

Supplementary Figure 239: Multi-HW-characteristic figure according to SSP2-4.5 CMIP6 multi-model ensemble (MME) per model for Pacific Northwest USA & Canada (Tx90p) - Each panel shows regional projections according to SSP2-4.5 model runs in the CMIP6 multi-model ensemble (MME) of four combined heatwave characteristics for each projected heatwave in the ensemble between 2020 and 2100. Each dot represents an individual heatwave in the projections, characterized by Median Heatwave Area (%) (x-axis) as latitude-weighted percentage of grid boxes affected by extreme heat, Heatwave Length (days) (y-axis) in days, Median Heatwave Severity (color), and Cumulative Heat (°C) (dot size). The characteristics of the observed event according to the Berkeley Earth observational data (x symbol) and the ERA5 reanalysis data (+ symbol), respectively, are pointed to by an arrow and a figure inset shows a zoom-in on this figure section. The figure inset shows the observed heatwave in the two reference data sets and projections in the indicated section of the respective sub-plot. It facilitates readability of color and size of the reference values and comparison to each other and the neighboring projections. Note that the zoom-in causes the dots to overlap more in the inset than in the main sub-plot. The univariate kernel density estimates along the x- (Median Heatwave Area (%)) and y-axis (Heatwave Length (days)) are on the top and right side of the subplots, respectively, with vertical lines in dark blue (Berkeley Earth) and light blue (ERA5) indicating the observed event. The respective region is indicated above the color bar.

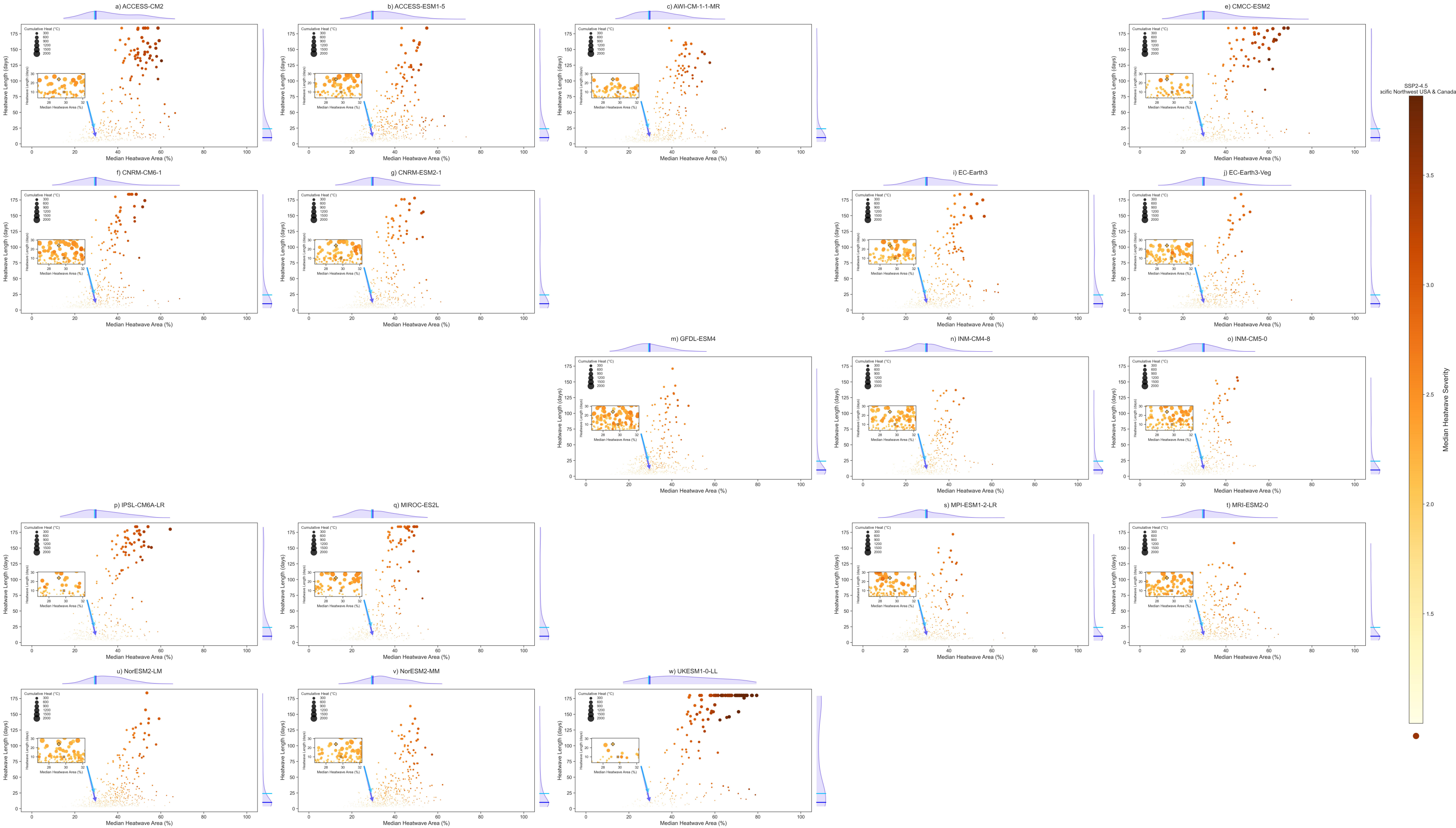

a) ACCESS-CM2
b) ACCESS-ESM1-5
c) AWI-CM-1-1-MR
e) CMCC-ESM2
f) CNRM-CM6-1
g) CNRM-ESM2-1
i) EC-Earth3
j) EC-Earth3-Veg
m) GFDL-ESM4
n) INM-CM4-8
o) INM-CM5-0
p) IPSL-CM6A-LR
q) MIROC-ES2L
s) MPI-ESM1-2-LR
t) MRI-ESM2-0
u) NorESM2-LM
v) NorESM2-MM
w) UKESM1-0-LL
Cumulative Heat (°C)
300
600
900
1200
1500
2000
Heatwave Length (days)
Median Heatwave Area (%)
SSP2-4.5
acific Northwest USA & Canada
Median Heatwave Severity

Supplementary Figure 240: Multi-HW-characteristic figure according to SSP2-4.5 CMIP6 multi-model ensemble (MME) per model for Russia (Tx90p) - Each panel shows regional projections according to SSP2-4.5 model runs in the CMIP6 multi-model ensemble (MME) of four combined heatwave characteristics for each projected heatwave in the ensemble between 2020 and 2100. Each dot represents an individual heatwave in the projections, characterized by Median Heatwave Area (%) (x-axis) as latitude-weighted percentage of grid boxes affected by extreme heat, Heatwave Length (days) (y-axis) in days, Median Heatwave Severity (color), and Cumulative Heat (°C) (dot size). The characteristics of the observed event according to the Berkeley Earth observational data (x symbol) and the ERA5 reanalysis data (+ symbol), respectively, are pointed to by an arrow and a figure inset shows a zoom-in on this figure section. The figure inset shows the observed heatwave in the two reference data sets and projections in the indicated section of the respective sub-plot. It facilitates readability of color and size of the reference values and comparison to each other and the neighboring projections. Note that the zoom-in causes the dots to overlap more in the inset than in the main sub-plot. The univariate kernel density estimates along the x- (Median Heatwave Area (%)) and y-axis (Heatwave Length (days)) are on the top and right side of the subplots, respectively, with vertical lines in dark blue (Berkeley Earth) and light blue (ERA5) indicating the observed event. The respective region is indicated above the color bar.

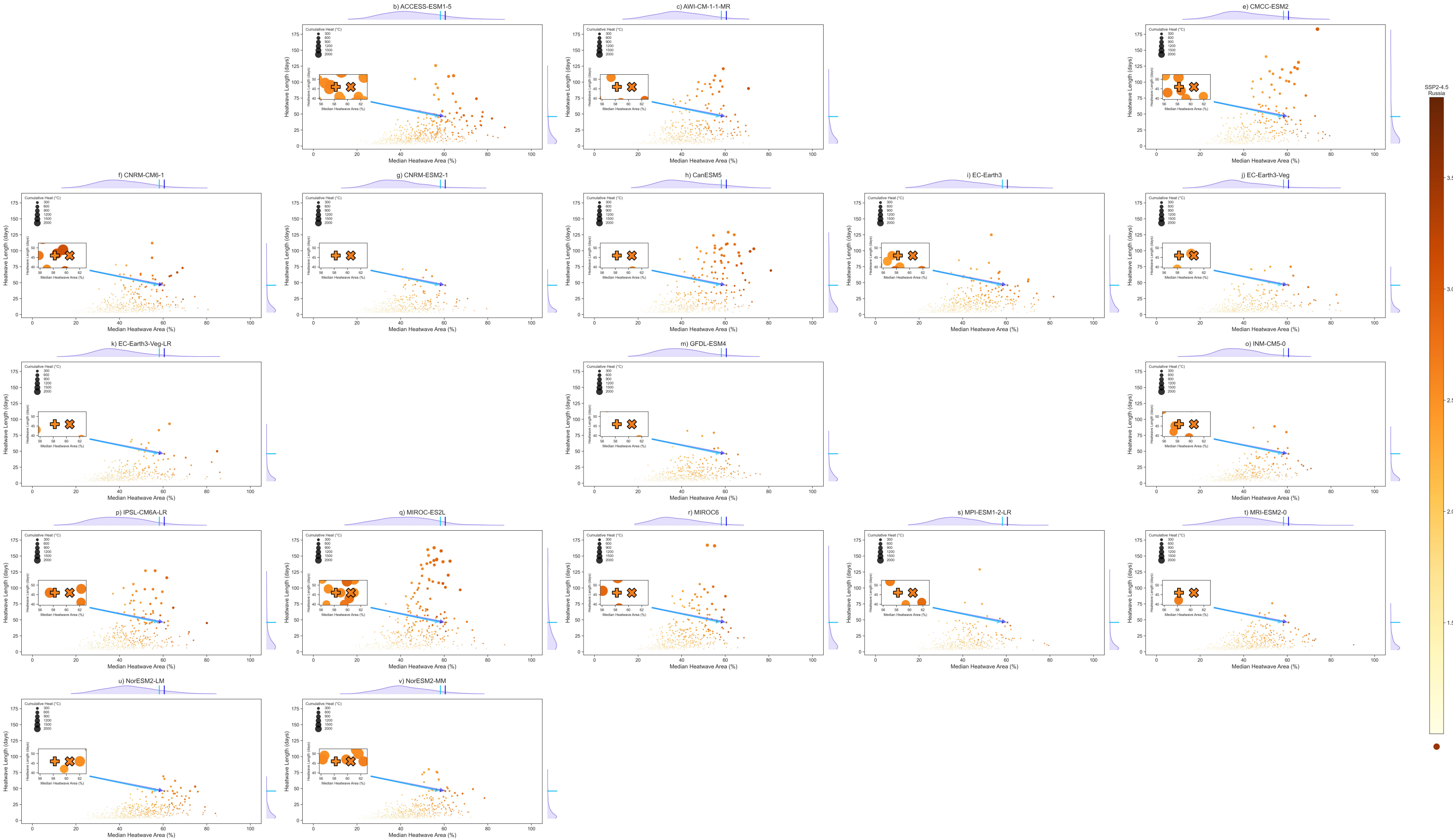

b) ACCESS-ESM1-5
c) AWI-CM-1-1-MR
e) CMCC-ESM2
f) CNRM-CM6-1
g) CNRM-ESM2-1
h) CanESM5
i) EC-Earth3
j) EC-Earth3-Veg
k) EC-Earth3-Veg-LR
m) GFDL-ESM4
o) INM-CM5-0
p) IPSL-CM6A-LR
q) MIROC-ES2L
r) MIROC6
s) MPI-ESM1-2-LR
t) MRI-ESM2-0
u) NorESM2-LM
v) NorESM2-MM
Cumulative Heat (°C)
300
600
900
1200
1500
2000
Heatwave Length (days)
Median Heatwave Area (%)
SSP2-4.5
Russia
Median Heatwave Severity
1.5
2.0
2.5
3.0
3.5

Supplementary Figure 241: Multi-HW-characteristic figure according to SSP2-4.5 CMIP6 multi-model ensemble (MME) per model for Southwestern USA & Mexico (Tx90p) - Each panel shows regional projections according to SSP2-4.5 model runs in the CMIP6 multi-model ensemble (MME) of four combined heatwave characteristics for each projected heatwave in the ensemble between 2020 and 2100. Each dot represents an individual heatwave in the projections, characterized by Median Heatwave Area (%) (x-axis) as latitude-weighted percentage of grid boxes affected by extreme heat, Heatwave Length (days) (y-axis) in days, Median Heatwave Severity (color), and Cumulative Heat (°C) (dot size). The characteristics of the observed event according to the Berkeley Earth observational data (x symbol) and the ERA5 reanalysis data (+ symbol), respectively, are pointed to by an arrow and a figure inset shows a zoom-in on this figure section. The figure inset shows the observed heatwave in the two reference data sets and projections in the indicated section of the respective sub-plot. It facilitates readability of color and size of the reference values and comparison to each other and the neighboring projections. Note that the zoom-in causes the dots to overlap more in the inset than in the main sub-plot. The univariate kernel density estimates along the x- (Median Heatwave Area (%)) and y-axis (Heatwave Length (days)) are on the top and right side of the subplots, respectively, with vertical lines in dark blue (Berkeley Earth) and light blue (ERA5) indicating the observed event. The respective region is indicated above the color bar.

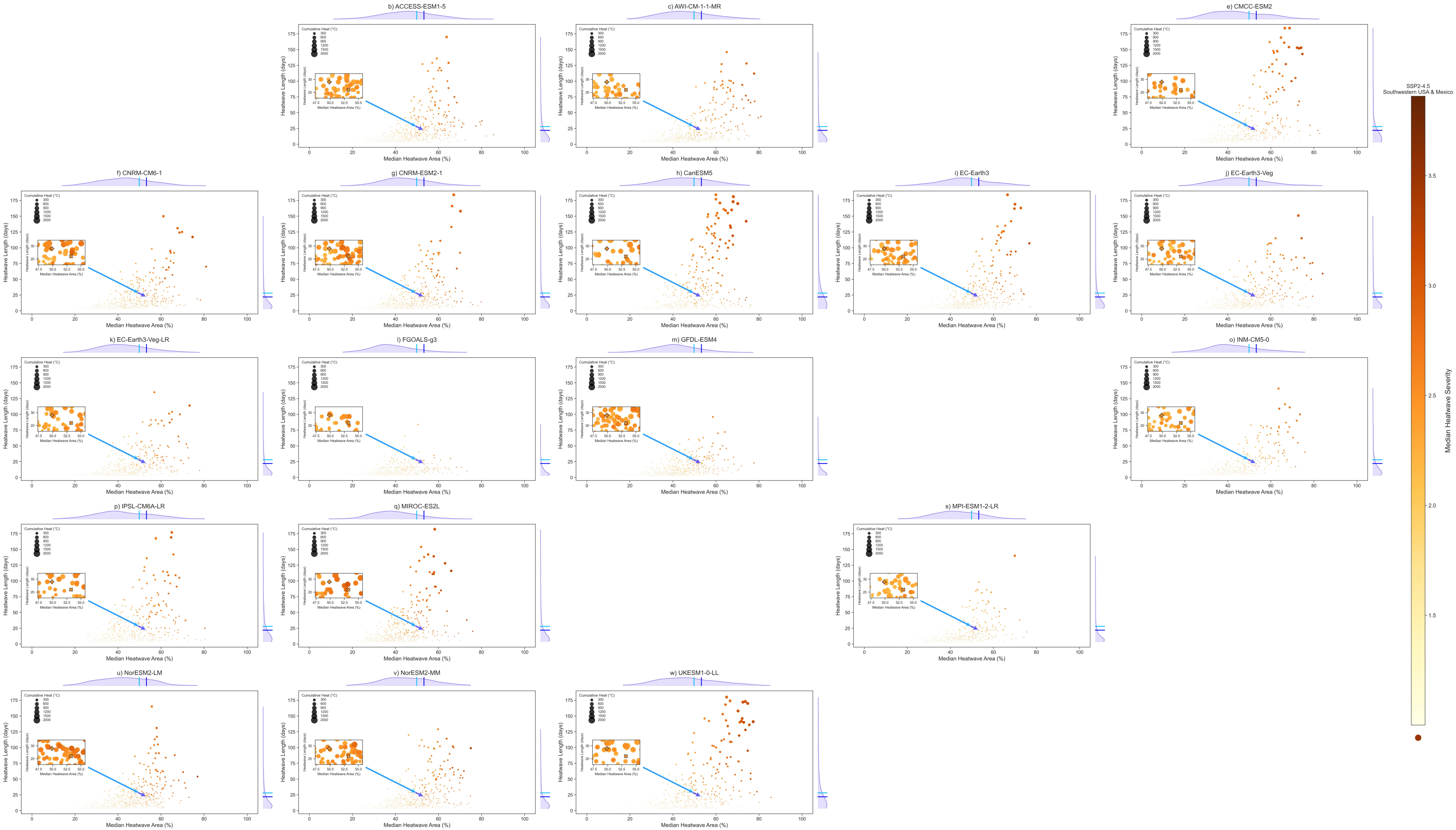
b) ACCESS-ESM1-5
c) AWI-CM-1-1-MR
e) CMCC-ESM2
f) CNRM-CM6-1
g) CNRM-ESM2-1
h) CanESM5
i) EC-Earth3
j) EC-Earth3-Veg
k) EC-Earth3-Veg-LR
l) FGOALS-g3
m) GFDL-ESM4
o) INM-CM5-0
p) IPSL-CM6A-LR
q) MIROC-ES2L
s) MPI-ESM1-2-LR
u) NorESM2-LM
v) NorESM2-MM
w) UKESM1-0-LL
Heatwave Length (days)
Median Heatwave Area (%)
Cumulative Heat (°C)
300
600
900
1200
1500
2000
SSP2-4.5
Southwestern USA & Mexico
Median Heatwave Severity

Supplementary Figure 242: Multi-HW-characteristic figure according to SSP2-4.5 CMIP6 multi-model ensemble (MME) per model for Siberia (Tx90p) - Each panel shows regional projections according to SSP2-4.5 model runs in the CMIP6 multi-model ensemble (MME) of four combined heatwave characteristics for each projected heatwave in the ensemble between 2020 and 2100. Each dot represents an individual heatwave in the projections, characterized by Median Heatwave Area (%) (x-axis) as latitude-weighted percentage of grid boxes affected by extreme heat, Heatwave Length (days) (y-axis) in days, Median Heatwave Severity (color), and Cumulative Heat (°C) (dot size). The characteristics of the observed event according to the Berkeley Earth observational data (x symbol) and the ERA5 reanalysis data (+ symbol), respectively, are pointed to by an arrow and a figure inset shows a zoom-in on this figure section. The figure inset shows the observed heatwave in the two reference data sets and projections in the indicated section of the respective sub-plot. It facilitates readability of color and size of the reference values and comparison to each other and the neighboring projections. Note that the zoom-in causes the dots to overlap more in the inset than in the main sub-plot. The univariate kernel density estimates along the x- (Median Heatwave Area (%)) and y-axis (Heatwave Length (days)) are on the top and right side of the subplots, respectively, with vertical lines in dark blue (Berkeley Earth) and light blue (ERA5) indicating the observed event. The respective region is indicated above the color bar.

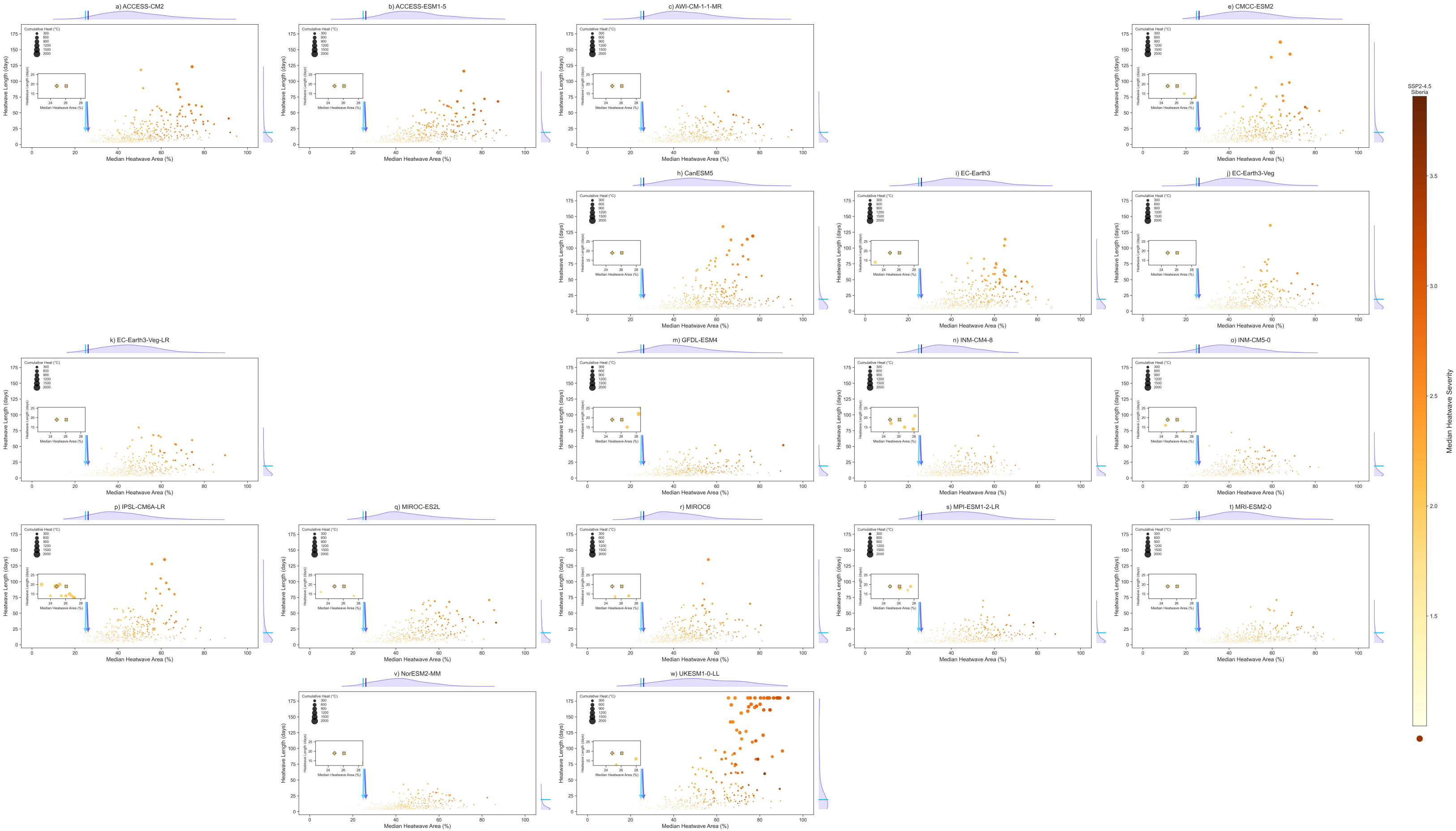

a) ACCESS-CM2
b) ACCESS-ESM1-5
c) AWI-CM-1-1-MR
e) CMCC-ESM2
h) CanESM5
i) EC-Earth3
j) EC-Earth3-Veg
k) EC-Earth3-Veg-LR
m) GFDL-ESM4
n) INM-CM4-8
o) INM-CM5-0
p) IPSL-CM6A-LR
q) MIROC-ES2L
r) MIROC6
s) MPI-ESM1-2-LR
t) MRI-ESM2-0
v) NorESM2-MM
w) UKESM1-0-LL
Cumulative Heat (°C)
300
600
900
1200
1500
2000
Heatwave Length (days)
Median Heatwave Area (%)
SSP2-4.5
Siberia
Median Heatwave Severity

Supplementary Figure 243: Multi-HW-characteristic figure according to SSP2-4.5 CMIP6 multi-model ensemble (MME) per model for South America (Tx90p) - Each panel shows regional projections according to SSP2-4.5 model runs in the CMIP6 multi-model ensemble (MME) of four combined heatwave characteristics for each projected heatwave in the ensemble between 2020 and 2100. Each dot represents an individual heatwave in the projections, characterized by Median Heatwave Area (%) (x-axis) as latitude-weighted percentage of grid boxes affected by extreme heat, Heatwave Length (days) (y-axis) in days, Median Heatwave Severity (color), and Cumulative Heat (°C) (dot size). The characteristics of the observed event according to the Berkeley Earth observational data (x symbol) and the ERA5 reanalysis data (+ symbol), respectively, are pointed to by an arrow and a figure inset shows a zoom-in on this figure section. The figure inset shows the observed heatwave in the two reference data sets and projections in the indicated section of the respective sub-plot. It facilitates readability of color and size of the reference values and comparison to each other and the neighboring projections. Note that the zoom-in causes the dots to overlap more in the inset than in the main sub-plot. The univariate kernel density estimates along the x- (Median Heatwave Area (%)) and y-axis (Heatwave Length (days)) are on the top and right side of the subplots, respectively, with vertical lines in dark blue (Berkeley Earth) and light blue (ERA5) indicating the observed event. The respective region is indicated above the color bar.

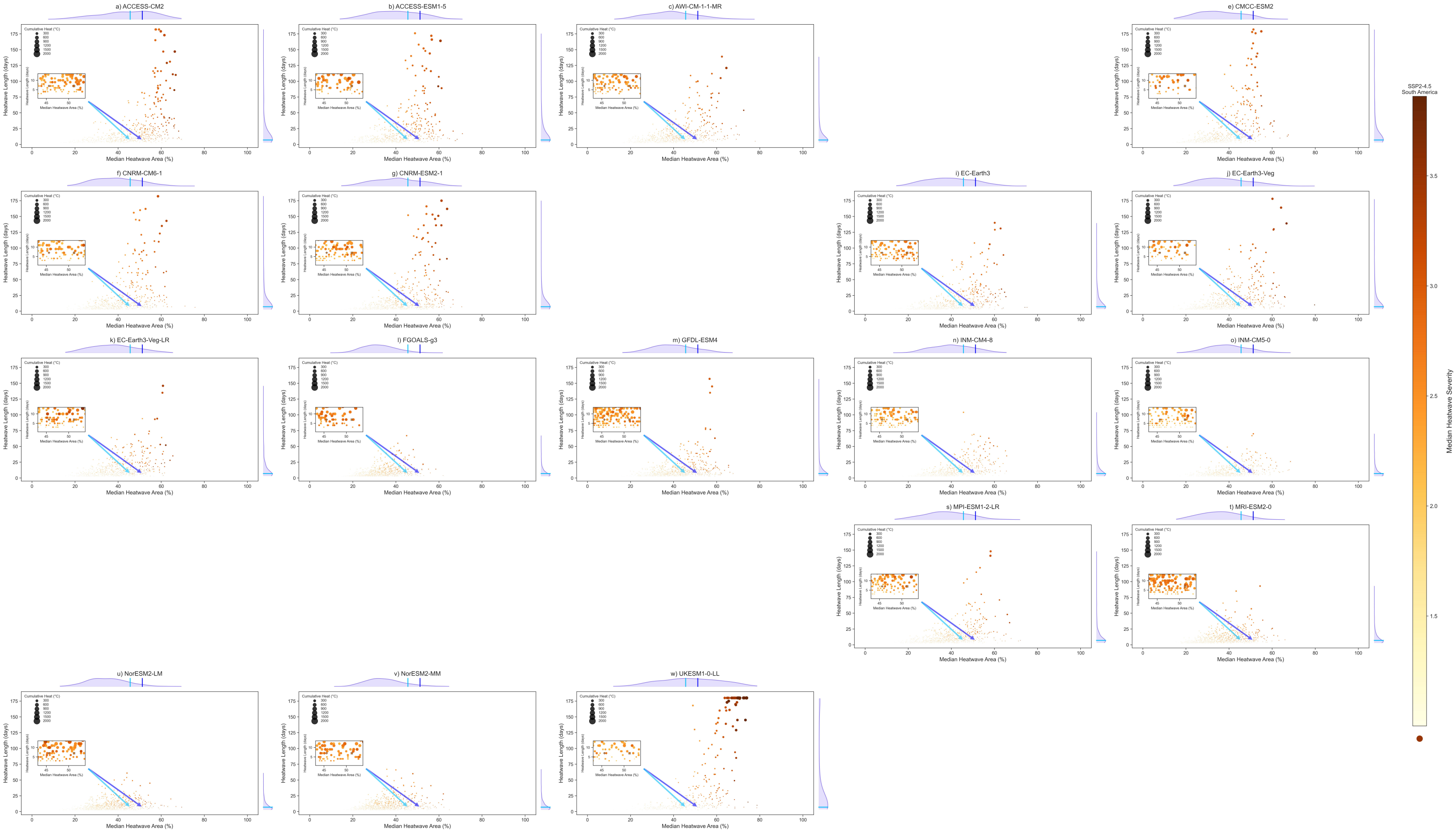
a) ACCESS-CM2
b) ACCESS-ESM1-5
c) AWI-CM-1-1-MR
e) CMCC-ESM2
f) CNRM-CM6-1
g) CNRM-ESM2-1
i) EC-Earth3
j) EC-Earth3-Veg
k) EC-Earth3-Veg-LR
l) FGOALS-g3
m) GFDL-ESM4
n) INM-CM4-8
o) INM-CM5-0
s) MPI-ESM1-2-LR
t) MRI-ESM2-0
u) NorESM2-LM
v) NorESM2-MM
w) UKESM1-0-LL
Cumulative Heat (°C)
300
600
900
1200
1500
2000
Heatwave Length (days)
Median Heatwave Area (%)
SSP2-4.5
South America
Median Heatwave Severity
1.5
2.0
2.5
3.0
3.5

Supplementary Figure 244: Multi-HW-characteristic figure according to SSP2-4.5 CMIP6 multi-model ensemble (MME) per model for Northern Southeast Asia (Tx90p) - Each panel shows regional projections according to SSP2-4.5 model runs in the CMIP6 multi-model ensemble (MME) of four combined heatwave characteristics for each projected heatwave in the ensemble between 2020 and 2100. Each dot represents an individual heatwave in the projections, characterized by Median Heatwave Area (%) (x-axis) as latitude-weighted percentage of grid boxes affected by extreme heat, Heatwave Length (days) (y-axis) in days, Median Heatwave Severity (color), and Cumulative Heat (°C) (dot size). The characteristics of the observed event according to the Berkeley Earth observational data (x symbol) and the ERA5 reanalysis data (+ symbol), respectively, are pointed to by an arrow and a figure inset shows a zoom-in on this figure section. The figure inset shows the observed heatwave in the two reference data sets and projections in the indicated section of the respective sub-plot. It facilitates readability of color and size of the reference values and comparison to each other and the neighboring projections. Note that the zoom-in causes the dots to overlap more in the inset than in the main sub-plot. The univariate kernel density estimates along the x- (Median Heatwave Area (%)) and y-axis (Heatwave Length (days)) are on the top and right side of the subplots, respectively, with vertical lines in dark blue (Berkeley Earth) and light blue (ERA5) indicating the observed event. The respective region is indicated above the color bar.

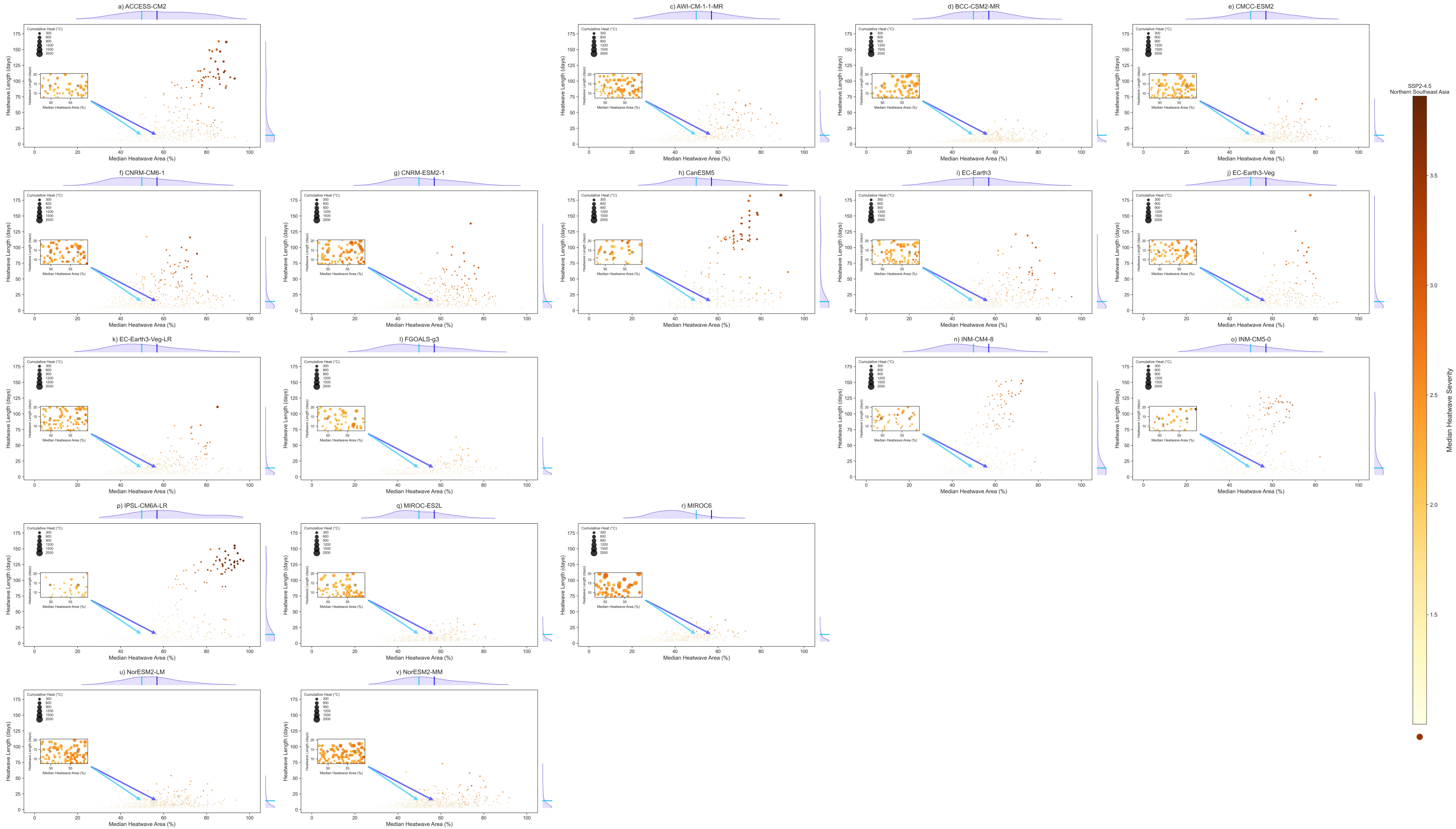

a) ACCESS-CM2
c) AWI-CM-1-1-MR
d) BCC-CSM2-MR
e) CMCC-ESM2
f) CNRM-CM6-1
g) CNRM-ESM2-1
h) CanESM5
i) EC-Earth3
j) EC-Earth3-Veg
k) EC-Earth3-Veg-LR
l) FGOALS-g3
n) INM-CM4-8
o) INM-CM5-0
p) IPSL-CM6A-LR
q) MIROC-ES2L
r) MIROC6
u) NorESM2-LM
v) NorESM2-MM
Heatwave Length (days)
Median Heatwave Area (%)
Cumulative Heat (°C)
300
600
900
1200
1500
2000
SSP2-4.5
Northern Southeast Asia
Median Heatwave Severity

Supplementary Figure 245: Multi-HW-characteristic figure according to SSP2-4.5 CMIP6 multi-model ensemble (MME) per model for Southern Africa (Tx90p) - Each panel shows regional projections according to SSP2-4.5 model runs in the CMIP6 multi-model ensemble (MME) of four combined heatwave characteristics for each projected heatwave in the ensemble between 2020 and 2100. Each dot represents an individual heatwave in the projections, characterized by Median Heatwave Area (%) (x-axis) as latitude-weighted percentage of grid boxes affected by extreme heat, Heatwave Length (days) (y-axis) in days, Median Heatwave Severity (color), and Cumulative Heat (°C) (dot size). The characteristics of the observed event according to the Berkeley Earth observational data (x symbol) and the ERA5 reanalysis data (+ symbol), respectively, are pointed to by an arrow and a figure inset shows a zoom-in on this figure section. The figure inset shows the observed heatwave in the two reference data sets and projections in the indicated section of the respective sub-plot. It facilitates readability of color and size of the reference values and comparison to each other and the neighboring projections. Note that the zoom-in causes the dots to overlap more in the inset than in the main sub-plot. The univariate kernel density estimates along the x- (Median Heatwave Area (%)) and y-axis (Heatwave Length (days)) are on the top and right side of the subplots, respectively, with vertical lines in dark blue (Berkeley Earth) and light blue (ERA5) indicating the observed event. The respective region is indicated above the color bar.

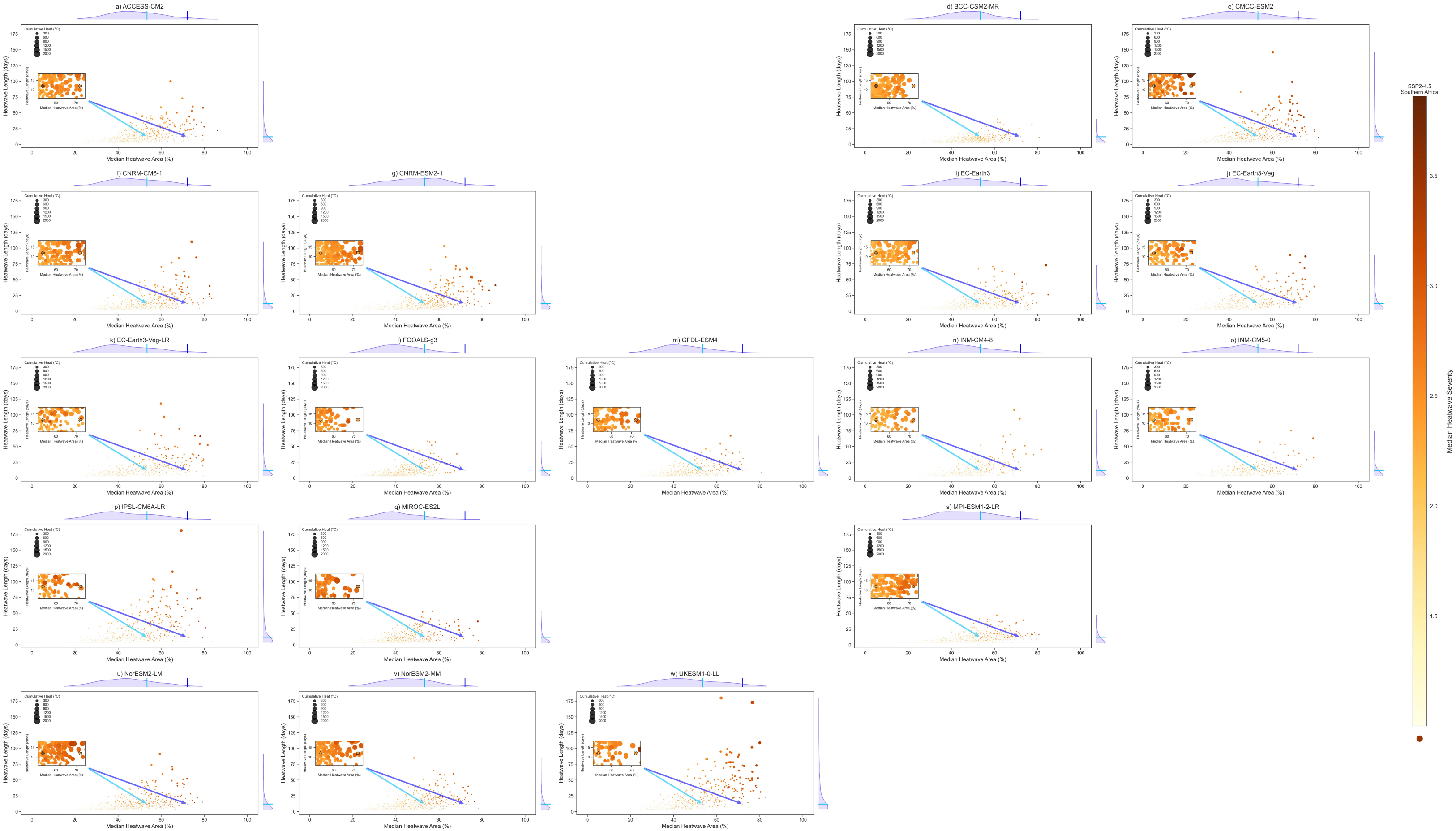
a) ACCESS-CM2
d) BCC-CSM2-MR
e) CMCC-ESM2
f) CNRM-CM6-1
g) CNRM-ESM2-1
i) EC-Earth3
j) EC-Earth3-Veg
k) EC-Earth3-Veg-LR
l) FGOALS-g3
m) GFDL-ESM4
n) INM-CM4-8
o) INM-CM5-0
p) IPSL-CM6A-LR
q) MIROC-ES2L
s) MPI-ESM1-2-LR
u) NorESM2-LM
v) NorESM2-MM
w) UKESM1-0-LL
Cumulative Heat (°C)
300
600
900
1200
1500
2000
Heatwave Length (days)
Median Heatwave Area (%)
SSP2-4.5
Southern Africa
Median Heatwave Severity

Supplementary Figure 246: Multi-HW-characteristic figure according to SSP2-4.5 CMIP6 multi-model ensemble (MME) per model for Eastern USA (Tx90p) - Each panel shows regional projections according to SSP2-4.5 model runs in the CMIP6 multi-model ensemble (MME) of four combined heatwave characteristics for each projected heatwave in the ensemble between 2020 and 2100. Each dot represents an individual heatwave in the projections, characterized by Median Heatwave Area (%) (x-axis) as latitude-weighted percentage of grid boxes affected by extreme heat, Heatwave Length (days) (y-axis) in days, Median Heatwave Severity (color), and Cumulative Heat (°C) (dot size). The characteristics of the observed event according to the Berkeley Earth observational data (x symbol) and the ERA5 reanalysis data (+ symbol), respectively, are pointed to by an arrow and a figure inset shows a zoom-in on this figure section. The figure inset shows the observed heatwave in the two reference data sets and projections in the indicated section of the respective sub-plot. It facilitates readability of color and size of the reference values and comparison to each other and the neighboring projections. Note that the zoom-in causes the dots to overlap more in the inset than in the main sub-plot. The univariate kernel density estimates along the x- (Median Heatwave Area (%)) and y-axis (Heatwave Length (days)) are on the top and right side of the subplots, respectively, with vertical lines in dark blue (Berkeley Earth) and light blue (ERA5) indicating the observed event. The respective region is indicated above the color bar.

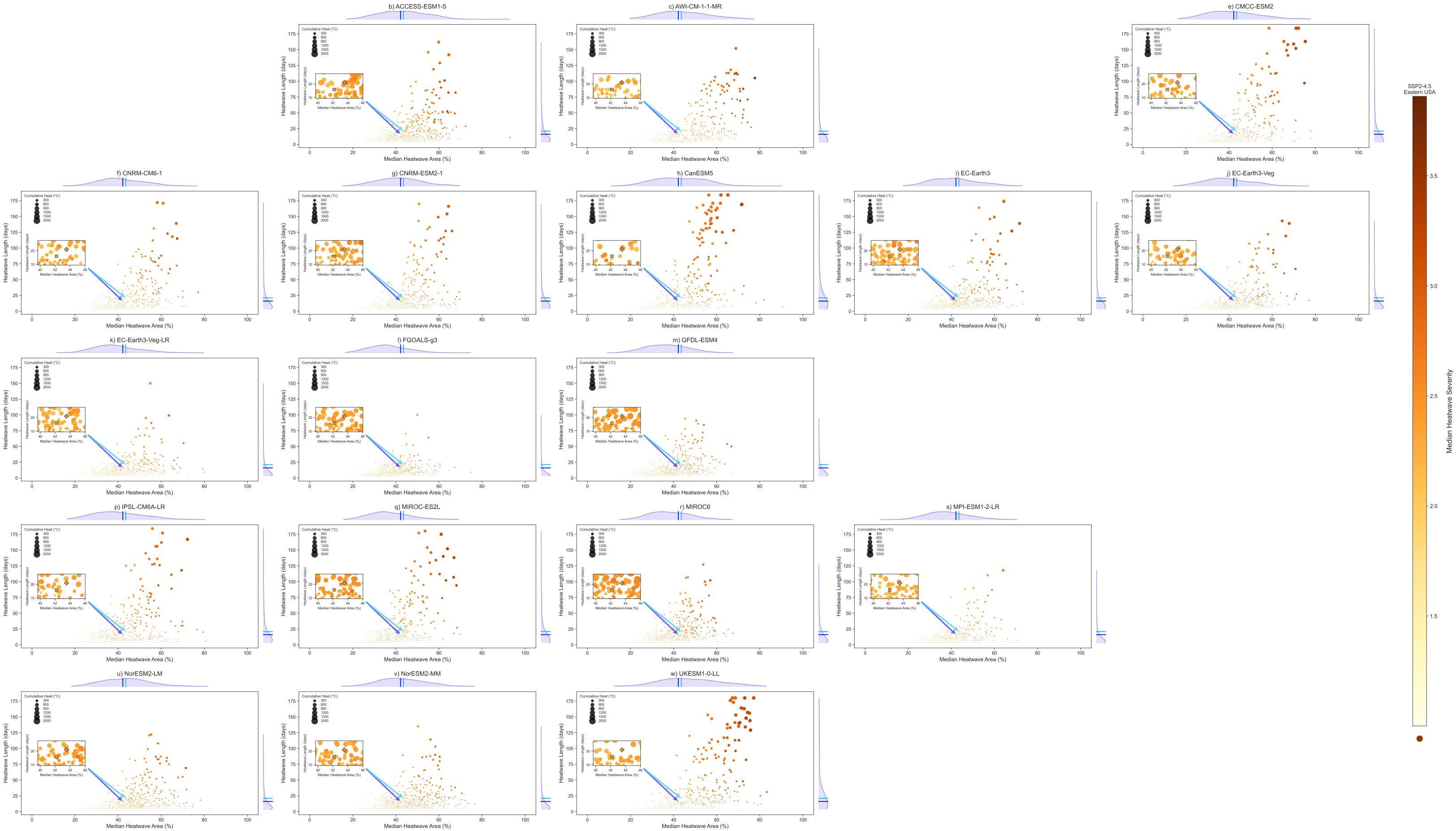
b) ACCESS-ESM1-5
c) AWI-CM-1-1-MR
e) CMCC-ESM2
f) CNRM-CM6-1
g) CNRM-ESM2-1
h) CanESM5
i) EC-Earth3
j) EC-Earth3-Veg
k) EC-Earth3-Veg-LR
l) FGOALS-g3
m) GFDL-ESM4
p) IPSL-CM6A-LR
q) MIROC-ES2L
r) MIROC6
s) MPI-ESM1-2-LR
u) NorESM2-LM
v) NorESM2-MM
w) UKESM1-0-LL
Cumulative Heat (°C)
300
600
900
1200
1500
2000
Heatwave Length (days)
Median Heatwave Area (%)
SSP2-4.5
Eastern USA
Median Heatwave Severity

Supplementary Figure 247: Multi-HW-characteristic figure according to SSP3-7.0 CMIP6 multi-model ensemble (MME) per model for Alaska & Canada (Tx90p) - Each panel shows regional projections according to SSP3-7.0 model runs in the CMIP6 multi-model ensemble (MME) of four combined heatwave characteristics for each projected heatwave in the ensemble between 2020 and 2100. Each dot represents an individual heatwave in the projections, characterized by Median Heatwave Area (%) (x-axis) as latitude-weighted percentage of grid boxes affected by extreme heat, Heatwave Length (days) (y-axis) in days, Median Heatwave Severity (color), and Cumulative Heat (°C) (dot size). The characteristics of the observed event according to the Berkeley Earth observational data (x symbol) and the ERA5 reanalysis data (+ symbol), respectively, are pointed to by an arrow and a figure inset shows a zoom-in on this figure section. The figure inset shows the observed heatwave in the two reference data sets and projections in the indicated section of the respective sub-plot. It facilitates readability of color and size of the reference values and comparison to each other and the neighboring projections. Note that the zoom-in causes the dots to overlap more in the inset than in the main sub-plot. The univariate kernel density estimates along the x- (Median Heatwave Area (%)) and y-axis (Heatwave Length (days)) are on the top and right side of the subplots, respectively, with vertical lines in dark blue (Berkeley Earth) and light blue (ERA5) indicating the observed event. The respective region is indicated above the color bar.

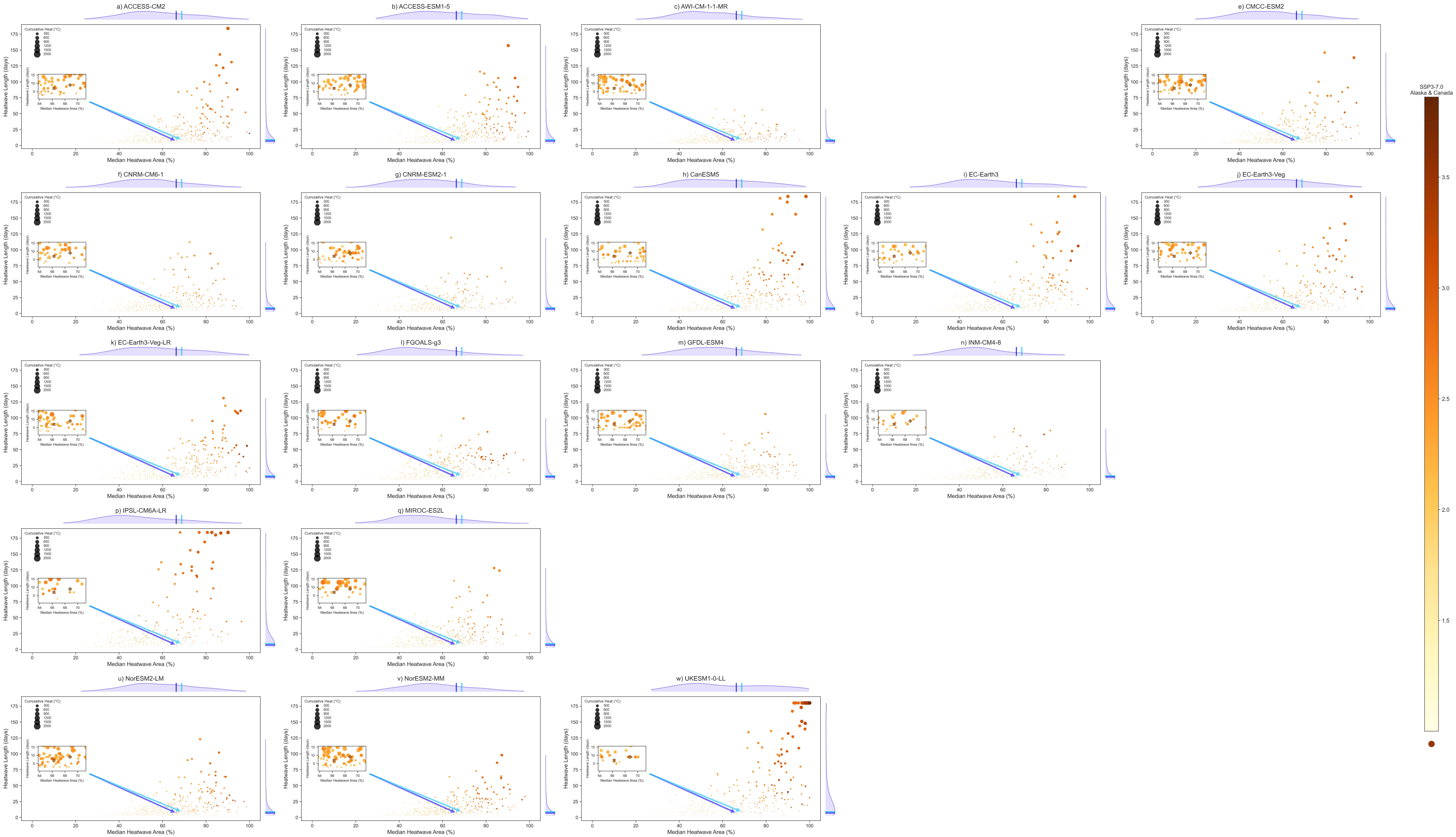

a) ACCESS-CM2
b) ACCESS-ESM1-5
c) AWI-CM-1-1-MR
e) CMCC-ESM2
f) CNRM-CM6-1
g) CNRM-ESM2-1
h) CanESM5
i) EC-Earth3
j) EC-Earth3-Veg
k) EC-Earth3-Veg-LR
l) FGOALS-g3
m) GFDL-ESM4
n) INM-CM4-8
p) IPSL-CM6A-LR
q) MIROC-ES2L
u) NorESM2-LM
v) NorESM2-MM
w) UKESM1-0-LL
Cumulative Heat (°C)
300
600
900
1200
1500
2000
Heatwave Length (days)
Median Heatwave Area (%)
SSP3-7.0
Alaska & Canada
Median Heatwave Severity

Supplementary Figure 248: Multi-HW-characteristic figure according to SSP3-7.0 CMIP6 multi-model ensemble (MME) per model for Argentina, Bolivia, Chile, Paraguay & Uruguay (Tx90p) - Each panel shows regional projections according to SSP3-7.0 model runs in the CMIP6 multi-model ensemble (MME) of four combined heatwave characteristics for each projected heatwave in the ensemble between 2020 and 2100. Each dot represents an individual heatwave in the projections, characterized by Median Heatwave Area (%) (x-axis) as latitude-weighted percentage of grid boxes affected by extreme heat, Heatwave Length (days) (y-axis) in days, Median Heatwave Severity (color), and Cumulative Heat (°C) (dot size). The characteristics of the observed event according to the Berkeley Earth observational data (x symbol) and the ERA5 reanalysis data (+ symbol), respectively, are pointed to by an arrow and a figure inset shows a zoom-in on this figure section. The figure inset shows the observed heatwave in the two reference data sets and projections in the indicated section of the respective sub-plot. It facilitates readability of color and size of the reference values and comparison to each other and the neighboring projections. Note that the zoom-in causes the dots to overlap more in the inset than in the main sub-plot. The univariate kernel density estimates along the x- (Median Heatwave Area (%)) and y-axis (Heatwave Length (days)) are on the top and right side of the subplots, respectively, with vertical lines in dark blue (Berkeley Earth) and light blue (ERA5) indicating the observed event. The respective region is indicated above the color bar.

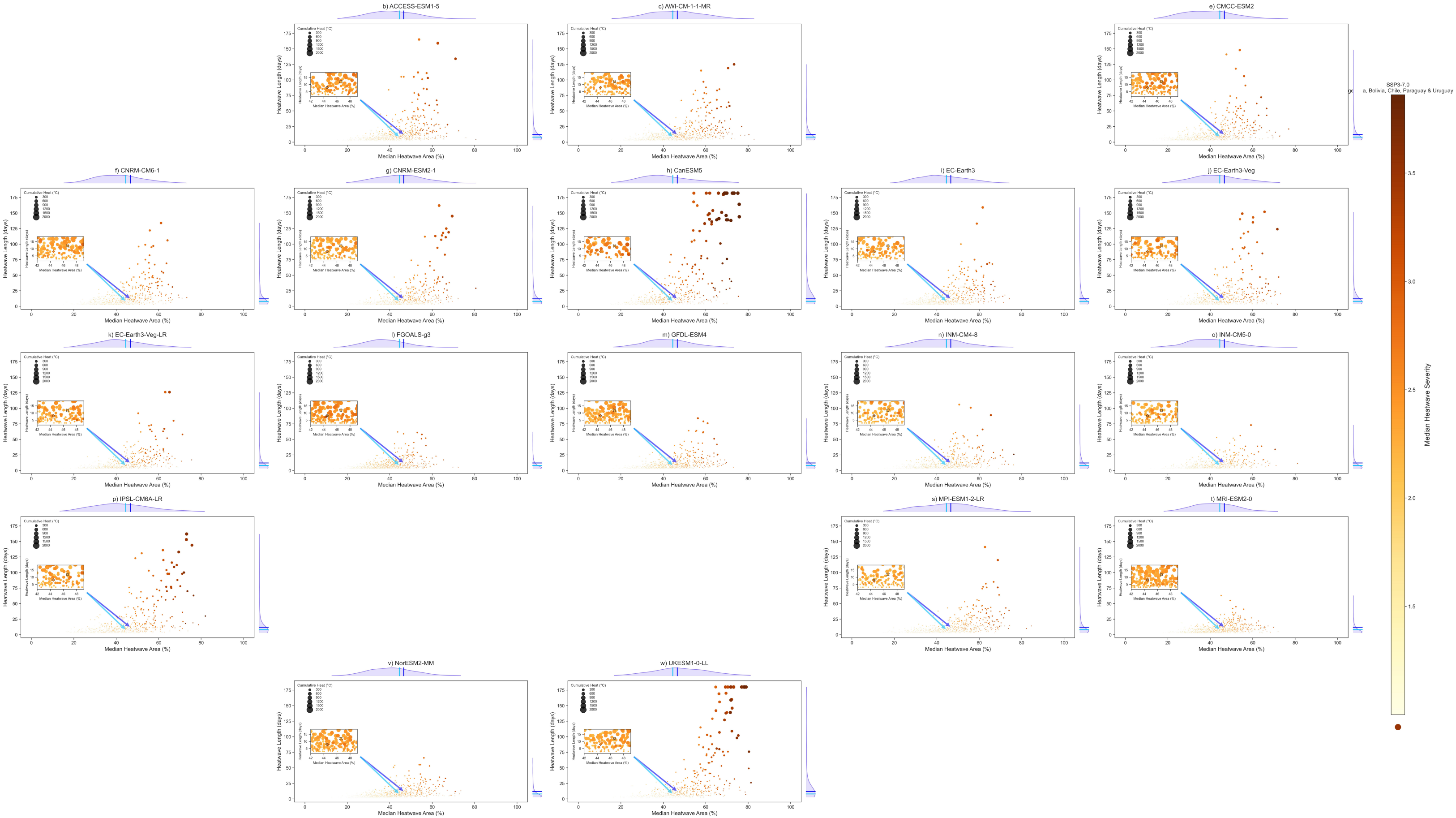

SSP3-7.0
a, Bolivia, Chile, Paraguay & Uruguay
b) ACCESS-ESM1-5
c) AWI-CM-1-1-MR
e) CMCC-ESM2
f) CNRM-CM6-1
g) CNRM-ESM2-1
h) CanESM5
i) EC-Earth3
j) EC-Earth3-Veg
k) EC-Earth3-Veg-LR
l) FGOALS-g3
m) GFDL-ESM4
n) INM-CM4-8
o) INM-CM5-0
p) IPSL-CM6A-LR
s) MPI-ESM1-2-LR
t) MRI-ESM2-0
v) NorESM2-MM
w) UKESM1-0-LL
Cumulative Heat (°C)
300
600
900
1200
1500
2000
Heatwave Length (days)
Median Heatwave Area (%)
Median Heatwave Severity

Supplementary Figure 249: Multi-HW-characteristic figure according to SSP3-7.0 CMIP6 multi-model ensemble (MME) per model for Argentina, Uruguay & Brazil (Tx90p) - Each panel shows regional projections according to SSP3-7.0 model runs in the CMIP6 multi-model ensemble (MME) of four combined heatwave characteristics for each projected heatwave in the ensemble between 2020 and 2100. Each dot represents an individual heatwave in the projections, characterized by Median Heatwave Area (%) (x-axis) as latitude-weighted percentage of grid boxes affected by extreme heat, Heatwave Length (days) (y-axis) in days, Median Heatwave Severity (color), and Cumulative Heat (°C) (dot size). The characteristics of the observed event according to the Berkeley Earth observational data (x symbol) and the ERA5 reanalysis data (+ symbol), respectively, are pointed to by an arrow and a figure inset shows a zoom-in on this figure section. The figure inset shows the observed heatwave in the two reference data sets and projections in the indicated section of the respective sub-plot. It facilitates readability of color and size of the reference values and comparison to each other and the neighboring projections. Note that the zoom-in causes the dots to overlap more in the inset than in the main sub-plot. The univariate kernel density estimates along the x- (Median Heatwave Area (%)) and y-axis (Heatwave Length (days)) are on the top and right side of the subplots, respectively, with vertical lines in dark blue (Berkeley Earth) and light blue (ERA5) indicating the observed event. The respective region is indicated above the color bar.

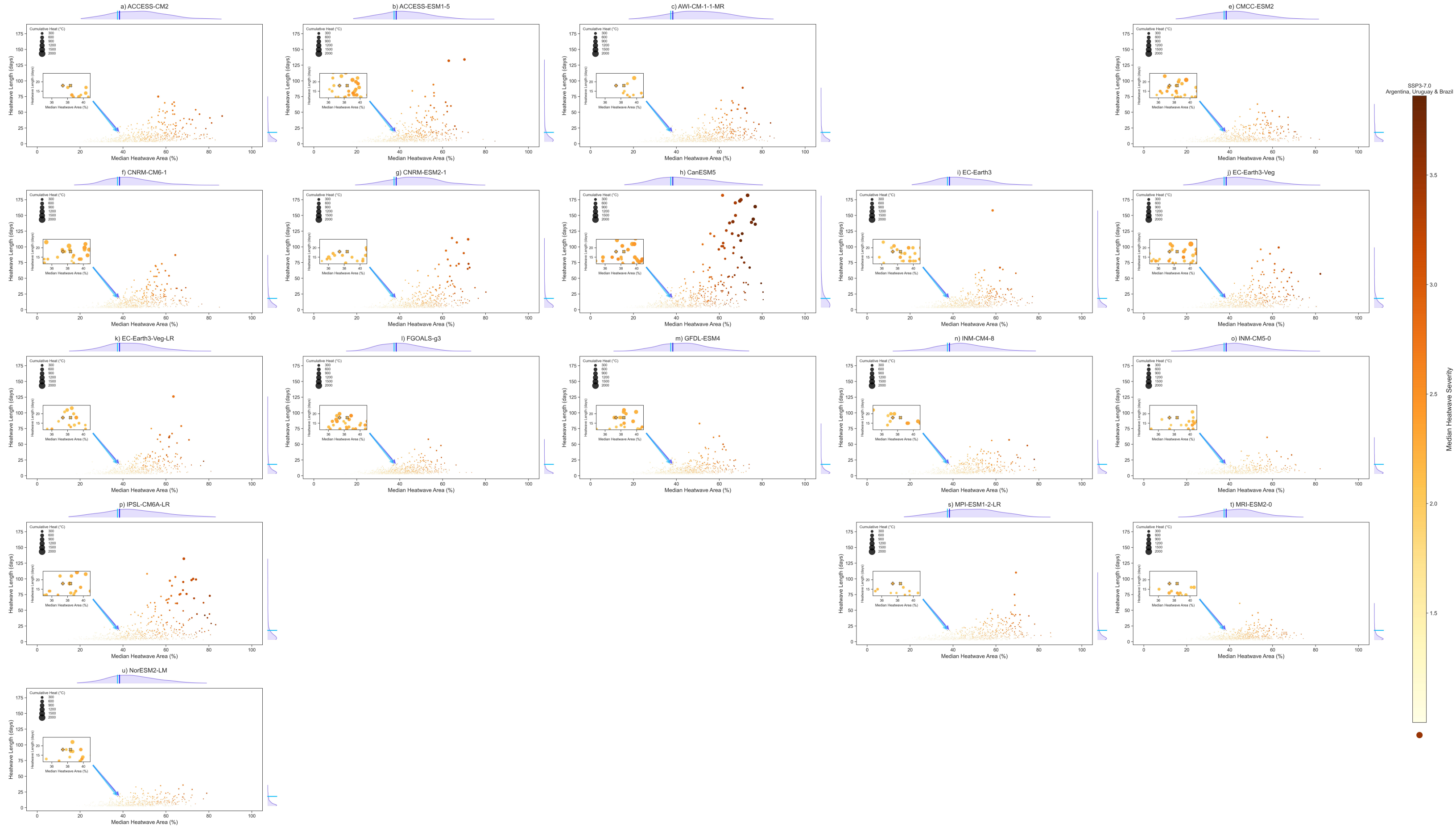

a) ACCESS-CM2
b) ACCESS-ESM1-5
c) AWI-CM-1-1-MR
e) CMCC-ESM2
f) CNRM-CM6-1
g) CNRM-ESM2-1
h) CanESM5
i) EC-Earth3
j) EC-Earth3-Veg
k) EC-Earth3-Veg-LR
l) FGOALS-g3
m) GFDL-ESM4
n) INM-CM4-8
o) INM-CM5-0
p) IPSL-CM6A-LR
s) MPI-ESM1-2-LR
t) MRI-ESM2-0
u) NorESM2-LM
Cumulative Heat (°C)
300
600
900
1200
1500
2000
Heatwave Length (days)
Median Heatwave Area (%)
SSP3-7.0
Argentina, Uruguay & Brazil
Median Heatwave Severity

Supplementary Figure 250: Multi-HW-characteristic figure according to SSP3-7.0 CMIP6 multi-model ensemble (MME) per model for Australia (Tx90p) - Each panel shows regional projections according to SSP3-7.0 model runs in the CMIP6 multi-model ensemble (MME) of four combined heatwave characteristics for each projected heatwave in the ensemble between 2020 and 2100. Each dot represents an individual heatwave in the projections, characterized by Median Heatwave Area (%) (x-axis) as latitude-weighted percentage of grid boxes affected by extreme heat, Heatwave Length (days) (y-axis) in days, Median Heatwave Severity (color), and Cumulative Heat (°C) (dot size). The characteristics of the observed event according to the Berkeley Earth observational data (x symbol) and the ERA5 reanalysis data (+ symbol), respectively, are pointed to by an arrow and a figure inset shows a zoom-in on this figure section. The figure inset shows the observed heatwave in the two reference data sets and projections in the indicated section of the respective sub-plot. It facilitates readability of color and size of the reference values and comparison to each other and the neighboring projections. Note that the zoom-in causes the dots to overlap more in the inset than in the main sub-plot. The univariate kernel density estimates along the x- (Median Heatwave Area (%)) and y-axis (Heatwave Length (days)) are on the top and right side of the subplots, respectively, with vertical lines in dark blue (Berkeley Earth) and light blue (ERA5) indicating the observed event. The respective region is indicated above the color bar.

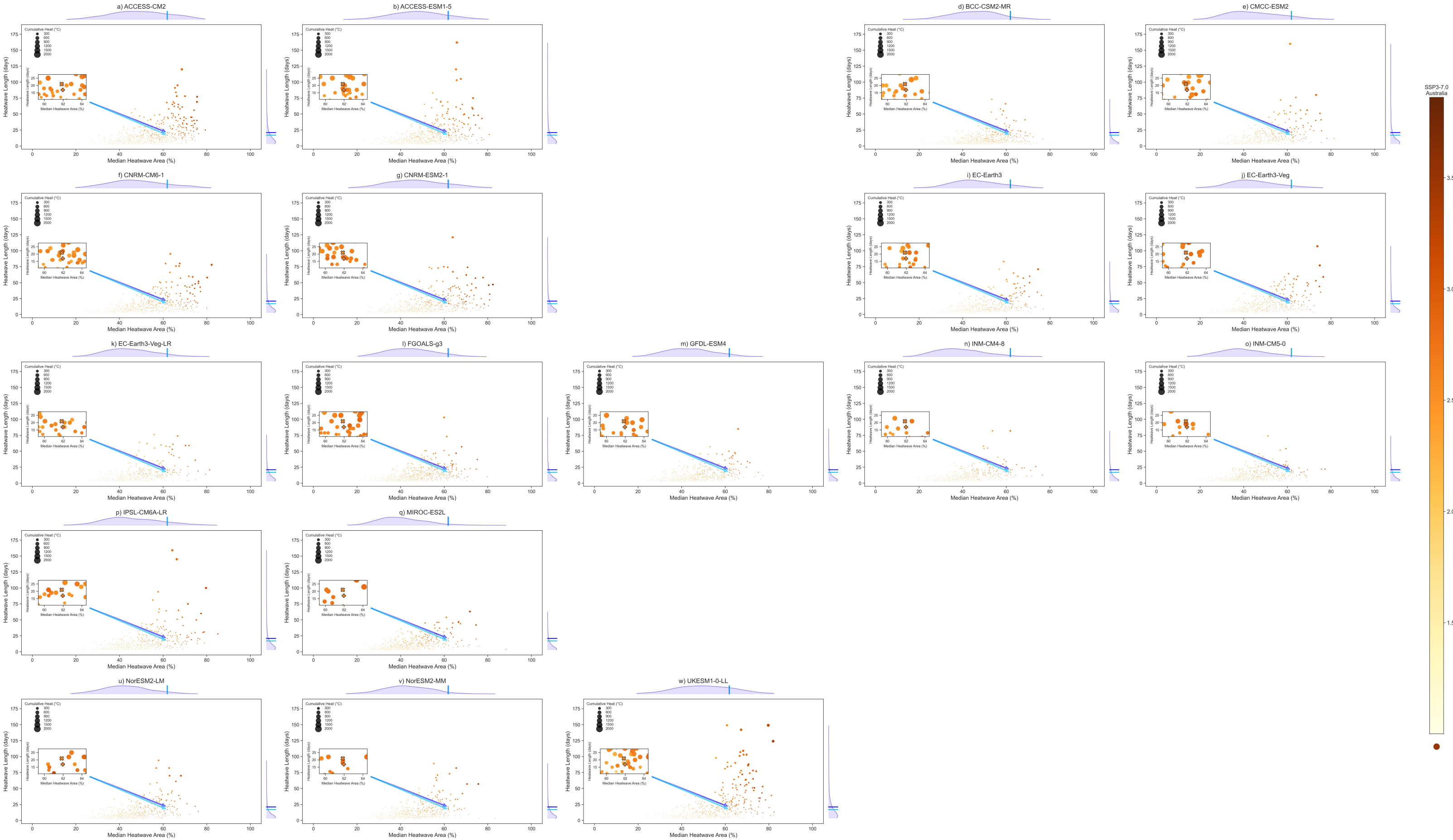

a) ACCESS-CM2
b) ACCESS-ESM1-5
d) BCC-CSM2-MR
e) CMCC-ESM2
f) CNRM-CM6-1
g) CNRM-ESM2-1
i) EC-Earth3
j) EC-Earth3-Veg
k) EC-Earth3-Veg-LR
l) FGOALS-g3
m) GFDL-ESM4
n) INM-CM4-8
o) INM-CM5-0
p) IPSL-CM6A-LR
q) MIROC-ES2L
u) NorESM2-LM
v) NorESM2-MM
w) UKESM1-0-LL
Cumulative Heat (°C)
300
600
900
1200
1500
2000
Heatwave Length (days)
Median Heatwave Area (%)
SSP3-7.0
Australia
Median Heatwave Severity

Supplementary Figure 251: Multi-HW-characteristic figure according to SSP3-7.0 CMIP6 multi-model ensemble (MME) per model for Central Siberia & Central Asia (Tx90p) - Each panel shows regional projections according to SSP3-7.0 model runs in the CMIP6 multi-model ensemble (MME) of four combined heatwave characteristics for each projected heatwave in the ensemble between 2020 and 2100. Each dot represents an individual heatwave in the projections, characterized by Median Heatwave Area (%) (x-axis) as latitude-weighted percentage of grid boxes affected by extreme heat, Heatwave Length (days) (y-axis) in days, Median Heatwave Severity (color), and Cumulative Heat (°C) (dot size). The characteristics of the observed event according to the Berkeley Earth observational data (x symbol) and the ERA5 reanalysis data (+ symbol), respectively, are pointed to by an arrow and a figure inset shows a zoom-in on this figure section. The figure inset shows the observed heatwave in the two reference data sets and projections in the indicated section of the respective sub-plot. It facilitates readability of color and size of the reference values and comparison to each other and the neighboring projections. Note that the zoom-in causes the dots to overlap more in the inset than in the main sub-plot. The univariate kernel density estimates along the x- (Median Heatwave Area (%)) and y-axis (Heatwave Length (days)) are on the top and right side of the subplots, respectively, with vertical lines in dark blue (Berkeley Earth) and light blue (ERA5) indicating the observed event. The respective region is indicated above the color bar.

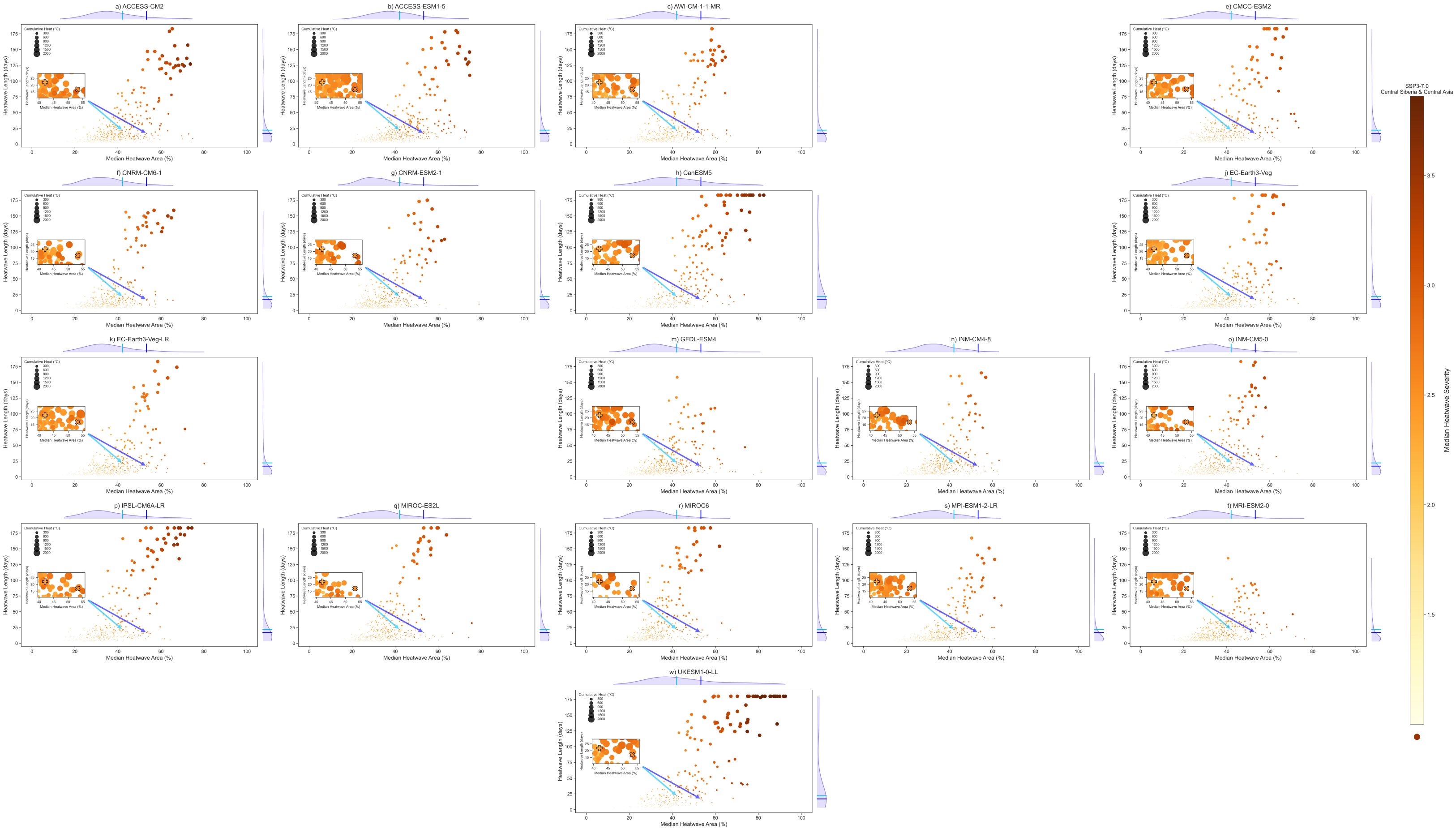
a) ACCESS-CM2
b) ACCESS-ESM1-5
c) AWI-CM-1-1-MR
e) CMCC-ESM2
f) CNRM-CM6-1
g) CNRM-ESM2-1
h) CanESM5
j) EC-Earth3-Veg
k) EC-Earth3-Veg-LR
m) GFDL-ESM4
n) INM-CM4-8
o) INM-CM5-0
p) IPSL-CM6A-LR
q) MIROC-ES2L
r) MIROC6
s) MPI-ESM1-2-LR
t) MRI-ESM2-0
w) UKESM1-0-LL
Heatwave Length (days)
Median Heatwave Area (%)
Cumulative Heat (°C)
300
600
900
1200
1500
2000
SSP3-7.0
Central Siberia & Central Asia
Median Heatwave Severity

Supplementary Figure 252: Multi-HW-characteristic figure according to SSP3-7.0 CMIP6 multi-model ensemble (MME) per model for China (2017) (Tx90p) - Each panel shows regional projections according to SSP3-7.0 model runs in the CMIP6 multi-model ensemble (MME) of four combined heatwave characteristics for each projected heatwave in the ensemble between 2020 and 2100. Each dot represents an individual heatwave in the projections, characterized by Median Heatwave Area (%) (x-axis) as latitude-weighted percentage of grid boxes affected by extreme heat, Heatwave Length (days) (y-axis) in days, Median Heatwave Severity (color), and Cumulative Heat (°C) (dot size). The characteristics of the observed event according to the Berkeley Earth observational data (x symbol) and the ERA5 reanalysis data (+ symbol), respectively, are pointed to by an arrow and a figure inset shows a zoom-in on this figure section. The figure inset shows the observed heatwave in the two reference data sets and projections in the indicated section of the respective sub-plot. It facilitates readability of color and size of the reference values and comparison to each other and the neighboring projections. Note that the zoom-in causes the dots to overlap more in the inset than in the main sub-plot. The univariate kernel density estimates along the x- (Median Heatwave Area (%)) and y-axis (Heatwave Length (days)) are on the top and right side of the subplots, respectively, with vertical lines in dark blue (Berkeley Earth) and light blue (ERA5) indicating the observed event. The respective region is indicated above the color bar.

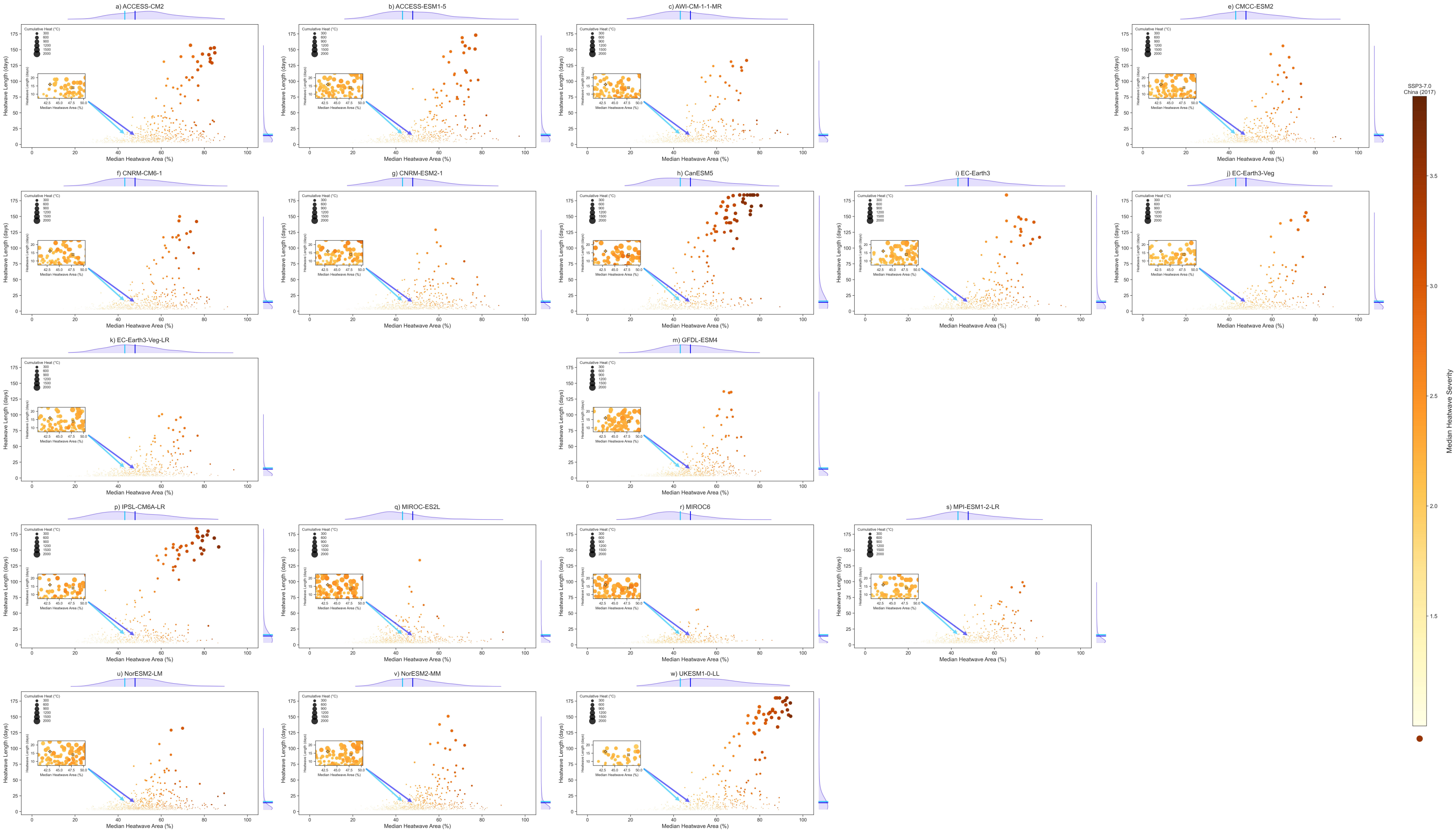

a) ACCESS-CM2
b) ACCESS-ESM1-5
c) AWI-CM-1-1-MR
e) CMCC-ESM2
f) CNRM-CM6-1
g) CNRM-ESM2-1
h) CanESM5
i) EC-Earth3
j) EC-Earth3-Veg
k) EC-Earth3-Veg-LR
m) GFDL-ESM4
p) IPSL-CM6A-LR
q) MIROC-ES2L
r) MIROC6
s) MPI-ESM1-2-LR
u) NorESM2-LM
v) NorESM2-MM
w) UKESM1-0-LL
Cumulative Heat (°C)
300
600
900
1200
1500
2000
Heatwave Length (days)
Median Heatwave Area (%)
SSP3-7.0
China (2017)
Median Heatwave Severity

Supplementary Figure 253: Multi-HW-characteristic figure according to SSP3-7.0 CMIP6 multi-model ensemble (MME) per model for China (Tx90p) - Each panel shows regional projections according to SSP3-7.0 model runs in the CMIP6 multi-model ensemble (MME) of four combined heatwave characteristics for each projected heatwave in the ensemble between 2020 and 2100. Each dot represents an individual heatwave in the projections, characterized by Median Heatwave Area (%) (x-axis) as latitude-weighted percentage of grid boxes affected by extreme heat, Heatwave Length (days) (y-axis) in days, Median Heatwave Severity (color), and Cumulative Heat (°C) (dot size). The characteristics of the observed event according to the Berkeley Earth observational data (x symbol) and the ERA5 reanalysis data (+ symbol), respectively, are pointed to by an arrow and a figure inset shows a zoom-in on this figure section. The figure inset shows the observed heatwave in the two reference data sets and projections in the indicated section of the respective sub-plot. It facilitates readability of color and size of the reference values and comparison to each other and the neighboring projections. Note that the zoom-in causes the dots to overlap more in the inset than in the main sub-plot. The univariate kernel density estimates along the x- (Median Heatwave Area (%)) and y-axis (Heatwave Length (days)) are on the top and right side of the subplots, respectively, with vertical lines in dark blue (Berkeley Earth) and light blue (ERA5) indicating the observed event. The respective region is indicated above the color bar.

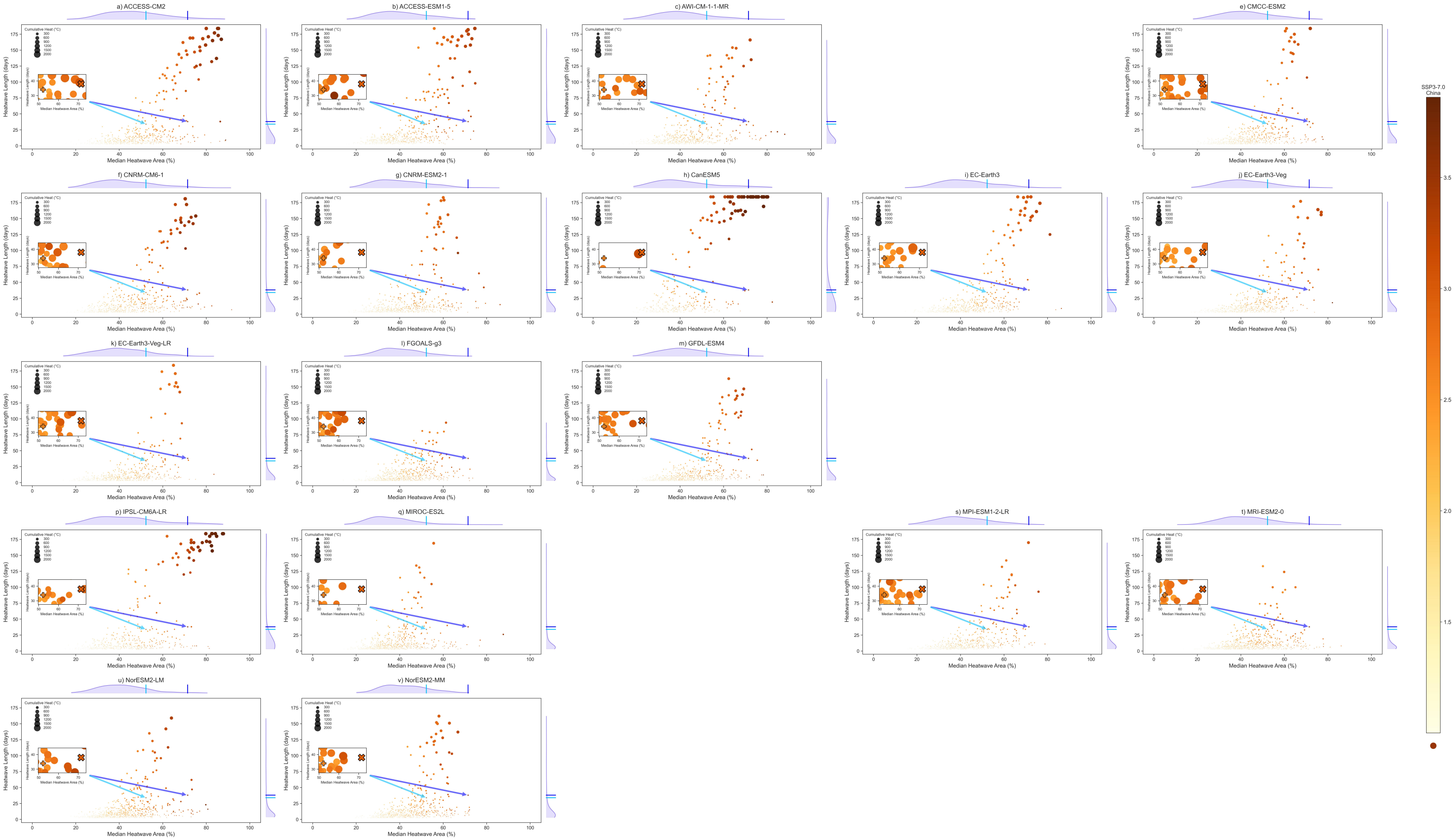

a) ACCESS-CM2
b) ACCESS-ESM1-5
c) AWI-CM-1-1-MR
e) CMCC-ESM2
f) CNRM-CM6-1
g) CNRM-ESM2-1
h) CanESM5
i) EC-Earth3
j) EC-Earth3-Veg
k) EC-Earth3-Veg-LR
l) FGOALS-g3
m) GFDL-ESM4
p) IPSL-CM6A-LR
q) MIROC-ES2L
s) MPI-ESM1-2-LR
t) MRI-ESM2-0
u) NorESM2-LM
v) NorESM2-MM
Cumulative Heat (°C)
300
600
900
1200
1500
2000
Heatwave Length (days)
Median Heatwave Area (%)
SSP3-7.0
China
Median Heatwave Severity
1.5
2.0
2.5
3.0
3.5

Supplementary Figure 254: Multi-HW-characteristic figure according to SSP3-7.0 CMIP6 multi-model ensemble (MME) per model for Central & Eastern Europe (Tx90p) - Each panel shows regional projections according to SSP3-7.0 model runs in the CMIP6 multi-model ensemble (MME) of four combined heatwave characteristics for each projected heatwave in the ensemble between 2020 and 2100. Each dot represents an individual heatwave in the projections, characterized by Median Heatwave Area (%) (x-axis) as latitude-weighted percentage of grid boxes affected by extreme heat, Heatwave Length (days) (y-axis) in days, Median Heatwave Severity (color), and Cumulative Heat (°C) (dot size). The characteristics of the observed event according to the Berkeley Earth observational data (x symbol) and the ERA5 reanalysis data (+ symbol), respectively, are pointed to by an arrow and a figure inset shows a zoom-in on this figure section. The figure inset shows the observed heatwave in the two reference data sets and projections in the indicated section of the respective sub-plot. It facilitates readability of color and size of the reference values and comparison to each other and the neighboring projections. Note that the zoom-in causes the dots to overlap more in the inset than in the main sub-plot. The univariate kernel density estimates along the x- (Median Heatwave Area (%)) and y-axis (Heatwave Length (days)) are on the top and right side of the subplots, respectively, with vertical lines in dark blue (Berkeley Earth) and light blue (ERA5) indicating the observed event. The respective region is indicated above the color bar.

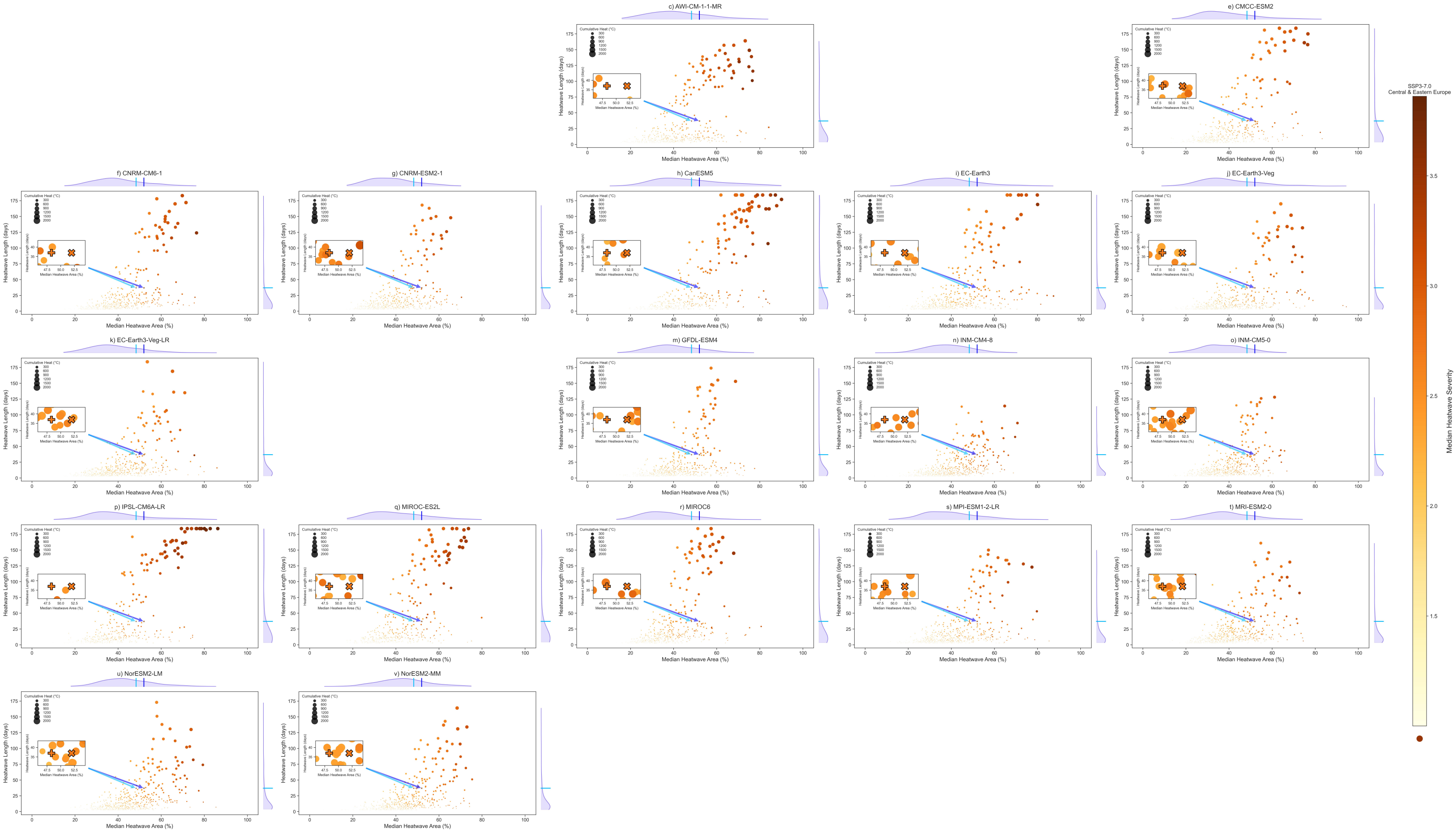

c) AWI-CM-1-1-MR
e) CMCC-ESM2
f) CNRM-CM6-1
g) CNRM-ESM2-1
h) CanESM5
i) EC-Earth3
j) EC-Earth3-Veg
k) EC-Earth3-Veg-LR
m) GFDL-ESM4
n) INM-CM4-8
o) INM-CM5-0
p) IPSL-CM6A-LR
q) MIROC-ES2L
r) MIROC6
s) MPI-ESM1-2-LR
t) MRI-ESM2-0
u) NorESM2-LM
v) NorESM2-MM
Cumulative Heat (°C)
300
600
900
1200
1500
2000
Heatwave Length (days)
Median Heatwave Area (%)
SSP3-7.0
Central & Eastern Europe
Median Heatwave Severity

Supplementary Figure 255: Multi-HW-characteristic figure according to SSP3-7.0 CMIP6 multi-model ensemble (MME) per model for Europe (Tx90p) - Each panel shows regional projections according to SSP3-7.0 model runs in the CMIP6 multi-model ensemble (MME) of four combined heatwave characteristics for each projected heatwave in the ensemble between 2020 and 2100. Each dot represents an individual heatwave in the projections, characterized by Median Heatwave Area (%) (x-axis) as latitude-weighted percentage of grid boxes affected by extreme heat, Heatwave Length (days) (y-axis) in days, Median Heatwave Severity (color), and Cumulative Heat (°C) (dot size). The characteristics of the observed event according to the Berkeley Earth observational data (x symbol) and the ERA5 reanalysis data (+ symbol), respectively, are pointed to by an arrow and a figure inset shows a zoom-in on this figure section. The figure inset shows the observed heatwave in the two reference data sets and projections in the indicated section of the respective sub-plot. It facilitates readability of color and size of the reference values and comparison to each other and the neighboring projections. Note that the zoom-in causes the dots to overlap more in the inset than in the main sub-plot. The univariate kernel density estimates along the x- (Median Heatwave Area (%)) and y-axis (Heatwave Length (days)) are on the top and right side of the subplots, respectively, with vertical lines in dark blue (Berkeley Earth) and light blue (ERA5) indicating the observed event. The respective region is indicated above the color bar.

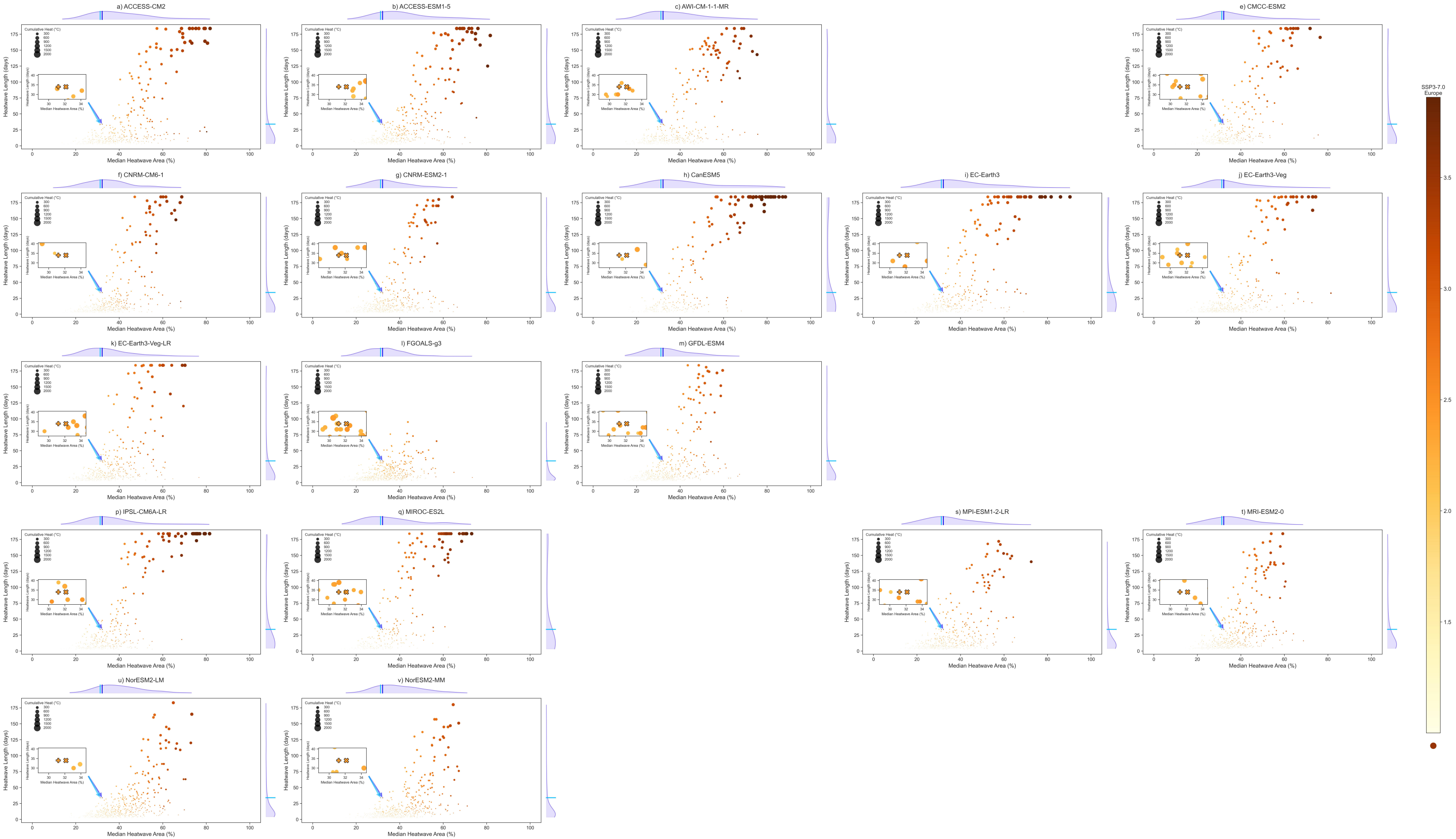
a) ACCESS-CM2
b) ACCESS-ESM1-5
c) AWI-CM-1-1-MR
e) CMCC-ESM2
f) CNRM-CM6-1
g) CNRM-ESM2-1
h) CanESM5
i) EC-Earth3
j) EC-Earth3-Veg
k) EC-Earth3-Veg-LR
l) FGOALS-g3
m) GFDL-ESM4
p) IPSL-CM6A-LR
q) MIROC-ES2L
s) MPI-ESM1-2-LR
t) MRI-ESM2-0
u) NorESM2-LM
v) NorESM2-MM
Cumulative Heat (°C)
300
600
900
1200
1500
2000
Heatwave Length (days)
Median Heatwave Area (%)
SSP3-7.0
Europe
Median Heatwave Severity

Supplementary Figure 256: Multi-HW-characteristic figure according to SSP3-7.0 CMIP6 multi-model ensemble (MME) per model for India & Middle East (Tx90p) - Each panel shows regional projections according to SSP3-7.0 model runs in the CMIP6 multi-model ensemble (MME) of four combined heatwave characteristics for each projected heatwave in the ensemble between 2020 and 2100. Each dot represents an individual heatwave in the projections, characterized by Median Heatwave Area (%) (x-axis) as latitude-weighted percentage of grid boxes affected by extreme heat, Heatwave Length (days) (y-axis) in days, Median Heatwave Severity (color), and Cumulative Heat (°C) (dot size). The characteristics of the observed event according to the Berkeley Earth observational data (x symbol) and the ERA5 reanalysis data (+ symbol), respectively, are pointed to by an arrow and a figure inset shows a zoom-in on this figure section. The figure inset shows the observed heatwave in the two reference data sets and projections in the indicated section of the respective sub-plot. It facilitates readability of color and size of the reference values and comparison to each other and the neighboring projections. Note that the zoom-in causes the dots to overlap more in the inset than in the main sub-plot. The univariate kernel density estimates along the x- (Median Heatwave Area (%)) and y-axis (Heatwave Length (days)) are on the top and right side of the subplots, respectively, with vertical lines in dark blue (Berkeley Earth) and light blue (ERA5) indicating the observed event. The respective region is indicated above the color bar.

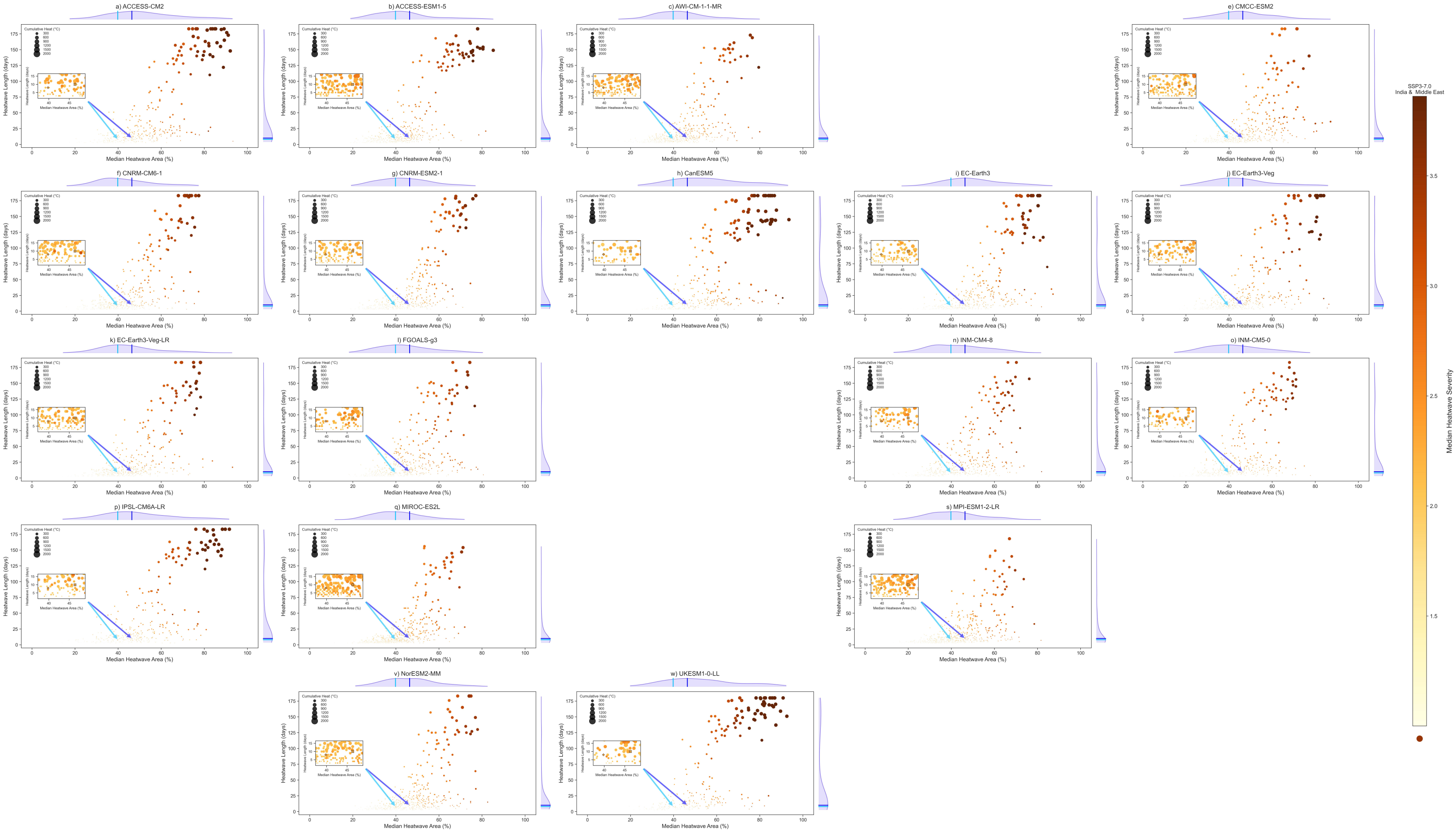

a) ACCESS-CM2
b) ACCESS-ESM1-5
c) AWI-CM-1-1-MR
e) CMCC-ESM2
f) CNRM-CM6-1
g) CNRM-ESM2-1
h) CanESM5
i) EC-Earth3
j) EC-Earth3-Veg
k) EC-Earth3-Veg-LR
l) FGOALS-g3
n) INM-CM4-8
o) INM-CM5-0
p) IPSL-CM6A-LR
q) MIROC-ES2L
s) MPI-ESM1-2-LR
v) NorESM2-MM
w) UKESM1-0-LL
Cumulative Heat (°C)
300
600
900
1200
1500
2000
Heatwave Length (days)
Median Heatwave Area (%)
SSP3-7.0
India & Middle East
Median Heatwave Severity

Supplementary Figure 257: Multi-HW-characteristic figure according to SSP3-7.0 CMIP6 multi-model ensemble (MME) per model for India & Pakistan (Tx90p) - Each panel shows regional projections according to SSP3-7.0 model runs in the CMIP6 multi-model ensemble (MME) of four combined heatwave characteristics for each projected heatwave in the ensemble between 2020 and 2100. Each dot represents an individual heatwave in the projections, characterized by Median Heatwave Area (%) (x-axis) as latitude-weighted percentage of grid boxes affected by extreme heat, Heatwave Length (days) (y-axis) in days, Median Heatwave Severity (color), and Cumulative Heat (°C) (dot size). The characteristics of the observed event according to the Berkeley Earth observational data (x symbol) and the ERA5 reanalysis data (+ symbol), respectively, are pointed to by an arrow and a figure inset shows a zoom-in on this figure section. The figure inset shows the observed heatwave in the two reference data sets and projections in the indicated section of the respective sub-plot. It facilitates readability of color and size of the reference values and comparison to each other and the neighboring projections. Note that the zoom-in causes the dots to overlap more in the inset than in the main sub-plot. The univariate kernel density estimates along the x- (Median Heatwave Area (%)) and y-axis (Heatwave Length (days)) are on the top and right side of the subplots, respectively, with vertical lines in dark blue (Berkeley Earth) and light blue (ERA5) indicating the observed event. The respective region is indicated above the color bar.

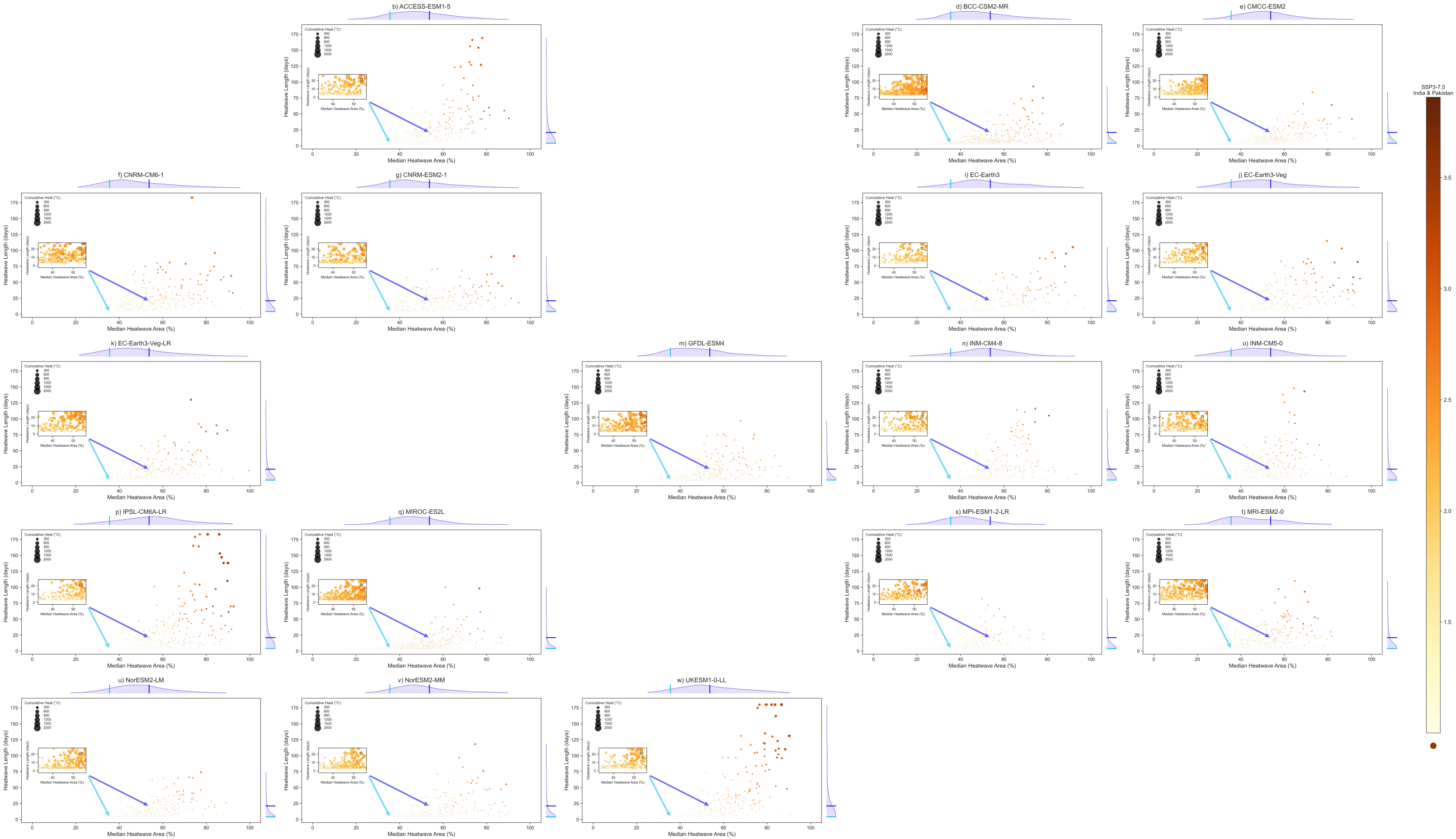

b) ACCESS-ESM1-5
d) BCC-CSM2-MR
e) CMCC-ESM2
f) CNRM-CM6-1
g) CNRM-ESM2-1
i) EC-Earth3
j) EC-Earth3-Veg
k) EC-Earth3-Veg-LR
m) GFDL-ESM4
n) INM-CM4-8
o) INM-CM5-0
p) IPSL-CM6A-LR
q) MIROC-ES2L
s) MPI-ESM1-2-LR
t) MRI-ESM2-0
u) NorESM2-LM
v) NorESM2-MM
w) UKESM1-0-LL
Cumulative Heat (°C)
300
600
900
1200
1500
2000
Heatwave Length (days)
Median Heatwave Area (%)
SSP3-7.0
India & Pakistan
Median Heatwave Severity
1.5
2.0
2.5
3.0
3.5

Supplementary Figure 258: Multi-HW-characteristic figure according to SSP3-7.0 CMIP6 multi-model ensemble (MME) per model for Middle East (Tx90p) - Each panel shows regional projections according to SSP3-7.0 model runs in the CMIP6 multi-model ensemble (MME) of four combined heatwave characteristics for each projected heatwave in the ensemble between 2020 and 2100. Each dot represents an individual heatwave in the projections, characterized by Median Heatwave Area (%) (x-axis) as latitude-weighted percentage of grid boxes affected by extreme heat, Heatwave Length (days) (y-axis) in days, Median Heatwave Severity (color), and Cumulative Heat (°C) (dot size). The characteristics of the observed event according to the Berkeley Earth observational data (x symbol) and the ERA5 reanalysis data (+ symbol), respectively, are pointed to by an arrow and a figure inset shows a zoom-in on this figure section. The figure inset shows the observed heatwave in the two reference data sets and projections in the indicated section of the respective sub-plot. It facilitates readability of color and size of the reference values and comparison to each other and the neighboring projections. Note that the zoom-in causes the dots to overlap more in the inset than in the main sub-plot. The univariate kernel density estimates along the x- (Median Heatwave Area (%)) and y-axis (Heatwave Length (days)) are on the top and right side of the subplots, respectively, with vertical lines in dark blue (Berkeley Earth) and light blue (ERA5) indicating the observed event. The respective region is indicated above the color bar.

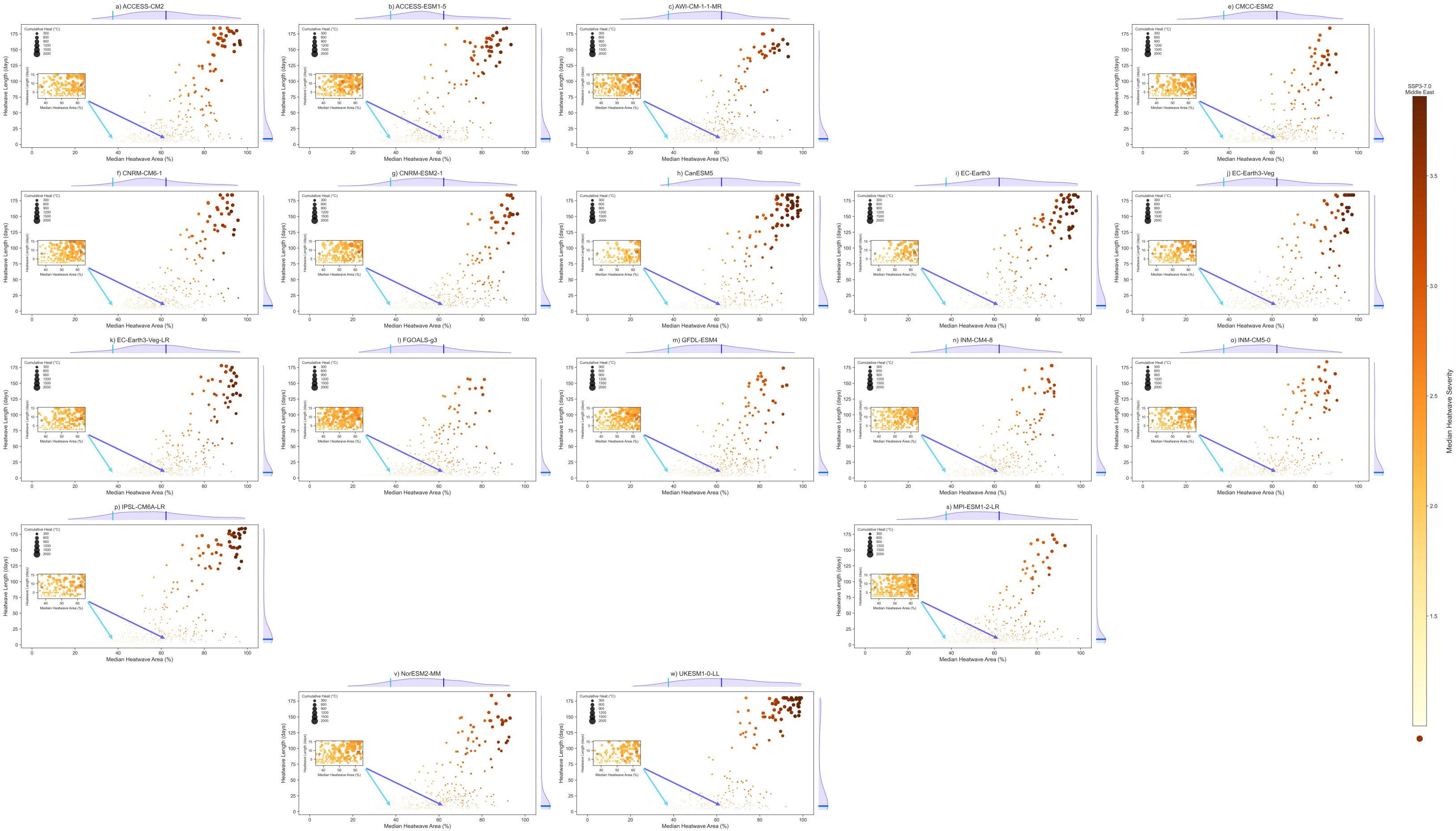
a) ACCESS-CM2
b) ACCESS-ESM1-5
c) AWI-CM-1-1-MR
e) CMCC-ESM2
f) CNRM-CM6-1
g) CNRM-ESM2-1
h) CanESM5
i) EC-Earth3
j) EC-Earth3-Veg
k) EC-Earth3-Veg-LR
l) FGOALS-g3
m) GFDL-ESM4
n) INM-CM4-8
o) INM-CM5-0
p) IPSL-CM6A-LR
s) MPI-ESM1-2-LR
v) NorESM2-MM
w) UKESM1-0-LL
Cumulative Heat (°C)
300
600
900
1200
1500
2000
Heatwave Length (days)
Median Heatwave Area (%)
SSP3-7.0
Middle East
Median Heatwave Severity

Supplementary Figure 259: Multi-HW-characteristic figure according to SSP3-7.0 CMIP6 multi-model ensemble (MME) per model for Northeast Siberia (Tx90p) - Each panel shows regional projections according to SSP3-7.0 model runs in the CMIP6 multi-model ensemble (MME) of four combined heatwave characteristics for each projected heatwave in the ensemble between 2020 and 2100. Each dot represents an individual heatwave in the projections, characterized by Median Heatwave Area (%) (x-axis) as latitude-weighted percentage of grid boxes affected by extreme heat, Heatwave Length (days) (y-axis) in days, Median Heatwave Severity (color), and Cumulative Heat (°C) (dot size). The characteristics of the observed event according to the Berkeley Earth observational data (x symbol) and the ERA5 reanalysis data (+ symbol), respectively, are pointed to by an arrow and a figure inset shows a zoom-in on this figure section. The figure inset shows the observed heatwave in the two reference data sets and projections in the indicated section of the respective sub-plot. It facilitates readability of color and size of the reference values and comparison to each other and the neighboring projections. Note that the zoom-in causes the dots to overlap more in the inset than in the main sub-plot. The univariate kernel density estimates along the x- (Median Heatwave Area (%)) and y-axis (Heatwave Length (days)) are on the top and right side of the subplots, respectively, with vertical lines in dark blue (Berkeley Earth) and light blue (ERA5) indicating the observed event. The respective region is indicated above the color bar.

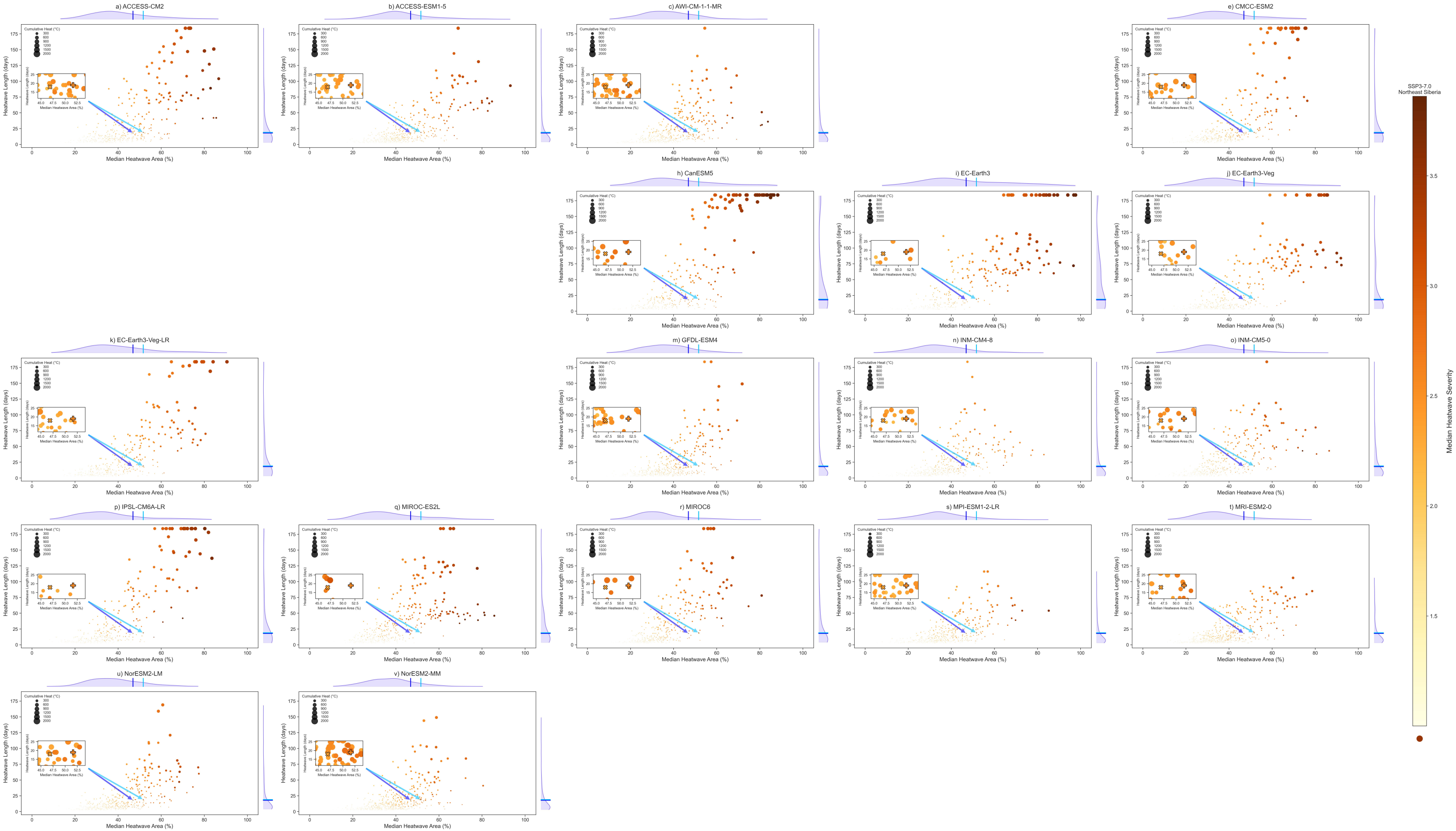

a) ACCESS-CM2
b) ACCESS-ESM1-5
c) AWI-CM-1-1-MR
e) CMCC-ESM2
h) CanESM5
i) EC-Earth3
j) EC-Earth3-Veg
k) EC-Earth3-Veg-LR
m) GFDL-ESM4
n) INM-CM4-8
o) INM-CM5-0
p) IPSL-CM6A-LR
q) MIROC-ES2L
r) MIROC6
s) MPI-ESM1-2-LR
t) MRI-ESM2-0
u) NorESM2-LM
v) NorESM2-MM
Heatwave Length (days)
Median Heatwave Area (%)
Cumulative Heat (°C)
300
600
900
1200
1500
2000
SSP3-7.0
Northeast Siberia
Median Heatwave Severity

Supplementary Figure 260: Multi-HW-characteristic figure according to SSP3-7.0 CMIP6 multi-model ensemble (MME) per model for Northern Africa & Middle East (Tx90p) - Each panel shows regional projections according to SSP3-7.0 model runs in the CMIP6 multi-model ensemble (MME) of four combined heatwave characteristics for each projected heatwave in the ensemble between 2020 and 2100. Each dot represents an individual heatwave in the projections, characterized by Median Heatwave Area (%) (x-axis) as latitude-weighted percentage of grid boxes affected by extreme heat, Heatwave Length (days) (y-axis) in days, Median Heatwave Severity (color), and Cumulative Heat (°C) (dot size). The characteristics of the observed event according to the Berkeley Earth observational data (x symbol) and the ERA5 reanalysis data (+ symbol), respectively, are pointed to by an arrow and a figure inset shows a zoom-in on this figure section. The figure inset shows the observed heatwave in the two reference data sets and projections in the indicated section of the respective sub-plot. It facilitates readability of color and size of the reference values and comparison to each other and the neighboring projections. Note that the zoom-in causes the dots to overlap more in the inset than in the main sub-plot. The univariate kernel density estimates along the x- (Median Heatwave Area (%)) and y-axis (Heatwave Length (days)) are on the top and right side of the subplots, respectively, with vertical lines in dark blue (Berkeley Earth) and light blue (ERA5) indicating the observed event. The respective region is indicated above the color bar.

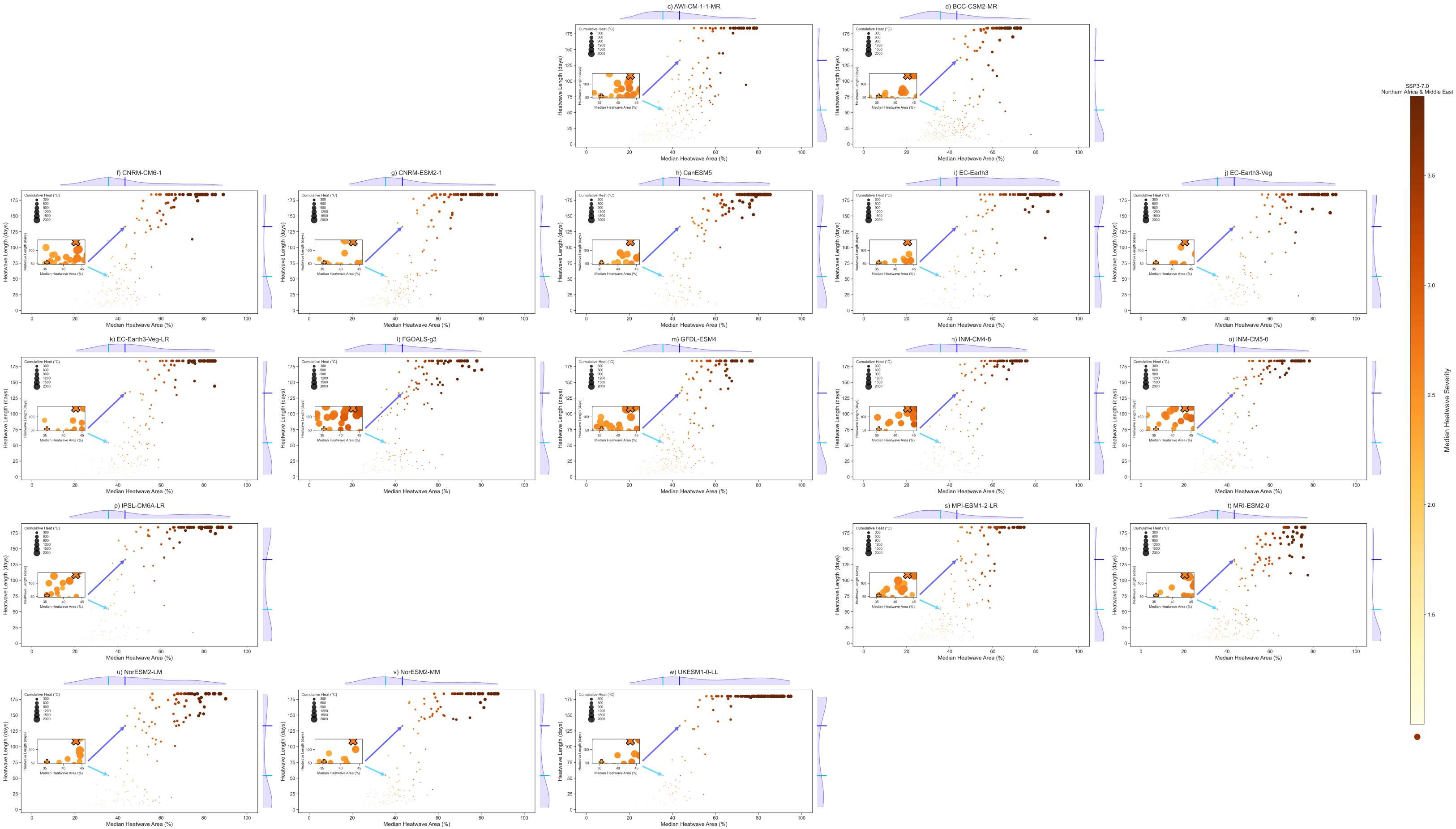

c) AWI-CM-1-1-MR
d) BCC-CSM2-MR
f) CNRM-CM6-1
g) CNRM-ESM2-1
h) CanESM5
i) EC-Earth3
j) EC-Earth3-Veg
k) EC-Earth3-Veg-LR
l) FGOALS-g3
m) GFDL-ESM4
n) INM-CM4-8
o) INM-CM5-0
p) IPSL-CM6A-LR
s) MPI-ESM1-2-LR
t) MRI-ESM2-0
u) NorESM2-LM
v) NorESM2-MM
w) UKESM1-0-LL
Cumulative Heat (°C)
300
600
900
1200
1500
2000
Heatwave Length (days)
Median Heatwave Area (%)
SSP3-7.0
Northern Africa & Middle East
Median Heatwave Severity

Supplementary Figure 261: Multi-HW-characteristic figure according to SSP3-7.0 CMIP6 multi-model ensemble (MME) per model for Pacific Northwest USA & Canada (Tx90p) - Each panel shows regional projections according to SSP3-7.0 model runs in the CMIP6 multi-model ensemble (MME) of four combined heatwave characteristics for each projected heatwave in the ensemble between 2020 and 2100. Each dot represents an individual heatwave in the projections, characterized by Median Heatwave Area (%) (x-axis) as latitude-weighted percentage of grid boxes affected by extreme heat, Heatwave Length (days) (y-axis) in days, Median Heatwave Severity (color), and Cumulative Heat (°C) (dot size). The characteristics of the observed event according to the Berkeley Earth observational data (x symbol) and the ERA5 reanalysis data (+ symbol), respectively, are pointed to by an arrow and a figure inset shows a zoom-in on this figure section. The figure inset shows the observed heatwave in the two reference data sets and projections in the indicated section of the respective sub-plot. It facilitates readability of color and size of the reference values and comparison to each other and the neighboring projections. Note that the zoom-in causes the dots to overlap more in the inset than in the main sub-plot. The univariate kernel density estimates along the x- (Median Heatwave Area (%)) and y-axis (Heatwave Length (days)) are on the top and right side of the subplots, respectively, with vertical lines in dark blue (Berkeley Earth) and light blue (ERA5) indicating the observed event. The respective region is indicated above the color bar.

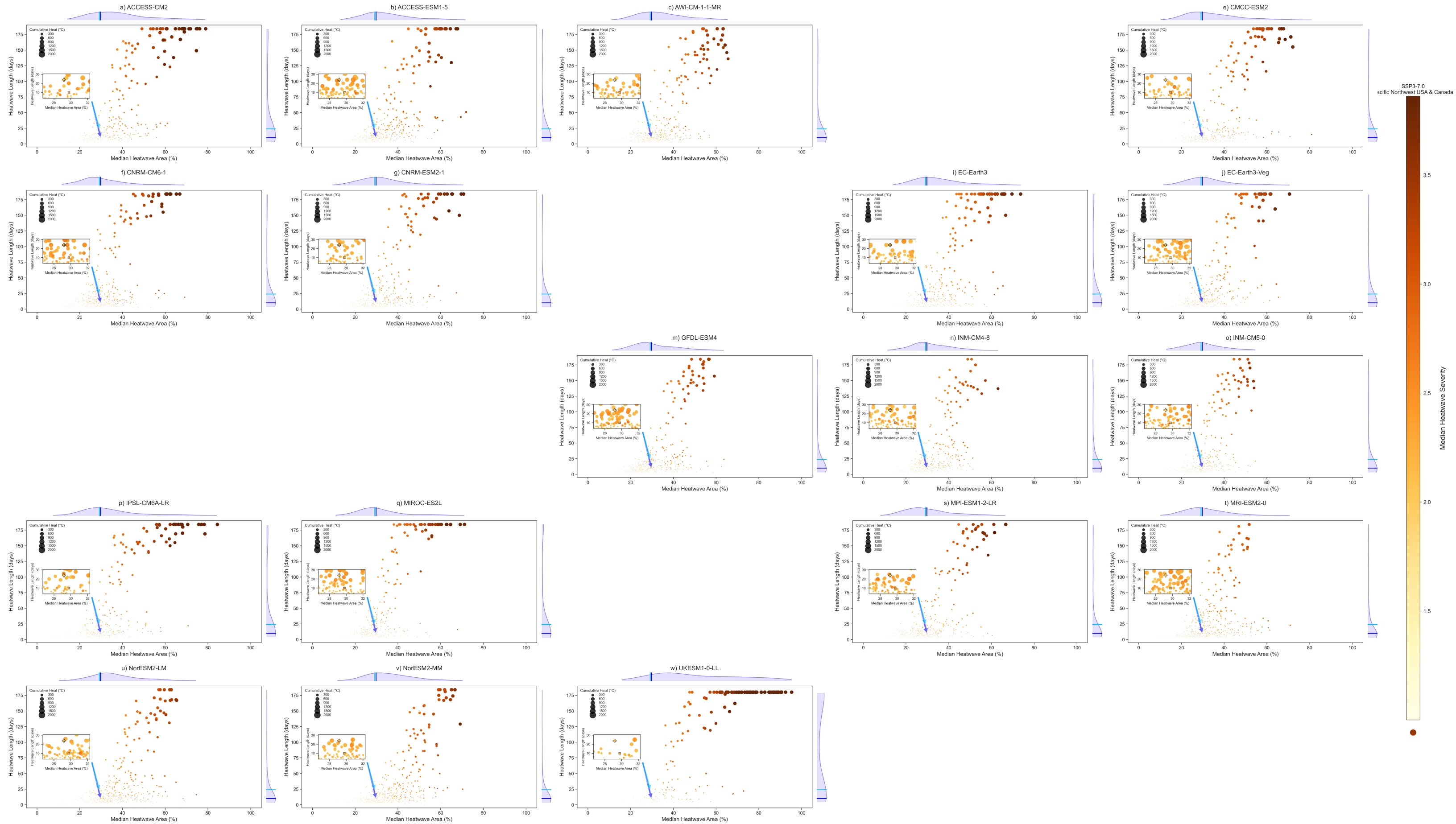
a) ACCESS-CM2
b) ACCESS-ESM1-5
c) AWI-CM-1-1-MR
e) CMCC-ESM2
f) CNRM-CM6-1
g) CNRM-ESM2-1
i) EC-Earth3
j) EC-Earth3-Veg
m) GFDL-ESM4
n) INM-CM4-8
o) INM-CM5-0
p) IPSL-CM6A-LR
q) MIROC-ES2L
s) MPI-ESM1-2-LR
t) MRI-ESM2-0
u) NorESM2-LM
v) NorESM2-MM
w) UKESM1-0-LL
Cumulative Heat (°C)
300
600
900
1200
1500
2000
Heatwave Length (days)
Median Heatwave Area (%)
SSP3-7.0
acific Northwest USA & Canada
Median Heatwave Severity
1.5
2.0
2.5
3.0
3.5

Supplementary Figure 262: Multi-HW-characteristic figure according to SSP3-7.0 CMIP6 multi-model ensemble (MME) per model for Russia (Tx90p) - Each panel shows regional projections according to SSP3-7.0 model runs in the CMIP6 multi-model ensemble (MME) of four combined heatwave characteristics for each projected heatwave in the ensemble between 2020 and 2100. Each dot represents an individual heatwave in the projections, characterized by Median Heatwave Area (%) (x-axis) as latitude-weighted percentage of grid boxes affected by extreme heat, Heatwave Length (days) (y-axis) in days, Median Heatwave Severity (color), and Cumulative Heat (°C) (dot size). The characteristics of the observed event according to the Berkeley Earth observational data (x symbol) and the ERA5 reanalysis data (+ symbol), respectively, are pointed to by an arrow and a figure inset shows a zoom-in on this figure section. The figure inset shows the observed heatwave in the two reference data sets and projections in the indicated section of the respective sub-plot. It facilitates readability of color and size of the reference values and comparison to each other and the neighboring projections. Note that the zoom-in causes the dots to overlap more in the inset than in the main sub-plot. The univariate kernel density estimates along the x- (Median Heatwave Area (%)) and y-axis (Heatwave Length (days)) are on the top and right side of the subplots, respectively, with vertical lines in dark blue (Berkeley Earth) and light blue (ERA5) indicating the observed event. The respective region is indicated above the color bar.

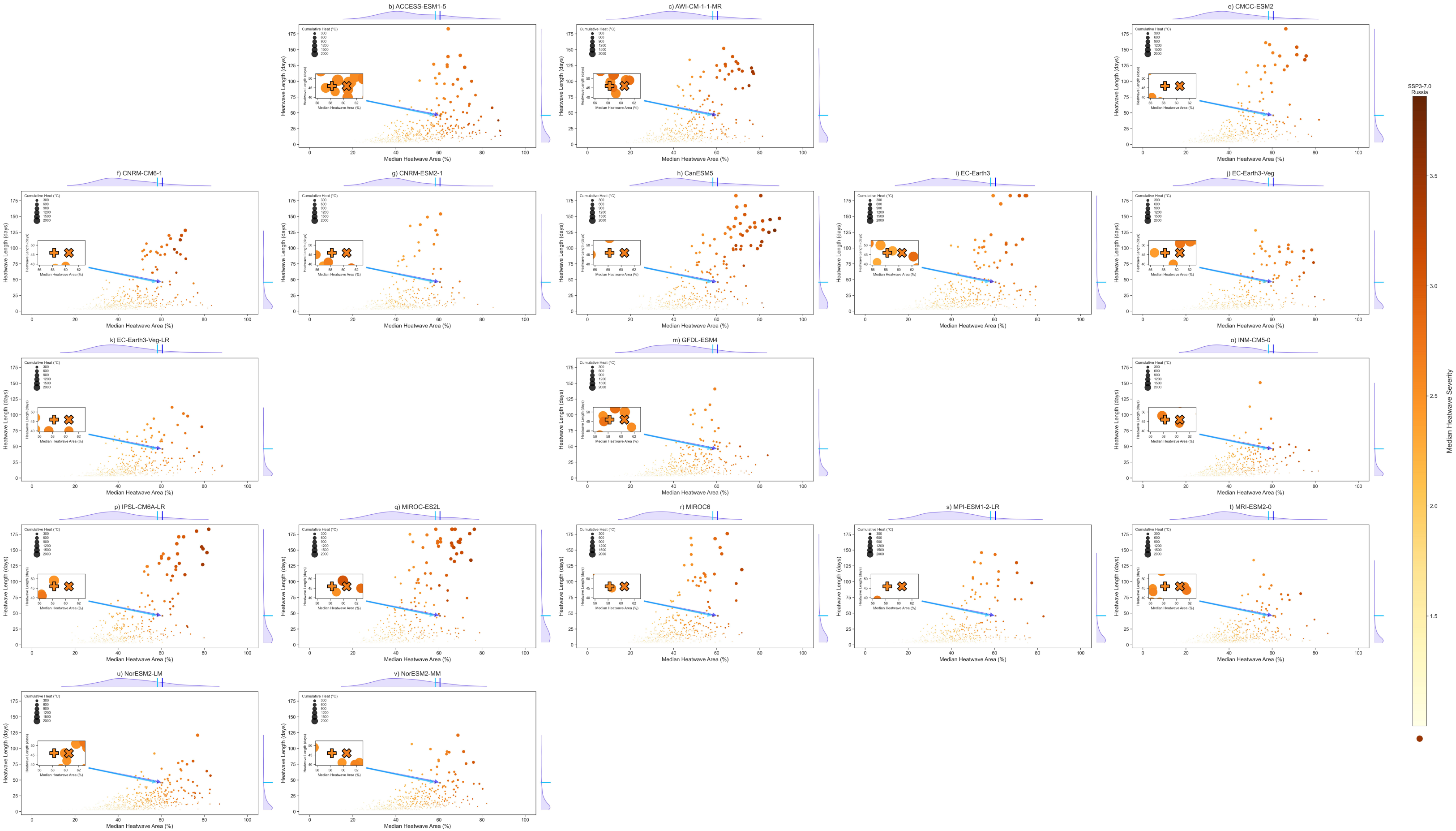
b) ACCESS-ESM1-5
c) AWI-CM-1-1-MR
e) CMCC-ESM2
f) CNRM-CM6-1
g) CNRM-ESM2-1
h) CanESM5
i) EC-Earth3
j) EC-Earth3-Veg
k) EC-Earth3-Veg-LR
m) GFDL-ESM4
o) INM-CM5-0
p) IPSL-CM6A-LR
q) MIROC-ES2L
r) MIROC6
s) MPI-ESM1-2-LR
t) MRI-ESM2-0
u) NorESM2-LM
v) NorESM2-MM
Cumulative Heat (°C)
300
600
900
1200
1500
2000
Heatwave Length (days)
Median Heatwave Area (%)
SSP3-7.0
Russia
Median Heatwave Severity

Supplementary Figure 263: Multi-HW-characteristic figure according to SSP3-7.0 CMIP6 multi-model ensemble (MME) per model for Southwestern USA & Mexico (Tx90p) - Each panel shows regional projections according to SSP3-7.0 model runs in the CMIP6 multi-model ensemble (MME) of four combined heatwave characteristics for each projected heatwave in the ensemble between 2020 and 2100. Each dot represents an individual heatwave in the projections, characterized by Median Heatwave Area (%) (x-axis) as latitude-weighted percentage of grid boxes affected by extreme heat, Heatwave Length (days) (y-axis) in days, Median Heatwave Severity (color), and Cumulative Heat (°C) (dot size). The characteristics of the observed event according to the Berkeley Earth observational data (x symbol) and the ERA5 reanalysis data (+ symbol), respectively, are pointed to by an arrow and a figure inset shows a zoom-in on this figure section. The figure inset shows the observed heatwave in the two reference data sets and projections in the indicated section of the respective sub-plot. It facilitates readability of color and size of the reference values and comparison to each other and the neighboring projections. Note that the zoom-in causes the dots to overlap more in the inset than in the main sub-plot. The univariate kernel density estimates along the x- (Median Heatwave Area (%)) and y-axis (Heatwave Length (days)) are on the top and right side of the subplots, respectively, with vertical lines in dark blue (Berkeley Earth) and light blue (ERA5) indicating the observed event. The respective region is indicated above the color bar.

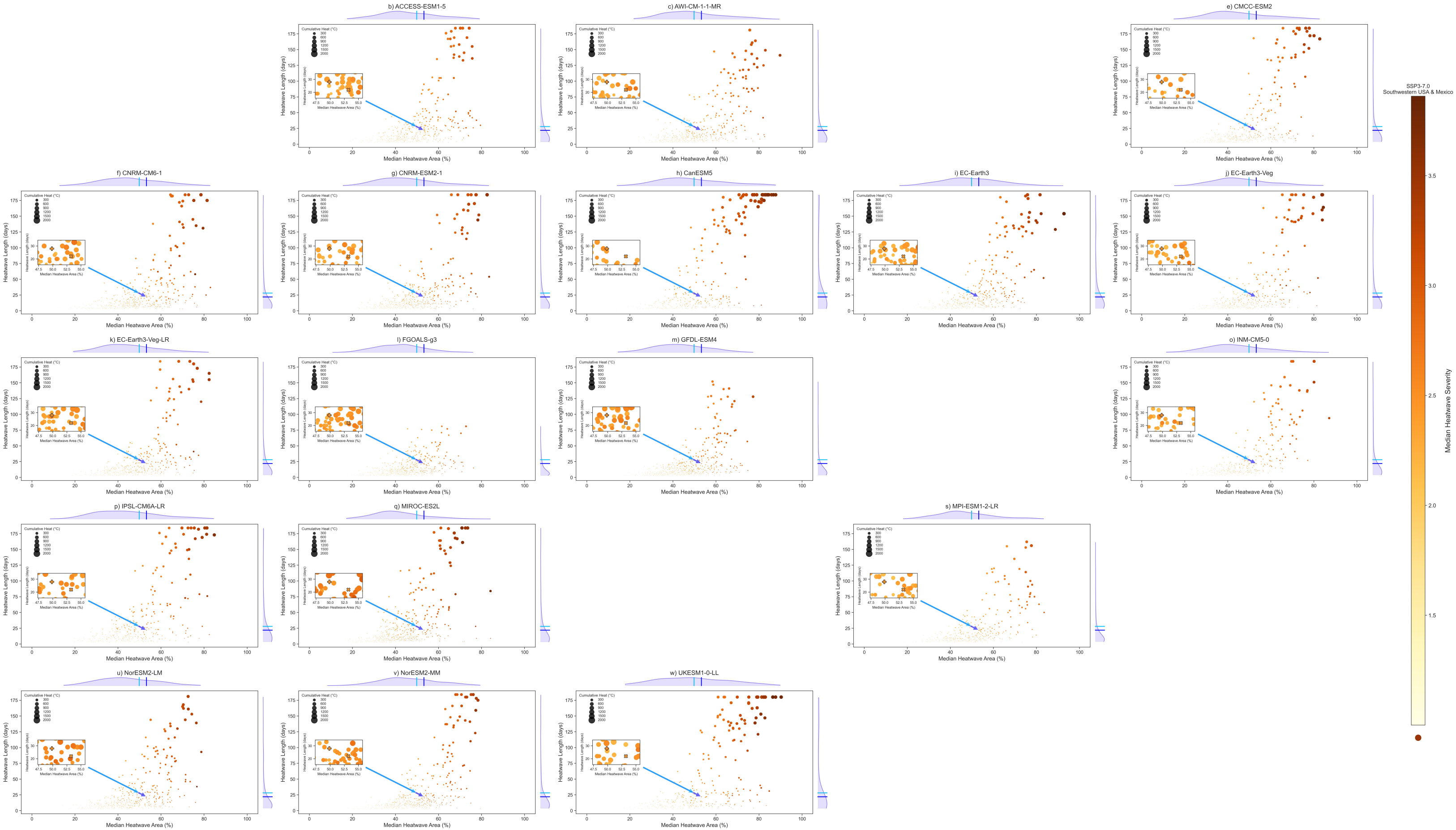
b) ACCESS-ESM1-5
c) AWI-CM-1-1-MR
e) CMCC-ESM2
f) CNRM-CM6-1
g) CNRM-ESM2-1
h) CanESM5
i) EC-Earth3
j) EC-Earth3-Veg
k) EC-Earth3-Veg-LR
l) FGOALS-g3
m) GFDL-ESM4
o) INM-CM5-0
p) IPSL-CM6A-LR
q) MIROC-ES2L
s) MPI-ESM1-2-LR
u) NorESM2-LM
v) NorESM2-MM
w) UKESM1-0-LL
Cumulative Heat (°C)
300
600
900
1200
1500
2000
Heatwave Length (days)
Median Heatwave Area (%)
SSP3-7.0
Southwestern USA & Mexico
Median Heatwave Severity
1.5
2.0
2.5
3.0
3.5

Supplementary Figure 264: Multi-HW-characteristic figure according to SSP3-7.0 CMIP6 multi-model ensemble (MME) per model for Siberia (Tx90p) - Each panel shows regional projections according to SSP3-7.0 model runs in the CMIP6 multi-model ensemble (MME) of four combined heatwave characteristics for each projected heatwave in the ensemble between 2020 and 2100. Each dot represents an individual heatwave in the projections, characterized by Median Heatwave Area (%) (x-axis) as latitude-weighted percentage of grid boxes affected by extreme heat, Heatwave Length (days) (y-axis) in days, Median Heatwave Severity (color), and Cumulative Heat (°C) (dot size). The characteristics of the observed event according to the Berkeley Earth observational data (x symbol) and the ERA5 reanalysis data (+ symbol), respectively, are pointed to by an arrow and a figure inset shows a zoom-in on this figure section. The figure inset shows the observed heatwave in the two reference data sets and projections in the indicated section of the respective sub-plot. It facilitates readability of color and size of the reference values and comparison to each other and the neighboring projections. Note that the zoom-in causes the dots to overlap more in the inset than in the main sub-plot. The univariate kernel density estimates along the x- (Median Heatwave Area (%)) and y-axis (Heatwave Length (days)) are on the top and right side of the subplots, respectively, with vertical lines in dark blue (Berkeley Earth) and light blue (ERA5) indicating the observed event. The respective region is indicated above the color bar.

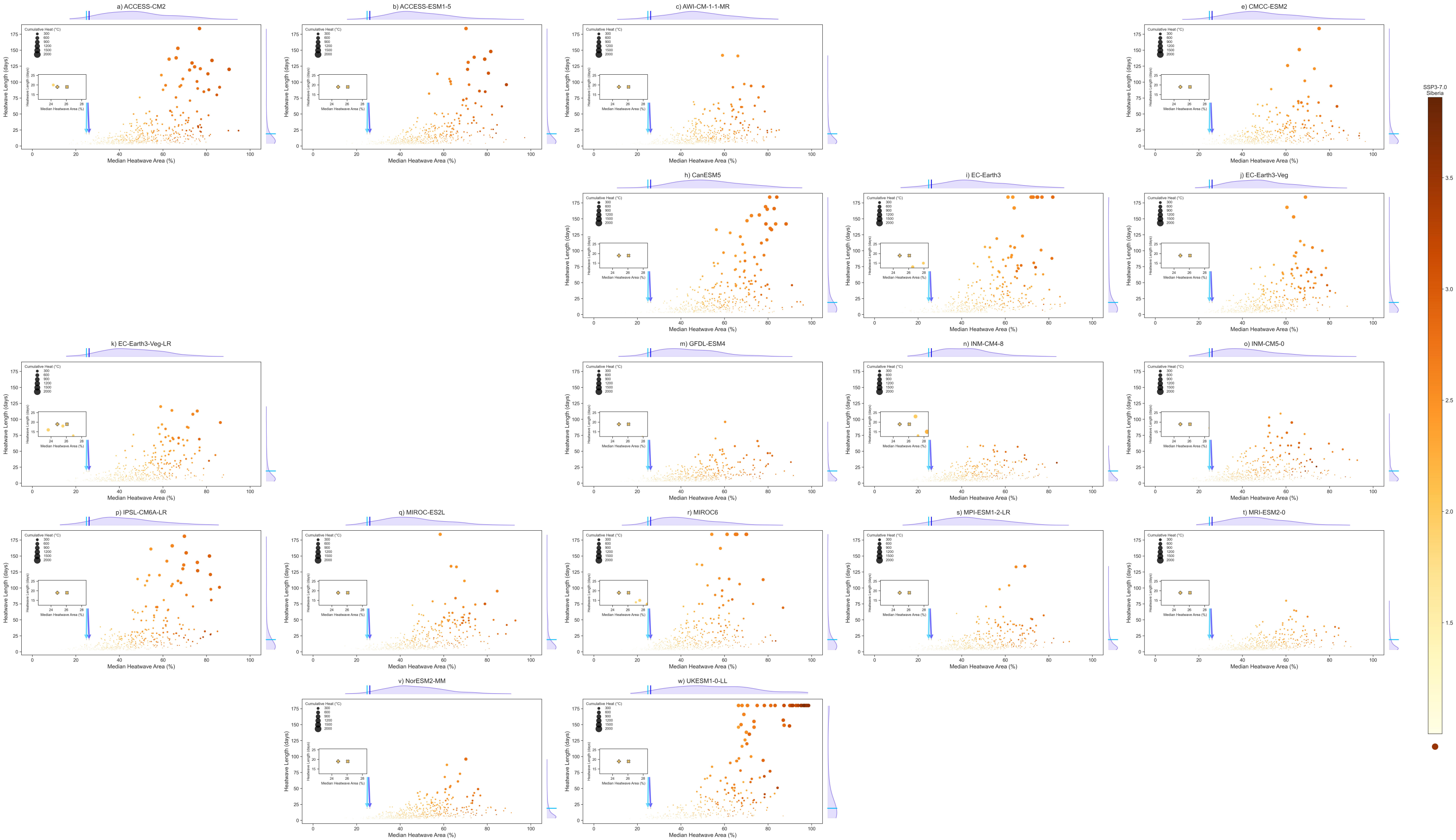
a) ACCESS-CM2
b) ACCESS-ESM1-5
c) AWI-CM-1-1-MR
e) CMCC-ESM2
h) CanESM5
i) EC-Earth3
j) EC-Earth3-Veg
k) EC-Earth3-Veg-LR
m) GFDL-ESM4
n) INM-CM4-8
o) INM-CM5-0
p) IPSL-CM6A-LR
q) MIROC-ES2L
r) MIROC6
s) MPI-ESM1-2-LR
t) MRI-ESM2-0
v) NorESM2-MM
w) UKESM1-0-LL
Heatwave Length (days)
Median Heatwave Area (%)
Cumulative Heat (°C)
300
600
900
1200
1500
2000
SSP3-7.0
Siberia
Median Heatwave Severity

Supplementary Figure 265: Multi-HW-characteristic figure according to SSP3-7.0 CMIP6 multi-model ensemble (MME) per model for South America (Tx90p) - Each panel shows regional projections according to SSP3-7.0 model runs in the CMIP6 multi-model ensemble (MME) of four combined heatwave characteristics for each projected heatwave in the ensemble between 2020 and 2100. Each dot represents an individual heatwave in the projections, characterized by Median Heatwave Area (%) (x-axis) as latitude-weighted percentage of grid boxes affected by extreme heat, Heatwave Length (days) (y-axis) in days, Median Heatwave Severity (color), and Cumulative Heat (°C) (dot size). The characteristics of the observed event according to the Berkeley Earth observational data (x symbol) and the ERA5 reanalysis data (+ symbol), respectively, are pointed to by an arrow and a figure inset shows a zoom-in on this figure section. The figure inset shows the observed heatwave in the two reference data sets and projections in the indicated section of the respective sub-plot. It facilitates readability of color and size of the reference values and comparison to each other and the neighboring projections. Note that the zoom-in causes the dots to overlap more in the inset than in the main sub-plot. The univariate kernel density estimates along the x- (Median Heatwave Area (%)) and y-axis (Heatwave Length (days)) are on the top and right side of the subplots, respectively, with vertical lines in dark blue (Berkeley Earth) and light blue (ERA5) indicating the observed event. The respective region is indicated above the color bar.

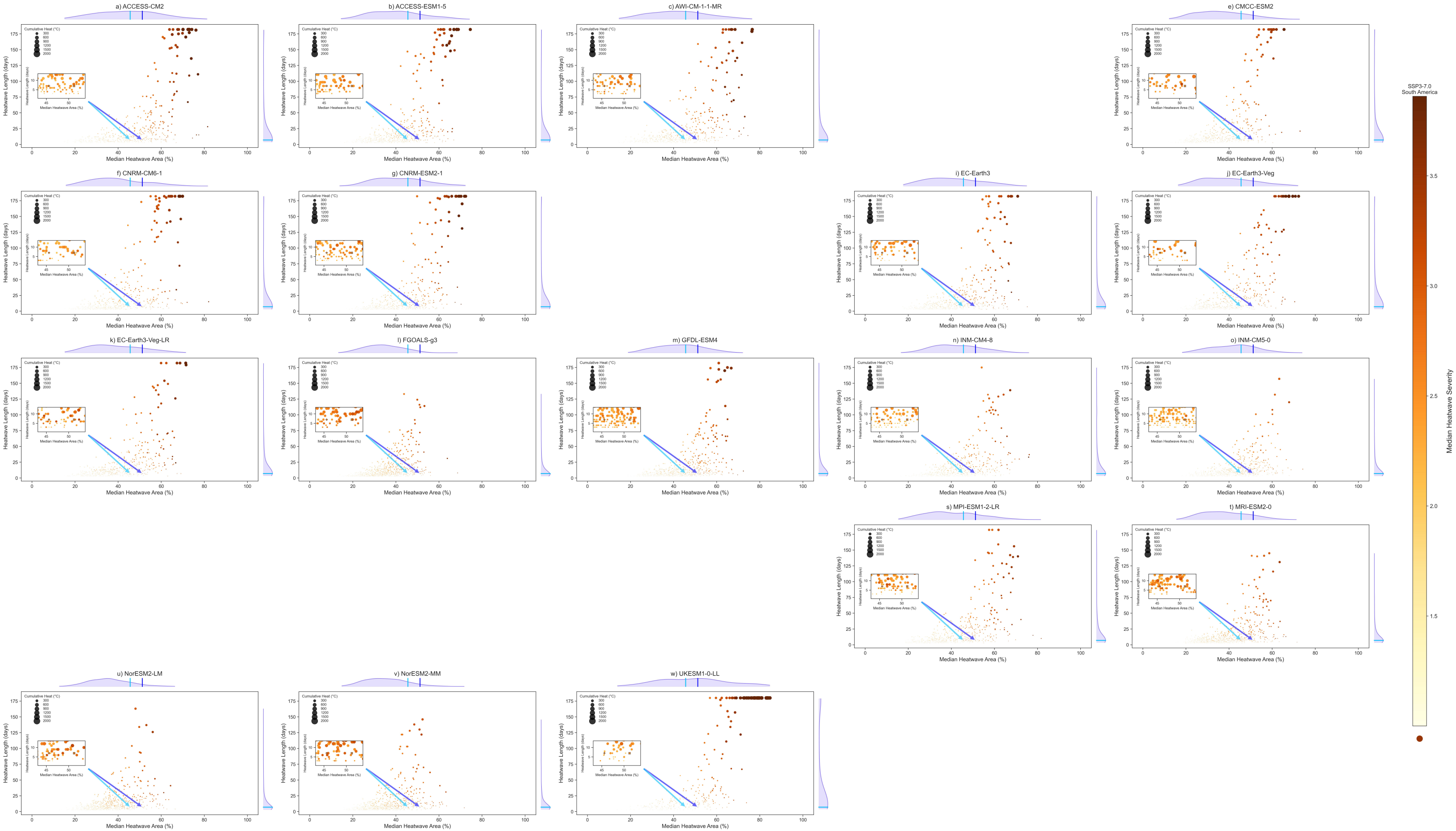

a) ACCESS-CM2
b) ACCESS-ESM1-5
c) AWI-CM-1-1-MR
e) CMCC-ESM2
f) CNRM-CM6-1
g) CNRM-ESM2-1
i) EC-Earth3
j) EC-Earth3-Veg
k) EC-Earth3-Veg-LR
l) FGOALS-g3
m) GFDL-ESM4
n) INM-CM4-8
o) INM-CM5-0
s) MPI-ESM1-2-LR
t) MRI-ESM2-0
u) NorESM2-LM
v) NorESM2-MM
w) UKESM1-0-LL
Cumulative Heat (°C)
300
600
900
1200
1500
2000
Heatwave Length (days)
Median Heatwave Area (%)
SSP3-7.0
South America
Median Heatwave Severity
1.5
2.0
2.5
3.0
3.5

Supplementary Figure 266: Multi-HW-characteristic figure according to SSP3-7.0 CMIP6 multi-model ensemble (MME) per model for Northern Southeast Asia (Tx90p) - Each panel shows regional projections according to SSP3-7.0 model runs in the CMIP6 multi-model ensemble (MME) of four combined heatwave characteristics for each projected heatwave in the ensemble between 2020 and 2100. Each dot represents an individual heatwave in the projections, characterized by Median Heatwave Area (%) (x-axis) as latitude-weighted percentage of grid boxes affected by extreme heat, Heatwave Length (days) (y-axis) in days, Median Heatwave Severity (color), and Cumulative Heat (°C) (dot size). The characteristics of the observed event according to the Berkeley Earth observational data (x symbol) and the ERA5 reanalysis data (+ symbol), respectively, are pointed to by an arrow and a figure inset shows a zoom-in on this figure section. The figure inset shows the observed heatwave in the two reference data sets and projections in the indicated section of the respective sub-plot. It facilitates readability of color and size of the reference values and comparison to each other and the neighboring projections. Note that the zoom-in causes the dots to overlap more in the inset than in the main sub-plot. The univariate kernel density estimates along the x- (Median Heatwave Area (%)) and y-axis (Heatwave Length (days)) are on the top and right side of the subplots, respectively, with vertical lines in dark blue (Berkeley Earth) and light blue (ERA5) indicating the observed event. The respective region is indicated above the color bar.

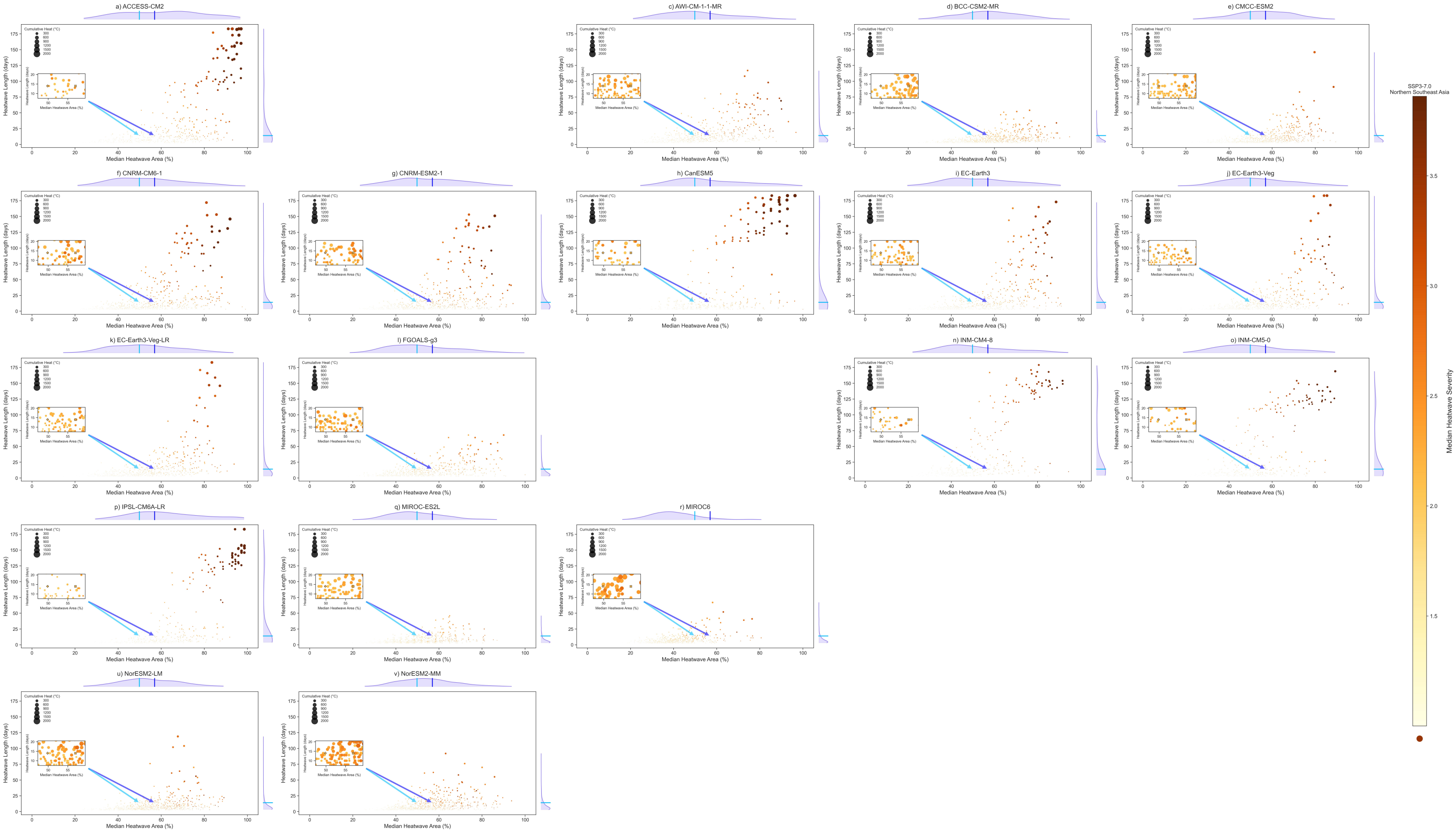

a) ACCESS-CM2
c) AWI-CM-1-1-MR
d) BCC-CSM2-MR
e) CMCC-ESM2
f) CNRM-CM6-1
g) CNRM-ESM2-1
h) CanESM5
i) EC-Earth3
j) EC-Earth3-Veg
k) EC-Earth3-Veg-LR
l) FGOALS-g3
n) INM-CM4-8
o) INM-CM5-0
p) IPSL-CM6A-LR
q) MIROC-ES2L
r) MIROC6
u) NorESM2-LM
v) NorESM2-MM
Cumulative Heat (°C)
300
600
900
1200
1500
2000
Heatwave Length (days)
Median Heatwave Area (%)
SSP3-7.0
Northern Southeast Asia
Median Heatwave Severity

Supplementary Figure 267: Multi-HW-characteristic figure according to SSP3-7.0 CMIP6 multi-model ensemble (MME) per model for Southern Africa (Tx90p) - Each panel shows regional projections according to SSP3-7.0 model runs in the CMIP6 multi-model ensemble (MME) of four combined heatwave characteristics for each projected heatwave in the ensemble between 2020 and 2100. Each dot represents an individual heatwave in the projections, characterized by Median Heatwave Area (%) (x-axis) as latitude-weighted percentage of grid boxes affected by extreme heat, Heatwave Length (days) (y-axis) in days, Median Heatwave Severity (color), and Cumulative Heat (°C) (dot size). The characteristics of the observed event according to the Berkeley Earth observational data (x symbol) and the ERA5 reanalysis data (+ symbol), respectively, are pointed to by an arrow and a figure inset shows a zoom-in on this figure section. The figure inset shows the observed heatwave in the two reference data sets and projections in the indicated section of the respective sub-plot. It facilitates readability of color and size of the reference values and comparison to each other and the neighboring projections. Note that the zoom-in causes the dots to overlap more in the inset than in the main sub-plot. The univariate kernel density estimates along the x- (Median Heatwave Area (%)) and y-axis (Heatwave Length (days)) are on the top and right side of the subplots, respectively, with vertical lines in dark blue (Berkeley Earth) and light blue (ERA5) indicating the observed event. The respective region is indicated above the color bar.

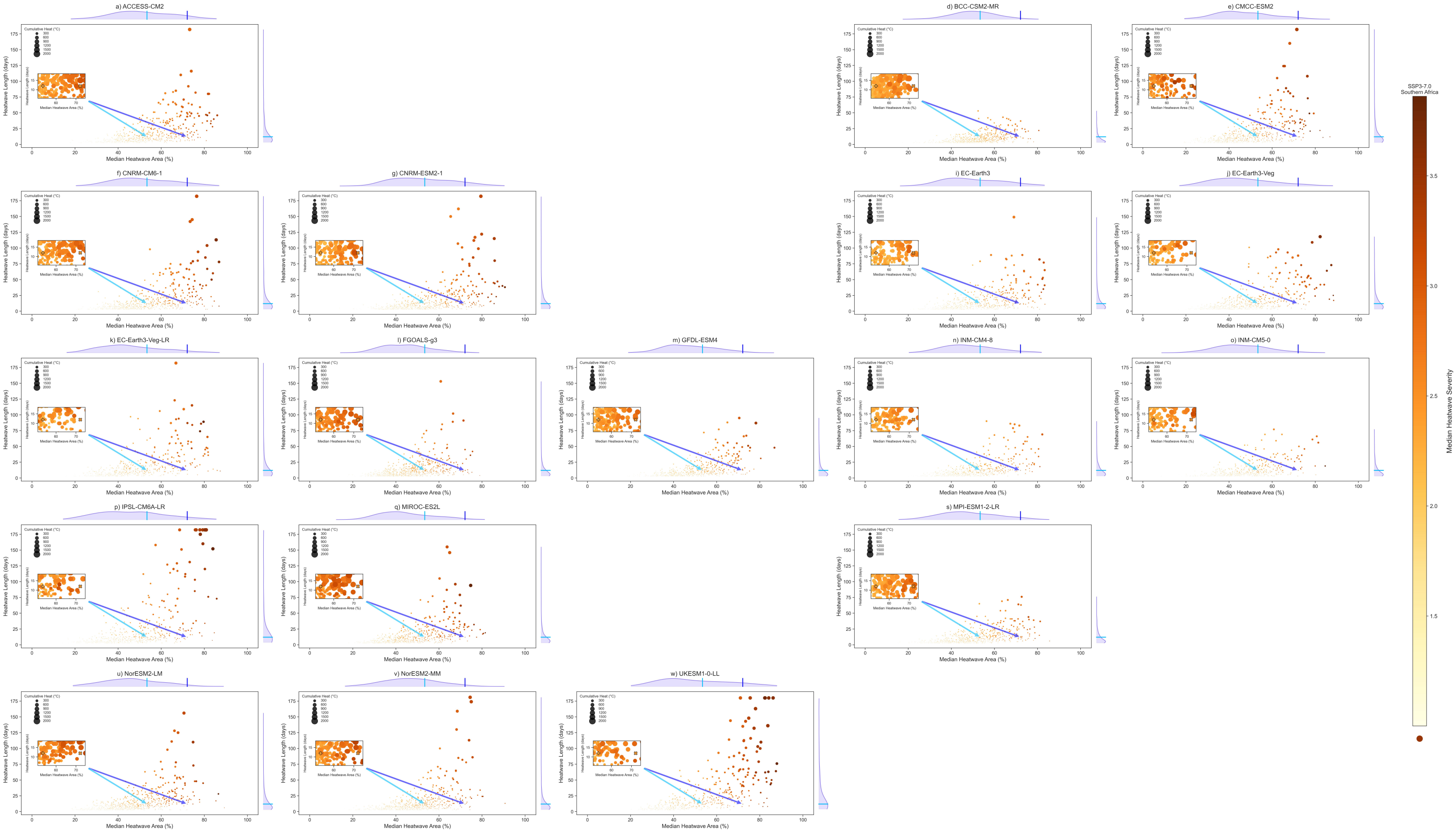
a) ACCESS-CM2
d) BCC-CSM2-MR
e) CMCC-ESM2
f) CNRM-CM6-1
g) CNRM-ESM2-1
i) EC-Earth3
j) EC-Earth3-Veg
k) EC-Earth3-Veg-LR
l) FGOALS-g3
m) GFDL-ESM4
n) INM-CM4-8
o) INM-CM5-0
p) IPSL-CM6A-LR
q) MIROC-ES2L
s) MPI-ESM1-2-LR
u) NorESM2-LM
v) NorESM2-MM
w) UKESM1-0-LL
Cumulative Heat (°C)
300
600
900
1200
1500
2000
Heatwave Length (days)
Median Heatwave Area (%)
SSP3-7.0
Southern Africa
Median Heatwave Severity
1.5
2.0
2.5
3.0
3.5

Supplementary Figure 268: Multi-HW-characteristic figure according to SSP3-7.0 CMIP6 multi-model ensemble (MME) per model for Eastern USA (Tx90p) - Each panel shows regional projections according to SSP3-7.0 model runs in the CMIP6 multi-model ensemble (MME) of four combined heatwave characteristics for each projected heatwave in the ensemble between 2020 and 2100. Each dot represents an individual heatwave in the projections, characterized by Median Heatwave Area (%) (x-axis) as latitude-weighted percentage of grid boxes affected by extreme heat, Heatwave Length (days) (y-axis) in days, Median Heatwave Severity (color), and Cumulative Heat (°C) (dot size). The characteristics of the observed event according to the Berkeley Earth observational data (x symbol) and the ERA5 reanalysis data (+ symbol), respectively, are pointed to by an arrow and a figure inset shows a zoom-in on this figure section. The figure inset shows the observed heatwave in the two reference data sets and projections in the indicated section of the respective sub-plot. It facilitates readability of color and size of the reference values and comparison to each other and the neighboring projections. Note that the zoom-in causes the dots to overlap more in the inset than in the main sub-plot. The univariate kernel density estimates along the x- (Median Heatwave Area (%)) and y-axis (Heatwave Length (days)) are on the top and right side of the subplots, respectively, with vertical lines in dark blue (Berkeley Earth) and light blue (ERA5) indicating the observed event. The respective region is indicated above the color bar.

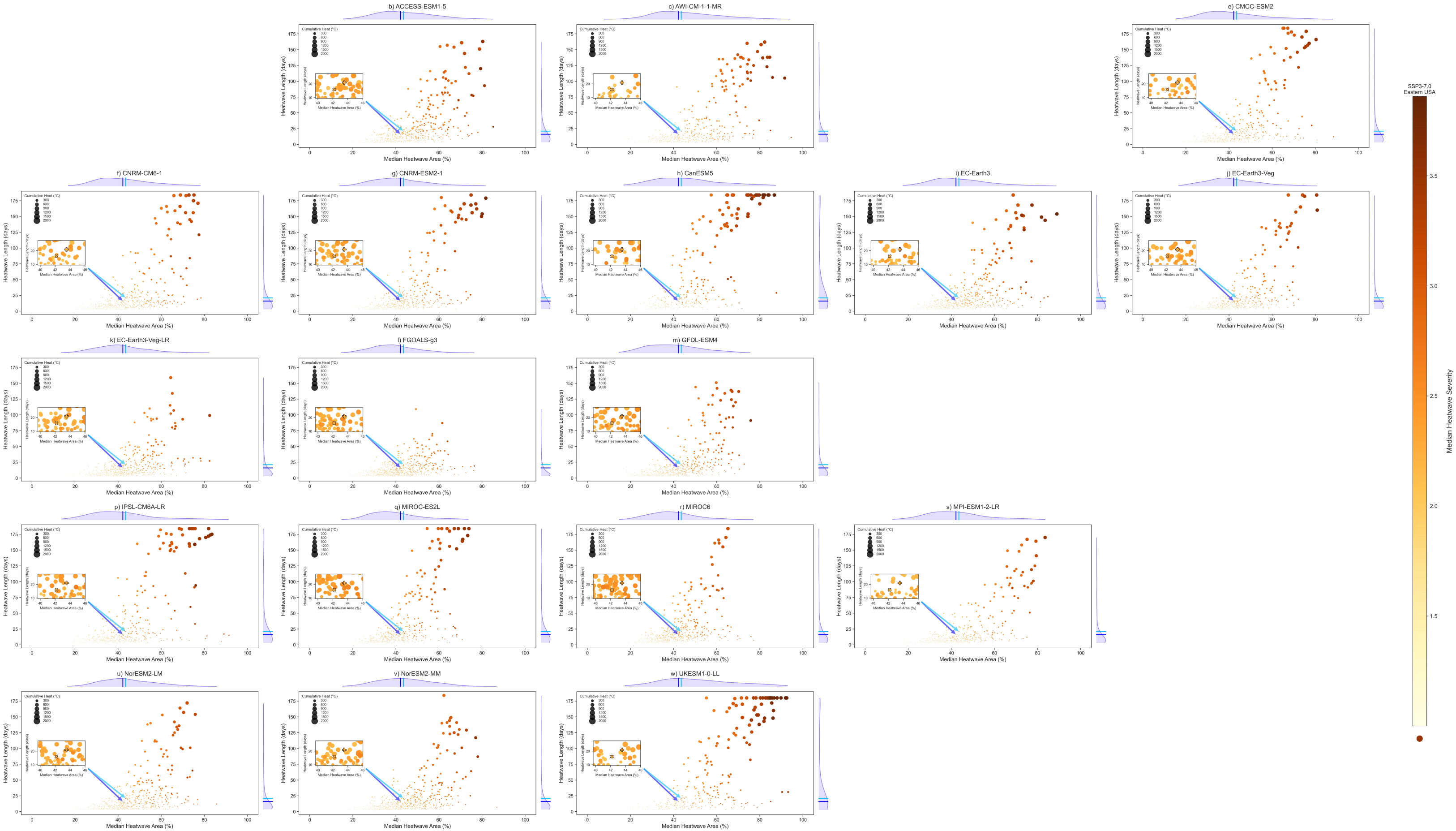

b) ACCESS-ESM1-5
c) AWI-CM-1-1-MR
e) CMCC-ESM2
f) CNRM-CM6-1
g) CNRM-ESM2-1
h) CanESM5
i) EC-Earth3
j) EC-Earth3-Veg
k) EC-Earth3-Veg-LR
l) FGOALS-g3
m) GFDL-ESM4
p) IPSL-CM6A-LR
q) MIROC-ES2L
r) MIROC6
s) MPI-ESM1-2-LR
u) NorESM2-LM
v) NorESM2-MM
w) UKESM1-0-LL
Cumulative Heat (°C)
300
600
900
1200
1500
2000
Heatwave Length (days)
Median Heatwave Area (%)
SSP3-7.0
Eastern USA
Median Heatwave Severity

Supplementary Figure 269: Multi-HW-characteristic figure according to SSP5-8.5 CMIP6 multi-model ensemble (MME) per model for Alaska & Canada (Tx90p) - Each panel shows regional projections according to SSP5-8.5 model runs in the CMIP6 multi-model ensemble (MME) of four combined heatwave characteristics for each projected heatwave in the ensemble between 2020 and 2100. Each dot represents an individual heatwave in the projections, characterized by Median Heatwave Area (%) (x-axis) as latitude-weighted percentage of grid boxes affected by extreme heat, Heatwave Length (days) (y-axis) in days, Median Heatwave Severity (color), and Cumulative Heat (°C) (dot size). The characteristics of the observed event according to the Berkeley Earth observational data (x symbol) and the ERA5 reanalysis data (+ symbol), respectively, are pointed to by an arrow and a figure inset shows a zoom-in on this figure section. The figure inset shows the observed heatwave in the two reference data sets and projections in the indicated section of the respective sub-plot. It facilitates readability of color and size of the reference values and comparison to each other and the neighboring projections. Note that the zoom-in causes the dots to overlap more in the inset than in the main sub-plot. The univariate kernel density estimates along the x- (Median Heatwave Area (%)) and y-axis (Heatwave Length (days)) are on the top and right side of the subplots, respectively, with vertical lines in dark blue (Berkeley Earth) and light blue (ERA5) indicating the observed event. The respective region is indicated above the color bar.

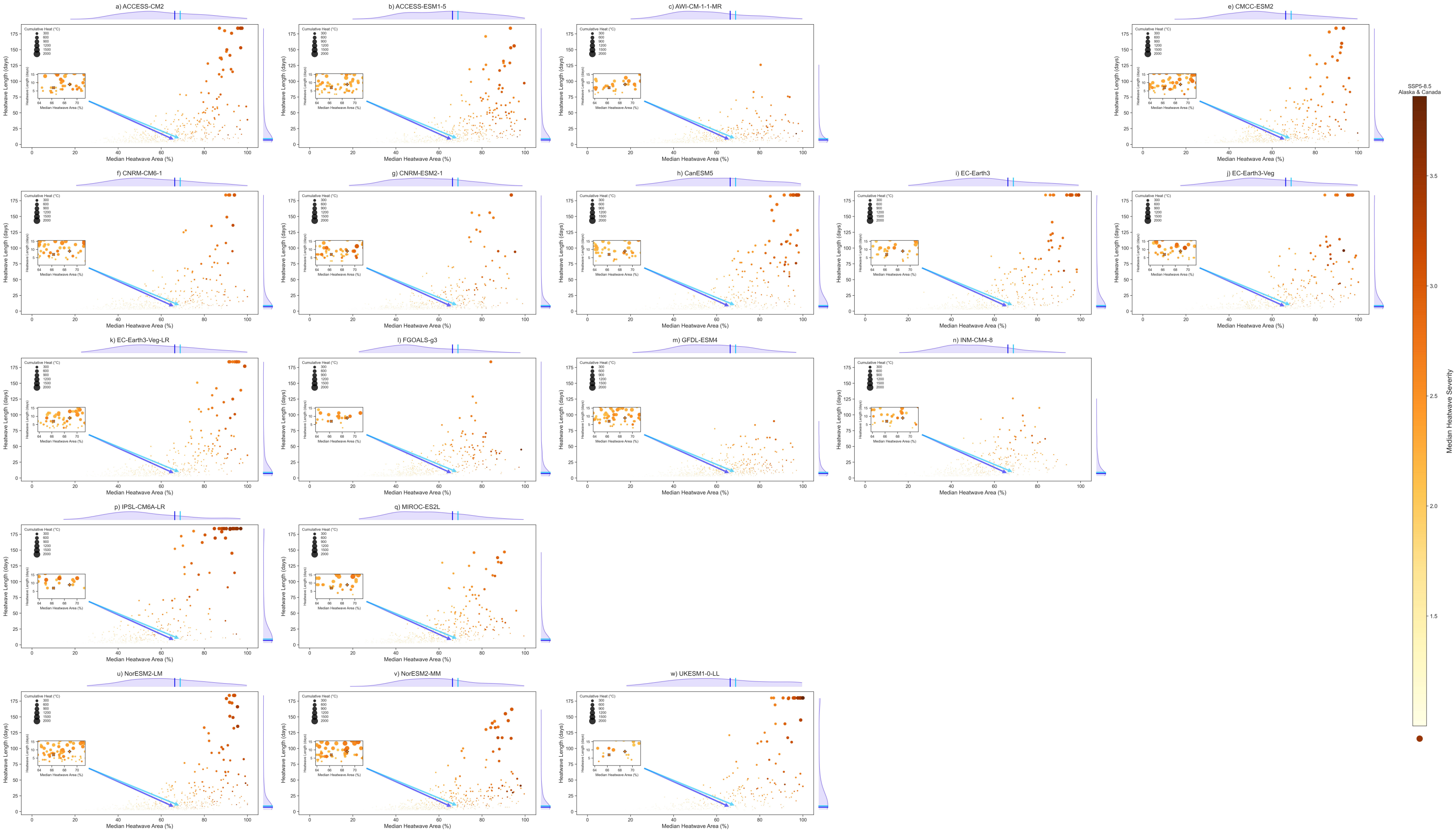

a) ACCESS-CM2
b) ACCESS-ESM1-5
c) AWI-CM-1-1-MR
e) CMCC-ESM2
f) CNRM-CM6-1
g) CNRM-ESM2-1
h) CanESM5
i) EC-Earth3
j) EC-Earth3-Veg
k) EC-Earth3-Veg-LR
l) FGOALS-g3
m) GFDL-ESM4
n) INM-CM4-8
p) IPSL-CM6A-LR
q) MIROC-ES2L
u) NorESM2-LM
v) NorESM2-MM
w) UKESM1-0-LL
Cumulative Heat (°C)
300
600
900
1200
1500
2000
Heatwave Length (days)
Median Heatwave Area (%)
SSP5-8.5
Alaska & Canada
Median Heatwave Severity

Supplementary Figure 270: Multi-HW-characteristic figure according to SSP5-8.5 CMIP6 multi-model ensemble (MME) per model for Argentina, Bolivia, Chile, Paraguay & Uruguay (Tx90p) - Each panel shows regional projections according to SSP5-8.5 model runs in the CMIP6 multi-model ensemble (MME) of four combined heatwave characteristics for each projected heatwave in the ensemble between 2020 and 2100. Each dot represents an individual heatwave in the projections, characterized by Median Heatwave Area (%) (x-axis) as latitude-weighted percentage of grid boxes affected by extreme heat, Heatwave Length (days) (y-axis) in days, Median Heatwave Severity (color), and Cumulative Heat (°C) (dot size). The characteristics of the observed event according to the Berkeley Earth observational data (x symbol) and the ERA5 reanalysis data (+ symbol), respectively, are pointed to by an arrow and a figure inset shows a zoom-in on this figure section. The figure inset shows the observed heatwave in the two reference data sets and projections in the indicated section of the respective sub-plot. It facilitates readability of color and size of the reference values and comparison to each other and the neighboring projections. Note that the zoom-in causes the dots to overlap more in the inset than in the main sub-plot. The univariate kernel density estimates along the x- (Median Heatwave Area (%)) and y-axis (Heatwave Length (days)) are on the top and right side of the subplots, respectively, with vertical lines in dark blue (Berkeley Earth) and light blue (ERA5) indicating the observed event. The respective region is indicated above the color bar.

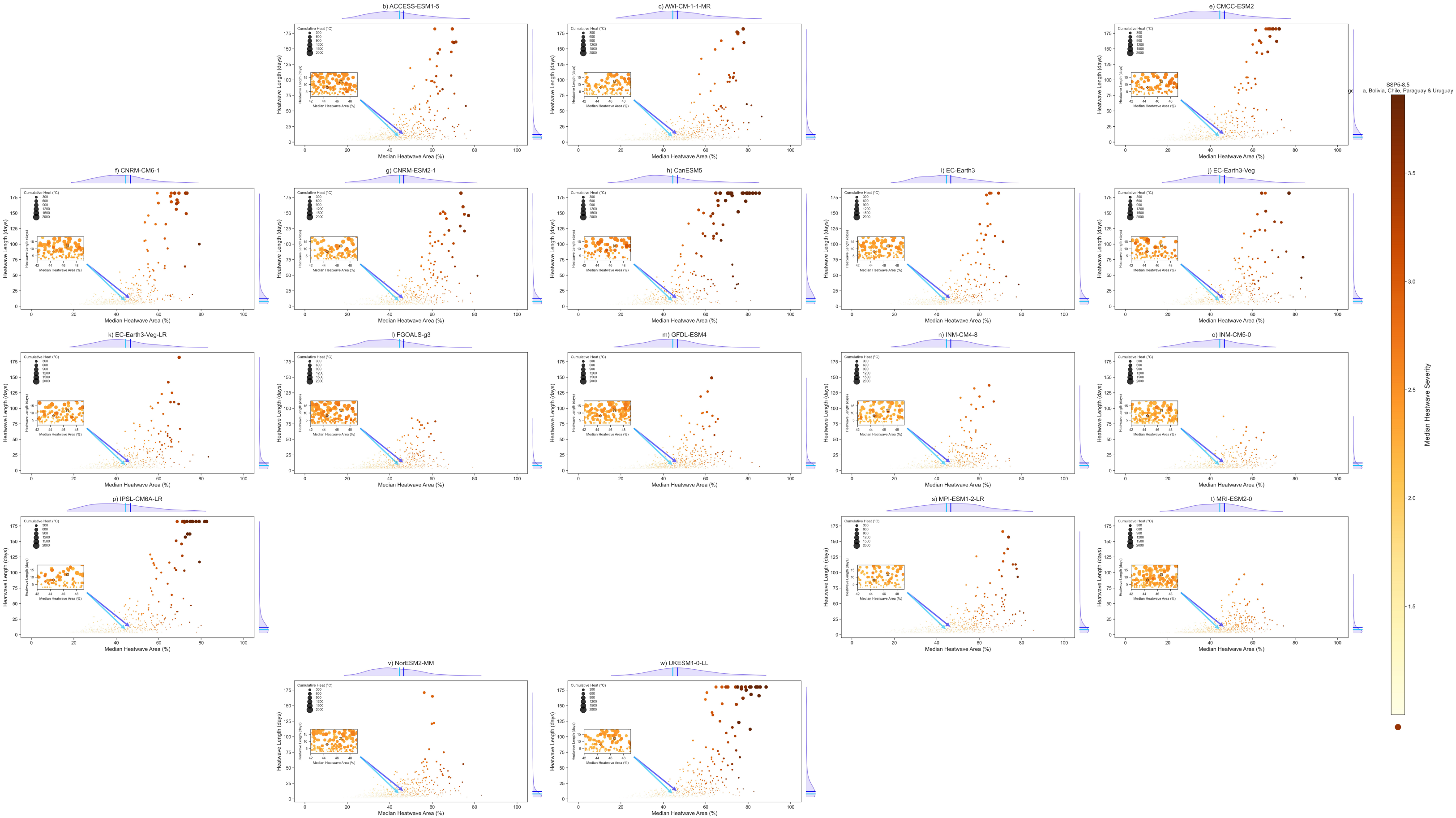
b) ACCESS-ESM1-5
c) AWI-CM-1-1-MR
e) CMCC-ESM2
f) CNRM-CM6-1
g) CNRM-ESM2-1
h) CanESM5
i) EC-Earth3
j) EC-Earth3-Veg
k) EC-Earth3-Veg-LR
l) FGOALS-g3
m) GFDL-ESM4
n) INM-CM4-8
o) INM-CM5-0
p) IPSL-CM6A-LR
s) MPI-ESM1-2-LR
t) MRI-ESM2-0
v) NorESM2-MM
w) UKESM1-0-LL
Cumulative Heat (°C)
300
600
900
1200
1500
2000
Heatwave Length (days)
Median Heatwave Area (%)
SSP5-8.5
a, Bolivia, Chile, Paraguay & Uruguay
Median Heatwave Severity

Supplementary Figure 271: Multi-HW-characteristic figure according to SSP5-8.5 CMIP6 multi-model ensemble (MME) per model for Argentina, Uruguay & Brazil (Tx90p) - Each panel shows regional projections according to SSP5-8.5 model runs in the CMIP6 multi-model ensemble (MME) of four combined heatwave characteristics for each projected heatwave in the ensemble between 2020 and 2100. Each dot represents an individual heatwave in the projections, characterized by Median Heatwave Area (%) (x-axis) as latitude-weighted percentage of grid boxes affected by extreme heat, Heatwave Length (days) (y-axis) in days, Median Heatwave Severity (color), and Cumulative Heat (°C) (dot size). The characteristics of the observed event according to the Berkeley Earth observational data (x symbol) and the ERA5 reanalysis data (+ symbol), respectively, are pointed to by an arrow and a figure inset shows a zoom-in on this figure section. The figure inset shows the observed heatwave in the two reference data sets and projections in the indicated section of the respective sub-plot. It facilitates readability of color and size of the reference values and comparison to each other and the neighboring projections. Note that the zoom-in causes the dots to overlap more in the inset than in the main sub-plot. The univariate kernel density estimates along the x- (Median Heatwave Area (%)) and y-axis (Heatwave Length (days)) are on the top and right side of the subplots, respectively, with vertical lines in dark blue (Berkeley Earth) and light blue (ERA5) indicating the observed event. The respective region is indicated above the color bar.

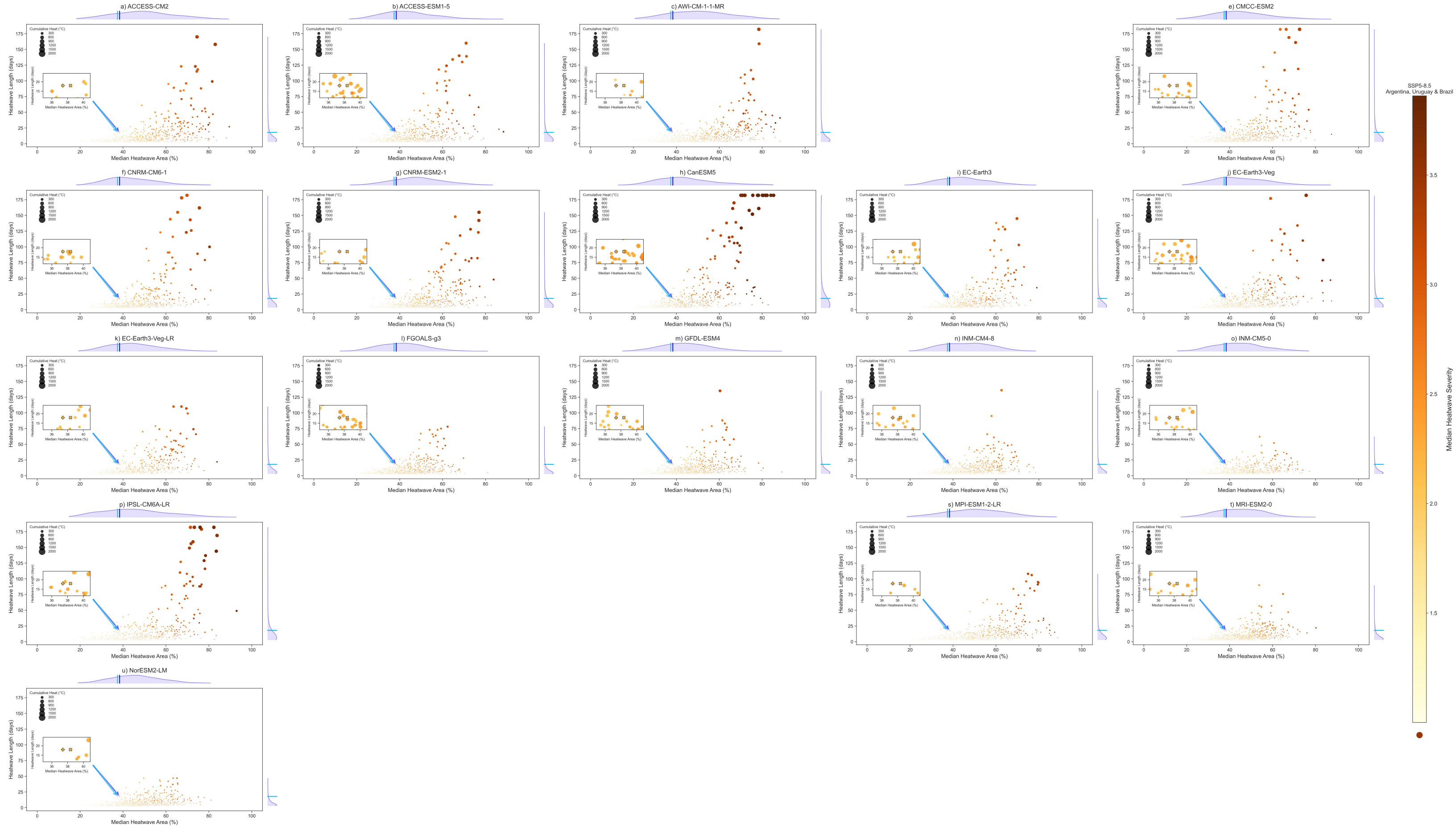

a) ACCESS-CM2
b) ACCESS-ESM1-5
c) AWI-CM-1-1-MR
e) CMCC-ESM2
f) CNRM-CM6-1
g) CNRM-ESM2-1
h) CanESM5
i) EC-Earth3
j) EC-Earth3-Veg
k) EC-Earth3-Veg-LR
l) FGOALS-g3
m) GFDL-ESM4
n) INM-CM4-8
o) INM-CM5-0
p) IPSL-CM6A-LR
s) MPI-ESM1-2-LR
t) MRI-ESM2-0
u) NorESM2-LM
Cumulative Heat (°C)
300
600
900
1200
1500
2000
Heatwave Length (days)
Median Heatwave Area (%)
SSP5-8.5
Argentina, Uruguay & Brazil
Median Heatwave Severity

Supplementary Figure 272: Multi-HW-characteristic figure according to SSP5-8.5 CMIP6 multi-model ensemble (MME) per model for Australia (Tx90p) - Each panel shows regional projections according to SSP5-8.5 model runs in the CMIP6 multi-model ensemble (MME) of four combined heatwave characteristics for each projected heatwave in the ensemble between 2020 and 2100. Each dot represents an individual heatwave in the projections, characterized by Median Heatwave Area (%) (x-axis) as latitude-weighted percentage of grid boxes affected by extreme heat, Heatwave Length (days) (y-axis) in days, Median Heatwave Severity (color), and Cumulative Heat (°C) (dot size). The characteristics of the observed event according to the Berkeley Earth observational data (x symbol) and the ERA5 reanalysis data (+ symbol), respectively, are pointed to by an arrow and a figure inset shows a zoom-in on this figure section. The figure inset shows the observed heatwave in the two reference data sets and projections in the indicated section of the respective sub-plot. It facilitates readability of color and size of the reference values and comparison to each other and the neighboring projections. Note that the zoom-in causes the dots to overlap more in the inset than in the main sub-plot. The univariate kernel density estimates along the x- (Median Heatwave Area (%)) and y-axis (Heatwave Length (days)) are on the top and right side of the subplots, respectively, with vertical lines in dark blue (Berkeley Earth) and light blue (ERA5) indicating the observed event. The respective region is indicated above the color bar.

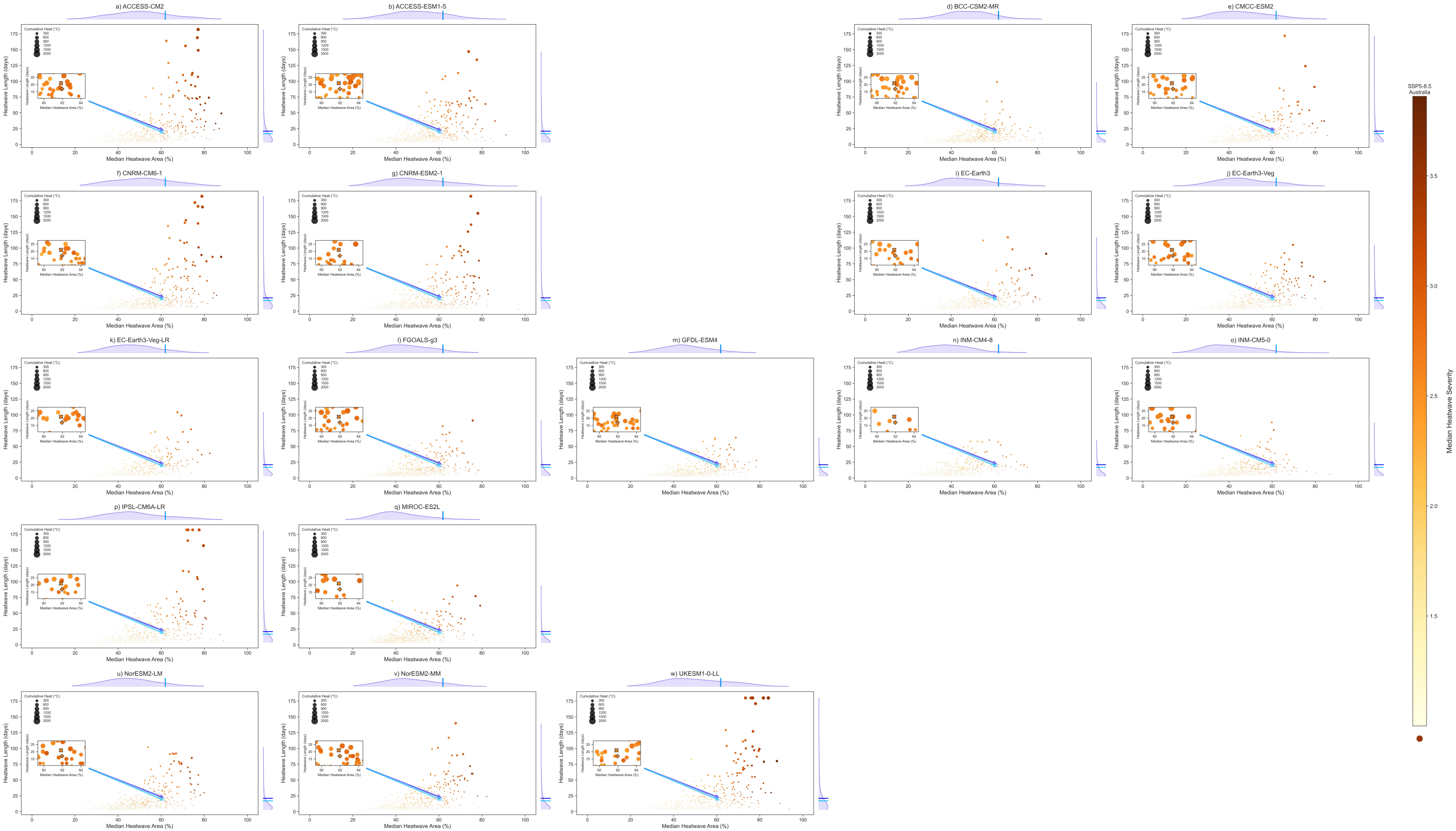
a) ACCESS-CM2
b) ACCESS-ESM1-5
d) BCC-CSM2-MR
e) CMCC-ESM2
f) CNRM-CM6-1
g) CNRM-ESM2-1
i) EC-Earth3
j) EC-Earth3-Veg
k) EC-Earth3-Veg-LR
l) FGOALS-g3
m) GFDL-ESM4
n) INM-CM4-8
o) INM-CM5-0
p) IPSL-CM6A-LR
q) MIROC-ES2L
u) NorESM2-LM
v) NorESM2-MM
w) UKESM1-0-LL
Cumulative Heat (°C)
300
600
900
1200
1500
2000
Heatwave Length (days)
Median Heatwave Area (%)
SSP5-8.5
Australia
Median Heatwave Severity
3.5
3.0
2.5
2.0
1.5

Supplementary Figure 273: Multi-HW-characteristic figure according to SSP5-8.5 CMIP6 multi-model ensemble (MME) per model for Central Siberia & Central Asia (Tx90p) - Each panel shows regional projections according to SSP5-8.5 model runs in the CMIP6 multi-model ensemble (MME) of four combined heatwave characteristics for each projected heatwave in the ensemble between 2020 and 2100. Each dot represents an individual heatwave in the projections, characterized by Median Heatwave Area (%) (x-axis) as latitude-weighted percentage of grid boxes affected by extreme heat, Heatwave Length (days) (y-axis) in days, Median Heatwave Severity (color), and Cumulative Heat (°C) (dot size). The characteristics of the observed event according to the Berkeley Earth observational data (x symbol) and the ERA5 reanalysis data (+ symbol), respectively, are pointed to by an arrow and a figure inset shows a zoom-in on this figure section. The figure inset shows the observed heatwave in the two reference data sets and projections in the indicated section of the respective sub-plot. It facilitates readability of color and size of the reference values and comparison to each other and the neighboring projections. Note that the zoom-in causes the dots to overlap more in the inset than in the main sub-plot. The univariate kernel density estimates along the x- (Median Heatwave Area (%)) and y-axis (Heatwave Length (days)) are on the top and right side of the subplots, respectively, with vertical lines in dark blue (Berkeley Earth) and light blue (ERA5) indicating the observed event. The respective region is indicated above the color bar.

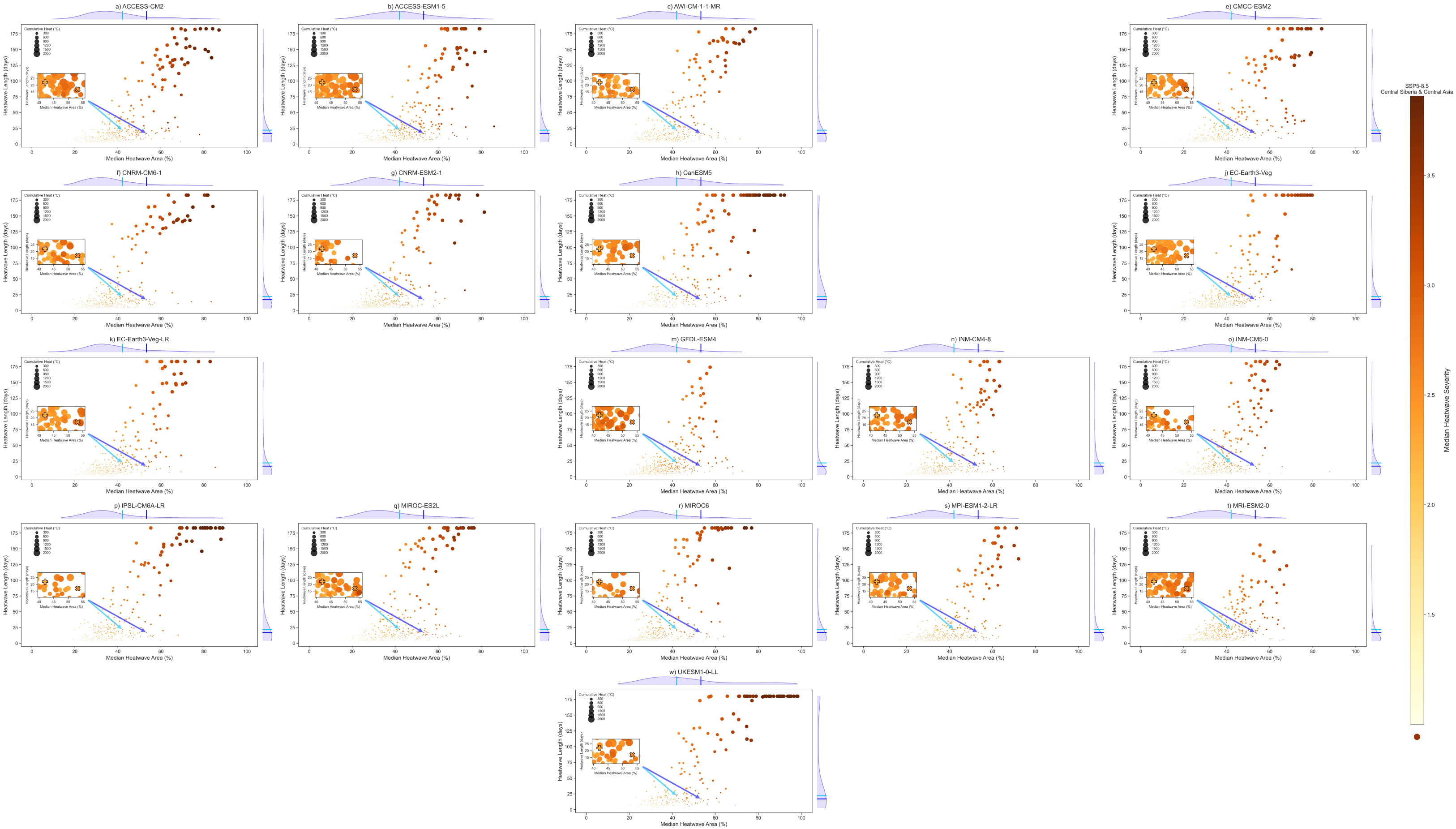
a) ACCESS-CM2
b) ACCESS-ESM1-5
c) AWI-CM-1-1-MR
e) CMCC-ESM2
f) CNRM-CM6-1
g) CNRM-ESM2-1
h) CanESM5
j) EC-Earth3-Veg
k) EC-Earth3-Veg-LR
m) GFDL-ESM4
n) INM-CM4-8
o) INM-CM5-0
p) IPSL-CM6A-LR
q) MIROC-ES2L
r) MIROC6
s) MPI-ESM1-2-LR
t) MRI-ESM2-0
w) UKESM1-0-LL
Cumulative Heat (°C)
300
600
900
1200
1500
2000
Heatwave Length (days)
Median Heatwave Area (%)
SSP5-8.5
Central Siberia & Central Asia
Median Heatwave Severity

Supplementary Figure 274: Multi-HW-characteristic figure according to SSP5-8.5 CMIP6 multi-model ensemble (MME) per model for China (2017) (Tx90p) - Each panel shows regional projections according to SSP5-8.5 model runs in the CMIP6 multi-model ensemble (MME) of four combined heatwave characteristics for each projected heatwave in the ensemble between 2020 and 2100. Each dot represents an individual heatwave in the projections, characterized by Median Heatwave Area (%) (x-axis) as latitude-weighted percentage of grid boxes affected by extreme heat, Heatwave Length (days) (y-axis) in days, Median Heatwave Severity (color), and Cumulative Heat (°C) (dot size). The characteristics of the observed event according to the Berkeley Earth observational data (x symbol) and the ERA5 reanalysis data (+ symbol), respectively, are pointed to by an arrow and a figure inset shows a zoom-in on this figure section. The figure inset shows the observed heatwave in the two reference data sets and projections in the indicated section of the respective sub-plot. It facilitates readability of color and size of the reference values and comparison to each other and the neighboring projections. Note that the zoom-in causes the dots to overlap more in the inset than in the main sub-plot. The univariate kernel density estimates along the x- (Median Heatwave Area (%)) and y-axis (Heatwave Length (days)) are on the top and right side of the subplots, respectively, with vertical lines in dark blue (Berkeley Earth) and light blue (ERA5) indicating the observed event. The respective region is indicated above the color bar.

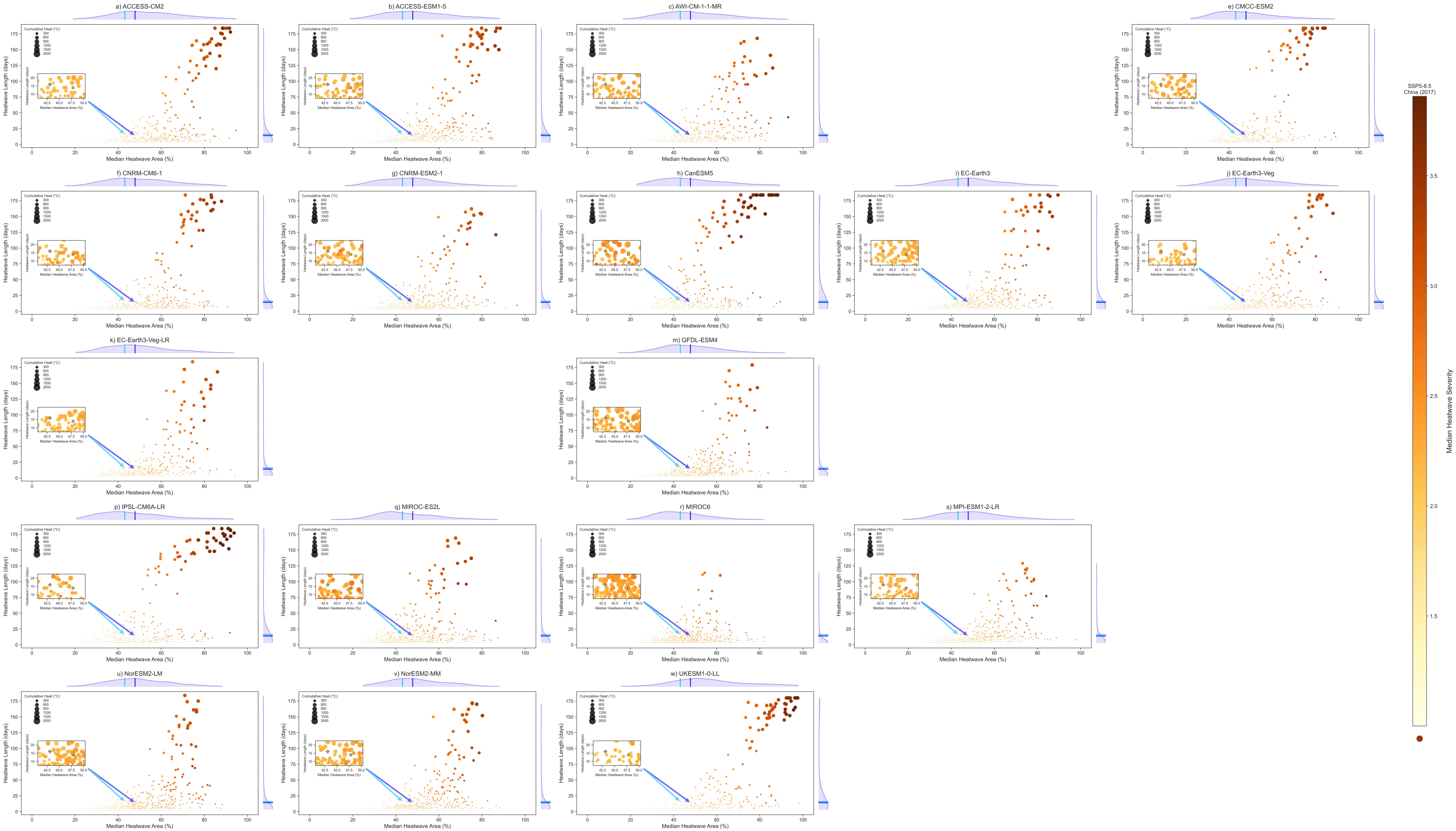

a) ACCESS-CM2
b) ACCESS-ESM1-5
c) AWI-CM-1-1-MR
e) CMCC-ESM2
f) CNRM-CM6-1
g) CNRM-ESM2-1
h) CanESM5
i) EC-Earth3
j) EC-Earth3-Veg
k) EC-Earth3-Veg-LR
m) GFDL-ESM4
p) IPSL-CM6A-LR
q) MIROC-ES2L
r) MIROC6
s) MPI-ESM1-2-LR
u) NorESM2-LM
v) NorESM2-MM
w) UKESM1-0-LL
Cumulative Heat (°C)
300
600
900
1200
1500
2000
Heatwave Length (days)
Median Heatwave Area (%)
SSP5-8.5
China (2017)
Median Heatwave Severity
1.5
2.0
2.5
3.0
3.5

Supplementary Figure 275: Multi-HW-characteristic figure according to SSP5-8.5 CMIP6 multi-model ensemble (MME) per model for China (Tx90p) - Each panel shows regional projections according to SSP5-8.5 model runs in the CMIP6 multi-model ensemble (MME) of four combined heatwave characteristics for each projected heatwave in the ensemble between 2020 and 2100. Each dot represents an individual heatwave in the projections, characterized by Median Heatwave Area (%) (x-axis) as latitude-weighted percentage of grid boxes affected by extreme heat, Heatwave Length (days) (y-axis) in days, Median Heatwave Severity (color), and Cumulative Heat (°C) (dot size). The characteristics of the observed event according to the Berkeley Earth observational data (x symbol) and the ERA5 reanalysis data (+ symbol), respectively, are pointed to by an arrow and a figure inset shows a zoom-in on this figure section. The figure inset shows the observed heatwave in the two reference data sets and projections in the indicated section of the respective sub-plot. It facilitates readability of color and size of the reference values and comparison to each other and the neighboring projections. Note that the zoom-in causes the dots to overlap more in the inset than in the main sub-plot. The univariate kernel density estimates along the x- (Median Heatwave Area (%)) and y-axis (Heatwave Length (days)) are on the top and right side of the subplots, respectively, with vertical lines in dark blue (Berkeley Earth) and light blue (ERA5) indicating the observed event. The respective region is indicated above the color bar.

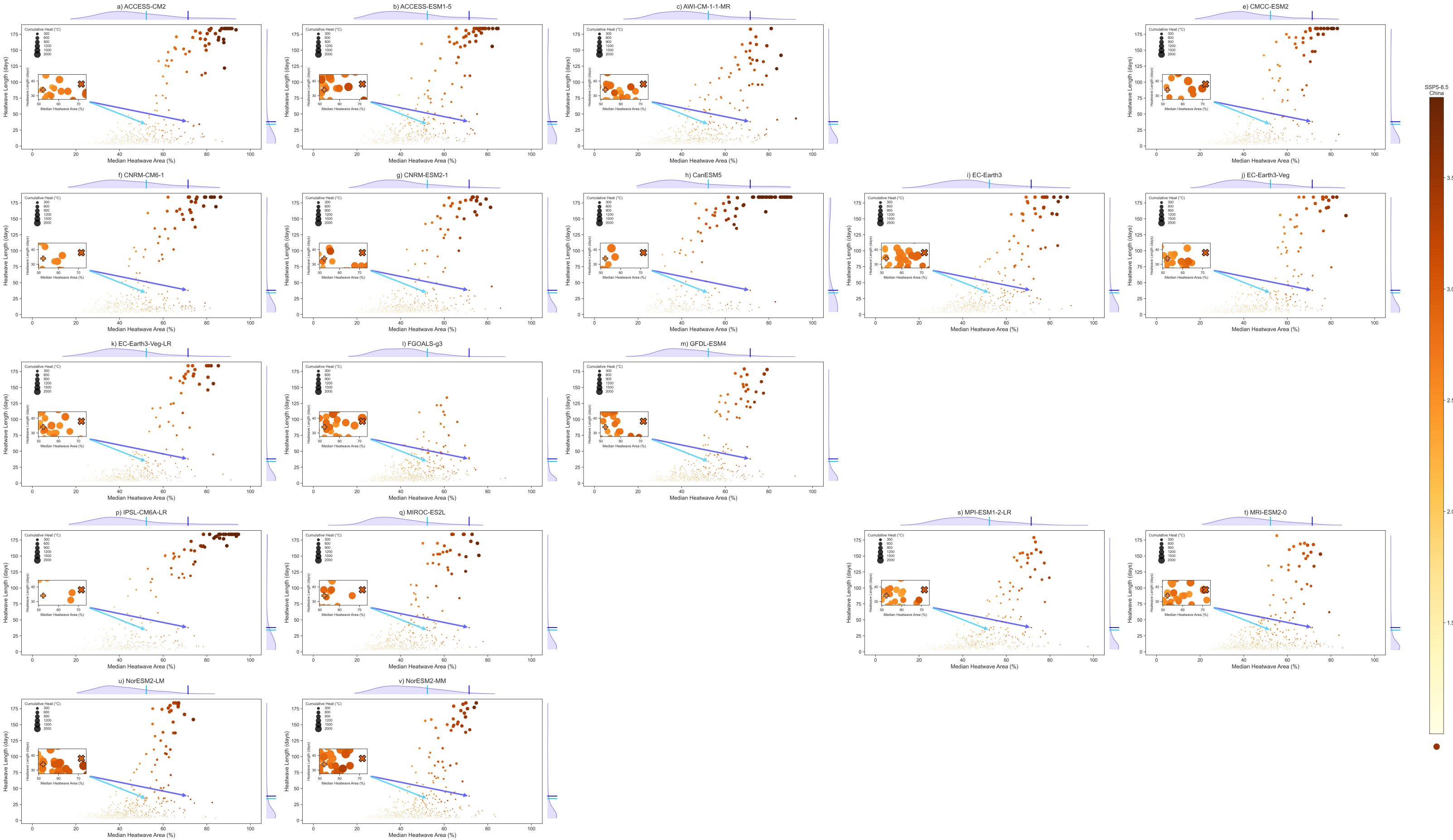

a) ACCESS-CM2
b) ACCESS-ESM1-5
c) AWI-CM-1-1-MR
e) CMCC-ESM2
f) CNRM-CM6-1
g) CNRM-ESM2-1
h) CanESM5
i) EC-Earth3
j) EC-Earth3-Veg
k) EC-Earth3-Veg-LR
l) FGOALS-g3
m) GFDL-ESM4
p) IPSL-CM6A-LR
q) MIROC-ES2L
s) MPI-ESM1-2-LR
t) MRI-ESM2-0
u) NorESM2-LM
v) NorESM2-MM
Cumulative Heat (°C)
300
600
900
1200
1500
2000
Heatwave Length (days)
Median Heatwave Area (%)
SSP5-8.5
China
Median Heatwave Severity
1.5
2.0
2.5
3.0
3.5

Supplementary Figure 276: Multi-HW-characteristic figure according to SSP5-8.5 CMIP6 multi-model ensemble (MME) per model for Central & Eastern Europe (Tx90p) - Each panel shows regional projections according to SSP5-8.5 model runs in the CMIP6 multi-model ensemble (MME) of four combined heatwave characteristics for each projected heatwave in the ensemble between 2020 and 2100. Each dot represents an individual heatwave in the projections, characterized by Median Heatwave Area (%) (x-axis) as latitude-weighted percentage of grid boxes affected by extreme heat, Heatwave Length (days) (y-axis) in days, Median Heatwave Severity (color), and Cumulative Heat (°C) (dot size). The characteristics of the observed event according to the Berkeley Earth observational data (x symbol) and the ERA5 reanalysis data (+ symbol), respectively, are pointed to by an arrow and a figure inset shows a zoom-in on this figure section. The figure inset shows the observed heatwave in the two reference data sets and projections in the indicated section of the respective sub-plot. It facilitates readability of color and size of the reference values and comparison to each other and the neighboring projections. Note that the zoom-in causes the dots to overlap more in the inset than in the main sub-plot. The univariate kernel density estimates along the x- (Median Heatwave Area (%)) and y-axis (Heatwave Length (days)) are on the top and right side of the subplots, respectively, with vertical lines in dark blue (Berkeley Earth) and light blue (ERA5) indicating the observed event. The respective region is indicated above the color bar.

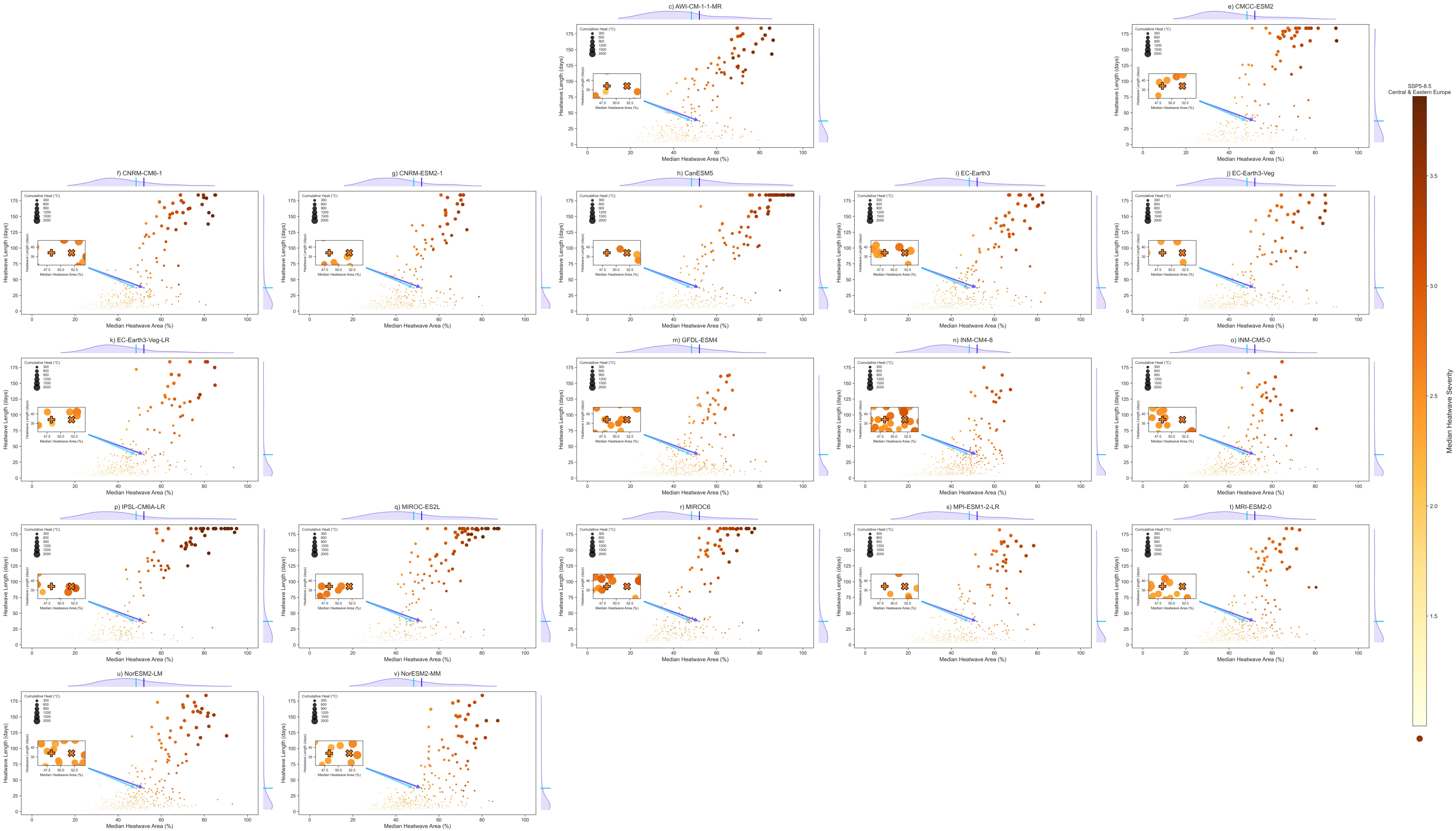

c) AWI-CM-1-1-MR
e) CMCC-ESM2
f) CNRM-CM6-1
g) CNRM-ESM2-1
h) CanESM5
i) EC-Earth3
j) EC-Earth3-Veg
k) EC-Earth3-Veg-LR
m) GFDL-ESM4
n) INM-CM4-8
o) INM-CM5-0
p) IPSL-CM6A-LR
q) MIROC-ES2L
r) MIROC6
s) MPI-ESM1-2-LR
t) MRI-ESM2-0
u) NorESM2-LM
v) NorESM2-MM
Cumulative Heat (°C)
300
600
900
1200
1500
2000
Heatwave Length (days)
Median Heatwave Area (%)
SSP5-8.5
Central & Eastern Europe
Median Heatwave Severity

Supplementary Figure 277: Multi-HW-characteristic figure according to SSP5-8.5 CMIP6 multi-model ensemble (MME) per model for Europe (Tx90p) - Each panel shows regional projections according to SSP5-8.5 model runs in the CMIP6 multi-model ensemble (MME) of four combined heatwave characteristics for each projected heatwave in the ensemble between 2020 and 2100. Each dot represents an individual heatwave in the projections, characterized by Median Heatwave Area (%) (x-axis) as latitude-weighted percentage of grid boxes affected by extreme heat, Heatwave Length (days) (y-axis) in days, Median Heatwave Severity (color), and Cumulative Heat (°C) (dot size). The characteristics of the observed event according to the Berkeley Earth observational data (x symbol) and the ERA5 reanalysis data (+ symbol), respectively, are pointed to by an arrow and a figure inset shows a zoom-in on this figure section. The figure inset shows the observed heatwave in the two reference data sets and projections in the indicated section of the respective sub-plot. It facilitates readability of color and size of the reference values and comparison to each other and the neighboring projections. Note that the zoom-in causes the dots to overlap more in the inset than in the main sub-plot. The univariate kernel density estimates along the x- (Median Heatwave Area (%)) and y-axis (Heatwave Length (days)) are on the top and right side of the subplots, respectively, with vertical lines in dark blue (Berkeley Earth) and light blue (ERA5) indicating the observed event. The respective region is indicated above the color bar.

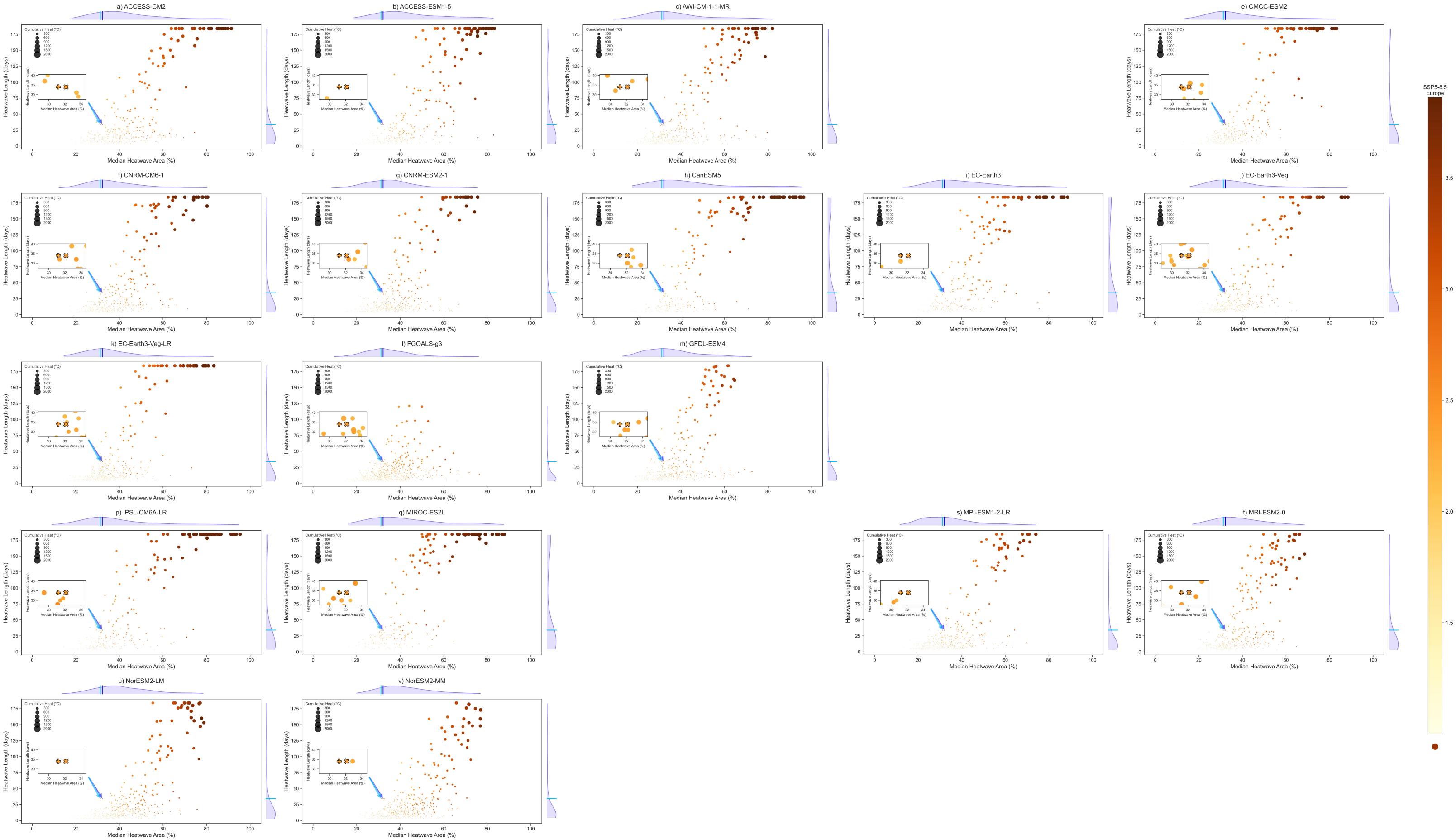

a) ACCESS-CM2
b) ACCESS-ESM1-5
c) AWI-CM-1-1-MR
e) CMCC-ESM2
f) CNRM-CM6-1
g) CNRM-ESM2-1
h) CanESM5
i) EC-Earth3
j) EC-Earth3-Veg
k) EC-Earth3-Veg-LR
l) FGOALS-g3
m) GFDL-ESM4
p) IPSL-CM6A-LR
q) MIROC-ES2L
s) MPI-ESM1-2-LR
t) MRI-ESM2-0
u) NorESM2-LM
v) NorESM2-MM
Cumulative Heat (°C)
300
600
900
1200
1500
2000
Heatwave Length (days)
Median Heatwave Area (%)
SSP5-8.5
Europe
Median Heatwave Severity

Supplementary Figure 278: Multi-HW-characteristic figure according to SSP5-8.5 CMIP6 multi-model ensemble (MME) per model for India & Middle East (Tx90p) - Each panel shows regional projections according to SSP5-8.5 model runs in the CMIP6 multi-model ensemble (MME) of four combined heatwave characteristics for each projected heatwave in the ensemble between 2020 and 2100. Each dot represents an individual heatwave in the projections, characterized by Median Heatwave Area (%) (x-axis) as latitude-weighted percentage of grid boxes affected by extreme heat, Heatwave Length (days) (y-axis) in days, Median Heatwave Severity (color), and Cumulative Heat (°C) (dot size). The characteristics of the observed event according to the Berkeley Earth observational data (x symbol) and the ERA5 reanalysis data (+ symbol), respectively, are pointed to by an arrow and a figure inset shows a zoom-in on this figure section. The figure inset shows the observed heatwave in the two reference data sets and projections in the indicated section of the respective sub-plot. It facilitates readability of color and size of the reference values and comparison to each other and the neighboring projections. Note that the zoom-in causes the dots to overlap more in the inset than in the main sub-plot. The univariate kernel density estimates along the x- (Median Heatwave Area (%)) and y-axis (Heatwave Length (days)) are on the top and right side of the subplots, respectively, with vertical lines in dark blue (Berkeley Earth) and light blue (ERA5) indicating the observed event. The respective region is indicated above the color bar.

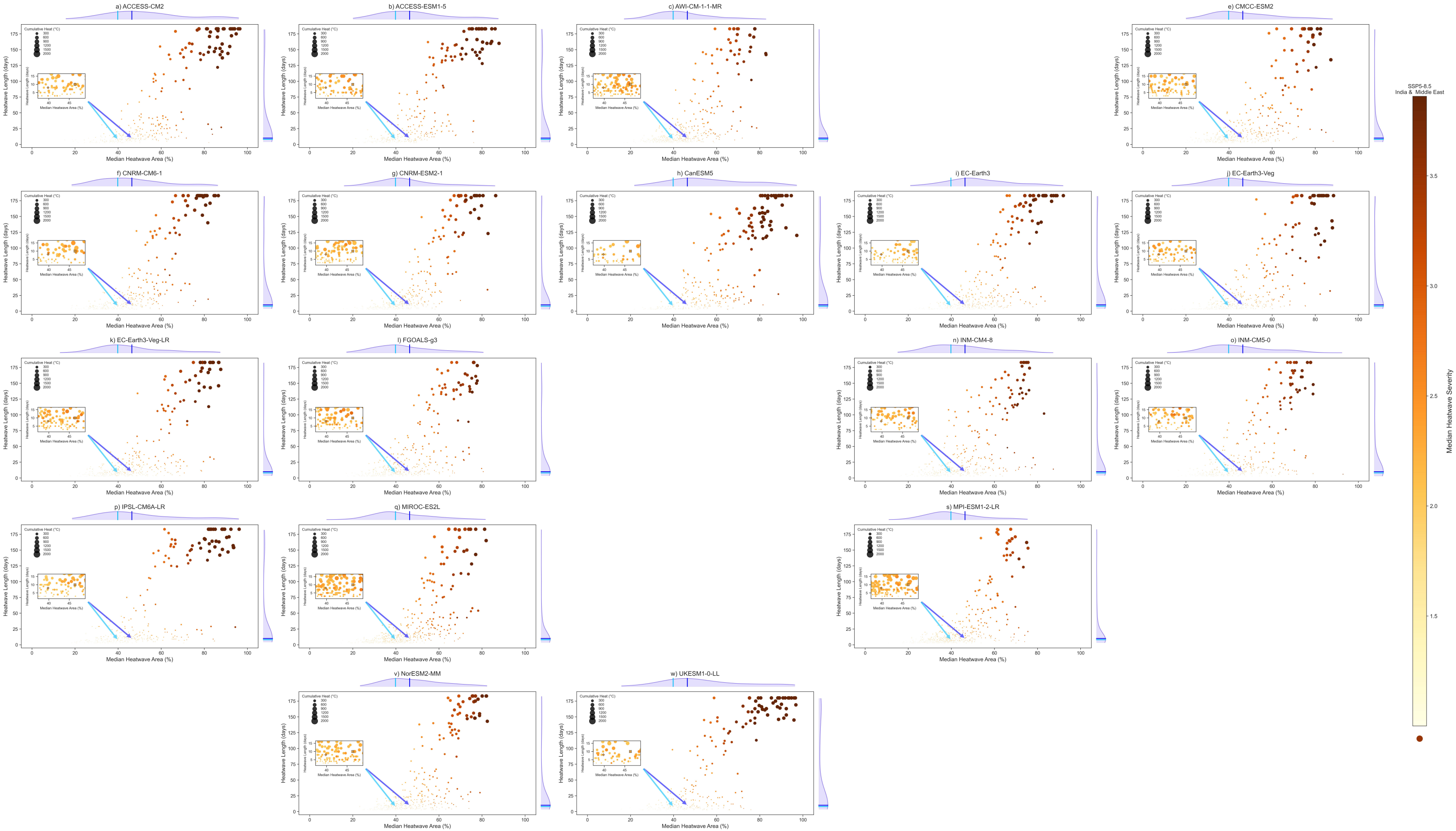

a) ACCESS-CM2
b) ACCESS-ESM1-5
c) AWI-CM-1-1-MR
e) CMCC-ESM2
f) CNRM-CM6-1
g) CNRM-ESM2-1
h) CanESM5
i) EC-Earth3
j) EC-Earth3-Veg
k) EC-Earth3-Veg-LR
l) FGOALS-g3
n) INM-CM4-8
o) INM-CM5-0
p) IPSL-CM6A-LR
q) MIROC-ES2L
s) MPI-ESM1-2-LR
v) NorESM2-MM
w) UKESM1-0-LL
Cumulative Heat (°C)
300
600
900
1200
1500
2000
Heatwave Length (days)
Median Heatwave Area (%)
SSP5-8.5
India & Middle East
Median Heatwave Severity

Supplementary Figure 279: Multi-HW-characteristic figure according to SSP5-8.5 CMIP6 multi-model ensemble (MME) per model for India & Pakistan (Tx90p) - Each panel shows regional projections according to SSP5-8.5 model runs in the CMIP6 multi-model ensemble (MME) of four combined heatwave characteristics for each projected heatwave in the ensemble between 2020 and 2100. Each dot represents an individual heatwave in the projections, characterized by Median Heatwave Area (%) (x-axis) as latitude-weighted percentage of grid boxes affected by extreme heat, Heatwave Length (days) (y-axis) in days, Median Heatwave Severity (color), and Cumulative Heat (°C) (dot size). The characteristics of the observed event according to the Berkeley Earth observational data (x symbol) and the ERA5 reanalysis data (+ symbol), respectively, are pointed to by an arrow and a figure inset shows a zoom-in on this figure section. The figure inset shows the observed heatwave in the two reference data sets and projections in the indicated section of the respective sub-plot. It facilitates readability of color and size of the reference values and comparison to each other and the neighboring projections. Note that the zoom-in causes the dots to overlap more in the inset than in the main sub-plot. The univariate kernel density estimates along the x- (Median Heatwave Area (%)) and y-axis (Heatwave Length (days)) are on the top and right side of the subplots, respectively, with vertical lines in dark blue (Berkeley Earth) and light blue (ERA5) indicating the observed event. The respective region is indicated above the color bar.

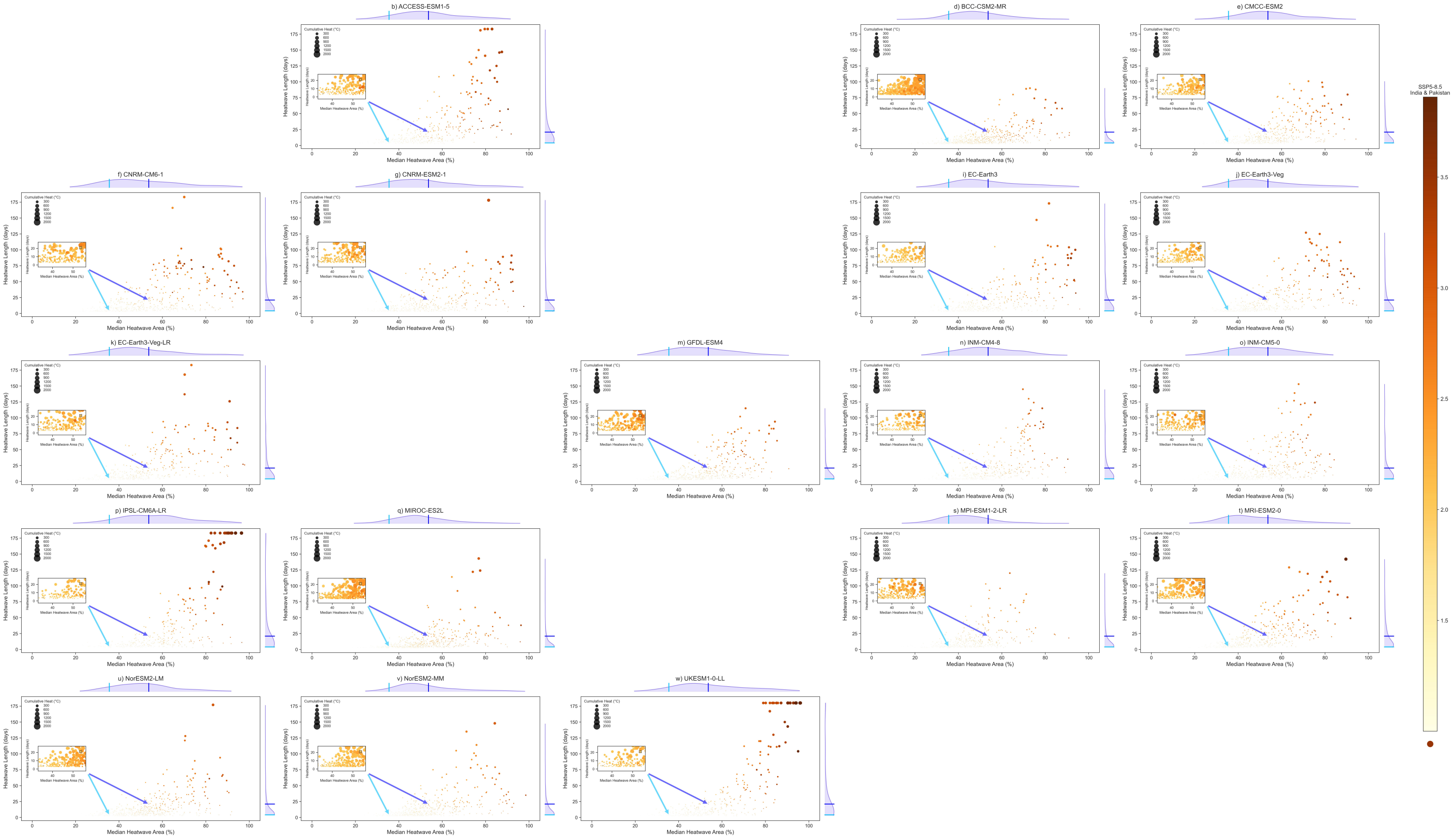

b) ACCESS-ESM1-5
d) BCC-CSM2-MR
e) CMCC-ESM2
f) CNRM-CM6-1
g) CNRM-ESM2-1
i) EC-Earth3
j) EC-Earth3-Veg
k) EC-Earth3-Veg-LR
m) GFDL-ESM4
n) INM-CM4-8
o) INM-CM5-0
p) IPSL-CM6A-LR
q) MIROC-ES2L
s) MPI-ESM1-2-LR
t) MRI-ESM2-0
u) NorESM2-LM
v) NorESM2-MM
w) UKESM1-0-LL
Heatwave Length (days)
Median Heatwave Area (%)
Cumulative Heat (°C)
300
600
900
1200
1500
2000
SSP5-8.5
India & Pakistan
Median Heatwave Severity

Supplementary Figure 280: Multi-HW-characteristic figure according to SSP5-8.5 CMIP6 multi-model ensemble (MME) per model for Middle East (Tx90p) - Each panel shows regional projections according to SSP5-8.5 model runs in the CMIP6 multi-model ensemble (MME) of four combined heatwave characteristics for each projected heatwave in the ensemble between 2020 and 2100. Each dot represents an individual heatwave in the projections, characterized by Median Heatwave Area (%) (x-axis) as latitude-weighted percentage of grid boxes affected by extreme heat, Heatwave Length (days) (y-axis) in days, Median Heatwave Severity (color), and Cumulative Heat (°C) (dot size). The characteristics of the observed event according to the Berkeley Earth observational data (x symbol) and the ERA5 reanalysis data (+ symbol), respectively, are pointed to by an arrow and a figure inset shows a zoom-in on this figure section. The figure inset shows the observed heatwave in the two reference data sets and projections in the indicated section of the respective sub-plot. It facilitates readability of color and size of the reference values and comparison to each other and the neighboring projections. Note that the zoom-in causes the dots to overlap more in the inset than in the main sub-plot. The univariate kernel density estimates along the x- (Median Heatwave Area (%)) and y-axis (Heatwave Length (days)) are on the top and right side of the subplots, respectively, with vertical lines in dark blue (Berkeley Earth) and light blue (ERA5) indicating the observed event. The respective region is indicated above the color bar.

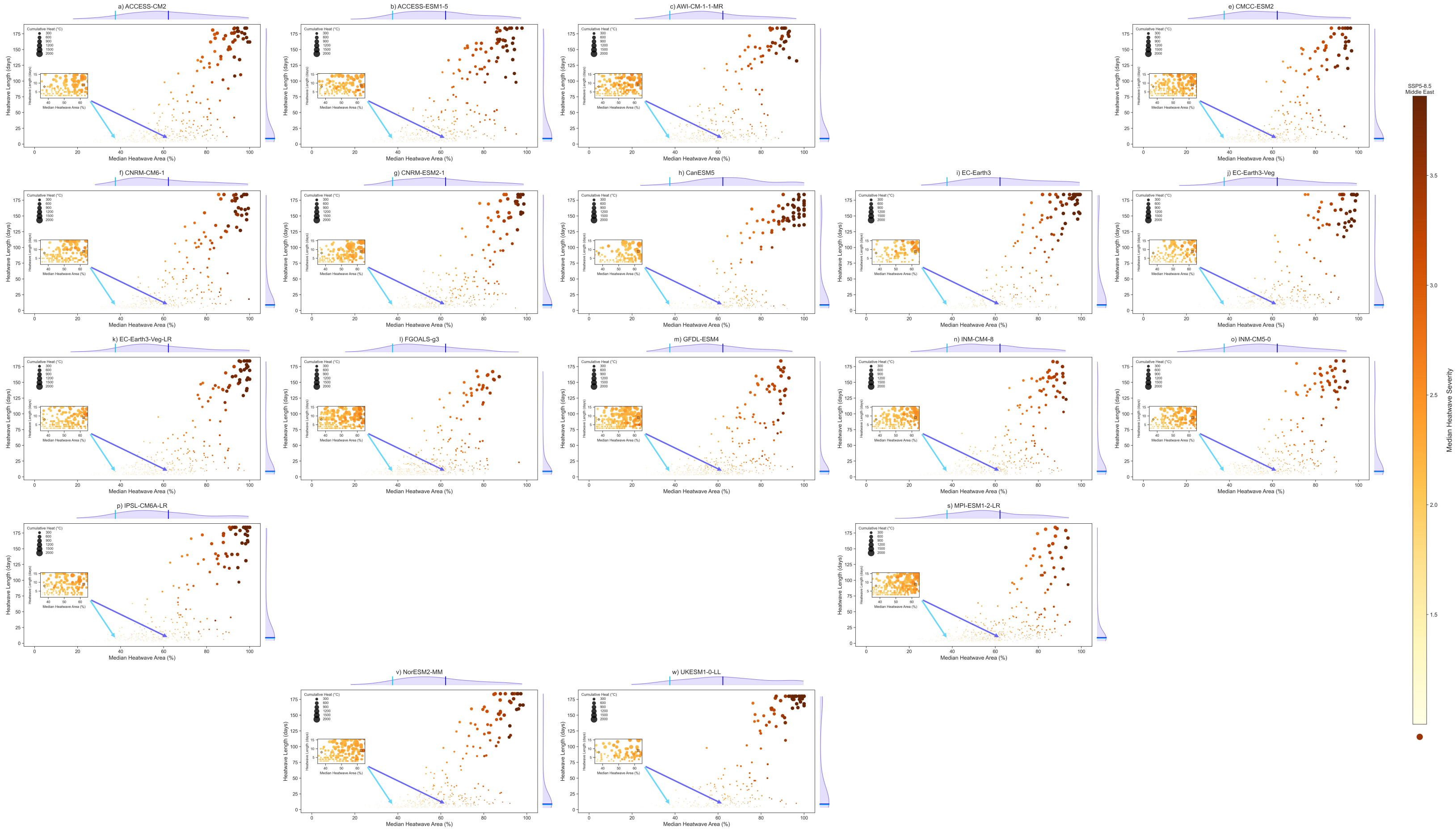

a) ACCESS-CM2
b) ACCESS-ESM1-5
c) AWI-CM-1-1-MR
e) CMCC-ESM2
f) CNRM-CM6-1
g) CNRM-ESM2-1
h) CanESM5
i) EC-Earth3
j) EC-Earth3-Veg
k) EC-Earth3-Veg-LR
l) FGOALS-g3
m) GFDL-ESM4
n) INM-CM4-8
o) INM-CM5-0
p) IPSL-CM6A-LR
s) MPI-ESM1-2-LR
v) NorESM2-MM
w) UKESM1-0-LL
Cumulative Heat (°C)
Heatwave Length (days)
Median Heatwave Area (%)
SSP5-8.5
Middle East
Median Heatwave Severity

Supplementary Figure 281: Multi-HW-characteristic figure according to SSP5-8.5 CMIP6 multi-model ensemble (MME) per model for Northeast Siberia (Tx90p) - Each panel shows regional projections according to SSP5-8.5 model runs in the CMIP6 multi-model ensemble (MME) of four combined heatwave characteristics for each projected heatwave in the ensemble between 2020 and 2100. Each dot represents an individual heatwave in the projections, characterized by Median Heatwave Area (%) (x-axis) as latitude-weighted percentage of grid boxes affected by extreme heat, Heatwave Length (days) (y-axis) in days, Median Heatwave Severity (color), and Cumulative Heat (°C) (dot size). The characteristics of the observed event according to the Berkeley Earth observational data (x symbol) and the ERA5 reanalysis data (+ symbol), respectively, are pointed to by an arrow and a figure inset shows a zoom-in on this figure section. The figure inset shows the observed heatwave in the two reference data sets and projections in the indicated section of the respective sub-plot. It facilitates readability of color and size of the reference values and comparison to each other and the neighboring projections. Note that the zoom-in causes the dots to overlap more in the inset than in the main sub-plot. The univariate kernel density estimates along the x- (Median Heatwave Area (%)) and y-axis (Heatwave Length (days)) are on the top and right side of the subplots, respectively, with vertical lines in dark blue (Berkeley Earth) and light blue (ERA5) indicating the observed event. The respective region is indicated above the color bar.

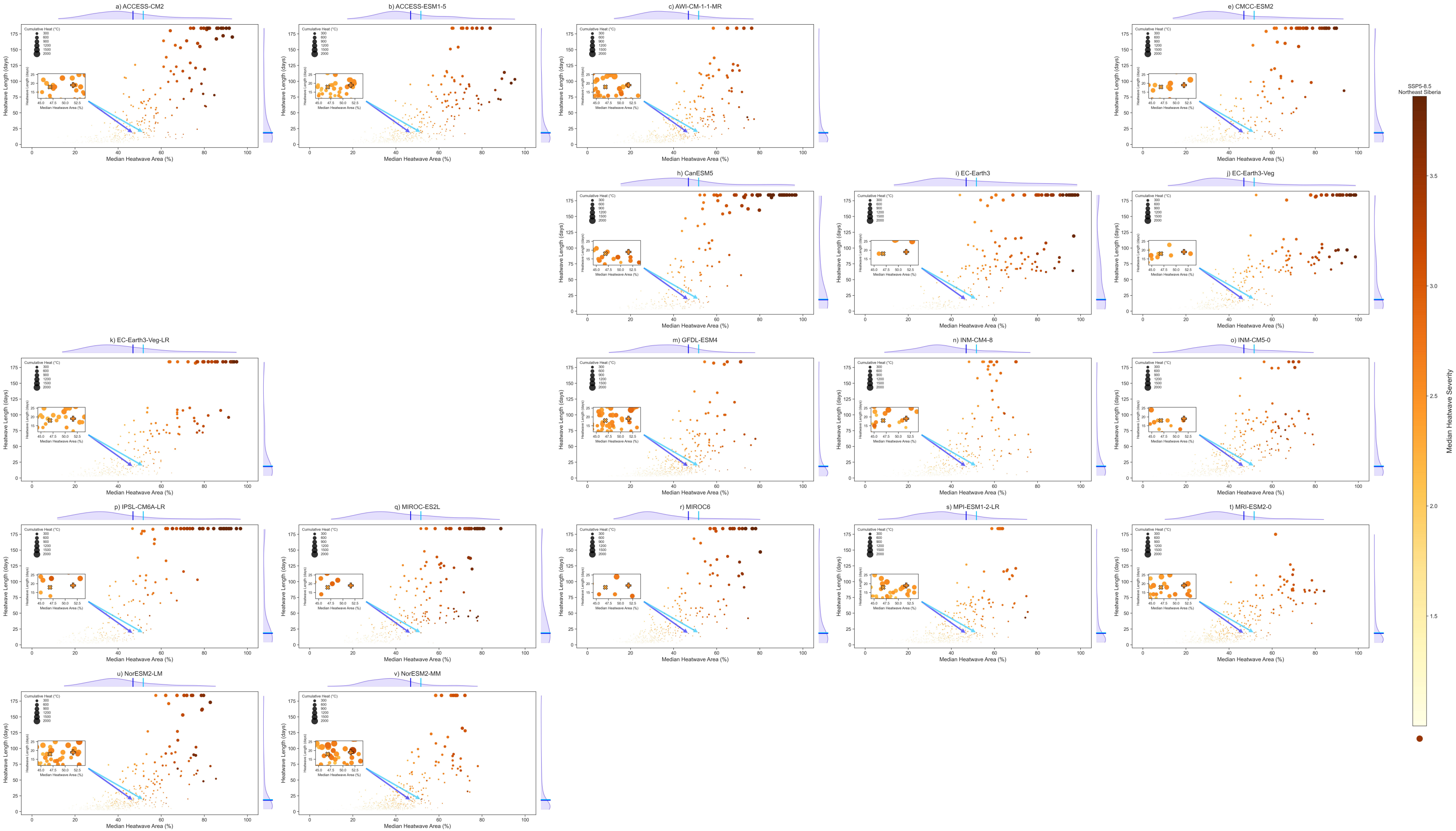

a) ACCESS-CM2
b) ACCESS-ESM1-5
c) AWI-CM-1-1-MR
e) CMCC-ESM2
h) CanESM5
i) EC-Earth3
j) EC-Earth3-Veg
k) EC-Earth3-Veg-LR
m) GFDL-ESM4
n) INM-CM4-8
o) INM-CM5-0
p) IPSL-CM6A-LR
q) MIROC-ES2L
r) MIROC6
s) MPI-ESM1-2-LR
t) MRI-ESM2-0
u) NorESM2-LM
v) NorESM2-MM
Cumulative Heat (°C)
300
600
900
1200
1500
2000
Heatwave Length (days)
Median Heatwave Area (%)
SSP5-8.5
Northeast Siberia
Median Heatwave Severity

Supplementary Figure 282: Multi-HW-characteristic figure according to SSP5-8.5 CMIP6 multi-model ensemble (MME) per model for Northern Africa & Middle East (Tx90p) - Each panel shows regional projections according to SSP5-8.5 model runs in the CMIP6 multi-model ensemble (MME) of four combined heatwave characteristics for each projected heatwave in the ensemble between 2020 and 2100. Each dot represents an individual heatwave in the projections, characterized by Median Heatwave Area (%) (x-axis) as latitude-weighted percentage of grid boxes affected by extreme heat, Heatwave Length (days) (y-axis) in days, Median Heatwave Severity (color), and Cumulative Heat (°C) (dot size). The characteristics of the observed event according to the Berkeley Earth observational data (x symbol) and the ERA5 reanalysis data (+ symbol), respectively, are pointed to by an arrow and a figure inset shows a zoom-in on this figure section. The figure inset shows the observed heatwave in the two reference data sets and projections in the indicated section of the respective sub-plot. It facilitates readability of color and size of the reference values and comparison to each other and the neighboring projections. Note that the zoom-in causes the dots to overlap more in the inset than in the main sub-plot. The univariate kernel density estimates along the x- (Median Heatwave Area (%)) and y-axis (Heatwave Length (days)) are on the top and right side of the subplots, respectively, with vertical lines in dark blue (Berkeley Earth) and light blue (ERA5) indicating the observed event. The respective region is indicated above the color bar.

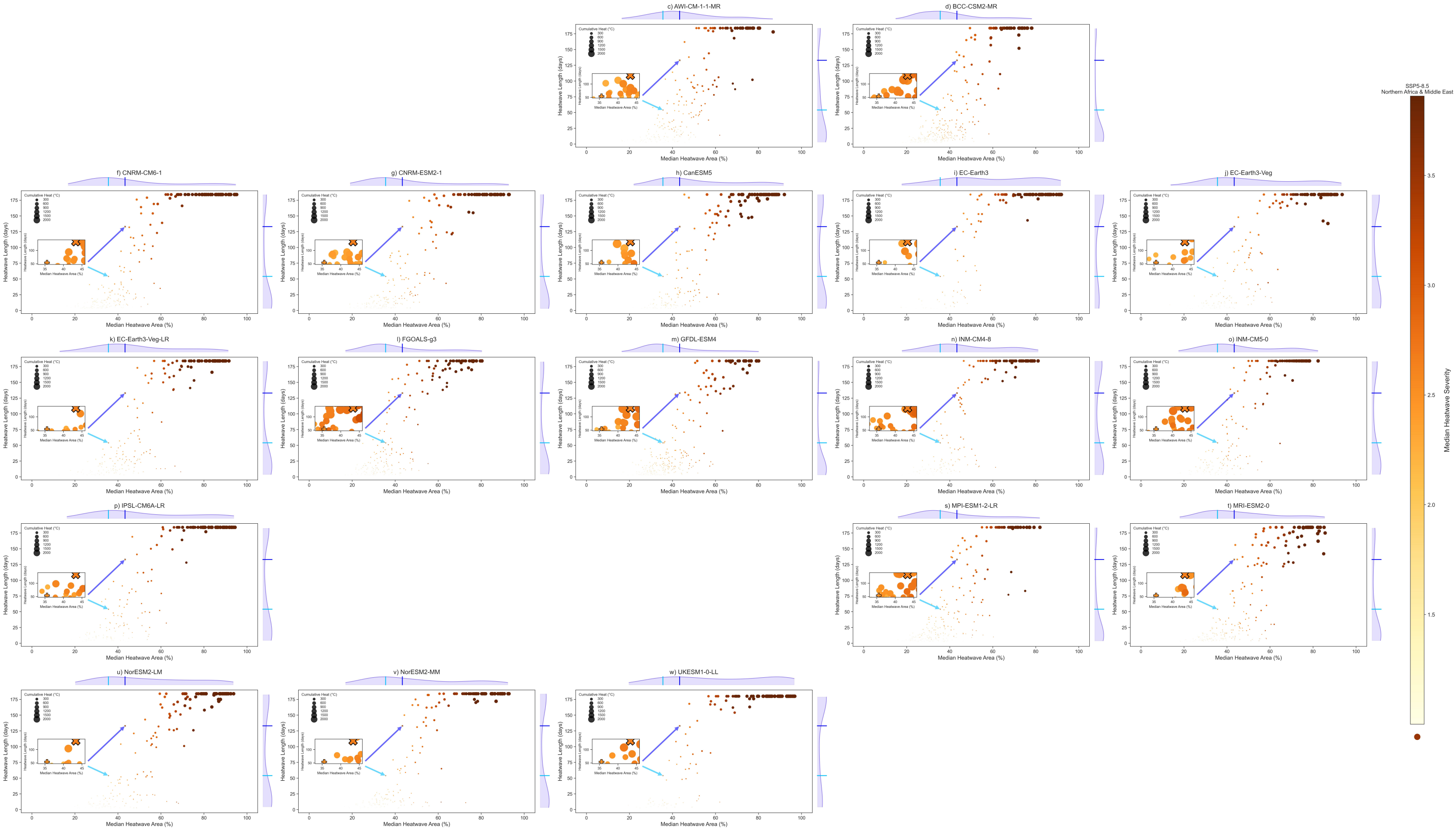

c) AWI-CM-1-1-MR
d) BCC-CSM2-MR
f) CNRM-CM6-1
g) CNRM-ESM2-1
h) CanESM5
i) EC-Earth3
j) EC-Earth3-Veg
k) EC-Earth3-Veg-LR
l) FGOALS-g3
m) GFDL-ESM4
n) INM-CM4-8
o) INM-CM5-0
p) IPSL-CM6A-LR
s) MPI-ESM1-2-LR
t) MRI-ESM2-0
u) NorESM2-LM
v) NorESM2-MM
w) UKESM1-0-LL
Cumulative Heat (°C)
300
600
900
1200
1500
2000
Heatwave Length (days)
Median Heatwave Area (%)
SSP5-8.5
Northern Africa & Middle East
Median Heatwave Severity

Supplementary Figure 283: Multi-HW-characteristic figure according to SSP5-8.5 CMIP6 multi-model ensemble (MME) per model for Pacific Northwest USA & Canada (Tx90p) - Each panel shows regional projections according to SSP5-8.5 model runs in the CMIP6 multi-model ensemble (MME) of four combined heatwave characteristics for each projected heatwave in the ensemble between 2020 and 2100. Each dot represents an individual heatwave in the projections, characterized by Median Heatwave Area (%) (x-axis) as latitude-weighted percentage of grid boxes affected by extreme heat, Heatwave Length (days) (y-axis) in days, Median Heatwave Severity (color), and Cumulative Heat (°C) (dot size). The characteristics of the observed event according to the Berkeley Earth observational data (x symbol) and the ERA5 reanalysis data (+ symbol), respectively, are pointed to by an arrow and a figure inset shows a zoom-in on this figure section. The figure inset shows the observed heatwave in the two reference data sets and projections in the indicated section of the respective sub-plot. It facilitates readability of color and size of the reference values and comparison to each other and the neighboring projections. Note that the zoom-in causes the dots to overlap more in the inset than in the main sub-plot. The univariate kernel density estimates along the x- (Median Heatwave Area (%)) and y-axis (Heatwave Length (days)) are on the top and right side of the subplots, respectively, with vertical lines in dark blue (Berkeley Earth) and light blue (ERA5) indicating the observed event. The respective region is indicated above the color bar.

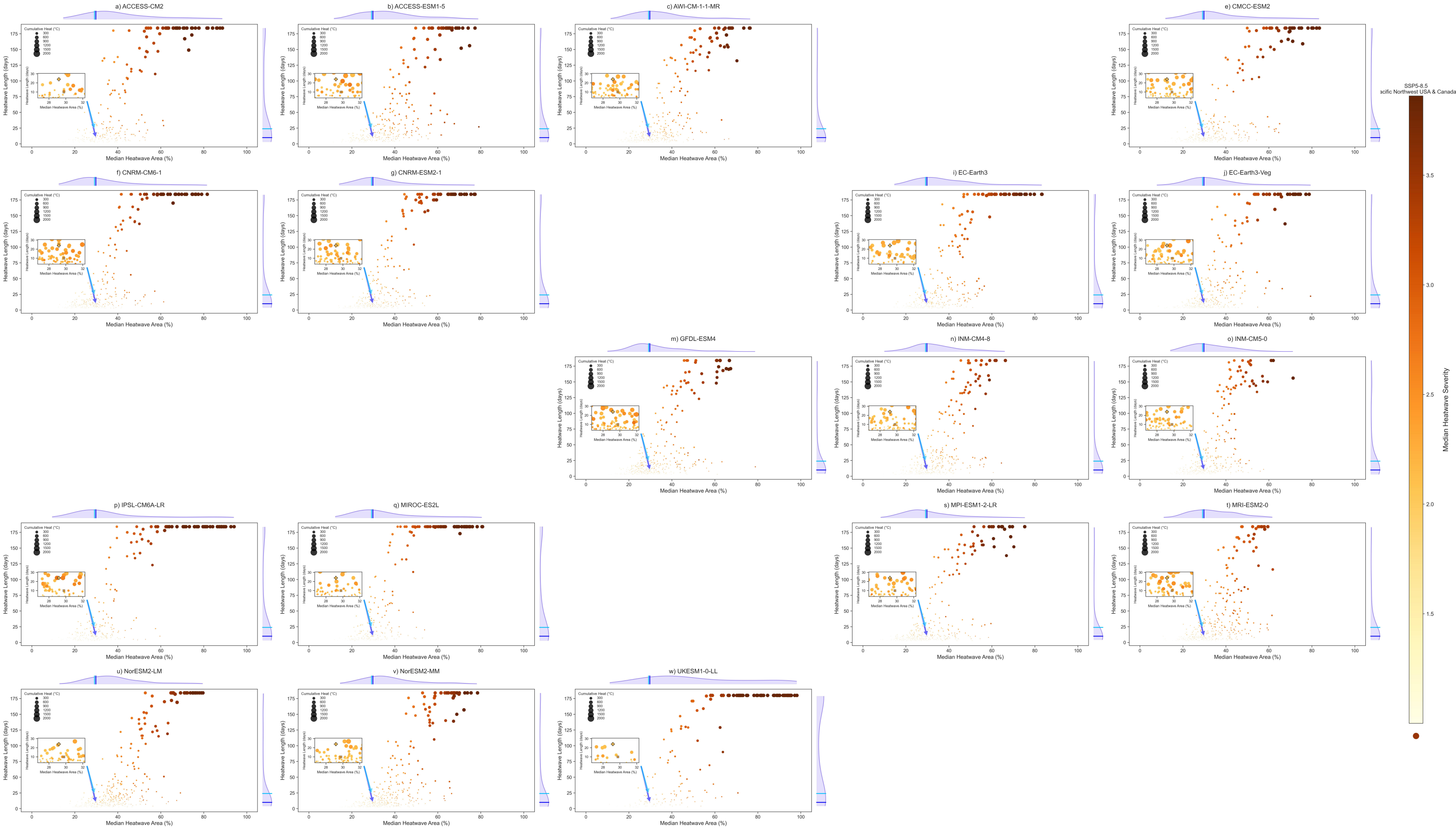

a) ACCESS-CM2
b) ACCESS-ESM1-5
c) AWI-CM-1-1-MR
e) CMCC-ESM2
f) CNRM-CM6-1
g) CNRM-ESM2-1
i) EC-Earth3
j) EC-Earth3-Veg
m) GFDL-ESM4
n) INM-CM4-8
o) INM-CM5-0
p) IPSL-CM6A-LR
q) MIROC-ES2L
s) MPI-ESM1-2-LR
t) MRI-ESM2-0
u) NorESM2-LM
v) NorESM2-MM
w) UKESM1-0-LL
Heatwave Length (days)
Median Heatwave Area (%)
Cumulative Heat (°C)
300
600
900
1200
1500
2000
SSP5-8.5
acific Northwest USA & Canada
Median Heatwave Severity

Supplementary Figure 284: Multi-HW-characteristic figure according to SSP5-8.5 CMIP6 multi-model ensemble (MME) per model for Russia (Tx90p) - Each panel shows regional projections according to SSP5-8.5 model runs in the CMIP6 multi-model ensemble (MME) of four combined heatwave characteristics for each projected heatwave in the ensemble between 2020 and 2100. Each dot represents an individual heatwave in the projections, characterized by Median Heatwave Area (%) (x-axis) as latitude-weighted percentage of grid boxes affected by extreme heat, Heatwave Length (days) (y-axis) in days, Median Heatwave Severity (color), and Cumulative Heat (°C) (dot size). The characteristics of the observed event according to the Berkeley Earth observational data (x symbol) and the ERA5 reanalysis data (+ symbol), respectively, are pointed to by an arrow and a figure inset shows a zoom-in on this figure section. The figure inset shows the observed heatwave in the two reference data sets and projections in the indicated section of the respective sub-plot. It facilitates readability of color and size of the reference values and comparison to each other and the neighboring projections. Note that the zoom-in causes the dots to overlap more in the inset than in the main sub-plot. The univariate kernel density estimates along the x- (Median Heatwave Area (%)) and y-axis (Heatwave Length (days)) are on the top and right side of the subplots, respectively, with vertical lines in dark blue (Berkeley Earth) and light blue (ERA5) indicating the observed event. The respective region is indicated above the color bar.

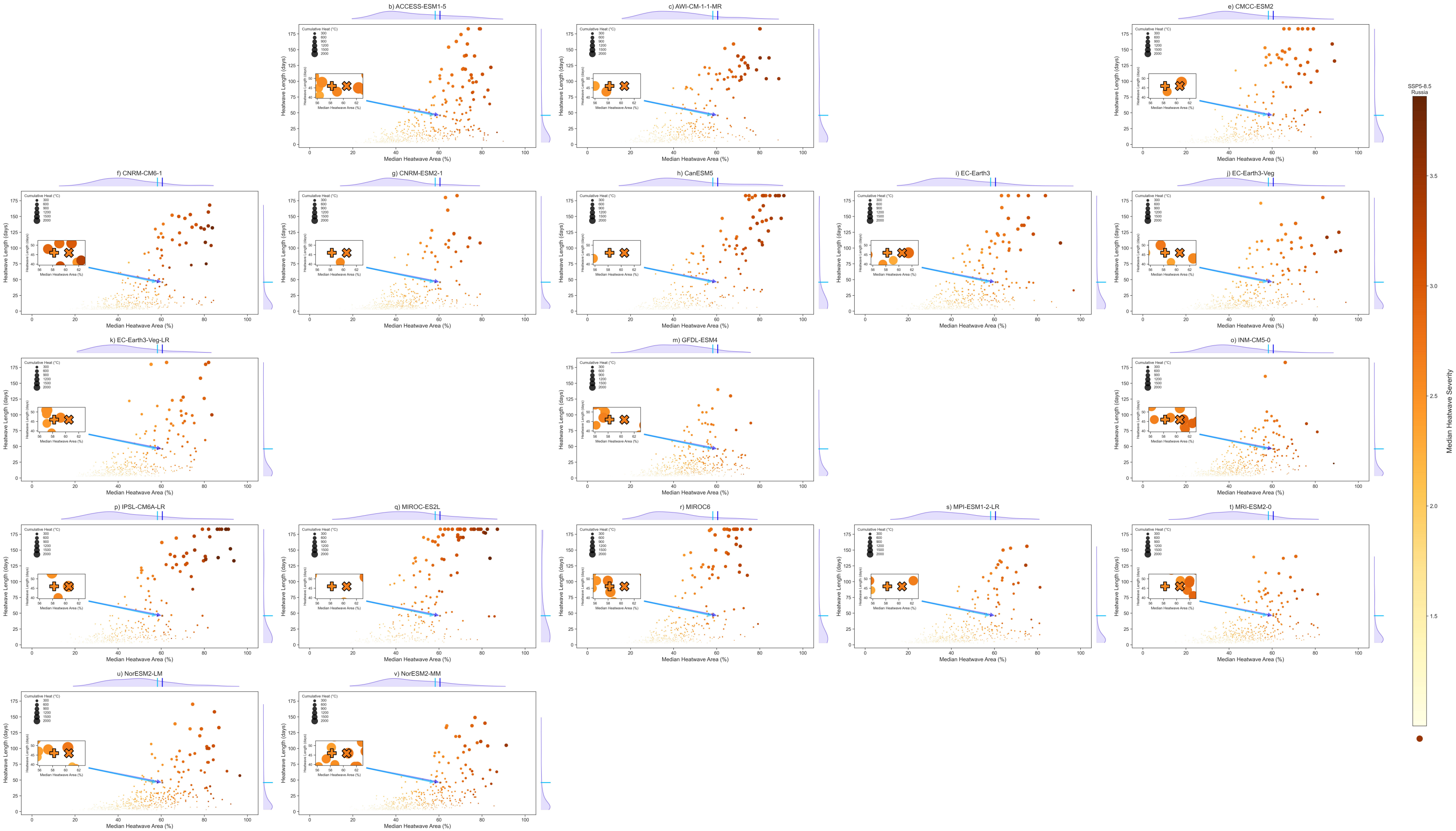
b) ACCESS-ESM1-5
c) AWI-CM-1-1-MR
e) CMCC-ESM2
f) CNRM-CM6-1
g) CNRM-ESM2-1
h) CanESM5
i) EC-Earth3
j) EC-Earth3-Veg
k) EC-Earth3-Veg-LR
m) GFDL-ESM4
o) INM-CM5-0
p) IPSL-CM6A-LR
q) MIROC-ES2L
r) MIROC6
s) MPI-ESM1-2-LR
t) MRI-ESM2-0
u) NorESM2-LM
v) NorESM2-MM
Cumulative Heat (°C)
300
600
900
1200
1500
2000
Heatwave Length (days)
Median Heatwave Area (%)
SSP5-8.5
Russia
Median Heatwave Severity
1.5
2.0
2.5
3.0
3.5

Supplementary Figure 285: Multi-HW-characteristic figure according to SSP5-8.5 CMIP6 multi-model ensemble (MME) per model for Southwestern USA & Mexico (Tx90p) - Each panel shows regional projections according to SSP5-8.5 model runs in the CMIP6 multi-model ensemble (MME) of four combined heatwave characteristics for each projected heatwave in the ensemble between 2020 and 2100. Each dot represents an individual heatwave in the projections, characterized by Median Heatwave Area (%) (x-axis) as latitude-weighted percentage of grid boxes affected by extreme heat, Heatwave Length (days) (y-axis) in days, Median Heatwave Severity (color), and Cumulative Heat (°C) (dot size). The characteristics of the observed event according to the Berkeley Earth observational data (x symbol) and the ERA5 reanalysis data (+ symbol), respectively, are pointed to by an arrow and a figure inset shows a zoom-in on this figure section. The figure inset shows the observed heatwave in the two reference data sets and projections in the indicated section of the respective sub-plot. It facilitates readability of color and size of the reference values and comparison to each other and the neighboring projections. Note that the zoom-in causes the dots to overlap more in the inset than in the main sub-plot. The univariate kernel density estimates along the x- (Median Heatwave Area (%)) and y-axis (Heatwave Length (days)) are on the top and right side of the subplots, respectively, with vertical lines in dark blue (Berkeley Earth) and light blue (ERA5) indicating the observed event. The respective region is indicated above the color bar.

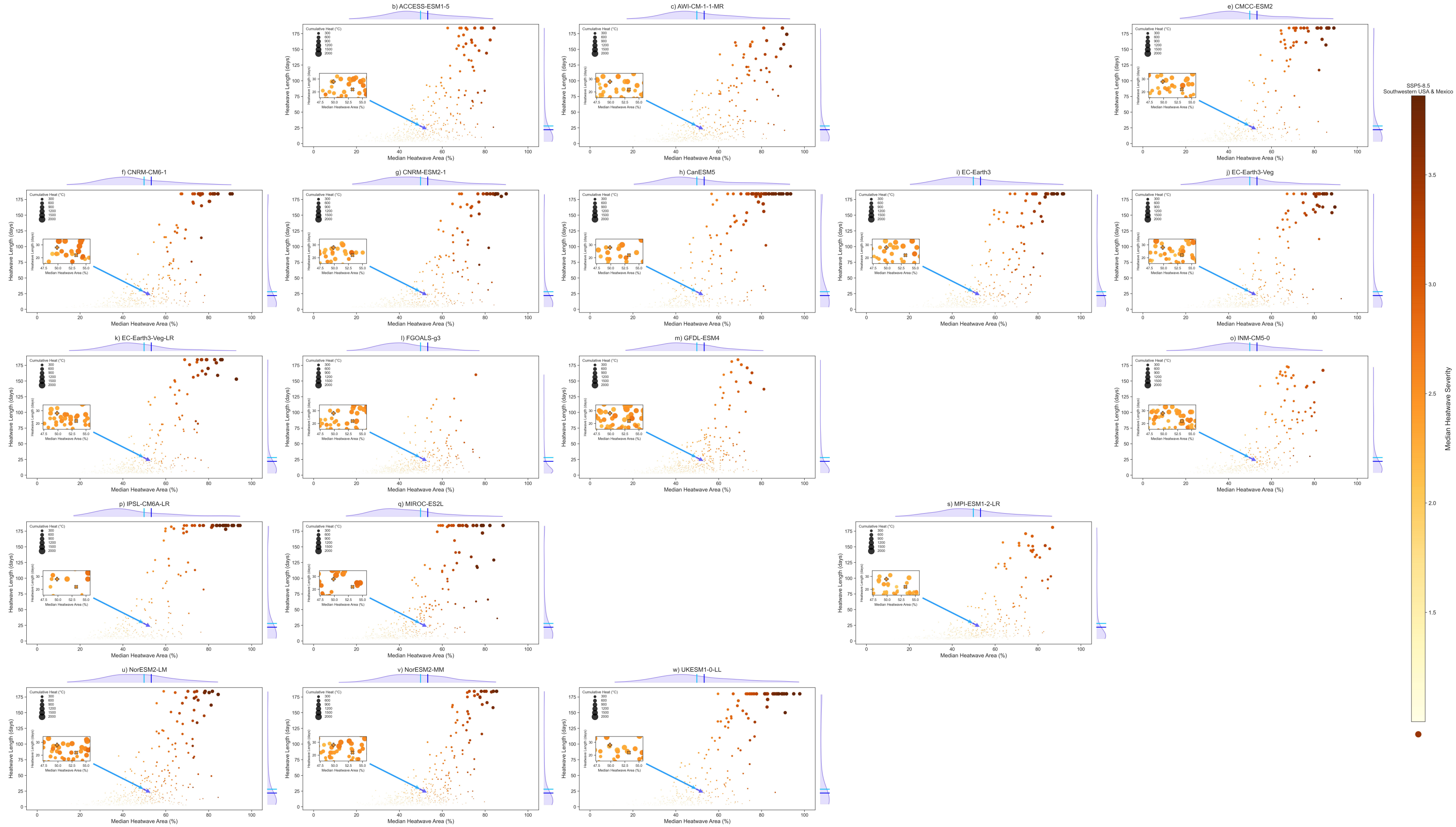

b) ACCESS-ESM1-5
c) AWI-CM-1-1-MR
e) CMCC-ESM2
f) CNRM-CM6-1
g) CNRM-ESM2-1
h) CanESM5
i) EC-Earth3
j) EC-Earth3-Veg
k) EC-Earth3-Veg-LR
l) FGOALS-g3
m) GFDL-ESM4
o) INM-CM5-0
p) IPSL-CM6A-LR
q) MIROC-ES2L
s) MPI-ESM1-2-LR
u) NorESM2-LM
v) NorESM2-MM
w) UKESM1-0-LL
Cumulative Heat (°C)
300
600
900
1200
1500
2000
Heatwave Length (days)
Median Heatwave Area (%)
SSP5-8.5
Southwestern USA & Mexico
Median Heatwave Severity

Supplementary Figure 286: Multi-HW-characteristic figure according to SSP5-8.5 CMIP6 multi-model ensemble (MME) per model for Siberia (Tx90p) - Each panel shows regional projections according to SSP5-8.5 model runs in the CMIP6 multi-model ensemble (MME) of four combined heatwave characteristics for each projected heatwave in the ensemble between 2020 and 2100. Each dot represents an individual heatwave in the projections, characterized by Median Heatwave Area (%) (x-axis) as latitude-weighted percentage of grid boxes affected by extreme heat, Heatwave Length (days) (y-axis) in days, Median Heatwave Severity (color), and Cumulative Heat (°C) (dot size). The characteristics of the observed event according to the Berkeley Earth observational data (x symbol) and the ERA5 reanalysis data (+ symbol), respectively, are pointed to by an arrow and a figure inset shows a zoom-in on this figure section. The figure inset shows the observed heatwave in the two reference data sets and projections in the indicated section of the respective sub-plot. It facilitates readability of color and size of the reference values and comparison to each other and the neighboring projections. Note that the zoom-in causes the dots to overlap more in the inset than in the main sub-plot. The univariate kernel density estimates along the x- (Median Heatwave Area (%)) and y-axis (Heatwave Length (days)) are on the top and right side of the subplots, respectively, with vertical lines in dark blue (Berkeley Earth) and light blue (ERA5) indicating the observed event. The respective region is indicated above the color bar.

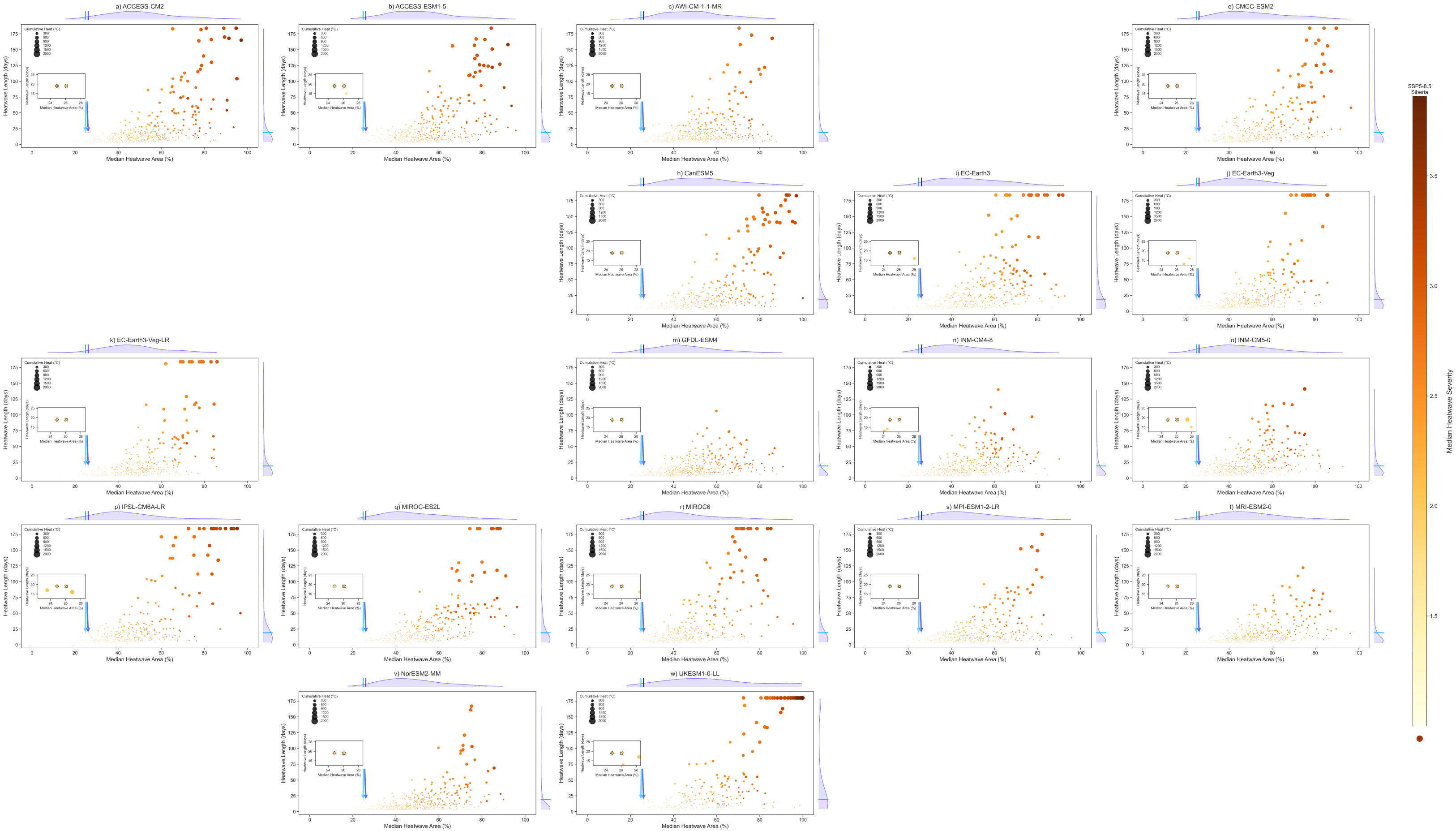

a) ACCESS-CM2
b) ACCESS-ESM1-5
c) AWI-CM-1-1-MR
e) CMCC-ESM2
h) CanESM5
i) EC-Earth3
j) EC-Earth3-Veg
k) EC-Earth3-Veg-LR
m) GFDL-ESM4
n) INM-CM4-8
o) INM-CM5-0
p) IPSL-CM6A-LR
q) MIROC-ES2L
r) MIROC6
s) MPI-ESM1-2-LR
t) MRI-ESM2-0
v) NorESM2-MM
w) UKESM1-0-LL
Cumulative Heat (°C)
300
600
900
1200
1500
2000
Heatwave Length (days)
Median Heatwave Area (%)
SSP5-8.5
Siberia
Median Heatwave Severity
1.5
2.0
2.5
3.0
3.5

Supplementary Figure 287: Multi-HW-characteristic figure according to SSP5-8.5 CMIP6 multi-model ensemble (MME) per model for South America (Tx90p) - Each panel shows regional projections according to SSP5-8.5 model runs in the CMIP6 multi-model ensemble (MME) of four combined heatwave characteristics for each projected heatwave in the ensemble between 2020 and 2100. Each dot represents an individual heatwave in the projections, characterized by Median Heatwave Area (%) (x-axis) as latitude-weighted percentage of grid boxes affected by extreme heat, Heatwave Length (days) (y-axis) in days, Median Heatwave Severity (color), and Cumulative Heat (°C) (dot size). The characteristics of the observed event according to the Berkeley Earth observational data (x symbol) and the ERA5 reanalysis data (+ symbol), respectively, are pointed to by an arrow and a figure inset shows a zoom-in on this figure section. The figure inset shows the observed heatwave in the two reference data sets and projections in the indicated section of the respective sub-plot. It facilitates readability of color and size of the reference values and comparison to each other and the neighboring projections. Note that the zoom-in causes the dots to overlap more in the inset than in the main sub-plot. The univariate kernel density estimates along the x- (Median Heatwave Area (%)) and y-axis (Heatwave Length (days)) are on the top and right side of the subplots, respectively, with vertical lines in dark blue (Berkeley Earth) and light blue (ERA5) indicating the observed event. The respective region is indicated above the color bar.

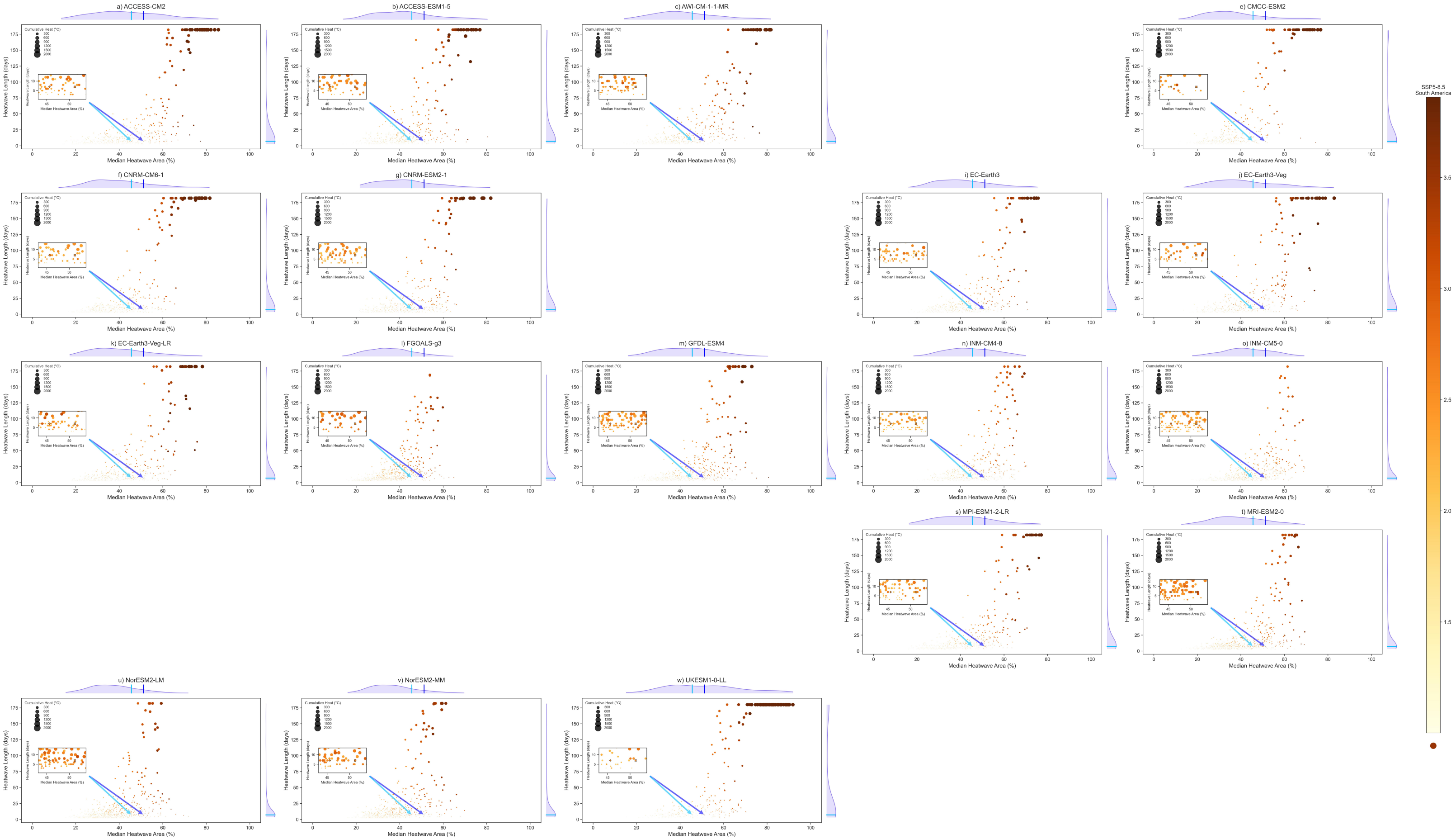

a) ACCESS-CM2
b) ACCESS-ESM1-5
c) AWI-CM-1-1-MR
e) CMCC-ESM2
f) CNRM-CM6-1
g) CNRM-ESM2-1
i) EC-Earth3
j) EC-Earth3-Veg
k) EC-Earth3-Veg-LR
l) FGOALS-g3
m) GFDL-ESM4
n) INM-CM4-8
o) INM-CM5-0
s) MPI-ESM1-2-LR
t) MRI-ESM2-0
u) NorESM2-LM
v) NorESM2-MM
w) UKESM1-0-LL
Heatwave Length (days)
Median Heatwave Area (%)
Cumulative Heat (°C)
300
600
900
1200
1500
2000
SSP5-8.5
South America
Median Heatwave Severity

Supplementary Figure 288: Multi-HW-characteristic figure according to SSP5-8.5 CMIP6 multi-model ensemble (MME) per model for Northern Southeast Asia (Tx90p) - Each panel shows regional projections according to SSP5-8.5 model runs in the CMIP6 multi-model ensemble (MME) of four combined heatwave characteristics for each projected heatwave in the ensemble between 2020 and 2100. Each dot represents an individual heatwave in the projections, characterized by Median Heatwave Area (%) (x-axis) as latitude-weighted percentage of grid boxes affected by extreme heat, Heatwave Length (days) (y-axis) in days, Median Heatwave Severity (color), and Cumulative Heat (°C) (dot size). The characteristics of the observed event according to the Berkeley Earth observational data (x symbol) and the ERA5 reanalysis data (+ symbol), respectively, are pointed to by an arrow and a figure inset shows a zoom-in on this figure section. The figure inset shows the observed heatwave in the two reference data sets and projections in the indicated section of the respective sub-plot. It facilitates readability of color and size of the reference values and comparison to each other and the neighboring projections. Note that the zoom-in causes the dots to overlap more in the inset than in the main sub-plot. The univariate kernel density estimates along the x- (Median Heatwave Area (%)) and y-axis (Heatwave Length (days)) are on the top and right side of the subplots, respectively, with vertical lines in dark blue (Berkeley Earth) and light blue (ERA5) indicating the observed event. The respective region is indicated above the color bar.

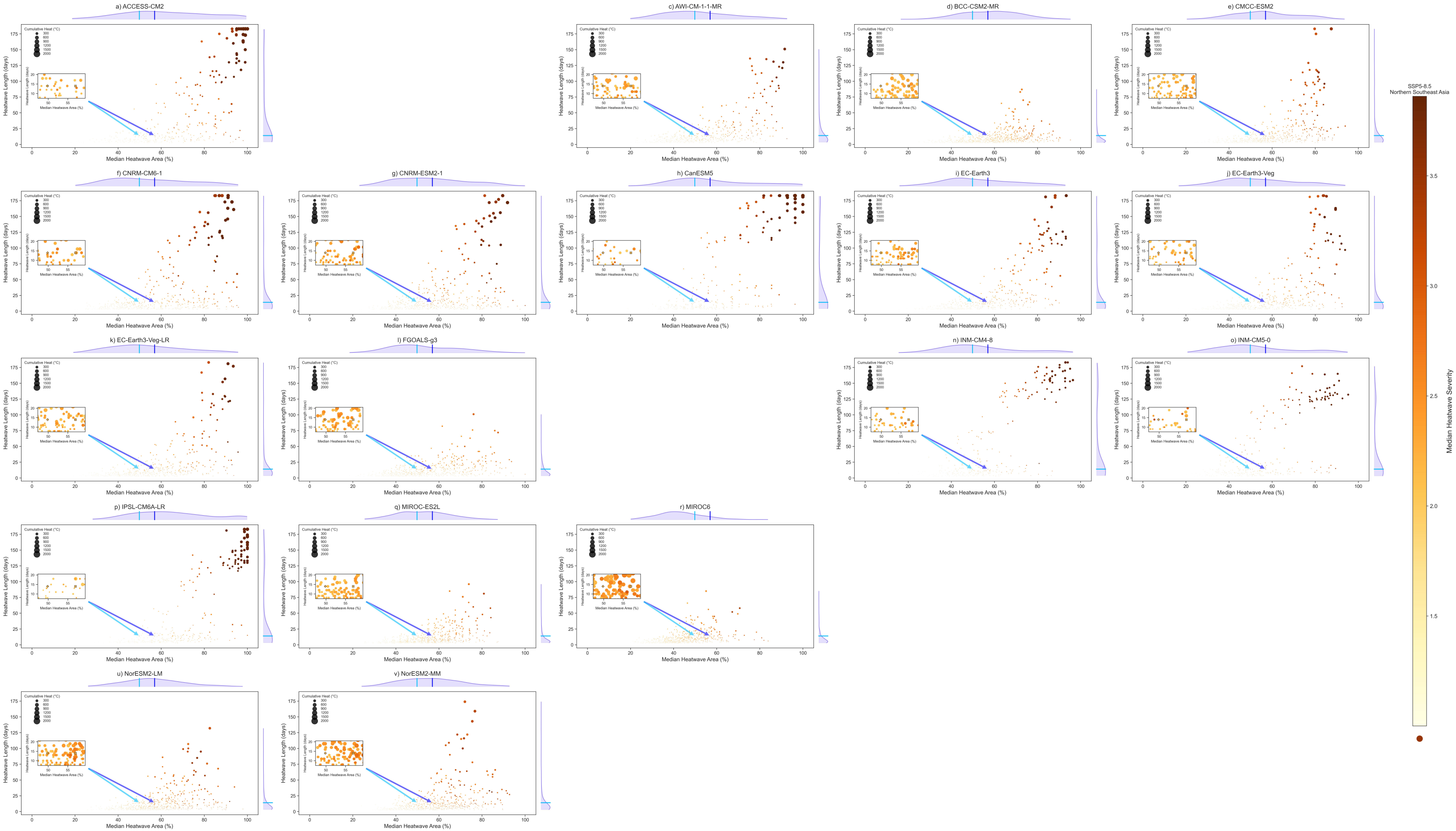
a) ACCESS-CM2
c) AWI-CM-1-1-MR
d) BCC-CSM2-MR
e) CMCC-ESM2
f) CNRM-CM6-1
g) CNRM-ESM2-1
h) CanESM5
i) EC-Earth3
j) EC-Earth3-Veg
k) EC-Earth3-Veg-LR
l) FGOALS-g3
n) INM-CM4-8
o) INM-CM5-0
p) IPSL-CM6A-LR
q) MIROC-ES2L
r) MIROC6
u) NorESM2-LM
v) NorESM2-MM
Cumulative Heat (°C)
300
600
900
1200
1500
2000
Heatwave Length (days)
Median Heatwave Area (%)
SSP5-8.5
Northern Southeast Asia
Median Heatwave Severity

Supplementary Figure 289: Multi-HW-characteristic figure according to SSP5-8.5 CMIP6 multi-model ensemble (MME) per model for Southern Africa (Tx90p) - Each panel shows regional projections according to SSP5-8.5 model runs in the CMIP6 multi-model ensemble (MME) of four combined heatwave characteristics for each projected heatwave in the ensemble between 2020 and 2100. Each dot represents an individual heatwave in the projections, characterized by Median Heatwave Area (%) (x-axis) as latitude-weighted percentage of grid boxes affected by extreme heat, Heatwave Length (days) (y-axis) in days, Median Heatwave Severity (color), and Cumulative Heat (°C) (dot size). The characteristics of the observed event according to the Berkeley Earth observational data (x symbol) and the ERA5 reanalysis data (+ symbol), respectively, are pointed to by an arrow and a figure inset shows a zoom-in on this figure section. The figure inset shows the observed heatwave in the two reference data sets and projections in the indicated section of the respective sub-plot. It facilitates readability of color and size of the reference values and comparison to each other and the neighboring projections. Note that the zoom-in causes the dots to overlap more in the inset than in the main sub-plot. The univariate kernel density estimates along the x- (Median Heatwave Area (%)) and y-axis (Heatwave Length (days)) are on the top and right side of the subplots, respectively, with vertical lines in dark blue (Berkeley Earth) and light blue (ERA5) indicating the observed event. The respective region is indicated above the color bar.

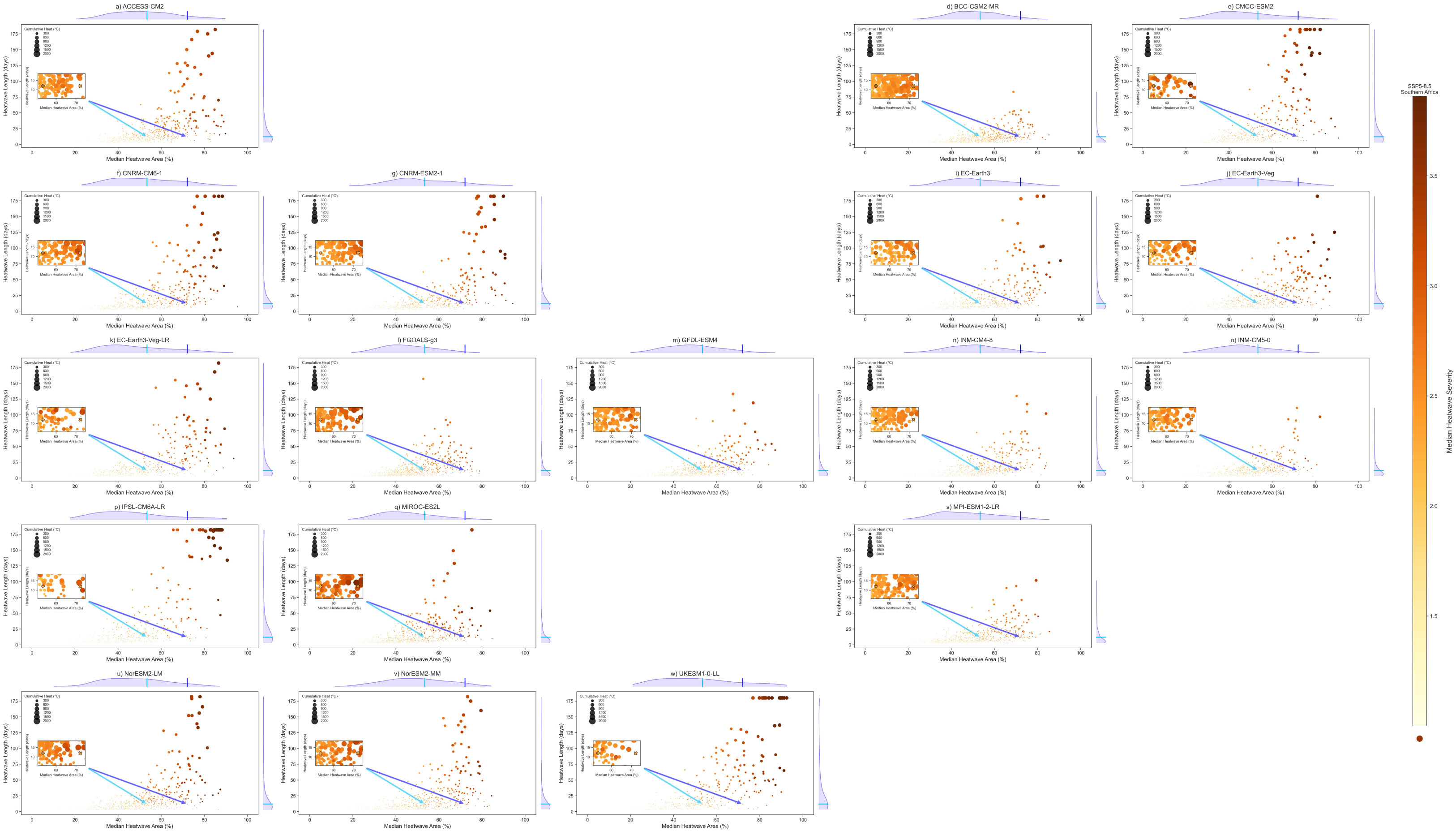
a) ACCESS-CM2
d) BCC-CSM2-MR
e) CMCC-ESM2
f) CNRM-CM6-1
g) CNRM-ESM2-1
i) EC-Earth3
j) EC-Earth3-Veg
k) EC-Earth3-Veg-LR
l) FGOALS-g3
m) GFDL-ESM4
n) INM-CM4-8
o) INM-CM5-0
p) IPSL-CM6A-LR
q) MIROC-ES2L
s) MPI-ESM1-2-LR
u) NorESM2-LM
v) NorESM2-MM
w) UKESM1-0-LL
Cumulative Heat (°C)
300
600
900
1200
1500
2000
Heatwave Length (days)
Median Heatwave Area (%)
SSP5-8.5
Southern Africa
Median Heatwave Severity
1.5
2.0
2.5
3.0
3.5

Supplementary Figure 290: Multi-HW-characteristic figure according to SSP5-8.5 CMIP6 multi-model ensemble (MME) per model for Eastern USA (Tx90p) - Each panel shows regional projections according to SSP5-8.5 model runs in the CMIP6 multi-model ensemble (MME) of four combined heatwave characteristics for each projected heatwave in the ensemble between 2020 and 2100. Each dot represents an individual heatwave in the projections, characterized by Median Heatwave Area (%) (x-axis) as latitude-weighted percentage of grid boxes affected by extreme heat, Heatwave Length (days) (y-axis) in days, Median Heatwave Severity (color), and Cumulative Heat (°C) (dot size). The characteristics of the observed event according to the Berkeley Earth observational data (x symbol) and the ERA5 reanalysis data (+ symbol), respectively, are pointed to by an arrow and a figure inset shows a zoom-in on this figure section. The figure inset shows the observed heatwave in the two reference data sets and projections in the indicated section of the respective sub-plot. It facilitates readability of color and size of the reference values and comparison to each other and the neighboring projections. Note that the zoom-in causes the dots to overlap more in the inset than in the main sub-plot. The univariate kernel density estimates along the x- (Median Heatwave Area (%)) and y-axis (Heatwave Length (days)) are on the top and right side of the subplots, respectively, with vertical lines in dark blue (Berkeley Earth) and light blue (ERA5) indicating the observed event. The respective region is indicated above the color bar.

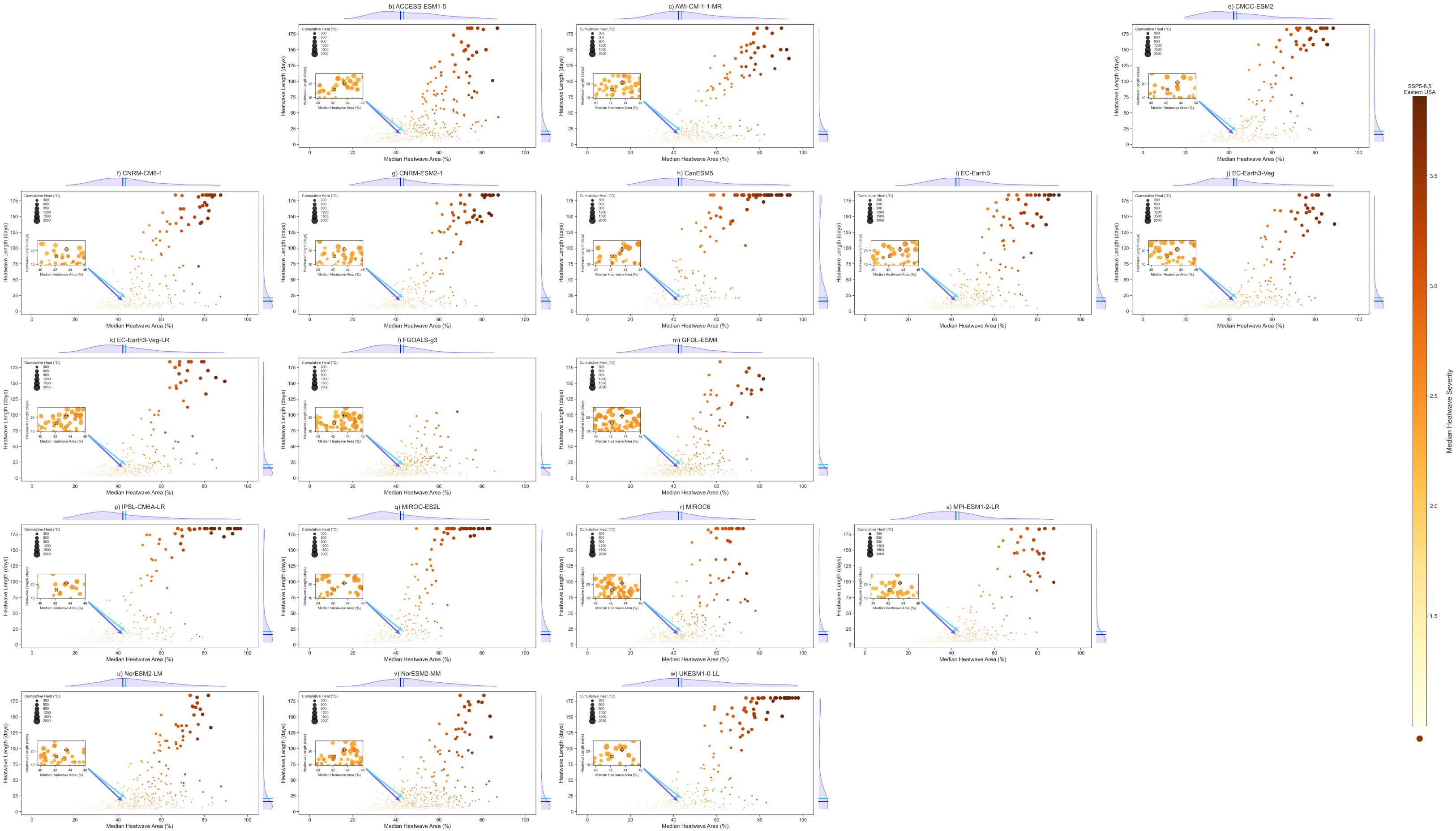

b) ACCESS-ESM1-5
c) AWI-CM-1-1-MR
e) CMCC-ESM2
f) CNRM-CM6-1
g) CNRM-ESM2-1
h) CanESM5
i) EC-Earth3
j) EC-Earth3-Veg
k) EC-Earth3-Veg-LR
l) FGOALS-g3
m) GFDL-ESM4
p) IPSL-CM6A-LR
q) MIROC-ES2L
r) MIROC6
s) MPI-ESM1-2-LR
u) NorESM2-LM
v) NorESM2-MM
w) UKESM1-0-LL
Cumulative Heat (°C)
300
600
900
1200
1500
2000
Heatwave Length (days)
Median Heatwave Area (%)
SSP5-8.5
Eastern USA
Median Heatwave Severity

Supplementary Figure 291: Kernel Density Estimates (KDE) across global warming levels (GWL)s for Maximum Heatwave Area (%) in SSP5-8.5 CMIP6 multi-model ensemble (MME) (Tx90p) - Kernel Density Estimates (KDE) of Maximum Heatwave Area (%) for different global warming levels (GWL)s in the CMIP6 multi-model ensemble (MME). Area under each curve is normalized to 1. Maximum Heatwave Area (%) of the observed event is indicated by vertical lines in dark blue (Berkeley Earth) and light blue (ERA5).

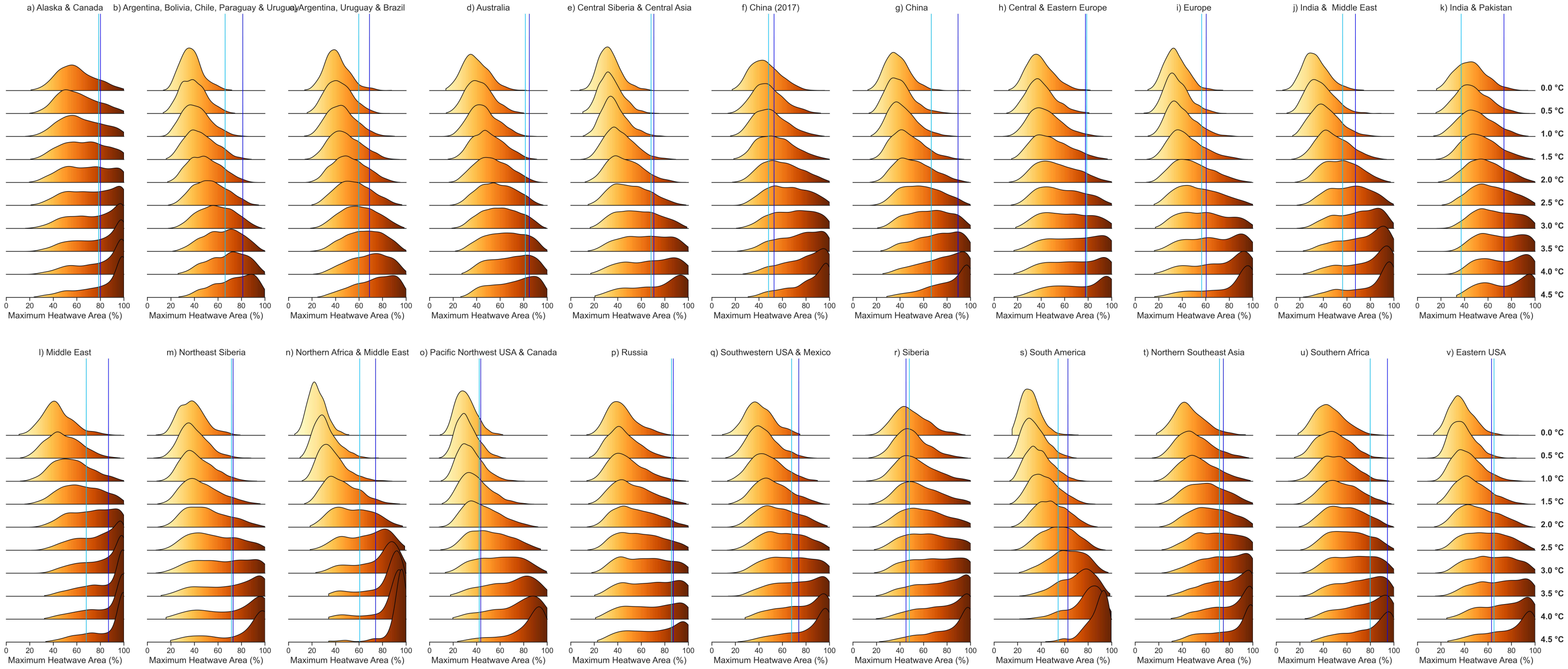
a) Alaska & Canada
b) Argentina, Bolivia, Chile, Paraguay & Uruguay
c) Argentina, Uruguay & Brazil
d) Australia
e) Central Siberia & Central Asia
f) China (2017)
g) China
h) Central & Eastern Europe
i) Europe
j) India & Middle East
k) India & Pakistan
l) Middle East
m) Northeast Siberia
n) Northern Africa & Middle East
o) Pacific Northwest USA & Canada
p) Russia
q) Southwestern USA & Mexico
r) Siberia
s) South America
t) Northern Southeast Asia
u) Southern Africa
v) Eastern USA
0.0 °C
0.5 °C
1.0 °C
1.5 °C
2.0 °C
2.5 °C
3.0 °C
3.5 °C
4.0 °C
4.5 °C
0 20 40 60 80 100
Maximum Heatwave Area (%)

Supplementary Figure 292: Kernel Density Estimates (KDE) across global warming levels (GWL)s for Maximum Heatwave Severity in SSP5-8.5 CMIP6 multi-model ensemble (MME) (Tx90p) - Kernel Density Estimates (KDE) of Maximum Heatwave Severity for different global warming levels (GWL)s in the CMIP6 multi-model ensemble (MME). Area under each curve is normalized to 1. Maximum Heatwave Severity of the observed event is indicated by vertical lines in dark blue (Berkeley Earth) and light blue (ERA5).

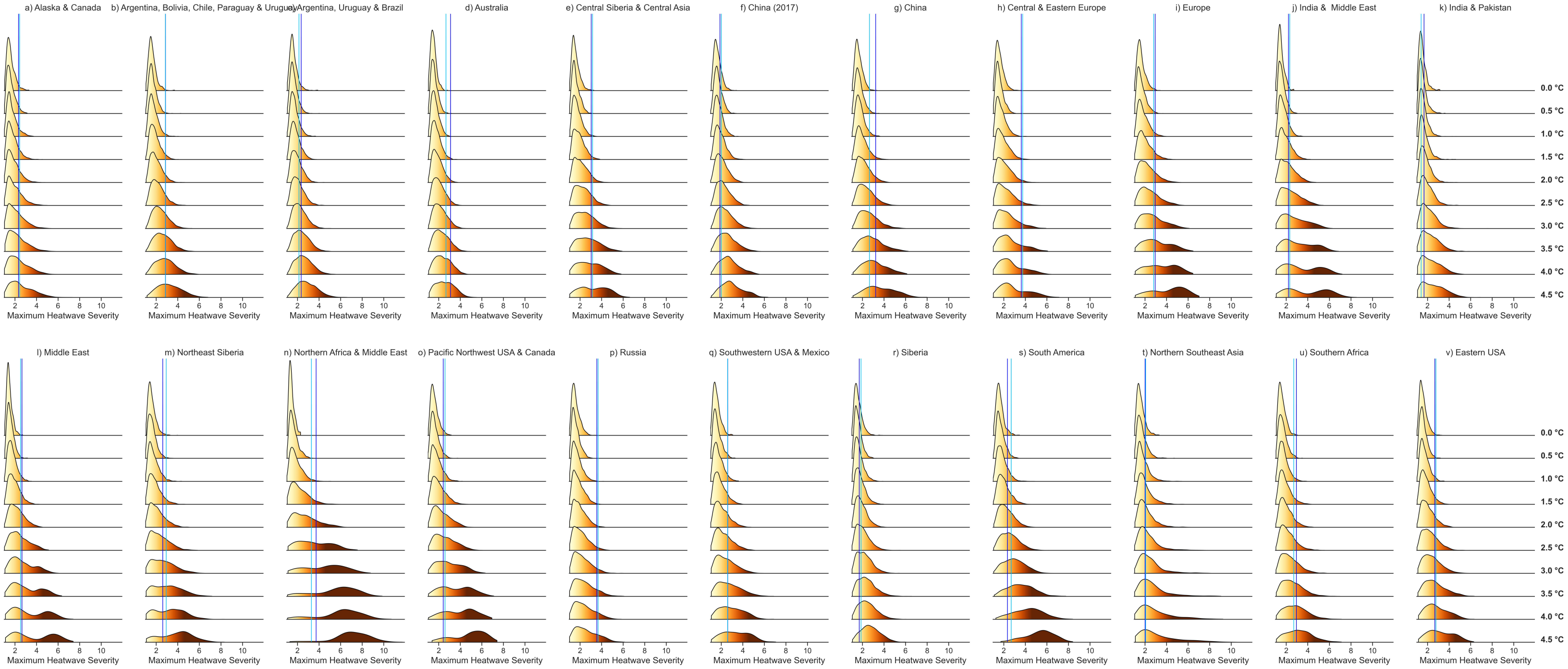

a) Alaska & Canada
b) Argentina, Bolivia, Chile, Paraguay & Uruguay
c) Argentina, Uruguay & Brazil
d) Australia
e) Central Siberia & Central Asia
f) China (2017)
g) China
h) Central & Eastern Europe
i) Europe
j) India & Middle East
k) India & Pakistan
l) Middle East
m) Northeast Siberia
n) Northern Africa & Middle East
o) Pacific Northwest USA & Canada
p) Russia
q) Southwestern USA & Mexico
r) Siberia
s) South America
t) Northern Southeast Asia
u) Southern Africa
v) Eastern USA
0.0 °C
0.5 °C
1.0 °C
1.5 °C
2.0 °C
2.5 °C
3.0 °C
3.5 °C
4.0 °C
4.5 °C
2 4 6 8 10
Maximum Heatwave Severity

Supplementary Figure 293: Kernel Density Estimates (KDE) across global warming levels (GWL)s for Median Heatwave Area (%) in SSP5-8.5 CMIP6 multi-model ensemble (MME) (Tx90p) - Kernel Density Estimates (KDE) of Median Heatwave Area (%) for different global warming levels (GWL)s in the CMIP6 multi-model ensemble (MME). Area under each curve is normalized to 1. Median Heatwave Area (%) of the observed event is indicated by vertical lines in dark blue (Berkeley Earth) and light blue (ERA5).

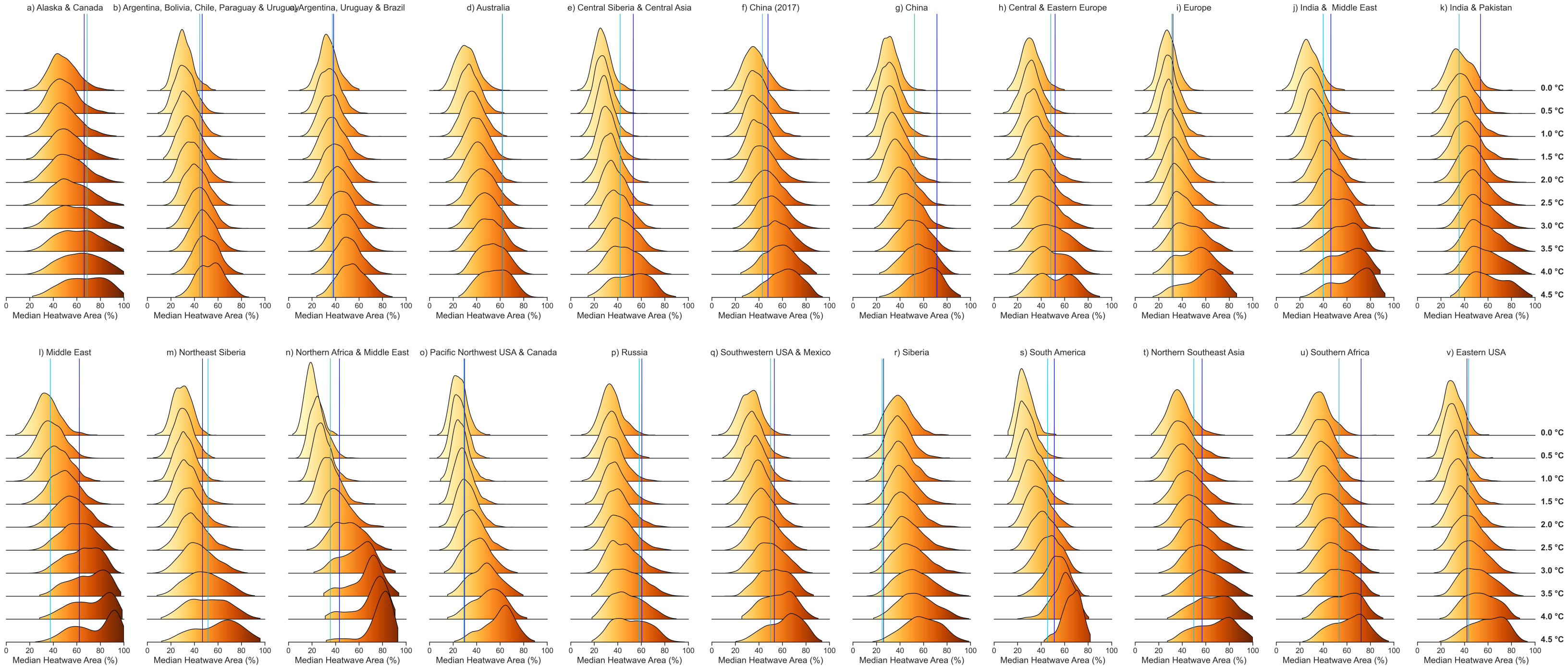
a) Alaska & Canada
b) Argentina, Bolivia, Chile, Paraguay & Uruguay
c) Argentina, Uruguay & Brazil
d) Australia
e) Central Siberia & Central Asia
f) China (2017)
g) China
h) Central & Eastern Europe
i) Europe
j) India & Middle East
k) India & Pakistan
l) Middle East
m) Northeast Siberia
n) Northern Africa & Middle East
o) Pacific Northwest USA & Canada
p) Russia
q) Southwestern USA & Mexico
r) Siberia
s) South America
t) Northern Southeast Asia
u) Southern Africa
v) Eastern USA
0.0 °C
0.5 °C
1.0 °C
1.5 °C
2.0 °C
2.5 °C
3.0 °C
3.5 °C
4.0 °C
4.5 °C
0 20 40 60 80 100
Median Heatwave Area (%)

Supplementary Figure 294: Kernel Density Estimates (KDE) across global warming levels (GWL)s for Median Heatwave Severity in SSP5-8.5 CMIP6 multi-model ensemble (MME) (Tx90p) - Kernel Density Estimates (KDE) of Median Heatwave Severity for different global warming levels (GWL)s in the CMIP6 multi-model ensemble (MME). Area under each curve is normalized to 1. Median Heatwave Severity of the observed event is indicated by vertical lines in dark blue (Berkeley Earth) and light blue (ERA5).

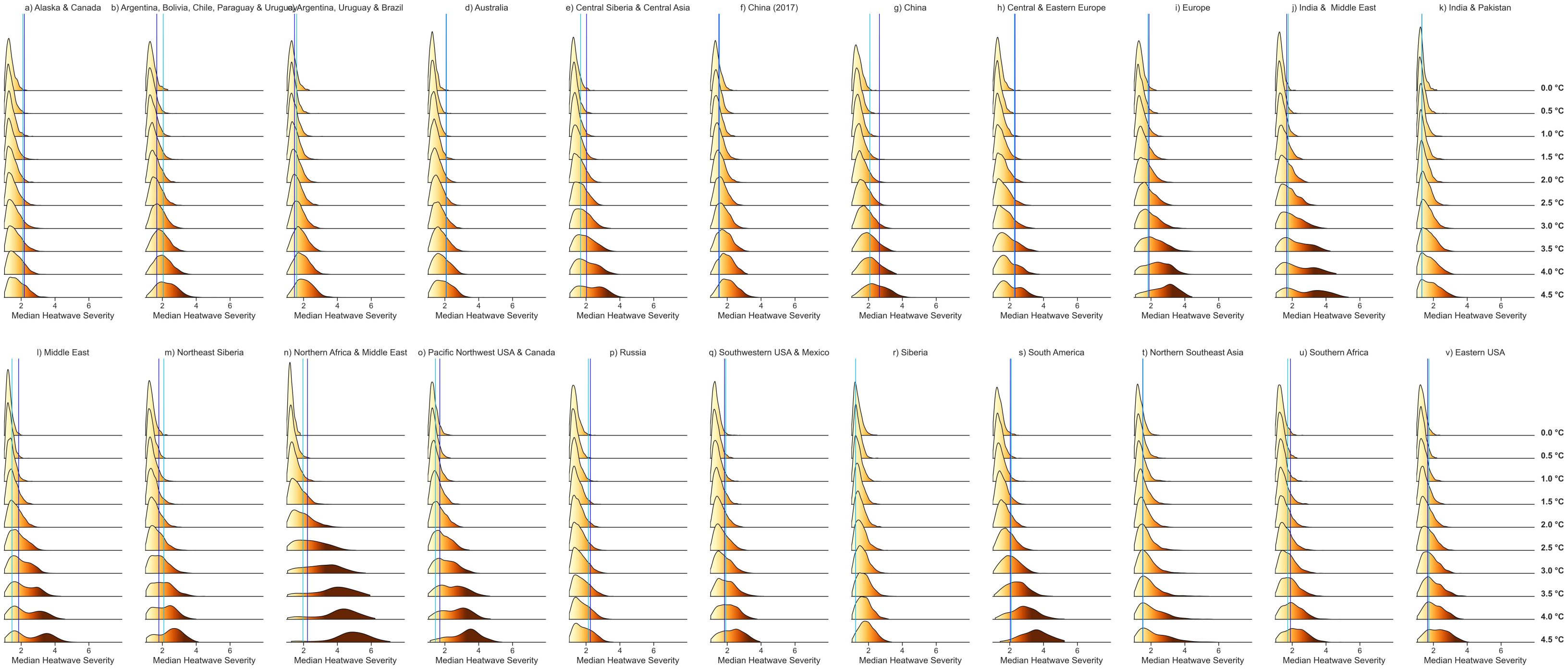
a) Alaska & Canada
b) Argentina, Bolivia, Chile, Paraguay & Uruguay
c) Argentina, Uruguay & Brazil
d) Australia
e) Central Siberia & Central Asia
f) China (2017)
g) China
h) Central & Eastern Europe
i) Europe
j) India & Middle East
k) India & Pakistan
l) Middle East
m) Northeast Siberia
n) Northern Africa & Middle East
o) Pacific Northwest USA & Canada
p) Russia
q) Southwestern USA & Mexico
r) Siberia
s) South America
t) Northern Southeast Asia
u) Southern Africa
v) Eastern USA
0.0 °C
0.5 °C
1.0 °C
1.5 °C
2.0 °C
2.5 °C
3.0 °C
3.5 °C
4.0 °C
4.5 °C
Median Heatwave Severity

Supplementary Figure 295: Kernel Density Estimates (KDE) across global warming levels (GWL)s for Heatwave Length (days) in SSP5-8.5 CMIP6 multi-model ensemble (MME) (Tx90p) - Kernel Density Estimates (KDE) of Heatwave Length (days) for different global warming levels (GWL)s in the CMIP6 multi-model ensemble (MME). Area under each curve is normalized to 1. Heatwave Length (days) of the observed event is indicated by vertical lines in dark blue (Berkeley Earth) and light blue (ERA5).

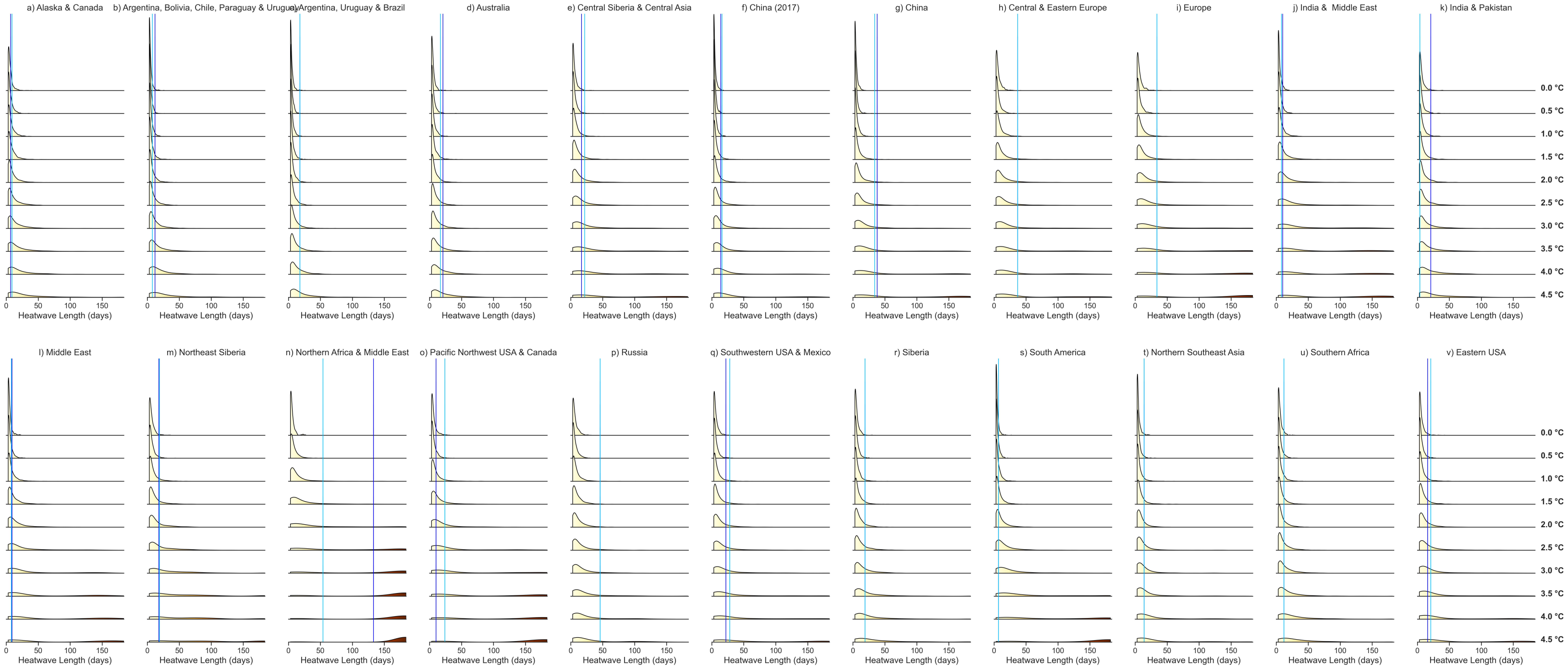
a) Alaska & Canada
b) Argentina, Bolivia, Chile, Paraguay & Uruguay
c) Argentina, Uruguay & Brazil
d) Australia
e) Central Siberia & Central Asia
f) China (2017)
g) China
h) Central & Eastern Europe
i) Europe
j) India & Middle East
k) India & Pakistan
l) Middle East
m) Northeast Siberia
n) Northern Africa & Middle East
o) Pacific Northwest USA & Canada
p) Russia
q) Southwestern USA & Mexico
r) Siberia
s) South America
t) Northern Southeast Asia
u) Southern Africa
v) Eastern USA
0.0 °C
0.5 °C
1.0 °C
1.5 °C
2.0 °C
2.5 °C
3.0 °C
3.5 °C
4.0 °C
4.5 °C
0
50
100
150
Heatwave Length (days)

Supplementary Figure 296: Kernel Density Estimates (KDE) across global warming levels (GWL)s for Cumulative Heat (°C) in SSP5-8.5 CMIP6 multi-model ensemble (MME) (Tx90p) - Kernel Density Estimates (KDE) of Cumulative Heat (°C) for different global warming levels (GWL)s in the CMIP6 multi-model ensemble (MME). Area under each curve is normalized to 1. Cumulative Heat (°C) of the observed event is indicated by vertical lines in dark blue (Berkeley Earth) and light blue (ERA5).

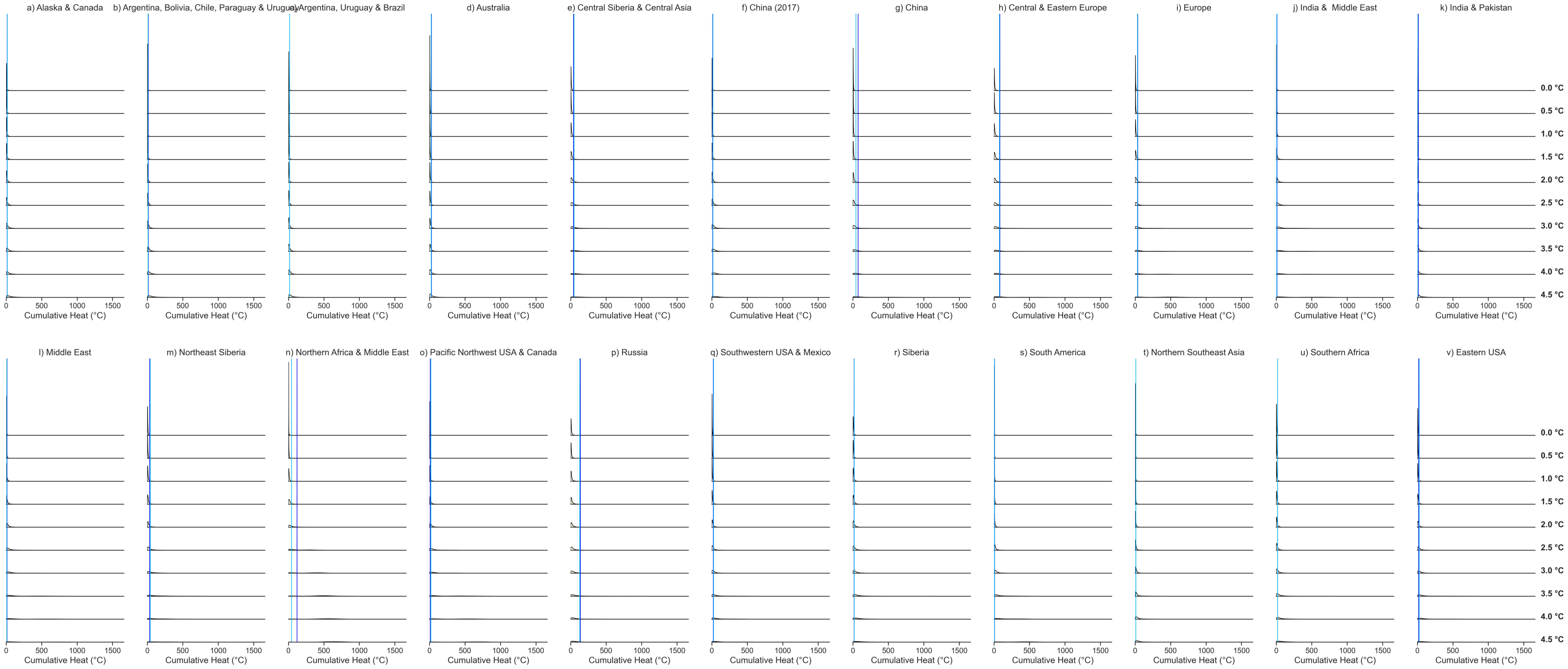

a) Alaska & Canada
b) Argentina, Bolivia, Chile, Paraguay & Uruguay
c) Argentina, Uruguay & Brazil
d) Australia
e) Central Siberia & Central Asia
f) China (2017)
g) China
h) Central & Eastern Europe
i) Europe
j) India & Middle East
k) India & Pakistan
l) Middle East
m) Northeast Siberia
n) Northern Africa & Middle East
o) Pacific Northwest USA & Canada
p) Russia
q) Southwestern USA & Mexico
r) Siberia
s) South America
t) Northern Southeast Asia
u) Southern Africa
v) Eastern USA
0.0 °C
0.5 °C
1.0 °C
1.5 °C
2.0 °C
2.5 °C
3.0 °C
3.5 °C
4.0 °C
4.5 °C
0
500
1000
1500
Cumulative Heat (°C)

Supplementary Figure 297: Kernel Density Estimates (KDE) across global warming levels (GWL)s for Maximum Heatwave Area (%) in RCP 8.5 CMIP5 multi-model ensemble (MME) (Tx90p) - Kernel Density Estimates (KDE) of Maximum Heatwave Area (%) for different global warming levels (GWL)s in the CMIP5 multi-model ensemble (MME). Area under each curve is normalized to 1. Maximum Heatwave Area (%) of the observed event is indicated by vertical lines in dark blue (Berkeley Earth) and light blue (ERA5).

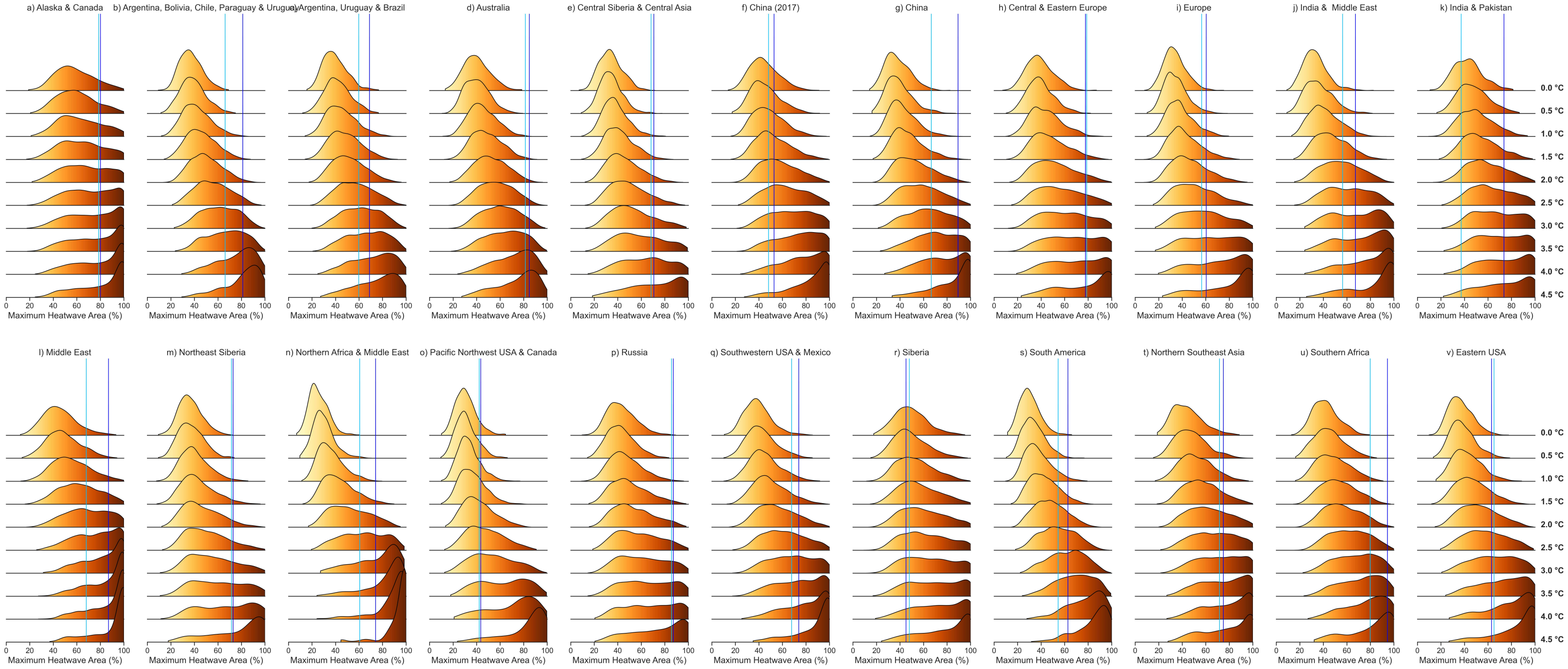

a) Alaska & Canada
b) Argentina, Bolivia, Chile, Paraguay & Uruguay
c) Argentina, Uruguay & Brazil
d) Australia
e) Central Siberia & Central Asia
f) China (2017)
g) China
h) Central & Eastern Europe
i) Europe
j) India & Middle East
k) India & Pakistan
l) Middle East
m) Northeast Siberia
n) Northern Africa & Middle East
o) Pacific Northwest USA & Canada
p) Russia
q) Southwestern USA & Mexico
r) Siberia
s) South America
t) Northern Southeast Asia
u) Southern Africa
v) Eastern USA
0.0 °C
0.5 °C
1.0 °C
1.5 °C
2.0 °C
2.5 °C
3.0 °C
3.5 °C
4.0 °C
4.5 °C
Maximum Heatwave Area (%)
0
20
40
60
80
100

Supplementary Figure 298: Kernel Density Estimates (KDE) across global warming levels (GWL)s for Maximum Heatwave Severity in RCP 8.5 CMIP5 multi-model ensemble (MME) (Tx90p) - Kernel Density Estimates (KDE) of Maximum Heatwave Severity for different global warming levels (GWL)s in the CMIP5 multi-model ensemble (MME). Area under each curve is normalized to 1. Maximum Heatwave Severity of the observed event is indicated by vertical lines in dark blue (Berkeley Earth) and light blue (ERA5).

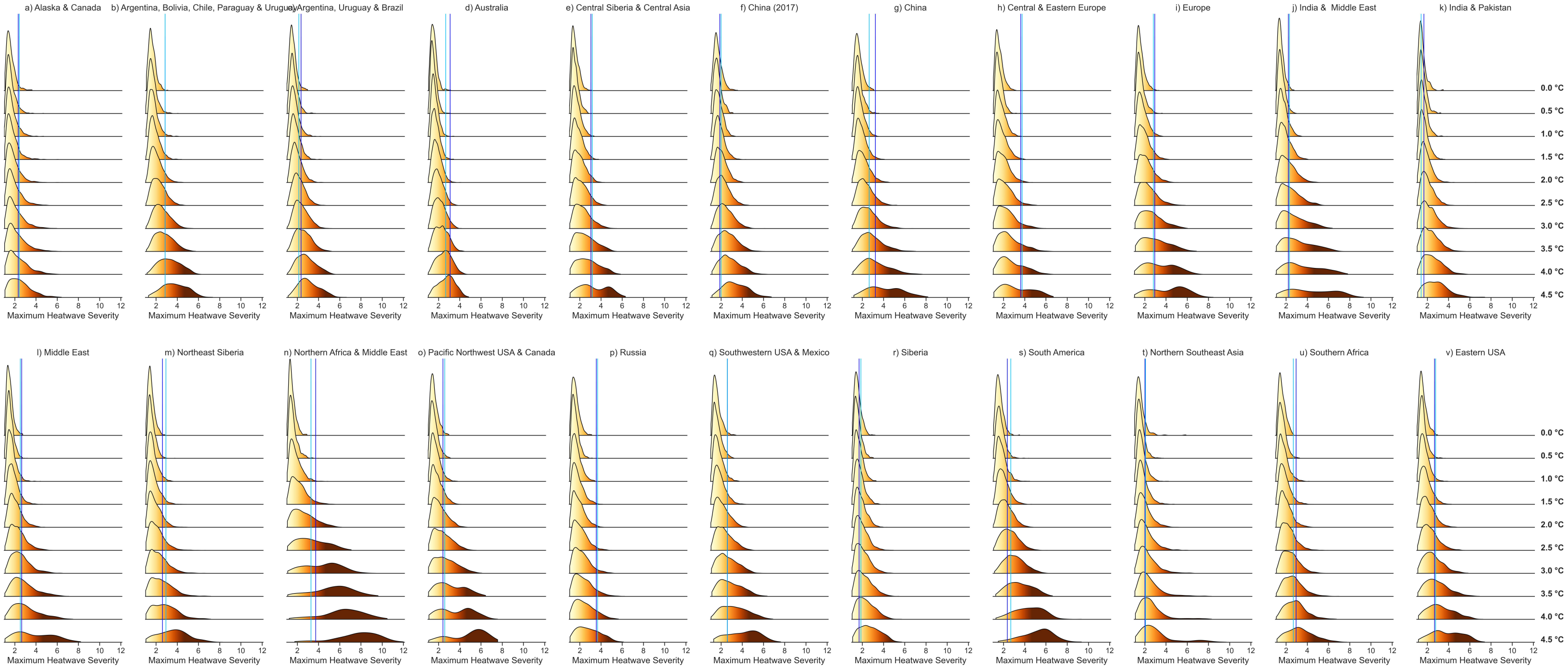

a) Alaska & Canada
b) Argentina, Bolivia, Chile, Paraguay & Uruguay
c) Argentina, Uruguay & Brazil
d) Australia
e) Central Siberia & Central Asia
f) China (2017)
g) China
h) Central & Eastern Europe
i) Europe
j) India & Middle East
k) India & Pakistan
l) Middle East
m) Northeast Siberia
n) Northern Africa & Middle East
o) Pacific Northwest USA & Canada
p) Russia
q) Southwestern USA & Mexico
r) Siberia
s) South America
t) Northern Southeast Asia
u) Southern Africa
v) Eastern USA
0.0 °C
0.5 °C
1.0 °C
1.5 °C
2.0 °C
2.5 °C
3.0 °C
3.5 °C
4.0 °C
4.5 °C
Maximum Heatwave Severity
2 4 6 8 10 12

Supplementary Figure 299: Kernel Density Estimates (KDE) across global warming levels (GWL)s for Median Heatwave Area (%) in RCP 8.5 CMIP5 multi-model ensemble (MME) (Tx90p) - Kernel Density Estimates (KDE) of Median Heatwave Area (%) for different global warming levels (GWL)s in the CMIP5 multi-model ensemble (MME). Area under each curve is normalized to 1. Median Heatwave Area (%) of the observed event is indicated by vertical lines in dark blue (Berkeley Earth) and light blue (ERA5).

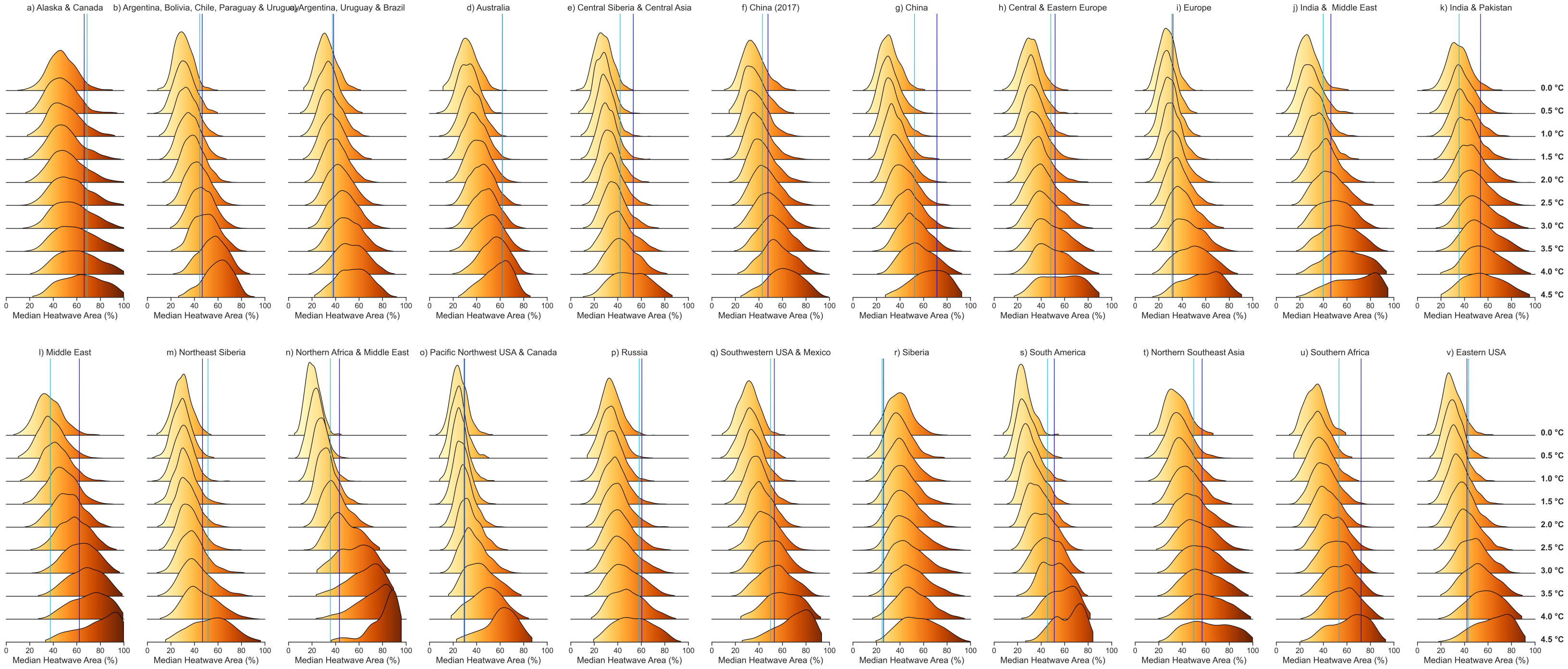
a) Alaska & Canada
b) Argentina, Bolivia, Chile, Paraguay & Uruguay
c) Argentina, Uruguay & Brazil
d) Australia
e) Central Siberia & Central Asia
f) China (2017)
g) China
h) Central & Eastern Europe
i) Europe
j) India & Middle East
k) India & Pakistan
l) Middle East
m) Northeast Siberia
n) Northern Africa & Middle East
o) Pacific Northwest USA & Canada
p) Russia
q) Southwestern USA & Mexico
r) Siberia
s) South America
t) Northern Southeast Asia
u) Southern Africa
v) Eastern USA
0.0 °C
0.5 °C
1.0 °C
1.5 °C
2.0 °C
2.5 °C
3.0 °C
3.5 °C
4.0 °C
4.5 °C
0 20 40 60 80 100
Median Heatwave Area (%)

Supplementary Figure 300: Kernel Density Estimates (KDE) across global warming levels (GWL)s for Median Heatwave Severity in RCP 8.5 CMIP5 multi-model ensemble (MME) (Tx90p) - Kernel Density Estimates (KDE) of Median Heatwave Severity for different global warming levels (GWL)s in the CMIP5 multi-model ensemble (MME). Area under each curve is normalized to 1. Median Heatwave Severity of the observed event is indicated by vertical lines in dark blue (Berkeley Earth) and light blue (ERA5).

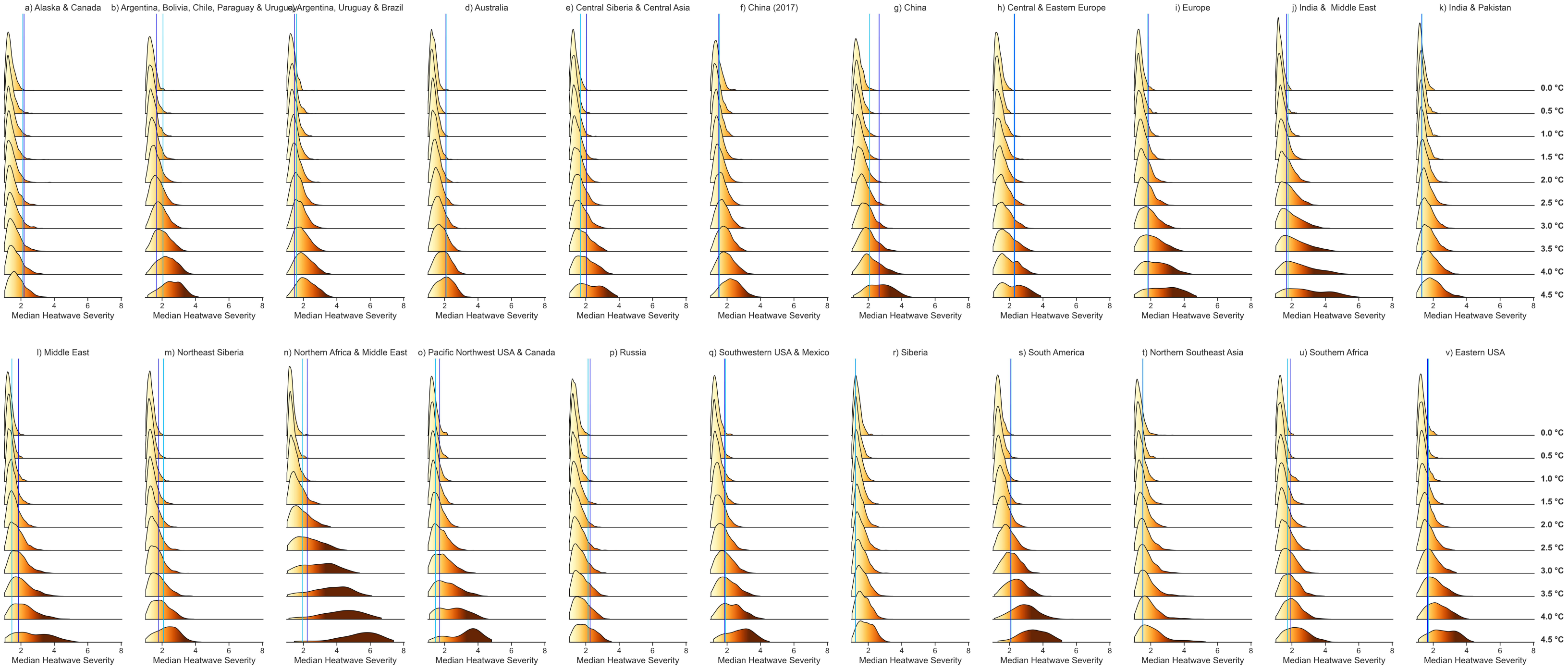

a) Alaska & Canada
b) Argentina, Bolivia, Chile, Paraguay & Uruguay
c) Argentina, Uruguay & Brazil
d) Australia
e) Central Siberia & Central Asia
f) China (2017)
g) China
h) Central & Eastern Europe
i) Europe
j) India & Middle East
k) India & Pakistan
l) Middle East
m) Northeast Siberia
n) Northern Africa & Middle East
o) Pacific Northwest USA & Canada
p) Russia
q) Southwestern USA & Mexico
r) Siberia
s) South America
t) Northern Southeast Asia
u) Southern Africa
v) Eastern USA
Median Heatwave Severity
0.0 °C
0.5 °C
1.0 °C
1.5 °C
2.0 °C
2.5 °C
3.0 °C
3.5 °C
4.0 °C
4.5 °C

Supplementary Figure 301: Kernel Density Estimates (KDE) across global warming levels (GWL)s for Heatwave Length (days) in RCP 8.5 CMIP5 multi-model ensemble (MME) (Tx90p) - Kernel Density Estimates (KDE) of Heatwave Length (days) for different global warming levels (GWL)s in the CMIP5 multi-model ensemble (MME). Area under each curve is normalized to 1. Heatwave Length (days) of the observed event is indicated by vertical lines in dark blue (Berkeley Earth) and light blue (ERA5).

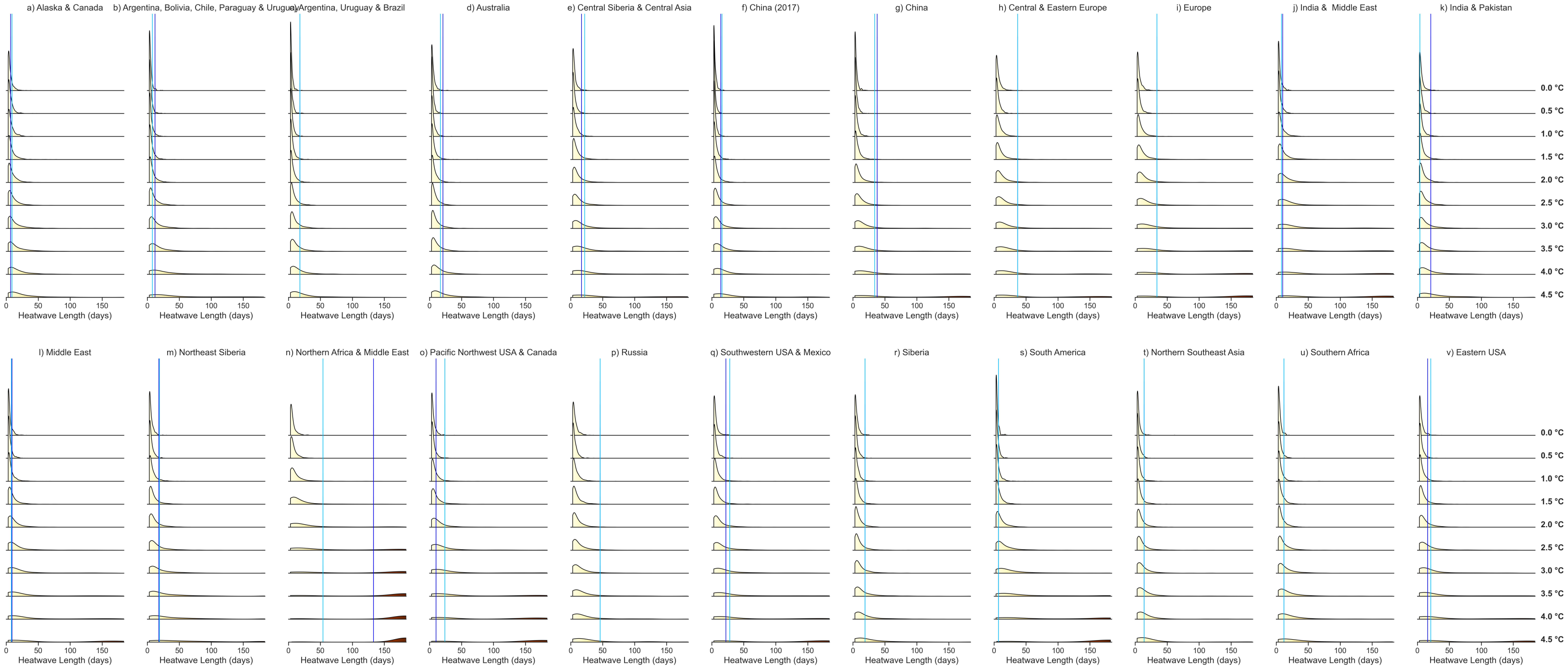

a) Alaska & Canada
b) Argentina, Bolivia, Chile, Paraguay & Uruguay
c) Argentina, Uruguay & Brazil
d) Australia
e) Central Siberia & Central Asia
f) China (2017)
g) China
h) Central & Eastern Europe
i) Europe
j) India & Middle East
k) India & Pakistan
l) Middle East
m) Northeast Siberia
n) Northern Africa & Middle East
o) Pacific Northwest USA & Canada
p) Russia
q) Southwestern USA & Mexico
r) Siberia
s) South America
t) Northern Southeast Asia
u) Southern Africa
v) Eastern USA
0.0 °C
0.5 °C
1.0 °C
1.5 °C
2.0 °C
2.5 °C
3.0 °C
3.5 °C
4.0 °C
4.5 °C
0
50
100
150
Heatwave Length (days)

Supplementary Figure 302: Kernel Density Estimates (KDE) across global warming levels (GWL)s for Cumulative Heat (°C) in RCP 8.5 CMIP5 multi-model ensemble (MME) (Tx90p) - Kernel Density Estimates (KDE) of Cumulative Heat (°C) for different global warming levels (GWL)s in the CMIP5 multi-model ensemble (MME). Area under each curve is normalized to 1. Cumulative Heat (°C) of the observed event is indicated by vertical lines in dark blue (Berkeley Earth) and light blue (ERA5).

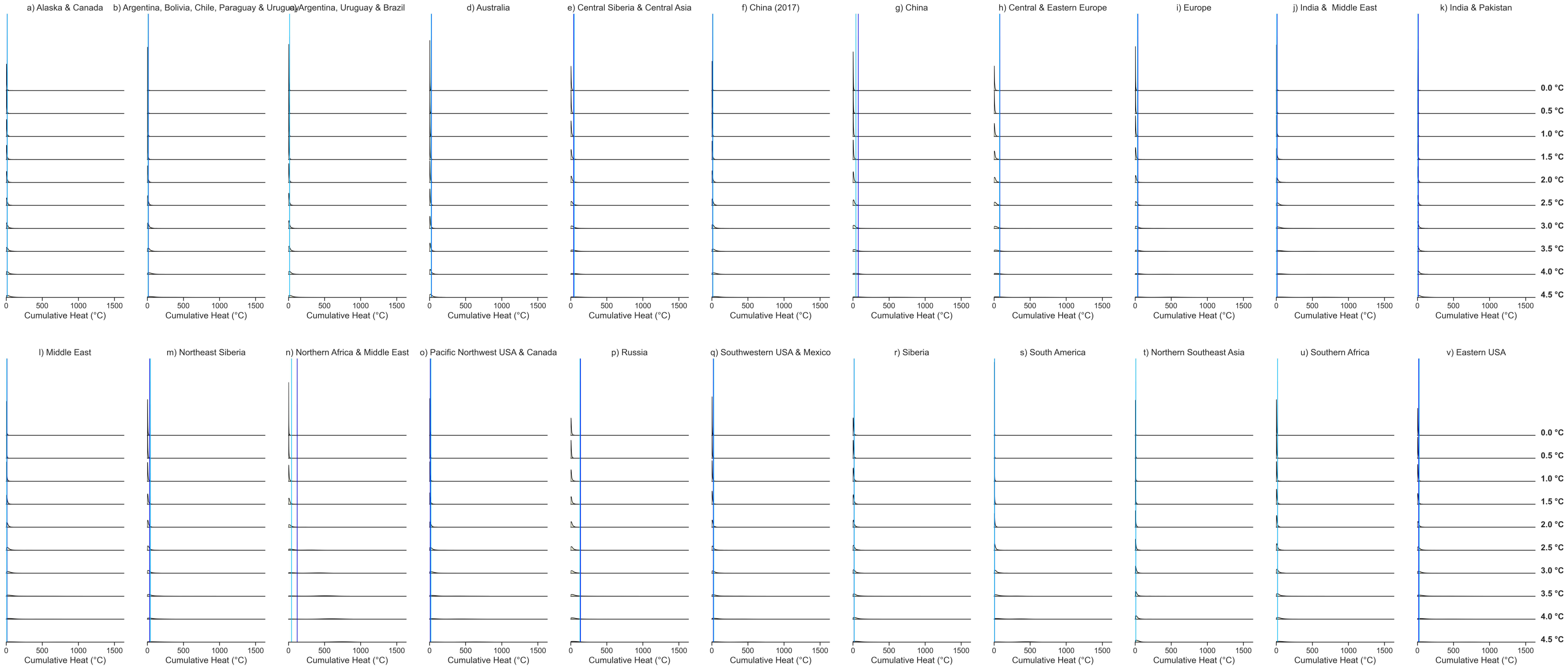

a) Alaska & Canada
b) Argentina, Bolivia, Chile, Paraguay & Uruguay
c) Argentina, Uruguay & Brazil
d) Australia
e) Central Siberia & Central Asia
f) China (2017)
g) China
h) Central & Eastern Europe
i) Europe
j) India & Middle East
k) India & Pakistan
l) Middle East
m) Northeast Siberia
n) Northern Africa & Middle East
o) Pacific Northwest USA & Canada
p) Russia
q) Southwestern USA & Mexico
r) Siberia
s) South America
t) Northern Southeast Asia
u) Southern Africa
v) Eastern USA
0.0 °C
0.5 °C
1.0 °C
1.5 °C
2.0 °C
2.5 °C
3.0 °C
3.5 °C
4.0 °C
4.5 °C
0
500
1000
1500
Cumulative Heat (°C)

Supplementary Figure 303: Reference Event Mapping for Pacific Northwest USA & Canada based on Berkeley Earth - Spatio-temporal evolution of local heat-affected grid boxes during reference heatwave in Pacific Northwest USA & Canada based on data in Berkeley Earth. Colors and color-bar indicate the local severity values for each day of the event. The respective date of the heatwave day is indicated above each sub-figure and x- and y-axis show the longitude and latitude coordinates.

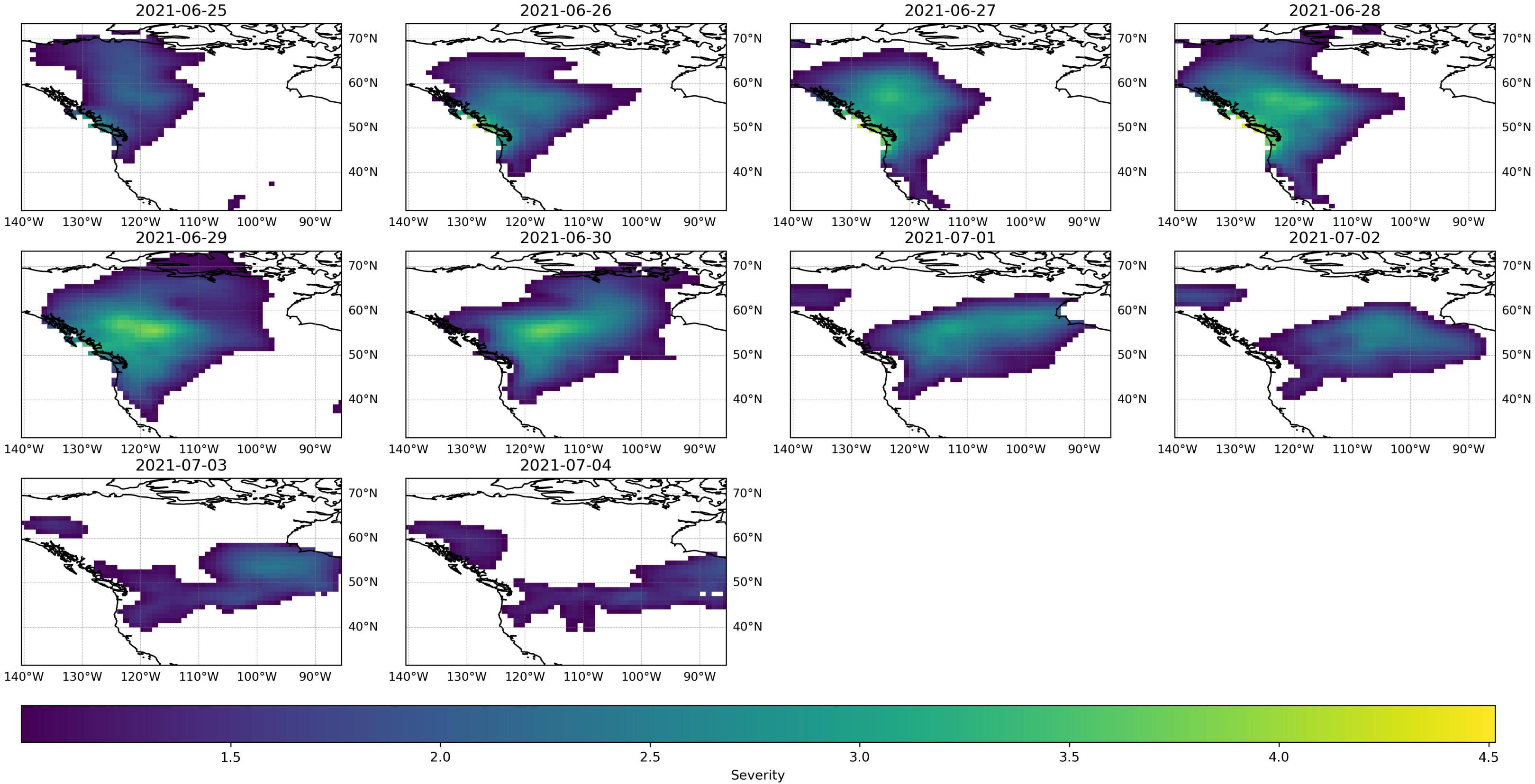
2021-06-25
2021-06-26
2021-06-27
2021-06-28
2021-06-29
2021-06-30
2021-07-01
2021-07-02
2021-07-03
2021-07-04
70°N
60°N
50°N
40°N
140°W
130°W
120°W
110°W
100°W
90°W
1.5
2.0
2.5
3.0
3.5
4.0
4.5
Severity

Supplementary Figure 304: Reference Event Mapping for Pacific Northwest USA & Canada based on ERA5 - Spatio-temporal evolution of local heat-affected grid boxes during reference heatwave in Pacific Northwest USA & Canada based on data in ERA5. Colors and color-bar indicate the local severity values for each day of the event. The respective date of the heatwave day is indicated above each sub-figure and x- and y-axis show the longitude and latitude coordinates.

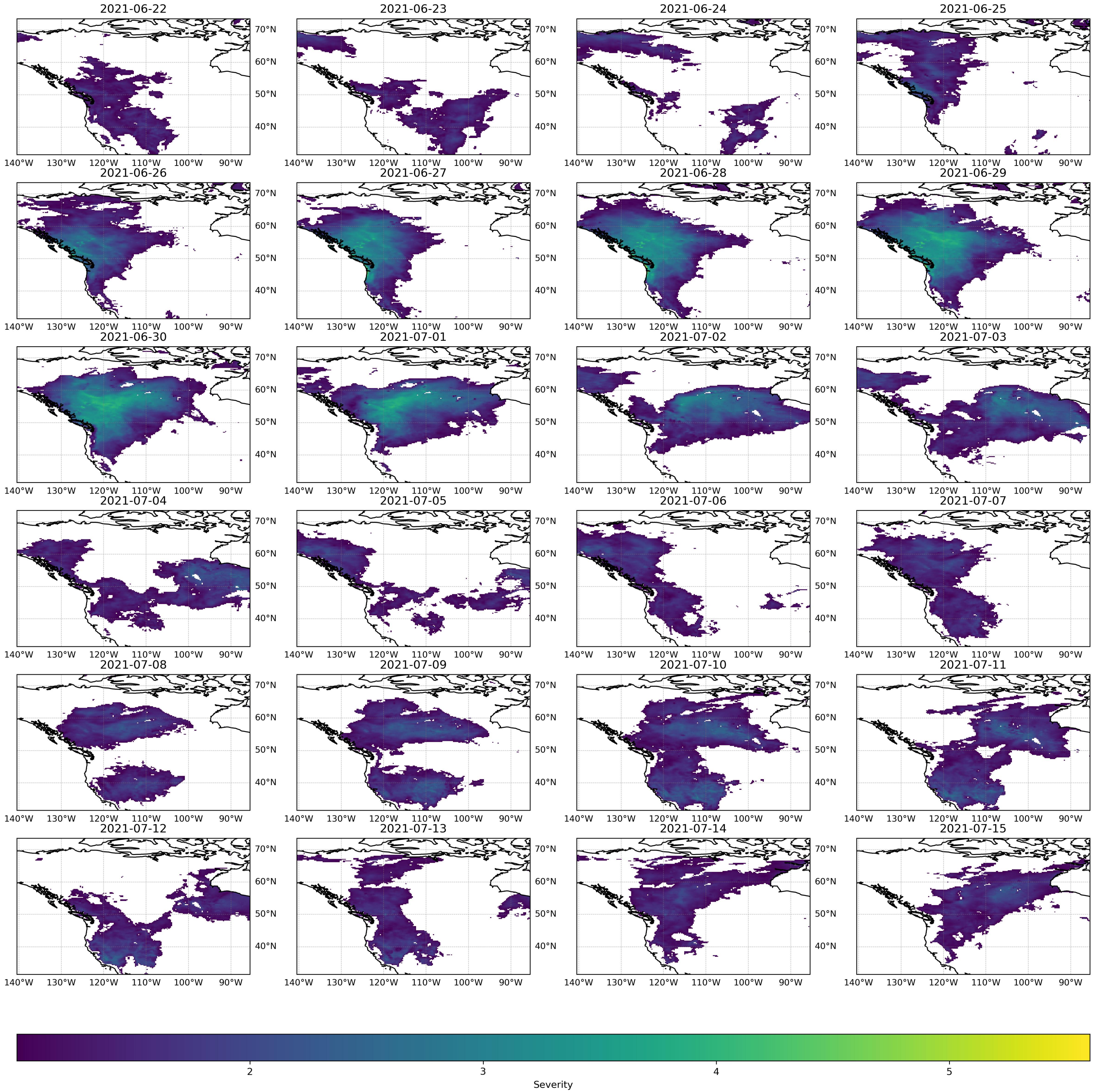

2021-06-22
2021-06-23
2021-06-24
2021-06-25
2021-06-26
2021-06-27
2021-06-28
2021-06-29
2021-06-30
2021-07-01
2021-07-02
2021-07-03
2021-07-04
2021-07-05
2021-07-06
2021-07-07
2021-07-08
2021-07-09
2021-07-10
2021-07-11
2021-07-12
2021-07-13
2021-07-14
2021-07-15
70°N
60°N
50°N
40°N
140°W
130°W
120°W
110°W
100°W
90°W
2
3
4
5
Severity

Supplementary Figure 305: Return times (RT) during historical (1951-2005) based on Berkeley Earth (Tx90p) - Return times (RT) (in years) during the historical period between 1951 and 2005 for Cumulative Heat (°C) of the respective observed event. For each region, values are shown for the different scenarios (increasing in emissions from left to right for the CMIP5 (green), CMIP6 (red), CanESM5 (dark blue), and ACCESS-ESM1-5 (light blue) ensembles. The error bars indicate the 5th and 95th percentile of the respective sample. Where the respective ensemble contains models not able to simulate the magnitude of the respective observed event's Cumulative Heat (°C), the proportion of models that are able to simulate an event with a value of Cumulative Heat (°C) at least as high as the observed event is indicated by the percentage values above the individual bars. The observed reference values are calculated using data from Berkeley Earth.

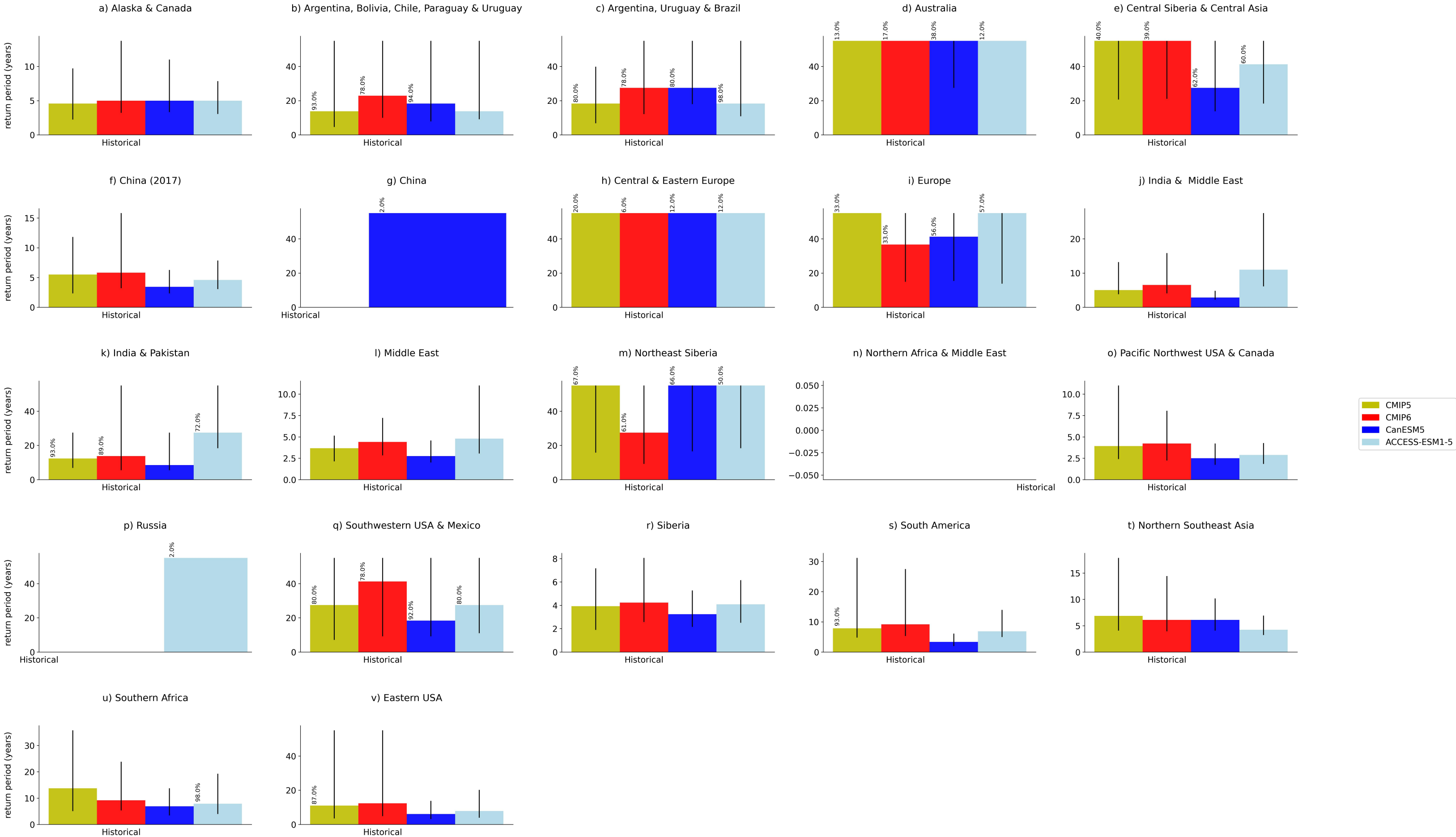
a) Alaska & Canada
b) Argentina, Bolivia, Chile, Paraguay & Uruguay
93.0% 78.0% 94.0%
c) Argentina, Uruguay & Brazil
80.0% 78.0% 80.0% 98.0%
d) Australia
13.0% 17.0% 38.0% 12.0%
e) Central Siberia & Central Asia
40.0% 39.0% 62.0% 60.0%
f) China (2017)
g) China
2.0%
h) Central & Eastern Europe
20.0% 6.0% 12.0% 12.0%
i) Europe
33.0% 33.0% 56.0% 57.0%
j) India & Middle East
k) India & Pakistan
93.0% 89.0% 72.0%
l) Middle East
m) Northeast Siberia
67.0% 61.0% 66.0% 50.0%
n) Northern Africa & Middle East
o) Pacific Northwest USA & Canada
p) Russia
2.0%
q) Southwestern USA & Mexico
80.0% 78.0% 92.0% 80.0%
r) Siberia
s) South America
93.0%
t) Northern Southeast Asia
u) Southern Africa
98.0%
v) Eastern USA
87.0%
return period (years)
Historical
CMIP5
CMIP6
CanESM5
ACCESS-ESM1-5

Supplementary Figure 306: Return times (RT) during historical (1951-2005) based on ERA5 (Tx90p) - Return times (RT) (in years) during the historical period between 1951 and 2005 for Cumulative Heat (°C) of the respective observed event. For each region, values are shown for the different scenarios (increasing in emissions from left to right for the CMIP5 (green), CMIP6 (red), CanESM5 (dark blue), and ACCESS-ESM1-5 (light blue) ensembles. The error bars indicate the 5th and 95th percentile of the respective sample. Where the respective ensemble contains models not able to simulate the magnitude of the respective observed event's Cumulative Heat (°C), the proportion of models that are able to simulate an event with a value of Cumulative Heat (°C) at least as high as the observed event is indicated by the percentage values above the individual bars. The observed reference values are calculated using data from ERA5.

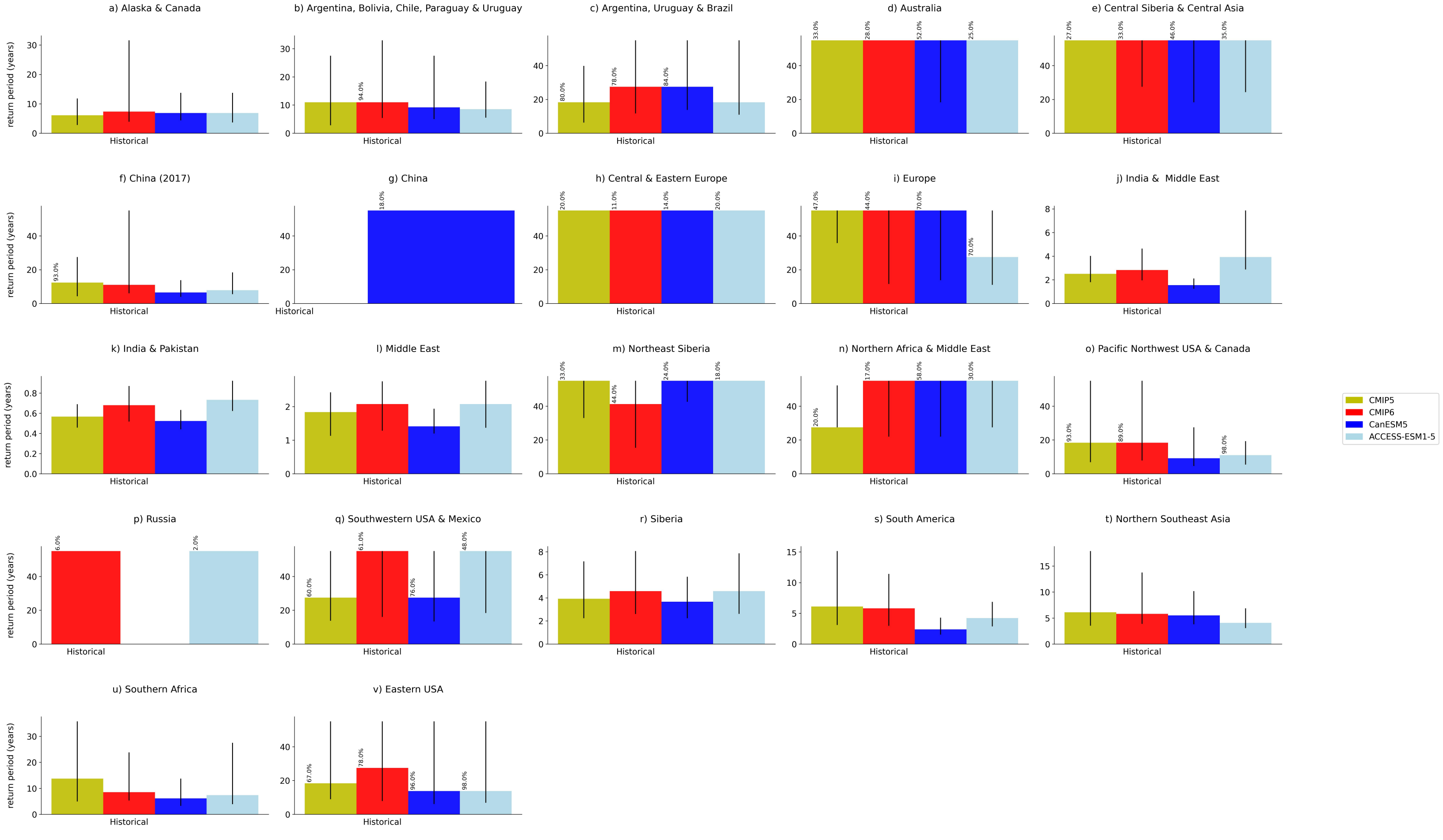

a) Alaska & Canada
b) Argentina, Bolivia, Chile, Paraguay & Uruguay
c) Argentina, Uruguay & Brazil
d) Australia
e) Central Siberia & Central Asia
f) China (2017)
g) China
h) Central & Eastern Europe
i) Europe
j) India & Middle East
k) India & Pakistan
l) Middle East
m) Northeast Siberia
n) Northern Africa & Middle East
o) Pacific Northwest USA & Canada
p) Russia
q) Southwestern USA & Mexico
r) Siberia
s) South America
t) Northern Southeast Asia
u) Southern Africa
v) Eastern USA
return period (years)
Historical
CMIP5
CMIP6
CanESM5
ACCESS-ESM1-5

Supplementary Figure 307: Return times (RT) during historical (1951-2005) based on Berkeley Earth (Tx90p) - Return times (RT) (in years) during the historical period between 1951 and 2005 for Heatwave Length (days) of the respective observed event. For each region, values are shown for the different scenarios (increasing in emissions from left to right for the CMIP5 (green), CMIP6 (red), CanESM5 (dark blue), and ACCESS-ESM1-5 (light blue) ensembles. The error bars indicate the 5th and 95th percentile of the respective sample. Where the respective ensemble contains models not able to simulate the magnitude of the respective observed event's Heatwave Length (days), the proportion of models that are able to simulate an event with a value of Heatwave Length (days) at least as high as the observed event is indicated by the percentage values above the individual bars. The observed reference values are calculated using data from Berkeley Earth.

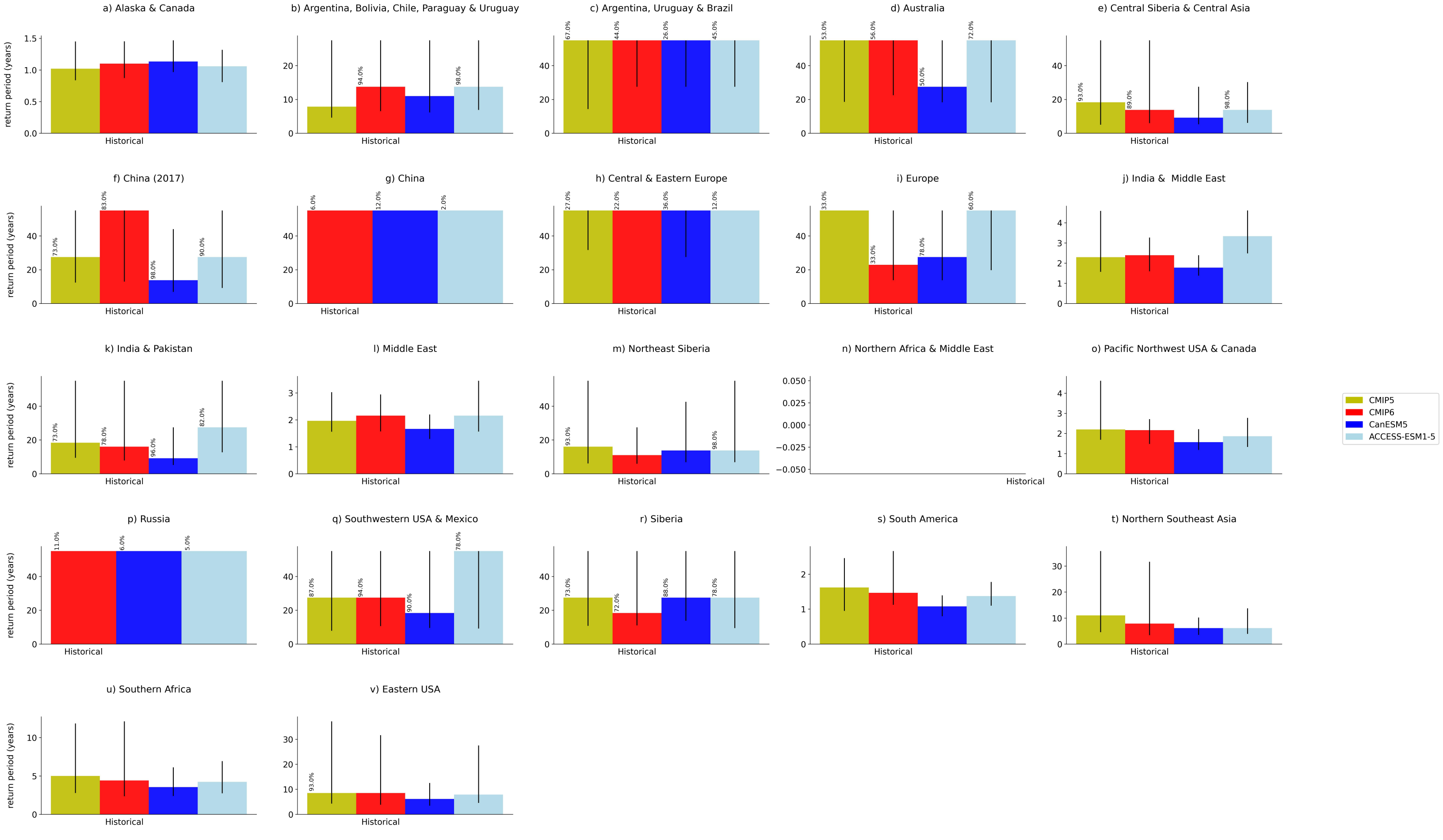

a) Alaska & Canada
b) Argentina, Bolivia, Chile, Paraguay & Uruguay
c) Argentina, Uruguay & Brazil
d) Australia
e) Central Siberia & Central Asia
f) China (2017)
g) China
h) Central & Eastern Europe
i) Europe
j) India & Middle East
k) India & Pakistan
l) Middle East
m) Northeast Siberia
n) Northern Africa & Middle East
o) Pacific Northwest USA & Canada
p) Russia
q) Southwestern USA & Mexico
r) Siberia
s) South America
t) Northern Southeast Asia
u) Southern Africa
v) Eastern USA
return period (years)
Historical
CMIP5
CMIP6
CanESM5
ACCESS-ESM1-5

Supplementary Figure 308: Return times (RT) during historical (1951-2005) based on ERA5 (Tx90p) - Return times (RT) (in years) during the historical period between 1951 and 2005 for Heatwave Length (days) of the respective observed event. For each region, values are shown for the different scenarios (increasing in emissions from left to right for the CMIP5 (green), CMIP6 (red), CanESM5 (dark blue), and ACCESS-ESM1-5 (light blue) ensembles. The error bars indicate the 5th and 95th percentile of the respective sample. Where the respective ensemble contains models not able to simulate the magnitude of the respective observed event's Heatwave Length (days), the proportion of models that are able to simulate an event with a value of Heatwave Length (days) at least as high as the observed event is indicated by the percentage values above the individual bars. The observed reference values are calculated using data from ERA5.

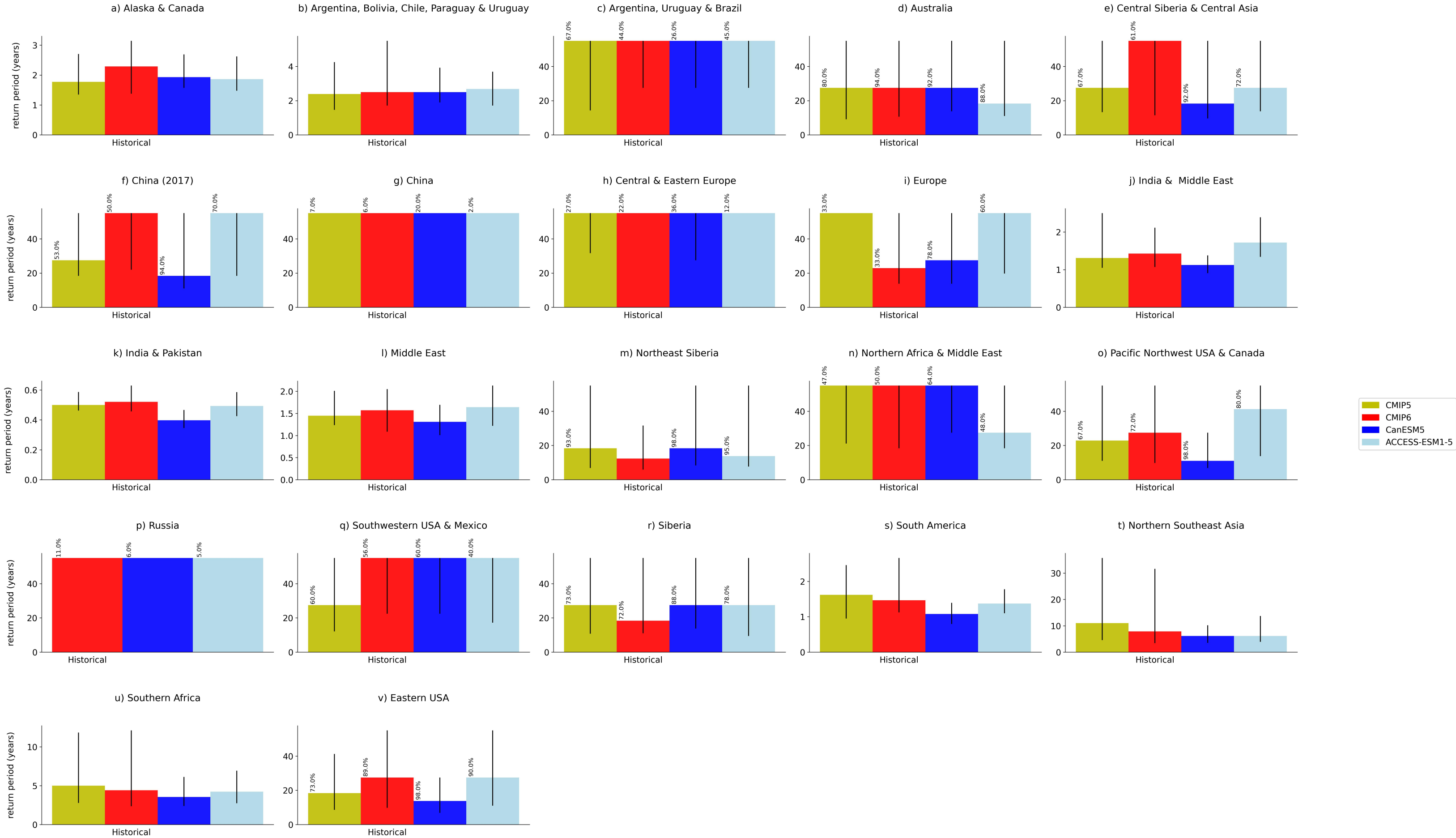
a) Alaska & Canada
b) Argentina, Bolivia, Chile, Paraguay & Uruguay
c) Argentina, Uruguay & Brazil
d) Australia
e) Central Siberia & Central Asia
f) China (2017)
g) China
h) Central & Eastern Europe
i) Europe
j) India & Middle East
k) India & Pakistan
l) Middle East
m) Northeast Siberia
n) Northern Africa & Middle East
o) Pacific Northwest USA & Canada
p) Russia
q) Southwestern USA & Mexico
r) Siberia
s) South America
t) Northern Southeast Asia
u) Southern Africa
v) Eastern USA
return period (years)
Historical
CMIP5
CMIP6
CanESM5
ACCESS-ESM1-5

Supplementary Figure 309: Return times (RT) during historical (1951-2005) based on Berkeley Earth (Tx90p) - Return times (RT) (in years) during the historical period between 1951 and 2005 for Maximum Heatwave Area (%) of the respective observed event. For each region, values are shown for the different scenarios (increasing in emissions from left to right for the CMIP5 (green), CMIP6 (red), CanESM5 (dark blue), and ACCESS-ESM1-5 (light blue) ensembles. The error bars indicate the 5th and 95th percentile of the respective sample. Where the respective ensemble contains models not able to simulate the magnitude of the respective observed event's Maximum Heatwave Area (%), the proportion of models that are able to simulate an event with a value of Maximum Heatwave Area (%) at least as high as the observed event is indicated by the percentage values above the individual bars. The observed reference values are calculated using data from Berkeley Earth.

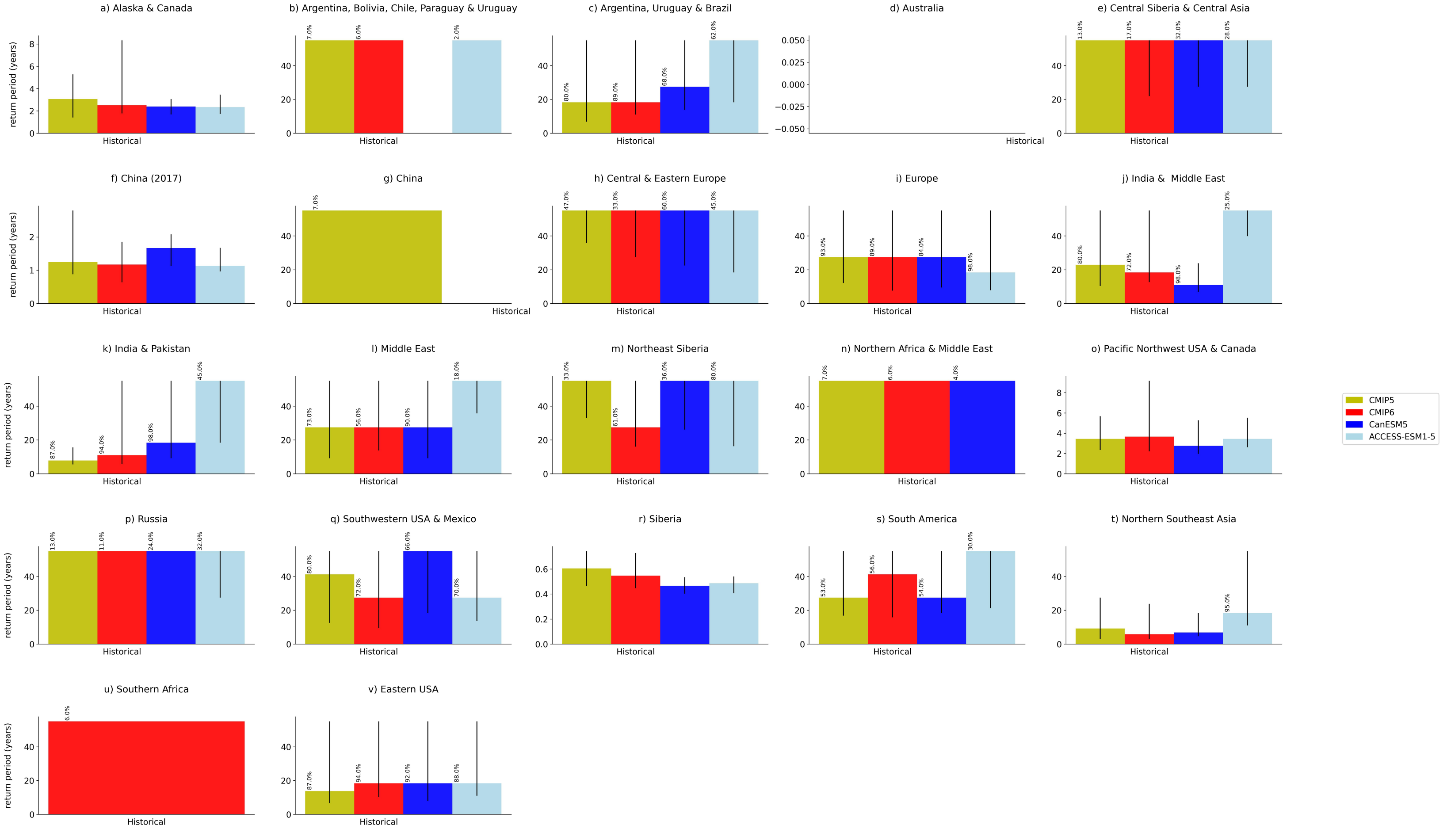
a) Alaska & Canada
b) Argentina, Bolivia, Chile, Paraguay & Uruguay
c) Argentina, Uruguay & Brazil
d) Australia
e) Central Siberia & Central Asia
f) China (2017)
g) China
h) Central & Eastern Europe
i) Europe
j) India & Middle East
k) India & Pakistan
l) Middle East
m) Northeast Siberia
n) Northern Africa & Middle East
o) Pacific Northwest USA & Canada
p) Russia
q) Southwestern USA & Mexico
r) Siberia
s) South America
t) Northern Southeast Asia
u) Southern Africa
v) Eastern USA
return period (years)
Historical
CMIP5
CMIP6
CanESM5
ACCESS-ESM1-5

Supplementary Figure 310: Return times (RT) during historical (1951-2005) based on ERA5 (Tx90p) - Return times (RT) (in years) during the historical period between 1951 and 2005 for Maximum Heatwave Area (%) of the respective observed event. For each region, values are shown for the different scenarios (increasing in emissions from left to right for the CMIP5 (green), CMIP6 (red), CanESM5 (dark blue), and ACCESS-ESM1-5 (light blue) ensembles. The error bars indicate the 5th and 95th percentile of the respective sample. Where the respective ensemble contains models not able to simulate the magnitude of the respective observed event's Maximum Heatwave Area (%), the proportion of models that are able to simulate an event with a value of Maximum Heatwave Area (%) at least as high as the observed event is indicated by the percentage values above the individual bars. The observed reference values are calculated using data from ERA5.

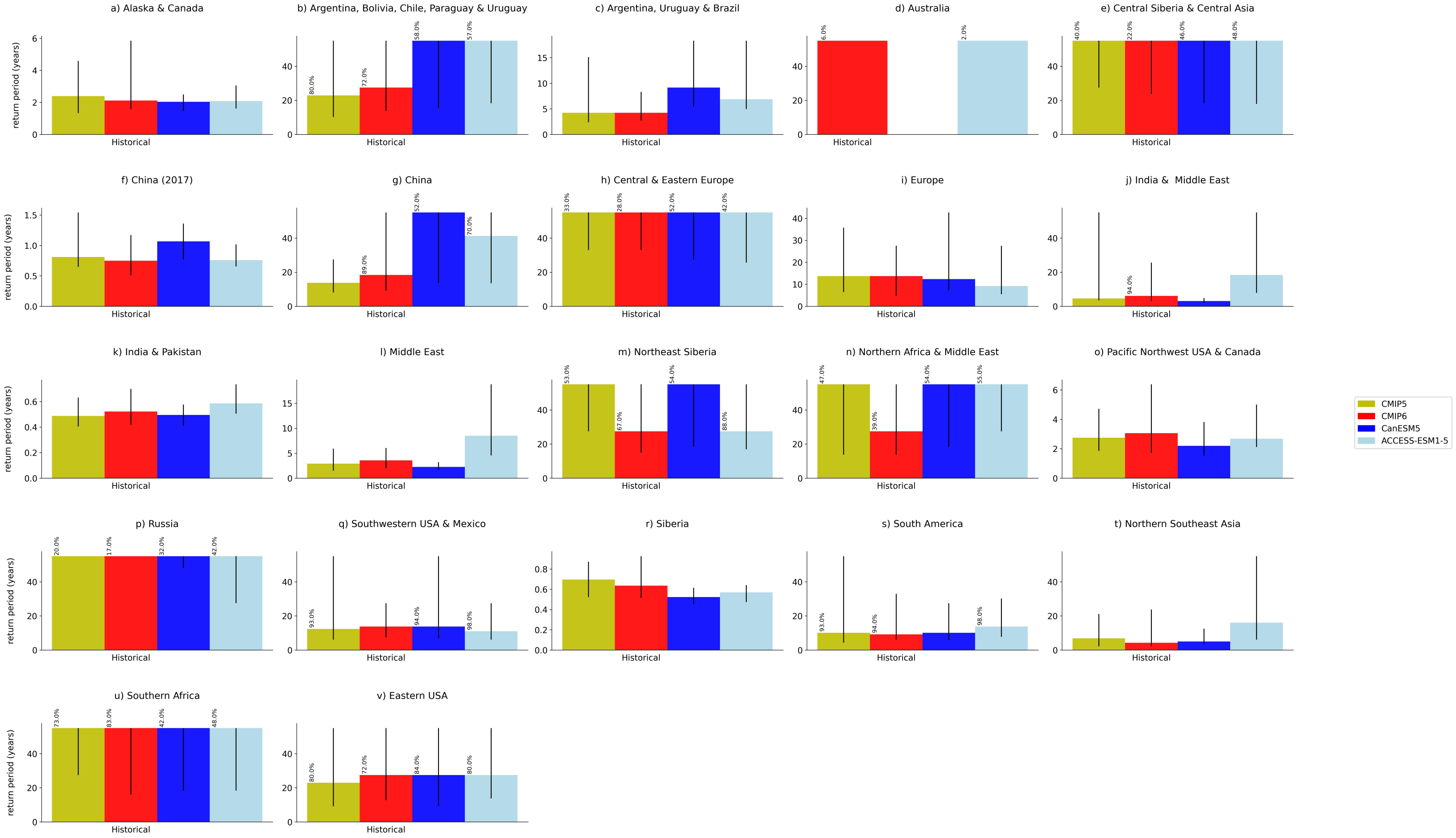

a) Alaska & Canada
b) Argentina, Bolivia, Chile, Paraguay & Uruguay
c) Argentina, Uruguay & Brazil
d) Australia
e) Central Siberia & Central Asia
f) China (2017)
g) China
h) Central & Eastern Europe
i) Europe
j) India & Middle East
k) India & Pakistan
l) Middle East
m) Northeast Siberia
n) Northern Africa & Middle East
o) Pacific Northwest USA & Canada
p) Russia
q) Southwestern USA & Mexico
r) Siberia
s) South America
t) Northern Southeast Asia
u) Southern Africa
v) Eastern USA
return period (years)
Historical
CMIP5
CMIP6
CanESM5
ACCESS-ESM1-5

Supplementary Figure 311: Return times (RT) during historical (1951-2005) based on Berkeley Earth (Tx90p) - Return times (RT) (in years) during the historical period between 1951 and 2005 for Maximum Heatwave Severity of the respective observed event. For each region, values are shown for the different scenarios (increasing in emissions from left to right for the CMIP5 (green), CMIP6 (red), CanESM5 (dark blue), and ACCESS-ESM1-5 (light blue) ensembles. The error bars indicate the 5th and 95th percentile of the respective sample. Where the respective ensemble contains models not able to simulate the magnitude of the respective observed event's Maximum Heatwave Severity, the proportion of models that are able to simulate an event with a value of Maximum Heatwave Severity at least as high as the observed event is indicated by the percentage values above the individual bars. The observed reference values are calculated using data from Berkeley Earth.

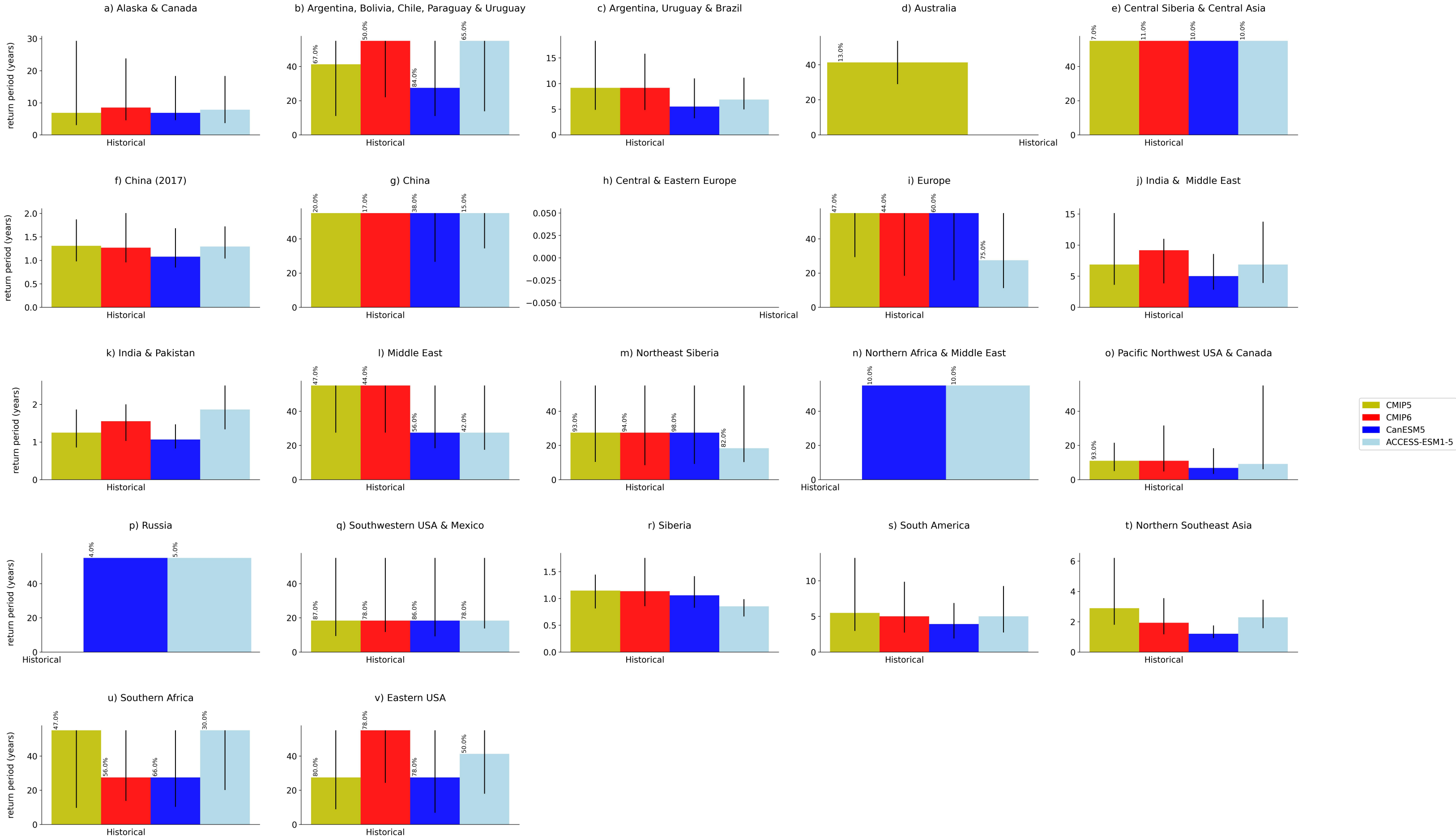
a) Alaska & Canada
b) Argentina, Bolivia, Chile, Paraguay & Uruguay
c) Argentina, Uruguay & Brazil
d) Australia
e) Central Siberia & Central Asia
f) China (2017)
g) China
h) Central & Eastern Europe
i) Europe
j) India & Middle East
k) India & Pakistan
l) Middle East
m) Northeast Siberia
n) Northern Africa & Middle East
o) Pacific Northwest USA & Canada
p) Russia
q) Southwestern USA & Mexico
r) Siberia
s) South America
t) Northern Southeast Asia
u) Southern Africa
v) Eastern USA
return period (years)
Historical
CMIP5
CMIP6
CanESM5
ACCESS-ESM1-5

Supplementary Figure 312: Return times (RT) during historical (1951-2005) based on ERA5 (Tx90p) - Return times (RT) (in years) during the historical period between 1951 and 2005 for Maximum Heatwave Severity of the respective observed event. For each region, values are shown for the different scenarios (increasing in emissions from left to right for the CMIP5 (green), CMIP6 (red), CanESM5 (dark blue), and ACCESS-ESM1-5 (light blue) ensembles. The error bars indicate the 5th and 95th percentile of the respective sample. Where the respective ensemble contains models not able to simulate the magnitude of the respective observed event's Maximum Heatwave Severity, the proportion of models that are able to simulate an event with a value of Maximum Heatwave Severity at least as high as the observed event is indicated by the percentage values above the individual bars. The observed reference values are calculated using data from ERA5.

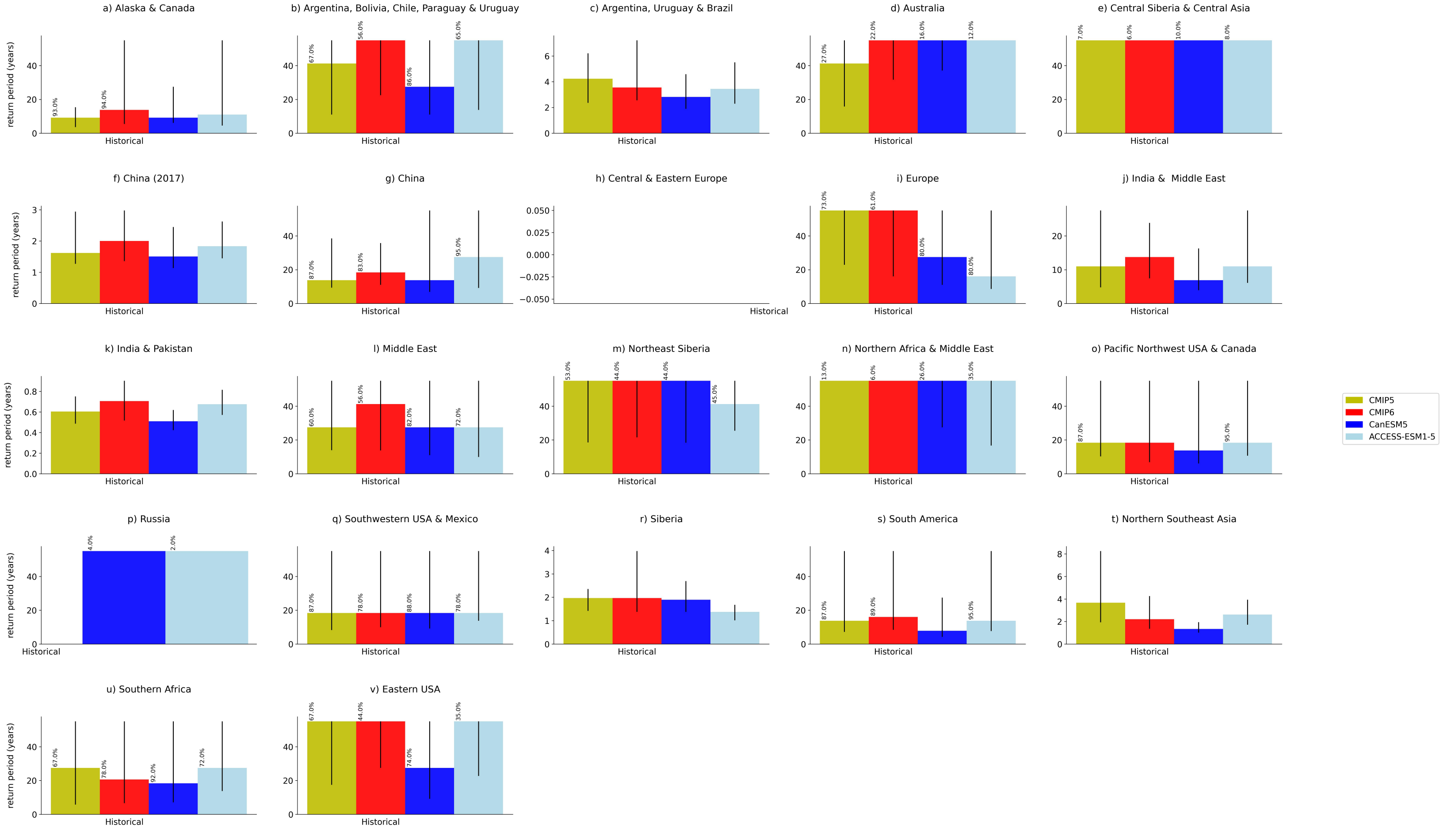

a) Alaska & Canada
b) Argentina, Bolivia, Chile, Paraguay & Uruguay
c) Argentina, Uruguay & Brazil
d) Australia
e) Central Siberia & Central Asia
f) China (2017)
g) China
h) Central & Eastern Europe
i) Europe
j) India & Middle East
k) India & Pakistan
l) Middle East
m) Northeast Siberia
n) Northern Africa & Middle East
o) Pacific Northwest USA & Canada
p) Russia
q) Southwestern USA & Mexico
r) Siberia
s) South America
t) Northern Southeast Asia
u) Southern Africa
v) Eastern USA
return period (years)
Historical
CMIP5
CMIP6
CanESM5
ACCESS-ESM1-5

Supplementary Figure 313: Return times (RT) during historical (1951-2005) based on Berkeley Earth (Tx90p) - Return times (RT) (in years) during the historical period between 1951 and 2005 for Median Heatwave Area (%) of the respective observed event. For each region, values are shown for the different scenarios (increasing in emissions from left to right for the CMIP5 (green), CMIP6 (red), CanESM5 (dark blue), and ACCESS-ESM1-5 (light blue) ensembles. The error bars indicate the 5th and 95th percentile of the respective sample. Where the respective ensemble contains models not able to simulate the magnitude of the respective observed event's Median Heatwave Area (%), the proportion of models that are able to simulate an event with a value of Median Heatwave Area (%) at least as high as the observed event is indicated by the percentage values above the individual bars. The observed reference values are calculated using data from Berkeley Earth.

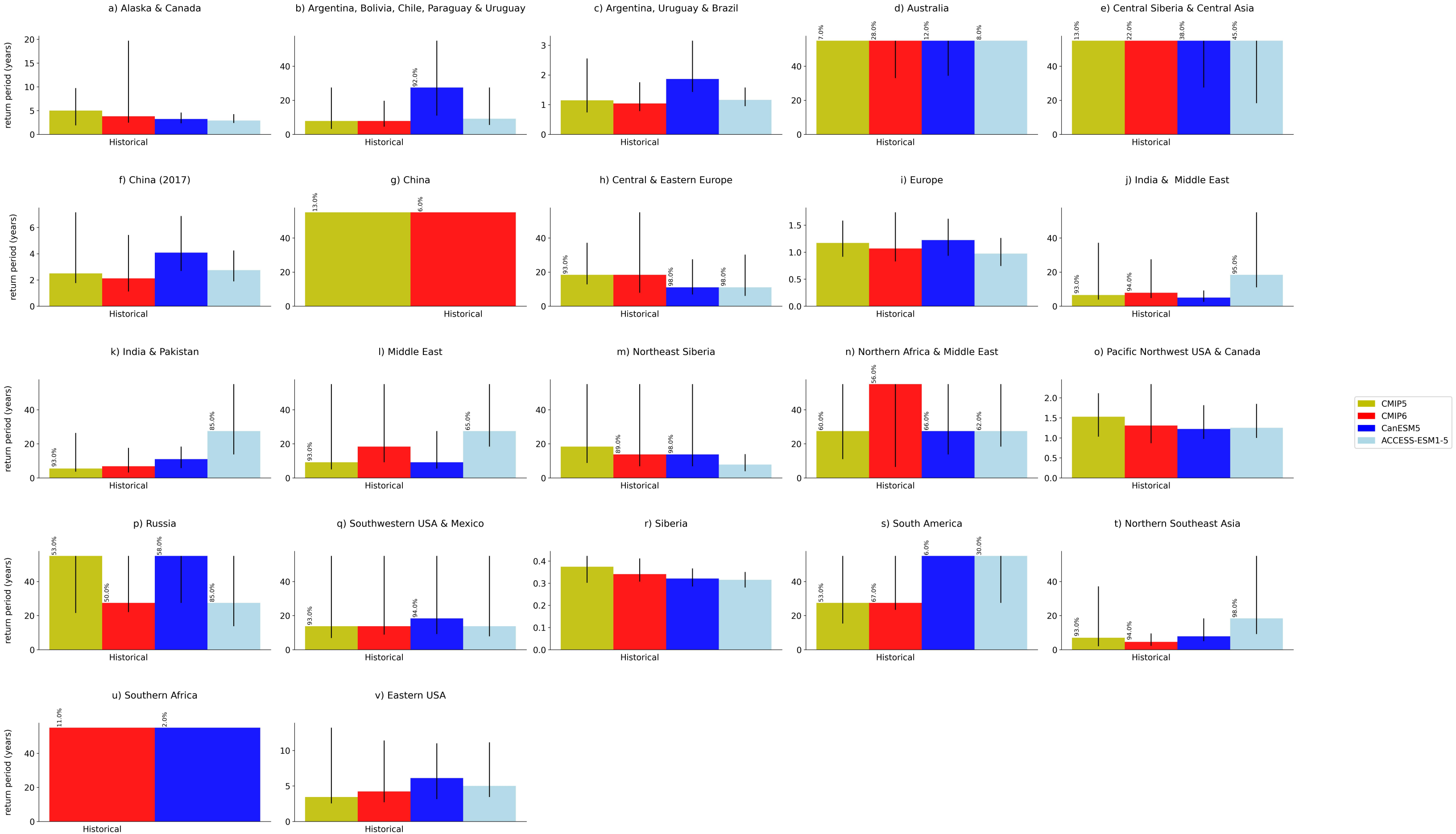
a) Alaska & Canada
b) Argentina, Bolivia, Chile, Paraguay & Uruguay
92.0%
c) Argentina, Uruguay & Brazil
d) Australia
7.0%
28.0%
12.0%
8.0%
e) Central Siberia & Central Asia
13.0%
22.0%
38.0%
45.0%
f) China (2017)
g) China
13.0%
6.0%
h) Central & Eastern Europe
93.0%
98.0%
98.0%
i) Europe
j) India & Middle East
93.0%
94.0%
95.0%
k) India & Pakistan
93.0%
85.0%
l) Middle East
93.0%
65.0%
m) Northeast Siberia
89.0%
98.0%
n) Northern Africa & Middle East
60.0%
56.0%
66.0%
62.0%
o) Pacific Northwest USA & Canada
p) Russia
53.0%
50.0%
58.0%
85.0%
q) Southwestern USA & Mexico
93.0%
94.0%
r) Siberia
s) South America
53.0%
67.0%
6.0%
30.0%
t) Northern Southeast Asia
93.0%
94.0%
98.0%
u) Southern Africa
11.0%
2.0%
v) Eastern USA
return period (years)
Historical
CMIP5
CMIP6
CanESM5
ACCESS-ESM1-5

Supplementary Figure 314: Return times (RT) during historical (1951-2005) based on ERA5 (Tx90p) - Return times (RT) (in years) during the historical period between 1951 and 2005 for Median Heatwave Area (%) of the respective observed event. For each region, values are shown for the different scenarios (increasing in emissions from left to right for the CMIP5 (green), CMIP6 (red), CanESM5 (dark blue), and ACCESS-ESM1-5 (light blue) ensembles. The error bars indicate the 5th and 95th percentile of the respective sample. Where the respective ensemble contains models not able to simulate the magnitude of the respective observed event's Median Heatwave Area (%), the proportion of models that are able to simulate an event with a value of Median Heatwave Area (%) at least as high as the observed event is indicated by the percentage values above the individual bars. The observed reference values are calculated using data from ERA5.

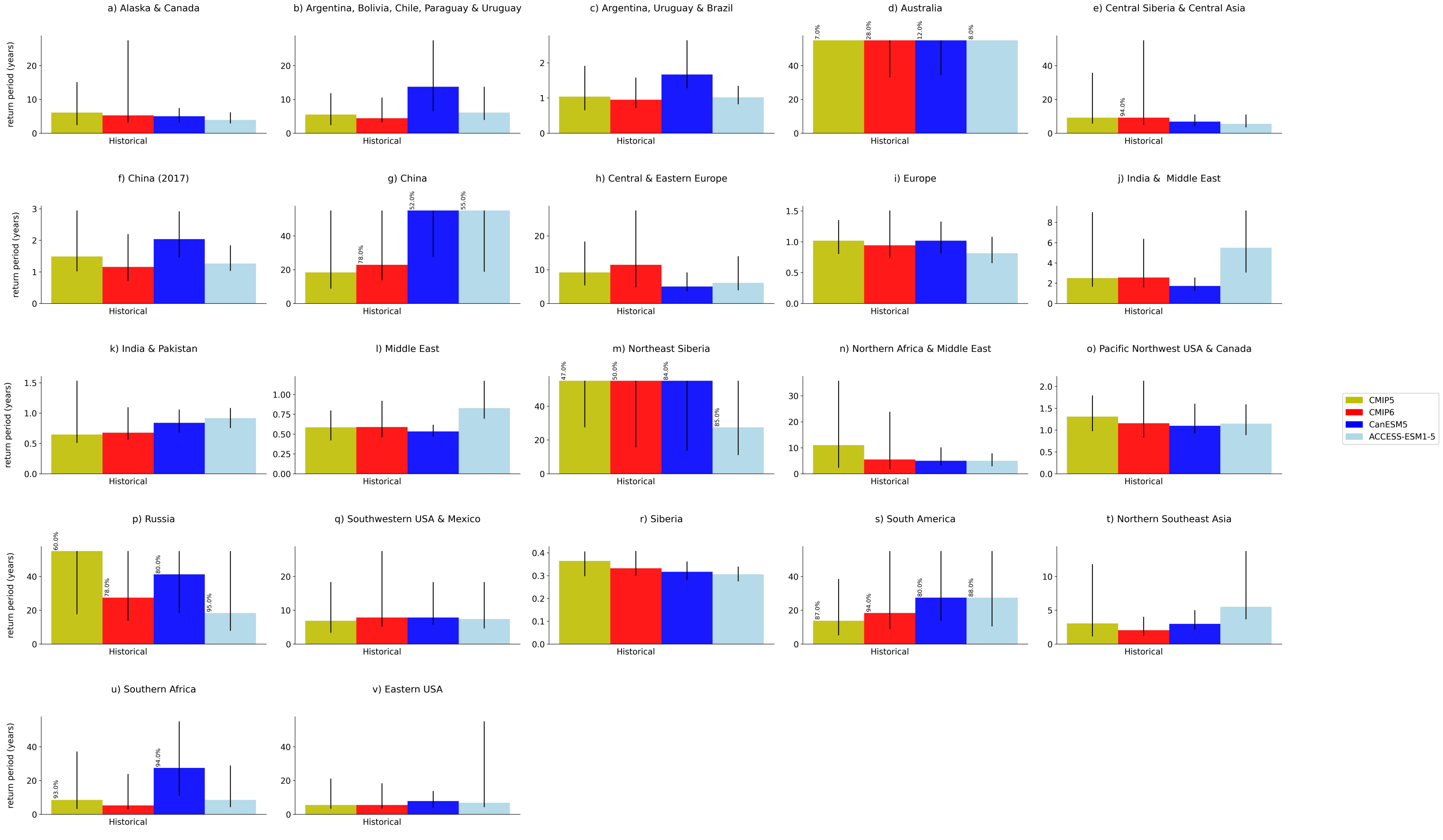
a) Alaska & Canada
b) Argentina, Bolivia, Chile, Paraguay & Uruguay
c) Argentina, Uruguay & Brazil
d) Australia
e) Central Siberia & Central Asia
f) China (2017)
g) China
h) Central & Eastern Europe
i) Europe
j) India & Middle East
k) India & Pakistan
l) Middle East
m) Northeast Siberia
n) Northern Africa & Middle East
o) Pacific Northwest USA & Canada
p) Russia
q) Southwestern USA & Mexico
r) Siberia
s) South America
t) Northern Southeast Asia
u) Southern Africa
v) Eastern USA
return period (years)
Historical
CMIP5
CMIP6
CanESM5
ACCESS-ESM1-5
7.0%
28.0%
12.0%
8.0%
94.0%
78.0%
52.0%
55.0%
47.0%
50.0%
84.0%
85.0%
60.0%
78.0%
80.0%
95.0%
87.0%
94.0%
80.0%
88.0%
93.0%
94.0%

Supplementary Figure 315: Return times (RT) during historical (1951-2005) based on Berkeley Earth (Tx90p) - Return times (RT) (in years) during the historical period between 1951 and 2005 for Median Heatwave Severity of the respective observed event. For each region, values are shown for the different scenarios (increasing in emissions from left to right for the CMIP5 (green), CMIP6 (red), CanESM5 (dark blue), and ACCESS-ESM1-5 (light blue) ensembles. The error bars indicate the 5th and 95th percentile of the respective sample. Where the respective ensemble contains models not able to simulate the magnitude of the respective observed event's Median Heatwave Severity, the proportion of models that are able to simulate an event with a value of Median Heatwave Severity at least as high as the observed event is indicated by the percentage values above the individual bars. The observed reference values are calculated using data from Berkeley Earth.

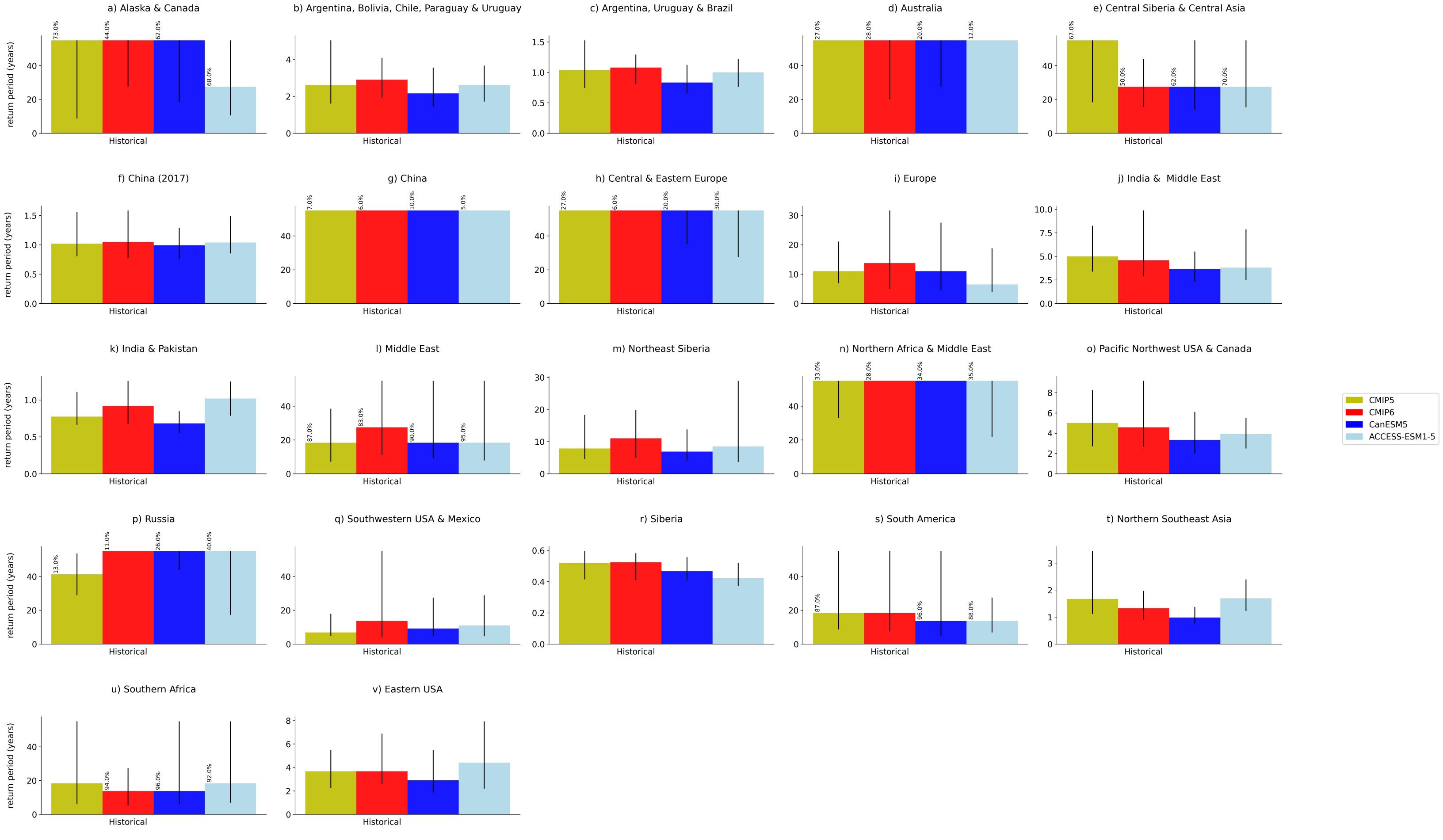
a) Alaska & Canada
b) Argentina, Bolivia, Chile, Paraguay & Uruguay
c) Argentina, Uruguay & Brazil
d) Australia
e) Central Siberia & Central Asia
f) China (2017)
g) China
h) Central & Eastern Europe
i) Europe
j) India & Middle East
k) India & Pakistan
l) Middle East
m) Northeast Siberia
n) Northern Africa & Middle East
o) Pacific Northwest USA & Canada
p) Russia
q) Southwestern USA & Mexico
r) Siberia
s) South America
t) Northern Southeast Asia
u) Southern Africa
v) Eastern USA
return period (years)
Historical
CMIP5
CMIP6
CanESM5
ACCESS-ESM1-5

Supplementary Figure 316: Return times (RT) during historical (1951-2005) based on ERA5 (Tx90p) - Return times (RT) (in years) during the historical period between 1951 and 2005 for Median Heatwave Severity of the respective observed event. For each region, values are shown for the different scenarios (increasing in emissions from left to right for the CMIP5 (green), CMIP6 (red), CanESM5 (dark blue), and ACCESS-ESM1-5 (light blue) ensembles. The error bars indicate the 5th and 95th percentile of the respective sample. Where the respective ensemble contains models not able to simulate the magnitude of the respective observed event's Median Heatwave Severity, the proportion of models that are able to simulate an event with a value of Median Heatwave Severity at least as high as the observed event is indicated by the percentage values above the individual bars. The observed reference values are calculated using data from ERA5.

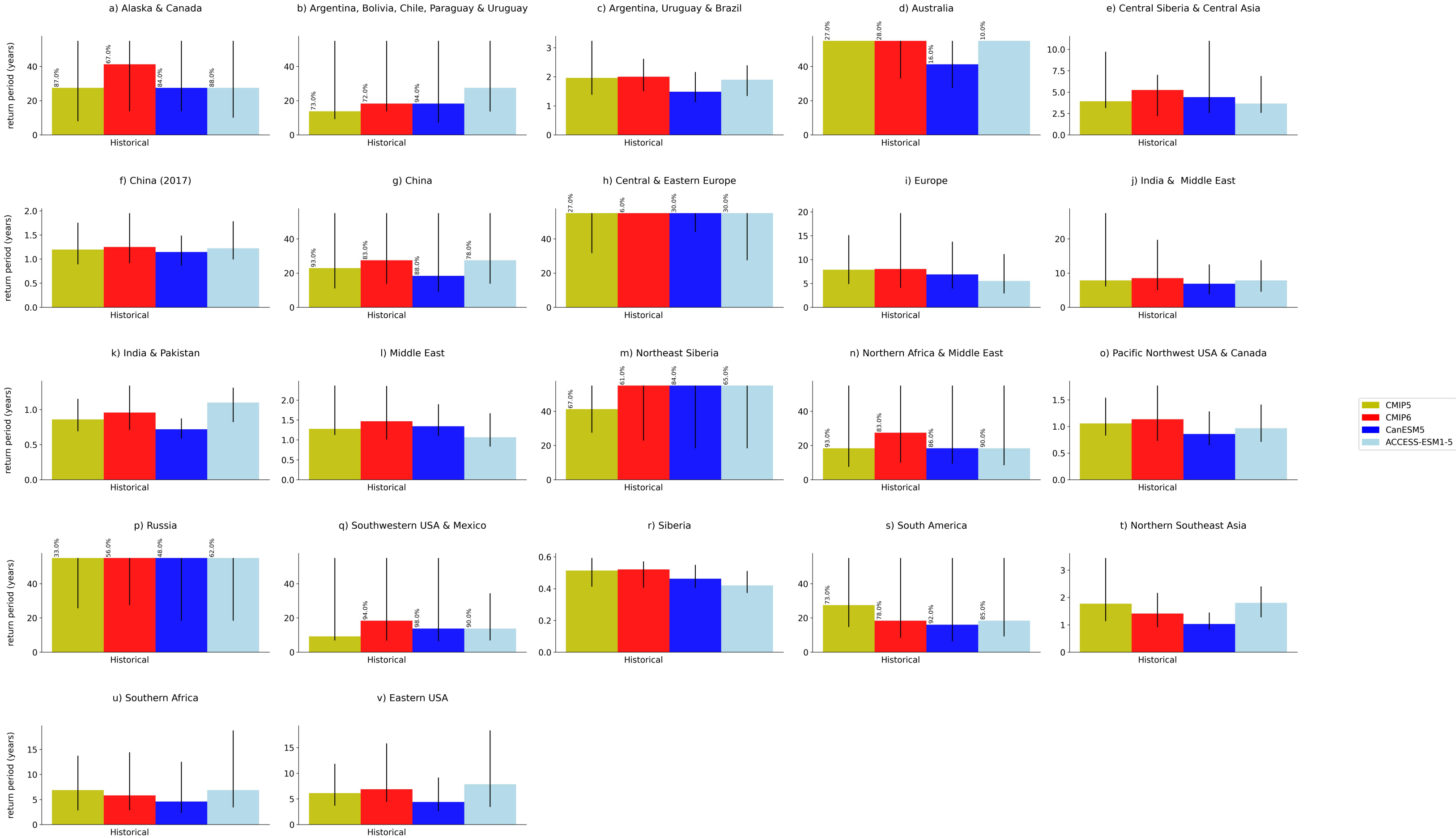
a) Alaska & Canada
b) Argentina, Bolivia, Chile, Paraguay & Uruguay
c) Argentina, Uruguay & Brazil
d) Australia
e) Central Siberia & Central Asia
f) China (2017)
g) China
h) Central & Eastern Europe
i) Europe
j) India & Middle East
k) India & Pakistan
l) Middle East
m) Northeast Siberia
n) Northern Africa & Middle East
o) Pacific Northwest USA & Canada
p) Russia
q) Southwestern USA & Mexico
r) Siberia
s) South America
t) Northern Southeast Asia
u) Southern Africa
v) Eastern USA
return period (years)
Historical
CMIP5
CMIP6
CanESM5
ACCESS-ESM1-5

Supplementary Figure 317: Return times (RT) during present-to-near-future (2015-2054) based on Berkeley Earth (Tx90p) - Return times (RT) (in years) during the present-to-near-future period between 2015 and 2054 for Cumulative Heat (°C) of the respective observed event. For each region, values are shown for the different scenarios (increasing in emissions from left to right for the CMIP5 (green), CMIP6 (red), CanESM5 (dark blue), and ACCESS-ESM1-5 (light blue) ensembles. The error bars indicate the 5th and 95th percentile of the respective sample. Where the respective ensemble contains models not able to simulate the magnitude of the respective observed event's Cumulative Heat (°C), the proportion of models that are able to simulate an event with a value of Cumulative Heat (°C) at least as high as the observed event is indicated by the percentage values above the individual bars. The observed reference values are calculated using data from Berkeley Earth.

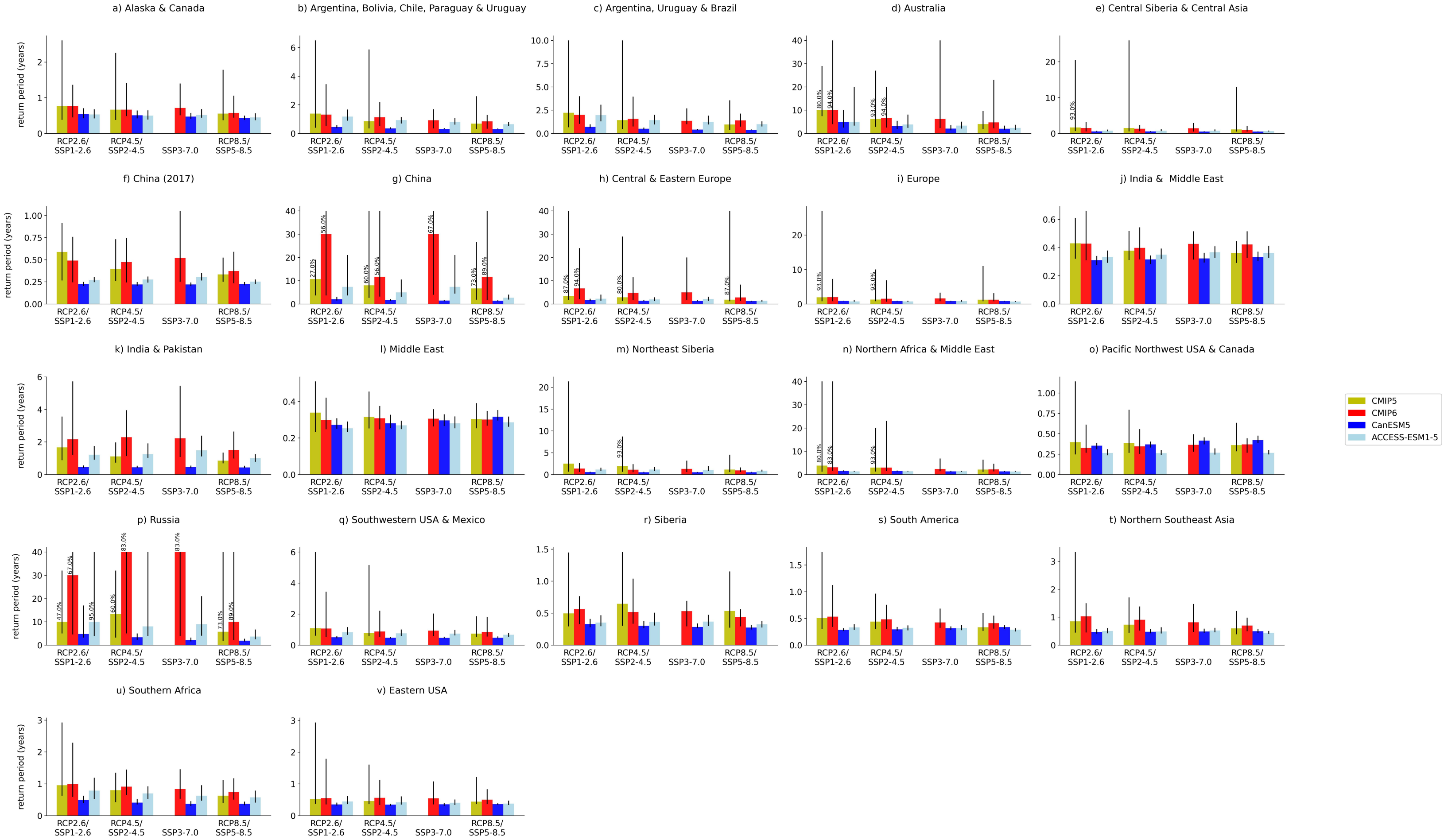
a) Alaska & Canada
b) Argentina, Bolivia, Chile, Paraguay & Uruguay
c) Argentina, Uruguay & Brazil
d) Australia
80.0%
94.0%
93.0%
94.0%
e) Central Siberia & Central Asia
93.0%
f) China (2017)
g) China
27.0%
56.0%
60.0%
56.0%
67.0%
73.0%
89.0%
h) Central & Eastern Europe
87.0%
94.0%
80.0%
87.0%
i) Europe
93.0%
93.0%
j) India & Middle East
k) India & Pakistan
l) Middle East
m) Northeast Siberia
93.0%
n) Northern Africa & Middle East
80.0%
83.0%
93.0%
o) Pacific Northwest USA & Canada
p) Russia
47.0%
67.0%
95.0%
60.0%
83.0%
83.0%
73.0%
89.0%
q) Southwestern USA & Mexico
r) Siberia
s) South America
t) Northern Southeast Asia
u) Southern Africa
v) Eastern USA
return period (years)
RCP2.6/ SSP1-2.6
RCP4.5/ SSP2-4.5
SSP3-7.0
RCP8.5/ SSP5-8.5
CMIP5
CMIP6
CanESM5
ACCESS-ESM1-5

Supplementary Figure 318: Return times (RT) during present-to-near-future (2015-2054) based on ERA5 (Tx90p) - Return times (RT) (in years) during the present-to-near-future period between 2015 and 2054 for Cumulative Heat (°C) of the respective observed event. For each region, values are shown for the different scenarios (increasing in emissions from left to right for the CMIP5 (green), CMIP6 (red), CanESM5 (dark blue), and ACCESS-ESM1-5 (light blue) ensembles. The error bars indicate the 5th and 95th percentile of the respective sample. Where the respective ensemble contains models not able to simulate the magnitude of the respective observed event's Cumulative Heat (°C), the proportion of models that are able to simulate an event with a value of Cumulative Heat (°C) at least as high as the observed event is indicated by the percentage values above the individual bars. The observed reference values are calculated using data from ERA5.

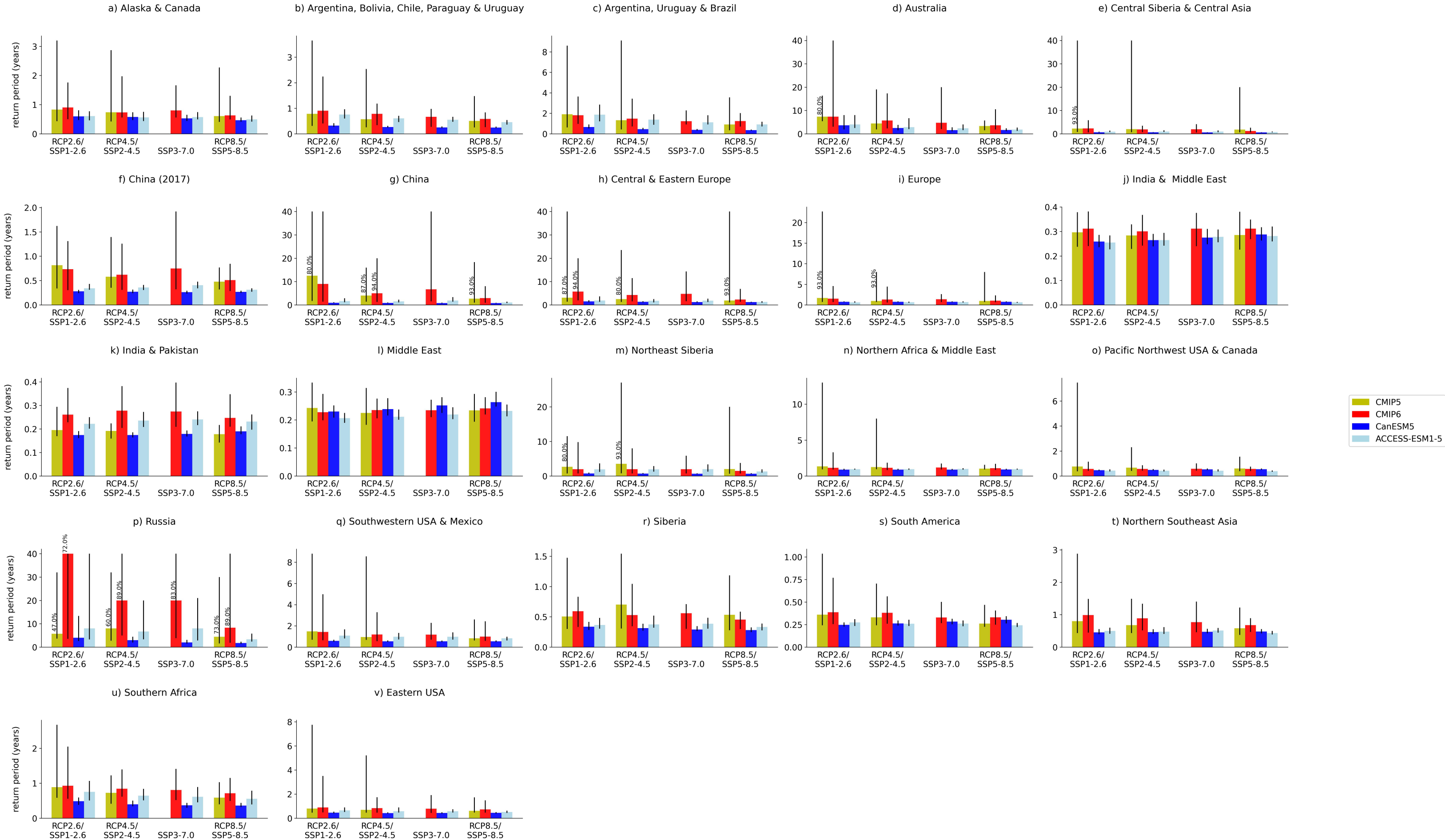
a) Alaska & Canada
b) Argentina, Bolivia, Chile, Paraguay & Uruguay
c) Argentina, Uruguay & Brazil
d) Australia
e) Central Siberia & Central Asia
f) China (2017)
g) China
h) Central & Eastern Europe
i) Europe
j) India & Middle East
k) India & Pakistan
l) Middle East
m) Northeast Siberia
n) Northern Africa & Middle East
o) Pacific Northwest USA & Canada
p) Russia
q) Southwestern USA & Mexico
r) Siberia
s) South America
t) Northern Southeast Asia
u) Southern Africa
v) Eastern USA
return period (years)
RCP2.6/ SSP1-2.6
RCP4.5/ SSP2-4.5
SSP3-7.0
RCP8.5/ SSP5-8.5
80.0%
93.0%
87.0%
94.0%
47.0%
72.0%
60.0%
89.0%
83.0%
73.0%
CMIP5
CMIP6
CanESM5
ACCESS-ESM1-5

Supplementary Figure 319: Return times (RT) during present-to-near-future (2015-2054) based on Berkeley Earth (Tx90p) - Return times (RT) (in years) during the present-to-near-future period between 2015 and 2054 for Heatwave Length (days) of the respective observed event. For each region, values are shown for the different scenarios (increasing in emissions from left to right for the CMIP5 (green), CMIP6 (red), CanESM5 (dark blue), and ACCESS-ESM1-5 (light blue) ensembles. The error bars indicate the 5th and 95th percentile of the respective sample. Where the respective ensemble contains models not able to simulate the magnitude of the respective observed event's Heatwave Length (days), the proportion of models that are able to simulate an event with a value of Heatwave Length (days) at least as high as the observed event is indicated by the percentage values above the individual bars. The observed reference values are calculated using data from Berkeley Earth.

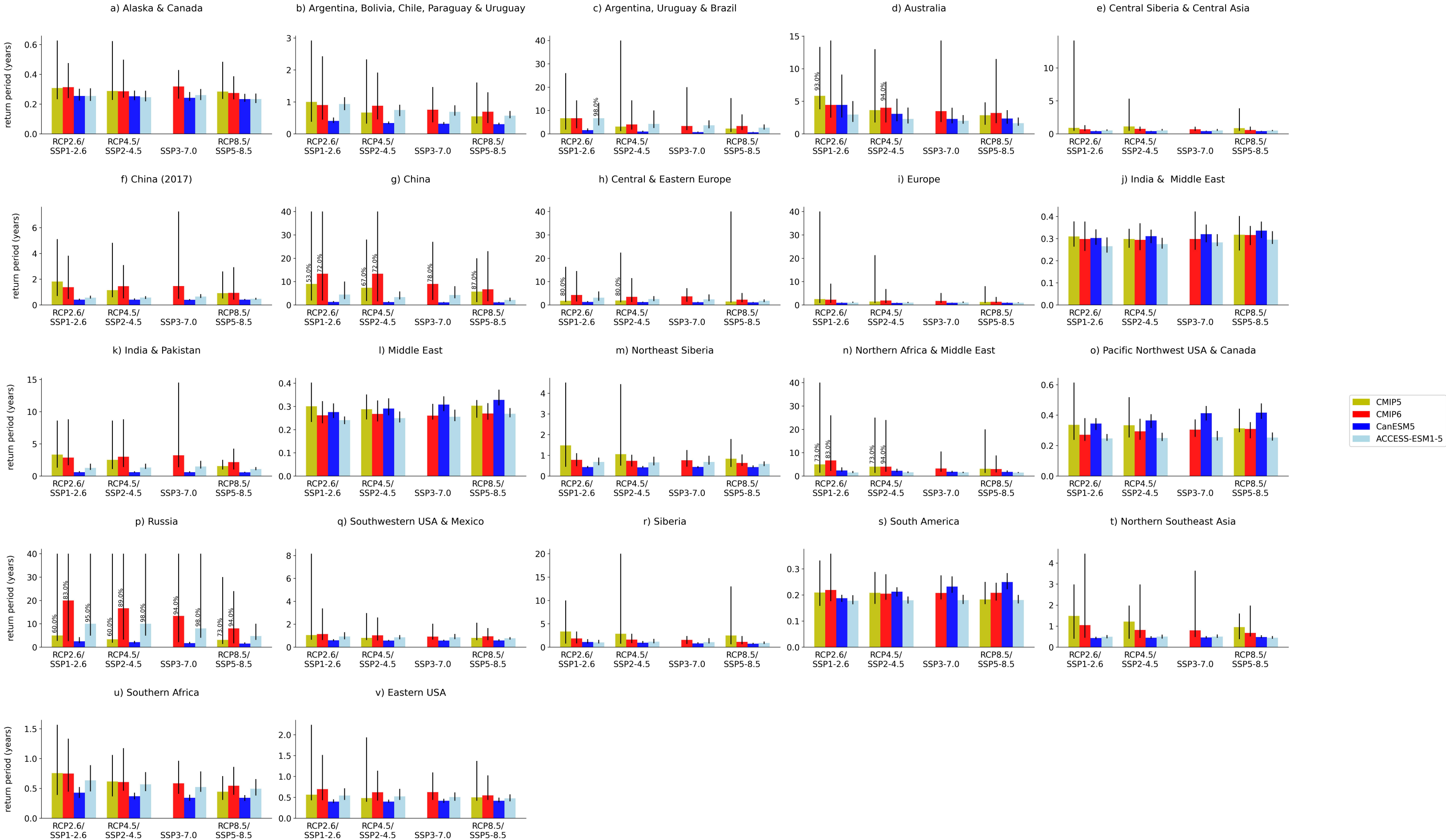
a) Alaska & Canada
b) Argentina, Bolivia, Chile, Paraguay & Uruguay
c) Argentina, Uruguay & Brazil
d) Australia
e) Central Siberia & Central Asia
f) China (2017)
g) China
h) Central & Eastern Europe
i) Europe
j) India & Middle East
k) India & Pakistan
l) Middle East
m) Northeast Siberia
n) Northern Africa & Middle East
o) Pacific Northwest USA & Canada
p) Russia
q) Southwestern USA & Mexico
r) Siberia
s) South America
t) Northern Southeast Asia
u) Southern Africa
v) Eastern USA
return period (years)
RCP2.6/
SSP1-2.6
RCP4.5/
SSP2-4.5
SSP3-7.0
RCP8.5/
SSP5-8.5
CMIP5
CMIP6
CanESM5
ACCESS-ESM1-5

Supplementary Figure 320: Return times (RT) during present-to-near-future (2015-2054) based on ERA5 (Tx90p) - Return times (RT) (in years) during the present-to-near-future period between 2015 and 2054 for Heatwave Length (days) of the respective observed event. For each region, values are shown for the different scenarios (increasing in emissions from left to right for the CMIP5 (green), CMIP6 (red), CanESM5 (dark blue), and ACCESS-ESM1-5 (light blue) ensembles. The error bars indicate the 5th and 95th percentile of the respective sample. Where the respective ensemble contains models not able to simulate the magnitude of the respective observed event's Heatwave Length (days), the proportion of models that are able to simulate an event with a value of Heatwave Length (days) at least as high as the observed event is indicated by the percentage values above the individual bars. The observed reference values are calculated using data from ERA5.

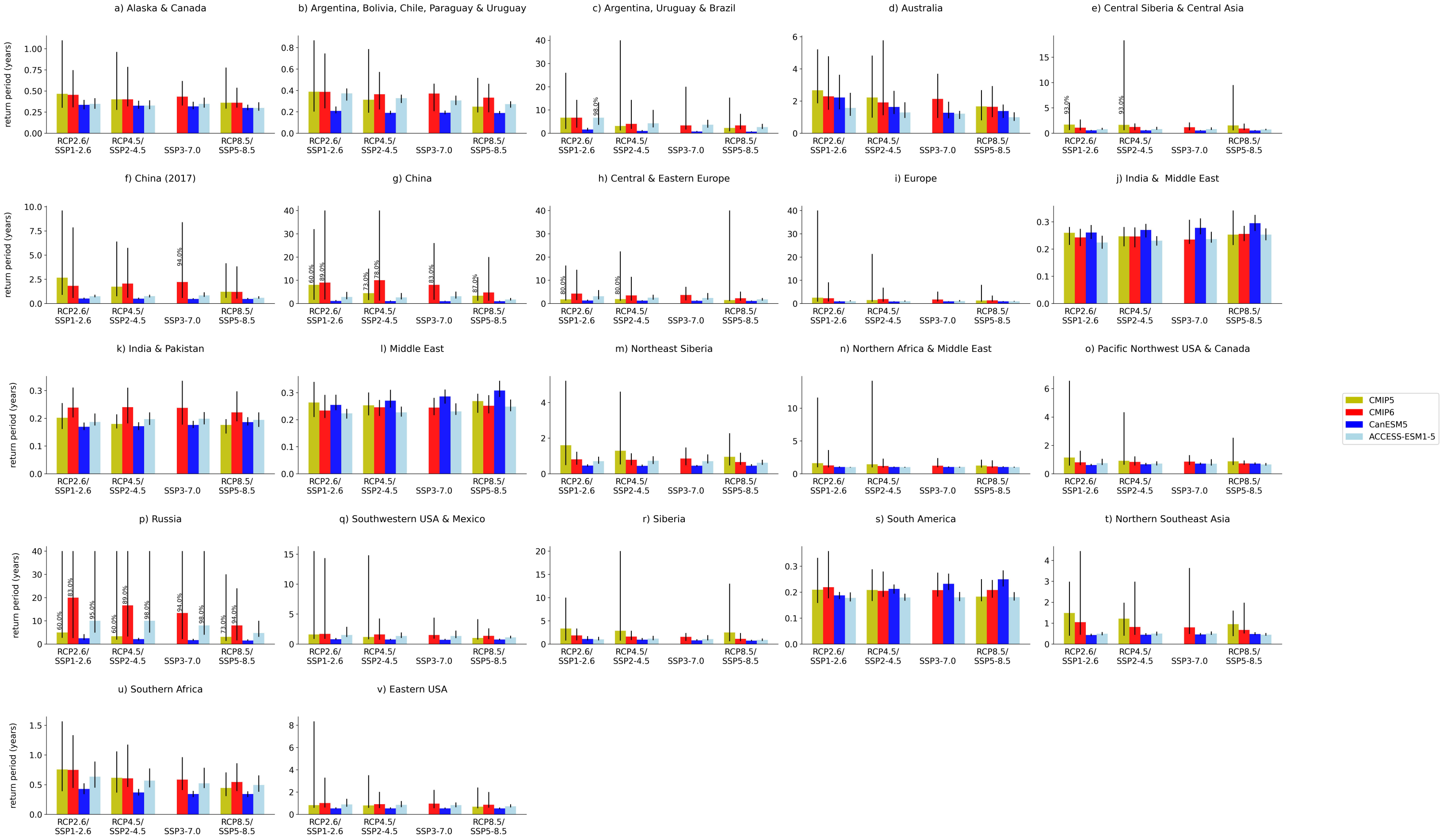
a) Alaska & Canada
b) Argentina, Bolivia, Chile, Paraguay & Uruguay
c) Argentina, Uruguay & Brazil
d) Australia
e) Central Siberia & Central Asia
f) China (2017)
g) China
h) Central & Eastern Europe
i) Europe
j) India & Middle East
k) India & Pakistan
l) Middle East
m) Northeast Siberia
n) Northern Africa & Middle East
o) Pacific Northwest USA & Canada
p) Russia
q) Southwestern USA & Mexico
r) Siberia
s) South America
t) Northern Southeast Asia
u) Southern Africa
v) Eastern USA
return period (years)
RCP2.6/
SSP1-2.6
RCP4.5/
SSP2-4.5
SSP3-7.0
RCP8.5/
SSP5-8.5
CMIP5
CMIP6
CanESM5
ACCESS-ESM1-5
98.0%
93.0%
94.0%
60.0%
89.0%
73.0%
78.0%
83.0%
87.0%
80.0%
95.0%

Supplementary Figure 321: Return times (RT) during present-to-near-future (2015-2054) based on Berkeley Earth (Tx90p) - Return times (RT) (in years) during the present-to-near-future period between 2015 and 2054 for Maximum Heatwave Area (%) of the respective observed event. For each region, values are shown for the different scenarios (increasing in emissions from left to right for the CMIP5 (green), CMIP6 (red), CanESM5 (dark blue), and ACCESS-ESM1-5 (light blue) ensembles. The error bars indicate the 5th and 95th percentile of the respective sample. Where the respective ensemble contains models not able to simulate the magnitude of the respective observed event's Maximum Heatwave Area (%), the proportion of models that are able to simulate an event with a value of Maximum Heatwave Area (%) at least as high as the observed event is indicated by the percentage values above the individual bars. The observed reference values are calculated using data from Berkeley Earth.

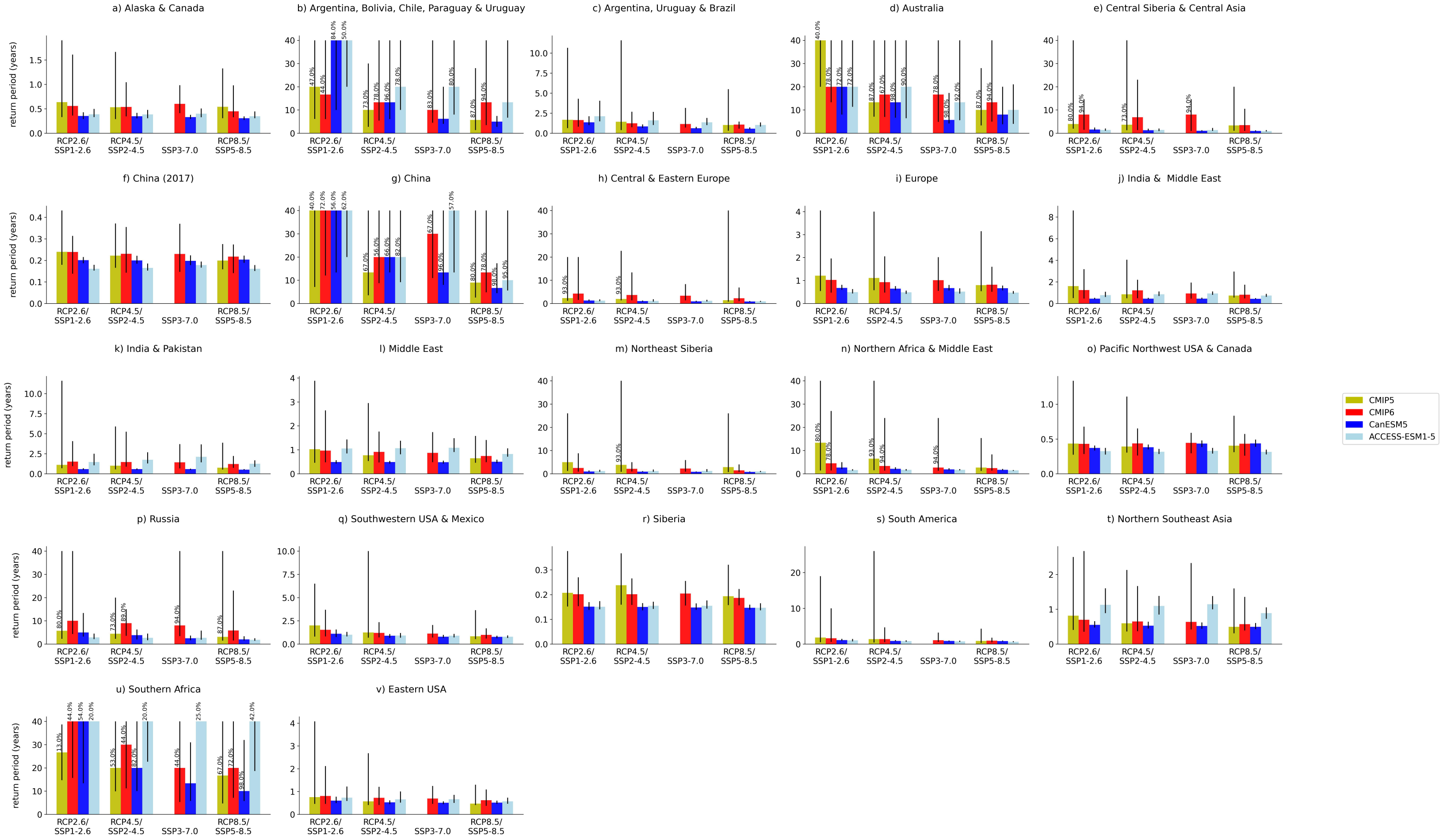

a) Alaska & Canada
b) Argentina, Bolivia, Chile, Paraguay & Uruguay
c) Argentina, Uruguay & Brazil
d) Australia
e) Central Siberia & Central Asia
f) China (2017)
g) China
h) Central & Eastern Europe
i) Europe
j) India & Middle East
k) India & Pakistan
l) Middle East
m) Northeast Siberia
n) Northern Africa & Middle East
o) Pacific Northwest USA & Canada
p) Russia
q) Southwestern USA & Mexico
r) Siberia
s) South America
t) Northern Southeast Asia
u) Southern Africa
v) Eastern USA
return period (years)
RCP2.6/ SSP1-2.6
RCP4.5/ SSP2-4.5
SSP3-7.0
RCP8.5/ SSP5-8.5
CMIP5
CMIP6
CanESM5
ACCESS-ESM1-5

Supplementary Figure 322: Return times (RT) during present-to-near-future (2015-2054) based on ERA5 (Tx90p) - Return times (RT) (in years) during the present-to-near-future period between 2015 and 2054 for Maximum Heatwave Area (%) of the respective observed event. For each region, values are shown for the different scenarios (increasing in emissions from left to right for the CMIP5 (green), CMIP6 (red), CanESM5 (dark blue), and ACCESS-ESM1-5 (light blue) ensembles. The error bars indicate the 5th and 95th percentile of the respective sample. Where the respective ensemble contains models not able to simulate the magnitude of the respective observed event's Maximum Heatwave Area (%), the proportion of models that are able to simulate an event with a value of Maximum Heatwave Area (%) at least as high as the observed event is indicated by the percentage values above the individual bars. The observed reference values are calculated using data from ERA5.

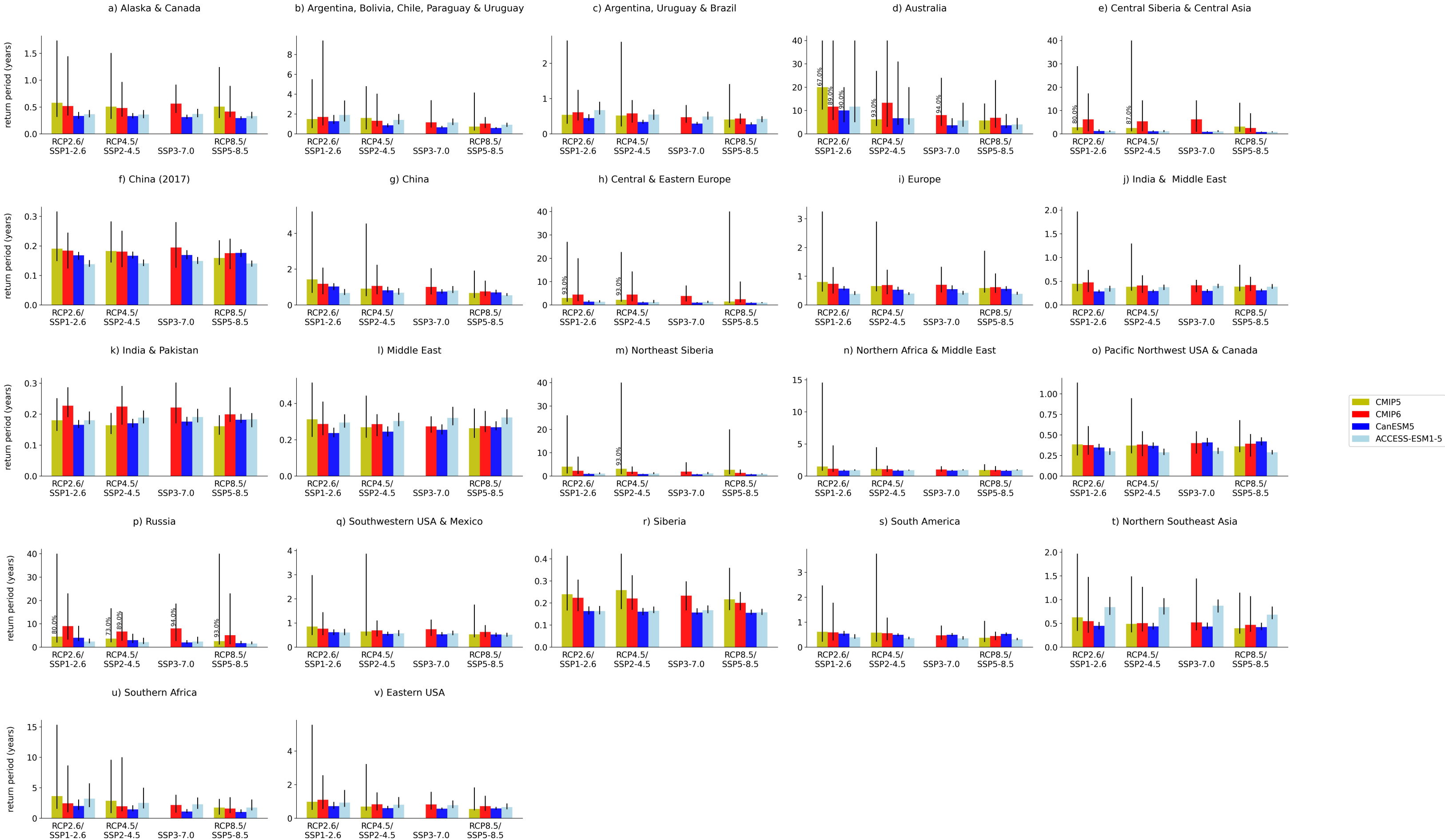
a) Alaska & Canada
b) Argentina, Bolivia, Chile, Paraguay & Uruguay
c) Argentina, Uruguay & Brazil
d) Australia
e) Central Siberia & Central Asia
f) China (2017)
g) China
h) Central & Eastern Europe
i) Europe
j) India & Middle East
k) India & Pakistan
l) Middle East
m) Northeast Siberia
n) Northern Africa & Middle East
o) Pacific Northwest USA & Canada
p) Russia
q) Southwestern USA & Mexico
r) Siberia
s) South America
t) Northern Southeast Asia
u) Southern Africa
v) Eastern USA
return period (years)
RCP2.6/ SSP1-2.6
RCP4.5/ SSP2-4.5
SSP3-7.0
RCP8.5/ SSP5-8.5
CMIP5
CMIP6
CanESM5
ACCESS-ESM1-5
67.0%
89.0%
90.0%
93.0%
94.0%
80.0%
87.0%
73.0%

Supplementary Figure 323: Return times (RT) during present-to-near-future (2015-2054) based on Berkeley Earth (Tx90p) - Return times (RT) (in years) during the present-to-near-future period between 2015 and 2054 for Maximum Heatwave Severity of the respective observed event. For each region, values are shown for the different scenarios (increasing in emissions from left to right for the CMIP5 (green), CMIP6 (red), CanESM5 (dark blue), and ACCESS-ESM1-5 (light blue) ensembles. The error bars indicate the 5th and 95th percentile of the respective sample. Where the respective ensemble contains models not able to simulate the magnitude of the respective observed event's Maximum Heatwave Severity, the proportion of models that are able to simulate an event with a value of Maximum Heatwave Severity at least as high as the observed event is indicated by the percentage values above the individual bars. The observed reference values are calculated using data from Berkeley Earth.

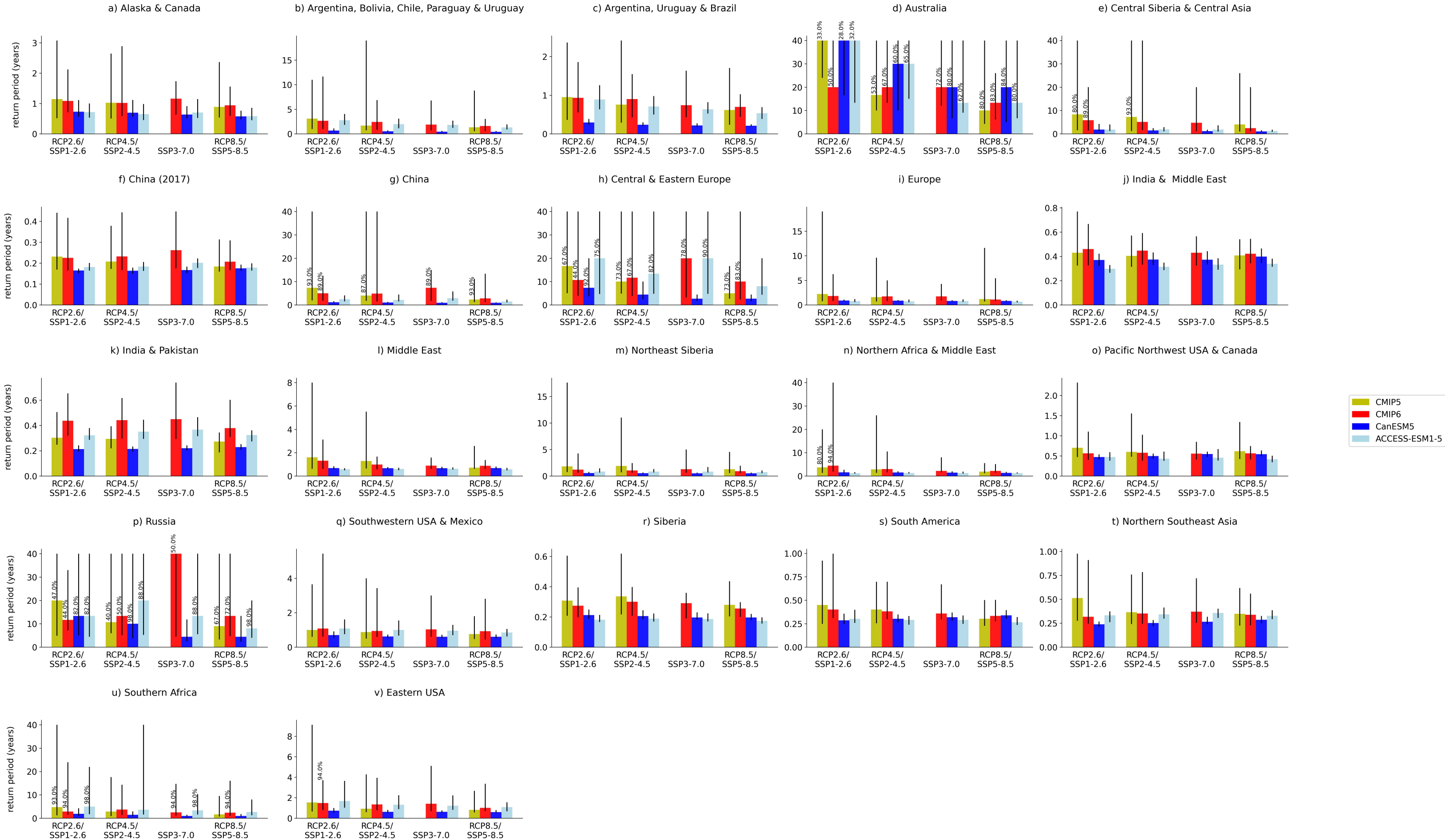
a) Alaska & Canada
b) Argentina, Bolivia, Chile, Paraguay & Uruguay
c) Argentina, Uruguay & Brazil
d) Australia
e) Central Siberia & Central Asia
f) China (2017)
g) China
h) Central & Eastern Europe
i) Europe
j) India & Middle East
k) India & Pakistan
l) Middle East
m) Northeast Siberia
n) Northern Africa & Middle East
o) Pacific Northwest USA & Canada
p) Russia
q) Southwestern USA & Mexico
r) Siberia
s) South America
t) Northern Southeast Asia
u) Southern Africa
v) Eastern USA
return period (years)
RCP2.6/ SSP1-2.6
RCP4.5/ SSP2-4.5
SSP3-7.0
RCP8.5/ SSP5-8.5
CMIP5
CMIP6
CanESM5
ACCESS-ESM1-5

Supplementary Figure 324: Return times (RT) during present-to-near-future (2015-2054) based on ERA5 (Tx90p) - Return times (RT) (in years) during the present-to-near-future period between 2015 and 2054 for Maximum Heatwave Severity of the respective observed event. For each region, values are shown for the different scenarios (increasing in emissions from left to right for the CMIP5 (green), CMIP6 (red), CanESM5 (dark blue), and ACCESS-ESM1-5 (light blue) ensembles. The error bars indicate the 5th and 95th percentile of the respective sample. Where the respective ensemble contains models not able to simulate the magnitude of the respective observed event's Maximum Heatwave Severity, the proportion of models that are able to simulate an event with a value of Maximum Heatwave Severity at least as high as the observed event is indicated by the percentage values above the individual bars. The observed reference values are calculated using data from ERA5.

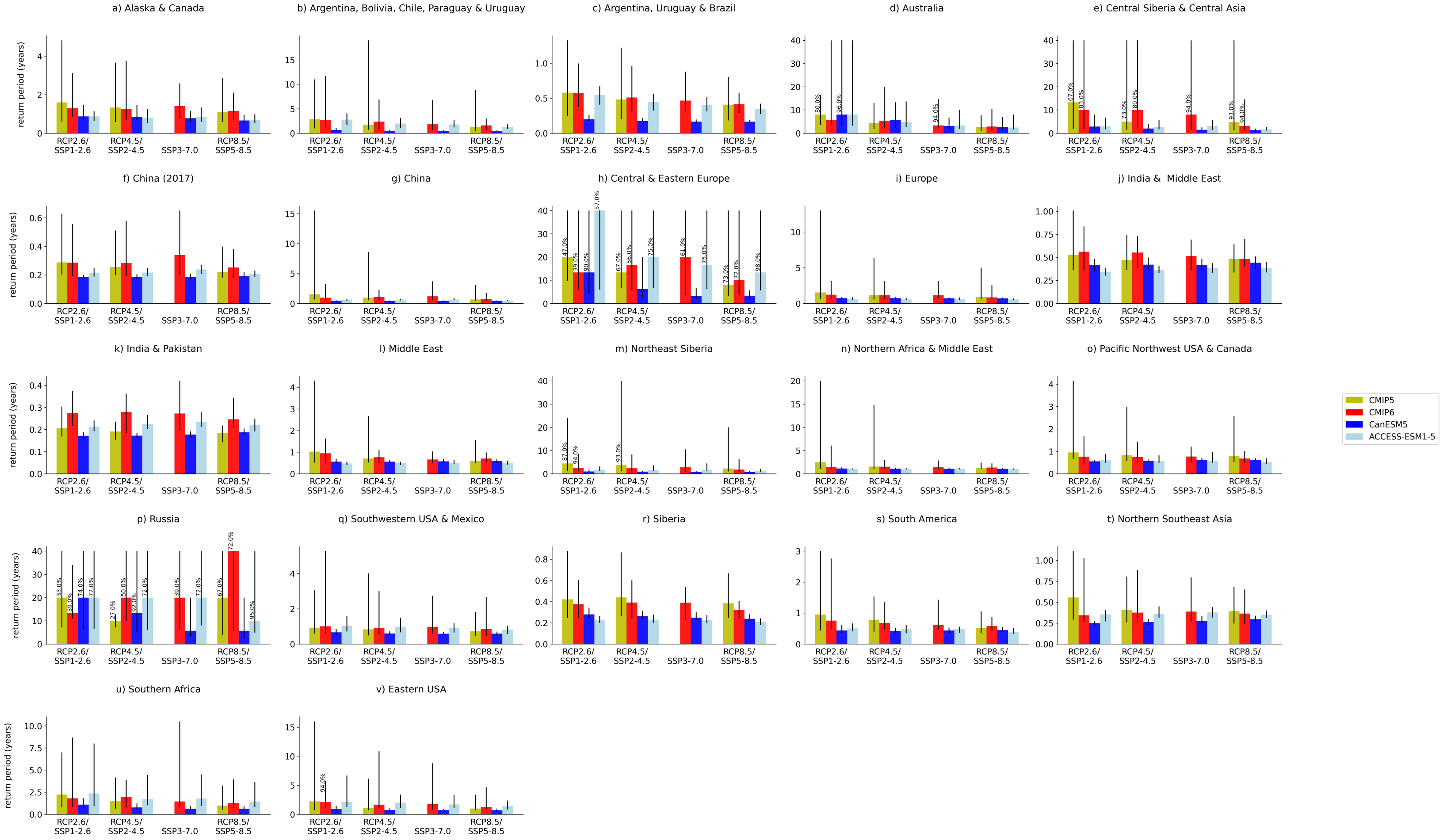
a) Alaska & Canada
b) Argentina, Bolivia, Chile, Paraguay & Uruguay
c) Argentina, Uruguay & Brazil
d) Australia
e) Central Siberia & Central Asia
f) China (2017)
g) China
h) Central & Eastern Europe
i) Europe
j) India & Middle East
k) India & Pakistan
l) Middle East
m) Northeast Siberia
n) Northern Africa & Middle East
o) Pacific Northwest USA & Canada
p) Russia
q) Southwestern USA & Mexico
r) Siberia
s) South America
t) Northern Southeast Asia
u) Southern Africa
v) Eastern USA
return period (years)
RCP2.6/ SSP1-2.6
RCP4.5/ SSP2-4.5
SSP3-7.0
RCP8.5/ SSP5-8.5
CMIP5
CMIP6
CanESM5
ACCESS-ESM1-5

Supplementary Figure 325: Return times (RT) during present-to-near-future (2015-2054) based on Berkeley Earth (Tx90p) - Return times (RT) (in years) during the present-to-near-future period between 2015 and 2054 for Median Heatwave Area (%) of the respective observed event. For each region, values are shown for the different scenarios (increasing in emissions from left to right for the CMIP5 (green), CMIP6 (red), CanESM5 (dark blue), and ACCESS-ESM1-5 (light blue) ensembles. The error bars indicate the 5th and 95th percentile of the respective sample. Where the respective ensemble contains models not able to simulate the magnitude of the respective observed event's Median Heatwave Area (%), the proportion of models that are able to simulate an event with a value of Median Heatwave Area (%) at least as high as the observed event is indicated by the percentage values above the individual bars. The observed reference values are calculated using data from Berkeley Earth.

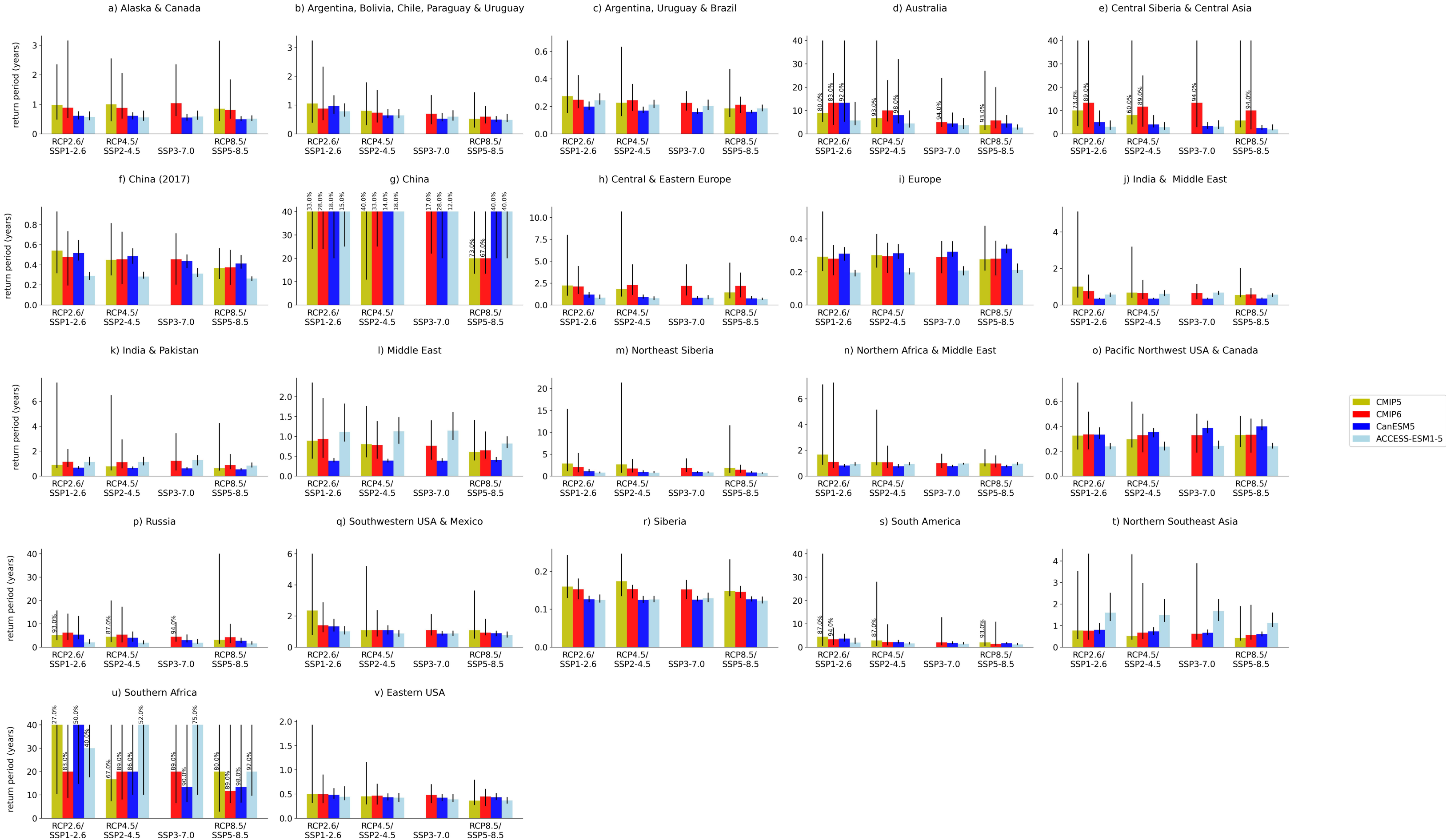
a) Alaska & Canada
b) Argentina, Bolivia, Chile, Paraguay & Uruguay
c) Argentina, Uruguay & Brazil
d) Australia
e) Central Siberia & Central Asia
f) China (2017)
g) China
h) Central & Eastern Europe
i) Europe
j) India & Middle East
k) India & Pakistan
l) Middle East
m) Northeast Siberia
n) Northern Africa & Middle East
o) Pacific Northwest USA & Canada
p) Russia
q) Southwestern USA & Mexico
r) Siberia
s) South America
t) Northern Southeast Asia
u) Southern Africa
v) Eastern USA
return period (years)
RCP2.6/ SSP1-2.6
RCP4.5/ SSP2-4.5
SSP3-7.0
RCP8.5/ SSP5-8.5
CMIP5
CMIP6
CanESM5
ACCESS-ESM1-5

Supplementary Figure 326: Return times (RT) during present-to-near-future (2015-2054) based on ERA5 (Tx90p) - Return times (RT) (in years) during the present-to-near-future period between 2015 and 2054 for Median Heatwave Area (%) of the respective observed event. For each region, values are shown for the different scenarios (increasing in emissions from left to right for the CMIP5 (green), CMIP6 (red), CanESM5 (dark blue), and ACCESS-ESM1-5 (light blue) ensembles. The error bars indicate the 5th and 95th percentile of the respective sample. Where the respective ensemble contains models not able to simulate the magnitude of the respective observed event's Median Heatwave Area (%), the proportion of models that are able to simulate an event with a value of Median Heatwave Area (%) at least as high as the observed event is indicated by the percentage values above the individual bars. The observed reference values are calculated using data from ERA5.

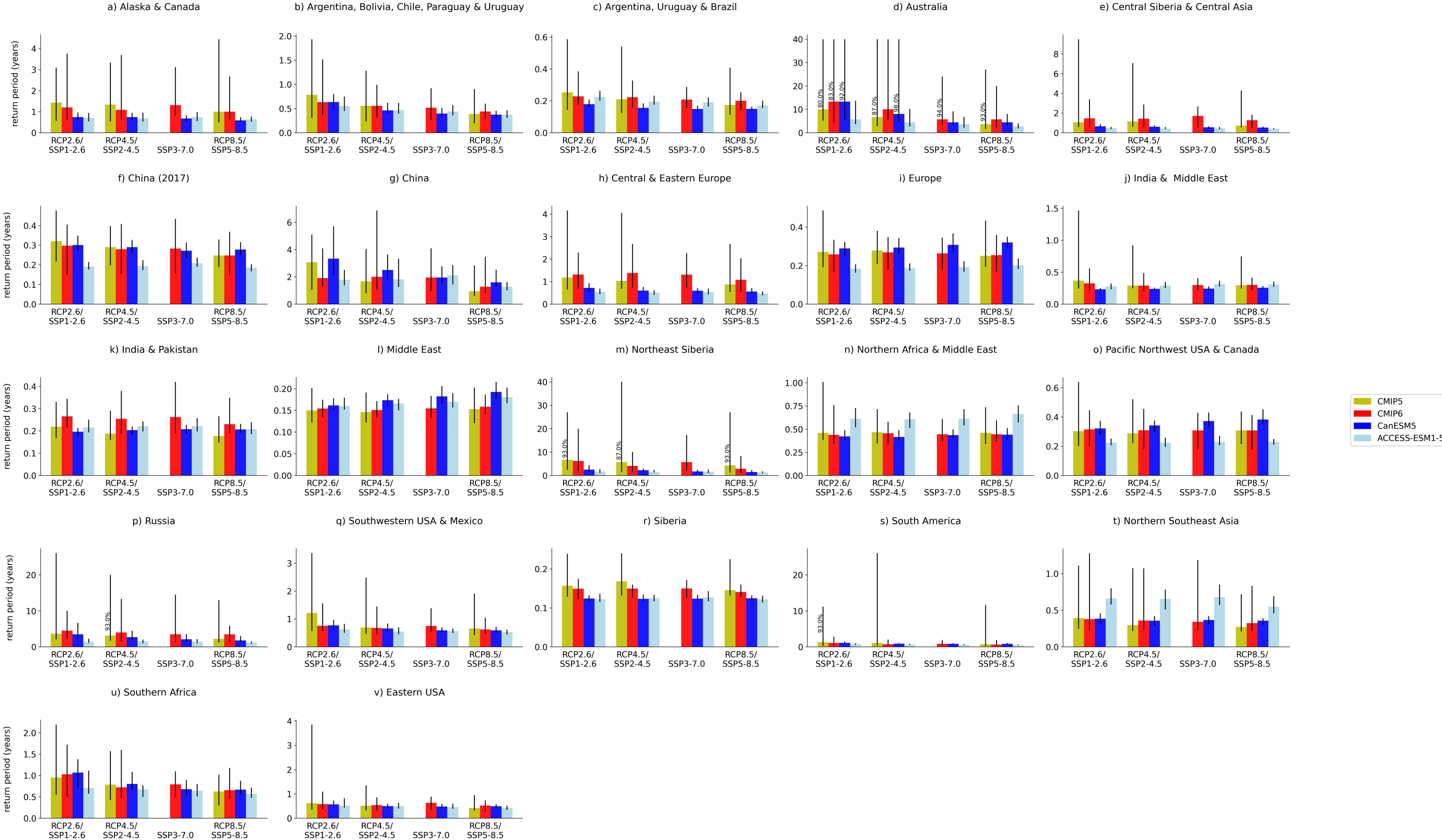
a) Alaska & Canada
b) Argentina, Bolivia, Chile, Paraguay & Uruguay
c) Argentina, Uruguay & Brazil
d) Australia
e) Central Siberia & Central Asia
f) China (2017)
g) China
h) Central & Eastern Europe
i) Europe
j) India & Middle East
k) India & Pakistan
l) Middle East
m) Northeast Siberia
n) Northern Africa & Middle East
o) Pacific Northwest USA & Canada
p) Russia
q) Southwestern USA & Mexico
r) Siberia
s) South America
t) Northern Southeast Asia
u) Southern Africa
v) Eastern USA
return period (years)
RCP2.6/
SSP1-2.6
RCP4.5/
SSP2-4.5
SSP3-7.0
RCP8.5/
SSP5-8.5
80.0%
83.0%
92.0%
87.0%
98.0%
94.0%
93.0%
CMIP5
CMIP6
CanESM5
ACCESS-ESM1-5

Supplementary Figure 327: Return times (RT) during present-to-near-future (2015-2054) based on Berkeley Earth (Tx90p) - Return times (RT) (in years) during the present-to-near-future period between 2015 and 2054 for Median Heatwave Severity of the respective observed event. For each region, values are shown for the different scenarios (increasing in emissions from left to right for the CMIP5 (green), CMIP6 (red), CanESM5 (dark blue), and ACCESS-ESM1-5 (light blue) ensembles. The error bars indicate the 5th and 95th percentile of the respective sample. Where the respective ensemble contains models not able to simulate the magnitude of the respective observed event's Median Heatwave Severity, the proportion of models that are able to simulate an event with a value of Median Heatwave Severity at least as high as the observed event is indicated by the percentage values above the individual bars. The observed reference values are calculated using data from Berkeley Earth.

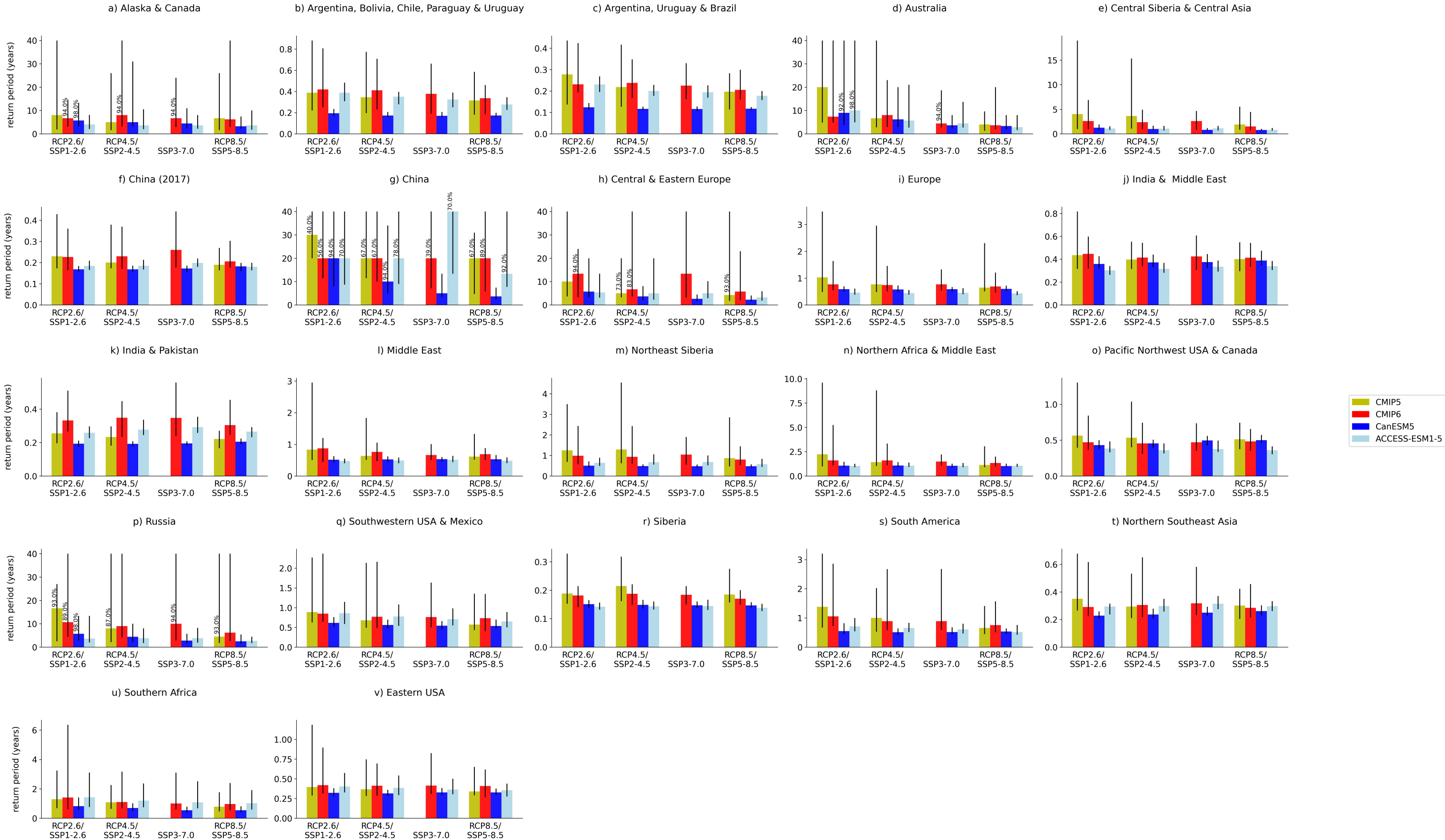
a) Alaska & Canada
b) Argentina, Bolivia, Chile, Paraguay & Uruguay
c) Argentina, Uruguay & Brazil
d) Australia
e) Central Siberia & Central Asia
f) China (2017)
g) China
h) Central & Eastern Europe
i) Europe
j) India & Middle East
k) India & Pakistan
l) Middle East
m) Northeast Siberia
n) Northern Africa & Middle East
o) Pacific Northwest USA & Canada
p) Russia
q) Southwestern USA & Mexico
r) Siberia
s) South America
t) Northern Southeast Asia
u) Southern Africa
v) Eastern USA
return period (years)
RCP2.6/
SSP1-2.6
RCP4.5/
SSP2-4.5
SSP3-7.0
RCP8.5/
SSP5-8.5
CMIP5
CMIP6
CanESM5
ACCESS-ESM1-5

Supplementary Figure 328: Return times (RT) during present-to-near-future (2015-2054) based on ERA5 (Tx90p) - Return times (RT) (in years) during the present-to-near-future period between 2015 and 2054 for Median Heatwave Severity of the respective observed event. For each region, values are shown for the different scenarios (increasing in emissions from left to right for the CMIP5 (green), CMIP6 (red), CanESM5 (dark blue), and ACCESS-ESM1-5 (light blue) ensembles. The error bars indicate the 5th and 95th percentile of the respective sample. Where the respective ensemble contains models not able to simulate the magnitude of the respective observed event's Median Heatwave Severity, the proportion of models that are able to simulate an event with a value of Median Heatwave Severity at least as high as the observed event is indicated by the percentage values above the individual bars. The observed reference values are calculated using data from ERA5.

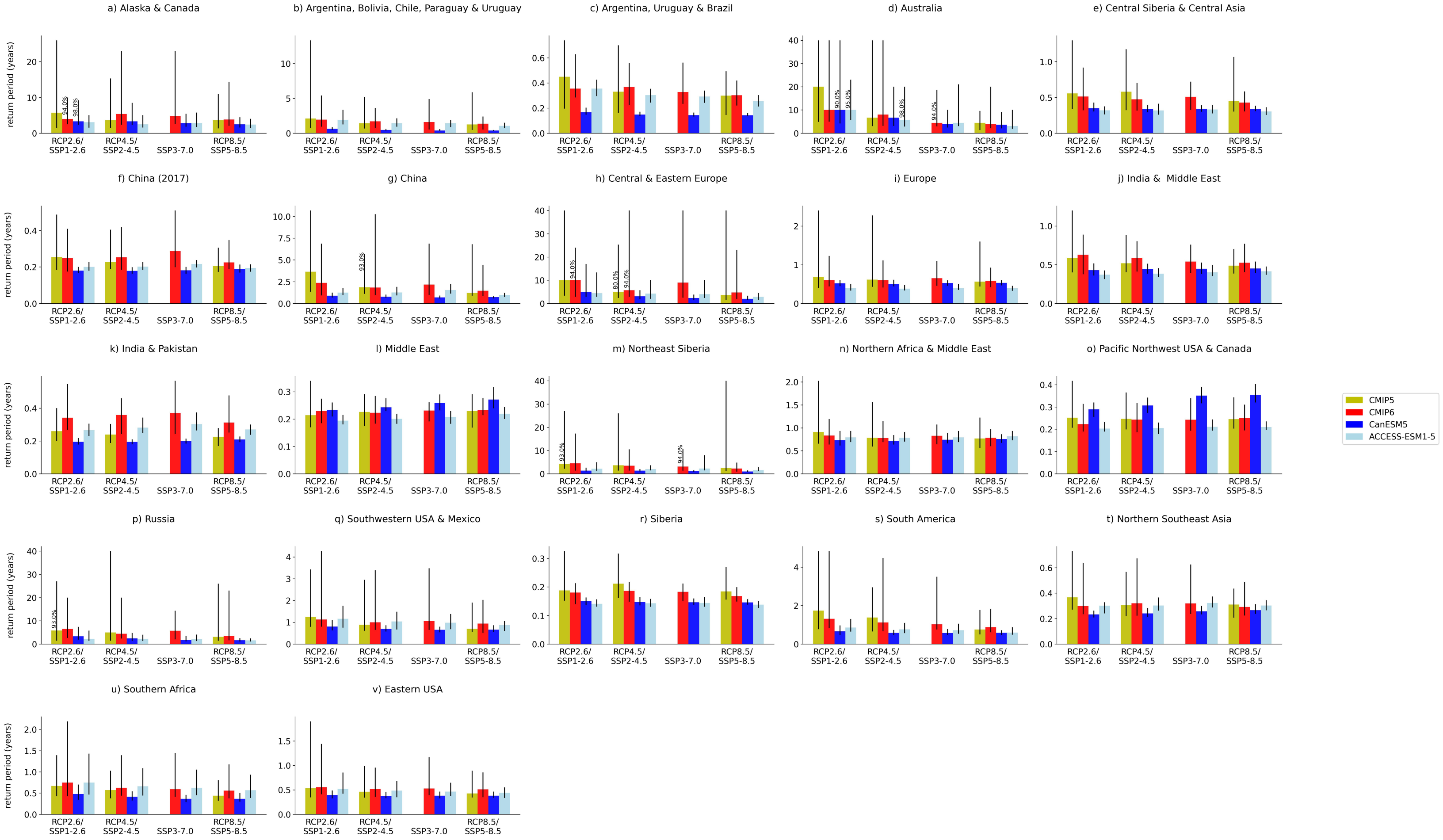

a) Alaska & Canada
b) Argentina, Bolivia, Chile, Paraguay & Uruguay
c) Argentina, Uruguay & Brazil
d) Australia
e) Central Siberia & Central Asia
f) China (2017)
g) China
h) Central & Eastern Europe
i) Europe
j) India & Middle East
k) India & Pakistan
l) Middle East
m) Northeast Siberia
n) Northern Africa & Middle East
o) Pacific Northwest USA & Canada
p) Russia
q) Southwestern USA & Mexico
r) Siberia
s) South America
t) Northern Southeast Asia
u) Southern Africa
v) Eastern USA
return period (years)
RCP2.6/
SSP1-2.6
RCP4.5/
SSP2-4.5
SSP3-7.0
RCP8.5/
SSP5-8.5
CMIP5
CMIP6
CanESM5
ACCESS-ESM1-5
94.0%
98.0%
90.0%
95.0%
93.0%
80.0%

Supplementary Figure 329: Return times (RT) during end-of-century (2060-2099) based on Berkeley Earth (Tx90p) - Return times (RT) (in years) during the end-of-century period between 2060 and 2099 for Cumulative Heat (°C) of the respective observed event. For each region, values are shown for the different scenarios (increasing in emissions from left to right for the CMIP5 (green), CMIP6 (red), CanESM5 (dark blue), and ACCESS-ESM1-5 (light blue) ensembles. The error bars indicate the 5th and 95th percentile of the respective sample. Where the respective ensemble contains models not able to simulate the magnitude of the respective observed event's Cumulative Heat (°C), the proportion of models that are able to simulate an event with a value of Cumulative Heat (°C) at least as high as the observed event is indicated by the percentage values above the individual bars. The observed reference values are calculated using data from Berkeley Earth.

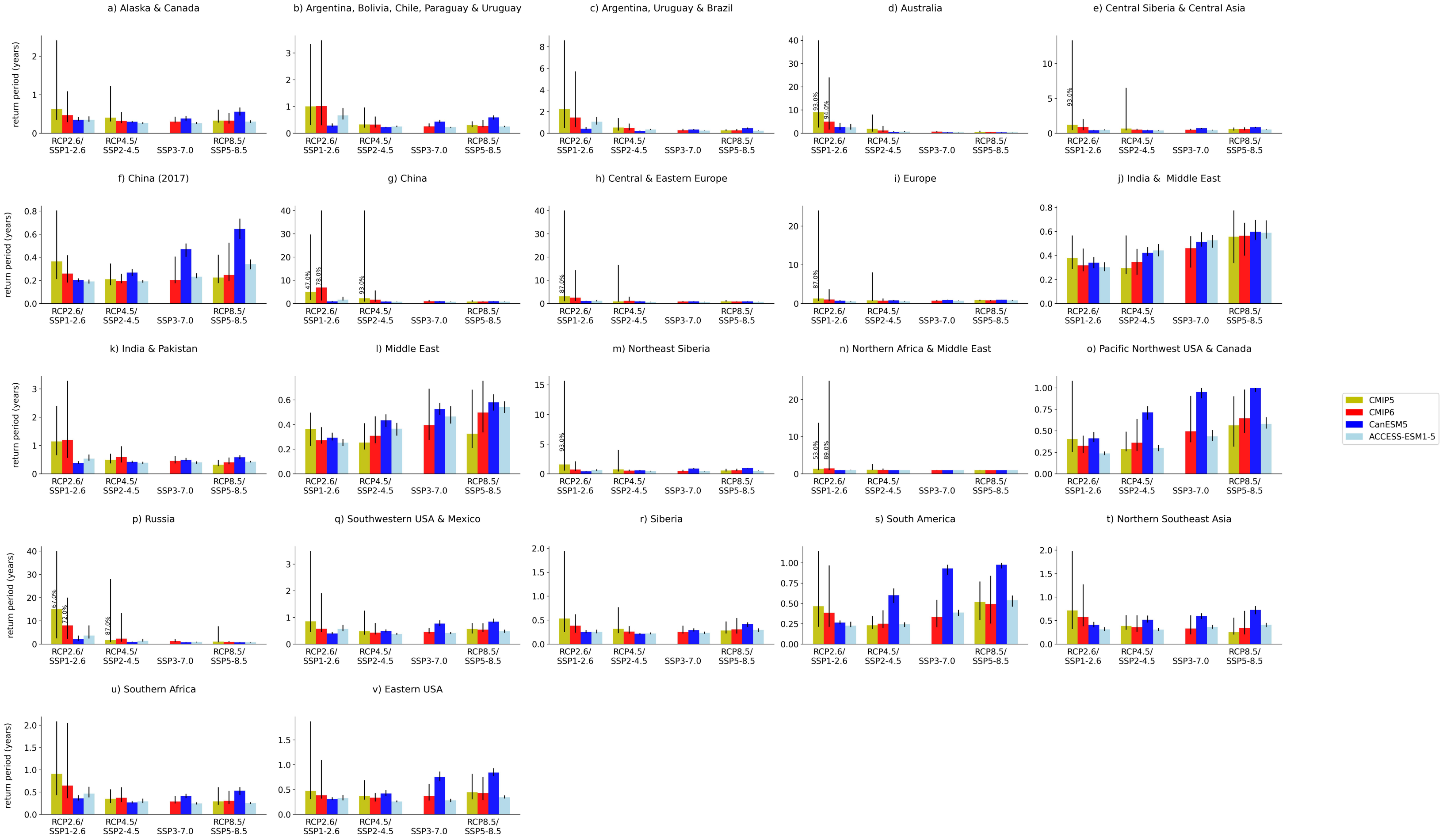
a) Alaska & Canada
b) Argentina, Bolivia, Chile, Paraguay & Uruguay
c) Argentina, Uruguay & Brazil
d) Australia
e) Central Siberia & Central Asia
f) China (2017)
g) China
h) Central & Eastern Europe
i) Europe
j) India & Middle East
k) India & Pakistan
l) Middle East
m) Northeast Siberia
n) Northern Africa & Middle East
o) Pacific Northwest USA & Canada
p) Russia
q) Southwestern USA & Mexico
r) Siberia
s) South America
t) Northern Southeast Asia
u) Southern Africa
v) Eastern USA
return period (years)
RCP2.6/
SSP1-2.6
RCP4.5/
SSP2-4.5
SSP3-7.0
RCP8.5/
SSP5-8.5
CMIP5
CMIP6
CanESM5
ACCESS-ESM1-5
93.0%
94.0%
47.0%
78.0%
87.0%
53.0%
89.0%
67.0%
72.0%

Supplementary Figure 330: Return times (RT) during end-of-century (2060-2099) based on ERA5 (Tx90p) - Return times (RT) (in years) during the end-of-century period between 2060 and 2099 for Cumulative Heat (°C) of the respective observed event. For each region, values are shown for the different scenarios (increasing in emissions from left to right for the CMIP5 (green), CMIP6 (red), CanESM5 (dark blue), and ACCESS-ESM1-5 (light blue) ensembles. The error bars indicate the 5th and 95th percentile of the respective sample. Where the respective ensemble contains models not able to simulate the magnitude of the respective observed event's Cumulative Heat (°C), the proportion of models that are able to simulate an event with a value of Cumulative Heat (°C) at least as high as the observed event is indicated by the percentage values above the individual bars. The observed reference values are calculated using data from ERA5.

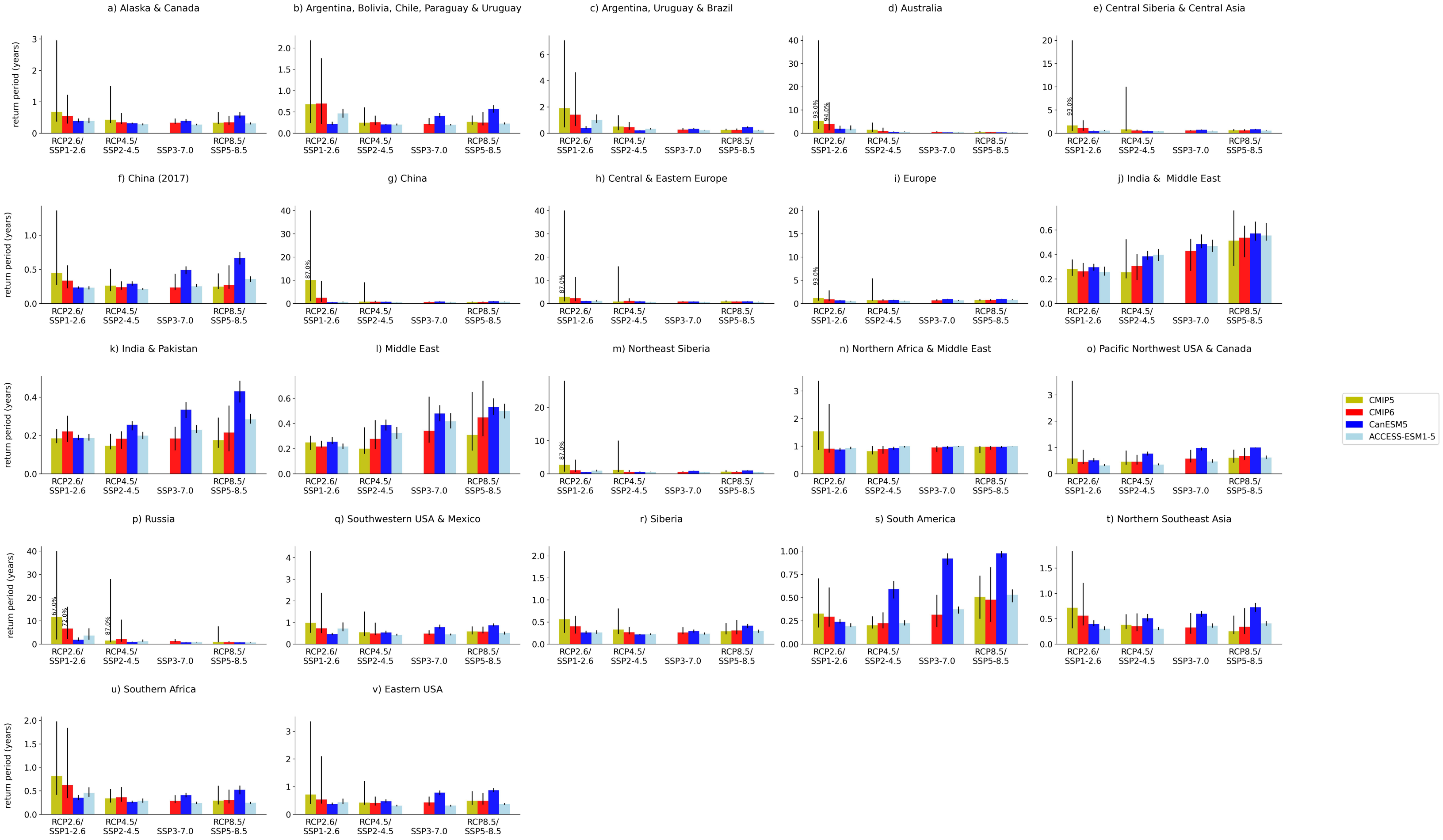
a) Alaska & Canada
b) Argentina, Bolivia, Chile, Paraguay & Uruguay
c) Argentina, Uruguay & Brazil
d) Australia
e) Central Siberia & Central Asia
f) China (2017)
g) China
h) Central & Eastern Europe
i) Europe
j) India & Middle East
k) India & Pakistan
l) Middle East
m) Northeast Siberia
n) Northern Africa & Middle East
o) Pacific Northwest USA & Canada
p) Russia
q) Southwestern USA & Mexico
r) Siberia
s) South America
t) Northern Southeast Asia
u) Southern Africa
v) Eastern USA
return period (years)
RCP2.6/ SSP1-2.6
RCP4.5/ SSP2-4.5
SSP3-7.0
RCP8.5/ SSP5-8.5
93.0%
94.0%
87.0%
67.0%
72.0%
CMIP5
CMIP6
CanESM5
ACCESS-ESM1-5

Supplementary Figure 331: Return times (RT) during end-of-century (2060-2099) based on Berkeley Earth (Tx90p) - Return times (RT) (in years) during the end-of-century period between 2060 and 2099 for Heatwave Length (days) of the respective observed event. For each region, values are shown for the different scenarios (increasing in emissions from left to right for the CMIP5 (green), CMIP6 (red), CanESM5 (dark blue), and ACCESS-ESM1-5 (light blue) ensembles. The error bars indicate the 5th and 95th percentile of the respective sample. Where the respective ensemble contains models not able to simulate the magnitude of the respective observed event's Heatwave Length (days), the proportion of models that are able to simulate an event with a value of Heatwave Length (days) at least as high as the observed event is indicated by the percentage values above the individual bars. The observed reference values are calculated using data from Berkeley Earth.

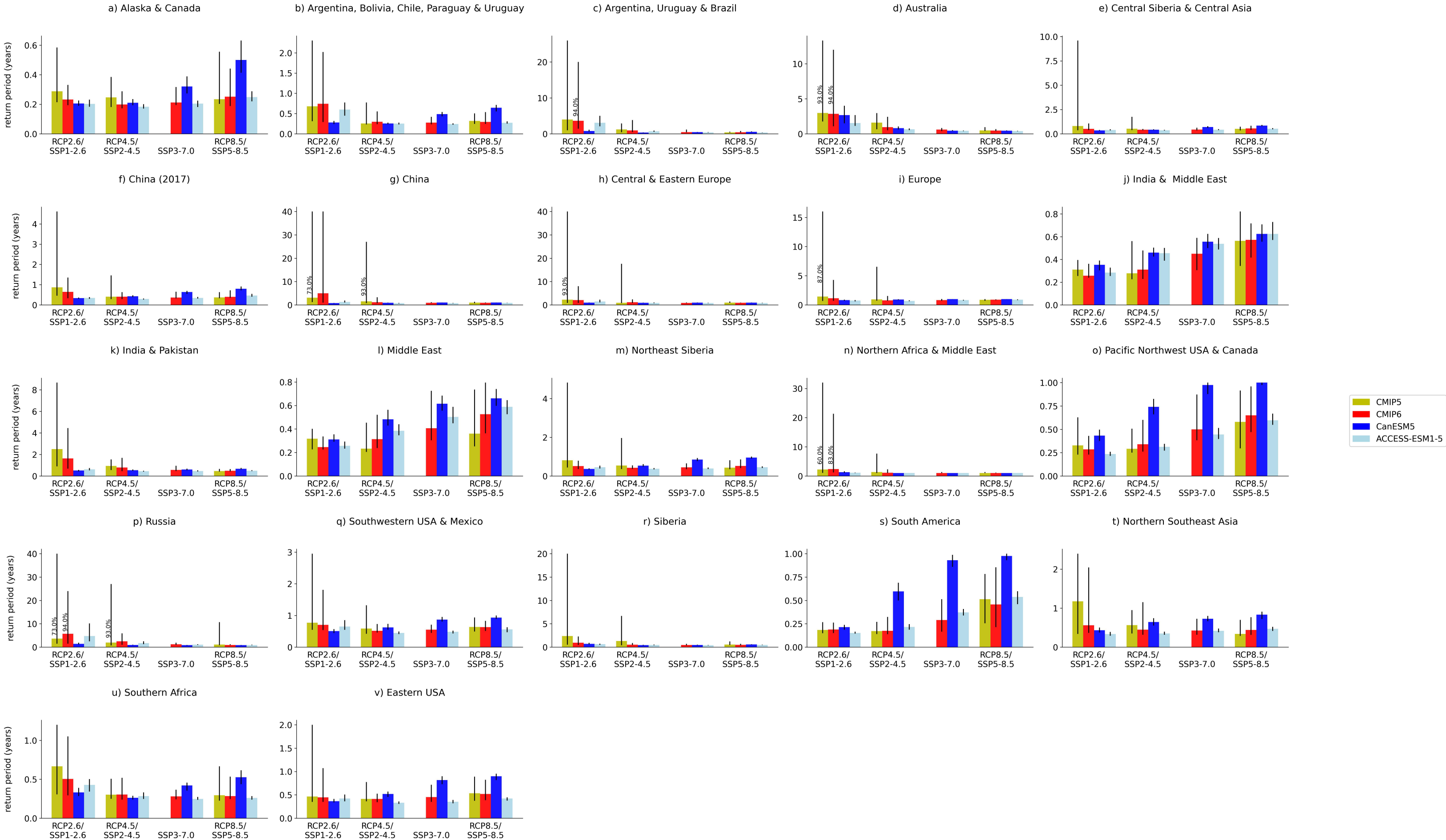

a) Alaska & Canada
b) Argentina, Bolivia, Chile, Paraguay & Uruguay
c) Argentina, Uruguay & Brazil
d) Australia
e) Central Siberia & Central Asia
f) China (2017)
g) China
h) Central & Eastern Europe
i) Europe
j) India & Middle East
k) India & Pakistan
l) Middle East
m) Northeast Siberia
n) Northern Africa & Middle East
o) Pacific Northwest USA & Canada
p) Russia
q) Southwestern USA & Mexico
r) Siberia
s) South America
t) Northern Southeast Asia
u) Southern Africa
v) Eastern USA
return period (years)
RCP2.6/ SSP1-2.6
RCP4.5/ SSP2-4.5
SSP3-7.0
RCP8.5/ SSP5-8.5
CMIP5
CMIP6
CanESM5
ACCESS-ESM1-5

Supplementary Figure 332: Return times (RT) during end-of-century (2060-2099) based on ERA5 (Tx90p) - Return times (RT) (in years) during the end-of-century period between 2060 and 2099 for Heatwave Length (days) of the respective observed event. For each region, values are shown for the different scenarios (increasing in emissions from left to right for the CMIP5 (green), CMIP6 (red), CanESM5 (dark blue), and ACCESS-ESM1-5 (light blue) ensembles. The error bars indicate the 5th and 95th percentile of the respective sample. Where the respective ensemble contains models not able to simulate the magnitude of the respective observed event's Heatwave Length (days), the proportion of models that are able to simulate an event with a value of Heatwave Length (days) at least as high as the observed event is indicated by the percentage values above the individual bars. The observed reference values are calculated using data from ERA5.

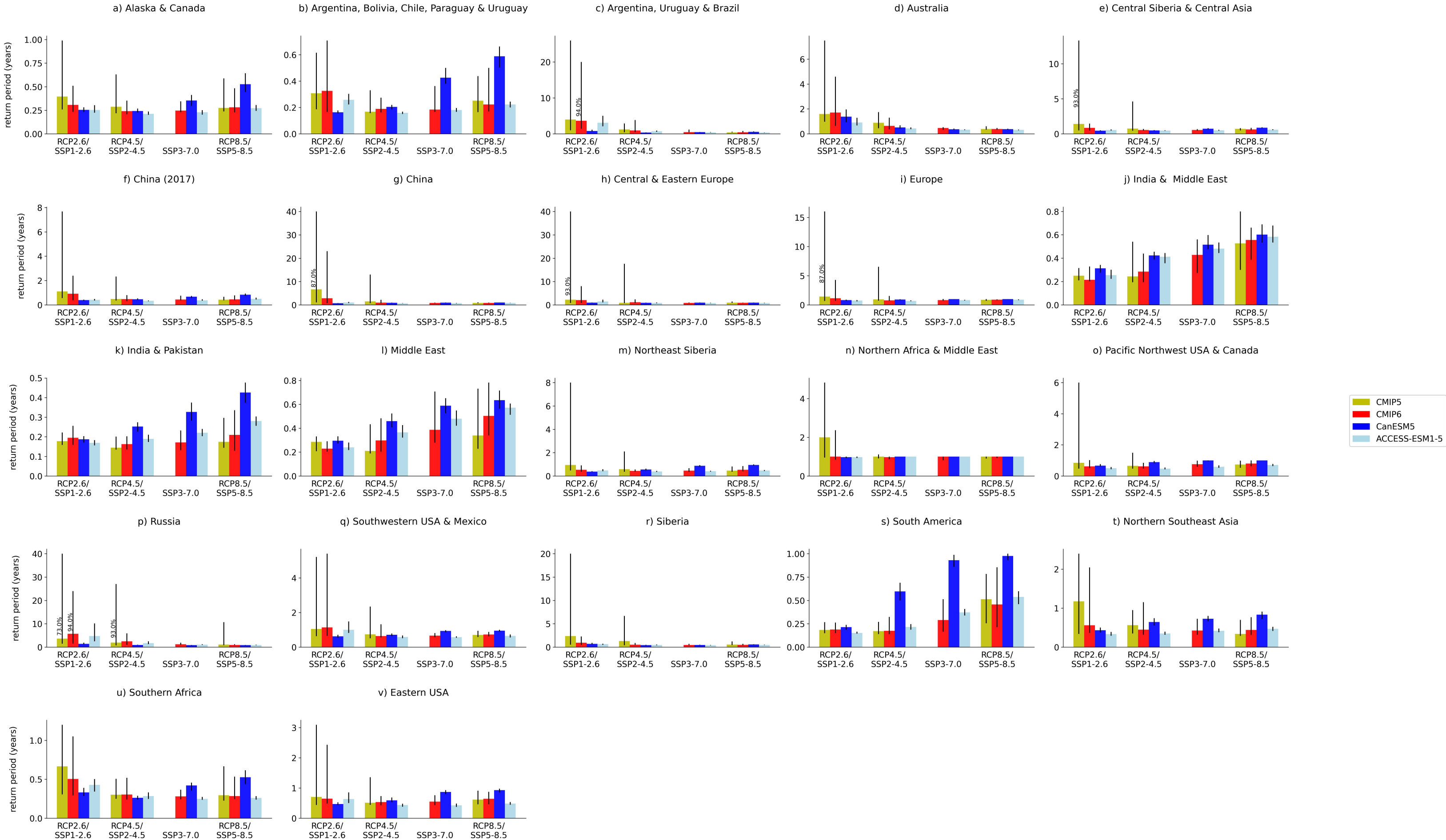

a) Alaska & Canada
b) Argentina, Bolivia, Chile, Paraguay & Uruguay
c) Argentina, Uruguay & Brazil
d) Australia
e) Central Siberia & Central Asia
f) China (2017)
g) China
h) Central & Eastern Europe
i) Europe
j) India & Middle East
k) India & Pakistan
l) Middle East
m) Northeast Siberia
n) Northern Africa & Middle East
o) Pacific Northwest USA & Canada
p) Russia
q) Southwestern USA & Mexico
r) Siberia
s) South America
t) Northern Southeast Asia
u) Southern Africa
v) Eastern USA
return period (years)
RCP2.6/
SSP1-2.6
RCP4.5/
SSP2-4.5
SSP3-7.0
RCP8.5/
SSP5-8.5
94.0%
93.0%
87.0%
73.0%
CMIP5
CMIP6
CanESM5
ACCESS-ESM1-5

Supplementary Figure 333: Return times (RT) during end-of-century (2060-2099) based on Berkeley Earth (Tx90p) - Return times (RT) (in years) during the end-of-century period between 2060 and 2099 for Maximum Heatwave Area (%) of the respective observed event. For each region, values are shown for the different scenarios (increasing in emissions from left to right for the CMIP5 (green), CMIP6 (red), CanESM5 (dark blue), and ACCESS-ESM1-5 (light blue) ensembles. The error bars indicate the 5th and 95th percentile of the respective sample. Where the respective ensemble contains models not able to simulate the magnitude of the respective observed event's Maximum Heatwave Area (%), the proportion of models that are able to simulate an event with a value of Maximum Heatwave Area (%) at least as high as the observed event is indicated by the percentage values above the individual bars. The observed reference values are calculated using data from Berkeley Earth.

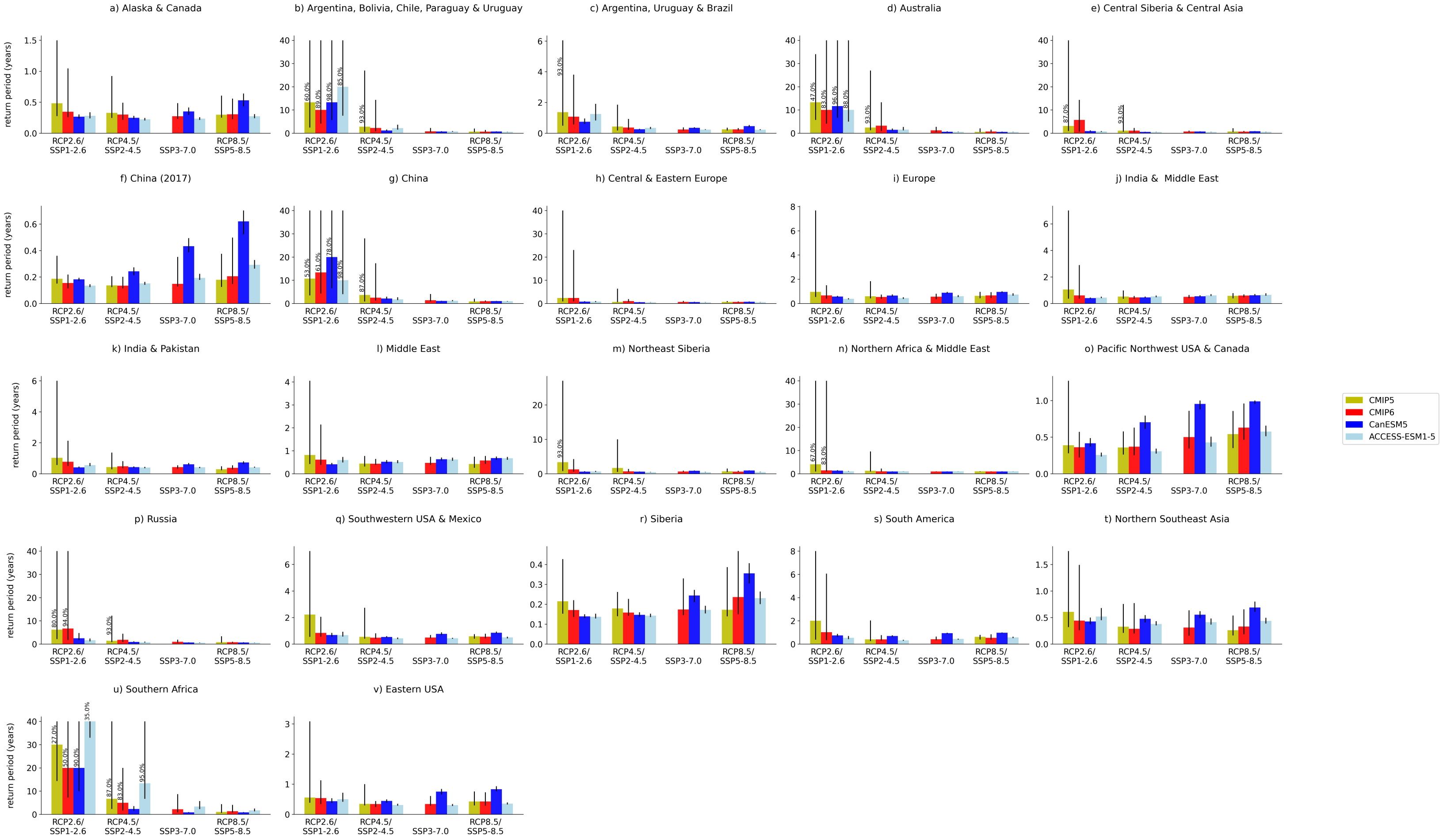
a) Alaska & Canada
b) Argentina, Bolivia, Chile, Paraguay & Uruguay
c) Argentina, Uruguay & Brazil
d) Australia
e) Central Siberia & Central Asia
f) China (2017)
g) China
h) Central & Eastern Europe
i) Europe
j) India & Middle East
k) India & Pakistan
l) Middle East
m) Northeast Siberia
n) Northern Africa & Middle East
o) Pacific Northwest USA & Canada
p) Russia
q) Southwestern USA & Mexico
r) Siberia
s) South America
t) Northern Southeast Asia
u) Southern Africa
v) Eastern USA
return period (years)
RCP2.6/ SSP1-2.6
RCP4.5/ SSP2-4.5
SSP3-7.0
RCP8.5/ SSP5-8.5
CMIP5
CMIP6
CanESM5
ACCESS-ESM1-5

Supplementary Figure 334: Return times (RT) during end-of-century (2060-2099) based on ERA5 (Tx90p) - Return times (RT) (in years) during the end-of-century period between 2060 and 2099 for Maximum Heatwave Area (%) of the respective observed event. For each region, values are shown for the different scenarios (increasing in emissions from left to right for the CMIP5 (green), CMIP6 (red), CanESM5 (dark blue), and ACCESS-ESM1-5 (light blue) ensembles. The error bars indicate the 5th and 95th percentile of the respective sample. Where the respective ensemble contains models not able to simulate the magnitude of the respective observed event's Maximum Heatwave Area (%), the proportion of models that are able to simulate an event with a value of Maximum Heatwave Area (%) at least as high as the observed event is indicated by the percentage values above the individual bars. The observed reference values are calculated using data from ERA5.

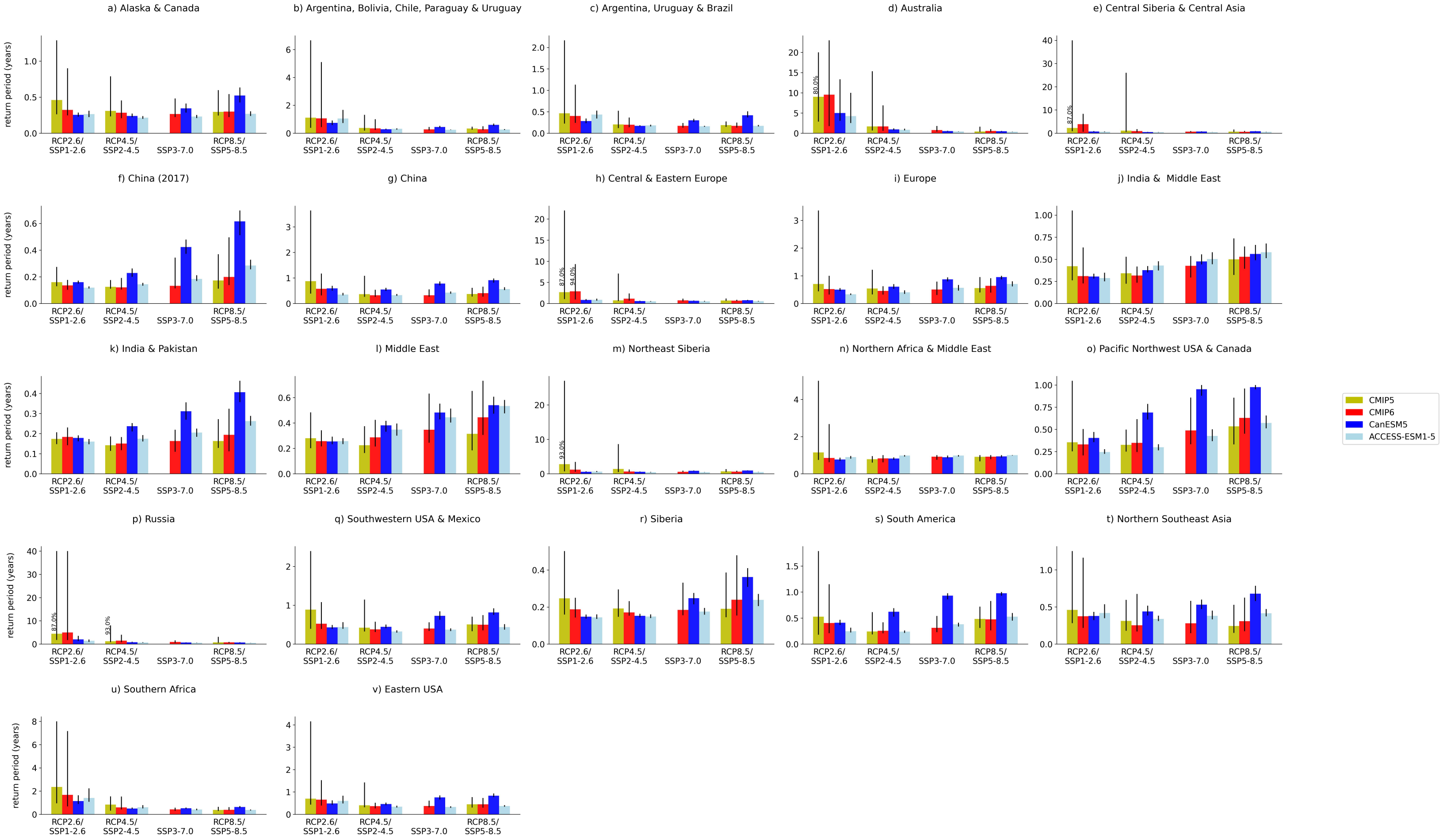

a) Alaska & Canada
b) Argentina, Bolivia, Chile, Paraguay & Uruguay
c) Argentina, Uruguay & Brazil
d) Australia
e) Central Siberia & Central Asia
f) China (2017)
g) China
h) Central & Eastern Europe
i) Europe
j) India & Middle East
k) India & Pakistan
l) Middle East
m) Northeast Siberia
n) Northern Africa & Middle East
o) Pacific Northwest USA & Canada
p) Russia
q) Southwestern USA & Mexico
r) Siberia
s) South America
t) Northern Southeast Asia
u) Southern Africa
v) Eastern USA
return period (years)
RCP2.6/ SSP1-2.6
RCP4.5/ SSP2-4.5
SSP3-7.0
RCP8.5/ SSP5-8.5
80.0%
87.0%
94.0%
93.0%
CMIP5
CMIP6
CanESM5
ACCESS-ESM1-5

Supplementary Figure 335: Return times (RT) during end-of-century (2060-2099) based on Berkeley Earth (Tx90p) - Return times (RT) (in years) during the end-of-century period between 2060 and 2099 for Maximum Heatwave Severity of the respective observed event. For each region, values are shown for the different scenarios (increasing in emissions from left to right for the CMIP5 (green), CMIP6 (red), CanESM5 (dark blue), and ACCESS-ESM1-5 (light blue) ensembles. The error bars indicate the 5th and 95th percentile of the respective sample. Where the respective ensemble contains models not able to simulate the magnitude of the respective observed event's Maximum Heatwave Severity, the proportion of models that are able to simulate an event with a value of Maximum Heatwave Severity at least as high as the observed event is indicated by the percentage values above the individual bars. The observed reference values are calculated using data from Berkeley Earth.

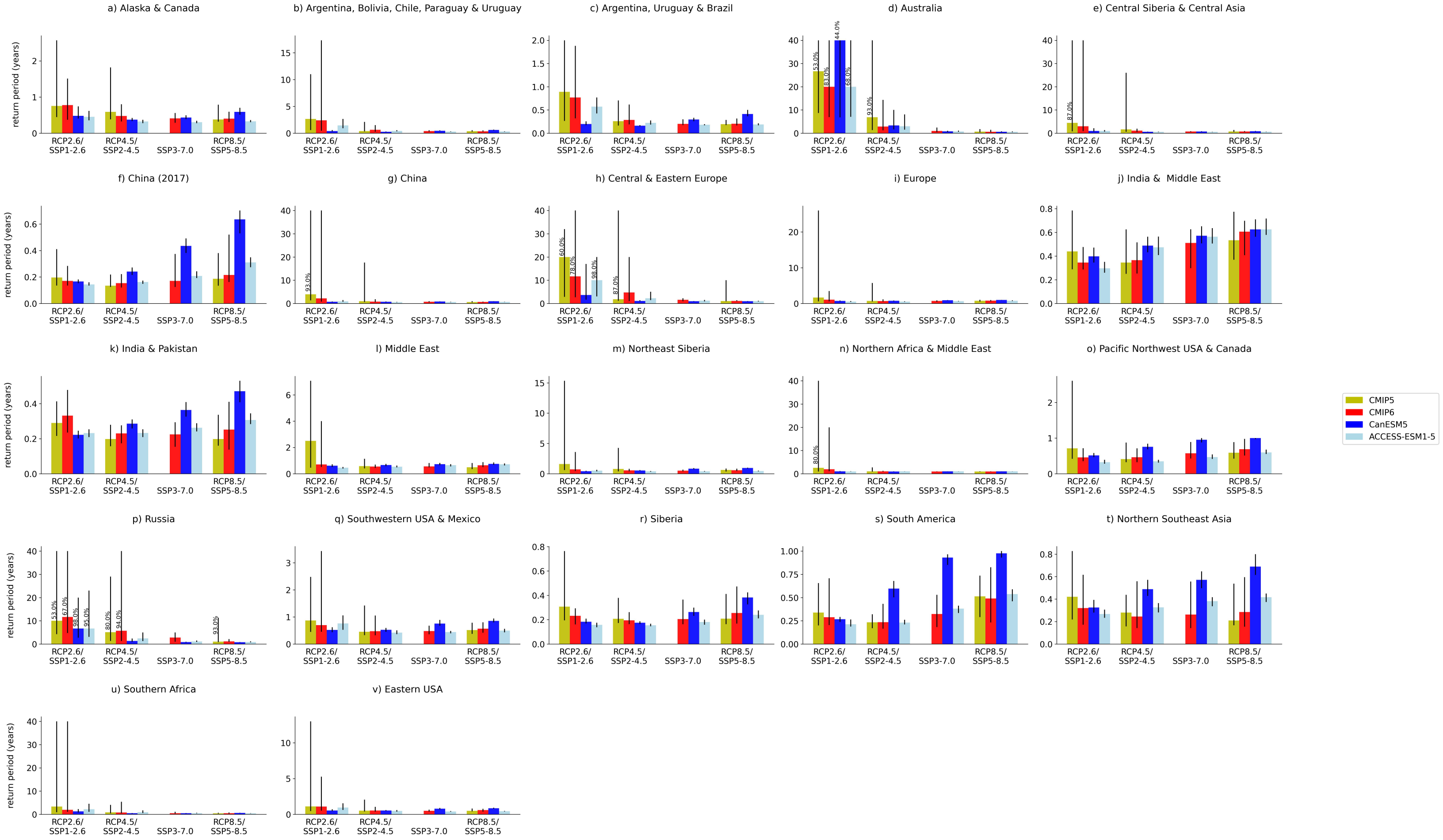

a) Alaska & Canada
b) Argentina, Bolivia, Chile, Paraguay & Uruguay
c) Argentina, Uruguay & Brazil
d) Australia
e) Central Siberia & Central Asia
f) China (2017)
g) China
h) Central & Eastern Europe
i) Europe
j) India & Middle East
k) India & Pakistan
l) Middle East
m) Northeast Siberia
n) Northern Africa & Middle East
o) Pacific Northwest USA & Canada
p) Russia
q) Southwestern USA & Mexico
r) Siberia
s) South America
t) Northern Southeast Asia
u) Southern Africa
v) Eastern USA
return period (years)
RCP2.6/
SSP1-2.6
RCP4.5/
SSP2-4.5
SSP3-7.0
RCP8.5/
SSP5-8.5
CMIP5
CMIP6
CanESM5
ACCESS-ESM1-5

Supplementary Figure 336: Return times (RT) during end-of-century (2060-2099) based on ERA5 (Tx90p) - Return times (RT) (in years) during the end-of-century period between 2060 and 2099 for Maximum Heatwave Severity of the respective observed event. For each region, values are shown for the different scenarios (increasing in emissions from left to right for the CMIP5 (green), CMIP6 (red), CanESM5 (dark blue), and ACCESS-ESM1-5 (light blue) ensembles. The error bars indicate the 5th and 95th percentile of the respective sample. Where the respective ensemble contains models not able to simulate the magnitude of the respective observed event's Maximum Heatwave Severity, the proportion of models that are able to simulate an event with a value of Maximum Heatwave Severity at least as high as the observed event is indicated by the percentage values above the individual bars. The observed reference values are calculated using data from ERA5.

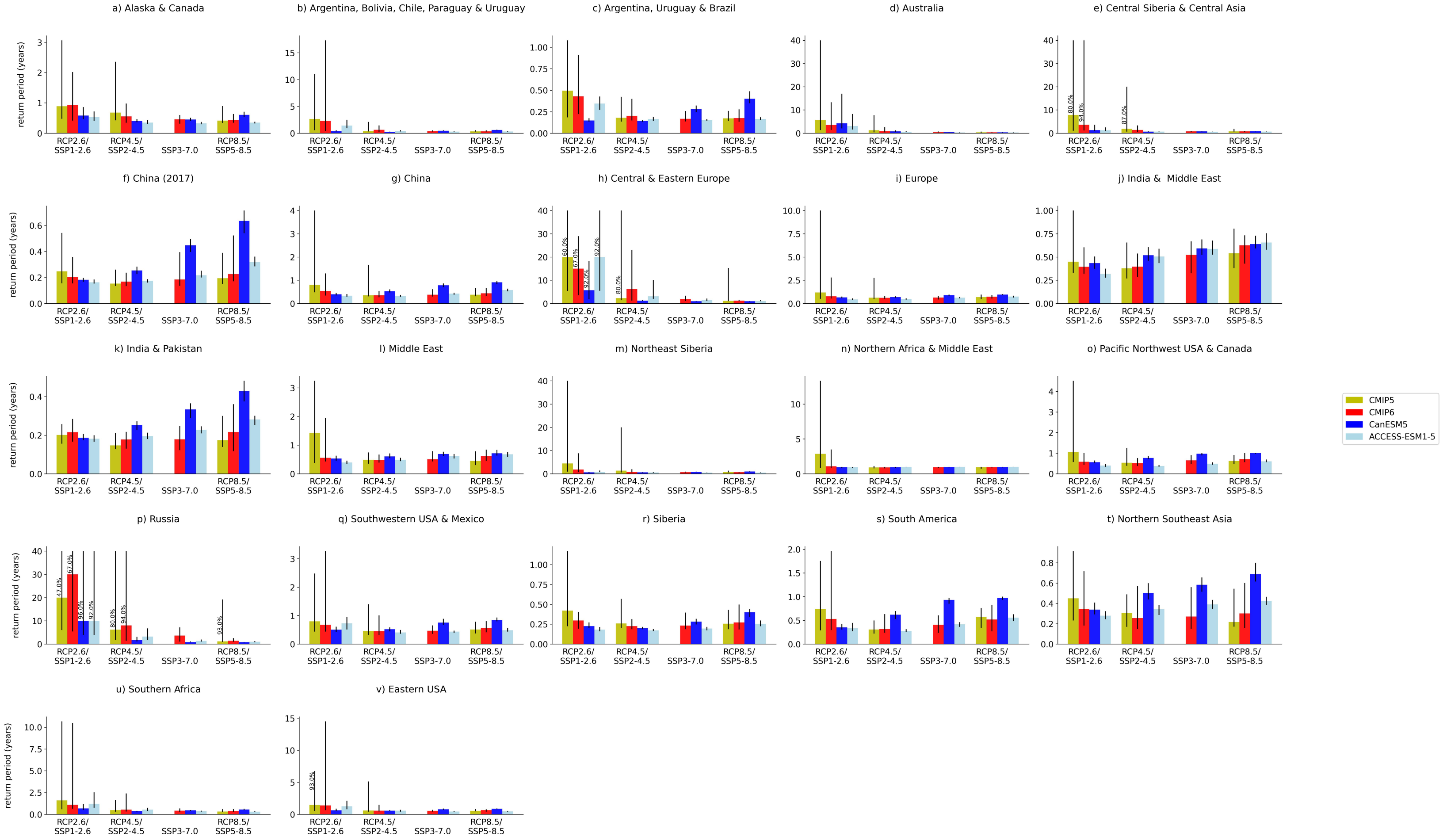
a) Alaska & Canada
b) Argentina, Bolivia, Chile, Paraguay & Uruguay
c) Argentina, Uruguay & Brazil
d) Australia
e) Central Siberia & Central Asia
f) China (2017)
g) China
h) Central & Eastern Europe
i) Europe
j) India & Middle East
k) India & Pakistan
l) Middle East
m) Northeast Siberia
n) Northern Africa & Middle East
o) Pacific Northwest USA & Canada
p) Russia
q) Southwestern USA & Mexico
r) Siberia
s) South America
t) Northern Southeast Asia
u) Southern Africa
v) Eastern USA
return period (years)
RCP2.6/ SSP1-2.6
RCP4.5/ SSP2-4.5
SSP3-7.0
RCP8.5/ SSP5-8.5
CMIP5
CMIP6
CanESM5
ACCESS-ESM1-5

Supplementary Figure 337: Return times (RT) during end-of-century (2060-2099) based on Berkeley Earth (Tx90p) - Return times (RT) (in years) during the end-of-century period between 2060 and 2099 for Median Heatwave Area (%) of the respective observed event. For each region, values are shown for the different scenarios (increasing in emissions from left to right for the CMIP5 (green), CMIP6 (red), CanESM5 (dark blue), and ACCESS-ESM1-5 (light blue) ensembles. The error bars indicate the 5th and 95th percentile of the respective sample. Where the respective ensemble contains models not able to simulate the magnitude of the respective observed event's Median Heatwave Area (%), the proportion of models that are able to simulate an event with a value of Median Heatwave Area (%) at least as high as the observed event is indicated by the percentage values above the individual bars. The observed reference values are calculated using data from Berkeley Earth.

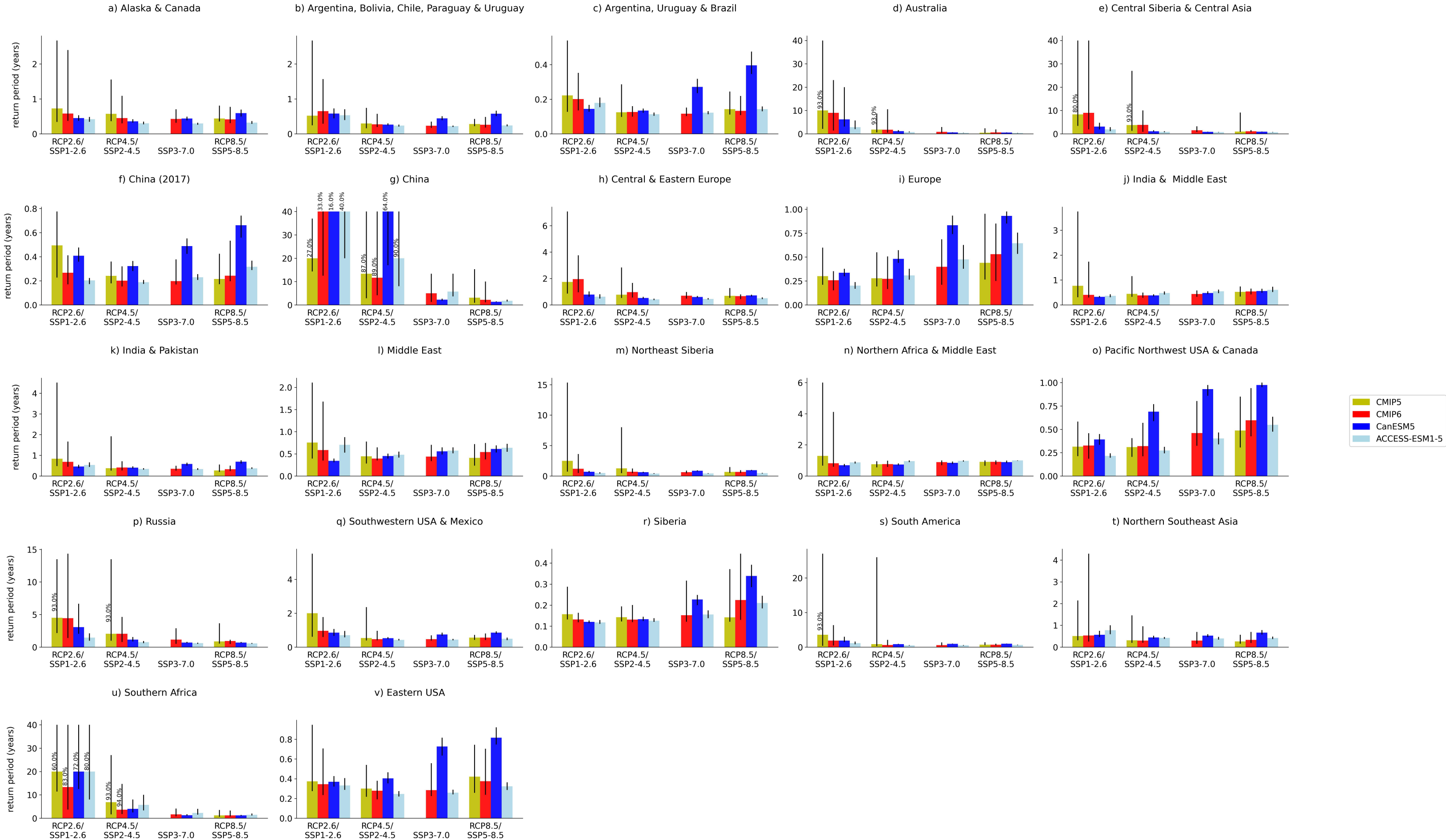
a) Alaska & Canada
b) Argentina, Bolivia, Chile, Paraguay & Uruguay
c) Argentina, Uruguay & Brazil
d) Australia
e) Central Siberia & Central Asia
f) China (2017)
g) China
h) Central & Eastern Europe
i) Europe
j) India & Middle East
k) India & Pakistan
l) Middle East
m) Northeast Siberia
n) Northern Africa & Middle East
o) Pacific Northwest USA & Canada
p) Russia
q) Southwestern USA & Mexico
r) Siberia
s) South America
t) Northern Southeast Asia
u) Southern Africa
v) Eastern USA
return period (years)
RCP2.6/ SSP1-2.6
RCP4.5/ SSP2-4.5
SSP3-7.0
RCP8.5/ SSP5-8.5
CMIP5
CMIP6
CanESM5
ACCESS-ESM1-5

Supplementary Figure 338: Return times (RT) during end-of-century (2060-2099) based on ERA5 (Tx90p) - Return times (RT) (in years) during the end-of-century period between 2060 and 2099 for Median Heatwave Area (%) of the respective observed event. For each region, values are shown for the different scenarios (increasing in emissions from left to right for the CMIP5 (green), CMIP6 (red), CanESM5 (dark blue), and ACCESS-ESM1-5 (light blue) ensembles. The error bars indicate the 5th and 95th percentile of the respective sample. Where the respective ensemble contains models not able to simulate the magnitude of the respective observed event's Median Heatwave Area (%), the proportion of models that are able to simulate an event with a value of Median Heatwave Area (%) at least as high as the observed event is indicated by the percentage values above the individual bars. The observed reference values are calculated using data from ERA5.

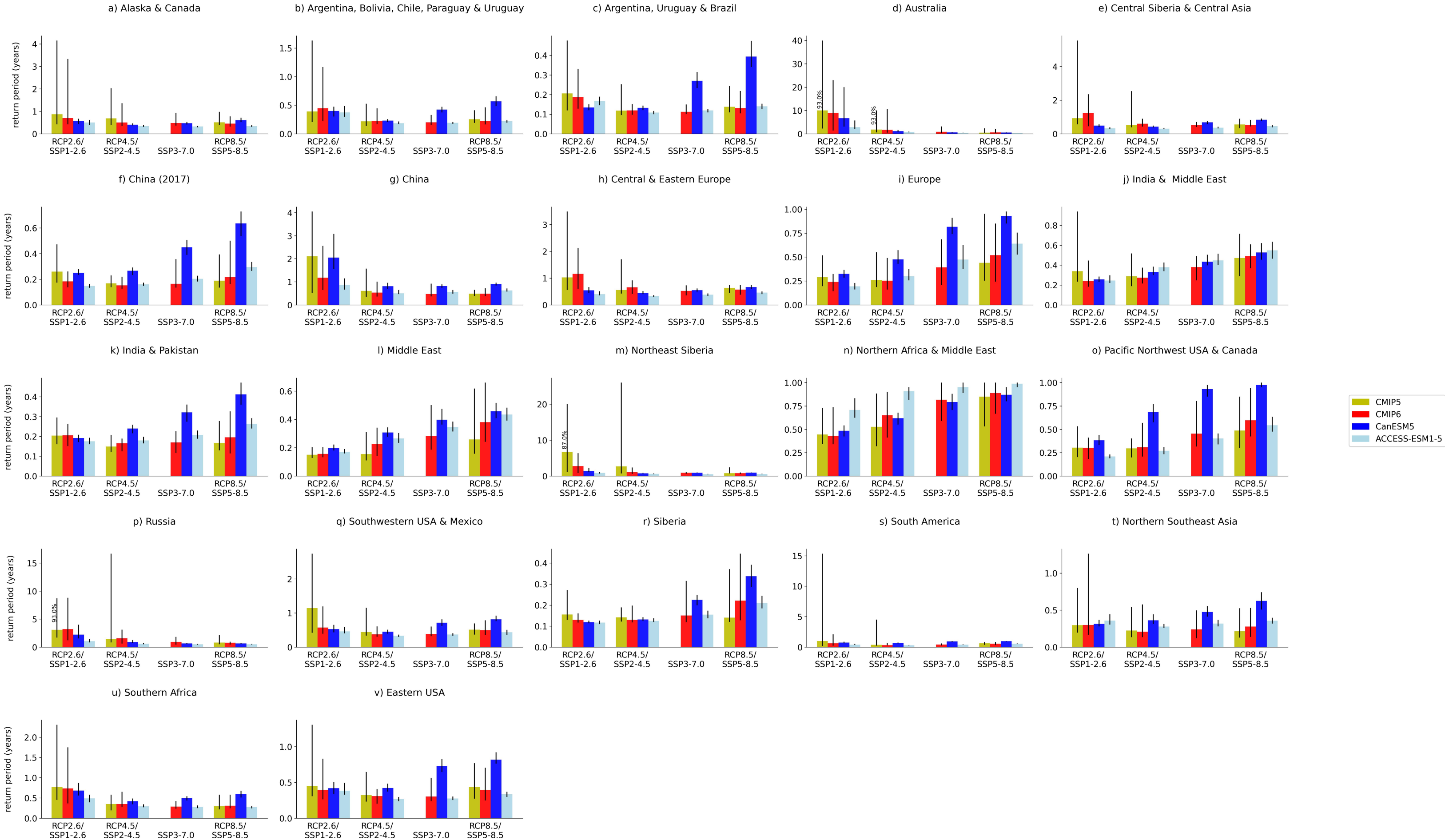
a) Alaska & Canada
b) Argentina, Bolivia, Chile, Paraguay & Uruguay
c) Argentina, Uruguay & Brazil
d) Australia
e) Central Siberia & Central Asia
f) China (2017)
g) China
h) Central & Eastern Europe
i) Europe
j) India & Middle East
k) India & Pakistan
l) Middle East
m) Northeast Siberia
n) Northern Africa & Middle East
o) Pacific Northwest USA & Canada
p) Russia
q) Southwestern USA & Mexico
r) Siberia
s) South America
t) Northern Southeast Asia
u) Southern Africa
v) Eastern USA
return period (years)
RCP2.6/ SSP1-2.6
RCP4.5/ SSP2-4.5
SSP3-7.0
RCP8.5/ SSP5-8.5
93.0%
87.0%
CMIP5
CMIP6
CanESM5
ACCESS-ESM1-5

Supplementary Figure 339: Return times (RT) during end-of-century (2060-2099) based on Berkeley Earth (Tx90p) - Return times (RT) (in years) during the end-of-century period between 2060 and 2099 for Median Heatwave Severity of the respective observed event. For each region, values are shown for the different scenarios (increasing in emissions from left to right for the CMIP5 (green), CMIP6 (red), CanESM5 (dark blue), and ACCESS-ESM1-5 (light blue) ensembles. The error bars indicate the 5th and 95th percentile of the respective sample. Where the respective ensemble contains models not able to simulate the magnitude of the respective observed event's Median Heatwave Severity, the proportion of models that are able to simulate an event with a value of Median Heatwave Severity at least as high as the observed event is indicated by the percentage values above the individual bars. The observed reference values are calculated using data from Berkeley Earth.

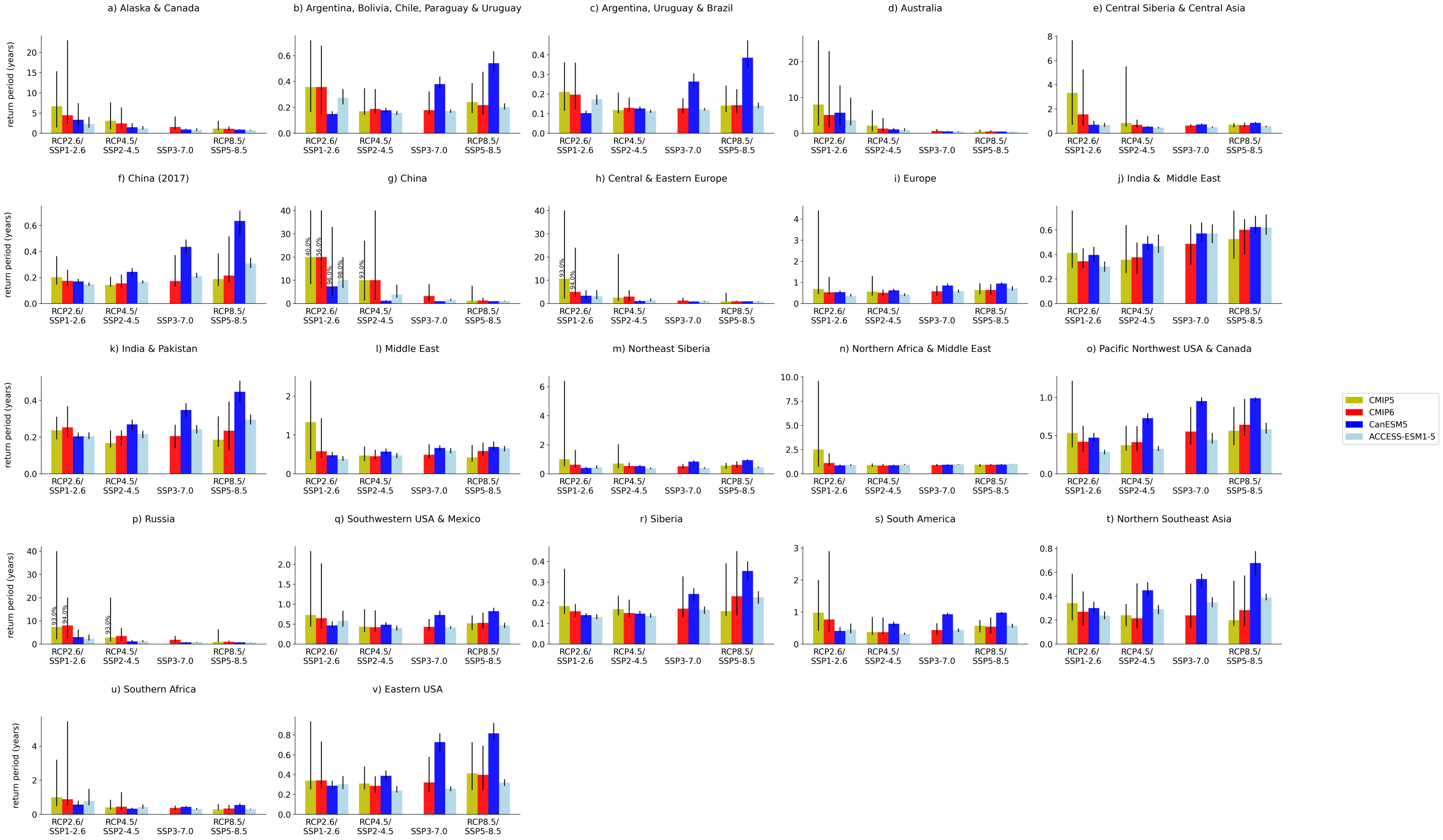

a) Alaska & Canada
b) Argentina, Bolivia, Chile, Paraguay & Uruguay
c) Argentina, Uruguay & Brazil
d) Australia
e) Central Siberia & Central Asia
f) China (2017)
g) China
h) Central & Eastern Europe
i) Europe
j) India & Middle East
k) India & Pakistan
l) Middle East
m) Northeast Siberia
n) Northern Africa & Middle East
o) Pacific Northwest USA & Canada
p) Russia
q) Southwestern USA & Mexico
r) Siberia
s) South America
t) Northern Southeast Asia
u) Southern Africa
v) Eastern USA
return period (years)
RCP2.6/
SSP1-2.6
RCP4.5/
SSP2-4.5
SSP3-7.0
RCP8.5/
SSP5-8.5
40.0%
56.0%
96.0%
98.0%
93.0%
94.0%
CMIP5
CMIP6
CanESM5
ACCESS-ESM1-5

Supplementary Figure 340: Return times (RT) during end-of-century (2060-2099) based on ERA5 (Tx90p) - Return times (RT) (in years) during the end-of-century period between 2060 and 2099 for Median Heatwave Severity of the respective observed event. For each region, values are shown for the different scenarios (increasing in emissions from left to right for the CMIP5 (green), CMIP6 (red), CanESM5 (dark blue), and ACCESS-ESM1-5 (light blue) ensembles. The error bars indicate the 5th and 95th percentile of the respective sample. Where the respective ensemble contains models not able to simulate the magnitude of the respective observed event's Median Heatwave Severity, the proportion of models that are able to simulate an event with a value of Median Heatwave Severity at least as high as the observed event is indicated by the percentage values above the individual bars. The observed reference values are calculated using data from ERA5.

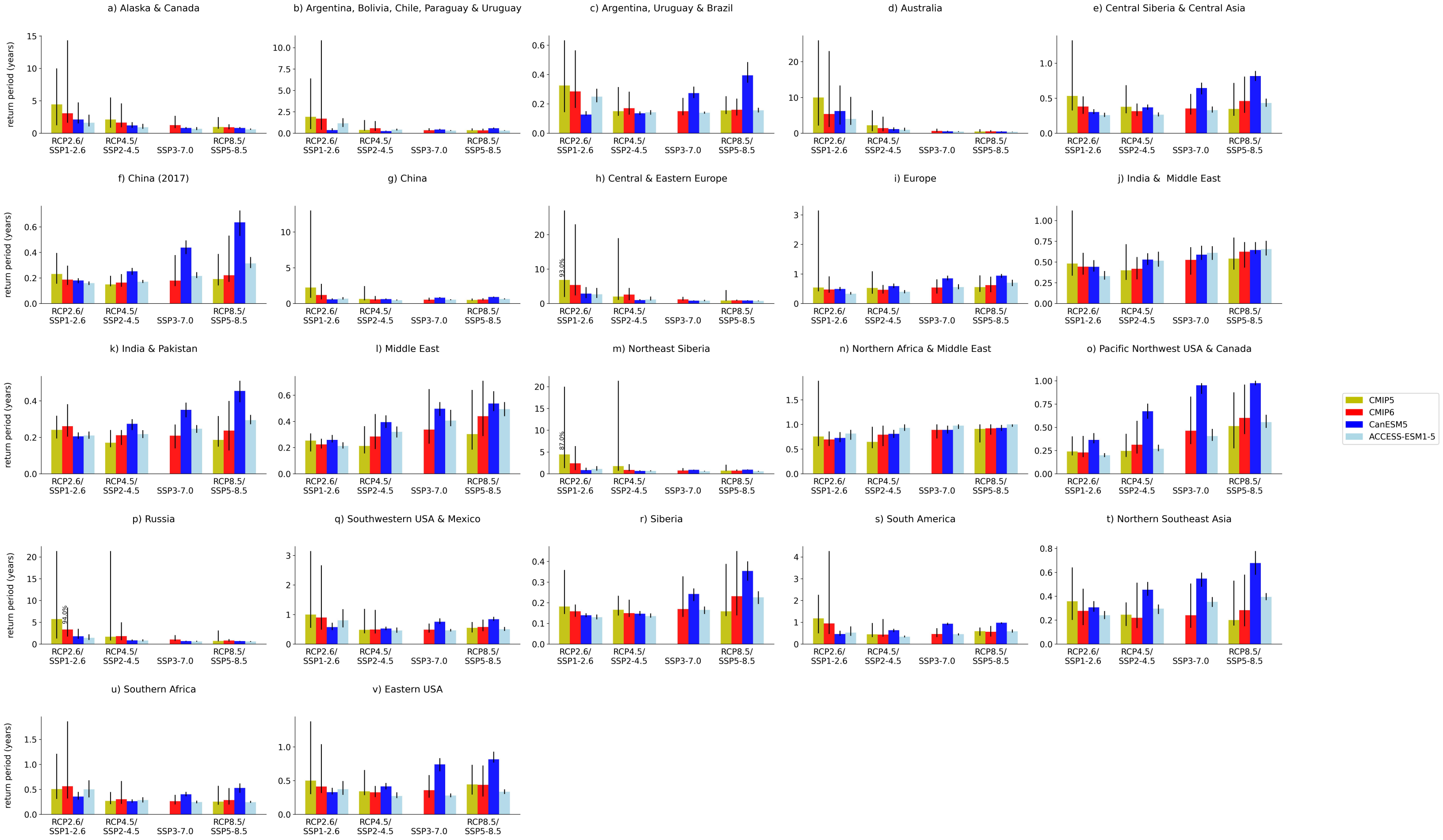
a) Alaska & Canada
b) Argentina, Bolivia, Chile, Paraguay & Uruguay
c) Argentina, Uruguay & Brazil
d) Australia
e) Central Siberia & Central Asia
f) China (2017)
g) China
h) Central & Eastern Europe
i) Europe
j) India & Middle East
k) India & Pakistan
l) Middle East
m) Northeast Siberia
n) Northern Africa & Middle East
o) Pacific Northwest USA & Canada
p) Russia
q) Southwestern USA & Mexico
r) Siberia
s) South America
t) Northern Southeast Asia
u) Southern Africa
v) Eastern USA
return period (years)
RCP2.6/ SSP1-2.6
RCP4.5/ SSP2-4.5
SSP3-7.0
RCP8.5/ SSP5-8.5
93.0%
87.0%
94.0%
CMIP5
CMIP6
CanESM5
ACCESS-ESM1-5

Supplementary Figure 341: Return times (RT) during historical (1951-2005) based on Berkeley Earth (Tx95.0p) - Return times (RT) (in years) during the historical period between 1951 and 2005 for Cumulative Heat (°C) of the respective observed event. For each region, values are shown for the different scenarios (increasing in emissions from left to right for the CMIP5 (green), CMIP6 (red), CanESM5 (dark blue), and ACCESS-ESM1-5 (light blue) ensembles. The error bars indicate the 5th and 95th percentile of the respective sample. Where the respective ensemble contains models not able to simulate the magnitude of the respective observed event's Cumulative Heat (°C), the proportion of models that are able to simulate an event with a value of Cumulative Heat (°C) at least as high as the observed event is indicated by the percentage values above the individual bars. The observed reference values are calculated using data from Berkeley Earth.

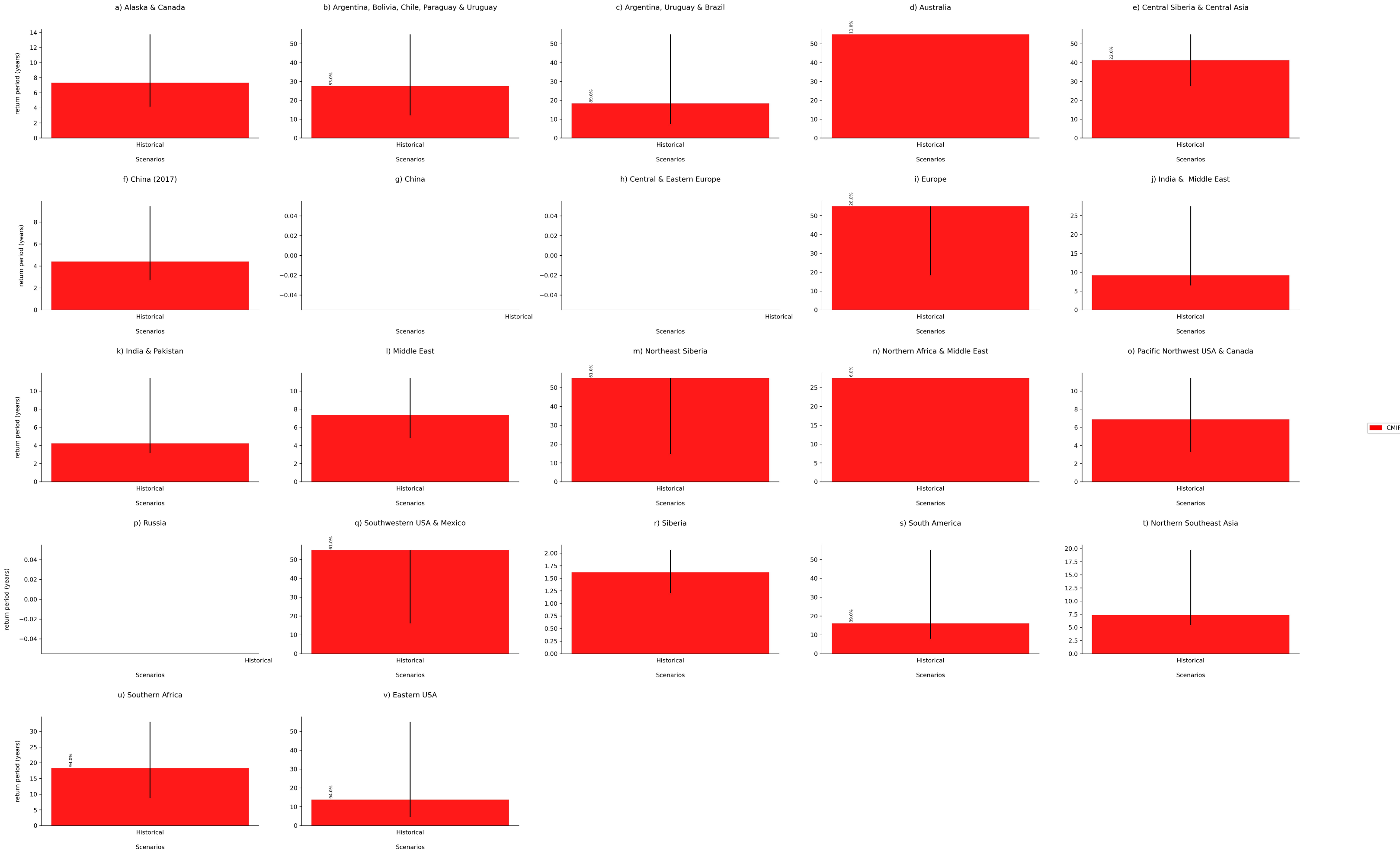
a) Alaska & Canada
b) Argentina, Bolivia, Chile, Paraguay & Uruguay
c) Argentina, Uruguay & Brazil
d) Australia
e) Central Siberia & Central Asia
f) China (2017)
g) China
h) Central & Eastern Europe
i) Europe
j) India & Middle East
k) India & Pakistan
l) Middle East
m) Northeast Siberia
n) Northern Africa & Middle East
o) Pacific Northwest USA & Canada
p) Russia
q) Southwestern USA & Mexico
r) Siberia
s) South America
t) Northern Southeast Asia
u) Southern Africa
v) Eastern USA
return period (years)
Historical
Scenarios
83.0%
89.0%
11.0%
22.0%
28.0%
61.0%
6.0%
61.0%
89.0%
94.0%
94.0%
CMIP6

Supplementary Figure 342: Return times (RT) during historical (1951-2005) based on Berkeley Earth (Tx97.5p) - Return times (RT) (in years) during the historical period between 1951 and 2005 for Cumulative Heat (°C) of the respective observed event. For each region, values are shown for the different scenarios (increasing in emissions from left to right for the CMIP5 (green), CMIP6 (red), CanESM5 (dark blue), and ACCESS-ESM1-5 (light blue) ensembles. The error bars indicate the 5th and 95th percentile of the respective sample. Where the respective ensemble contains models not able to simulate the magnitude of the respective observed event's Cumulative Heat (°C), the proportion of models that are able to simulate an event with a value of Cumulative Heat (°C) at least as high as the observed event is indicated by the percentage values above the individual bars. The observed reference values are calculated using data from Berkeley Earth.

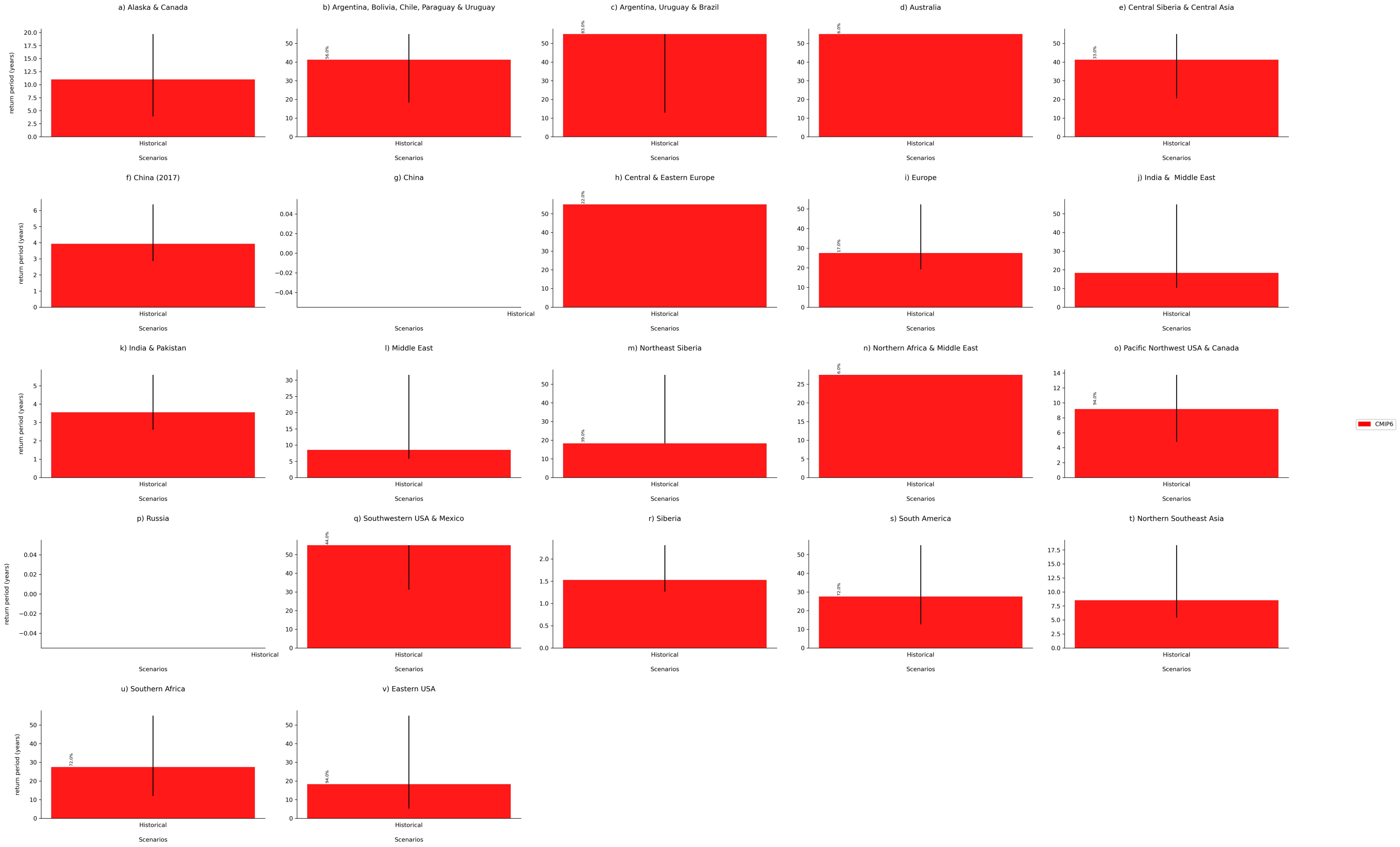
a) Alaska & Canada
b) Argentina, Bolivia, Chile, Paraguay & Uruguay
c) Argentina, Uruguay & Brazil
d) Australia
e) Central Siberia & Central Asia
f) China (2017)
g) China
h) Central & Eastern Europe
i) Europe
j) India & Middle East
k) India & Pakistan
l) Middle East
m) Northeast Siberia
n) Northern Africa & Middle East
o) Pacific Northwest USA & Canada
p) Russia
q) Southwestern USA & Mexico
r) Siberia
s) South America
t) Northern Southeast Asia
u) Southern Africa
v) Eastern USA
return period (years)
Historical
Scenarios
CMIP6
56.0%
83.0%
6.0%
33.0%
22.0%
17.0%
39.0%
6.0%
94.0%
44.0%
72.0%
72.0%
94.0%

Supplementary Figure 343: Return times (RT) during historical (1951-2005) based on Berkeley Earth (Tx99.0p) - Return times (RT) (in years) during the historical period between 1951 and 2005 for Cumulative Heat (°C) of the respective observed event. For each region, values are shown for the different scenarios (increasing in emissions from left to right for the CMIP5 (green), CMIP6 (red), CanESM5 (dark blue), and ACCESS-ESM1-5 (light blue) ensembles. The error bars indicate the 5th and 95th percentile of the respective sample. Where the respective ensemble contains models not able to simulate the magnitude of the respective observed event's Cumulative Heat (°C), the proportion of models that are able to simulate an event with a value of Cumulative Heat (°C) at least as high as the observed event is indicated by the percentage values above the individual bars. The observed reference values are calculated using data from Berkeley Earth.

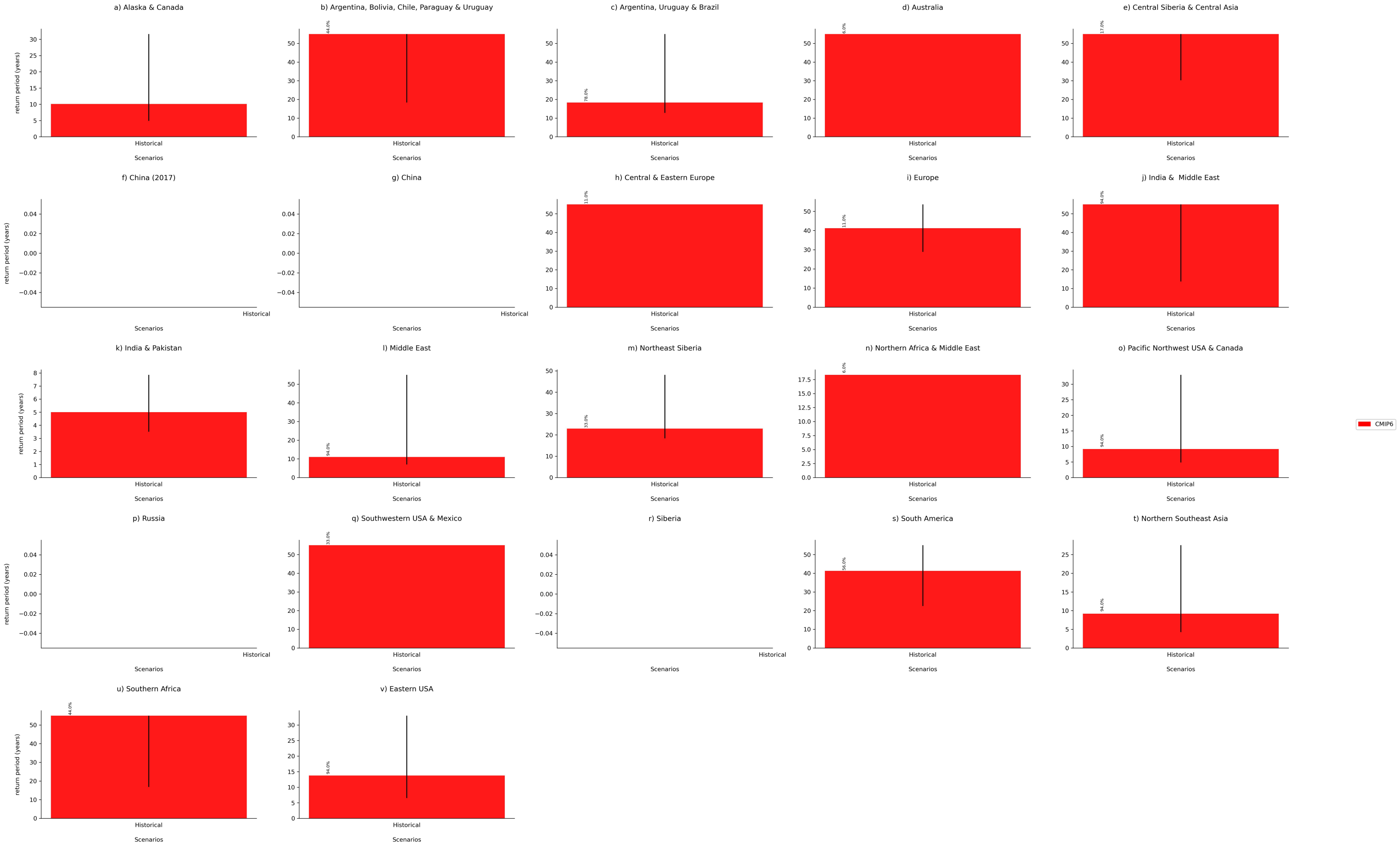

a) Alaska & Canada
b) Argentina, Bolivia, Chile, Paraguay & Uruguay
c) Argentina, Uruguay & Brazil
d) Australia
e) Central Siberia & Central Asia
f) China (2017)
g) China
h) Central & Eastern Europe
i) Europe
j) India & Middle East
k) India & Pakistan
l) Middle East
m) Northeast Siberia
n) Northern Africa & Middle East
o) Pacific Northwest USA & Canada
p) Russia
q) Southwestern USA & Mexico
r) Siberia
s) South America
t) Northern Southeast Asia
u) Southern Africa
v) Eastern USA
return period (years)
Historical
Scenarios
CMIP6

Supplementary Figure 344: Return times (RT) during present-to-near-future (2015-2054) based on Berkeley Earth (Tx95.0p) - Return times (RT) (in years) during the present-to-near-future period between 2015 and 2054 for Cumulative Heat (°C) of the respective observed event. For each region, values are shown for the different scenarios (increasing in emissions from left to right for the CMIP5 (green), CMIP6 (red), CanESM5 (dark blue), and ACCESS-ESM1-5 (light blue) ensembles. The error bars indicate the 5th and 95th percentile of the respective sample. Where the respective ensemble contains models not able to simulate the magnitude of the respective observed event's Cumulative Heat (°C), the proportion of models that are able to simulate an event with a value of Cumulative Heat (°C) at least as high as the observed event is indicated by the percentage values above the individual bars. The observed reference values are calculated using data from Berkeley Earth.

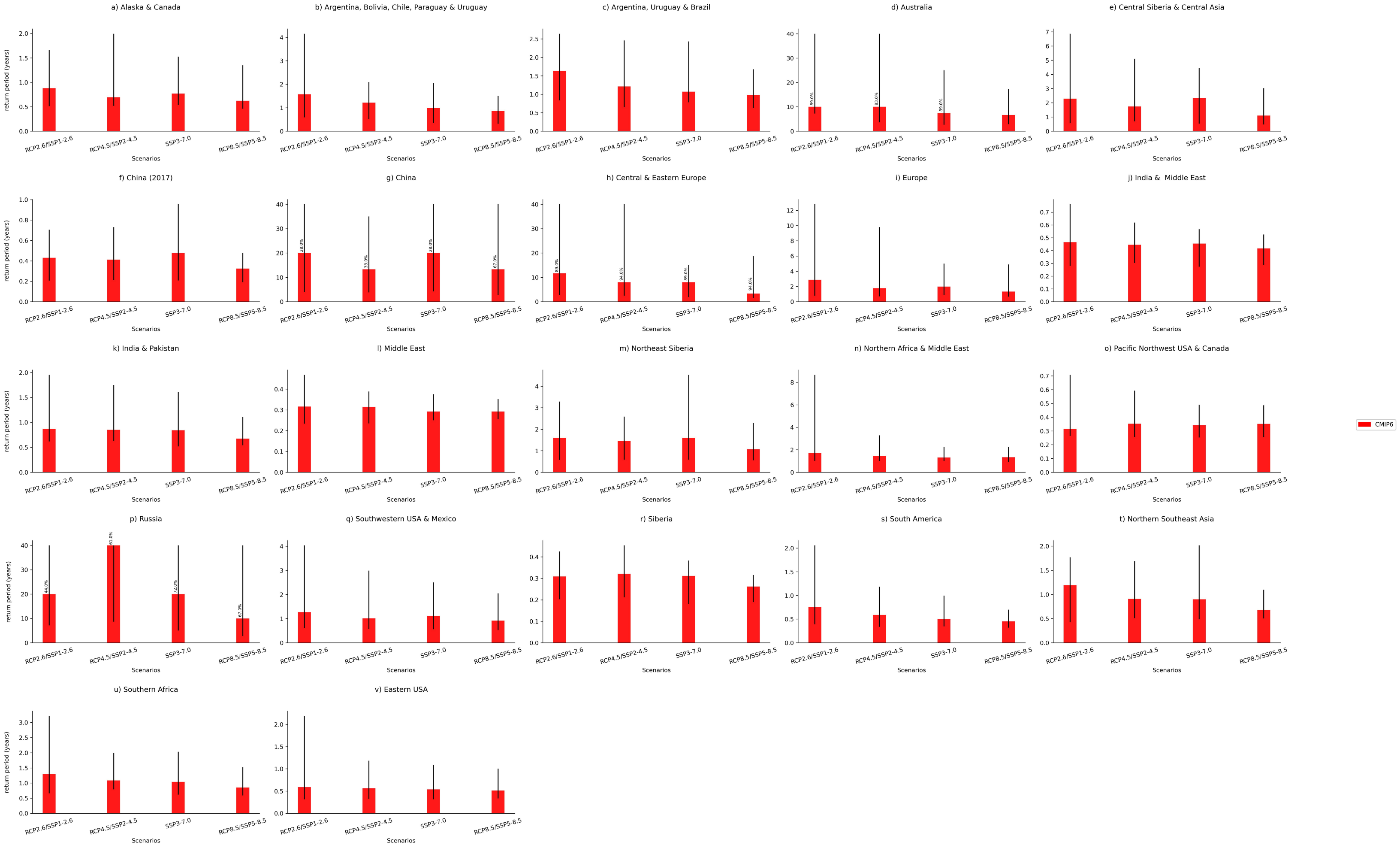
a) Alaska & Canada
b) Argentina, Bolivia, Chile, Paraguay & Uruguay
c) Argentina, Uruguay & Brazil
d) Australia
89.0%
83.0%
89.0%
e) Central Siberia & Central Asia
f) China (2017)
g) China
28.0%
33.0%
28.0%
67.0%
h) Central & Eastern Europe
89.0%
94.0%
89.0%
94.0%
i) Europe
j) India & Middle East
k) India & Pakistan
l) Middle East
m) Northeast Siberia
n) Northern Africa & Middle East
o) Pacific Northwest USA & Canada
p) Russia
44.0%
61.0%
72.0%
67.0%
q) Southwestern USA & Mexico
r) Siberia
s) South America
t) Northern Southeast Asia
u) Southern Africa
v) Eastern USA
return period (years)
Scenarios
RCP2.6/SSP1-2.6
RCP4.5/SSP2-4.5
SSP3-7.0
RCP8.5/SSP5-8.5
CMIP6

Supplementary Figure 345: Return times (RT) during present-to-near-future (2015-2054) based on Berkeley Earth (Tx97.5p) - Return times (RT) (in years) during the present-to-near-future period between 2015 and 2054 for Cumulative Heat (°C) of the respective observed event. For each region, values are shown for the different scenarios (increasing in emissions from left to right for the CMIP5 (green), CMIP6 (red), CanESM5 (dark blue), and ACCESS-ESM1-5 (light blue) ensembles. The error bars indicate the 5th and 95th percentile of the respective sample. Where the respective ensemble contains models not able to simulate the magnitude of the respective observed event's Cumulative Heat (°C), the proportion of models that are able to simulate an event with a value of Cumulative Heat (°C) at least as high as the observed event is indicated by the percentage values above the individual bars. The observed reference values are calculated using data from Berkeley Earth.

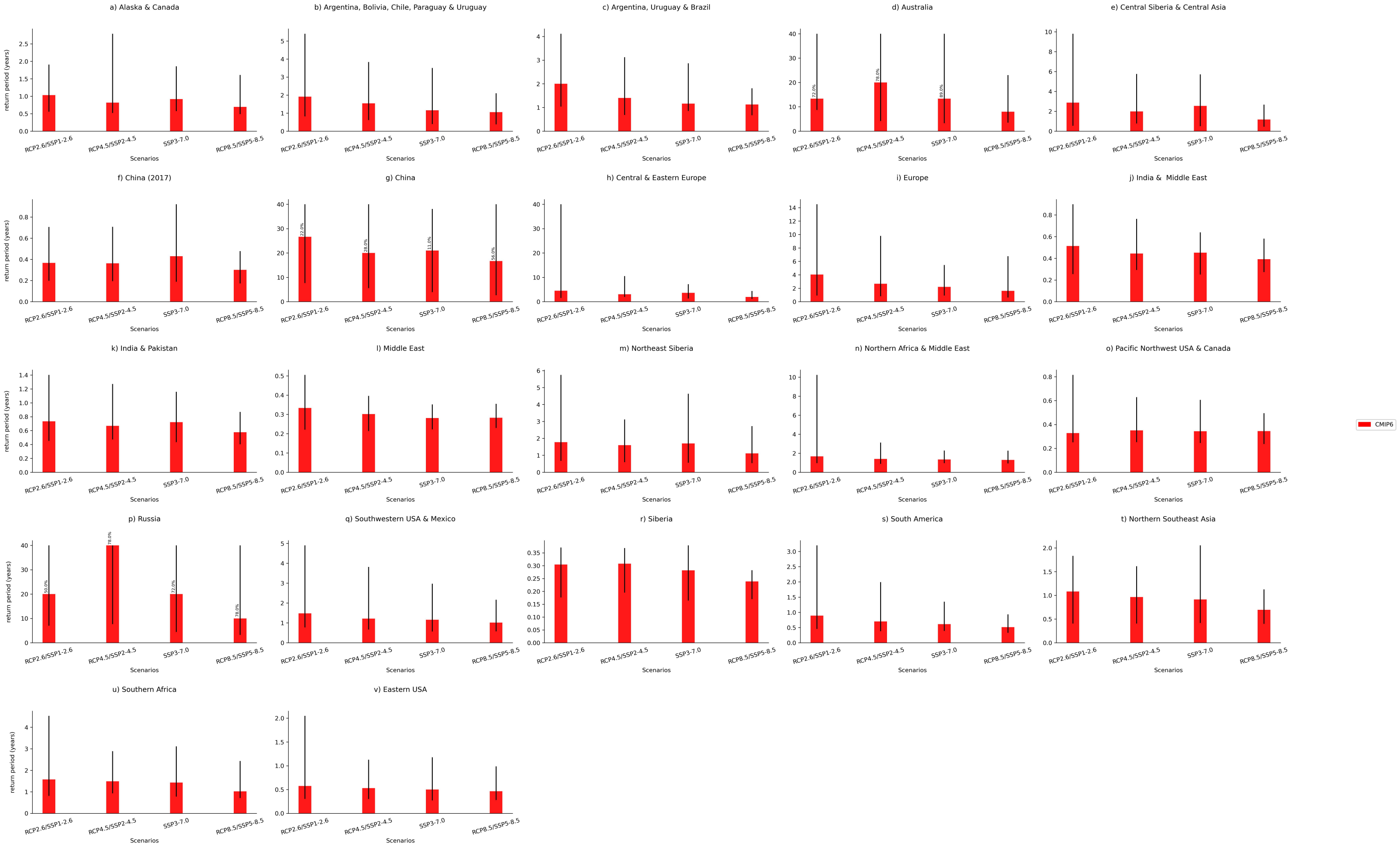
a) Alaska & Canada
b) Argentina, Bolivia, Chile, Paraguay & Uruguay
c) Argentina, Uruguay & Brazil
d) Australia
e) Central Siberia & Central Asia
f) China (2017)
g) China
h) Central & Eastern Europe
i) Europe
j) India & Middle East
k) India & Pakistan
l) Middle East
m) Northeast Siberia
n) Northern Africa & Middle East
o) Pacific Northwest USA & Canada
p) Russia
q) Southwestern USA & Mexico
r) Siberia
s) South America
t) Northern Southeast Asia
u) Southern Africa
v) Eastern USA
return period (years)
Scenarios
RCP2.6/SSP1-2.6
RCP4.5/SSP2-4.5
SSP3-7.0
RCP8.5/SSP5-8.5
CMIP6
72.0%
78.0%
89.0%
22.0%
28.0%
11.0%
56.0%
50.0%
78.0%
72.0%
78.0%

Supplementary Figure 346: Return times (RT) during present-to-near-future (2015-2054) based on Berkeley Earth (Tx99.0p) - Return times (RT) (in years) during the present-to-near-future period between 2015 and 2054 for Cumulative Heat (°C) of the respective observed event. For each region, values are shown for the different scenarios (increasing in emissions from left to right for the CMIP5 (green), CMIP6 (red), CanESM5 (dark blue), and ACCESS-ESM1-5 (light blue) ensembles. The error bars indicate the 5th and 95th percentile of the respective sample. Where the respective ensemble contains models not able to simulate the magnitude of the respective observed event's Cumulative Heat (°C), the proportion of models that are able to simulate an event with a value of Cumulative Heat (°C) at least as high as the observed event is indicated by the percentage values above the individual bars. The observed reference values are calculated using data from Berkeley Earth.

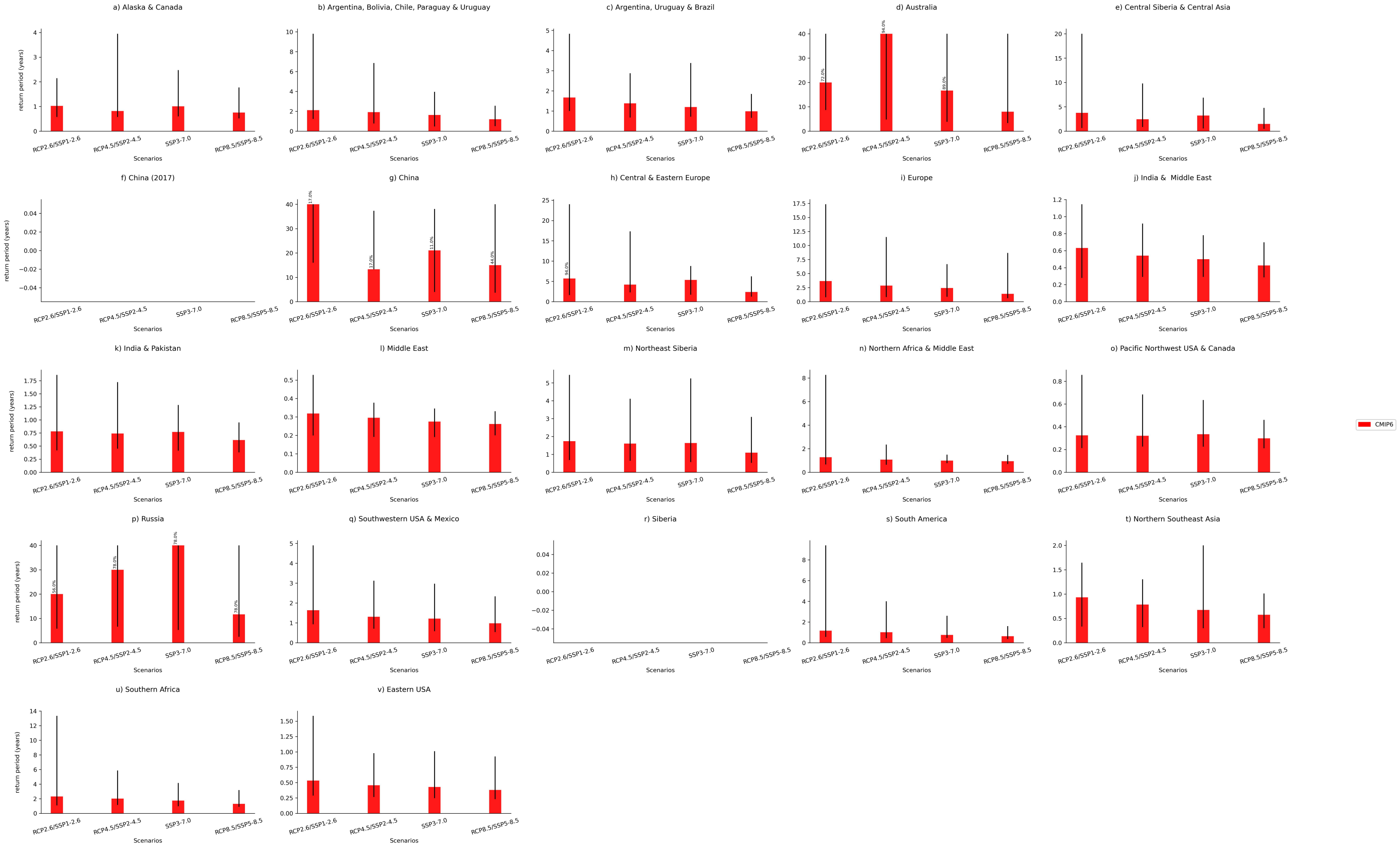

a) Alaska & Canada
b) Argentina, Bolivia, Chile, Paraguay & Uruguay
c) Argentina, Uruguay & Brazil
d) Australia
72.0%
94.0%
89.0%
e) Central Siberia & Central Asia
f) China (2017)
g) China
17.0%
17.0%
11.0%
44.0%
h) Central & Eastern Europe
94.0%
i) Europe
j) India & Middle East
k) India & Pakistan
l) Middle East
m) Northeast Siberia
n) Northern Africa & Middle East
o) Pacific Northwest USA & Canada
p) Russia
56.0%
78.0%
78.0%
78.0%
q) Southwestern USA & Mexico
r) Siberia
s) South America
t) Northern Southeast Asia
u) Southern Africa
v) Eastern USA
return period (years)
Scenarios
RCP2.6/SSP1-2.6
RCP4.5/SSP2-4.5
SSP3-7.0
RCP8.5/SSP5-8.5
CMIP6

Supplementary Figure 347: Return times (RT) during end-of-century (2060-2099) based on Berkeley Earth (Tx95.0p) - Return times (RT) (in years) during the end-of-century period between 2060 and 2099 for Cumulative Heat (°C) of the respective observed event. For each region, values are shown for the different scenarios (increasing in emissions from left to right for the CMIP5 (green), CMIP6 (red), CanESM5 (dark blue), and ACCESS-ESM1-5 (light blue) ensembles. The error bars indicate the 5th and 95th percentile of the respective sample. Where the respective ensemble contains models not able to simulate the magnitude of the respective observed event's Cumulative Heat (°C), the proportion of models that are able to simulate an event with a value of Cumulative Heat (°C) at least as high as the observed event is indicated by the percentage values above the individual bars. The observed reference values are calculated using data from Berkeley Earth.

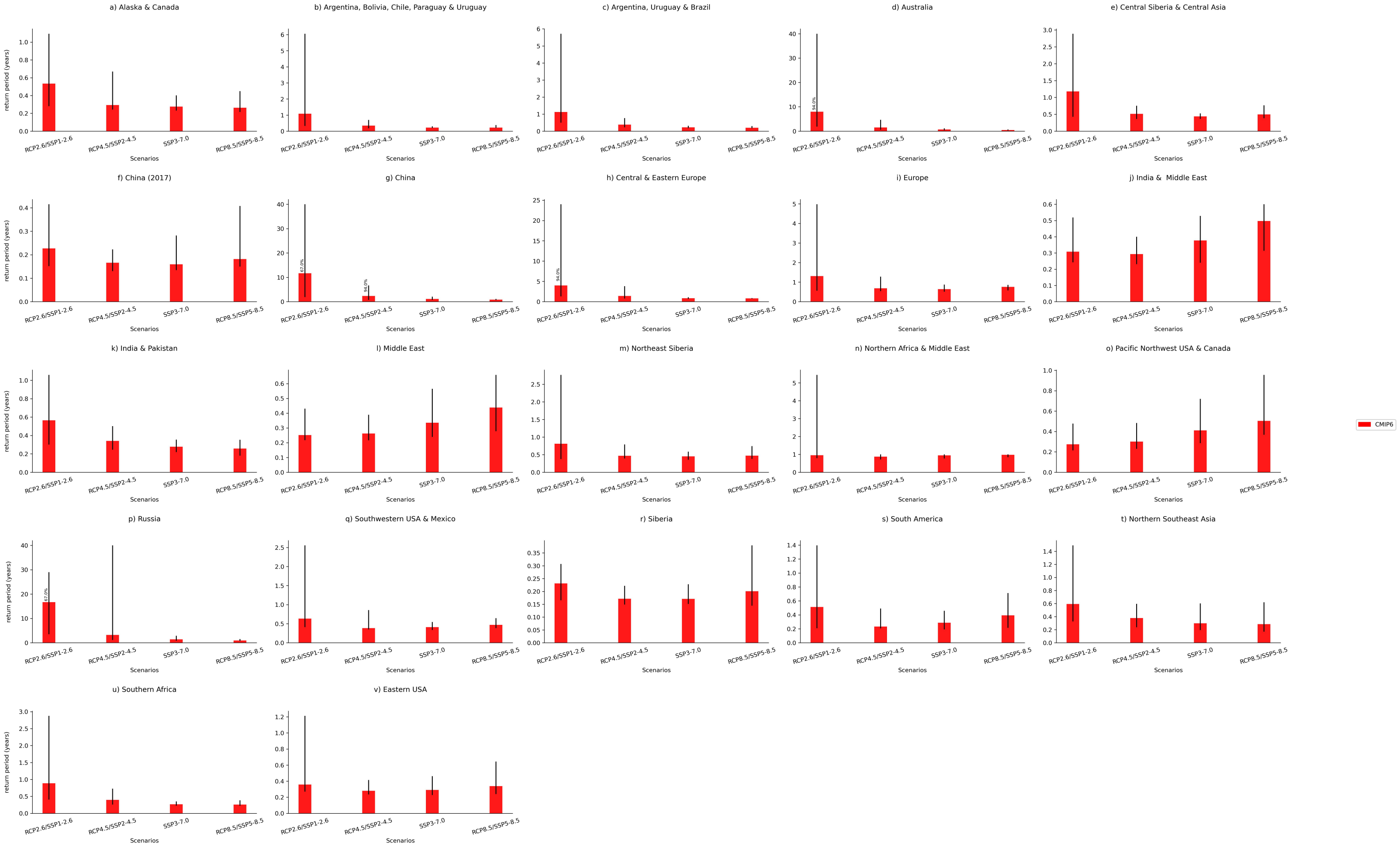
a) Alaska & Canada
b) Argentina, Bolivia, Chile, Paraguay & Uruguay
c) Argentina, Uruguay & Brazil
d) Australia
e) Central Siberia & Central Asia
f) China (2017)
g) China
h) Central & Eastern Europe
i) Europe
j) India & Middle East
k) India & Pakistan
l) Middle East
m) Northeast Siberia
n) Northern Africa & Middle East
o) Pacific Northwest USA & Canada
p) Russia
q) Southwestern USA & Mexico
r) Siberia
s) South America
t) Northern Southeast Asia
u) Southern Africa
v) Eastern USA
return period (years)
Scenarios
RCP2.6/SSP1-2.6
RCP4.5/SSP2-4.5
SSP3-7.0
RCP8.5/SSP5-8.5
94.0%
67.0%
CMIP6

Supplementary Figure 348: Return times (RT) during end-of-century (2060-2099) based on Berkeley Earth (Tx97.5p) - Return times (RT) (in years) during the end-of-century period between 2060 and 2099 for Cumulative Heat (°C) of the respective observed event. For each region, values are shown for the different scenarios (increasing in emissions from left to right for the CMIP5 (green), CMIP6 (red), CanESM5 (dark blue), and ACCESS-ESM1-5 (light blue) ensembles. The error bars indicate the 5th and 95th percentile of the respective sample. Where the respective ensemble contains models not able to simulate the magnitude of the respective observed event's Cumulative Heat (°C), the proportion of models that are able to simulate an event with a value of Cumulative Heat (°C) at least as high as the observed event is indicated by the percentage values above the individual bars. The observed reference values are calculated using data from Berkeley Earth.

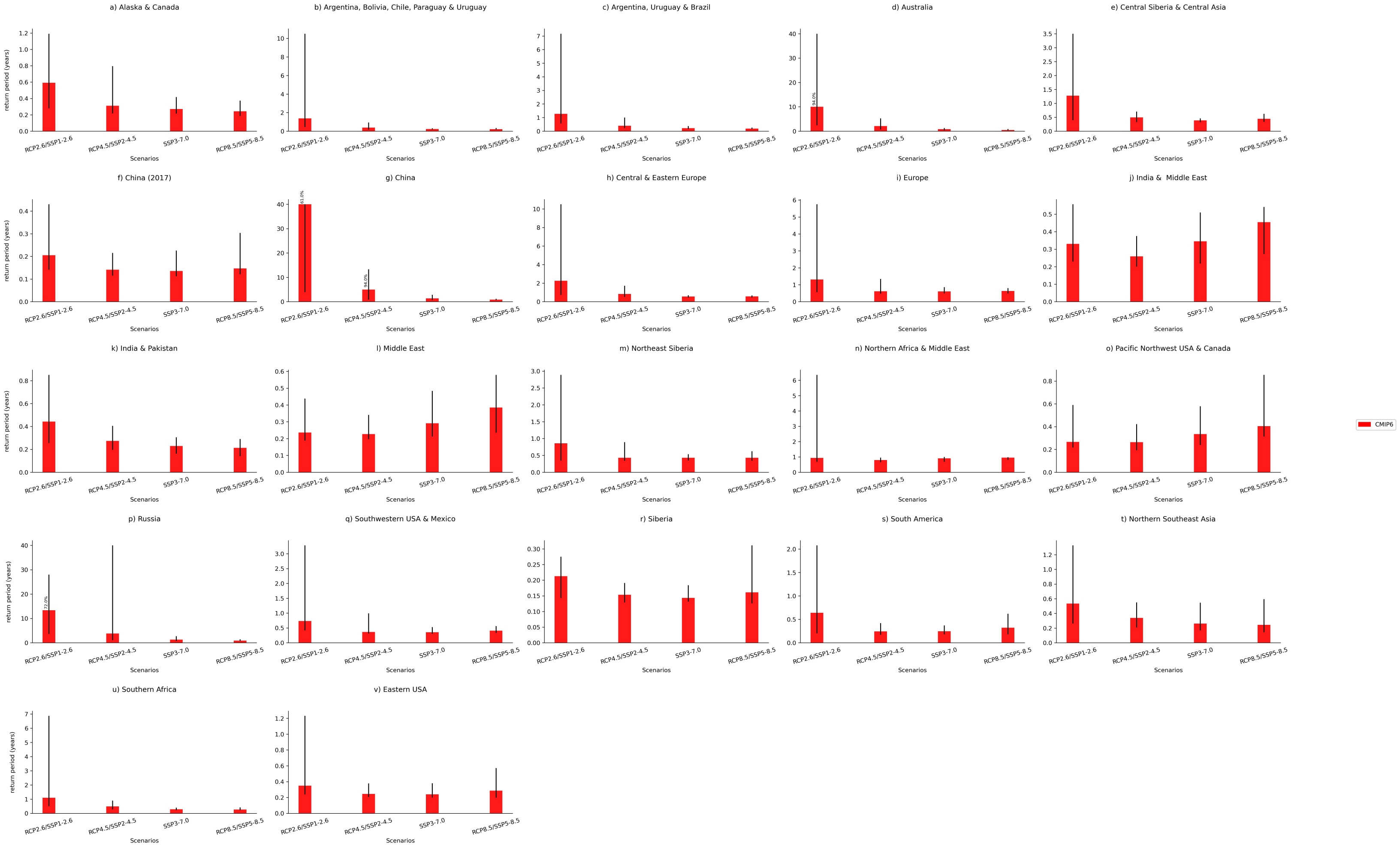

a) Alaska & Canada
b) Argentina, Bolivia, Chile, Paraguay & Uruguay
c) Argentina, Uruguay & Brazil
d) Australia
94.0%
e) Central Siberia & Central Asia
f) China (2017)
g) China
61.0%
94.0%
h) Central & Eastern Europe
i) Europe
j) India & Middle East
k) India & Pakistan
l) Middle East
m) Northeast Siberia
n) Northern Africa & Middle East
o) Pacific Northwest USA & Canada
p) Russia
72.0%
q) Southwestern USA & Mexico
r) Siberia
s) South America
t) Northern Southeast Asia
u) Southern Africa
v) Eastern USA
return period (years)
Scenarios
RCP2.6/SSP1-2.6
RCP4.5/SSP2-4.5
SSP3-7.0
RCP8.5/SSP5-8.5
CMIP6

Supplementary Figure 349: Return times (RT) during end-of-century (2060-2099) based on Berkeley Earth (Tx99.0p) - Return times (RT) (in years) during the end-of-century period between 2060 and 2099 for Cumulative Heat (°C) of the respective observed event. For each region, values are shown for the different scenarios (increasing in emissions from left to right for the CMIP5 (green), CMIP6 (red), CanESM5 (dark blue), and ACCESS-ESM1-5 (light blue) ensembles. The error bars indicate the 5th and 95th percentile of the respective sample. Where the respective ensemble contains models not able to simulate the magnitude of the respective observed event's Cumulative Heat (°C), the proportion of models that are able to simulate an event with a value of Cumulative Heat (°C) at least as high as the observed event is indicated by the percentage values above the individual bars. The observed reference values are calculated using data from Berkeley Earth.

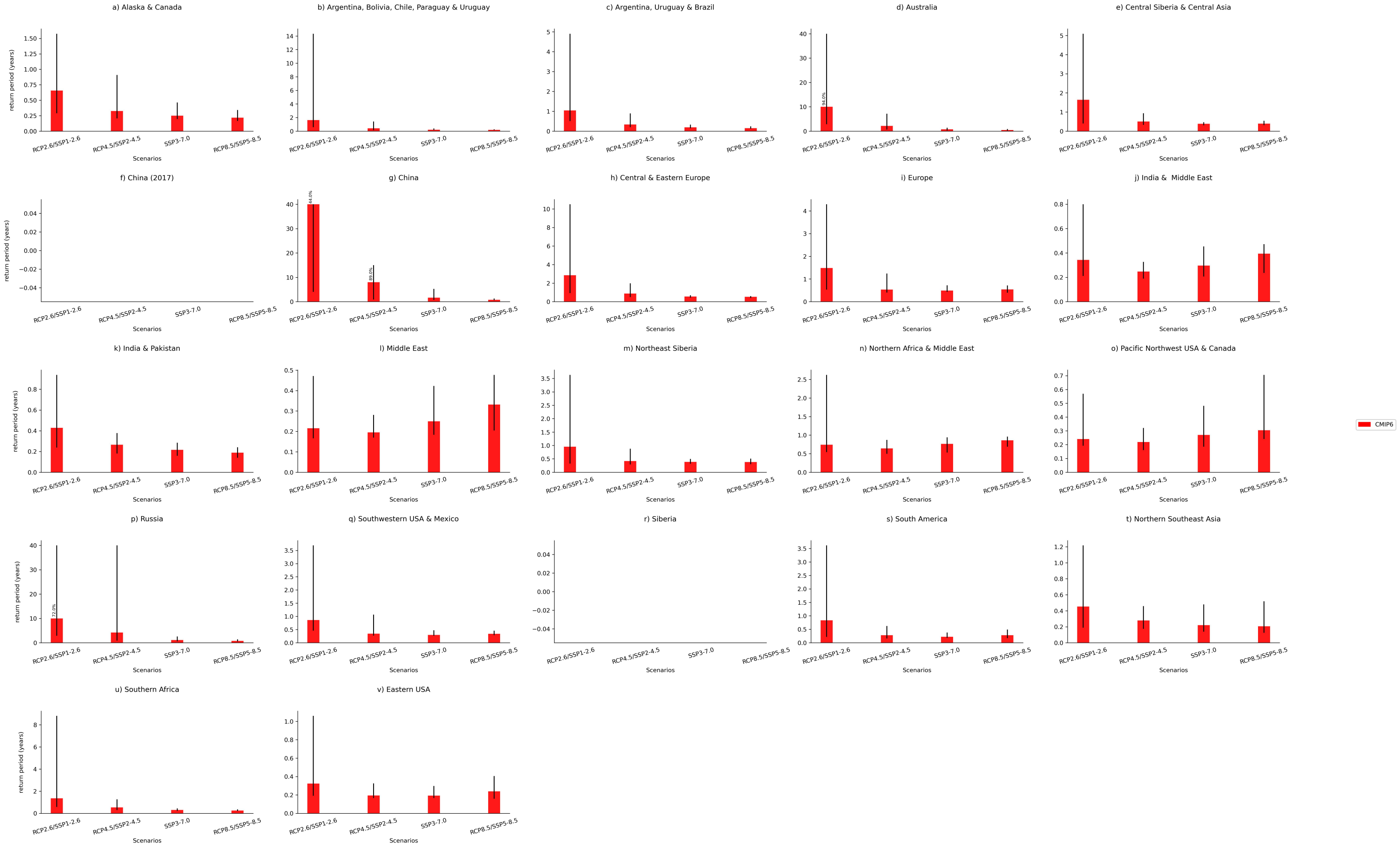
a) Alaska & Canada
b) Argentina, Bolivia, Chile, Paraguay & Uruguay
c) Argentina, Uruguay & Brazil
d) Australia
e) Central Siberia & Central Asia
f) China (2017)
g) China
h) Central & Eastern Europe
i) Europe
j) India & Middle East
k) India & Pakistan
l) Middle East
m) Northeast Siberia
n) Northern Africa & Middle East
o) Pacific Northwest USA & Canada
p) Russia
q) Southwestern USA & Mexico
r) Siberia
s) South America
t) Northern Southeast Asia
u) Southern Africa
v) Eastern USA
return period (years)
Scenarios
RCP2.6/SSP1-2.6
RCP4.5/SSP2-4.5
SSP3-7.0
RCP8.5/SSP5-8.5
94.0%
44.0%
89.0%
72.0%
CMIP6

Supplementary Figure 350: Return times (RT) during historical (1951-2005) based on ERA5 (Tx95.0p) - Return times (RT) (in years) during the historical period between 1951 and 2005 for Cumulative Heat (°C) of the respective observed event. For each region, values are shown for the different scenarios (increasing in emissions from left to right for the CMIP5 (green), CMIP6 (red), CanESM5 (dark blue), and ACCESS-ESM1-5 (light blue) ensembles. The error bars indicate the 5th and 95th percentile of the respective sample. Where the respective ensemble contains models not able to simulate the magnitude of the respective observed event's Cumulative Heat (°C), the proportion of models that are able to simulate an event with a value of Cumulative Heat (°C) at least as high as the observed event is indicated by the percentage values above the individual bars. The observed reference values are calculated using data from ERA5.

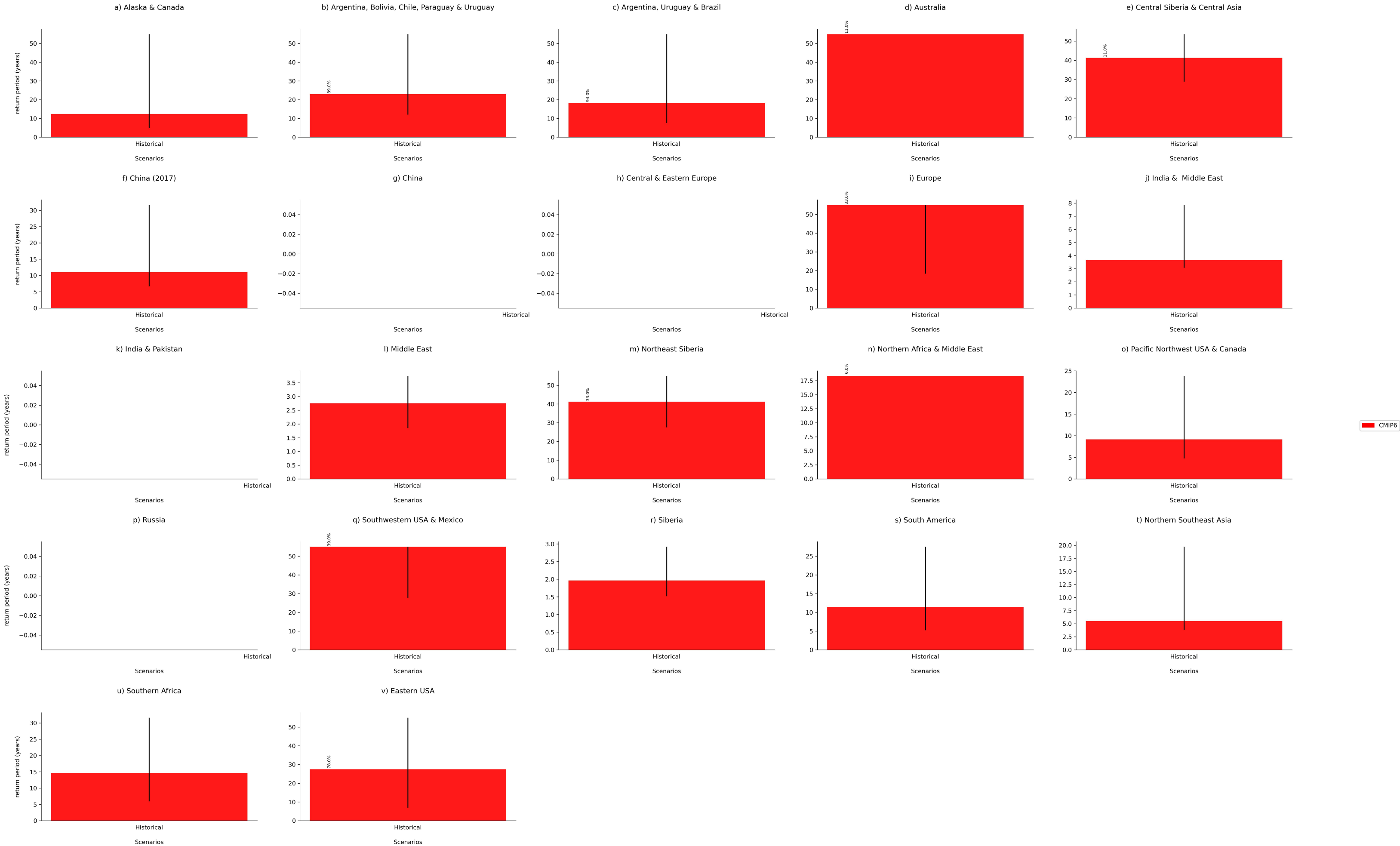
a) Alaska & Canada
b) Argentina, Bolivia, Chile, Paraguay & Uruguay
89.0%
c) Argentina, Uruguay & Brazil
94.0%
d) Australia
11.0%
e) Central Siberia & Central Asia
11.0%
f) China (2017)
g) China
h) Central & Eastern Europe
i) Europe
33.0%
j) India & Middle East
k) India & Pakistan
l) Middle East
m) Northeast Siberia
33.0%
n) Northern Africa & Middle East
6.0%
o) Pacific Northwest USA & Canada
p) Russia
q) Southwestern USA & Mexico
39.0%
r) Siberia
s) South America
t) Northern Southeast Asia
u) Southern Africa
v) Eastern USA
78.0%
return period (years)
Historical
Scenarios
CMIP6

Supplementary Figure 351: Return times (RT) during historical (1951-2005) based on ERA5 (Tx97.5p) - Return times (RT) (in years) during the historical period between 1951 and 2005 for Cumulative Heat (°C) of the respective observed event. For each region, values are shown for the different scenarios (increasing in emissions from left to right for the CMIP5 (green), CMIP6 (red), CanESM5 (dark blue), and ACCESS-ESM1-5 (light blue) ensembles. The error bars indicate the 5th and 95th percentile of the respective sample. Where the respective ensemble contains models not able to simulate the magnitude of the respective observed event's Cumulative Heat (°C), the proportion of models that are able to simulate an event with a value of Cumulative Heat (°C) at least as high as the observed event is indicated by the percentage values above the individual bars. The observed reference values are calculated using data from ERA5.

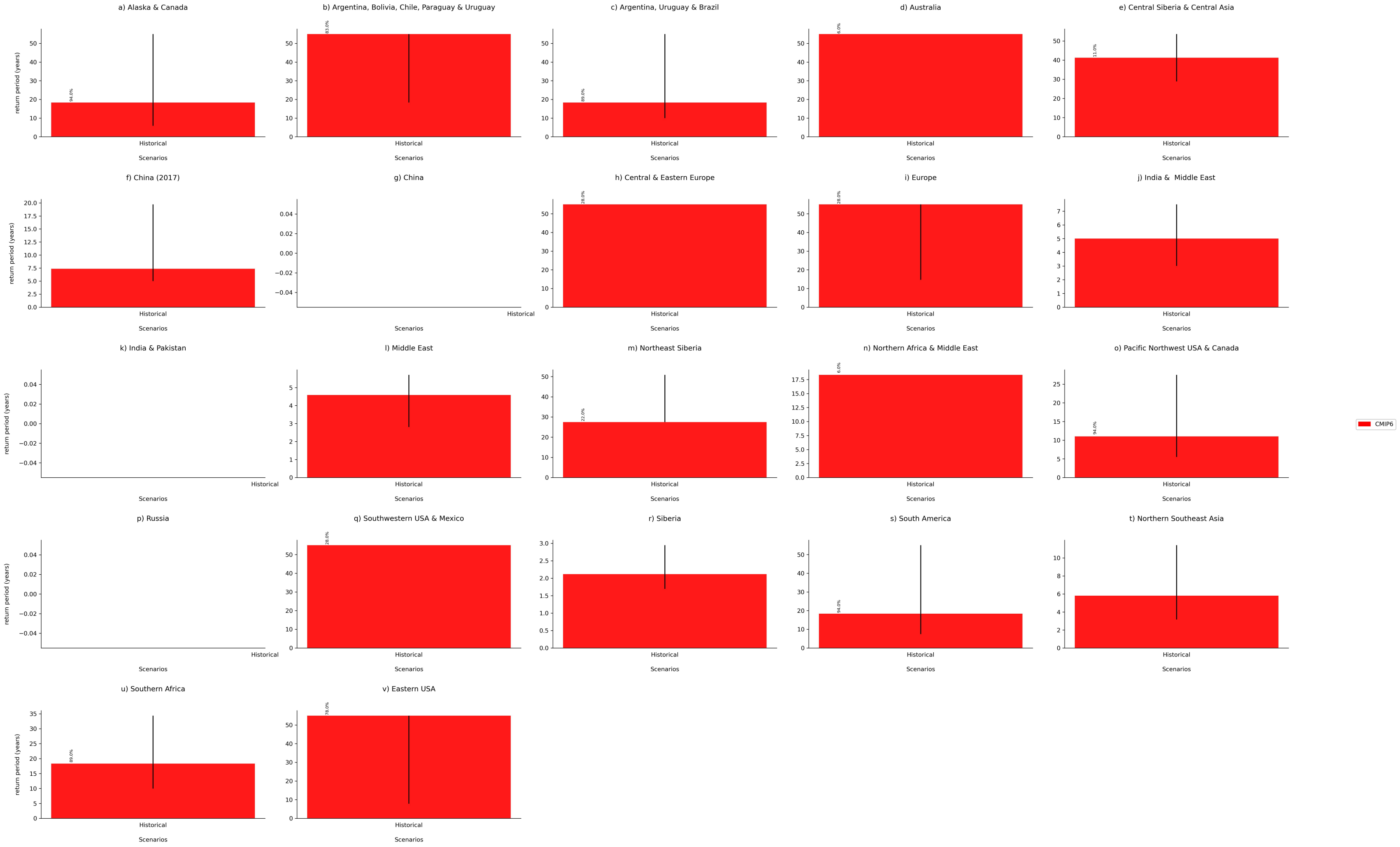
a) Alaska & Canada
b) Argentina, Bolivia, Chile, Paraguay & Uruguay
c) Argentina, Uruguay & Brazil
d) Australia
e) Central Siberia & Central Asia
f) China (2017)
g) China
h) Central & Eastern Europe
i) Europe
j) India & Middle East
k) India & Pakistan
l) Middle East
m) Northeast Siberia
n) Northern Africa & Middle East
o) Pacific Northwest USA & Canada
p) Russia
q) Southwestern USA & Mexico
r) Siberia
s) South America
t) Northern Southeast Asia
u) Southern Africa
v) Eastern USA
return period (years)
Historical
Scenarios
CMIP6
94.0%
83.0%
89.0%
6.0%
11.0%
28.0%
28.0%
22.0%
6.0%
94.0%
28.0%
94.0%
89.0%
78.0%

Supplementary Figure 352: Return times (RT) during historical (1951-2005) based on ERA5 (Tx99.0p) - Return times (RT) (in years) during the historical period between 1951 and 2005 for Cumulative Heat (°C) of the respective observed event. For each region, values are shown for the different scenarios (increasing in emissions from left to right for the CMIP5 (green), CMIP6 (red), CanESM5 (dark blue), and ACCESS-ESM1-5 (light blue) ensembles. The error bars indicate the 5th and 95th percentile of the respective sample. Where the respective ensemble contains models not able to simulate the magnitude of the respective observed event's Cumulative Heat (°C), the proportion of models that are able to simulate an event with a value of Cumulative Heat (°C) at least as high as the observed event is indicated by the percentage values above the individual bars. The observed reference values are calculated using data from ERA5.

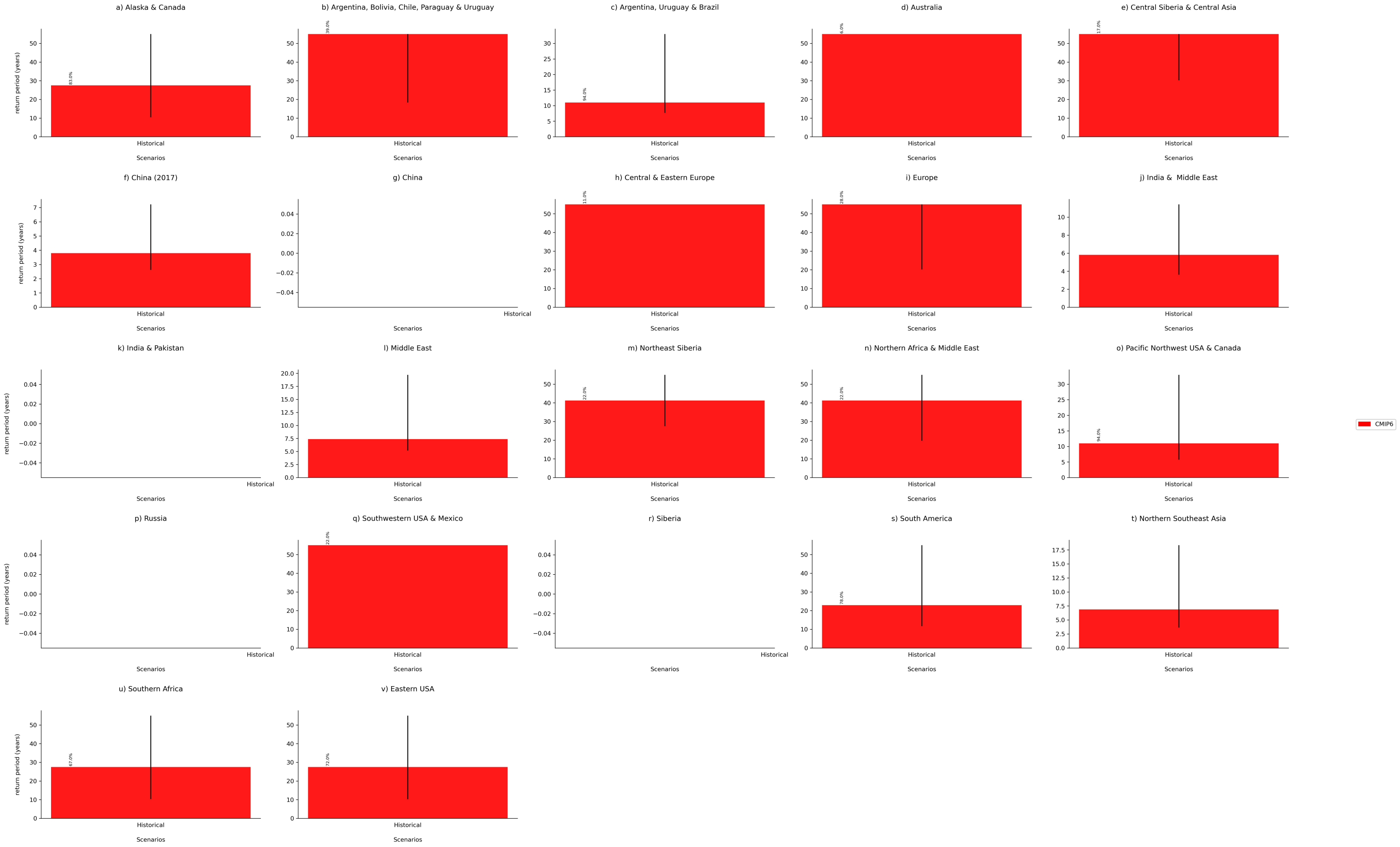
a) Alaska & Canada
83.0%
b) Argentina, Bolivia, Chile, Paraguay & Uruguay
39.0%
c) Argentina, Uruguay & Brazil
94.0%
d) Australia
6.0%
e) Central Siberia & Central Asia
17.0%
f) China (2017)
g) China
h) Central & Eastern Europe
11.0%
i) Europe
28.0%
j) India & Middle East
k) India & Pakistan
l) Middle East
m) Northeast Siberia
22.0%
n) Northern Africa & Middle East
22.0%
o) Pacific Northwest USA & Canada
94.0%
p) Russia
q) Southwestern USA & Mexico
22.0%
r) Siberia
s) South America
78.0%
t) Northern Southeast Asia
u) Southern Africa
67.0%
v) Eastern USA
72.0%
return period (years)
Historical
Scenarios
CMIP6

Supplementary Figure 353: Return times (RT) during present-to-near-future (2015-2054) based on ERA5 (Tx95.0p) - Return times (RT) (in years) during the present-to-near-future period between 2015 and 2054 for Cumulative Heat (°C) of the respective observed event. For each region, values are shown for the different scenarios (increasing in emissions from left to right for the CMIP5 (green), CMIP6 (red), CanESM5 (dark blue), and ACCESS-ESM1-5 (light blue) ensembles. The error bars indicate the 5th and 95th percentile of the respective sample. Where the respective ensemble contains models not able to simulate the magnitude of the respective observed event's Cumulative Heat (°C), the proportion of models that are able to simulate an event with a value of Cumulative Heat (°C) at least as high as the observed event is indicated by the percentage values above the individual bars. The observed reference values are calculated using data from ERA5.

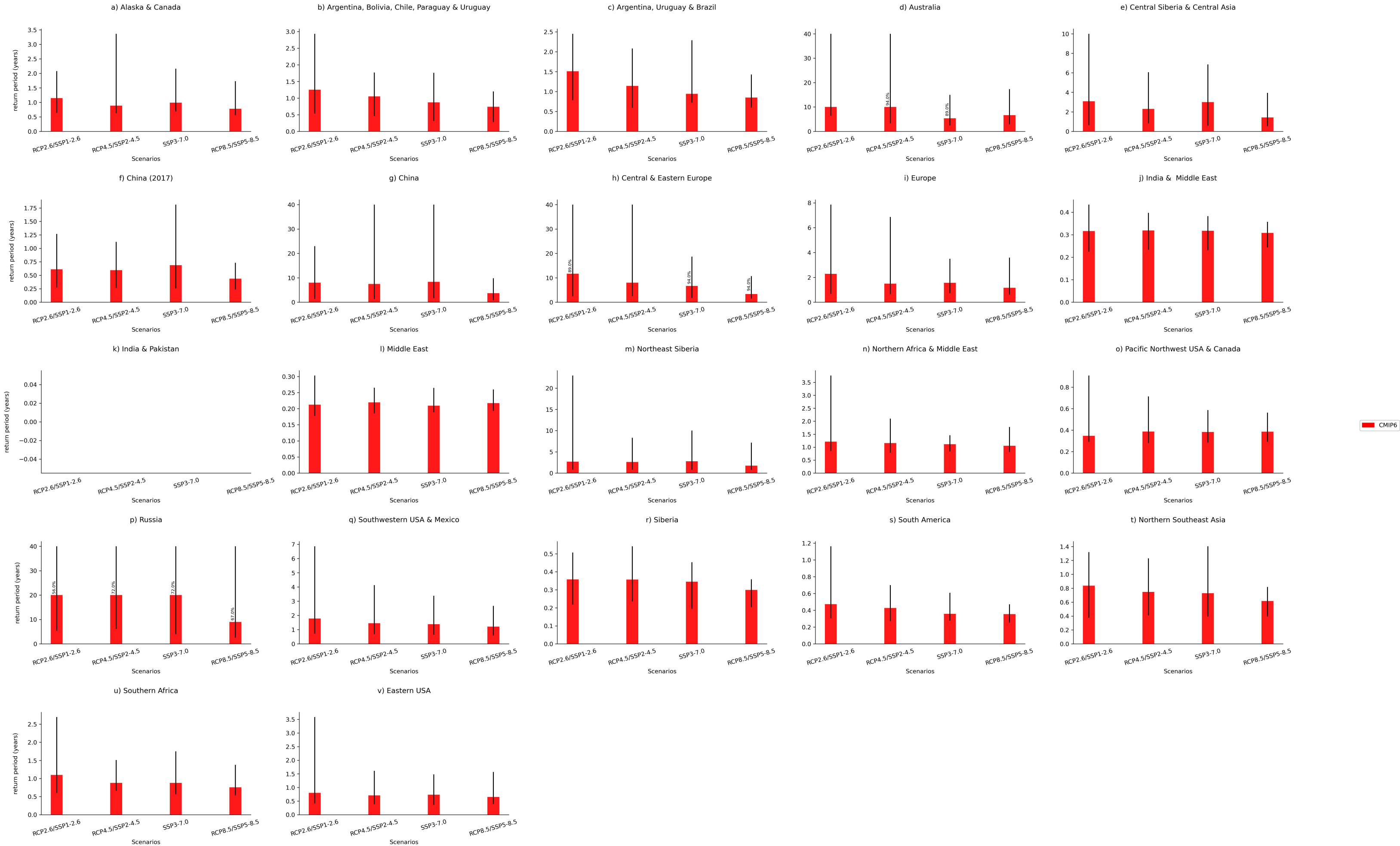

a) Alaska & Canada
b) Argentina, Bolivia, Chile, Paraguay & Uruguay
c) Argentina, Uruguay & Brazil
d) Australia
e) Central Siberia & Central Asia
f) China (2017)
g) China
h) Central & Eastern Europe
i) Europe
j) India & Middle East
k) India & Pakistan
l) Middle East
m) Northeast Siberia
n) Northern Africa & Middle East
o) Pacific Northwest USA & Canada
p) Russia
q) Southwestern USA & Mexico
r) Siberia
s) South America
t) Northern Southeast Asia
u) Southern Africa
v) Eastern USA
return period (years)
Scenarios
RCP2.6/SSP1-2.6
RCP4.5/SSP2-4.5
SSP3-7.0
RCP8.5/SSP5-8.5
94.0%
89.0%
89.0%
94.0%
94.0%
56.0%
72.0%
72.0%
67.0%
CMIP6

Supplementary Figure 354: Return times (RT) during present-to-near-future (2015-2054) based on ERA5 (Tx97.5p) - Return times (RT) (in years) during the present-to-near-future period between 2015 and 2054 for Cumulative Heat (°C) of the respective observed event. For each region, values are shown for the different scenarios (increasing in emissions from left to right for the CMIP5 (green), CMIP6 (red), CanESM5 (dark blue), and ACCESS-ESM1-5 (light blue) ensembles. The error bars indicate the 5th and 95th percentile of the respective sample. Where the respective ensemble contains models not able to simulate the magnitude of the respective observed event's Cumulative Heat (°C), the proportion of models that are able to simulate an event with a value of Cumulative Heat (°C) at least as high as the observed event is indicated by the percentage values above the individual bars. The observed reference values are calculated using data from ERA5.

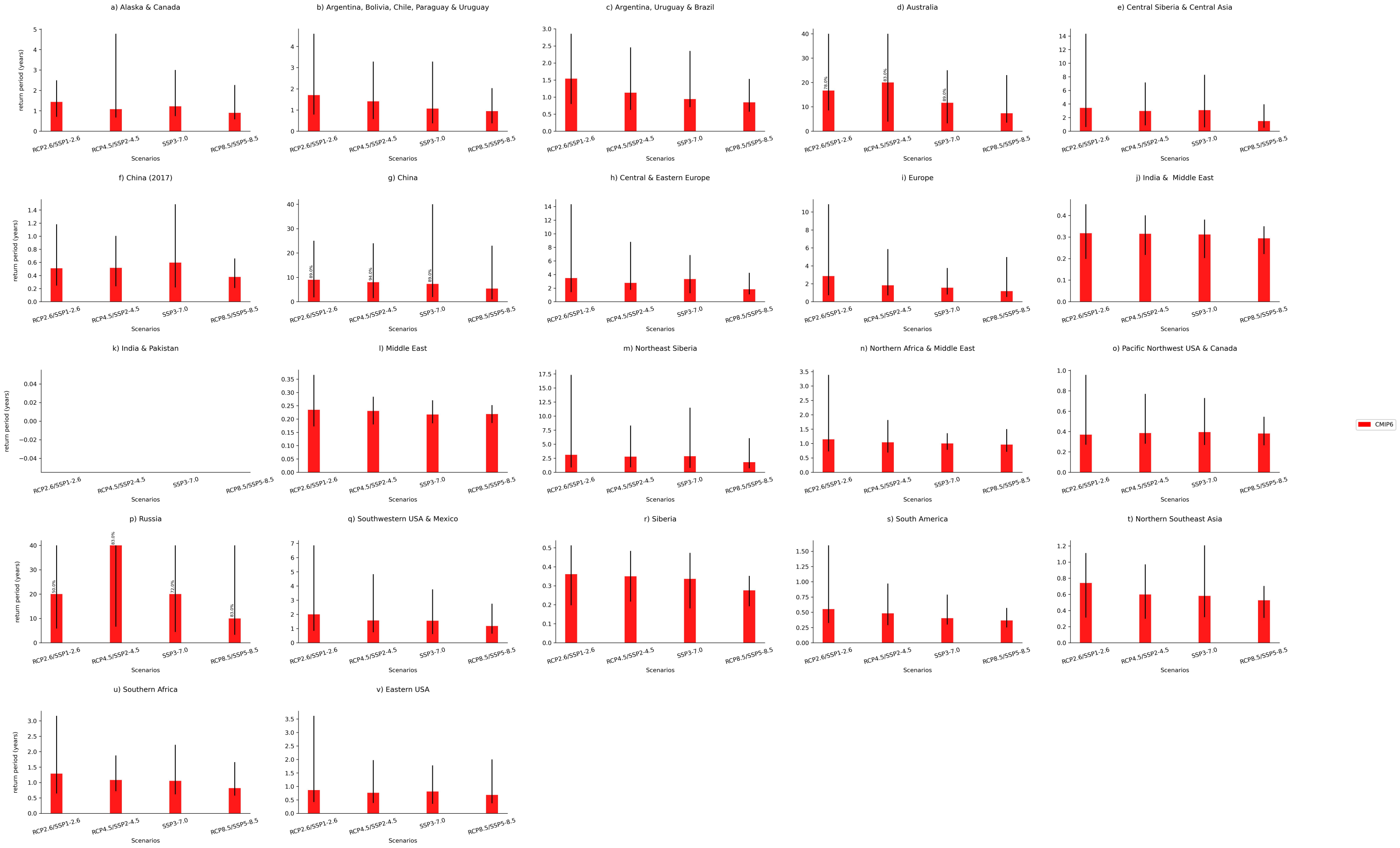
a) Alaska & Canada
b) Argentina, Bolivia, Chile, Paraguay & Uruguay
c) Argentina, Uruguay & Brazil
d) Australia
e) Central Siberia & Central Asia
f) China (2017)
g) China
h) Central & Eastern Europe
i) Europe
j) India & Middle East
k) India & Pakistan
l) Middle East
m) Northeast Siberia
n) Northern Africa & Middle East
o) Pacific Northwest USA & Canada
p) Russia
q) Southwestern USA & Mexico
r) Siberia
s) South America
t) Northern Southeast Asia
u) Southern Africa
v) Eastern USA
return period (years)
Scenarios
RCP2.6/SSP1-2.6
RCP4.5/SSP2-4.5
SSP3-7.0
RCP8.5/SSP5-8.5
CMIP6

Supplementary Figure 355: Return times (RT) during present-to-near-future (2015-2054) based on ERA5 (Tx99.0p) - Return times (RT) (in years) during the present-to-near-future period between 2015 and 2054 for Cumulative Heat (°C) of the respective observed event. For each region, values are shown for the different scenarios (increasing in emissions from left to right for the CMIP5 (green), CMIP6 (red), CanESM5 (dark blue), and ACCESS-ESM1-5 (light blue) ensembles. The error bars indicate the 5th and 95th percentile of the respective sample. Where the respective ensemble contains models not able to simulate the magnitude of the respective observed event's Cumulative Heat (°C), the proportion of models that are able to simulate an event with a value of Cumulative Heat (°C) at least as high as the observed event is indicated by the percentage values above the individual bars. The observed reference values are calculated using data from ERA5.

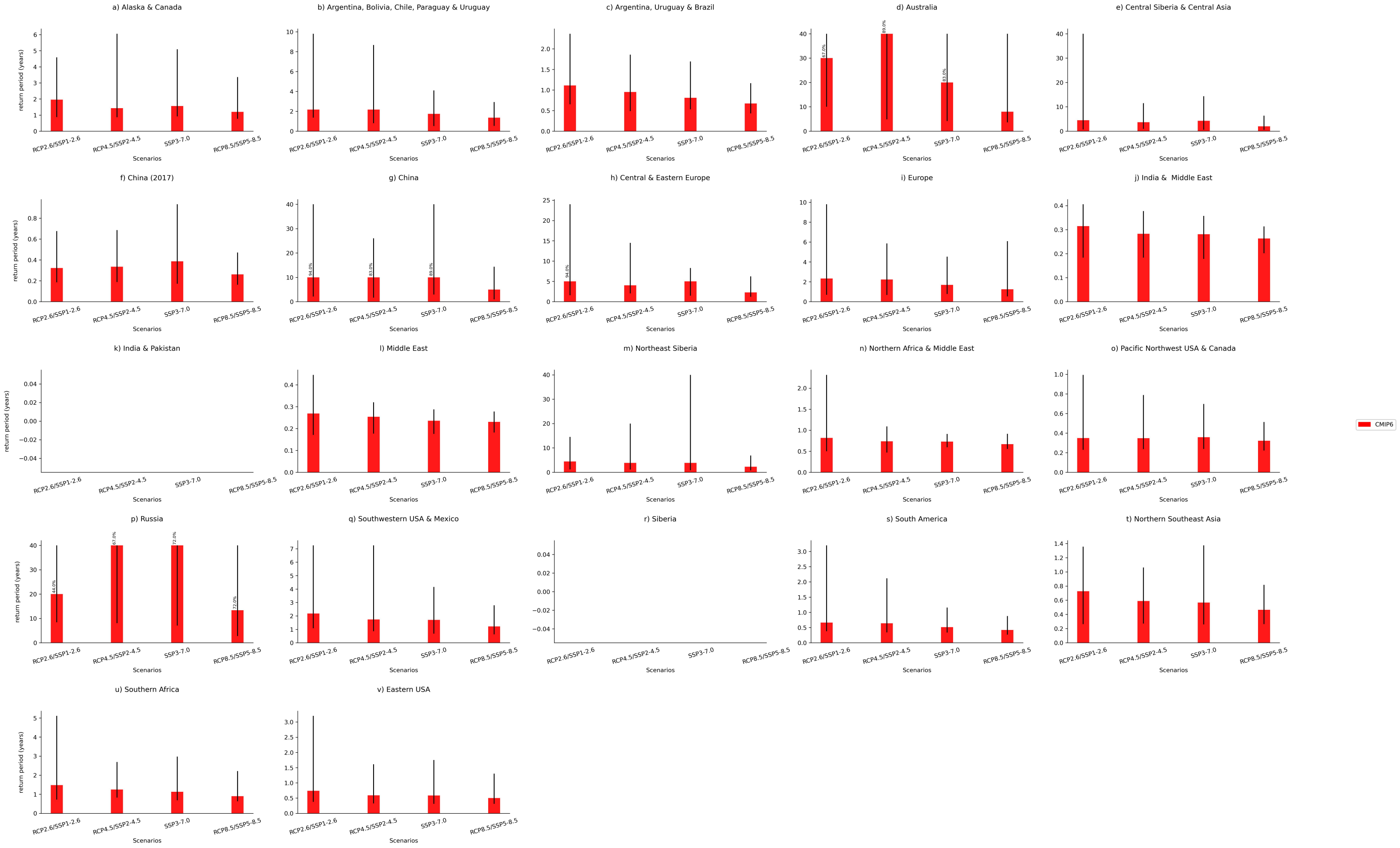

a) Alaska & Canada
b) Argentina, Bolivia, Chile, Paraguay & Uruguay
c) Argentina, Uruguay & Brazil
d) Australia
e) Central Siberia & Central Asia
f) China (2017)
g) China
h) Central & Eastern Europe
i) Europe
j) India & Middle East
k) India & Pakistan
l) Middle East
m) Northeast Siberia
n) Northern Africa & Middle East
o) Pacific Northwest USA & Canada
p) Russia
q) Southwestern USA & Mexico
r) Siberia
s) South America
t) Northern Southeast Asia
u) Southern Africa
v) Eastern USA
return period (years)
Scenarios
RCP2.6/SSP1-2.6
RCP4.5/SSP2-4.5
SSP3-7.0
RCP8.5/SSP5-8.5
CMIP6

Supplementary Figure 356: Return times (RT) during end-of-century (2060-2099) based on ERA5 (Tx95.0p) - Return times (RT) (in years) during the end-of-century period between 2060 and 2099 for Cumulative Heat (°C) of the respective observed event. For each region, values are shown for the different scenarios (increasing in emissions from left to right for the CMIP5 (green), CMIP6 (red), CanESM5 (dark blue), and ACCESS-ESM1-5 (light blue) ensembles. The error bars indicate the 5th and 95th percentile of the respective sample. Where the respective ensemble contains models not able to simulate the magnitude of the respective observed event's Cumulative Heat (°C), the proportion of models that are able to simulate an event with a value of Cumulative Heat (°C) at least as high as the observed event is indicated by the percentage values above the individual bars. The observed reference values are calculated using data from ERA5.

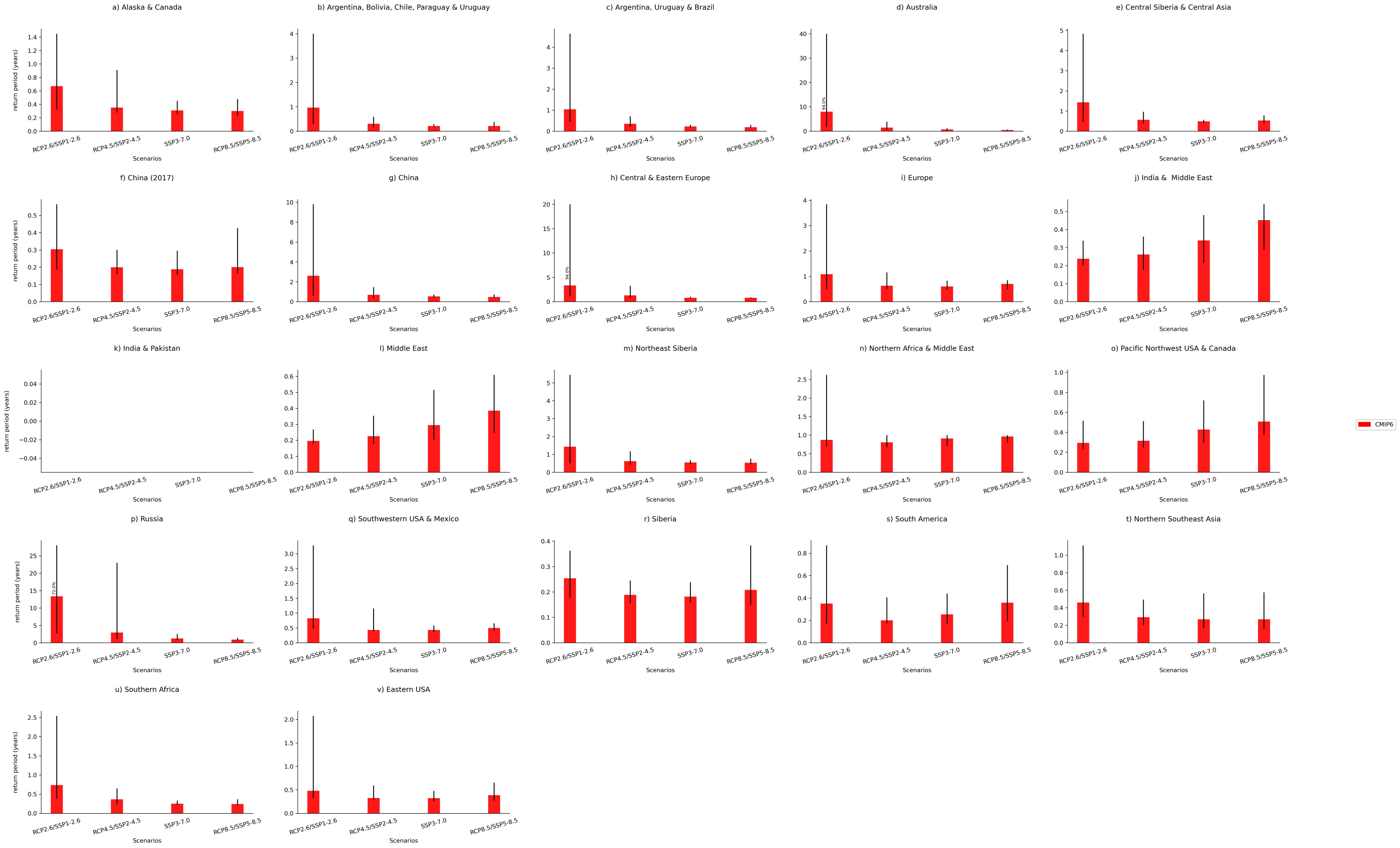
a) Alaska & Canada
b) Argentina, Bolivia, Chile, Paraguay & Uruguay
c) Argentina, Uruguay & Brazil
d) Australia
e) Central Siberia & Central Asia
f) China (2017)
g) China
h) Central & Eastern Europe
i) Europe
j) India & Middle East
k) India & Pakistan
l) Middle East
m) Northeast Siberia
n) Northern Africa & Middle East
o) Pacific Northwest USA & Canada
p) Russia
q) Southwestern USA & Mexico
r) Siberia
s) South America
t) Northern Southeast Asia
u) Southern Africa
v) Eastern USA
return period (years)
Scenarios
RCP2.6/SSP1-2.6
RCP4.5/SSP2-4.5
SSP3-7.0
RCP8.5/SSP5-8.5
94.0%
94.0%
72.0%
CMIP6

Supplementary Figure 357: Return times (RT) during end-of-century (2060-2099) based on ERA5 (Tx97.5p) - Return times (RT) (in years) during the end-of-century period between 2060 and 2099 for Cumulative Heat (°C) of the respective observed event. For each region, values are shown for the different scenarios (increasing in emissions from left to right for the CMIP5 (green), CMIP6 (red), CanESM5 (dark blue), and ACCESS-ESM1-5 (light blue) ensembles. The error bars indicate the 5th and 95th percentile of the respective sample. Where the respective ensemble contains models not able to simulate the magnitude of the respective observed event's Cumulative Heat (°C), the proportion of models that are able to simulate an event with a value of Cumulative Heat (°C) at least as high as the observed event is indicated by the percentage values above the individual bars. The observed reference values are calculated using data from ERA5.

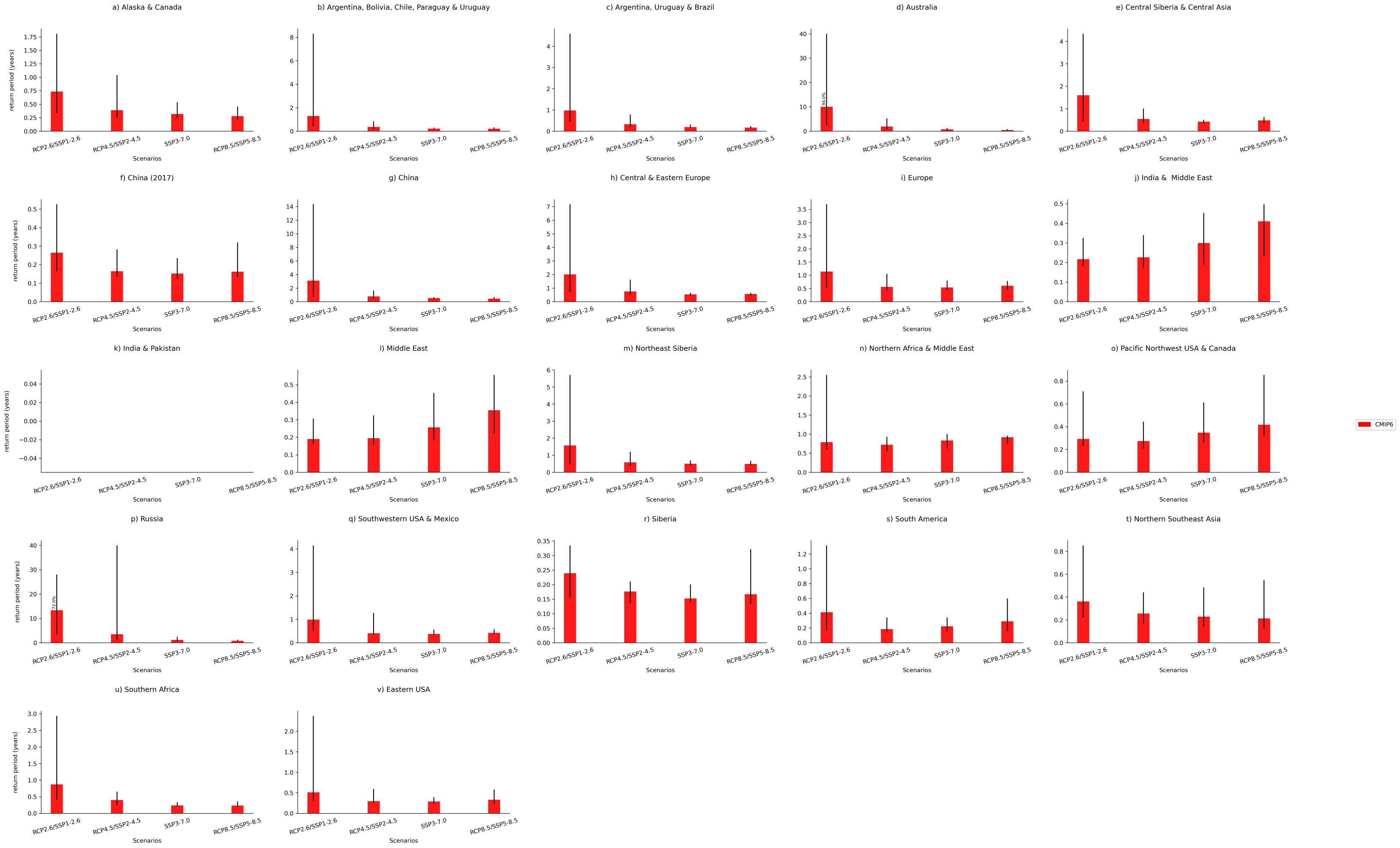
a) Alaska & Canada
b) Argentina, Bolivia, Chile, Paraguay & Uruguay
c) Argentina, Uruguay & Brazil
d) Australia
e) Central Siberia & Central Asia
f) China (2017)
g) China
h) Central & Eastern Europe
i) Europe
j) India & Middle East
k) India & Pakistan
l) Middle East
m) Northeast Siberia
n) Northern Africa & Middle East
o) Pacific Northwest USA & Canada
p) Russia
q) Southwestern USA & Mexico
r) Siberia
s) South America
t) Northern Southeast Asia
u) Southern Africa
v) Eastern USA
return period (years)
Scenarios
RCP2.6/SSP1-2.6
RCP4.5/SSP2-4.5
SSP3-7.0
RCP8.5/SSP5-8.5
94.0%
72.0%
CMIP6

Supplementary Figure 358: Return times (RT) during end-of-century (2060-2099) based on ERA5 (Tx99.0p) - Return times (RT) (in years) during the end-of-century period between 2060 and 2099 for Cumulative Heat (°C) of the respective observed event. For each region, values are shown for the different scenarios (increasing in emissions from left to right for the CMIP5 (green), CMIP6 (red), CanESM5 (dark blue), and ACCESS-ESM1-5 (light blue) ensembles. The error bars indicate the 5th and 95th percentile of the respective sample. Where the respective ensemble contains models not able to simulate the magnitude of the respective observed event's Cumulative Heat (°C), the proportion of models that are able to simulate an event with a value of Cumulative Heat (°C) at least as high as the observed event is indicated by the percentage values above the individual bars. The observed reference values are calculated using data from ERA5.

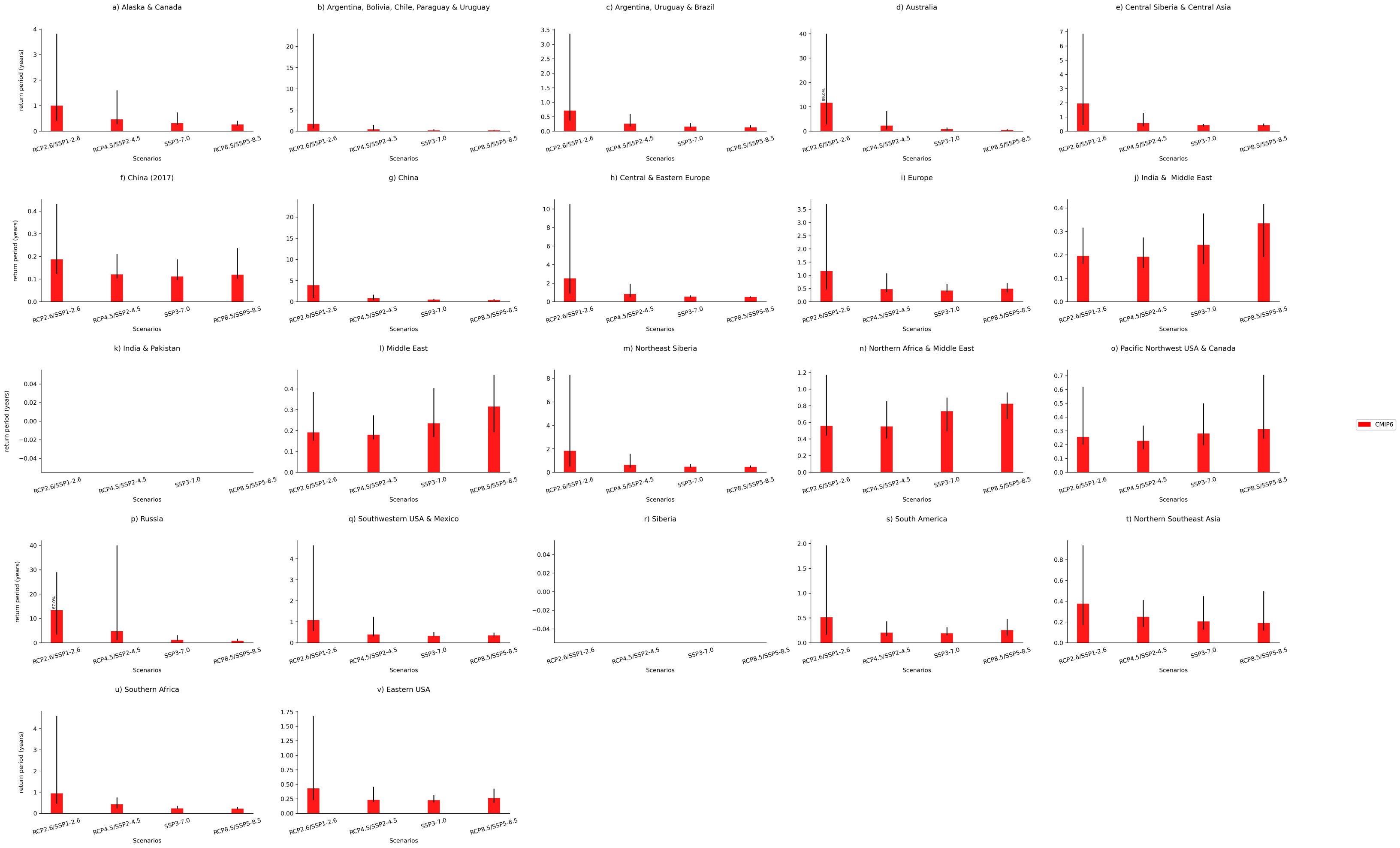

a) Alaska & Canada
b) Argentina, Bolivia, Chile, Paraguay & Uruguay
c) Argentina, Uruguay & Brazil
d) Australia
e) Central Siberia & Central Asia
f) China (2017)
g) China
h) Central & Eastern Europe
i) Europe
j) India & Middle East
k) India & Pakistan
l) Middle East
m) Northeast Siberia
n) Northern Africa & Middle East
o) Pacific Northwest USA & Canada
p) Russia
q) Southwestern USA & Mexico
r) Siberia
s) South America
t) Northern Southeast Asia
u) Southern Africa
v) Eastern USA
return period (years)
Scenarios
RCP2.6/SSP1-2.6
RCP4.5/SSP2-4.5
SSP3-7.0
RCP8.5/SSP5-8.5
89.0%
67.0%
CMIP6

Supplementary Figure 359: Return times (RT) during historical (1951-2005) based on Berkeley Earth (Tx95.0p) - Return times (RT) (in years) during the historical period between 1951 and 2005 for Heatwave Length (days) of the respective observed event. For each region, values are shown for the different scenarios (increasing in emissions from left to right for the CMIP5 (green), CMIP6 (red), CanESM5 (dark blue), and ACCESS-ESM1-5 (light blue) ensembles. The error bars indicate the 5th and 95th percentile of the respective sample. Where the respective ensemble contains models not able to simulate the magnitude of the respective observed event's Heatwave Length (days), the proportion of models that are able to simulate an event with a value of Heatwave Length (days) at least as high as the observed event is indicated by the percentage values above the individual bars. The observed reference values are calculated using data from Berkeley Earth.

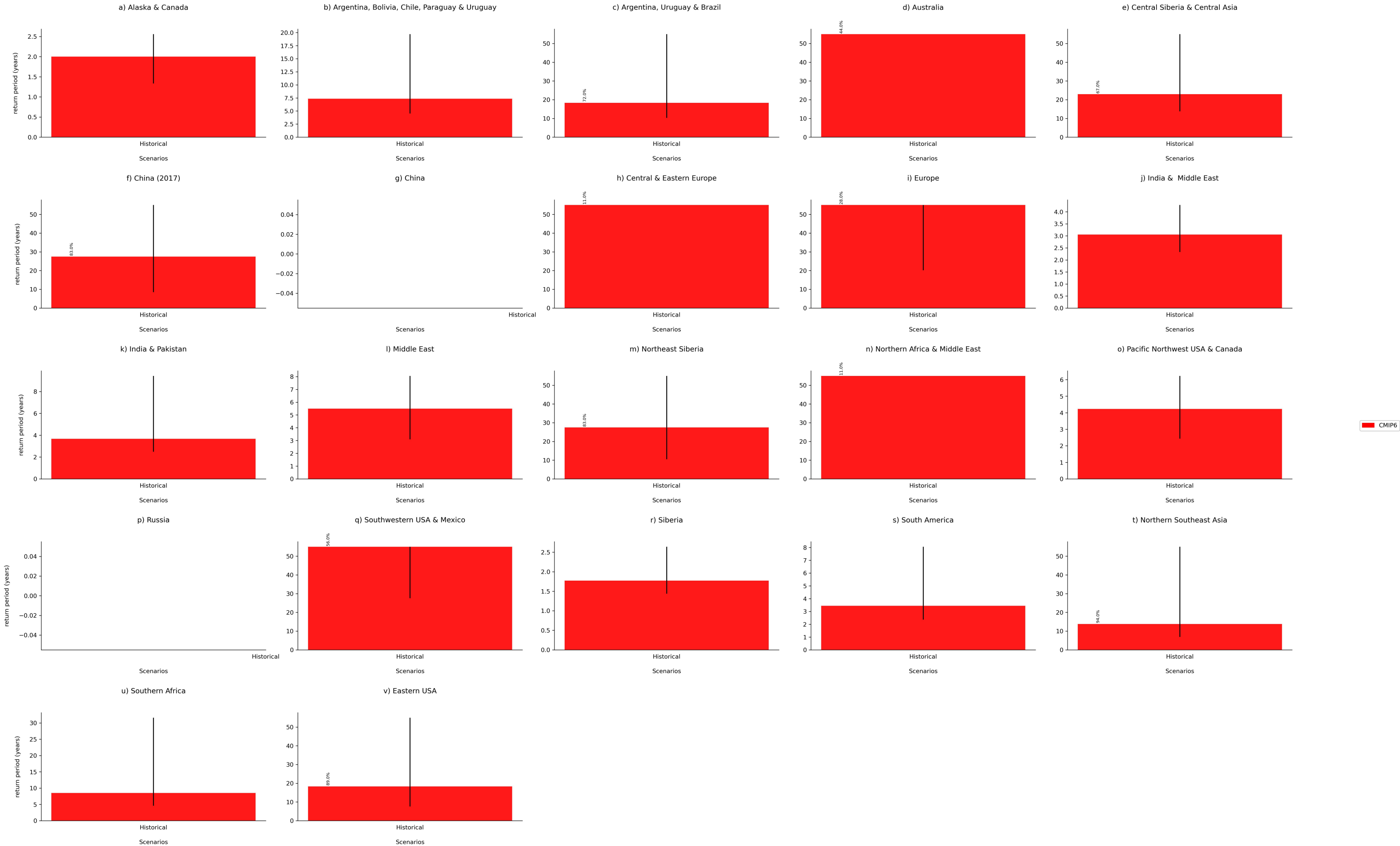
a) Alaska & Canada
b) Argentina, Bolivia, Chile, Paraguay & Uruguay
c) Argentina, Uruguay & Brazil
72.0%
d) Australia
44.0%
e) Central Siberia & Central Asia
67.0%
f) China (2017)
83.0%
g) China
h) Central & Eastern Europe
11.0%
i) Europe
28.0%
j) India & Middle East
k) India & Pakistan
l) Middle East
m) Northeast Siberia
83.0%
n) Northern Africa & Middle East
11.0%
o) Pacific Northwest USA & Canada
p) Russia
q) Southwestern USA & Mexico
56.0%
r) Siberia
s) South America
t) Northern Southeast Asia
94.0%
u) Southern Africa
v) Eastern USA
89.0%
return period (years)
Historical
Scenarios
CMIP6

Supplementary Figure 360: Return times (RT) during historical (1951-2005) based on Berkeley Earth (Tx97.5p) - Return times (RT) (in years) during the historical period between 1951 and 2005 for Heatwave Length (days) of the respective observed event. For each region, values are shown for the different scenarios (increasing in emissions from left to right for the CMIP5 (green), CMIP6 (red), CanESM5 (dark blue), and ACCESS-ESM1-5 (light blue) ensembles. The error bars indicate the 5th and 95th percentile of the respective sample. Where the respective ensemble contains models not able to simulate the magnitude of the respective observed event's Heatwave Length (days), the proportion of models that are able to simulate an event with a value of Heatwave Length (days) at least as high as the observed event is indicated by the percentage values above the individual bars. The observed reference values are calculated using data from Berkeley Earth.

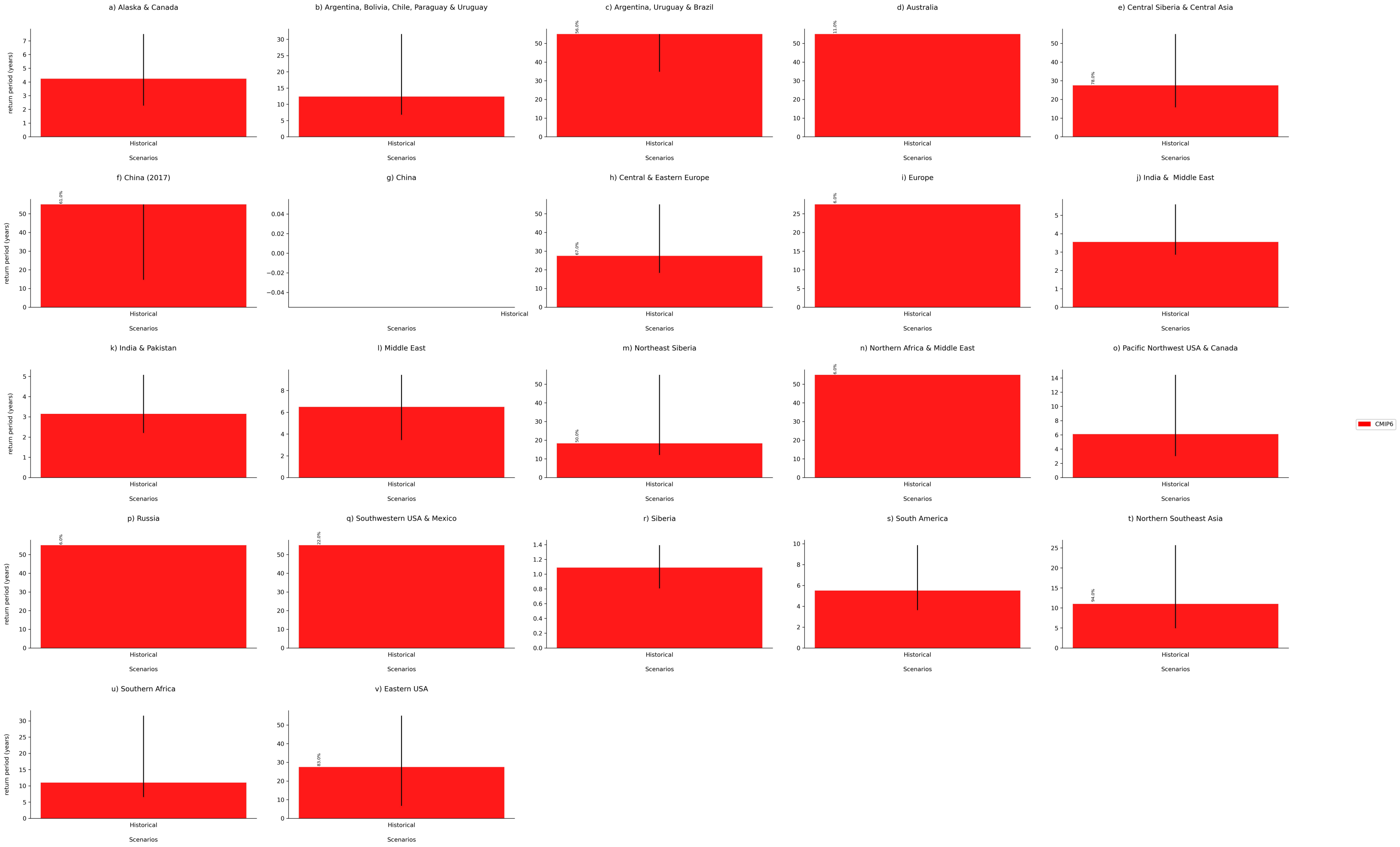
a) Alaska & Canada
b) Argentina, Bolivia, Chile, Paraguay & Uruguay
c) Argentina, Uruguay & Brazil
d) Australia
e) Central Siberia & Central Asia
f) China (2017)
g) China
h) Central & Eastern Europe
i) Europe
j) India & Middle East
k) India & Pakistan
l) Middle East
m) Northeast Siberia
n) Northern Africa & Middle East
o) Pacific Northwest USA & Canada
p) Russia
q) Southwestern USA & Mexico
r) Siberia
s) South America
t) Northern Southeast Asia
u) Southern Africa
v) Eastern USA
return period (years)
Historical
Scenarios
CMIP6
56.0%
11.0%
78.0%
61.0%
67.0%
6.0%
50.0%
6.0%
6.0%
22.0%
94.0%
83.0%

Supplementary Figure 361: Return times (RT) during historical (1951-2005) based on Berkeley Earth (Tx99.0p) - Return times (RT) (in years) during the historical period between 1951 and 2005 for Heatwave Length (days) of the respective observed event. For each region, values are shown for the different scenarios (increasing in emissions from left to right for the CMIP5 (green), CMIP6 (red), CanESM5 (dark blue), and ACCESS-ESM1-5 (light blue) ensembles. The error bars indicate the 5th and 95th percentile of the respective sample. Where the respective ensemble contains models not able to simulate the magnitude of the respective observed event's Heatwave Length (days), the proportion of models that are able to simulate an event with a value of Heatwave Length (days) at least as high as the observed event is indicated by the percentage values above the individual bars. The observed reference values are calculated using data from Berkeley Earth.

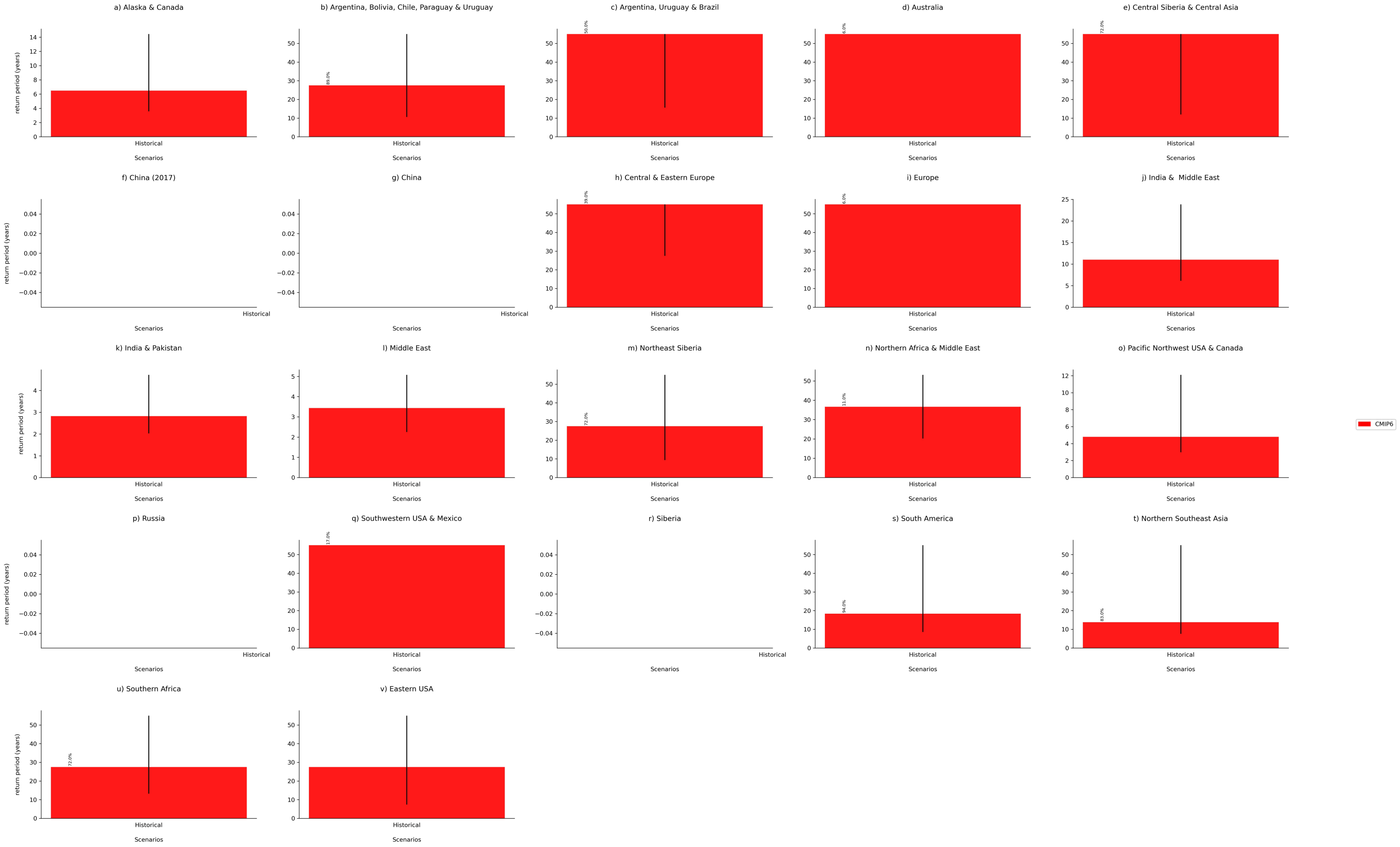
a) Alaska & Canada
b) Argentina, Bolivia, Chile, Paraguay & Uruguay
89.0%
c) Argentina, Uruguay & Brazil
50.0%
d) Australia
6.0%
e) Central Siberia & Central Asia
72.0%
f) China (2017)
g) China
h) Central & Eastern Europe
39.0%
i) Europe
6.0%
j) India & Middle East
k) India & Pakistan
l) Middle East
m) Northeast Siberia
72.0%
n) Northern Africa & Middle East
11.0%
o) Pacific Northwest USA & Canada
p) Russia
q) Southwestern USA & Mexico
17.0%
r) Siberia
s) South America
94.0%
t) Northern Southeast Asia
83.0%
u) Southern Africa
72.0%
v) Eastern USA
return period (years)
Historical
Scenarios
CMIP6

Supplementary Figure 362: Return times (RT) during present-to-near-future (2015-2054) based on Berkeley Earth (Tx95.0p) - Return times (RT) (in years) during the present-to-near-future period between 2015 and 2054 for Heatwave Length (days) of the respective observed event. For each region, values are shown for the different scenarios (increasing in emissions from left to right for the CMIP5 (green), CMIP6 (red), CanESM5 (dark blue), and ACCESS-ESM1-5 (light blue) ensembles. The error bars indicate the 5th and 95th percentile of the respective sample. Where the respective ensemble contains models not able to simulate the magnitude of the respective observed event's Heatwave Length (days), the proportion of models that are able to simulate an event with a value of Heatwave Length (days) at least as high as the observed event is indicated by the percentage values above the individual bars. The observed reference values are calculated using data from Berkeley Earth.

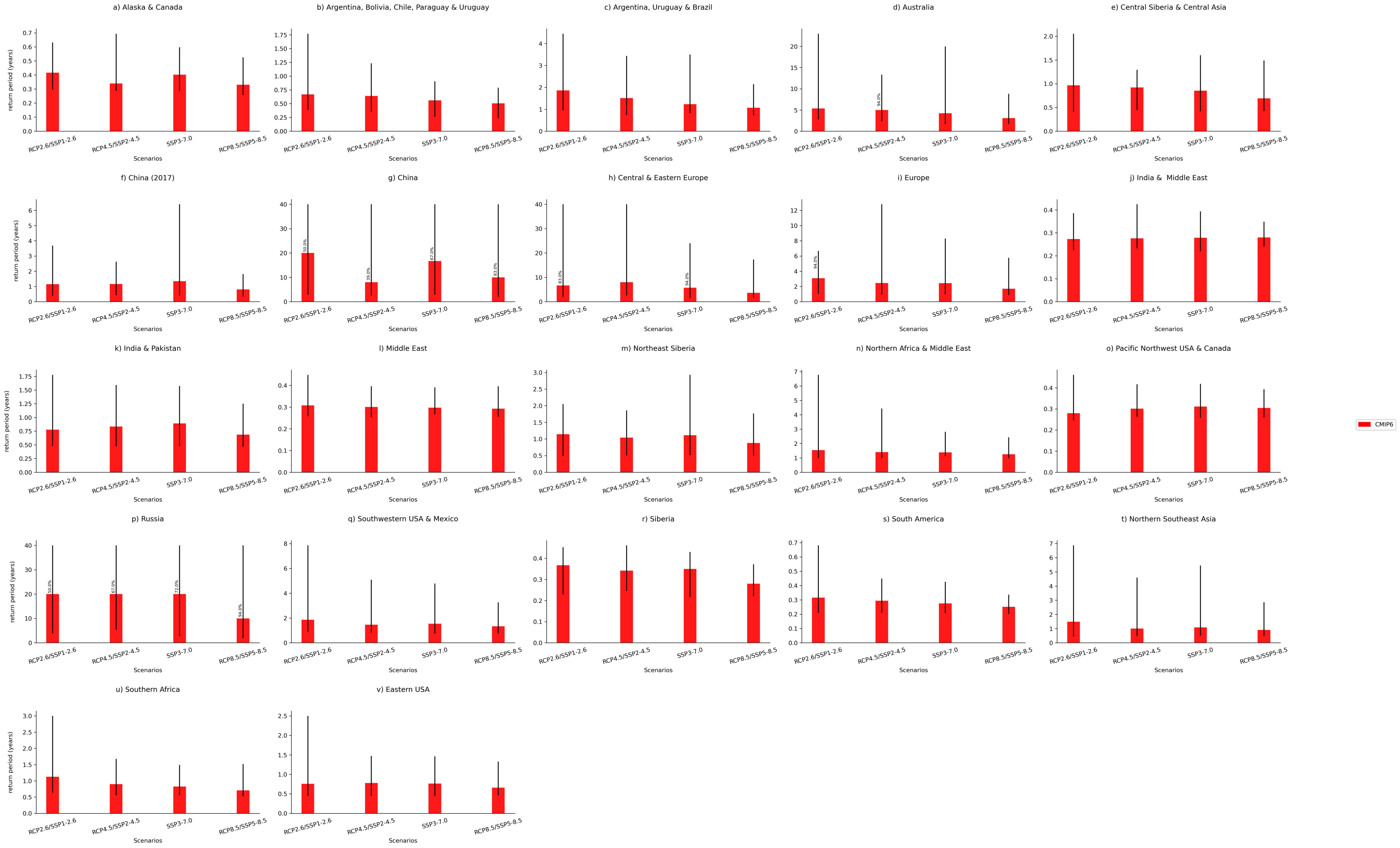
a) Alaska & Canada
b) Argentina, Bolivia, Chile, Paraguay & Uruguay
c) Argentina, Uruguay & Brazil
d) Australia
e) Central Siberia & Central Asia
f) China (2017)
g) China
h) Central & Eastern Europe
i) Europe
j) India & Middle East
k) India & Pakistan
l) Middle East
m) Northeast Siberia
n) Northern Africa & Middle East
o) Pacific Northwest USA & Canada
p) Russia
q) Southwestern USA & Mexico
r) Siberia
s) South America
t) Northern Southeast Asia
u) Southern Africa
v) Eastern USA
return period (years)
Scenarios
RCP2.6/SSP1-2.6
RCP4.5/SSP2-4.5
SSP3-7.0
RCP8.5/SSP5-8.5
94.0%
50.0%
39.0%
67.0%
83.0%
83.0%
94.0%
94.0%
50.0%
67.0%
72.0%
94.0%
CMIP6

Supplementary Figure 363: Return times (RT) during present-to-near-future (2015-2054) based on Berkeley Earth (Tx97.5p) - Return times (RT) (in years) during the present-to-near-future period between 2015 and 2054 for Heatwave Length (days) of the respective observed event. For each region, values are shown for the different scenarios (increasing in emissions from left to right for the CMIP5 (green), CMIP6 (red), CanESM5 (dark blue), and ACCESS-ESM1-5 (light blue) ensembles. The error bars indicate the 5th and 95th percentile of the respective sample. Where the respective ensemble contains models not able to simulate the magnitude of the respective observed event's Heatwave Length (days), the proportion of models that are able to simulate an event with a value of Heatwave Length (days) at least as high as the observed event is indicated by the percentage values above the individual bars. The observed reference values are calculated using data from Berkeley Earth.

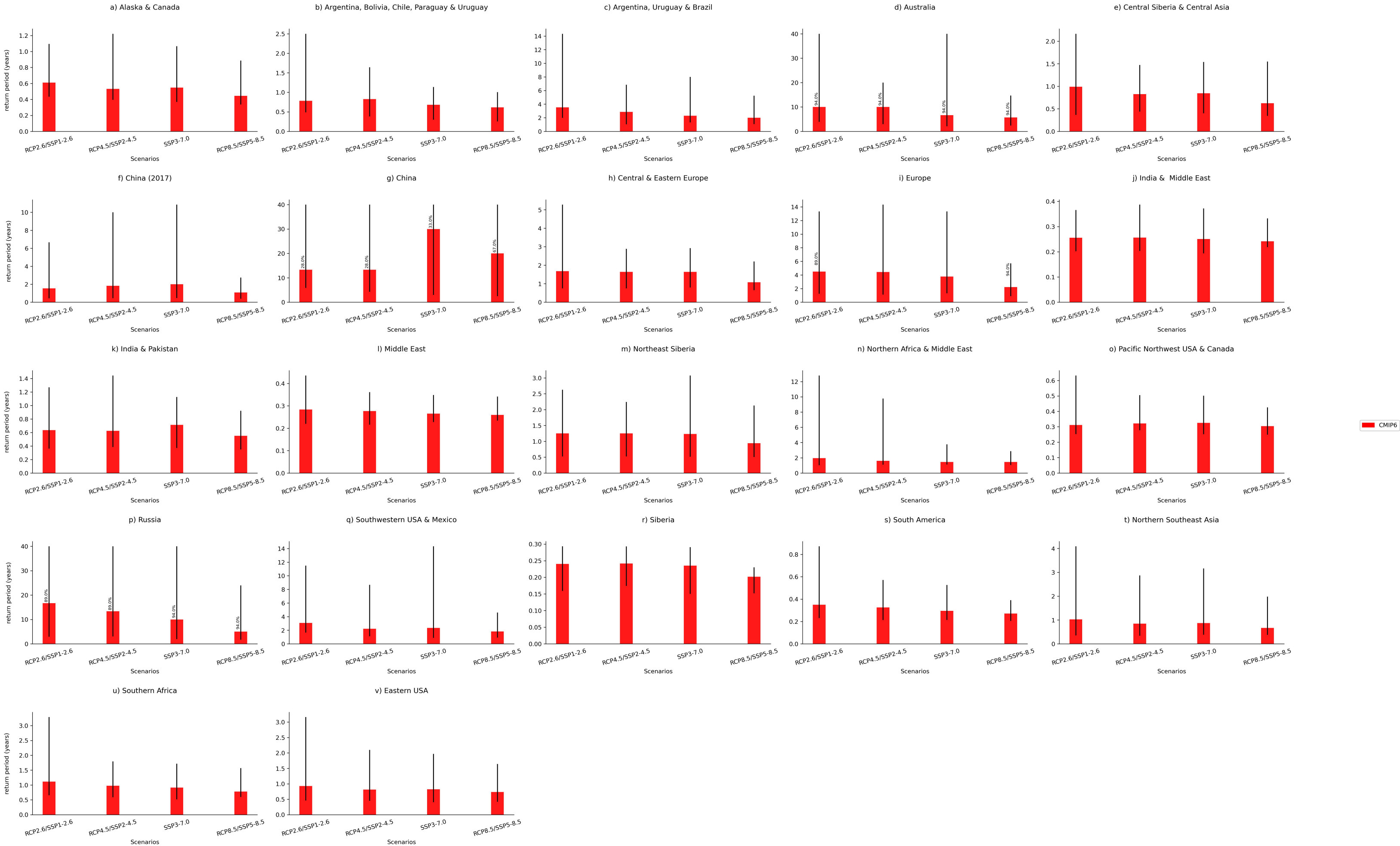
a) Alaska & Canada
b) Argentina, Bolivia, Chile, Paraguay & Uruguay
c) Argentina, Uruguay & Brazil
d) Australia
e) Central Siberia & Central Asia
f) China (2017)
g) China
h) Central & Eastern Europe
i) Europe
j) India & Middle East
k) India & Pakistan
l) Middle East
m) Northeast Siberia
n) Northern Africa & Middle East
o) Pacific Northwest USA & Canada
p) Russia
q) Southwestern USA & Mexico
r) Siberia
s) South America
t) Northern Southeast Asia
u) Southern Africa
v) Eastern USA
return period (years)
Scenarios
RCP2.6/SSP1-2.6
RCP4.5/SSP2-4.5
SSP3-7.0
RCP8.5/SSP5-8.5
94.0%
28.0%
33.0%
67.0%
89.0%
CMIP6

Supplementary Figure 364: Return times (RT) during present-to-near-future (2015-2054) based on Berkeley Earth (Tx99.0p) - Return times (RT) (in years) during the present-to-near-future period between 2015 and 2054 for Heatwave Length (days) of the respective observed event. For each region, values are shown for the different scenarios (increasing in emissions from left to right for the CMIP5 (green), CMIP6 (red), CanESM5 (dark blue), and ACCESS-ESM1-5 (light blue) ensembles. The error bars indicate the 5th and 95th percentile of the respective sample. Where the respective ensemble contains models not able to simulate the magnitude of the respective observed event's Heatwave Length (days), the proportion of models that are able to simulate an event with a value of Heatwave Length (days) at least as high as the observed event is indicated by the percentage values above the individual bars. The observed reference values are calculated using data from Berkeley Earth.

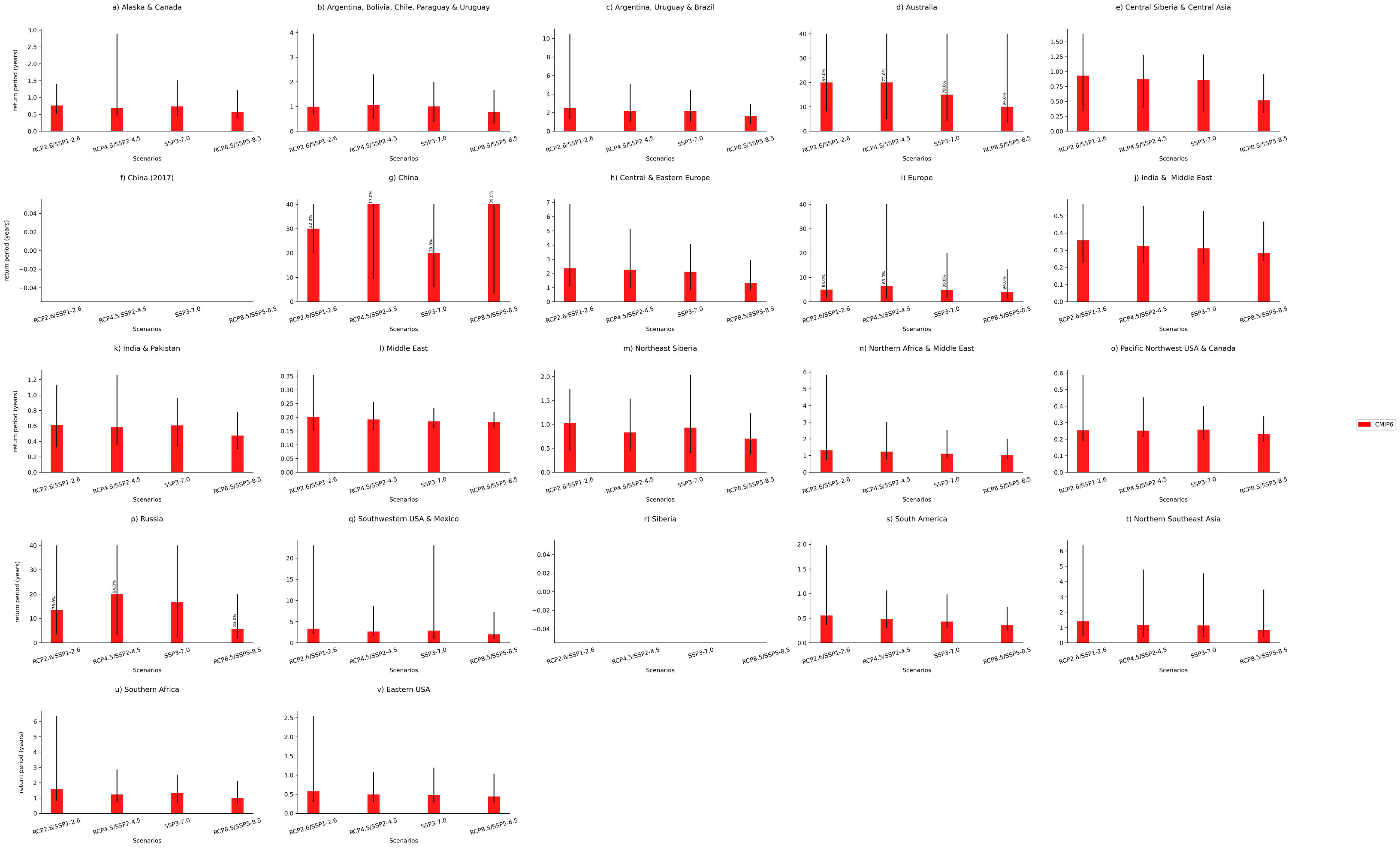
a) Alaska & Canada
b) Argentina, Bolivia, Chile, Paraguay & Uruguay
c) Argentina, Uruguay & Brazil
d) Australia
67.0%
72.0%
78.0%
94.0%
e) Central Siberia & Central Asia
f) China (2017)
g) China
22.0%
17.0%
28.0%
39.0%
h) Central & Eastern Europe
i) Europe
83.0%
89.0%
89.0%
94.0%
j) India & Middle East
k) India & Pakistan
l) Middle East
m) Northeast Siberia
n) Northern Africa & Middle East
o) Pacific Northwest USA & Canada
p) Russia
78.0%
94.0%
83.0%
q) Southwestern USA & Mexico
r) Siberia
s) South America
t) Northern Southeast Asia
u) Southern Africa
v) Eastern USA
return period (years)
Scenarios
RCP2.6/SSP1-2.6
RCP4.5/SSP2-4.5
SSP3-7.0
RCP8.5/SSP5-8.5
CMIP6

Supplementary Figure 365: Return times (RT) during end-of-century (2060-2099) based on Berkeley Earth (Tx95.0p) - Return times (RT) (in years) during the end-of-century period between 2060 and 2099 for Heatwave Length (days) of the respective observed event. For each region, values are shown for the different scenarios (increasing in emissions from left to right for the CMIP5 (green), CMIP6 (red), CanESM5 (dark blue), and ACCESS-ESM1-5 (light blue) ensembles. The error bars indicate the 5th and 95th percentile of the respective sample. Where the respective ensemble contains models not able to simulate the magnitude of the respective observed event's Heatwave Length (days), the proportion of models that are able to simulate an event with a value of Heatwave Length (days) at least as high as the observed event is indicated by the percentage values above the individual bars. The observed reference values are calculated using data from Berkeley Earth.

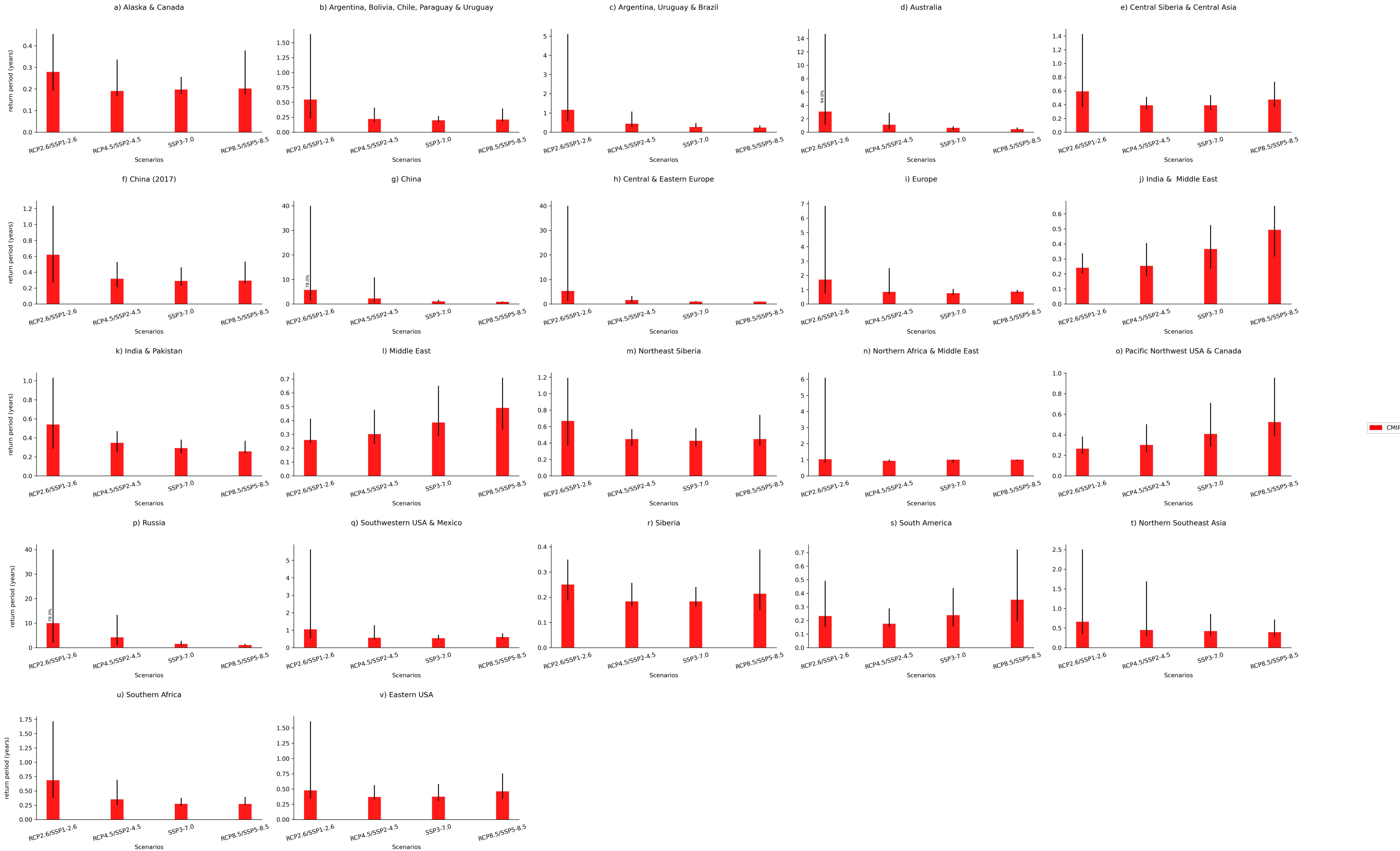
a) Alaska & Canada
b) Argentina, Bolivia, Chile, Paraguay & Uruguay
c) Argentina, Uruguay & Brazil
d) Australia
e) Central Siberia & Central Asia
f) China (2017)
g) China
h) Central & Eastern Europe
i) Europe
j) India & Middle East
k) India & Pakistan
l) Middle East
m) Northeast Siberia
n) Northern Africa & Middle East
o) Pacific Northwest USA & Canada
p) Russia
q) Southwestern USA & Mexico
r) Siberia
s) South America
t) Northern Southeast Asia
u) Southern Africa
v) Eastern USA
return period (years)
Scenarios
RCP2.6/SSP1-2.6
RCP4.5/SSP2-4.5
SSP3-7.0
RCP8.5/SSP5-8.5
94.0%
78.0%
78.0%
CMIP6

Supplementary Figure 366: Return times (RT) during end-of-century (2060-2099) based on Berkeley Earth (Tx97.5p) - Return times (RT) (in years) during the end-of-century period between 2060 and 2099 for Heatwave Length (days) of the respective observed event. For each region, values are shown for the different scenarios (increasing in emissions from left to right for the CMIP5 (green), CMIP6 (red), CanESM5 (dark blue), and ACCESS-ESM1-5 (light blue) ensembles. The error bars indicate the 5th and 95th percentile of the respective sample. Where the respective ensemble contains models not able to simulate the magnitude of the respective observed event's Heatwave Length (days), the proportion of models that are able to simulate an event with a value of Heatwave Length (days) at least as high as the observed event is indicated by the percentage values above the individual bars. The observed reference values are calculated using data from Berkeley Earth.

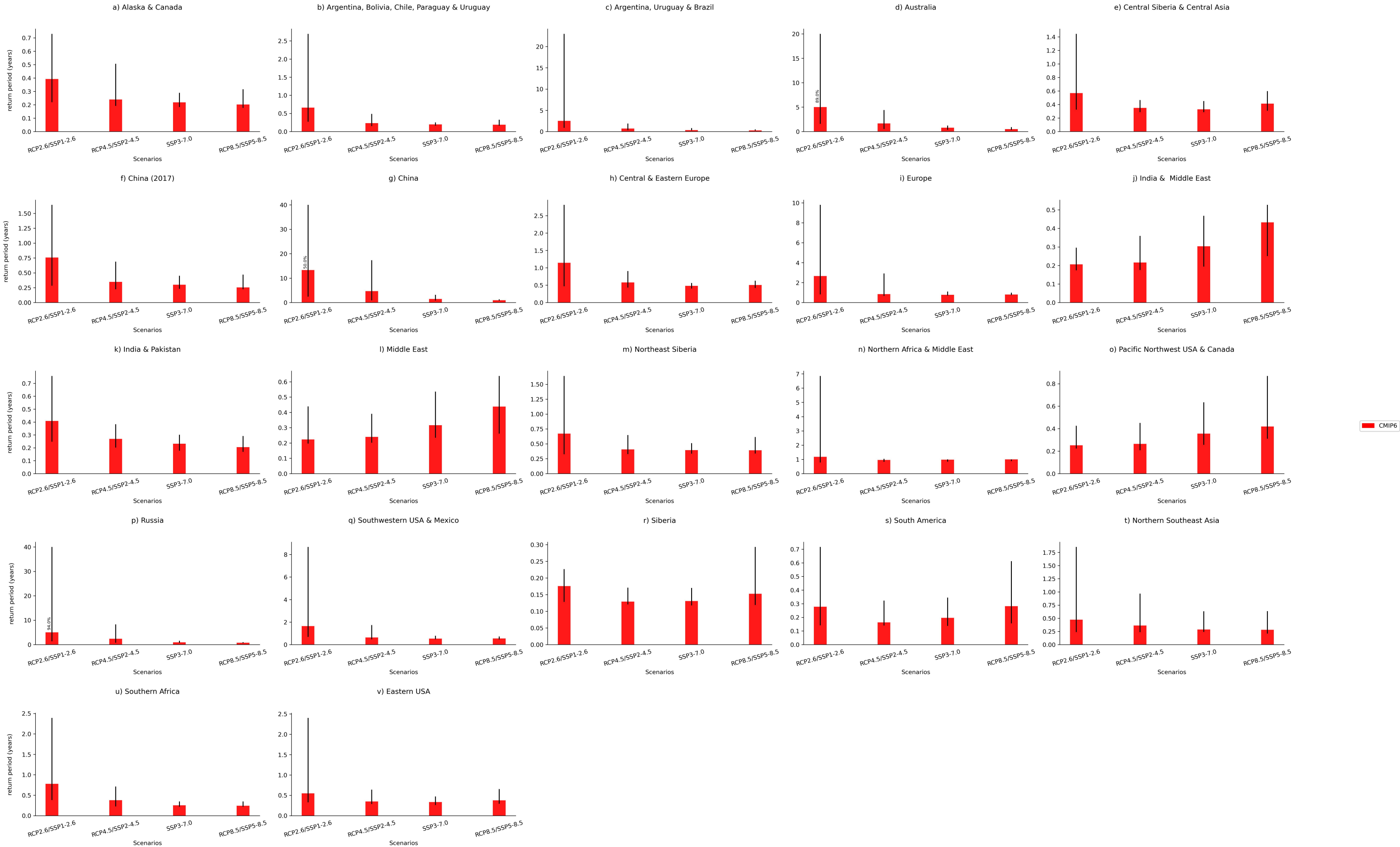

a) Alaska & Canada
b) Argentina, Bolivia, Chile, Paraguay & Uruguay
c) Argentina, Uruguay & Brazil
d) Australia
e) Central Siberia & Central Asia
f) China (2017)
g) China
h) Central & Eastern Europe
i) Europe
j) India & Middle East
k) India & Pakistan
l) Middle East
m) Northeast Siberia
n) Northern Africa & Middle East
o) Pacific Northwest USA & Canada
p) Russia
q) Southwestern USA & Mexico
r) Siberia
s) South America
t) Northern Southeast Asia
u) Southern Africa
v) Eastern USA
return period (years)
Scenarios
RCP2.6/SSP1-2.6
RCP4.5/SSP2-4.5
SSP3-7.0
RCP8.5/SSP5-8.5
89.0%
50.0%
94.0%
CMIP6

Supplementary Figure 367: Return times (RT) during end-of-century (2060-2099) based on Berkeley Earth (Tx99.0p) - Return times (RT) (in years) during the end-of-century period between 2060 and 2099 for Heatwave Length (days) of the respective observed event. For each region, values are shown for the different scenarios (increasing in emissions from left to right for the CMIP5 (green), CMIP6 (red), CanESM5 (dark blue), and ACCESS-ESM1-5 (light blue) ensembles. The error bars indicate the 5th and 95th percentile of the respective sample. Where the respective ensemble contains models not able to simulate the magnitude of the respective observed event's Heatwave Length (days), the proportion of models that are able to simulate an event with a value of Heatwave Length (days) at least as high as the observed event is indicated by the percentage values above the individual bars. The observed reference values are calculated using data from Berkeley Earth.

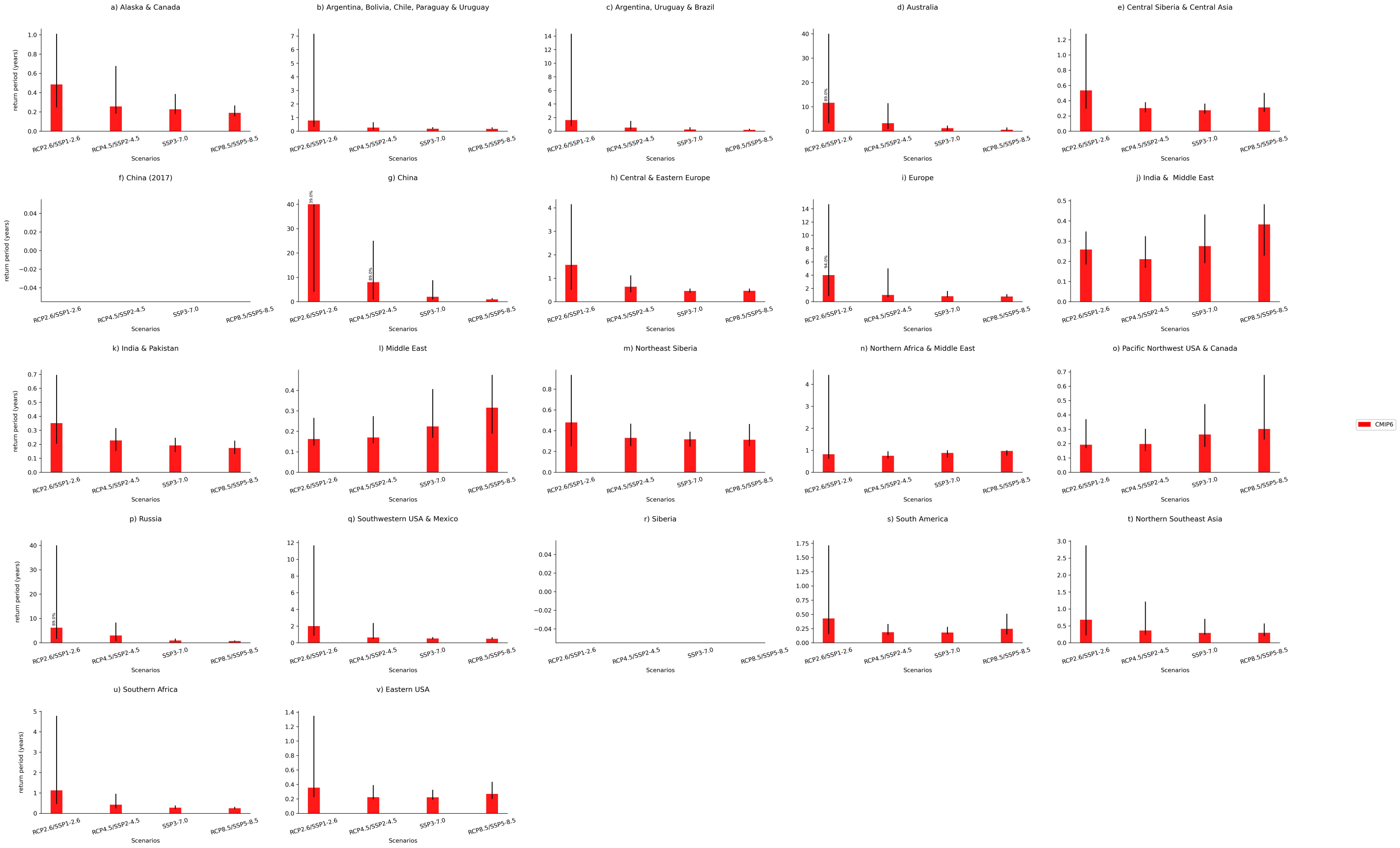

a) Alaska & Canada
b) Argentina, Bolivia, Chile, Paraguay & Uruguay
c) Argentina, Uruguay & Brazil
d) Australia
e) Central Siberia & Central Asia
f) China (2017)
g) China
h) Central & Eastern Europe
i) Europe
j) India & Middle East
k) India & Pakistan
l) Middle East
m) Northeast Siberia
n) Northern Africa & Middle East
o) Pacific Northwest USA & Canada
p) Russia
q) Southwestern USA & Mexico
r) Siberia
s) South America
t) Northern Southeast Asia
u) Southern Africa
v) Eastern USA
return period (years)
Scenarios
RCP2.6/SSP1-2.6
RCP4.5/SSP2-4.5
SSP3-7.0
RCP8.5/SSP5-8.5
89.0%
39.0%
94.0%
CMIP6

Supplementary Figure 368: Return times (RT) during historical (1951-2005) based on ERA5 (Tx95.0p) - Return times (RT) (in years) during the historical period between 1951 and 2005 for Heatwave Length (days) of the respective observed event. For each region, values are shown for the different scenarios (increasing in emissions from left to right for the CMIP5 (green), CMIP6 (red), CanESM5 (dark blue), and ACCESS-ESM1-5 (light blue) ensembles. The error bars indicate the 5th and 95th percentile of the respective sample. Where the respective ensemble contains models not able to simulate the magnitude of the respective observed event's Heatwave Length (days), the proportion of models that are able to simulate an event with a value of Heatwave Length (days) at least as high as the observed event is indicated by the percentage values above the individual bars. The observed reference values are calculated using data from ERA5.

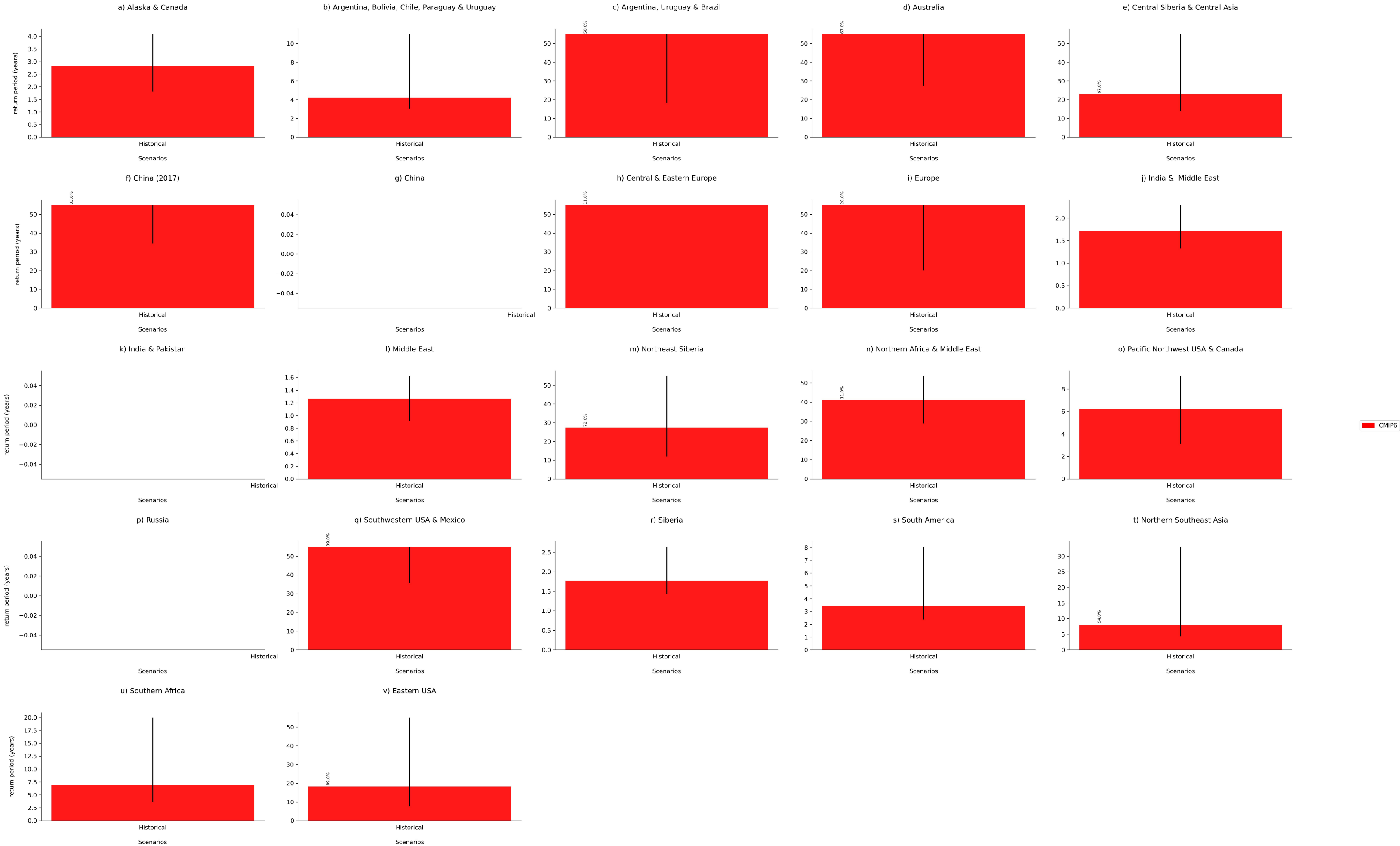
a) Alaska & Canada
b) Argentina, Bolivia, Chile, Paraguay & Uruguay
c) Argentina, Uruguay & Brazil
50.0%
d) Australia
67.0%
e) Central Siberia & Central Asia
67.0%
f) China (2017)
33.0%
g) China
h) Central & Eastern Europe
11.0%
i) Europe
28.0%
j) India & Middle East
k) India & Pakistan
l) Middle East
m) Northeast Siberia
72.0%
n) Northern Africa & Middle East
11.0%
o) Pacific Northwest USA & Canada
p) Russia
q) Southwestern USA & Mexico
39.0%
r) Siberia
s) South America
t) Northern Southeast Asia
94.0%
u) Southern Africa
v) Eastern USA
89.0%
return period (years)
Historical
Scenarios
CMIP6

Supplementary Figure 369: Return times (RT) during historical (1951-2005) based on ERA5 (Tx97.5p) - Return times (RT) (in years) during the historical period between 1951 and 2005 for Heatwave Length (days) of the respective observed event. For each region, values are shown for the different scenarios (increasing in emissions from left to right for the CMIP5 (green), CMIP6 (red), CanESM5 (dark blue), and ACCESS-ESM1-5 (light blue) ensembles. The error bars indicate the 5th and 95th percentile of the respective sample. Where the respective ensemble contains models not able to simulate the magnitude of the respective observed event's Heatwave Length (days), the proportion of models that are able to simulate an event with a value of Heatwave Length (days) at least as high as the observed event is indicated by the percentage values above the individual bars. The observed reference values are calculated using data from ERA5.

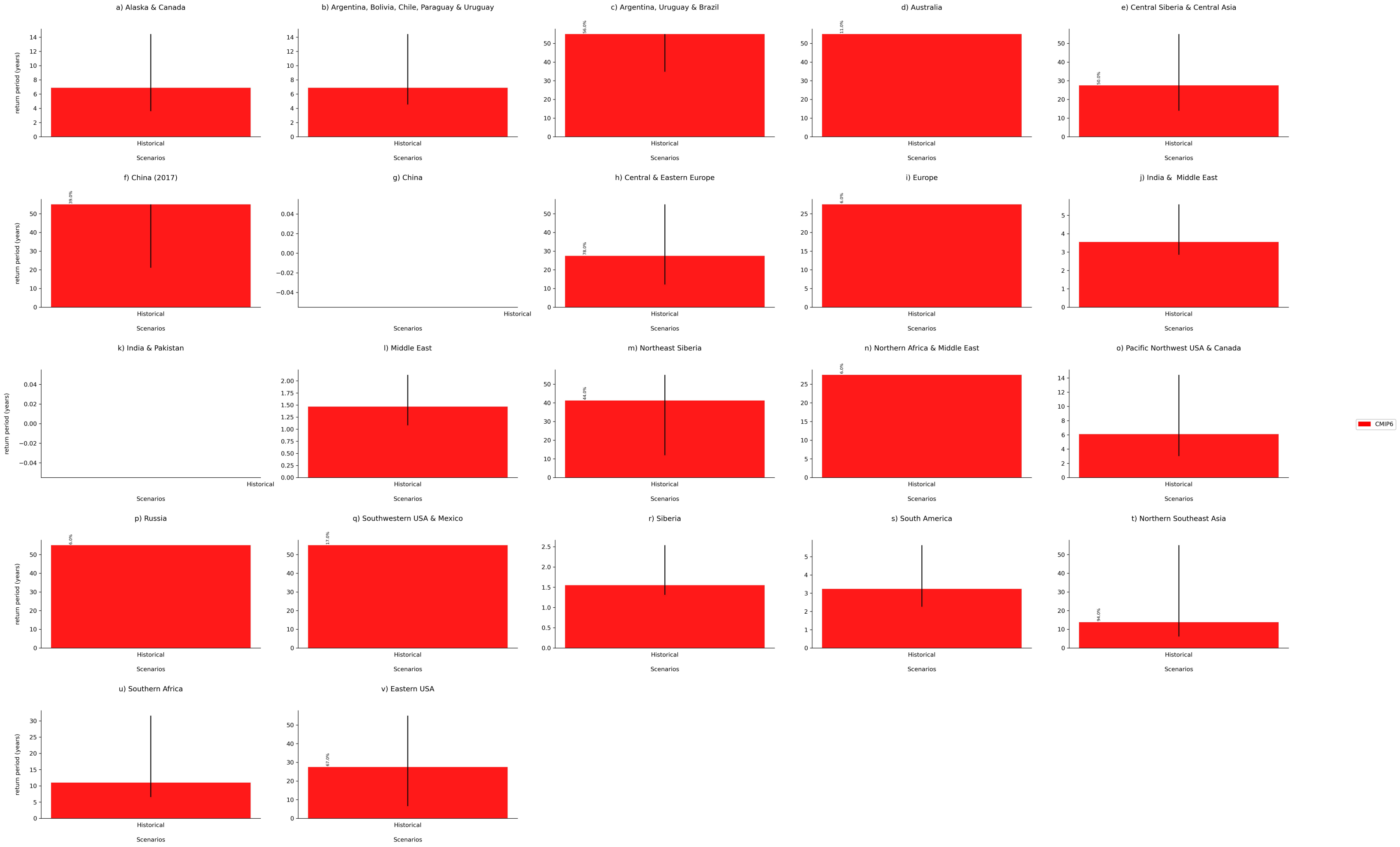

a) Alaska & Canada
b) Argentina, Bolivia, Chile, Paraguay & Uruguay
c) Argentina, Uruguay & Brazil
56.0%
d) Australia
11.0%
e) Central Siberia & Central Asia
50.0%
f) China (2017)
39.0%
g) China
h) Central & Eastern Europe
78.0%
i) Europe
6.0%
j) India & Middle East
k) India & Pakistan
l) Middle East
m) Northeast Siberia
44.0%
n) Northern Africa & Middle East
6.0%
o) Pacific Northwest USA & Canada
p) Russia
6.0%
q) Southwestern USA & Mexico
17.0%
r) Siberia
s) South America
t) Northern Southeast Asia
94.0%
u) Southern Africa
v) Eastern USA
67.0%
return period (years)
Historical
Scenarios
CMIP6

Supplementary Figure 370: Return times (RT) during historical (1951-2005) based on ERA5 (Tx99.0p) - Return times (RT) (in years) during the historical period between 1951 and 2005 for Heatwave Length (days) of the respective observed event. For each region, values are shown for the different scenarios (increasing in emissions from left to right for the CMIP5 (green), CMIP6 (red), CanESM5 (dark blue), and ACCESS-ESM1-5 (light blue) ensembles. The error bars indicate the 5th and 95th percentile of the respective sample. Where the respective ensemble contains models not able to simulate the magnitude of the respective observed event's Heatwave Length (days), the proportion of models that are able to simulate an event with a value of Heatwave Length (days) at least as high as the observed event is indicated by the percentage values above the individual bars. The observed reference values are calculated using data from ERA5.

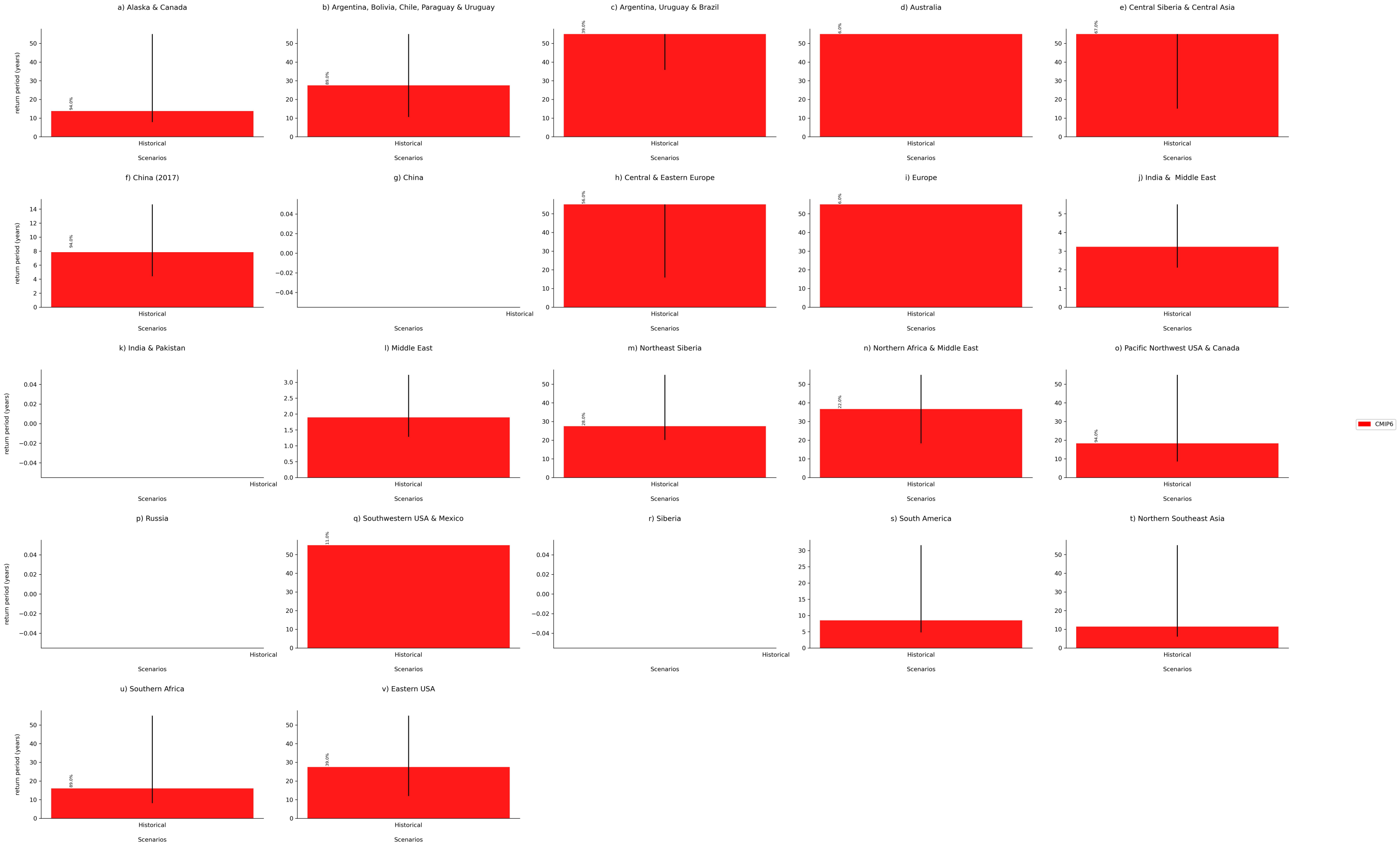
a) Alaska & Canada
b) Argentina, Bolivia, Chile, Paraguay & Uruguay
c) Argentina, Uruguay & Brazil
d) Australia
e) Central Siberia & Central Asia
f) China (2017)
g) China
h) Central & Eastern Europe
i) Europe
j) India & Middle East
k) India & Pakistan
l) Middle East
m) Northeast Siberia
n) Northern Africa & Middle East
o) Pacific Northwest USA & Canada
p) Russia
q) Southwestern USA & Mexico
r) Siberia
s) South America
t) Northern Southeast Asia
u) Southern Africa
v) Eastern USA
return period (years)
Historical
Scenarios
CMIP6
94.0%
89.0%
39.0%
6.0%
67.0%
94.0%
56.0%
6.0%
28.0%
22.0%
94.0%
11.0%
89.0%
39.0%

Supplementary Figure 371: Return times (RT) during present-to-near-future (2015-2054) based on ERA5 (Tx95.0p) - Return times (RT) (in years) during the present-to-near-future period between 2015 and 2054 for Heatwave Length (days) of the respective observed event. For each region, values are shown for the different scenarios (increasing in emissions from left to right for the CMIP5 (green), CMIP6 (red), CanESM5 (dark blue), and ACCESS-ESM1-5 (light blue) ensembles. The error bars indicate the 5th and 95th percentile of the respective sample. Where the respective ensemble contains models not able to simulate the magnitude of the respective observed event's Heatwave Length (days), the proportion of models that are able to simulate an event with a value of Heatwave Length (days) at least as high as the observed event is indicated by the percentage values above the individual bars. The observed reference values are calculated using data from ERA5.

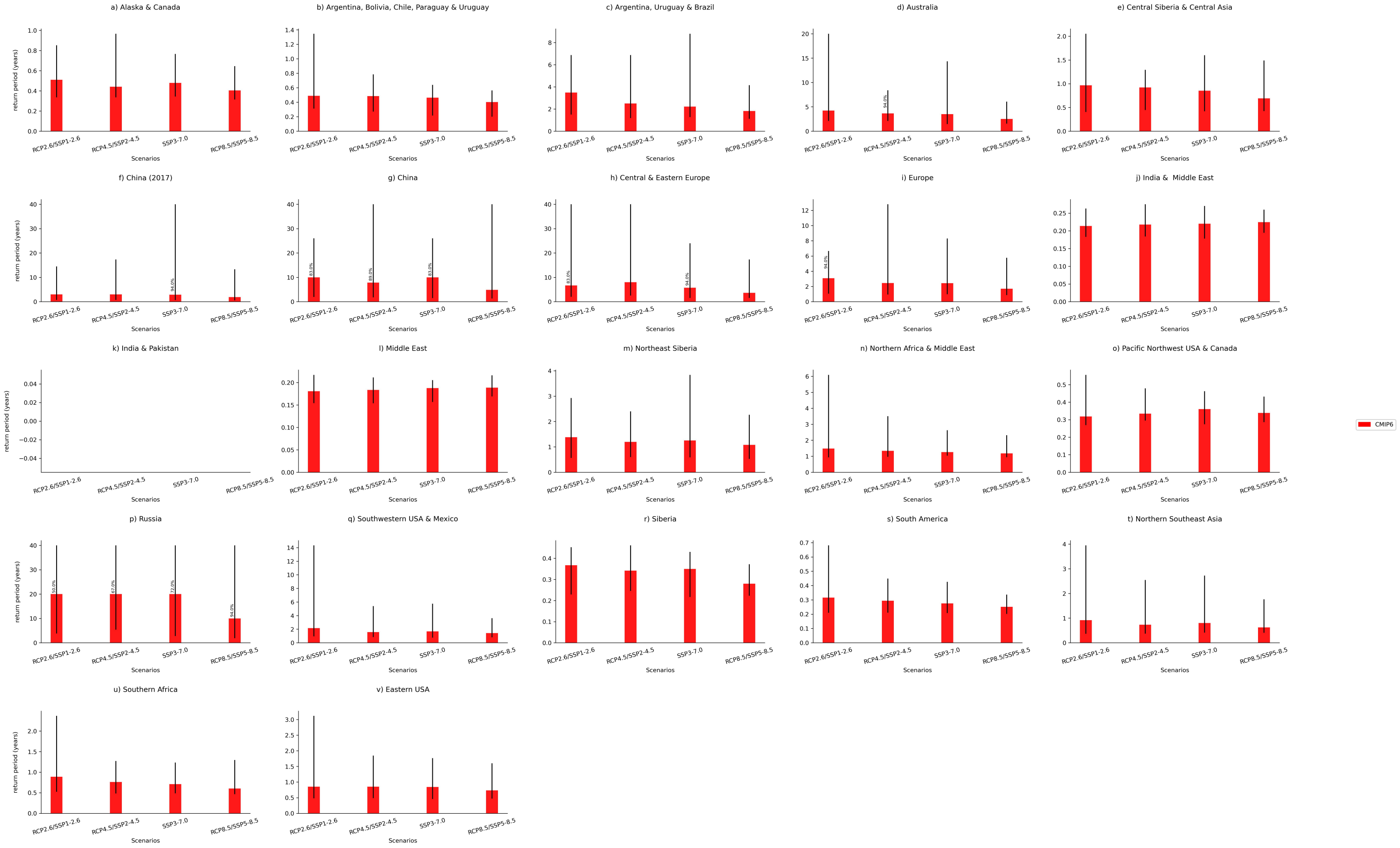
a) Alaska & Canada
b) Argentina, Bolivia, Chile, Paraguay & Uruguay
c) Argentina, Uruguay & Brazil
d) Australia
e) Central Siberia & Central Asia
f) China (2017)
g) China
h) Central & Eastern Europe
i) Europe
j) India & Middle East
k) India & Pakistan
l) Middle East
m) Northeast Siberia
n) Northern Africa & Middle East
o) Pacific Northwest USA & Canada
p) Russia
q) Southwestern USA & Mexico
r) Siberia
s) South America
t) Northern Southeast Asia
u) Southern Africa
v) Eastern USA
return period (years)
Scenarios
RCP2.6/SSP1-2.6
RCP4.5/SSP2-4.5
SSP3-7.0
RCP8.5/SSP5-8.5
CMIP6
94.0%
83.0%
89.0%
50.0%
67.0%
72.0%

Supplementary Figure 372: Return times (RT) during present-to-near-future (2015-2054) based on ERA5 (Tx97.5p) - Return times (RT) (in years) during the present-to-near-future period between 2015 and 2054 for Heatwave Length (days) of the respective observed event. For each region, values are shown for the different scenarios (increasing in emissions from left to right for the CMIP5 (green), CMIP6 (red), CanESM5 (dark blue), and ACCESS-ESM1-5 (light blue) ensembles. The error bars indicate the 5th and 95th percentile of the respective sample. Where the respective ensemble contains models not able to simulate the magnitude of the respective observed event's Heatwave Length (days), the proportion of models that are able to simulate an event with a value of Heatwave Length (days) at least as high as the observed event is indicated by the percentage values above the individual bars. The observed reference values are calculated using data from ERA5.

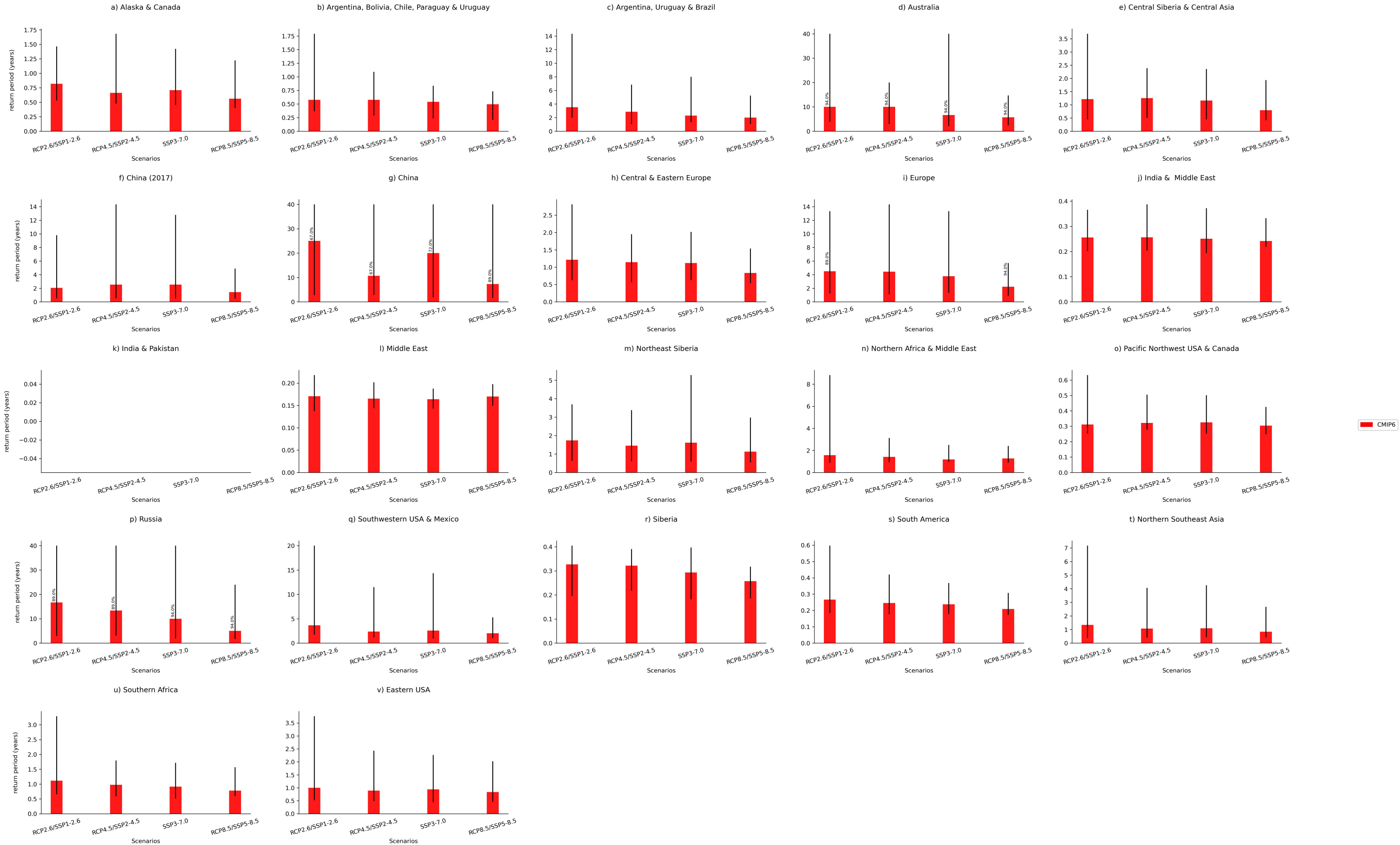
a) Alaska & Canada
b) Argentina, Bolivia, Chile, Paraguay & Uruguay
c) Argentina, Uruguay & Brazil
d) Australia
e) Central Siberia & Central Asia
f) China (2017)
g) China
h) Central & Eastern Europe
i) Europe
j) India & Middle East
k) India & Pakistan
l) Middle East
m) Northeast Siberia
n) Northern Africa & Middle East
o) Pacific Northwest USA & Canada
p) Russia
q) Southwestern USA & Mexico
r) Siberia
s) South America
t) Northern Southeast Asia
u) Southern Africa
v) Eastern USA
return period (years)
Scenarios
RCP2.6/SSP1-2.6
RCP4.5/SSP2-4.5
SSP3-7.0
RCP8.5/SSP5-8.5
94.0%
67.0%
72.0%
89.0%
CMIP6

Supplementary Figure 373: Return times (RT) during present-to-near-future (2015-2054) based on ERA5 (Tx99.0p) - Return times (RT) (in years) during the present-to-near-future period between 2015 and 2054 for Heatwave Length (days) of the respective observed event. For each region, values are shown for the different scenarios (increasing in emissions from left to right for the CMIP5 (green), CMIP6 (red), CanESM5 (dark blue), and ACCESS-ESM1-5 (light blue) ensembles. The error bars indicate the 5th and 95th percentile of the respective sample. Where the respective ensemble contains models not able to simulate the magnitude of the respective observed event's Heatwave Length (days), the proportion of models that are able to simulate an event with a value of Heatwave Length (days) at least as high as the observed event is indicated by the percentage values above the individual bars. The observed reference values are calculated using data from ERA5.

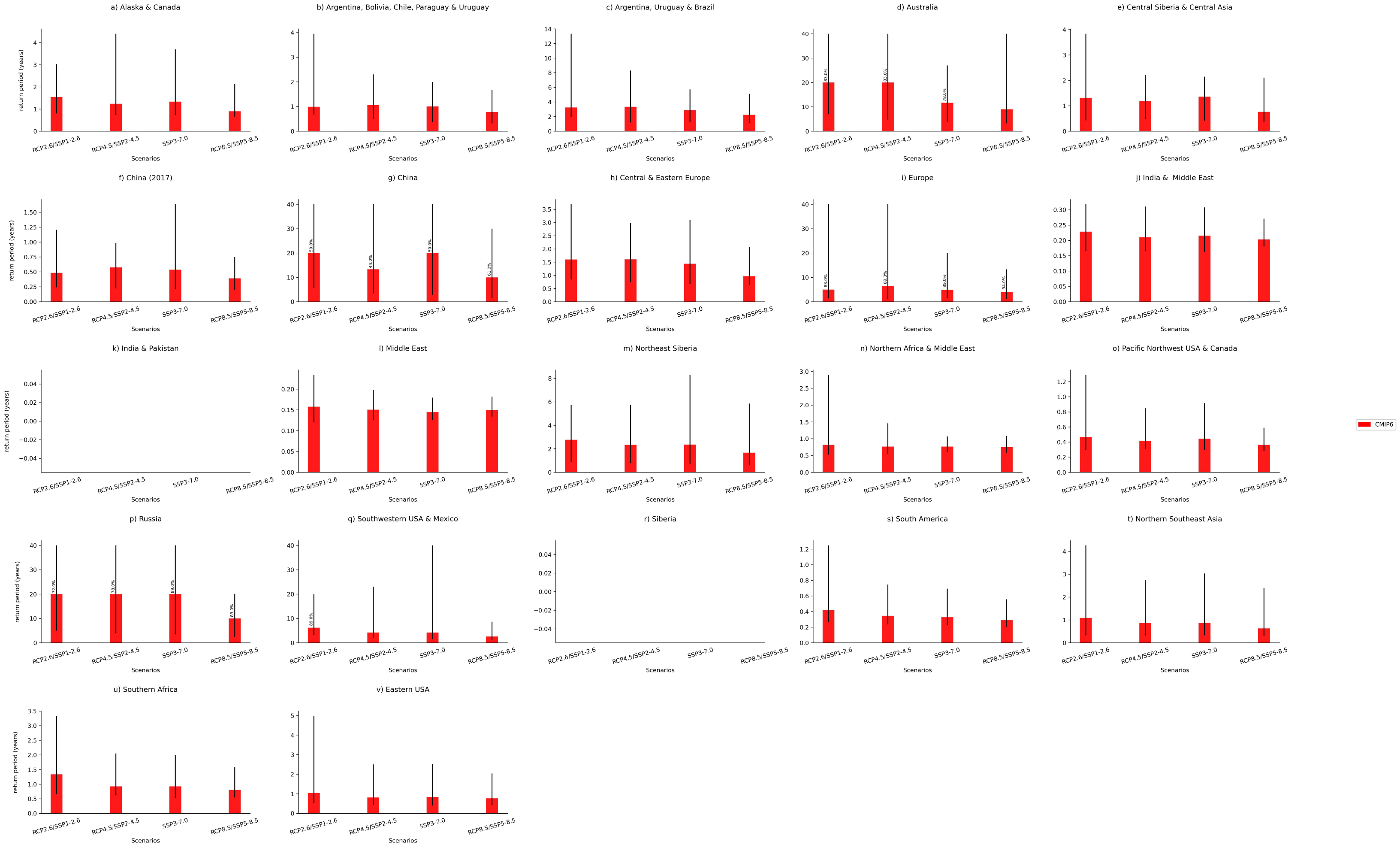

a) Alaska & Canada
b) Argentina, Bolivia, Chile, Paraguay & Uruguay
c) Argentina, Uruguay & Brazil
d) Australia
e) Central Siberia & Central Asia
f) China (2017)
g) China
h) Central & Eastern Europe
i) Europe
j) India & Middle East
k) India & Pakistan
l) Middle East
m) Northeast Siberia
n) Northern Africa & Middle East
o) Pacific Northwest USA & Canada
p) Russia
q) Southwestern USA & Mexico
r) Siberia
s) South America
t) Northern Southeast Asia
u) Southern Africa
v) Eastern USA
return period (years)
Scenarios
RCP2.6/SSP1-2.6
RCP4.5/SSP2-4.5
SSP3-7.0
RCP8.5/SSP5-8.5
CMIP6

Supplementary Figure 374: Return times (RT) during end-of-century (2060-2099) based on ERA5 (Tx95.0p) - Return times (RT) (in years) during the end-of-century period between 2060 and 2099 for Heatwave Length (days) of the respective observed event. For each region, values are shown for the different scenarios (increasing in emissions from left to right for the CMIP5 (green), CMIP6 (red), CanESM5 (dark blue), and ACCESS-ESM1-5 (light blue) ensembles. The error bars indicate the 5th and 95th percentile of the respective sample. Where the respective ensemble contains models not able to simulate the magnitude of the respective observed event's Heatwave Length (days), the proportion of models that are able to simulate an event with a value of Heatwave Length (days) at least as high as the observed event is indicated by the percentage values above the individual bars. The observed reference values are calculated using data from ERA5.

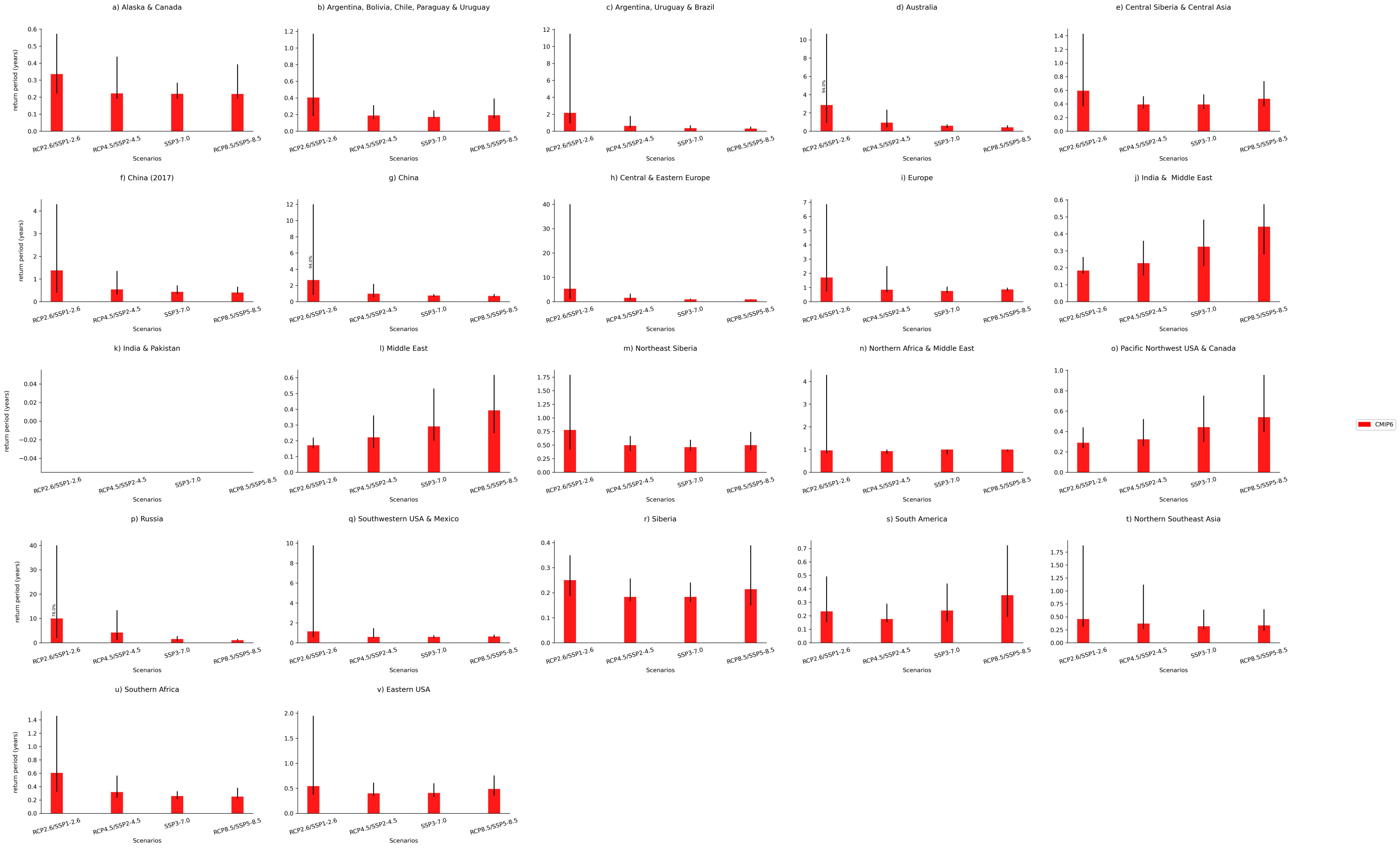

a) Alaska & Canada
b) Argentina, Bolivia, Chile, Paraguay & Uruguay
c) Argentina, Uruguay & Brazil
d) Australia
e) Central Siberia & Central Asia
f) China (2017)
g) China
h) Central & Eastern Europe
i) Europe
j) India & Middle East
k) India & Pakistan
l) Middle East
m) Northeast Siberia
n) Northern Africa & Middle East
o) Pacific Northwest USA & Canada
p) Russia
q) Southwestern USA & Mexico
r) Siberia
s) South America
t) Northern Southeast Asia
u) Southern Africa
v) Eastern USA
return period (years)
Scenarios
RCP2.6/SSP1-2.6
RCP4.5/SSP2-4.5
SSP3-7.0
RCP8.5/SSP5-8.5
94.0%
94.0%
78.0%
CMIP6

Supplementary Figure 375: Return times (RT) during end-of-century (2060-2099) based on ERA5 (Tx97.5p) - Return times (RT) (in years) during the end-of-century period between 2060 and 2099 for Heatwave Length (days) of the respective observed event. For each region, values are shown for the different scenarios (increasing in emissions from left to right for the CMIP5 (green), CMIP6 (red), CanESM5 (dark blue), and ACCESS-ESM1-5 (light blue) ensembles. The error bars indicate the 5th and 95th percentile of the respective sample. Where the respective ensemble contains models not able to simulate the magnitude of the respective observed event's Heatwave Length (days), the proportion of models that are able to simulate an event with a value of Heatwave Length (days) at least as high as the observed event is indicated by the percentage values above the individual bars. The observed reference values are calculated using data from ERA5.

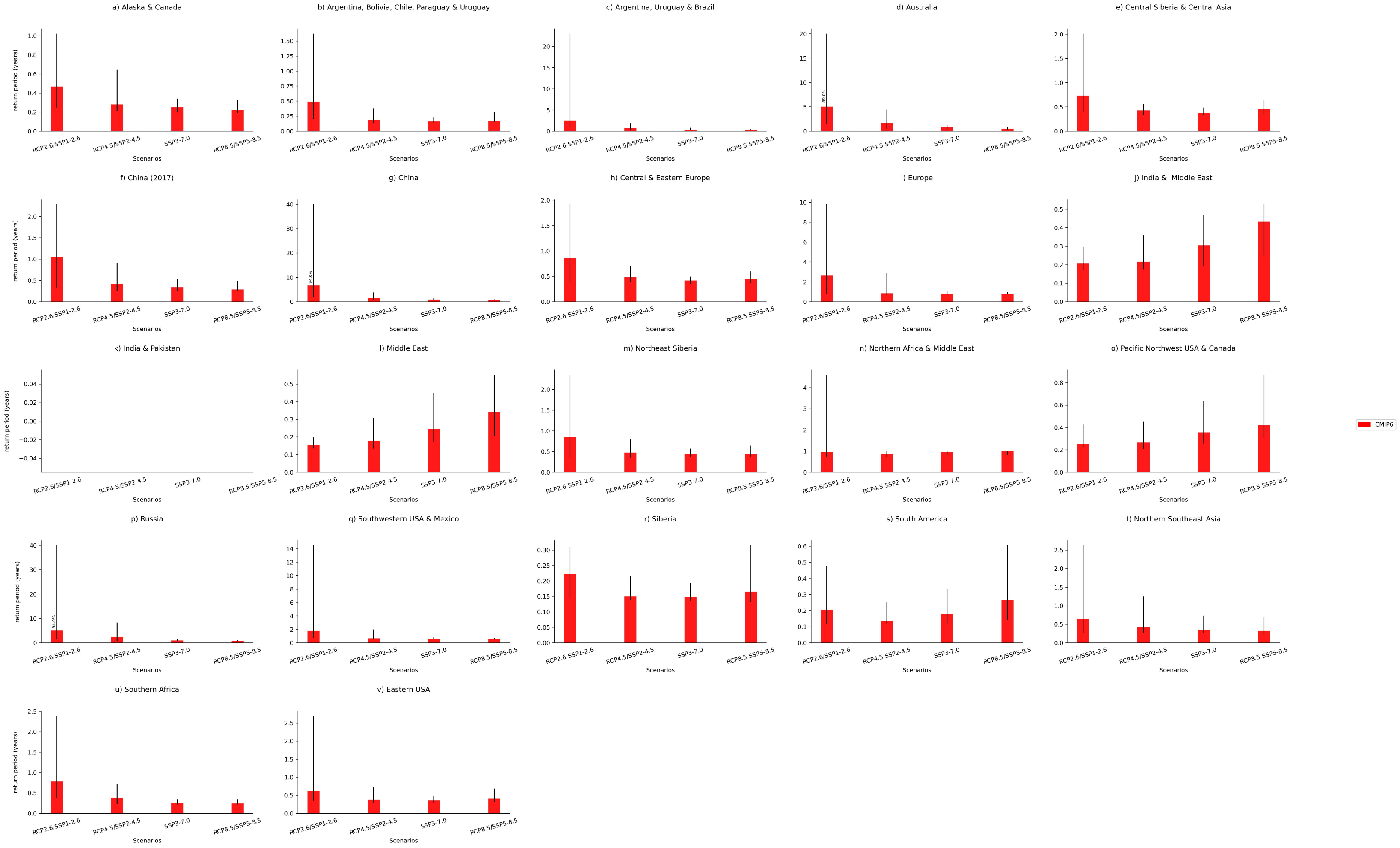

a) Alaska & Canada
b) Argentina, Bolivia, Chile, Paraguay & Uruguay
c) Argentina, Uruguay & Brazil
d) Australia
e) Central Siberia & Central Asia
f) China (2017)
g) China
h) Central & Eastern Europe
i) Europe
j) India & Middle East
k) India & Pakistan
l) Middle East
m) Northeast Siberia
n) Northern Africa & Middle East
o) Pacific Northwest USA & Canada
p) Russia
q) Southwestern USA & Mexico
r) Siberia
s) South America
t) Northern Southeast Asia
u) Southern Africa
v) Eastern USA
return period (years)
Scenarios
RCP2.6/SSP1-2.6
RCP4.5/SSP2-4.5
SSP3-7.0
RCP8.5/SSP5-8.5
89.0%
94.0%
94.0%
CMIP6

Supplementary Figure 376: Return times (RT) during end-of-century (2060-2099) based on ERA5 (Tx99.0p) - Return times (RT) (in years) during the end-of-century period between 2060 and 2099 for Heatwave Length (days) of the respective observed event. For each region, values are shown for the different scenarios (increasing in emissions from left to right for the CMIP5 (green), CMIP6 (red), CanESM5 (dark blue), and ACCESS-ESM1-5 (light blue) ensembles. The error bars indicate the 5th and 95th percentile of the respective sample. Where the respective ensemble contains models not able to simulate the magnitude of the respective observed event's Heatwave Length (days), the proportion of models that are able to simulate an event with a value of Heatwave Length (days) at least as high as the observed event is indicated by the percentage values above the individual bars. The observed reference values are calculated using data from ERA5.

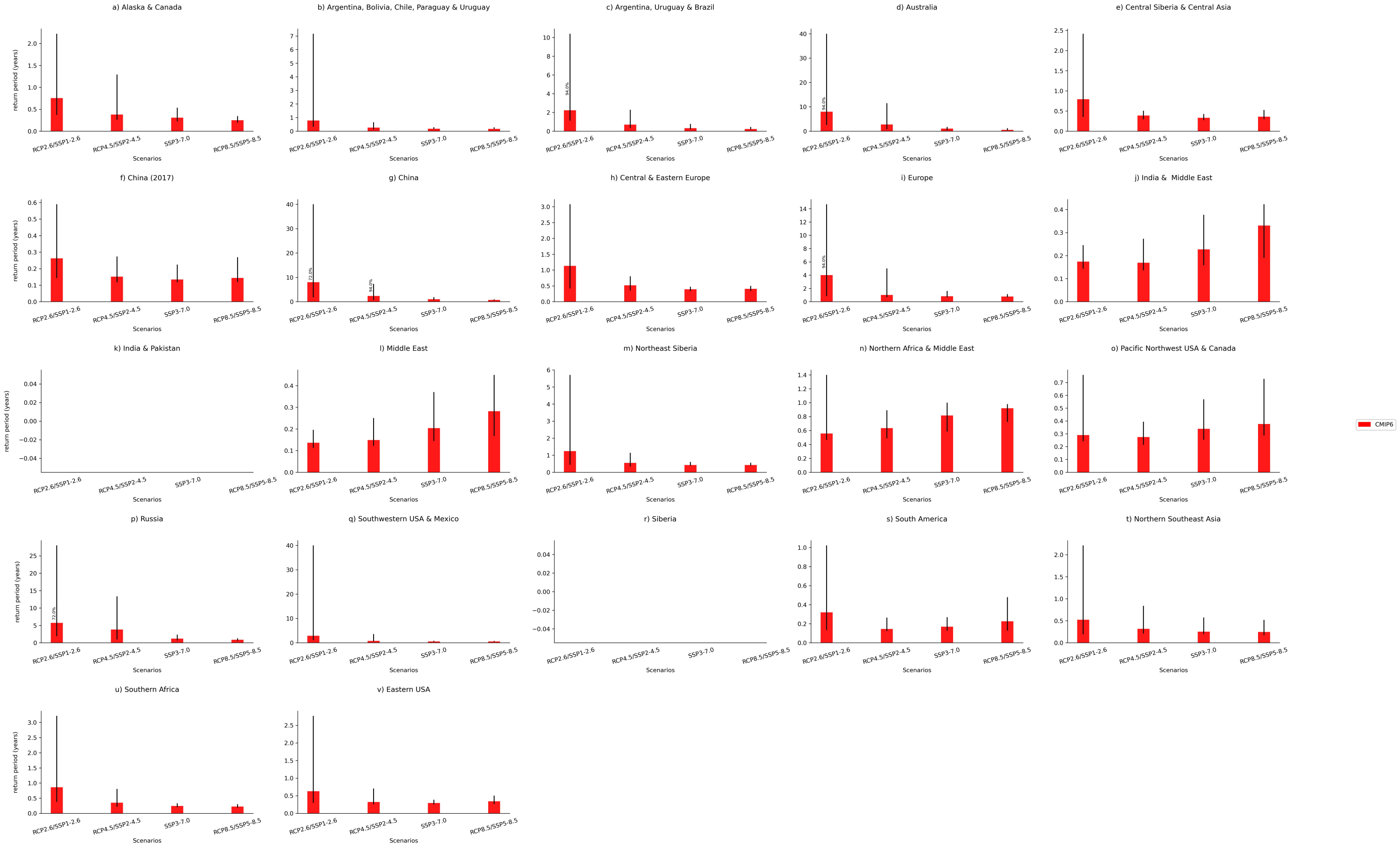

a) Alaska & Canada
b) Argentina, Bolivia, Chile, Paraguay & Uruguay
c) Argentina, Uruguay & Brazil
d) Australia
e) Central Siberia & Central Asia
f) China (2017)
g) China
h) Central & Eastern Europe
i) Europe
j) India & Middle East
k) India & Pakistan
l) Middle East
m) Northeast Siberia
n) Northern Africa & Middle East
o) Pacific Northwest USA & Canada
p) Russia
q) Southwestern USA & Mexico
r) Siberia
s) South America
t) Northern Southeast Asia
u) Southern Africa
v) Eastern USA
return period (years)
Scenarios
RCP2.6/SSP1-2.6
RCP4.5/SSP2-4.5
SSP3-7.0
RCP8.5/SSP5-8.5
94.0%
72.0%
CMIP6

Supplementary Figure 377: Return times (RT) during historical (1951-2005) based on Berkeley Earth (Tx95.0p) - Return times (RT) (in years) during the historical period between 1951 and 2005 for Maximum Heatwave Area (%) of the respective observed event. For each region, values are shown for the different scenarios (increasing in emissions from left to right for the CMIP5 (green), CMIP6 (red), CanESM5 (dark blue), and ACCESS-ESM1-5 (light blue) ensembles. The error bars indicate the 5th and 95th percentile of the respective sample. Where the respective ensemble contains models not able to simulate the magnitude of the respective observed event's Maximum Heatwave Area (%), the proportion of models that are able to simulate an event with a value of Maximum Heatwave Area (%) at least as high as the observed event is indicated by the percentage values above the individual bars. The observed reference values are calculated using data from Berkeley Earth.

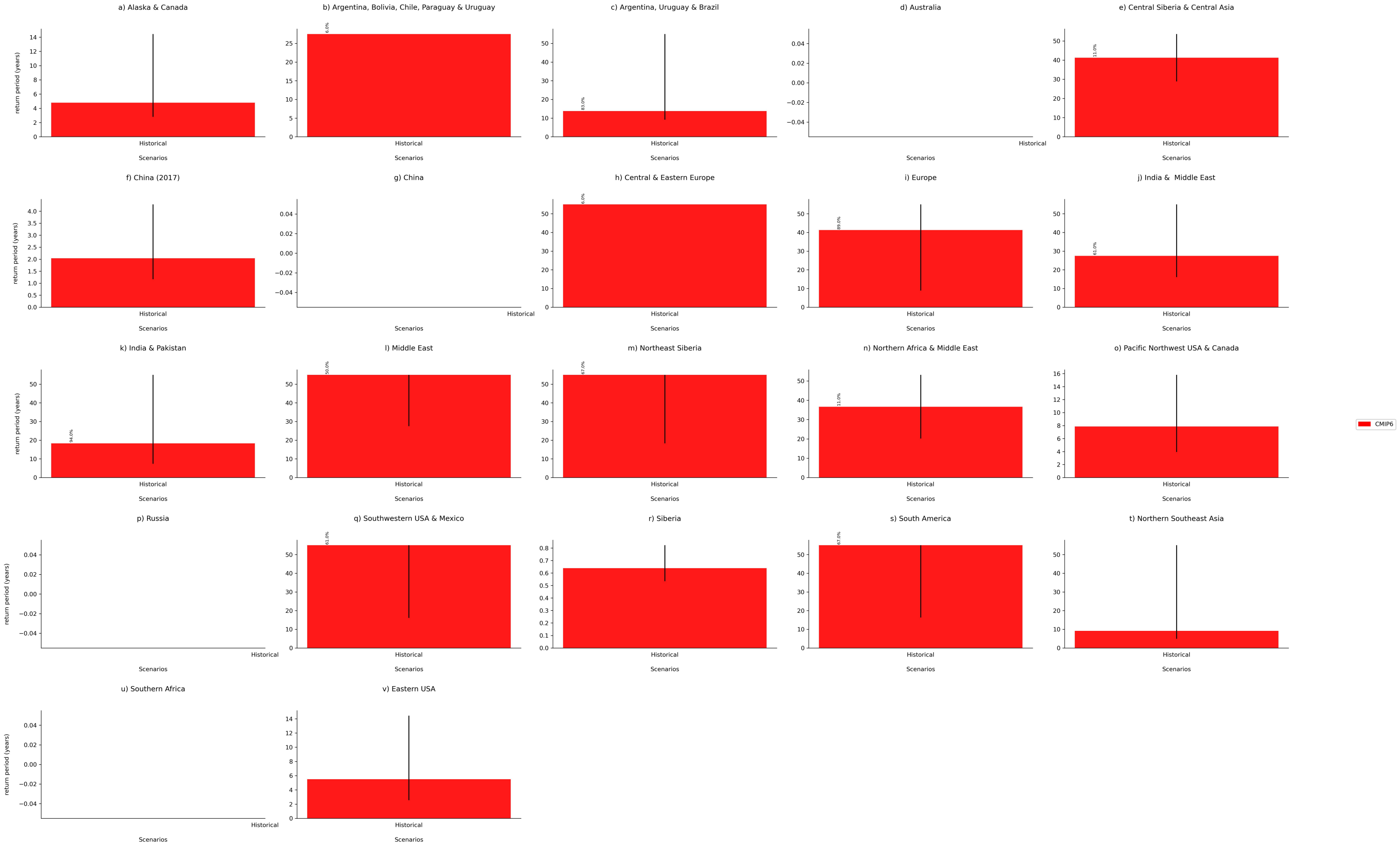
a) Alaska & Canada
b) Argentina, Bolivia, Chile, Paraguay & Uruguay
c) Argentina, Uruguay & Brazil
d) Australia
e) Central Siberia & Central Asia
f) China (2017)
g) China
h) Central & Eastern Europe
i) Europe
j) India & Middle East
k) India & Pakistan
l) Middle East
m) Northeast Siberia
n) Northern Africa & Middle East
o) Pacific Northwest USA & Canada
p) Russia
q) Southwestern USA & Mexico
r) Siberia
s) South America
t) Northern Southeast Asia
u) Southern Africa
v) Eastern USA
return period (years)
Historical
Scenarios
6.0%
83.0%
11.0%
6.0%
89.0%
61.0%
94.0%
50.0%
67.0%
11.0%
61.0%
67.0%
CMIP6

Supplementary Figure 378: Return times (RT) during historical (1951-2005) based on Berkeley Earth (Tx97.5p) - Return times (RT) (in years) during the historical period between 1951 and 2005 for Maximum Heatwave Area (%) of the respective observed event. For each region, values are shown for the different scenarios (increasing in emissions from left to right for the CMIP5 (green), CMIP6 (red), CanESM5 (dark blue), and ACCESS-ESM1-5 (light blue) ensembles. The error bars indicate the 5th and 95th percentile of the respective sample. Where the respective ensemble contains models not able to simulate the magnitude of the respective observed event's Maximum Heatwave Area (%), the proportion of models that are able to simulate an event with a value of Maximum Heatwave Area (%) at least as high as the observed event is indicated by the percentage values above the individual bars. The observed reference values are calculated using data from Berkeley Earth.

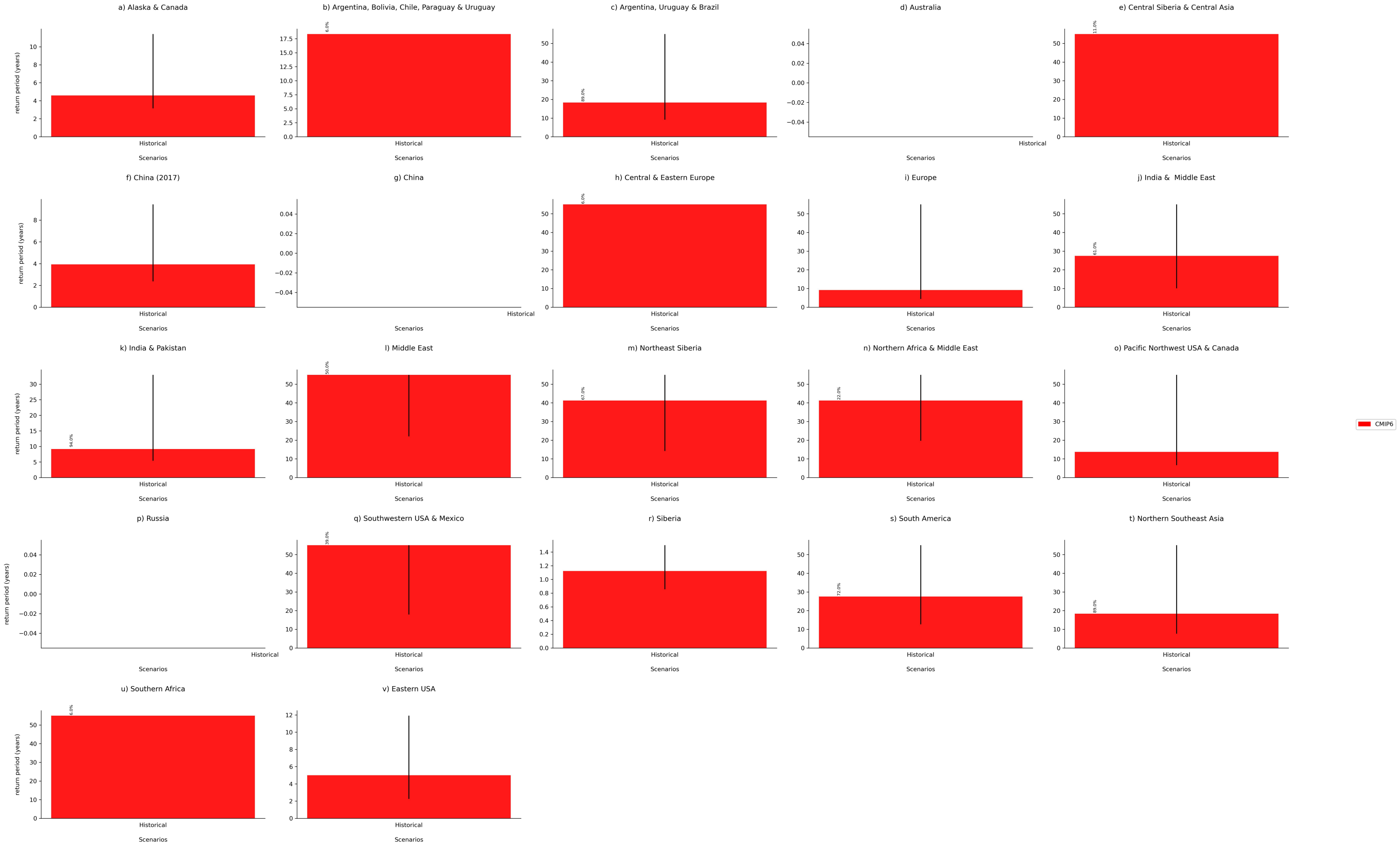

a) Alaska & Canada
b) Argentina, Bolivia, Chile, Paraguay & Uruguay
6.0%
c) Argentina, Uruguay & Brazil
89.0%
d) Australia
e) Central Siberia & Central Asia
11.0%
f) China (2017)
g) China
h) Central & Eastern Europe
6.0%
i) Europe
j) India & Middle East
61.0%
k) India & Pakistan
94.0%
l) Middle East
50.0%
m) Northeast Siberia
67.0%
n) Northern Africa & Middle East
22.0%
o) Pacific Northwest USA & Canada
p) Russia
q) Southwestern USA & Mexico
39.0%
r) Siberia
s) South America
72.0%
t) Northern Southeast Asia
89.0%
u) Southern Africa
6.0%
v) Eastern USA
return period (years)
Historical
Scenarios
CMIP6

Supplementary Figure 379: Return times (RT) during historical (1951-2005) based on Berkeley Earth (Tx99.0p) - Return times (RT) (in years) during the historical period between 1951 and 2005 for Maximum Heatwave Area (%) of the respective observed event. For each region, values are shown for the different scenarios (increasing in emissions from left to right for the CMIP5 (green), CMIP6 (red), CanESM5 (dark blue), and ACCESS-ESM1-5 (light blue) ensembles. The error bars indicate the 5th and 95th percentile of the respective sample. Where the respective ensemble contains models not able to simulate the magnitude of the respective observed event's Maximum Heatwave Area (%), the proportion of models that are able to simulate an event with a value of Maximum Heatwave Area (%) at least as high as the observed event is indicated by the percentage values above the individual bars. The observed reference values are calculated using data from Berkeley Earth.

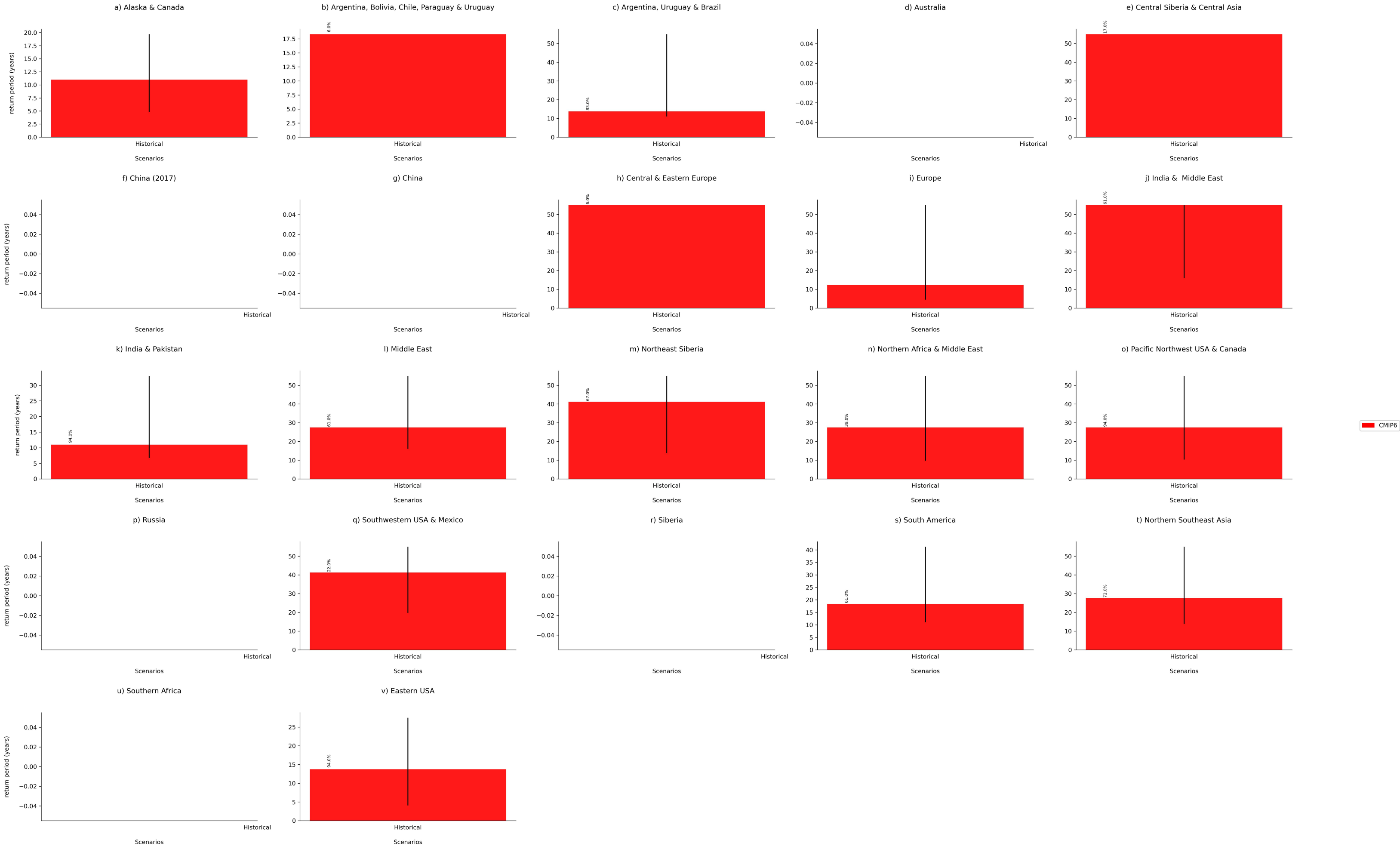

a) Alaska & Canada
b) Argentina, Bolivia, Chile, Paraguay & Uruguay
c) Argentina, Uruguay & Brazil
d) Australia
e) Central Siberia & Central Asia
f) China (2017)
g) China
h) Central & Eastern Europe
i) Europe
j) India & Middle East
k) India & Pakistan
l) Middle East
m) Northeast Siberia
n) Northern Africa & Middle East
o) Pacific Northwest USA & Canada
p) Russia
q) Southwestern USA & Mexico
r) Siberia
s) South America
t) Northern Southeast Asia
u) Southern Africa
v) Eastern USA
return period (years)
Historical
Scenarios
6.0%
83.0%
17.0%
6.0%
61.0%
94.0%
61.0%
67.0%
39.0%
94.0%
22.0%
61.0%
72.0%
94.0%
CMIP6

Supplementary Figure 380: Return times (RT) during present-to-near-future (2015-2054) based on Berkeley Earth (Tx95.0p) - Return times (RT) (in years) during the present-to-near-future period between 2015 and 2054 for Maximum Heatwave Area (%) of the respective observed event. For each region, values are shown for the different scenarios (increasing in emissions from left to right for the CMIP5 (green), CMIP6 (red), CanESM5 (dark blue), and ACCESS-ESM1-5 (light blue) ensembles. The error bars indicate the 5th and 95th percentile of the respective sample. Where the respective ensemble contains models not able to simulate the magnitude of the respective observed event's Maximum Heatwave Area (%), the proportion of models that are able to simulate an event with a value of Maximum Heatwave Area (%) at least as high as the observed event is indicated by the percentage values above the individual bars. The observed reference values are calculated using data from Berkeley Earth.

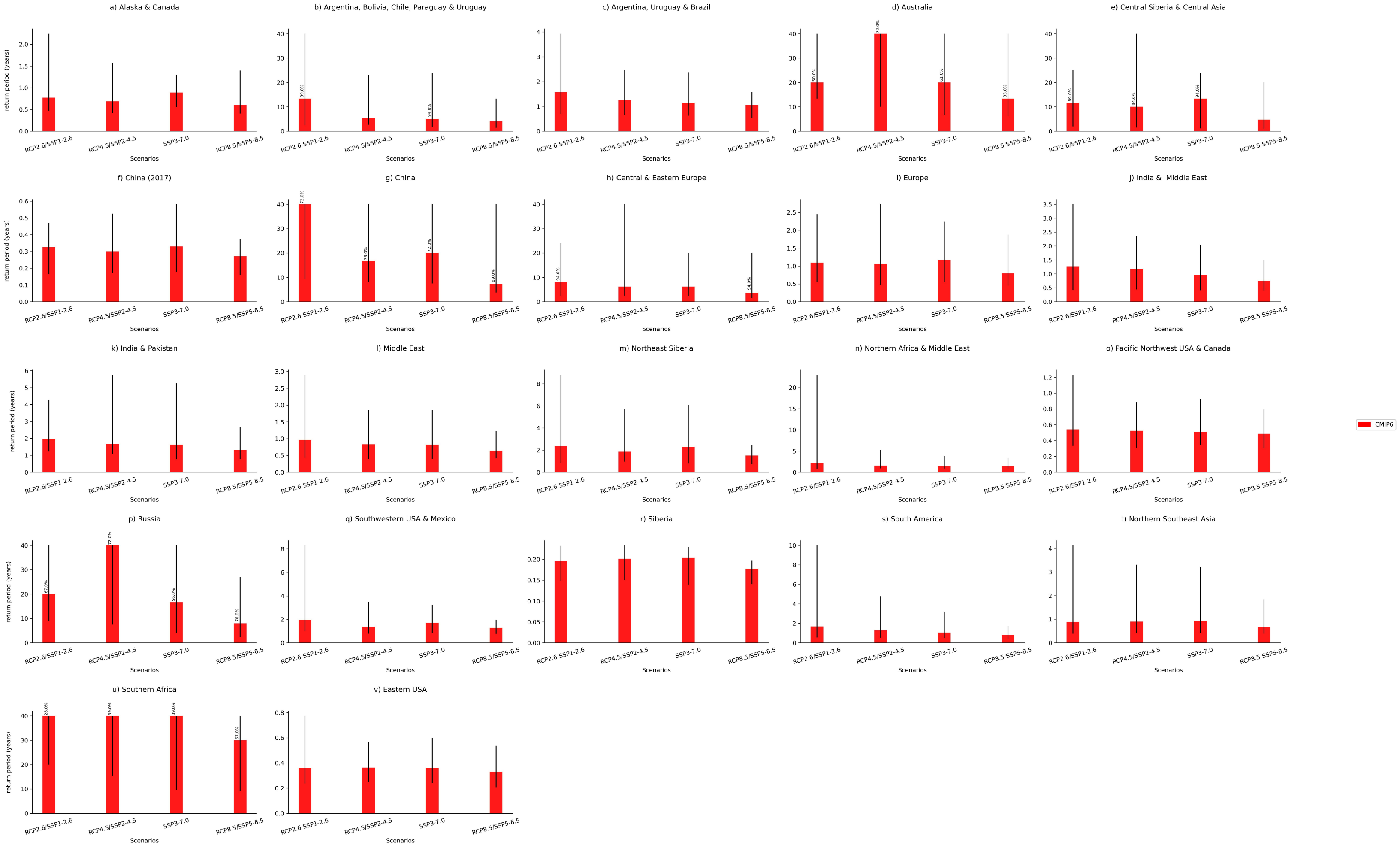

a) Alaska & Canada
b) Argentina, Bolivia, Chile, Paraguay & Uruguay
c) Argentina, Uruguay & Brazil
d) Australia
e) Central Siberia & Central Asia
f) China (2017)
g) China
h) Central & Eastern Europe
i) Europe
j) India & Middle East
k) India & Pakistan
l) Middle East
m) Northeast Siberia
n) Northern Africa & Middle East
o) Pacific Northwest USA & Canada
p) Russia
q) Southwestern USA & Mexico
r) Siberia
s) South America
t) Northern Southeast Asia
u) Southern Africa
v) Eastern USA
return period (years)
Scenarios
RCP2.6/SSP1-2.6
RCP4.5/SSP2-4.5
SSP3-7.0
RCP8.5/SSP5-8.5
CMIP6

Supplementary Figure 381: Return times (RT) during present-to-near-future (2015-2054) based on Berkeley Earth (Tx97.5p) - Return times (RT) (in years) during the present-to-near-future period between 2015 and 2054 for Maximum Heatwave Area (%) of the respective observed event. For each region, values are shown for the different scenarios (increasing in emissions from left to right for the CMIP5 (green), CMIP6 (red), CanESM5 (dark blue), and ACCESS-ESM1-5 (light blue) ensembles. The error bars indicate the 5th and 95th percentile of the respective sample. Where the respective ensemble contains models not able to simulate the magnitude of the respective observed event's Maximum Heatwave Area (%), the proportion of models that are able to simulate an event with a value of Maximum Heatwave Area (%) at least as high as the observed event is indicated by the percentage values above the individual bars. The observed reference values are calculated using data from Berkeley Earth.

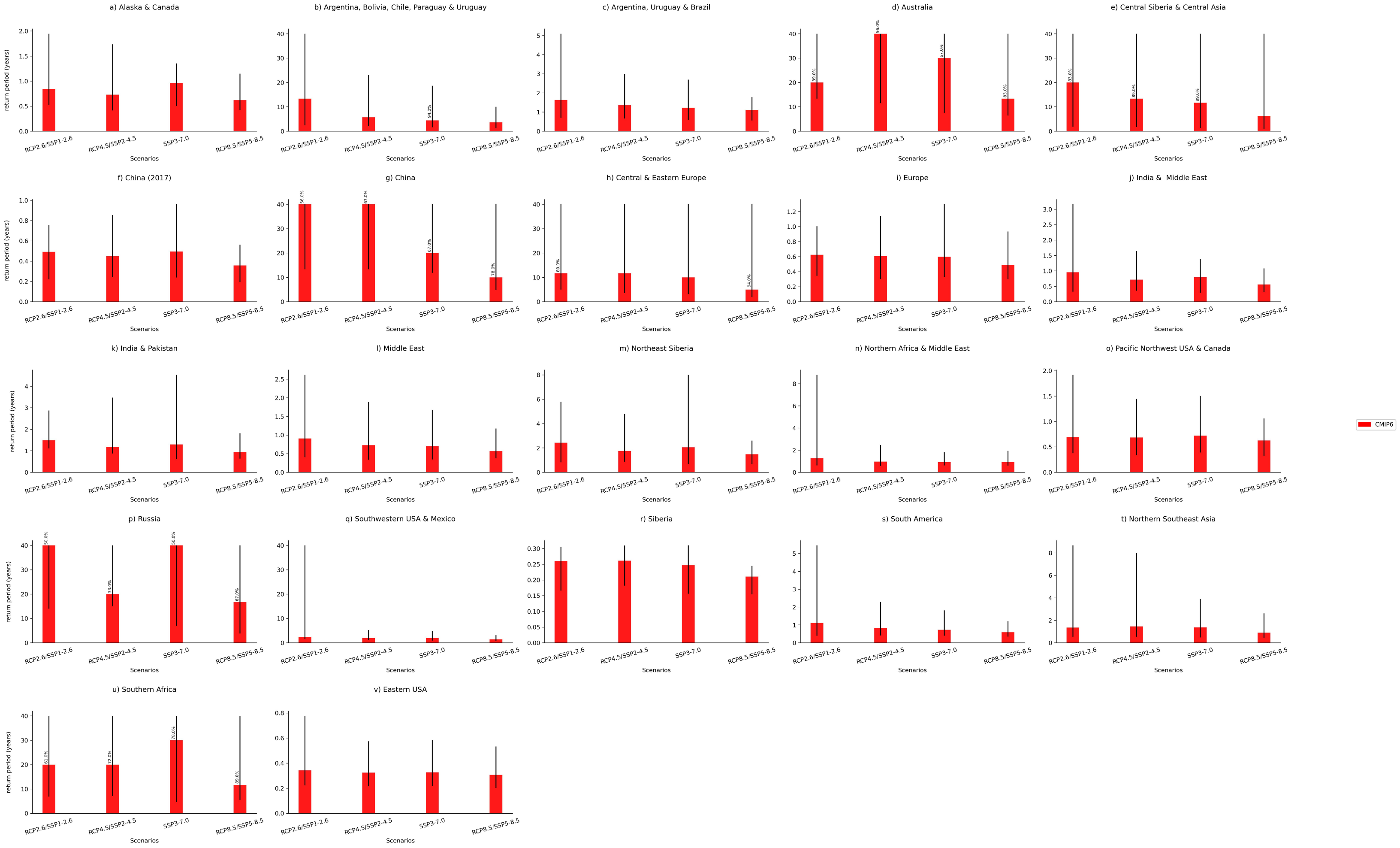
a) Alaska & Canada
b) Argentina, Bolivia, Chile, Paraguay & Uruguay
94.0%
c) Argentina, Uruguay & Brazil
d) Australia
39.0%
56.0%
67.0%
83.0%
e) Central Siberia & Central Asia
83.0%
89.0%
89.0%
f) China (2017)
g) China
56.0%
67.0%
67.0%
78.0%
h) Central & Eastern Europe
89.0%
94.0%
i) Europe
j) India & Middle East
k) India & Pakistan
l) Middle East
m) Northeast Siberia
n) Northern Africa & Middle East
o) Pacific Northwest USA & Canada
p) Russia
50.0%
33.0%
50.0%
67.0%
q) Southwestern USA & Mexico
r) Siberia
s) South America
t) Northern Southeast Asia
u) Southern Africa
61.0%
72.0%
78.0%
89.0%
v) Eastern USA
return period (years)
Scenarios
RCP2.6/SSP1-2.6
RCP4.5/SSP2-4.5
SSP3-7.0
RCP8.5/SSP5-8.5
CMIP6

Supplementary Figure 382: Return times (RT) during present-to-near-future (2015-2054) based on Berkeley Earth (Tx99.0p) - Return times (RT) (in years) during the present-to-near-future period between 2015 and 2054 for Maximum Heatwave Area (%) of the respective observed event. For each region, values are shown for the different scenarios (increasing in emissions from left to right for the CMIP5 (green), CMIP6 (red), CanESM5 (dark blue), and ACCESS-ESM1-5 (light blue) ensembles. The error bars indicate the 5th and 95th percentile of the respective sample. Where the respective ensemble contains models not able to simulate the magnitude of the respective observed event's Maximum Heatwave Area (%), the proportion of models that are able to simulate an event with a value of Maximum Heatwave Area (%) at least as high as the observed event is indicated by the percentage values above the individual bars. The observed reference values are calculated using data from Berkeley Earth.

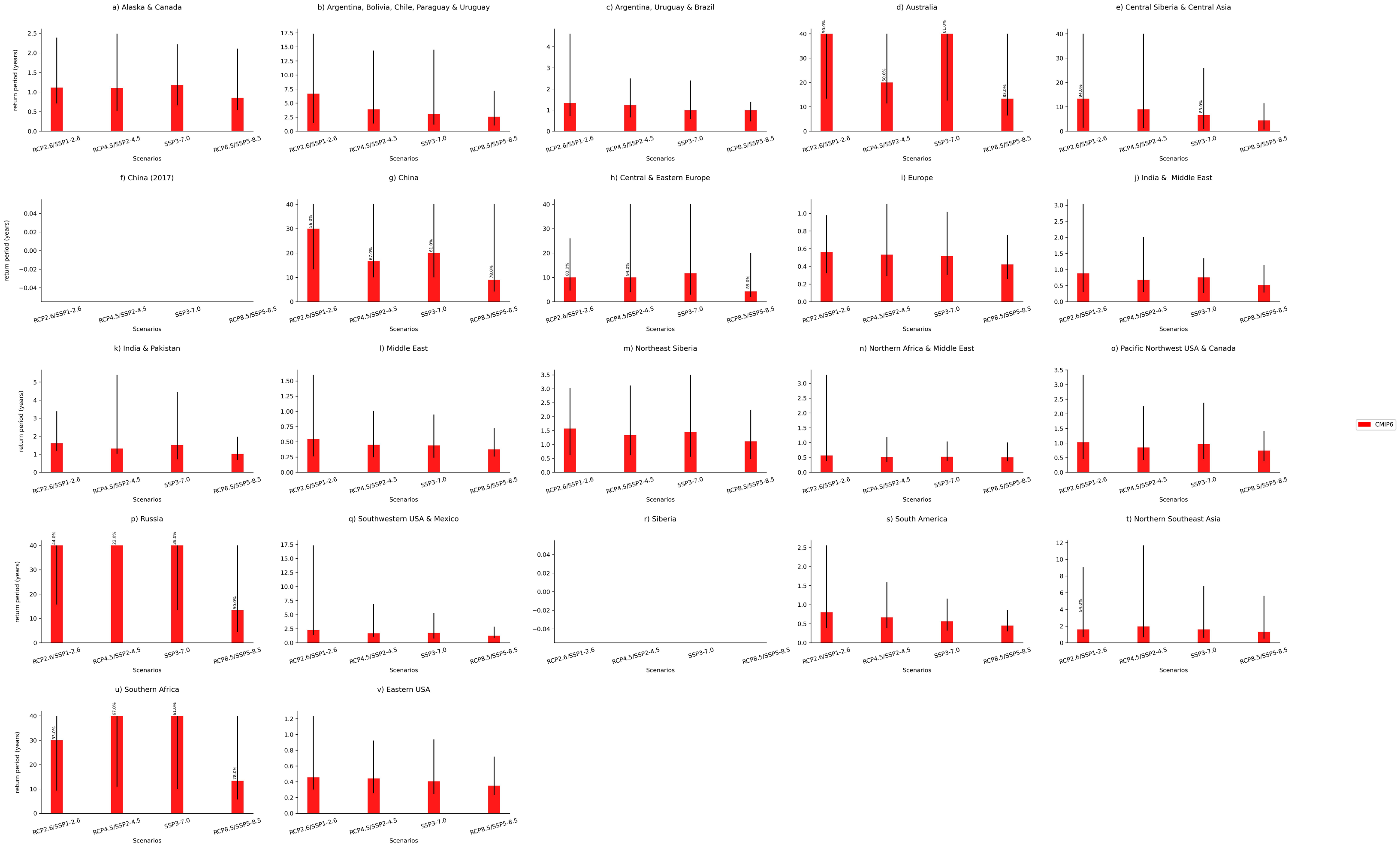
a) Alaska & Canada
b) Argentina, Bolivia, Chile, Paraguay & Uruguay
c) Argentina, Uruguay & Brazil
d) Australia
e) Central Siberia & Central Asia
f) China (2017)
g) China
h) Central & Eastern Europe
i) Europe
j) India & Middle East
k) India & Pakistan
l) Middle East
m) Northeast Siberia
n) Northern Africa & Middle East
o) Pacific Northwest USA & Canada
p) Russia
q) Southwestern USA & Mexico
r) Siberia
s) South America
t) Northern Southeast Asia
u) Southern Africa
v) Eastern USA
return period (years)
Scenarios
RCP2.6/SSP1-2.6
RCP4.5/SSP2-4.5
SSP3-7.0
RCP8.5/SSP5-8.5
CMIP6
50.0%
61.0%
83.0%
94.0%
56.0%
67.0%
78.0%
89.0%
44.0%
22.0%
39.0%
33.0%

Supplementary Figure 383: Return times (RT) during end-of-century (2060-2099) based on Berkeley Earth (Tx95.0p) - Return times (RT) (in years) during the end-of-century period between 2060 and 2099 for Maximum Heatwave Area (%) of the respective observed event. For each region, values are shown for the different scenarios (increasing in emissions from left to right for the CMIP5 (green), CMIP6 (red), CanESM5 (dark blue), and ACCESS-ESM1-5 (light blue) ensembles. The error bars indicate the 5th and 95th percentile of the respective sample. Where the respective ensemble contains models not able to simulate the magnitude of the respective observed event's Maximum Heatwave Area (%), the proportion of models that are able to simulate an event with a value of Maximum Heatwave Area (%) at least as high as the observed event is indicated by the percentage values above the individual bars. The observed reference values are calculated using data from Berkeley Earth.

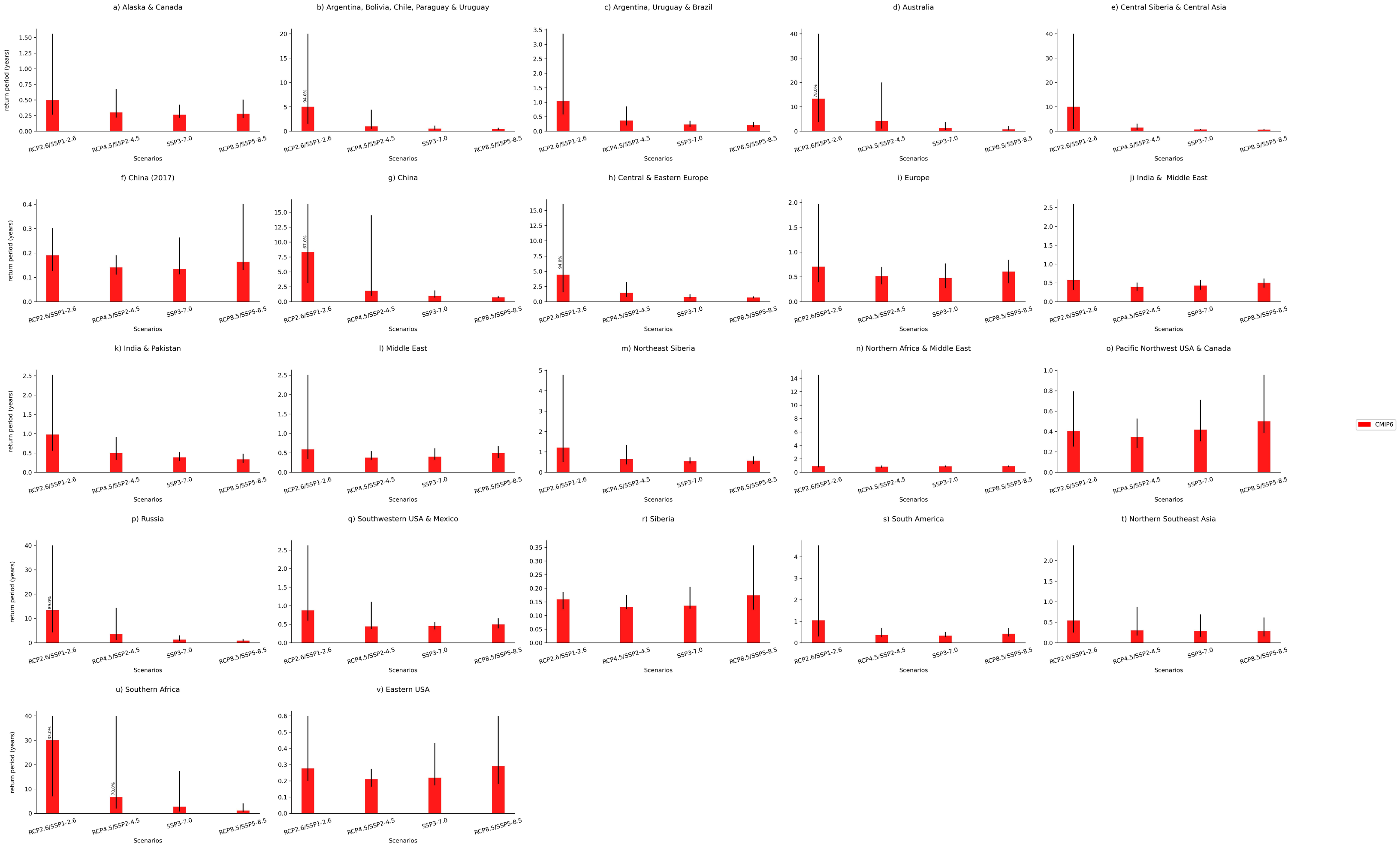
a) Alaska & Canada
b) Argentina, Bolivia, Chile, Paraguay & Uruguay
94.0%
c) Argentina, Uruguay & Brazil
d) Australia
78.0%
e) Central Siberia & Central Asia
f) China (2017)
g) China
67.0%
h) Central & Eastern Europe
94.0%
i) Europe
j) India & Middle East
k) India & Pakistan
l) Middle East
m) Northeast Siberia
n) Northern Africa & Middle East
o) Pacific Northwest USA & Canada
p) Russia
89.0%
q) Southwestern USA & Mexico
r) Siberia
s) South America
t) Northern Southeast Asia
u) Southern Africa
33.0%
78.0%
v) Eastern USA
return period (years)
Scenarios
RCP2.6/SSP1-2.6
RCP4.5/SSP2-4.5
SSP3-7.0
RCP8.5/SSP5-8.5
CMIP6

Supplementary Figure 384: Return times (RT) during end-of-century (2060-2099) based on Berkeley Earth (Tx97.5p) - Return times (RT) (in years) during the end-of-century period between 2060 and 2099 for Maximum Heatwave Area (%) of the respective observed event. For each region, values are shown for the different scenarios (increasing in emissions from left to right for the CMIP5 (green), CMIP6 (red), CanESM5 (dark blue), and ACCESS-ESM1-5 (light blue) ensembles. The error bars indicate the 5th and 95th percentile of the respective sample. Where the respective ensemble contains models not able to simulate the magnitude of the respective observed event's Maximum Heatwave Area (%), the proportion of models that are able to simulate an event with a value of Maximum Heatwave Area (%) at least as high as the observed event is indicated by the percentage values above the individual bars. The observed reference values are calculated using data from Berkeley Earth.

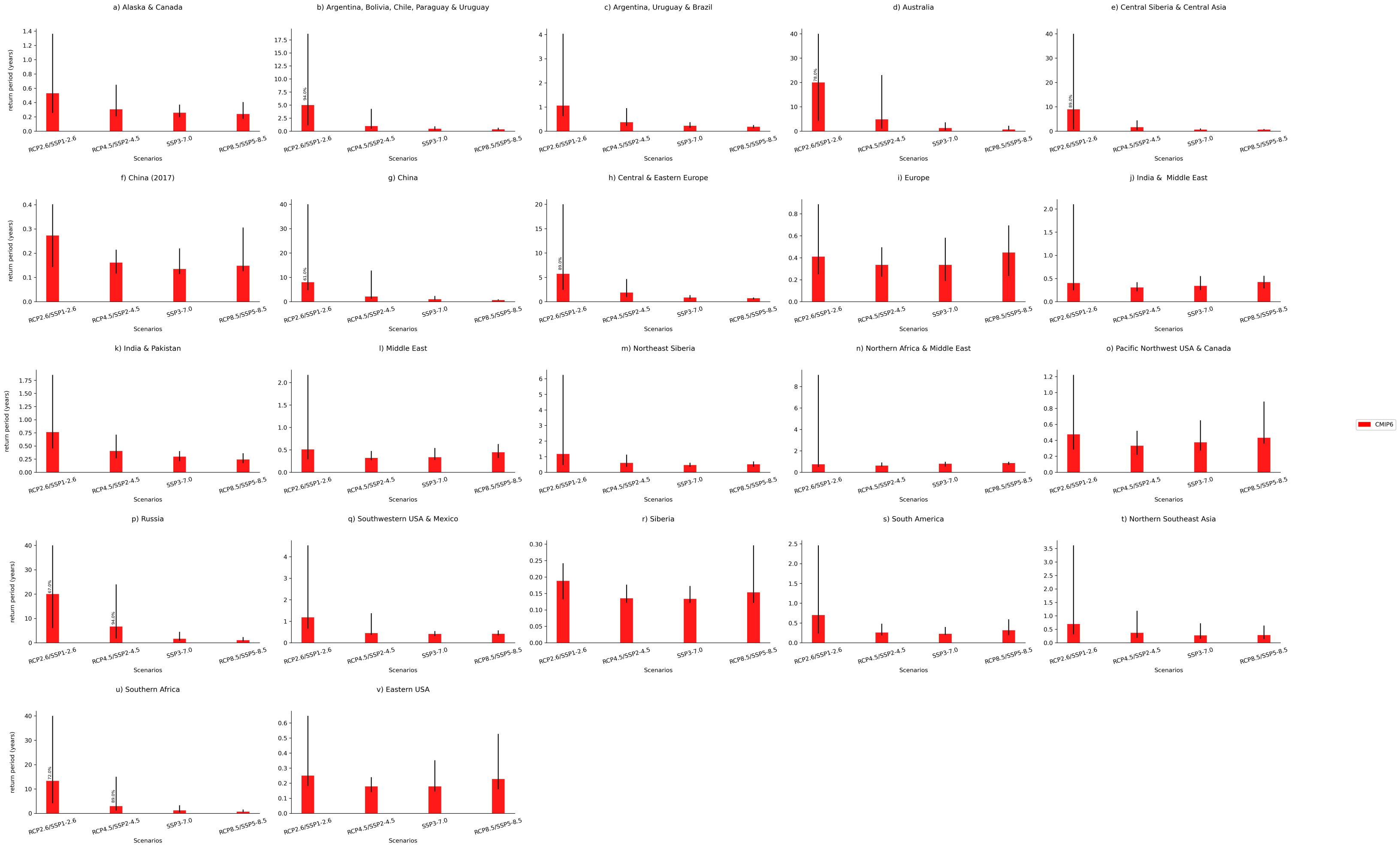
a) Alaska & Canada
b) Argentina, Bolivia, Chile, Paraguay & Uruguay
94.0%
c) Argentina, Uruguay & Brazil
d) Australia
78.0%
e) Central Siberia & Central Asia
89.0%
f) China (2017)
g) China
61.0%
h) Central & Eastern Europe
89.0%
i) Europe
j) India & Middle East
k) India & Pakistan
l) Middle East
m) Northeast Siberia
n) Northern Africa & Middle East
o) Pacific Northwest USA & Canada
p) Russia
67.0%
94.0%
q) Southwestern USA & Mexico
r) Siberia
s) South America
t) Northern Southeast Asia
u) Southern Africa
72.0%
89.0%
v) Eastern USA
return period (years)
RCP2.6/SSP1-2.6
RCP4.5/SSP2-4.5
SSP3-7.0
RCP8.5/SSP5-8.5
Scenarios
CMIP6

Supplementary Figure 385: Return times (RT) during end-of-century (2060-2099) based on Berkeley Earth (Tx99.0p) - Return times (RT) (in years) during the end-of-century period between 2060 and 2099 for Maximum Heatwave Area (%) of the respective observed event. For each region, values are shown for the different scenarios (increasing in emissions from left to right for the CMIP5 (green), CMIP6 (red), CanESM5 (dark blue), and ACCESS-ESM1-5 (light blue) ensembles. The error bars indicate the 5th and 95th percentile of the respective sample. Where the respective ensemble contains models not able to simulate the magnitude of the respective observed event's Maximum Heatwave Area (%), the proportion of models that are able to simulate an event with a value of Maximum Heatwave Area (%) at least as high as the observed event is indicated by the percentage values above the individual bars. The observed reference values are calculated using data from Berkeley Earth.

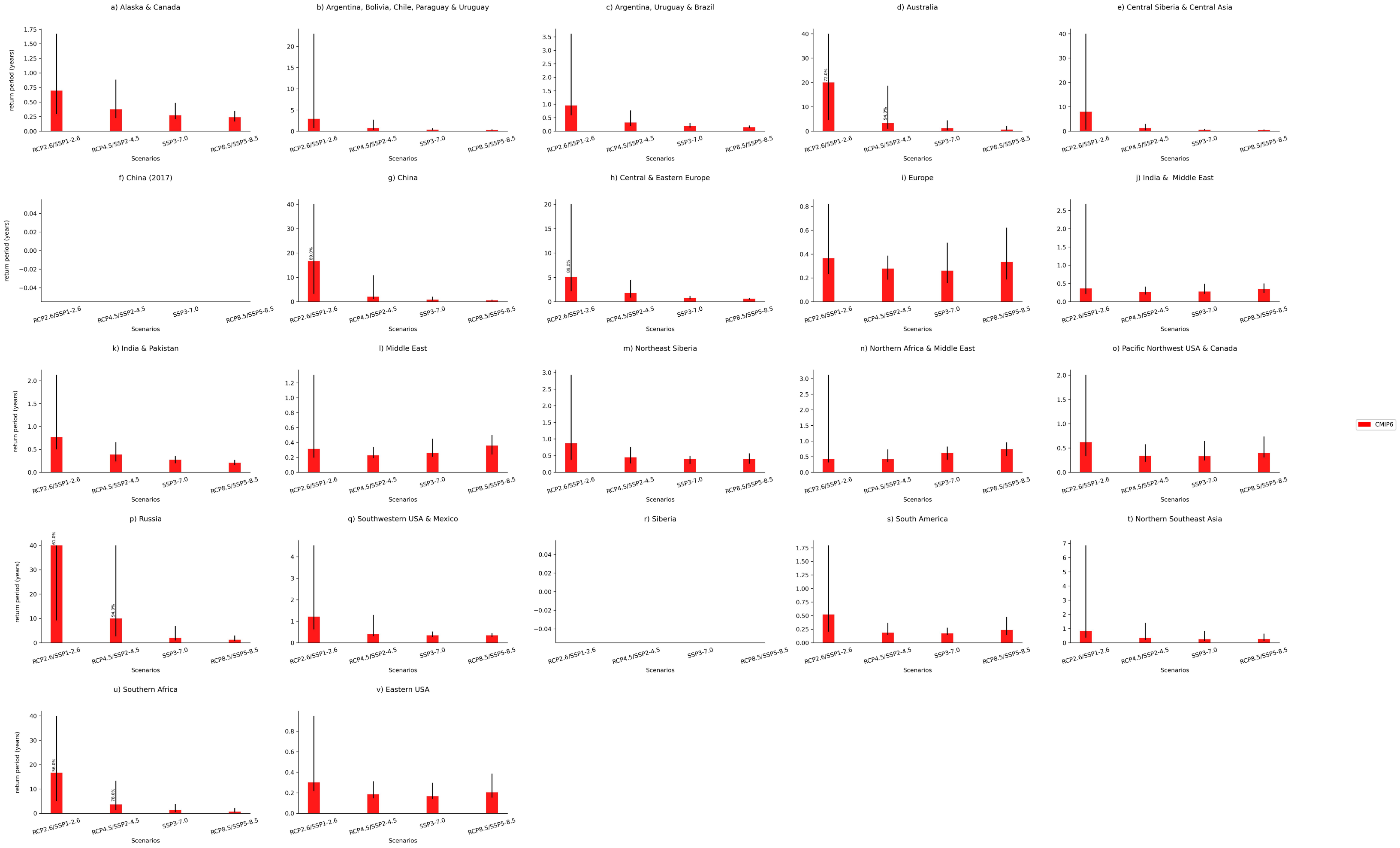
a) Alaska & Canada
b) Argentina, Bolivia, Chile, Paraguay & Uruguay
c) Argentina, Uruguay & Brazil
d) Australia
e) Central Siberia & Central Asia
f) China (2017)
g) China
h) Central & Eastern Europe
i) Europe
j) India & Middle East
k) India & Pakistan
l) Middle East
m) Northeast Siberia
n) Northern Africa & Middle East
o) Pacific Northwest USA & Canada
p) Russia
q) Southwestern USA & Mexico
r) Siberia
s) South America
t) Northern Southeast Asia
u) Southern Africa
v) Eastern USA
return period (years)
Scenarios
RCP2.6/SSP1-2.6
RCP4.5/SSP2-4.5
SSP3-7.0
RCP8.5/SSP5-8.5
72.0%
94.0%
89.0%
89.0%
61.0%
94.0%
56.0%
78.0%
CMIP6

Supplementary Figure 386: Return times (RT) during historical (1951-2005) based on ERA5 (Tx95.0p) - Return times (RT) (in years) during the historical period between 1951 and 2005 for Maximum Heatwave Area (%) of the respective observed event. For each region, values are shown for the different scenarios (increasing in emissions from left to right for the CMIP5 (green), CMIP6 (red), CanESM5 (dark blue), and ACCESS-ESM1-5 (light blue) ensembles. The error bars indicate the 5th and 95th percentile of the respective sample. Where the respective ensemble contains models not able to simulate the magnitude of the respective observed event's Maximum Heatwave Area (%), the proportion of models that are able to simulate an event with a value of Maximum Heatwave Area (%) at least as high as the observed event is indicated by the percentage values above the individual bars. The observed reference values are calculated using data from ERA5.

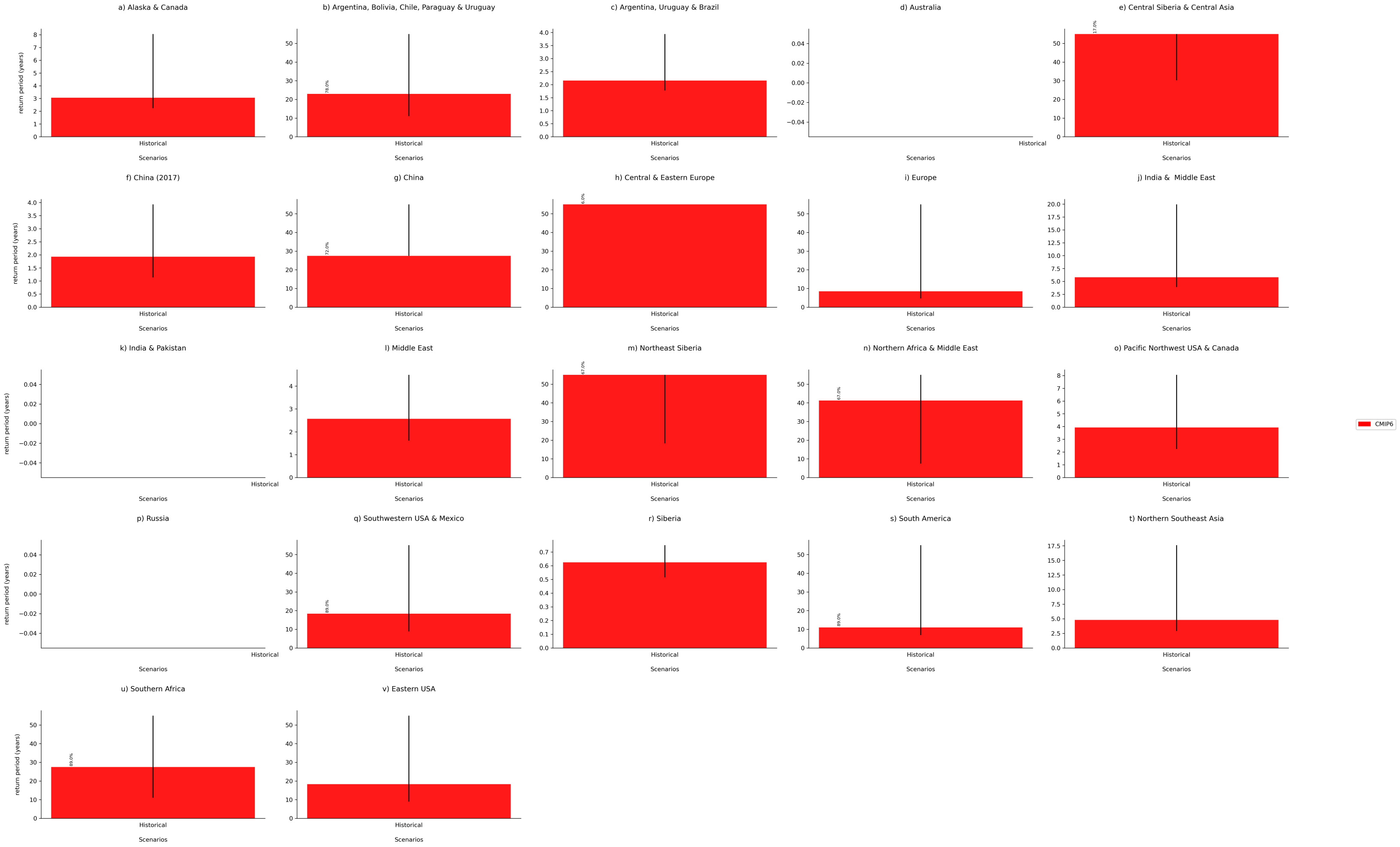

a) Alaska & Canada
b) Argentina, Bolivia, Chile, Paraguay & Uruguay
c) Argentina, Uruguay & Brazil
d) Australia
e) Central Siberia & Central Asia
f) China (2017)
g) China
h) Central & Eastern Europe
i) Europe
j) India & Middle East
k) India & Pakistan
l) Middle East
m) Northeast Siberia
n) Northern Africa & Middle East
o) Pacific Northwest USA & Canada
p) Russia
q) Southwestern USA & Mexico
r) Siberia
s) South America
t) Northern Southeast Asia
u) Southern Africa
v) Eastern USA
return period (years)
Historical
Scenarios
78.0%
17.0%
72.0%
6.0%
67.0%
67.0%
89.0%
89.0%
89.0%
CMIP6

Supplementary Figure 387: Return times (RT) during historical (1951-2005) based on ERA5 (Tx97.5p) - Return times (RT) (in years) during the historical period between 1951 and 2005 for Maximum Heatwave Area (%) of the respective observed event. For each region, values are shown for the different scenarios (increasing in emissions from left to right for the CMIP5 (green), CMIP6 (red), CanESM5 (dark blue), and ACCESS-ESM1-5 (light blue) ensembles. The error bars indicate the 5th and 95th percentile of the respective sample. Where the respective ensemble contains models not able to simulate the magnitude of the respective observed event's Maximum Heatwave Area (%), the proportion of models that are able to simulate an event with a value of Maximum Heatwave Area (%) at least as high as the observed event is indicated by the percentage values above the individual bars. The observed reference values are calculated using data from ERA5.

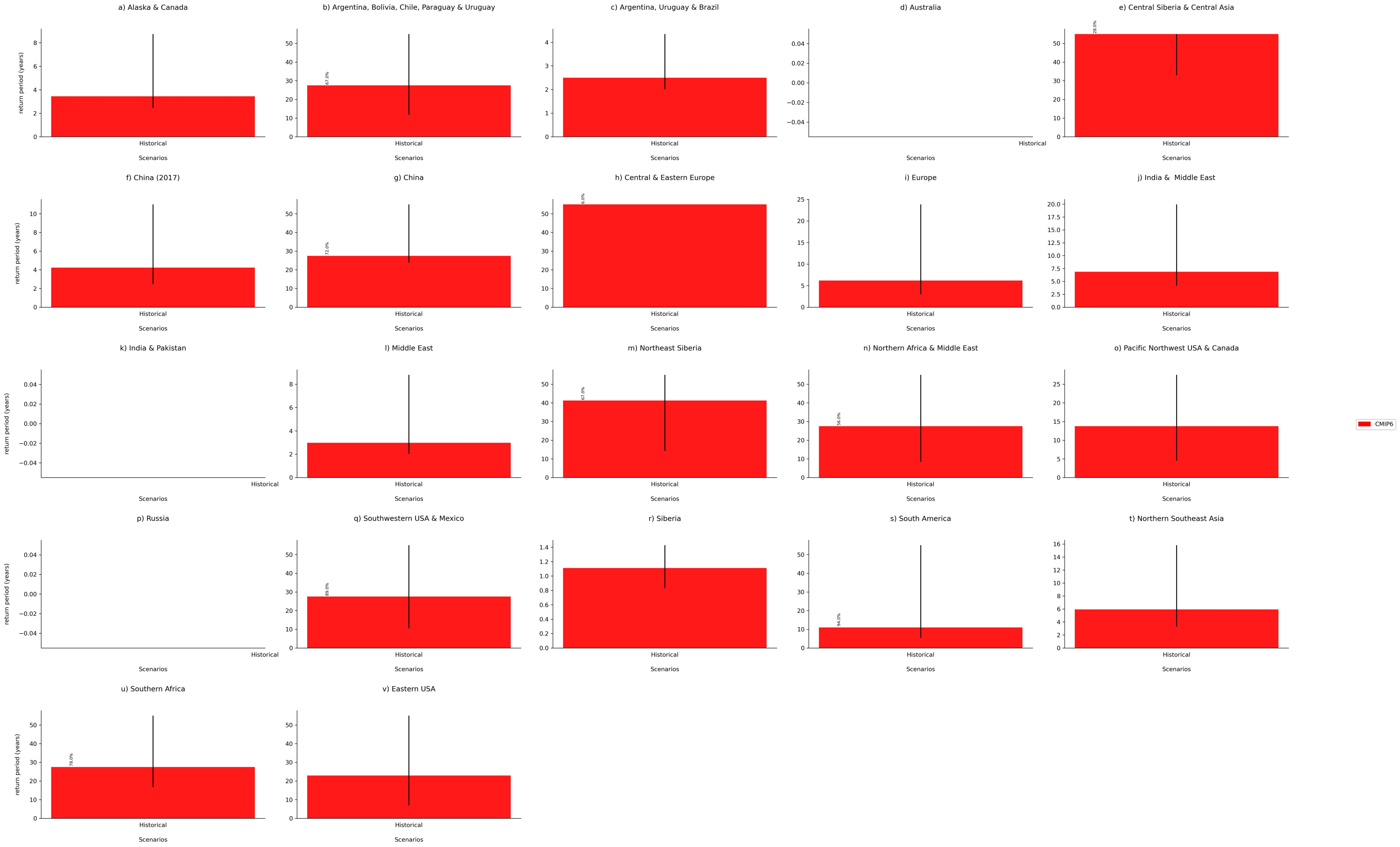

a) Alaska & Canada
b) Argentina, Bolivia, Chile, Paraguay & Uruguay
c) Argentina, Uruguay & Brazil
d) Australia
e) Central Siberia & Central Asia
f) China (2017)
g) China
h) Central & Eastern Europe
i) Europe
j) India & Middle East
k) India & Pakistan
l) Middle East
m) Northeast Siberia
n) Northern Africa & Middle East
o) Pacific Northwest USA & Canada
p) Russia
q) Southwestern USA & Mexico
r) Siberia
s) South America
t) Northern Southeast Asia
u) Southern Africa
v) Eastern USA
return period (years)
Historical
Scenarios
CMIP6
67.0%
28.0%
72.0%
67.0%
56.0%
89.0%
94.0%
78.0%

Supplementary Figure 388: Return times (RT) during historical (1951-2005) based on ERA5 (Tx99.0p) - Return times (RT) (in years) during the historical period between 1951 and 2005 for Maximum Heatwave Area (%) of the respective observed event. For each region, values are shown for the different scenarios (increasing in emissions from left to right for the CMIP5 (green), CMIP6 (red), CanESM5 (dark blue), and ACCESS-ESM1-5 (light blue) ensembles. The error bars indicate the 5th and 95th percentile of the respective sample. Where the respective ensemble contains models not able to simulate the magnitude of the respective observed event's Maximum Heatwave Area (%), the proportion of models that are able to simulate an event with a value of Maximum Heatwave Area (%) at least as high as the observed event is indicated by the percentage values above the individual bars. The observed reference values are calculated using data from ERA5.

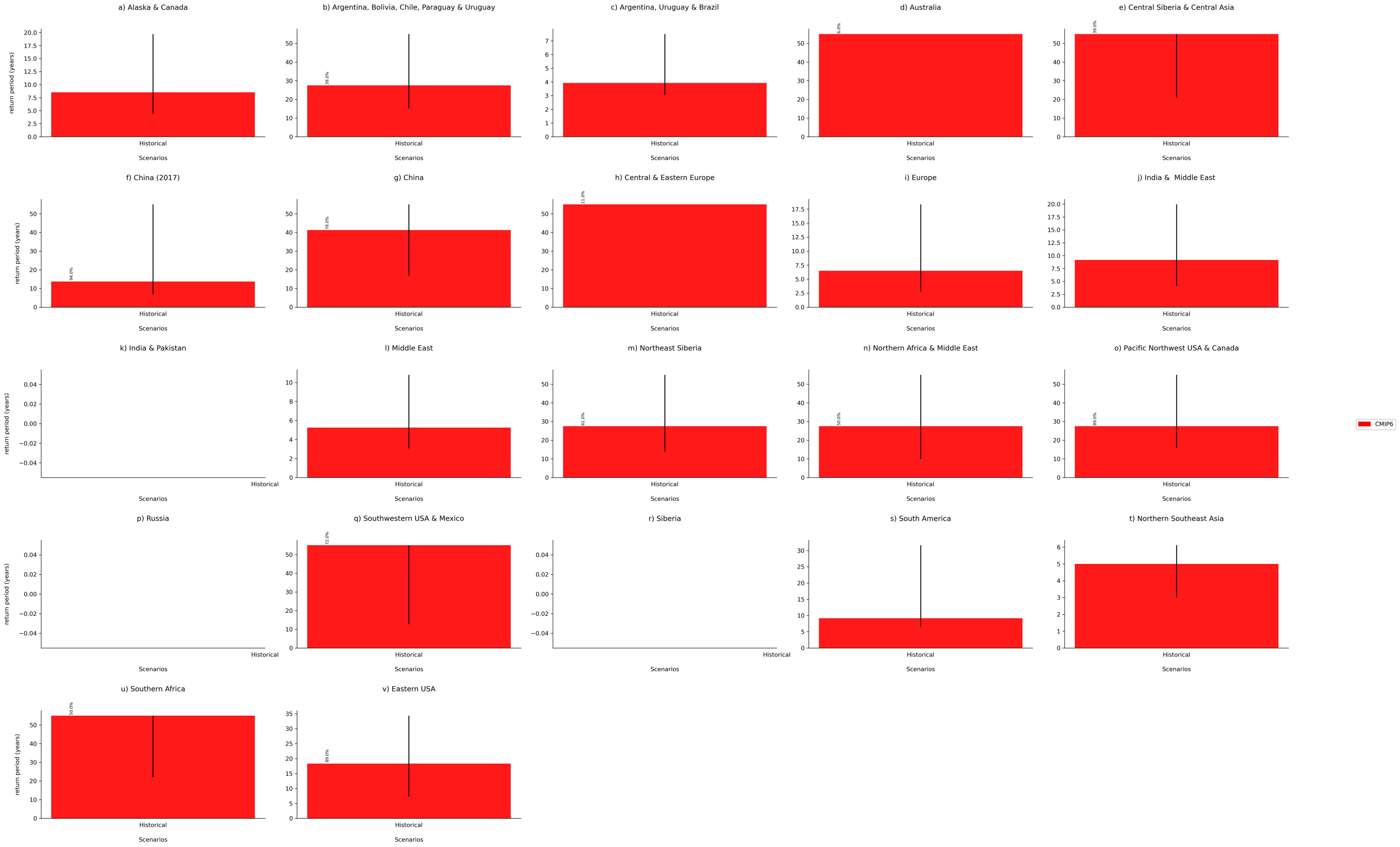

a) Alaska & Canada
b) Argentina, Bolivia, Chile, Paraguay & Uruguay
c) Argentina, Uruguay & Brazil
d) Australia
e) Central Siberia & Central Asia
f) China (2017)
g) China
h) Central & Eastern Europe
i) Europe
j) India & Middle East
k) India & Pakistan
l) Middle East
m) Northeast Siberia
n) Northern Africa & Middle East
o) Pacific Northwest USA & Canada
p) Russia
q) Southwestern USA & Mexico
r) Siberia
s) South America
t) Northern Southeast Asia
u) Southern Africa
v) Eastern USA
return period (years)
Historical
Scenarios
39.0%
6.0%
39.0%
94.0%
78.0%
11.0%
61.0%
50.0%
89.0%
72.0%
50.0%
89.0%
CMIP6

Supplementary Figure 389: Return times (RT) during present-to-near-future (2015-2054) based on ERA5 (Tx95.0p) - Return times (RT) (in years) during the present-to-near-future period between 2015 and 2054 for Maximum Heatwave Area (%) of the respective observed event. For each region, values are shown for the different scenarios (increasing in emissions from left to right for the CMIP5 (green), CMIP6 (red), CanESM5 (dark blue), and ACCESS-ESM1-5 (light blue) ensembles. The error bars indicate the 5th and 95th percentile of the respective sample. Where the respective ensemble contains models not able to simulate the magnitude of the respective observed event's Maximum Heatwave Area (%), the proportion of models that are able to simulate an event with a value of Maximum Heatwave Area (%) at least as high as the observed event is indicated by the percentage values above the individual bars. The observed reference values are calculated using data from ERA5.

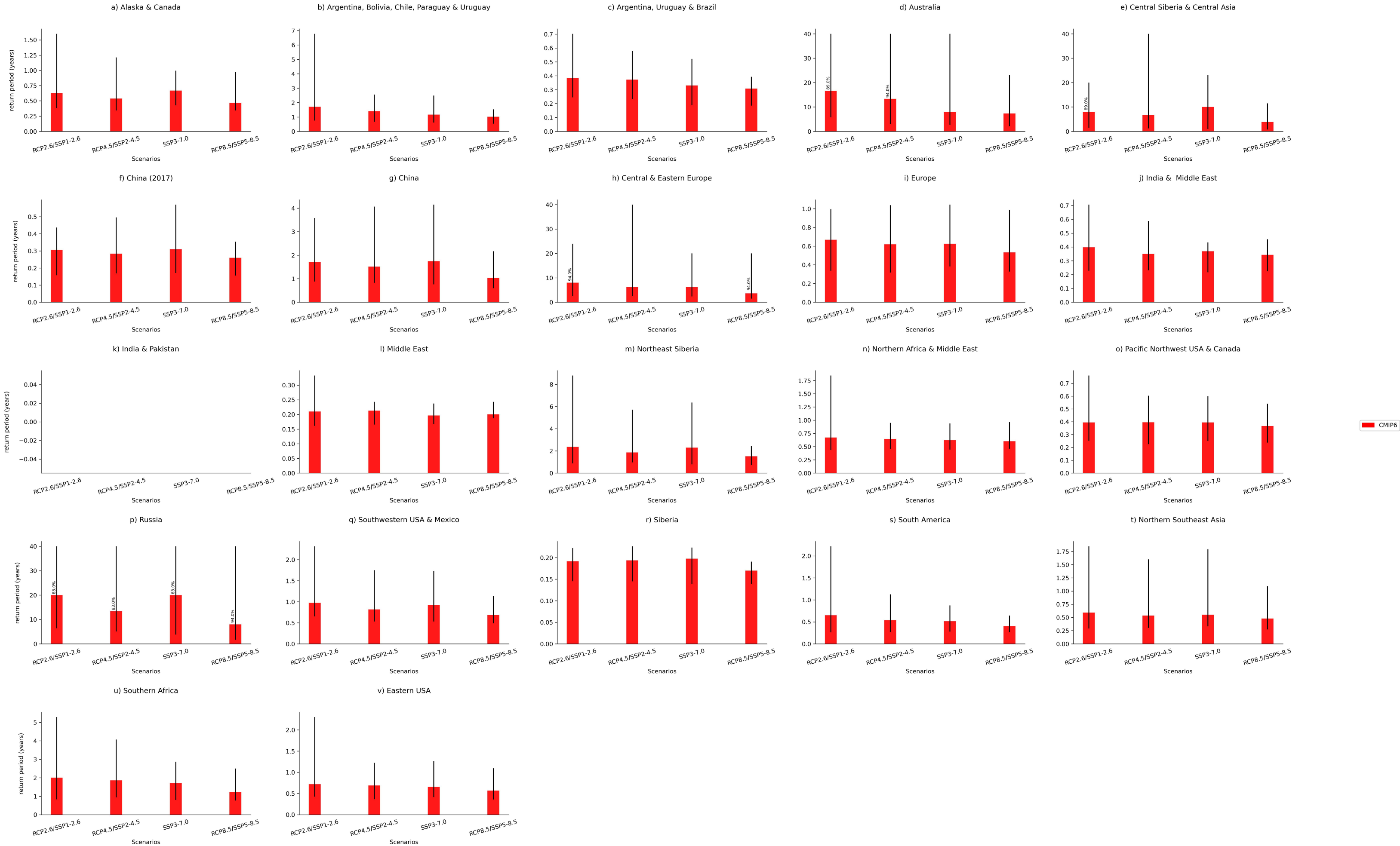

a) Alaska & Canada
b) Argentina, Bolivia, Chile, Paraguay & Uruguay
c) Argentina, Uruguay & Brazil
d) Australia
e) Central Siberia & Central Asia
f) China (2017)
g) China
h) Central & Eastern Europe
i) Europe
j) India & Middle East
k) India & Pakistan
l) Middle East
m) Northeast Siberia
n) Northern Africa & Middle East
o) Pacific Northwest USA & Canada
p) Russia
q) Southwestern USA & Mexico
r) Siberia
s) South America
t) Northern Southeast Asia
u) Southern Africa
v) Eastern USA
return period (years)
Scenarios
RCP2.6/SSP1-2.6
RCP4.5/SSP2-4.5
SSP3-7.0
RCP8.5/SSP5-8.5
89.0%
94.0%
83.0%
CMIP6

Supplementary Figure 390: Return times (RT) during present-to-near-future (2015-2054) based on ERA5 (Tx97.5p) - Return times (RT) (in years) during the present-to-near-future period between 2015 and 2054 for Maximum Heatwave Area (%) of the respective observed event. For each region, values are shown for the different scenarios (increasing in emissions from left to right for the CMIP5 (green), CMIP6 (red), CanESM5 (dark blue), and ACCESS-ESM1-5 (light blue) ensembles. The error bars indicate the 5th and 95th percentile of the respective sample. Where the respective ensemble contains models not able to simulate the magnitude of the respective observed event's Maximum Heatwave Area (%), the proportion of models that are able to simulate an event with a value of Maximum Heatwave Area (%) at least as high as the observed event is indicated by the percentage values above the individual bars. The observed reference values are calculated using data from ERA5.

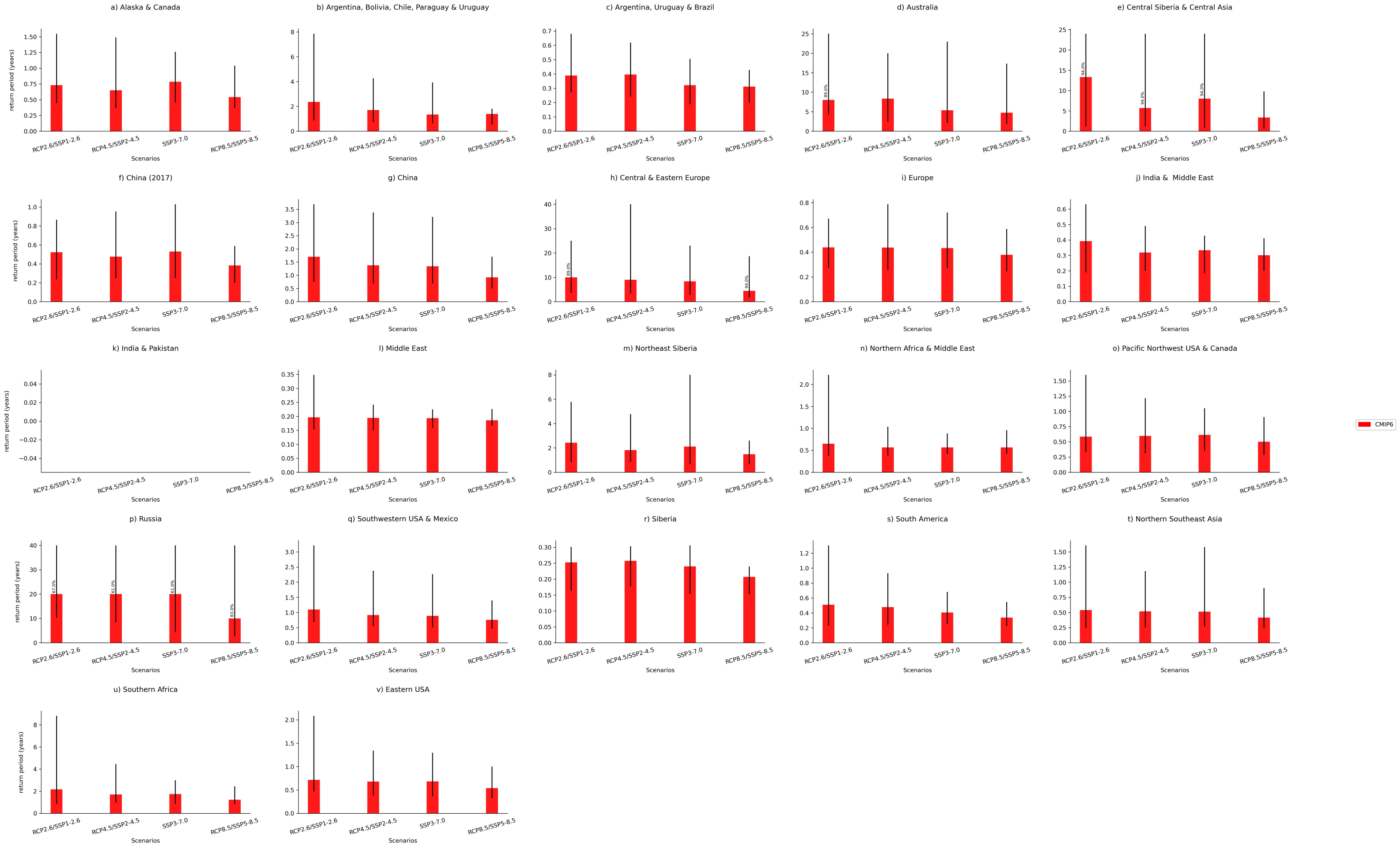

a) Alaska & Canada
b) Argentina, Bolivia, Chile, Paraguay & Uruguay
c) Argentina, Uruguay & Brazil
d) Australia
e) Central Siberia & Central Asia
f) China (2017)
g) China
h) Central & Eastern Europe
i) Europe
j) India & Middle East
k) India & Pakistan
l) Middle East
m) Northeast Siberia
n) Northern Africa & Middle East
o) Pacific Northwest USA & Canada
p) Russia
q) Southwestern USA & Mexico
r) Siberia
s) South America
t) Northern Southeast Asia
u) Southern Africa
v) Eastern USA
return period (years)
Scenarios
RCP2.6/SSP1-2.6
RCP4.5/SSP2-4.5
SSP3-7.0
RCP8.5/SSP5-8.5
CMIP6

Supplementary Figure 391: Return times (RT) during present-to-near-future (2015-2054) based on ERA5 (Tx99.0p) - Return times (RT) (in years) during the present-to-near-future period between 2015 and 2054 for Maximum Heatwave Area (%) of the respective observed event. For each region, values are shown for the different scenarios (increasing in emissions from left to right for the CMIP5 (green), CMIP6 (red), CanESM5 (dark blue), and ACCESS-ESM1-5 (light blue) ensembles. The error bars indicate the 5th and 95th percentile of the respective sample. Where the respective ensemble contains models not able to simulate the magnitude of the respective observed event's Maximum Heatwave Area (%), the proportion of models that are able to simulate an event with a value of Maximum Heatwave Area (%) at least as high as the observed event is indicated by the percentage values above the individual bars. The observed reference values are calculated using data from ERA5.

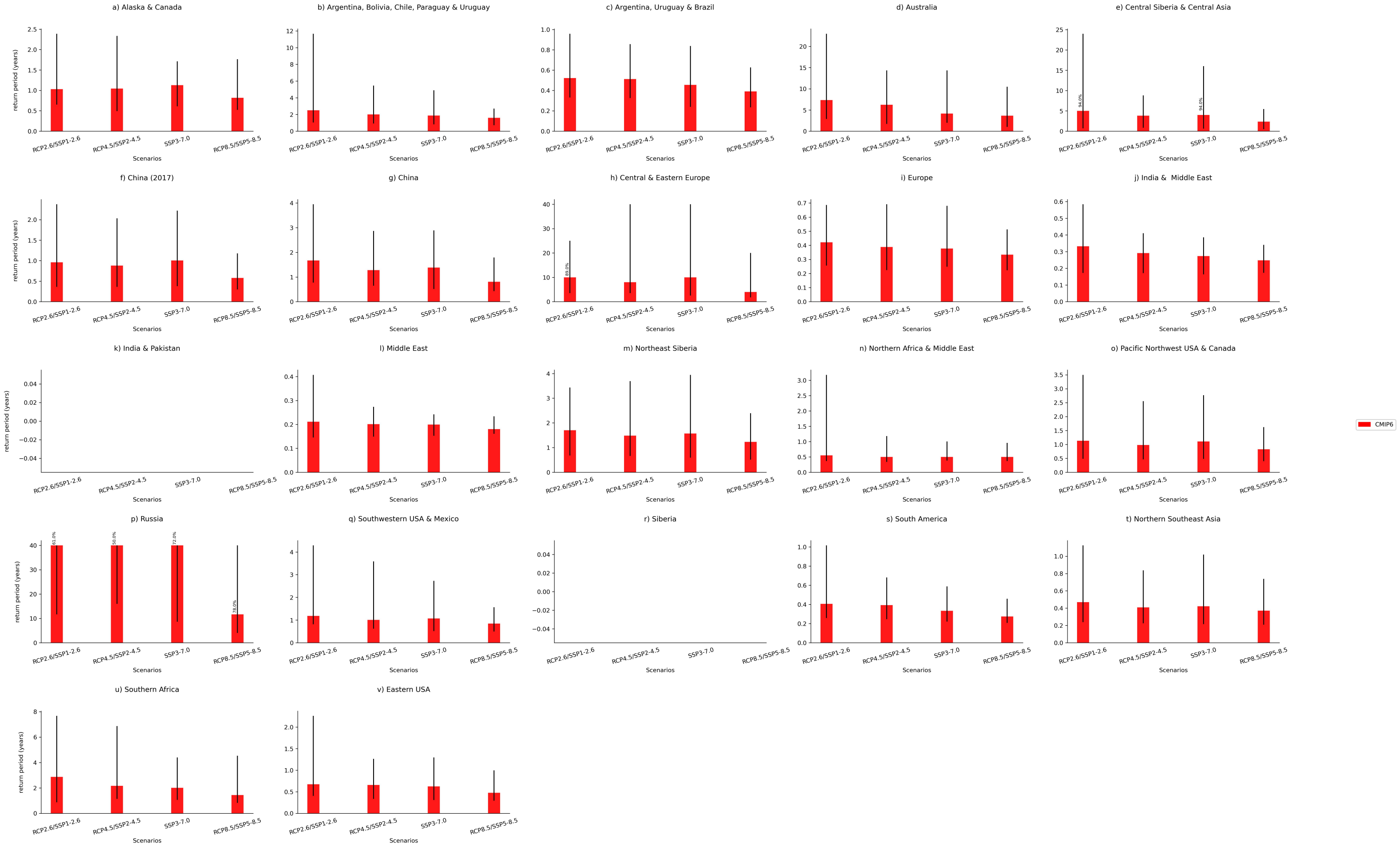

a) Alaska & Canada
b) Argentina, Bolivia, Chile, Paraguay & Uruguay
c) Argentina, Uruguay & Brazil
d) Australia
e) Central Siberia & Central Asia
f) China (2017)
g) China
h) Central & Eastern Europe
i) Europe
j) India & Middle East
k) India & Pakistan
l) Middle East
m) Northeast Siberia
n) Northern Africa & Middle East
o) Pacific Northwest USA & Canada
p) Russia
q) Southwestern USA & Mexico
r) Siberia
s) South America
t) Northern Southeast Asia
u) Southern Africa
v) Eastern USA
return period (years)
Scenarios
RCP2.6/SSP1-2.6
RCP4.5/SSP2-4.5
SSP3-7.0
RCP8.5/SSP5-8.5
94.0%
94.0%
89.0%
61.0%
50.0%
72.0%
78.0%
CMIP6

Supplementary Figure 392: Return times (RT) during end-of-century (2060-2099) based on ERA5 (Tx95.0p) - Return times (RT) (in years) during the end-of-century period between 2060 and 2099 for Maximum Heatwave Area (%) of the respective observed event. For each region, values are shown for the different scenarios (increasing in emissions from left to right for the CMIP5 (green), CMIP6 (red), CanESM5 (dark blue), and ACCESS-ESM1-5 (light blue) ensembles. The error bars indicate the 5th and 95th percentile of the respective sample. Where the respective ensemble contains models not able to simulate the magnitude of the respective observed event's Maximum Heatwave Area (%), the proportion of models that are able to simulate an event with a value of Maximum Heatwave Area (%) at least as high as the observed event is indicated by the percentage values above the individual bars. The observed reference values are calculated using data from ERA5.

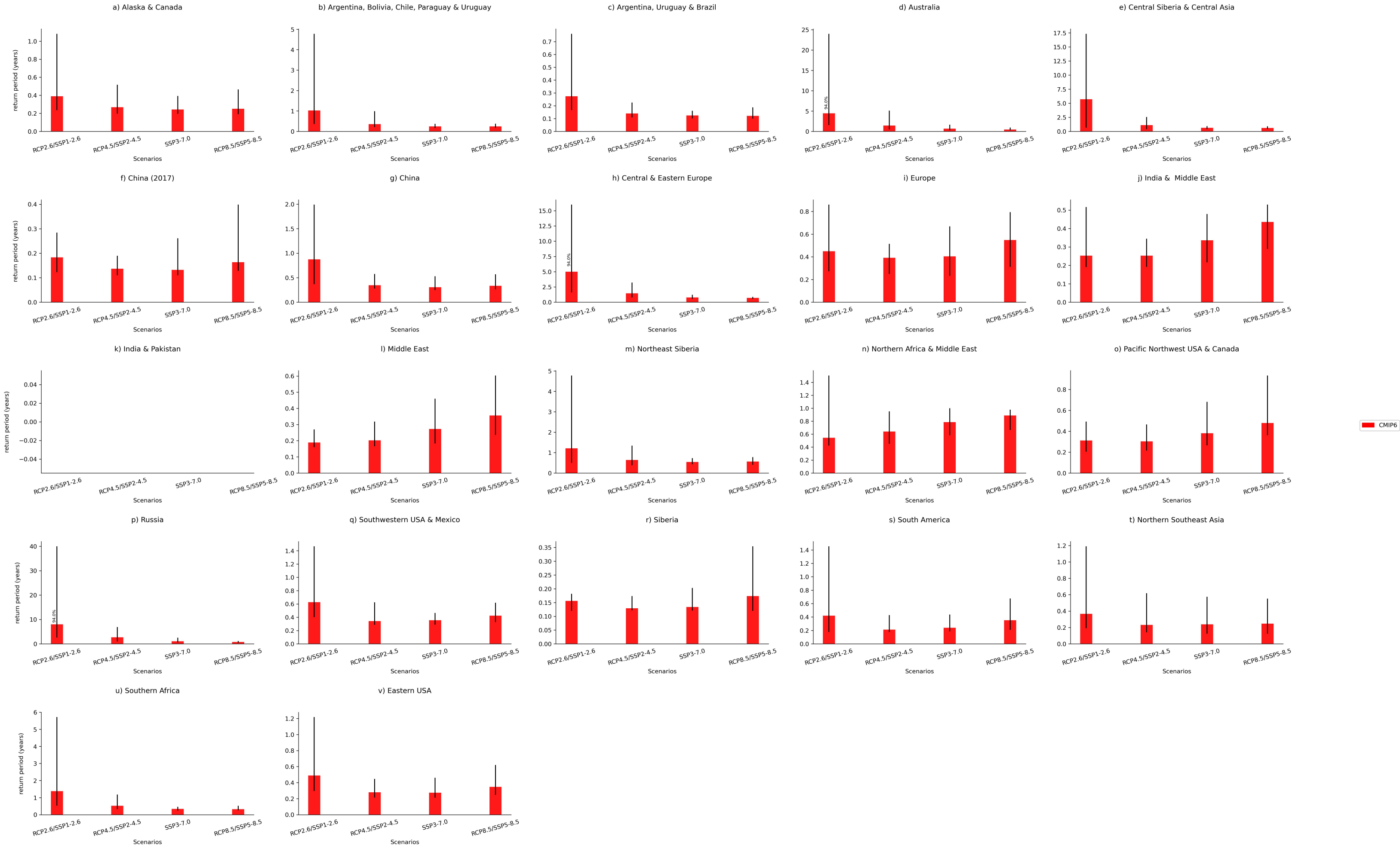
a) Alaska & Canada
b) Argentina, Bolivia, Chile, Paraguay & Uruguay
c) Argentina, Uruguay & Brazil
d) Australia
e) Central Siberia & Central Asia
f) China (2017)
g) China
h) Central & Eastern Europe
i) Europe
j) India & Middle East
k) India & Pakistan
l) Middle East
m) Northeast Siberia
n) Northern Africa & Middle East
o) Pacific Northwest USA & Canada
p) Russia
q) Southwestern USA & Mexico
r) Siberia
s) South America
t) Northern Southeast Asia
u) Southern Africa
v) Eastern USA
return period (years)
Scenarios
RCP2.6/SSP1-2.6
RCP4.5/SSP2-4.5
SSP3-7.0
RCP8.5/SSP5-8.5
94.0%
CMIP6

Supplementary Figure 393: Return times (RT) during end-of-century (2060-2099) based on ERA5 (Tx97.5p) - Return times (RT) (in years) during the end-of-century period between 2060 and 2099 for Maximum Heatwave Area (%) of the respective observed event. For each region, values are shown for the different scenarios (increasing in emissions from left to right for the CMIP5 (green), CMIP6 (red), CanESM5 (dark blue), and ACCESS-ESM1-5 (light blue) ensembles. The error bars indicate the 5th and 95th percentile of the respective sample. Where the respective ensemble contains models not able to simulate the magnitude of the respective observed event's Maximum Heatwave Area (%), the proportion of models that are able to simulate an event with a value of Maximum Heatwave Area (%) at least as high as the observed event is indicated by the percentage values above the individual bars. The observed reference values are calculated using data from ERA5.

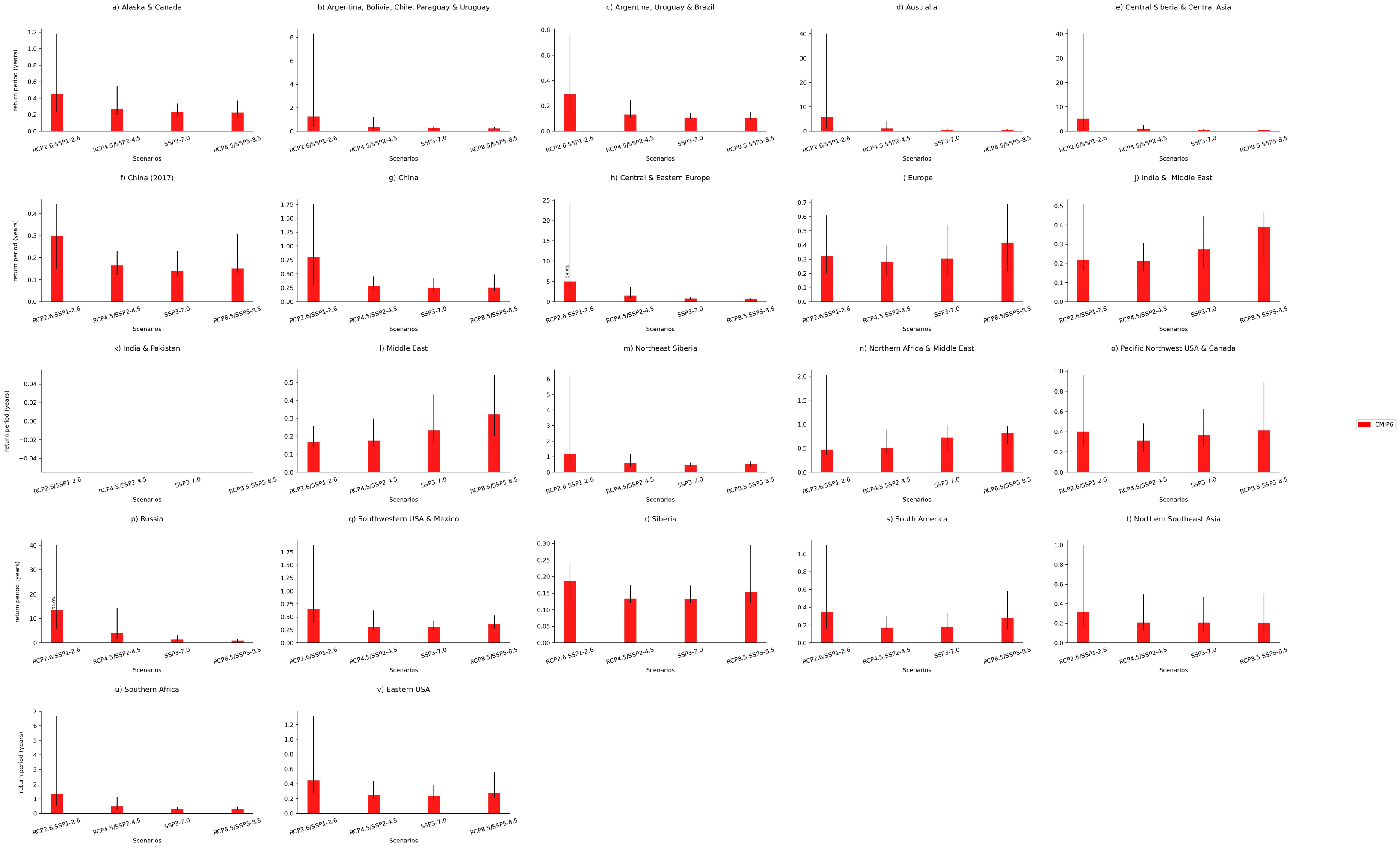
a) Alaska & Canada
b) Argentina, Bolivia, Chile, Paraguay & Uruguay
c) Argentina, Uruguay & Brazil
d) Australia
e) Central Siberia & Central Asia
f) China (2017)
g) China
h) Central & Eastern Europe
i) Europe
j) India & Middle East
k) India & Pakistan
l) Middle East
m) Northeast Siberia
n) Northern Africa & Middle East
o) Pacific Northwest USA & Canada
p) Russia
q) Southwestern USA & Mexico
r) Siberia
s) South America
t) Northern Southeast Asia
u) Southern Africa
v) Eastern USA
return period (years)
Scenarios
RCP2.6/SSP1-2.6
RCP4.5/SSP2-4.5
SSP3-7.0
RCP8.5/SSP5-8.5
94.0%
CMIP6

Supplementary Figure 394: Return times (RT) during end-of-century (2060-2099) based on ERA5 (Tx99.0p) - Return times (RT) (in years) during the end-of-century period between 2060 and 2099 for Maximum Heatwave Area (%) of the respective observed event. For each region, values are shown for the different scenarios (increasing in emissions from left to right for the CMIP5 (green), CMIP6 (red), CanESM5 (dark blue), and ACCESS-ESM1-5 (light blue) ensembles. The error bars indicate the 5th and 95th percentile of the respective sample. Where the respective ensemble contains models not able to simulate the magnitude of the respective observed event's Maximum Heatwave Area (%), the proportion of models that are able to simulate an event with a value of Maximum Heatwave Area (%) at least as high as the observed event is indicated by the percentage values above the individual bars. The observed reference values are calculated using data from ERA5.

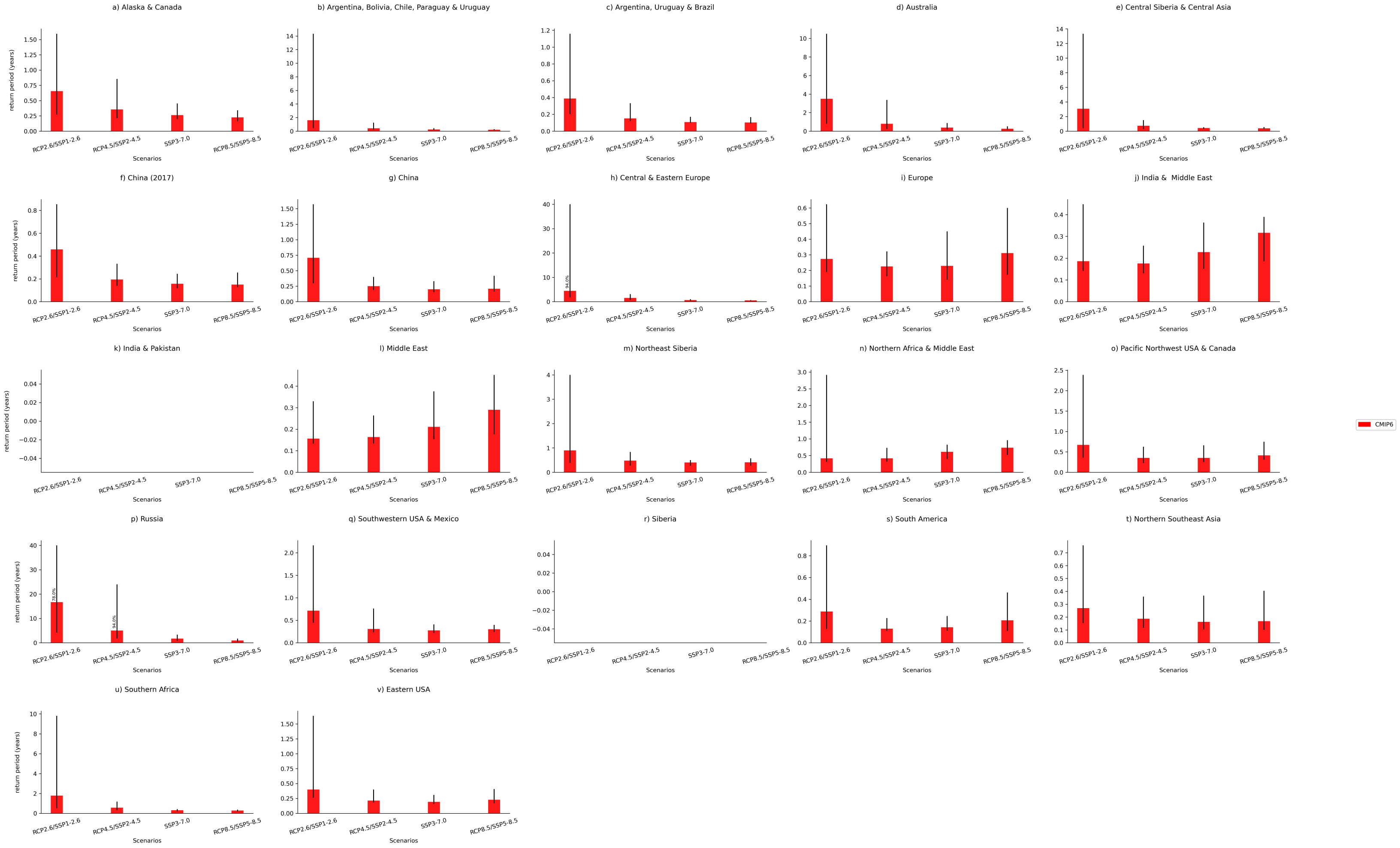
a) Alaska & Canada
b) Argentina, Bolivia, Chile, Paraguay & Uruguay
c) Argentina, Uruguay & Brazil
d) Australia
e) Central Siberia & Central Asia
f) China (2017)
g) China
h) Central & Eastern Europe
i) Europe
j) India & Middle East
k) India & Pakistan
l) Middle East
m) Northeast Siberia
n) Northern Africa & Middle East
o) Pacific Northwest USA & Canada
p) Russia
q) Southwestern USA & Mexico
r) Siberia
s) South America
t) Northern Southeast Asia
u) Southern Africa
v) Eastern USA
return period (years)
Scenarios
RCP2.6/SSP1-2.6
RCP4.5/SSP2-4.5
SSP3-7.0
RCP8.5/SSP5-8.5
94.0%
78.0%
94.0%
CMIP6

Supplementary Figure 395: Return times (RT) during historical (1951-2005) based on Berkeley Earth (Tx95.0p) - Return times (RT) (in years) during the historical period between 1951 and 2005 for Maximum Heatwave Severity of the respective observed event. For each region, values are shown for the different scenarios (increasing in emissions from left to right for the CMIP5 (green), CMIP6 (red), CanESM5 (dark blue), and ACCESS-ESM1-5 (light blue) ensembles. The error bars indicate the 5th and 95th percentile of the respective sample. Where the respective ensemble contains models not able to simulate the magnitude of the respective observed event's Maximum Heatwave Severity, the proportion of models that are able to simulate an event with a value of Maximum Heatwave Severity at least as high as the observed event is indicated by the percentage values above the individual bars. The observed reference values are calculated using data from Berkeley Earth.

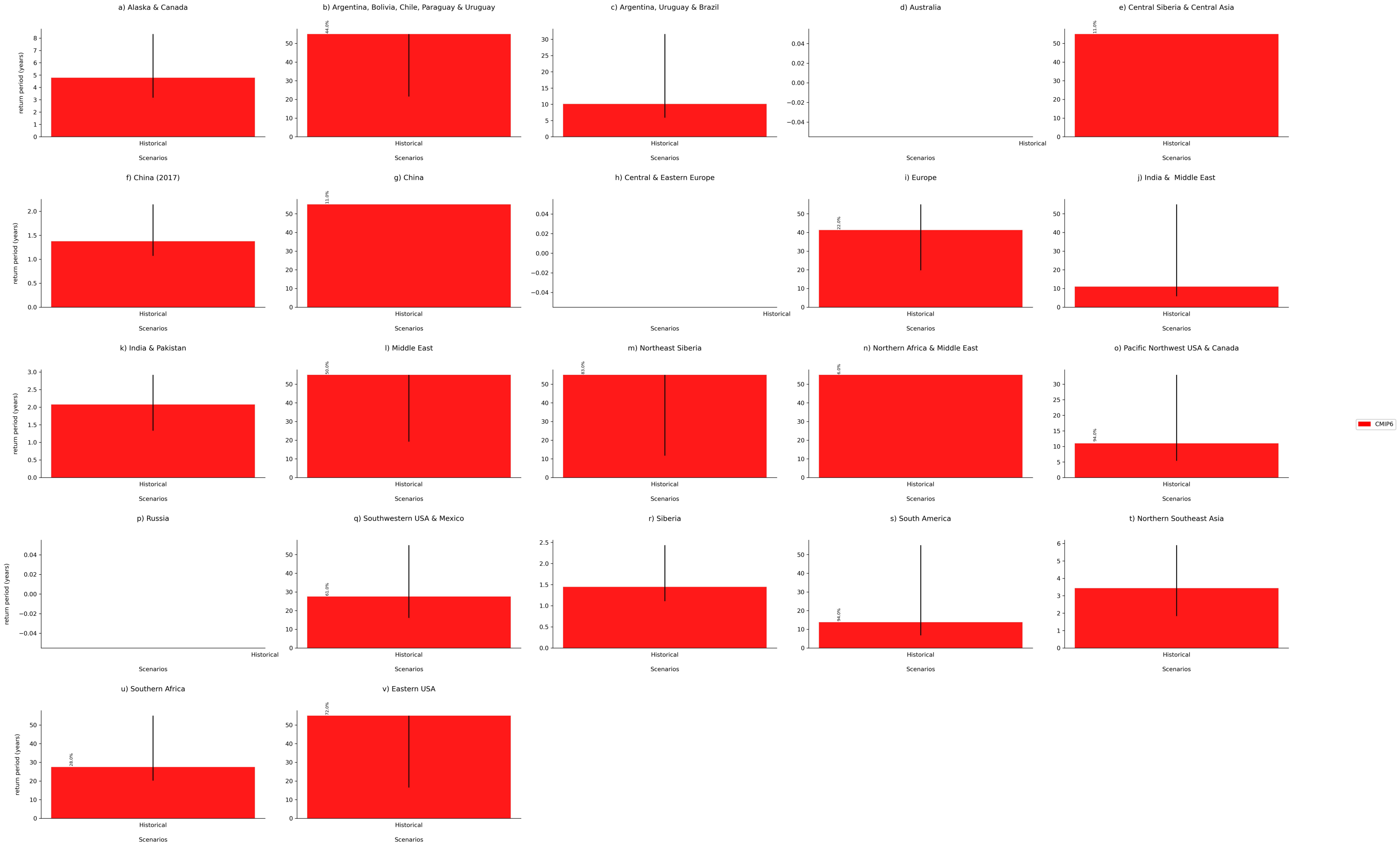

a) Alaska & Canada
b) Argentina, Bolivia, Chile, Paraguay & Uruguay
c) Argentina, Uruguay & Brazil
d) Australia
e) Central Siberia & Central Asia
f) China (2017)
g) China
h) Central & Eastern Europe
i) Europe
j) India & Middle East
k) India & Pakistan
l) Middle East
m) Northeast Siberia
n) Northern Africa & Middle East
o) Pacific Northwest USA & Canada
p) Russia
q) Southwestern USA & Mexico
r) Siberia
s) South America
t) Northern Southeast Asia
u) Southern Africa
v) Eastern USA
return period (years)
Historical
Scenarios
CMIP6

Supplementary Figure 396: Return times (RT) during historical (1951-2005) based on Berkeley Earth (Tx97.5p) - Return times (RT) (in years) during the historical period between 1951 and 2005 for Maximum Heatwave Severity of the respective observed event. For each region, values are shown for the different scenarios (increasing in emissions from left to right for the CMIP5 (green), CMIP6 (red), CanESM5 (dark blue), and ACCESS-ESM1-5 (light blue) ensembles. The error bars indicate the 5th and 95th percentile of the respective sample. Where the respective ensemble contains models not able to simulate the magnitude of the respective observed event's Maximum Heatwave Severity, the proportion of models that are able to simulate an event with a value of Maximum Heatwave Severity at least as high as the observed event is indicated by the percentage values above the individual bars. The observed reference values are calculated using data from Berkeley Earth.

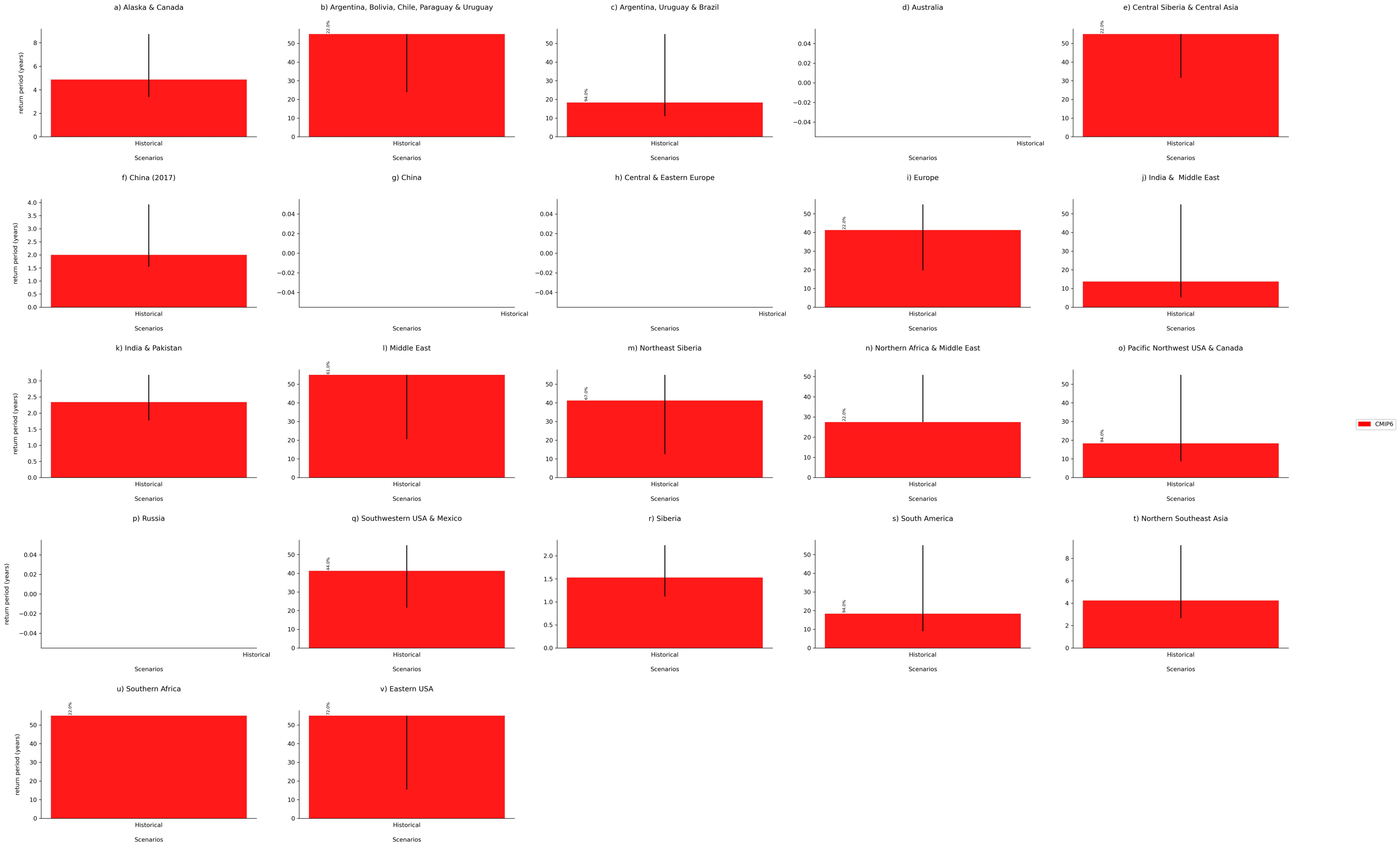

a) Alaska & Canada
b) Argentina, Bolivia, Chile, Paraguay & Uruguay
c) Argentina, Uruguay & Brazil
d) Australia
e) Central Siberia & Central Asia
f) China (2017)
g) China
h) Central & Eastern Europe
i) Europe
j) India & Middle East
k) India & Pakistan
l) Middle East
m) Northeast Siberia
n) Northern Africa & Middle East
o) Pacific Northwest USA & Canada
p) Russia
q) Southwestern USA & Mexico
r) Siberia
s) South America
t) Northern Southeast Asia
u) Southern Africa
v) Eastern USA
return period (years)
Historical
Scenarios
CMIP6
22.0%
94.0%
22.0%
22.0%
61.0%
67.0%
22.0%
94.0%
44.0%
94.0%
22.0%
72.0%

Supplementary Figure 397: Return times (RT) during historical (1951-2005) based on Berkeley Earth (Tx99.0p) - Return times (RT) (in years) during the historical period between 1951 and 2005 for Maximum Heatwave Severity of the respective observed event. For each region, values are shown for the different scenarios (increasing in emissions from left to right for the CMIP5 (green), CMIP6 (red), CanESM5 (dark blue), and ACCESS-ESM1-5 (light blue) ensembles. The error bars indicate the 5th and 95th percentile of the respective sample. Where the respective ensemble contains models not able to simulate the magnitude of the respective observed event's Maximum Heatwave Severity, the proportion of models that are able to simulate an event with a value of Maximum Heatwave Severity at least as high as the observed event is indicated by the percentage values above the individual bars. The observed reference values are calculated using data from Berkeley Earth.

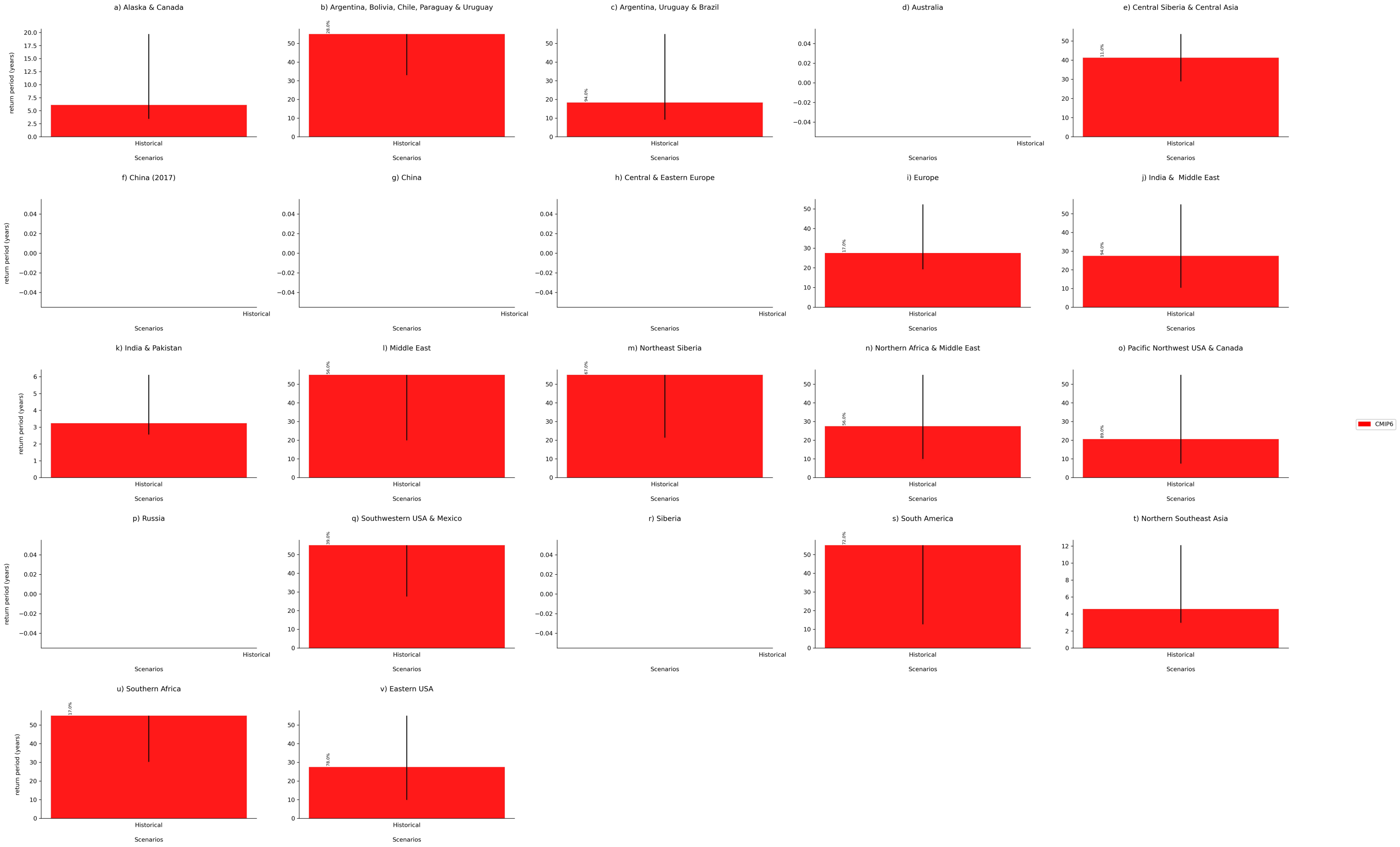

a) Alaska & Canada
b) Argentina, Bolivia, Chile, Paraguay & Uruguay
c) Argentina, Uruguay & Brazil
d) Australia
e) Central Siberia & Central Asia
f) China (2017)
g) China
h) Central & Eastern Europe
i) Europe
j) India & Middle East
k) India & Pakistan
l) Middle East
m) Northeast Siberia
n) Northern Africa & Middle East
o) Pacific Northwest USA & Canada
p) Russia
q) Southwestern USA & Mexico
r) Siberia
s) South America
t) Northern Southeast Asia
u) Southern Africa
v) Eastern USA
return period (years)
Scenarios
Historical
28.0%
94.0%
11.0%
17.0%
94.0%
56.0%
67.0%
56.0%
89.0%
39.0%
72.0%
17.0%
78.0%
CMIP6

Supplementary Figure 398: Return times (RT) during present-to-near-future (2015-2054) based on Berkeley Earth (Tx95.0p) - Return times (RT) (in years) during the present-to-near-future period between 2015 and 2054 for Maximum Heatwave Severity of the respective observed event. For each region, values are shown for the different scenarios (increasing in emissions from left to right for the CMIP5 (green), CMIP6 (red), CanESM5 (dark blue), and ACCESS-ESM1-5 (light blue) ensembles. The error bars indicate the 5th and 95th percentile of the respective sample. Where the respective ensemble contains models not able to simulate the magnitude of the respective observed event's Maximum Heatwave Severity, the proportion of models that are able to simulate an event with a value of Maximum Heatwave Severity at least as high as the observed event is indicated by the percentage values above the individual bars. The observed reference values are calculated using data from Berkeley Earth.

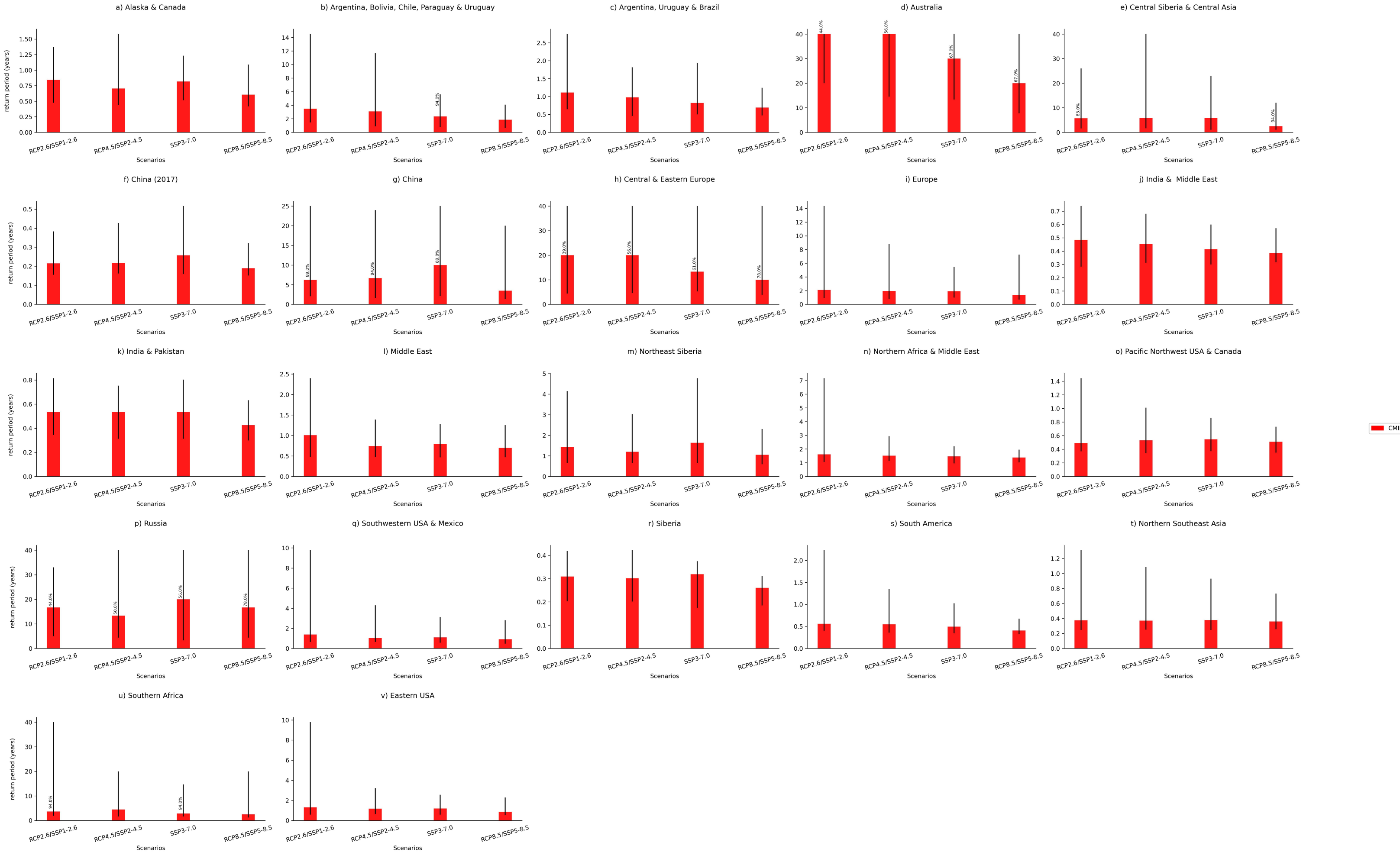
a) Alaska & Canada
b) Argentina, Bolivia, Chile, Paraguay & Uruguay
c) Argentina, Uruguay & Brazil
d) Australia
e) Central Siberia & Central Asia
f) China (2017)
g) China
h) Central & Eastern Europe
i) Europe
j) India & Middle East
k) India & Pakistan
l) Middle East
m) Northeast Siberia
n) Northern Africa & Middle East
o) Pacific Northwest USA & Canada
p) Russia
q) Southwestern USA & Mexico
r) Siberia
s) South America
t) Northern Southeast Asia
u) Southern Africa
v) Eastern USA
return period (years)
Scenarios
RCP2.6/SSP1-2.6
RCP4.5/SSP2-4.5
SSP3-7.0
RCP8.5/SSP5-8.5
CMIP6

Supplementary Figure 399: Return times (RT) during present-to-near-future (2015-2054) based on Berkeley Earth (Tx97.5p) - Return times (RT) (in years) during the present-to-near-future period between 2015 and 2054 for Maximum Heatwave Severity of the respective observed event. For each region, values are shown for the different scenarios (increasing in emissions from left to right for the CMIP5 (green), CMIP6 (red), CanESM5 (dark blue), and ACCESS-ESM1-5 (light blue) ensembles. The error bars indicate the 5th and 95th percentile of the respective sample. Where the respective ensemble contains models not able to simulate the magnitude of the respective observed event's Maximum Heatwave Severity, the proportion of models that are able to simulate an event with a value of Maximum Heatwave Severity at least as high as the observed event is indicated by the percentage values above the individual bars. The observed reference values are calculated using data from Berkeley Earth.

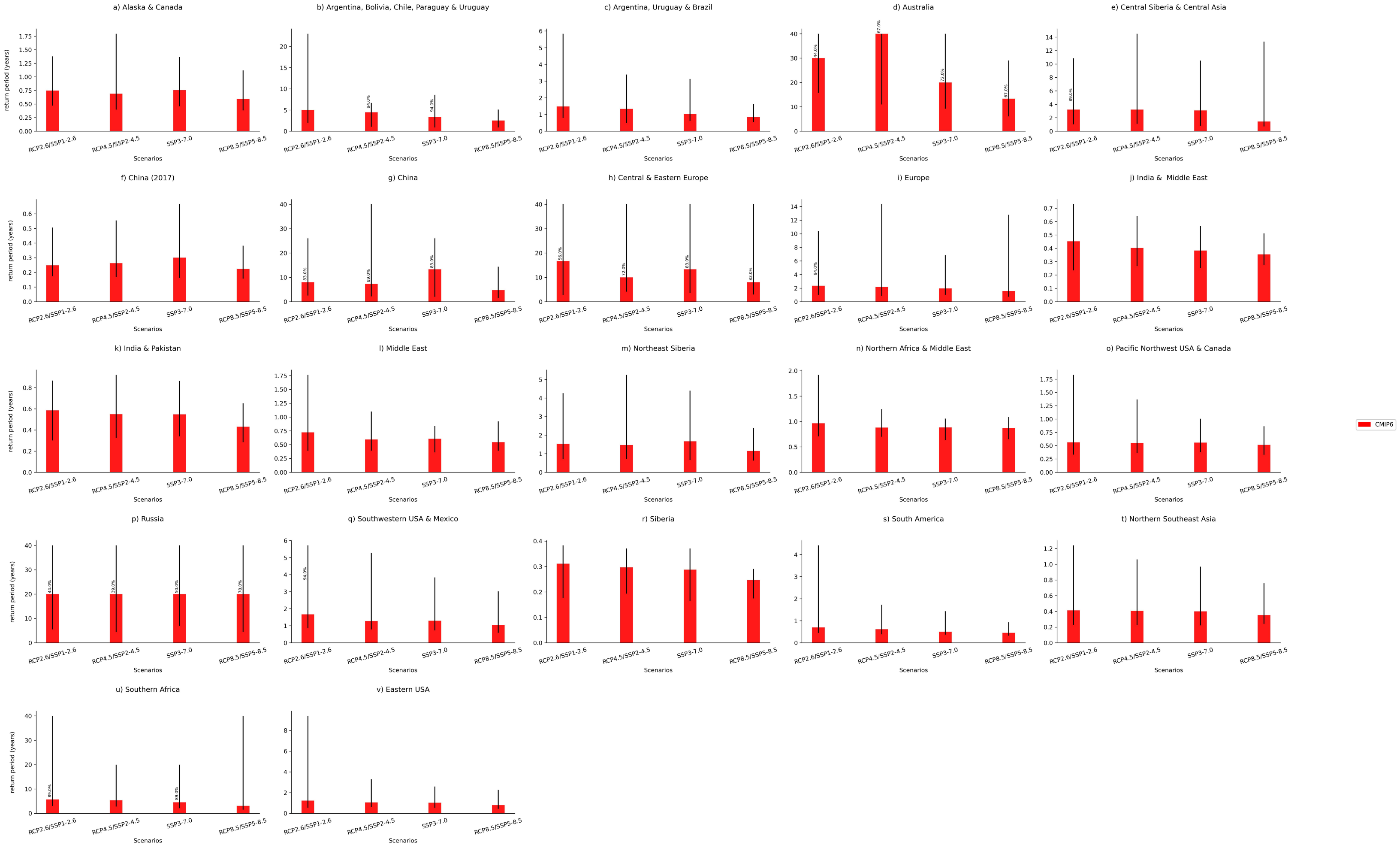
a) Alaska & Canada
b) Argentina, Bolivia, Chile, Paraguay & Uruguay
c) Argentina, Uruguay & Brazil
d) Australia
e) Central Siberia & Central Asia
f) China (2017)
g) China
h) Central & Eastern Europe
i) Europe
j) India & Middle East
k) India & Pakistan
l) Middle East
m) Northeast Siberia
n) Northern Africa & Middle East
o) Pacific Northwest USA & Canada
p) Russia
q) Southwestern USA & Mexico
r) Siberia
s) South America
t) Northern Southeast Asia
u) Southern Africa
v) Eastern USA
return period (years)
Scenarios
RCP2.6/SSP1-2.6
RCP4.5/SSP2-4.5
SSP3-7.0
RCP8.5/SSP5-8.5
CMIP6

Supplementary Figure 400: Return times (RT) during present-to-near-future (2015-2054) based on Berkeley Earth (Tx99.0p) - Return times (RT) (in years) during the present-to-near-future period between 2015 and 2054 for Maximum Heatwave Severity of the respective observed event. For each region, values are shown for the different scenarios (increasing in emissions from left to right for the CMIP5 (green), CMIP6 (red), CanESM5 (dark blue), and ACCESS-ESM1-5 (light blue) ensembles. The error bars indicate the 5th and 95th percentile of the respective sample. Where the respective ensemble contains models not able to simulate the magnitude of the respective observed event's Maximum Heatwave Severity, the proportion of models that are able to simulate an event with a value of Maximum Heatwave Severity at least as high as the observed event is indicated by the percentage values above the individual bars. The observed reference values are calculated using data from Berkeley Earth.

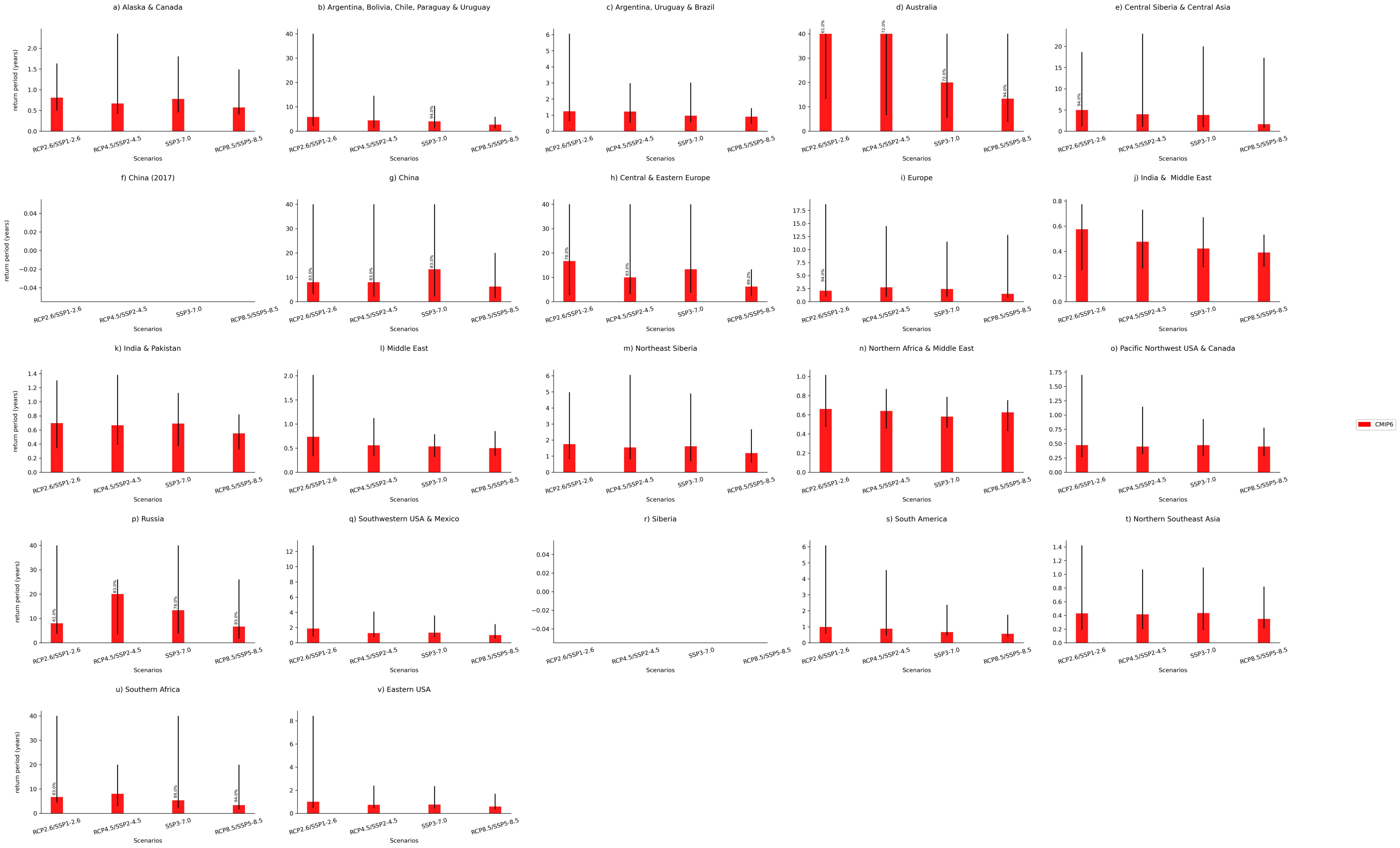

a) Alaska & Canada
b) Argentina, Bolivia, Chile, Paraguay & Uruguay
c) Argentina, Uruguay & Brazil
d) Australia
e) Central Siberia & Central Asia
f) China (2017)
g) China
h) Central & Eastern Europe
i) Europe
j) India & Middle East
k) India & Pakistan
l) Middle East
m) Northeast Siberia
n) Northern Africa & Middle East
o) Pacific Northwest USA & Canada
p) Russia
q) Southwestern USA & Mexico
r) Siberia
s) South America
t) Northern Southeast Asia
u) Southern Africa
v) Eastern USA
return period (years)
Scenarios
RCP2.6/SSP1-2.6
RCP4.5/SSP2-4.5
SSP3-7.0
RCP8.5/SSP5-8.5
CMIP6

Supplementary Figure 401: Return times (RT) during end-of-century (2060-2099) based on Berkeley Earth (Tx95.0p) - Return times (RT) (in years) during the end-of-century period between 2060 and 2099 for Maximum Heatwave Severity of the respective observed event. For each region, values are shown for the different scenarios (increasing in emissions from left to right for the CMIP5 (green), CMIP6 (red), CanESM5 (dark blue), and ACCESS-ESM1-5 (light blue) ensembles. The error bars indicate the 5th and 95th percentile of the respective sample. Where the respective ensemble contains models not able to simulate the magnitude of the respective observed event's Maximum Heatwave Severity, the proportion of models that are able to simulate an event with a value of Maximum Heatwave Severity at least as high as the observed event is indicated by the percentage values above the individual bars. The observed reference values are calculated using data from Berkeley Earth.

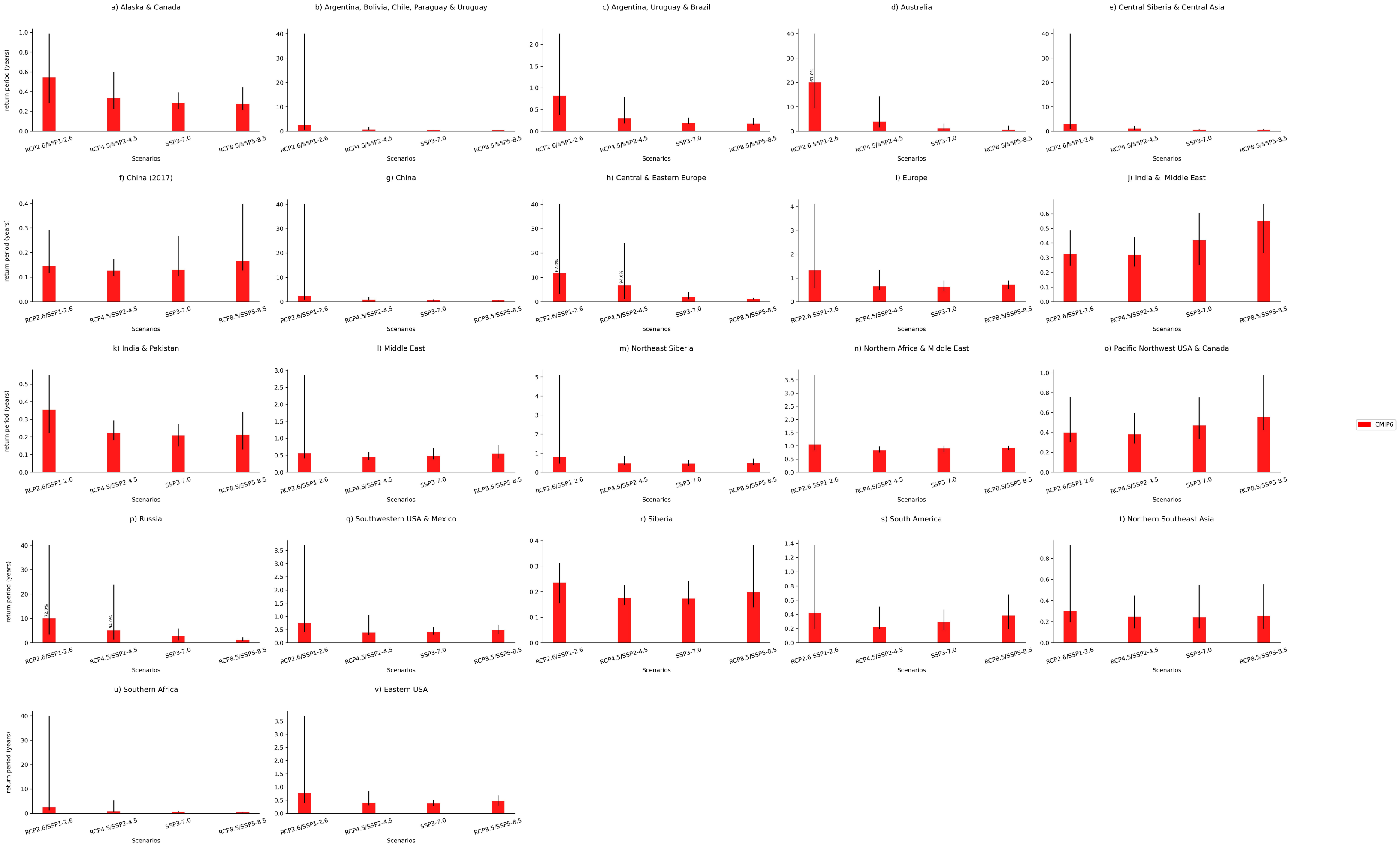
a) Alaska & Canada
b) Argentina, Bolivia, Chile, Paraguay & Uruguay
c) Argentina, Uruguay & Brazil
d) Australia
e) Central Siberia & Central Asia
f) China (2017)
g) China
h) Central & Eastern Europe
i) Europe
j) India & Middle East
k) India & Pakistan
l) Middle East
m) Northeast Siberia
n) Northern Africa & Middle East
o) Pacific Northwest USA & Canada
p) Russia
q) Southwestern USA & Mexico
r) Siberia
s) South America
t) Northern Southeast Asia
u) Southern Africa
v) Eastern USA
return period (years)
Scenarios
RCP2.6/SSP1-2.6
RCP4.5/SSP2-4.5
SSP3-7.0
RCP8.5/SSP5-8.5
61.0%
67.0%
94.0%
72.0%
94.0%
CMIP6

Supplementary Figure 402: Return times (RT) during end-of-century (2060-2099) based on Berkeley Earth (Tx97.5p) - Return times (RT) (in years) during the end-of-century period between 2060 and 2099 for Maximum Heatwave Severity of the respective observed event. For each region, values are shown for the different scenarios (increasing in emissions from left to right for the CMIP5 (green), CMIP6 (red), CanESM5 (dark blue), and ACCESS-ESM1-5 (light blue) ensembles. The error bars indicate the 5th and 95th percentile of the respective sample. Where the respective ensemble contains models not able to simulate the magnitude of the respective observed event's Maximum Heatwave Severity, the proportion of models that are able to simulate an event with a value of Maximum Heatwave Severity at least as high as the observed event is indicated by the percentage values above the individual bars. The observed reference values are calculated using data from Berkeley Earth.

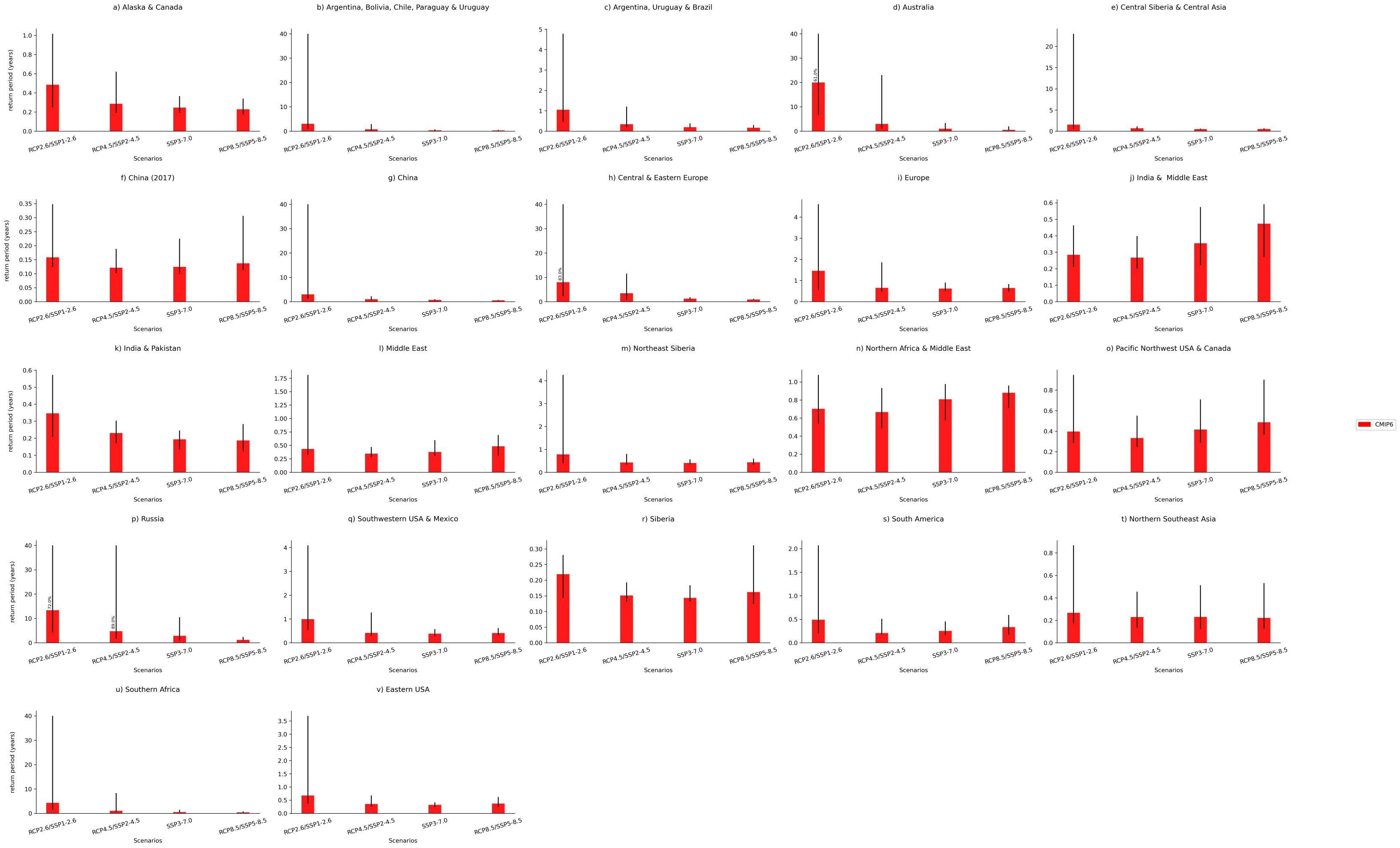
a) Alaska & Canada
b) Argentina, Bolivia, Chile, Paraguay & Uruguay
c) Argentina, Uruguay & Brazil
d) Australia
61.0%
e) Central Siberia & Central Asia
f) China (2017)
g) China
h) Central & Eastern Europe
83.0%
i) Europe
j) India & Middle East
k) India & Pakistan
l) Middle East
m) Northeast Siberia
n) Northern Africa & Middle East
o) Pacific Northwest USA & Canada
p) Russia
72.0%
89.0%
q) Southwestern USA & Mexico
r) Siberia
s) South America
t) Northern Southeast Asia
u) Southern Africa
v) Eastern USA
return period (years)
Scenarios
RCP2.6/SSP1-2.6
RCP4.5/SSP2-4.5
SSP3-7.0
RCP8.5/SSP5-8.5
CMIP6

Supplementary Figure 403: Return times (RT) during end-of-century (2060-2099) based on Berkeley Earth (Tx99.0p) - Return times (RT) (in years) during the end-of-century period between 2060 and 2099 for Maximum Heatwave Severity of the respective observed event. For each region, values are shown for the different scenarios (increasing in emissions from left to right for the CMIP5 (green), CMIP6 (red), CanESM5 (dark blue), and ACCESS-ESM1-5 (light blue) ensembles. The error bars indicate the 5th and 95th percentile of the respective sample. Where the respective ensemble contains models not able to simulate the magnitude of the respective observed event's Maximum Heatwave Severity, the proportion of models that are able to simulate an event with a value of Maximum Heatwave Severity at least as high as the observed event is indicated by the percentage values above the individual bars. The observed reference values are calculated using data from Berkeley Earth.

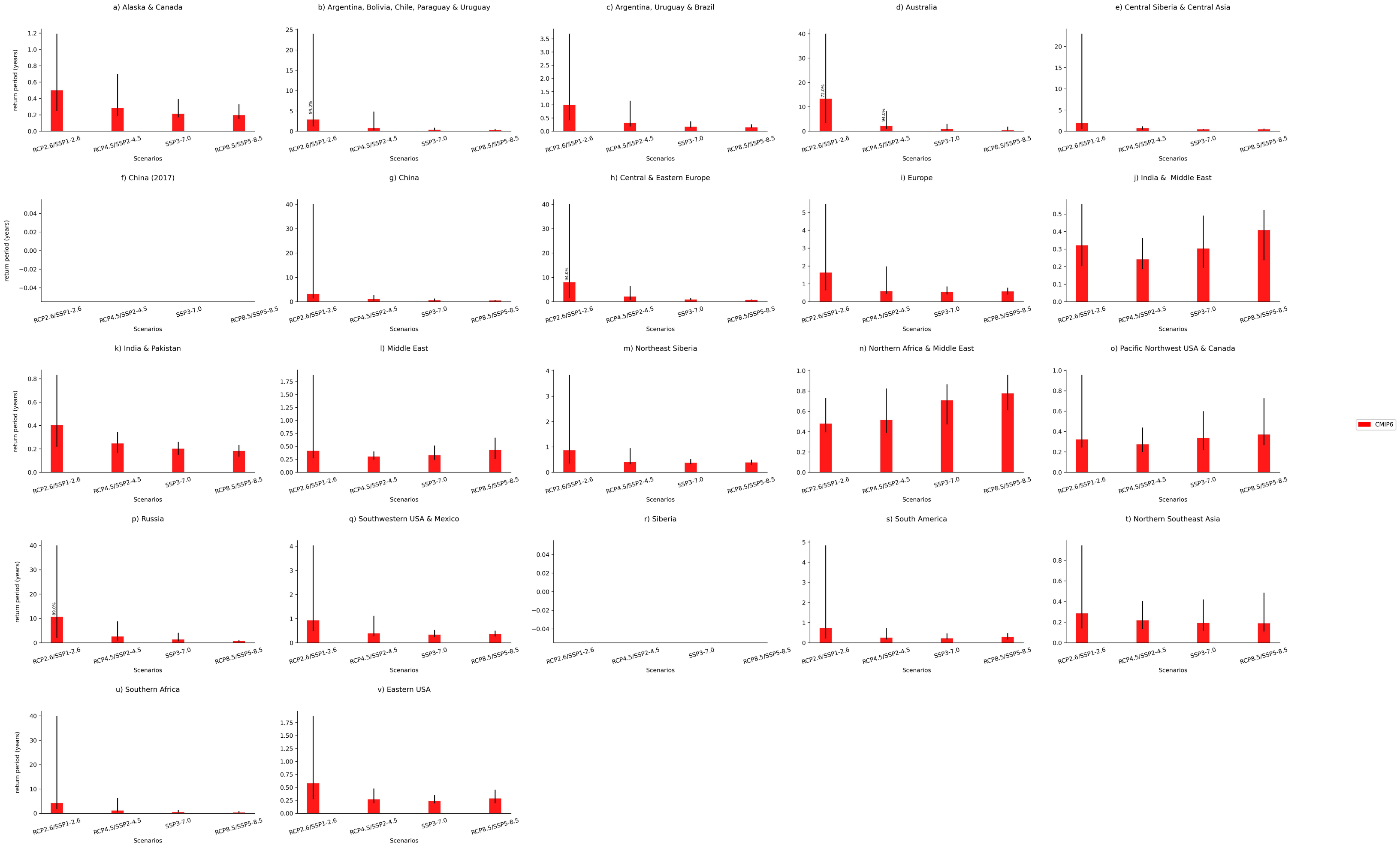
a) Alaska & Canada
b) Argentina, Bolivia, Chile, Paraguay & Uruguay
c) Argentina, Uruguay & Brazil
d) Australia
e) Central Siberia & Central Asia
f) China (2017)
g) China
h) Central & Eastern Europe
i) Europe
j) India & Middle East
k) India & Pakistan
l) Middle East
m) Northeast Siberia
n) Northern Africa & Middle East
o) Pacific Northwest USA & Canada
p) Russia
q) Southwestern USA & Mexico
r) Siberia
s) South America
t) Northern Southeast Asia
u) Southern Africa
v) Eastern USA
return period (years)
Scenarios
RCP2.6/SSP1-2.6
RCP4.5/SSP2-4.5
SSP3-7.0
RCP8.5/SSP5-8.5
94.0%
72.0%
89.0%
CMIP6

Supplementary Figure 404: Return times (RT) during historical (1951-2005) based on ERA5 (Tx95.0p) - Return times (RT) (in years) during the historical period between 1951 and 2005 for Maximum Heatwave Severity of the respective observed event. For each region, values are shown for the different scenarios (increasing in emissions from left to right for the CMIP5 (green), CMIP6 (red), CanESM5 (dark blue), and ACCESS-ESM1-5 (light blue) ensembles. The error bars indicate the 5th and 95th percentile of the respective sample. Where the respective ensemble contains models not able to simulate the magnitude of the respective observed event's Maximum Heatwave Severity, the proportion of models that are able to simulate an event with a value of Maximum Heatwave Severity at least as high as the observed event is indicated by the percentage values above the individual bars. The observed reference values are calculated using data from ERA5.

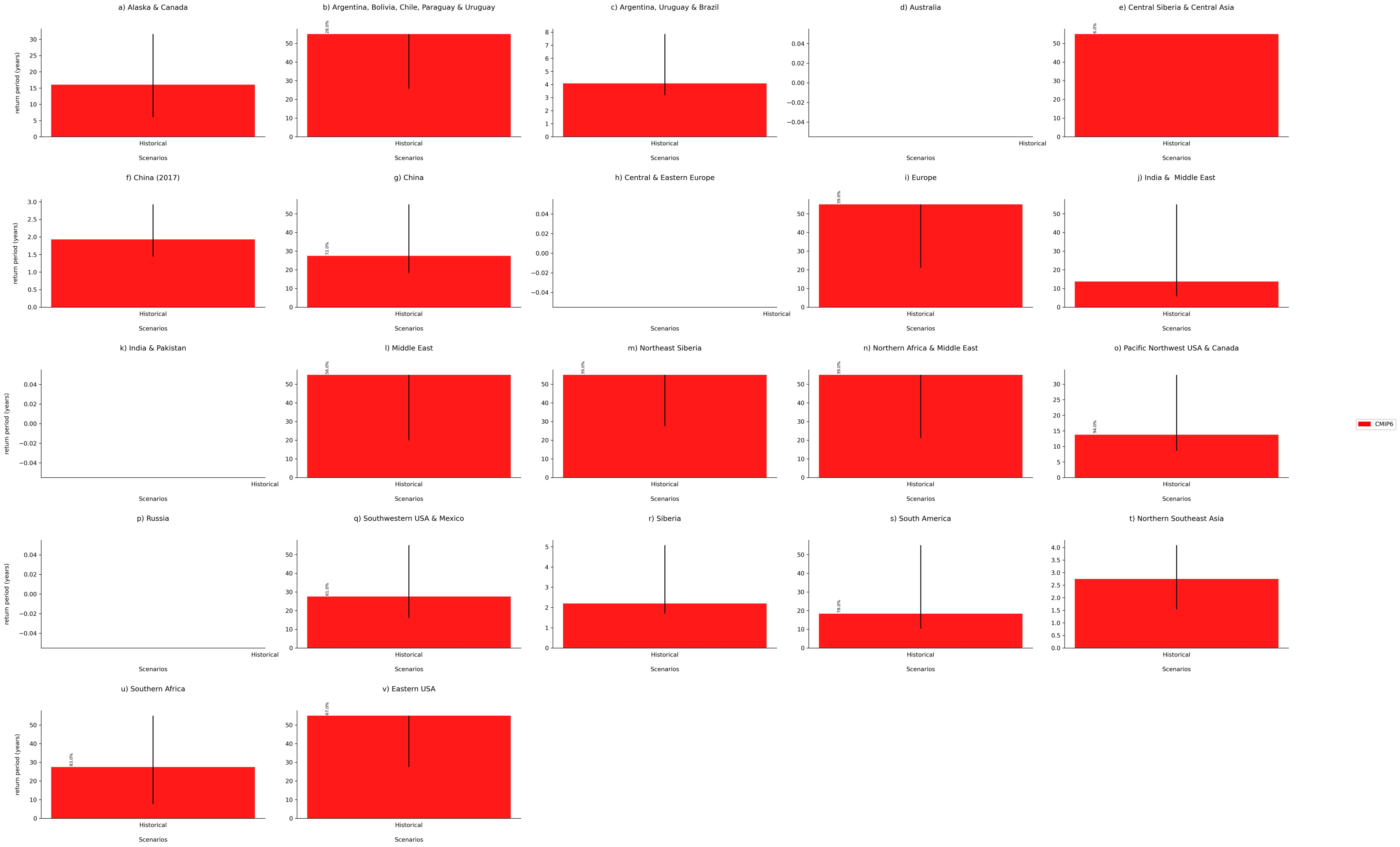

a) Alaska & Canada
b) Argentina, Bolivia, Chile, Paraguay & Uruguay
c) Argentina, Uruguay & Brazil
d) Australia
e) Central Siberia & Central Asia
f) China (2017)
g) China
h) Central & Eastern Europe
i) Europe
j) India & Middle East
k) India & Pakistan
l) Middle East
m) Northeast Siberia
n) Northern Africa & Middle East
o) Pacific Northwest USA & Canada
p) Russia
q) Southwestern USA & Mexico
r) Siberia
s) South America
t) Northern Southeast Asia
u) Southern Africa
v) Eastern USA
return period (years)
Historical
Scenarios
CMIP6
28.0%
6.0%
72.0%
39.0%
56.0%
39.0%
39.0%
94.0%
61.0%
78.0%
83.0%
67.0%

Supplementary Figure 405: Return times (RT) during historical (1951-2005) based on ERA5 (Tx97.5p) - Return times (RT) (in years) during the historical period between 1951 and 2005 for Maximum Heatwave Severity of the respective observed event. For each region, values are shown for the different scenarios (increasing in emissions from left to right for the CMIP5 (green), CMIP6 (red), CanESM5 (dark blue), and ACCESS-ESM1-5 (light blue) ensembles. The error bars indicate the 5th and 95th percentile of the respective sample. Where the respective ensemble contains models not able to simulate the magnitude of the respective observed event's Maximum Heatwave Severity, the proportion of models that are able to simulate an event with a value of Maximum Heatwave Severity at least as high as the observed event is indicated by the percentage values above the individual bars. The observed reference values are calculated using data from ERA5.

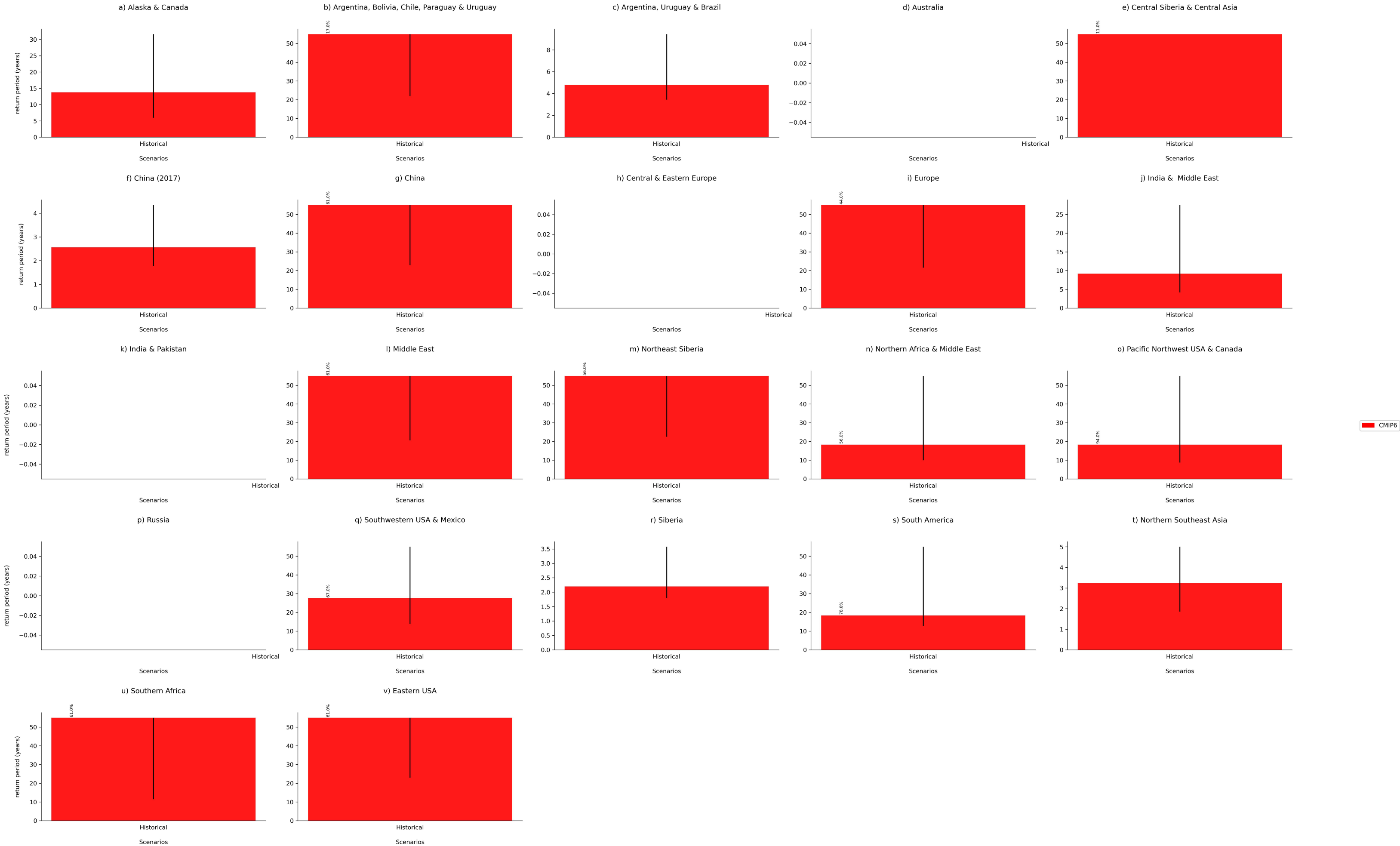

a) Alaska & Canada
b) Argentina, Bolivia, Chile, Paraguay & Uruguay
17.0%
c) Argentina, Uruguay & Brazil
d) Australia
e) Central Siberia & Central Asia
11.0%
f) China (2017)
g) China
61.0%
h) Central & Eastern Europe
i) Europe
44.0%
j) India & Middle East
k) India & Pakistan
l) Middle East
61.0%
m) Northeast Siberia
56.0%
n) Northern Africa & Middle East
56.0%
o) Pacific Northwest USA & Canada
94.0%
p) Russia
q) Southwestern USA & Mexico
67.0%
r) Siberia
s) South America
78.0%
t) Northern Southeast Asia
u) Southern Africa
61.0%
v) Eastern USA
61.0%
return period (years)
Historical
Scenarios
CMIP6

Supplementary Figure 406: Return times (RT) during historical (1951-2005) based on ERA5 (Tx99.0p) - Return times (RT) (in years) during the historical period between 1951 and 2005 for Maximum Heatwave Severity of the respective observed event. For each region, values are shown for the different scenarios (increasing in emissions from left to right for the CMIP5 (green), CMIP6 (red), CanESM5 (dark blue), and ACCESS-ESM1-5 (light blue) ensembles. The error bars indicate the 5th and 95th percentile of the respective sample. Where the respective ensemble contains models not able to simulate the magnitude of the respective observed event's Maximum Heatwave Severity, the proportion of models that are able to simulate an event with a value of Maximum Heatwave Severity at least as high as the observed event is indicated by the percentage values above the individual bars. The observed reference values are calculated using data from ERA5.

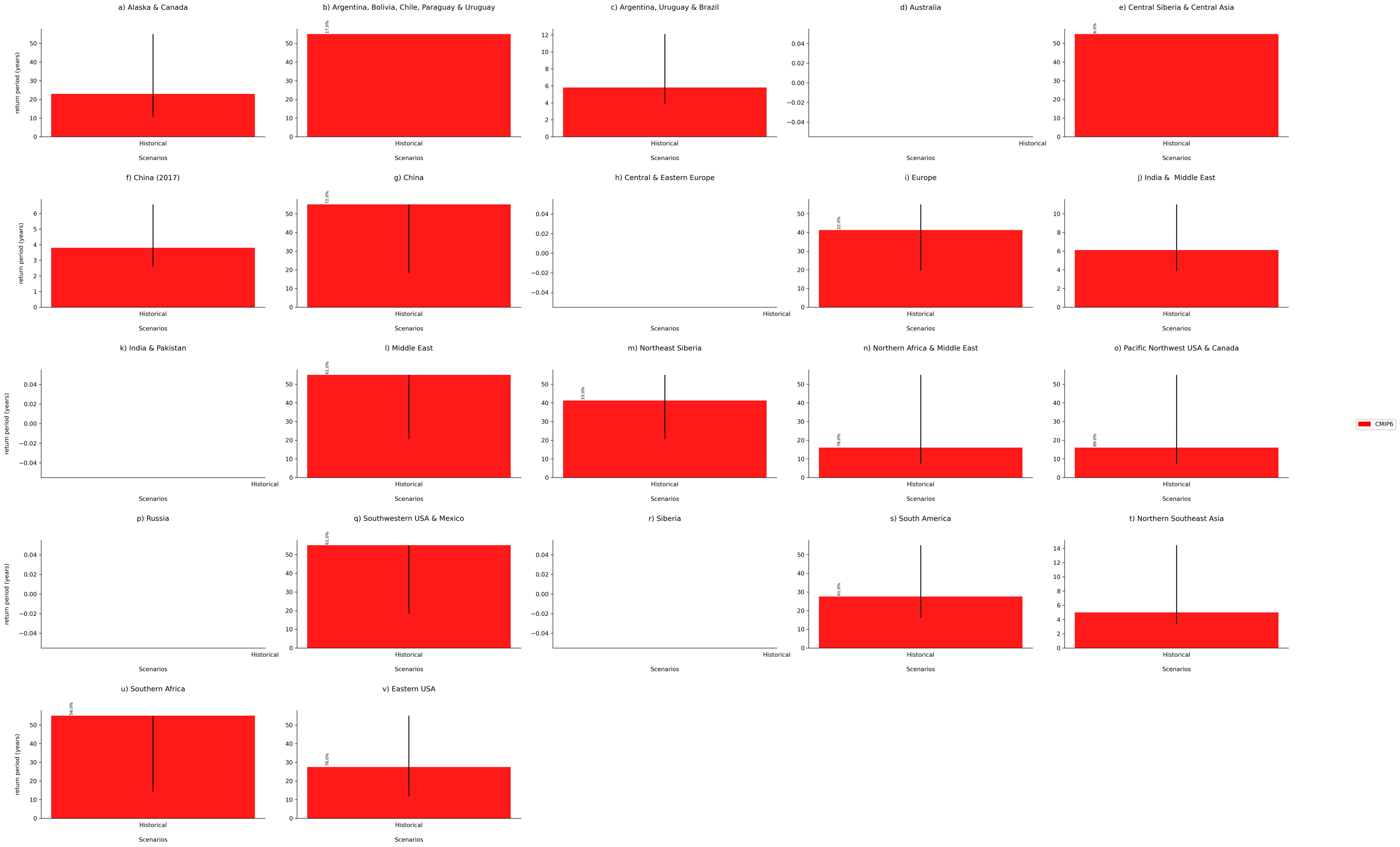

a) Alaska & Canada
b) Argentina, Bolivia, Chile, Paraguay & Uruguay
17.0%
c) Argentina, Uruguay & Brazil
d) Australia
e) Central Siberia & Central Asia
6.0%
f) China (2017)
g) China
72.0%
h) Central & Eastern Europe
i) Europe
22.0%
j) India & Middle East
k) India & Pakistan
l) Middle East
61.0%
m) Northeast Siberia
33.0%
n) Northern Africa & Middle East
78.0%
o) Pacific Northwest USA & Canada
89.0%
p) Russia
q) Southwestern USA & Mexico
61.0%
r) Siberia
s) South America
61.0%
t) Northern Southeast Asia
u) Southern Africa
56.0%
v) Eastern USA
78.0%
return period (years)
Historical
Scenarios
CMIP6

Supplementary Figure 407: Return times (RT) during present-to-near-future (2015-2054) based on ERA5 (Tx95.0p) - Return times (RT) (in years) during the present-to-near-future period between 2015 and 2054 for Maximum Heatwave Severity of the respective observed event. For each region, values are shown for the different scenarios (increasing in emissions from left to right for the CMIP5 (green), CMIP6 (red), CanESM5 (dark blue), and ACCESS-ESM1-5 (light blue) ensembles. The error bars indicate the 5th and 95th percentile of the respective sample. Where the respective ensemble contains models not able to simulate the magnitude of the respective observed event's Maximum Heatwave Severity, the proportion of models that are able to simulate an event with a value of Maximum Heatwave Severity at least as high as the observed event is indicated by the percentage values above the individual bars. The observed reference values are calculated using data from ERA5.

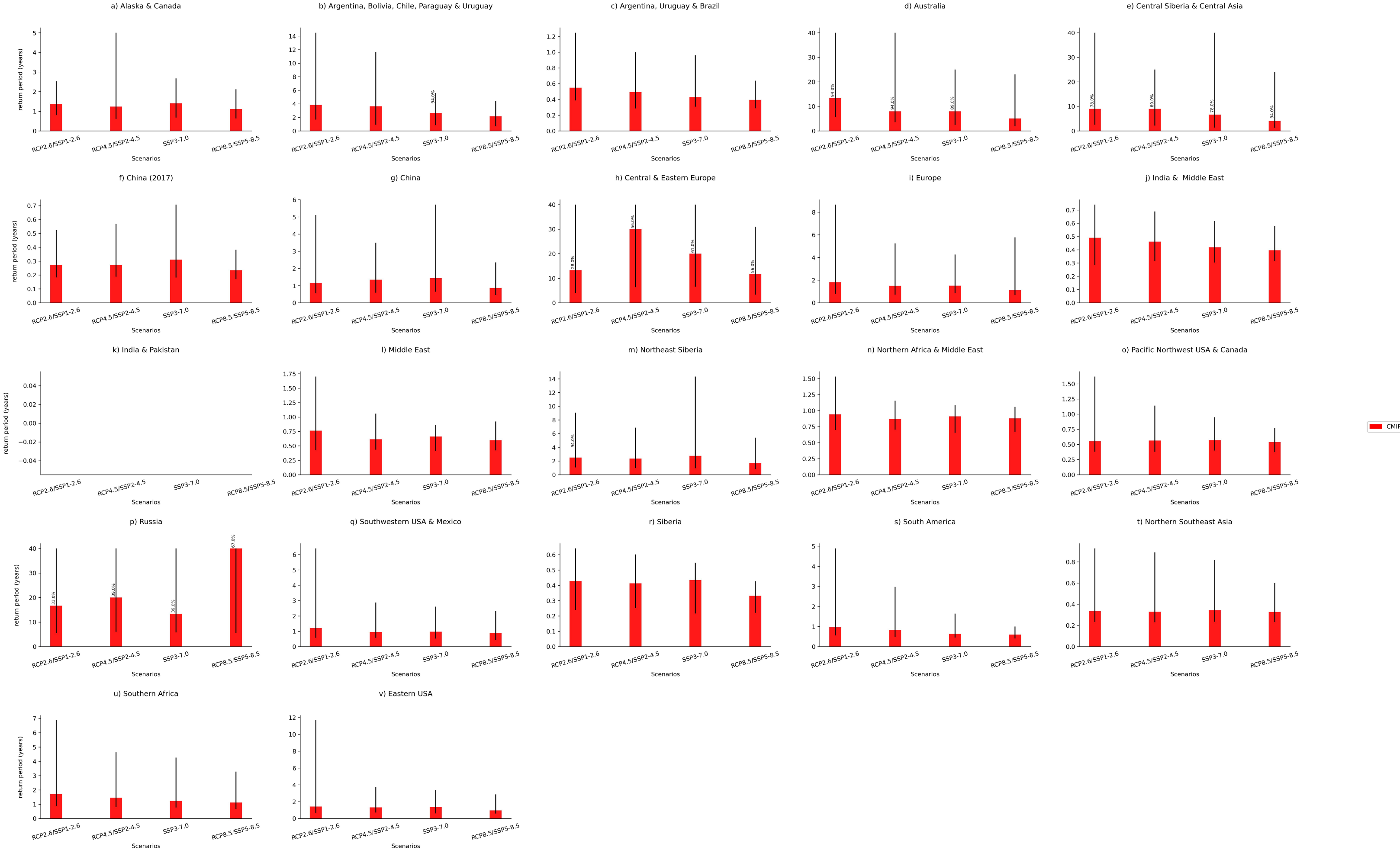
a) Alaska & Canada
b) Argentina, Bolivia, Chile, Paraguay & Uruguay
c) Argentina, Uruguay & Brazil
d) Australia
e) Central Siberia & Central Asia
f) China (2017)
g) China
h) Central & Eastern Europe
i) Europe
j) India & Middle East
k) India & Pakistan
l) Middle East
m) Northeast Siberia
n) Northern Africa & Middle East
o) Pacific Northwest USA & Canada
p) Russia
q) Southwestern USA & Mexico
r) Siberia
s) South America
t) Northern Southeast Asia
u) Southern Africa
v) Eastern USA
return period (years)
Scenarios
RCP2.6/SSP1-2.6
RCP4.5/SSP2-4.5
SSP3-7.0
RCP8.5/SSP5-8.5
CMIP6

Supplementary Figure 408: Return times (RT) during present-to-near-future (2015-2054) based on ERA5 (Tx97.5p) - Return times (RT) (in years) during the present-to-near-future period between 2015 and 2054 for Maximum Heatwave Severity of the respective observed event. For each region, values are shown for the different scenarios (increasing in emissions from left to right for the CMIP5 (green), CMIP6 (red), CanESM5 (dark blue), and ACCESS-ESM1-5 (light blue) ensembles. The error bars indicate the 5th and 95th percentile of the respective sample. Where the respective ensemble contains models not able to simulate the magnitude of the respective observed event's Maximum Heatwave Severity, the proportion of models that are able to simulate an event with a value of Maximum Heatwave Severity at least as high as the observed event is indicated by the percentage values above the individual bars. The observed reference values are calculated using data from ERA5.

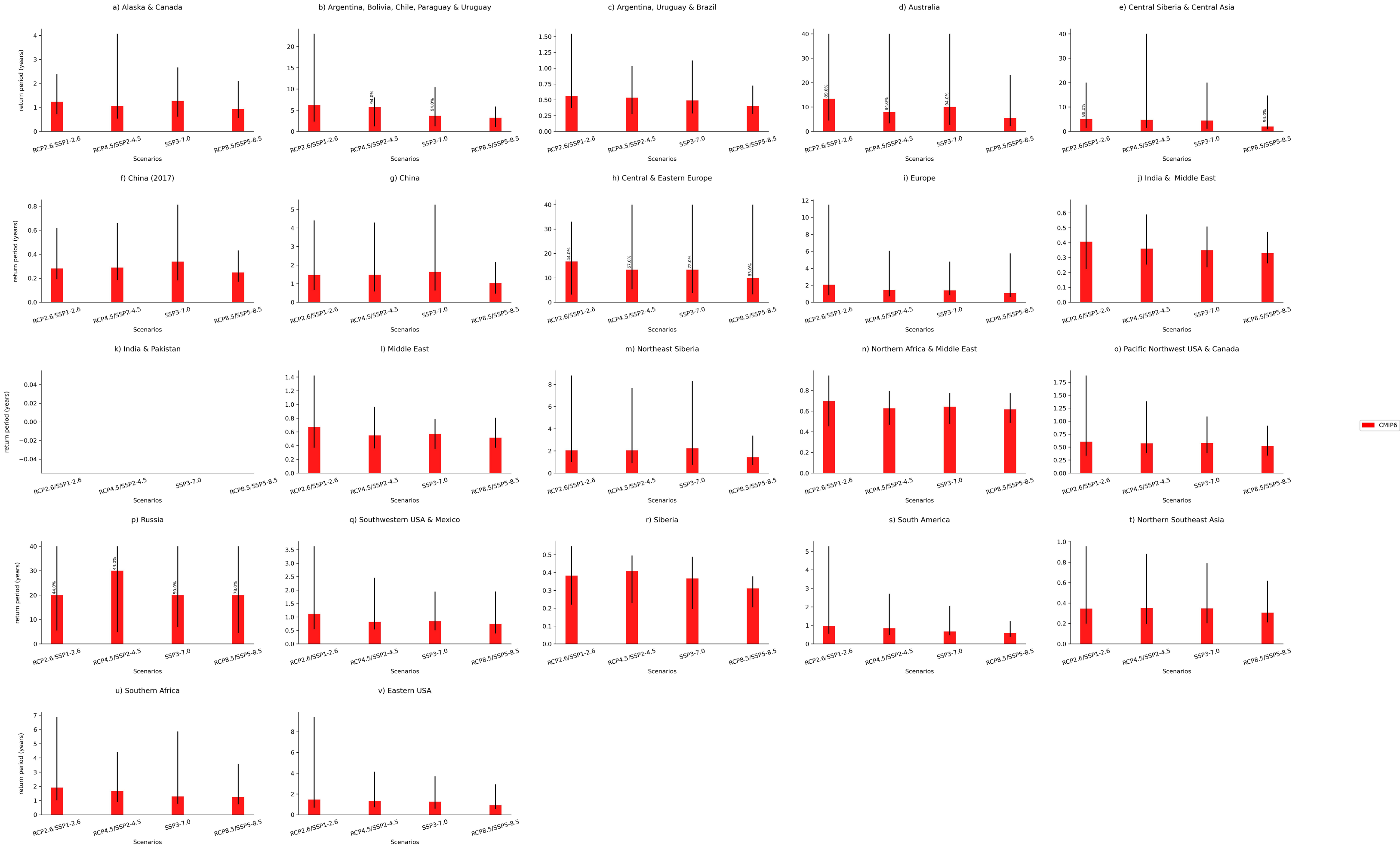
a) Alaska & Canada
b) Argentina, Bolivia, Chile, Paraguay & Uruguay
c) Argentina, Uruguay & Brazil
d) Australia
e) Central Siberia & Central Asia
f) China (2017)
g) China
h) Central & Eastern Europe
i) Europe
j) India & Middle East
k) India & Pakistan
l) Middle East
m) Northeast Siberia
n) Northern Africa & Middle East
o) Pacific Northwest USA & Canada
p) Russia
q) Southwestern USA & Mexico
r) Siberia
s) South America
t) Northern Southeast Asia
u) Southern Africa
v) Eastern USA
return period (years)
Scenarios
RCP2.6/SSP1-2.6
RCP4.5/SSP2-4.5
SSP3-7.0
RCP8.5/SSP5-8.5
CMIP6

Supplementary Figure 409: Return times (RT) during present-to-near-future (2015-2054) based on ERA5 (Tx99.0p) - Return times (RT) (in years) during the present-to-near-future period between 2015 and 2054 for Maximum Heatwave Severity of the respective observed event. For each region, values are shown for the different scenarios (increasing in emissions from left to right for the CMIP5 (green), CMIP6 (red), CanESM5 (dark blue), and ACCESS-ESM1-5 (light blue) ensembles. The error bars indicate the 5th and 95th percentile of the respective sample. Where the respective ensemble contains models not able to simulate the magnitude of the respective observed event's Maximum Heatwave Severity, the proportion of models that are able to simulate an event with a value of Maximum Heatwave Severity at least as high as the observed event is indicated by the percentage values above the individual bars. The observed reference values are calculated using data from ERA5.

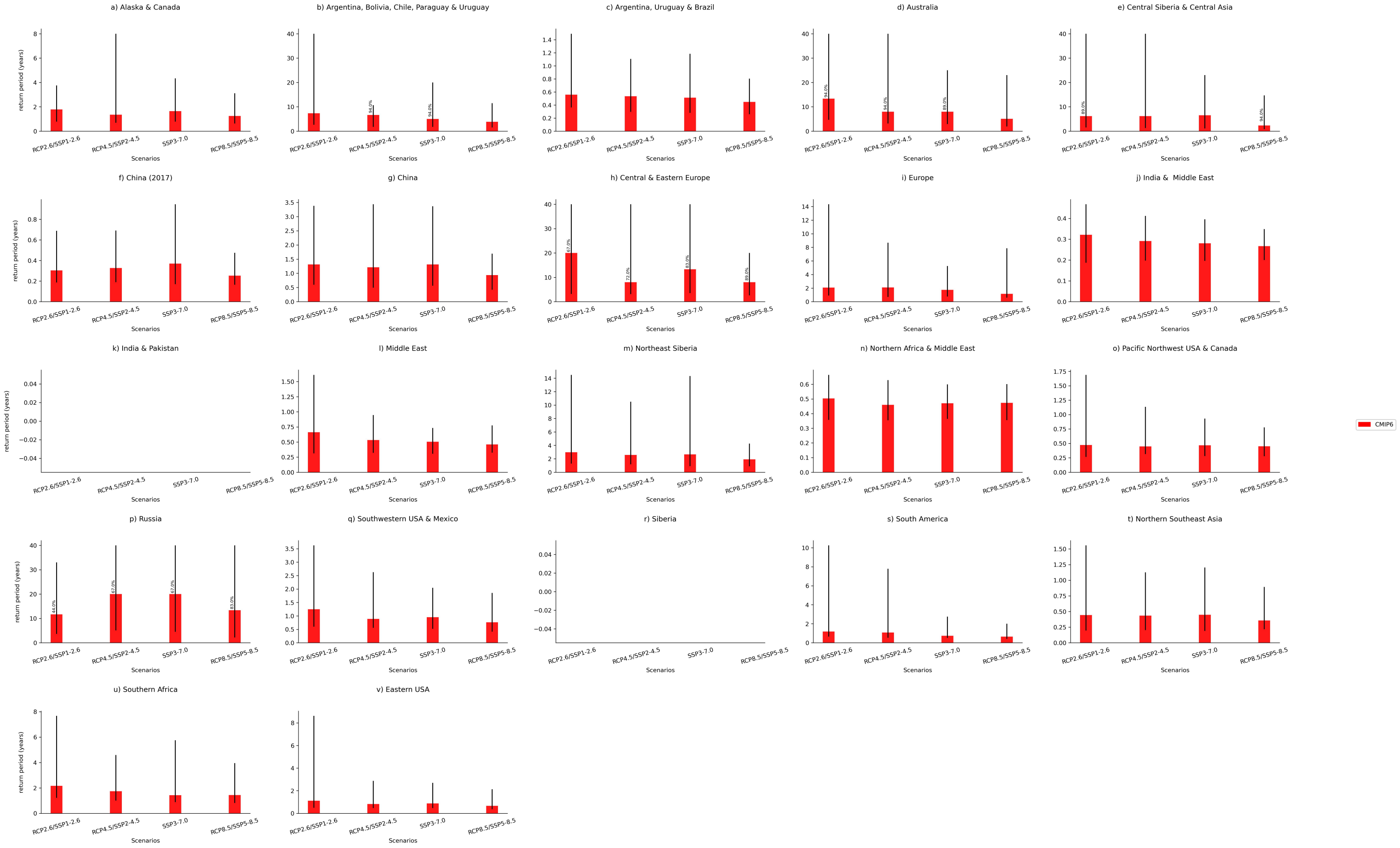

a) Alaska & Canada
b) Argentina, Bolivia, Chile, Paraguay & Uruguay
c) Argentina, Uruguay & Brazil
d) Australia
e) Central Siberia & Central Asia
f) China (2017)
g) China
h) Central & Eastern Europe
i) Europe
j) India & Middle East
k) India & Pakistan
l) Middle East
m) Northeast Siberia
n) Northern Africa & Middle East
o) Pacific Northwest USA & Canada
p) Russia
q) Southwestern USA & Mexico
r) Siberia
s) South America
t) Northern Southeast Asia
u) Southern Africa
v) Eastern USA
return period (years)
Scenarios
RCP2.6/SSP1-2.6
RCP4.5/SSP2-4.5
SSP3-7.0
RCP8.5/SSP5-8.5
CMIP6

Supplementary Figure 410: Return times (RT) during end-of-century (2060-2099) based on ERA5 (Tx95.0p) - Return times (RT) (in years) during the end-of-century period between 2060 and 2099 for Maximum Heatwave Severity of the respective observed event. For each region, values are shown for the different scenarios (increasing in emissions from left to right for the CMIP5 (green), CMIP6 (red), CanESM5 (dark blue), and ACCESS-ESM1-5 (light blue) ensembles. The error bars indicate the 5th and 95th percentile of the respective sample. Where the respective ensemble contains models not able to simulate the magnitude of the respective observed event's Maximum Heatwave Severity, the proportion of models that are able to simulate an event with a value of Maximum Heatwave Severity at least as high as the observed event is indicated by the percentage values above the individual bars. The observed reference values are calculated using data from ERA5.

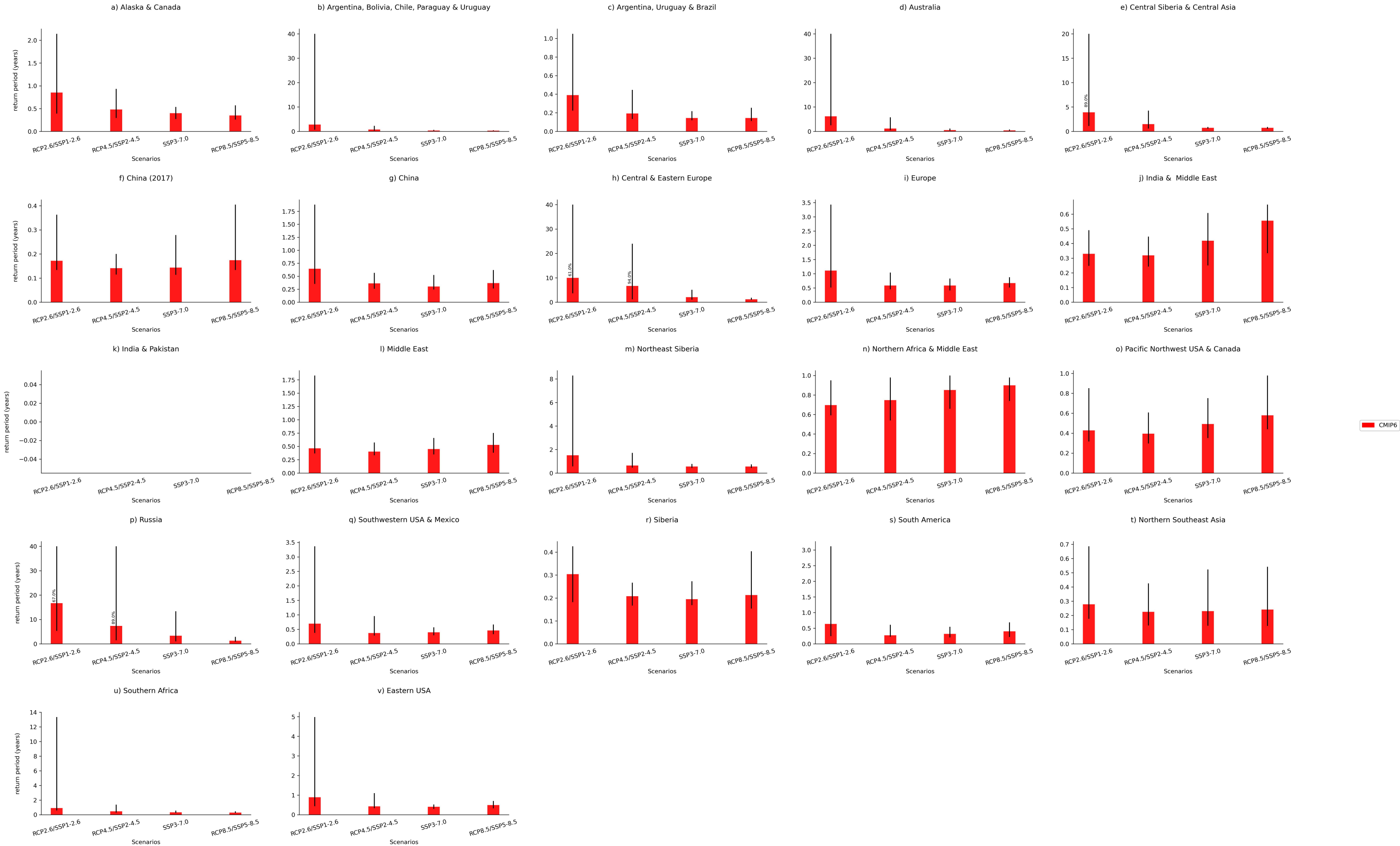
a) Alaska & Canada
b) Argentina, Bolivia, Chile, Paraguay & Uruguay
c) Argentina, Uruguay & Brazil
d) Australia
e) Central Siberia & Central Asia
f) China (2017)
g) China
h) Central & Eastern Europe
i) Europe
j) India & Middle East
k) India & Pakistan
l) Middle East
m) Northeast Siberia
n) Northern Africa & Middle East
o) Pacific Northwest USA & Canada
p) Russia
q) Southwestern USA & Mexico
r) Siberia
s) South America
t) Northern Southeast Asia
u) Southern Africa
v) Eastern USA
return period (years)
Scenarios
RCP2.6/SSP1-2.6
RCP4.5/SSP2-4.5
SSP3-7.0
RCP8.5/SSP5-8.5
89.0%
61.0%
94.0%
67.0%
89.0%
CMIP6

Supplementary Figure 411: Return times (RT) during end-of-century (2060-2099) based on ERA5 (Tx97.5p) - Return times (RT) (in years) during the end-of-century period between 2060 and 2099 for Maximum Heatwave Severity of the respective observed event. For each region, values are shown for the different scenarios (increasing in emissions from left to right for the CMIP5 (green), CMIP6 (red), CanESM5 (dark blue), and ACCESS-ESM1-5 (light blue) ensembles. The error bars indicate the 5th and 95th percentile of the respective sample. Where the respective ensemble contains models not able to simulate the magnitude of the respective observed event's Maximum Heatwave Severity, the proportion of models that are able to simulate an event with a value of Maximum Heatwave Severity at least as high as the observed event is indicated by the percentage values above the individual bars. The observed reference values are calculated using data from ERA5.

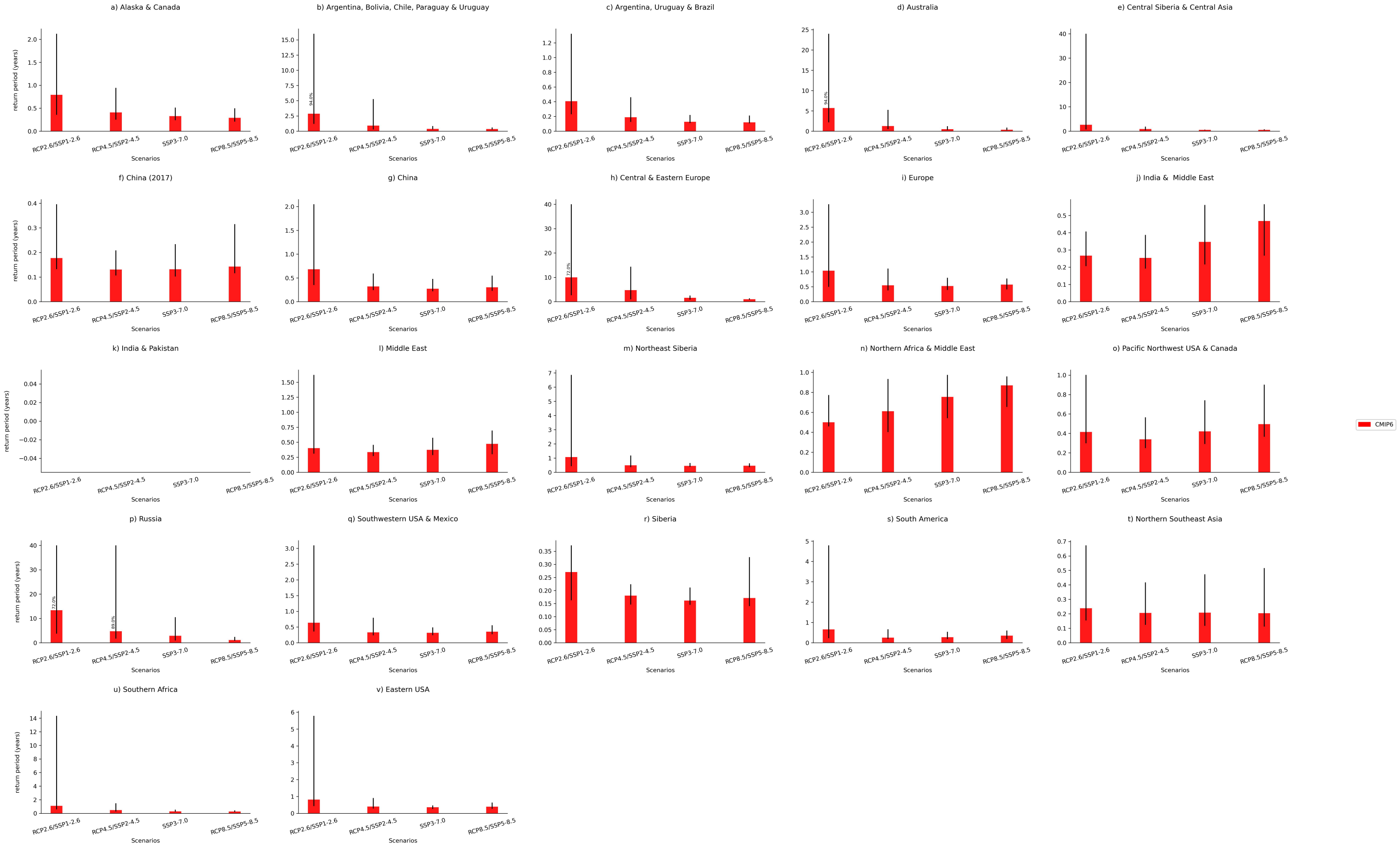

a) Alaska & Canada
b) Argentina, Bolivia, Chile, Paraguay & Uruguay
c) Argentina, Uruguay & Brazil
d) Australia
e) Central Siberia & Central Asia
f) China (2017)
g) China
h) Central & Eastern Europe
i) Europe
j) India & Middle East
k) India & Pakistan
l) Middle East
m) Northeast Siberia
n) Northern Africa & Middle East
o) Pacific Northwest USA & Canada
p) Russia
q) Southwestern USA & Mexico
r) Siberia
s) South America
t) Northern Southeast Asia
u) Southern Africa
v) Eastern USA
return period (years)
Scenarios
RCP2.6/SSP1-2.6
RCP4.5/SSP2-4.5
SSP3-7.0
RCP8.5/SSP5-8.5
CMIP6

Supplementary Figure 412: Return times (RT) during end-of-century (2060-2099) based on ERA5 (Tx99.0p) - Return times (RT) (in years) during the end-of-century period between 2060 and 2099 for Maximum Heatwave Severity of the respective observed event. For each region, values are shown for the different scenarios (increasing in emissions from left to right for the CMIP5 (green), CMIP6 (red), CanESM5 (dark blue), and ACCESS-ESM1-5 (light blue) ensembles. The error bars indicate the 5th and 95th percentile of the respective sample. Where the respective ensemble contains models not able to simulate the magnitude of the respective observed event's Maximum Heatwave Severity, the proportion of models that are able to simulate an event with a value of Maximum Heatwave Severity at least as high as the observed event is indicated by the percentage values above the individual bars. The observed reference values are calculated using data from ERA5.

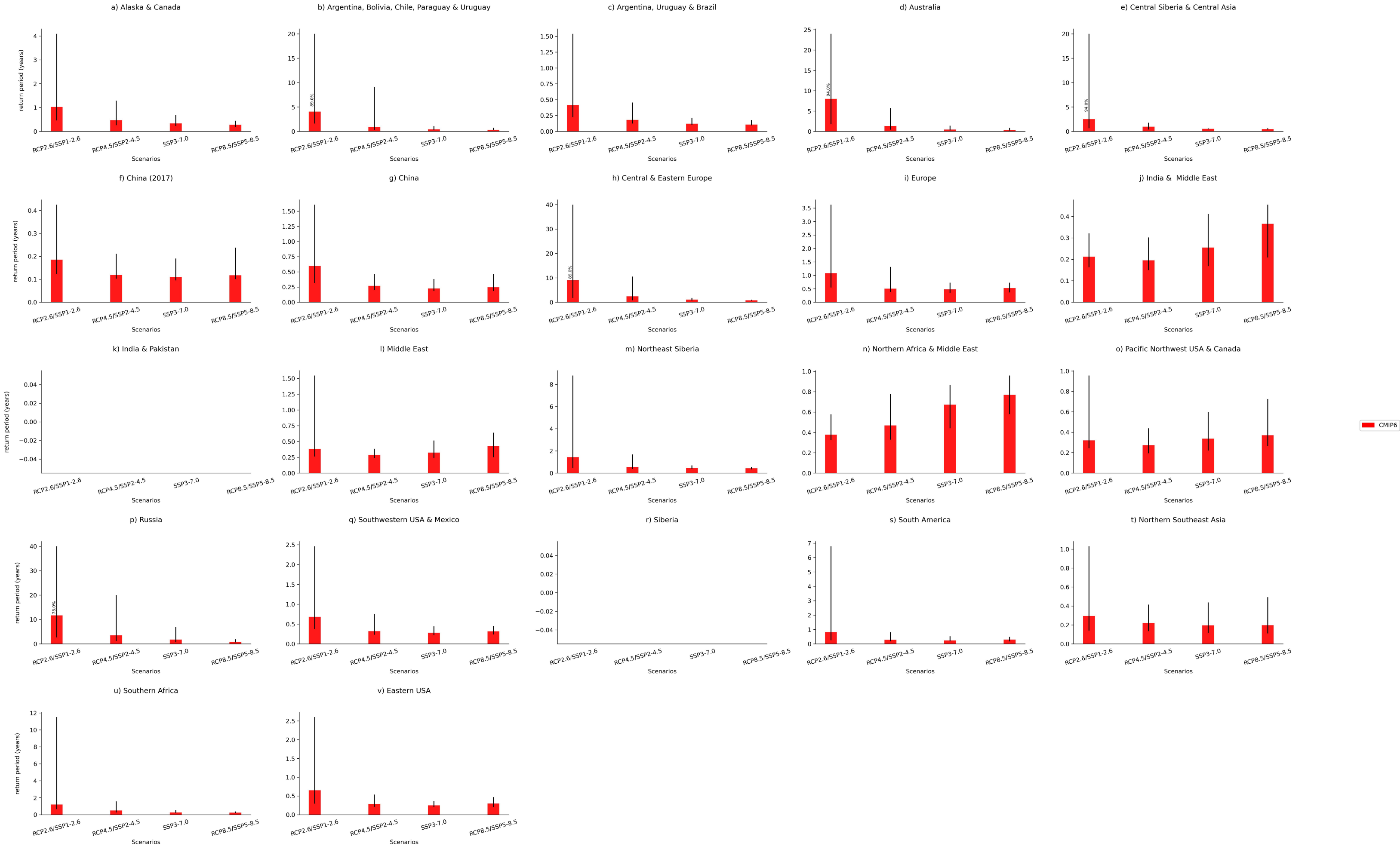
a) Alaska & Canada
b) Argentina, Bolivia, Chile, Paraguay & Uruguay
c) Argentina, Uruguay & Brazil
d) Australia
e) Central Siberia & Central Asia
f) China (2017)
g) China
h) Central & Eastern Europe
i) Europe
j) India & Middle East
k) India & Pakistan
l) Middle East
m) Northeast Siberia
n) Northern Africa & Middle East
o) Pacific Northwest USA & Canada
p) Russia
q) Southwestern USA & Mexico
r) Siberia
s) South America
t) Northern Southeast Asia
u) Southern Africa
v) Eastern USA
return period (years)
Scenarios
RCP2.6/SSP1-2.6
RCP4.5/SSP2-4.5
SSP3-7.0
RCP8.5/SSP5-8.5
89.0%
94.0%
94.0%
89.0%
78.0%
CMIP6

Supplementary Figure 413: Return times (RT) during historical (1951-2005) based on Berkeley Earth (Tx95.0p) - Return times (RT) (in years) during the historical period between 1951 and 2005 for Median Heatwave Area (%) of the respective observed event. For each region, values are shown for the different scenarios (increasing in emissions from left to right for the CMIP5 (green), CMIP6 (red), CanESM5 (dark blue), and ACCESS-ESM1-5 (light blue) ensembles. The error bars indicate the 5th and 95th percentile of the respective sample. Where the respective ensemble contains models not able to simulate the magnitude of the respective observed event's Median Heatwave Area (%), the proportion of models that are able to simulate an event with a value of Median Heatwave Area (%) at least as high as the observed event is indicated by the percentage values above the individual bars. The observed reference values are calculated using data from Berkeley Earth.

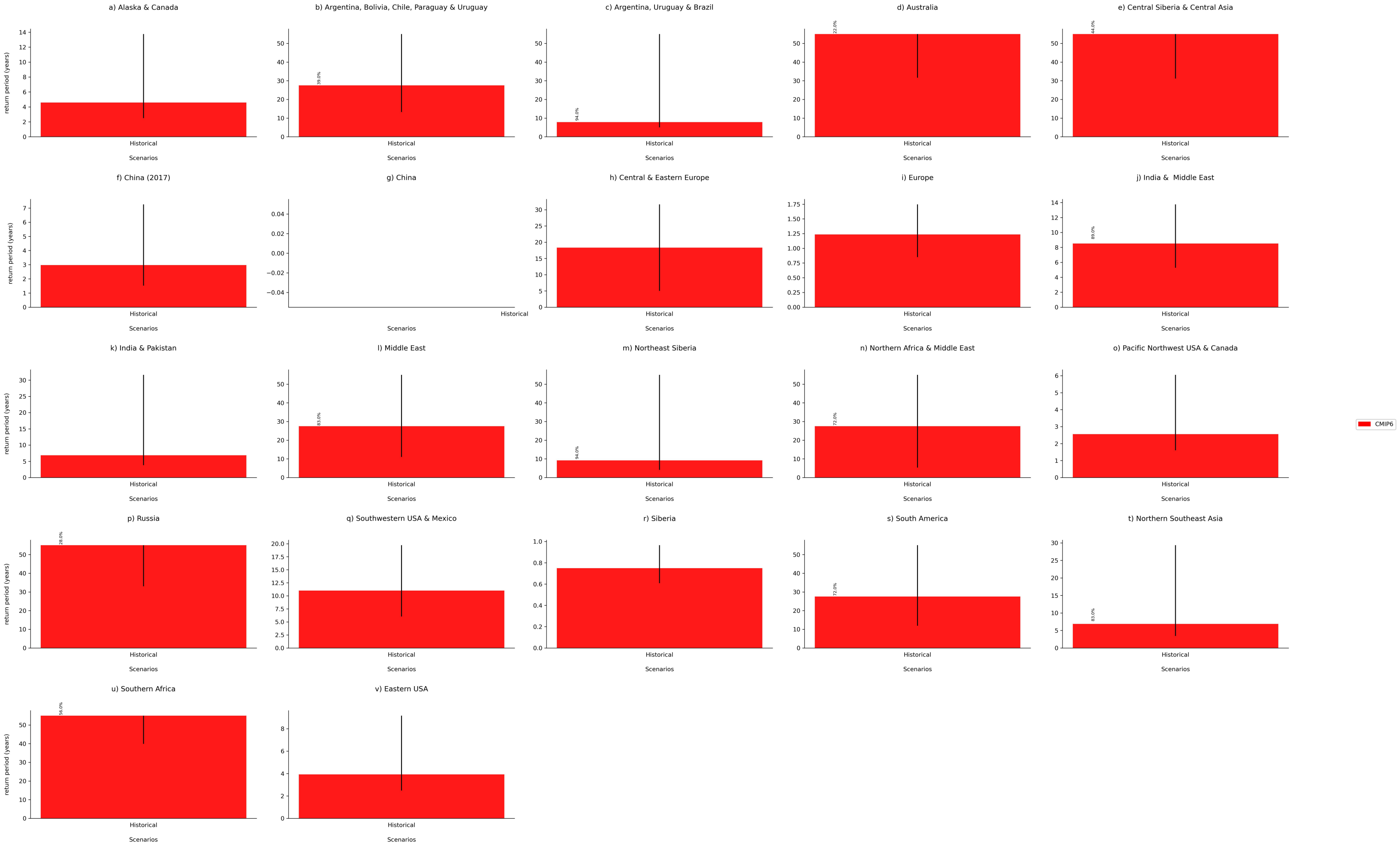
a) Alaska & Canada
b) Argentina, Bolivia, Chile, Paraguay & Uruguay
39.0%
c) Argentina, Uruguay & Brazil
94.0%
d) Australia
22.0%
e) Central Siberia & Central Asia
44.0%
f) China (2017)
g) China
h) Central & Eastern Europe
i) Europe
j) India & Middle East
89.0%
k) India & Pakistan
l) Middle East
83.0%
m) Northeast Siberia
94.0%
n) Northern Africa & Middle East
72.0%
o) Pacific Northwest USA & Canada
p) Russia
28.0%
q) Southwestern USA & Mexico
r) Siberia
s) South America
72.0%
t) Northern Southeast Asia
83.0%
u) Southern Africa
56.0%
v) Eastern USA
return period (years)
Historical
Scenarios
CMIP6

Supplementary Figure 414: Return times (RT) during historical (1951-2005) based on Berkeley Earth (Tx97.5p) - Return times (RT) (in years) during the historical period between 1951 and 2005 for Median Heatwave Area (%) of the respective observed event. For each region, values are shown for the different scenarios (increasing in emissions from left to right for the CMIP5 (green), CMIP6 (red), CanESM5 (dark blue), and ACCESS-ESM1-5 (light blue) ensembles. The error bars indicate the 5th and 95th percentile of the respective sample. Where the respective ensemble contains models not able to simulate the magnitude of the respective observed event's Median Heatwave Area (%), the proportion of models that are able to simulate an event with a value of Median Heatwave Area (%) at least as high as the observed event is indicated by the percentage values above the individual bars. The observed reference values are calculated using data from Berkeley Earth.

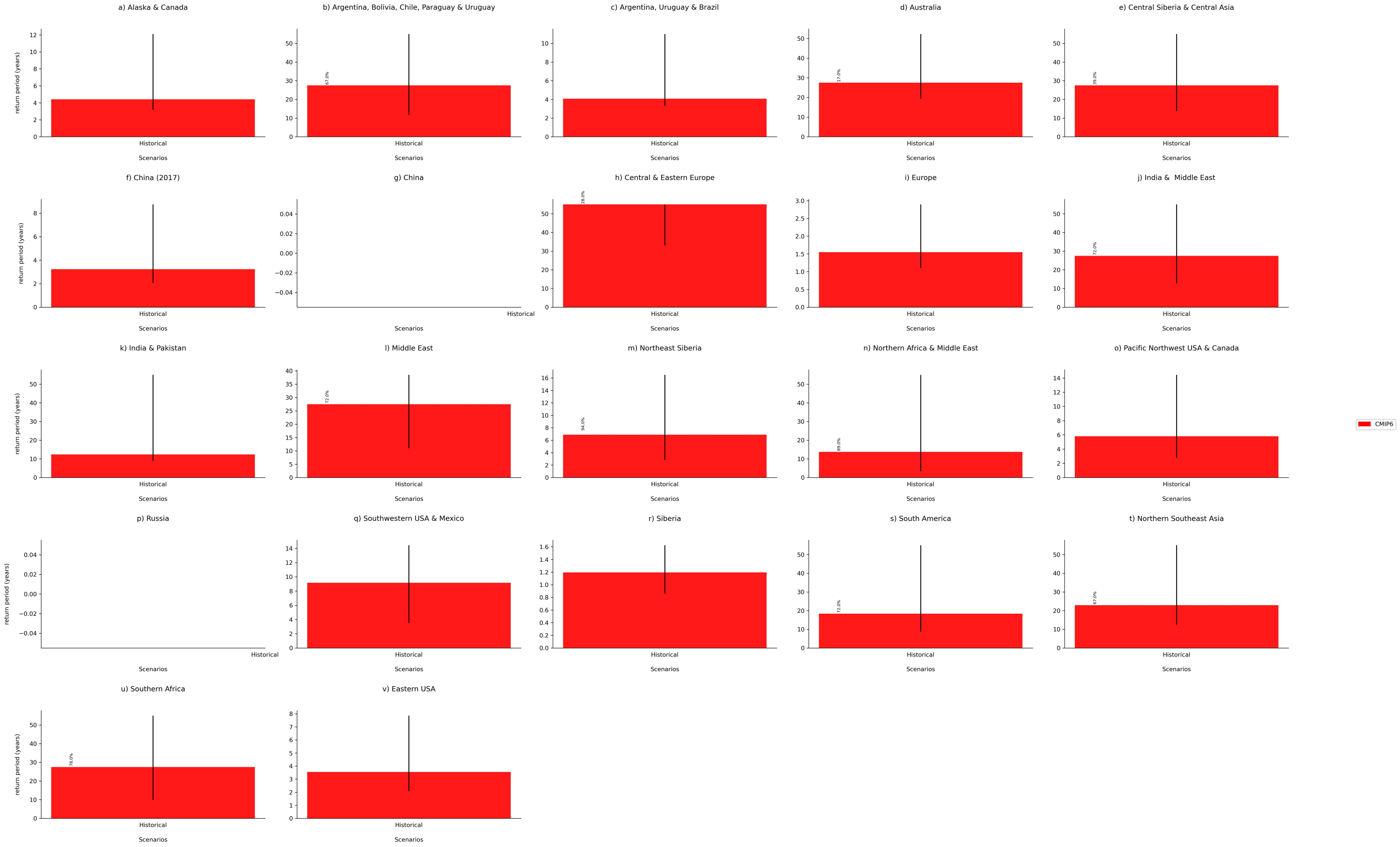

a) Alaska & Canada
b) Argentina, Bolivia, Chile, Paraguay & Uruguay
c) Argentina, Uruguay & Brazil
d) Australia
e) Central Siberia & Central Asia
f) China (2017)
g) China
h) Central & Eastern Europe
i) Europe
j) India & Middle East
k) India & Pakistan
l) Middle East
m) Northeast Siberia
n) Northern Africa & Middle East
o) Pacific Northwest USA & Canada
p) Russia
q) Southwestern USA & Mexico
r) Siberia
s) South America
t) Northern Southeast Asia
u) Southern Africa
v) Eastern USA
return period (years)
Historical
Scenarios
CMIP6

Supplementary Figure 415: Return times (RT) during historical (1951-2005) based on Berkeley Earth (Tx99.0p) - Return times (RT) (in years) during the historical period between 1951 and 2005 for Median Heatwave Area (%) of the respective observed event. For each region, values are shown for the different scenarios (increasing in emissions from left to right for the CMIP5 (green), CMIP6 (red), CanESM5 (dark blue), and ACCESS-ESM1-5 (light blue) ensembles. The error bars indicate the 5th and 95th percentile of the respective sample. Where the respective ensemble contains models not able to simulate the magnitude of the respective observed event's Median Heatwave Area (%), the proportion of models that are able to simulate an event with a value of Median Heatwave Area (%) at least as high as the observed event is indicated by the percentage values above the individual bars. The observed reference values are calculated using data from Berkeley Earth.

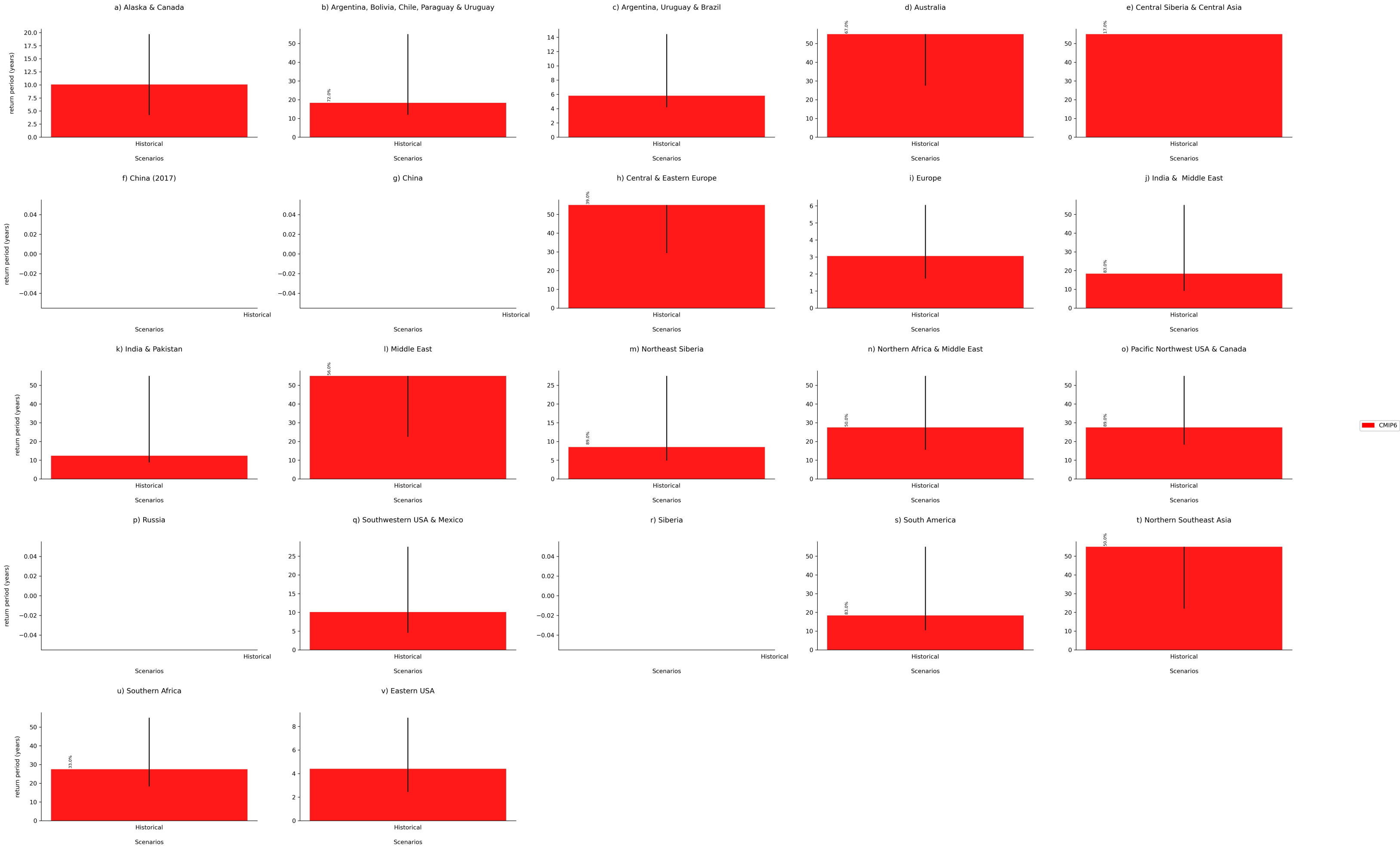

a) Alaska & Canada
b) Argentina, Bolivia, Chile, Paraguay & Uruguay
c) Argentina, Uruguay & Brazil
d) Australia
e) Central Siberia & Central Asia
f) China (2017)
g) China
h) Central & Eastern Europe
i) Europe
j) India & Middle East
k) India & Pakistan
l) Middle East
m) Northeast Siberia
n) Northern Africa & Middle East
o) Pacific Northwest USA & Canada
p) Russia
q) Southwestern USA & Mexico
r) Siberia
s) South America
t) Northern Southeast Asia
u) Southern Africa
v) Eastern USA
return period (years)
Historical
Scenarios
72.0%
67.0%
17.0%
39.0%
83.0%
56.0%
89.0%
50.0%
89.0%
83.0%
50.0%
33.0%
CMIP6

Supplementary Figure 416: Return times (RT) during present-to-near-future (2015-2054) based on Berkeley Earth (Tx95.0p) - Return times (RT) (in years) during the present-to-near-future period between 2015 and 2054 for Median Heatwave Area (%) of the respective observed event. For each region, values are shown for the different scenarios (increasing in emissions from left to right for the CMIP5 (green), CMIP6 (red), CanESM5 (dark blue), and ACCESS-ESM1-5 (light blue) ensembles. The error bars indicate the 5th and 95th percentile of the respective sample. Where the respective ensemble contains models not able to simulate the magnitude of the respective observed event's Median Heatwave Area (%), the proportion of models that are able to simulate an event with a value of Median Heatwave Area (%) at least as high as the observed event is indicated by the percentage values above the individual bars. The observed reference values are calculated using data from Berkeley Earth.

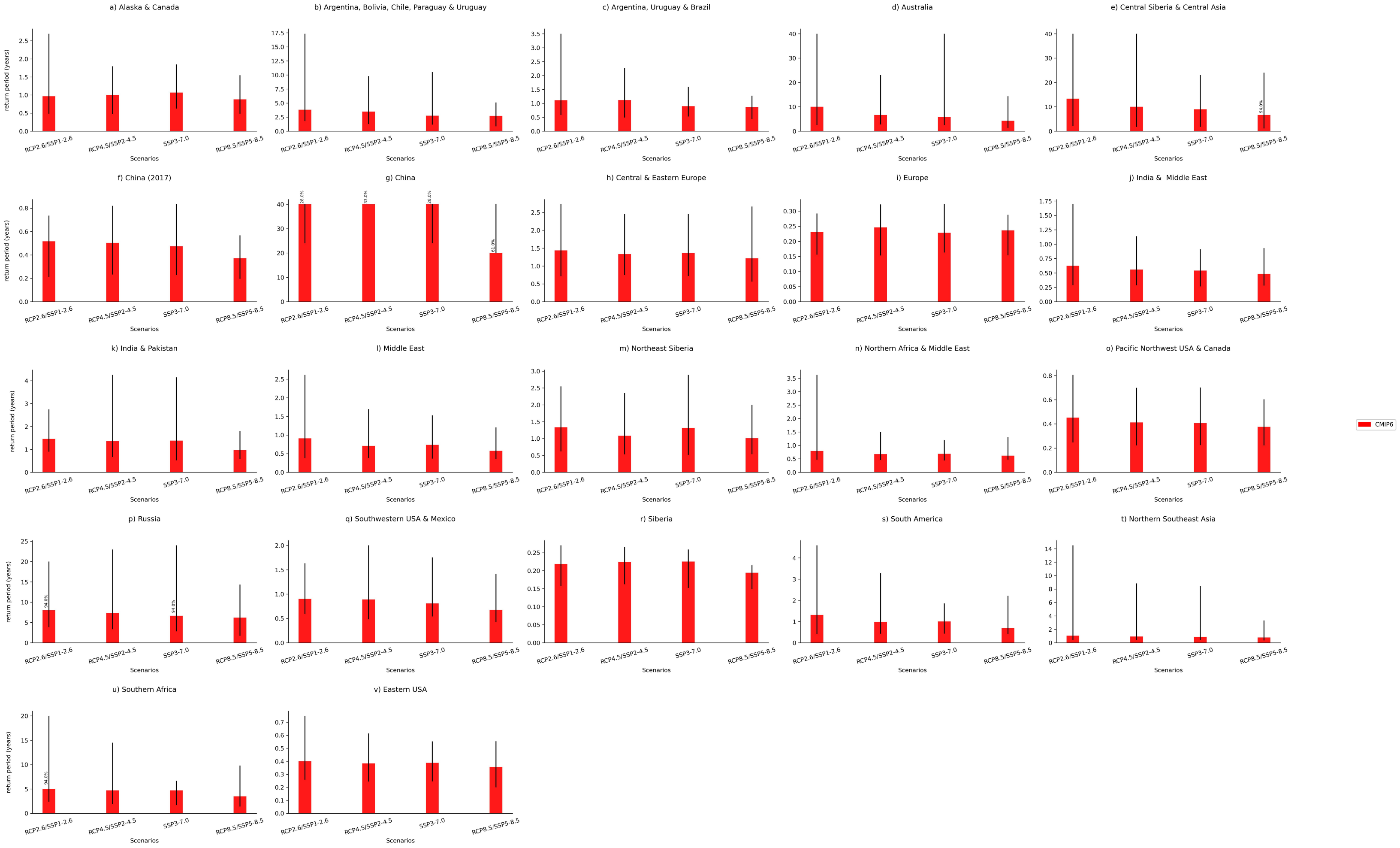

a) Alaska & Canada
b) Argentina, Bolivia, Chile, Paraguay & Uruguay
c) Argentina, Uruguay & Brazil
d) Australia
e) Central Siberia & Central Asia
f) China (2017)
g) China
h) Central & Eastern Europe
i) Europe
j) India & Middle East
k) India & Pakistan
l) Middle East
m) Northeast Siberia
n) Northern Africa & Middle East
o) Pacific Northwest USA & Canada
p) Russia
q) Southwestern USA & Mexico
r) Siberia
s) South America
t) Northern Southeast Asia
u) Southern Africa
v) Eastern USA
return period (years)
Scenarios
RCP2.6/SSP1-2.6
RCP4.5/SSP2-4.5
SSP3-7.0
RCP8.5/SSP5-8.5
CMIP6

Supplementary Figure 417: Return times (RT) during present-to-near-future (2015-2054) based on Berkeley Earth (Tx97.5p) - Return times (RT) (in years) during the present-to-near-future period between 2015 and 2054 for Median Heatwave Area (%) of the respective observed event. For each region, values are shown for the different scenarios (increasing in emissions from left to right for the CMIP5 (green), CMIP6 (red), CanESM5 (dark blue), and ACCESS-ESM1-5 (light blue) ensembles. The error bars indicate the 5th and 95th percentile of the respective sample. Where the respective ensemble contains models not able to simulate the magnitude of the respective observed event's Median Heatwave Area (%), the proportion of models that are able to simulate an event with a value of Median Heatwave Area (%) at least as high as the observed event is indicated by the percentage values above the individual bars. The observed reference values are calculated using data from Berkeley Earth.

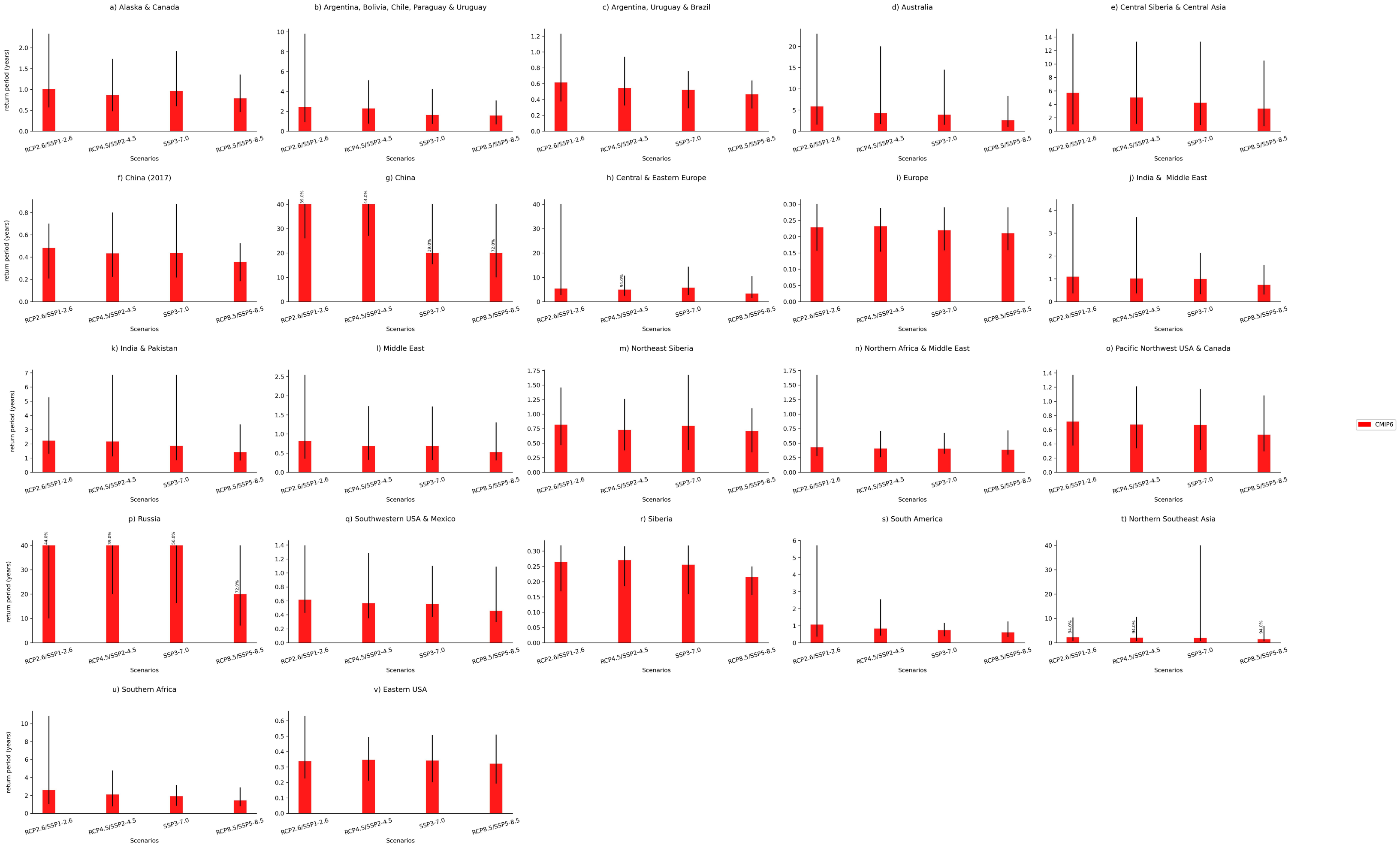

a) Alaska & Canada
b) Argentina, Bolivia, Chile, Paraguay & Uruguay
c) Argentina, Uruguay & Brazil
d) Australia
e) Central Siberia & Central Asia
f) China (2017)
g) China
h) Central & Eastern Europe
i) Europe
j) India & Middle East
k) India & Pakistan
l) Middle East
m) Northeast Siberia
n) Northern Africa & Middle East
o) Pacific Northwest USA & Canada
p) Russia
q) Southwestern USA & Mexico
r) Siberia
s) South America
t) Northern Southeast Asia
u) Southern Africa
v) Eastern USA
return period (years)
Scenarios
RCP2.6/SSP1-2.6
RCP4.5/SSP2-4.5
SSP3-7.0
RCP8.5/SSP5-8.5
CMIP6

Supplementary Figure 418: Return times (RT) during present-to-near-future (2015-2054) based on Berkeley Earth (Tx99.0p) - Return times (RT) (in years) during the present-to-near-future period between 2015 and 2054 for Median Heatwave Area (%) of the respective observed event. For each region, values are shown for the different scenarios (increasing in emissions from left to right for the CMIP5 (green), CMIP6 (red), CanESM5 (dark blue), and ACCESS-ESM1-5 (light blue) ensembles. The error bars indicate the 5th and 95th percentile of the respective sample. Where the respective ensemble contains models not able to simulate the magnitude of the respective observed event's Median Heatwave Area (%), the proportion of models that are able to simulate an event with a value of Median Heatwave Area (%) at least as high as the observed event is indicated by the percentage values above the individual bars. The observed reference values are calculated using data from Berkeley Earth.

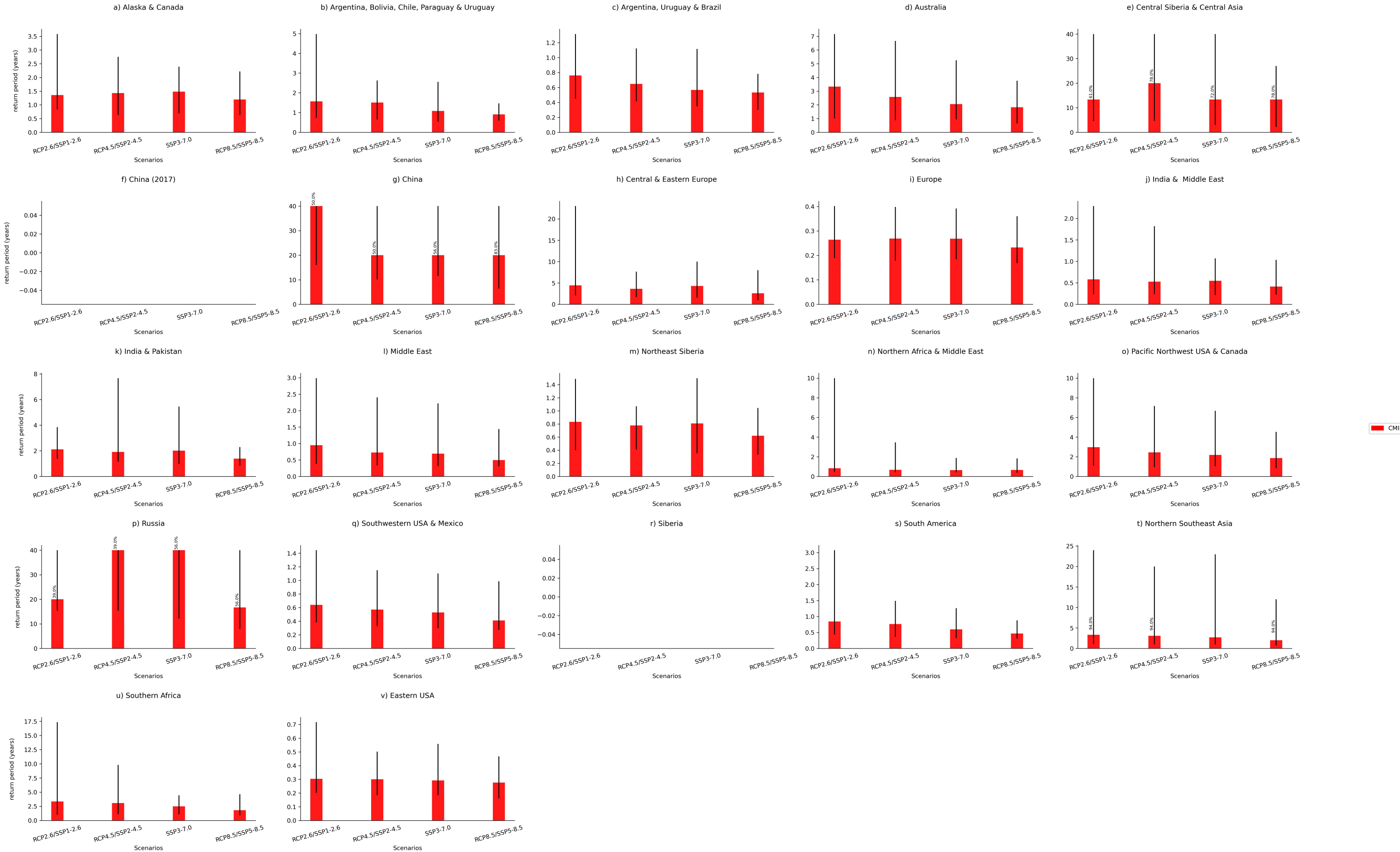
a) Alaska & Canada
b) Argentina, Bolivia, Chile, Paraguay & Uruguay
c) Argentina, Uruguay & Brazil
d) Australia
e) Central Siberia & Central Asia
61.0%
78.0%
72.0%
78.0%
f) China (2017)
g) China
50.0%
50.0%
56.0%
83.0%
h) Central & Eastern Europe
i) Europe
j) India & Middle East
k) India & Pakistan
l) Middle East
m) Northeast Siberia
n) Northern Africa & Middle East
o) Pacific Northwest USA & Canada
CMIP6
p) Russia
39.0%
39.0%
56.0%
56.0%
q) Southwestern USA & Mexico
r) Siberia
s) South America
t) Northern Southeast Asia
94.0%
94.0%
94.0%
u) Southern Africa
v) Eastern USA
return period (years)
Scenarios
RCP2.6/SSP1-2.6
RCP4.5/SSP2-4.5
SSP3-7.0
RCP8.5/SSP5-8.5

Supplementary Figure 419: Return times (RT) during end-of-century (2060-2099) based on Berkeley Earth (Tx95.0p) - Return times (RT) (in years) during the end-of-century period between 2060 and 2099 for Median Heatwave Area (%) of the respective observed event. For each region, values are shown for the different scenarios (increasing in emissions from left to right for the CMIP5 (green), CMIP6 (red), CanESM5 (dark blue), and ACCESS-ESM1-5 (light blue) ensembles. The error bars indicate the 5th and 95th percentile of the respective sample. Where the respective ensemble contains models not able to simulate the magnitude of the respective observed event's Median Heatwave Area (%), the proportion of models that are able to simulate an event with a value of Median Heatwave Area (%) at least as high as the observed event is indicated by the percentage values above the individual bars. The observed reference values are calculated using data from Berkeley Earth.

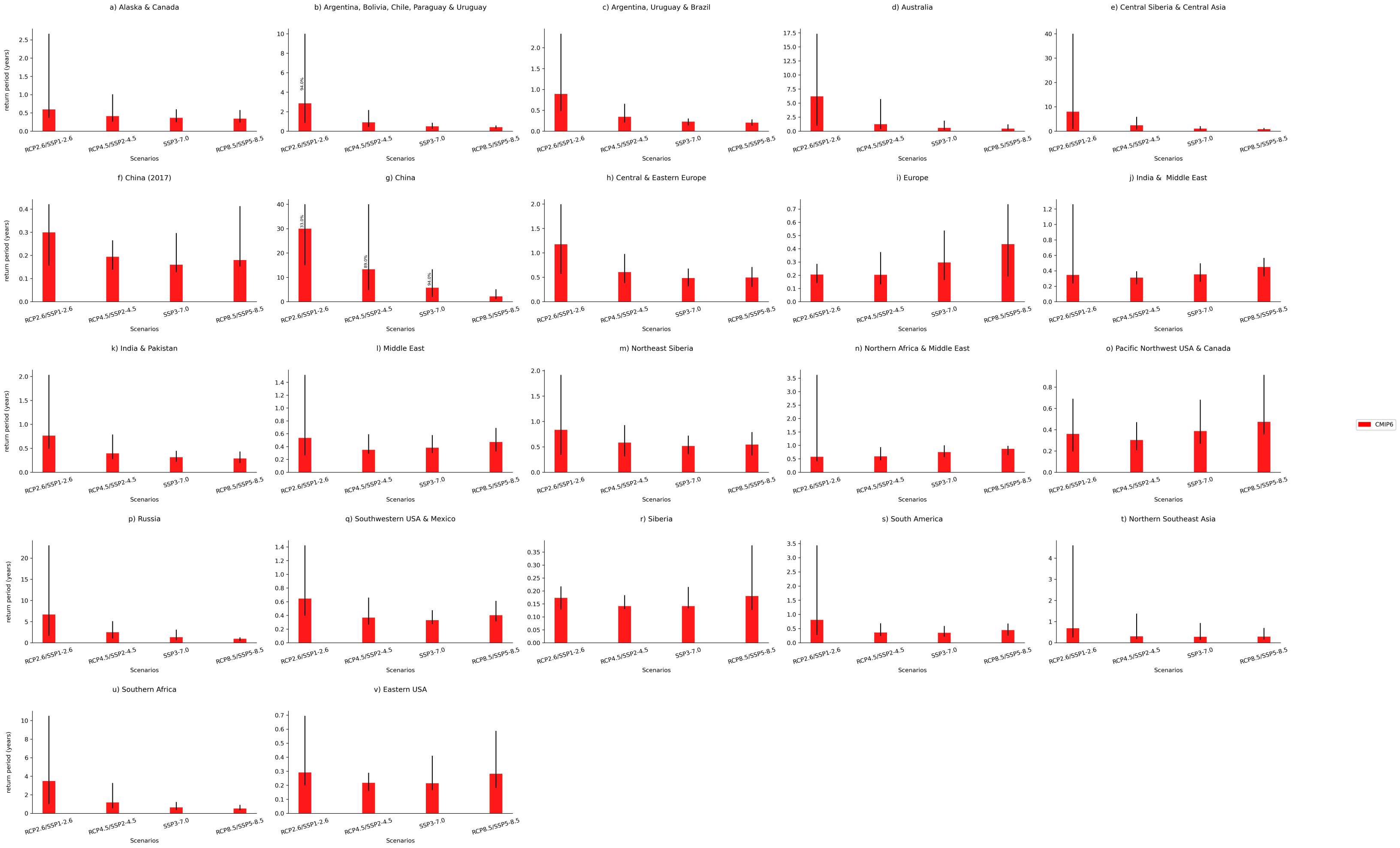
a) Alaska & Canada
b) Argentina, Bolivia, Chile, Paraguay & Uruguay
c) Argentina, Uruguay & Brazil
d) Australia
e) Central Siberia & Central Asia
f) China (2017)
g) China
h) Central & Eastern Europe
i) Europe
j) India & Middle East
k) India & Pakistan
l) Middle East
m) Northeast Siberia
n) Northern Africa & Middle East
o) Pacific Northwest USA & Canada
p) Russia
q) Southwestern USA & Mexico
r) Siberia
s) South America
t) Northern Southeast Asia
u) Southern Africa
v) Eastern USA
return period (years)
Scenarios
RCP2.6/SSP1-2.6
RCP4.5/SSP2-4.5
SSP3-7.0
RCP8.5/SSP5-8.5
94.0%
33.0%
89.0%
CMIP6

Supplementary Figure 420: Return times (RT) during end-of-century (2060-2099) based on Berkeley Earth (Tx97.5p) - Return times (RT) (in years) during the end-of-century period between 2060 and 2099 for Median Heatwave Area (%) of the respective observed event. For each region, values are shown for the different scenarios (increasing in emissions from left to right for the CMIP5 (green), CMIP6 (red), CanESM5 (dark blue), and ACCESS-ESM1-5 (light blue) ensembles. The error bars indicate the 5th and 95th percentile of the respective sample. Where the respective ensemble contains models not able to simulate the magnitude of the respective observed event's Median Heatwave Area (%), the proportion of models that are able to simulate an event with a value of Median Heatwave Area (%) at least as high as the observed event is indicated by the percentage values above the individual bars. The observed reference values are calculated using data from Berkeley Earth.

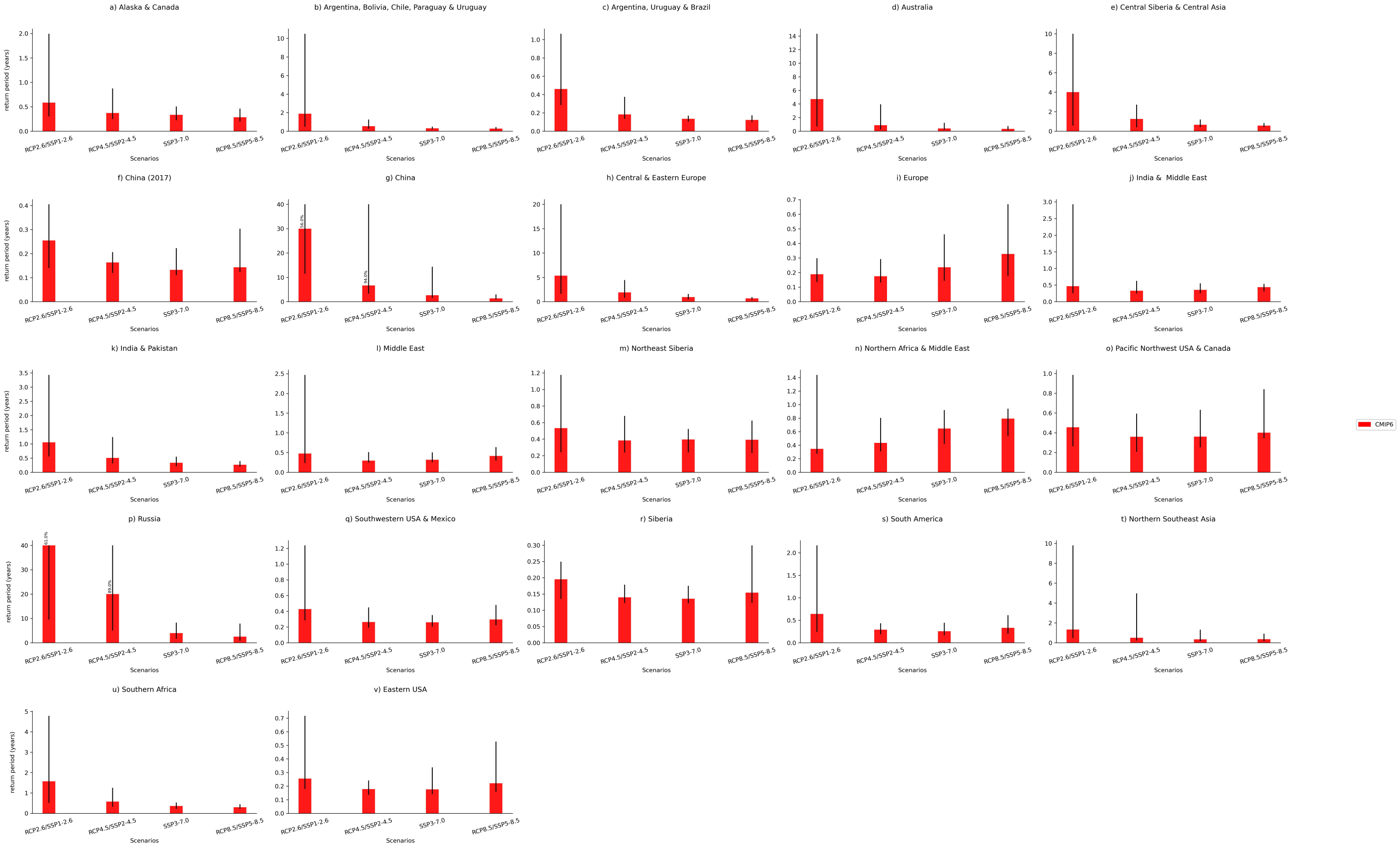

a) Alaska & Canada
b) Argentina, Bolivia, Chile, Paraguay & Uruguay
c) Argentina, Uruguay & Brazil
d) Australia
e) Central Siberia & Central Asia
f) China (2017)
g) China
h) Central & Eastern Europe
i) Europe
j) India & Middle East
k) India & Pakistan
l) Middle East
m) Northeast Siberia
n) Northern Africa & Middle East
o) Pacific Northwest USA & Canada
p) Russia
q) Southwestern USA & Mexico
r) Siberia
s) South America
t) Northern Southeast Asia
u) Southern Africa
v) Eastern USA
return period (years)
Scenarios
RCP2.6/SSP1-2.6
RCP4.5/SSP2-4.5
SSP3-7.0
RCP8.5/SSP5-8.5
56.0%
94.0%
61.0%
89.0%
CMIP6

Supplementary Figure 421: Return times (RT) during end-of-century (2060-2099) based on Berkeley Earth (Tx99.0p) - Return times (RT) (in years) during the end-of-century period between 2060 and 2099 for Median Heatwave Area (%) of the respective observed event. For each region, values are shown for the different scenarios (increasing in emissions from left to right for the CMIP5 (green), CMIP6 (red), CanESM5 (dark blue), and ACCESS-ESM1-5 (light blue) ensembles. The error bars indicate the 5th and 95th percentile of the respective sample. Where the respective ensemble contains models not able to simulate the magnitude of the respective observed event's Median Heatwave Area (%), the proportion of models that are able to simulate an event with a value of Median Heatwave Area (%) at least as high as the observed event is indicated by the percentage values above the individual bars. The observed reference values are calculated using data from Berkeley Earth.

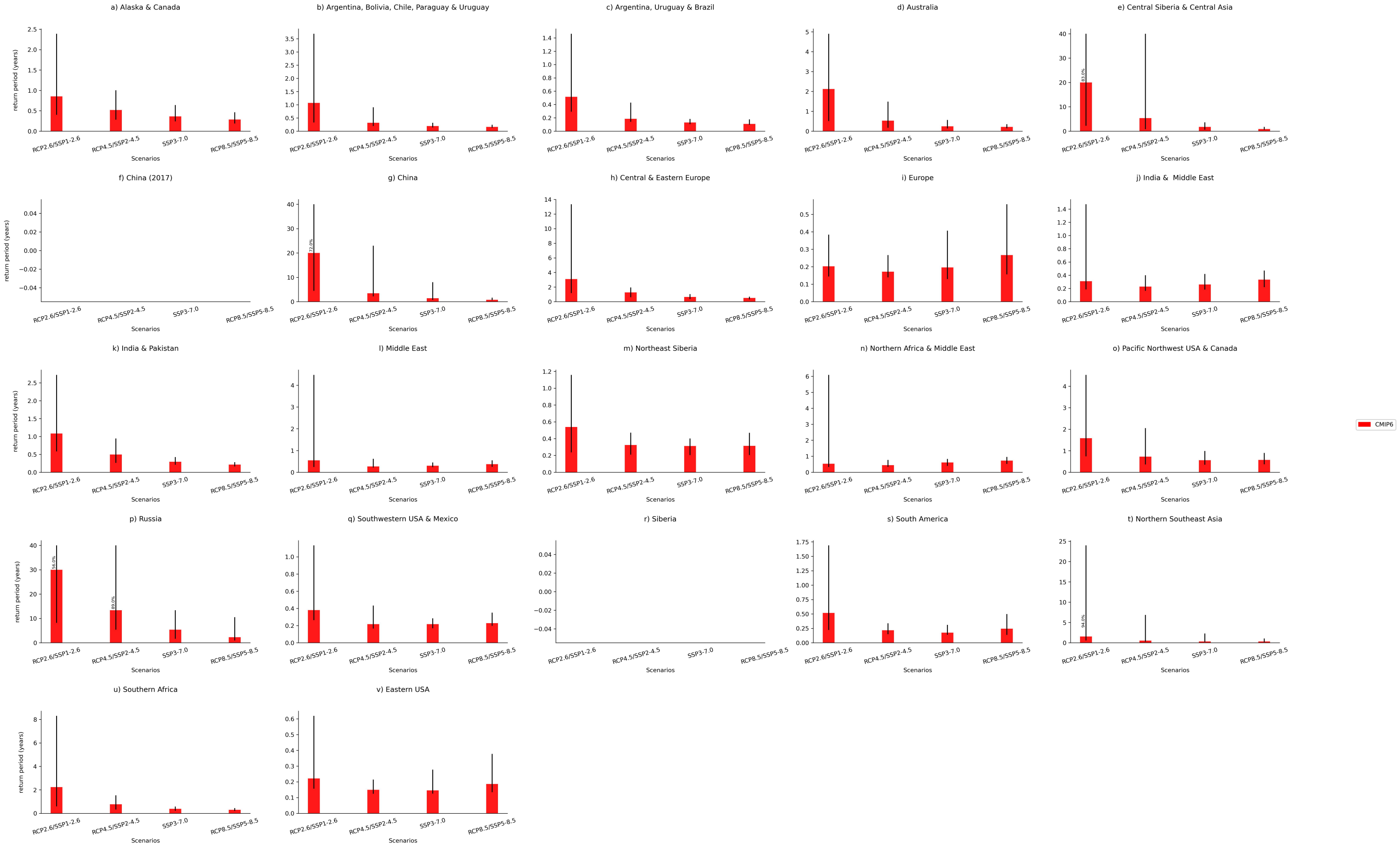
a) Alaska & Canada
b) Argentina, Bolivia, Chile, Paraguay & Uruguay
c) Argentina, Uruguay & Brazil
d) Australia
e) Central Siberia & Central Asia
f) China (2017)
g) China
h) Central & Eastern Europe
i) Europe
j) India & Middle East
k) India & Pakistan
l) Middle East
m) Northeast Siberia
n) Northern Africa & Middle East
o) Pacific Northwest USA & Canada
p) Russia
q) Southwestern USA & Mexico
r) Siberia
s) South America
t) Northern Southeast Asia
u) Southern Africa
v) Eastern USA
return period (years)
Scenarios
RCP2.6/SSP1-2.6
RCP4.5/SSP2-4.5
SSP3-7.0
RCP8.5/SSP5-8.5
83.0%
72.0%
56.0%
89.0%
94.0%
CMIP6

Supplementary Figure 422: Return times (RT) during historical (1951-2005) based on ERA5 (Tx95.0p) - Return times (RT) (in years) during the historical period between 1951 and 2005 for Median Heatwave Area (%) of the respective observed event. For each region, values are shown for the different scenarios (increasing in emissions from left to right for the CMIP5 (green), CMIP6 (red), CanESM5 (dark blue), and ACCESS-ESM1-5 (light blue) ensembles. The error bars indicate the 5th and 95th percentile of the respective sample. Where the respective ensemble contains models not able to simulate the magnitude of the respective observed event's Median Heatwave Area (%), the proportion of models that are able to simulate an event with a value of Median Heatwave Area (%) at least as high as the observed event is indicated by the percentage values above the individual bars. The observed reference values are calculated using data from ERA5.

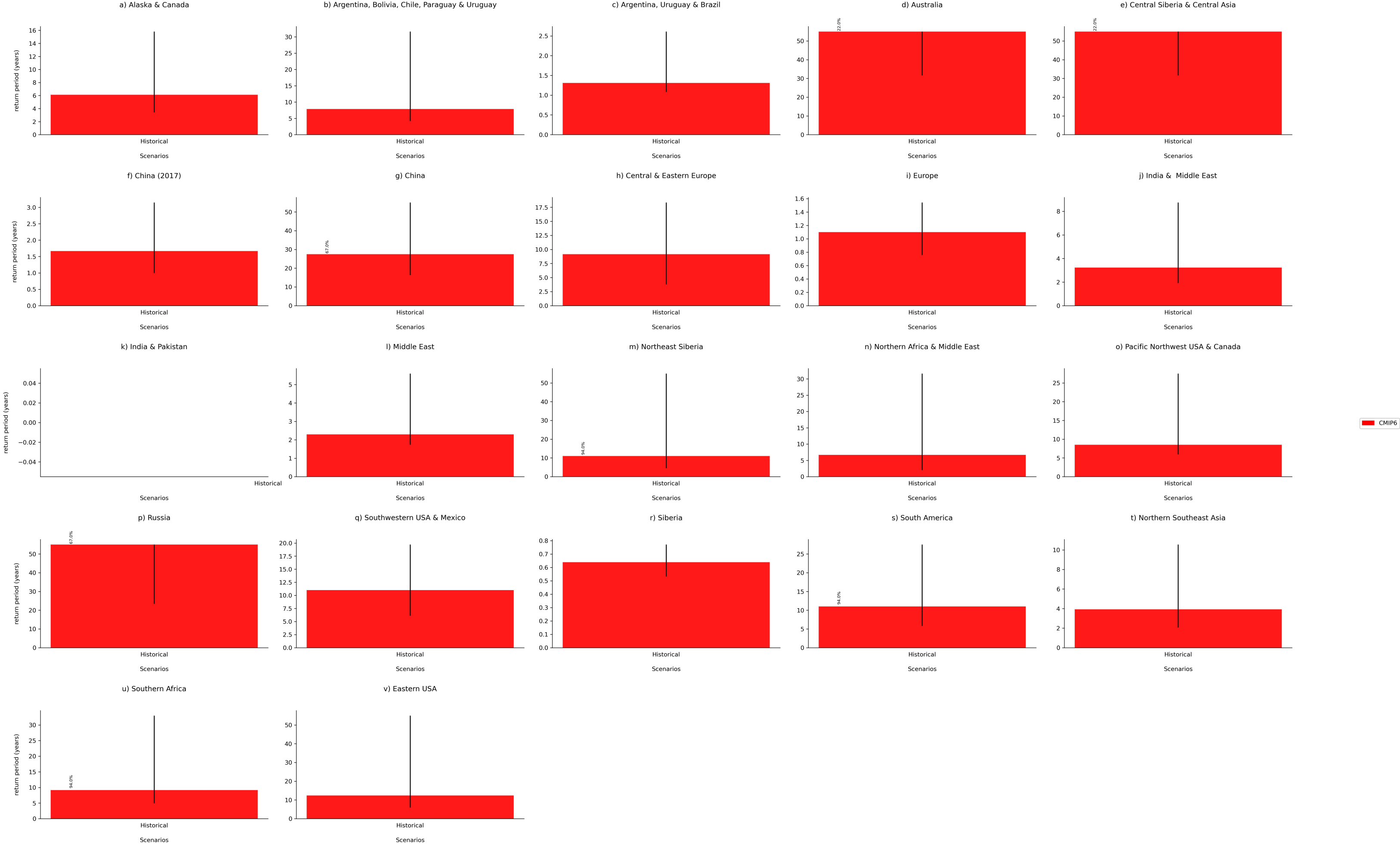

a) Alaska & Canada
b) Argentina, Bolivia, Chile, Paraguay & Uruguay
c) Argentina, Uruguay & Brazil
d) Australia
22.0%
e) Central Siberia & Central Asia
22.0%
f) China (2017)
g) China
67.0%
h) Central & Eastern Europe
i) Europe
j) India & Middle East
k) India & Pakistan
l) Middle East
m) Northeast Siberia
94.0%
n) Northern Africa & Middle East
o) Pacific Northwest USA & Canada
p) Russia
67.0%
q) Southwestern USA & Mexico
r) Siberia
s) South America
94.0%
t) Northern Southeast Asia
u) Southern Africa
94.0%
v) Eastern USA
return period (years)
Historical
Scenarios
CMIP6

Supplementary Figure 423: Return times (RT) during historical (1951-2005) based on ERA5 (Tx97.5p) - Return times (RT) (in years) during the historical period between 1951 and 2005 for Median Heatwave Area (%) of the respective observed event. For each region, values are shown for the different scenarios (increasing in emissions from left to right for the CMIP5 (green), CMIP6 (red), CanESM5 (dark blue), and ACCESS-ESM1-5 (light blue) ensembles. The error bars indicate the 5th and 95th percentile of the respective sample. Where the respective ensemble contains models not able to simulate the magnitude of the respective observed event's Median Heatwave Area (%), the proportion of models that are able to simulate an event with a value of Median Heatwave Area (%) at least as high as the observed event is indicated by the percentage values above the individual bars. The observed reference values are calculated using data from ERA5.

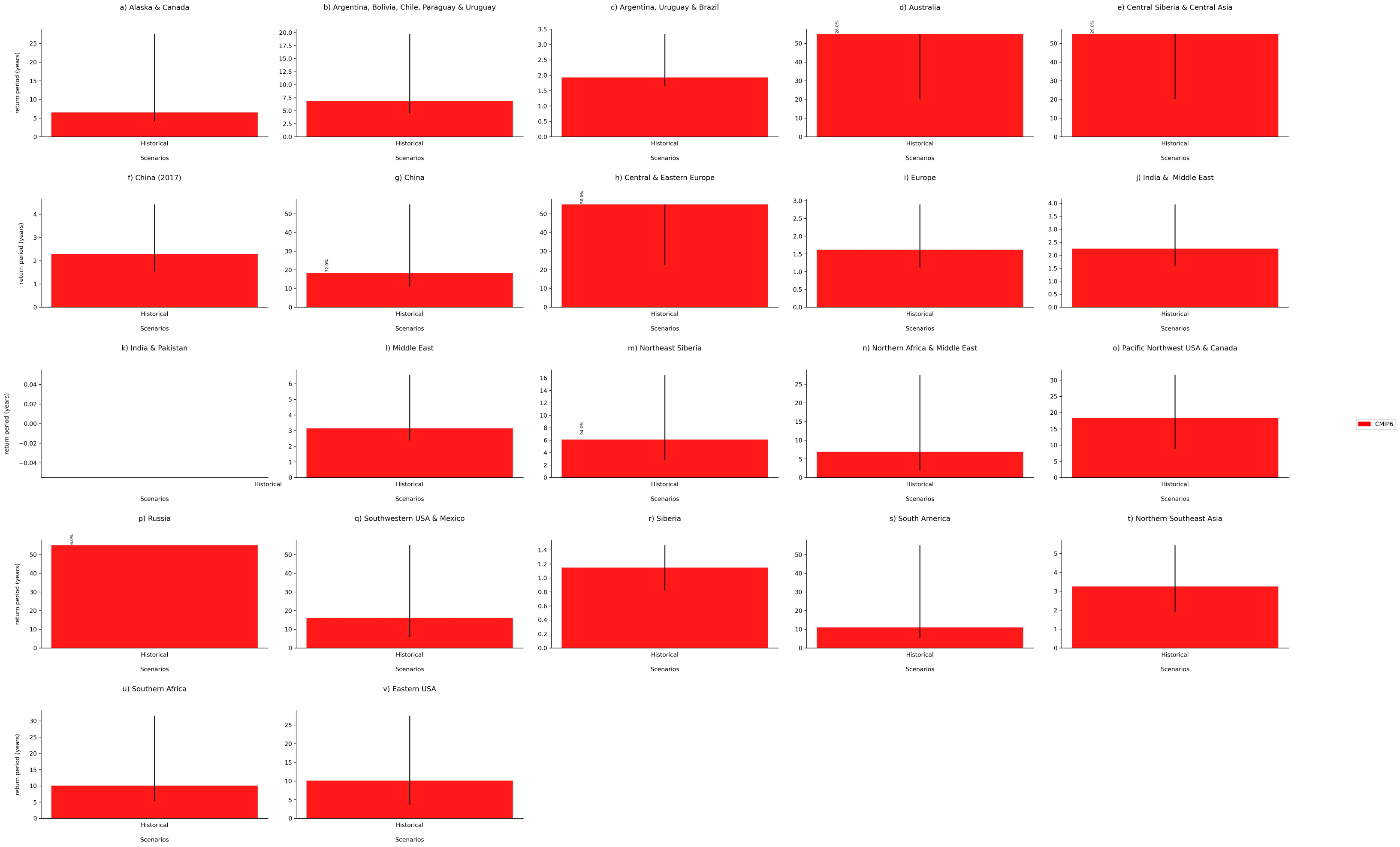

a) Alaska & Canada
b) Argentina, Bolivia, Chile, Paraguay & Uruguay
c) Argentina, Uruguay & Brazil
d) Australia
28.0%
e) Central Siberia & Central Asia
28.0%
f) China (2017)
g) China
72.0%
h) Central & Eastern Europe
56.0%
i) Europe
j) India & Middle East
k) India & Pakistan
l) Middle East
m) Northeast Siberia
94.0%
n) Northern Africa & Middle East
o) Pacific Northwest USA & Canada
p) Russia
6.0%
q) Southwestern USA & Mexico
r) Siberia
s) South America
t) Northern Southeast Asia
u) Southern Africa
v) Eastern USA
return period (years)
Historical
Scenarios
CMIP6

Supplementary Figure 424: Return times (RT) during historical (1951-2005) based on ERA5 (Tx99.0p) - Return times (RT) (in years) during the historical period between 1951 and 2005 for Median Heatwave Area (%) of the respective observed event. For each region, values are shown for the different scenarios (increasing in emissions from left to right for the CMIP5 (green), CMIP6 (red), CanESM5 (dark blue), and ACCESS-ESM1-5 (light blue) ensembles. The error bars indicate the 5th and 95th percentile of the respective sample. Where the respective ensemble contains models not able to simulate the magnitude of the respective observed event's Median Heatwave Area (%), the proportion of models that are able to simulate an event with a value of Median Heatwave Area (%) at least as high as the observed event is indicated by the percentage values above the individual bars. The observed reference values are calculated using data from ERA5.

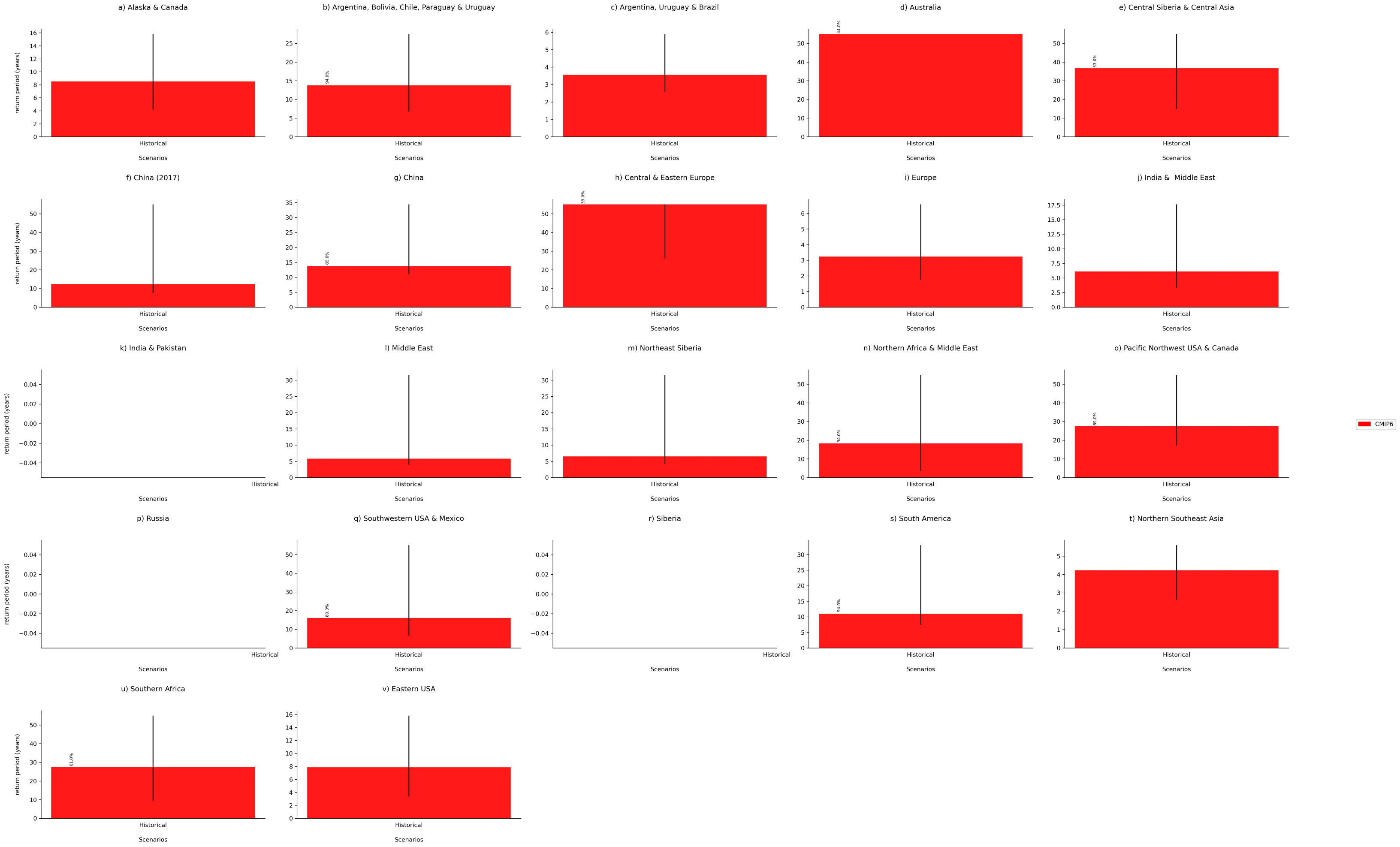

a) Alaska & Canada
b) Argentina, Bolivia, Chile, Paraguay & Uruguay
c) Argentina, Uruguay & Brazil
d) Australia
e) Central Siberia & Central Asia
f) China (2017)
g) China
h) Central & Eastern Europe
i) Europe
j) India & Middle East
k) India & Pakistan
l) Middle East
m) Northeast Siberia
n) Northern Africa & Middle East
o) Pacific Northwest USA & Canada
p) Russia
q) Southwestern USA & Mexico
r) Siberia
s) South America
t) Northern Southeast Asia
u) Southern Africa
v) Eastern USA
return period (years)
Historical
Scenarios
94.0%
44.0%
33.0%
89.0%
39.0%
61.0%
CMIP6

Supplementary Figure 425: Return times (RT) during present-to-near-future (2015-2054) based on ERA5 (Tx95.0p) - Return times (RT) (in years) during the present-to-near-future period between 2015 and 2054 for Median Heatwave Area (%) of the respective observed event. For each region, values are shown for the different scenarios (increasing in emissions from left to right for the CMIP5 (green), CMIP6 (red), CanESM5 (dark blue), and ACCESS-ESM1-5 (light blue) ensembles. The error bars indicate the 5th and 95th percentile of the respective sample. Where the respective ensemble contains models not able to simulate the magnitude of the respective observed event's Median Heatwave Area (%), the proportion of models that are able to simulate an event with a value of Median Heatwave Area (%) at least as high as the observed event is indicated by the percentage values above the individual bars. The observed reference values are calculated using data from ERA5.

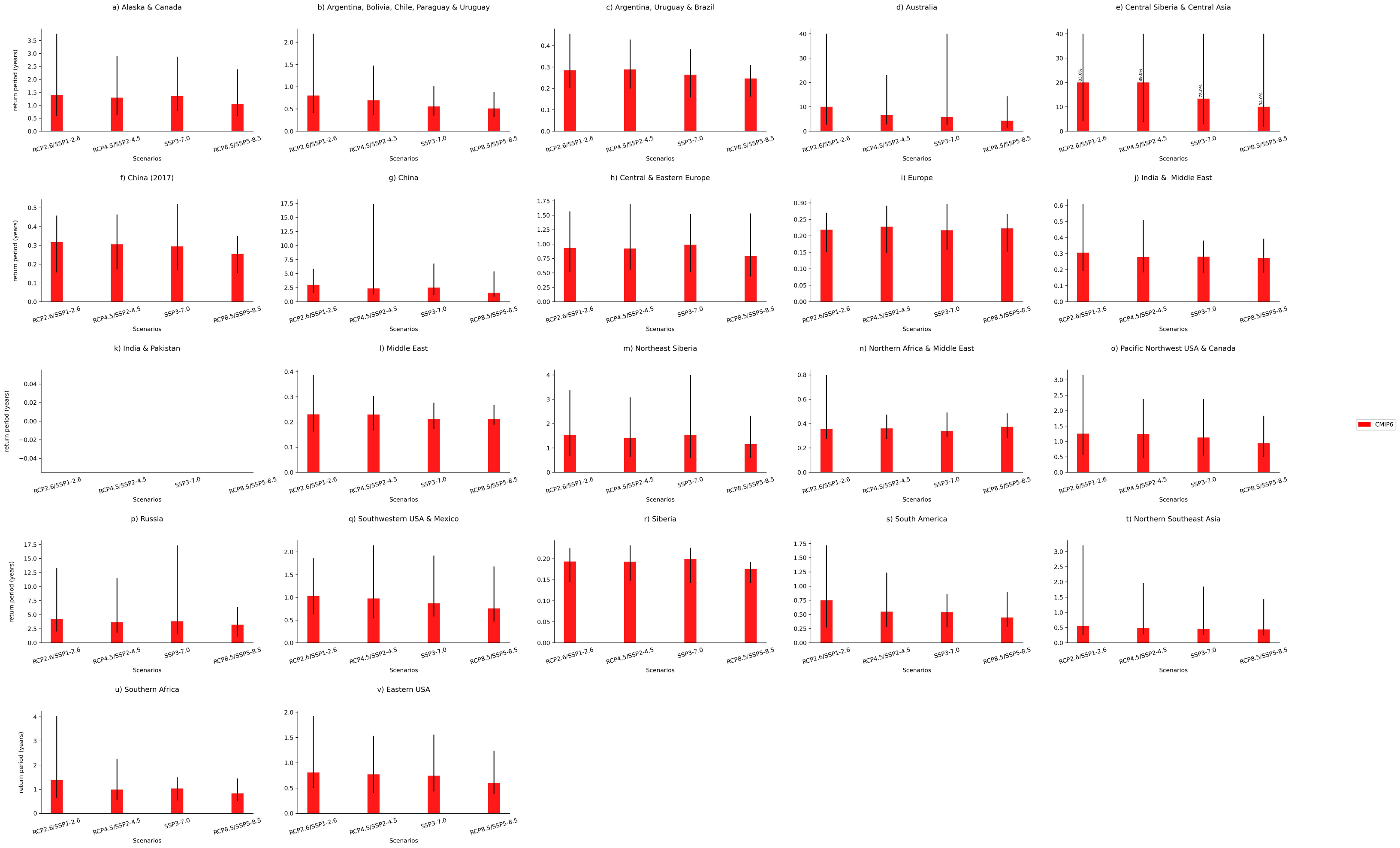

a) Alaska & Canada
b) Argentina, Bolivia, Chile, Paraguay & Uruguay
c) Argentina, Uruguay & Brazil
d) Australia
e) Central Siberia & Central Asia
83.0%
89.0%
78.0%
94.0%
f) China (2017)
g) China
h) Central & Eastern Europe
i) Europe
j) India & Middle East
k) India & Pakistan
l) Middle East
m) Northeast Siberia
n) Northern Africa & Middle East
o) Pacific Northwest USA & Canada
p) Russia
q) Southwestern USA & Mexico
r) Siberia
s) South America
t) Northern Southeast Asia
u) Southern Africa
v) Eastern USA
return period (years)
Scenarios
RCP2.6/SSP1-2.6
RCP4.5/SSP2-4.5
SSP3-7.0
RCP8.5/SSP5-8.5
CMIP6

Supplementary Figure 426: Return times (RT) during present-to-near-future (2015-2054) based on ERA5 (Tx97.5p) - Return times (RT) (in years) during the present-to-near-future period between 2015 and 2054 for Median Heatwave Area (%) of the respective observed event. For each region, values are shown for the different scenarios (increasing in emissions from left to right for the CMIP5 (green), CMIP6 (red), CanESM5 (dark blue), and ACCESS-ESM1-5 (light blue) ensembles. The error bars indicate the 5th and 95th percentile of the respective sample. Where the respective ensemble contains models not able to simulate the magnitude of the respective observed event's Median Heatwave Area (%), the proportion of models that are able to simulate an event with a value of Median Heatwave Area (%) at least as high as the observed event is indicated by the percentage values above the individual bars. The observed reference values are calculated using data from ERA5.

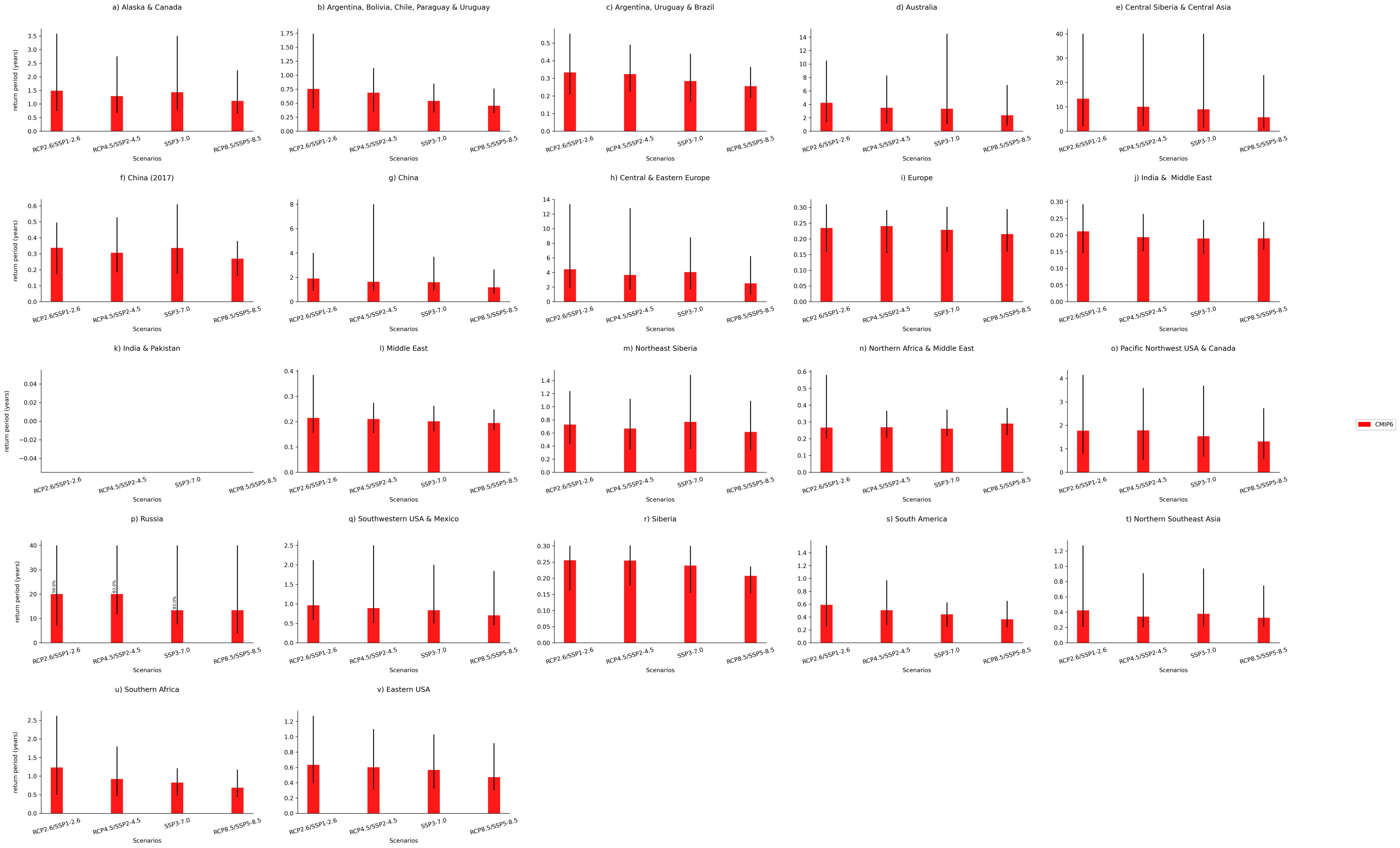

a) Alaska & Canada
b) Argentina, Bolivia, Chile, Paraguay & Uruguay
c) Argentina, Uruguay & Brazil
d) Australia
e) Central Siberia & Central Asia
f) China (2017)
g) China
h) Central & Eastern Europe
i) Europe
j) India & Middle East
k) India & Pakistan
l) Middle East
m) Northeast Siberia
n) Northern Africa & Middle East
o) Pacific Northwest USA & Canada
p) Russia
q) Southwestern USA & Mexico
r) Siberia
s) South America
t) Northern Southeast Asia
u) Southern Africa
v) Eastern USA
return period (years)
Scenarios
RCP2.6/SSP1-2.6
RCP4.5/SSP2-4.5
SSP3-7.0
RCP8.5/SSP5-8.5
56.0%
83.0%
83.0%
CMIP6

Supplementary Figure 427: Return times (RT) during present-to-near-future (2015-2054) based on ERA5 (Tx99.0p) - Return times (RT) (in years) during the present-to-near-future period between 2015 and 2054 for Median Heatwave Area (%) of the respective observed event. For each region, values are shown for the different scenarios (increasing in emissions from left to right for the CMIP5 (green), CMIP6 (red), CanESM5 (dark blue), and ACCESS-ESM1-5 (light blue) ensembles. The error bars indicate the 5th and 95th percentile of the respective sample. Where the respective ensemble contains models not able to simulate the magnitude of the respective observed event's Median Heatwave Area (%), the proportion of models that are able to simulate an event with a value of Median Heatwave Area (%) at least as high as the observed event is indicated by the percentage values above the individual bars. The observed reference values are calculated using data from ERA5.

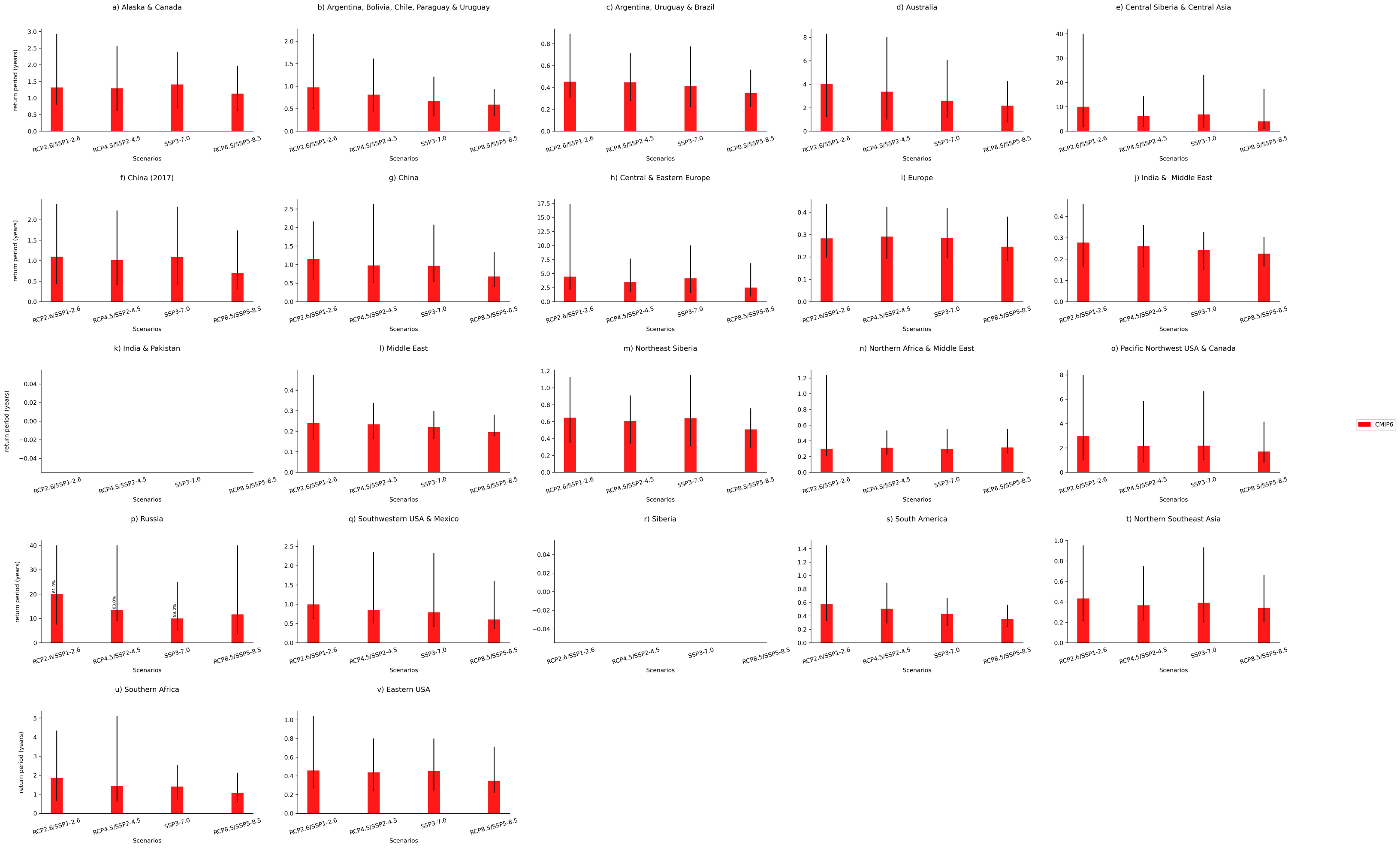

a) Alaska & Canada
b) Argentina, Bolivia, Chile, Paraguay & Uruguay
c) Argentina, Uruguay & Brazil
d) Australia
e) Central Siberia & Central Asia
f) China (2017)
g) China
h) Central & Eastern Europe
i) Europe
j) India & Middle East
k) India & Pakistan
l) Middle East
m) Northeast Siberia
n) Northern Africa & Middle East
o) Pacific Northwest USA & Canada
p) Russia
q) Southwestern USA & Mexico
r) Siberia
s) South America
t) Northern Southeast Asia
u) Southern Africa
v) Eastern USA
return period (years)
Scenarios
RCP2.6/SSP1-2.6
RCP4.5/SSP2-4.5
SSP3-7.0
RCP8.5/SSP5-8.5
61.0%
83.0%
89.0%
CMIP6

Supplementary Figure 428: Return times (RT) during end-of-century (2060-2099) based on ERA5 (Tx95.0p) - Return times (RT) (in years) during the end-of-century period between 2060 and 2099 for Median Heatwave Area (%) of the respective observed event. For each region, values are shown for the different scenarios (increasing in emissions from left to right for the CMIP5 (green), CMIP6 (red), CanESM5 (dark blue), and ACCESS-ESM1-5 (light blue) ensembles. The error bars indicate the 5th and 95th percentile of the respective sample. Where the respective ensemble contains models not able to simulate the magnitude of the respective observed event's Median Heatwave Area (%), the proportion of models that are able to simulate an event with a value of Median Heatwave Area (%) at least as high as the observed event is indicated by the percentage values above the individual bars. The observed reference values are calculated using data from ERA5.

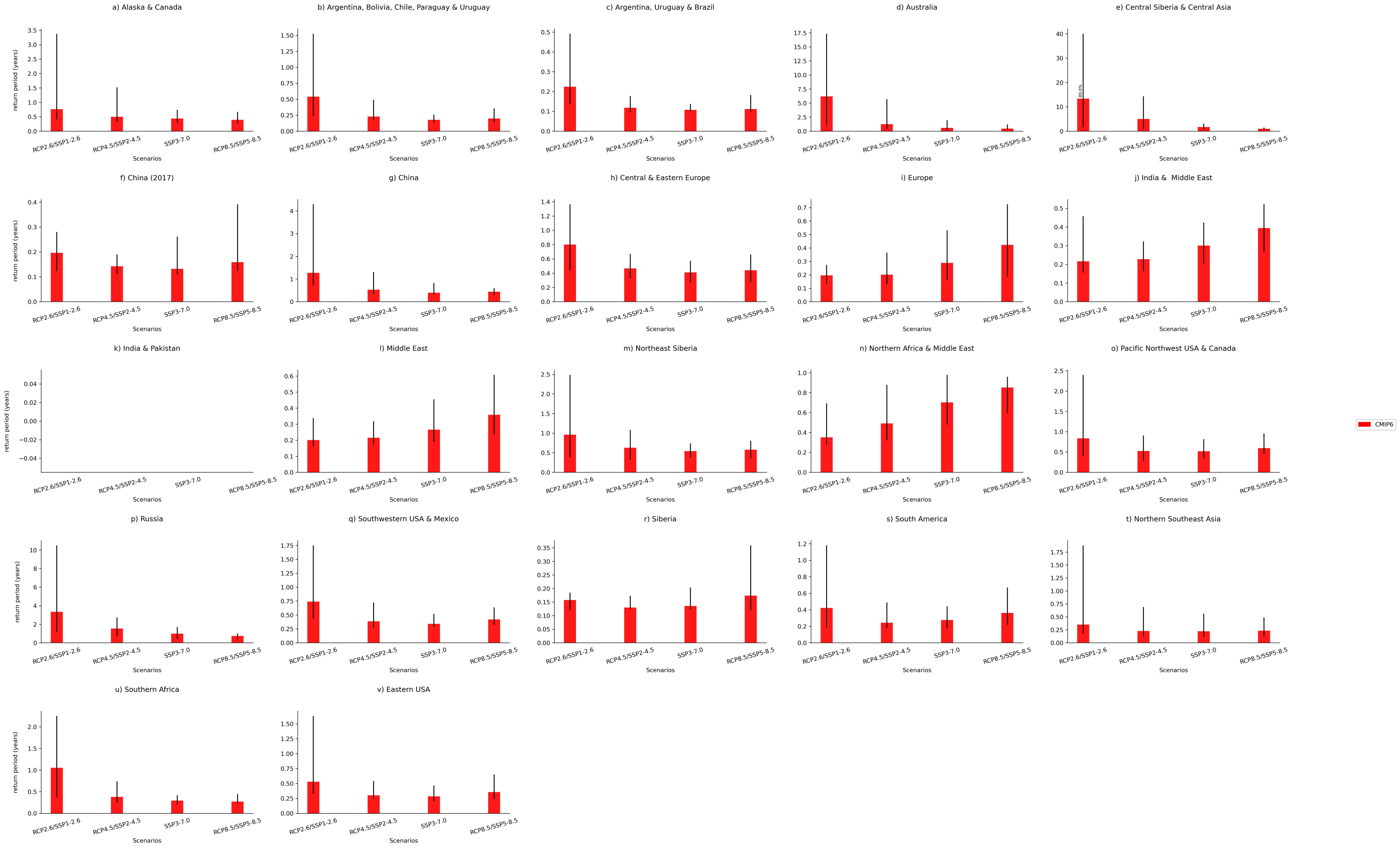

a) Alaska & Canada
b) Argentina, Bolivia, Chile, Paraguay & Uruguay
c) Argentina, Uruguay & Brazil
d) Australia
e) Central Siberia & Central Asia
f) China (2017)
g) China
h) Central & Eastern Europe
i) Europe
j) India & Middle East
k) India & Pakistan
l) Middle East
m) Northeast Siberia
n) Northern Africa & Middle East
o) Pacific Northwest USA & Canada
p) Russia
q) Southwestern USA & Mexico
r) Siberia
s) South America
t) Northern Southeast Asia
u) Southern Africa
v) Eastern USA
return period (years)
Scenarios
RCP2.6/SSP1-2.6
RCP4.5/SSP2-4.5
SSP3-7.0
RCP8.5/SSP5-8.5
89.0%
CMIP6

Supplementary Figure 429: Return times (RT) during end-of-century (2060-2099) based on ERA5 (Tx97.5p) - Return times (RT) (in years) during the end-of-century period between 2060 and 2099 for Median Heatwave Area (%) of the respective observed event. For each region, values are shown for the different scenarios (increasing in emissions from left to right for the CMIP5 (green), CMIP6 (red), CanESM5 (dark blue), and ACCESS-ESM1-5 (light blue) ensembles. The error bars indicate the 5th and 95th percentile of the respective sample. Where the respective ensemble contains models not able to simulate the magnitude of the respective observed event's Median Heatwave Area (%), the proportion of models that are able to simulate an event with a value of Median Heatwave Area (%) at least as high as the observed event is indicated by the percentage values above the individual bars. The observed reference values are calculated using data from ERA5.

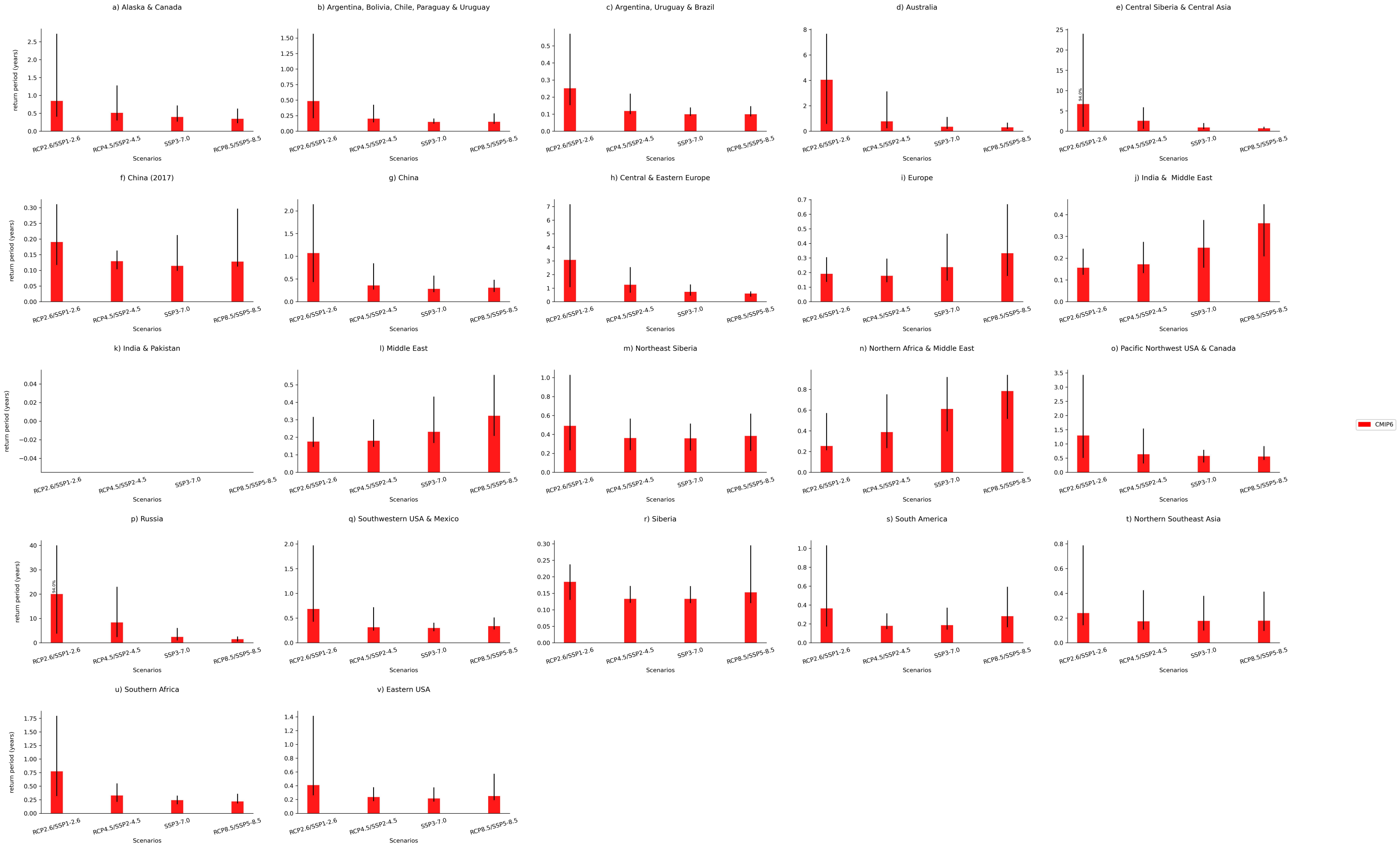
a) Alaska & Canada
b) Argentina, Bolivia, Chile, Paraguay & Uruguay
c) Argentina, Uruguay & Brazil
d) Australia
e) Central Siberia & Central Asia
f) China (2017)
g) China
h) Central & Eastern Europe
i) Europe
j) India & Middle East
k) India & Pakistan
l) Middle East
m) Northeast Siberia
n) Northern Africa & Middle East
o) Pacific Northwest USA & Canada
p) Russia
q) Southwestern USA & Mexico
r) Siberia
s) South America
t) Northern Southeast Asia
u) Southern Africa
v) Eastern USA
return period (years)
Scenarios
RCP2.6/SSP1-2.6
RCP4.5/SSP2-4.5
SSP3-7.0
RCP8.5/SSP5-8.5
94.0%
CMIP6

Supplementary Figure 430: Return times (RT) during end-of-century (2060-2099) based on ERA5 (Tx99.0p) - Return times (RT) (in years) during the end-of-century period between 2060 and 2099 for Median Heatwave Area (%) of the respective observed event. For each region, values are shown for the different scenarios (increasing in emissions from left to right for the CMIP5 (green), CMIP6 (red), CanESM5 (dark blue), and ACCESS-ESM1-5 (light blue) ensembles. The error bars indicate the 5th and 95th percentile of the respective sample. Where the respective ensemble contains models not able to simulate the magnitude of the respective observed event's Median Heatwave Area (%), the proportion of models that are able to simulate an event with a value of Median Heatwave Area (%) at least as high as the observed event is indicated by the percentage values above the individual bars. The observed reference values are calculated using data from ERA5.

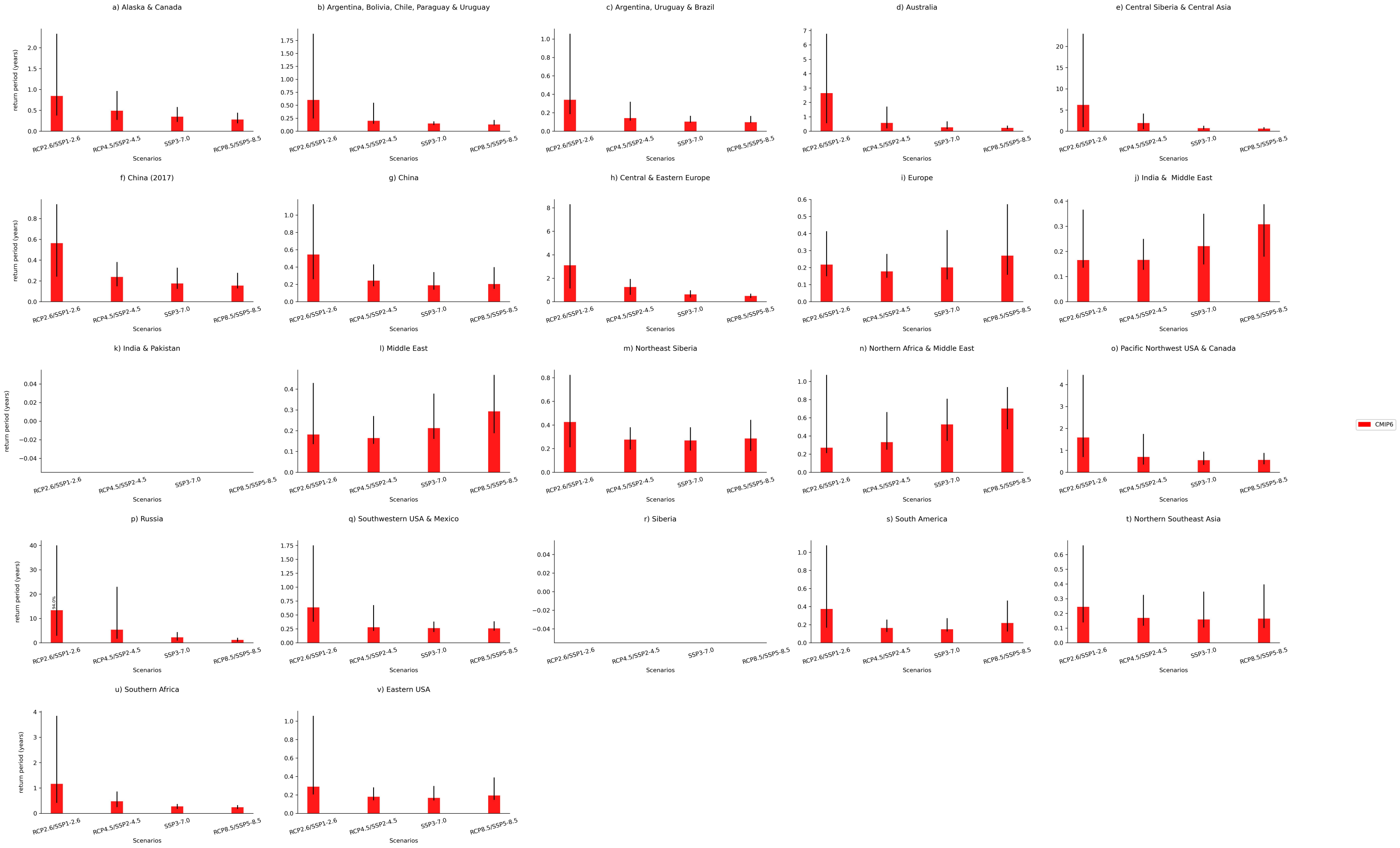

a) Alaska & Canada
b) Argentina, Bolivia, Chile, Paraguay & Uruguay
c) Argentina, Uruguay & Brazil
d) Australia
e) Central Siberia & Central Asia
f) China (2017)
g) China
h) Central & Eastern Europe
i) Europe
j) India & Middle East
k) India & Pakistan
l) Middle East
m) Northeast Siberia
n) Northern Africa & Middle East
o) Pacific Northwest USA & Canada
p) Russia
q) Southwestern USA & Mexico
r) Siberia
s) South America
t) Northern Southeast Asia
u) Southern Africa
v) Eastern USA
return period (years)
Scenarios
RCP2.6/SSP1-2.6
RCP4.5/SSP2-4.5
SSP3-7.0
RCP8.5/SSP5-8.5
94.0%
CMIP6

Supplementary Figure 431: Return times (RT) during historical (1951-2005) based on Berkeley Earth (Tx95.0p) - Return times (RT) (in years) during the historical period between 1951 and 2005 for Median Heatwave Severity of the respective observed event. For each region, values are shown for the different scenarios (increasing in emissions from left to right for the CMIP5 (green), CMIP6 (red), CanESM5 (dark blue), and ACCESS-ESM1-5 (light blue) ensembles. The error bars indicate the 5th and 95th percentile of the respective sample. Where the respective ensemble contains models not able to simulate the magnitude of the respective observed event's Median Heatwave Severity, the proportion of models that are able to simulate an event with a value of Median Heatwave Severity at least as high as the observed event is indicated by the percentage values above the individual bars. The observed reference values are calculated using data from Berkeley Earth.

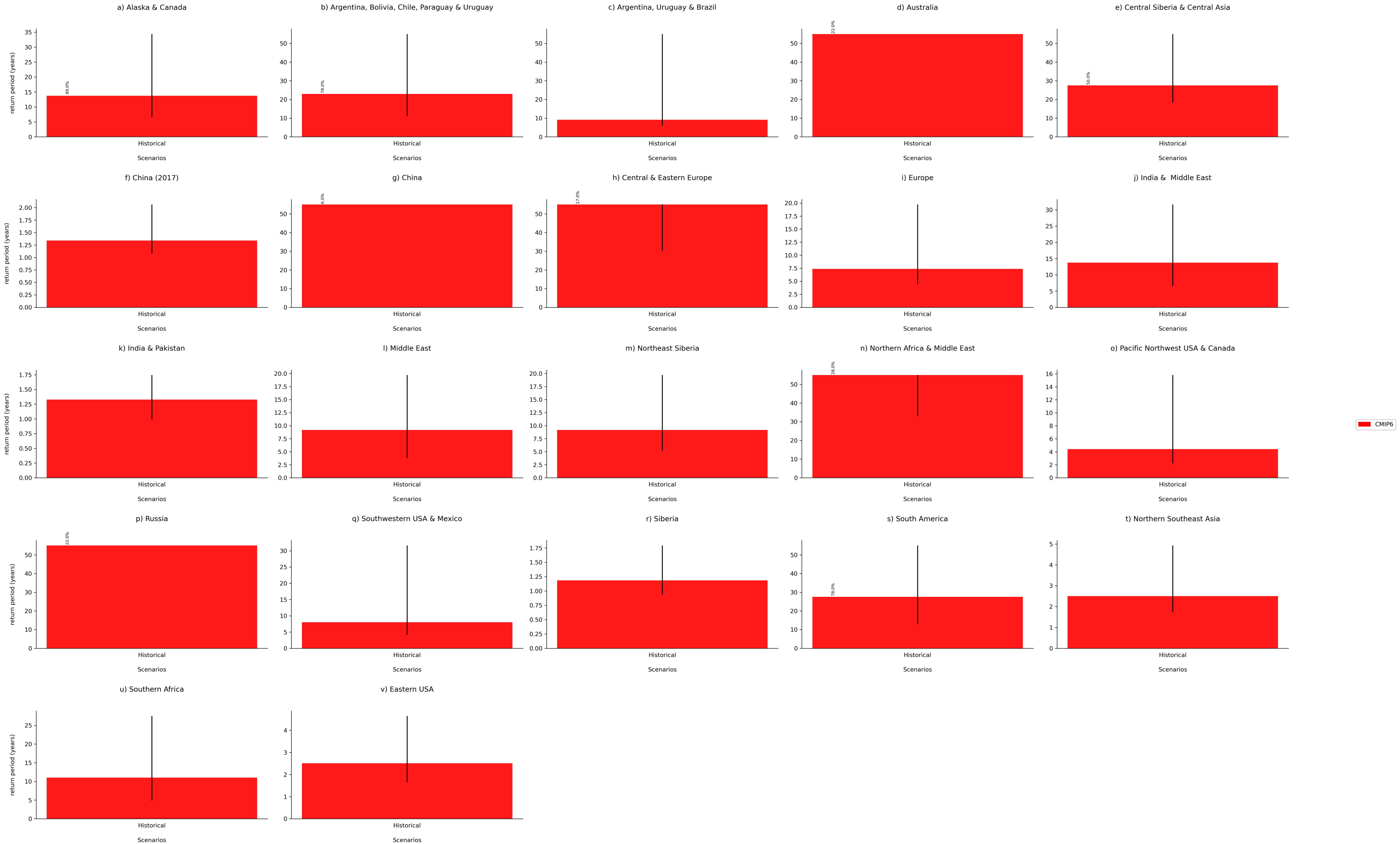
a) Alaska & Canada
b) Argentina, Bolivia, Chile, Paraguay & Uruguay
c) Argentina, Uruguay & Brazil
d) Australia
e) Central Siberia & Central Asia
f) China (2017)
g) China
h) Central & Eastern Europe
i) Europe
j) India & Middle East
k) India & Pakistan
l) Middle East
m) Northeast Siberia
n) Northern Africa & Middle East
o) Pacific Northwest USA & Canada
p) Russia
q) Southwestern USA & Mexico
r) Siberia
s) South America
t) Northern Southeast Asia
u) Southern Africa
v) Eastern USA
return period (years)
Historical
Scenarios
CMIP6

Supplementary Figure 432: Return times (RT) during historical (1951-2005) based on Berkeley Earth (Tx97.5p) - Return times (RT) (in years) during the historical period between 1951 and 2005 for Median Heatwave Severity of the respective observed event. For each region, values are shown for the different scenarios (increasing in emissions from left to right for the CMIP5 (green), CMIP6 (red), CanESM5 (dark blue), and ACCESS-ESM1-5 (light blue) ensembles. The error bars indicate the 5th and 95th percentile of the respective sample. Where the respective ensemble contains models not able to simulate the magnitude of the respective observed event's Median Heatwave Severity, the proportion of models that are able to simulate an event with a value of Median Heatwave Severity at least as high as the observed event is indicated by the percentage values above the individual bars. The observed reference values are calculated using data from Berkeley Earth.

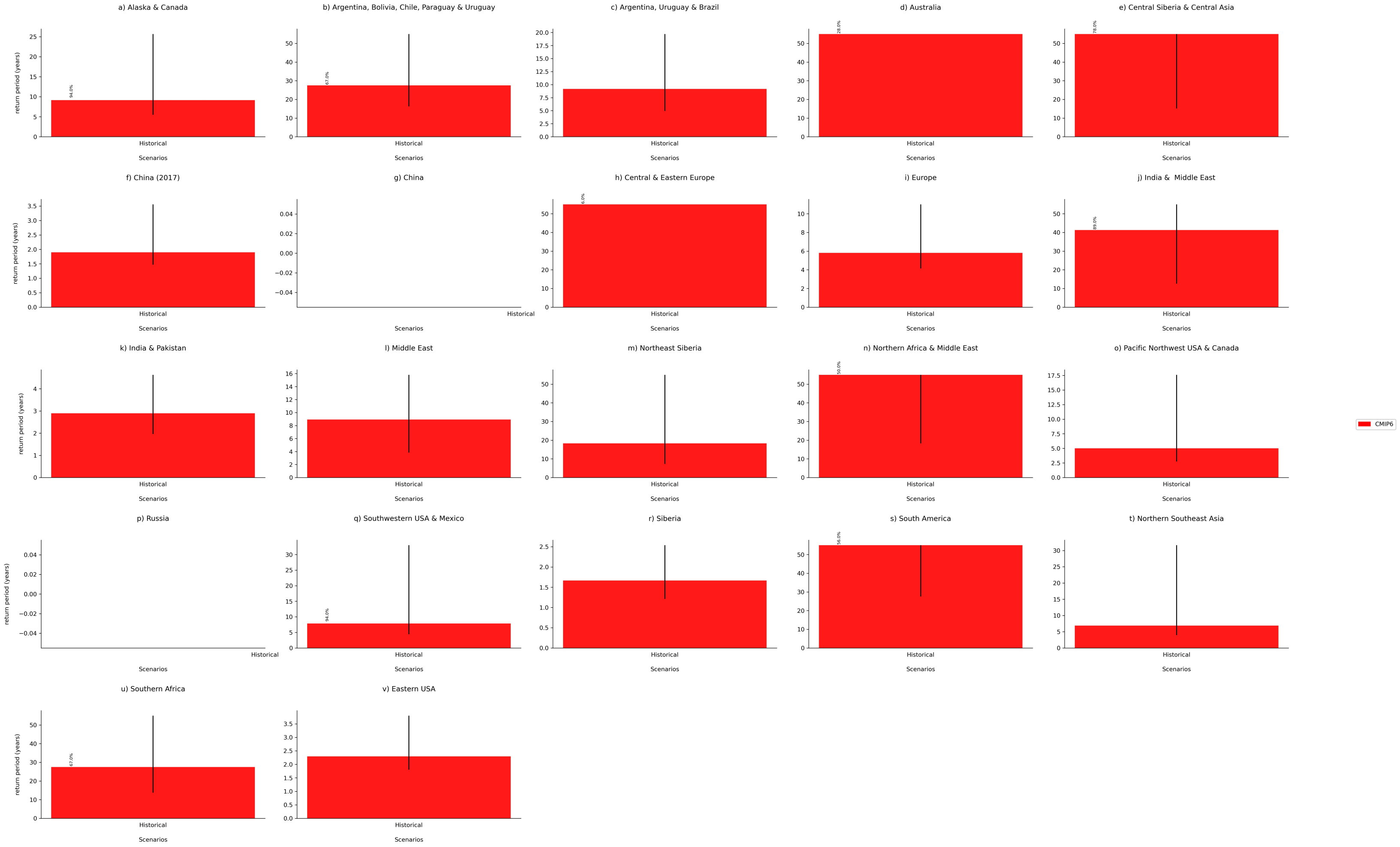
a) Alaska & Canada
94.0%
b) Argentina, Bolivia, Chile, Paraguay & Uruguay
67.0%
c) Argentina, Uruguay & Brazil
d) Australia
28.0%
e) Central Siberia & Central Asia
78.0%
f) China (2017)
g) China
h) Central & Eastern Europe
6.0%
i) Europe
j) India & Middle East
89.0%
k) India & Pakistan
l) Middle East
m) Northeast Siberia
n) Northern Africa & Middle East
50.0%
o) Pacific Northwest USA & Canada
p) Russia
q) Southwestern USA & Mexico
94.0%
r) Siberia
s) South America
56.0%
t) Northern Southeast Asia
u) Southern Africa
67.0%
v) Eastern USA
return period (years)
Historical
Scenarios
CMIP6

Supplementary Figure 433: Return times (RT) during historical (1951-2005) based on Berkeley Earth (Tx99.0p) - Return times (RT) (in years) during the historical period between 1951 and 2005 for Median Heatwave Severity of the respective observed event. For each region, values are shown for the different scenarios (increasing in emissions from left to right for the CMIP5 (green), CMIP6 (red), CanESM5 (dark blue), and ACCESS-ESM1-5 (light blue) ensembles. The error bars indicate the 5th and 95th percentile of the respective sample. Where the respective ensemble contains models not able to simulate the magnitude of the respective observed event's Median Heatwave Severity, the proportion of models that are able to simulate an event with a value of Median Heatwave Severity at least as high as the observed event is indicated by the percentage values above the individual bars. The observed reference values are calculated using data from Berkeley Earth.

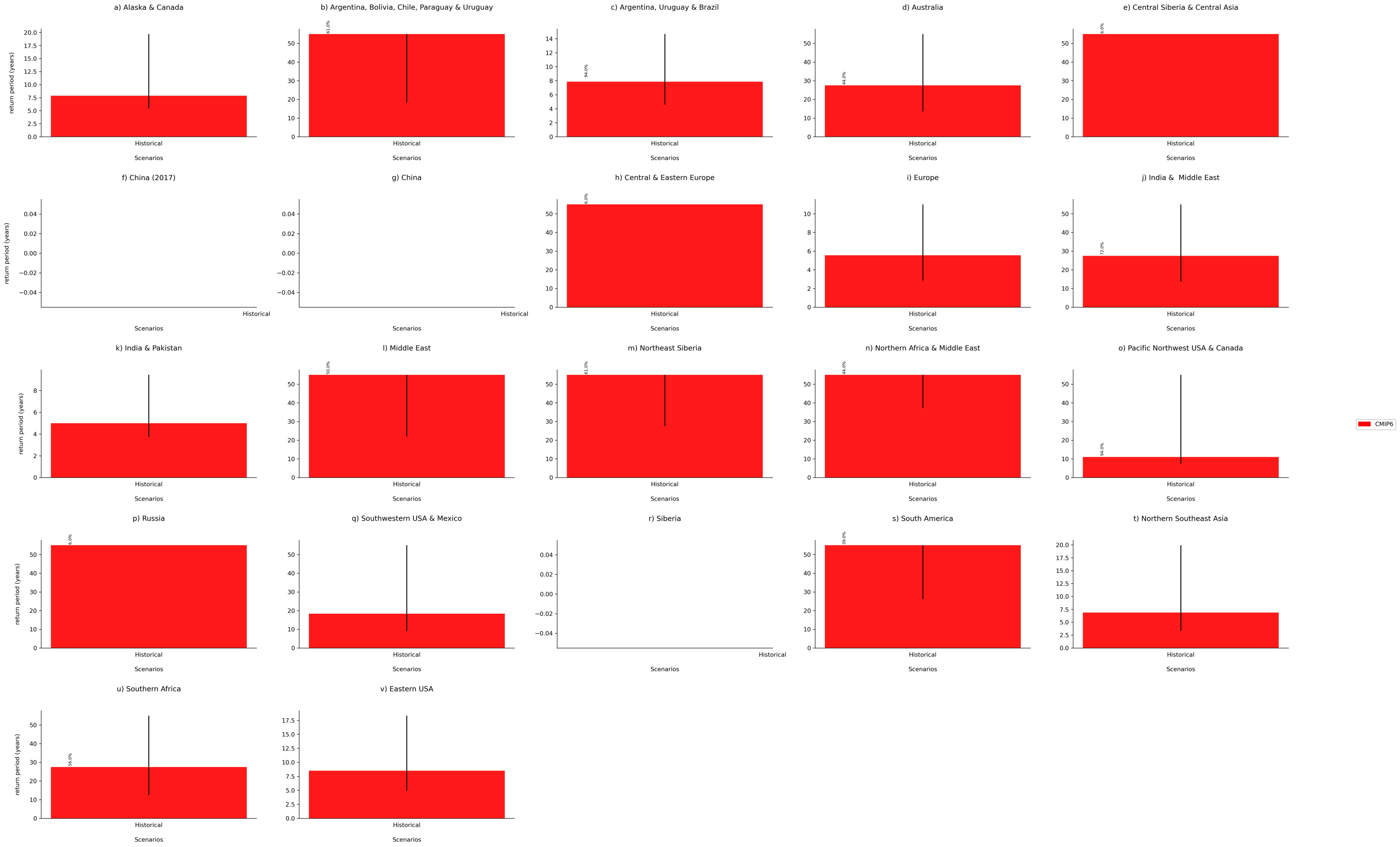
a) Alaska & Canada
b) Argentina, Bolivia, Chile, Paraguay & Uruguay
c) Argentina, Uruguay & Brazil
d) Australia
e) Central Siberia & Central Asia
f) China (2017)
g) China
h) Central & Eastern Europe
i) Europe
j) India & Middle East
k) India & Pakistan
l) Middle East
m) Northeast Siberia
n) Northern Africa & Middle East
o) Pacific Northwest USA & Canada
p) Russia
q) Southwestern USA & Mexico
r) Siberia
s) South America
t) Northern Southeast Asia
u) Southern Africa
v) Eastern USA
return period (years)
Historical
Scenarios
CMIP6

Supplementary Figure 434: Return times (RT) during present-to-near-future (2015-2054) based on Berkeley Earth (Tx95.0p) - Return times (RT) (in years) during the present-to-near-future period between 2015 and 2054 for Median Heatwave Severity of the respective observed event. For each region, values are shown for the different scenarios (increasing in emissions from left to right for the CMIP5 (green), CMIP6 (red), CanESM5 (dark blue), and ACCESS-ESM1-5 (light blue) ensembles. The error bars indicate the 5th and 95th percentile of the respective sample. Where the respective ensemble contains models not able to simulate the magnitude of the respective observed event's Median Heatwave Severity, the proportion of models that are able to simulate an event with a value of Median Heatwave Severity at least as high as the observed event is indicated by the percentage values above the individual bars. The observed reference values are calculated using data from Berkeley Earth.

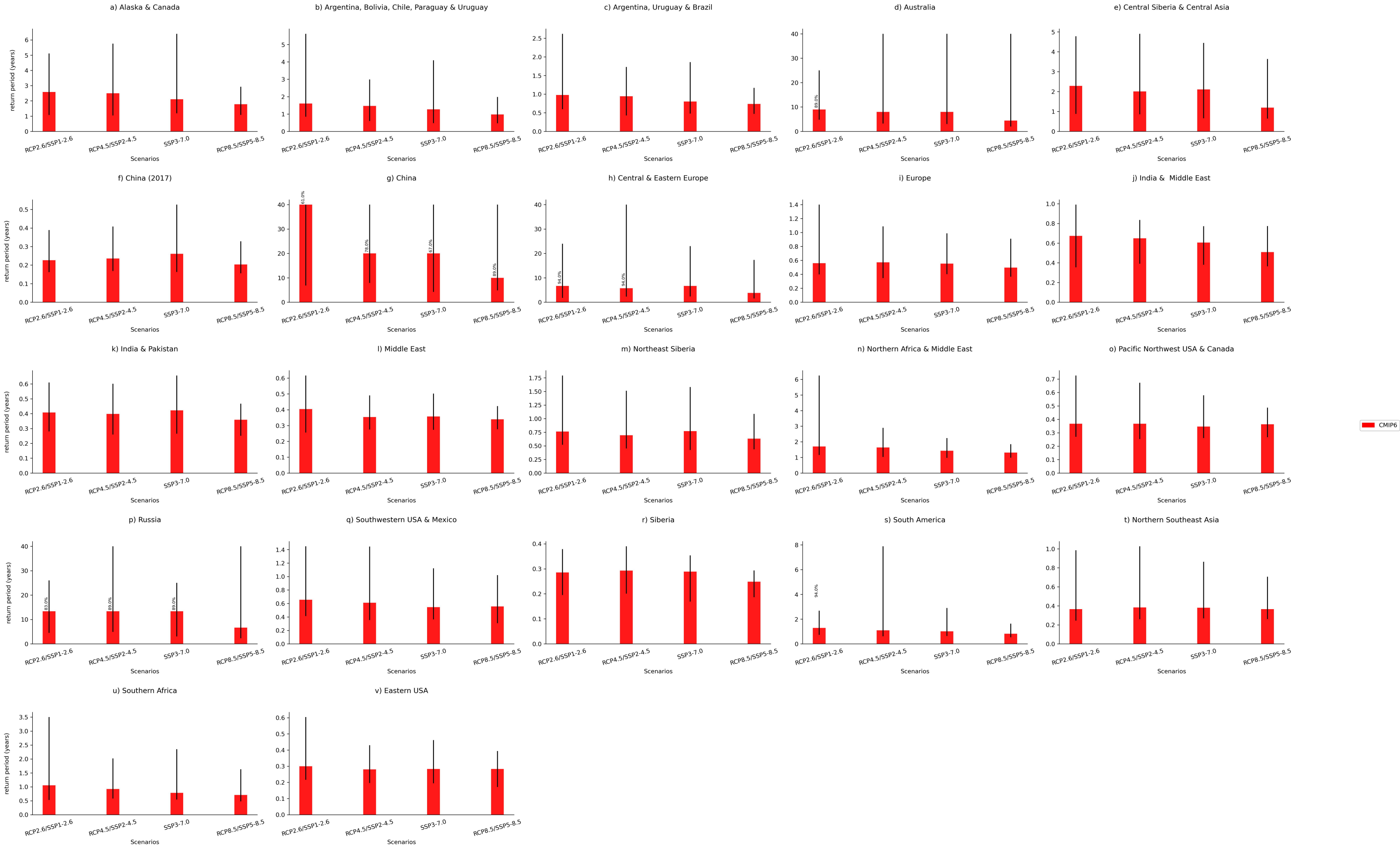
a) Alaska & Canada
b) Argentina, Bolivia, Chile, Paraguay & Uruguay
c) Argentina, Uruguay & Brazil
d) Australia
e) Central Siberia & Central Asia
f) China (2017)
g) China
h) Central & Eastern Europe
i) Europe
j) India & Middle East
k) India & Pakistan
l) Middle East
m) Northeast Siberia
n) Northern Africa & Middle East
o) Pacific Northwest USA & Canada
p) Russia
q) Southwestern USA & Mexico
r) Siberia
s) South America
t) Northern Southeast Asia
u) Southern Africa
v) Eastern USA
return period (years)
Scenarios
RCP2.6/SSP1-2.6
RCP4.5/SSP2-4.5
SSP3-7.0
RCP8.5/SSP5-8.5
CMIP6
89.0%
61.0%
78.0%
67.0%
89.0%
94.0%
94.0%
83.0%
89.0%
89.0%
94.0%

Supplementary Figure 435: Return times (RT) during present-to-near-future (2015-2054) based on Berkeley Earth (Tx97.5p) - Return times (RT) (in years) during the present-to-near-future period between 2015 and 2054 for Median Heatwave Severity of the respective observed event. For each region, values are shown for the different scenarios (increasing in emissions from left to right for the CMIP5 (green), CMIP6 (red), CanESM5 (dark blue), and ACCESS-ESM1-5 (light blue) ensembles. The error bars indicate the 5th and 95th percentile of the respective sample. Where the respective ensemble contains models not able to simulate the magnitude of the respective observed event's Median Heatwave Severity, the proportion of models that are able to simulate an event with a value of Median Heatwave Severity at least as high as the observed event is indicated by the percentage values above the individual bars. The observed reference values are calculated using data from Berkeley Earth.

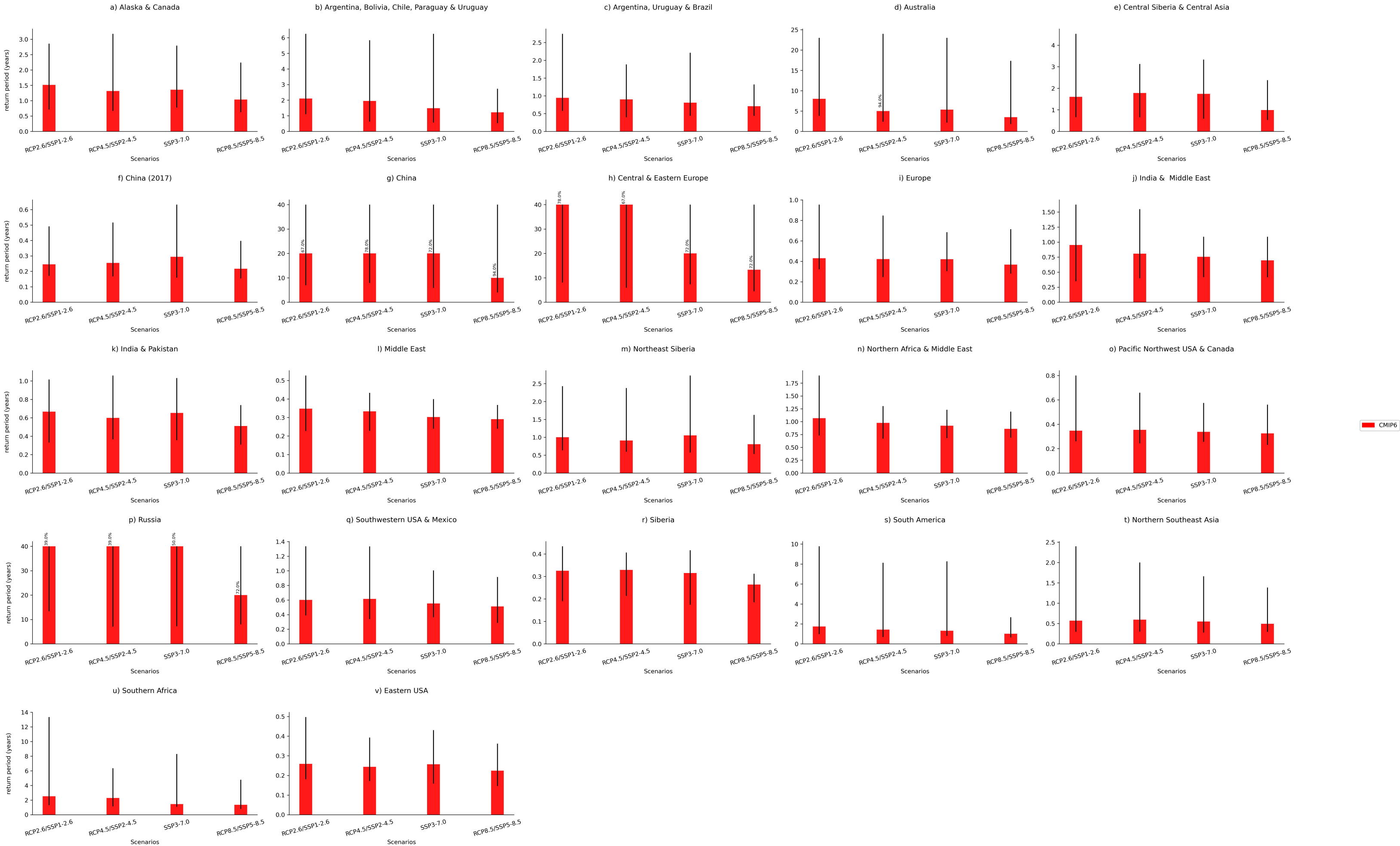
a) Alaska & Canada
b) Argentina, Bolivia, Chile, Paraguay & Uruguay
c) Argentina, Uruguay & Brazil
d) Australia
e) Central Siberia & Central Asia
f) China (2017)
g) China
h) Central & Eastern Europe
i) Europe
j) India & Middle East
k) India & Pakistan
l) Middle East
m) Northeast Siberia
n) Northern Africa & Middle East
o) Pacific Northwest USA & Canada
p) Russia
q) Southwestern USA & Mexico
r) Siberia
s) South America
t) Northern Southeast Asia
u) Southern Africa
v) Eastern USA
return period (years)
Scenarios
RCP2.6/SSP1-2.6
RCP4.5/SSP2-4.5
SSP3-7.0
RCP8.5/SSP5-8.5
94.0%
67.0%
78.0%
72.0%
39.0%
50.0%
CMIP6

Supplementary Figure 436: Return times (RT) during present-to-near-future (2015-2054) based on Berkeley Earth (Tx99.0p) - Return times (RT) (in years) during the present-to-near-future period between 2015 and 2054 for Median Heatwave Severity of the respective observed event. For each region, values are shown for the different scenarios (increasing in emissions from left to right for the CMIP5 (green), CMIP6 (red), CanESM5 (dark blue), and ACCESS-ESM1-5 (light blue) ensembles. The error bars indicate the 5th and 95th percentile of the respective sample. Where the respective ensemble contains models not able to simulate the magnitude of the respective observed event's Median Heatwave Severity, the proportion of models that are able to simulate an event with a value of Median Heatwave Severity at least as high as the observed event is indicated by the percentage values above the individual bars. The observed reference values are calculated using data from Berkeley Earth.

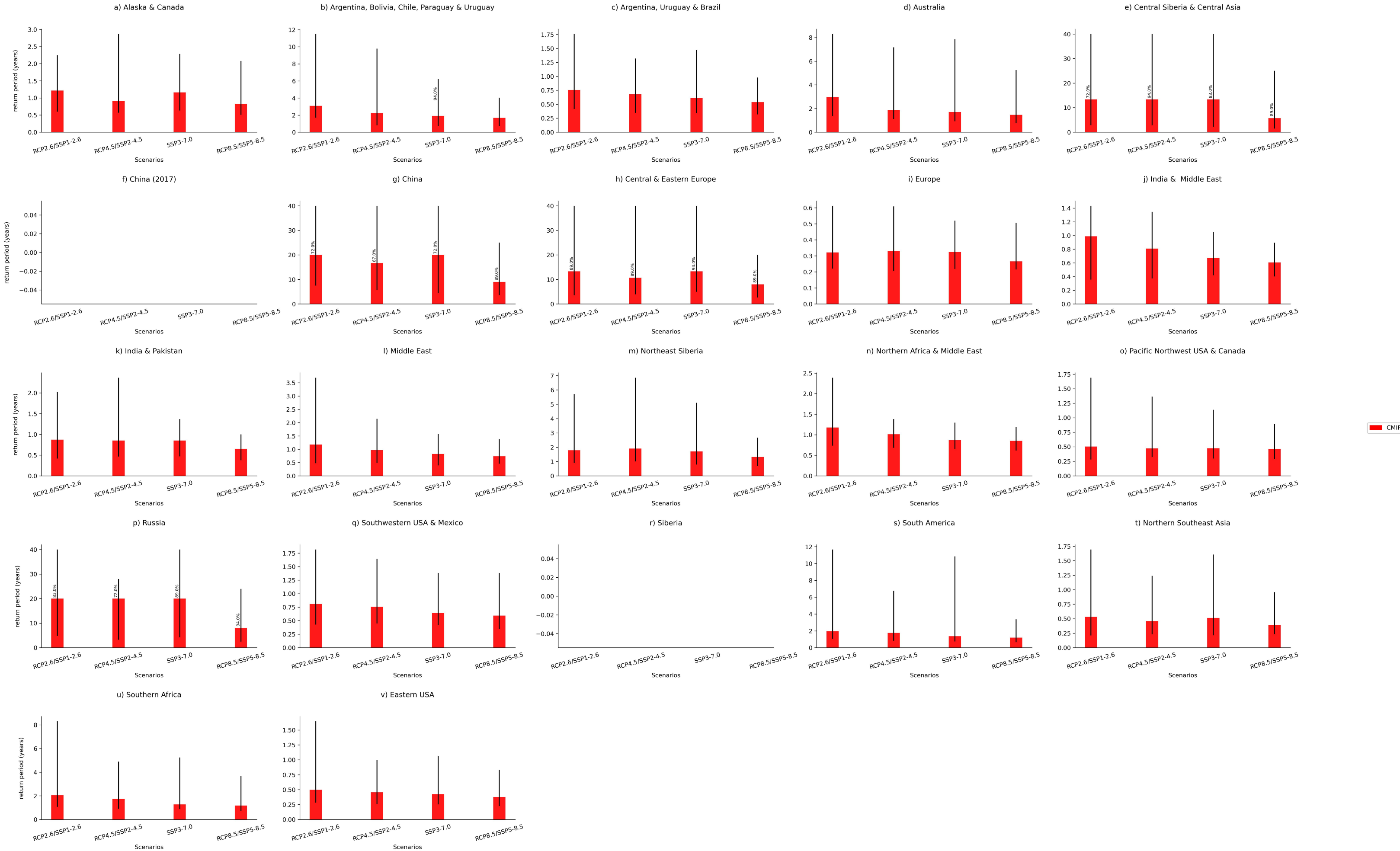

a) Alaska & Canada
b) Argentina, Bolivia, Chile, Paraguay & Uruguay
c) Argentina, Uruguay & Brazil
d) Australia
e) Central Siberia & Central Asia
f) China (2017)
g) China
h) Central & Eastern Europe
i) Europe
j) India & Middle East
k) India & Pakistan
l) Middle East
m) Northeast Siberia
n) Northern Africa & Middle East
o) Pacific Northwest USA & Canada
p) Russia
q) Southwestern USA & Mexico
r) Siberia
s) South America
t) Northern Southeast Asia
u) Southern Africa
v) Eastern USA
return period (years)
Scenarios
RCP2.6/SSP1-2.6
RCP4.5/SSP2-4.5
SSP3-7.0
RCP8.5/SSP5-8.5
CMIP6

Supplementary Figure 437: Return times (RT) during end-of-century (2060-2099) based on Berkeley Earth (Tx95.0p) - Return times (RT) (in years) during the end-of-century period between 2060 and 2099 for Median Heatwave Severity of the respective observed event. For each region, values are shown for the different scenarios (increasing in emissions from left to right for the CMIP5 (green), CMIP6 (red), CanESM5 (dark blue), and ACCESS-ESM1-5 (light blue) ensembles. The error bars indicate the 5th and 95th percentile of the respective sample. Where the respective ensemble contains models not able to simulate the magnitude of the respective observed event's Median Heatwave Severity, the proportion of models that are able to simulate an event with a value of Median Heatwave Severity at least as high as the observed event is indicated by the percentage values above the individual bars. The observed reference values are calculated using data from Berkeley Earth.

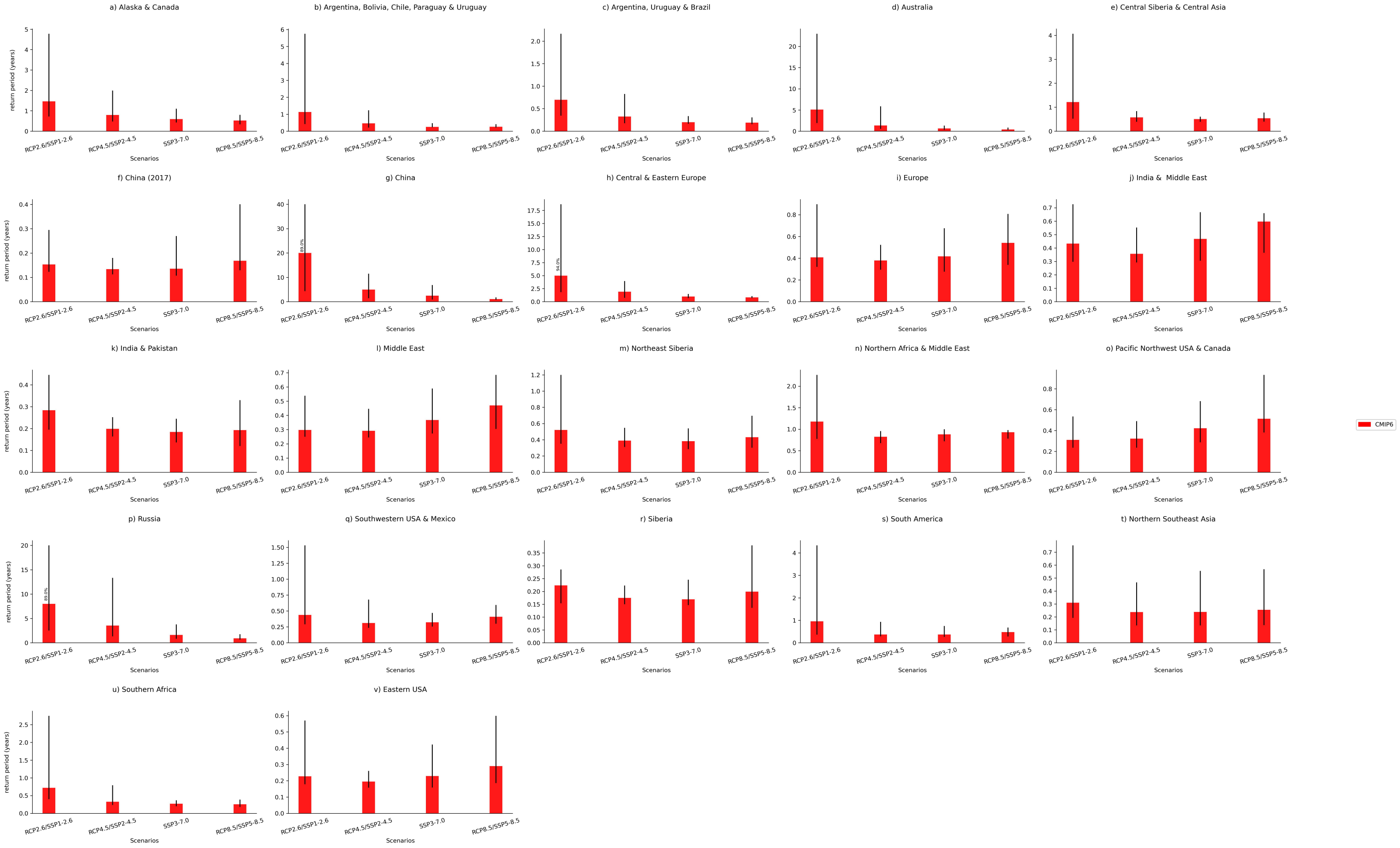

a) Alaska & Canada
b) Argentina, Bolivia, Chile, Paraguay & Uruguay
c) Argentina, Uruguay & Brazil
d) Australia
e) Central Siberia & Central Asia
f) China (2017)
g) China
h) Central & Eastern Europe
i) Europe
j) India & Middle East
k) India & Pakistan
l) Middle East
m) Northeast Siberia
n) Northern Africa & Middle East
o) Pacific Northwest USA & Canada
p) Russia
q) Southwestern USA & Mexico
r) Siberia
s) South America
t) Northern Southeast Asia
u) Southern Africa
v) Eastern USA
return period (years)
Scenarios
RCP2.6/SSP1-2.6
RCP4.5/SSP2-4.5
SSP3-7.0
RCP8.5/SSP5-8.5
89.0%
94.0%
CMIP6

Supplementary Figure 438: Return times (RT) during end-of-century (2060-2099) based on Berkeley Earth (Tx97.5p) - Return times (RT) (in years) during the end-of-century period between 2060 and 2099 for Median Heatwave Severity of the respective observed event. For each region, values are shown for the different scenarios (increasing in emissions from left to right for the CMIP5 (green), CMIP6 (red), CanESM5 (dark blue), and ACCESS-ESM1-5 (light blue) ensembles. The error bars indicate the 5th and 95th percentile of the respective sample. Where the respective ensemble contains models not able to simulate the magnitude of the respective observed event's Median Heatwave Severity, the proportion of models that are able to simulate an event with a value of Median Heatwave Severity at least as high as the observed event is indicated by the percentage values above the individual bars. The observed reference values are calculated using data from Berkeley Earth.

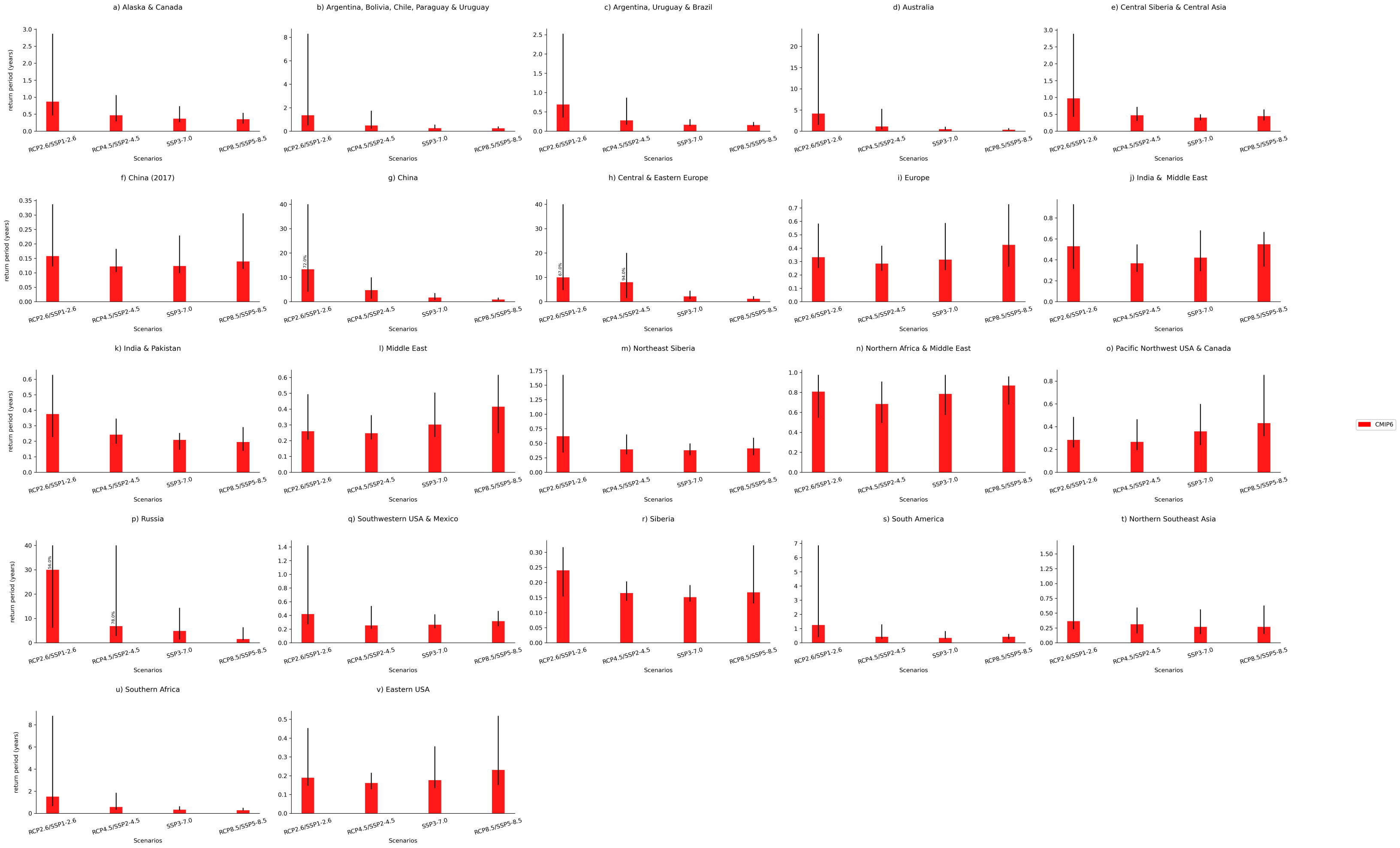

a) Alaska & Canada
b) Argentina, Bolivia, Chile, Paraguay & Uruguay
c) Argentina, Uruguay & Brazil
d) Australia
e) Central Siberia & Central Asia
f) China (2017)
g) China
h) Central & Eastern Europe
i) Europe
j) India & Middle East
k) India & Pakistan
l) Middle East
m) Northeast Siberia
n) Northern Africa & Middle East
o) Pacific Northwest USA & Canada
p) Russia
q) Southwestern USA & Mexico
r) Siberia
s) South America
t) Northern Southeast Asia
u) Southern Africa
v) Eastern USA
return period (years)
Scenarios
RCP2.6/SSP1-2.6
RCP4.5/SSP2-4.5
SSP3-7.0
RCP8.5/SSP5-8.5
72.0%
67.0%
94.0%
56.0%
78.0%
CMIP6

Supplementary Figure 439: Return times (RT) during end-of-century (2060-2099) based on Berkeley Earth (Tx99.0p) - Return times (RT) (in years) during the end-of-century period between 2060 and 2099 for Median Heatwave Severity of the respective observed event. For each region, values are shown for the different scenarios (increasing in emissions from left to right for the CMIP5 (green), CMIP6 (red), CanESM5 (dark blue), and ACCESS-ESM1-5 (light blue) ensembles. The error bars indicate the 5th and 95th percentile of the respective sample. Where the respective ensemble contains models not able to simulate the magnitude of the respective observed event's Median Heatwave Severity, the proportion of models that are able to simulate an event with a value of Median Heatwave Severity at least as high as the observed event is indicated by the percentage values above the individual bars. The observed reference values are calculated using data from Berkeley Earth.

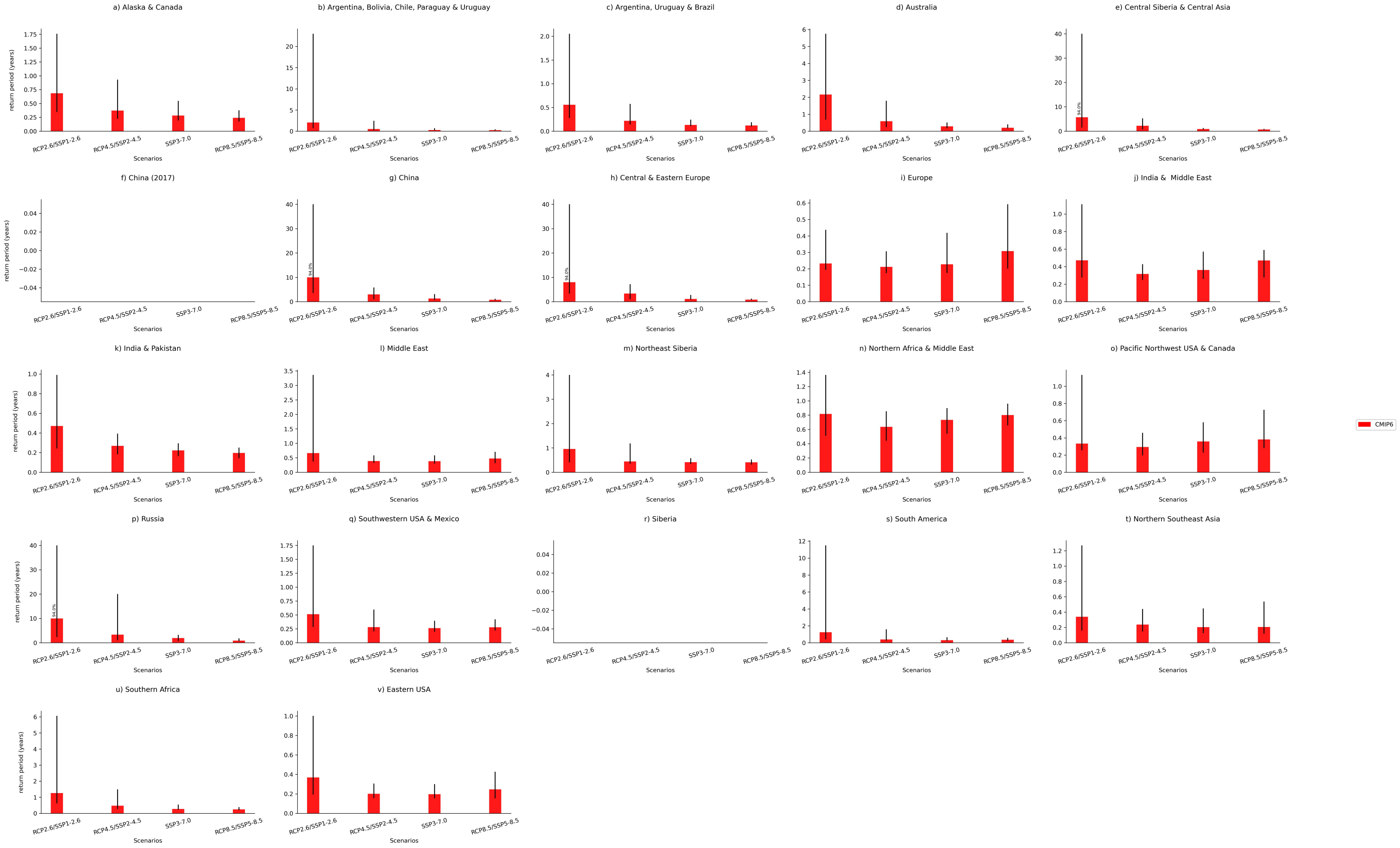
a) Alaska & Canada
b) Argentina, Bolivia, Chile, Paraguay & Uruguay
c) Argentina, Uruguay & Brazil
d) Australia
e) Central Siberia & Central Asia
f) China (2017)
g) China
h) Central & Eastern Europe
i) Europe
j) India & Middle East
k) India & Pakistan
l) Middle East
m) Northeast Siberia
n) Northern Africa & Middle East
o) Pacific Northwest USA & Canada
p) Russia
q) Southwestern USA & Mexico
r) Siberia
s) South America
t) Northern Southeast Asia
u) Southern Africa
v) Eastern USA
return period (years)
Scenarios
RCP2.6/SSP1-2.6
RCP4.5/SSP2-4.5
SSP3-7.0
RCP8.5/SSP5-8.5
94.0%
CMIP6

Supplementary Figure 440: Return times (RT) during historical (1951-2005) based on ERA5 (Tx95.0p) - Return times (RT) (in years) during the historical period between 1951 and 2005 for Median Heatwave Severity of the respective observed event. For each region, values are shown for the different scenarios (increasing in emissions from left to right for the CMIP5 (green), CMIP6 (red), CanESM5 (dark blue), and ACCESS-ESM1-5 (light blue) ensembles. The error bars indicate the 5th and 95th percentile of the respective sample. Where the respective ensemble contains models not able to simulate the magnitude of the respective observed event's Median Heatwave Severity, the proportion of models that are able to simulate an event with a value of Median Heatwave Severity at least as high as the observed event is indicated by the percentage values above the individual bars. The observed reference values are calculated using data from ERA5.

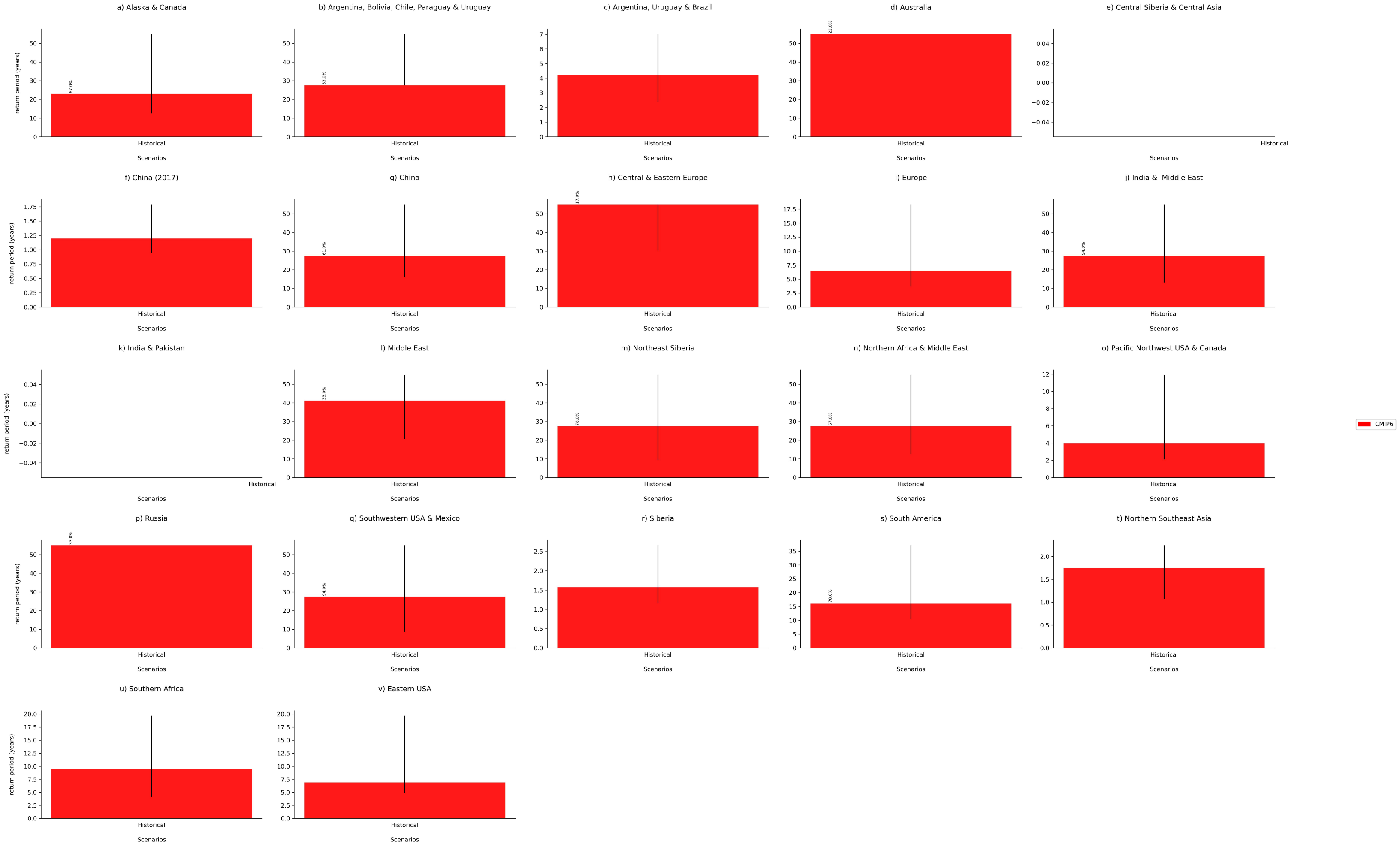
a) Alaska & Canada
67.0%
b) Argentina, Bolivia, Chile, Paraguay & Uruguay
33.0%
c) Argentina, Uruguay & Brazil
d) Australia
22.0%
e) Central Siberia & Central Asia
f) China (2017)
g) China
61.0%
h) Central & Eastern Europe
17.0%
i) Europe
j) India & Middle East
94.0%
k) India & Pakistan
l) Middle East
33.0%
m) Northeast Siberia
78.0%
n) Northern Africa & Middle East
67.0%
o) Pacific Northwest USA & Canada
p) Russia
33.0%
q) Southwestern USA & Mexico
94.0%
r) Siberia
s) South America
78.0%
t) Northern Southeast Asia
u) Southern Africa
v) Eastern USA
return period (years)
Historical
Scenarios
CMIP6

Supplementary Figure 441: Return times (RT) during historical (1951-2005) based on ERA5 (Tx97.5p) - Return times (RT) (in years) during the historical period between 1951 and 2005 for Median Heatwave Severity of the respective observed event. For each region, values are shown for the different scenarios (increasing in emissions from left to right for the CMIP5 (green), CMIP6 (red), CanESM5 (dark blue), and ACCESS-ESM1-5 (light blue) ensembles. The error bars indicate the 5th and 95th percentile of the respective sample. Where the respective ensemble contains models not able to simulate the magnitude of the respective observed event's Median Heatwave Severity, the proportion of models that are able to simulate an event with a value of Median Heatwave Severity at least as high as the observed event is indicated by the percentage values above the individual bars. The observed reference values are calculated using data from ERA5.

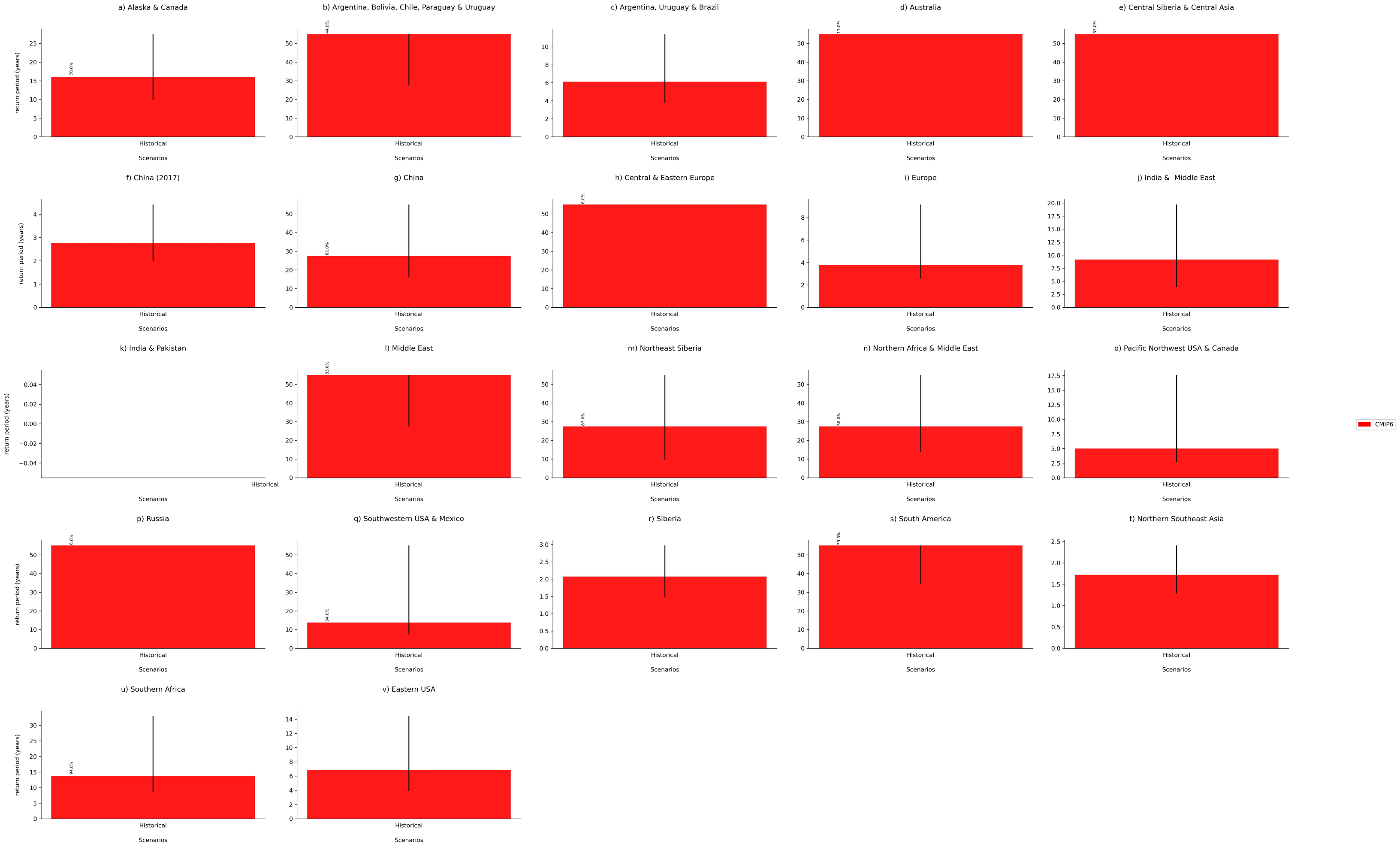
a) Alaska & Canada
b) Argentina, Bolivia, Chile, Paraguay & Uruguay
c) Argentina, Uruguay & Brazil
d) Australia
e) Central Siberia & Central Asia
f) China (2017)
g) China
h) Central & Eastern Europe
i) Europe
j) India & Middle East
k) India & Pakistan
l) Middle East
m) Northeast Siberia
n) Northern Africa & Middle East
o) Pacific Northwest USA & Canada
p) Russia
q) Southwestern USA & Mexico
r) Siberia
s) South America
t) Northern Southeast Asia
u) Southern Africa
v) Eastern USA
return period (years)
Historical
Scenarios
CMIP6

Supplementary Figure 442: Return times (RT) during historical (1951-2005) based on ERA5 (Tx99.0p) - Return times (RT) (in years) during the historical period between 1951 and 2005 for Median Heatwave Severity of the respective observed event. For each region, values are shown for the different scenarios (increasing in emissions from left to right for the CMIP5 (green), CMIP6 (red), CanESM5 (dark blue), and ACCESS-ESM1-5 (light blue) ensembles. The error bars indicate the 5th and 95th percentile of the respective sample. Where the respective ensemble contains models not able to simulate the magnitude of the respective observed event's Median Heatwave Severity, the proportion of models that are able to simulate an event with a value of Median Heatwave Severity at least as high as the observed event is indicated by the percentage values above the individual bars. The observed reference values are calculated using data from ERA5.

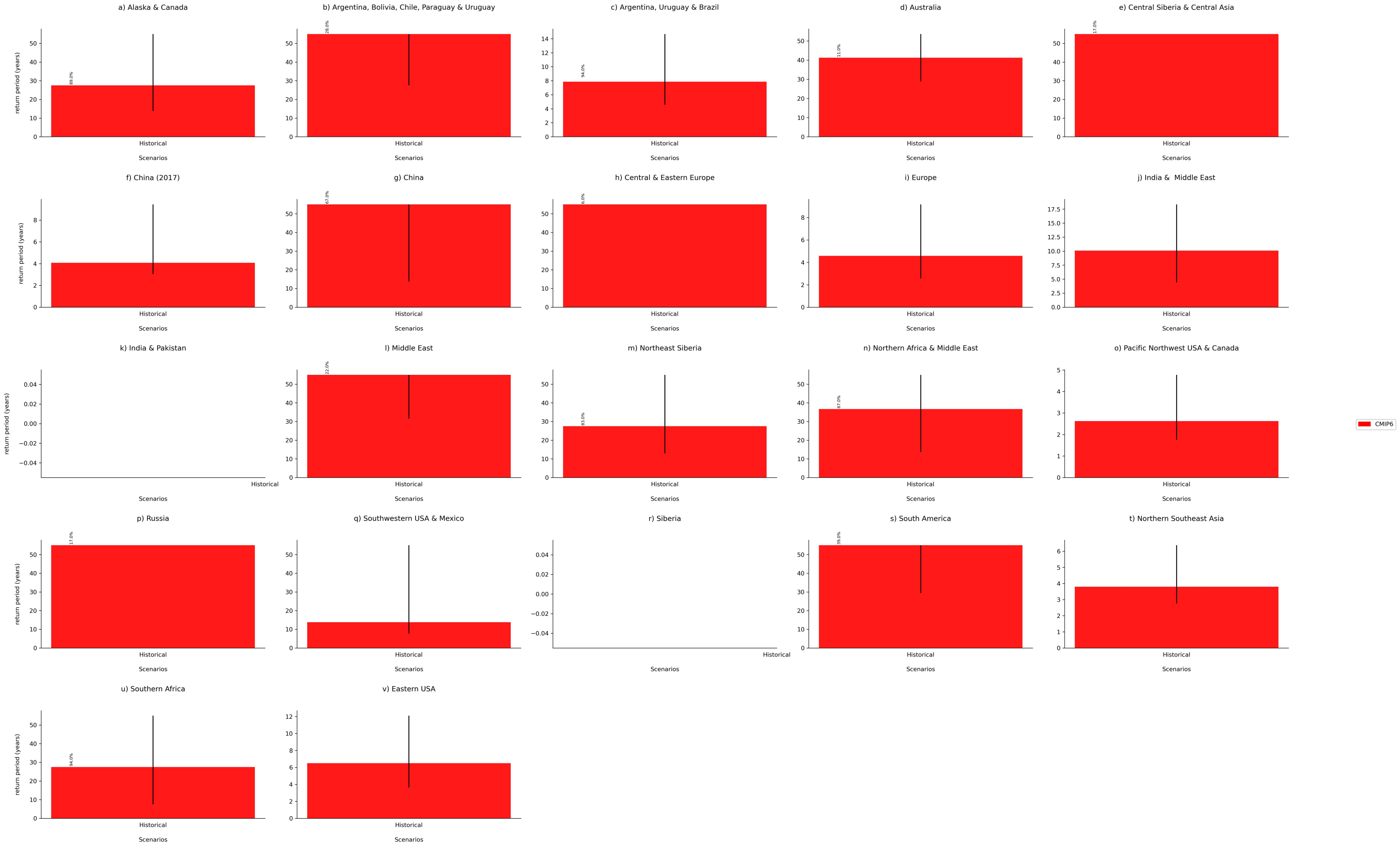

a) Alaska & Canada
b) Argentina, Bolivia, Chile, Paraguay & Uruguay
c) Argentina, Uruguay & Brazil
d) Australia
e) Central Siberia & Central Asia
f) China (2017)
g) China
h) Central & Eastern Europe
i) Europe
j) India & Middle East
k) India & Pakistan
l) Middle East
m) Northeast Siberia
n) Northern Africa & Middle East
o) Pacific Northwest USA & Canada
p) Russia
q) Southwestern USA & Mexico
r) Siberia
s) South America
t) Northern Southeast Asia
u) Southern Africa
v) Eastern USA
return period (years)
Historical
Scenarios
CMIP6

Supplementary Figure 443: Return times (RT) during present-to-near-future (2015-2054) based on ERA5 (Tx95.0p) - Return times (RT) (in years) during the present-to-near-future period between 2015 and 2054 for Median Heatwave Severity of the respective observed event. For each region, values are shown for the different scenarios (increasing in emissions from left to right for the CMIP5 (green), CMIP6 (red), CanESM5 (dark blue), and ACCESS-ESM1-5 (light blue) ensembles. The error bars indicate the 5th and 95th percentile of the respective sample. Where the respective ensemble contains models not able to simulate the magnitude of the respective observed event's Median Heatwave Severity, the proportion of models that are able to simulate an event with a value of Median Heatwave Severity at least as high as the observed event is indicated by the percentage values above the individual bars. The observed reference values are calculated using data from ERA5.

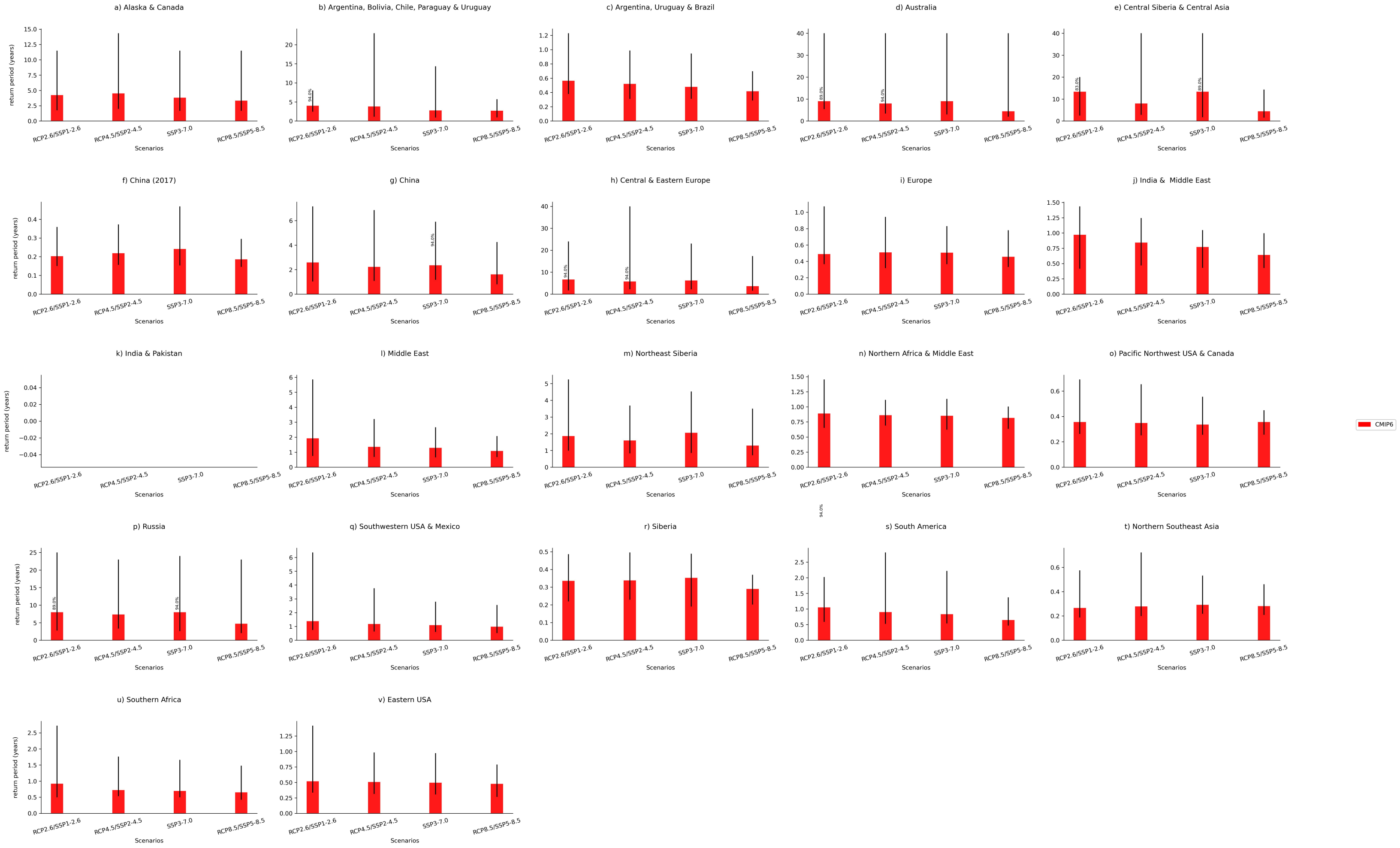
a) Alaska & Canada
b) Argentina, Bolivia, Chile, Paraguay & Uruguay
c) Argentina, Uruguay & Brazil
d) Australia
e) Central Siberia & Central Asia
f) China (2017)
g) China
h) Central & Eastern Europe
i) Europe
j) India & Middle East
k) India & Pakistan
l) Middle East
m) Northeast Siberia
n) Northern Africa & Middle East
o) Pacific Northwest USA & Canada
p) Russia
q) Southwestern USA & Mexico
r) Siberia
s) South America
t) Northern Southeast Asia
u) Southern Africa
v) Eastern USA
return period (years)
Scenarios
RCP2.6/SSP1-2.6
RCP4.5/SSP2-4.5
SSP3-7.0
RCP8.5/SSP5-8.5
CMIP6

Supplementary Figure 444: Return times (RT) during present-to-near-future (2015-2054) based on ERA5 (Tx97.5p) - Return times (RT) (in years) during the present-to-near-future period between 2015 and 2054 for Median Heatwave Severity of the respective observed event. For each region, values are shown for the different scenarios (increasing in emissions from left to right for the CMIP5 (green), CMIP6 (red), CanESM5 (dark blue), and ACCESS-ESM1-5 (light blue) ensembles. The error bars indicate the 5th and 95th percentile of the respective sample. Where the respective ensemble contains models not able to simulate the magnitude of the respective observed event's Median Heatwave Severity, the proportion of models that are able to simulate an event with a value of Median Heatwave Severity at least as high as the observed event is indicated by the percentage values above the individual bars. The observed reference values are calculated using data from ERA5.

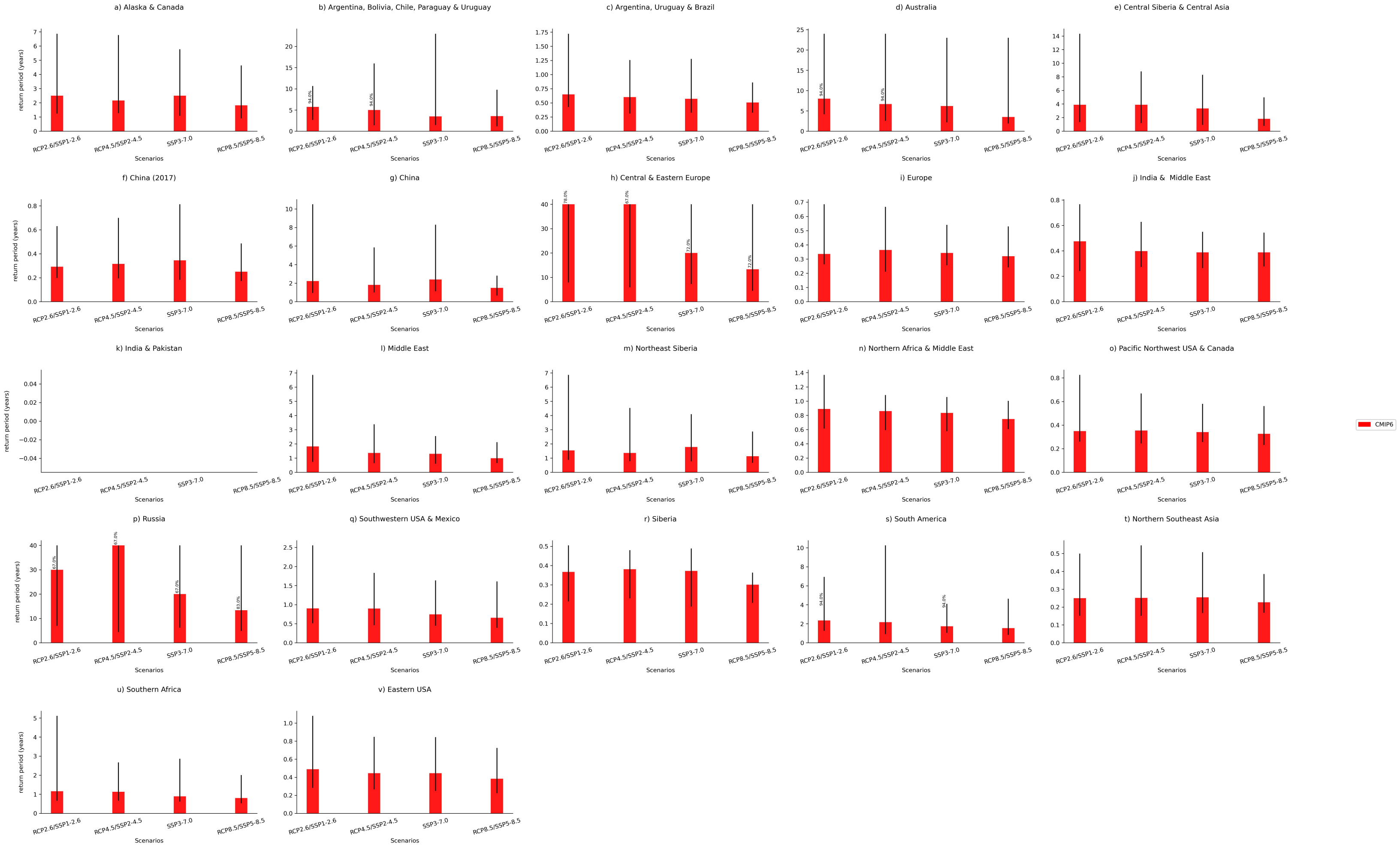

a) Alaska & Canada
b) Argentina, Bolivia, Chile, Paraguay & Uruguay
c) Argentina, Uruguay & Brazil
d) Australia
e) Central Siberia & Central Asia
f) China (2017)
g) China
h) Central & Eastern Europe
i) Europe
j) India & Middle East
k) India & Pakistan
l) Middle East
m) Northeast Siberia
n) Northern Africa & Middle East
o) Pacific Northwest USA & Canada
p) Russia
q) Southwestern USA & Mexico
r) Siberia
s) South America
t) Northern Southeast Asia
u) Southern Africa
v) Eastern USA
return period (years)
Scenarios
RCP2.6/SSP1-2.6
RCP4.5/SSP2-4.5
SSP3-7.0
RCP8.5/SSP5-8.5
94.0%
78.0%
67.0%
72.0%
83.0%
CMIP6

Supplementary Figure 445: Return times (RT) during present-to-near-future (2015-2054) based on ERA5 (Tx99.0p) - Return times (RT) (in years) during the present-to-near-future period between 2015 and 2054 for Median Heatwave Severity of the respective observed event. For each region, values are shown for the different scenarios (increasing in emissions from left to right for the CMIP5 (green), CMIP6 (red), CanESM5 (dark blue), and ACCESS-ESM1-5 (light blue) ensembles. The error bars indicate the 5th and 95th percentile of the respective sample. Where the respective ensemble contains models not able to simulate the magnitude of the respective observed event's Median Heatwave Severity, the proportion of models that are able to simulate an event with a value of Median Heatwave Severity at least as high as the observed event is indicated by the percentage values above the individual bars. The observed reference values are calculated using data from ERA5.

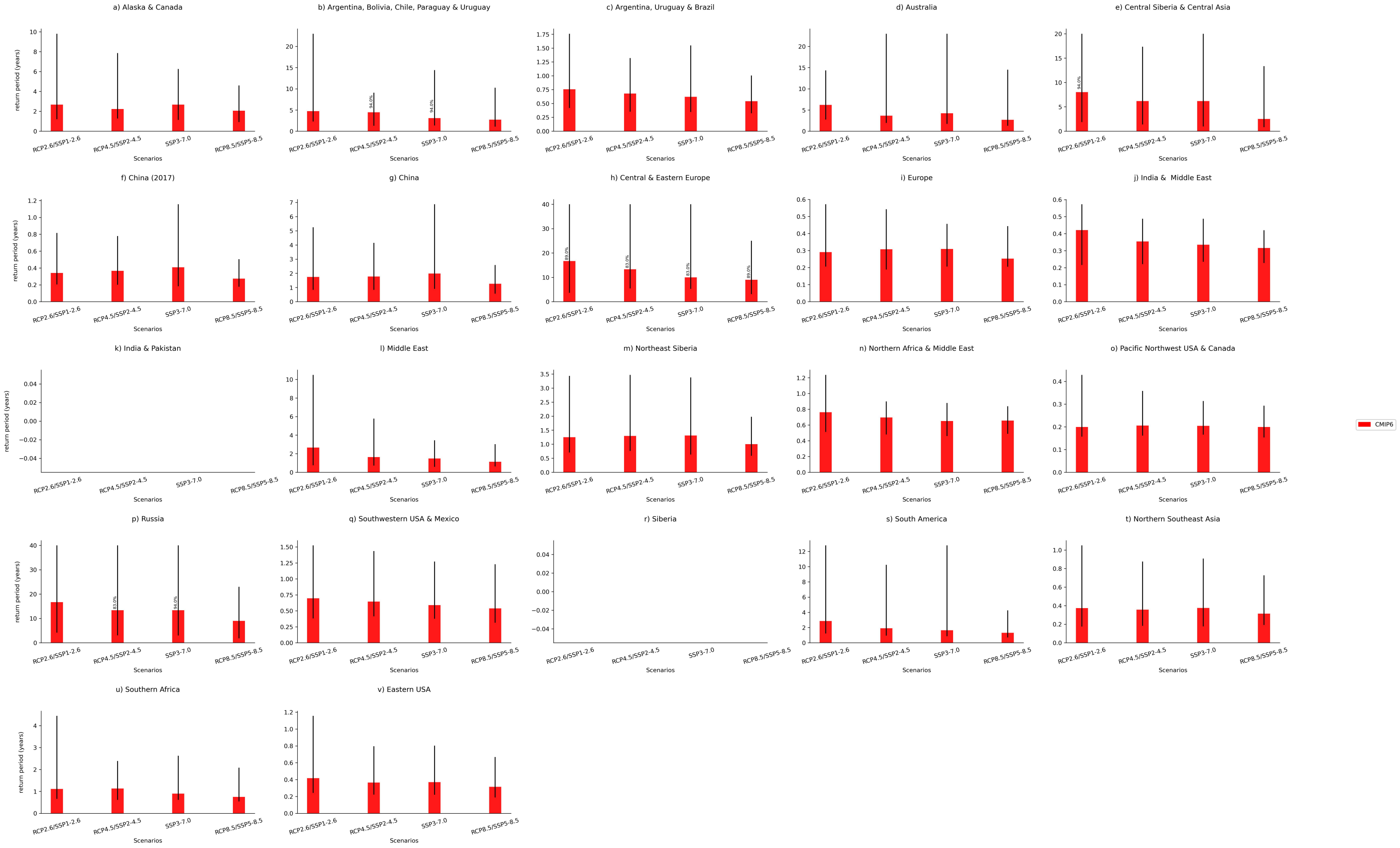

a) Alaska & Canada
b) Argentina, Bolivia, Chile, Paraguay & Uruguay
94.0%
94.0%
c) Argentina, Uruguay & Brazil
d) Australia
e) Central Siberia & Central Asia
94.0%
f) China (2017)
g) China
h) Central & Eastern Europe
89.0%
83.0%
83.0%
89.0%
i) Europe
j) India & Middle East
k) India & Pakistan
l) Middle East
m) Northeast Siberia
n) Northern Africa & Middle East
o) Pacific Northwest USA & Canada
p) Russia
83.0%
94.0%
q) Southwestern USA & Mexico
r) Siberia
s) South America
t) Northern Southeast Asia
u) Southern Africa
v) Eastern USA
return period (years)
Scenarios
RCP2.6/SSP1-2.6
RCP4.5/SSP2-4.5
SSP3-7.0
RCP8.5/SSP5-8.5
CMIP6

Supplementary Figure 446: Return times (RT) during end-of-century (2060-2099) based on ERA5 (Tx95.0p) - Return times (RT) (in years) during the end-of-century period between 2060 and 2099 for Median Heatwave Severity of the respective observed event. For each region, values are shown for the different scenarios (increasing in emissions from left to right for the CMIP5 (green), CMIP6 (red), CanESM5 (dark blue), and ACCESS-ESM1-5 (light blue) ensembles. The error bars indicate the 5th and 95th percentile of the respective sample. Where the respective ensemble contains models not able to simulate the magnitude of the respective observed event's Median Heatwave Severity, the proportion of models that are able to simulate an event with a value of Median Heatwave Severity at least as high as the observed event is indicated by the percentage values above the individual bars. The observed reference values are calculated using data from ERA5.

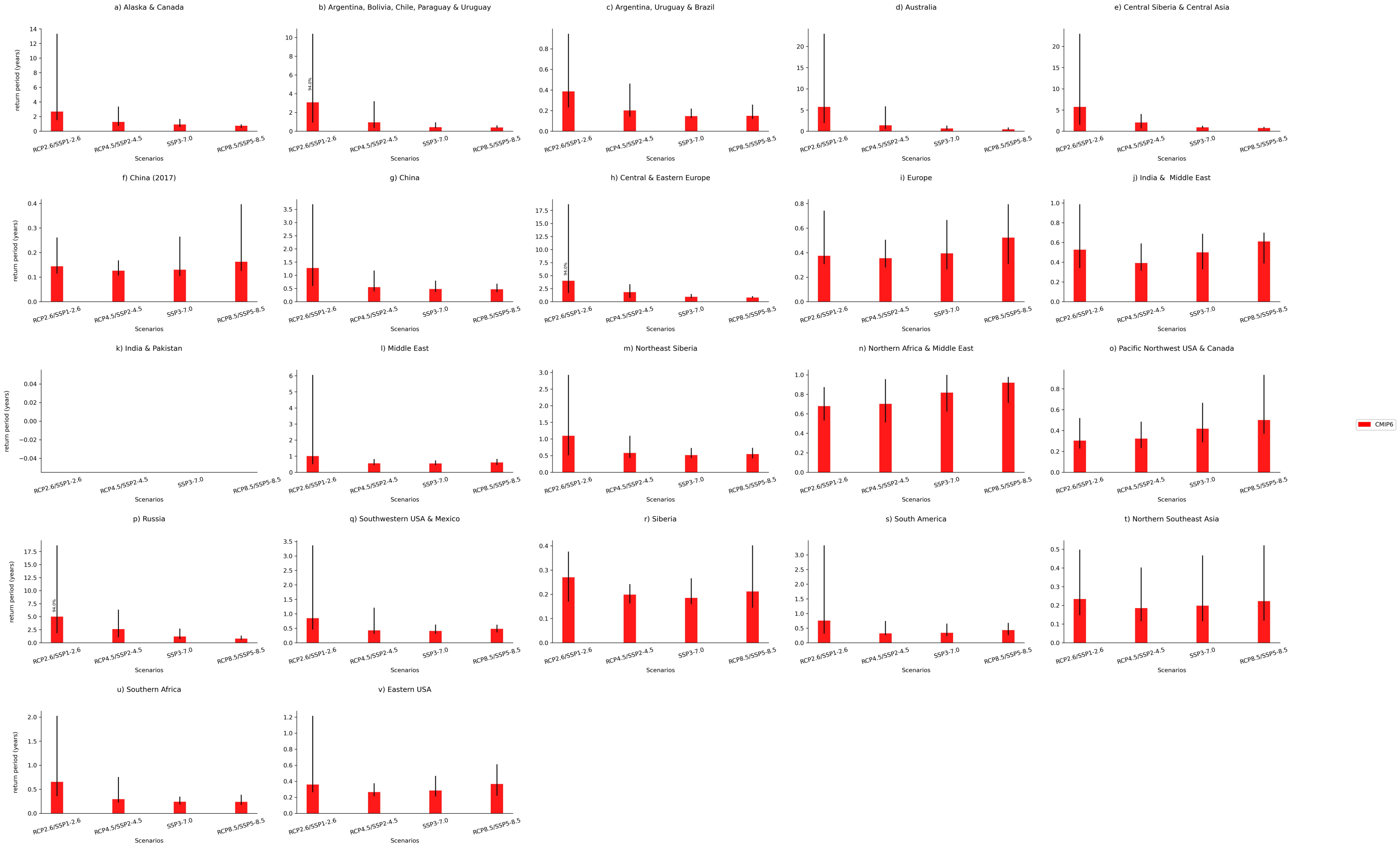

a) Alaska & Canada
b) Argentina, Bolivia, Chile, Paraguay & Uruguay
c) Argentina, Uruguay & Brazil
d) Australia
e) Central Siberia & Central Asia
f) China (2017)
g) China
h) Central & Eastern Europe
i) Europe
j) India & Middle East
k) India & Pakistan
l) Middle East
m) Northeast Siberia
n) Northern Africa & Middle East
o) Pacific Northwest USA & Canada
p) Russia
q) Southwestern USA & Mexico
r) Siberia
s) South America
t) Northern Southeast Asia
u) Southern Africa
v) Eastern USA
return period (years)
Scenarios
RCP2.6/SSP1-2.6
RCP4.5/SSP2-4.5
SSP3-7.0
RCP8.5/SSP5-8.5
94.0%
CMIP6

Supplementary Figure 447: Return times (RT) during end-of-century (2060-2099) based on ERA5 (Tx97.5p) - Return times (RT) (in years) during the end-of-century period between 2060 and 2099 for Median Heatwave Severity of the respective observed event. For each region, values are shown for the different scenarios (increasing in emissions from left to right for the CMIP5 (green), CMIP6 (red), CanESM5 (dark blue), and ACCESS-ESM1-5 (light blue) ensembles. The error bars indicate the 5th and 95th percentile of the respective sample. Where the respective ensemble contains models not able to simulate the magnitude of the respective observed event's Median Heatwave Severity, the proportion of models that are able to simulate an event with a value of Median Heatwave Severity at least as high as the observed event is indicated by the percentage values above the individual bars. The observed reference values are calculated using data from ERA5.

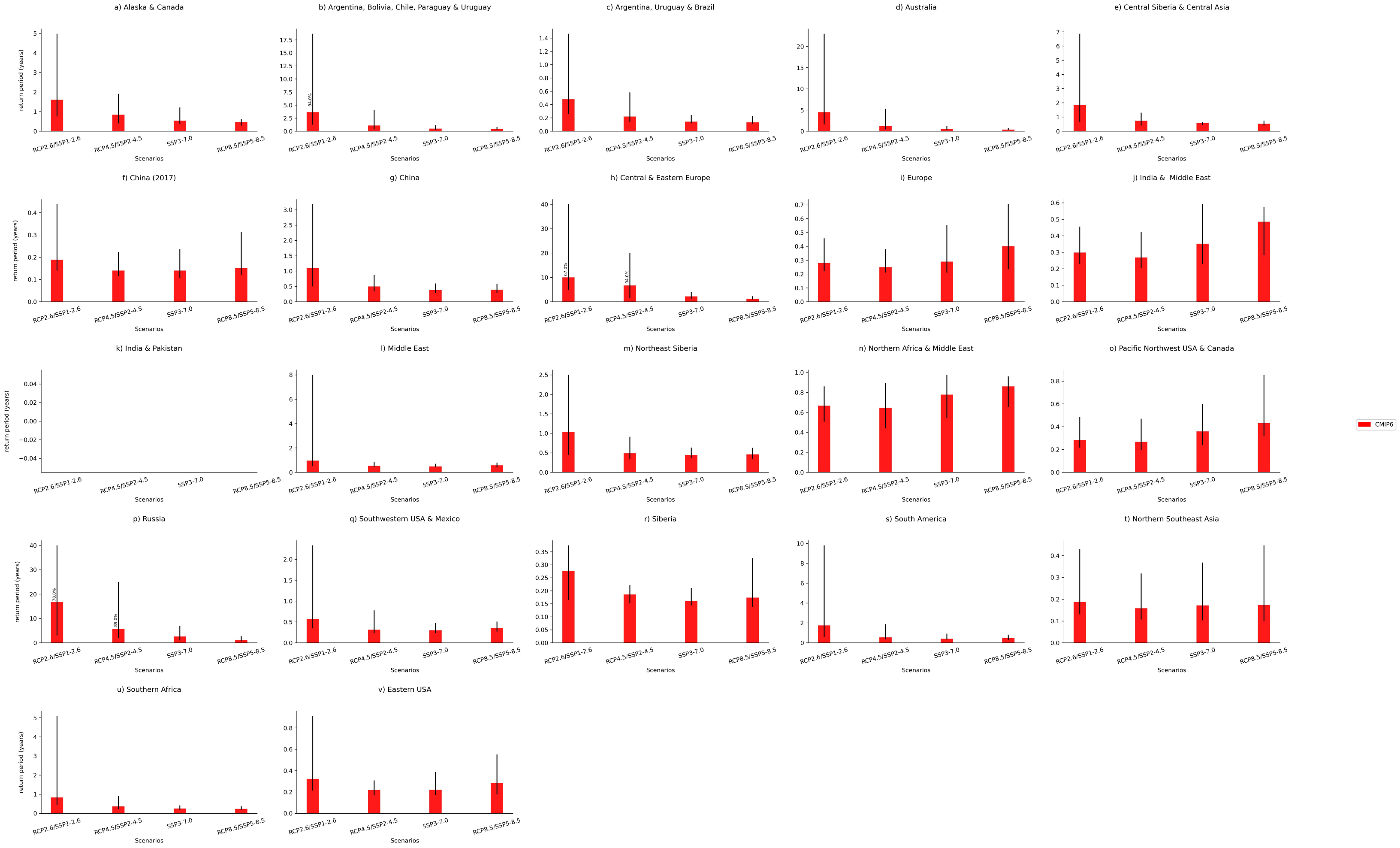

a) Alaska & Canada
b) Argentina, Bolivia, Chile, Paraguay & Uruguay
c) Argentina, Uruguay & Brazil
d) Australia
e) Central Siberia & Central Asia
f) China (2017)
g) China
h) Central & Eastern Europe
i) Europe
j) India & Middle East
k) India & Pakistan
l) Middle East
m) Northeast Siberia
n) Northern Africa & Middle East
o) Pacific Northwest USA & Canada
p) Russia
q) Southwestern USA & Mexico
r) Siberia
s) South America
t) Northern Southeast Asia
u) Southern Africa
v) Eastern USA
return period (years)
Scenarios
RCP2.6/SSP1-2.6
RCP4.5/SSP2-4.5
SSP3-7.0
RCP8.5/SSP5-8.5
94.0%
67.0%
94.0%
78.0%
89.0%
CMIP6

Supplementary Figure 448: Return times (RT) during end-of-century (2060-2099) based on ERA5 (Tx99.0p) - Return times (RT) (in years) during the end-of-century period between 2060 and 2099 for Median Heatwave Severity of the respective observed event. For each region, values are shown for the different scenarios (increasing in emissions from left to right for the CMIP5 (green), CMIP6 (red), CanESM5 (dark blue), and ACCESS-ESM1-5 (light blue) ensembles. The error bars indicate the 5th and 95th percentile of the respective sample. Where the respective ensemble contains models not able to simulate the magnitude of the respective observed event's Median Heatwave Severity, the proportion of models that are able to simulate an event with a value of Median Heatwave Severity at least as high as the observed event is indicated by the percentage values above the individual bars. The observed reference values are calculated using data from ERA5.

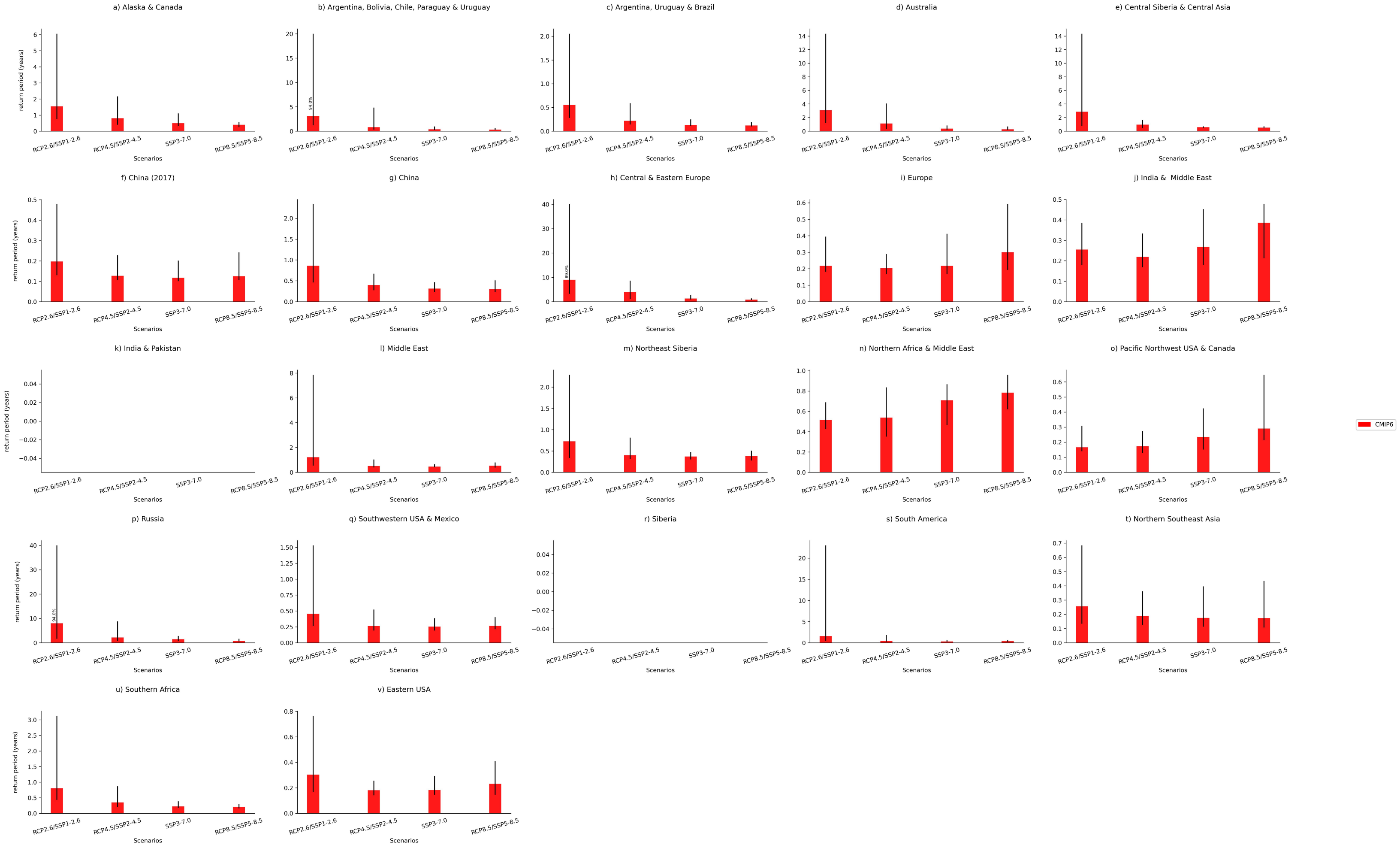

a) Alaska & Canada
b) Argentina, Bolivia, Chile, Paraguay & Uruguay
c) Argentina, Uruguay & Brazil
d) Australia
e) Central Siberia & Central Asia
f) China (2017)
g) China
h) Central & Eastern Europe
i) Europe
j) India & Middle East
k) India & Pakistan
l) Middle East
m) Northeast Siberia
n) Northern Africa & Middle East
o) Pacific Northwest USA & Canada
p) Russia
q) Southwestern USA & Mexico
r) Siberia
s) South America
t) Northern Southeast Asia
u) Southern Africa
v) Eastern USA
return period (years)
Scenarios
RCP2.6/SSP1-2.6
RCP4.5/SSP2-4.5
SSP3-7.0
RCP8.5/SSP5-8.5
CMIP6

Supplementary Figure 449: Reference Event Mapping for Alaska & Canada based on Berkeley Earth - Spatio-temporal evolution of local heat-affected grid boxes during reference heatwave in Alaska & Canada based on data in Berkeley Earth. Colors and color-bar indicate the local severity values for each day of the event. The respective date of the heatwave day is indicated above each sub-figure and x- and y-axis show the longitude and latitude coordinates.

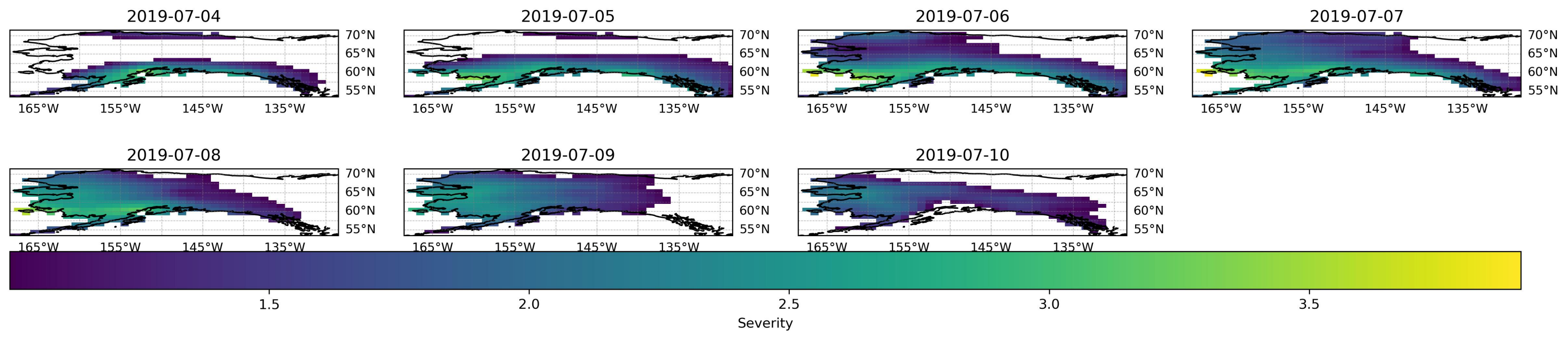
2019-07-04
2019-07-05
2019-07-06
2019-07-07
2019-07-08
2019-07-09
2019-07-10
70°N
65°N
60°N
55°N
165°W
155°W
145°W
135°W
1.5
2.0
2.5
3.0
3.5
Severity

Supplementary Figure 450: Reference Event Mapping for Alaska & Canada based on ERA5 - Spatio-temporal evolution of local heat-affected grid boxes during reference heatwave in Alaska & Canada based on data in ERA5. Colors and color-bar indicate the local severity values for each day of the event. The respective date of the heatwave day is indicated above each sub-figure and x- and y-axis show the longitude and latitude coordinates.

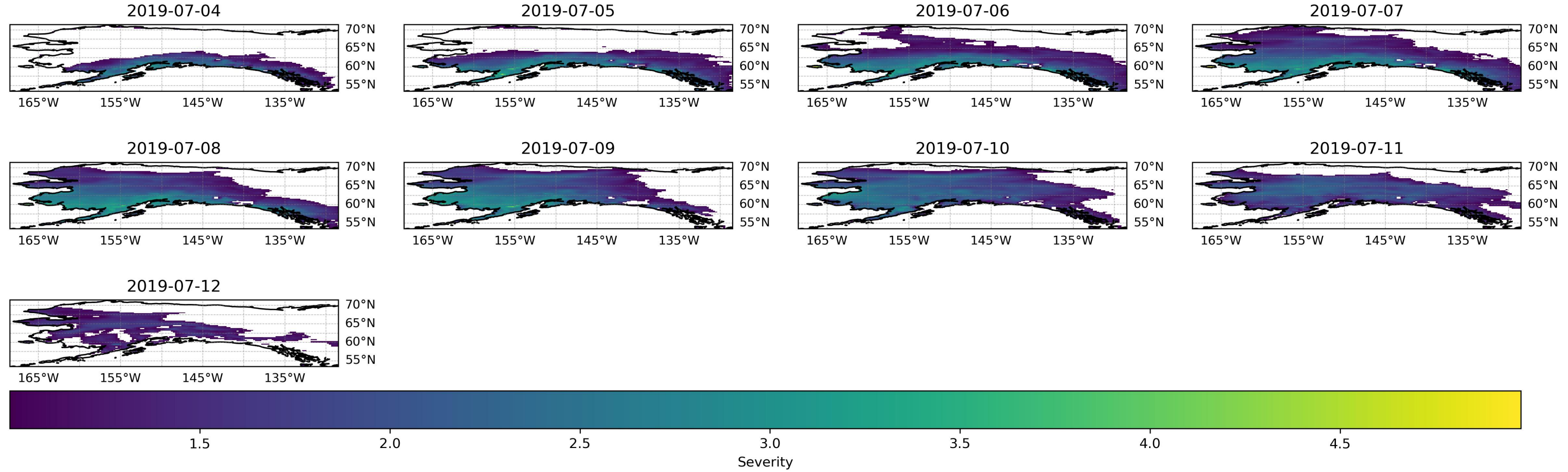

2019-07-04
2019-07-05
2019-07-06
2019-07-07
2019-07-08
2019-07-09
2019-07-10
2019-07-11
2019-07-12
70°N
65°N
60°N
55°N
165°W
155°W
145°W
135°W
1.5
2.0
2.5
3.0
3.5
4.0
4.5
Severity

Supplementary Figure 451: Reference Event Mapping for Argentina, Bolivia, Chile, Paraguay & Uruguay based on Berkeley Earth - Spatio-temporal evolution of local heat-affected grid boxes during reference heatwave in Argentina, Bolivia, Chile, Paraguay & Uruguay based on data in Berkeley Earth. Colors and color-bar indicate the local severity values for each day of the event. The respective date of the heatwave day is indicated above each sub-figure and x- and y-axis show the longitude and latitude coordinates.

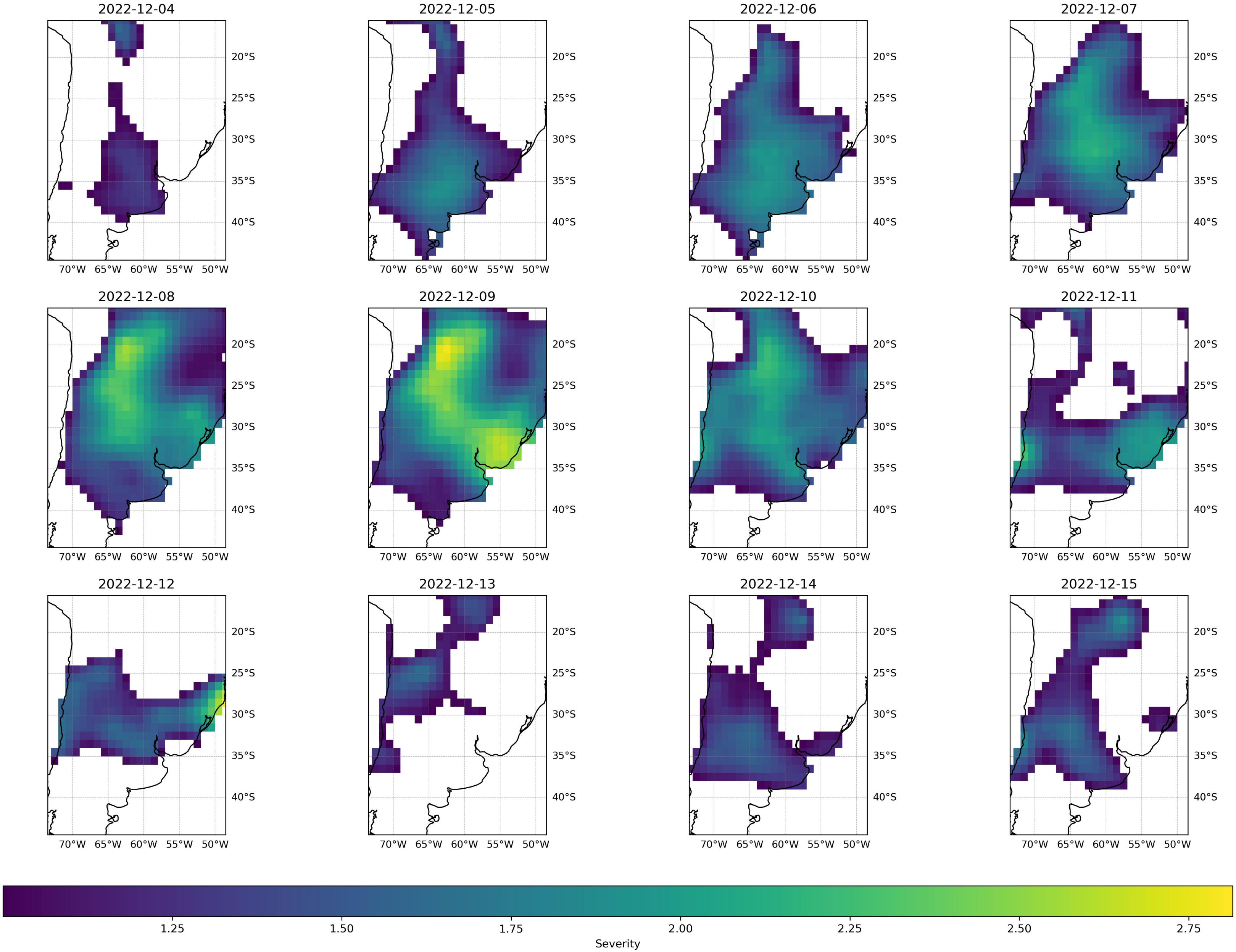
2022-12-04
2022-12-05
2022-12-06
2022-12-07
2022-12-08
2022-12-09
2022-12-10
2022-12-11
2022-12-12
2022-12-13
2022-12-14
2022-12-15
20°S
25°S
30°S
35°S
40°S
70°W
65°W
60°W
55°W
50°W
1.25
1.50
1.75
2.00
2.25
2.50
2.75
Severity

Supplementary Figure 452: Reference Event Mapping for Argentina, Bolivia, Chile, Paraguay & Uruguay based on ERA5 - Spatio-temporal evolution of local heat-affected grid boxes during reference heatwave in Argentina, Bolivia, Chile, Paraguay & Uruguay based on data in ERA5. Colors and color-bar indicate the local severity values for each day of the event. The respective date of the heatwave day is indicated above each sub-figure and x- and y-axis show the longitude and latitude coordinates.

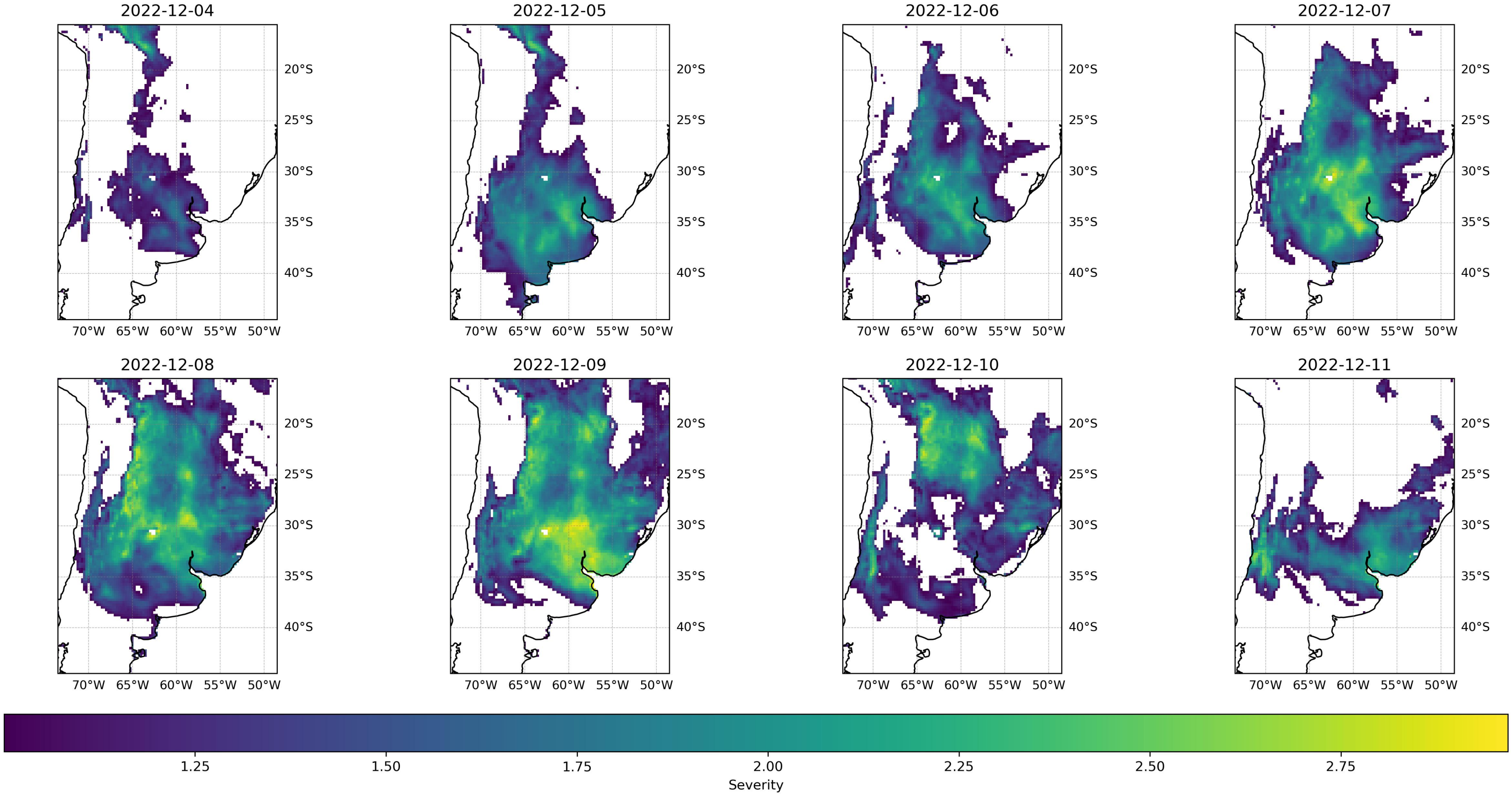
2022-12-04
2022-12-05
2022-12-06
2022-12-07
2022-12-08
2022-12-09
2022-12-10
2022-12-11
20°S
25°S
30°S
35°S
40°S
70°W
65°W
60°W
55°W
50°W
1.25
1.50
1.75
2.00
2.25
2.50
2.75
Severity

Supplementary Figure 453: Reference Event Mapping for Argentina, Uruguay & Brazil based on Berkeley Earth - Spatio-temporal evolution of local heat-affected grid boxes during reference heatwave in Argentina, Uruguay & Brazil based on data in Berkeley Earth. Colors and color-bar indicate the local severity values for each day of the event. The respective date of the heatwave day is indicated above each sub-figure and x- and y-axis show the longitude and latitude coordinates.

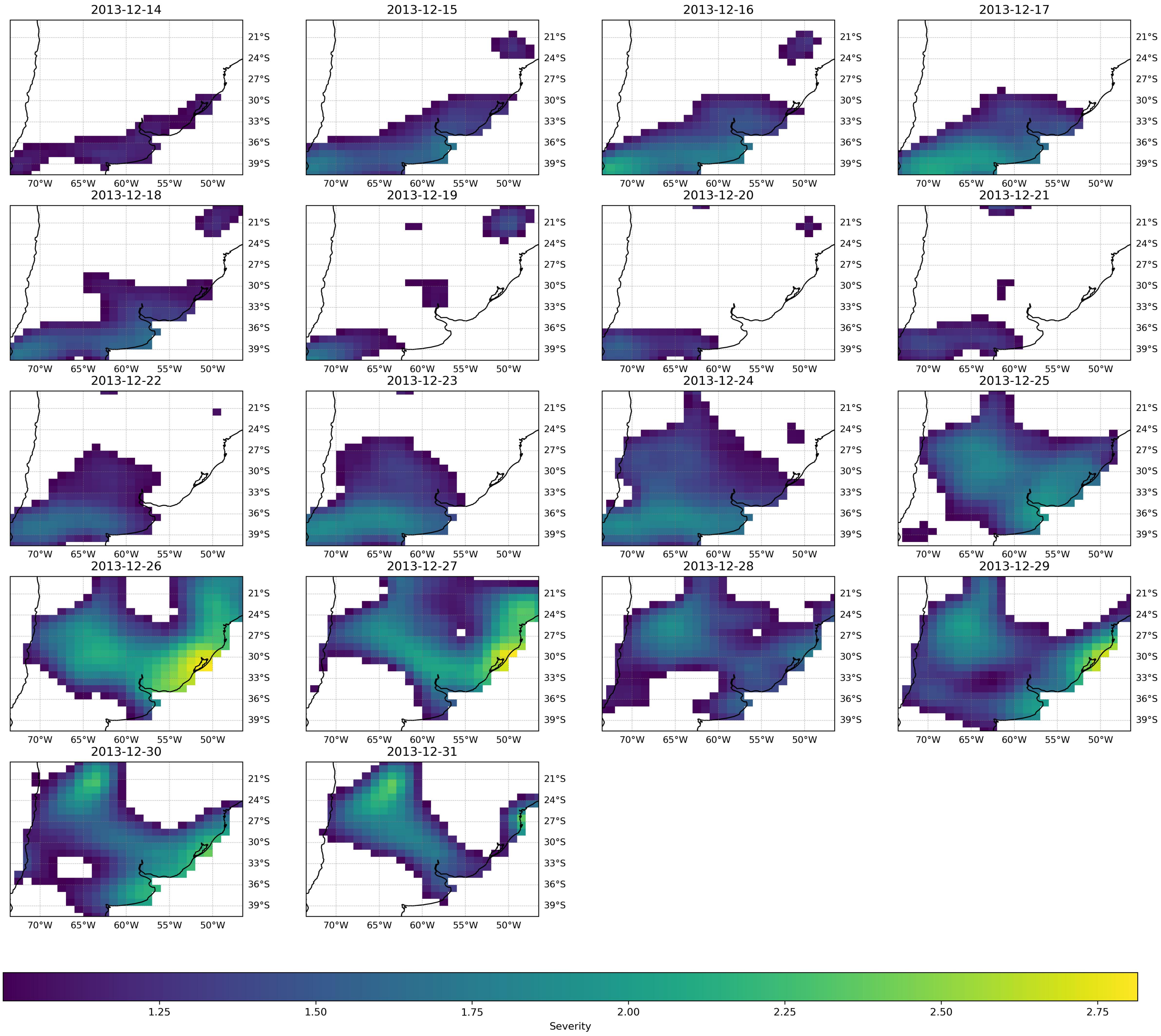

2013-12-14
2013-12-15
2013-12-16
2013-12-17
2013-12-18
2013-12-19
2013-12-20
2013-12-21
2013-12-22
2013-12-23
2013-12-24
2013-12-25
2013-12-26
2013-12-27
2013-12-28
2013-12-29
2013-12-30
2013-12-31
21°S
24°S
27°S
30°S
33°S
36°S
39°S
70°W
65°W
60°W
55°W
50°W
1.25
1.50
1.75
2.00
2.25
2.50
2.75
Severity

Supplementary Figure 454: Reference Event Mapping for Argentina, Uruguay & Brazil based on ERA5 - Spatio-temporal evolution of local heat-affected grid boxes during reference heatwave in Argentina, Uruguay & Brazil based on data in ERA5. Colors and color-bar indicate the local severity values for each day of the event. The respective date of the heatwave day is indicated above each sub-figure and x- and y-axis show the longitude and latitude coordinates.

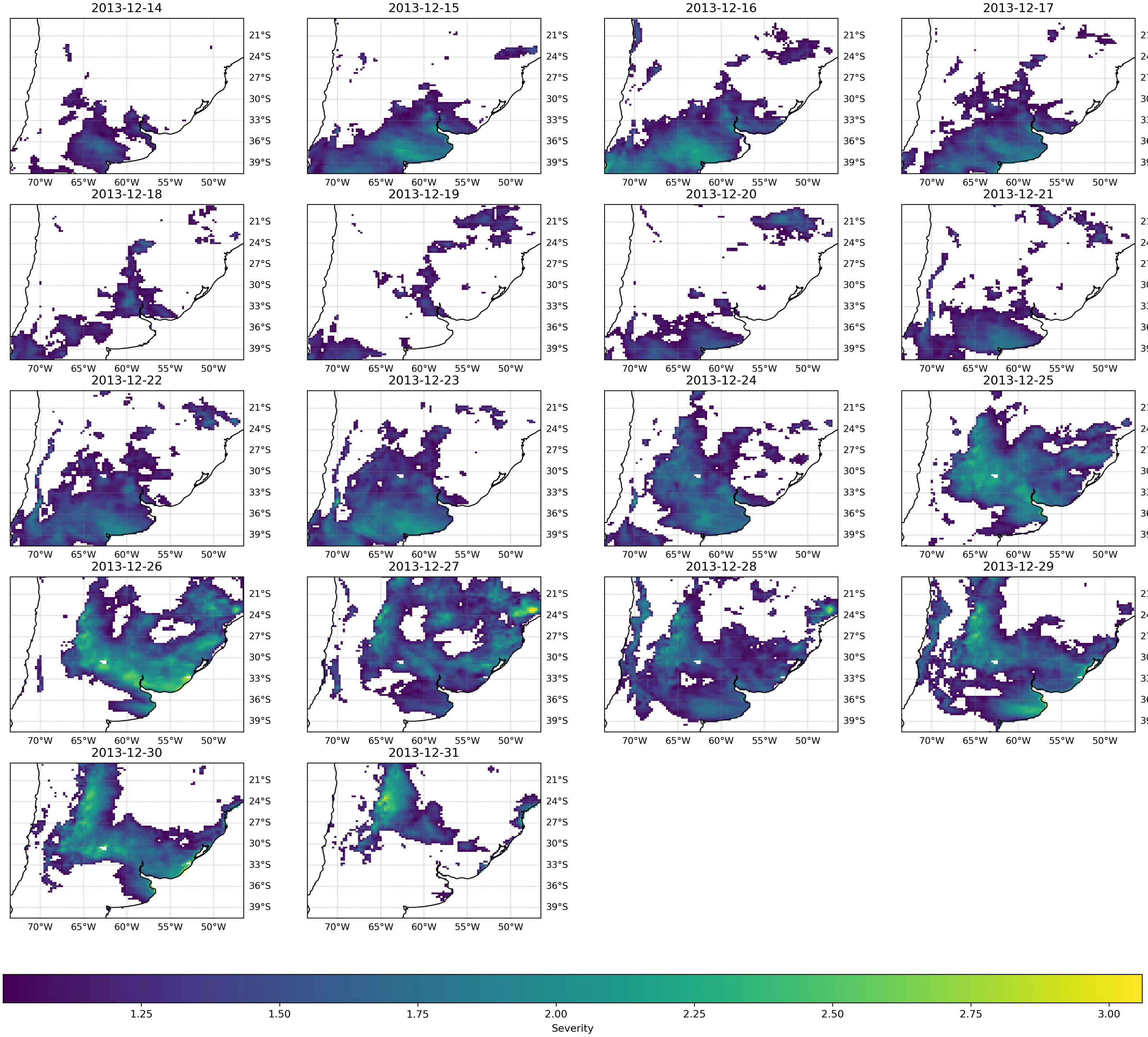

2013-12-14
2013-12-15
2013-12-16
2013-12-17
2013-12-18
2013-12-19
2013-12-20
2013-12-21
2013-12-22
2013-12-23
2013-12-24
2013-12-25
2013-12-26
2013-12-27
2013-12-28
2013-12-29
2013-12-30
2013-12-31
21°S
24°S
27°S
30°S
33°S
36°S
39°S
70°W
65°W
60°W
55°W
50°W
1.25
1.50
1.75
2.00
2.25
2.50
2.75
3.00
Severity

Supplementary Figure 455: Reference Event Mapping for Australia based on Berkeley Earth - Spatio-temporal evolution of local heat-affected grid boxes during reference heatwave in Australia based on data in Berkeley Earth. Colors and color-bar indicate the local severity values for each day of the event. The respective date of the heatwave day is indicated above each sub-figure and x- and y-axis show the longitude and latitude coordinates.

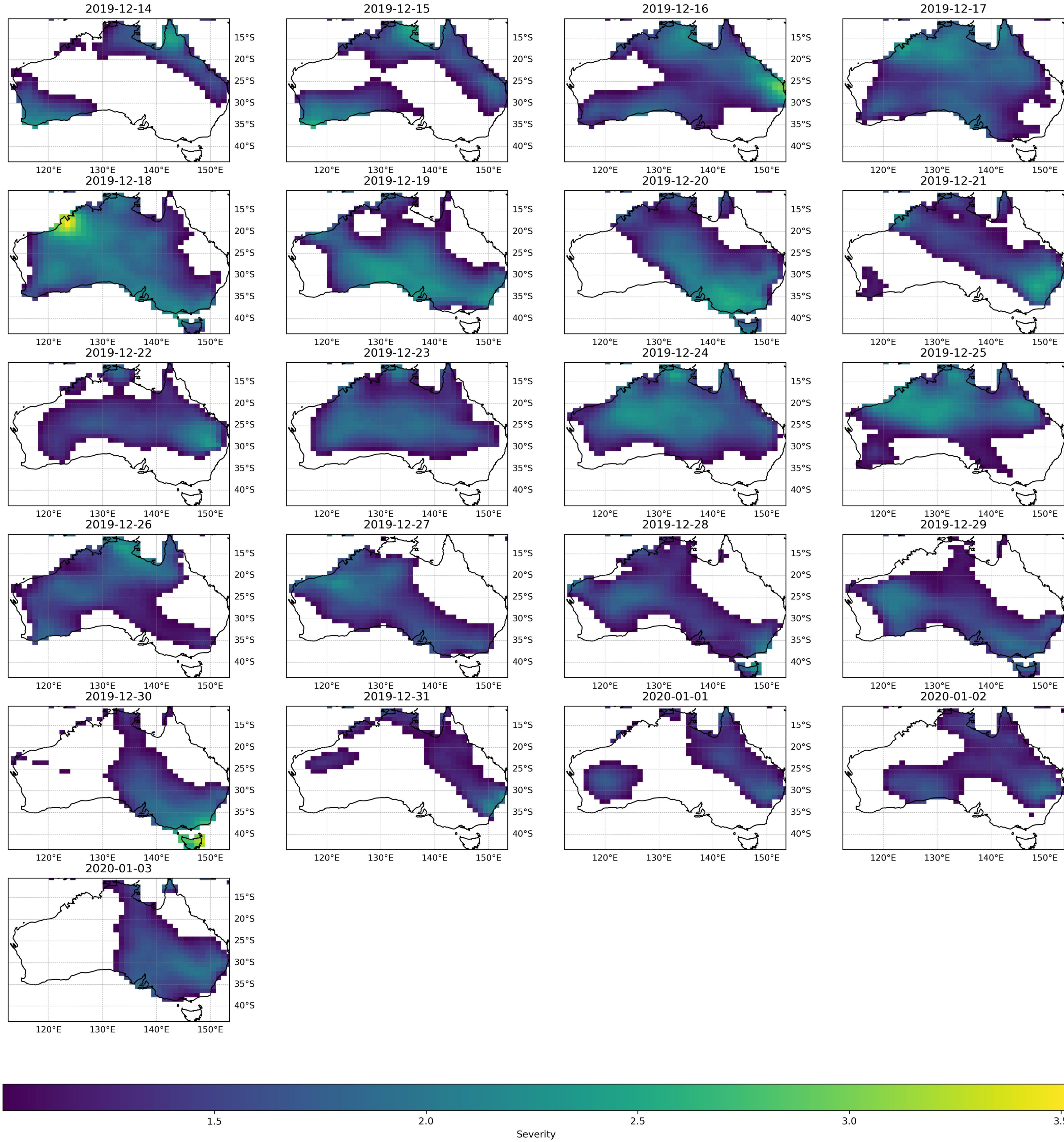

2019-12-14
2019-12-15
2019-12-16
2019-12-17
2019-12-18
2019-12-19
2019-12-20
2019-12-21
2019-12-22
2019-12-23
2019-12-24
2019-12-25
2019-12-26
2019-12-27
2019-12-28
2019-12-29
2019-12-30
2019-12-31
2020-01-01
2020-01-02
2020-01-03
15°S
20°S
25°S
30°S
35°S
40°S
120°E
130°E
140°E
150°E
1.5
2.0
2.5
3.0
3.5
Severity

Supplementary Figure 456: Reference Event Mapping for Australia based on ERA5 - Spatio-temporal evolution of local heat-affected grid boxes during reference heatwave in Australia based on data in ERA5. Colors and color-bar indicate the local severity values for each day of the event. The respective date of the heatwave day is indicated above each sub-figure and x- and y-axis show the longitude and latitude coordinates.

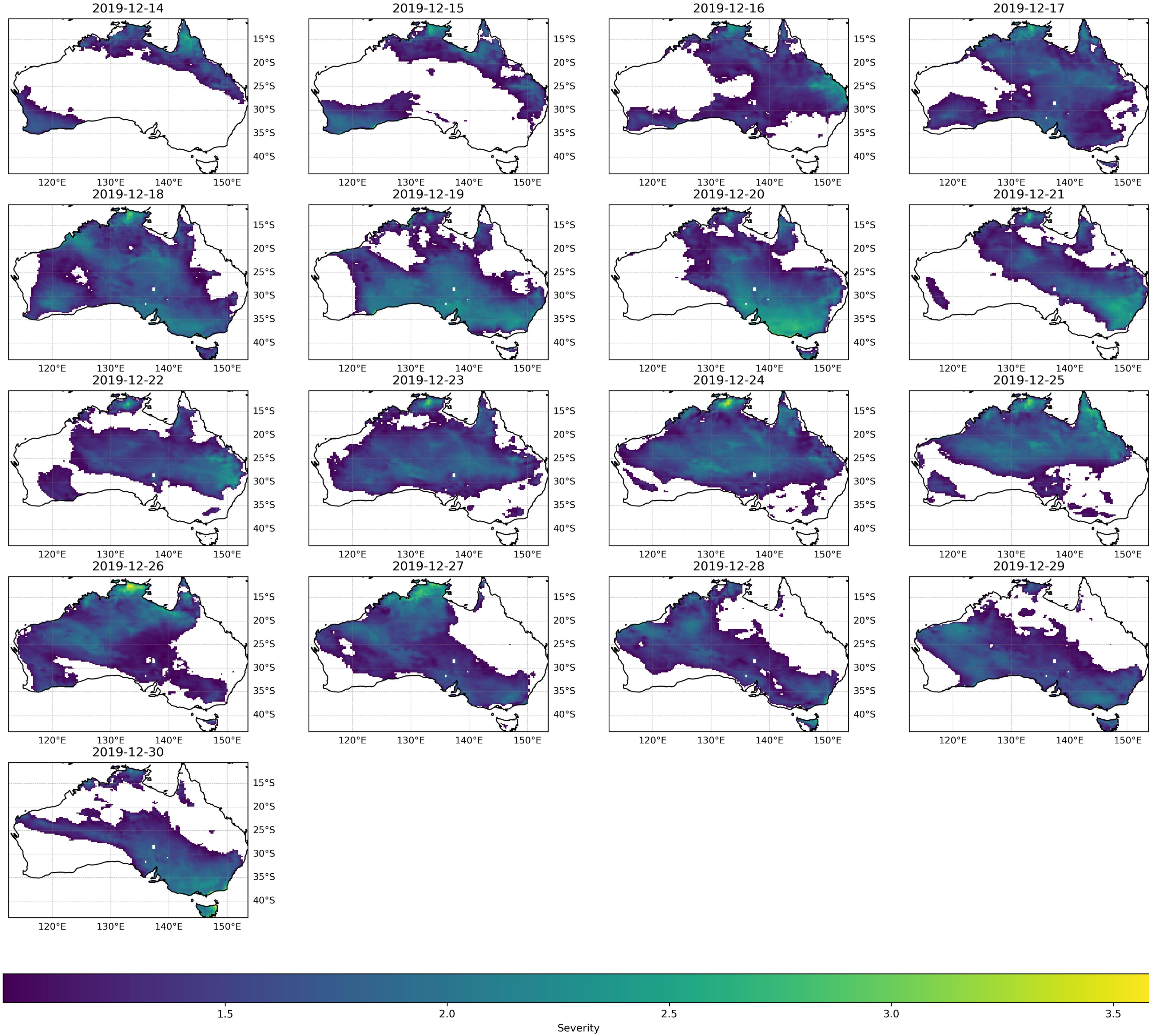

2019-12-14
2019-12-15
2019-12-16
2019-12-17
2019-12-18
2019-12-19
2019-12-20
2019-12-21
2019-12-22
2019-12-23
2019-12-24
2019-12-25
2019-12-26
2019-12-27
2019-12-28
2019-12-29
2019-12-30
15°S
20°S
25°S
30°S
35°S
40°S
120°E
130°E
140°E
150°E
1.5
2.0
2.5
3.0
3.5
Severity

Supplementary Figure 457: Reference Event Mapping for Central Siberia & Central Asia based on Berkeley Earth - Spatio-temporal evolution of local heat-affected grid boxes during reference heatwave in Central Siberia & Central Asia based on data in Berkeley Earth. Colors and color-bar indicate the local severity values for each day of the event. The respective date of the heatwave day is indicated above each sub-figure and x- and y-axis show the longitude and latitude coordinates.

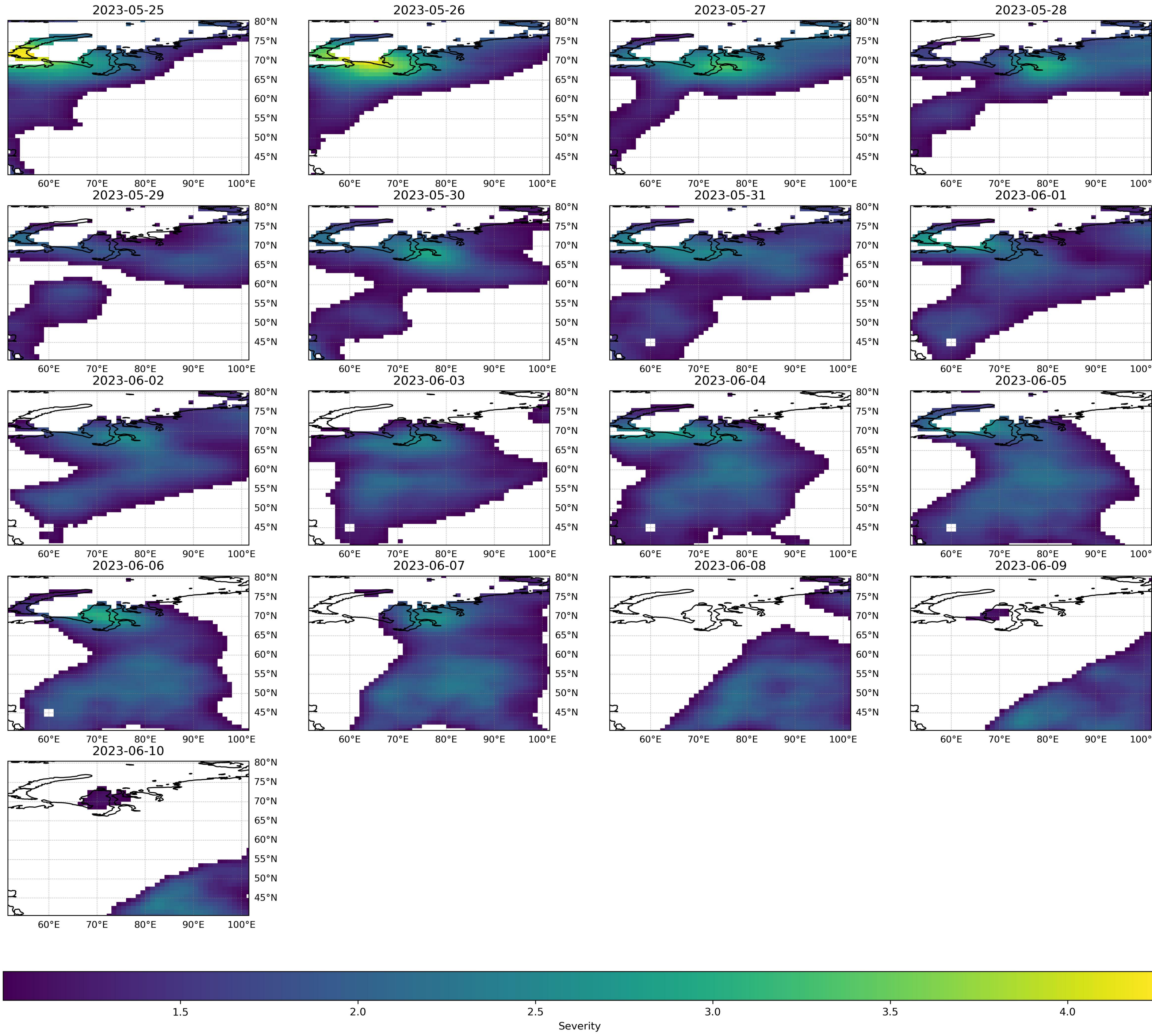
2023-05-25
2023-05-26
2023-05-27
2023-05-28
2023-05-29
2023-05-30
2023-05-31
2023-06-01
2023-06-02
2023-06-03
2023-06-04
2023-06-05
2023-06-06
2023-06-07
2023-06-08
2023-06-09
2023-06-10
80°N
75°N
70°N
65°N
60°N
55°N
50°N
45°N
60°E
70°E
80°E
90°E
100°E
1.5
2.0
2.5
3.0
3.5
4.0
Severity

Supplementary Figure 458: Reference Event Mapping for Central Siberia & Central Asia based on ERA5 - Spatio-temporal evolution of local heat-affected grid boxes during reference heatwave in Central Siberia & Central Asia based on data in ERA5. Colors and color-bar indicate the local severity values for each day of the event. The respective date of the heatwave day is indicated above each sub-figure and x- and y-axis show the longitude and latitude coordinates.

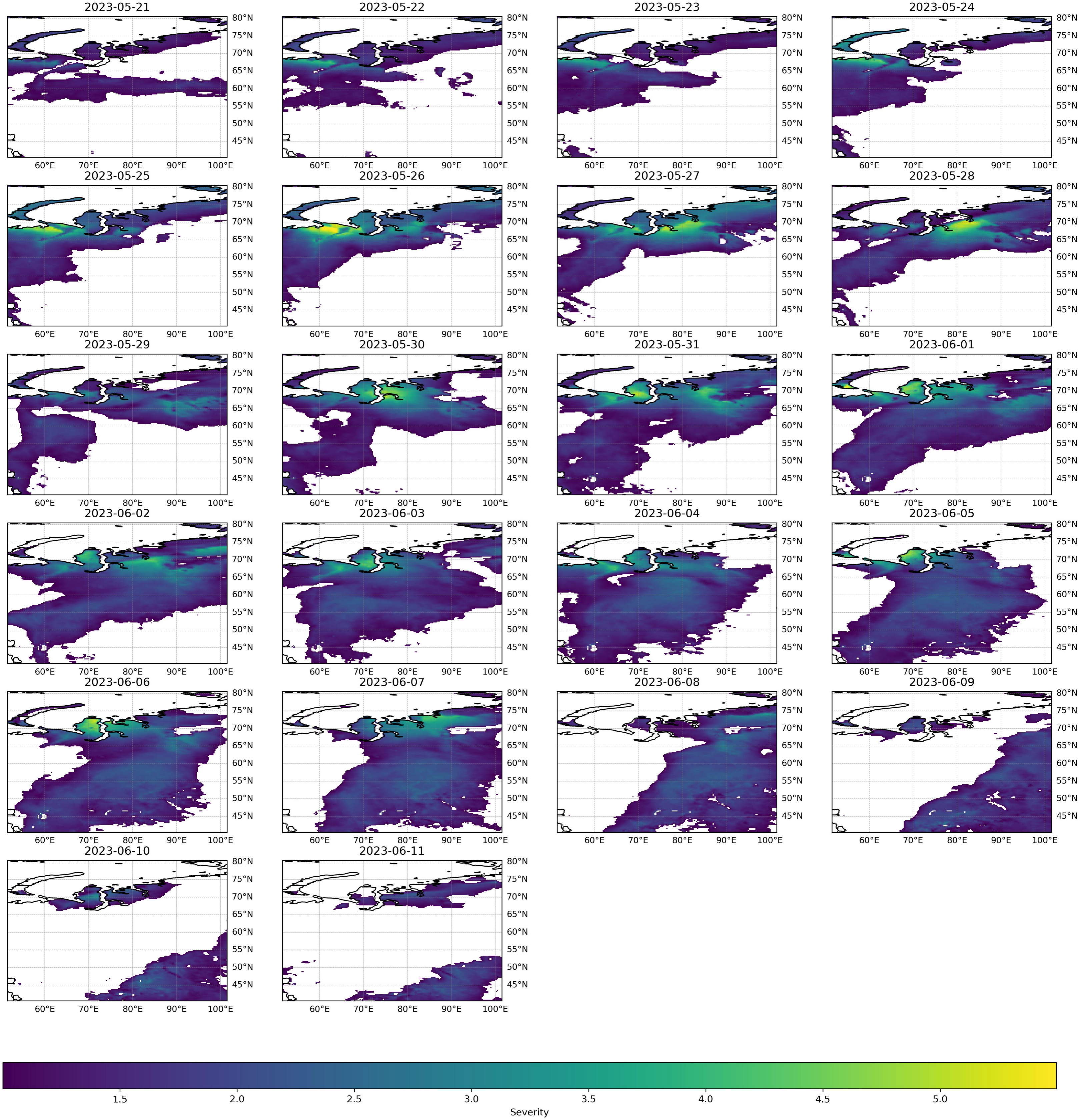

2023-05-21
2023-05-22
2023-05-23
2023-05-24
2023-05-25
2023-05-26
2023-05-27
2023-05-28
2023-05-29
2023-05-30
2023-05-31
2023-06-01
2023-06-02
2023-06-03
2023-06-04
2023-06-05
2023-06-06
2023-06-07
2023-06-08
2023-06-09
2023-06-10
2023-06-11
80°N
75°N
70°N
65°N
60°N
55°N
50°N
45°N
60°E
70°E
80°E
90°E
100°E
1.5
2.0
2.5
3.0
3.5
4.0
4.5
5.0
Severity

Supplementary Figure 459: Reference Event Mapping for China (2017) based on Berkeley Earth - Spatio-temporal evolution of local heat-affected grid boxes during reference heatwave in China (2017) based on data in Berkeley Earth. Colors and color-bar indicate the local severity values for each day of the event. The respective date of the heatwave day is indicated above each sub-figure and x- and y-axis show the longitude and latitude coordinates.

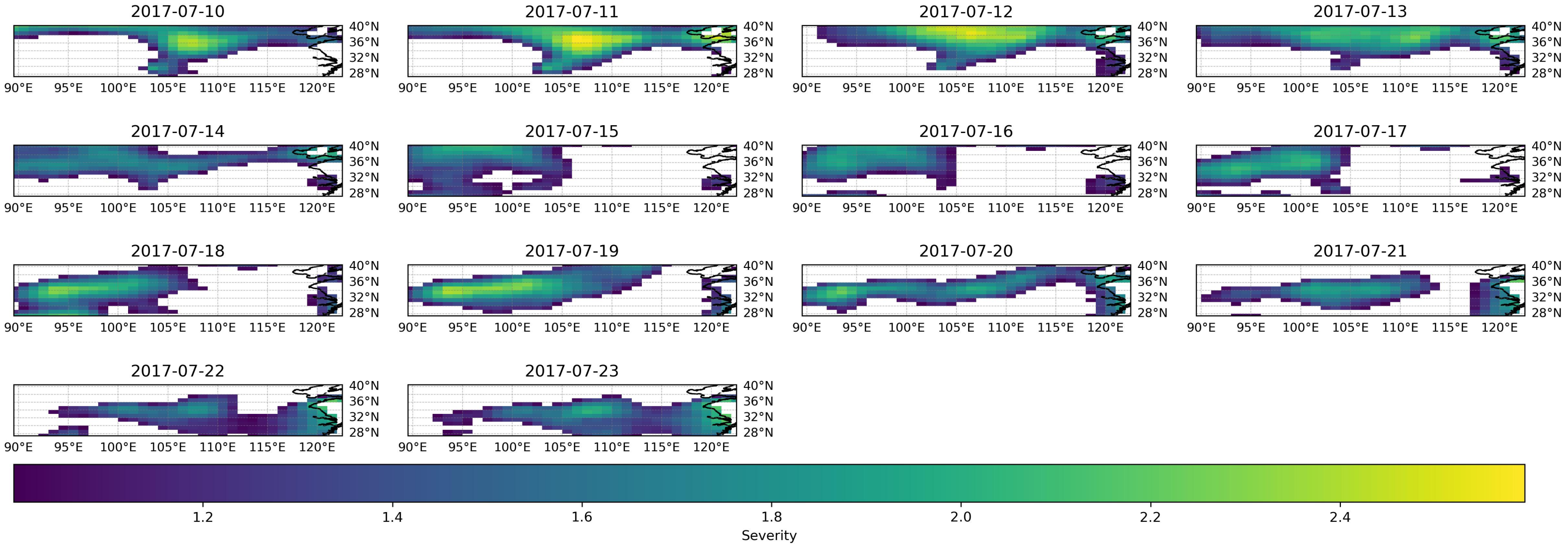
2017-07-10
2017-07-11
2017-07-12
2017-07-13
2017-07-14
2017-07-15
2017-07-16
2017-07-17
2017-07-18
2017-07-19
2017-07-20
2017-07-21
2017-07-22
2017-07-23
40°N
36°N
32°N
28°N
90°E
95°E
100°E
105°E
110°E
115°E
120°E
1.2
1.4
1.6
1.8
2.0
2.2
2.4
Severity

Supplementary Figure 460: Reference Event Mapping for China (2017) based on ERA5 - Spatio-temporal evolution of local heat-affected grid boxes during reference heatwave in China (2017) based on data in ERA5. Colors and color-bar indicate the local severity values for each day of the event. The respective date of the heatwave day is indicated above each sub-figure and x- and y-axis show the longitude and latitude coordinates.

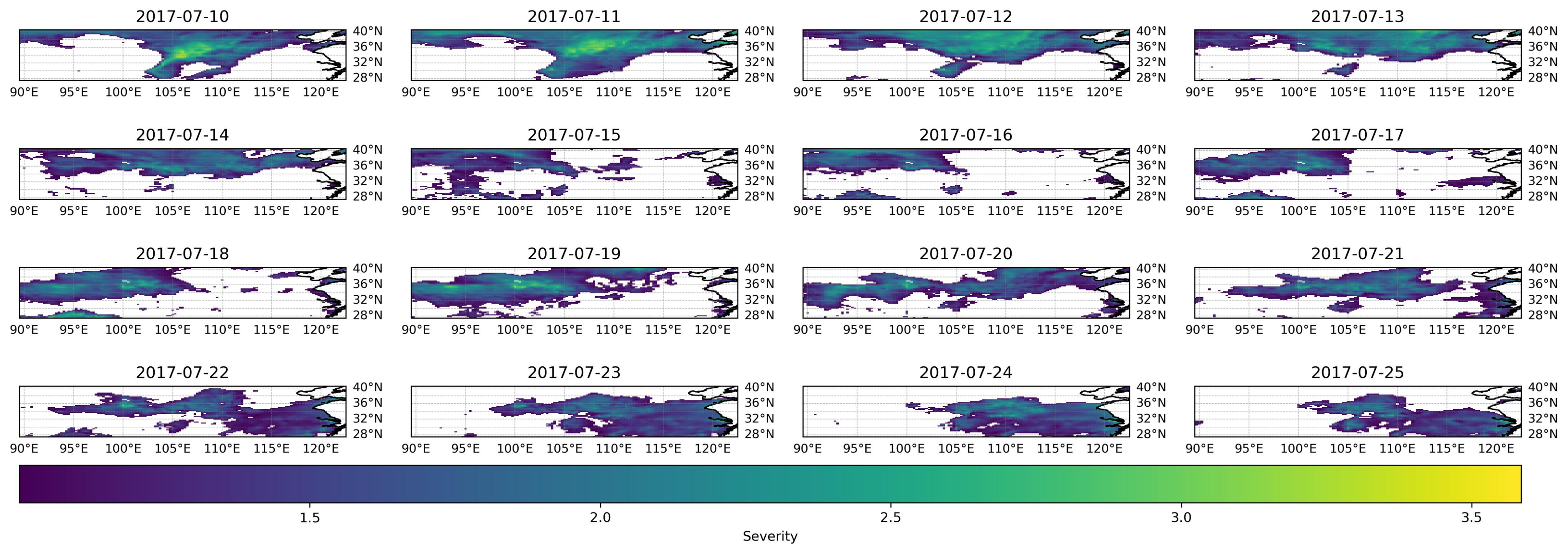

2017-07-10
2017-07-11
2017-07-12
2017-07-13
2017-07-14
2017-07-15
2017-07-16
2017-07-17
2017-07-18
2017-07-19
2017-07-20
2017-07-21
2017-07-22
2017-07-23
2017-07-24
2017-07-25
40°N
36°N
32°N
28°N
90°E
95°E
100°E
105°E
110°E
115°E
120°E
1.5
2.0
2.5
3.0
3.5
Severity

Supplementary Figure 461: Reference Event Mapping for China based on Berkeley Earth - Spatio-temporal evolution of local heat-affected grid boxes during reference heatwave in China based on data in Berkeley Earth. Colors and color-bar indicate the local severity values for each day of the event. The respective date of the heatwave day is indicated above each sub-figure and x- and y-axis show the longitude and latitude coordinates.

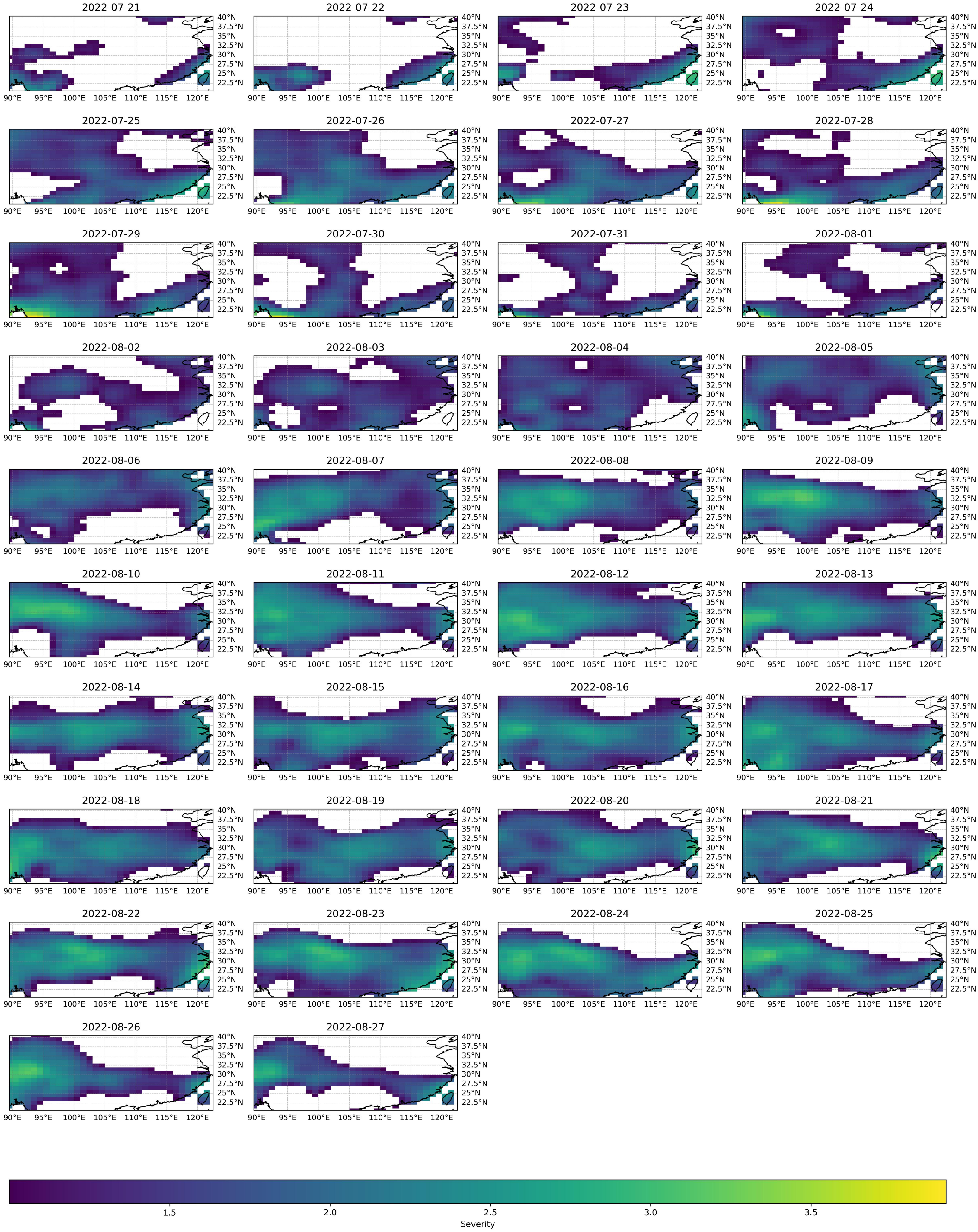
2022-07-21
2022-07-22
2022-07-23
2022-07-24
2022-07-25
2022-07-26
2022-07-27
2022-07-28
2022-07-29
2022-07-30
2022-07-31
2022-08-01
2022-08-02
2022-08-03
2022-08-04
2022-08-05
2022-08-06
2022-08-07
2022-08-08
2022-08-09
2022-08-10
2022-08-11
2022-08-12
2022-08-13
2022-08-14
2022-08-15
2022-08-16
2022-08-17
2022-08-18
2022-08-19
2022-08-20
2022-08-21
2022-08-22
2022-08-23
2022-08-24
2022-08-25
2022-08-26
2022-08-27
40°N
37.5°N
35°N
32.5°N
30°N
27.5°N
25°N
22.5°N
90°E
95°E
100°E
105°E
110°E
115°E
120°E
1.5
2.0
2.5
3.0
3.5
Severity

Supplementary Figure 462: Reference Event Mapping for China based on ERA5 - Spatio-temporal evolution of local heat-affected grid boxes during reference heatwave in China based on data in ERA5. Colors and color-bar indicate the local severity values for each day of the event. The respective date of the heatwave day is indicated above each sub-figure and x- and y-axis show the longitude and latitude coordinates.

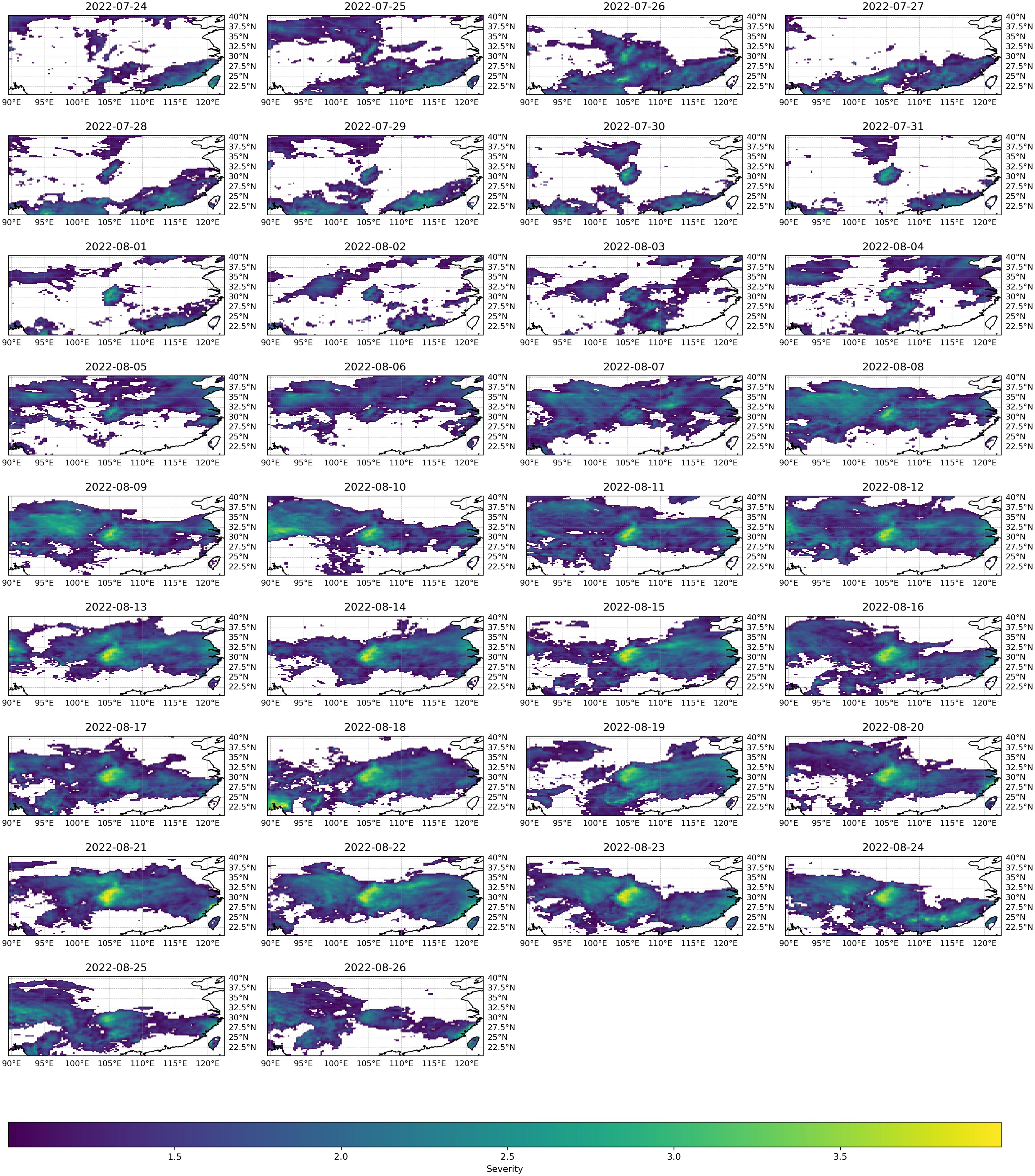
2022-07-24
2022-07-25
2022-07-26
2022-07-27
2022-07-28
2022-07-29
2022-07-30
2022-07-31
2022-08-01
2022-08-02
2022-08-03
2022-08-04
2022-08-05
2022-08-06
2022-08-07
2022-08-08
2022-08-09
2022-08-10
2022-08-11
2022-08-12
2022-08-13
2022-08-14
2022-08-15
2022-08-16
2022-08-17
2022-08-18
2022-08-19
2022-08-20
2022-08-21
2022-08-22
2022-08-23
2022-08-24
2022-08-25
2022-08-26
40°N
37.5°N
35°N
32.5°N
30°N
27.5°N
25°N
22.5°N
90°E
95°E
100°E
105°E
110°E
115°E
120°E
1.5
2.0
2.5
3.0
3.5
Severity

Supplementary Figure 463: Reference Event Mapping for Central & Eastern Europe based on Berkeley Earth - Spatio-temporal evolution of local heat-affected grid boxes during reference heatwave in Central & Eastern Europe based on data in Berkeley Earth. Colors and color-bar indicate the local severity values for each day of the event. The respective date of the heatwave day is indicated above each sub-figure and x- and y-axis show the longitude and latitude coordinates.

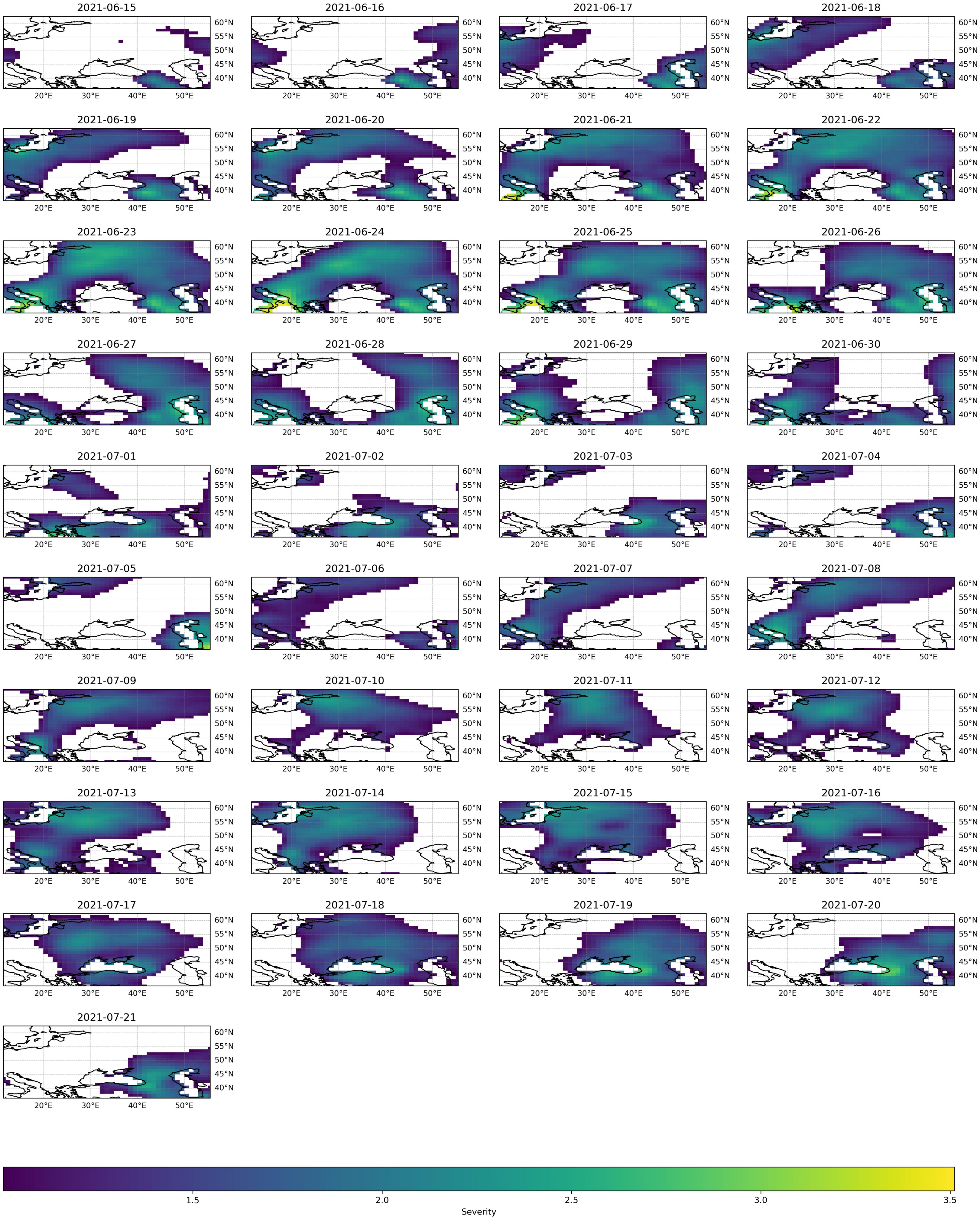
2021-06-15
2021-06-16
2021-06-17
2021-06-18
2021-06-19
2021-06-20
2021-06-21
2021-06-22
2021-06-23
2021-06-24
2021-06-25
2021-06-26
2021-06-27
2021-06-28
2021-06-29
2021-06-30
2021-07-01
2021-07-02
2021-07-03
2021-07-04
2021-07-05
2021-07-06
2021-07-07
2021-07-08
2021-07-09
2021-07-10
2021-07-11
2021-07-12
2021-07-13
2021-07-14
2021-07-15
2021-07-16
2021-07-17
2021-07-18
2021-07-19
2021-07-20
2021-07-21
60°N
55°N
50°N
45°N
40°N
20°E
30°E
40°E
50°E
1.5
2.0
2.5
3.0
3.5
Severity

Supplementary Figure 464: Reference Event Mapping for Central & Eastern Europe based on ERA5 - Spatio-temporal evolution of local heat-affected grid boxes during reference heatwave in Central & Eastern Europe based on data in ERA5. Colors and color-bar indicate the local severity values for each day of the event. The respective date of the heatwave day is indicated above each sub-figure and x- and y-axis show the longitude and latitude coordinates.

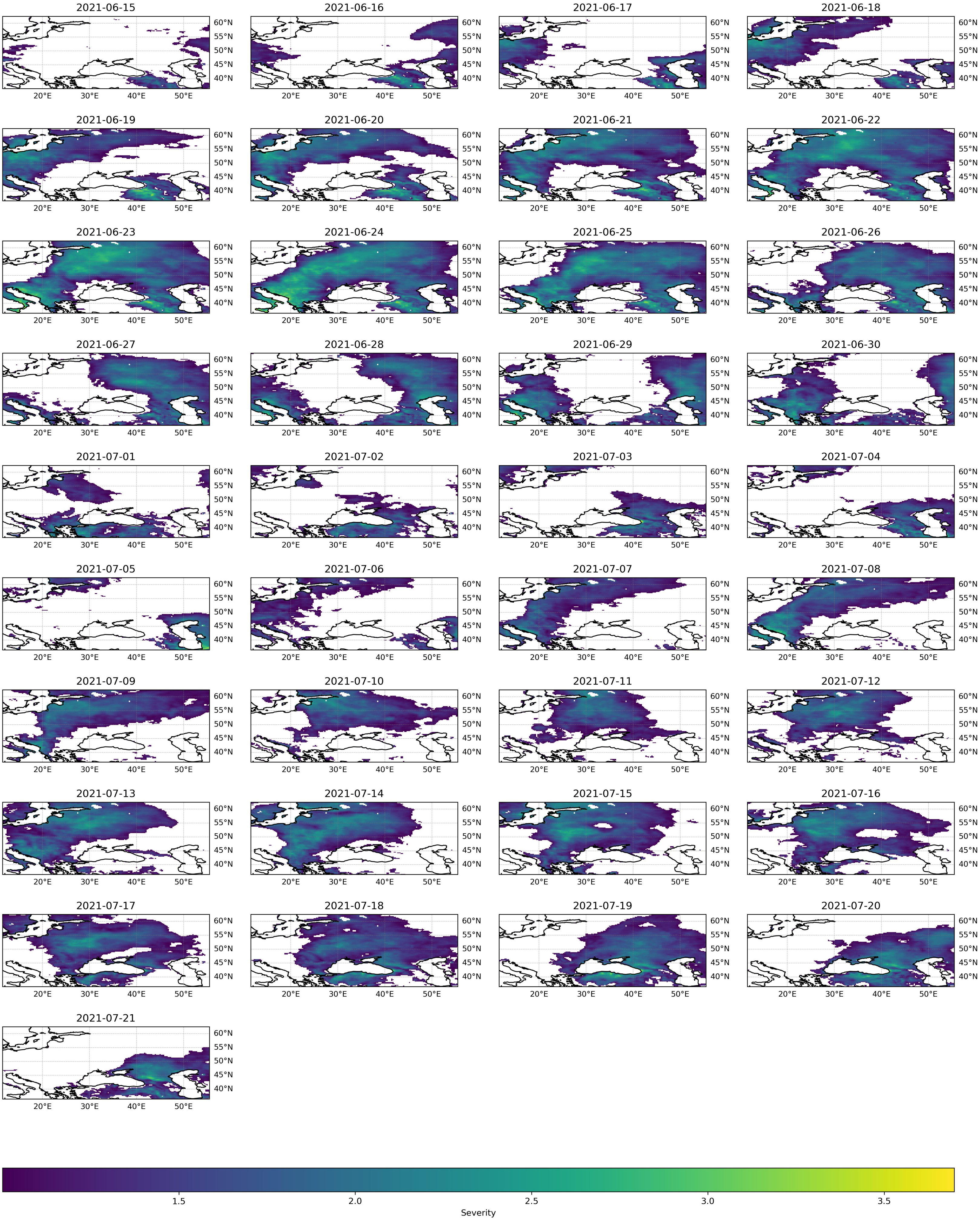
2021-06-15
2021-06-16
2021-06-17
2021-06-18
2021-06-19
2021-06-20
2021-06-21
2021-06-22
2021-06-23
2021-06-24
2021-06-25
2021-06-26
2021-06-27
2021-06-28
2021-06-29
2021-06-30
2021-07-01
2021-07-02
2021-07-03
2021-07-04
2021-07-05
2021-07-06
2021-07-07
2021-07-08
2021-07-09
2021-07-10
2021-07-11
2021-07-12
2021-07-13
2021-07-14
2021-07-15
2021-07-16
2021-07-17
2021-07-18
2021-07-19
2021-07-20
2021-07-21
60°N
55°N
50°N
45°N
40°N
20°E
30°E
40°E
50°E
1.5
2.0
2.5
3.0
3.5
Severity

Supplementary Figure 465: Reference Event Mapping for Europe based on Berkeley Earth - Spatio-temporal evolution of local heat-affected grid boxes during reference heatwave in Europe based on data in Berkeley Earth. Colors and color-bar indicate the local severity values for each day of the event. The respective date of the heatwave day is indicated above each sub-figure and x- and y-axis show the longitude and latitude coordinates.

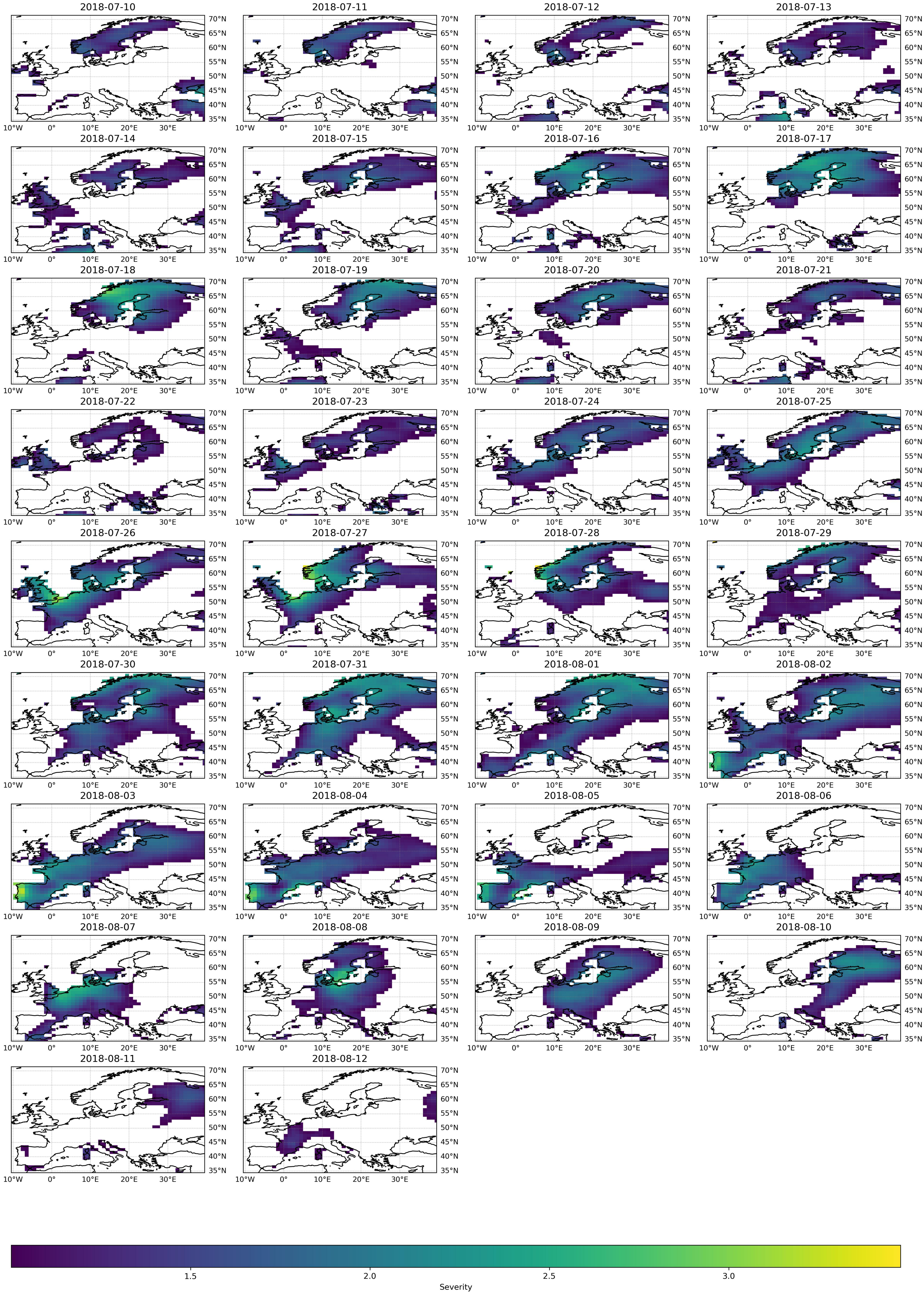
2018-07-10
2018-07-11
2018-07-12
2018-07-13
2018-07-14
2018-07-15
2018-07-16
2018-07-17
2018-07-18
2018-07-19
2018-07-20
2018-07-21
2018-07-22
2018-07-23
2018-07-24
2018-07-25
2018-07-26
2018-07-27
2018-07-28
2018-07-29
2018-07-30
2018-07-31
2018-08-01
2018-08-02
2018-08-03
2018-08-04
2018-08-05
2018-08-06
2018-08-07
2018-08-08
2018-08-09
2018-08-10
2018-08-11
2018-08-12
70°N
65°N
60°N
55°N
50°N
45°N
40°N
35°N
10°W
0°
10°E
20°E
30°E
1.5
2.0
2.5
3.0
Severity

Supplementary Figure 466: Reference Event Mapping for Europe based on ERA5 - Spatio-temporal evolution of local heat-affected grid boxes during reference heatwave in Europe based on data in ERA5. Colors and color-bar indicate the local severity values for each day of the event. The respective date of the heatwave day is indicated above each sub-figure and x- and y-axis show the longitude and latitude coordinates.

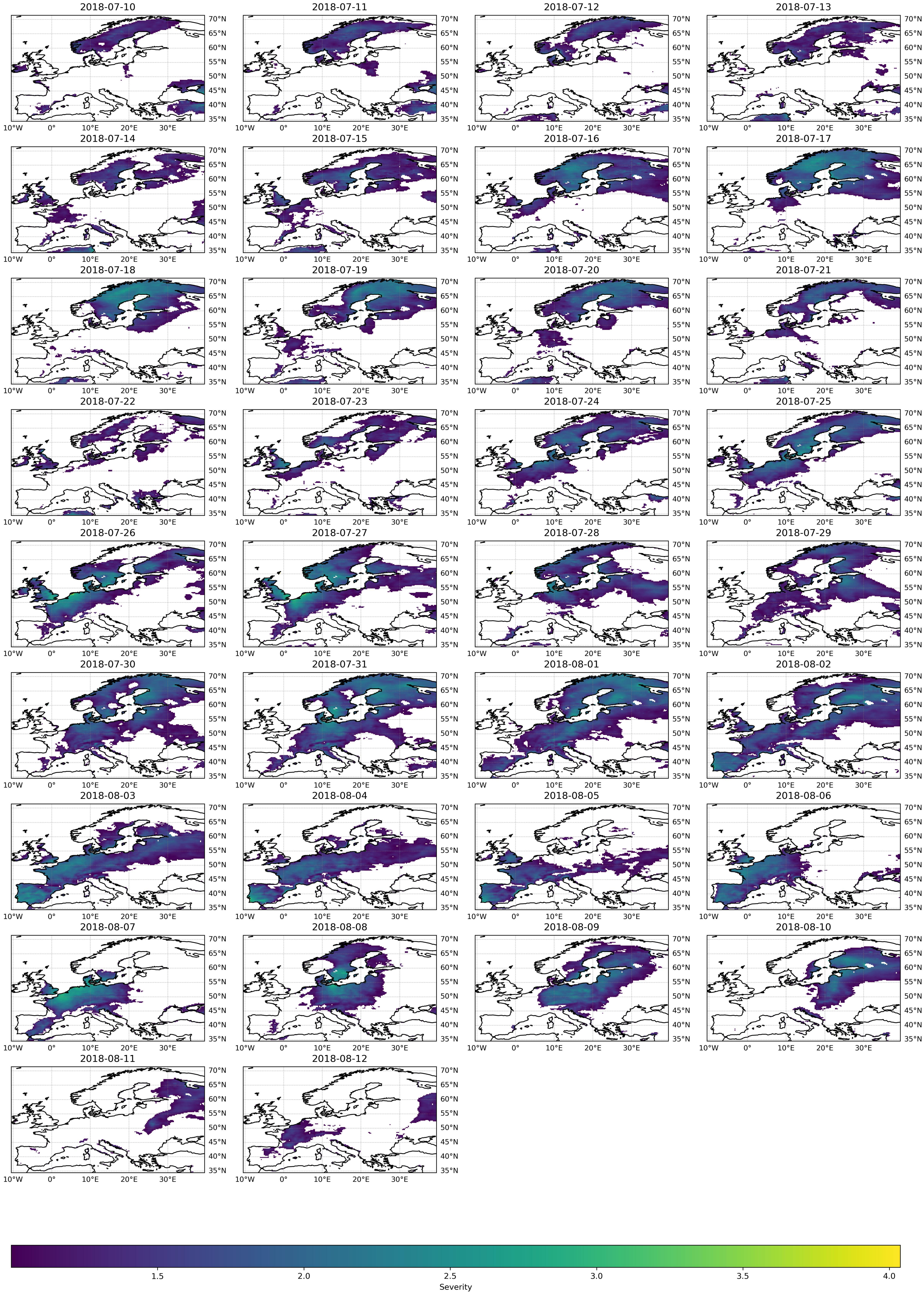

2018-07-10
2018-07-11
2018-07-12
2018-07-13
2018-07-14
2018-07-15
2018-07-16
2018-07-17
2018-07-18
2018-07-19
2018-07-20
2018-07-21
2018-07-22
2018-07-23
2018-07-24
2018-07-25
2018-07-26
2018-07-27
2018-07-28
2018-07-29
2018-07-30
2018-07-31
2018-08-01
2018-08-02
2018-08-03
2018-08-04
2018-08-05
2018-08-06
2018-08-07
2018-08-08
2018-08-09
2018-08-10
2018-08-11
2018-08-12
70°N
65°N
60°N
55°N
50°N
45°N
40°N
35°N
10°W
0°
10°E
20°E
30°E
1.5
2.0
2.5
3.0
3.5
4.0
Severity

Supplementary Figure 467: Reference Event Mapping for India & Middle East based on Berkeley Earth - Spatio-temporal evolution of local heat-affected grid boxes during reference heatwave in India & Middle East based on data in Berkeley Earth. Colors and color-bar indicate the local severity values for each day of the event. The respective date of the heatwave day is indicated above each sub-figure and x- and y-axis show the longitude and latitude coordinates.

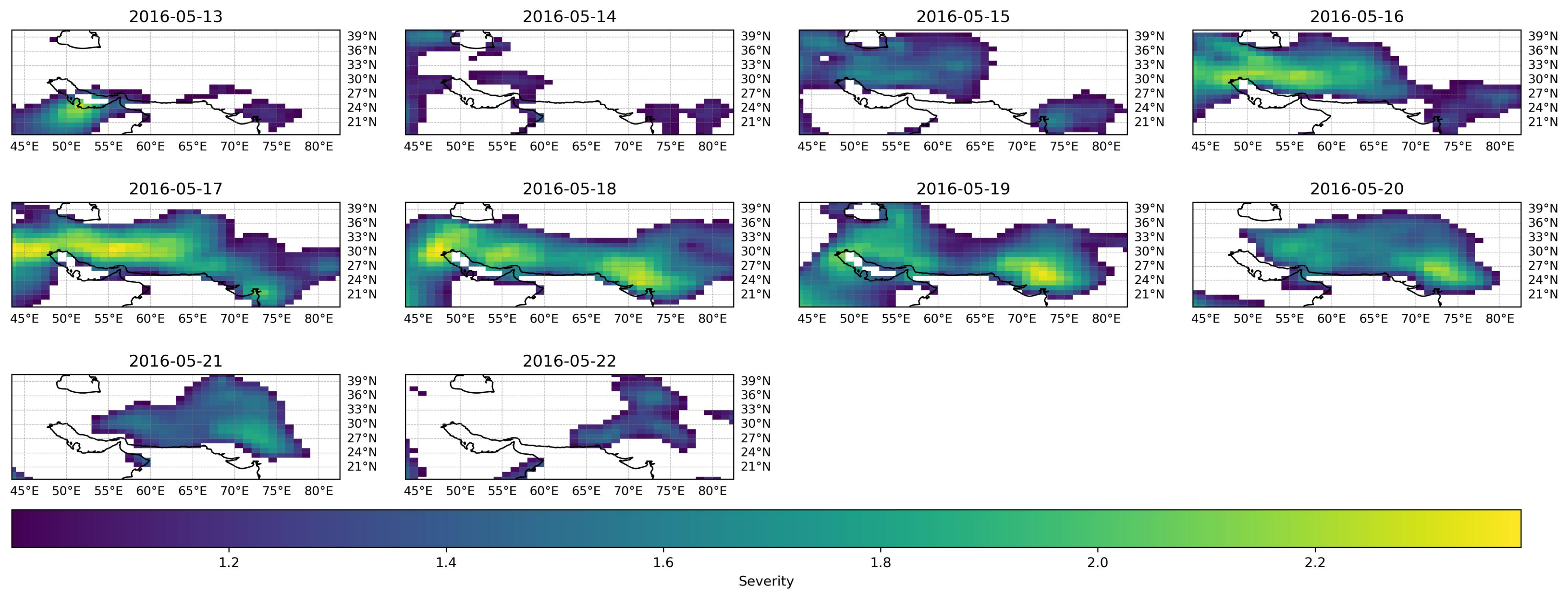

2016-05-13
2016-05-14
2016-05-15
2016-05-16
2016-05-17
2016-05-18
2016-05-19
2016-05-20
2016-05-21
2016-05-22
39°N
36°N
33°N
30°N
27°N
24°N
21°N
45°E
50°E
55°E
60°E
65°E
70°E
75°E
80°E
1.2
1.4
1.6
1.8
2.0
2.2
Severity

Supplementary Figure 468: Reference Event Mapping for India & Middle East based on ERA5 - Spatio-temporal evolution of local heat-affected grid boxes during reference heatwave in India & Middle East based on data in ERA5. Colors and color-bar indicate the local severity values for each day of the event. The respective date of the heatwave day is indicated above each sub-figure and x- and y-axis show the longitude and latitude coordinates.

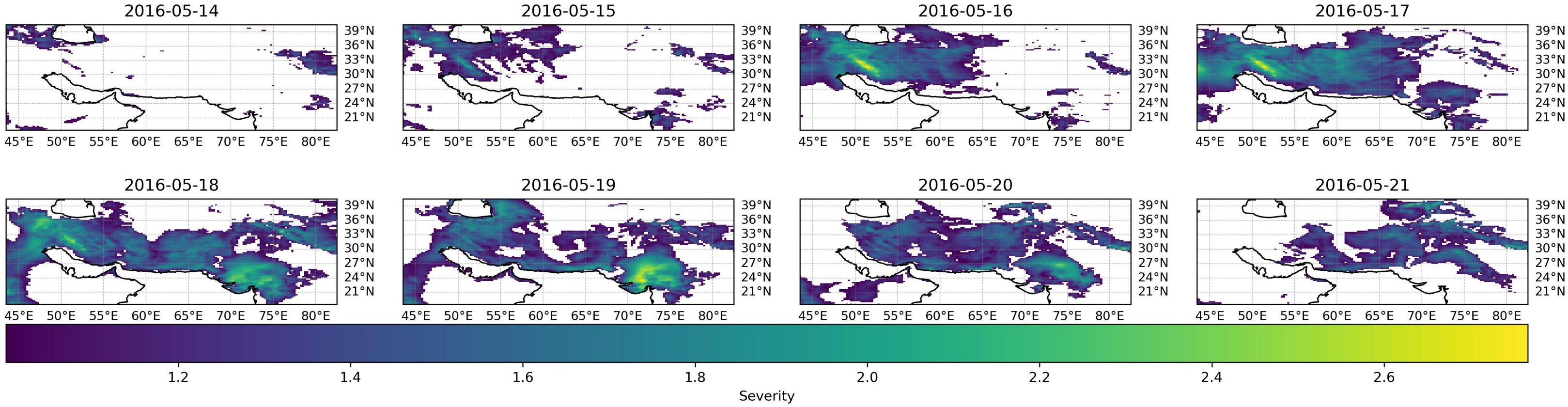
2016-05-14
2016-05-15
2016-05-16
2016-05-17
2016-05-18
2016-05-19
2016-05-20
2016-05-21
39°N
36°N
33°N
30°N
27°N
24°N
21°N
45°E
50°E
55°E
60°E
65°E
70°E
75°E
80°E
1.2
1.4
1.6
1.8
2.0
2.2
2.4
2.6
Severity

Supplementary Figure 469: Reference Event Mapping for India & Pakistan based on Berkeley Earth - Spatio-temporal evolution of local heat-affected grid boxes during reference heatwave in India & Pakistan based on data in Berkeley Earth. Colors and color-bar indicate the local severity values for each day of the event. The respective date of the heatwave day is indicated above each sub-figure and x- and y-axis show the longitude and latitude coordinates.

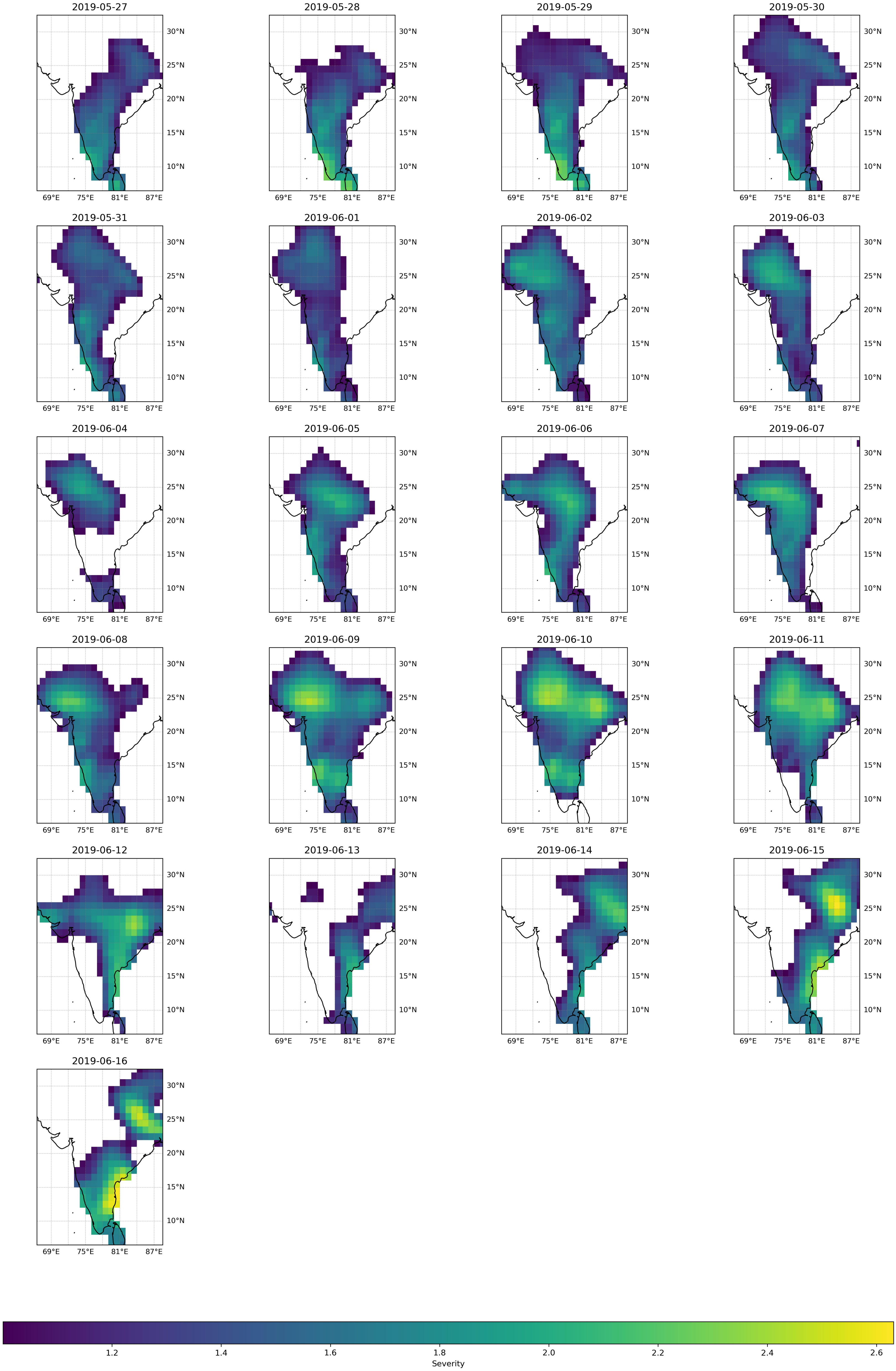
2019-05-27
2019-05-28
2019-05-29
2019-05-30
2019-05-31
2019-06-01
2019-06-02
2019-06-03
2019-06-04
2019-06-05
2019-06-06
2019-06-07
2019-06-08
2019-06-09
2019-06-10
2019-06-11
2019-06-12
2019-06-13
2019-06-14
2019-06-15
2019-06-16
30°N
25°N
20°N
15°N
10°N
69°E
75°E
81°E
87°E
1.2
1.4
1.6
1.8
2.0
2.2
2.4
2.6
Severity

Supplementary Figure 470: Reference Event Mapping for India & Pakistan based on ERA5 - Spatio-temporal evolution of local heat-affected grid boxes during reference heatwave in India & Pakistan based on data in ERA5. Colors and color-bar indicate the local severity values for each day of the event. The respective date of the heatwave day is indicated above each sub-figure and x- and y-axis show the longitude and latitude coordinates.

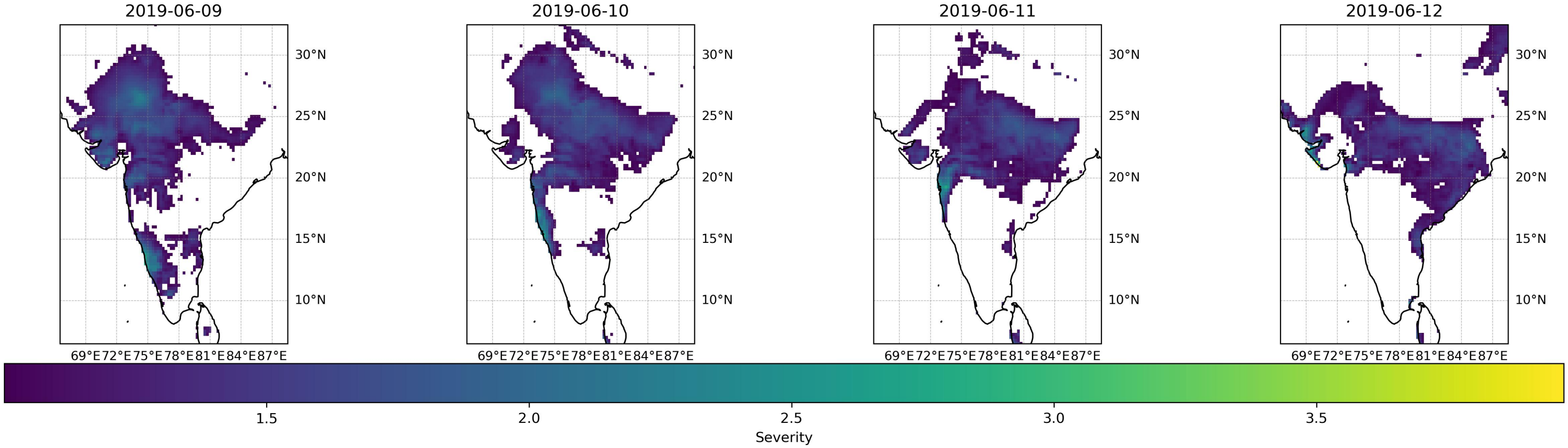

2019-06-09
2019-06-10
2019-06-11
2019-06-12
30°N
25°N
20°N
15°N
10°N
69°E72°E75°E78°E81°E84°E87°E
1.5
2.0
2.5
3.0
3.5
Severity

Supplementary Figure 471: Reference Event Mapping for Middle East based on Berkeley Earth - Spatio-temporal evolution of local heat-affected grid boxes during reference heatwave in Middle East based on data in Berkeley Earth. Colors and color-bar indicate the local severity values for each day of the event. The respective date of the heatwave day is indicated above each sub-figure and x- and y-axis show the longitude and latitude coordinates.

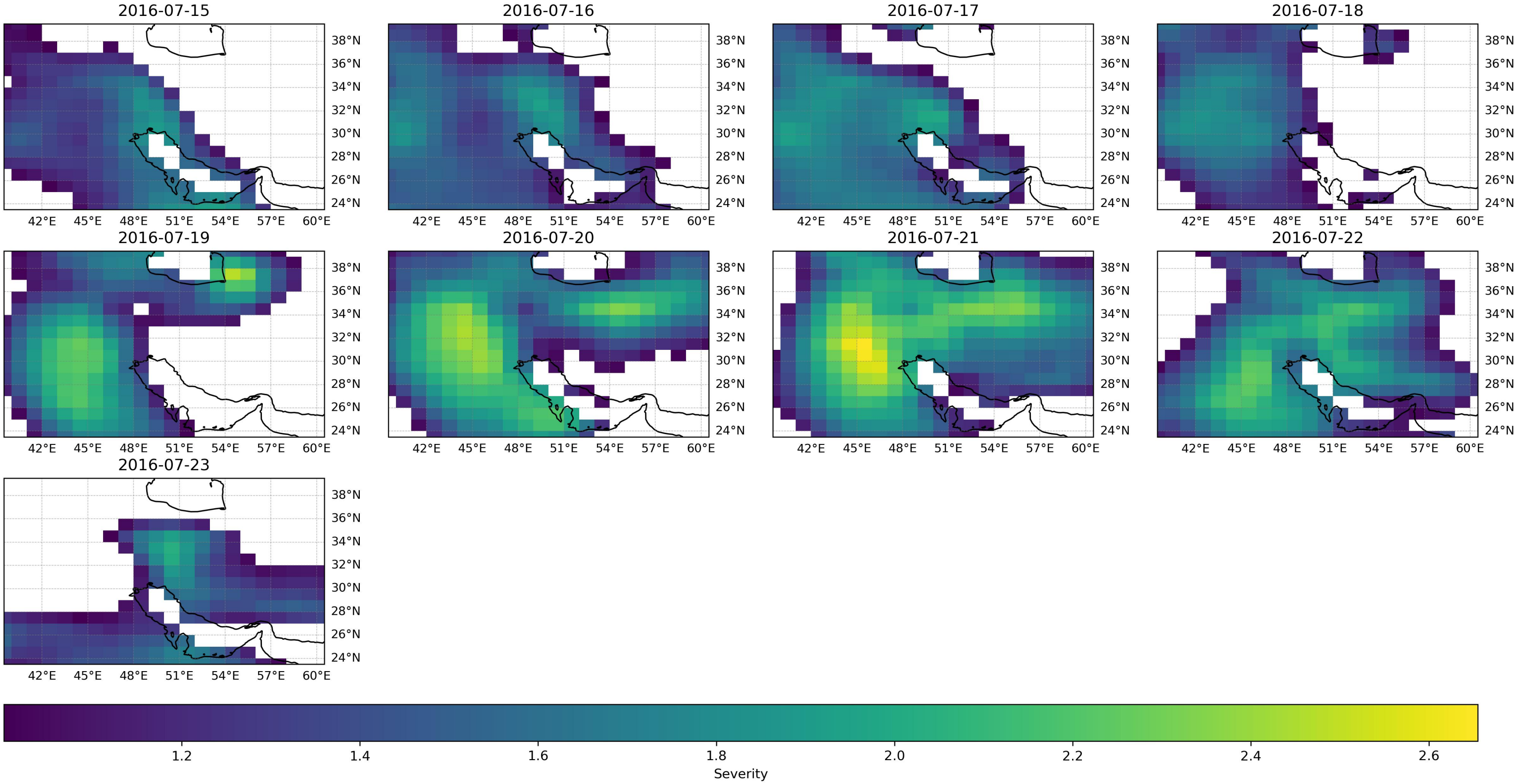
2016-07-15
2016-07-16
2016-07-17
2016-07-18
2016-07-19
2016-07-20
2016-07-21
2016-07-22
2016-07-23
38°N
36°N
34°N
32°N
30°N
28°N
26°N
24°N
42°E
45°E
48°E
51°E
54°E
57°E
60°E
1.2
1.4
1.6
1.8
2.0
2.2
2.4
2.6
Severity

Supplementary Figure 472: Reference Event Mapping for Middle East based on ERA5 - Spatio-temporal evolution of local heat-affected grid boxes during reference heatwave in Middle East based on data in ERA5. Colors and color-bar indicate the local severity values for each day of the event. The respective date of the heatwave day is indicated above each sub-figure and x- and y-axis show the longitude and latitude coordinates.

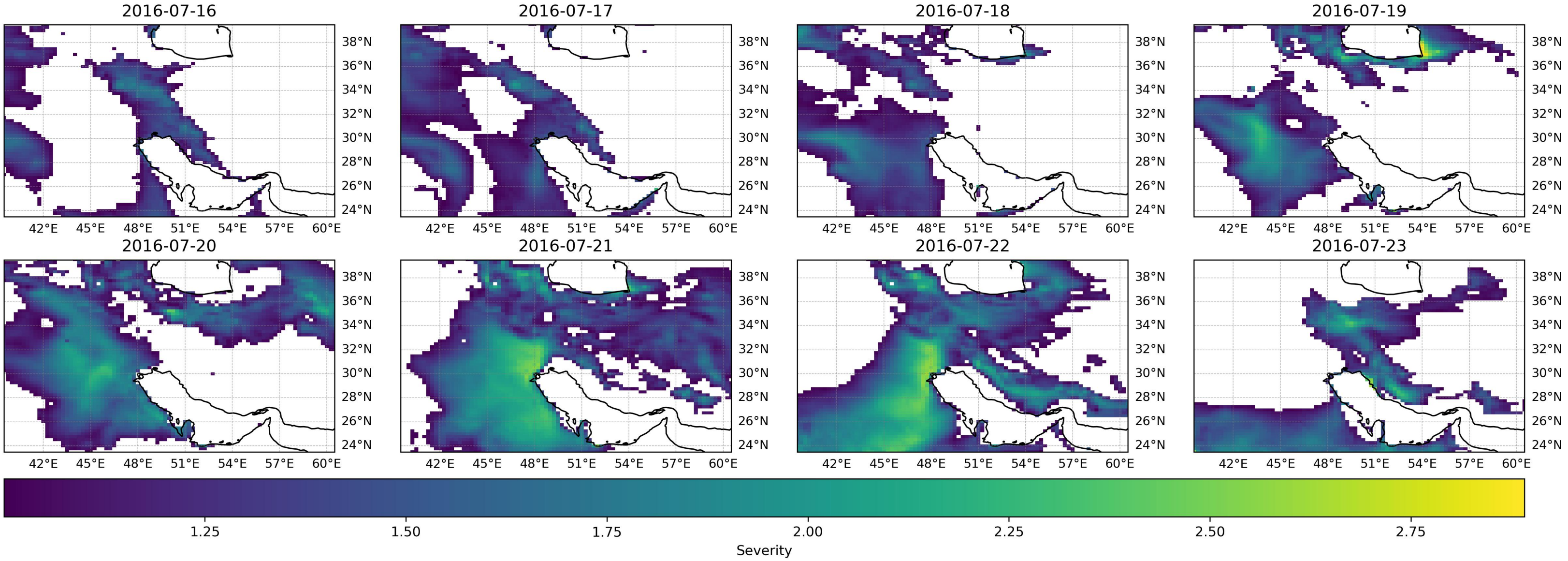
2016-07-16
2016-07-17
2016-07-18
2016-07-19
2016-07-20
2016-07-21
2016-07-22
2016-07-23
38°N
36°N
34°N
32°N
30°N
28°N
26°N
24°N
42°E
45°E
48°E
51°E
54°E
57°E
60°E
1.25
1.50
1.75
2.00
2.25
2.50
2.75
Severity

Supplementary Figure 473: Reference Event Mapping for Northeast Siberia based on Berkeley Earth - Spatio-temporal evolution of local heat-affected grid boxes during reference heatwave in Northeast Siberia based on data in Berkeley Earth. Colors and color-bar indicate the local severity values for each day of the event. The respective date of the heatwave day is indicated above each sub-figure and x- and y-axis show the longitude and latitude coordinates.

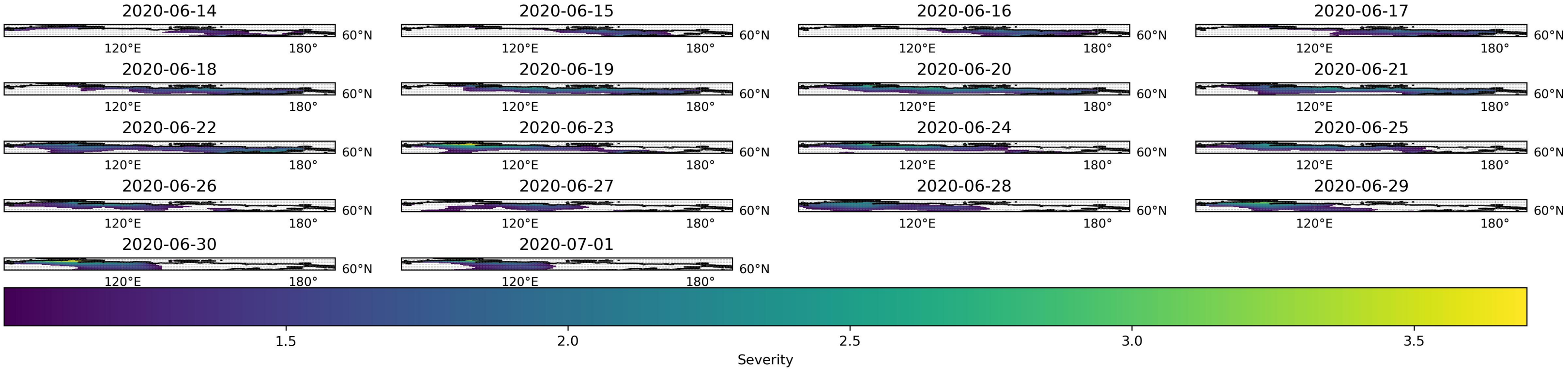

2020-06-14
2020-06-15
2020-06-16
2020-06-17
2020-06-18
2020-06-19
2020-06-20
2020-06-21
2020-06-22
2020-06-23
2020-06-24
2020-06-25
2020-06-26
2020-06-27
2020-06-28
2020-06-29
2020-06-30
2020-07-01
60°N
120°E
180°
1.5
2.0
2.5
3.0
3.5
Severity

Supplementary Figure 474: Reference Event Mapping for Northeast Siberia based on ERA5 - Spatio-temporal evolution of local heat-affected grid boxes during reference heatwave in Northeast Siberia based on data in ERA5. Colors and color-bar indicate the local severity values for each day of the event. The respective date of the heatwave day is indicated above each sub-figure and x- and y-axis show the longitude and latitude coordinates.

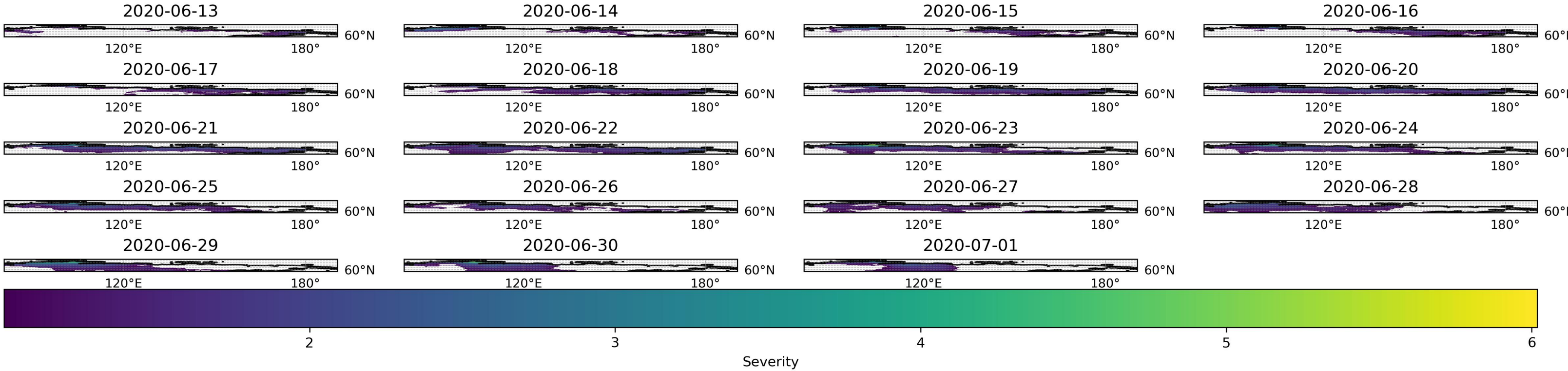
2020-06-13
2020-06-14
2020-06-15
2020-06-16
2020-06-17
2020-06-18
2020-06-19
2020-06-20
2020-06-21
2020-06-22
2020-06-23
2020-06-24
2020-06-25
2020-06-26
2020-06-27
2020-06-28
2020-06-29
2020-06-30
2020-07-01
60°N
120°E
180°
2
3
4
5
6
Severity

Supplementary Figure 475: Reference Event Mapping for Northern Africa & Middle East based on Berkeley Earth - Spatio-temporal evolution of local heat-affected grid boxes during reference heatwave in Northern Africa & Middle East based on data in Berkeley Earth. Colors and color-bar indicate the local severity values for each day of the event. The respective date of the heatwave day is indicated above each sub-figure and x- and y-axis show the longitude and latitude coordinates.

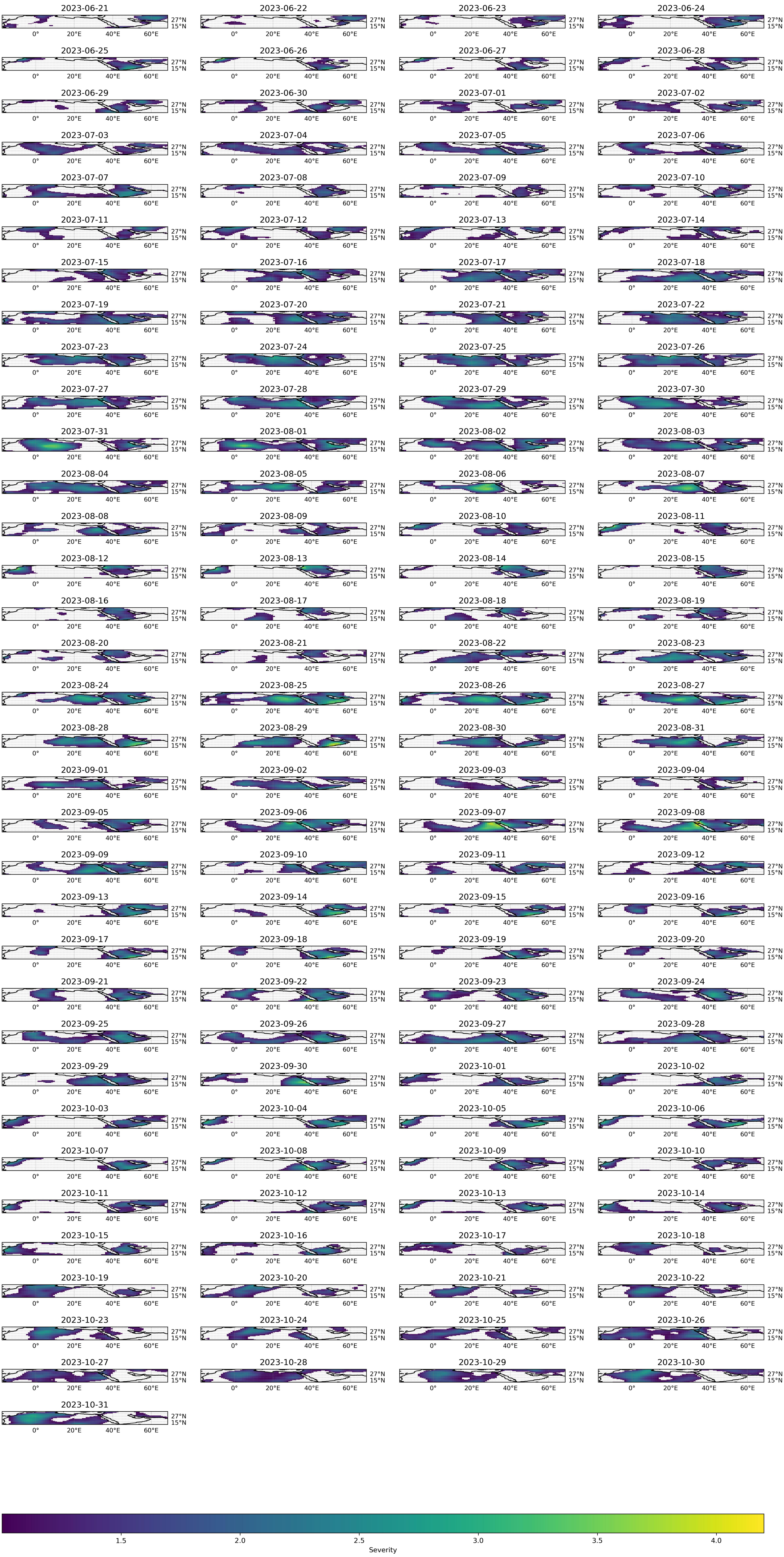
2023-06-21
2023-06-22
2023-06-23
2023-06-24
2023-06-25
2023-06-26
2023-06-27
2023-06-28
2023-06-29
2023-06-30
2023-07-01
2023-07-02
2023-07-03
2023-07-04
2023-07-05
2023-07-06
2023-07-07
2023-07-08
2023-07-09
2023-07-10
2023-07-11
2023-07-12
2023-07-13
2023-07-14
2023-07-15
2023-07-16
2023-07-17
2023-07-18
2023-07-19
2023-07-20
2023-07-21
2023-07-22
2023-07-23
2023-07-24
2023-07-25
2023-07-26
2023-07-27
2023-07-28
2023-07-29
2023-07-30
2023-07-31
2023-08-01
2023-08-02
2023-08-03
2023-08-04
2023-08-05
2023-08-06
2023-08-07
2023-08-08
2023-08-09
2023-08-10
2023-08-11
2023-08-12
2023-08-13
2023-08-14
2023-08-15
2023-08-16
2023-08-17
2023-08-18
2023-08-19
2023-08-20
2023-08-21
2023-08-22
2023-08-23
2023-08-24
2023-08-25
2023-08-26
2023-08-27
2023-08-28
2023-08-29
2023-08-30
2023-08-31
2023-09-01
2023-09-02
2023-09-03
2023-09-04
2023-09-05
2023-09-06
2023-09-07
2023-09-08
2023-09-09
2023-09-10
2023-09-11
2023-09-12
2023-09-13
2023-09-14
2023-09-15
2023-09-16
2023-09-17
2023-09-18
2023-09-19
2023-09-20
2023-09-21
2023-09-22
2023-09-23
2023-09-24
2023-09-25
2023-09-26
2023-09-27
2023-09-28
2023-09-29
2023-09-30
2023-10-01
2023-10-02
2023-10-03
2023-10-04
2023-10-05
2023-10-06
2023-10-07
2023-10-08
2023-10-09
2023-10-10
2023-10-11
2023-10-12
2023-10-13
2023-10-14
2023-10-15
2023-10-16
2023-10-17
2023-10-18
2023-10-19
2023-10-20
2023-10-21
2023-10-22
2023-10-23
2023-10-24
2023-10-25
2023-10-26
2023-10-27
2023-10-28
2023-10-29
2023-10-30
2023-10-31
27°N
15°N
0°
20°E
40°E
60°E
1.5
2.0
2.5
3.0
3.5
4.0
Severity

Supplementary Figure 476: Reference Event Mapping for Northern Africa & Middle East based on ERA5 - Spatio-temporal evolution of local heat-affected grid boxes during reference heatwave in Northern Africa & Middle East based on data in ERA5. Colors and color-bar indicate the local severity values for each day of the event. The respective date of the heatwave day is indicated above each sub-figure and x- and y-axis show the longitude and latitude coordinates.

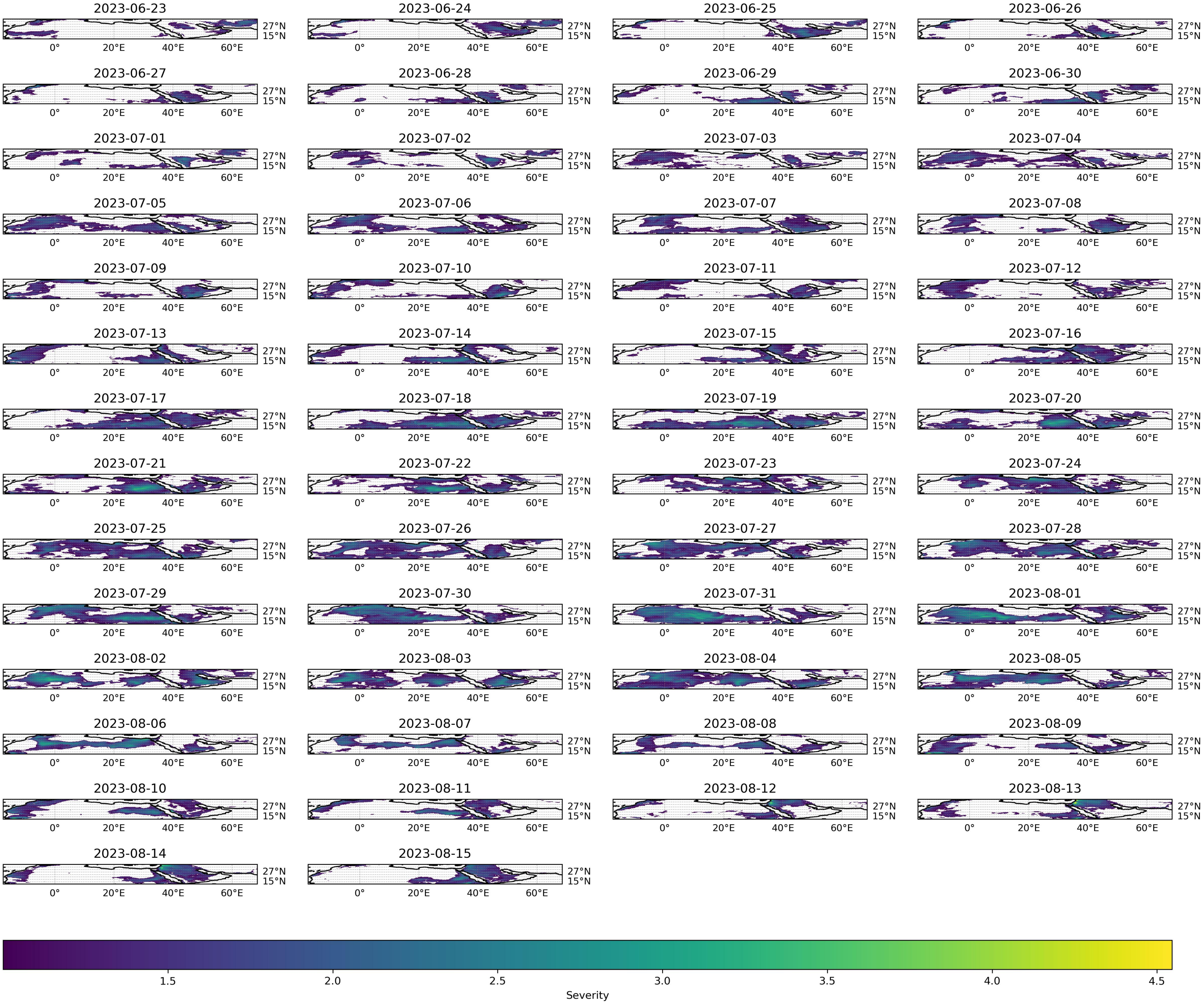
2023-06-23
2023-06-24
2023-06-25
2023-06-26
2023-06-27
2023-06-28
2023-06-29
2023-06-30
2023-07-01
2023-07-02
2023-07-03
2023-07-04
2023-07-05
2023-07-06
2023-07-07
2023-07-08
2023-07-09
2023-07-10
2023-07-11
2023-07-12
2023-07-13
2023-07-14
2023-07-15
2023-07-16
2023-07-17
2023-07-18
2023-07-19
2023-07-20
2023-07-21
2023-07-22
2023-07-23
2023-07-24
2023-07-25
2023-07-26
2023-07-27
2023-07-28
2023-07-29
2023-07-30
2023-07-31
2023-08-01
2023-08-02
2023-08-03
2023-08-04
2023-08-05
2023-08-06
2023-08-07
2023-08-08
2023-08-09
2023-08-10
2023-08-11
2023-08-12
2023-08-13
2023-08-14
2023-08-15
27°N
15°N
0°
20°E
40°E
60°E
1.5
2.0
2.5
3.0
3.5
4.0
4.5
Severity

Supplementary Figure 477: Reference Event Mapping for Pacific Northwest USA & Canada based on Berkeley Earth - Spatio-temporal evolution of local heat-affected grid boxes during reference heatwave in Pacific Northwest USA & Canada based on data in Berkeley Earth. Colors and color-bar indicate the local severity values for each day of the event. The respective date of the heatwave day is indicated above each sub-figure and x- and y-axis show the longitude and latitude coordinates.

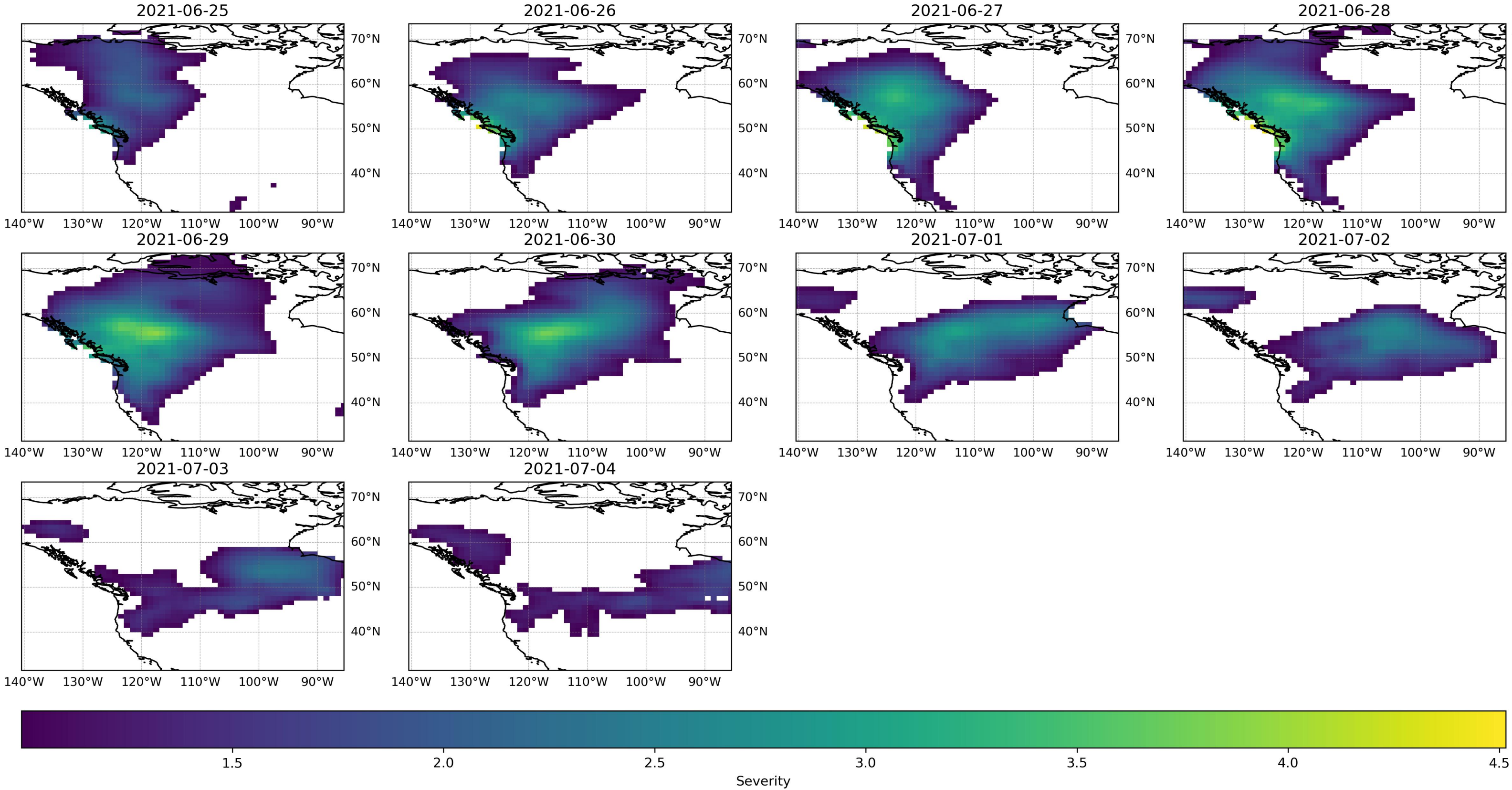

2021-06-25
2021-06-26
2021-06-27
2021-06-28
2021-06-29
2021-06-30
2021-07-01
2021-07-02
2021-07-03
2021-07-04
70°N
60°N
50°N
40°N
140°W
130°W
120°W
110°W
100°W
90°W
1.5
2.0
2.5
3.0
3.5
4.0
4.5
Severity

Supplementary Figure 478: Reference Event Mapping for Pacific Northwest USA & Canada based on ERA5 - Spatio-temporal evolution of local heat-affected grid boxes during reference heatwave in Pacific Northwest USA & Canada based on data in ERA5. Colors and color-bar indicate the local severity values for each day of the event. The respective date of the heatwave day is indicated above each sub-figure and x- and y-axis show the longitude and latitude coordinates.

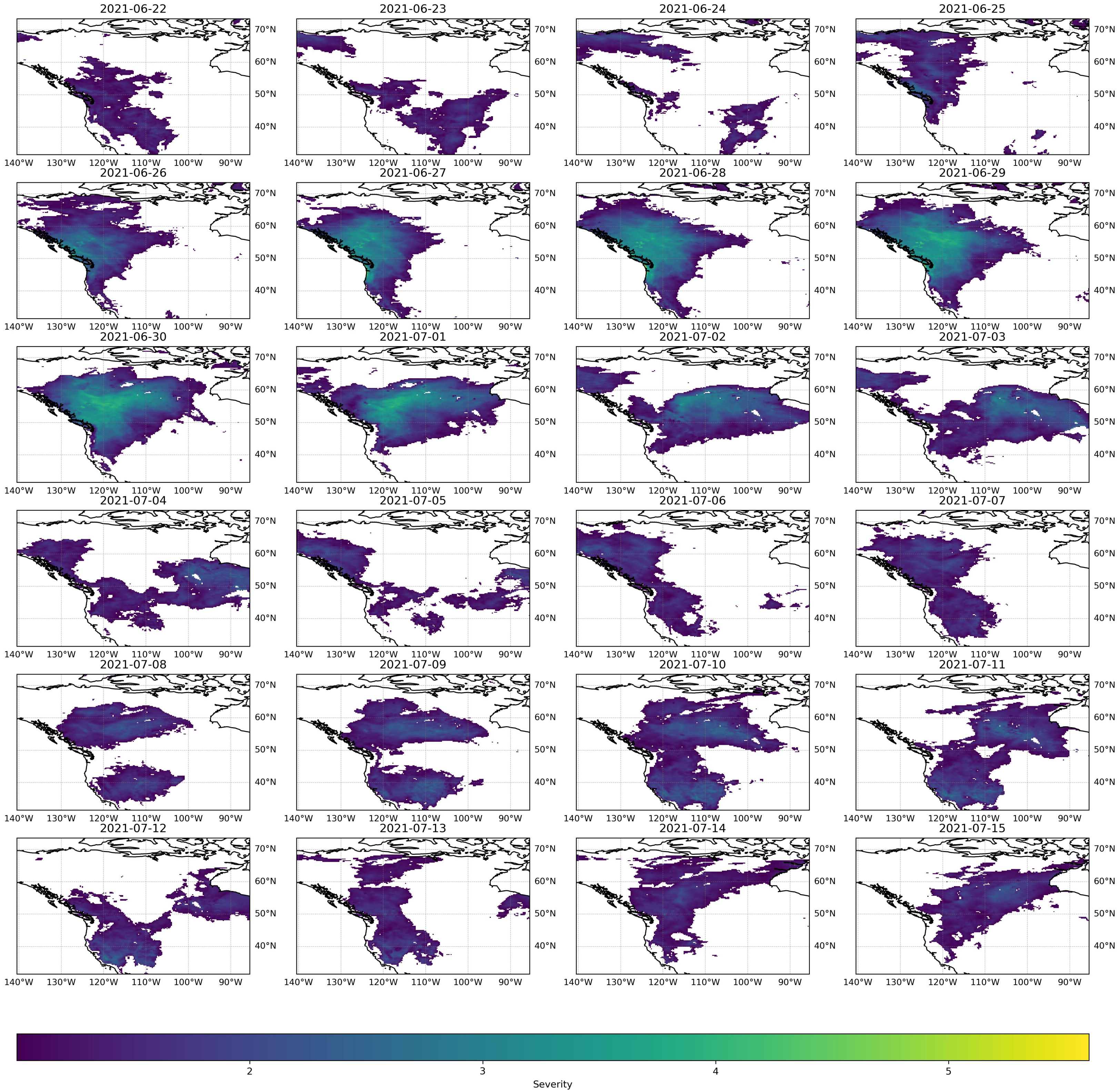
2021-06-22
2021-06-23
2021-06-24
2021-06-25
2021-06-26
2021-06-27
2021-06-28
2021-06-29
2021-06-30
2021-07-01
2021-07-02
2021-07-03
2021-07-04
2021-07-05
2021-07-06
2021-07-07
2021-07-08
2021-07-09
2021-07-10
2021-07-11
2021-07-12
2021-07-13
2021-07-14
2021-07-15
70°N
60°N
50°N
40°N
140°W
130°W
120°W
110°W
100°W
90°W
2
3
4
5
Severity

Supplementary Figure 479: Reference Event Mapping for Russia based on Berkeley Earth - Spatio-temporal evolution of local heat-affected grid boxes during reference heatwave in Russia based on data in Berkeley Earth. Colors and color-bar indicate the local severity values for each day of the event. The respective date of the heatwave day is indicated above each sub-figure and x- and y-axis show the longitude and latitude coordinates.

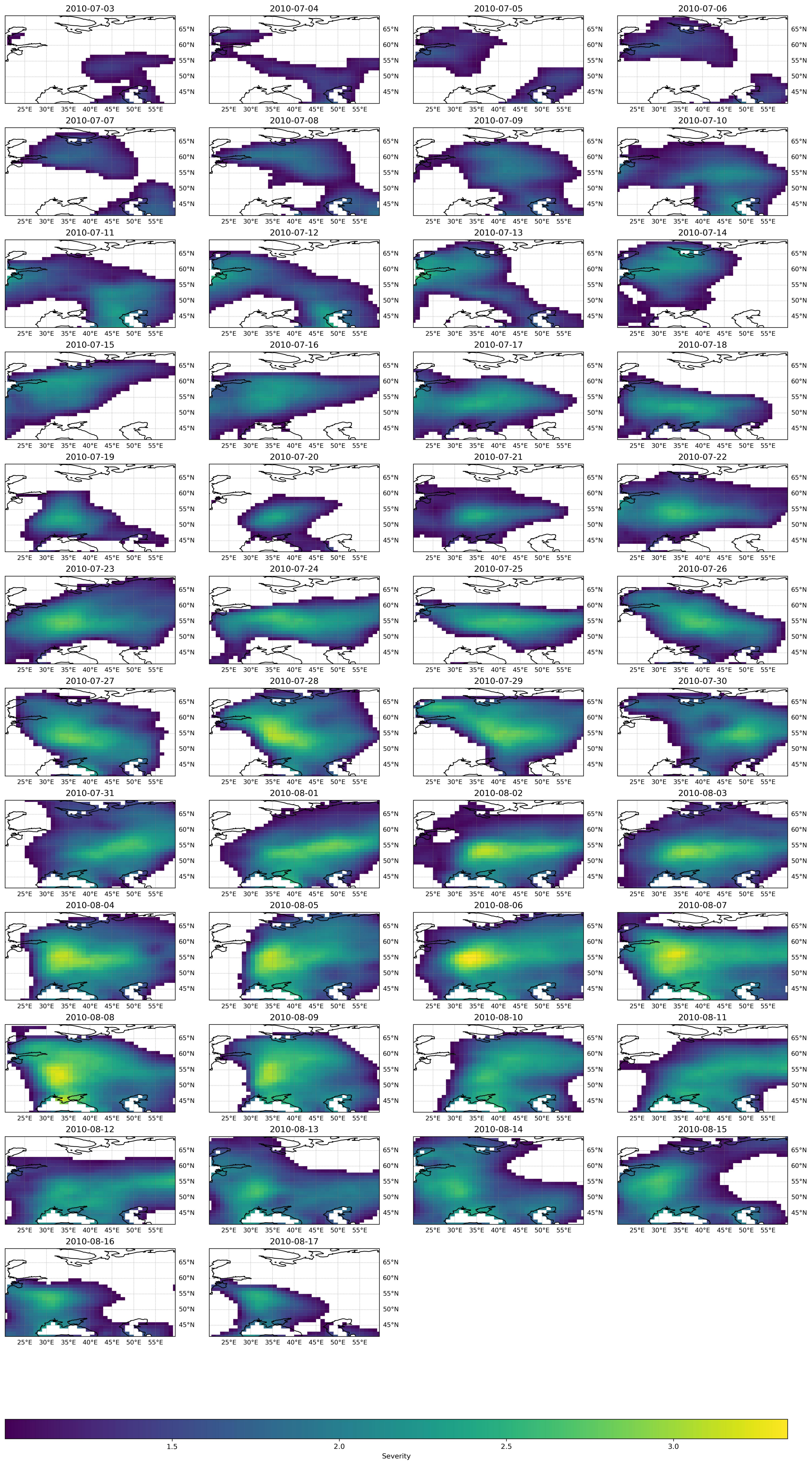

2010-07-03
2010-07-04
2010-07-05
2010-07-06
2010-07-07
2010-07-08
2010-07-09
2010-07-10
2010-07-11
2010-07-12
2010-07-13
2010-07-14
2010-07-15
2010-07-16
2010-07-17
2010-07-18
2010-07-19
2010-07-20
2010-07-21
2010-07-22
2010-07-23
2010-07-24
2010-07-25
2010-07-26
2010-07-27
2010-07-28
2010-07-29
2010-07-30
2010-07-31
2010-08-01
2010-08-02
2010-08-03
2010-08-04
2010-08-05
2010-08-06
2010-08-07
2010-08-08
2010-08-09
2010-08-10
2010-08-11
2010-08-12
2010-08-13
2010-08-14
2010-08-15
2010-08-16
2010-08-17
65°N
60°N
55°N
50°N
45°N
25°E 30°E 35°E 40°E 45°E 50°E 55°E
1.5
2.0
2.5
3.0
Severity

Supplementary Figure 480: Reference Event Mapping for Russia based on ERA5 - Spatio-temporal evolution of local heat-affected grid boxes during reference heatwave in Russia based on data in ERA5. Colors and color-bar indicate the local severity values for each day of the event. The respective date of the heatwave day is indicated above each sub-figure and x- and y-axis show the longitude and latitude coordinates.

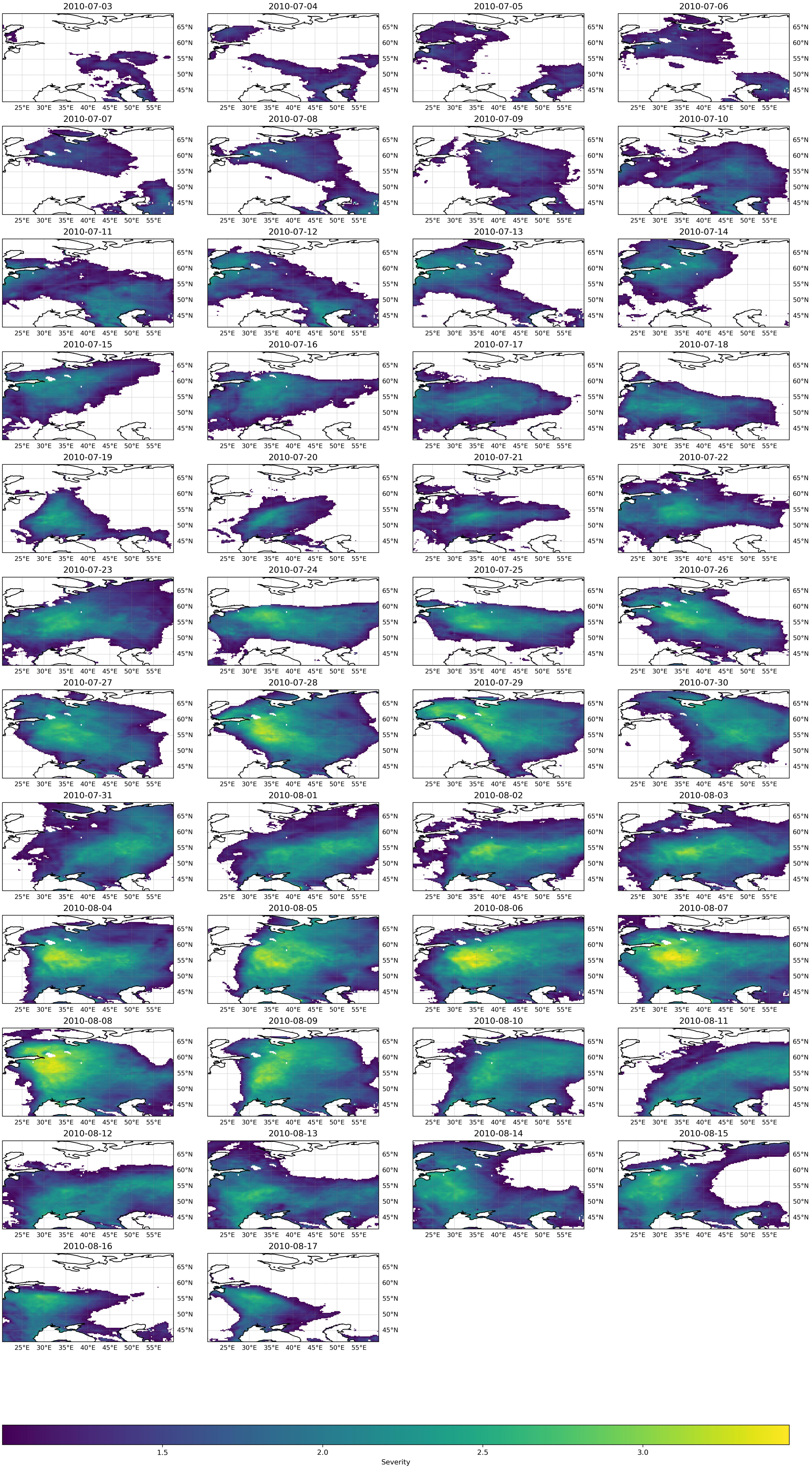
2010-07-03
2010-07-04
2010-07-05
2010-07-06
2010-07-07
2010-07-08
2010-07-09
2010-07-10
2010-07-11
2010-07-12
2010-07-13
2010-07-14
2010-07-15
2010-07-16
2010-07-17
2010-07-18
2010-07-19
2010-07-20
2010-07-21
2010-07-22
2010-07-23
2010-07-24
2010-07-25
2010-07-26
2010-07-27
2010-07-28
2010-07-29
2010-07-30
2010-07-31
2010-08-01
2010-08-02
2010-08-03
2010-08-04
2010-08-05
2010-08-06
2010-08-07
2010-08-08
2010-08-09
2010-08-10
2010-08-11
2010-08-12
2010-08-13
2010-08-14
2010-08-15
2010-08-16
2010-08-17
65°N
60°N
55°N
50°N
45°N
25°E
30°E
35°E
40°E
45°E
50°E
55°E
1.5
2.0
2.5
3.0
Severity

Supplementary Figure 481: Reference Event Mapping for Southwestern USA & Mexico based on Berkeley Earth - Spatio-temporal evolution of local heat-affected grid boxes during reference heatwave in Southwestern USA & Mexico based on data in Berkeley Earth. Colors and color-bar indicate the local severity values for each day of the event. The respective date of the heatwave day is indicated above each sub-figure and x- and y-axis show the longitude and latitude coordinates.

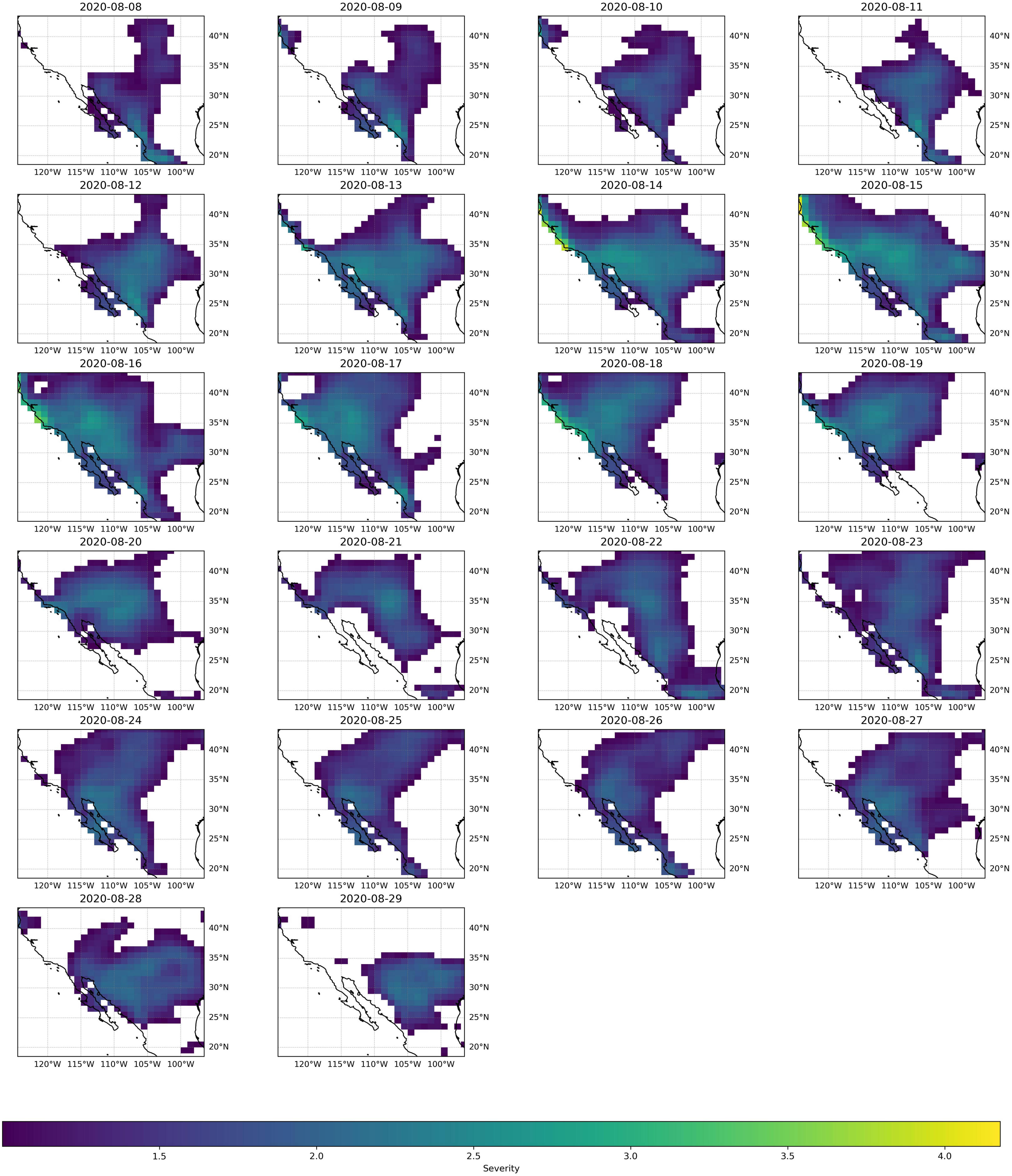
2020-08-08
2020-08-09
2020-08-10
2020-08-11
2020-08-12
2020-08-13
2020-08-14
2020-08-15
2020-08-16
2020-08-17
2020-08-18
2020-08-19
2020-08-20
2020-08-21
2020-08-22
2020-08-23
2020-08-24
2020-08-25
2020-08-26
2020-08-27
2020-08-28
2020-08-29
40°N
35°N
30°N
25°N
20°N
120°W 115°W 110°W 105°W 100°W
1.5
2.0
2.5
3.0
3.5
4.0
Severity

Supplementary Figure 482: Reference Event Mapping for Southwestern USA & Mexico based on ERA5 - Spatio-temporal evolution of local heat-affected grid boxes during reference heatwave in Southwestern USA & Mexico based on data in ERA5. Colors and color-bar indicate the local severity values for each day of the event. The respective date of the heatwave day is indicated above each sub-figure and x- and y-axis show the longitude and latitude coordinates.

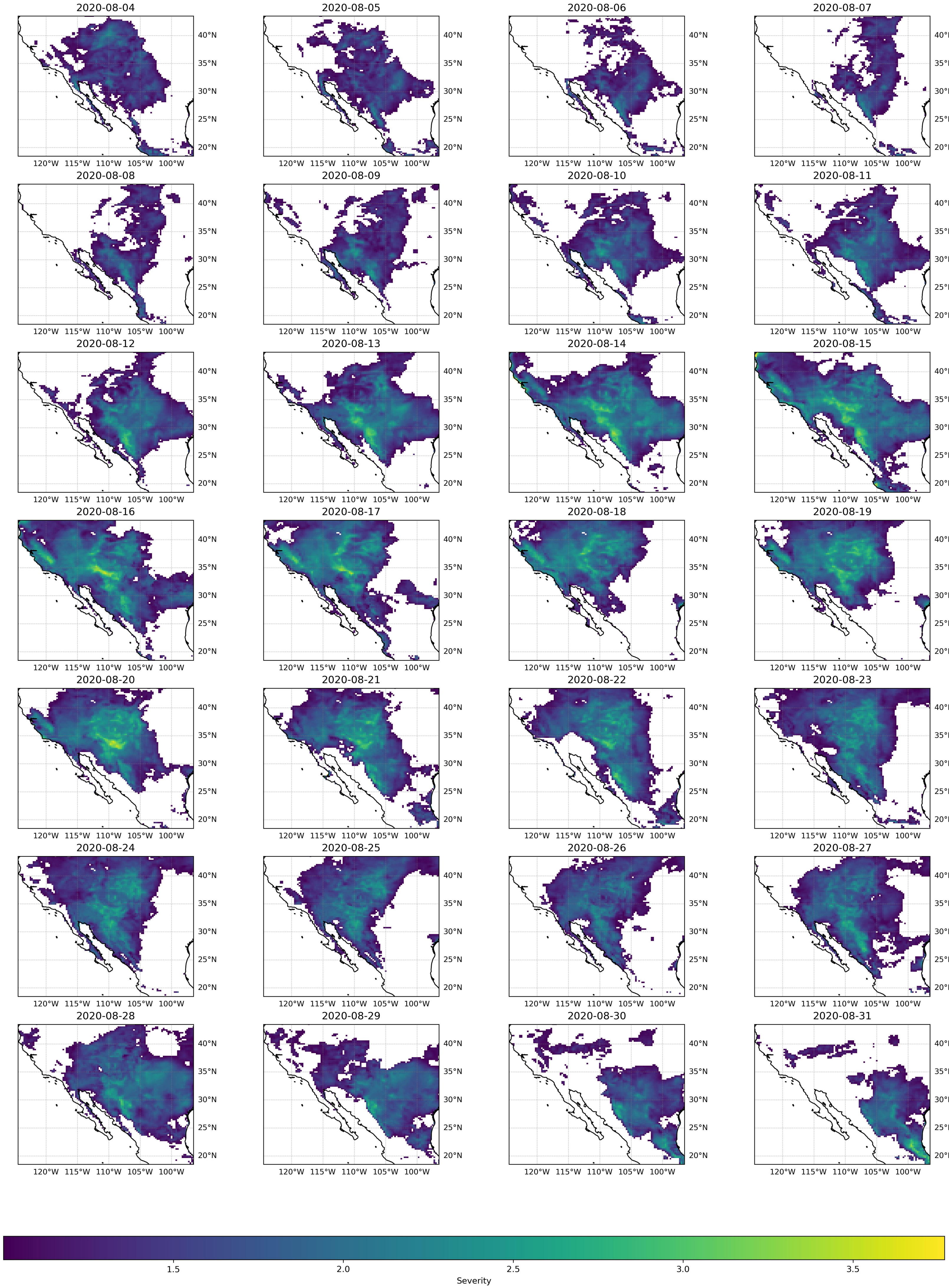
2020-08-04
2020-08-05
2020-08-06
2020-08-07
2020-08-08
2020-08-09
2020-08-10
2020-08-11
2020-08-12
2020-08-13
2020-08-14
2020-08-15
2020-08-16
2020-08-17
2020-08-18
2020-08-19
2020-08-20
2020-08-21
2020-08-22
2020-08-23
2020-08-24
2020-08-25
2020-08-26
2020-08-27
2020-08-28
2020-08-29
2020-08-30
2020-08-31
120°W 115°W 110°W 105°W 100°W
40°N 35°N 30°N 25°N 20°N
1.5 2.0 2.5 3.0 3.5
Severity

Supplementary Figure 483: Reference Event Mapping for Siberia based on Berkeley Earth - Spatio-temporal evolution of local heat-affected grid boxes during reference heatwave in Siberia based on data in Berkeley Earth. Colors and color-bar indicate the local severity values for each day of the event. The respective date of the heatwave day is indicated above each sub-figure and x- and y-axis show the longitude and latitude coordinates.

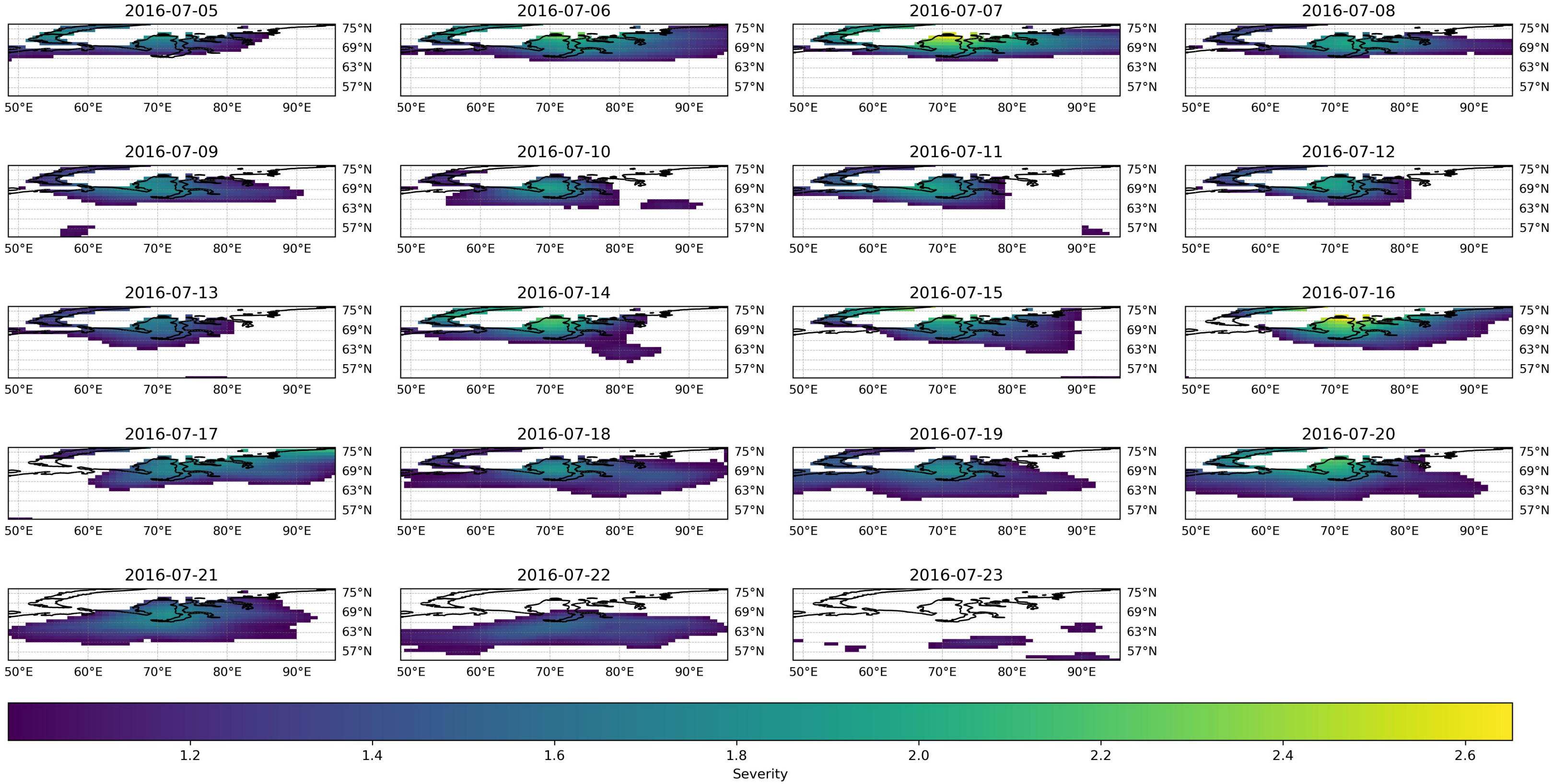
2016-07-05
2016-07-06
2016-07-07
2016-07-08
2016-07-09
2016-07-10
2016-07-11
2016-07-12
2016-07-13
2016-07-14
2016-07-15
2016-07-16
2016-07-17
2016-07-18
2016-07-19
2016-07-20
2016-07-21
2016-07-22
2016-07-23
75°N
69°N
63°N
57°N
50°E
60°E
70°E
80°E
90°E
1.2
1.4
1.6
1.8
2.0
2.2
2.4
2.6
Severity

Supplementary Figure 484: Reference Event Mapping for Siberia based on ERA5 - Spatio-temporal evolution of local heat-affected grid boxes during reference heatwave in Siberia based on data in ERA5. Colors and color-bar indicate the local severity values for each day of the event. The respective date of the heatwave day is indicated above each sub-figure and x- and y-axis show the longitude and latitude coordinates.

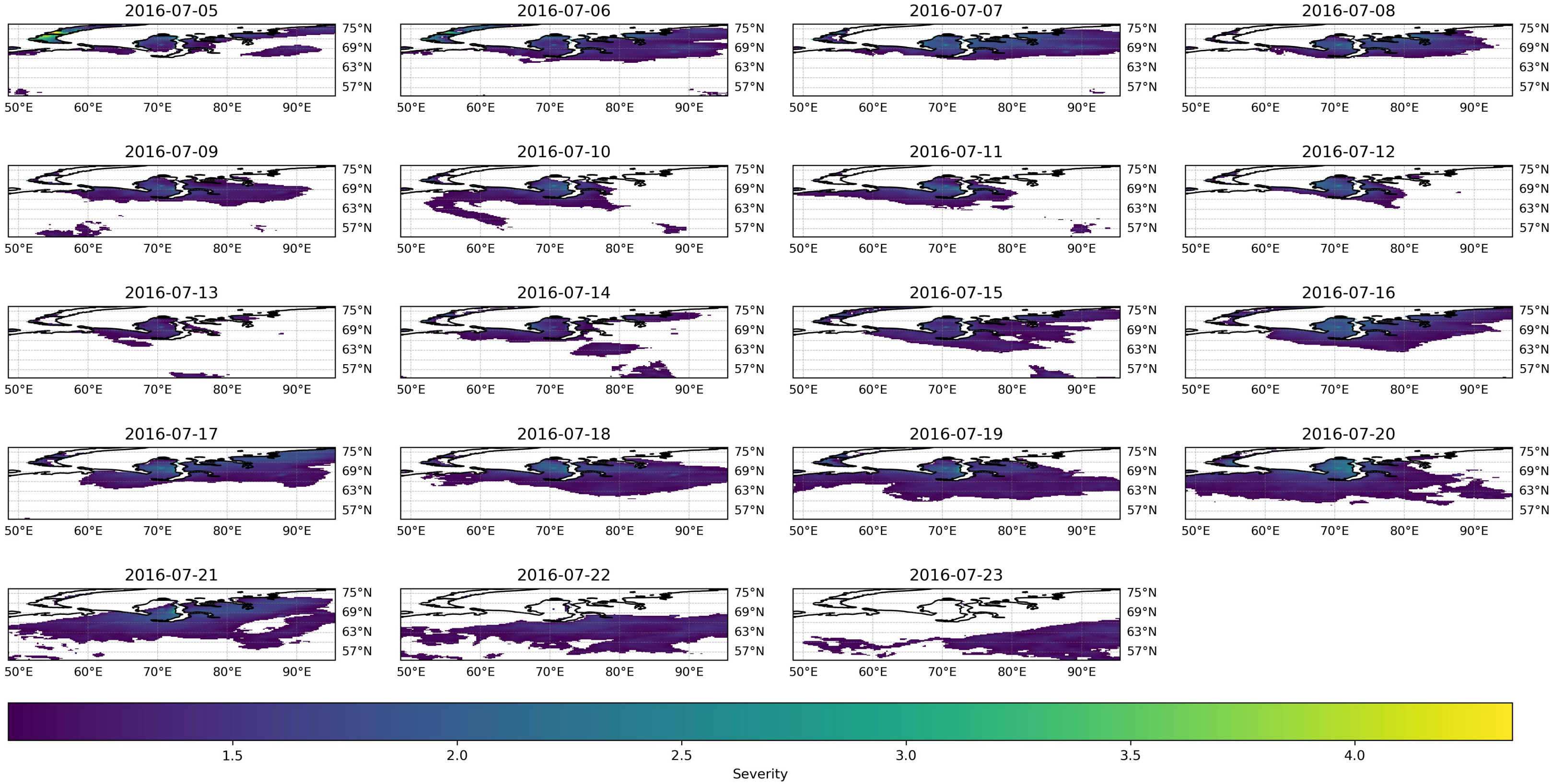
2016-07-05
2016-07-06
2016-07-07
2016-07-08
2016-07-09
2016-07-10
2016-07-11
2016-07-12
2016-07-13
2016-07-14
2016-07-15
2016-07-16
2016-07-17
2016-07-18
2016-07-19
2016-07-20
2016-07-21
2016-07-22
2016-07-23
75°N
69°N
63°N
57°N
50°E
60°E
70°E
80°E
90°E
1.5
2.0
2.5
3.0
3.5
4.0
Severity

Supplementary Figure 485: Reference Event Mapping for South America based on Berkeley Earth - Spatio-temporal evolution of local heat-affected grid boxes during reference heatwave in South America based on data in Berkeley Earth. Colors and color-bar indicate the local severity values for each day of the event. The respective date of the heatwave day is indicated above each sub-figure and x- and y-axis show the longitude and latitude coordinates.

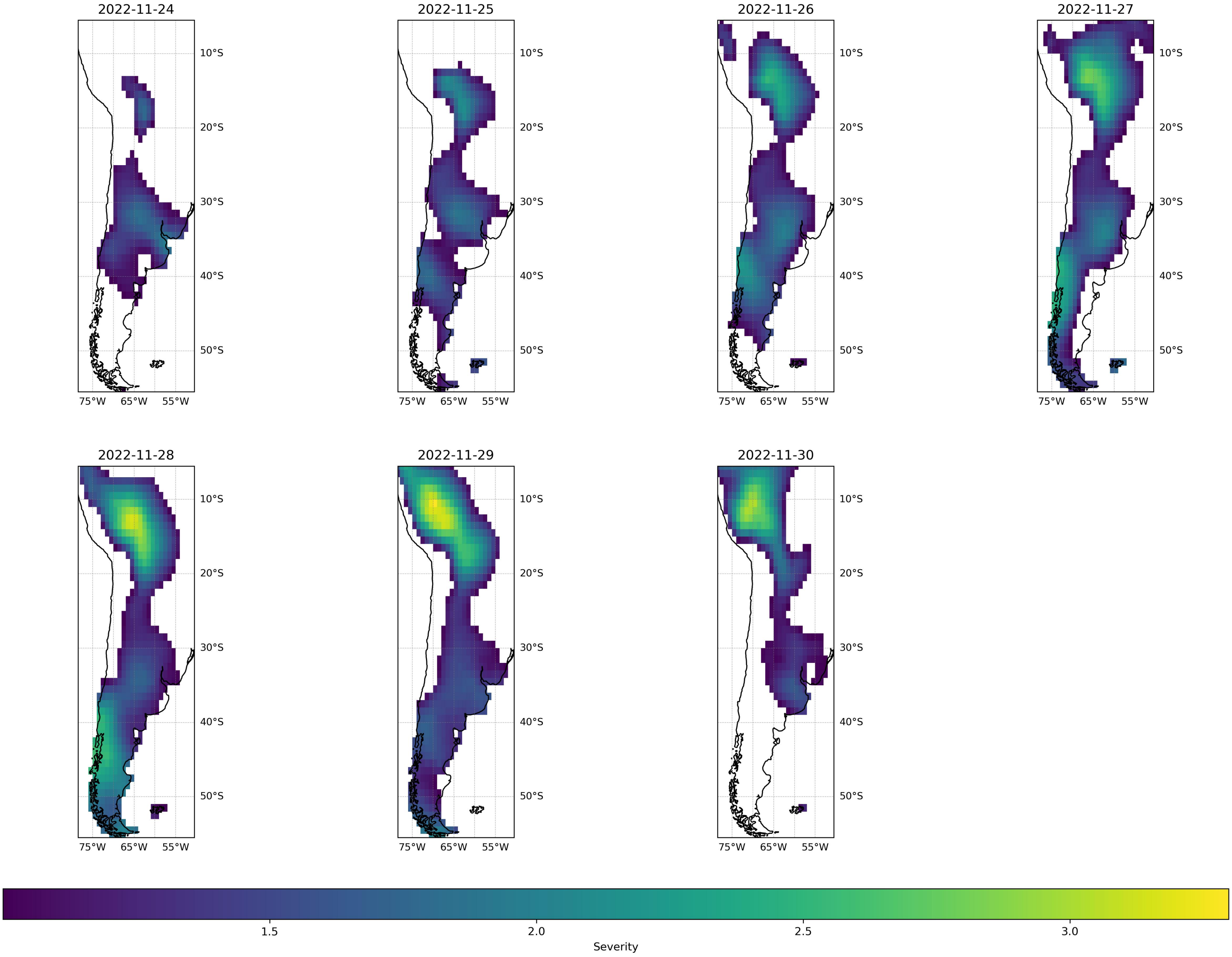

2022-11-24
2022-11-25
2022-11-26
2022-11-27
2022-11-28
2022-11-29
2022-11-30
10°S
20°S
30°S
40°S
50°S
75°W
65°W
55°W
1.5
2.0
2.5
3.0
Severity

Supplementary Figure 486: Reference Event Mapping for South America based on ERA5 - Spatio-temporal evolution of local heat-affected grid boxes during reference heatwave in South America based on data in ERA5. Colors and color-bar indicate the local severity values for each day of the event. The respective date of the heatwave day is indicated above each sub-figure and x- and y-axis show the longitude and latitude coordinates.

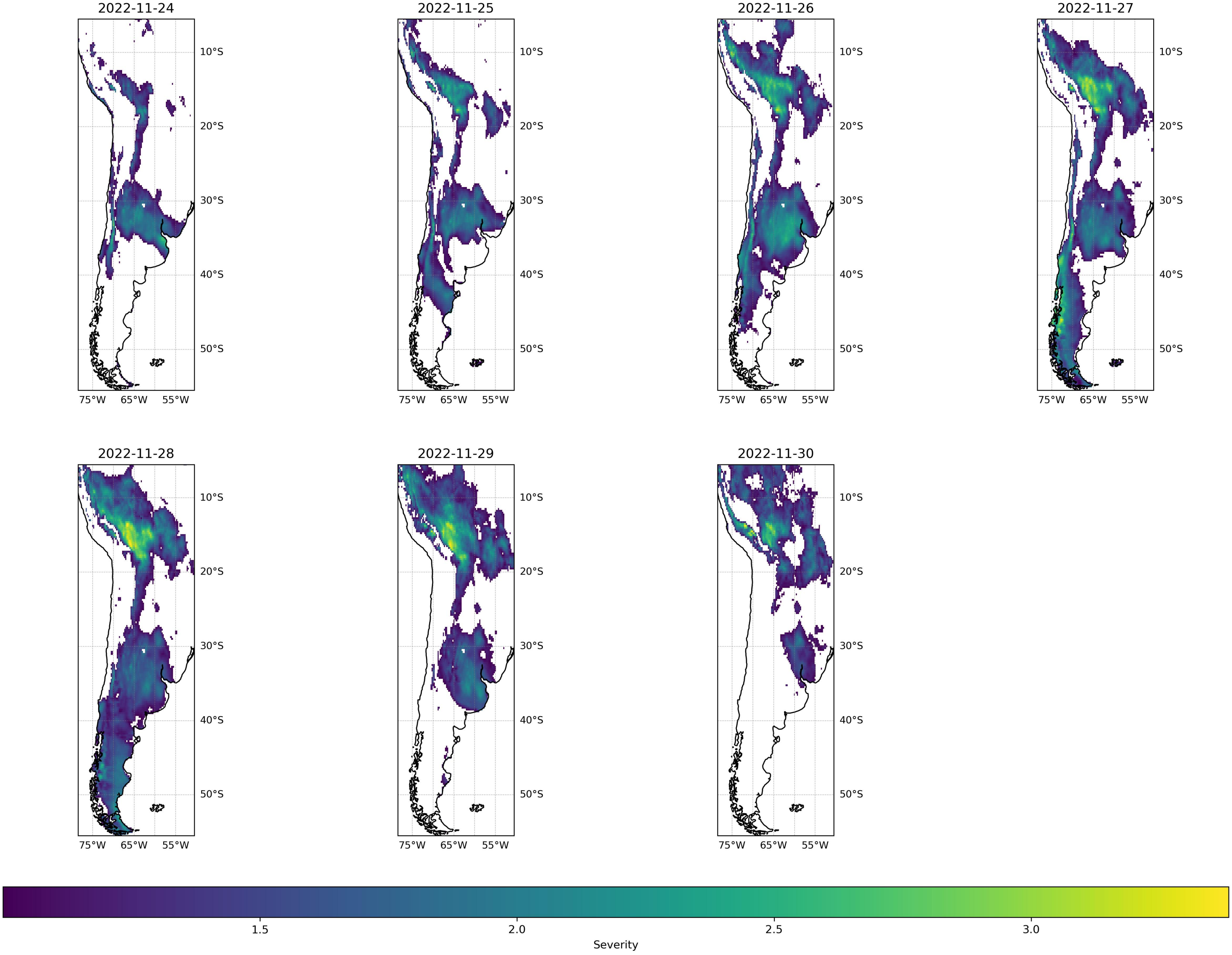
2022-11-24
2022-11-25
2022-11-26
2022-11-27
2022-11-28
2022-11-29
2022-11-30
10°S
20°S
30°S
40°S
50°S
75°W
65°W
55°W
1.5
2.0
2.5
3.0
Severity

Supplementary Figure 487: Reference Event Mapping for Northern Southeast Asia based on Berkeley Earth - Spatio-temporal evolution of local heat-affected grid boxes during reference heatwave in Northern Southeast Asia based on data in Berkeley Earth. Colors and color-bar indicate the local severity values for each day of the event. The respective date of the heatwave day is indicated above each sub-figure and x- and y-axis show the longitude and latitude coordinates.

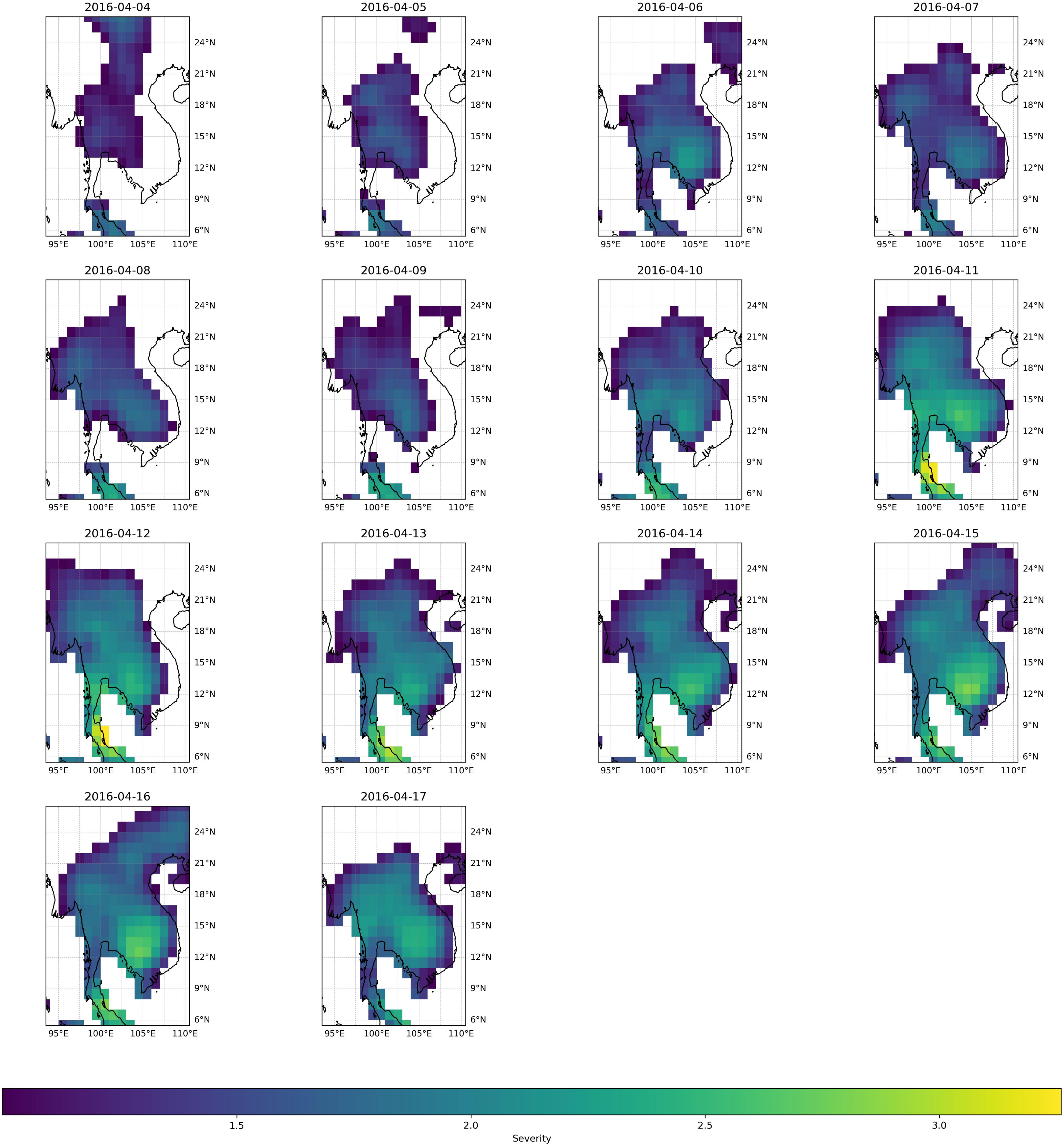
2016-04-04
2016-04-05
2016-04-06
2016-04-07
2016-04-08
2016-04-09
2016-04-10
2016-04-11
2016-04-12
2016-04-13
2016-04-14
2016-04-15
2016-04-16
2016-04-17
24°N
21°N
18°N
15°N
12°N
9°N
6°N
95°E
100°E
105°E
110°E
1.5
2.0
2.5
3.0
Severity

Supplementary Figure 488: Reference Event Mapping for Northern Southeast Asia based on ERA5 - Spatio-temporal evolution of local heat-affected grid boxes during reference heatwave in Northern Southeast Asia based on data in ERA5. Colors and color-bar indicate the local severity values for each day of the event. The respective date of the heatwave day is indicated above each sub-figure and x- and y-axis show the longitude and latitude coordinates.

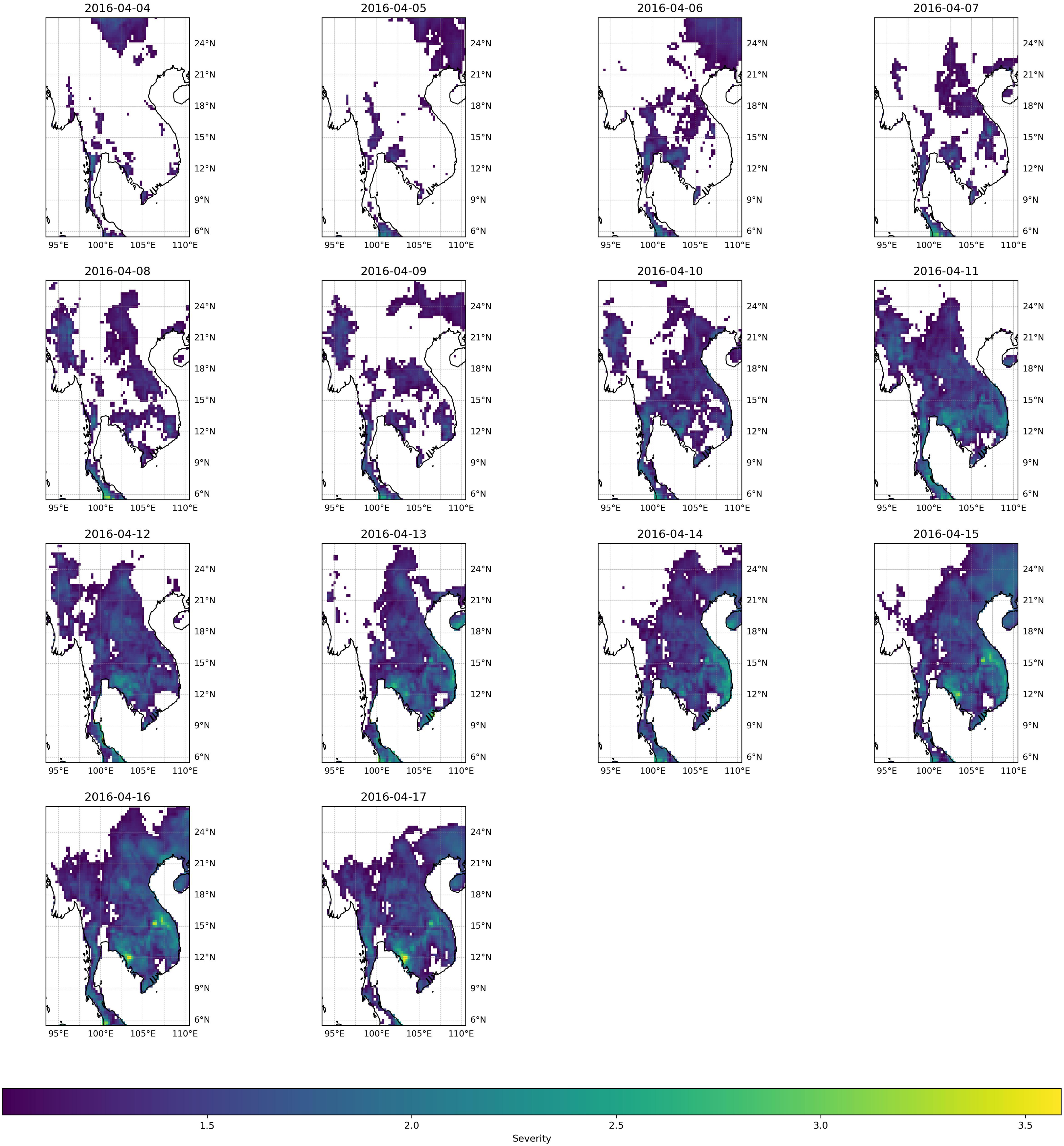
2016-04-04
2016-04-05
2016-04-06
2016-04-07
2016-04-08
2016-04-09
2016-04-10
2016-04-11
2016-04-12
2016-04-13
2016-04-14
2016-04-15
2016-04-16
2016-04-17
24°N
21°N
18°N
15°N
12°N
9°N
6°N
95°E
100°E
105°E
110°E
1.5
2.0
2.5
3.0
3.5
Severity

Supplementary Figure 489: Reference Event Mapping for Southern Africa based on Berkeley Earth - Spatio-temporal evolution of local heat-affected grid boxes during reference heatwave in Southern Africa based on data in Berkeley Earth. Colors and color-bar indicate the local severity values for each day of the event. The respective date of the heatwave day is indicated above each sub-figure and x- and y-axis show the longitude and latitude coordinates.

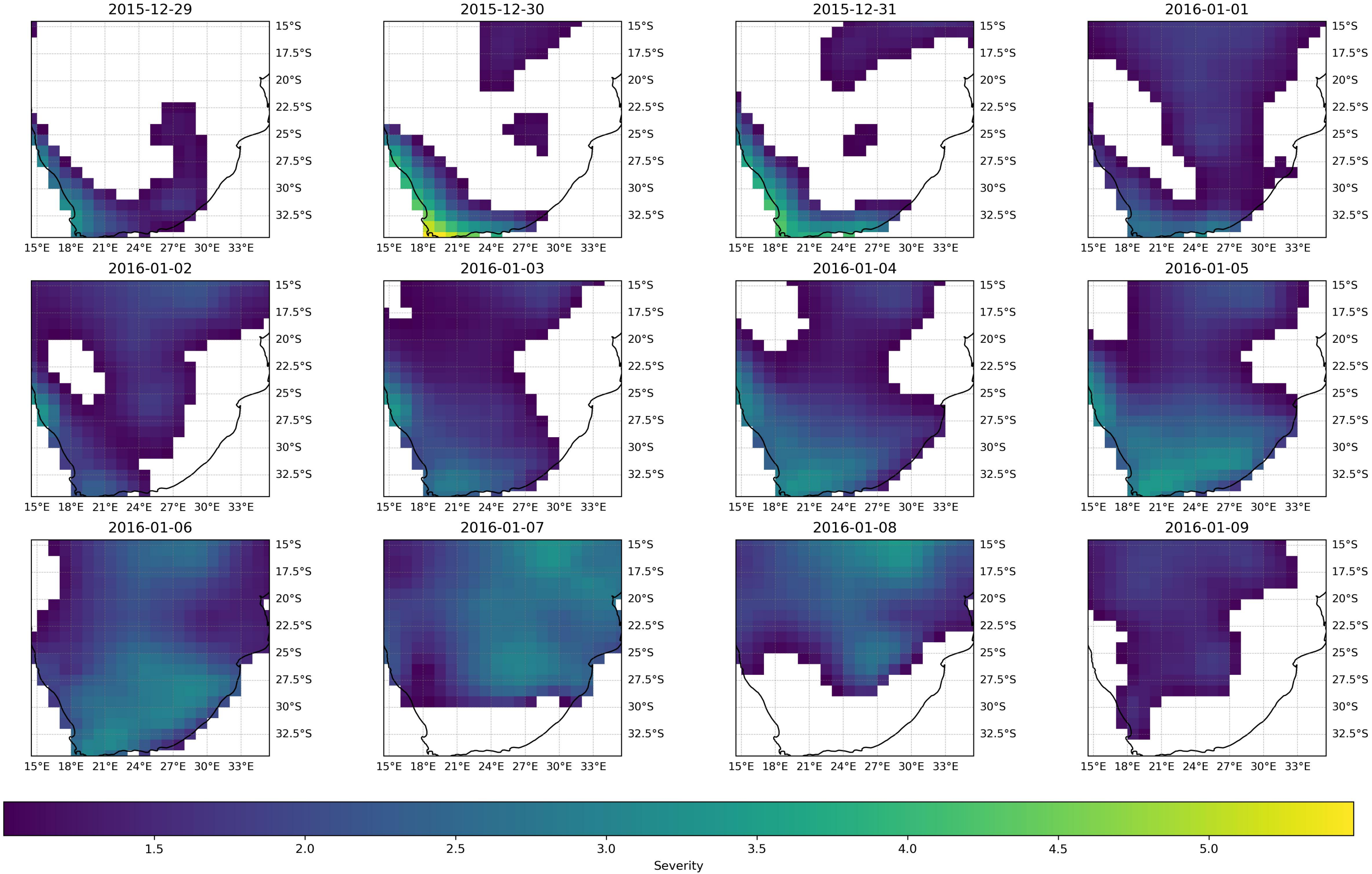
2015-12-29
2015-12-30
2015-12-31
2016-01-01
2016-01-02
2016-01-03
2016-01-04
2016-01-05
2016-01-06
2016-01-07
2016-01-08
2016-01-09
15°S
17.5°S
20°S
22.5°S
25°S
27.5°S
30°S
32.5°S
15°E
18°E
21°E
24°E
27°E
30°E
33°E
1.5
2.0
2.5
3.0
3.5
4.0
4.5
5.0
Severity

Supplementary Figure 490: Reference Event Mapping for Southern Africa based on ERA5 - Spatio-temporal evolution of local heat-affected grid boxes during reference heatwave in Southern Africa based on data in ERA5. Colors and color-bar indicate the local severity values for each day of the event. The respective date of the heatwave day is indicated above each sub-figure and x- and y-axis show the longitude and latitude coordinates.

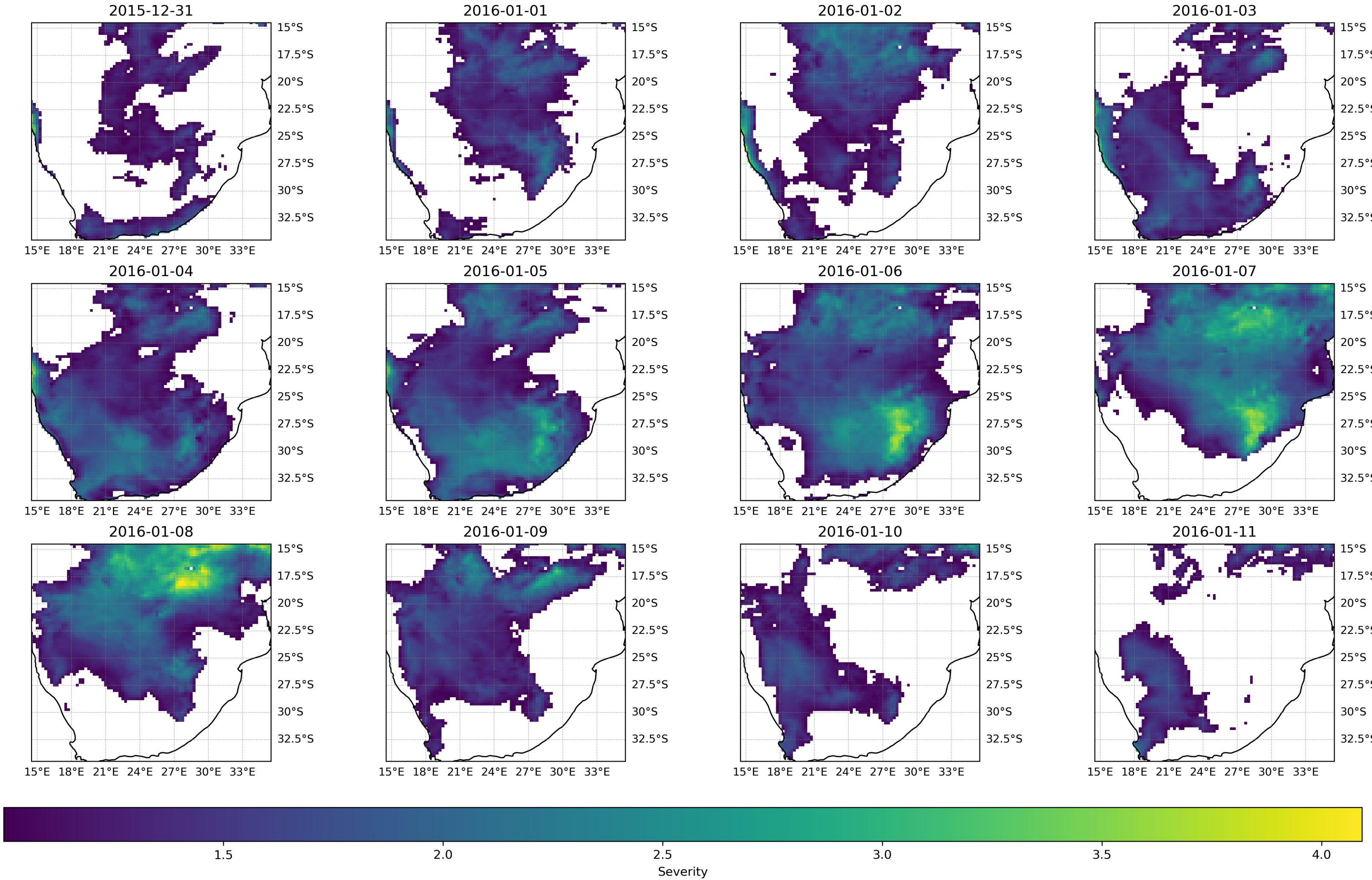
2015-12-31
2016-01-01
2016-01-02
2016-01-03
2016-01-04
2016-01-05
2016-01-06
2016-01-07
2016-01-08
2016-01-09
2016-01-10
2016-01-11
15°S
17.5°S
20°S
22.5°S
25°S
27.5°S
30°S
32.5°S
15°E
18°E
21°E
24°E
27°E
30°E
33°E
1.5
2.0
2.5
3.0
3.5
4.0
Severity

Supplementary Figure 491: Reference Event Mapping for Eastern USA based on Berkeley Earth - Spatio-temporal evolution of local heat-affected grid boxes during reference heatwave in Eastern USA based on data in Berkeley Earth. Colors and color-bar indicate the local severity values for each day of the event. The respective date of the heatwave day is indicated above each sub-figure and x- and y-axis show the longitude and latitude coordinates.

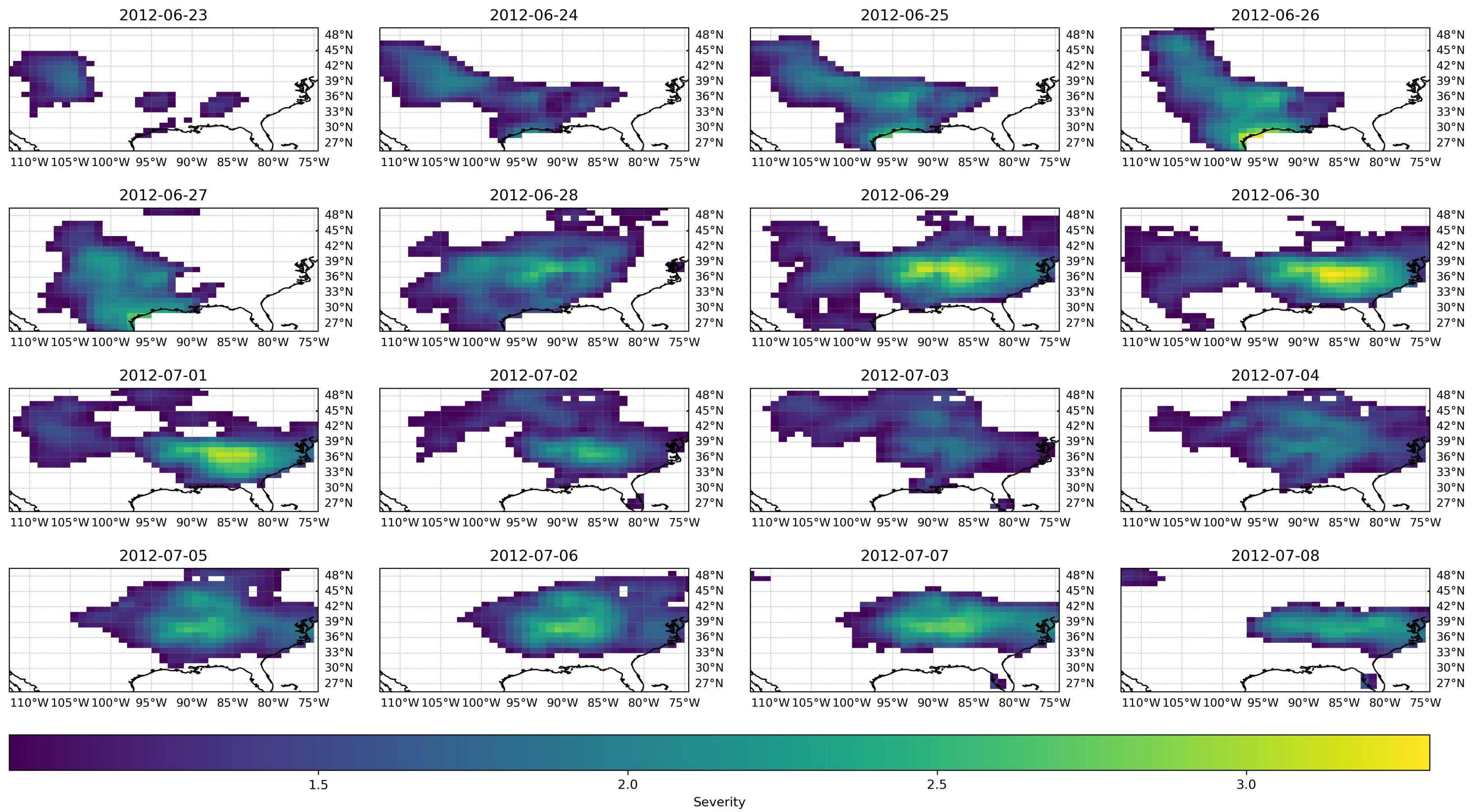
2012-06-23
2012-06-24
2012-06-25
2012-06-26
2012-06-27
2012-06-28
2012-06-29
2012-06-30
2012-07-01
2012-07-02
2012-07-03
2012-07-04
2012-07-05
2012-07-06
2012-07-07
2012-07-08
48°N
45°N
42°N
39°N
36°N
33°N
30°N
27°N
110°W 105°W 100°W 95°W 90°W 85°W 80°W 75°W
1.5
2.0
2.5
3.0
Severity

Supplementary Figure 492: Reference Event Mapping for Eastern USA based on ERA5 - Spatio-temporal evolution of local heat-affected grid boxes during reference heatwave in Eastern USA based on data in ERA5. Colors and color-bar indicate the local severity values for each day of the event. The respective date of the heatwave day is indicated above each sub-figure and x- and y-axis show the longitude and latitude coordinates.

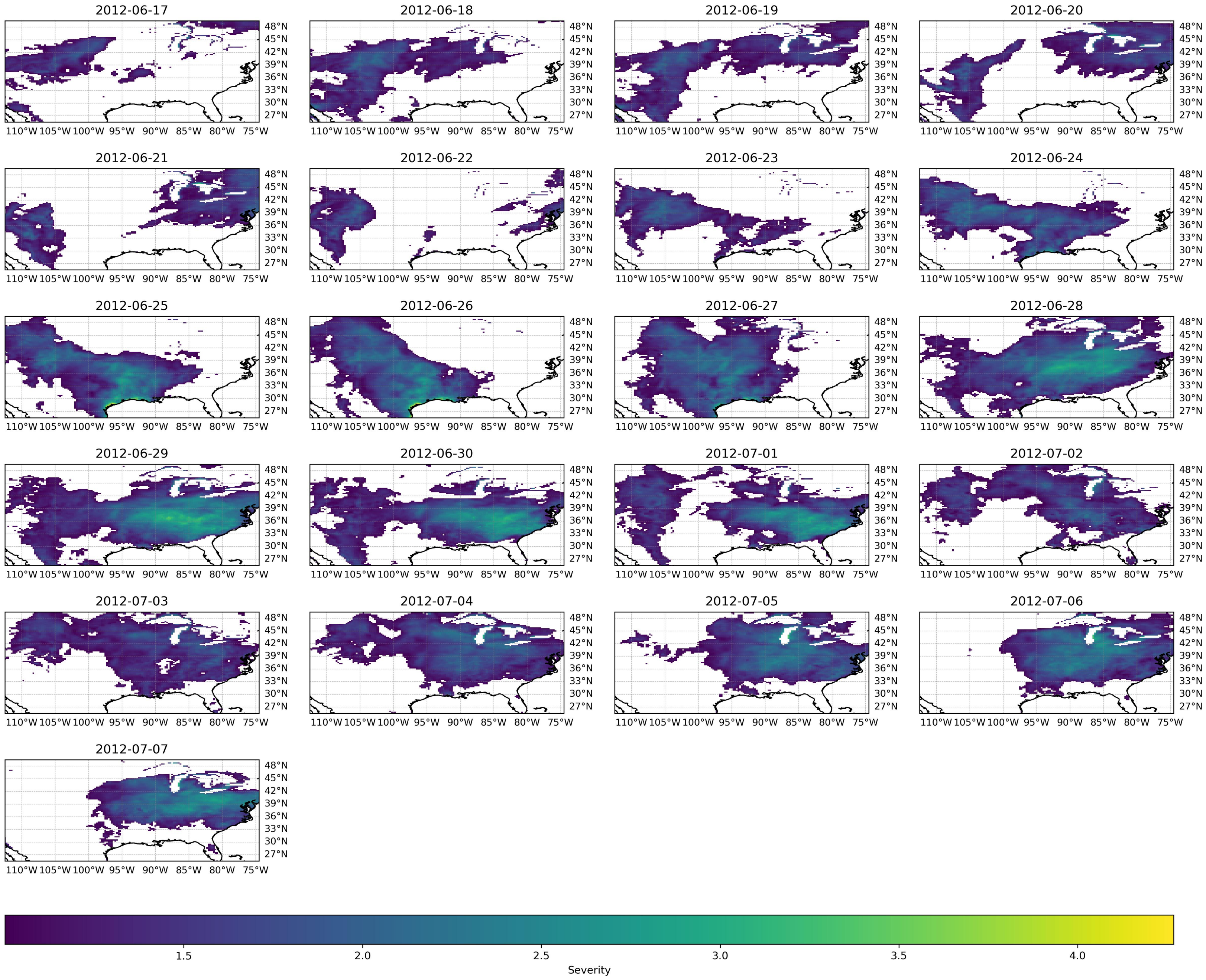
2012-06-17
2012-06-18
2012-06-19
2012-06-20
2012-06-21
2012-06-22
2012-06-23
2012-06-24
2012-06-25
2012-06-26
2012-06-27
2012-06-28
2012-06-29
2012-06-30
2012-07-01
2012-07-02
2012-07-03
2012-07-04
2012-07-05
2012-07-06
2012-07-07
48°N
45°N
42°N
39°N
36°N
33°N
30°N
27°N
110°W
105°W
100°W
95°W
90°W
85°W
80°W
75°W
1.5
2.0
2.5
3.0
3.5
4.0
Severity

Supplementary Figure 493: Temperature extreme frequency in a single ERA5 grid cell. Shown are 30 annual cycles of maximum temperature (black lines), as well as the 90th percentile threshold using a 15-day running window (red line) and a subsequent 31-day smoothing (blue line). Daily exceedances of the first threshold are shown as red shading. The bottom part of the plot shows the monthly averaged exceedance frequency for both thresholds. The figure is adapted from figure 1b in Brunner and Voigt (2024), for more details see their methods.

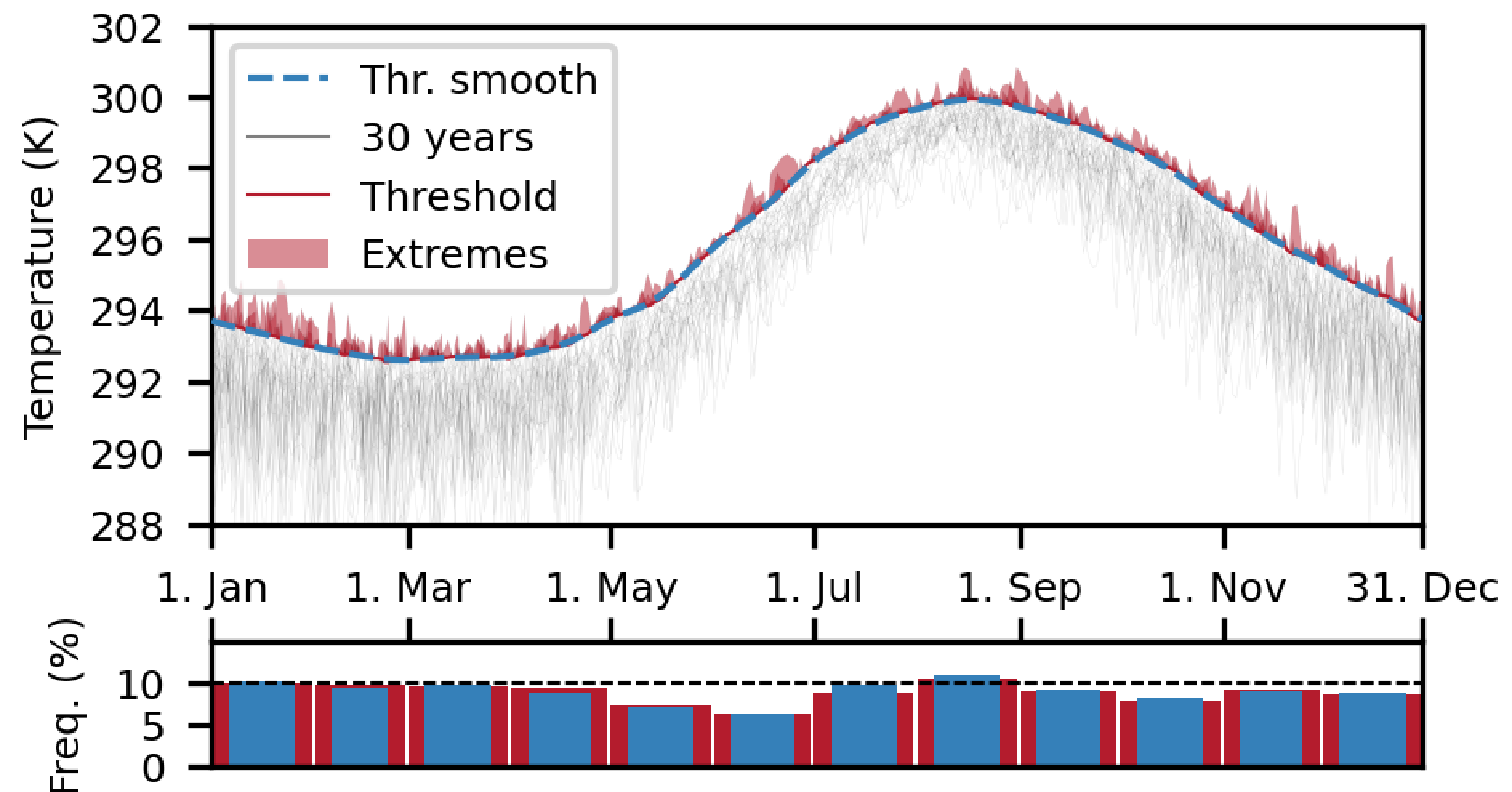

Thr. smooth
30 years
Threshold
Extremes
Temperature (K)
302
300
298
296
294
292
290
288
1. Jan
1. Mar
1. May
1. Jul
1. Sep
1. Nov
31. Dec
Freq. (%)
10
5
0

Supplementary Figure 494: Grid Resolution Sensitivity Test for EC-Earth3-Veg SSP1-2.6 - Figure shows the heatwave area distributions for each region per grid resolution used for regridding, i.e. r320x160, r256x128, r128x64. This figure shows the results for SSP1-2.6 in EC-Earth3-Veg.

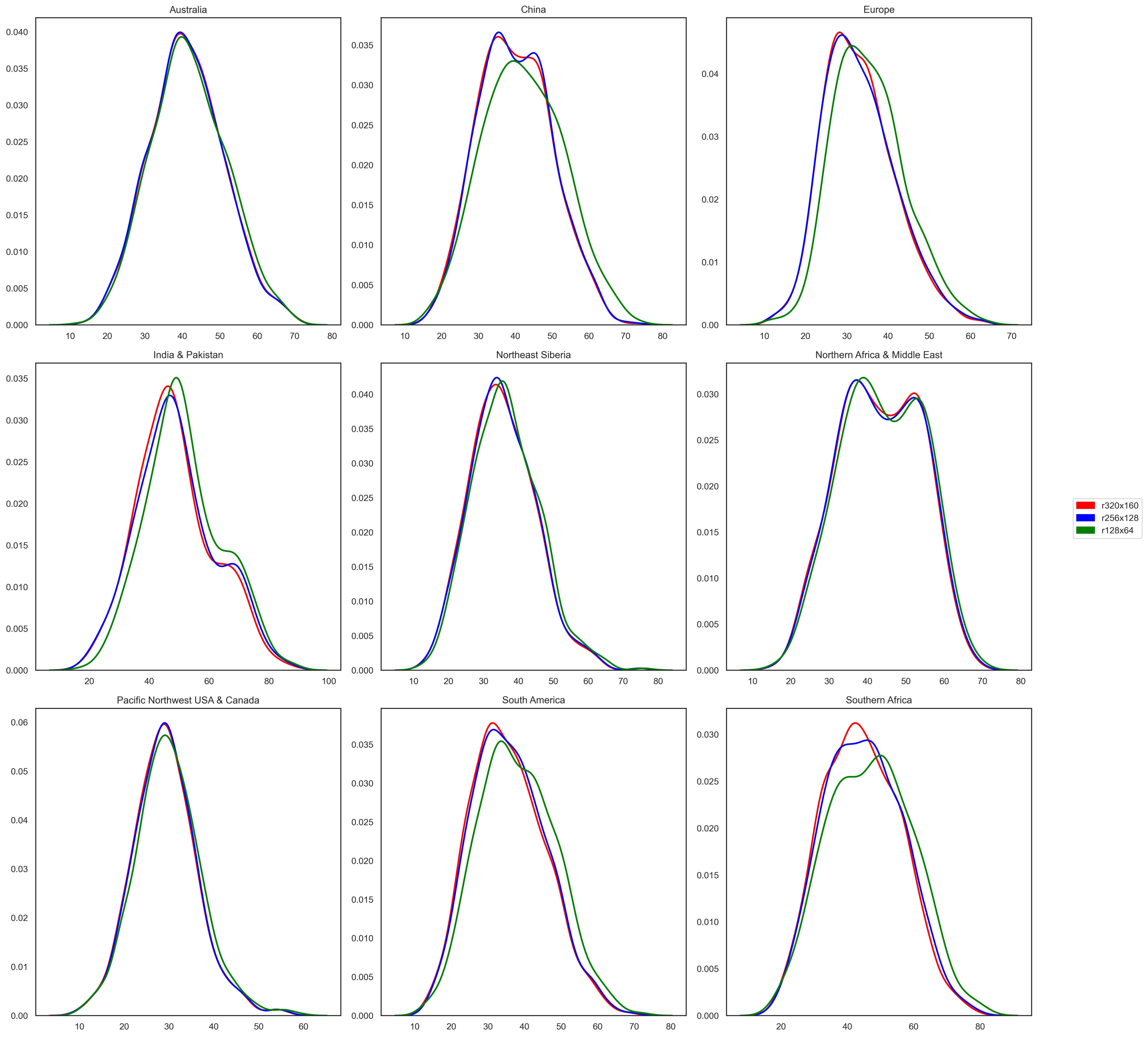

Australia
China
Europe
India & Pakistan
Northeast Siberia
Northern Africa & Middle East
Pacific Northwest USA & Canada
South America
Southern Africa
r320x160
r256x128
r128x64

Supplementary Figure 495: Grid Resolution Sensitivity Test for EC-Earth3-Veg SSP3-7.0 - Figure shows the heatwave area distributions for each region per grid resolution used for regridding, i.e. r320x160, r256x128, r128x64. This figure shows the results for SSP3-7.0 in EC-Earth3-Veg.

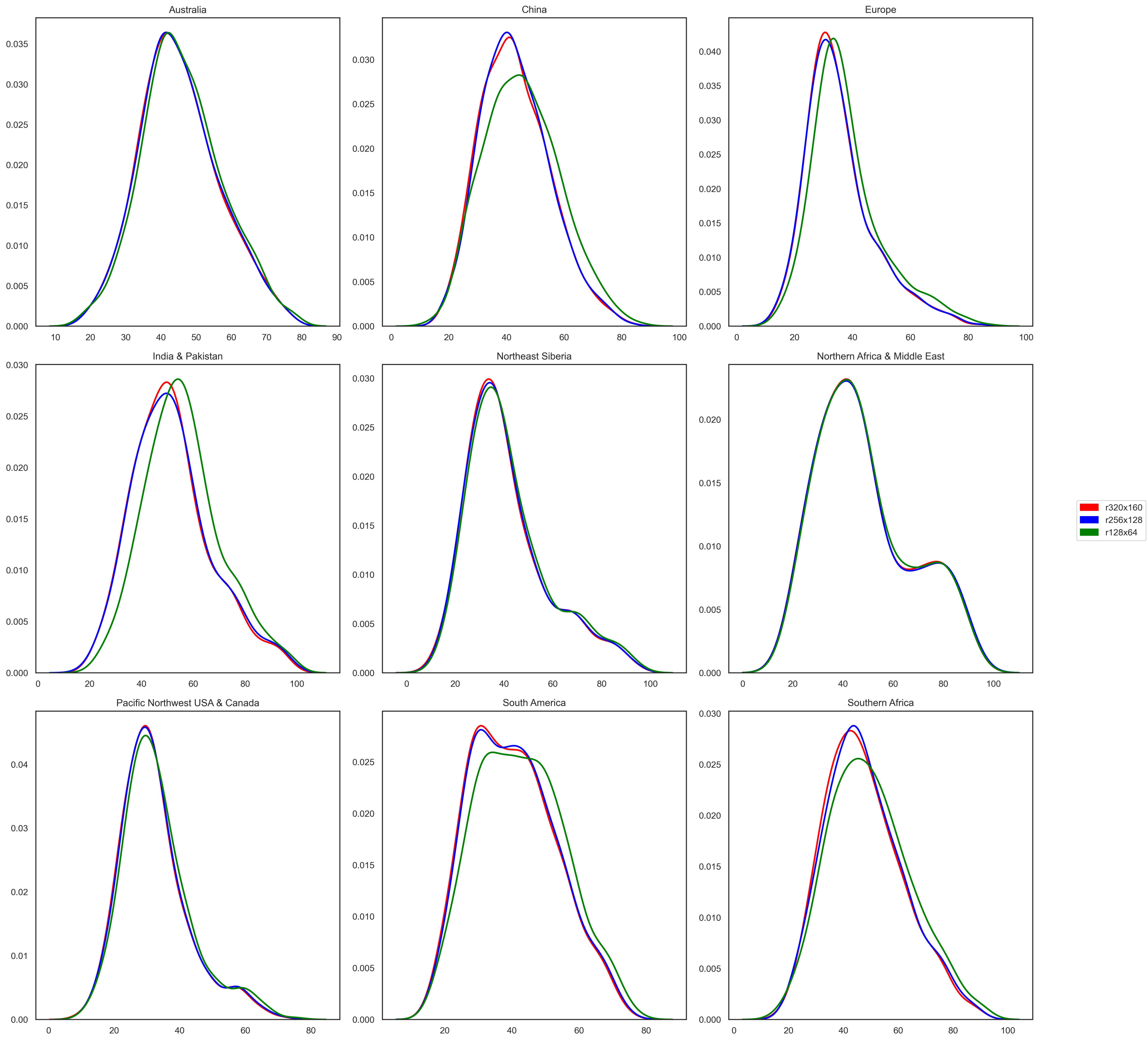

Australia
China
Europe
India & Pakistan
Northeast Siberia
Northern Africa & Middle East
Pacific Northwest USA & Canada
South America
Southern Africa
r320x160
r256x128
r128x64
0.000
0.005
0.010
0.015
0.020
0.025
0.030
0.035
0.040
0.00
0.01
0.02
0.03
0.04
0
10
20
30
40
50
60
70
80
90
100

Supplementary Figure 496: Grid Resolution Sensitivity Test for MIROC-ES2L SSP1-2.6 - Figure shows the heatwave area distributions for each region per grid resolution used for regridding, i.e. r320x160, r256x128, r128x64. This figure shows the results for SSP1-2.6 in MIROC-ES2L.

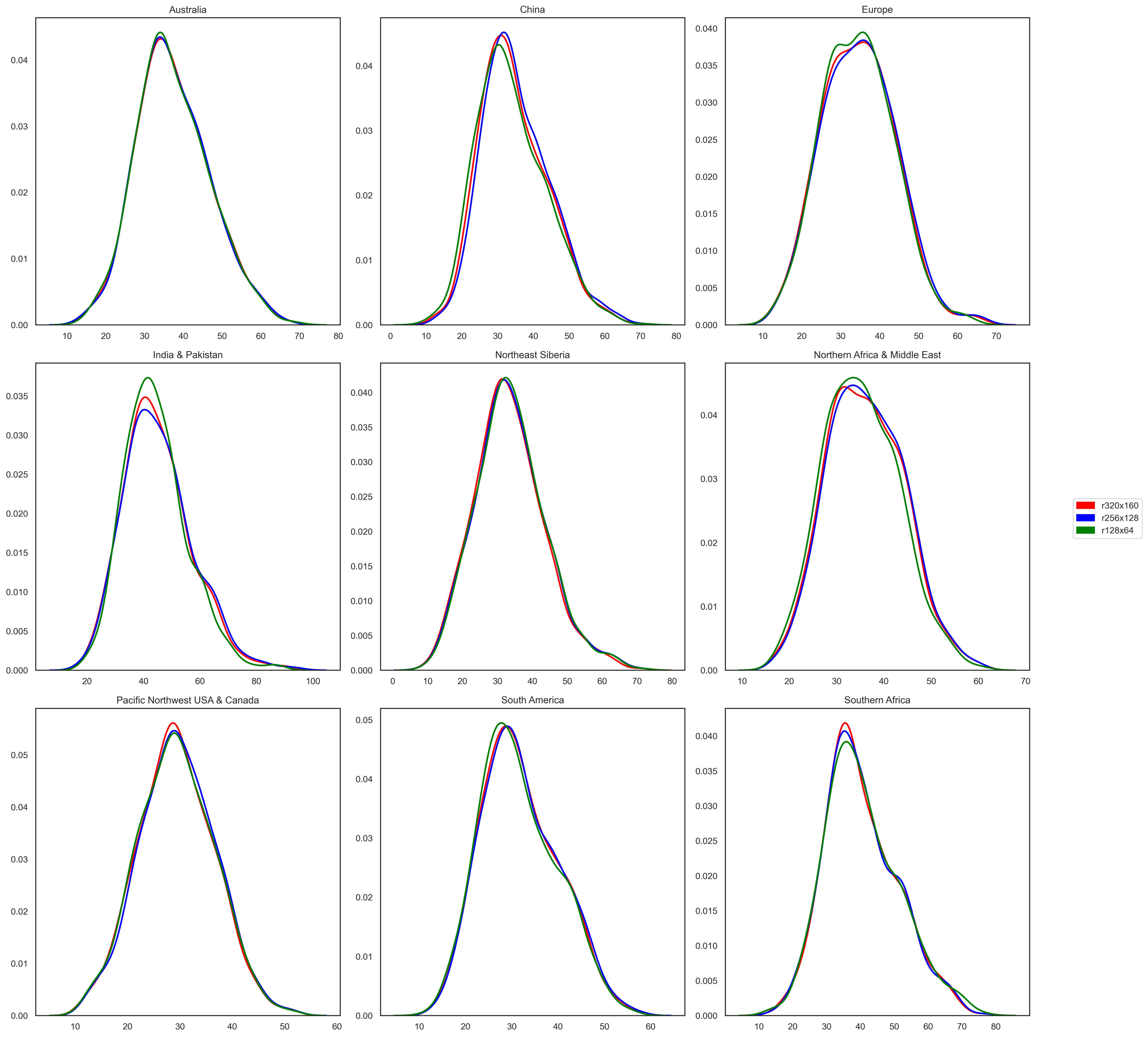

Australia
China
Europe
India & Pakistan
Northeast Siberia
Northern Africa & Middle East
Pacific Northwest USA & Canada
South America
Southern Africa
r320x160
r256x128
r128x64

Supplementary Figure 497: Grid Resolution Sensitivity Test for MIROC-ES2L SSP3-7.0 - Figure shows the heatwave area distributions for each region per grid resolution used for regridding, i.e. r320x160, r256x128, r128x64. This figure shows the results for SSP3-7.0 in MIROC-ES2L.

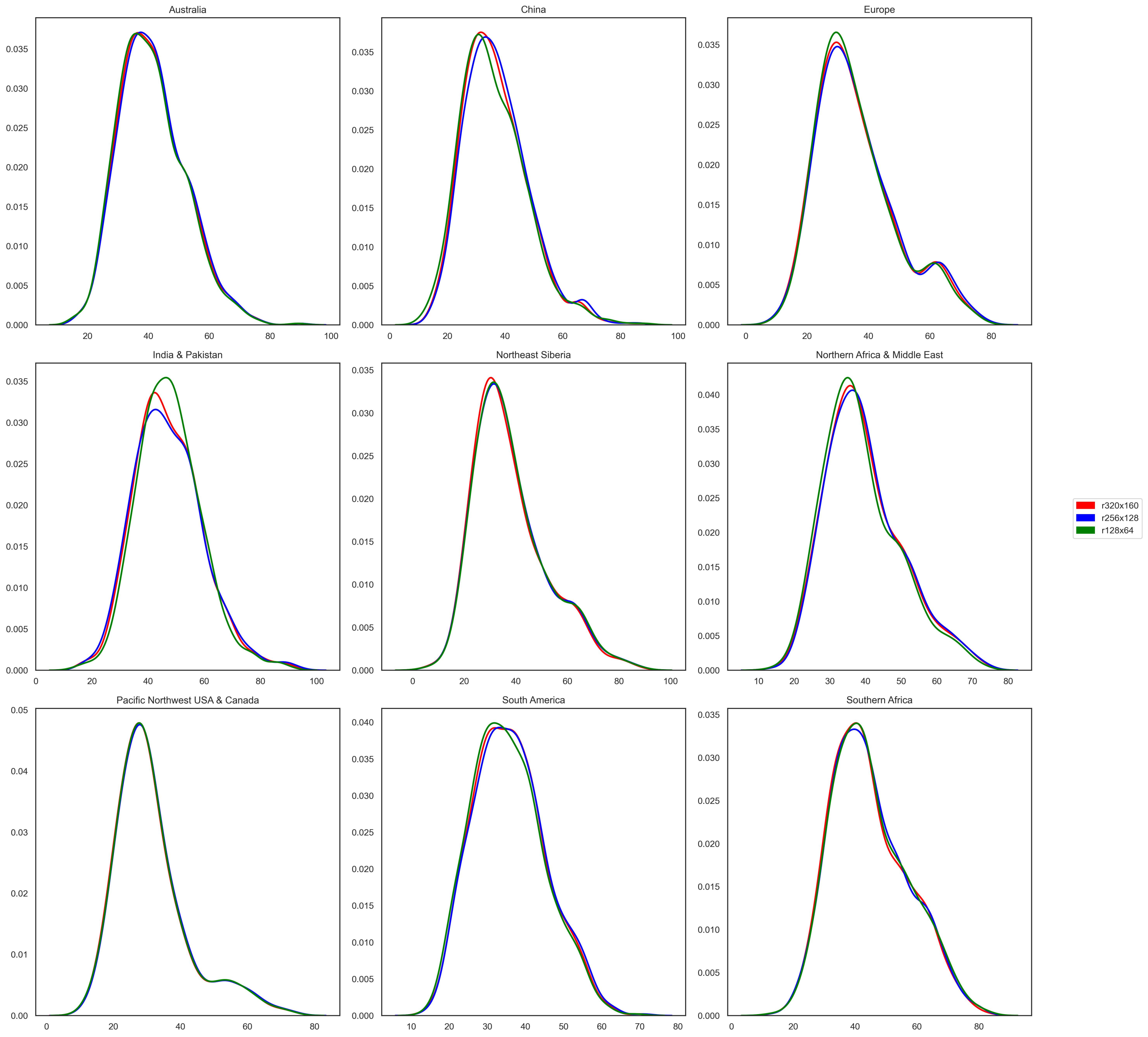
Australia
China
Europe
India & Pakistan
Northeast Siberia
Northern Africa & Middle East
Pacific Northwest USA & Canada
South America
Southern Africa
r320x160
r256x128
r128x64
