# Supplementary material for: Recent extreme heatwaves are no match for future events
Source: Nat Commun. 2026 Nov 5;17:11456. doi: 10.1038/s41467-026-76809-5 (PMC13740290; doi:10.1038/s41467-026-76809-5)
Supplement: Supplementary file 2 [file 41467_2026_76809_MOESM2_ESM.pdf]

Description of Additional Supplementary Files

Supplementary Data 1: Region References - Table lists reports that were used as a first indicator where and when a reference heatwave occurred. This list includes all 22 reference heatwaves analyzed for this study.

Supplementary Data 2: Reference Heatwave Characteristics - Table lists dates, regional boundaries, and all six heatwave characteristics of all 22 reference heatwaves ananlyzed for this study. The table also includes indicators as in Table 2 of the paper: Symbols indicating characteristics among the top 3.0% from 1961 to date of reference heatwave (`), top 3.0% in entire reference dataset from 1961 to 2023 (-i-), peak value from 1961 to date of reference heatwave (*) or peak value in entire reference dataset from 1961 to 2023 (**). Heatwave characteristics are based on Berkeley_Earth.

Supplementary Data 3: Region References - Table lists reports that were used as a first indicator where and when a reference heatwave occurred. This list includes all 22 reference heatwaves analyzed for this study.

Supplementary Data 4: Reference Heatwave Characteristics - Table lists dates, regional boundaries, and all six heatwave characteristics of all 22 reference heatwaves ananlyzed for this study. The table also includes indicators as in Table 2 of the paper: Symbols indicating characteristics among the top 3.0% from 1961 to date of reference heatwave (`), top 3.0% in entire reference dataset from 1961 to 2023 (-i-), peak value from 1961 to date of reference heatwave (*) or peak value in entire reference dataset from 1961 to 2023 (**). Heatwave characteristics are based on ERA5.

Supplementary Data 5: Reference Event Characteristic Ranking (Tx90p) - Table lists heatwave characteristics of all reference events based on both Berkeley Earth and ERA5. The Table shows the region name, dates of the reference event, name of the heatwave characteristic ('Parameter_Name'), the reference value of the respective characteristic ('Parameter'), the respective reference data set ('Reanalysis'), and the exceedance percentages for the respective time interval: (1) 'time_window_to_ref' indicates the time window from the start of the climatological baseline (1961) to the start date of the reference heatwave. (2) 'entire_time_window' indicates the entire time window from 1961 to 2023. (3) 'gt' means greater than and 'gte' greater than or equal, which were used to indicate if a specific characteristic is a peak value ('gt' is 0) or among the top X% ('gte').

Supplementary Data 6: Reference Event Characteristic Ranking (Tx95.0p) - Table lists heatwave characteristics of all reference events based on both Berkeley Earth and ERA5. The Table shows the region name, dates of the reference event, name of the heatwave characteristic ('Parameter_Name'), the reference value of the respective characteristic ('Parameter'), the respective reference data set ('Reanalysis'), and the exceedance percentages for the respective time interval: (1) 'time_window_to_ref' indicates the time window from the start of the climatological baseline (1961) to the start date of the reference heatwave. (2) 'entire_time_window' indicates the entire time window from 1961 to 2023. (3) 'gt' means greater than and 'gte' greater than or equal, which were used to indicate if a specific characteristic is a peak value ('gt' is 0) or among the top X% ('gte').

Supplementary Data 7: Reference Event Characteristic Ranking (Tx97.5p) - Table lists heatwave characteristics of all reference events based on both Berkeley Earth and ERA5. The Table shows the region name, dates of the reference event, name of the heatwave characteristic ('Parameter_Name'), the reference value of the respective characteristic ('Parameter'), the respective reference data set ('Reanalysis'), and the exceedance percentages for the respective time interval: (1) 'time_window_to_ref' indicates the time window from the start of the climatological baseline (1961) to the start date of the reference heatwave. (2) 'entire_time_window' indicates the entire time window from 1961 to 2023. (3) 'gt' means greater than and 'gte' greater than or equal, which were used to indicate if a specific characteristic is a peak value ('gt' is 0) or among the top X% ('gte').

Supplementary Data 8: Reference Event Characteristic Ranking (Tx99.0p) - Table lists heatwave characteristics of all reference events based on both Berkeley Earth and ERA5. The Table shows the region name, dates of the reference event, name of the heatwave characteristic ('Parameter_Name'), the reference value of the respective characteristic ('Parameter'), the respective reference data set ('Reanalysis'), and the exceedance percentages for the respective time interval: (1) 'time_window_to_ref' indicates the time window from the start of the climatological baseline (1961) to the start date of the reference heatwave. (2) 'entire_time_window' indicates the entire time window from 1961 to 2023. (3) 'gt' means greater than and 'gte' greater than or equal, which were used to indicate if a specific characteristic is a peak value ('gt' is 0) or among the top X% ('gte').

Supplementary Data 9: Heatwave Characteristic Exceedange Percentages (Tx90p) - Table lists the exceedance percentages of the respective heatwave characteristic for CMIP6 multi-model ensemble (MME) SSP1-2.6 for entire dataset from 2020 to 2100. The Table shows the region name, dates of the reference event, name of the heatwave characteristic ('Parameter_Name'), the reference value of the respective characteristic ('Parameter'), the respective reference data set ('Reanalysis'), the total number of heatwaves in the respective sample ('total_HW_num'), the number of heatwaves in the sample exceeding the reference value ('Parameter_Exceedance_Number'), and the exceedance percentage ('Parameter_Exceedance_Percentage'), the data subset type, the CMIP generation, and the repsective experiment.

Supplementary Data 10: Heatwave Characteristic Exceedange Percentages (Tx90p) - Table lists the exceedance percentages of the respective heatwave characteristic for CMIP6 multi-model ensemble (MME) SSP3-7.0 for entire dataset from 2020 to 2100. The Table shows the region name, dates of the reference event, name of the heatwave characteristic ('Parameter_Name'), the reference value of the respective characteristic ('Parameter'), the respective reference data set ('Reanalysis'), the total number of heatwaves in the respective sample ('total_HW_num'), the number of heatwaves in the sample exceeding the reference value ('Parameter_Exceedance_Number'), and the exceedance percentage ('Parameter_Exceedance_Percentage'), the data subset type, the CMIP generation, and the repsective experiment.

Supplementary Data 11: Distribution of Projected Heatwave Characteristics (Tx90p) - Table lists distribution of the projected heatwave characteristic for CMIP6 multi-model ensemble (MME) SSP1-2.6 for entire dataset from 2020 to 2100. The table shows the projected value of the respective heatwave characteristic for each integer percentile in the respective sample.

Supplementary Data 12: Distribution of Projected Heatwave Characteristics (Tx90p) - Table lists distribution of the projected heatwave characteristic for CMIP6 multi-model ensemble (MME) SSP3-7.0 for entire dataset from 2020 to 2100. The table shows the projected value of the respective heatwave characteristic for each integer percentile in the respective sample.

Supplementary Data 13: Heatwave Characteristic Exceedange Percentages (Tx99.0p) - Table lists the exceedance percentages of the respective heatwave characteristic for CMIP6 multi-model ensemble (MME) SSP1-2.6 for entire dataset from 2020 to 2100. The Table shows the region name, dates of the reference event, name of the heatwave characteristic ('Parameter_Name'), the reference value of the respective characteristic ('Parameter'), the respective reference data set ('Reanalysis'), the total number of heatwaves in the respective sample ('total_HW_num'), the number of heatwaves in the sample exceeding the reference value ('Parameter_Exceedance_Number'), and the exceedance percentage ('Parameter_Exceedance_Percentage'), the data subset type, the CMIP generation, and the repsective experiment.

Supplementary Data 14: Heatwave Characteristic Exceedange Percentages (Tx99.0p) - Table lists the exceedance percentages of the respective heatwave characteristic for CMIP6 multi-model ensemble (MME) SSP3-7.0 for entire dataset from 2020 to 2100. The Table shows the region name, dates of the reference event, name of the heatwave characteristic ('Parameter_Name'), the reference value of the respective characteristic ('Parameter'), the respective reference data set ('Reanalysis'), the total number of heatwaves in the respective sample ('total_HW_num'), the number of heatwaves in the sample exceeding the reference value ('Parameter_Exceedance_Number'), and the exceedance percentage ('Parameter_Exceedance_Percentage'), the data subset type, the CMIP generation, and the repsective experiment.

Supplementary Data 15: Heatwave Characteristic Exceedange Percentages (Tx90p) - Table lists the exceedance percentages of the respective heatwave characteristic for CMIP6 multi-model ensemble (MME) SSP1-2.6 for entire dataset from 2020 to 2100. The Table shows the region name, dates of the reference event, name of the heatwave characteristic ('Parameter_Name'), the reference value of the respective characteristic ('Parameter'), the respective reference data set ('Reanalysis'), the total number of heatwaves in the respective sample ('total_HW_num'), the number of heatwaves in the sample exceeding the reference value ('Parameter_Exceedance_Number'), and the exceedance percentage ('Parameter_Exceedance_Percentage'), the data subset type, the CMIP generation, and the repsective experiment.

Supplementary Data 16: Heatwave Characteristic Exceedange Percentages (Tx90p) - Table lists the exceedance percentages of the respective heatwave characteristic for CMIP6 multi-model ensemble (MME) SSP3-7.0 for entire dataset from 2020 to 2100. The Table shows the region name, dates of the reference event, name of the heatwave characteristic ('Parameter_Name'), the reference value of the respective characteristic ('Parameter'), the respective reference data set ('Reanalysis'), the total number of heatwaves in the respective sample ('total_HW_num'), the number of heatwaves in the sample exceeding the reference value ('Parameter_Exceedance_Number'), and the exceedance percentage ('Parameter_Exceedance_Percentage'), the data subset type, the CMIP generation, and the repsective experiment.

Supplementary Data 17: Distribution of Projected Heatwave Characteristics (Tx90p) - Table lists distribution of the projected heatwave characteristic for CMIP6 multi-model ensemble (MME) SSP1-2.6 for entire dataset from 2020 to 2100. The table shows the projected value of the respective heatwave characteristic for each integer percentile in the respective sample.

Supplementary Data 18: Distribution of Projected Heatwave Characteristics (Tx90p) - Table lists distribution of the projected heatwave characteristic for CMIP6 multi-model ensemble (MME) SSP3-7.0 for entire dataset from 2020 to 2100. The table shows the projected value of the respective heatwave characteristic for each integer percentile in the respective sample.

Supplementary Data 19: Heatwave Characteristic Exceedange Percentages (Tx90p) - Table lists the exceedance percentages of the respective heatwave characteristic for CMIP6 multi-model ensemble (MME) SSP1-2.6 for entire dataset from 2020 to 2100. The Table shows the region name, dates of the reference event, name of the heatwave characteristic ('Parameter_Name'), the reference value of the respective characteristic ('Parameter'), the respective reference data set ('Reanalysis'), the total number of heatwaves in the respective sample ('total_HW_num'), the number of heatwaves in the sample exceeding the reference value ('Parameter_Exceedance_Number'), and the exceedance percentage ('Parameter_Exceedance_Percentage'), the data subset type, the CMIP generation, and the repsective experiment.

Supplementary Data 20: Heatwave Characteristic Exceedange Percentages (Tx90p) - Table lists the exceedance percentages of the respective heatwave characteristic for CMIP6 multi-model ensemble (MME) SSP3-7.0 for entire dataset from 2020 to 2100. The Table shows the region name, dates of the reference event, name of the heatwave characteristic ('Parameter_Name'), the reference value of the respective characteristic ('Parameter'), the respective reference data set ('Reanalysis'), the total number of heatwaves in the respective sample ('total_HW_num'), the number of heatwaves in the sample exceeding the reference value ('Parameter_Exceedance_Number'), and the exceedance percentage ('Parameter_Exceedance_Percentage'), the data subset type, the CMIP generation, and the repsective experiment.

Supplementary Data 21: Heatwave Characteristic Exceedange Percentages (Tx90p) - Table lists the exceedance percentages of the respective heatwave characteristic for CMIP6 multi-model ensemble (MME) SSP1-2.6 for entire dataset from 2020 to 2100. The Table shows the region name, dates of the reference event, name of the heatwave characteristic ('Parameter_Name'), the reference value of the respective characteristic ('Parameter'), the respective reference data set ('Reanalysis'), the total number of heatwaves in the respective sample ('total_HW_num'), the number of heatwaves in the sample exceeding the reference value ('Parameter_Exceedance_Number'), and the exceedance percentage ('Parameter_Exceedance_Percentage'), the data subset type, the CMIP generation, and the repsective experiment.

Supplementary Data 22: Heatwave Characteristic Exceedange Percentages (Tx90p) - Table lists the exceedance percentages of the respective heatwave characteristic for CMIP6 multi-model ensemble (MME) SSP3-7.0 for entire dataset from 2020 to 2100. The Table shows the region name, dates of the reference event, name of the heatwave characteristic ('Parameter_Name'), the reference value of the respective characteristic ('Parameter'), the respective reference data set ('Reanalysis'), the total number of heatwaves in the respective sample ('total_HW_num'), the number of heatwaves in the sample exceeding the reference value

('Parameter_Exceedance_Number'), and the exceedance percentage ('Parameter_Exceedance_Percentage'), the data subset type, the CMIP generation, and the repsective experiment.

Supplementary Data 23: Heatwave Characteristic Exceedange Percentages (Tx90p) - Table lists the percentages of projected heatwaves where the projected heatwave characteristic is several times as high as the reference characteristic for CMIP6 multi-model ensemble (MME) SSP1-2.6 for entire dataset from 2020 to 2100. The Table shows the region name, dates of the reference event, name of the heatwave characteristic ('Parameter_Name'), the reference value of the respective characteristic ('Parameter'), the respective reference data set ('Reanalysis'), the total number of heatwaves in the respective sample ('total_HW_num'), the number of heatwaves in the sample exceeding the reference value ('Parameter_Exceedance_Number'), and the exceedance percentage ('Parameter_Exceedance_Percentage'), the data subset type, the CMIP generation, and the repsective experiment.

Supplementary Data 24: Heatwave Characteristic Exceedange Percentages (Tx90p) - Table lists the percentages of projected heatwaves where the projected heatwave characteristic is several times as high as the reference characteristic for CMIP6 multi-model ensemble (MME) SSP3-7.0 for entire dataset from 2020 to 2100. The Table shows the region name, dates of the reference event, name of the heatwave characteristic ('Parameter_Name'), the reference value of the respective characteristic ('Parameter'), the respective reference data set ('Reanalysis'), the total number of heatwaves in the respective sample ('total_HW_num'), the number of heatwaves in the sample exceeding the reference value ('Parameter_Exceedance_Number'), and the exceedance percentage ('Parameter_Exceedance_Percentage'), the data subset type, the CMIP generation, and the repsective experiment.

Supplementary Data 25: Heatwave Characteristic Exceedange Percentages (Tx90p) - Table lists the exceedance percentages of the respective heatwave characteristic for CMIP6 multi-model ensemble (MME) SSP5-8.5 for data subdivided by Global Warming Levels (GWLs). The Table shows the region name, dates of the reference event, name of the heatwave characteristic ('Parameter_Name'), the reference value of the respective characteristic ('Parameter'), the respective reference data set ('Reanalysis'), the total number of heatwaves in the respective sample ('total_HW_num'), the number of heatwaves in the sample exceeding the reference value ('Parameter_Exceedance_Number'), and the exceedance percentage ('Parameter_Exceedance_Percentage'), the data subset type, the CMIP generation, and the repsective experiment.

Supplementary Data 26: Heatwave Characteristic Exceedange Percentages (Tx90p) - Table lists the shifts in exceedance percentages of the respective heatwave characteristic per data subdivided by Global Warming Levels (GWLs) for CMIP6 multi-model ensemble (MME) SSP5-8.5. The Table shows the region name, dates of the reference event, name of the heatwave characteristic ('Parameter_Name'), the reference value of the respective characteristic ('Parameter'), the respective reference data set ('Reanalysis'), the total number of heatwaves in the respective sample ('total_HW_num'), the number of heatwaves in the sample exceeding the reference value ('Parameter_Exceedance_Number'), and the exceedance percentage ('Parameter_Exceedance_Percentage'), the data subset type, the CMIP generation, and the repsective experiment.

Supplementary Data 27: Heatwave Characteristic Exceedange Percentages (Tx90p) - Table lists the shifts in exceedance percentages of the respective heatwave characteristic per data subdivided by Global Warming Levels (GWLs) for CMIP6 multi-model ensemble (MME) SSP5-8.5. The Table shows the region name, dates of the reference event, name of the heatwave characteristic ('Parameter_Name'), the reference value of the respective characteristic ('Parameter'), the respective reference data set ('Reanalysis'), the total number of heatwaves in the respective sample ('total_HW_num'), the number of heatwaves in the sample exceeding the reference value ('Parameter_Exceedance_Number'), and the exceedance percentage ('Parameter_Exceedance_Percentage'), the data subset type, the CMIP generation, and the repsective experiment.

Supplementary Data 28: Heatwave Characteristic Exceedange Percentages (Tx90p) - Table lists the percentages of projected heatwaves that excced the respective heatwave characteristic every single day for CMIP6 multi-model ensemble (MME) SSP5-8.5 for data subdivided by Global Warming Levels (GWLs). The Table shows the region name, dates of the reference event, name of the heatwave characteristic ('Parameter_Name'), the reference value of the respective characteristic ('Parameter'), the respective reference data set ('Reanalysis'), the total number of heatwaves in the respective sample ('total_HW_num'), the number of heatwaves in the sample exceeding the reference value ('Parameter_Exceedance_Number'), and the exceedance percentage ('Parameter_Exceedance_Percentage'), the data subset type, the CMIP generation, and the repsective experiment.

Supplementary Data 29: Distribution of Projected Heatwave Characteristics (Tx90p) - Table lists distribution of the projected heatwave characteristic for CMIP6 multi-model ensemble (MME) SSP5-8.5 for data subdivided by Global Warming Levels (GWLs). The table shows the projected value of the respective heatwave characteristic for each integer percentile in the respective sample.

Supplementary Data 30: Heatwave Characteristic Exceedange Percentages (Tx90p) - Table lists the exceedance percentages of the respective heatwave characteristic for CMIP6 multi-model ensemble (MME) SSP5-8.5 for data subdivided by Global Warming Levels (GWLs). The Table shows the region name, dates of the reference event, name of the heatwave characteristic ('Parameter_Name'), the reference value of the respective characteristic ('Parameter'), the respective reference data set ('Reanalysis'), the total number of heatwaves in the respective sample ('total_HW_num'), the number of heatwaves in the sample exceeding the reference value ('Parameter_Exceedance_Number'), and the exceedance percentage ('Parameter_Exceedance_Percentage'), the data subset type, the CMIP generation, and the repsective experiment.

Supplementary Data 31: Heatwave Characteristic Exceedange Percentages (Tx90p) - Table lists the shifts in exceedance percentages of the respective heatwave characteristic per data subdivided by Global Warming Levels (GWLs) for CMIP6 multi-model ensemble (MME) SSP5-8.5. The Table shows the region name, dates of the reference event, name of the heatwave characteristic ('Parameter_Name'), the reference value of the respective characteristic ('Parameter'), the respective reference data set ('Reanalysis'), the total number of heatwaves in the respective sample ('total_HW_num'), the number of heatwaves in the sample exceeding the reference value ('Parameter_Exceedance_Number'), and the exceedance percentage ('Parameter_Exceedance_Percentage'), the data subset type, the CMIP generation, and the repsective experiment.

Supplementary Data 32: Heatwave Characteristic Exceedange Percentages (Tx90p) - Table lists the shifts in exceedance percentages of the respective heatwave characteristic per data subdivided by Global Warming Levels (GWLs) for CMIP6 multi-model ensemble (MME) SSP5-8.5. The Table shows the region name, dates of the reference event, name of the heatwave characteristic ('Parameter_Name'), the reference value of the respective characteristic ('Parameter'), the respective reference data set ('Reanalysis'), the total number of heatwaves in the respective sample ('total_HW_num'), the number of heatwaves in the sample exceeding the reference value ('Parameter_Exceedance_Number'), and the exceedance percentage ('Parameter_Exceedance_Percentage'), the data subset type, the CMIP generation, and the repsective experiment.

Supplementary Data 33: Distribution of Projected Heatwave Characteristics (Tx99.0p) - Table lists distribution of the projected heatwave characteristic for CMIP6 multi-model ensemble (MME) SSP5-8.5 for data subdivided by Global Warming Levels (GWLs). The table shows the projected value of the respective heatwave characteristic for each integer percentile in the respective sample.

Supplementary Data 34: Heatwave Characteristic Exceedange Percentages (Tx90p) - Table lists the percentages of projected heatwaves that excced the respective heatwave for all six characteristics at once for CMIP6 multi-model ensemble (MME) SSP5-8.5 for data subdivided by Global Warming Levels (GWLs). The Table shows the region name, dates of the reference event, name of the heatwave characteristic ('Parameter_Name'), the reference value of the respective characteristic ('Parameter'), the respective reference data set ('Reanalysis'), the total number of heatwaves in the respective sample ('total_HW_num'), the number of heatwaves in the sample exceeding the reference value ('Parameter_Exceedance_Number'), and the exceedance percentage ('Parameter_Exceedance_Percentage'), the data subset type, the CMIP generation, and the repsective experiment.

Supplementary Data 35: Model Sampling for CMIP6 - Table lists the models in the sample for each region, including the modeling center, model name, model run, and the version numbers per model scenario.

Supplementary Data 36: Model Sampling for CMIP5 - Table lists the models in the sample for each region, including the modeling center, model name, model run, and the version numbers per model scenario.

Supplementary Data 37: Heatwave Characteristic Exceedange Percentages (Tx90p) - Table lists the exceedance percentages of the respective heatwave characteristic for CMIP6 multi-model ensemble (MME) SSP1-2.6 for data subdivided by decade. The Table shows the region name, dates of the reference event, name of the heatwave characteristic ('Parameter_Name'), the reference value of the respective characteristic ('Parameter'), the respective reference data set ('Reanalysis'), the total number of heatwaves in the respective sample ('total_HW_num'), the number of heatwaves in the sample exceeding the reference value ('Parameter_Exceedance_Number'), and the exceedance percentage ('Parameter_Exceedance_Percentage'), the data subset type, the CMIP generation, and the repsective experiment. Data based on ERA5

Supplementary Data 38: Heatwave Characteristic Exceedange Percentages (Tx90p) - Table lists the exceedance percentages of the respective heatwave characteristic for CMIP6 multi-model ensemble (MME) SSP1-2.6 for data subdivided by decade. The Table shows the region name, dates of the reference event, name of the heatwave characteristic ('Parameter_Name'), the reference value of the respective characteristic ('Parameter'), the respective reference data set ('Reanalysis'), the total number of heatwaves in the respective sample ('total_HW_num'), the number of heatwaves in the sample exceeding the reference value ('Parameter_Exceedance_Number'), and the exceedance percentage ('Parameter_Exceedance_Percentage'), the data subset type, the CMIP generation, and the repsective experiment. Data based on ERA5

Supplementary Data 39: Heatwave Characteristic Exceedange Percentages (Tx90p) - Table lists the exceedance percentages of the respective heatwave characteristic for CMIP6 multi-model ensemble (MME) SSP1-2.6 for data subdivided by decade. The Table shows the region name, dates of the reference event, name of the heatwave characteristic ('Parameter_Name'), the reference value of the respective characteristic ('Parameter'), the respective reference data set ('Reanalysis'), the total number of heatwaves in the respective sample ('total_HW_num'), the number of heatwaves in the sample exceeding the reference value ('Parameter_Exceedance_Number'), and the exceedance percentage ('Parameter_Exceedance_Percentage'), the data subset type, the CMIP generation, and the repsective experiment. Data based on ERA5

Supplementary Data 40: Heatwave Characteristic Exceedange Percentages (Tx90p) - Table lists the exceedance percentages of the respective heatwave characteristic for CMIP6 multi-model ensemble (MME) SSP1-2.6 for data subdivided by decade. The Table shows the region name, dates of the reference event, name of the heatwave characteristic ('Parameter_Name'), the reference value of the respective characteristic ('Parameter'), the respective reference data set ('Reanalysis'), the total number of heatwaves in the respective sample ('total_HW_num'), the number of heatwaves in the sample exceeding the reference value ('Parameter_Exceedance_Number'), and the exceedance percentage ('Parameter_Exceedance_Percentage'), the data subset type, the CMIP generation, and the repsective experiment. Data based on ERA5

Supplementary Data 41: Heatwave Characteristic Exceedange Percentages (Tx90p) - Table lists the exceedance percentages of the respective heatwave characteristic for CMIP6 multi-model ensemble (MME) SSP1-2.6 for data subdivided by decade. The Table shows the region name, dates of the reference event, name of the heatwave characteristic ('Parameter_Name'), the reference value of the respective characteristic ('Parameter'), the respective reference data set ('Reanalysis'), the total number of heatwaves in the respective sample ('total_HW_num'), the number of heatwaves in the sample exceeding the reference value ('Parameter_Exceedance_Number'), and the exceedance percentage ('Parameter_Exceedance_Percentage'), the data subset type, the CMIP generation, and the repsective experiment. Data based on ERA5

Supplementary Data 42: Heatwave Characteristic Exceedange Percentages (Tx90p) - Table lists the exceedance percentages of the respective heatwave characteristic for CMIP6 multi-model ensemble (MME) SSP1-2.6 for data subdivided by decade. The Table shows the region name, dates of the reference event, name of the heatwave characteristic ('Parameter_Name'), the reference value of the respective characteristic ('Parameter'), the respective reference data set ('Reanalysis'), the total number of heatwaves in the respective sample ('total_HW_num'), the number of heatwaves in the sample exceeding the reference value ('Parameter_Exceedance_Number'), and the exceedance percentage ('Parameter_Exceedance_Percentage'), the data subset type, the CMIP generation, and the repsective experiment. Data based on ERA5

Supplementary Data 43: Heatwave Characteristic Exceedange Percentages (Tx90p) - Table lists the exceedance percentages of the respective heatwave characteristic for CMIP6 multi-model ensemble (MME) SSP2-4.5 for data subdivided by decade. The Table shows the region name, dates of the reference event, name of the heatwave characteristic ('Parameter_Name'), the reference value of the respective characteristic ('Parameter'), the respective reference data set ('Reanalysis'), the total number of heatwaves in the respective sample ('total_HW_num'), the number of heatwaves in the sample exceeding the reference value ('Parameter_Exceedance_Number'), and the exceedance percentage ('Parameter_Exceedance_Percentage'), the data subset type, the CMIP generation, and the repsective experiment. Data based on ERA5

Supplementary Data 44: Heatwave Characteristic Exceedange Percentages (Tx90p) - Table lists the exceedance percentages of the respective heatwave characteristic for CMIP6 multi-model ensemble (MME) SSP2-4.5 for data subdivided by decade. The Table shows the region name, dates of the reference event, name of the heatwave characteristic ('Parameter_Name'), the reference value of the respective characteristic ('Parameter'), the respective reference data set ('Reanalysis'), the total number of heatwaves in the respective sample ('total_HW_num'), the number of heatwaves in the sample exceeding the reference value ('Parameter_Exceedance_Number'), and the exceedance percentage ('Parameter_Exceedance_Percentage'), the data subset type, the CMIP generation, and the repsective experiment. Data based on ERA5

Supplementary Data 45: Heatwave Characteristic Exceedange Percentages (Tx90p) - Table lists the exceedance percentages of the respective heatwave characteristic for CMIP6 multi-model ensemble (MME) SSP2-4.5 for data subdivided by decade. The Table shows the region name, dates of the reference event, name of the heatwave characteristic ('Parameter_Name'), the reference value of the respective characteristic ('Parameter'), the respective reference data set ('Reanalysis'), the total number of heatwaves in the respective sample ('total_HW_num'), the number of heatwaves in the sample exceeding the reference value ('Parameter_Exceedance_Number'), and the exceedance percentage ('Parameter_Exceedance_Percentage'), the data subset type, the CMIP generation, and the repsective experiment. Data based on ERA5

Supplementary Data 46: Heatwave Characteristic Exceedange Percentages (Tx90p) - Table lists the exceedance percentages of the respective heatwave characteristic for CMIP6 multi-model ensemble (MME) SSP2-4.5 for data subdivided by decade. The Table shows the region name, dates of the reference event, name of the heatwave characteristic ('Parameter_Name'), the reference value of the respective characteristic ('Parameter'), the respective reference data set ('Reanalysis'), the total number of heatwaves in the respective sample ('total_HW_num'), the number of heatwaves in the sample exceeding the reference value ('Parameter_Exceedance_Number'), and the exceedance percentage ('Parameter_Exceedance_Percentage'), the data subset type, the CMIP generation, and the repsective experiment. Data based on ERA5

Supplementary Data 47: Heatwave Characteristic Exceedange Percentages (Tx90p) - Table lists the exceedance percentages of the respective heatwave characteristic for CMIP6 multi-model ensemble (MME) SSP2-4.5 for data subdivided by decade. The Table shows the region name, dates of the reference event, name of the heatwave characteristic ('Parameter_Name'), the reference value of the respective characteristic ('Parameter'), the respective reference data set ('Reanalysis'), the total number of heatwaves in the respective sample ('total_HW_num'), the number of heatwaves in the sample exceeding the reference value

('Parameter_Exceedance_Number'), and the exceedance percentage ('Parameter_Exceedance_Percentage'), the data subset type, the CMIP generation, and the repsective experiment. Data based on ERA5

Supplementary Data 48: Heatwave Characteristic Exceedange Percentages (Tx90p) - Table lists the exceedance percentages of the respective heatwave characteristic for CMIP6 multi-model ensemble (MME) SSP2-4.5 for data subdivided by decade. The Table shows the region name, dates of the reference event, name of the heatwave characteristic ('Parameter_Name'), the reference value of the respective characteristic ('Parameter'), the respective reference data set ('Reanalysis'), the total number of heatwaves in the respective sample ('total_HW_num'), the number of heatwaves in the sample exceeding the reference value ('Parameter_Exceedance_Number'), and the exceedance percentage ('Parameter_Exceedance_Percentage'), the data subset type, the CMIP generation, and the repsective experiment. Data based on ERA5

Supplementary Data 49: Heatwave Characteristic Exceedange Percentages (Tx90p) - Table lists the exceedance percentages of the respective heatwave characteristic for CMIP6 multi-model ensemble (MME) SSP3-7.0 for data subdivided by decade. The Table shows the region name, dates of the reference event, name of the heatwave characteristic ('Parameter_Name'), the reference value of the respective characteristic ('Parameter'), the respective reference data set ('Reanalysis'), the total number of heatwaves in the respective sample ('total_HW_num'), the number of heatwaves in the sample exceeding the reference value ('Parameter_Exceedance_Number'), and the exceedance percentage ('Parameter_Exceedance_Percentage'), the data subset type, the CMIP generation, and the repsective experiment. Data based on ERA5

Supplementary Data 50: Heatwave Characteristic Exceedange Percentages (Tx90p) - Table lists the exceedance percentages of the respective heatwave characteristic for CMIP6 multi-model ensemble (MME) SSP3-7.0 for data subdivided by decade. The Table shows the region name, dates of the reference event, name of the heatwave characteristic ('Parameter_Name'), the reference value of the respective characteristic ('Parameter'), the respective reference data set ('Reanalysis'), the total number of heatwaves in the respective sample ('total_HW_num'), the number of heatwaves in the sample exceeding the reference value ('Parameter_Exceedance_Number'), and the exceedance percentage ('Parameter_Exceedance_Percentage'), the data subset type, the CMIP generation, and the repsective experiment. Data based on ERA5

Supplementary Data 51: Heatwave Characteristic Exceedange Percentages (Tx90p) - Table lists the exceedance percentages of the respective heatwave characteristic for CMIP6 multi-model ensemble (MME) SSP3-7.0 for data subdivided by decade. The Table shows the region name, dates of the reference event, name of the heatwave characteristic ('Parameter_Name'), the reference value of the respective characteristic ('Parameter'), the respective reference data set ('Reanalysis'), the total number of heatwaves in the respective sample ('total_HW_num'), the number of heatwaves in the sample exceeding the reference value ('Parameter_Exceedance_Number'), and the exceedance percentage ('Parameter_Exceedance_Percentage'), the data subset type, the CMIP generation, and the repsective experiment. Data based on ERA5

Supplementary Data 52: Heatwave Characteristic Exceedange Percentages (Tx90p) - Table lists the exceedance percentages of the respective heatwave characteristic for CMIP6 multi-model ensemble (MME) SSP3-7.0 for data subdivided by decade. The Table shows the region name, dates of the reference event, name of the heatwave characteristic ('Parameter_Name'), the reference value of the respective characteristic ('Parameter'),

the respective reference data set ('Reanalysis'), the total number of heatwaves in the respective sample ('total_HW_num'), the number of heatwaves in the sample exceeding the reference value ('Parameter_Exceedance_Number'), and the exceedance percentage ('Parameter_Exceedance_Percentage'), the data subset type, the CMIP generation, and the repsective experiment. Data based on ERA5

Supplementary Data 53: Heatwave Characteristic Exceedange Percentages (Tx90p) - Table lists the exceedance percentages of the respective heatwave characteristic for CMIP6 multi-model ensemble (MME) SSP3-7.0 for data subdivided by decade. The Table shows the region name, dates of the reference event, name of the heatwave characteristic ('Parameter_Name'), the reference value of the respective characteristic ('Parameter'), the respective reference data set ('Reanalysis'), the total number of heatwaves in the respective sample ('total_HW_num'), the number of heatwaves in the sample exceeding the reference value ('Parameter_Exceedance_Number'), and the exceedance percentage ('Parameter_Exceedance_Percentage'), the data subset type, the CMIP generation, and the repsective experiment. Data based on ERA5

Supplementary Data 54: Heatwave Characteristic Exceedange Percentages (Tx90p) - Table lists the exceedance percentages of the respective heatwave characteristic for CMIP6 multi-model ensemble (MME) SSP3-7.0 for data subdivided by decade. The Table shows the region name, dates of the reference event, name of the heatwave characteristic ('Parameter_Name'), the reference value of the respective characteristic ('Parameter'), the respective reference data set ('Reanalysis'), the total number of heatwaves in the respective sample ('total_HW_num'), the number of heatwaves in the sample exceeding the reference value ('Parameter_Exceedance_Number'), and the exceedance percentage ('Parameter_Exceedance_Percentage'), the data subset type, the CMIP generation, and the repsective experiment. Data based on ERA5

Supplementary Data 55: Heatwave Characteristic Exceedange Percentages (Tx90p) - Table lists the exceedance percentages of the respective heatwave characteristic for CMIP6 multi-model ensemble (MME) SSP5-8.5 for data subdivided by decade. The Table shows the region name, dates of the reference event, name of the heatwave characteristic ('Parameter_Name'), the reference value of the respective characteristic ('Parameter'), the respective reference data set ('Reanalysis'), the total number of heatwaves in the respective sample ('total_HW_num'), the number of heatwaves in the sample exceeding the reference value ('Parameter_Exceedance_Number'), and the exceedance percentage ('Parameter_Exceedance_Percentage'), the data subset type, the CMIP generation, and the repsective experiment. Data based on ERA5

Supplementary Data 56: Heatwave Characteristic Exceedange Percentages (Tx90p) - Table lists the exceedance percentages of the respective heatwave characteristic for CMIP6 multi-model ensemble (MME) SSP5-8.5 for data subdivided by decade. The Table shows the region name, dates of the reference event, name of the heatwave characteristic ('Parameter_Name'), the reference value of the respective characteristic ('Parameter'), the respective reference data set ('Reanalysis'), the total number of heatwaves in the respective sample ('total_HW_num'), the number of heatwaves in the sample exceeding the reference value ('Parameter_Exceedance_Number'), and the exceedance percentage ('Parameter_Exceedance_Percentage'), the data subset type, the CMIP generation, and the repsective experiment. Data based on ERA5

Supplementary Data 57: Heatwave Characteristic Exceedange Percentages (Tx90p) - Table lists the exceedance percentages of the respective heatwave characteristic for CMIP6 multi-model ensemble (MME) SSP5-8.5 for

data subdivided by decade. The Table shows the region name, dates of the reference event, name of the heatwave characteristic ('Parameter_Name'), the reference value of the respective characteristic ('Parameter'), the respective reference data set ('Reanalysis'), the total number of heatwaves in the respective sample ('total_HW_num'), the number of heatwaves in the sample exceeding the reference value ('Parameter_Exceedance_Number'), and the exceedance percentage ('Parameter_Exceedance_Percentage'), the data subset type, the CMIP generation, and the repsective experiment. Data based on ERA5

Supplementary Data 58: Heatwave Characteristic Exceedange Percentages (Tx90p) - Table lists the exceedance percentages of the respective heatwave characteristic for CMIP6 multi-model ensemble (MME) SSP5-8.5 for data subdivided by decade. The Table shows the region name, dates of the reference event, name of the heatwave characteristic ('Parameter_Name'), the reference value of the respective characteristic ('Parameter'), the respective reference data set ('Reanalysis'), the total number of heatwaves in the respective sample ('total_HW_num'), the number of heatwaves in the sample exceeding the reference value ('Parameter_Exceedance_Number'), and the exceedance percentage ('Parameter_Exceedance_Percentage'), the data subset type, the CMIP generation, and the repsective experiment. Data based on ERA5

Supplementary Data 59: Heatwave Characteristic Exceedange Percentages (Tx90p) - Table lists the exceedance percentages of the respective heatwave characteristic for CMIP6 multi-model ensemble (MME) SSP5-8.5 for data subdivided by decade. The Table shows the region name, dates of the reference event, name of the heatwave characteristic ('Parameter_Name'), the reference value of the respective characteristic ('Parameter'), the respective reference data set ('Reanalysis'), the total number of heatwaves in the respective sample ('total_HW_num'), the number of heatwaves in the sample exceeding the reference value ('Parameter_Exceedance_Number'), and the exceedance percentage ('Parameter_Exceedance_Percentage'), the data subset type, the CMIP generation, and the repsective experiment. Data based on ERA5

Supplementary Data 60: Heatwave Characteristic Exceedange Percentages (Tx90p) - Table lists the exceedance percentages of the respective heatwave characteristic for CMIP6 multi-model ensemble (MME) SSP5-8.5 for data subdivided by decade. The Table shows the region name, dates of the reference event, name of the heatwave characteristic ('Parameter_Name'), the reference value of the respective characteristic ('Parameter'), the respective reference data set ('Reanalysis'), the total number of heatwaves in the respective sample ('total_HW_num'), the number of heatwaves in the sample exceeding the reference value ('Parameter_Exceedance_Number'), and the exceedance percentage ('Parameter_Exceedance_Percentage'), the data subset type, the CMIP generation, and the repsective experiment. Data based on ERA5

Supplementary Data 61: Heatwave Characteristic Exceedange Percentages (Tx90p) - Table lists the exceedance percentages of the respective heatwave characteristic for CMIP6 multi-model ensemble (MME) SSP1-2.6 for data subdivided by Global Warming Levels (GWLs). The Table shows the region name, dates of the reference event, name of the heatwave characteristic ('Parameter_Name'), the reference value of the respective characteristic ('Parameter'), the respective reference data set ('Reanalysis'), the total number of heatwaves in the respective sample ('total_HW_num'), the number of heatwaves in the sample exceeding the reference value ('Parameter_Exceedance_Number'), and the exceedance percentage ('Parameter_Exceedance_Percentage'), the data subset type, the CMIP generation, and the repsective experiment. Data based on ERA5

Supplementary Data 62: Heatwave Characteristic Exceedange Percentages (Tx90p) - Table lists the exceedance percentages of the respective heatwave characteristic for CMIP6 multi-model ensemble (MME) SSP1-2.6 for data subdivided by Global Warming Levels (GWLs). The Table shows the region name, dates of the reference event, name of the heatwave characteristic ('Parameter_Name'), the reference value of the respective characteristic ('Parameter'), the respective reference data set ('Reanalysis'), the total number of heatwaves in the respective sample ('total_HW_num'), the number of heatwaves in the sample exceeding the reference value ('Parameter_Exceedance_Number'), and the exceedance percentage ('Parameter_Exceedance_Percentage'), the data subset type, the CMIP generation, and the repsective experiment. Data based on ERA5

Supplementary Data 63: Heatwave Characteristic Exceedange Percentages (Tx90p) - Table lists the exceedance percentages of the respective heatwave characteristic for CMIP6 multi-model ensemble (MME) SSP1-2.6 for data subdivided by Global Warming Levels (GWLs). The Table shows the region name, dates of the reference event, name of the heatwave characteristic ('Parameter_Name'), the reference value of the respective characteristic ('Parameter'), the respective reference data set ('Reanalysis'), the total number of heatwaves in the respective sample ('total_HW_num'), the number of heatwaves in the sample exceeding the reference value ('Parameter_Exceedance_Number'), and the exceedance percentage ('Parameter_Exceedance_Percentage'), the data subset type, the CMIP generation, and the repsective experiment. Data based on ERA5

Supplementary Data 64: Heatwave Characteristic Exceedange Percentages (Tx90p) - Table lists the exceedance percentages of the respective heatwave characteristic for CMIP6 multi-model ensemble (MME) SSP1-2.6 for data subdivided by Global Warming Levels (GWLs). The Table shows the region name, dates of the reference event, name of the heatwave characteristic ('Parameter_Name'), the reference value of the respective characteristic ('Parameter'), the respective reference data set ('Reanalysis'), the total number of heatwaves in the respective sample ('total_HW_num'), the number of heatwaves in the sample exceeding the reference value ('Parameter_Exceedance_Number'), and the exceedance percentage ('Parameter_Exceedance_Percentage'), the data subset type, the CMIP generation, and the repsective experiment. Data based on ERA5

Supplementary Data 65: Heatwave Characteristic Exceedange Percentages (Tx90p) - Table lists the exceedance percentages of the respective heatwave characteristic for CMIP6 multi-model ensemble (MME) SSP1-2.6 for data subdivided by Global Warming Levels (GWLs). The Table shows the region name, dates of the reference event, name of the heatwave characteristic ('Parameter_Name'), the reference value of the respective characteristic ('Parameter'), the respective reference data set ('Reanalysis'), the total number of heatwaves in the respective sample ('total_HW_num'), the number of heatwaves in the sample exceeding the reference value ('Parameter_Exceedance_Number'), and the exceedance percentage ('Parameter_Exceedance_Percentage'), the data subset type, the CMIP generation, and the repsective experiment. Data based on ERA5

Supplementary Data 66: Heatwave Characteristic Exceedange Percentages (Tx90p) - Table lists the exceedance percentages of the respective heatwave characteristic for CMIP6 multi-model ensemble (MME) SSP1-2.6 for data subdivided by Global Warming Levels (GWLs). The Table shows the region name, dates of the reference event, name of the heatwave characteristic ('Parameter_Name'), the reference value of the respective

characteristic ('Parameter'), the respective reference data set ('Reanalysis'), the total number of heatwaves in the respective sample ('total_HW_num'), the number of heatwaves in the sample exceeding the reference value ('Parameter_Exceedance_Number'), and the exceedance percentage ('Parameter_Exceedance_Percentage'), the data subset type, the CMIP generation, and the repsective experiment. Data based on ERA5

Supplementary Data 67: Heatwave Characteristic Exceedange Percentages (Tx90p) - Table lists the exceedance percentages of the respective heatwave characteristic for CMIP6 multi-model ensemble (MME) SSP2-4.5 for data subdivided by Global Warming Levels (GWLs). The Table shows the region name, dates of the reference event, name of the heatwave characteristic ('Parameter_Name'), the reference value of the respective characteristic ('Parameter'), the respective reference data set ('Reanalysis'), the total number of heatwaves in the respective sample ('total_HW_num'), the number of heatwaves in the sample exceeding the reference value ('Parameter_Exceedance_Number'), and the exceedance percentage ('Parameter_Exceedance_Percentage'), the data subset type, the CMIP generation, and the repsective experiment. Data based on ERA5

Supplementary Data 68: Heatwave Characteristic Exceedange Percentages (Tx90p) - Table lists the exceedance percentages of the respective heatwave characteristic for CMIP6 multi-model ensemble (MME) SSP2-4.5 for data subdivided by Global Warming Levels (GWLs). The Table shows the region name, dates of the reference event, name of the heatwave characteristic ('Parameter_Name'), the reference value of the respective characteristic ('Parameter'), the respective reference data set ('Reanalysis'), the total number of heatwaves in the respective sample ('total_HW_num'), the number of heatwaves in the sample exceeding the reference value ('Parameter_Exceedance_Number'), and the exceedance percentage ('Parameter_Exceedance_Percentage'), the data subset type, the CMIP generation, and the repsective experiment. Data based on ERA5

Supplementary Data 69: Heatwave Characteristic Exceedange Percentages (Tx90p) - Table lists the exceedance percentages of the respective heatwave characteristic for CMIP6 multi-model ensemble (MME) SSP2-4.5 for data subdivided by Global Warming Levels (GWLs). The Table shows the region name, dates of the reference event, name of the heatwave characteristic ('Parameter_Name'), the reference value of the respective characteristic ('Parameter'), the respective reference data set ('Reanalysis'), the total number of heatwaves in the respective sample ('total_HW_num'), the number of heatwaves in the sample exceeding the reference value ('Parameter_Exceedance_Number'), and the exceedance percentage ('Parameter_Exceedance_Percentage'), the data subset type, the CMIP generation, and the repsective experiment. Data based on ERA5

Supplementary Data 70: Heatwave Characteristic Exceedange Percentages (Tx90p) - Table lists the exceedance percentages of the respective heatwave characteristic for CMIP6 multi-model ensemble (MME) SSP2-4.5 for data subdivided by Global Warming Levels (GWLs). The Table shows the region name, dates of the reference event, name of the heatwave characteristic ('Parameter_Name'), the reference value of the respective characteristic ('Parameter'), the respective reference data set ('Reanalysis'), the total number of heatwaves in the respective sample ('total_HW_num'), the number of heatwaves in the sample exceeding the reference value ('Parameter_Exceedance_Number'), and the exceedance percentage

('Parameter_Exceedance_Percentage'), the data subset type, the CMIP generation, and the repsective experiment. Data based on ERA5

Supplementary Data 71: Heatwave Characteristic Exceedange Percentages (Tx90p) - Table lists the exceedance percentages of the respective heatwave characteristic for CMIP6 multi-model ensemble (MME) SSP2-4.5 for data subdivided by Global Warming Levels (GWLs). The Table shows the region name, dates of the reference event, name of the heatwave characteristic ('Parameter_Name'), the reference value of the respective characteristic ('Parameter'), the respective reference data set ('Reanalysis'), the total number of heatwaves in the respective sample ('total_HW_num'), the number of heatwaves in the sample exceeding the reference value ('Parameter_Exceedance_Number'), and the exceedance percentage ('Parameter_Exceedance_Percentage'), the data subset type, the CMIP generation, and the repsective experiment. Data based on ERA5

Supplementary Data 72: Heatwave Characteristic Exceedange Percentages (Tx90p) - Table lists the exceedance percentages of the respective heatwave characteristic for CMIP6 multi-model ensemble (MME) SSP2-4.5 for data subdivided by Global Warming Levels (GWLs). The Table shows the region name, dates of the reference event, name of the heatwave characteristic ('Parameter_Name'), the reference value of the respective characteristic ('Parameter'), the respective reference data set ('Reanalysis'), the total number of heatwaves in the respective sample ('total_HW_num'), the number of heatwaves in the sample exceeding the reference value ('Parameter_Exceedance_Number'), and the exceedance percentage ('Parameter_Exceedance_Percentage'), the data subset type, the CMIP generation, and the repsective experiment. Data based on ERA5

Supplementary Data 73: Heatwave Characteristic Exceedange Percentages (Tx90p) - Table lists the exceedance percentages of the respective heatwave characteristic for CMIP6 multi-model ensemble (MME) SSP3-7.0 for data subdivided by Global Warming Levels (GWLs). The Table shows the region name, dates of the reference event, name of the heatwave characteristic ('Parameter_Name'), the reference value of the respective characteristic ('Parameter'), the respective reference data set ('Reanalysis'), the total number of heatwaves in the respective sample ('total_HW_num'), the number of heatwaves in the sample exceeding the reference value ('Parameter_Exceedance_Number'), and the exceedance percentage ('Parameter_Exceedance_Percentage'), the data subset type, the CMIP generation, and the repsective experiment. Data based on ERA5

Supplementary Data 74: Heatwave Characteristic Exceedange Percentages (Tx90p) - Table lists the exceedance percentages of the respective heatwave characteristic for CMIP6 multi-model ensemble (MME) SSP3-7.0 for data subdivided by Global Warming Levels (GWLs). The Table shows the region name, dates of the reference event, name of the heatwave characteristic ('Parameter_Name'), the reference value of the respective characteristic ('Parameter'), the respective reference data set ('Reanalysis'), the total number of heatwaves in the respective sample ('total_HW_num'), the number of heatwaves in the sample exceeding the reference value ('Parameter_Exceedance_Number'), and the exceedance percentage ('Parameter_Exceedance_Percentage'), the data subset type, the CMIP generation, and the repsective experiment. Data based on ERA5

Supplementary Data 75: Heatwave Characteristic Exceedange Percentages (Tx90p) - Table lists the exceedance percentages of the respective heatwave characteristic for CMIP6 multi-model ensemble (MME) SSP3-7.0 for data subdivided by Global Warming Levels (GWLs). The Table shows the region name, dates of the reference event, name of the heatwave characteristic ('Parameter_Name'), the reference value of the respective characteristic ('Parameter'), the respective reference data set ('Reanalysis'), the total number of heatwaves in the respective sample ('total_HW_num'), the number of heatwaves in the sample exceeding the reference value ('Parameter_Exceedance_Number'), and the exceedance percentage ('Parameter_Exceedance_Percentage'), the data subset type, the CMIP generation, and the repsective experiment. Data based on ERA5

Supplementary Data 76: Heatwave Characteristic Exceedange Percentages (Tx90p) - Table lists the exceedance percentages of the respective heatwave characteristic for CMIP6 multi-model ensemble (MME) SSP3-7.0 for data subdivided by Global Warming Levels (GWLs). The Table shows the region name, dates of the reference event, name of the heatwave characteristic ('Parameter_Name'), the reference value of the respective characteristic ('Parameter'), the respective reference data set ('Reanalysis'), the total number of heatwaves in the respective sample ('total_HW_num'), the number of heatwaves in the sample exceeding the reference value ('Parameter_Exceedance_Number'), and the exceedance percentage ('Parameter_Exceedance_Percentage'), the data subset type, the CMIP generation, and the repsective experiment. Data based on ERA5

Supplementary Data 77: Heatwave Characteristic Exceedange Percentages (Tx90p) - Table lists the exceedance percentages of the respective heatwave characteristic for CMIP6 multi-model ensemble (MME) SSP3-7.0 for data subdivided by Global Warming Levels (GWLs). The Table shows the region name, dates of the reference event, name of the heatwave characteristic ('Parameter_Name'), the reference value of the respective characteristic ('Parameter'), the respective reference data set ('Reanalysis'), the total number of heatwaves in the respective sample ('total_HW_num'), the number of heatwaves in the sample exceeding the reference value ('Parameter_Exceedance_Number'), and the exceedance percentage ('Parameter_Exceedance_Percentage'), the data subset type, the CMIP generation, and the repsective experiment. Data based on ERA5

Supplementary Data 78: Heatwave Characteristic Exceedange Percentages (Tx90p) - Table lists the exceedance percentages of the respective heatwave characteristic for CMIP6 multi-model ensemble (MME) SSP3-7.0 for data subdivided by Global Warming Levels (GWLs). The Table shows the region name, dates of the reference event, name of the heatwave characteristic ('Parameter_Name'), the reference value of the respective characteristic ('Parameter'), the respective reference data set ('Reanalysis'), the total number of heatwaves in the respective sample ('total_HW_num'), the number of heatwaves in the sample exceeding the reference value ('Parameter_Exceedance_Number'), and the exceedance percentage ('Parameter_Exceedance_Percentage'), the data subset type, the CMIP generation, and the repsective experiment. Data based on ERA5

Supplementary Data 79: Heatwave Characteristic Exceedange Percentages (Tx90p) - Table lists the exceedance percentages of the respective heatwave characteristic for CMIP6 multi-model ensemble (MME) SSP5-8.5 for data subdivided by Global Warming Levels (GWLs). The Table shows the region name, dates of the reference event, name of the heatwave characteristic ('Parameter_Name'), the reference value of the respective

characteristic ('Parameter'), the respective reference data set ('Reanalysis'), the total number of heatwaves in the respective sample ('total_HW_num'), the number of heatwaves in the sample exceeding the reference value ('Parameter_Exceedance_Number'), and the exceedance percentage ('Parameter_Exceedance_Percentage'), the data subset type, the CMIP generation, and the repsective experiment. Data based on ERA5

Supplementary Data 80: Heatwave Characteristic Exceedange Percentages (Tx90p) - Table lists the exceedance percentages of the respective heatwave characteristic for CMIP6 multi-model ensemble (MME) SSP5-8.5 for data subdivided by Global Warming Levels (GWLs). The Table shows the region name, dates of the reference event, name of the heatwave characteristic ('Parameter_Name'), the reference value of the respective characteristic ('Parameter'), the respective reference data set ('Reanalysis'), the total number of heatwaves in the respective sample ('total_HW_num'), the number of heatwaves in the sample exceeding the reference value ('Parameter_Exceedance_Number'), and the exceedance percentage ('Parameter_Exceedance_Percentage'), the data subset type, the CMIP generation, and the repsective experiment. Data based on ERA5

Supplementary Data 81: Heatwave Characteristic Exceedange Percentages (Tx90p) - Table lists the exceedance percentages of the respective heatwave characteristic for CMIP6 multi-model ensemble (MME) SSP5-8.5 for data subdivided by Global Warming Levels (GWLs). The Table shows the region name, dates of the reference event, name of the heatwave characteristic ('Parameter_Name'), the reference value of the respective characteristic ('Parameter'), the respective reference data set ('Reanalysis'), the total number of heatwaves in the respective sample ('total_HW_num'), the number of heatwaves in the sample exceeding the reference value ('Parameter_Exceedance_Number'), and the exceedance percentage ('Parameter_Exceedance_Percentage'), the data subset type, the CMIP generation, and the repsective experiment. Data based on ERA5

Supplementary Data 82: Heatwave Characteristic Exceedange Percentages (Tx90p) - Table lists the exceedance percentages of the respective heatwave characteristic for CMIP6 multi-model ensemble (MME) SSP5-8.5 for data subdivided by Global Warming Levels (GWLs). The Table shows the region name, dates of the reference event, name of the heatwave characteristic ('Parameter_Name'), the reference value of the respective characteristic ('Parameter'), the respective reference data set ('Reanalysis'), the total number of heatwaves in the respective sample ('total_HW_num'), the number of heatwaves in the sample exceeding the reference value ('Parameter_Exceedance_Number'), and the exceedance percentage ('Parameter_Exceedance_Percentage'), the data subset type, the CMIP generation, and the repsective experiment. Data based on ERA5

Supplementary Data 83: Heatwave Characteristic Exceedange Percentages (Tx90p) - Table lists the exceedance percentages of the respective heatwave characteristic for CMIP6 multi-model ensemble (MME) SSP5-8.5 for data subdivided by Global Warming Levels (GWLs). The Table shows the region name, dates of the reference event, name of the heatwave characteristic ('Parameter_Name'), the reference value of the respective characteristic ('Parameter'), the respective reference data set ('Reanalysis'), the total number of heatwaves in the respective sample ('total_HW_num'), the number of heatwaves in the sample exceeding the reference value ('Parameter_Exceedance_Number'), and the exceedance percentage

('Parameter_Exceedance_Percentage'), the data subset type, the CMIP generation, and the repsective experiment. Data based on ERA5

Supplementary Data 84: Heatwave Characteristic Exceedange Percentages (Tx90p) - Table lists the exceedance percentages of the respective heatwave characteristic for CMIP6 multi-model ensemble (MME) SSP5-8.5 for data subdivided by Global Warming Levels (GWLs). The Table shows the region name, dates of the reference event, name of the heatwave characteristic ('Parameter_Name'), the reference value of the respective characteristic ('Parameter'), the respective reference data set ('Reanalysis'), the total number of heatwaves in the respective sample ('total_HW_num'), the number of heatwaves in the sample exceeding the reference value ('Parameter_Exceedance_Number'), and the exceedance percentage ('Parameter_Exceedance_Percentage'), the data subset type, the CMIP generation, and the repsective experiment. Data based on ERA5

Supplementary Data 85: Heatwave Characteristic Exceedange Percentages (Tx90p) - Table lists the exceedance percentages of the respective heatwave characteristic for CMIP6 multi-model ensemble (MME) SSP1-2.6 for entire dataset from 2020 to 2100. The Table shows the region name, dates of the reference event, name of the heatwave characteristic ('Parameter_Name'), the reference value of the respective characteristic ('Parameter'), the respective reference data set ('Reanalysis'), the total number of heatwaves in the respective sample ('total_HW_num'), the number of heatwaves in the sample exceeding the reference value ('Parameter_Exceedance_Number'), and the exceedance percentage ('Parameter_Exceedance_Percentage'), the data subset type, the CMIP generation, and the repsective experiment. Data based on ERA5

Supplementary Data 86: Heatwave Characteristic Exceedange Percentages (Tx90p) - Table lists the exceedance percentages of the respective heatwave characteristic for CMIP6 multi-model ensemble (MME) SSP1-2.6 for entire dataset from 2020 to 2100. The Table shows the region name, dates of the reference event, name of the heatwave characteristic ('Parameter_Name'), the reference value of the respective characteristic ('Parameter'), the respective reference data set ('Reanalysis'), the total number of heatwaves in the respective sample ('total_HW_num'), the number of heatwaves in the sample exceeding the reference value ('Parameter_Exceedance_Number'), and the exceedance percentage ('Parameter_Exceedance_Percentage'), the data subset type, the CMIP generation, and the repsective experiment. Data based on ERA5

Supplementary Data 87: Heatwave Characteristic Exceedange Percentages (Tx90p) - Table lists the exceedance percentages of the respective heatwave characteristic for CMIP6 multi-model ensemble (MME) SSP1-2.6 for entire dataset from 2020 to 2100. The Table shows the region name, dates of the reference event, name of the heatwave characteristic ('Parameter_Name'), the reference value of the respective characteristic ('Parameter'), the respective reference data set ('Reanalysis'), the total number of heatwaves in the respective sample ('total_HW_num'), the number of heatwaves in the sample exceeding the reference value ('Parameter_Exceedance_Number'), and the exceedance percentage ('Parameter_Exceedance_Percentage'), the data subset type, the CMIP generation, and the repsective experiment. Data based on ERA5

Supplementary Data 88: Heatwave Characteristic Exceedange Percentages (Tx90p) - Table lists the exceedance percentages of the respective heatwave characteristic for CMIP6 multi-model ensemble (MME) SSP1-2.6 for entire dataset from 2020 to 2100. The Table shows the region name, dates of the reference event, name of the

heatwave characteristic ('Parameter_Name'), the reference value of the respective characteristic ('Parameter'), the respective reference data set ('Reanalysis'), the total number of heatwaves in the respective sample ('total_HW_num'), the number of heatwaves in the sample exceeding the reference value ('Parameter_Exceedance_Number'), and the exceedance percentage ('Parameter_Exceedance_Percentage'), the data subset type, the CMIP generation, and the repsective experiment. Data based on ERA5

Supplementary Data 89: Heatwave Characteristic Exceedange Percentages (Tx90p) - Table lists the exceedance percentages of the respective heatwave characteristic for CMIP6 multi-model ensemble (MME) SSP1-2.6 for entire dataset from 2020 to 2100. The Table shows the region name, dates of the reference event, name of the heatwave characteristic ('Parameter_Name'), the reference value of the respective characteristic ('Parameter'), the respective reference data set ('Reanalysis'), the total number of heatwaves in the respective sample ('total_HW_num'), the number of heatwaves in the sample exceeding the reference value ('Parameter_Exceedance_Number'), and the exceedance percentage ('Parameter_Exceedance_Percentage'), the data subset type, the CMIP generation, and the repsective experiment. Data based on ERA5

Supplementary Data 90: Heatwave Characteristic Exceedange Percentages (Tx90p) - Table lists the exceedance percentages of the respective heatwave characteristic for CMIP6 multi-model ensemble (MME) SSP1-2.6 for entire dataset from 2020 to 2100. The Table shows the region name, dates of the reference event, name of the heatwave characteristic ('Parameter_Name'), the reference value of the respective characteristic ('Parameter'), the respective reference data set ('Reanalysis'), the total number of heatwaves in the respective sample ('total_HW_num'), the number of heatwaves in the sample exceeding the reference value ('Parameter_Exceedance_Number'), and the exceedance percentage ('Parameter_Exceedance_Percentage'), the data subset type, the CMIP generation, and the repsective experiment. Data based on ERA5

Supplementary Data 91: Heatwave Characteristic Exceedange Percentages (Tx90p) - Table lists the exceedance percentages of the respective heatwave characteristic for CMIP6 multi-model ensemble (MME) SSP2-4.5 for entire dataset from 2020 to 2100. The Table shows the region name, dates of the reference event, name of the heatwave characteristic ('Parameter_Name'), the reference value of the respective characteristic ('Parameter'), the respective reference data set ('Reanalysis'), the total number of heatwaves in the respective sample ('total_HW_num'), the number of heatwaves in the sample exceeding the reference value ('Parameter_Exceedance_Number'), and the exceedance percentage ('Parameter_Exceedance_Percentage'), the data subset type, the CMIP generation, and the repsective experiment. Data based on ERA5

Supplementary Data 92: Heatwave Characteristic Exceedange Percentages (Tx90p) - Table lists the exceedance percentages of the respective heatwave characteristic for CMIP6 multi-model ensemble (MME) SSP2-4.5 for entire dataset from 2020 to 2100. The Table shows the region name, dates of the reference event, name of the heatwave characteristic ('Parameter_Name'), the reference value of the respective characteristic ('Parameter'), the respective reference data set ('Reanalysis'), the total number of heatwaves in the respective sample ('total_HW_num'), the number of heatwaves in the sample exceeding the reference value ('Parameter_Exceedance_Number'), and the exceedance percentage ('Parameter_Exceedance_Percentage'), the data subset type, the CMIP generation, and the repsective experiment. Data based on ERA5

Supplementary Data 93: Heatwave Characteristic Exceedange Percentages (Tx90p) - Table lists the exceedance percentages of the respective heatwave characteristic for CMIP6 multi-model ensemble (MME) SSP2-4.5 for entire dataset from 2020 to 2100. The Table shows the region name, dates of the reference event, name of the heatwave characteristic ('Parameter_Name'), the reference value of the respective characteristic ('Parameter'), the respective reference data set ('Reanalysis'), the total number of heatwaves in the respective sample ('total_HW_num'), the number of heatwaves in the sample exceeding the reference value ('Parameter_Exceedance_Number'), and the exceedance percentage ('Parameter_Exceedance_Percentage'), the data subset type, the CMIP generation, and the repsective experiment. Data based on ERA5

Supplementary Data 94: Heatwave Characteristic Exceedange Percentages (Tx90p) - Table lists the exceedance percentages of the respective heatwave characteristic for CMIP6 multi-model ensemble (MME) SSP2-4.5 for entire dataset from 2020 to 2100. The Table shows the region name, dates of the reference event, name of the heatwave characteristic ('Parameter_Name'), the reference value of the respective characteristic ('Parameter'), the respective reference data set ('Reanalysis'), the total number of heatwaves in the respective sample ('total_HW_num'), the number of heatwaves in the sample exceeding the reference value ('Parameter_Exceedance_Number'), and the exceedance percentage ('Parameter_Exceedance_Percentage'), the data subset type, the CMIP generation, and the repsective experiment. Data based on ERA5

Supplementary Data 95: Heatwave Characteristic Exceedange Percentages (Tx90p) - Table lists the exceedance percentages of the respective heatwave characteristic for CMIP6 multi-model ensemble (MME) SSP2-4.5 for entire dataset from 2020 to 2100. The Table shows the region name, dates of the reference event, name of the heatwave characteristic ('Parameter_Name'), the reference value of the respective characteristic ('Parameter'), the respective reference data set ('Reanalysis'), the total number of heatwaves in the respective sample ('total_HW_num'), the number of heatwaves in the sample exceeding the reference value ('Parameter_Exceedance_Number'), and the exceedance percentage ('Parameter_Exceedance_Percentage'), the data subset type, the CMIP generation, and the repsective experiment. Data based on ERA5

Supplementary Data 96: Heatwave Characteristic Exceedange Percentages (Tx90p) - Table lists the exceedance percentages of the respective heatwave characteristic for CMIP6 multi-model ensemble (MME) SSP2-4.5 for entire dataset from 2020 to 2100. The Table shows the region name, dates of the reference event, name of the heatwave characteristic ('Parameter_Name'), the reference value of the respective characteristic ('Parameter'), the respective reference data set ('Reanalysis'), the total number of heatwaves in the respective sample ('total_HW_num'), the number of heatwaves in the sample exceeding the reference value ('Parameter_Exceedance_Number'), and the exceedance percentage ('Parameter_Exceedance_Percentage'), the data subset type, the CMIP generation, and the repsective experiment. Data based on ERA5

Supplementary Data 97: Heatwave Characteristic Exceedange Percentages (Tx90p) - Table lists the exceedance percentages of the respective heatwave characteristic for CMIP6 multi-model ensemble (MME) SSP3-7.0 for entire dataset from 2020 to 2100. The Table shows the region name, dates of the reference event, name of the heatwave characteristic ('Parameter_Name'), the reference value of the respective characteristic ('Parameter'), the respective reference data set ('Reanalysis'), the total number of heatwaves in the respective sample ('total_HW_num'), the number of heatwaves in the sample exceeding the reference value ('Parameter_Exceedance_Number'), and the exceedance percentage ('Parameter_Exceedance_Percentage'), the data subset type, the CMIP generation, and the repsective experiment. Data based on ERA5

Supplementary Data 98: Heatwave Characteristic Exceedange Percentages (Tx90p) - Table lists the exceedance percentages of the respective heatwave characteristic for CMIP6 multi-model ensemble (MME) SSP3-7.0 for entire dataset from 2020 to 2100. The Table shows the region name, dates of the reference event, name of the heatwave characteristic ('Parameter_Name'), the reference value of the respective characteristic ('Parameter'), the respective reference data set ('Reanalysis'), the total number of heatwaves in the respective sample ('total_HW_num'), the number of heatwaves in the sample exceeding the reference value ('Parameter_Exceedance_Number'), and the exceedance percentage ('Parameter_Exceedance_Percentage'), the data subset type, the CMIP generation, and the repsective experiment. Data based on ERA5

Supplementary Data 99: Heatwave Characteristic Exceedange Percentages (Tx90p) - Table lists the exceedance percentages of the respective heatwave characteristic for CMIP6 multi-model ensemble (MME) SSP3-7.0 for entire dataset from 2020 to 2100. The Table shows the region name, dates of the reference event, name of the heatwave characteristic ('Parameter_Name'), the reference value of the respective characteristic ('Parameter'), the respective reference data set ('Reanalysis'), the total number of heatwaves in the respective sample ('total_HW_num'), the number of heatwaves in the sample exceeding the reference value ('Parameter_Exceedance_Number'), and the exceedance percentage ('Parameter_Exceedance_Percentage'), the data subset type, the CMIP generation, and the repsective experiment. Data based on ERA5

Supplementary Data 100: Heatwave Characteristic Exceedange Percentages (Tx90p) - Table lists the exceedance percentages of the respective heatwave characteristic for CMIP6 multi-model ensemble (MME) SSP3-7.0 for entire dataset from 2020 to 2100. The Table shows the region name, dates of the reference event, name of the heatwave characteristic ('Parameter_Name'), the reference value of the respective characteristic ('Parameter'), the respective reference data set ('Reanalysis'), the total number of heatwaves in the respective sample ('total_HW_num'), the number of heatwaves in the sample exceeding the reference value ('Parameter_Exceedance_Number'), and the exceedance percentage ('Parameter_Exceedance_Percentage'), the data subset type, the CMIP generation, and the repsective experiment. Data based on ERA5

Supplementary Data 101: Heatwave Characteristic Exceedange Percentages (Tx90p) - Table lists the exceedance percentages of the respective heatwave characteristic for CMIP6 multi-model ensemble (MME) SSP3-7.0 for entire dataset from 2020 to 2100. The Table shows the region name, dates of the reference event, name of the heatwave characteristic ('Parameter_Name'), the reference value of the respective characteristic ('Parameter'), the respective reference data set ('Reanalysis'), the total number of heatwaves in the respective sample ('total_HW_num'), the number of heatwaves in the sample exceeding the reference value ('Parameter_Exceedance_Number'), and the exceedance percentage ('Parameter_Exceedance_Percentage'), the data subset type, the CMIP generation, and the repsective experiment. Data based on ERA5

Supplementary Data 102: Heatwave Characteristic Exceedange Percentages (Tx90p) - Table lists the exceedance percentages of the respective heatwave characteristic for CMIP6 multi-model ensemble (MME) SSP3-7.0 for entire dataset from 2020 to 2100. The Table shows the region name, dates of the reference event, name of the heatwave characteristic ('Parameter_Name'), the reference value of the respective characteristic ('Parameter'), the respective reference data set ('Reanalysis'), the total number of heatwaves in the respective sample ('total_HW_num'), the number of heatwaves in the sample exceeding the reference value

('Parameter_Exceedance_Number'), and the exceedance percentage ('Parameter_Exceedance_Percentage'), the data subset type, the CMIP generation, and the repsective experiment. Data based on ERA5

Supplementary Data 103: Heatwave Characteristic Exceedange Percentages (Tx90p) - Table lists the exceedance percentages of the respective heatwave characteristic for CMIP6 multi-model ensemble (MME) SSP5-8.5 for entire dataset from 2020 to 2100. The Table shows the region name, dates of the reference event, name of the heatwave characteristic ('Parameter_Name'), the reference value of the respective characteristic ('Parameter'), the respective reference data set ('Reanalysis'), the total number of heatwaves in the respective sample ('total_HW_num'), the number of heatwaves in the sample exceeding the reference value ('Parameter_Exceedance_Number'), and the exceedance percentage ('Parameter_Exceedance_Percentage'), the data subset type, the CMIP generation, and the repsective experiment. Data based on ERA5

Supplementary Data 104: Heatwave Characteristic Exceedange Percentages (Tx90p) - Table lists the exceedance percentages of the respective heatwave characteristic for CMIP6 multi-model ensemble (MME) SSP5-8.5 for entire dataset from 2020 to 2100. The Table shows the region name, dates of the reference event, name of the heatwave characteristic ('Parameter_Name'), the reference value of the respective characteristic ('Parameter'), the respective reference data set ('Reanalysis'), the total number of heatwaves in the respective sample ('total_HW_num'), the number of heatwaves in the sample exceeding the reference value ('Parameter_Exceedance_Number'), and the exceedance percentage ('Parameter_Exceedance_Percentage'), the data subset type, the CMIP generation, and the repsective experiment. Data based on ERA5

Supplementary Data 105: Heatwave Characteristic Exceedange Percentages (Tx90p) - Table lists the exceedance percentages of the respective heatwave characteristic for CMIP6 multi-model ensemble (MME) SSP5-8.5 for entire dataset from 2020 to 2100. The Table shows the region name, dates of the reference event, name of the heatwave characteristic ('Parameter_Name'), the reference value of the respective characteristic ('Parameter'), the respective reference data set ('Reanalysis'), the total number of heatwaves in the respective sample ('total_HW_num'), the number of heatwaves in the sample exceeding the reference value ('Parameter_Exceedance_Number'), and the exceedance percentage ('Parameter_Exceedance_Percentage'), the data subset type, the CMIP generation, and the repsective experiment. Data based on ERA5

Supplementary Data 106: Heatwave Characteristic Exceedange Percentages (Tx90p) - Table lists the exceedance percentages of the respective heatwave characteristic for CMIP6 multi-model ensemble (MME) SSP5-8.5 for entire dataset from 2020 to 2100. The Table shows the region name, dates of the reference event, name of the heatwave characteristic ('Parameter_Name'), the reference value of the respective characteristic ('Parameter'), the respective reference data set ('Reanalysis'), the total number of heatwaves in the respective sample ('total_HW_num'), the number of heatwaves in the sample exceeding the reference value ('Parameter_Exceedance_Number'), and the exceedance percentage ('Parameter_Exceedance_Percentage'), the data subset type, the CMIP generation, and the repsective experiment. Data based on ERA5

Supplementary Data 107: Heatwave Characteristic Exceedange Percentages (Tx90p) - Table lists the exceedance percentages of the respective heatwave characteristic for CMIP6 multi-model ensemble (MME) SSP5-8.5 for entire dataset from 2020 to 2100. The Table shows the region name, dates of the reference event, name of the heatwave characteristic ('Parameter_Name'), the reference value of the respective characteristic ('Parameter'),

the respective reference data set ('Reanalysis'), the total number of heatwaves in the respective sample ('total_HW_num'), the number of heatwaves in the sample exceeding the reference value ('Parameter_Exceedance_Number'), and the exceedance percentage ('Parameter_Exceedance_Percentage'), the data subset type, the CMIP generation, and the repsective experiment. Data based on ERA5

Supplementary Data 108: Heatwave Characteristic Exceedange Percentages (Tx90p) - Table lists the exceedance percentages of the respective heatwave characteristic for CMIP6 multi-model ensemble (MME) SSP5-8.5 for entire dataset from 2020 to 2100. The Table shows the region name, dates of the reference event, name of the heatwave characteristic ('Parameter_Name'), the reference value of the respective characteristic ('Parameter'), the respective reference data set ('Reanalysis'), the total number of heatwaves in the respective sample ('total_HW_num'), the number of heatwaves in the sample exceeding the reference value ('Parameter_Exceedance_Number'), and the exceedance percentage ('Parameter_Exceedance_Percentage'), the data subset type, the CMIP generation, and the repsective experiment. Data based on ERA5

Supplementary Data 109: Heatwave Characteristic Exceedange Percentages (Tx90p) - Table lists the percentages of projected heatwaves where the projected heatwave characteristic is several times as high as the reference characteristic for CMIP6 multi-model ensemble (MME) SSP1-2.6 for data subdivided by decade. The Table shows the region name, dates of the reference event, name of the heatwave characteristic ('Parameter_Name'), the reference value of the respective characteristic ('Parameter'), the respective reference data set ('Reanalysis'), the total number of heatwaves in the respective sample ('total_HW_num'), the number of heatwaves in the sample exceeding the reference value ('Parameter_Exceedance_Number'), and the exceedance percentage ('Parameter_Exceedance_Percentage'), the data subset type, the CMIP generation, and the repsective experiment. Data based on ERA5

Supplementary Data 110: Heatwave Characteristic Exceedange Percentages (Tx90p) - Table lists the percentages of projected heatwaves where the projected heatwave characteristic is several times as high as the reference characteristic for CMIP6 multi-model ensemble (MME) SSP1-2.6 for data subdivided by decade. The Table shows the region name, dates of the reference event, name of the heatwave characteristic ('Parameter_Name'), the reference value of the respective characteristic ('Parameter'), the respective reference data set ('Reanalysis'), the total number of heatwaves in the respective sample ('total_HW_num'), the number of heatwaves in the sample exceeding the reference value ('Parameter_Exceedance_Number'), and the exceedance percentage ('Parameter_Exceedance_Percentage'), the data subset type, the CMIP generation, and the repsective experiment. Data based on ERA5

Supplementary Data 111: Heatwave Characteristic Exceedange Percentages (Tx90p) - Table lists the percentages of projected heatwaves where the projected heatwave characteristic is several times as high as the reference characteristic for CMIP6 multi-model ensemble (MME) SSP1-2.6 for data subdivided by decade. The Table shows the region name, dates of the reference event, name of the heatwave characteristic ('Parameter_Name'), the reference value of the respective characteristic ('Parameter'), the respective reference data set ('Reanalysis'), the total number of heatwaves in the respective sample ('total_HW_num'), the number of heatwaves in the sample exceeding the reference value ('Parameter_Exceedance_Number'), and the exceedance percentage ('Parameter_Exceedance_Percentage'), the data subset type, the CMIP generation, and the repsective experiment. Data based on ERA5

Supplementary Data 112: Heatwave Characteristic Exceedange Percentages (Tx90p) - Table lists the percentages of projected heatwaves where the projected heatwave characteristic is several times as high as the reference characteristic for CMIP6 multi-model ensemble (MME) SSP1-2.6 for data subdivided by decade. The Table shows the region name, dates of the reference event, name of the heatwave characteristic ('Parameter_Name'), the reference value of the respective characteristic ('Parameter'), the respective reference data set ('Reanalysis'), the total number of heatwaves in the respective sample ('total_HW_num'), the number of heatwaves in the sample exceeding the reference value ('Parameter_Exceedance_Number'), and the exceedance percentage ('Parameter_Exceedance_Percentage'), the data subset type, the CMIP generation, and the repsective experiment. Data based on ERA5

Supplementary Data 113: Heatwave Characteristic Exceedange Percentages (Tx90p) - Table lists the percentages of projected heatwaves where the projected heatwave characteristic is several times as high as the reference characteristic for CMIP6 multi-model ensemble (MME) SSP1-2.6 for data subdivided by decade. The Table shows the region name, dates of the reference event, name of the heatwave characteristic ('Parameter_Name'), the reference value of the respective characteristic ('Parameter'), the respective reference data set ('Reanalysis'), the total number of heatwaves in the respective sample ('total_HW_num'), the number of heatwaves in the sample exceeding the reference value ('Parameter_Exceedance_Number'), and the exceedance percentage ('Parameter_Exceedance_Percentage'), the data subset type, the CMIP generation, and the repsective experiment. Data based on ERA5

Supplementary Data 114: Heatwave Characteristic Exceedange Percentages (Tx90p) - Table lists the percentages of projected heatwaves where the projected heatwave characteristic is several times as high as the reference characteristic for CMIP6 multi-model ensemble (MME) SSP1-2.6 for data subdivided by decade. The Table shows the region name, dates of the reference event, name of the heatwave characteristic ('Parameter_Name'), the reference value of the respective characteristic ('Parameter'), the respective reference data set ('Reanalysis'), the total number of heatwaves in the respective sample ('total_HW_num'), the number of heatwaves in the sample exceeding the reference value ('Parameter_Exceedance_Number'), and the exceedance percentage ('Parameter_Exceedance_Percentage'), the data subset type, the CMIP generation, and the repsective experiment. Data based on ERA5

Supplementary Data 115: Heatwave Characteristic Exceedange Percentages (Tx90p) - Table lists the percentages of projected heatwaves where the projected heatwave characteristic is several times as high as the reference characteristic for CMIP6 multi-model ensemble (MME) SSP2-4.5 for data subdivided by decade. The Table shows the region name, dates of the reference event, name of the heatwave characteristic ('Parameter_Name'), the reference value of the respective characteristic ('Parameter'), the respective reference data set ('Reanalysis'), the total number of heatwaves in the respective sample ('total_HW_num'), the number of heatwaves in the sample exceeding the reference value ('Parameter_Exceedance_Number'), and the exceedance percentage ('Parameter_Exceedance_Percentage'), the data subset type, the CMIP generation, and the repsective experiment. Data based on ERA5

Supplementary Data 116: Heatwave Characteristic Exceedange Percentages (Tx90p) - Table lists the percentages of projected heatwaves where the projected heatwave characteristic is several times as high as the reference characteristic for CMIP6 multi-model ensemble (MME) SSP2-4.5 for data subdivided by decade. The Table shows the region name, dates of the reference event, name of the heatwave characteristic

('Parameter_Name'), the reference value of the respective characteristic ('Parameter'), the respective reference data set ('Reanalysis'), the total number of heatwaves in the respective sample ('total_HW_num'), the number of heatwaves in the sample exceeding the reference value ('Parameter_Exceedance_Number'), and the exceedance percentage ('Parameter_Exceedance_Percentage'), the data subset type, the CMIP generation, and the repsective experiment. Data based on ERA5

Supplementary Data 117: Heatwave Characteristic Exceedange Percentages (Tx90p) - Table lists the percentages of projected heatwaves where the projected heatwave characteristic is several times as high as the reference characteristic for CMIP6 multi-model ensemble (MME) SSP2-4.5 for data subdivided by decade. The Table shows the region name, dates of the reference event, name of the heatwave characteristic ('Parameter_Name'), the reference value of the respective characteristic ('Parameter'), the respective reference data set ('Reanalysis'), the total number of heatwaves in the respective sample ('total_HW_num'), the number of heatwaves in the sample exceeding the reference value ('Parameter_Exceedance_Number'), and the exceedance percentage ('Parameter_Exceedance_Percentage'), the data subset type, the CMIP generation, and the repsective experiment. Data based on ERA5

Supplementary Data 118: Heatwave Characteristic Exceedange Percentages (Tx90p) - Table lists the percentages of projected heatwaves where the projected heatwave characteristic is several times as high as the reference characteristic for CMIP6 multi-model ensemble (MME) SSP2-4.5 for data subdivided by decade. The Table shows the region name, dates of the reference event, name of the heatwave characteristic ('Parameter_Name'), the reference value of the respective characteristic ('Parameter'), the respective reference data set ('Reanalysis'), the total number of heatwaves in the respective sample ('total_HW_num'), the number of heatwaves in the sample exceeding the reference value ('Parameter_Exceedance_Number'), and the exceedance percentage ('Parameter_Exceedance_Percentage'), the data subset type, the CMIP generation, and the repsective experiment. Data based on ERA5

Supplementary Data 119: Heatwave Characteristic Exceedange Percentages (Tx90p) - Table lists the percentages of projected heatwaves where the projected heatwave characteristic is several times as high as the reference characteristic for CMIP6 multi-model ensemble (MME) SSP2-4.5 for data subdivided by decade. The Table shows the region name, dates of the reference event, name of the heatwave characteristic ('Parameter_Name'), the reference value of the respective characteristic ('Parameter'), the respective reference data set ('Reanalysis'), the total number of heatwaves in the respective sample ('total_HW_num'), the number of heatwaves in the sample exceeding the reference value ('Parameter_Exceedance_Number'), and the exceedance percentage ('Parameter_Exceedance_Percentage'), the data subset type, the CMIP generation, and the repsective experiment. Data based on ERA5

Supplementary Data 120: Heatwave Characteristic Exceedange Percentages (Tx90p) - Table lists the percentages of projected heatwaves where the projected heatwave characteristic is several times as high as the reference characteristic for CMIP6 multi-model ensemble (MME) SSP2-4.5 for data subdivided by decade. The Table shows the region name, dates of the reference event, name of the heatwave characteristic ('Parameter_Name'), the reference value of the respective characteristic ('Parameter'), the respective reference data set ('Reanalysis'), the total number of heatwaves in the respective sample ('total_HW_num'), the number of heatwaves in the sample exceeding the reference value ('Parameter_Exceedance_Number'), and the

exceedance percentage ('Parameter_Exceedance_Percentage'), the data subset type, the CMIP generation, and the repsective experiment. Data based on ERA5

Supplementary Data 121: Heatwave Characteristic Exceedange Percentages (Tx90p) - Table lists the percentages of projected heatwaves where the projected heatwave characteristic is several times as high as the reference characteristic for CMIP6 multi-model ensemble (MME) SSP3-7.0 for data subdivided by decade. The Table shows the region name, dates of the reference event, name of the heatwave characteristic ('Parameter_Name'), the reference value of the respective characteristic ('Parameter'), the respective reference data set ('Reanalysis'), the total number of heatwaves in the respective sample ('total_HW_num'), the number of heatwaves in the sample exceeding the reference value ('Parameter_Exceedance_Number'), and the exceedance percentage ('Parameter_Exceedance_Percentage'), the data subset type, the CMIP generation, and the repsective experiment. Data based on ERA5

Supplementary Data 122: Heatwave Characteristic Exceedange Percentages (Tx90p) - Table lists the percentages of projected heatwaves where the projected heatwave characteristic is several times as high as the reference characteristic for CMIP6 multi-model ensemble (MME) SSP3-7.0 for data subdivided by decade. The Table shows the region name, dates of the reference event, name of the heatwave characteristic ('Parameter_Name'), the reference value of the respective characteristic ('Parameter'), the respective reference data set ('Reanalysis'), the total number of heatwaves in the respective sample ('total_HW_num'), the number of heatwaves in the sample exceeding the reference value ('Parameter_Exceedance_Number'), and the exceedance percentage ('Parameter_Exceedance_Percentage'), the data subset type, the CMIP generation, and the repsective experiment. Data based on ERA5

Supplementary Data 123: Heatwave Characteristic Exceedange Percentages (Tx90p) - Table lists the percentages of projected heatwaves where the projected heatwave characteristic is several times as high as the reference characteristic for CMIP6 multi-model ensemble (MME) SSP3-7.0 for data subdivided by decade. The Table shows the region name, dates of the reference event, name of the heatwave characteristic ('Parameter_Name'), the reference value of the respective characteristic ('Parameter'), the respective reference data set ('Reanalysis'), the total number of heatwaves in the respective sample ('total_HW_num'), the number of heatwaves in the sample exceeding the reference value ('Parameter_Exceedance_Number'), and the exceedance percentage ('Parameter_Exceedance_Percentage'), the data subset type, the CMIP generation, and the repsective experiment. Data based on ERA5

Supplementary Data 124: Heatwave Characteristic Exceedange Percentages (Tx90p) - Table lists the percentages of projected heatwaves where the projected heatwave characteristic is several times as high as the reference characteristic for CMIP6 multi-model ensemble (MME) SSP3-7.0 for data subdivided by decade. The Table shows the region name, dates of the reference event, name of the heatwave characteristic ('Parameter_Name'), the reference value of the respective characteristic ('Parameter'), the respective reference data set ('Reanalysis'), the total number of heatwaves in the respective sample ('total_HW_num'), the number of heatwaves in the sample exceeding the reference value ('Parameter_Exceedance_Number'), and the exceedance percentage ('Parameter_Exceedance_Percentage'), the data subset type, the CMIP generation, and the repsective experiment. Data based on ERA5

Supplementary Data 125: Heatwave Characteristic Exceedange Percentages (Tx90p) - Table lists the percentages of projected heatwaves where the projected heatwave characteristic is several times as high as the reference characteristic for CMIP6 multi-model ensemble (MME) SSP3-7.0 for data subdivided by decade. The Table shows the region name, dates of the reference event, name of the heatwave characteristic ('Parameter_Name'), the reference value of the respective characteristic ('Parameter'), the respective reference data set ('Reanalysis'), the total number of heatwaves in the respective sample ('total_HW_num'), the number of heatwaves in the sample exceeding the reference value ('Parameter_Exceedance_Number'), and the exceedance percentage ('Parameter_Exceedance_Percentage'), the data subset type, the CMIP generation, and the repsective experiment. Data based on ERA5

Supplementary Data 126: Heatwave Characteristic Exceedange Percentages (Tx90p) - Table lists the percentages of projected heatwaves where the projected heatwave characteristic is several times as high as the reference characteristic for CMIP6 multi-model ensemble (MME) SSP3-7.0 for data subdivided by decade. The Table shows the region name, dates of the reference event, name of the heatwave characteristic ('Parameter_Name'), the reference value of the respective characteristic ('Parameter'), the respective reference data set ('Reanalysis'), the total number of heatwaves in the respective sample ('total_HW_num'), the number of heatwaves in the sample exceeding the reference value ('Parameter_Exceedance_Number'), and the exceedance percentage ('Parameter_Exceedance_Percentage'), the data subset type, the CMIP generation, and the repsective experiment. Data based on ERA5

Supplementary Data 127: Heatwave Characteristic Exceedange Percentages (Tx90p) - Table lists the percentages of projected heatwaves where the projected heatwave characteristic is several times as high as the reference characteristic for CMIP6 multi-model ensemble (MME) SSP5-8.5 for data subdivided by decade. The Table shows the region name, dates of the reference event, name of the heatwave characteristic ('Parameter_Name'), the reference value of the respective characteristic ('Parameter'), the respective reference data set ('Reanalysis'), the total number of heatwaves in the respective sample ('total_HW_num'), the number of heatwaves in the sample exceeding the reference value ('Parameter_Exceedance_Number'), and the exceedance percentage ('Parameter_Exceedance_Percentage'), the data subset type, the CMIP generation, and the repsective experiment. Data based on ERA5

Supplementary Data 128: Heatwave Characteristic Exceedange Percentages (Tx90p) - Table lists the percentages of projected heatwaves where the projected heatwave characteristic is several times as high as the reference characteristic for CMIP6 multi-model ensemble (MME) SSP5-8.5 for data subdivided by decade. The Table shows the region name, dates of the reference event, name of the heatwave characteristic ('Parameter_Name'), the reference value of the respective characteristic ('Parameter'), the respective reference data set ('Reanalysis'), the total number of heatwaves in the respective sample ('total_HW_num'), the number of heatwaves in the sample exceeding the reference value ('Parameter_Exceedance_Number'), and the exceedance percentage ('Parameter_Exceedance_Percentage'), the data subset type, the CMIP generation, and the repsective experiment. Data based on ERA5

Supplementary Data 129: Heatwave Characteristic Exceedange Percentages (Tx90p) - Table lists the percentages of projected heatwaves where the projected heatwave characteristic is several times as high as the reference characteristic for CMIP6 multi-model ensemble (MME) SSP5-8.5 for data subdivided by decade. The Table shows the region name, dates of the reference event, name of the heatwave characteristic

('Parameter_Name'), the reference value of the respective characteristic ('Parameter'), the respective reference data set ('Reanalysis'), the total number of heatwaves in the respective sample ('total_HW_num'), the number of heatwaves in the sample exceeding the reference value ('Parameter_Exceedance_Number'), and the exceedance percentage ('Parameter_Exceedance_Percentage'), the data subset type, the CMIP generation, and the repsective experiment. Data based on ERA5

Supplementary Data 130: Heatwave Characteristic Exceedange Percentages (Tx90p) - Table lists the percentages of projected heatwaves where the projected heatwave characteristic is several times as high as the reference characteristic for CMIP6 multi-model ensemble (MME) SSP5-8.5 for data subdivided by decade. The Table shows the region name, dates of the reference event, name of the heatwave characteristic ('Parameter_Name'), the reference value of the respective characteristic ('Parameter'), the respective reference data set ('Reanalysis'), the total number of heatwaves in the respective sample ('total_HW_num'), the number of heatwaves in the sample exceeding the reference value ('Parameter_Exceedance_Number'), and the exceedance percentage ('Parameter_Exceedance_Percentage'), the data subset type, the CMIP generation, and the repsective experiment. Data based on ERA5

Supplementary Data 131: Heatwave Characteristic Exceedange Percentages (Tx90p) - Table lists the percentages of projected heatwaves where the projected heatwave characteristic is several times as high as the reference characteristic for CMIP6 multi-model ensemble (MME) SSP5-8.5 for data subdivided by decade. The Table shows the region name, dates of the reference event, name of the heatwave characteristic ('Parameter_Name'), the reference value of the respective characteristic ('Parameter'), the respective reference data set ('Reanalysis'), the total number of heatwaves in the respective sample ('total_HW_num'), the number of heatwaves in the sample exceeding the reference value ('Parameter_Exceedance_Number'), and the exceedance percentage ('Parameter_Exceedance_Percentage'), the data subset type, the CMIP generation, and the repsective experiment. Data based on ERA5

Supplementary Data 132: Heatwave Characteristic Exceedange Percentages (Tx90p) - Table lists the percentages of projected heatwaves where the projected heatwave characteristic is several times as high as the reference characteristic for CMIP6 multi-model ensemble (MME) SSP5-8.5 for data subdivided by decade. The Table shows the region name, dates of the reference event, name of the heatwave characteristic ('Parameter_Name'), the reference value of the respective characteristic ('Parameter'), the respective reference data set ('Reanalysis'), the total number of heatwaves in the respective sample ('total_HW_num'), the number of heatwaves in the sample exceeding the reference value ('Parameter_Exceedance_Number'), and the exceedance percentage ('Parameter_Exceedance_Percentage'), the data subset type, the CMIP generation, and the repsective experiment. Data based on ERA5

Supplementary Data 133: Heatwave Characteristic Exceedange Percentages (Tx90p) - Table lists the percentages of projected heatwaves where the projected heatwave characteristic is several times as high as the reference characteristic for CMIP6 multi-model ensemble (MME) SSP1-2.6 for data subdivided by Global Warming Levels (GWLs). The Table shows the region name, dates of the reference event, name of the heatwave characteristic ('Parameter_Name'), the reference value of the respective characteristic ('Parameter'), the respective reference data set ('Reanalysis'), the total number of heatwaves in the respective sample ('total_HW_num'), the number of heatwaves in the sample exceeding the reference value

('Parameter_Exceedance_Number'), and the exceedance percentage ('Parameter_Exceedance_Percentage'), the data subset type, the CMIP generation, and the repsective experiment. Data based on ERA5

Supplementary Data 134: Heatwave Characteristic Exceedange Percentages (Tx90p) - Table lists the percentages of projected heatwaves where the projected heatwave characteristic is several times as high as the reference characteristic for CMIP6 multi-model ensemble (MME) SSP1-2.6 for data subdivided by Global Warming Levels (GWLs). The Table shows the region name, dates of the reference event, name of the heatwave characteristic ('Parameter_Name'), the reference value of the respective characteristic ('Parameter'), the respective reference data set ('Reanalysis'), the total number of heatwaves in the respective sample ('total_HW_num'), the number of heatwaves in the sample exceeding the reference value ('Parameter_Exceedance_Number'), and the exceedance percentage ('Parameter_Exceedance_Percentage'), the data subset type, the CMIP generation, and the repsective experiment. Data based on ERA5

Supplementary Data 135: Heatwave Characteristic Exceedange Percentages (Tx90p) - Table lists the percentages of projected heatwaves where the projected heatwave characteristic is several times as high as the reference characteristic for CMIP6 multi-model ensemble (MME) SSP1-2.6 for data subdivided by Global Warming Levels (GWLs). The Table shows the region name, dates of the reference event, name of the heatwave characteristic ('Parameter_Name'), the reference value of the respective characteristic ('Parameter'), the respective reference data set ('Reanalysis'), the total number of heatwaves in the respective sample ('total_HW_num'), the number of heatwaves in the sample exceeding the reference value ('Parameter_Exceedance_Number'), and the exceedance percentage ('Parameter_Exceedance_Percentage'), the data subset type, the CMIP generation, and the repsective experiment. Data based on ERA5

Supplementary Data 136: Heatwave Characteristic Exceedange Percentages (Tx90p) - Table lists the percentages of projected heatwaves where the projected heatwave characteristic is several times as high as the reference characteristic for CMIP6 multi-model ensemble (MME) SSP1-2.6 for data subdivided by Global Warming Levels (GWLs). The Table shows the region name, dates of the reference event, name of the heatwave characteristic ('Parameter_Name'), the reference value of the respective characteristic ('Parameter'), the respective reference data set ('Reanalysis'), the total number of heatwaves in the respective sample ('total_HW_num'), the number of heatwaves in the sample exceeding the reference value ('Parameter_Exceedance_Number'), and the exceedance percentage ('Parameter_Exceedance_Percentage'), the data subset type, the CMIP generation, and the repsective experiment. Data based on ERA5

Supplementary Data 137: Heatwave Characteristic Exceedange Percentages (Tx90p) - Table lists the percentages of projected heatwaves where the projected heatwave characteristic is several times as high as the reference characteristic for CMIP6 multi-model ensemble (MME) SSP1-2.6 for data subdivided by Global Warming Levels (GWLs). The Table shows the region name, dates of the reference event, name of the heatwave characteristic ('Parameter_Name'), the reference value of the respective characteristic ('Parameter'), the respective reference data set ('Reanalysis'), the total number of heatwaves in the respective sample ('total_HW_num'), the number of heatwaves in the sample exceeding the reference value ('Parameter_Exceedance_Number'), and the exceedance percentage ('Parameter_Exceedance_Percentage'), the data subset type, the CMIP generation, and the repsective experiment. Data based on ERA5

Supplementary Data 138: Heatwave Characteristic Exceedange Percentages (Tx90p) - Table lists the percentages of projected heatwaves where the projected heatwave characteristic is several times as high as the reference characteristic for CMIP6 multi-model ensemble (MME) SSP1-2.6 for data subdivided by Global Warming Levels (GWLs). The Table shows the region name, dates of the reference event, name of the heatwave characteristic ('Parameter_Name'), the reference value of the respective characteristic ('Parameter'), the respective reference data set ('Reanalysis'), the total number of heatwaves in the respective sample ('total_HW_num'), the number of heatwaves in the sample exceeding the reference value ('Parameter_Exceedance_Number'), and the exceedance percentage ('Parameter_Exceedance_Percentage'), the data subset type, the CMIP generation, and the repsective experiment. Data based on ERA5

Supplementary Data 139: Heatwave Characteristic Exceedange Percentages (Tx90p) - Table lists the percentages of projected heatwaves where the projected heatwave characteristic is several times as high as the reference characteristic for CMIP6 multi-model ensemble (MME) SSP2-4.5 for data subdivided by Global Warming Levels (GWLs). The Table shows the region name, dates of the reference event, name of the heatwave characteristic ('Parameter_Name'), the reference value of the respective characteristic ('Parameter'), the respective reference data set ('Reanalysis'), the total number of heatwaves in the respective sample ('total_HW_num'), the number of heatwaves in the sample exceeding the reference value ('Parameter_Exceedance_Number'), and the exceedance percentage ('Parameter_Exceedance_Percentage'), the data subset type, the CMIP generation, and the repsective experiment. Data based on ERA5

Supplementary Data 140: Heatwave Characteristic Exceedange Percentages (Tx90p) - Table lists the percentages of projected heatwaves where the projected heatwave characteristic is several times as high as the reference characteristic for CMIP6 multi-model ensemble (MME) SSP2-4.5 for data subdivided by Global Warming Levels (GWLs). The Table shows the region name, dates of the reference event, name of the heatwave characteristic ('Parameter_Name'), the reference value of the respective characteristic ('Parameter'), the respective reference data set ('Reanalysis'), the total number of heatwaves in the respective sample ('total_HW_num'), the number of heatwaves in the sample exceeding the reference value ('Parameter_Exceedance_Number'), and the exceedance percentage ('Parameter_Exceedance_Percentage'), the data subset type, the CMIP generation, and the repsective experiment. Data based on ERA5

Supplementary Data 141: Heatwave Characteristic Exceedange Percentages (Tx90p) - Table lists the percentages of projected heatwaves where the projected heatwave characteristic is several times as high as the reference characteristic for CMIP6 multi-model ensemble (MME) SSP2-4.5 for data subdivided by Global Warming Levels (GWLs). The Table shows the region name, dates of the reference event, name of the heatwave characteristic ('Parameter_Name'), the reference value of the respective characteristic ('Parameter'), the respective reference data set ('Reanalysis'), the total number of heatwaves in the respective sample ('total_HW_num'), the number of heatwaves in the sample exceeding the reference value ('Parameter_Exceedance_Number'), and the exceedance percentage ('Parameter_Exceedance_Percentage'), the data subset type, the CMIP generation, and the repsective experiment. Data based on ERA5

Supplementary Data 142: Heatwave Characteristic Exceedange Percentages (Tx90p) - Table lists the percentages of projected heatwaves where the projected heatwave characteristic is several times as high as the reference characteristic for CMIP6 multi-model ensemble (MME) SSP2-4.5 for data subdivided by Global Warming Levels (GWLs). The Table shows the region name, dates of the reference event, name of the heatwave

characteristic ('Parameter_Name'), the reference value of the respective characteristic ('Parameter'), the respective reference data set ('Reanalysis'), the total number of heatwaves in the respective sample ('total_HW_num'), the number of heatwaves in the sample exceeding the reference value ('Parameter_Exceedance_Number'), and the exceedance percentage ('Parameter_Exceedance_Percentage'), the data subset type, the CMIP generation, and the repsective experiment. Data based on ERA5

Supplementary Data 143: Heatwave Characteristic Exceedange Percentages (Tx90p) - Table lists the percentages of projected heatwaves where the projected heatwave characteristic is several times as high as the reference characteristic for CMIP6 multi-model ensemble (MME) SSP2-4.5 for data subdivided by Global Warming Levels (GWLs). The Table shows the region name, dates of the reference event, name of the heatwave characteristic ('Parameter_Name'), the reference value of the respective characteristic ('Parameter'), the respective reference data set ('Reanalysis'), the total number of heatwaves in the respective sample ('total_HW_num'), the number of heatwaves in the sample exceeding the reference value ('Parameter_Exceedance_Number'), and the exceedance percentage ('Parameter_Exceedance_Percentage'), the data subset type, the CMIP generation, and the repsective experiment. Data based on ERA5

Supplementary Data 144: Heatwave Characteristic Exceedange Percentages (Tx90p) - Table lists the percentages of projected heatwaves where the projected heatwave characteristic is several times as high as the reference characteristic for CMIP6 multi-model ensemble (MME) SSP2-4.5 for data subdivided by Global Warming Levels (GWLs). The Table shows the region name, dates of the reference event, name of the heatwave characteristic ('Parameter_Name'), the reference value of the respective characteristic ('Parameter'), the respective reference data set ('Reanalysis'), the total number of heatwaves in the respective sample ('total_HW_num'), the number of heatwaves in the sample exceeding the reference value ('Parameter_Exceedance_Number'), and the exceedance percentage ('Parameter_Exceedance_Percentage'), the data subset type, the CMIP generation, and the repsective experiment. Data based on ERA5

Supplementary Data 145: Heatwave Characteristic Exceedange Percentages (Tx90p) - Table lists the percentages of projected heatwaves where the projected heatwave characteristic is several times as high as the reference characteristic for CMIP6 multi-model ensemble (MME) SSP3-7.0 for data subdivided by Global Warming Levels (GWLs). The Table shows the region name, dates of the reference event, name of the heatwave characteristic ('Parameter_Name'), the reference value of the respective characteristic ('Parameter'), the respective reference data set ('Reanalysis'), the total number of heatwaves in the respective sample ('total_HW_num'), the number of heatwaves in the sample exceeding the reference value ('Parameter_Exceedance_Number'), and the exceedance percentage ('Parameter_Exceedance_Percentage'), the data subset type, the CMIP generation, and the repsective experiment. Data based on ERA5

Supplementary Data 146: Heatwave Characteristic Exceedange Percentages (Tx90p) - Table lists the percentages of projected heatwaves where the projected heatwave characteristic is several times as high as the reference characteristic for CMIP6 multi-model ensemble (MME) SSP3-7.0 for data subdivided by Global Warming Levels (GWLs). The Table shows the region name, dates of the reference event, name of the heatwave characteristic ('Parameter_Name'), the reference value of the respective characteristic ('Parameter'), the respective reference data set ('Reanalysis'), the total number of heatwaves in the respective sample ('total_HW_num'), the number of heatwaves in the sample exceeding the reference value

('Parameter_Exceedance_Number'), and the exceedance percentage ('Parameter_Exceedance_Percentage'), the data subset type, the CMIP generation, and the repsective experiment. Data based on ERA5

Supplementary Data 147: Heatwave Characteristic Exceedange Percentages (Tx90p) - Table lists the percentages of projected heatwaves where the projected heatwave characteristic is several times as high as the reference characteristic for CMIP6 multi-model ensemble (MME) SSP3-7.0 for data subdivided by Global Warming Levels (GWLs). The Table shows the region name, dates of the reference event, name of the heatwave characteristic ('Parameter_Name'), the reference value of the respective characteristic ('Parameter'), the respective reference data set ('Reanalysis'), the total number of heatwaves in the respective sample ('total_HW_num'), the number of heatwaves in the sample exceeding the reference value ('Parameter_Exceedance_Number'), and the exceedance percentage ('Parameter_Exceedance_Percentage'), the data subset type, the CMIP generation, and the repsective experiment. Data based on ERA5

Supplementary Data 148: Heatwave Characteristic Exceedange Percentages (Tx90p) - Table lists the percentages of projected heatwaves where the projected heatwave characteristic is several times as high as the reference characteristic for CMIP6 multi-model ensemble (MME) SSP3-7.0 for data subdivided by Global Warming Levels (GWLs). The Table shows the region name, dates of the reference event, name of the heatwave characteristic ('Parameter_Name'), the reference value of the respective characteristic ('Parameter'), the respective reference data set ('Reanalysis'), the total number of heatwaves in the respective sample ('total_HW_num'), the number of heatwaves in the sample exceeding the reference value ('Parameter_Exceedance_Number'), and the exceedance percentage ('Parameter_Exceedance_Percentage'), the data subset type, the CMIP generation, and the repsective experiment. Data based on ERA5

Supplementary Data 149: Heatwave Characteristic Exceedange Percentages (Tx90p) - Table lists the percentages of projected heatwaves where the projected heatwave characteristic is several times as high as the reference characteristic for CMIP6 multi-model ensemble (MME) SSP3-7.0 for data subdivided by Global Warming Levels (GWLs). The Table shows the region name, dates of the reference event, name of the heatwave characteristic ('Parameter_Name'), the reference value of the respective characteristic ('Parameter'), the respective reference data set ('Reanalysis'), the total number of heatwaves in the respective sample ('total_HW_num'), the number of heatwaves in the sample exceeding the reference value ('Parameter_Exceedance_Number'), and the exceedance percentage ('Parameter_Exceedance_Percentage'), the data subset type, the CMIP generation, and the repsective experiment. Data based on ERA5

Supplementary Data 150: Heatwave Characteristic Exceedange Percentages (Tx90p) - Table lists the percentages of projected heatwaves where the projected heatwave characteristic is several times as high as the reference characteristic for CMIP6 multi-model ensemble (MME) SSP3-7.0 for data subdivided by Global Warming Levels (GWLs). The Table shows the region name, dates of the reference event, name of the heatwave characteristic ('Parameter_Name'), the reference value of the respective characteristic ('Parameter'), the respective reference data set ('Reanalysis'), the total number of heatwaves in the respective sample ('total_HW_num'), the number of heatwaves in the sample exceeding the reference value ('Parameter_Exceedance_Number'), and the exceedance percentage ('Parameter_Exceedance_Percentage'), the data subset type, the CMIP generation, and the repsective experiment. Data based on ERA5

Supplementary Data 151: Heatwave Characteristic Exceedange Percentages (Tx90p) - Table lists the percentages of projected heatwaves where the projected heatwave characteristic is several times as high as the reference characteristic for CMIP6 multi-model ensemble (MME) SSP5-8.5 for data subdivided by Global Warming Levels (GWLs). The Table shows the region name, dates of the reference event, name of the heatwave characteristic ('Parameter_Name'), the reference value of the respective characteristic ('Parameter'), the respective reference data set ('Reanalysis'), the total number of heatwaves in the respective sample ('total_HW_num'), the number of heatwaves in the sample exceeding the reference value ('Parameter_Exceedance_Number'), and the exceedance percentage ('Parameter_Exceedance_Percentage'), the data subset type, the CMIP generation, and the repsective experiment. Data based on ERA5

Supplementary Data 152: Heatwave Characteristic Exceedange Percentages (Tx90p) - Table lists the percentages of projected heatwaves where the projected heatwave characteristic is several times as high as the reference characteristic for CMIP6 multi-model ensemble (MME) SSP5-8.5 for data subdivided by Global Warming Levels (GWLs). The Table shows the region name, dates of the reference event, name of the heatwave characteristic ('Parameter_Name'), the reference value of the respective characteristic ('Parameter'), the respective reference data set ('Reanalysis'), the total number of heatwaves in the respective sample ('total_HW_num'), the number of heatwaves in the sample exceeding the reference value ('Parameter_Exceedance_Number'), and the exceedance percentage ('Parameter_Exceedance_Percentage'), the data subset type, the CMIP generation, and the repsective experiment. Data based on ERA5

Supplementary Data 153: Heatwave Characteristic Exceedange Percentages (Tx90p) - Table lists the percentages of projected heatwaves where the projected heatwave characteristic is several times as high as the reference characteristic for CMIP6 multi-model ensemble (MME) SSP5-8.5 for data subdivided by Global Warming Levels (GWLs). The Table shows the region name, dates of the reference event, name of the heatwave characteristic ('Parameter_Name'), the reference value of the respective characteristic ('Parameter'), the respective reference data set ('Reanalysis'), the total number of heatwaves in the respective sample ('total_HW_num'), the number of heatwaves in the sample exceeding the reference value ('Parameter_Exceedance_Number'), and the exceedance percentage ('Parameter_Exceedance_Percentage'), the data subset type, the CMIP generation, and the repsective experiment. Data based on ERA5

Supplementary Data 154: Heatwave Characteristic Exceedange Percentages (Tx90p) - Table lists the percentages of projected heatwaves where the projected heatwave characteristic is several times as high as the reference characteristic for CMIP6 multi-model ensemble (MME) SSP5-8.5 for data subdivided by Global Warming Levels (GWLs). The Table shows the region name, dates of the reference event, name of the heatwave characteristic ('Parameter_Name'), the reference value of the respective characteristic ('Parameter'), the respective reference data set ('Reanalysis'), the total number of heatwaves in the respective sample ('total_HW_num'), the number of heatwaves in the sample exceeding the reference value ('Parameter_Exceedance_Number'), and the exceedance percentage ('Parameter_Exceedance_Percentage'), the data subset type, the CMIP generation, and the repsective experiment. Data based on ERA5

Supplementary Data 155: Heatwave Characteristic Exceedange Percentages (Tx90p) - Table lists the percentages of projected heatwaves where the projected heatwave characteristic is several times as high as the reference characteristic for CMIP6 multi-model ensemble (MME) SSP5-8.5 for data subdivided by Global Warming Levels (GWLs). The Table shows the region name, dates of the reference event, name of the heatwave

characteristic ('Parameter_Name'), the reference value of the respective characteristic ('Parameter'), the respective reference data set ('Reanalysis'), the total number of heatwaves in the respective sample ('total_HW_num'), the number of heatwaves in the sample exceeding the reference value ('Parameter_Exceedance_Number'), and the exceedance percentage ('Parameter_Exceedance_Percentage'), the data subset type, the CMIP generation, and the repsective experiment. Data based on ERA5

Supplementary Data 156: Heatwave Characteristic Exceedange Percentages (Tx90p) - Table lists the percentages of projected heatwaves where the projected heatwave characteristic is several times as high as the reference characteristic for CMIP6 multi-model ensemble (MME) SSP5-8.5 for data subdivided by Global Warming Levels (GWLs). The Table shows the region name, dates of the reference event, name of the heatwave characteristic ('Parameter_Name'), the reference value of the respective characteristic ('Parameter'), the respective reference data set ('Reanalysis'), the total number of heatwaves in the respective sample ('total_HW_num'), the number of heatwaves in the sample exceeding the reference value ('Parameter_Exceedance_Number'), and the exceedance percentage ('Parameter_Exceedance_Percentage'), the data subset type, the CMIP generation, and the repsective experiment. Data based on ERA5

Supplementary Data 157: Heatwave Characteristic Exceedange Percentages (Tx90p) - Table lists the percentages of projected heatwaves where the projected heatwave characteristic is several times as high as the reference characteristic for CMIP6 multi-model ensemble (MME) SSP1-2.6 for entire dataset from 2020 to 2100. The Table shows the region name, dates of the reference event, name of the heatwave characteristic ('Parameter_Name'), the reference value of the respective characteristic ('Parameter'), the respective reference data set ('Reanalysis'), the total number of heatwaves in the respective sample ('total_HW_num'), the number of heatwaves in the sample exceeding the reference value ('Parameter_Exceedance_Number'), and the exceedance percentage ('Parameter_Exceedance_Percentage'), the data subset type, the CMIP generation, and the repsective experiment. Data based on ERA5

Supplementary Data 158: Heatwave Characteristic Exceedange Percentages (Tx90p) - Table lists the percentages of projected heatwaves where the projected heatwave characteristic is several times as high as the reference characteristic for CMIP6 multi-model ensemble (MME) SSP1-2.6 for entire dataset from 2020 to 2100. The Table shows the region name, dates of the reference event, name of the heatwave characteristic ('Parameter_Name'), the reference value of the respective characteristic ('Parameter'), the respective reference data set ('Reanalysis'), the total number of heatwaves in the respective sample ('total_HW_num'), the number of heatwaves in the sample exceeding the reference value ('Parameter_Exceedance_Number'), and the exceedance percentage ('Parameter_Exceedance_Percentage'), the data subset type, the CMIP generation, and the repsective experiment. Data based on ERA5

Supplementary Data 159: Heatwave Characteristic Exceedange Percentages (Tx90p) - Table lists the percentages of projected heatwaves where the projected heatwave characteristic is several times as high as the reference characteristic for CMIP6 multi-model ensemble (MME) SSP1-2.6 for entire dataset from 2020 to 2100. The Table shows the region name, dates of the reference event, name of the heatwave characteristic ('Parameter_Name'), the reference value of the respective characteristic ('Parameter'), the respective reference data set ('Reanalysis'), the total number of heatwaves in the respective sample ('total_HW_num'), the number of heatwaves in the sample exceeding the reference value ('Parameter_Exceedance_Number'), and the

exceedance percentage ('Parameter_Exceedance_Percentage'), the data subset type, the CMIP generation, and the repsective experiment. Data based on ERA5

Supplementary Data 160: Heatwave Characteristic Exceedange Percentages (Tx90p) - Table lists the percentages of projected heatwaves where the projected heatwave characteristic is several times as high as the reference characteristic for CMIP6 multi-model ensemble (MME) SSP1-2.6 for entire dataset from 2020 to 2100. The Table shows the region name, dates of the reference event, name of the heatwave characteristic ('Parameter_Name'), the reference value of the respective characteristic ('Parameter'), the respective reference data set ('Reanalysis'), the total number of heatwaves in the respective sample ('total_HW_num'), the number of heatwaves in the sample exceeding the reference value ('Parameter_Exceedance_Number'), and the exceedance percentage ('Parameter_Exceedance_Percentage'), the data subset type, the CMIP generation, and the repsective experiment. Data based on ERA5

Supplementary Data 161: Heatwave Characteristic Exceedange Percentages (Tx90p) - Table lists the percentages of projected heatwaves where the projected heatwave characteristic is several times as high as the reference characteristic for CMIP6 multi-model ensemble (MME) SSP1-2.6 for entire dataset from 2020 to 2100. The Table shows the region name, dates of the reference event, name of the heatwave characteristic ('Parameter_Name'), the reference value of the respective characteristic ('Parameter'), the respective reference data set ('Reanalysis'), the total number of heatwaves in the respective sample ('total_HW_num'), the number of heatwaves in the sample exceeding the reference value ('Parameter_Exceedance_Number'), and the exceedance percentage ('Parameter_Exceedance_Percentage'), the data subset type, the CMIP generation, and the repsective experiment. Data based on ERA5

Supplementary Data 162: Heatwave Characteristic Exceedange Percentages (Tx90p) - Table lists the percentages of projected heatwaves where the projected heatwave characteristic is several times as high as the reference characteristic for CMIP6 multi-model ensemble (MME) SSP1-2.6 for entire dataset from 2020 to 2100. The Table shows the region name, dates of the reference event, name of the heatwave characteristic ('Parameter_Name'), the reference value of the respective characteristic ('Parameter'), the respective reference data set ('Reanalysis'), the total number of heatwaves in the respective sample ('total_HW_num'), the number of heatwaves in the sample exceeding the reference value ('Parameter_Exceedance_Number'), and the exceedance percentage ('Parameter_Exceedance_Percentage'), the data subset type, the CMIP generation, and the repsective experiment. Data based on ERA5

Supplementary Data 163: Heatwave Characteristic Exceedange Percentages (Tx90p) - Table lists the percentages of projected heatwaves where the projected heatwave characteristic is several times as high as the reference characteristic for CMIP6 multi-model ensemble (MME) SSP2-4.5 for entire dataset from 2020 to 2100. The Table shows the region name, dates of the reference event, name of the heatwave characteristic ('Parameter_Name'), the reference value of the respective characteristic ('Parameter'), the respective reference data set ('Reanalysis'), the total number of heatwaves in the respective sample ('total_HW_num'), the number of heatwaves in the sample exceeding the reference value ('Parameter_Exceedance_Number'), and the exceedance percentage ('Parameter_Exceedance_Percentage'), the data subset type, the CMIP generation, and the repsective experiment. Data based on ERA5

Supplementary Data 164: Heatwave Characteristic Exceedange Percentages (Tx90p) - Table lists the percentages of projected heatwaves where the projected heatwave characteristic is several times as high as the reference characteristic for CMIP6 multi-model ensemble (MME) SSP2-4.5 for entire dataset from 2020 to 2100. The Table shows the region name, dates of the reference event, name of the heatwave characteristic ('Parameter_Name'), the reference value of the respective characteristic ('Parameter'), the respective reference data set ('Reanalysis'), the total number of heatwaves in the respective sample ('total_HW_num'), the number of heatwaves in the sample exceeding the reference value ('Parameter_Exceedance_Number'), and the exceedance percentage ('Parameter_Exceedance_Percentage'), the data subset type, the CMIP generation, and the repsective experiment. Data based on ERA5

Supplementary Data 165: Heatwave Characteristic Exceedange Percentages (Tx90p) - Table lists the percentages of projected heatwaves where the projected heatwave characteristic is several times as high as the reference characteristic for CMIP6 multi-model ensemble (MME) SSP2-4.5 for entire dataset from 2020 to 2100. The Table shows the region name, dates of the reference event, name of the heatwave characteristic ('Parameter_Name'), the reference value of the respective characteristic ('Parameter'), the respective reference data set ('Reanalysis'), the total number of heatwaves in the respective sample ('total_HW_num'), the number of heatwaves in the sample exceeding the reference value ('Parameter_Exceedance_Number'), and the exceedance percentage ('Parameter_Exceedance_Percentage'), the data subset type, the CMIP generation, and the repsective experiment. Data based on ERA5

Supplementary Data 166: Heatwave Characteristic Exceedange Percentages (Tx90p) - Table lists the percentages of projected heatwaves where the projected heatwave characteristic is several times as high as the reference characteristic for CMIP6 multi-model ensemble (MME) SSP2-4.5 for entire dataset from 2020 to 2100. The Table shows the region name, dates of the reference event, name of the heatwave characteristic ('Parameter_Name'), the reference value of the respective characteristic ('Parameter'), the respective reference data set ('Reanalysis'), the total number of heatwaves in the respective sample ('total_HW_num'), the number of heatwaves in the sample exceeding the reference value ('Parameter_Exceedance_Number'), and the exceedance percentage ('Parameter_Exceedance_Percentage'), the data subset type, the CMIP generation, and the repsective experiment. Data based on ERA5

Supplementary Data 167: Heatwave Characteristic Exceedange Percentages (Tx90p) - Table lists the percentages of projected heatwaves where the projected heatwave characteristic is several times as high as the reference characteristic for CMIP6 multi-model ensemble (MME) SSP2-4.5 for entire dataset from 2020 to 2100. The Table shows the region name, dates of the reference event, name of the heatwave characteristic ('Parameter_Name'), the reference value of the respective characteristic ('Parameter'), the respective reference data set ('Reanalysis'), the total number of heatwaves in the respective sample ('total_HW_num'), the number of heatwaves in the sample exceeding the reference value ('Parameter_Exceedance_Number'), and the exceedance percentage ('Parameter_Exceedance_Percentage'), the data subset type, the CMIP generation, and the repsective experiment. Data based on ERA5

Supplementary Data 168: Heatwave Characteristic Exceedange Percentages (Tx90p) - Table lists the percentages of projected heatwaves where the projected heatwave characteristic is several times as high as the reference characteristic for CMIP6 multi-model ensemble (MME) SSP2-4.5 for entire dataset from 2020 to 2100. The Table shows the region name, dates of the reference event, name of the heatwave characteristic

('Parameter_Name'), the reference value of the respective characteristic ('Parameter'), the respective reference data set ('Reanalysis'), the total number of heatwaves in the respective sample ('total_HW_num'), the number of heatwaves in the sample exceeding the reference value ('Parameter_Exceedance_Number'), and the exceedance percentage ('Parameter_Exceedance_Percentage'), the data subset type, the CMIP generation, and the repsective experiment. Data based on ERA5

Supplementary Data 169: Heatwave Characteristic Exceedange Percentages (Tx90p) - Table lists the percentages of projected heatwaves where the projected heatwave characteristic is several times as high as the reference characteristic for CMIP6 multi-model ensemble (MME) SSP3-7.0 for entire dataset from 2020 to 2100. The Table shows the region name, dates of the reference event, name of the heatwave characteristic ('Parameter_Name'), the reference value of the respective characteristic ('Parameter'), the respective reference data set ('Reanalysis'), the total number of heatwaves in the respective sample ('total_HW_num'), the number of heatwaves in the sample exceeding the reference value ('Parameter_Exceedance_Number'), and the exceedance percentage ('Parameter_Exceedance_Percentage'), the data subset type, the CMIP generation, and the repsective experiment. Data based on ERA5

Supplementary Data 170: Heatwave Characteristic Exceedange Percentages (Tx90p) - Table lists the percentages of projected heatwaves where the projected heatwave characteristic is several times as high as the reference characteristic for CMIP6 multi-model ensemble (MME) SSP3-7.0 for entire dataset from 2020 to 2100. The Table shows the region name, dates of the reference event, name of the heatwave characteristic ('Parameter_Name'), the reference value of the respective characteristic ('Parameter'), the respective reference data set ('Reanalysis'), the total number of heatwaves in the respective sample ('total_HW_num'), the number of heatwaves in the sample exceeding the reference value ('Parameter_Exceedance_Number'), and the exceedance percentage ('Parameter_Exceedance_Percentage'), the data subset type, the CMIP generation, and the repsective experiment. Data based on ERA5

Supplementary Data 171: Heatwave Characteristic Exceedange Percentages (Tx90p) - Table lists the percentages of projected heatwaves where the projected heatwave characteristic is several times as high as the reference characteristic for CMIP6 multi-model ensemble (MME) SSP3-7.0 for entire dataset from 2020 to 2100. The Table shows the region name, dates of the reference event, name of the heatwave characteristic ('Parameter_Name'), the reference value of the respective characteristic ('Parameter'), the respective reference data set ('Reanalysis'), the total number of heatwaves in the respective sample ('total_HW_num'), the number of heatwaves in the sample exceeding the reference value ('Parameter_Exceedance_Number'), and the exceedance percentage ('Parameter_Exceedance_Percentage'), the data subset type, the CMIP generation, and the repsective experiment. Data based on ERA5

Supplementary Data 172: Heatwave Characteristic Exceedange Percentages (Tx90p) - Table lists the percentages of projected heatwaves where the projected heatwave characteristic is several times as high as the reference characteristic for CMIP6 multi-model ensemble (MME) SSP3-7.0 for entire dataset from 2020 to 2100. The Table shows the region name, dates of the reference event, name of the heatwave characteristic ('Parameter_Name'), the reference value of the respective characteristic ('Parameter'), the respective reference data set ('Reanalysis'), the total number of heatwaves in the respective sample ('total_HW_num'), the number of heatwaves in the sample exceeding the reference value ('Parameter_Exceedance_Number'), and the

exceedance percentage ('Parameter_Exceedance_Percentage'), the data subset type, the CMIP generation, and the repsective experiment. Data based on ERA5

Supplementary Data 173: Heatwave Characteristic Exceedange Percentages (Tx90p) - Table lists the percentages of projected heatwaves where the projected heatwave characteristic is several times as high as the reference characteristic for CMIP6 multi-model ensemble (MME) SSP3-7.0 for entire dataset from 2020 to 2100. The Table shows the region name, dates of the reference event, name of the heatwave characteristic ('Parameter_Name'), the reference value of the respective characteristic ('Parameter'), the respective reference data set ('Reanalysis'), the total number of heatwaves in the respective sample ('total_HW_num'), the number of heatwaves in the sample exceeding the reference value ('Parameter_Exceedance_Number'), and the exceedance percentage ('Parameter_Exceedance_Percentage'), the data subset type, the CMIP generation, and the repsective experiment. Data based on ERA5

Supplementary Data 174: Heatwave Characteristic Exceedange Percentages (Tx90p) - Table lists the percentages of projected heatwaves where the projected heatwave characteristic is several times as high as the reference characteristic for CMIP6 multi-model ensemble (MME) SSP3-7.0 for entire dataset from 2020 to 2100. The Table shows the region name, dates of the reference event, name of the heatwave characteristic ('Parameter_Name'), the reference value of the respective characteristic ('Parameter'), the respective reference data set ('Reanalysis'), the total number of heatwaves in the respective sample ('total_HW_num'), the number of heatwaves in the sample exceeding the reference value ('Parameter_Exceedance_Number'), and the exceedance percentage ('Parameter_Exceedance_Percentage'), the data subset type, the CMIP generation, and the repsective experiment. Data based on ERA5

Supplementary Data 175: Heatwave Characteristic Exceedange Percentages (Tx90p) - Table lists the percentages of projected heatwaves where the projected heatwave characteristic is several times as high as the reference characteristic for CMIP6 multi-model ensemble (MME) SSP5-8.5 for entire dataset from 2020 to 2100. The Table shows the region name, dates of the reference event, name of the heatwave characteristic ('Parameter_Name'), the reference value of the respective characteristic ('Parameter'), the respective reference data set ('Reanalysis'), the total number of heatwaves in the respective sample ('total_HW_num'), the number of heatwaves in the sample exceeding the reference value ('Parameter_Exceedance_Number'), and the exceedance percentage ('Parameter_Exceedance_Percentage'), the data subset type, the CMIP generation, and the repsective experiment. Data based on ERA5

Supplementary Data 176: Heatwave Characteristic Exceedange Percentages (Tx90p) - Table lists the percentages of projected heatwaves where the projected heatwave characteristic is several times as high as the reference characteristic for CMIP6 multi-model ensemble (MME) SSP5-8.5 for entire dataset from 2020 to 2100. The Table shows the region name, dates of the reference event, name of the heatwave characteristic ('Parameter_Name'), the reference value of the respective characteristic ('Parameter'), the respective reference data set ('Reanalysis'), the total number of heatwaves in the respective sample ('total_HW_num'), the number of heatwaves in the sample exceeding the reference value ('Parameter_Exceedance_Number'), and the exceedance percentage ('Parameter_Exceedance_Percentage'), the data subset type, the CMIP generation, and the repsective experiment. Data based on ERA5

Supplementary Data 177: Heatwave Characteristic Exceedange Percentages (Tx90p) - Table lists the percentages of projected heatwaves where the projected heatwave characteristic is several times as high as the reference characteristic for CMIP6 multi-model ensemble (MME) SSP5-8.5 for entire dataset from 2020 to 2100. The Table shows the region name, dates of the reference event, name of the heatwave characteristic ('Parameter_Name'), the reference value of the respective characteristic ('Parameter'), the respective reference data set ('Reanalysis'), the total number of heatwaves in the respective sample ('total_HW_num'), the number of heatwaves in the sample exceeding the reference value ('Parameter_Exceedance_Number'), and the exceedance percentage ('Parameter_Exceedance_Percentage'), the data subset type, the CMIP generation, and the repsective experiment. Data based on ERA5

Supplementary Data 178: Heatwave Characteristic Exceedange Percentages (Tx90p) - Table lists the percentages of projected heatwaves where the projected heatwave characteristic is several times as high as the reference characteristic for CMIP6 multi-model ensemble (MME) SSP5-8.5 for entire dataset from 2020 to 2100. The Table shows the region name, dates of the reference event, name of the heatwave characteristic ('Parameter_Name'), the reference value of the respective characteristic ('Parameter'), the respective reference data set ('Reanalysis'), the total number of heatwaves in the respective sample ('total_HW_num'), the number of heatwaves in the sample exceeding the reference value ('Parameter_Exceedance_Number'), and the exceedance percentage ('Parameter_Exceedance_Percentage'), the data subset type, the CMIP generation, and the repsective experiment. Data based on ERA5

Supplementary Data 179: Heatwave Characteristic Exceedange Percentages (Tx90p) - Table lists the percentages of projected heatwaves where the projected heatwave characteristic is several times as high as the reference characteristic for CMIP6 multi-model ensemble (MME) SSP5-8.5 for entire dataset from 2020 to 2100. The Table shows the region name, dates of the reference event, name of the heatwave characteristic ('Parameter_Name'), the reference value of the respective characteristic ('Parameter'), the respective reference data set ('Reanalysis'), the total number of heatwaves in the respective sample ('total_HW_num'), the number of heatwaves in the sample exceeding the reference value ('Parameter_Exceedance_Number'), and the exceedance percentage ('Parameter_Exceedance_Percentage'), the data subset type, the CMIP generation, and the repsective experiment. Data based on ERA5

Supplementary Data 180: Heatwave Characteristic Exceedange Percentages (Tx90p) - Table lists the percentages of projected heatwaves where the projected heatwave characteristic is several times as high as the reference characteristic for CMIP6 multi-model ensemble (MME) SSP5-8.5 for entire dataset from 2020 to 2100. The Table shows the region name, dates of the reference event, name of the heatwave characteristic ('Parameter_Name'), the reference value of the respective characteristic ('Parameter'), the respective reference data set ('Reanalysis'), the total number of heatwaves in the respective sample ('total_HW_num'), the number of heatwaves in the sample exceeding the reference value ('Parameter_Exceedance_Number'), and the exceedance percentage ('Parameter_Exceedance_Percentage'), the data subset type, the CMIP generation, and the repsective experiment. Data based on ERA5

Supplementary Data 181: Heatwave Characteristic Exceedange Percentages (Tx90p) - Table lists the shifts in exceedance percentages of the respective heatwave characteristic per data subdivided by decade for CMIP6 multi-model ensemble (MME) SSP1-2.6. The Table shows the region name, dates of the reference event, name of the heatwave characteristic ('Parameter_Name'), the reference value of the respective characteristic

('Parameter'), the respective reference data set ('Reanalysis'), the total number of heatwaves in the respective sample ('total_HW_num'), the number of heatwaves in the sample exceeding the reference value ('Parameter_Exceedance_Number'), and the exceedance percentage ('Parameter_Exceedance_Percentage'), the data subset type, the CMIP generation, and the repsective experiment. Data based on ERA5

Supplementary Data 182: Heatwave Characteristic Exceedange Percentages (Tx90p) - Table lists the shifts in exceedance percentages of the respective heatwave characteristic per data subdivided by decade for CMIP6 multi-model ensemble (MME) SSP1-2.6. The Table shows the region name, dates of the reference event, name of the heatwave characteristic ('Parameter_Name'), the reference value of the respective characteristic ('Parameter'), the respective reference data set ('Reanalysis'), the total number of heatwaves in the respective sample ('total_HW_num'), the number of heatwaves in the sample exceeding the reference value ('Parameter_Exceedance_Number'), and the exceedance percentage ('Parameter_Exceedance_Percentage'), the data subset type, the CMIP generation, and the repsective experiment. Data based on ERA5

Supplementary Data 183: Heatwave Characteristic Exceedange Percentages (Tx90p) - Table lists the shifts in exceedance percentages of the respective heatwave characteristic per data subdivided by decade for CMIP6 multi-model ensemble (MME) SSP1-2.6. The Table shows the region name, dates of the reference event, name of the heatwave characteristic ('Parameter_Name'), the reference value of the respective characteristic ('Parameter'), the respective reference data set ('Reanalysis'), the total number of heatwaves in the respective sample ('total_HW_num'), the number of heatwaves in the sample exceeding the reference value ('Parameter_Exceedance_Number'), and the exceedance percentage ('Parameter_Exceedance_Percentage'), the data subset type, the CMIP generation, and the repsective experiment. Data based on ERA5

Supplementary Data 184: Heatwave Characteristic Exceedange Percentages (Tx90p) - Table lists the shifts in exceedance percentages of the respective heatwave characteristic per data subdivided by decade for CMIP6 multi-model ensemble (MME) SSP1-2.6. The Table shows the region name, dates of the reference event, name of the heatwave characteristic ('Parameter_Name'), the reference value of the respective characteristic ('Parameter'), the respective reference data set ('Reanalysis'), the total number of heatwaves in the respective sample ('total_HW_num'), the number of heatwaves in the sample exceeding the reference value ('Parameter_Exceedance_Number'), and the exceedance percentage ('Parameter_Exceedance_Percentage'), the data subset type, the CMIP generation, and the repsective experiment. Data based on ERA5

Supplementary Data 185: Heatwave Characteristic Exceedange Percentages (Tx90p) - Table lists the shifts in exceedance percentages of the respective heatwave characteristic per data subdivided by decade for CMIP6 multi-model ensemble (MME) SSP1-2.6. The Table shows the region name, dates of the reference event, name of the heatwave characteristic ('Parameter_Name'), the reference value of the respective characteristic ('Parameter'), the respective reference data set ('Reanalysis'), the total number of heatwaves in the respective sample ('total_HW_num'), the number of heatwaves in the sample exceeding the reference value ('Parameter_Exceedance_Number'), and the exceedance percentage ('Parameter_Exceedance_Percentage'), the data subset type, the CMIP generation, and the repsective experiment. Data based on ERA5

Supplementary Data 186: Heatwave Characteristic Exceedange Percentages (Tx90p) - Table lists the shifts in exceedance percentages of the respective heatwave characteristic per data subdivided by decade for CMIP6

multi-model ensemble (MME) SSP1-2.6. The Table shows the region name, dates of the reference event, name of the heatwave characteristic ('Parameter_Name'), the reference value of the respective characteristic ('Parameter'), the respective reference data set ('Reanalysis'), the total number of heatwaves in the respective sample ('total_HW_num'), the number of heatwaves in the sample exceeding the reference value ('Parameter_Exceedance_Number'), and the exceedance percentage ('Parameter_Exceedance_Percentage'), the data subset type, the CMIP generation, and the repsective experiment. Data based on ERA5

Supplementary Data 187: Heatwave Characteristic Exceedange Percentages (Tx90p) - Table lists the shifts in exceedance percentages of the respective heatwave characteristic per data subdivided by decade for CMIP6 multi-model ensemble (MME) SSP1-2.6. The Table shows the region name, dates of the reference event, name of the heatwave characteristic ('Parameter_Name'), the reference value of the respective characteristic ('Parameter'), the respective reference data set ('Reanalysis'), the total number of heatwaves in the respective sample ('total_HW_num'), the number of heatwaves in the sample exceeding the reference value ('Parameter_Exceedance_Number'), and the exceedance percentage ('Parameter_Exceedance_Percentage'), the data subset type, the CMIP generation, and the repsective experiment. Data based on ERA5

Supplementary Data 188: Heatwave Characteristic Exceedange Percentages (Tx90p) - Table lists the shifts in exceedance percentages of the respective heatwave characteristic per data subdivided by decade for CMIP6 multi-model ensemble (MME) SSP1-2.6. The Table shows the region name, dates of the reference event, name of the heatwave characteristic ('Parameter_Name'), the reference value of the respective characteristic ('Parameter'), the respective reference data set ('Reanalysis'), the total number of heatwaves in the respective sample ('total_HW_num'), the number of heatwaves in the sample exceeding the reference value ('Parameter_Exceedance_Number'), and the exceedance percentage ('Parameter_Exceedance_Percentage'), the data subset type, the CMIP generation, and the repsective experiment. Data based on ERA5

Supplementary Data 189: Heatwave Characteristic Exceedange Percentages (Tx90p) - Table lists the shifts in exceedance percentages of the respective heatwave characteristic per data subdivided by decade for CMIP6 multi-model ensemble (MME) SSP1-2.6. The Table shows the region name, dates of the reference event, name of the heatwave characteristic ('Parameter_Name'), the reference value of the respective characteristic ('Parameter'), the respective reference data set ('Reanalysis'), the total number of heatwaves in the respective sample ('total_HW_num'), the number of heatwaves in the sample exceeding the reference value ('Parameter_Exceedance_Number'), and the exceedance percentage ('Parameter_Exceedance_Percentage'), the data subset type, the CMIP generation, and the repsective experiment. Data based on ERA5

Supplementary Data 190: Heatwave Characteristic Exceedange Percentages (Tx90p) - Table lists the shifts in exceedance percentages of the respective heatwave characteristic per data subdivided by decade for CMIP6 multi-model ensemble (MME) SSP1-2.6. The Table shows the region name, dates of the reference event, name of the heatwave characteristic ('Parameter_Name'), the reference value of the respective characteristic ('Parameter'), the respective reference data set ('Reanalysis'), the total number of heatwaves in the respective sample ('total_HW_num'), the number of heatwaves in the sample exceeding the reference value ('Parameter_Exceedance_Number'), and the exceedance percentage ('Parameter_Exceedance_Percentage'), the data subset type, the CMIP generation, and the repsective experiment. Data based on ERA5

Supplementary Data 191: Heatwave Characteristic Exceedange Percentages (Tx90p) - Table lists the shifts in exceedance percentages of the respective heatwave characteristic per data subdivided by decade for CMIP6 multi-model ensemble (MME) SSP1-2.6. The Table shows the region name, dates of the reference event, name of the heatwave characteristic ('Parameter_Name'), the reference value of the respective characteristic ('Parameter'), the respective reference data set ('Reanalysis'), the total number of heatwaves in the respective sample ('total_HW_num'), the number of heatwaves in the sample exceeding the reference value ('Parameter_Exceedance_Number'), and the exceedance percentage ('Parameter_Exceedance_Percentage'), the data subset type, the CMIP generation, and the repsective experiment. Data based on ERA5

Supplementary Data 192: Heatwave Characteristic Exceedange Percentages (Tx90p) - Table lists the shifts in exceedance percentages of the respective heatwave characteristic per data subdivided by decade for CMIP6 multi-model ensemble (MME) SSP1-2.6. The Table shows the region name, dates of the reference event, name of the heatwave characteristic ('Parameter_Name'), the reference value of the respective characteristic ('Parameter'), the respective reference data set ('Reanalysis'), the total number of heatwaves in the respective sample ('total_HW_num'), the number of heatwaves in the sample exceeding the reference value ('Parameter_Exceedance_Number'), and the exceedance percentage ('Parameter_Exceedance_Percentage'), the data subset type, the CMIP generation, and the repsective experiment. Data based on ERA5

Supplementary Data 193: Heatwave Characteristic Exceedange Percentages (Tx90p) - Table lists the shifts in exceedance percentages of the respective heatwave characteristic per data subdivided by decade for CMIP6 multi-model ensemble (MME) SSP2-4.5. The Table shows the region name, dates of the reference event, name of the heatwave characteristic ('Parameter_Name'), the reference value of the respective characteristic ('Parameter'), the respective reference data set ('Reanalysis'), the total number of heatwaves in the respective sample ('total_HW_num'), the number of heatwaves in the sample exceeding the reference value ('Parameter_Exceedance_Number'), and the exceedance percentage ('Parameter_Exceedance_Percentage'), the data subset type, the CMIP generation, and the repsective experiment. Data based on ERA5

Supplementary Data 194: Heatwave Characteristic Exceedange Percentages (Tx90p) - Table lists the shifts in exceedance percentages of the respective heatwave characteristic per data subdivided by decade for CMIP6 multi-model ensemble (MME) SSP2-4.5. The Table shows the region name, dates of the reference event, name of the heatwave characteristic ('Parameter_Name'), the reference value of the respective characteristic ('Parameter'), the respective reference data set ('Reanalysis'), the total number of heatwaves in the respective sample ('total_HW_num'), the number of heatwaves in the sample exceeding the reference value ('Parameter_Exceedance_Number'), and the exceedance percentage ('Parameter_Exceedance_Percentage'), the data subset type, the CMIP generation, and the repsective experiment. Data based on ERA5

Supplementary Data 195: Heatwave Characteristic Exceedange Percentages (Tx90p) - Table lists the shifts in exceedance percentages of the respective heatwave characteristic per data subdivided by decade for CMIP6 multi-model ensemble (MME) SSP2-4.5. The Table shows the region name, dates of the reference event, name of the heatwave characteristic ('Parameter_Name'), the reference value of the respective characteristic ('Parameter'), the respective reference data set ('Reanalysis'), the total number of heatwaves in the respective sample ('total_HW_num'), the number of heatwaves in the sample exceeding the reference value ('Parameter_Exceedance_Number'), and the exceedance percentage ('Parameter_Exceedance_Percentage'), the data subset type, the CMIP generation, and the repsective experiment. Data based on ERA5

Supplementary Data 196: Heatwave Characteristic Exceedange Percentages (Tx90p) - Table lists the shifts in exceedance percentages of the respective heatwave characteristic per data subdivided by decade for CMIP6 multi-model ensemble (MME) SSP2-4.5. The Table shows the region name, dates of the reference event, name of the heatwave characteristic ('Parameter_Name'), the reference value of the respective characteristic ('Parameter'), the respective reference data set ('Reanalysis'), the total number of heatwaves in the respective sample ('total_HW_num'), the number of heatwaves in the sample exceeding the reference value ('Parameter_Exceedance_Number'), and the exceedance percentage ('Parameter_Exceedance_Percentage'), the data subset type, the CMIP generation, and the repsective experiment. Data based on ERA5

Supplementary Data 197: Heatwave Characteristic Exceedange Percentages (Tx90p) - Table lists the shifts in exceedance percentages of the respective heatwave characteristic per data subdivided by decade for CMIP6 multi-model ensemble (MME) SSP2-4.5. The Table shows the region name, dates of the reference event, name of the heatwave characteristic ('Parameter_Name'), the reference value of the respective characteristic ('Parameter'), the respective reference data set ('Reanalysis'), the total number of heatwaves in the respective sample ('total_HW_num'), the number of heatwaves in the sample exceeding the reference value ('Parameter_Exceedance_Number'), and the exceedance percentage ('Parameter_Exceedance_Percentage'), the data subset type, the CMIP generation, and the repsective experiment. Data based on ERA5

Supplementary Data 198: Heatwave Characteristic Exceedange Percentages (Tx90p) - Table lists the shifts in exceedance percentages of the respective heatwave characteristic per data subdivided by decade for CMIP6 multi-model ensemble (MME) SSP2-4.5. The Table shows the region name, dates of the reference event, name of the heatwave characteristic ('Parameter_Name'), the reference value of the respective characteristic ('Parameter'), the respective reference data set ('Reanalysis'), the total number of heatwaves in the respective sample ('total_HW_num'), the number of heatwaves in the sample exceeding the reference value ('Parameter_Exceedance_Number'), and the exceedance percentage ('Parameter_Exceedance_Percentage'), the data subset type, the CMIP generation, and the repsective experiment. Data based on ERA5

Supplementary Data 199: Heatwave Characteristic Exceedange Percentages (Tx90p) - Table lists the shifts in exceedance percentages of the respective heatwave characteristic per data subdivided by decade for CMIP6 multi-model ensemble (MME) SSP2-4.5. The Table shows the region name, dates of the reference event, name of the heatwave characteristic ('Parameter_Name'), the reference value of the respective characteristic ('Parameter'), the respective reference data set ('Reanalysis'), the total number of heatwaves in the respective sample ('total_HW_num'), the number of heatwaves in the sample exceeding the reference value ('Parameter_Exceedance_Number'), and the exceedance percentage ('Parameter_Exceedance_Percentage'), the data subset type, the CMIP generation, and the repsective experiment. Data based on ERA5

Supplementary Data 200: Heatwave Characteristic Exceedange Percentages (Tx90p) - Table lists the shifts in exceedance percentages of the respective heatwave characteristic per data subdivided by decade for CMIP6 multi-model ensemble (MME) SSP2-4.5. The Table shows the region name, dates of the reference event, name of the heatwave characteristic ('Parameter_Name'), the reference value of the respective characteristic ('Parameter'), the respective reference data set ('Reanalysis'), the total number of heatwaves in the respective sample ('total_HW_num'), the number of heatwaves in the sample exceeding the reference value

('Parameter_Exceedance_Number'), and the exceedance percentage ('Parameter_Exceedance_Percentage'), the data subset type, the CMIP generation, and the repsective experiment. Data based on ERA5

Supplementary Data 201: Heatwave Characteristic Exceedange Percentages (Tx90p) - Table lists the shifts in exceedance percentages of the respective heatwave characteristic per data subdivided by decade for CMIP6 multi-model ensemble (MME) SSP2-4.5. The Table shows the region name, dates of the reference event, name of the heatwave characteristic ('Parameter_Name'), the reference value of the respective characteristic ('Parameter'), the respective reference data set ('Reanalysis'), the total number of heatwaves in the respective sample ('total_HW_num'), the number of heatwaves in the sample exceeding the reference value ('Parameter_Exceedance_Number'), and the exceedance percentage ('Parameter_Exceedance_Percentage'), the data subset type, the CMIP generation, and the repsective experiment. Data based on ERA5

Supplementary Data 202: Heatwave Characteristic Exceedange Percentages (Tx90p) - Table lists the shifts in exceedance percentages of the respective heatwave characteristic per data subdivided by decade for CMIP6 multi-model ensemble (MME) SSP2-4.5. The Table shows the region name, dates of the reference event, name of the heatwave characteristic ('Parameter_Name'), the reference value of the respective characteristic ('Parameter'), the respective reference data set ('Reanalysis'), the total number of heatwaves in the respective sample ('total_HW_num'), the number of heatwaves in the sample exceeding the reference value ('Parameter_Exceedance_Number'), and the exceedance percentage ('Parameter_Exceedance_Percentage'), the data subset type, the CMIP generation, and the repsective experiment. Data based on ERA5

Supplementary Data 203: Heatwave Characteristic Exceedange Percentages (Tx90p) - Table lists the shifts in exceedance percentages of the respective heatwave characteristic per data subdivided by decade for CMIP6 multi-model ensemble (MME) SSP2-4.5. The Table shows the region name, dates of the reference event, name of the heatwave characteristic ('Parameter_Name'), the reference value of the respective characteristic ('Parameter'), the respective reference data set ('Reanalysis'), the total number of heatwaves in the respective sample ('total_HW_num'), the number of heatwaves in the sample exceeding the reference value ('Parameter_Exceedance_Number'), and the exceedance percentage ('Parameter_Exceedance_Percentage'), the data subset type, the CMIP generation, and the repsective experiment. Data based on ERA5

Supplementary Data 204: Heatwave Characteristic Exceedange Percentages (Tx90p) - Table lists the shifts in exceedance percentages of the respective heatwave characteristic per data subdivided by decade for CMIP6 multi-model ensemble (MME) SSP2-4.5. The Table shows the region name, dates of the reference event, name of the heatwave characteristic ('Parameter_Name'), the reference value of the respective characteristic ('Parameter'), the respective reference data set ('Reanalysis'), the total number of heatwaves in the respective sample ('total_HW_num'), the number of heatwaves in the sample exceeding the reference value ('Parameter_Exceedance_Number'), and the exceedance percentage ('Parameter_Exceedance_Percentage'), the data subset type, the CMIP generation, and the repsective experiment. Data based on ERA5

Supplementary Data 205: Heatwave Characteristic Exceedange Percentages (Tx90p) - Table lists the shifts in exceedance percentages of the respective heatwave characteristic per data subdivided by decade for CMIP6 multi-model ensemble (MME) SSP3-7.0. The Table shows the region name, dates of the reference event, name of the heatwave characteristic ('Parameter_Name'), the reference value of the respective characteristic

('Parameter'), the respective reference data set ('Reanalysis'), the total number of heatwaves in the respective sample ('total_HW_num'), the number of heatwaves in the sample exceeding the reference value ('Parameter_Exceedance_Number'), and the exceedance percentage ('Parameter_Exceedance_Percentage'), the data subset type, the CMIP generation, and the repsective experiment. Data based on ERA5

Supplementary Data 206: Heatwave Characteristic Exceedange Percentages (Tx90p) - Table lists the shifts in exceedance percentages of the respective heatwave characteristic per data subdivided by decade for CMIP6 multi-model ensemble (MME) SSP3-7.0. The Table shows the region name, dates of the reference event, name of the heatwave characteristic ('Parameter_Name'), the reference value of the respective characteristic ('Parameter'), the respective reference data set ('Reanalysis'), the total number of heatwaves in the respective sample ('total_HW_num'), the number of heatwaves in the sample exceeding the reference value ('Parameter_Exceedance_Number'), and the exceedance percentage ('Parameter_Exceedance_Percentage'), the data subset type, the CMIP generation, and the repsective experiment. Data based on ERA5

Supplementary Data 207: Heatwave Characteristic Exceedange Percentages (Tx90p) - Table lists the shifts in exceedance percentages of the respective heatwave characteristic per data subdivided by decade for CMIP6 multi-model ensemble (MME) SSP3-7.0. The Table shows the region name, dates of the reference event, name of the heatwave characteristic ('Parameter_Name'), the reference value of the respective characteristic ('Parameter'), the respective reference data set ('Reanalysis'), the total number of heatwaves in the respective sample ('total_HW_num'), the number of heatwaves in the sample exceeding the reference value ('Parameter_Exceedance_Number'), and the exceedance percentage ('Parameter_Exceedance_Percentage'), the data subset type, the CMIP generation, and the repsective experiment. Data based on ERA5

Supplementary Data 208: Heatwave Characteristic Exceedange Percentages (Tx90p) - Table lists the shifts in exceedance percentages of the respective heatwave characteristic per data subdivided by decade for CMIP6 multi-model ensemble (MME) SSP3-7.0. The Table shows the region name, dates of the reference event, name of the heatwave characteristic ('Parameter_Name'), the reference value of the respective characteristic ('Parameter'), the respective reference data set ('Reanalysis'), the total number of heatwaves in the respective sample ('total_HW_num'), the number of heatwaves in the sample exceeding the reference value ('Parameter_Exceedance_Number'), and the exceedance percentage ('Parameter_Exceedance_Percentage'), the data subset type, the CMIP generation, and the repsective experiment. Data based on ERA5

Supplementary Data 209: Heatwave Characteristic Exceedange Percentages (Tx90p) - Table lists the shifts in exceedance percentages of the respective heatwave characteristic per data subdivided by decade for CMIP6 multi-model ensemble (MME) SSP3-7.0. The Table shows the region name, dates of the reference event, name of the heatwave characteristic ('Parameter_Name'), the reference value of the respective characteristic ('Parameter'), the respective reference data set ('Reanalysis'), the total number of heatwaves in the respective sample ('total_HW_num'), the number of heatwaves in the sample exceeding the reference value ('Parameter_Exceedance_Number'), and the exceedance percentage ('Parameter_Exceedance_Percentage'), the data subset type, the CMIP generation, and the repsective experiment. Data based on ERA5

Supplementary Data 210: Heatwave Characteristic Exceedange Percentages (Tx90p) - Table lists the shifts in exceedance percentages of the respective heatwave characteristic per data subdivided by decade for CMIP6

multi-model ensemble (MME) SSP3-7.0. The Table shows the region name, dates of the reference event, name of the heatwave characteristic ('Parameter_Name'), the reference value of the respective characteristic ('Parameter'), the respective reference data set ('Reanalysis'), the total number of heatwaves in the respective sample ('total_HW_num'), the number of heatwaves in the sample exceeding the reference value ('Parameter_Exceedance_Number'), and the exceedance percentage ('Parameter_Exceedance_Percentage'), the data subset type, the CMIP generation, and the repsective experiment. Data based on ERA5

Supplementary Data 211: Heatwave Characteristic Exceedange Percentages (Tx90p) - Table lists the shifts in exceedance percentages of the respective heatwave characteristic per data subdivided by decade for CMIP6 multi-model ensemble (MME) SSP3-7.0. The Table shows the region name, dates of the reference event, name of the heatwave characteristic ('Parameter_Name'), the reference value of the respective characteristic ('Parameter'), the respective reference data set ('Reanalysis'), the total number of heatwaves in the respective sample ('total_HW_num'), the number of heatwaves in the sample exceeding the reference value ('Parameter_Exceedance_Number'), and the exceedance percentage ('Parameter_Exceedance_Percentage'), the data subset type, the CMIP generation, and the repsective experiment. Data based on ERA5

Supplementary Data 212: Heatwave Characteristic Exceedange Percentages (Tx90p) - Table lists the shifts in exceedance percentages of the respective heatwave characteristic per data subdivided by decade for CMIP6 multi-model ensemble (MME) SSP3-7.0. The Table shows the region name, dates of the reference event, name of the heatwave characteristic ('Parameter_Name'), the reference value of the respective characteristic ('Parameter'), the respective reference data set ('Reanalysis'), the total number of heatwaves in the respective sample ('total_HW_num'), the number of heatwaves in the sample exceeding the reference value ('Parameter_Exceedance_Number'), and the exceedance percentage ('Parameter_Exceedance_Percentage'), the data subset type, the CMIP generation, and the repsective experiment. Data based on ERA5

Supplementary Data 213: Heatwave Characteristic Exceedange Percentages (Tx90p) - Table lists the shifts in exceedance percentages of the respective heatwave characteristic per data subdivided by decade for CMIP6 multi-model ensemble (MME) SSP3-7.0. The Table shows the region name, dates of the reference event, name of the heatwave characteristic ('Parameter_Name'), the reference value of the respective characteristic ('Parameter'), the respective reference data set ('Reanalysis'), the total number of heatwaves in the respective sample ('total_HW_num'), the number of heatwaves in the sample exceeding the reference value ('Parameter_Exceedance_Number'), and the exceedance percentage ('Parameter_Exceedance_Percentage'), the data subset type, the CMIP generation, and the repsective experiment. Data based on ERA5

Supplementary Data 214: Heatwave Characteristic Exceedange Percentages (Tx90p) - Table lists the shifts in exceedance percentages of the respective heatwave characteristic per data subdivided by decade for CMIP6 multi-model ensemble (MME) SSP3-7.0. The Table shows the region name, dates of the reference event, name of the heatwave characteristic ('Parameter_Name'), the reference value of the respective characteristic ('Parameter'), the respective reference data set ('Reanalysis'), the total number of heatwaves in the respective sample ('total_HW_num'), the number of heatwaves in the sample exceeding the reference value ('Parameter_Exceedance_Number'), and the exceedance percentage ('Parameter_Exceedance_Percentage'), the data subset type, the CMIP generation, and the repsective experiment. Data based on ERA5

Supplementary Data 215: Heatwave Characteristic Exceedange Percentages (Tx90p) - Table lists the shifts in exceedance percentages of the respective heatwave characteristic per data subdivided by decade for CMIP6 multi-model ensemble (MME) SSP3-7.0. The Table shows the region name, dates of the reference event, name of the heatwave characteristic ('Parameter_Name'), the reference value of the respective characteristic ('Parameter'), the respective reference data set ('Reanalysis'), the total number of heatwaves in the respective sample ('total_HW_num'), the number of heatwaves in the sample exceeding the reference value ('Parameter_Exceedance_Number'), and the exceedance percentage ('Parameter_Exceedance_Percentage'), the data subset type, the CMIP generation, and the repsective experiment. Data based on ERA5

Supplementary Data 216: Heatwave Characteristic Exceedange Percentages (Tx90p) - Table lists the shifts in exceedance percentages of the respective heatwave characteristic per data subdivided by decade for CMIP6 multi-model ensemble (MME) SSP3-7.0. The Table shows the region name, dates of the reference event, name of the heatwave characteristic ('Parameter_Name'), the reference value of the respective characteristic ('Parameter'), the respective reference data set ('Reanalysis'), the total number of heatwaves in the respective sample ('total_HW_num'), the number of heatwaves in the sample exceeding the reference value ('Parameter_Exceedance_Number'), and the exceedance percentage ('Parameter_Exceedance_Percentage'), the data subset type, the CMIP generation, and the repsective experiment. Data based on ERA5

Supplementary Data 217: Heatwave Characteristic Exceedange Percentages (Tx90p) - Table lists the shifts in exceedance percentages of the respective heatwave characteristic per data subdivided by decade for CMIP6 multi-model ensemble (MME) SSP5-8.5. The Table shows the region name, dates of the reference event, name of the heatwave characteristic ('Parameter_Name'), the reference value of the respective characteristic ('Parameter'), the respective reference data set ('Reanalysis'), the total number of heatwaves in the respective sample ('total_HW_num'), the number of heatwaves in the sample exceeding the reference value ('Parameter_Exceedance_Number'), and the exceedance percentage ('Parameter_Exceedance_Percentage'), the data subset type, the CMIP generation, and the repsective experiment. Data based on ERA5

Supplementary Data 218: Heatwave Characteristic Exceedange Percentages (Tx90p) - Table lists the shifts in exceedance percentages of the respective heatwave characteristic per data subdivided by decade for CMIP6 multi-model ensemble (MME) SSP5-8.5. The Table shows the region name, dates of the reference event, name of the heatwave characteristic ('Parameter_Name'), the reference value of the respective characteristic ('Parameter'), the respective reference data set ('Reanalysis'), the total number of heatwaves in the respective sample ('total_HW_num'), the number of heatwaves in the sample exceeding the reference value ('Parameter_Exceedance_Number'), and the exceedance percentage ('Parameter_Exceedance_Percentage'), the data subset type, the CMIP generation, and the repsective experiment. Data based on ERA5

Supplementary Data 219: Heatwave Characteristic Exceedange Percentages (Tx90p) - Table lists the shifts in exceedance percentages of the respective heatwave characteristic per data subdivided by decade for CMIP6 multi-model ensemble (MME) SSP5-8.5. The Table shows the region name, dates of the reference event, name of the heatwave characteristic ('Parameter_Name'), the reference value of the respective characteristic ('Parameter'), the respective reference data set ('Reanalysis'), the total number of heatwaves in the respective sample ('total_HW_num'), the number of heatwaves in the sample exceeding the reference value ('Parameter_Exceedance_Number'), and the exceedance percentage ('Parameter_Exceedance_Percentage'), the data subset type, the CMIP generation, and the repsective experiment. Data based on ERA5

Supplementary Data 220: Heatwave Characteristic Exceedange Percentages (Tx90p) - Table lists the shifts in exceedance percentages of the respective heatwave characteristic per data subdivided by decade for CMIP6 multi-model ensemble (MME) SSP5-8.5. The Table shows the region name, dates of the reference event, name of the heatwave characteristic ('Parameter_Name'), the reference value of the respective characteristic ('Parameter'), the respective reference data set ('Reanalysis'), the total number of heatwaves in the respective sample ('total_HW_num'), the number of heatwaves in the sample exceeding the reference value ('Parameter_Exceedance_Number'), and the exceedance percentage ('Parameter_Exceedance_Percentage'), the data subset type, the CMIP generation, and the repsective experiment. Data based on ERA5

Supplementary Data 221: Heatwave Characteristic Exceedange Percentages (Tx90p) - Table lists the shifts in exceedance percentages of the respective heatwave characteristic per data subdivided by decade for CMIP6 multi-model ensemble (MME) SSP5-8.5. The Table shows the region name, dates of the reference event, name of the heatwave characteristic ('Parameter_Name'), the reference value of the respective characteristic ('Parameter'), the respective reference data set ('Reanalysis'), the total number of heatwaves in the respective sample ('total_HW_num'), the number of heatwaves in the sample exceeding the reference value ('Parameter_Exceedance_Number'), and the exceedance percentage ('Parameter_Exceedance_Percentage'), the data subset type, the CMIP generation, and the repsective experiment. Data based on ERA5

Supplementary Data 222: Heatwave Characteristic Exceedange Percentages (Tx90p) - Table lists the shifts in exceedance percentages of the respective heatwave characteristic per data subdivided by decade for CMIP6 multi-model ensemble (MME) SSP5-8.5. The Table shows the region name, dates of the reference event, name of the heatwave characteristic ('Parameter_Name'), the reference value of the respective characteristic ('Parameter'), the respective reference data set ('Reanalysis'), the total number of heatwaves in the respective sample ('total_HW_num'), the number of heatwaves in the sample exceeding the reference value ('Parameter_Exceedance_Number'), and the exceedance percentage ('Parameter_Exceedance_Percentage'), the data subset type, the CMIP generation, and the repsective experiment. Data based on ERA5

Supplementary Data 223: Heatwave Characteristic Exceedange Percentages (Tx90p) - Table lists the shifts in exceedance percentages of the respective heatwave characteristic per data subdivided by decade for CMIP6 multi-model ensemble (MME) SSP5-8.5. The Table shows the region name, dates of the reference event, name of the heatwave characteristic ('Parameter_Name'), the reference value of the respective characteristic ('Parameter'), the respective reference data set ('Reanalysis'), the total number of heatwaves in the respective sample ('total_HW_num'), the number of heatwaves in the sample exceeding the reference value ('Parameter_Exceedance_Number'), and the exceedance percentage ('Parameter_Exceedance_Percentage'), the data subset type, the CMIP generation, and the repsective experiment. Data based on ERA5

Supplementary Data 224: Heatwave Characteristic Exceedange Percentages (Tx90p) - Table lists the shifts in exceedance percentages of the respective heatwave characteristic per data subdivided by decade for CMIP6 multi-model ensemble (MME) SSP5-8.5. The Table shows the region name, dates of the reference event, name of the heatwave characteristic ('Parameter_Name'), the reference value of the respective characteristic ('Parameter'), the respective reference data set ('Reanalysis'), the total number of heatwaves in the respective sample ('total_HW_num'), the number of heatwaves in the sample exceeding the reference value

('Parameter_Exceedance_Number'), and the exceedance percentage ('Parameter_Exceedance_Percentage'), the data subset type, the CMIP generation, and the repsective experiment. Data based on ERA5

Supplementary Data 225: Heatwave Characteristic Exceedange Percentages (Tx90p) - Table lists the shifts in exceedance percentages of the respective heatwave characteristic per data subdivided by decade for CMIP6 multi-model ensemble (MME) SSP5-8.5. The Table shows the region name, dates of the reference event, name of the heatwave characteristic ('Parameter_Name'), the reference value of the respective characteristic ('Parameter'), the respective reference data set ('Reanalysis'), the total number of heatwaves in the respective sample ('total_HW_num'), the number of heatwaves in the sample exceeding the reference value ('Parameter_Exceedance_Number'), and the exceedance percentage ('Parameter_Exceedance_Percentage'), the data subset type, the CMIP generation, and the repsective experiment. Data based on ERA5

Supplementary Data 226: Heatwave Characteristic Exceedange Percentages (Tx90p) - Table lists the shifts in exceedance percentages of the respective heatwave characteristic per data subdivided by decade for CMIP6 multi-model ensemble (MME) SSP5-8.5. The Table shows the region name, dates of the reference event, name of the heatwave characteristic ('Parameter_Name'), the reference value of the respective characteristic ('Parameter'), the respective reference data set ('Reanalysis'), the total number of heatwaves in the respective sample ('total_HW_num'), the number of heatwaves in the sample exceeding the reference value ('Parameter_Exceedance_Number'), and the exceedance percentage ('Parameter_Exceedance_Percentage'), the data subset type, the CMIP generation, and the repsective experiment. Data based on ERA5

Supplementary Data 227: Heatwave Characteristic Exceedange Percentages (Tx90p) - Table lists the shifts in exceedance percentages of the respective heatwave characteristic per data subdivided by decade for CMIP6 multi-model ensemble (MME) SSP5-8.5. The Table shows the region name, dates of the reference event, name of the heatwave characteristic ('Parameter_Name'), the reference value of the respective characteristic ('Parameter'), the respective reference data set ('Reanalysis'), the total number of heatwaves in the respective sample ('total_HW_num'), the number of heatwaves in the sample exceeding the reference value ('Parameter_Exceedance_Number'), and the exceedance percentage ('Parameter_Exceedance_Percentage'), the data subset type, the CMIP generation, and the repsective experiment. Data based on ERA5

Supplementary Data 228: Heatwave Characteristic Exceedange Percentages (Tx90p) - Table lists the shifts in exceedance percentages of the respective heatwave characteristic per data subdivided by decade for CMIP6 multi-model ensemble (MME) SSP5-8.5. The Table shows the region name, dates of the reference event, name of the heatwave characteristic ('Parameter_Name'), the reference value of the respective characteristic ('Parameter'), the respective reference data set ('Reanalysis'), the total number of heatwaves in the respective sample ('total_HW_num'), the number of heatwaves in the sample exceeding the reference value ('Parameter_Exceedance_Number'), and the exceedance percentage ('Parameter_Exceedance_Percentage'), the data subset type, the CMIP generation, and the repsective experiment. Data based on ERA5

Supplementary Data 229: Heatwave Characteristic Exceedange Percentages (Tx90p) - Table lists the shifts in exceedance percentages of the respective heatwave characteristic per data subdivided by Global Warming Levels (GWLs) for CMIP6 multi-model ensemble (MME) SSP1-2.6. The Table shows the region name, dates of the reference event, name of the heatwave characteristic ('Parameter_Name'), the reference value of the

respective characteristic ('Parameter'), the respective reference data set ('Reanalysis'), the total number of heatwaves in the respective sample ('total_HW_num'), the number of heatwaves in the sample exceeding the reference value ('Parameter_Exceedance_Number'), and the exceedance percentage ('Parameter_Exceedance_Percentage'), the data subset type, the CMIP generation, and the repsective experiment. Data based on ERA5

Supplementary Data 230: Heatwave Characteristic Exceedange Percentages (Tx90p) - Table lists the shifts in exceedance percentages of the respective heatwave characteristic per data subdivided by Global Warming Levels (GWLs) for CMIP6 multi-model ensemble (MME) SSP1-2.6. The Table shows the region name, dates of the reference event, name of the heatwave characteristic ('Parameter_Name'), the reference value of the respective characteristic ('Parameter'), the respective reference data set ('Reanalysis'), the total number of heatwaves in the respective sample ('total_HW_num'), the number of heatwaves in the sample exceeding the reference value ('Parameter_Exceedance_Number'), and the exceedance percentage ('Parameter_Exceedance_Percentage'), the data subset type, the CMIP generation, and the repsective experiment. Data based on ERA5

Supplementary Data 231: Heatwave Characteristic Exceedange Percentages (Tx90p) - Table lists the shifts in exceedance percentages of the respective heatwave characteristic per data subdivided by Global Warming Levels (GWLs) for CMIP6 multi-model ensemble (MME) SSP1-2.6. The Table shows the region name, dates of the reference event, name of the heatwave characteristic ('Parameter_Name'), the reference value of the respective characteristic ('Parameter'), the respective reference data set ('Reanalysis'), the total number of heatwaves in the respective sample ('total_HW_num'), the number of heatwaves in the sample exceeding the reference value ('Parameter_Exceedance_Number'), and the exceedance percentage ('Parameter_Exceedance_Percentage'), the data subset type, the CMIP generation, and the repsective experiment. Data based on ERA5

Supplementary Data 232: Heatwave Characteristic Exceedange Percentages (Tx90p) - Table lists the shifts in exceedance percentages of the respective heatwave characteristic per data subdivided by Global Warming Levels (GWLs) for CMIP6 multi-model ensemble (MME) SSP1-2.6. The Table shows the region name, dates of the reference event, name of the heatwave characteristic ('Parameter_Name'), the reference value of the respective characteristic ('Parameter'), the respective reference data set ('Reanalysis'), the total number of heatwaves in the respective sample ('total_HW_num'), the number of heatwaves in the sample exceeding the reference value ('Parameter_Exceedance_Number'), and the exceedance percentage ('Parameter_Exceedance_Percentage'), the data subset type, the CMIP generation, and the repsective experiment. Data based on ERA5

Supplementary Data 233: Heatwave Characteristic Exceedange Percentages (Tx90p) - Table lists the shifts in exceedance percentages of the respective heatwave characteristic per data subdivided by Global Warming Levels (GWLs) for CMIP6 multi-model ensemble (MME) SSP1-2.6. The Table shows the region name, dates of the reference event, name of the heatwave characteristic ('Parameter_Name'), the reference value of the respective characteristic ('Parameter'), the respective reference data set ('Reanalysis'), the total number of heatwaves in the respective sample ('total_HW_num'), the number of heatwaves in the sample exceeding the reference value ('Parameter_Exceedance_Number'), and the exceedance percentage

('Parameter_Exceedance_Percentage'), the data subset type, the CMIP generation, and the repsective experiment. Data based on ERA5

Supplementary Data 234: Heatwave Characteristic Exceedange Percentages (Tx90p) - Table lists the shifts in exceedance percentages of the respective heatwave characteristic per data subdivided by Global Warming Levels (GWLs) for CMIP6 multi-model ensemble (MME) SSP1-2.6. The Table shows the region name, dates of the reference event, name of the heatwave characteristic ('Parameter_Name'), the reference value of the respective characteristic ('Parameter'), the respective reference data set ('Reanalysis'), the total number of heatwaves in the respective sample ('total_HW_num'), the number of heatwaves in the sample exceeding the reference value ('Parameter_Exceedance_Number'), and the exceedance percentage ('Parameter_Exceedance_Percentage'), the data subset type, the CMIP generation, and the repsective experiment. Data based on ERA5

Supplementary Data 235: Heatwave Characteristic Exceedange Percentages (Tx90p) - Table lists the shifts in exceedance percentages of the respective heatwave characteristic per data subdivided by Global Warming Levels (GWLs) for CMIP6 multi-model ensemble (MME) SSP1-2.6. The Table shows the region name, dates of the reference event, name of the heatwave characteristic ('Parameter_Name'), the reference value of the respective characteristic ('Parameter'), the respective reference data set ('Reanalysis'), the total number of heatwaves in the respective sample ('total_HW_num'), the number of heatwaves in the sample exceeding the reference value ('Parameter_Exceedance_Number'), and the exceedance percentage ('Parameter_Exceedance_Percentage'), the data subset type, the CMIP generation, and the repsective experiment. Data based on ERA5

Supplementary Data 236: Heatwave Characteristic Exceedange Percentages (Tx90p) - Table lists the shifts in exceedance percentages of the respective heatwave characteristic per data subdivided by Global Warming Levels (GWLs) for CMIP6 multi-model ensemble (MME) SSP1-2.6. The Table shows the region name, dates of the reference event, name of the heatwave characteristic ('Parameter_Name'), the reference value of the respective characteristic ('Parameter'), the respective reference data set ('Reanalysis'), the total number of heatwaves in the respective sample ('total_HW_num'), the number of heatwaves in the sample exceeding the reference value ('Parameter_Exceedance_Number'), and the exceedance percentage ('Parameter_Exceedance_Percentage'), the data subset type, the CMIP generation, and the repsective experiment. Data based on ERA5

Supplementary Data 237: Heatwave Characteristic Exceedange Percentages (Tx90p) - Table lists the shifts in exceedance percentages of the respective heatwave characteristic per data subdivided by Global Warming Levels (GWLs) for CMIP6 multi-model ensemble (MME) SSP1-2.6. The Table shows the region name, dates of the reference event, name of the heatwave characteristic ('Parameter_Name'), the reference value of the respective characteristic ('Parameter'), the respective reference data set ('Reanalysis'), the total number of heatwaves in the respective sample ('total_HW_num'), the number of heatwaves in the sample exceeding the reference value ('Parameter_Exceedance_Number'), and the exceedance percentage ('Parameter_Exceedance_Percentage'), the data subset type, the CMIP generation, and the repsective experiment. Data based on ERA5

Supplementary Data 238: Heatwave Characteristic Exceedange Percentages (Tx90p) - Table lists the shifts in exceedance percentages of the respective heatwave characteristic per data subdivided by Global Warming Levels (GWLs) for CMIP6 multi-model ensemble (MME) SSP1-2.6. The Table shows the region name, dates of the reference event, name of the heatwave characteristic ('Parameter_Name'), the reference value of the respective characteristic ('Parameter'), the respective reference data set ('Reanalysis'), the total number of heatwaves in the respective sample ('total_HW_num'), the number of heatwaves in the sample exceeding the reference value ('Parameter_Exceedance_Number'), and the exceedance percentage ('Parameter_Exceedance_Percentage'), the data subset type, the CMIP generation, and the repsective experiment. Data based on ERA5

Supplementary Data 239: Heatwave Characteristic Exceedange Percentages (Tx90p) - Table lists the shifts in exceedance percentages of the respective heatwave characteristic per data subdivided by Global Warming Levels (GWLs) for CMIP6 multi-model ensemble (MME) SSP1-2.6. The Table shows the region name, dates of the reference event, name of the heatwave characteristic ('Parameter_Name'), the reference value of the respective characteristic ('Parameter'), the respective reference data set ('Reanalysis'), the total number of heatwaves in the respective sample ('total_HW_num'), the number of heatwaves in the sample exceeding the reference value ('Parameter_Exceedance_Number'), and the exceedance percentage ('Parameter_Exceedance_Percentage'), the data subset type, the CMIP generation, and the repsective experiment. Data based on ERA5

Supplementary Data 240: Heatwave Characteristic Exceedange Percentages (Tx90p) - Table lists the shifts in exceedance percentages of the respective heatwave characteristic per data subdivided by Global Warming Levels (GWLs) for CMIP6 multi-model ensemble (MME) SSP1-2.6. The Table shows the region name, dates of the reference event, name of the heatwave characteristic ('Parameter_Name'), the reference value of the respective characteristic ('Parameter'), the respective reference data set ('Reanalysis'), the total number of heatwaves in the respective sample ('total_HW_num'), the number of heatwaves in the sample exceeding the reference value ('Parameter_Exceedance_Number'), and the exceedance percentage ('Parameter_Exceedance_Percentage'), the data subset type, the CMIP generation, and the repsective experiment. Data based on ERA5

Supplementary Data 241: Heatwave Characteristic Exceedange Percentages (Tx90p) - Table lists the shifts in exceedance percentages of the respective heatwave characteristic per data subdivided by Global Warming Levels (GWLs) for CMIP6 multi-model ensemble (MME) SSP2-4.5. The Table shows the region name, dates of the reference event, name of the heatwave characteristic ('Parameter_Name'), the reference value of the respective characteristic ('Parameter'), the respective reference data set ('Reanalysis'), the total number of heatwaves in the respective sample ('total_HW_num'), the number of heatwaves in the sample exceeding the reference value ('Parameter_Exceedance_Number'), and the exceedance percentage ('Parameter_Exceedance_Percentage'), the data subset type, the CMIP generation, and the repsective experiment. Data based on ERA5

Supplementary Data 242: Heatwave Characteristic Exceedange Percentages (Tx90p) - Table lists the shifts in exceedance percentages of the respective heatwave characteristic per data subdivided by Global Warming Levels (GWLs) for CMIP6 multi-model ensemble (MME) SSP2-4.5. The Table shows the region name, dates of the reference event, name of the heatwave characteristic ('Parameter_Name'), the reference value of the

respective characteristic ('Parameter'), the respective reference data set ('Reanalysis'), the total number of heatwaves in the respective sample ('total_HW_num'), the number of heatwaves in the sample exceeding the reference value ('Parameter_Exceedance_Number'), and the exceedance percentage ('Parameter_Exceedance_Percentage'), the data subset type, the CMIP generation, and the repsective experiment. Data based on ERA5

Supplementary Data 243: Heatwave Characteristic Exceedange Percentages (Tx90p) - Table lists the shifts in exceedance percentages of the respective heatwave characteristic per data subdivided by Global Warming Levels (GWLs) for CMIP6 multi-model ensemble (MME) SSP2-4.5. The Table shows the region name, dates of the reference event, name of the heatwave characteristic ('Parameter_Name'), the reference value of the respective characteristic ('Parameter'), the respective reference data set ('Reanalysis'), the total number of heatwaves in the respective sample ('total_HW_num'), the number of heatwaves in the sample exceeding the reference value ('Parameter_Exceedance_Number'), and the exceedance percentage ('Parameter_Exceedance_Percentage'), the data subset type, the CMIP generation, and the repsective experiment. Data based on ERA5

Supplementary Data 244: Heatwave Characteristic Exceedange Percentages (Tx90p) - Table lists the shifts in exceedance percentages of the respective heatwave characteristic per data subdivided by Global Warming Levels (GWLs) for CMIP6 multi-model ensemble (MME) SSP2-4.5. The Table shows the region name, dates of the reference event, name of the heatwave characteristic ('Parameter_Name'), the reference value of the respective characteristic ('Parameter'), the respective reference data set ('Reanalysis'), the total number of heatwaves in the respective sample ('total_HW_num'), the number of heatwaves in the sample exceeding the reference value ('Parameter_Exceedance_Number'), and the exceedance percentage ('Parameter_Exceedance_Percentage'), the data subset type, the CMIP generation, and the repsective experiment. Data based on ERA5

Supplementary Data 245: Heatwave Characteristic Exceedange Percentages (Tx90p) - Table lists the shifts in exceedance percentages of the respective heatwave characteristic per data subdivided by Global Warming Levels (GWLs) for CMIP6 multi-model ensemble (MME) SSP2-4.5. The Table shows the region name, dates of the reference event, name of the heatwave characteristic ('Parameter_Name'), the reference value of the respective characteristic ('Parameter'), the respective reference data set ('Reanalysis'), the total number of heatwaves in the respective sample ('total_HW_num'), the number of heatwaves in the sample exceeding the reference value ('Parameter_Exceedance_Number'), and the exceedance percentage ('Parameter_Exceedance_Percentage'), the data subset type, the CMIP generation, and the repsective experiment. Data based on ERA5

Supplementary Data 246: Heatwave Characteristic Exceedange Percentages (Tx90p) - Table lists the shifts in exceedance percentages of the respective heatwave characteristic per data subdivided by Global Warming Levels (GWLs) for CMIP6 multi-model ensemble (MME) SSP2-4.5. The Table shows the region name, dates of the reference event, name of the heatwave characteristic ('Parameter_Name'), the reference value of the respective characteristic ('Parameter'), the respective reference data set ('Reanalysis'), the total number of heatwaves in the respective sample ('total_HW_num'), the number of heatwaves in the sample exceeding the reference value ('Parameter_Exceedance_Number'), and the exceedance percentage

('Parameter_Exceedance_Percentage'), the data subset type, the CMIP generation, and the repsective experiment. Data based on ERA5

Supplementary Data 247: Heatwave Characteristic Exceedange Percentages (Tx90p) - Table lists the shifts in exceedance percentages of the respective heatwave characteristic per data subdivided by Global Warming Levels (GWLs) for CMIP6 multi-model ensemble (MME) SSP2-4.5. The Table shows the region name, dates of the reference event, name of the heatwave characteristic ('Parameter_Name'), the reference value of the respective characteristic ('Parameter'), the respective reference data set ('Reanalysis'), the total number of heatwaves in the respective sample ('total_HW_num'), the number of heatwaves in the sample exceeding the reference value ('Parameter_Exceedance_Number'), and the exceedance percentage ('Parameter_Exceedance_Percentage'), the data subset type, the CMIP generation, and the repsective experiment. Data based on ERA5

Supplementary Data 248: Heatwave Characteristic Exceedange Percentages (Tx90p) - Table lists the shifts in exceedance percentages of the respective heatwave characteristic per data subdivided by Global Warming Levels (GWLs) for CMIP6 multi-model ensemble (MME) SSP2-4.5. The Table shows the region name, dates of the reference event, name of the heatwave characteristic ('Parameter_Name'), the reference value of the respective characteristic ('Parameter'), the respective reference data set ('Reanalysis'), the total number of heatwaves in the respective sample ('total_HW_num'), the number of heatwaves in the sample exceeding the reference value ('Parameter_Exceedance_Number'), and the exceedance percentage ('Parameter_Exceedance_Percentage'), the data subset type, the CMIP generation, and the repsective experiment. Data based on ERA5

Supplementary Data 249: Heatwave Characteristic Exceedange Percentages (Tx90p) - Table lists the shifts in exceedance percentages of the respective heatwave characteristic per data subdivided by Global Warming Levels (GWLs) for CMIP6 multi-model ensemble (MME) SSP2-4.5. The Table shows the region name, dates of the reference event, name of the heatwave characteristic ('Parameter_Name'), the reference value of the respective characteristic ('Parameter'), the respective reference data set ('Reanalysis'), the total number of heatwaves in the respective sample ('total_HW_num'), the number of heatwaves in the sample exceeding the reference value ('Parameter_Exceedance_Number'), and the exceedance percentage ('Parameter_Exceedance_Percentage'), the data subset type, the CMIP generation, and the repsective experiment. Data based on ERA5

Supplementary Data 250: Heatwave Characteristic Exceedange Percentages (Tx90p) - Table lists the shifts in exceedance percentages of the respective heatwave characteristic per data subdivided by Global Warming Levels (GWLs) for CMIP6 multi-model ensemble (MME) SSP2-4.5. The Table shows the region name, dates of the reference event, name of the heatwave characteristic ('Parameter_Name'), the reference value of the respective characteristic ('Parameter'), the respective reference data set ('Reanalysis'), the total number of heatwaves in the respective sample ('total_HW_num'), the number of heatwaves in the sample exceeding the reference value ('Parameter_Exceedance_Number'), and the exceedance percentage ('Parameter_Exceedance_Percentage'), the data subset type, the CMIP generation, and the repsective experiment. Data based on ERA5

Supplementary Data 251: Heatwave Characteristic Exceedange Percentages (Tx90p) - Table lists the shifts in exceedance percentages of the respective heatwave characteristic per data subdivided by Global Warming Levels (GWLs) for CMIP6 multi-model ensemble (MME) SSP2-4.5. The Table shows the region name, dates of the reference event, name of the heatwave characteristic ('Parameter_Name'), the reference value of the respective characteristic ('Parameter'), the respective reference data set ('Reanalysis'), the total number of heatwaves in the respective sample ('total_HW_num'), the number of heatwaves in the sample exceeding the reference value ('Parameter_Exceedance_Number'), and the exceedance percentage ('Parameter_Exceedance_Percentage'), the data subset type, the CMIP generation, and the repsective experiment. Data based on ERA5

Supplementary Data 252: Heatwave Characteristic Exceedange Percentages (Tx90p) - Table lists the shifts in exceedance percentages of the respective heatwave characteristic per data subdivided by Global Warming Levels (GWLs) for CMIP6 multi-model ensemble (MME) SSP2-4.5. The Table shows the region name, dates of the reference event, name of the heatwave characteristic ('Parameter_Name'), the reference value of the respective characteristic ('Parameter'), the respective reference data set ('Reanalysis'), the total number of heatwaves in the respective sample ('total_HW_num'), the number of heatwaves in the sample exceeding the reference value ('Parameter_Exceedance_Number'), and the exceedance percentage ('Parameter_Exceedance_Percentage'), the data subset type, the CMIP generation, and the repsective experiment. Data based on ERA5

Supplementary Data 253: Heatwave Characteristic Exceedange Percentages (Tx90p) - Table lists the shifts in exceedance percentages of the respective heatwave characteristic per data subdivided by Global Warming Levels (GWLs) for CMIP6 multi-model ensemble (MME) SSP3-7.0. The Table shows the region name, dates of the reference event, name of the heatwave characteristic ('Parameter_Name'), the reference value of the respective characteristic ('Parameter'), the respective reference data set ('Reanalysis'), the total number of heatwaves in the respective sample ('total_HW_num'), the number of heatwaves in the sample exceeding the reference value ('Parameter_Exceedance_Number'), and the exceedance percentage ('Parameter_Exceedance_Percentage'), the data subset type, the CMIP generation, and the repsective experiment. Data based on ERA5

Supplementary Data 254: Heatwave Characteristic Exceedange Percentages (Tx90p) - Table lists the shifts in exceedance percentages of the respective heatwave characteristic per data subdivided by Global Warming Levels (GWLs) for CMIP6 multi-model ensemble (MME) SSP3-7.0. The Table shows the region name, dates of the reference event, name of the heatwave characteristic ('Parameter_Name'), the reference value of the respective characteristic ('Parameter'), the respective reference data set ('Reanalysis'), the total number of heatwaves in the respective sample ('total_HW_num'), the number of heatwaves in the sample exceeding the reference value ('Parameter_Exceedance_Number'), and the exceedance percentage ('Parameter_Exceedance_Percentage'), the data subset type, the CMIP generation, and the repsective experiment. Data based on ERA5

Supplementary Data 255: Heatwave Characteristic Exceedange Percentages (Tx90p) - Table lists the shifts in exceedance percentages of the respective heatwave characteristic per data subdivided by Global Warming Levels (GWLs) for CMIP6 multi-model ensemble (MME) SSP3-7.0. The Table shows the region name, dates of the reference event, name of the heatwave characteristic ('Parameter_Name'), the reference value of the

respective characteristic ('Parameter'), the respective reference data set ('Reanalysis'), the total number of heatwaves in the respective sample ('total_HW_num'), the number of heatwaves in the sample exceeding the reference value ('Parameter_Exceedance_Number'), and the exceedance percentage ('Parameter_Exceedance_Percentage'), the data subset type, the CMIP generation, and the repsective experiment. Data based on ERA5

Supplementary Data 256: Heatwave Characteristic Exceedange Percentages (Tx90p) - Table lists the shifts in exceedance percentages of the respective heatwave characteristic per data subdivided by Global Warming Levels (GWLs) for CMIP6 multi-model ensemble (MME) SSP3-7.0. The Table shows the region name, dates of the reference event, name of the heatwave characteristic ('Parameter_Name'), the reference value of the respective characteristic ('Parameter'), the respective reference data set ('Reanalysis'), the total number of heatwaves in the respective sample ('total_HW_num'), the number of heatwaves in the sample exceeding the reference value ('Parameter_Exceedance_Number'), and the exceedance percentage ('Parameter_Exceedance_Percentage'), the data subset type, the CMIP generation, and the repsective experiment. Data based on ERA5

Supplementary Data 257: Heatwave Characteristic Exceedange Percentages (Tx90p) - Table lists the shifts in exceedance percentages of the respective heatwave characteristic per data subdivided by Global Warming Levels (GWLs) for CMIP6 multi-model ensemble (MME) SSP3-7.0. The Table shows the region name, dates of the reference event, name of the heatwave characteristic ('Parameter_Name'), the reference value of the respective characteristic ('Parameter'), the respective reference data set ('Reanalysis'), the total number of heatwaves in the respective sample ('total_HW_num'), the number of heatwaves in the sample exceeding the reference value ('Parameter_Exceedance_Number'), and the exceedance percentage ('Parameter_Exceedance_Percentage'), the data subset type, the CMIP generation, and the repsective experiment. Data based on ERA5

Supplementary Data 258: Heatwave Characteristic Exceedange Percentages (Tx90p) - Table lists the shifts in exceedance percentages of the respective heatwave characteristic per data subdivided by Global Warming Levels (GWLs) for CMIP6 multi-model ensemble (MME) SSP3-7.0. The Table shows the region name, dates of the reference event, name of the heatwave characteristic ('Parameter_Name'), the reference value of the respective characteristic ('Parameter'), the respective reference data set ('Reanalysis'), the total number of heatwaves in the respective sample ('total_HW_num'), the number of heatwaves in the sample exceeding the reference value ('Parameter_Exceedance_Number'), and the exceedance percentage ('Parameter_Exceedance_Percentage'), the data subset type, the CMIP generation, and the repsective experiment. Data based on ERA5

Supplementary Data 259: Heatwave Characteristic Exceedange Percentages (Tx90p) - Table lists the shifts in exceedance percentages of the respective heatwave characteristic per data subdivided by Global Warming Levels (GWLs) for CMIP6 multi-model ensemble (MME) SSP3-7.0. The Table shows the region name, dates of the reference event, name of the heatwave characteristic ('Parameter_Name'), the reference value of the respective characteristic ('Parameter'), the respective reference data set ('Reanalysis'), the total number of heatwaves in the respective sample ('total_HW_num'), the number of heatwaves in the sample exceeding the reference value ('Parameter_Exceedance_Number'), and the exceedance percentage

('Parameter_Exceedance_Percentage'), the data subset type, the CMIP generation, and the repsective experiment. Data based on ERA5

Supplementary Data 260: Heatwave Characteristic Exceedange Percentages (Tx90p) - Table lists the shifts in exceedance percentages of the respective heatwave characteristic per data subdivided by Global Warming Levels (GWLs) for CMIP6 multi-model ensemble (MME) SSP3-7.0. The Table shows the region name, dates of the reference event, name of the heatwave characteristic ('Parameter_Name'), the reference value of the respective characteristic ('Parameter'), the respective reference data set ('Reanalysis'), the total number of heatwaves in the respective sample ('total_HW_num'), the number of heatwaves in the sample exceeding the reference value ('Parameter_Exceedance_Number'), and the exceedance percentage ('Parameter_Exceedance_Percentage'), the data subset type, the CMIP generation, and the repsective experiment. Data based on ERA5

Supplementary Data 261: Heatwave Characteristic Exceedange Percentages (Tx90p) - Table lists the shifts in exceedance percentages of the respective heatwave characteristic per data subdivided by Global Warming Levels (GWLs) for CMIP6 multi-model ensemble (MME) SSP3-7.0. The Table shows the region name, dates of the reference event, name of the heatwave characteristic ('Parameter_Name'), the reference value of the respective characteristic ('Parameter'), the respective reference data set ('Reanalysis'), the total number of heatwaves in the respective sample ('total_HW_num'), the number of heatwaves in the sample exceeding the reference value ('Parameter_Exceedance_Number'), and the exceedance percentage ('Parameter_Exceedance_Percentage'), the data subset type, the CMIP generation, and the repsective experiment. Data based on ERA5

Supplementary Data 262: Heatwave Characteristic Exceedange Percentages (Tx90p) - Table lists the shifts in exceedance percentages of the respective heatwave characteristic per data subdivided by Global Warming Levels (GWLs) for CMIP6 multi-model ensemble (MME) SSP3-7.0. The Table shows the region name, dates of the reference event, name of the heatwave characteristic ('Parameter_Name'), the reference value of the respective characteristic ('Parameter'), the respective reference data set ('Reanalysis'), the total number of heatwaves in the respective sample ('total_HW_num'), the number of heatwaves in the sample exceeding the reference value ('Parameter_Exceedance_Number'), and the exceedance percentage ('Parameter_Exceedance_Percentage'), the data subset type, the CMIP generation, and the repsective experiment. Data based on ERA5

Supplementary Data 263: Heatwave Characteristic Exceedange Percentages (Tx90p) - Table lists the shifts in exceedance percentages of the respective heatwave characteristic per data subdivided by Global Warming Levels (GWLs) for CMIP6 multi-model ensemble (MME) SSP3-7.0. The Table shows the region name, dates of the reference event, name of the heatwave characteristic ('Parameter_Name'), the reference value of the respective characteristic ('Parameter'), the respective reference data set ('Reanalysis'), the total number of heatwaves in the respective sample ('total_HW_num'), the number of heatwaves in the sample exceeding the reference value ('Parameter_Exceedance_Number'), and the exceedance percentage ('Parameter_Exceedance_Percentage'), the data subset type, the CMIP generation, and the repsective experiment. Data based on ERA5

Supplementary Data 264: Heatwave Characteristic Exceedange Percentages (Tx90p) - Table lists the shifts in exceedance percentages of the respective heatwave characteristic per data subdivided by Global Warming Levels (GWLs) for CMIP6 multi-model ensemble (MME) SSP3-7.0. The Table shows the region name, dates of the reference event, name of the heatwave characteristic ('Parameter_Name'), the reference value of the respective characteristic ('Parameter'), the respective reference data set ('Reanalysis'), the total number of heatwaves in the respective sample ('total_HW_num'), the number of heatwaves in the sample exceeding the reference value ('Parameter_Exceedance_Number'), and the exceedance percentage ('Parameter_Exceedance_Percentage'), the data subset type, the CMIP generation, and the repsective experiment. Data based on ERA5

Supplementary Data 265: Heatwave Characteristic Exceedange Percentages (Tx90p) - Table lists the shifts in exceedance percentages of the respective heatwave characteristic per data subdivided by Global Warming Levels (GWLs) for CMIP6 multi-model ensemble (MME) SSP5-8.5. The Table shows the region name, dates of the reference event, name of the heatwave characteristic ('Parameter_Name'), the reference value of the respective characteristic ('Parameter'), the respective reference data set ('Reanalysis'), the total number of heatwaves in the respective sample ('total_HW_num'), the number of heatwaves in the sample exceeding the reference value ('Parameter_Exceedance_Number'), and the exceedance percentage ('Parameter_Exceedance_Percentage'), the data subset type, the CMIP generation, and the repsective experiment. Data based on ERA5

Supplementary Data 266: Heatwave Characteristic Exceedange Percentages (Tx90p) - Table lists the shifts in exceedance percentages of the respective heatwave characteristic per data subdivided by Global Warming Levels (GWLs) for CMIP6 multi-model ensemble (MME) SSP5-8.5. The Table shows the region name, dates of the reference event, name of the heatwave characteristic ('Parameter_Name'), the reference value of the respective characteristic ('Parameter'), the respective reference data set ('Reanalysis'), the total number of heatwaves in the respective sample ('total_HW_num'), the number of heatwaves in the sample exceeding the reference value ('Parameter_Exceedance_Number'), and the exceedance percentage ('Parameter_Exceedance_Percentage'), the data subset type, the CMIP generation, and the repsective experiment. Data based on ERA5

Supplementary Data 267: Heatwave Characteristic Exceedange Percentages (Tx90p) - Table lists the shifts in exceedance percentages of the respective heatwave characteristic per data subdivided by Global Warming Levels (GWLs) for CMIP6 multi-model ensemble (MME) SSP5-8.5. The Table shows the region name, dates of the reference event, name of the heatwave characteristic ('Parameter_Name'), the reference value of the respective characteristic ('Parameter'), the respective reference data set ('Reanalysis'), the total number of heatwaves in the respective sample ('total_HW_num'), the number of heatwaves in the sample exceeding the reference value ('Parameter_Exceedance_Number'), and the exceedance percentage ('Parameter_Exceedance_Percentage'), the data subset type, the CMIP generation, and the repsective experiment. Data based on ERA5

Supplementary Data 268: Heatwave Characteristic Exceedange Percentages (Tx90p) - Table lists the shifts in exceedance percentages of the respective heatwave characteristic per data subdivided by Global Warming Levels (GWLs) for CMIP6 multi-model ensemble (MME) SSP5-8.5. The Table shows the region name, dates of the reference event, name of the heatwave characteristic ('Parameter_Name'), the reference value of the

respective characteristic ('Parameter'), the respective reference data set ('Reanalysis'), the total number of heatwaves in the respective sample ('total_HW_num'), the number of heatwaves in the sample exceeding the reference value ('Parameter_Exceedance_Number'), and the exceedance percentage ('Parameter_Exceedance_Percentage'), the data subset type, the CMIP generation, and the repsective experiment. Data based on ERA5

Supplementary Data 269: Heatwave Characteristic Exceedange Percentages (Tx90p) - Table lists the shifts in exceedance percentages of the respective heatwave characteristic per data subdivided by Global Warming Levels (GWLs) for CMIP6 multi-model ensemble (MME) SSP5-8.5. The Table shows the region name, dates of the reference event, name of the heatwave characteristic ('Parameter_Name'), the reference value of the respective characteristic ('Parameter'), the respective reference data set ('Reanalysis'), the total number of heatwaves in the respective sample ('total_HW_num'), the number of heatwaves in the sample exceeding the reference value ('Parameter_Exceedance_Number'), and the exceedance percentage ('Parameter_Exceedance_Percentage'), the data subset type, the CMIP generation, and the repsective experiment. Data based on ERA5

Supplementary Data 270: Heatwave Characteristic Exceedange Percentages (Tx90p) - Table lists the shifts in exceedance percentages of the respective heatwave characteristic per data subdivided by Global Warming Levels (GWLs) for CMIP6 multi-model ensemble (MME) SSP5-8.5. The Table shows the region name, dates of the reference event, name of the heatwave characteristic ('Parameter_Name'), the reference value of the respective characteristic ('Parameter'), the respective reference data set ('Reanalysis'), the total number of heatwaves in the respective sample ('total_HW_num'), the number of heatwaves in the sample exceeding the reference value ('Parameter_Exceedance_Number'), and the exceedance percentage ('Parameter_Exceedance_Percentage'), the data subset type, the CMIP generation, and the repsective experiment. Data based on ERA5

Supplementary Data 271: Heatwave Characteristic Exceedange Percentages (Tx90p) - Table lists the shifts in exceedance percentages of the respective heatwave characteristic per data subdivided by Global Warming Levels (GWLs) for CMIP6 multi-model ensemble (MME) SSP5-8.5. The Table shows the region name, dates of the reference event, name of the heatwave characteristic ('Parameter_Name'), the reference value of the respective characteristic ('Parameter'), the respective reference data set ('Reanalysis'), the total number of heatwaves in the respective sample ('total_HW_num'), the number of heatwaves in the sample exceeding the reference value ('Parameter_Exceedance_Number'), and the exceedance percentage ('Parameter_Exceedance_Percentage'), the data subset type, the CMIP generation, and the repsective experiment. Data based on ERA5

Supplementary Data 272: Heatwave Characteristic Exceedange Percentages (Tx90p) - Table lists the shifts in exceedance percentages of the respective heatwave characteristic per data subdivided by Global Warming Levels (GWLs) for CMIP6 multi-model ensemble (MME) SSP5-8.5. The Table shows the region name, dates of the reference event, name of the heatwave characteristic ('Parameter_Name'), the reference value of the respective characteristic ('Parameter'), the respective reference data set ('Reanalysis'), the total number of heatwaves in the respective sample ('total_HW_num'), the number of heatwaves in the sample exceeding the reference value ('Parameter_Exceedance_Number'), and the exceedance percentage

('Parameter_Exceedance_Percentage'), the data subset type, the CMIP generation, and the repsective experiment. Data based on ERA5

Supplementary Data 273: Heatwave Characteristic Exceedange Percentages (Tx90p) - Table lists the shifts in exceedance percentages of the respective heatwave characteristic per data subdivided by Global Warming Levels (GWLs) for CMIP6 multi-model ensemble (MME) SSP5-8.5. The Table shows the region name, dates of the reference event, name of the heatwave characteristic ('Parameter_Name'), the reference value of the respective characteristic ('Parameter'), the respective reference data set ('Reanalysis'), the total number of heatwaves in the respective sample ('total_HW_num'), the number of heatwaves in the sample exceeding the reference value ('Parameter_Exceedance_Number'), and the exceedance percentage ('Parameter_Exceedance_Percentage'), the data subset type, the CMIP generation, and the repsective experiment. Data based on ERA5

Supplementary Data 274: Heatwave Characteristic Exceedange Percentages (Tx90p) - Table lists the shifts in exceedance percentages of the respective heatwave characteristic per data subdivided by Global Warming Levels (GWLs) for CMIP6 multi-model ensemble (MME) SSP5-8.5. The Table shows the region name, dates of the reference event, name of the heatwave characteristic ('Parameter_Name'), the reference value of the respective characteristic ('Parameter'), the respective reference data set ('Reanalysis'), the total number of heatwaves in the respective sample ('total_HW_num'), the number of heatwaves in the sample exceeding the reference value ('Parameter_Exceedance_Number'), and the exceedance percentage ('Parameter_Exceedance_Percentage'), the data subset type, the CMIP generation, and the repsective experiment. Data based on ERA5

Supplementary Data 275: Heatwave Characteristic Exceedange Percentages (Tx90p) - Table lists the shifts in exceedance percentages of the respective heatwave characteristic per data subdivided by Global Warming Levels (GWLs) for CMIP6 multi-model ensemble (MME) SSP5-8.5. The Table shows the region name, dates of the reference event, name of the heatwave characteristic ('Parameter_Name'), the reference value of the respective characteristic ('Parameter'), the respective reference data set ('Reanalysis'), the total number of heatwaves in the respective sample ('total_HW_num'), the number of heatwaves in the sample exceeding the reference value ('Parameter_Exceedance_Number'), and the exceedance percentage ('Parameter_Exceedance_Percentage'), the data subset type, the CMIP generation, and the repsective experiment. Data based on ERA5

Supplementary Data 276: Heatwave Characteristic Exceedange Percentages (Tx90p) - Table lists the shifts in exceedance percentages of the respective heatwave characteristic per data subdivided by Global Warming Levels (GWLs) for CMIP6 multi-model ensemble (MME) SSP5-8.5. The Table shows the region name, dates of the reference event, name of the heatwave characteristic ('Parameter_Name'), the reference value of the respective characteristic ('Parameter'), the respective reference data set ('Reanalysis'), the total number of heatwaves in the respective sample ('total_HW_num'), the number of heatwaves in the sample exceeding the reference value ('Parameter_Exceedance_Number'), and the exceedance percentage ('Parameter_Exceedance_Percentage'), the data subset type, the CMIP generation, and the repsective experiment. Data based on ERA5

Supplementary Data 277: Heatwave Characteristic Exceedange Percentages (Tx95.0p) - Table lists the exceedance percentages of the respective heatwave characteristic for CMIP6 multi-model ensemble (MME) SSP1-2.6 for data subdivided by decade. The Table shows the region name, dates of the reference event, name of the heatwave characteristic ('Parameter_Name'), the reference value of the respective characteristic ('Parameter'), the respective reference data set ('Reanalysis'), the total number of heatwaves in the respective sample ('total_HW_num'), the number of heatwaves in the sample exceeding the reference value ('Parameter_Exceedance_Number'), and the exceedance percentage ('Parameter_Exceedance_Percentage'), the data subset type, the CMIP generation, and the repsective experiment. Data based on ERA5

Supplementary Data 278: Heatwave Characteristic Exceedange Percentages (Tx97.5p) - Table lists the exceedance percentages of the respective heatwave characteristic for CMIP6 multi-model ensemble (MME) SSP1-2.6 for data subdivided by decade. The Table shows the region name, dates of the reference event, name of the heatwave characteristic ('Parameter_Name'), the reference value of the respective characteristic ('Parameter'), the respective reference data set ('Reanalysis'), the total number of heatwaves in the respective sample ('total_HW_num'), the number of heatwaves in the sample exceeding the reference value ('Parameter_Exceedance_Number'), and the exceedance percentage ('Parameter_Exceedance_Percentage'), the data subset type, the CMIP generation, and the repsective experiment. Data based on ERA5

Supplementary Data 279: Heatwave Characteristic Exceedange Percentages (Tx99.0p) - Table lists the exceedance percentages of the respective heatwave characteristic for CMIP6 multi-model ensemble (MME) SSP1-2.6 for data subdivided by decade. The Table shows the region name, dates of the reference event, name of the heatwave characteristic ('Parameter_Name'), the reference value of the respective characteristic ('Parameter'), the respective reference data set ('Reanalysis'), the total number of heatwaves in the respective sample ('total_HW_num'), the number of heatwaves in the sample exceeding the reference value ('Parameter_Exceedance_Number'), and the exceedance percentage ('Parameter_Exceedance_Percentage'), the data subset type, the CMIP generation, and the repsective experiment. Data based on ERA5

Supplementary Data 280: Heatwave Characteristic Exceedange Percentages (Tx95.0p) - Table lists the exceedance percentages of the respective heatwave characteristic for CMIP6 multi-model ensemble (MME) SSP1-2.6 for data subdivided by decade. The Table shows the region name, dates of the reference event, name of the heatwave characteristic ('Parameter_Name'), the reference value of the respective characteristic ('Parameter'), the respective reference data set ('Reanalysis'), the total number of heatwaves in the respective sample ('total_HW_num'), the number of heatwaves in the sample exceeding the reference value ('Parameter_Exceedance_Number'), and the exceedance percentage ('Parameter_Exceedance_Percentage'), the data subset type, the CMIP generation, and the repsective experiment. Data based on ERA5

Supplementary Data 281: Heatwave Characteristic Exceedange Percentages (Tx97.5p) - Table lists the exceedance percentages of the respective heatwave characteristic for CMIP6 multi-model ensemble (MME) SSP1-2.6 for data subdivided by decade. The Table shows the region name, dates of the reference event, name of the heatwave characteristic ('Parameter_Name'), the reference value of the respective characteristic ('Parameter'), the respective reference data set ('Reanalysis'), the total number of heatwaves in the respective sample ('total_HW_num'), the number of heatwaves in the sample exceeding the reference value ('Parameter_Exceedance_Number'), and the exceedance percentage ('Parameter_Exceedance_Percentage'), the data subset type, the CMIP generation, and the repsective experiment. Data based on ERA5

Supplementary Data 282: Heatwave Characteristic Exceedange Percentages (Tx99.0p) - Table lists the exceedance percentages of the respective heatwave characteristic for CMIP6 multi-model ensemble (MME) SSP1-2.6 for data subdivided by decade. The Table shows the region name, dates of the reference event, name of the heatwave characteristic ('Parameter_Name'), the reference value of the respective characteristic ('Parameter'), the respective reference data set ('Reanalysis'), the total number of heatwaves in the respective sample ('total_HW_num'), the number of heatwaves in the sample exceeding the reference value ('Parameter_Exceedance_Number'), and the exceedance percentage ('Parameter_Exceedance_Percentage'), the data subset type, the CMIP generation, and the repsective experiment. Data based on ERA5

Supplementary Data 283: Heatwave Characteristic Exceedange Percentages (Tx95.0p) - Table lists the exceedance percentages of the respective heatwave characteristic for CMIP6 multi-model ensemble (MME) SSP1-2.6 for data subdivided by decade. The Table shows the region name, dates of the reference event, name of the heatwave characteristic ('Parameter_Name'), the reference value of the respective characteristic ('Parameter'), the respective reference data set ('Reanalysis'), the total number of heatwaves in the respective sample ('total_HW_num'), the number of heatwaves in the sample exceeding the reference value ('Parameter_Exceedance_Number'), and the exceedance percentage ('Parameter_Exceedance_Percentage'), the data subset type, the CMIP generation, and the repsective experiment. Data based on ERA5

Supplementary Data 284: Heatwave Characteristic Exceedange Percentages (Tx97.5p) - Table lists the exceedance percentages of the respective heatwave characteristic for CMIP6 multi-model ensemble (MME) SSP1-2.6 for data subdivided by decade. The Table shows the region name, dates of the reference event, name of the heatwave characteristic ('Parameter_Name'), the reference value of the respective characteristic ('Parameter'), the respective reference data set ('Reanalysis'), the total number of heatwaves in the respective sample ('total_HW_num'), the number of heatwaves in the sample exceeding the reference value ('Parameter_Exceedance_Number'), and the exceedance percentage ('Parameter_Exceedance_Percentage'), the data subset type, the CMIP generation, and the repsective experiment. Data based on ERA5

Supplementary Data 285: Heatwave Characteristic Exceedange Percentages (Tx99.0p) - Table lists the exceedance percentages of the respective heatwave characteristic for CMIP6 multi-model ensemble (MME) SSP1-2.6 for data subdivided by decade. The Table shows the region name, dates of the reference event, name of the heatwave characteristic ('Parameter_Name'), the reference value of the respective characteristic ('Parameter'), the respective reference data set ('Reanalysis'), the total number of heatwaves in the respective sample ('total_HW_num'), the number of heatwaves in the sample exceeding the reference value ('Parameter_Exceedance_Number'), and the exceedance percentage ('Parameter_Exceedance_Percentage'), the data subset type, the CMIP generation, and the repsective experiment. Data based on ERA5

Supplementary Data 286: Heatwave Characteristic Exceedange Percentages (Tx95.0p) - Table lists the exceedance percentages of the respective heatwave characteristic for CMIP6 multi-model ensemble (MME) SSP1-2.6 for data subdivided by decade. The Table shows the region name, dates of the reference event, name of the heatwave characteristic ('Parameter_Name'), the reference value of the respective characteristic ('Parameter'), the respective reference data set ('Reanalysis'), the total number of heatwaves in the respective sample ('total_HW_num'), the number of heatwaves in the sample exceeding the reference value

('Parameter_Exceedance_Number'), and the exceedance percentage ('Parameter_Exceedance_Percentage'), the data subset type, the CMIP generation, and the repsective experiment. Data based on ERA5

Supplementary Data 287: Heatwave Characteristic Exceedange Percentages (Tx97.5p) - Table lists the exceedance percentages of the respective heatwave characteristic for CMIP6 multi-model ensemble (MME) SSP1-2.6 for data subdivided by decade. The Table shows the region name, dates of the reference event, name of the heatwave characteristic ('Parameter_Name'), the reference value of the respective characteristic ('Parameter'), the respective reference data set ('Reanalysis'), the total number of heatwaves in the respective sample ('total_HW_num'), the number of heatwaves in the sample exceeding the reference value ('Parameter_Exceedance_Number'), and the exceedance percentage ('Parameter_Exceedance_Percentage'), the data subset type, the CMIP generation, and the repsective experiment. Data based on ERA5

Supplementary Data 288: Heatwave Characteristic Exceedange Percentages (Tx99.0p) - Table lists the exceedance percentages of the respective heatwave characteristic for CMIP6 multi-model ensemble (MME) SSP1-2.6 for data subdivided by decade. The Table shows the region name, dates of the reference event, name of the heatwave characteristic ('Parameter_Name'), the reference value of the respective characteristic ('Parameter'), the respective reference data set ('Reanalysis'), the total number of heatwaves in the respective sample ('total_HW_num'), the number of heatwaves in the sample exceeding the reference value ('Parameter_Exceedance_Number'), and the exceedance percentage ('Parameter_Exceedance_Percentage'), the data subset type, the CMIP generation, and the repsective experiment. Data based on ERA5

Supplementary Data 289: Heatwave Characteristic Exceedange Percentages (Tx95.0p) - Table lists the exceedance percentages of the respective heatwave characteristic for CMIP6 multi-model ensemble (MME) SSP1-2.6 for data subdivided by decade. The Table shows the region name, dates of the reference event, name of the heatwave characteristic ('Parameter_Name'), the reference value of the respective characteristic ('Parameter'), the respective reference data set ('Reanalysis'), the total number of heatwaves in the respective sample ('total_HW_num'), the number of heatwaves in the sample exceeding the reference value ('Parameter_Exceedance_Number'), and the exceedance percentage ('Parameter_Exceedance_Percentage'), the data subset type, the CMIP generation, and the repsective experiment. Data based on ERA5

Supplementary Data 290: Heatwave Characteristic Exceedange Percentages (Tx97.5p) - Table lists the exceedance percentages of the respective heatwave characteristic for CMIP6 multi-model ensemble (MME) SSP1-2.6 for data subdivided by decade. The Table shows the region name, dates of the reference event, name of the heatwave characteristic ('Parameter_Name'), the reference value of the respective characteristic ('Parameter'), the respective reference data set ('Reanalysis'), the total number of heatwaves in the respective sample ('total_HW_num'), the number of heatwaves in the sample exceeding the reference value ('Parameter_Exceedance_Number'), and the exceedance percentage ('Parameter_Exceedance_Percentage'), the data subset type, the CMIP generation, and the repsective experiment. Data based on ERA5

Supplementary Data 291: Heatwave Characteristic Exceedange Percentages (Tx99.0p) - Table lists the exceedance percentages of the respective heatwave characteristic for CMIP6 multi-model ensemble (MME) SSP1-2.6 for data subdivided by decade. The Table shows the region name, dates of the reference event, name of the heatwave characteristic ('Parameter_Name'), the reference value of the respective characteristic

('Parameter'), the respective reference data set ('Reanalysis'), the total number of heatwaves in the respective sample ('total_HW_num'), the number of heatwaves in the sample exceeding the reference value ('Parameter_Exceedance_Number'), and the exceedance percentage ('Parameter_Exceedance_Percentage'), the data subset type, the CMIP generation, and the repsective experiment. Data based on ERA5

Supplementary Data 292: Heatwave Characteristic Exceedange Percentages (Tx95.0p) - Table lists the exceedance percentages of the respective heatwave characteristic for CMIP6 multi-model ensemble (MME) SSP1-2.6 for data subdivided by decade. The Table shows the region name, dates of the reference event, name of the heatwave characteristic ('Parameter_Name'), the reference value of the respective characteristic ('Parameter'), the respective reference data set ('Reanalysis'), the total number of heatwaves in the respective sample ('total_HW_num'), the number of heatwaves in the sample exceeding the reference value ('Parameter_Exceedance_Number'), and the exceedance percentage ('Parameter_Exceedance_Percentage'), the data subset type, the CMIP generation, and the repsective experiment. Data based on ERA5

Supplementary Data 293: Heatwave Characteristic Exceedange Percentages (Tx97.5p) - Table lists the exceedance percentages of the respective heatwave characteristic for CMIP6 multi-model ensemble (MME) SSP1-2.6 for data subdivided by decade. The Table shows the region name, dates of the reference event, name of the heatwave characteristic ('Parameter_Name'), the reference value of the respective characteristic ('Parameter'), the respective reference data set ('Reanalysis'), the total number of heatwaves in the respective sample ('total_HW_num'), the number of heatwaves in the sample exceeding the reference value ('Parameter_Exceedance_Number'), and the exceedance percentage ('Parameter_Exceedance_Percentage'), the data subset type, the CMIP generation, and the repsective experiment. Data based on ERA5

Supplementary Data 294: Heatwave Characteristic Exceedange Percentages (Tx99.0p) - Table lists the exceedance percentages of the respective heatwave characteristic for CMIP6 multi-model ensemble (MME) SSP1-2.6 for data subdivided by decade. The Table shows the region name, dates of the reference event, name of the heatwave characteristic ('Parameter_Name'), the reference value of the respective characteristic ('Parameter'), the respective reference data set ('Reanalysis'), the total number of heatwaves in the respective sample ('total_HW_num'), the number of heatwaves in the sample exceeding the reference value ('Parameter_Exceedance_Number'), and the exceedance percentage ('Parameter_Exceedance_Percentage'), the data subset type, the CMIP generation, and the repsective experiment. Data based on ERA5

Supplementary Data 295: Heatwave Characteristic Exceedange Percentages (Tx95.0p) - Table lists the exceedance percentages of the respective heatwave characteristic for CMIP6 multi-model ensemble (MME) SSP2-4.5 for data subdivided by decade. The Table shows the region name, dates of the reference event, name of the heatwave characteristic ('Parameter_Name'), the reference value of the respective characteristic ('Parameter'), the respective reference data set ('Reanalysis'), the total number of heatwaves in the respective sample ('total_HW_num'), the number of heatwaves in the sample exceeding the reference value ('Parameter_Exceedance_Number'), and the exceedance percentage ('Parameter_Exceedance_Percentage'), the data subset type, the CMIP generation, and the repsective experiment. Data based on ERA5

Supplementary Data 296: Heatwave Characteristic Exceedange Percentages (Tx97.5p) - Table lists the exceedance percentages of the respective heatwave characteristic for CMIP6 multi-model ensemble (MME)

SSP2-4.5 for data subdivided by decade. The Table shows the region name, dates of the reference event, name of the heatwave characteristic ('Parameter_Name'), the reference value of the respective characteristic ('Parameter'), the respective reference data set ('Reanalysis'), the total number of heatwaves in the respective sample ('total_HW_num'), the number of heatwaves in the sample exceeding the reference value ('Parameter_Exceedance_Number'), and the exceedance percentage ('Parameter_Exceedance_Percentage'), the data subset type, the CMIP generation, and the repsective experiment. Data based on ERA5

Supplementary Data 297: Heatwave Characteristic Exceedange Percentages (Tx99.0p) - Table lists the exceedance percentages of the respective heatwave characteristic for CMIP6 multi-model ensemble (MME) SSP2-4.5 for data subdivided by decade. The Table shows the region name, dates of the reference event, name of the heatwave characteristic ('Parameter_Name'), the reference value of the respective characteristic ('Parameter'), the respective reference data set ('Reanalysis'), the total number of heatwaves in the respective sample ('total_HW_num'), the number of heatwaves in the sample exceeding the reference value ('Parameter_Exceedance_Number'), and the exceedance percentage ('Parameter_Exceedance_Percentage'), the data subset type, the CMIP generation, and the repsective experiment. Data based on ERA5

Supplementary Data 298: Heatwave Characteristic Exceedange Percentages (Tx95.0p) - Table lists the exceedance percentages of the respective heatwave characteristic for CMIP6 multi-model ensemble (MME) SSP2-4.5 for data subdivided by decade. The Table shows the region name, dates of the reference event, name of the heatwave characteristic ('Parameter_Name'), the reference value of the respective characteristic ('Parameter'), the respective reference data set ('Reanalysis'), the total number of heatwaves in the respective sample ('total_HW_num'), the number of heatwaves in the sample exceeding the reference value ('Parameter_Exceedance_Number'), and the exceedance percentage ('Parameter_Exceedance_Percentage'), the data subset type, the CMIP generation, and the repsective experiment. Data based on ERA5

Supplementary Data 299: Heatwave Characteristic Exceedange Percentages (Tx97.5p) - Table lists the exceedance percentages of the respective heatwave characteristic for CMIP6 multi-model ensemble (MME) SSP2-4.5 for data subdivided by decade. The Table shows the region name, dates of the reference event, name of the heatwave characteristic ('Parameter_Name'), the reference value of the respective characteristic ('Parameter'), the respective reference data set ('Reanalysis'), the total number of heatwaves in the respective sample ('total_HW_num'), the number of heatwaves in the sample exceeding the reference value ('Parameter_Exceedance_Number'), and the exceedance percentage ('Parameter_Exceedance_Percentage'), the data subset type, the CMIP generation, and the repsective experiment. Data based on ERA5

Supplementary Data 300: Heatwave Characteristic Exceedange Percentages (Tx99.0p) - Table lists the exceedance percentages of the respective heatwave characteristic for CMIP6 multi-model ensemble (MME) SSP2-4.5 for data subdivided by decade. The Table shows the region name, dates of the reference event, name of the heatwave characteristic ('Parameter_Name'), the reference value of the respective characteristic ('Parameter'), the respective reference data set ('Reanalysis'), the total number of heatwaves in the respective sample ('total_HW_num'), the number of heatwaves in the sample exceeding the reference value ('Parameter_Exceedance_Number'), and the exceedance percentage ('Parameter_Exceedance_Percentage'), the data subset type, the CMIP generation, and the repsective experiment. Data based on ERA5

Supplementary Data 301: Heatwave Characteristic Exceedange Percentages (Tx95.0p) - Table lists the exceedance percentages of the respective heatwave characteristic for CMIP6 multi-model ensemble (MME) SSP2-4.5 for data subdivided by decade. The Table shows the region name, dates of the reference event, name of the heatwave characteristic ('Parameter_Name'), the reference value of the respective characteristic ('Parameter'), the respective reference data set ('Reanalysis'), the total number of heatwaves in the respective sample ('total_HW_num'), the number of heatwaves in the sample exceeding the reference value ('Parameter_Exceedance_Number'), and the exceedance percentage ('Parameter_Exceedance_Percentage'), the data subset type, the CMIP generation, and the repsective experiment. Data based on ERA5

Supplementary Data 302: Heatwave Characteristic Exceedange Percentages (Tx97.5p) - Table lists the exceedance percentages of the respective heatwave characteristic for CMIP6 multi-model ensemble (MME) SSP2-4.5 for data subdivided by decade. The Table shows the region name, dates of the reference event, name of the heatwave characteristic ('Parameter_Name'), the reference value of the respective characteristic ('Parameter'), the respective reference data set ('Reanalysis'), the total number of heatwaves in the respective sample ('total_HW_num'), the number of heatwaves in the sample exceeding the reference value ('Parameter_Exceedance_Number'), and the exceedance percentage ('Parameter_Exceedance_Percentage'), the data subset type, the CMIP generation, and the repsective experiment. Data based on ERA5

Supplementary Data 303: Heatwave Characteristic Exceedange Percentages (Tx99.0p) - Table lists the exceedance percentages of the respective heatwave characteristic for CMIP6 multi-model ensemble (MME) SSP2-4.5 for data subdivided by decade. The Table shows the region name, dates of the reference event, name of the heatwave characteristic ('Parameter_Name'), the reference value of the respective characteristic ('Parameter'), the respective reference data set ('Reanalysis'), the total number of heatwaves in the respective sample ('total_HW_num'), the number of heatwaves in the sample exceeding the reference value ('Parameter_Exceedance_Number'), and the exceedance percentage ('Parameter_Exceedance_Percentage'), the data subset type, the CMIP generation, and the repsective experiment. Data based on ERA5

Supplementary Data 304: Heatwave Characteristic Exceedange Percentages (Tx95.0p) - Table lists the exceedance percentages of the respective heatwave characteristic for CMIP6 multi-model ensemble (MME) SSP2-4.5 for data subdivided by decade. The Table shows the region name, dates of the reference event, name of the heatwave characteristic ('Parameter_Name'), the reference value of the respective characteristic ('Parameter'), the respective reference data set ('Reanalysis'), the total number of heatwaves in the respective sample ('total_HW_num'), the number of heatwaves in the sample exceeding the reference value ('Parameter_Exceedance_Number'), and the exceedance percentage ('Parameter_Exceedance_Percentage'), the data subset type, the CMIP generation, and the repsective experiment. Data based on ERA5

Supplementary Data 305: Heatwave Characteristic Exceedange Percentages (Tx97.5p) - Table lists the exceedance percentages of the respective heatwave characteristic for CMIP6 multi-model ensemble (MME) SSP2-4.5 for data subdivided by decade. The Table shows the region name, dates of the reference event, name of the heatwave characteristic ('Parameter_Name'), the reference value of the respective characteristic ('Parameter'), the respective reference data set ('Reanalysis'), the total number of heatwaves in the respective sample ('total_HW_num'), the number of heatwaves in the sample exceeding the reference value ('Parameter_Exceedance_Number'), and the exceedance percentage ('Parameter_Exceedance_Percentage'), the data subset type, the CMIP generation, and the repsective experiment. Data based on ERA5

Supplementary Data 306: Heatwave Characteristic Exceedange Percentages (Tx99.0p) - Table lists the exceedance percentages of the respective heatwave characteristic for CMIP6 multi-model ensemble (MME) SSP2-4.5 for data subdivided by decade. The Table shows the region name, dates of the reference event, name of the heatwave characteristic ('Parameter_Name'), the reference value of the respective characteristic ('Parameter'), the respective reference data set ('Reanalysis'), the total number of heatwaves in the respective sample ('total_HW_num'), the number of heatwaves in the sample exceeding the reference value ('Parameter_Exceedance_Number'), and the exceedance percentage ('Parameter_Exceedance_Percentage'), the data subset type, the CMIP generation, and the repsective experiment. Data based on ERA5

Supplementary Data 307: Heatwave Characteristic Exceedange Percentages (Tx95.0p) - Table lists the exceedance percentages of the respective heatwave characteristic for CMIP6 multi-model ensemble (MME) SSP2-4.5 for data subdivided by decade. The Table shows the region name, dates of the reference event, name of the heatwave characteristic ('Parameter_Name'), the reference value of the respective characteristic ('Parameter'), the respective reference data set ('Reanalysis'), the total number of heatwaves in the respective sample ('total_HW_num'), the number of heatwaves in the sample exceeding the reference value ('Parameter_Exceedance_Number'), and the exceedance percentage ('Parameter_Exceedance_Percentage'), the data subset type, the CMIP generation, and the repsective experiment. Data based on ERA5

Supplementary Data 308: Heatwave Characteristic Exceedange Percentages (Tx97.5p) - Table lists the exceedance percentages of the respective heatwave characteristic for CMIP6 multi-model ensemble (MME) SSP2-4.5 for data subdivided by decade. The Table shows the region name, dates of the reference event, name of the heatwave characteristic ('Parameter_Name'), the reference value of the respective characteristic ('Parameter'), the respective reference data set ('Reanalysis'), the total number of heatwaves in the respective sample ('total_HW_num'), the number of heatwaves in the sample exceeding the reference value ('Parameter_Exceedance_Number'), and the exceedance percentage ('Parameter_Exceedance_Percentage'), the data subset type, the CMIP generation, and the repsective experiment. Data based on ERA5

Supplementary Data 309: Heatwave Characteristic Exceedange Percentages (Tx99.0p) - Table lists the exceedance percentages of the respective heatwave characteristic for CMIP6 multi-model ensemble (MME) SSP2-4.5 for data subdivided by decade. The Table shows the region name, dates of the reference event, name of the heatwave characteristic ('Parameter_Name'), the reference value of the respective characteristic ('Parameter'), the respective reference data set ('Reanalysis'), the total number of heatwaves in the respective sample ('total_HW_num'), the number of heatwaves in the sample exceeding the reference value ('Parameter_Exceedance_Number'), and the exceedance percentage ('Parameter_Exceedance_Percentage'), the data subset type, the CMIP generation, and the repsective experiment. Data based on ERA5

Supplementary Data 310: Heatwave Characteristic Exceedange Percentages (Tx95.0p) - Table lists the exceedance percentages of the respective heatwave characteristic for CMIP6 multi-model ensemble (MME) SSP2-4.5 for data subdivided by decade. The Table shows the region name, dates of the reference event, name of the heatwave characteristic ('Parameter_Name'), the reference value of the respective characteristic ('Parameter'), the respective reference data set ('Reanalysis'), the total number of heatwaves in the respective sample ('total_HW_num'), the number of heatwaves in the sample exceeding the reference value

('Parameter_Exceedance_Number'), and the exceedance percentage ('Parameter_Exceedance_Percentage'), the data subset type, the CMIP generation, and the repsective experiment. Data based on ERA5

Supplementary Data 311: Heatwave Characteristic Exceedange Percentages (Tx97.5p) - Table lists the exceedance percentages of the respective heatwave characteristic for CMIP6 multi-model ensemble (MME) SSP2-4.5 for data subdivided by decade. The Table shows the region name, dates of the reference event, name of the heatwave characteristic ('Parameter_Name'), the reference value of the respective characteristic ('Parameter'), the respective reference data set ('Reanalysis'), the total number of heatwaves in the respective sample ('total_HW_num'), the number of heatwaves in the sample exceeding the reference value ('Parameter_Exceedance_Number'), and the exceedance percentage ('Parameter_Exceedance_Percentage'), the data subset type, the CMIP generation, and the repsective experiment. Data based on ERA5

Supplementary Data 312: Heatwave Characteristic Exceedange Percentages (Tx99.0p) - Table lists the exceedance percentages of the respective heatwave characteristic for CMIP6 multi-model ensemble (MME) SSP2-4.5 for data subdivided by decade. The Table shows the region name, dates of the reference event, name of the heatwave characteristic ('Parameter_Name'), the reference value of the respective characteristic ('Parameter'), the respective reference data set ('Reanalysis'), the total number of heatwaves in the respective sample ('total_HW_num'), the number of heatwaves in the sample exceeding the reference value ('Parameter_Exceedance_Number'), and the exceedance percentage ('Parameter_Exceedance_Percentage'), the data subset type, the CMIP generation, and the repsective experiment. Data based on ERA5

Supplementary Data 313: Heatwave Characteristic Exceedange Percentages (Tx95.0p) - Table lists the exceedance percentages of the respective heatwave characteristic for CMIP6 multi-model ensemble (MME) SSP3-7.0 for data subdivided by decade. The Table shows the region name, dates of the reference event, name of the heatwave characteristic ('Parameter_Name'), the reference value of the respective characteristic ('Parameter'), the respective reference data set ('Reanalysis'), the total number of heatwaves in the respective sample ('total_HW_num'), the number of heatwaves in the sample exceeding the reference value ('Parameter_Exceedance_Number'), and the exceedance percentage ('Parameter_Exceedance_Percentage'), the data subset type, the CMIP generation, and the repsective experiment. Data based on ERA5

Supplementary Data 314: Heatwave Characteristic Exceedange Percentages (Tx97.5p) - Table lists the exceedance percentages of the respective heatwave characteristic for CMIP6 multi-model ensemble (MME) SSP3-7.0 for data subdivided by decade. The Table shows the region name, dates of the reference event, name of the heatwave characteristic ('Parameter_Name'), the reference value of the respective characteristic ('Parameter'), the respective reference data set ('Reanalysis'), the total number of heatwaves in the respective sample ('total_HW_num'), the number of heatwaves in the sample exceeding the reference value ('Parameter_Exceedance_Number'), and the exceedance percentage ('Parameter_Exceedance_Percentage'), the data subset type, the CMIP generation, and the repsective experiment. Data based on ERA5

Supplementary Data 315: Heatwave Characteristic Exceedange Percentages (Tx99.0p) - Table lists the exceedance percentages of the respective heatwave characteristic for CMIP6 multi-model ensemble (MME) SSP3-7.0 for data subdivided by decade. The Table shows the region name, dates of the reference event, name of the heatwave characteristic ('Parameter_Name'), the reference value of the respective characteristic

('Parameter'), the respective reference data set ('Reanalysis'), the total number of heatwaves in the respective sample ('total_HW_num'), the number of heatwaves in the sample exceeding the reference value ('Parameter_Exceedance_Number'), and the exceedance percentage ('Parameter_Exceedance_Percentage'), the data subset type, the CMIP generation, and the repsective experiment. Data based on ERA5

Supplementary Data 316: Heatwave Characteristic Exceedange Percentages (Tx95.0p) - Table lists the exceedance percentages of the respective heatwave characteristic for CMIP6 multi-model ensemble (MME) SSP3-7.0 for data subdivided by decade. The Table shows the region name, dates of the reference event, name of the heatwave characteristic ('Parameter_Name'), the reference value of the respective characteristic ('Parameter'), the respective reference data set ('Reanalysis'), the total number of heatwaves in the respective sample ('total_HW_num'), the number of heatwaves in the sample exceeding the reference value ('Parameter_Exceedance_Number'), and the exceedance percentage ('Parameter_Exceedance_Percentage'), the data subset type, the CMIP generation, and the repsective experiment. Data based on ERA5

Supplementary Data 317: Heatwave Characteristic Exceedange Percentages (Tx97.5p) - Table lists the exceedance percentages of the respective heatwave characteristic for CMIP6 multi-model ensemble (MME) SSP3-7.0 for data subdivided by decade. The Table shows the region name, dates of the reference event, name of the heatwave characteristic ('Parameter_Name'), the reference value of the respective characteristic ('Parameter'), the respective reference data set ('Reanalysis'), the total number of heatwaves in the respective sample ('total_HW_num'), the number of heatwaves in the sample exceeding the reference value ('Parameter_Exceedance_Number'), and the exceedance percentage ('Parameter_Exceedance_Percentage'), the data subset type, the CMIP generation, and the repsective experiment. Data based on ERA5

Supplementary Data 318: Heatwave Characteristic Exceedange Percentages (Tx99.0p) - Table lists the exceedance percentages of the respective heatwave characteristic for CMIP6 multi-model ensemble (MME) SSP3-7.0 for data subdivided by decade. The Table shows the region name, dates of the reference event, name of the heatwave characteristic ('Parameter_Name'), the reference value of the respective characteristic ('Parameter'), the respective reference data set ('Reanalysis'), the total number of heatwaves in the respective sample ('total_HW_num'), the number of heatwaves in the sample exceeding the reference value ('Parameter_Exceedance_Number'), and the exceedance percentage ('Parameter_Exceedance_Percentage'), the data subset type, the CMIP generation, and the repsective experiment. Data based on ERA5

Supplementary Data 319: Heatwave Characteristic Exceedange Percentages (Tx95.0p) - Table lists the exceedance percentages of the respective heatwave characteristic for CMIP6 multi-model ensemble (MME) SSP3-7.0 for data subdivided by decade. The Table shows the region name, dates of the reference event, name of the heatwave characteristic ('Parameter_Name'), the reference value of the respective characteristic ('Parameter'), the respective reference data set ('Reanalysis'), the total number of heatwaves in the respective sample ('total_HW_num'), the number of heatwaves in the sample exceeding the reference value ('Parameter_Exceedance_Number'), and the exceedance percentage ('Parameter_Exceedance_Percentage'), the data subset type, the CMIP generation, and the repsective experiment. Data based on ERA5

Supplementary Data 320: Heatwave Characteristic Exceedange Percentages (Tx97.5p) - Table lists the exceedance percentages of the respective heatwave characteristic for CMIP6 multi-model ensemble (MME)

SSP3-7.0 for data subdivided by decade. The Table shows the region name, dates of the reference event, name of the heatwave characteristic ('Parameter_Name'), the reference value of the respective characteristic ('Parameter'), the respective reference data set ('Reanalysis'), the total number of heatwaves in the respective sample ('total_HW_num'), the number of heatwaves in the sample exceeding the reference value ('Parameter_Exceedance_Number'), and the exceedance percentage ('Parameter_Exceedance_Percentage'), the data subset type, the CMIP generation, and the repsective experiment. Data based on ERA5

Supplementary Data 321: Heatwave Characteristic Exceedange Percentages (Tx99.0p) - Table lists the exceedance percentages of the respective heatwave characteristic for CMIP6 multi-model ensemble (MME) SSP3-7.0 for data subdivided by decade. The Table shows the region name, dates of the reference event, name of the heatwave characteristic ('Parameter_Name'), the reference value of the respective characteristic ('Parameter'), the respective reference data set ('Reanalysis'), the total number of heatwaves in the respective sample ('total_HW_num'), the number of heatwaves in the sample exceeding the reference value ('Parameter_Exceedance_Number'), and the exceedance percentage ('Parameter_Exceedance_Percentage'), the data subset type, the CMIP generation, and the repsective experiment. Data based on ERA5

Supplementary Data 322: Heatwave Characteristic Exceedange Percentages (Tx95.0p) - Table lists the exceedance percentages of the respective heatwave characteristic for CMIP6 multi-model ensemble (MME) SSP3-7.0 for data subdivided by decade. The Table shows the region name, dates of the reference event, name of the heatwave characteristic ('Parameter_Name'), the reference value of the respective characteristic ('Parameter'), the respective reference data set ('Reanalysis'), the total number of heatwaves in the respective sample ('total_HW_num'), the number of heatwaves in the sample exceeding the reference value ('Parameter_Exceedance_Number'), and the exceedance percentage ('Parameter_Exceedance_Percentage'), the data subset type, the CMIP generation, and the repsective experiment. Data based on ERA5

Supplementary Data 323: Heatwave Characteristic Exceedange Percentages (Tx97.5p) - Table lists the exceedance percentages of the respective heatwave characteristic for CMIP6 multi-model ensemble (MME) SSP3-7.0 for data subdivided by decade. The Table shows the region name, dates of the reference event, name of the heatwave characteristic ('Parameter_Name'), the reference value of the respective characteristic ('Parameter'), the respective reference data set ('Reanalysis'), the total number of heatwaves in the respective sample ('total_HW_num'), the number of heatwaves in the sample exceeding the reference value ('Parameter_Exceedance_Number'), and the exceedance percentage ('Parameter_Exceedance_Percentage'), the data subset type, the CMIP generation, and the repsective experiment. Data based on ERA5

Supplementary Data 324: Heatwave Characteristic Exceedange Percentages (Tx99.0p) - Table lists the exceedance percentages of the respective heatwave characteristic for CMIP6 multi-model ensemble (MME) SSP3-7.0 for data subdivided by decade. The Table shows the region name, dates of the reference event, name of the heatwave characteristic ('Parameter_Name'), the reference value of the respective characteristic ('Parameter'), the respective reference data set ('Reanalysis'), the total number of heatwaves in the respective sample ('total_HW_num'), the number of heatwaves in the sample exceeding the reference value ('Parameter_Exceedance_Number'), and the exceedance percentage ('Parameter_Exceedance_Percentage'), the data subset type, the CMIP generation, and the repsective experiment. Data based on ERA5

Supplementary Data 325: Heatwave Characteristic Exceedange Percentages (Tx95.0p) - Table lists the exceedance percentages of the respective heatwave characteristic for CMIP6 multi-model ensemble (MME) SSP3-7.0 for data subdivided by decade. The Table shows the region name, dates of the reference event, name of the heatwave characteristic ('Parameter_Name'), the reference value of the respective characteristic ('Parameter'), the respective reference data set ('Reanalysis'), the total number of heatwaves in the respective sample ('total_HW_num'), the number of heatwaves in the sample exceeding the reference value ('Parameter_Exceedance_Number'), and the exceedance percentage ('Parameter_Exceedance_Percentage'), the data subset type, the CMIP generation, and the repsective experiment. Data based on ERA5

Supplementary Data 326: Heatwave Characteristic Exceedange Percentages (Tx97.5p) - Table lists the exceedance percentages of the respective heatwave characteristic for CMIP6 multi-model ensemble (MME) SSP3-7.0 for data subdivided by decade. The Table shows the region name, dates of the reference event, name of the heatwave characteristic ('Parameter_Name'), the reference value of the respective characteristic ('Parameter'), the respective reference data set ('Reanalysis'), the total number of heatwaves in the respective sample ('total_HW_num'), the number of heatwaves in the sample exceeding the reference value ('Parameter_Exceedance_Number'), and the exceedance percentage ('Parameter_Exceedance_Percentage'), the data subset type, the CMIP generation, and the repsective experiment. Data based on ERA5

Supplementary Data 327: Heatwave Characteristic Exceedange Percentages (Tx99.0p) - Table lists the exceedance percentages of the respective heatwave characteristic for CMIP6 multi-model ensemble (MME) SSP3-7.0 for data subdivided by decade. The Table shows the region name, dates of the reference event, name of the heatwave characteristic ('Parameter_Name'), the reference value of the respective characteristic ('Parameter'), the respective reference data set ('Reanalysis'), the total number of heatwaves in the respective sample ('total_HW_num'), the number of heatwaves in the sample exceeding the reference value ('Parameter_Exceedance_Number'), and the exceedance percentage ('Parameter_Exceedance_Percentage'), the data subset type, the CMIP generation, and the repsective experiment. Data based on ERA5

Supplementary Data 328: Heatwave Characteristic Exceedange Percentages (Tx95.0p) - Table lists the exceedance percentages of the respective heatwave characteristic for CMIP6 multi-model ensemble (MME) SSP3-7.0 for data subdivided by decade. The Table shows the region name, dates of the reference event, name of the heatwave characteristic ('Parameter_Name'), the reference value of the respective characteristic ('Parameter'), the respective reference data set ('Reanalysis'), the total number of heatwaves in the respective sample ('total_HW_num'), the number of heatwaves in the sample exceeding the reference value ('Parameter_Exceedance_Number'), and the exceedance percentage ('Parameter_Exceedance_Percentage'), the data subset type, the CMIP generation, and the repsective experiment. Data based on ERA5

Supplementary Data 329: Heatwave Characteristic Exceedange Percentages (Tx97.5p) - Table lists the exceedance percentages of the respective heatwave characteristic for CMIP6 multi-model ensemble (MME) SSP3-7.0 for data subdivided by decade. The Table shows the region name, dates of the reference event, name of the heatwave characteristic ('Parameter_Name'), the reference value of the respective characteristic ('Parameter'), the respective reference data set ('Reanalysis'), the total number of heatwaves in the respective sample ('total_HW_num'), the number of heatwaves in the sample exceeding the reference value ('Parameter_Exceedance_Number'), and the exceedance percentage ('Parameter_Exceedance_Percentage'), the data subset type, the CMIP generation, and the repsective experiment. Data based on ERA5

Supplementary Data 330: Heatwave Characteristic Exceedange Percentages (Tx99.0p) - Table lists the exceedance percentages of the respective heatwave characteristic for CMIP6 multi-model ensemble (MME) SSP3-7.0 for data subdivided by decade. The Table shows the region name, dates of the reference event, name of the heatwave characteristic ('Parameter_Name'), the reference value of the respective characteristic ('Parameter'), the respective reference data set ('Reanalysis'), the total number of heatwaves in the respective sample ('total_HW_num'), the number of heatwaves in the sample exceeding the reference value ('Parameter_Exceedance_Number'), and the exceedance percentage ('Parameter_Exceedance_Percentage'), the data subset type, the CMIP generation, and the repsective experiment. Data based on ERA5

Supplementary Data 331: Heatwave Characteristic Exceedange Percentages (Tx95.0p) - Table lists the exceedance percentages of the respective heatwave characteristic for CMIP6 multi-model ensemble (MME) SSP5-8.5 for data subdivided by decade. The Table shows the region name, dates of the reference event, name of the heatwave characteristic ('Parameter_Name'), the reference value of the respective characteristic ('Parameter'), the respective reference data set ('Reanalysis'), the total number of heatwaves in the respective sample ('total_HW_num'), the number of heatwaves in the sample exceeding the reference value ('Parameter_Exceedance_Number'), and the exceedance percentage ('Parameter_Exceedance_Percentage'), the data subset type, the CMIP generation, and the repsective experiment. Data based on ERA5

Supplementary Data 332: Heatwave Characteristic Exceedange Percentages (Tx97.5p) - Table lists the exceedance percentages of the respective heatwave characteristic for CMIP6 multi-model ensemble (MME) SSP5-8.5 for data subdivided by decade. The Table shows the region name, dates of the reference event, name of the heatwave characteristic ('Parameter_Name'), the reference value of the respective characteristic ('Parameter'), the respective reference data set ('Reanalysis'), the total number of heatwaves in the respective sample ('total_HW_num'), the number of heatwaves in the sample exceeding the reference value ('Parameter_Exceedance_Number'), and the exceedance percentage ('Parameter_Exceedance_Percentage'), the data subset type, the CMIP generation, and the repsective experiment. Data based on ERA5

Supplementary Data 333: Heatwave Characteristic Exceedange Percentages (Tx99.0p) - Table lists the exceedance percentages of the respective heatwave characteristic for CMIP6 multi-model ensemble (MME) SSP5-8.5 for data subdivided by decade. The Table shows the region name, dates of the reference event, name of the heatwave characteristic ('Parameter_Name'), the reference value of the respective characteristic ('Parameter'), the respective reference data set ('Reanalysis'), the total number of heatwaves in the respective sample ('total_HW_num'), the number of heatwaves in the sample exceeding the reference value ('Parameter_Exceedance_Number'), and the exceedance percentage ('Parameter_Exceedance_Percentage'), the data subset type, the CMIP generation, and the repsective experiment. Data based on ERA5

Supplementary Data 334: Heatwave Characteristic Exceedange Percentages (Tx95.0p) - Table lists the exceedance percentages of the respective heatwave characteristic for CMIP6 multi-model ensemble (MME) SSP5-8.5 for data subdivided by decade. The Table shows the region name, dates of the reference event, name of the heatwave characteristic ('Parameter_Name'), the reference value of the respective characteristic ('Parameter'), the respective reference data set ('Reanalysis'), the total number of heatwaves in the respective sample ('total_HW_num'), the number of heatwaves in the sample exceeding the reference value

('Parameter_Exceedance_Number'), and the exceedance percentage ('Parameter_Exceedance_Percentage'), the data subset type, the CMIP generation, and the repsective experiment. Data based on ERA5

Supplementary Data 335: Heatwave Characteristic Exceedange Percentages (Tx97.5p) - Table lists the exceedance percentages of the respective heatwave characteristic for CMIP6 multi-model ensemble (MME) SSP5-8.5 for data subdivided by decade. The Table shows the region name, dates of the reference event, name of the heatwave characteristic ('Parameter_Name'), the reference value of the respective characteristic ('Parameter'), the respective reference data set ('Reanalysis'), the total number of heatwaves in the respective sample ('total_HW_num'), the number of heatwaves in the sample exceeding the reference value ('Parameter_Exceedance_Number'), and the exceedance percentage ('Parameter_Exceedance_Percentage'), the data subset type, the CMIP generation, and the repsective experiment. Data based on ERA5

Supplementary Data 336: Heatwave Characteristic Exceedange Percentages (Tx99.0p) - Table lists the exceedance percentages of the respective heatwave characteristic for CMIP6 multi-model ensemble (MME) SSP5-8.5 for data subdivided by decade. The Table shows the region name, dates of the reference event, name of the heatwave characteristic ('Parameter_Name'), the reference value of the respective characteristic ('Parameter'), the respective reference data set ('Reanalysis'), the total number of heatwaves in the respective sample ('total_HW_num'), the number of heatwaves in the sample exceeding the reference value ('Parameter_Exceedance_Number'), and the exceedance percentage ('Parameter_Exceedance_Percentage'), the data subset type, the CMIP generation, and the repsective experiment. Data based on ERA5

Supplementary Data 337: Heatwave Characteristic Exceedange Percentages (Tx95.0p) - Table lists the exceedance percentages of the respective heatwave characteristic for CMIP6 multi-model ensemble (MME) SSP5-8.5 for data subdivided by decade. The Table shows the region name, dates of the reference event, name of the heatwave characteristic ('Parameter_Name'), the reference value of the respective characteristic ('Parameter'), the respective reference data set ('Reanalysis'), the total number of heatwaves in the respective sample ('total_HW_num'), the number of heatwaves in the sample exceeding the reference value ('Parameter_Exceedance_Number'), and the exceedance percentage ('Parameter_Exceedance_Percentage'), the data subset type, the CMIP generation, and the repsective experiment. Data based on ERA5

Supplementary Data 338: Heatwave Characteristic Exceedange Percentages (Tx97.5p) - Table lists the exceedance percentages of the respective heatwave characteristic for CMIP6 multi-model ensemble (MME) SSP5-8.5 for data subdivided by decade. The Table shows the region name, dates of the reference event, name of the heatwave characteristic ('Parameter_Name'), the reference value of the respective characteristic ('Parameter'), the respective reference data set ('Reanalysis'), the total number of heatwaves in the respective sample ('total_HW_num'), the number of heatwaves in the sample exceeding the reference value ('Parameter_Exceedance_Number'), and the exceedance percentage ('Parameter_Exceedance_Percentage'), the data subset type, the CMIP generation, and the repsective experiment. Data based on ERA5

Supplementary Data 339: Heatwave Characteristic Exceedange Percentages (Tx99.0p) - Table lists the exceedance percentages of the respective heatwave characteristic for CMIP6 multi-model ensemble (MME) SSP5-8.5 for data subdivided by decade. The Table shows the region name, dates of the reference event, name of the heatwave characteristic ('Parameter_Name'), the reference value of the respective characteristic

('Parameter'), the respective reference data set ('Reanalysis'), the total number of heatwaves in the respective sample ('total_HW_num'), the number of heatwaves in the sample exceeding the reference value ('Parameter_Exceedance_Number'), and the exceedance percentage ('Parameter_Exceedance_Percentage'), the data subset type, the CMIP generation, and the repsective experiment. Data based on ERA5

Supplementary Data 340: Heatwave Characteristic Exceedange Percentages (Tx95.0p) - Table lists the exceedance percentages of the respective heatwave characteristic for CMIP6 multi-model ensemble (MME) SSP5-8.5 for data subdivided by decade. The Table shows the region name, dates of the reference event, name of the heatwave characteristic ('Parameter_Name'), the reference value of the respective characteristic ('Parameter'), the respective reference data set ('Reanalysis'), the total number of heatwaves in the respective sample ('total_HW_num'), the number of heatwaves in the sample exceeding the reference value ('Parameter_Exceedance_Number'), and the exceedance percentage ('Parameter_Exceedance_Percentage'), the data subset type, the CMIP generation, and the repsective experiment. Data based on ERA5

Supplementary Data 341: Heatwave Characteristic Exceedange Percentages (Tx97.5p) - Table lists the exceedance percentages of the respective heatwave characteristic for CMIP6 multi-model ensemble (MME) SSP5-8.5 for data subdivided by decade. The Table shows the region name, dates of the reference event, name of the heatwave characteristic ('Parameter_Name'), the reference value of the respective characteristic ('Parameter'), the respective reference data set ('Reanalysis'), the total number of heatwaves in the respective sample ('total_HW_num'), the number of heatwaves in the sample exceeding the reference value ('Parameter_Exceedance_Number'), and the exceedance percentage ('Parameter_Exceedance_Percentage'), the data subset type, the CMIP generation, and the repsective experiment. Data based on ERA5

Supplementary Data 342: Heatwave Characteristic Exceedange Percentages (Tx99.0p) - Table lists the exceedance percentages of the respective heatwave characteristic for CMIP6 multi-model ensemble (MME) SSP5-8.5 for data subdivided by decade. The Table shows the region name, dates of the reference event, name of the heatwave characteristic ('Parameter_Name'), the reference value of the respective characteristic ('Parameter'), the respective reference data set ('Reanalysis'), the total number of heatwaves in the respective sample ('total_HW_num'), the number of heatwaves in the sample exceeding the reference value ('Parameter_Exceedance_Number'), and the exceedance percentage ('Parameter_Exceedance_Percentage'), the data subset type, the CMIP generation, and the repsective experiment. Data based on ERA5

Supplementary Data 343: Heatwave Characteristic Exceedange Percentages (Tx95.0p) - Table lists the exceedance percentages of the respective heatwave characteristic for CMIP6 multi-model ensemble (MME) SSP5-8.5 for data subdivided by decade. The Table shows the region name, dates of the reference event, name of the heatwave characteristic ('Parameter_Name'), the reference value of the respective characteristic ('Parameter'), the respective reference data set ('Reanalysis'), the total number of heatwaves in the respective sample ('total_HW_num'), the number of heatwaves in the sample exceeding the reference value ('Parameter_Exceedance_Number'), and the exceedance percentage ('Parameter_Exceedance_Percentage'), the data subset type, the CMIP generation, and the repsective experiment. Data based on ERA5

Supplementary Data 344: Heatwave Characteristic Exceedange Percentages (Tx97.5p) - Table lists the exceedance percentages of the respective heatwave characteristic for CMIP6 multi-model ensemble (MME)

SSP5-8.5 for data subdivided by decade. The Table shows the region name, dates of the reference event, name of the heatwave characteristic ('Parameter_Name'), the reference value of the respective characteristic ('Parameter'), the respective reference data set ('Reanalysis'), the total number of heatwaves in the respective sample ('total_HW_num'), the number of heatwaves in the sample exceeding the reference value ('Parameter_Exceedance_Number'), and the exceedance percentage ('Parameter_Exceedance_Percentage'), the data subset type, the CMIP generation, and the repsective experiment. Data based on ERA5

Supplementary Data 345: Heatwave Characteristic Exceedange Percentages (Tx99.0p) - Table lists the exceedance percentages of the respective heatwave characteristic for CMIP6 multi-model ensemble (MME) SSP5-8.5 for data subdivided by decade. The Table shows the region name, dates of the reference event, name of the heatwave characteristic ('Parameter_Name'), the reference value of the respective characteristic ('Parameter'), the respective reference data set ('Reanalysis'), the total number of heatwaves in the respective sample ('total_HW_num'), the number of heatwaves in the sample exceeding the reference value ('Parameter_Exceedance_Number'), and the exceedance percentage ('Parameter_Exceedance_Percentage'), the data subset type, the CMIP generation, and the repsective experiment. Data based on ERA5

Supplementary Data 346: Heatwave Characteristic Exceedange Percentages (Tx95.0p) - Table lists the exceedance percentages of the respective heatwave characteristic for CMIP6 multi-model ensemble (MME) SSP5-8.5 for data subdivided by decade. The Table shows the region name, dates of the reference event, name of the heatwave characteristic ('Parameter_Name'), the reference value of the respective characteristic ('Parameter'), the respective reference data set ('Reanalysis'), the total number of heatwaves in the respective sample ('total_HW_num'), the number of heatwaves in the sample exceeding the reference value ('Parameter_Exceedance_Number'), and the exceedance percentage ('Parameter_Exceedance_Percentage'), the data subset type, the CMIP generation, and the repsective experiment. Data based on ERA5

Supplementary Data 347: Heatwave Characteristic Exceedange Percentages (Tx97.5p) - Table lists the exceedance percentages of the respective heatwave characteristic for CMIP6 multi-model ensemble (MME) SSP5-8.5 for data subdivided by decade. The Table shows the region name, dates of the reference event, name of the heatwave characteristic ('Parameter_Name'), the reference value of the respective characteristic ('Parameter'), the respective reference data set ('Reanalysis'), the total number of heatwaves in the respective sample ('total_HW_num'), the number of heatwaves in the sample exceeding the reference value ('Parameter_Exceedance_Number'), and the exceedance percentage ('Parameter_Exceedance_Percentage'), the data subset type, the CMIP generation, and the repsective experiment. Data based on ERA5

Supplementary Data 348: Heatwave Characteristic Exceedange Percentages (Tx99.0p) - Table lists the exceedance percentages of the respective heatwave characteristic for CMIP6 multi-model ensemble (MME) SSP5-8.5 for data subdivided by decade. The Table shows the region name, dates of the reference event, name of the heatwave characteristic ('Parameter_Name'), the reference value of the respective characteristic ('Parameter'), the respective reference data set ('Reanalysis'), the total number of heatwaves in the respective sample ('total_HW_num'), the number of heatwaves in the sample exceeding the reference value ('Parameter_Exceedance_Number'), and the exceedance percentage ('Parameter_Exceedance_Percentage'), the data subset type, the CMIP generation, and the repsective experiment. Data based on ERA5

Supplementary Data 349: Heatwave Characteristic Exceedange Percentages (Tx95.0p) - Table lists the exceedance percentages of the respective heatwave characteristic for CMIP6 multi-model ensemble (MME) SSP1-2.6 for data subdivided by Global Warming Levels (GWLs). The Table shows the region name, dates of the reference event, name of the heatwave characteristic ('Parameter_Name'), the reference value of the respective characteristic ('Parameter'), the respective reference data set ('Reanalysis'), the total number of heatwaves in the respective sample ('total_HW_num'), the number of heatwaves in the sample exceeding the reference value ('Parameter_Exceedance_Number'), and the exceedance percentage ('Parameter_Exceedance_Percentage'), the data subset type, the CMIP generation, and the repsective experiment. Data based on ERA5

Supplementary Data 350: Heatwave Characteristic Exceedange Percentages (Tx97.5p) - Table lists the exceedance percentages of the respective heatwave characteristic for CMIP6 multi-model ensemble (MME) SSP1-2.6 for data subdivided by Global Warming Levels (GWLs). The Table shows the region name, dates of the reference event, name of the heatwave characteristic ('Parameter_Name'), the reference value of the respective characteristic ('Parameter'), the respective reference data set ('Reanalysis'), the total number of heatwaves in the respective sample ('total_HW_num'), the number of heatwaves in the sample exceeding the reference value ('Parameter_Exceedance_Number'), and the exceedance percentage ('Parameter_Exceedance_Percentage'), the data subset type, the CMIP generation, and the repsective experiment. Data based on ERA5

Supplementary Data 351: Heatwave Characteristic Exceedange Percentages (Tx99.0p) - Table lists the exceedance percentages of the respective heatwave characteristic for CMIP6 multi-model ensemble (MME) SSP1-2.6 for data subdivided by Global Warming Levels (GWLs). The Table shows the region name, dates of the reference event, name of the heatwave characteristic ('Parameter_Name'), the reference value of the respective characteristic ('Parameter'), the respective reference data set ('Reanalysis'), the total number of heatwaves in the respective sample ('total_HW_num'), the number of heatwaves in the sample exceeding the reference value ('Parameter_Exceedance_Number'), and the exceedance percentage ('Parameter_Exceedance_Percentage'), the data subset type, the CMIP generation, and the repsective experiment. Data based on ERA5

Supplementary Data 352: Heatwave Characteristic Exceedange Percentages (Tx95.0p) - Table lists the exceedance percentages of the respective heatwave characteristic for CMIP6 multi-model ensemble (MME) SSP1-2.6 for data subdivided by Global Warming Levels (GWLs). The Table shows the region name, dates of the reference event, name of the heatwave characteristic ('Parameter_Name'), the reference value of the respective characteristic ('Parameter'), the respective reference data set ('Reanalysis'), the total number of heatwaves in the respective sample ('total_HW_num'), the number of heatwaves in the sample exceeding the reference value ('Parameter_Exceedance_Number'), and the exceedance percentage ('Parameter_Exceedance_Percentage'), the data subset type, the CMIP generation, and the repsective experiment. Data based on ERA5

Supplementary Data 353: Heatwave Characteristic Exceedange Percentages (Tx97.5p) - Table lists the exceedance percentages of the respective heatwave characteristic for CMIP6 multi-model ensemble (MME) SSP1-2.6 for data subdivided by Global Warming Levels (GWLs). The Table shows the region name, dates of the reference event, name of the heatwave characteristic ('Parameter_Name'), the reference value of the

respective characteristic ('Parameter'), the respective reference data set ('Reanalysis'), the total number of heatwaves in the respective sample ('total_HW_num'), the number of heatwaves in the sample exceeding the reference value ('Parameter_Exceedance_Number'), and the exceedance percentage ('Parameter_Exceedance_Percentage'), the data subset type, the CMIP generation, and the repsective experiment. Data based on ERA5

Supplementary Data 354: Heatwave Characteristic Exceedange Percentages (Tx99.0p) - Table lists the exceedance percentages of the respective heatwave characteristic for CMIP6 multi-model ensemble (MME) SSP1-2.6 for data subdivided by Global Warming Levels (GWLs). The Table shows the region name, dates of the reference event, name of the heatwave characteristic ('Parameter_Name'), the reference value of the respective characteristic ('Parameter'), the respective reference data set ('Reanalysis'), the total number of heatwaves in the respective sample ('total_HW_num'), the number of heatwaves in the sample exceeding the reference value ('Parameter_Exceedance_Number'), and the exceedance percentage ('Parameter_Exceedance_Percentage'), the data subset type, the CMIP generation, and the repsective experiment. Data based on ERA5

Supplementary Data 355: Heatwave Characteristic Exceedange Percentages (Tx95.0p) - Table lists the exceedance percentages of the respective heatwave characteristic for CMIP6 multi-model ensemble (MME) SSP1-2.6 for data subdivided by Global Warming Levels (GWLs). The Table shows the region name, dates of the reference event, name of the heatwave characteristic ('Parameter_Name'), the reference value of the respective characteristic ('Parameter'), the respective reference data set ('Reanalysis'), the total number of heatwaves in the respective sample ('total_HW_num'), the number of heatwaves in the sample exceeding the reference value ('Parameter_Exceedance_Number'), and the exceedance percentage ('Parameter_Exceedance_Percentage'), the data subset type, the CMIP generation, and the repsective experiment. Data based on ERA5

Supplementary Data 356: Heatwave Characteristic Exceedange Percentages (Tx97.5p) - Table lists the exceedance percentages of the respective heatwave characteristic for CMIP6 multi-model ensemble (MME) SSP1-2.6 for data subdivided by Global Warming Levels (GWLs). The Table shows the region name, dates of the reference event, name of the heatwave characteristic ('Parameter_Name'), the reference value of the respective characteristic ('Parameter'), the respective reference data set ('Reanalysis'), the total number of heatwaves in the respective sample ('total_HW_num'), the number of heatwaves in the sample exceeding the reference value ('Parameter_Exceedance_Number'), and the exceedance percentage ('Parameter_Exceedance_Percentage'), the data subset type, the CMIP generation, and the repsective experiment. Data based on ERA5

Supplementary Data 357: Heatwave Characteristic Exceedange Percentages (Tx99.0p) - Table lists the exceedance percentages of the respective heatwave characteristic for CMIP6 multi-model ensemble (MME) SSP1-2.6 for data subdivided by Global Warming Levels (GWLs). The Table shows the region name, dates of the reference event, name of the heatwave characteristic ('Parameter_Name'), the reference value of the respective characteristic ('Parameter'), the respective reference data set ('Reanalysis'), the total number of heatwaves in the respective sample ('total_HW_num'), the number of heatwaves in the sample exceeding the reference value ('Parameter_Exceedance_Number'), and the exceedance percentage

('Parameter_Exceedance_Percentage'), the data subset type, the CMIP generation, and the repsective experiment. Data based on ERA5

Supplementary Data 358: Heatwave Characteristic Exceedange Percentages (Tx95.0p) - Table lists the exceedance percentages of the respective heatwave characteristic for CMIP6 multi-model ensemble (MME) SSP1-2.6 for data subdivided by Global Warming Levels (GWLs). The Table shows the region name, dates of the reference event, name of the heatwave characteristic ('Parameter_Name'), the reference value of the respective characteristic ('Parameter'), the respective reference data set ('Reanalysis'), the total number of heatwaves in the respective sample ('total_HW_num'), the number of heatwaves in the sample exceeding the reference value ('Parameter_Exceedance_Number'), and the exceedance percentage ('Parameter_Exceedance_Percentage'), the data subset type, the CMIP generation, and the repsective experiment. Data based on ERA5

Supplementary Data 359: Heatwave Characteristic Exceedange Percentages (Tx97.5p) - Table lists the exceedance percentages of the respective heatwave characteristic for CMIP6 multi-model ensemble (MME) SSP1-2.6 for data subdivided by Global Warming Levels (GWLs). The Table shows the region name, dates of the reference event, name of the heatwave characteristic ('Parameter_Name'), the reference value of the respective characteristic ('Parameter'), the respective reference data set ('Reanalysis'), the total number of heatwaves in the respective sample ('total_HW_num'), the number of heatwaves in the sample exceeding the reference value ('Parameter_Exceedance_Number'), and the exceedance percentage ('Parameter_Exceedance_Percentage'), the data subset type, the CMIP generation, and the repsective experiment. Data based on ERA5

Supplementary Data 360: Heatwave Characteristic Exceedange Percentages (Tx99.0p) - Table lists the exceedance percentages of the respective heatwave characteristic for CMIP6 multi-model ensemble (MME) SSP1-2.6 for data subdivided by Global Warming Levels (GWLs). The Table shows the region name, dates of the reference event, name of the heatwave characteristic ('Parameter_Name'), the reference value of the respective characteristic ('Parameter'), the respective reference data set ('Reanalysis'), the total number of heatwaves in the respective sample ('total_HW_num'), the number of heatwaves in the sample exceeding the reference value ('Parameter_Exceedance_Number'), and the exceedance percentage ('Parameter_Exceedance_Percentage'), the data subset type, the CMIP generation, and the repsective experiment. Data based on ERA5

Supplementary Data 361: Heatwave Characteristic Exceedange Percentages (Tx95.0p) - Table lists the exceedance percentages of the respective heatwave characteristic for CMIP6 multi-model ensemble (MME) SSP1-2.6 for data subdivided by Global Warming Levels (GWLs). The Table shows the region name, dates of the reference event, name of the heatwave characteristic ('Parameter_Name'), the reference value of the respective characteristic ('Parameter'), the respective reference data set ('Reanalysis'), the total number of heatwaves in the respective sample ('total_HW_num'), the number of heatwaves in the sample exceeding the reference value ('Parameter_Exceedance_Number'), and the exceedance percentage ('Parameter_Exceedance_Percentage'), the data subset type, the CMIP generation, and the repsective experiment. Data based on ERA5

Supplementary Data 362: Heatwave Characteristic Exceedange Percentages (Tx97.5p) - Table lists the exceedance percentages of the respective heatwave characteristic for CMIP6 multi-model ensemble (MME) SSP1-2.6 for data subdivided by Global Warming Levels (GWLs). The Table shows the region name, dates of the reference event, name of the heatwave characteristic ('Parameter_Name'), the reference value of the respective characteristic ('Parameter'), the respective reference data set ('Reanalysis'), the total number of heatwaves in the respective sample ('total_HW_num'), the number of heatwaves in the sample exceeding the reference value ('Parameter_Exceedance_Number'), and the exceedance percentage ('Parameter_Exceedance_Percentage'), the data subset type, the CMIP generation, and the repsective experiment. Data based on ERA5

Supplementary Data 363: Heatwave Characteristic Exceedange Percentages (Tx99.0p) - Table lists the exceedance percentages of the respective heatwave characteristic for CMIP6 multi-model ensemble (MME) SSP1-2.6 for data subdivided by Global Warming Levels (GWLs). The Table shows the region name, dates of the reference event, name of the heatwave characteristic ('Parameter_Name'), the reference value of the respective characteristic ('Parameter'), the respective reference data set ('Reanalysis'), the total number of heatwaves in the respective sample ('total_HW_num'), the number of heatwaves in the sample exceeding the reference value ('Parameter_Exceedance_Number'), and the exceedance percentage ('Parameter_Exceedance_Percentage'), the data subset type, the CMIP generation, and the repsective experiment. Data based on ERA5

Supplementary Data 364: Heatwave Characteristic Exceedange Percentages (Tx95.0p) - Table lists the exceedance percentages of the respective heatwave characteristic for CMIP6 multi-model ensemble (MME) SSP1-2.6 for data subdivided by Global Warming Levels (GWLs). The Table shows the region name, dates of the reference event, name of the heatwave characteristic ('Parameter_Name'), the reference value of the respective characteristic ('Parameter'), the respective reference data set ('Reanalysis'), the total number of heatwaves in the respective sample ('total_HW_num'), the number of heatwaves in the sample exceeding the reference value ('Parameter_Exceedance_Number'), and the exceedance percentage ('Parameter_Exceedance_Percentage'), the data subset type, the CMIP generation, and the repsective experiment. Data based on ERA5

Supplementary Data 365: Heatwave Characteristic Exceedange Percentages (Tx97.5p) - Table lists the exceedance percentages of the respective heatwave characteristic for CMIP6 multi-model ensemble (MME) SSP1-2.6 for data subdivided by Global Warming Levels (GWLs). The Table shows the region name, dates of the reference event, name of the heatwave characteristic ('Parameter_Name'), the reference value of the respective characteristic ('Parameter'), the respective reference data set ('Reanalysis'), the total number of heatwaves in the respective sample ('total_HW_num'), the number of heatwaves in the sample exceeding the reference value ('Parameter_Exceedance_Number'), and the exceedance percentage ('Parameter_Exceedance_Percentage'), the data subset type, the CMIP generation, and the repsective experiment. Data based on ERA5

Supplementary Data 366: Heatwave Characteristic Exceedange Percentages (Tx99.0p) - Table lists the exceedance percentages of the respective heatwave characteristic for CMIP6 multi-model ensemble (MME) SSP1-2.6 for data subdivided by Global Warming Levels (GWLs). The Table shows the region name, dates of the reference event, name of the heatwave characteristic ('Parameter_Name'), the reference value of the

respective characteristic ('Parameter'), the respective reference data set ('Reanalysis'), the total number of heatwaves in the respective sample ('total_HW_num'), the number of heatwaves in the sample exceeding the reference value ('Parameter_Exceedance_Number'), and the exceedance percentage ('Parameter_Exceedance_Percentage'), the data subset type, the CMIP generation, and the repsective experiment. Data based on ERA5

Supplementary Data 367: Heatwave Characteristic Exceedange Percentages (Tx95.0p) - Table lists the exceedance percentages of the respective heatwave characteristic for CMIP6 multi-model ensemble (MME) SSP2-4.5 for data subdivided by Global Warming Levels (GWLs). The Table shows the region name, dates of the reference event, name of the heatwave characteristic ('Parameter_Name'), the reference value of the respective characteristic ('Parameter'), the respective reference data set ('Reanalysis'), the total number of heatwaves in the respective sample ('total_HW_num'), the number of heatwaves in the sample exceeding the reference value ('Parameter_Exceedance_Number'), and the exceedance percentage ('Parameter_Exceedance_Percentage'), the data subset type, the CMIP generation, and the repsective experiment. Data based on ERA5

Supplementary Data 368: Heatwave Characteristic Exceedange Percentages (Tx97.5p) - Table lists the exceedance percentages of the respective heatwave characteristic for CMIP6 multi-model ensemble (MME) SSP2-4.5 for data subdivided by Global Warming Levels (GWLs). The Table shows the region name, dates of the reference event, name of the heatwave characteristic ('Parameter_Name'), the reference value of the respective characteristic ('Parameter'), the respective reference data set ('Reanalysis'), the total number of heatwaves in the respective sample ('total_HW_num'), the number of heatwaves in the sample exceeding the reference value ('Parameter_Exceedance_Number'), and the exceedance percentage ('Parameter_Exceedance_Percentage'), the data subset type, the CMIP generation, and the repsective experiment. Data based on ERA5

Supplementary Data 369: Heatwave Characteristic Exceedange Percentages (Tx99.0p) - Table lists the exceedance percentages of the respective heatwave characteristic for CMIP6 multi-model ensemble (MME) SSP2-4.5 for data subdivided by Global Warming Levels (GWLs). The Table shows the region name, dates of the reference event, name of the heatwave characteristic ('Parameter_Name'), the reference value of the respective characteristic ('Parameter'), the respective reference data set ('Reanalysis'), the total number of heatwaves in the respective sample ('total_HW_num'), the number of heatwaves in the sample exceeding the reference value ('Parameter_Exceedance_Number'), and the exceedance percentage ('Parameter_Exceedance_Percentage'), the data subset type, the CMIP generation, and the repsective experiment. Data based on ERA5

Supplementary Data 370: Heatwave Characteristic Exceedange Percentages (Tx95.0p) - Table lists the exceedance percentages of the respective heatwave characteristic for CMIP6 multi-model ensemble (MME) SSP2-4.5 for data subdivided by Global Warming Levels (GWLs). The Table shows the region name, dates of the reference event, name of the heatwave characteristic ('Parameter_Name'), the reference value of the respective characteristic ('Parameter'), the respective reference data set ('Reanalysis'), the total number of heatwaves in the respective sample ('total_HW_num'), the number of heatwaves in the sample exceeding the reference value ('Parameter_Exceedance_Number'), and the exceedance percentage

('Parameter_Exceedance_Percentage'), the data subset type, the CMIP generation, and the repsective experiment. Data based on ERA5

Supplementary Data 371: Heatwave Characteristic Exceedange Percentages (Tx97.5p) - Table lists the exceedance percentages of the respective heatwave characteristic for CMIP6 multi-model ensemble (MME) SSP2-4.5 for data subdivided by Global Warming Levels (GWLs). The Table shows the region name, dates of the reference event, name of the heatwave characteristic ('Parameter_Name'), the reference value of the respective characteristic ('Parameter'), the respective reference data set ('Reanalysis'), the total number of heatwaves in the respective sample ('total_HW_num'), the number of heatwaves in the sample exceeding the reference value ('Parameter_Exceedance_Number'), and the exceedance percentage ('Parameter_Exceedance_Percentage'), the data subset type, the CMIP generation, and the repsective experiment. Data based on ERA5

Supplementary Data 372: Heatwave Characteristic Exceedange Percentages (Tx99.0p) - Table lists the exceedance percentages of the respective heatwave characteristic for CMIP6 multi-model ensemble (MME) SSP2-4.5 for data subdivided by Global Warming Levels (GWLs). The Table shows the region name, dates of the reference event, name of the heatwave characteristic ('Parameter_Name'), the reference value of the respective characteristic ('Parameter'), the respective reference data set ('Reanalysis'), the total number of heatwaves in the respective sample ('total_HW_num'), the number of heatwaves in the sample exceeding the reference value ('Parameter_Exceedance_Number'), and the exceedance percentage ('Parameter_Exceedance_Percentage'), the data subset type, the CMIP generation, and the repsective experiment. Data based on ERA5

Supplementary Data 373: Heatwave Characteristic Exceedange Percentages (Tx95.0p) - Table lists the exceedance percentages of the respective heatwave characteristic for CMIP6 multi-model ensemble (MME) SSP2-4.5 for data subdivided by Global Warming Levels (GWLs). The Table shows the region name, dates of the reference event, name of the heatwave characteristic ('Parameter_Name'), the reference value of the respective characteristic ('Parameter'), the respective reference data set ('Reanalysis'), the total number of heatwaves in the respective sample ('total_HW_num'), the number of heatwaves in the sample exceeding the reference value ('Parameter_Exceedance_Number'), and the exceedance percentage ('Parameter_Exceedance_Percentage'), the data subset type, the CMIP generation, and the repsective experiment. Data based on ERA5

Supplementary Data 374: Heatwave Characteristic Exceedange Percentages (Tx97.5p) - Table lists the exceedance percentages of the respective heatwave characteristic for CMIP6 multi-model ensemble (MME) SSP2-4.5 for data subdivided by Global Warming Levels (GWLs). The Table shows the region name, dates of the reference event, name of the heatwave characteristic ('Parameter_Name'), the reference value of the respective characteristic ('Parameter'), the respective reference data set ('Reanalysis'), the total number of heatwaves in the respective sample ('total_HW_num'), the number of heatwaves in the sample exceeding the reference value ('Parameter_Exceedance_Number'), and the exceedance percentage ('Parameter_Exceedance_Percentage'), the data subset type, the CMIP generation, and the repsective experiment. Data based on ERA5

Supplementary Data 375: Heatwave Characteristic Exceedange Percentages (Tx99.0p) - Table lists the exceedance percentages of the respective heatwave characteristic for CMIP6 multi-model ensemble (MME) SSP2-4.5 for data subdivided by Global Warming Levels (GWLs). The Table shows the region name, dates of the reference event, name of the heatwave characteristic ('Parameter_Name'), the reference value of the respective characteristic ('Parameter'), the respective reference data set ('Reanalysis'), the total number of heatwaves in the respective sample ('total_HW_num'), the number of heatwaves in the sample exceeding the reference value ('Parameter_Exceedance_Number'), and the exceedance percentage ('Parameter_Exceedance_Percentage'), the data subset type, the CMIP generation, and the repsective experiment. Data based on ERA5

Supplementary Data 376: Heatwave Characteristic Exceedange Percentages (Tx95.0p) - Table lists the exceedance percentages of the respective heatwave characteristic for CMIP6 multi-model ensemble (MME) SSP2-4.5 for data subdivided by Global Warming Levels (GWLs). The Table shows the region name, dates of the reference event, name of the heatwave characteristic ('Parameter_Name'), the reference value of the respective characteristic ('Parameter'), the respective reference data set ('Reanalysis'), the total number of heatwaves in the respective sample ('total_HW_num'), the number of heatwaves in the sample exceeding the reference value ('Parameter_Exceedance_Number'), and the exceedance percentage ('Parameter_Exceedance_Percentage'), the data subset type, the CMIP generation, and the repsective experiment. Data based on ERA5

Supplementary Data 377: Heatwave Characteristic Exceedange Percentages (Tx97.5p) - Table lists the exceedance percentages of the respective heatwave characteristic for CMIP6 multi-model ensemble (MME) SSP2-4.5 for data subdivided by Global Warming Levels (GWLs). The Table shows the region name, dates of the reference event, name of the heatwave characteristic ('Parameter_Name'), the reference value of the respective characteristic ('Parameter'), the respective reference data set ('Reanalysis'), the total number of heatwaves in the respective sample ('total_HW_num'), the number of heatwaves in the sample exceeding the reference value ('Parameter_Exceedance_Number'), and the exceedance percentage ('Parameter_Exceedance_Percentage'), the data subset type, the CMIP generation, and the repsective experiment. Data based on ERA5

Supplementary Data 378: Heatwave Characteristic Exceedange Percentages (Tx99.0p) - Table lists the exceedance percentages of the respective heatwave characteristic for CMIP6 multi-model ensemble (MME) SSP2-4.5 for data subdivided by Global Warming Levels (GWLs). The Table shows the region name, dates of the reference event, name of the heatwave characteristic ('Parameter_Name'), the reference value of the respective characteristic ('Parameter'), the respective reference data set ('Reanalysis'), the total number of heatwaves in the respective sample ('total_HW_num'), the number of heatwaves in the sample exceeding the reference value ('Parameter_Exceedance_Number'), and the exceedance percentage ('Parameter_Exceedance_Percentage'), the data subset type, the CMIP generation, and the repsective experiment. Data based on ERA5

Supplementary Data 379: Heatwave Characteristic Exceedange Percentages (Tx95.0p) - Table lists the exceedance percentages of the respective heatwave characteristic for CMIP6 multi-model ensemble (MME) SSP2-4.5 for data subdivided by Global Warming Levels (GWLs). The Table shows the region name, dates of the reference event, name of the heatwave characteristic ('Parameter_Name'), the reference value of the

respective characteristic ('Parameter'), the respective reference data set ('Reanalysis'), the total number of heatwaves in the respective sample ('total_HW_num'), the number of heatwaves in the sample exceeding the reference value ('Parameter_Exceedance_Number'), and the exceedance percentage ('Parameter_Exceedance_Percentage'), the data subset type, the CMIP generation, and the repsective experiment. Data based on ERA5

Supplementary Data 380: Heatwave Characteristic Exceedange Percentages (Tx97.5p) - Table lists the exceedance percentages of the respective heatwave characteristic for CMIP6 multi-model ensemble (MME) SSP2-4.5 for data subdivided by Global Warming Levels (GWLs). The Table shows the region name, dates of the reference event, name of the heatwave characteristic ('Parameter_Name'), the reference value of the respective characteristic ('Parameter'), the respective reference data set ('Reanalysis'), the total number of heatwaves in the respective sample ('total_HW_num'), the number of heatwaves in the sample exceeding the reference value ('Parameter_Exceedance_Number'), and the exceedance percentage ('Parameter_Exceedance_Percentage'), the data subset type, the CMIP generation, and the repsective experiment. Data based on ERA5

Supplementary Data 381: Heatwave Characteristic Exceedange Percentages (Tx99.0p) - Table lists the exceedance percentages of the respective heatwave characteristic for CMIP6 multi-model ensemble (MME) SSP2-4.5 for data subdivided by Global Warming Levels (GWLs). The Table shows the region name, dates of the reference event, name of the heatwave characteristic ('Parameter_Name'), the reference value of the respective characteristic ('Parameter'), the respective reference data set ('Reanalysis'), the total number of heatwaves in the respective sample ('total_HW_num'), the number of heatwaves in the sample exceeding the reference value ('Parameter_Exceedance_Number'), and the exceedance percentage ('Parameter_Exceedance_Percentage'), the data subset type, the CMIP generation, and the repsective experiment. Data based on ERA5

Supplementary Data 382: Heatwave Characteristic Exceedange Percentages (Tx95.0p) - Table lists the exceedance percentages of the respective heatwave characteristic for CMIP6 multi-model ensemble (MME) SSP2-4.5 for data subdivided by Global Warming Levels (GWLs). The Table shows the region name, dates of the reference event, name of the heatwave characteristic ('Parameter_Name'), the reference value of the respective characteristic ('Parameter'), the respective reference data set ('Reanalysis'), the total number of heatwaves in the respective sample ('total_HW_num'), the number of heatwaves in the sample exceeding the reference value ('Parameter_Exceedance_Number'), and the exceedance percentage ('Parameter_Exceedance_Percentage'), the data subset type, the CMIP generation, and the repsective experiment. Data based on ERA5

Supplementary Data 383: Heatwave Characteristic Exceedange Percentages (Tx97.5p) - Table lists the exceedance percentages of the respective heatwave characteristic for CMIP6 multi-model ensemble (MME) SSP2-4.5 for data subdivided by Global Warming Levels (GWLs). The Table shows the region name, dates of the reference event, name of the heatwave characteristic ('Parameter_Name'), the reference value of the respective characteristic ('Parameter'), the respective reference data set ('Reanalysis'), the total number of heatwaves in the respective sample ('total_HW_num'), the number of heatwaves in the sample exceeding the reference value ('Parameter_Exceedance_Number'), and the exceedance percentage

('Parameter_Exceedance_Percentage'), the data subset type, the CMIP generation, and the repsective experiment. Data based on ERA5

Supplementary Data 384: Heatwave Characteristic Exceedange Percentages (Tx99.0p) - Table lists the exceedance percentages of the respective heatwave characteristic for CMIP6 multi-model ensemble (MME) SSP2-4.5 for data subdivided by Global Warming Levels (GWLs). The Table shows the region name, dates of the reference event, name of the heatwave characteristic ('Parameter_Name'), the reference value of the respective characteristic ('Parameter'), the respective reference data set ('Reanalysis'), the total number of heatwaves in the respective sample ('total_HW_num'), the number of heatwaves in the sample exceeding the reference value ('Parameter_Exceedance_Number'), and the exceedance percentage ('Parameter_Exceedance_Percentage'), the data subset type, the CMIP generation, and the repsective experiment. Data based on ERA5

Supplementary Data 385: Heatwave Characteristic Exceedange Percentages (Tx95.0p) - Table lists the exceedance percentages of the respective heatwave characteristic for CMIP6 multi-model ensemble (MME) SSP3-7.0 for data subdivided by Global Warming Levels (GWLs). The Table shows the region name, dates of the reference event, name of the heatwave characteristic ('Parameter_Name'), the reference value of the respective characteristic ('Parameter'), the respective reference data set ('Reanalysis'), the total number of heatwaves in the respective sample ('total_HW_num'), the number of heatwaves in the sample exceeding the reference value ('Parameter_Exceedance_Number'), and the exceedance percentage ('Parameter_Exceedance_Percentage'), the data subset type, the CMIP generation, and the repsective experiment. Data based on ERA5

Supplementary Data 386: Heatwave Characteristic Exceedange Percentages (Tx97.5p) - Table lists the exceedance percentages of the respective heatwave characteristic for CMIP6 multi-model ensemble (MME) SSP3-7.0 for data subdivided by Global Warming Levels (GWLs). The Table shows the region name, dates of the reference event, name of the heatwave characteristic ('Parameter_Name'), the reference value of the respective characteristic ('Parameter'), the respective reference data set ('Reanalysis'), the total number of heatwaves in the respective sample ('total_HW_num'), the number of heatwaves in the sample exceeding the reference value ('Parameter_Exceedance_Number'), and the exceedance percentage ('Parameter_Exceedance_Percentage'), the data subset type, the CMIP generation, and the repsective experiment. Data based on ERA5

Supplementary Data 387: Heatwave Characteristic Exceedange Percentages (Tx99.0p) - Table lists the exceedance percentages of the respective heatwave characteristic for CMIP6 multi-model ensemble (MME) SSP3-7.0 for data subdivided by Global Warming Levels (GWLs). The Table shows the region name, dates of the reference event, name of the heatwave characteristic ('Parameter_Name'), the reference value of the respective characteristic ('Parameter'), the respective reference data set ('Reanalysis'), the total number of heatwaves in the respective sample ('total_HW_num'), the number of heatwaves in the sample exceeding the reference value ('Parameter_Exceedance_Number'), and the exceedance percentage ('Parameter_Exceedance_Percentage'), the data subset type, the CMIP generation, and the repsective experiment. Data based on ERA5

Supplementary Data 388: Heatwave Characteristic Exceedange Percentages (Tx95.0p) - Table lists the exceedance percentages of the respective heatwave characteristic for CMIP6 multi-model ensemble (MME) SSP3-7.0 for data subdivided by Global Warming Levels (GWLs). The Table shows the region name, dates of the reference event, name of the heatwave characteristic ('Parameter_Name'), the reference value of the respective characteristic ('Parameter'), the respective reference data set ('Reanalysis'), the total number of heatwaves in the respective sample ('total_HW_num'), the number of heatwaves in the sample exceeding the reference value ('Parameter_Exceedance_Number'), and the exceedance percentage ('Parameter_Exceedance_Percentage'), the data subset type, the CMIP generation, and the repsective experiment. Data based on ERA5

Supplementary Data 389: Heatwave Characteristic Exceedange Percentages (Tx97.5p) - Table lists the exceedance percentages of the respective heatwave characteristic for CMIP6 multi-model ensemble (MME) SSP3-7.0 for data subdivided by Global Warming Levels (GWLs). The Table shows the region name, dates of the reference event, name of the heatwave characteristic ('Parameter_Name'), the reference value of the respective characteristic ('Parameter'), the respective reference data set ('Reanalysis'), the total number of heatwaves in the respective sample ('total_HW_num'), the number of heatwaves in the sample exceeding the reference value ('Parameter_Exceedance_Number'), and the exceedance percentage ('Parameter_Exceedance_Percentage'), the data subset type, the CMIP generation, and the repsective experiment. Data based on ERA5

Supplementary Data 390: Heatwave Characteristic Exceedange Percentages (Tx99.0p) - Table lists the exceedance percentages of the respective heatwave characteristic for CMIP6 multi-model ensemble (MME) SSP3-7.0 for data subdivided by Global Warming Levels (GWLs). The Table shows the region name, dates of the reference event, name of the heatwave characteristic ('Parameter_Name'), the reference value of the respective characteristic ('Parameter'), the respective reference data set ('Reanalysis'), the total number of heatwaves in the respective sample ('total_HW_num'), the number of heatwaves in the sample exceeding the reference value ('Parameter_Exceedance_Number'), and the exceedance percentage ('Parameter_Exceedance_Percentage'), the data subset type, the CMIP generation, and the repsective experiment. Data based on ERA5

Supplementary Data 391: Heatwave Characteristic Exceedange Percentages (Tx95.0p) - Table lists the exceedance percentages of the respective heatwave characteristic for CMIP6 multi-model ensemble (MME) SSP3-7.0 for data subdivided by Global Warming Levels (GWLs). The Table shows the region name, dates of the reference event, name of the heatwave characteristic ('Parameter_Name'), the reference value of the respective characteristic ('Parameter'), the respective reference data set ('Reanalysis'), the total number of heatwaves in the respective sample ('total_HW_num'), the number of heatwaves in the sample exceeding the reference value ('Parameter_Exceedance_Number'), and the exceedance percentage ('Parameter_Exceedance_Percentage'), the data subset type, the CMIP generation, and the repsective experiment. Data based on ERA5

Supplementary Data 392: Heatwave Characteristic Exceedange Percentages (Tx97.5p) - Table lists the exceedance percentages of the respective heatwave characteristic for CMIP6 multi-model ensemble (MME) SSP3-7.0 for data subdivided by Global Warming Levels (GWLs). The Table shows the region name, dates of the reference event, name of the heatwave characteristic ('Parameter_Name'), the reference value of the

respective characteristic ('Parameter'), the respective reference data set ('Reanalysis'), the total number of heatwaves in the respective sample ('total_HW_num'), the number of heatwaves in the sample exceeding the reference value ('Parameter_Exceedance_Number'), and the exceedance percentage ('Parameter_Exceedance_Percentage'), the data subset type, the CMIP generation, and the repsective experiment. Data based on ERA5

Supplementary Data 393: Heatwave Characteristic Exceedange Percentages (Tx99.0p) - Table lists the exceedance percentages of the respective heatwave characteristic for CMIP6 multi-model ensemble (MME) SSP3-7.0 for data subdivided by Global Warming Levels (GWLs). The Table shows the region name, dates of the reference event, name of the heatwave characteristic ('Parameter_Name'), the reference value of the respective characteristic ('Parameter'), the respective reference data set ('Reanalysis'), the total number of heatwaves in the respective sample ('total_HW_num'), the number of heatwaves in the sample exceeding the reference value ('Parameter_Exceedance_Number'), and the exceedance percentage ('Parameter_Exceedance_Percentage'), the data subset type, the CMIP generation, and the repsective experiment. Data based on ERA5

Supplementary Data 394: Heatwave Characteristic Exceedange Percentages (Tx95.0p) - Table lists the exceedance percentages of the respective heatwave characteristic for CMIP6 multi-model ensemble (MME) SSP3-7.0 for data subdivided by Global Warming Levels (GWLs). The Table shows the region name, dates of the reference event, name of the heatwave characteristic ('Parameter_Name'), the reference value of the respective characteristic ('Parameter'), the respective reference data set ('Reanalysis'), the total number of heatwaves in the respective sample ('total_HW_num'), the number of heatwaves in the sample exceeding the reference value ('Parameter_Exceedance_Number'), and the exceedance percentage ('Parameter_Exceedance_Percentage'), the data subset type, the CMIP generation, and the repsective experiment. Data based on ERA5

Supplementary Data 395: Heatwave Characteristic Exceedange Percentages (Tx97.5p) - Table lists the exceedance percentages of the respective heatwave characteristic for CMIP6 multi-model ensemble (MME) SSP3-7.0 for data subdivided by Global Warming Levels (GWLs). The Table shows the region name, dates of the reference event, name of the heatwave characteristic ('Parameter_Name'), the reference value of the respective characteristic ('Parameter'), the respective reference data set ('Reanalysis'), the total number of heatwaves in the respective sample ('total_HW_num'), the number of heatwaves in the sample exceeding the reference value ('Parameter_Exceedance_Number'), and the exceedance percentage ('Parameter_Exceedance_Percentage'), the data subset type, the CMIP generation, and the repsective experiment. Data based on ERA5

Supplementary Data 396: Heatwave Characteristic Exceedange Percentages (Tx99.0p) - Table lists the exceedance percentages of the respective heatwave characteristic for CMIP6 multi-model ensemble (MME) SSP3-7.0 for data subdivided by Global Warming Levels (GWLs). The Table shows the region name, dates of the reference event, name of the heatwave characteristic ('Parameter_Name'), the reference value of the respective characteristic ('Parameter'), the respective reference data set ('Reanalysis'), the total number of heatwaves in the respective sample ('total_HW_num'), the number of heatwaves in the sample exceeding the reference value ('Parameter_Exceedance_Number'), and the exceedance percentage

('Parameter_Exceedance_Percentage'), the data subset type, the CMIP generation, and the repsective experiment. Data based on ERA5

Supplementary Data 397: Heatwave Characteristic Exceedange Percentages (Tx95.0p) - Table lists the exceedance percentages of the respective heatwave characteristic for CMIP6 multi-model ensemble (MME) SSP3-7.0 for data subdivided by Global Warming Levels (GWLs). The Table shows the region name, dates of the reference event, name of the heatwave characteristic ('Parameter_Name'), the reference value of the respective characteristic ('Parameter'), the respective reference data set ('Reanalysis'), the total number of heatwaves in the respective sample ('total_HW_num'), the number of heatwaves in the sample exceeding the reference value ('Parameter_Exceedance_Number'), and the exceedance percentage ('Parameter_Exceedance_Percentage'), the data subset type, the CMIP generation, and the repsective experiment. Data based on ERA5

Supplementary Data 398: Heatwave Characteristic Exceedange Percentages (Tx97.5p) - Table lists the exceedance percentages of the respective heatwave characteristic for CMIP6 multi-model ensemble (MME) SSP3-7.0 for data subdivided by Global Warming Levels (GWLs). The Table shows the region name, dates of the reference event, name of the heatwave characteristic ('Parameter_Name'), the reference value of the respective characteristic ('Parameter'), the respective reference data set ('Reanalysis'), the total number of heatwaves in the respective sample ('total_HW_num'), the number of heatwaves in the sample exceeding the reference value ('Parameter_Exceedance_Number'), and the exceedance percentage ('Parameter_Exceedance_Percentage'), the data subset type, the CMIP generation, and the repsective experiment. Data based on ERA5

Supplementary Data 399: Heatwave Characteristic Exceedange Percentages (Tx99.0p) - Table lists the exceedance percentages of the respective heatwave characteristic for CMIP6 multi-model ensemble (MME) SSP3-7.0 for data subdivided by Global Warming Levels (GWLs). The Table shows the region name, dates of the reference event, name of the heatwave characteristic ('Parameter_Name'), the reference value of the respective characteristic ('Parameter'), the respective reference data set ('Reanalysis'), the total number of heatwaves in the respective sample ('total_HW_num'), the number of heatwaves in the sample exceeding the reference value ('Parameter_Exceedance_Number'), and the exceedance percentage ('Parameter_Exceedance_Percentage'), the data subset type, the CMIP generation, and the repsective experiment. Data based on ERA5

Supplementary Data 400: Heatwave Characteristic Exceedange Percentages (Tx95.0p) - Table lists the exceedance percentages of the respective heatwave characteristic for CMIP6 multi-model ensemble (MME) SSP3-7.0 for data subdivided by Global Warming Levels (GWLs). The Table shows the region name, dates of the reference event, name of the heatwave characteristic ('Parameter_Name'), the reference value of the respective characteristic ('Parameter'), the respective reference data set ('Reanalysis'), the total number of heatwaves in the respective sample ('total_HW_num'), the number of heatwaves in the sample exceeding the reference value ('Parameter_Exceedance_Number'), and the exceedance percentage ('Parameter_Exceedance_Percentage'), the data subset type, the CMIP generation, and the repsective experiment. Data based on ERA5

Supplementary Data 401: Heatwave Characteristic Exceedange Percentages (Tx97.5p) - Table lists the exceedance percentages of the respective heatwave characteristic for CMIP6 multi-model ensemble (MME) SSP3-7.0 for data subdivided by Global Warming Levels (GWLs). The Table shows the region name, dates of the reference event, name of the heatwave characteristic ('Parameter_Name'), the reference value of the respective characteristic ('Parameter'), the respective reference data set ('Reanalysis'), the total number of heatwaves in the respective sample ('total_HW_num'), the number of heatwaves in the sample exceeding the reference value ('Parameter_Exceedance_Number'), and the exceedance percentage ('Parameter_Exceedance_Percentage'), the data subset type, the CMIP generation, and the repsective experiment. Data based on ERA5

Supplementary Data 402: Heatwave Characteristic Exceedange Percentages (Tx99.0p) - Table lists the exceedance percentages of the respective heatwave characteristic for CMIP6 multi-model ensemble (MME) SSP3-7.0 for data subdivided by Global Warming Levels (GWLs). The Table shows the region name, dates of the reference event, name of the heatwave characteristic ('Parameter_Name'), the reference value of the respective characteristic ('Parameter'), the respective reference data set ('Reanalysis'), the total number of heatwaves in the respective sample ('total_HW_num'), the number of heatwaves in the sample exceeding the reference value ('Parameter_Exceedance_Number'), and the exceedance percentage ('Parameter_Exceedance_Percentage'), the data subset type, the CMIP generation, and the repsective experiment. Data based on ERA5

Supplementary Data 403: Heatwave Characteristic Exceedange Percentages (Tx95.0p) - Table lists the exceedance percentages of the respective heatwave characteristic for CMIP6 multi-model ensemble (MME) SSP5-8.5 for data subdivided by Global Warming Levels (GWLs). The Table shows the region name, dates of the reference event, name of the heatwave characteristic ('Parameter_Name'), the reference value of the respective characteristic ('Parameter'), the respective reference data set ('Reanalysis'), the total number of heatwaves in the respective sample ('total_HW_num'), the number of heatwaves in the sample exceeding the reference value ('Parameter_Exceedance_Number'), and the exceedance percentage ('Parameter_Exceedance_Percentage'), the data subset type, the CMIP generation, and the repsective experiment. Data based on ERA5

Supplementary Data 404: Heatwave Characteristic Exceedange Percentages (Tx97.5p) - Table lists the exceedance percentages of the respective heatwave characteristic for CMIP6 multi-model ensemble (MME) SSP5-8.5 for data subdivided by Global Warming Levels (GWLs). The Table shows the region name, dates of the reference event, name of the heatwave characteristic ('Parameter_Name'), the reference value of the respective characteristic ('Parameter'), the respective reference data set ('Reanalysis'), the total number of heatwaves in the respective sample ('total_HW_num'), the number of heatwaves in the sample exceeding the reference value ('Parameter_Exceedance_Number'), and the exceedance percentage ('Parameter_Exceedance_Percentage'), the data subset type, the CMIP generation, and the repsective experiment. Data based on ERA5

Supplementary Data 405: Heatwave Characteristic Exceedange Percentages (Tx99.0p) - Table lists the exceedance percentages of the respective heatwave characteristic for CMIP6 multi-model ensemble (MME) SSP5-8.5 for data subdivided by Global Warming Levels (GWLs). The Table shows the region name, dates of the reference event, name of the heatwave characteristic ('Parameter_Name'), the reference value of the

respective characteristic ('Parameter'), the respective reference data set ('Reanalysis'), the total number of heatwaves in the respective sample ('total_HW_num'), the number of heatwaves in the sample exceeding the reference value ('Parameter_Exceedance_Number'), and the exceedance percentage ('Parameter_Exceedance_Percentage'), the data subset type, the CMIP generation, and the repsective experiment. Data based on ERA5

Supplementary Data 406: Heatwave Characteristic Exceedange Percentages (Tx95.0p) - Table lists the exceedance percentages of the respective heatwave characteristic for CMIP6 multi-model ensemble (MME) SSP5-8.5 for data subdivided by Global Warming Levels (GWLs). The Table shows the region name, dates of the reference event, name of the heatwave characteristic ('Parameter_Name'), the reference value of the respective characteristic ('Parameter'), the respective reference data set ('Reanalysis'), the total number of heatwaves in the respective sample ('total_HW_num'), the number of heatwaves in the sample exceeding the reference value ('Parameter_Exceedance_Number'), and the exceedance percentage ('Parameter_Exceedance_Percentage'), the data subset type, the CMIP generation, and the repsective experiment. Data based on ERA5

Supplementary Data 407: Heatwave Characteristic Exceedange Percentages (Tx97.5p) - Table lists the exceedance percentages of the respective heatwave characteristic for CMIP6 multi-model ensemble (MME) SSP5-8.5 for data subdivided by Global Warming Levels (GWLs). The Table shows the region name, dates of the reference event, name of the heatwave characteristic ('Parameter_Name'), the reference value of the respective characteristic ('Parameter'), the respective reference data set ('Reanalysis'), the total number of heatwaves in the respective sample ('total_HW_num'), the number of heatwaves in the sample exceeding the reference value ('Parameter_Exceedance_Number'), and the exceedance percentage ('Parameter_Exceedance_Percentage'), the data subset type, the CMIP generation, and the repsective experiment. Data based on ERA5

Supplementary Data 408: Heatwave Characteristic Exceedange Percentages (Tx99.0p) - Table lists the exceedance percentages of the respective heatwave characteristic for CMIP6 multi-model ensemble (MME) SSP5-8.5 for data subdivided by Global Warming Levels (GWLs). The Table shows the region name, dates of the reference event, name of the heatwave characteristic ('Parameter_Name'), the reference value of the respective characteristic ('Parameter'), the respective reference data set ('Reanalysis'), the total number of heatwaves in the respective sample ('total_HW_num'), the number of heatwaves in the sample exceeding the reference value ('Parameter_Exceedance_Number'), and the exceedance percentage ('Parameter_Exceedance_Percentage'), the data subset type, the CMIP generation, and the repsective experiment. Data based on ERA5

Supplementary Data 409: Heatwave Characteristic Exceedange Percentages (Tx95.0p) - Table lists the exceedance percentages of the respective heatwave characteristic for CMIP6 multi-model ensemble (MME) SSP5-8.5 for data subdivided by Global Warming Levels (GWLs). The Table shows the region name, dates of the reference event, name of the heatwave characteristic ('Parameter_Name'), the reference value of the respective characteristic ('Parameter'), the respective reference data set ('Reanalysis'), the total number of heatwaves in the respective sample ('total_HW_num'), the number of heatwaves in the sample exceeding the reference value ('Parameter_Exceedance_Number'), and the exceedance percentage

('Parameter_Exceedance_Percentage'), the data subset type, the CMIP generation, and the repsective experiment. Data based on ERA5

Supplementary Data 410: Heatwave Characteristic Exceedange Percentages (Tx97.5p) - Table lists the exceedance percentages of the respective heatwave characteristic for CMIP6 multi-model ensemble (MME) SSP5-8.5 for data subdivided by Global Warming Levels (GWLs). The Table shows the region name, dates of the reference event, name of the heatwave characteristic ('Parameter_Name'), the reference value of the respective characteristic ('Parameter'), the respective reference data set ('Reanalysis'), the total number of heatwaves in the respective sample ('total_HW_num'), the number of heatwaves in the sample exceeding the reference value ('Parameter_Exceedance_Number'), and the exceedance percentage ('Parameter_Exceedance_Percentage'), the data subset type, the CMIP generation, and the repsective experiment. Data based on ERA5

Supplementary Data 411: Heatwave Characteristic Exceedange Percentages (Tx99.0p) - Table lists the exceedance percentages of the respective heatwave characteristic for CMIP6 multi-model ensemble (MME) SSP5-8.5 for data subdivided by Global Warming Levels (GWLs). The Table shows the region name, dates of the reference event, name of the heatwave characteristic ('Parameter_Name'), the reference value of the respective characteristic ('Parameter'), the respective reference data set ('Reanalysis'), the total number of heatwaves in the respective sample ('total_HW_num'), the number of heatwaves in the sample exceeding the reference value ('Parameter_Exceedance_Number'), and the exceedance percentage ('Parameter_Exceedance_Percentage'), the data subset type, the CMIP generation, and the repsective experiment. Data based on ERA5

Supplementary Data 412: Heatwave Characteristic Exceedange Percentages (Tx95.0p) - Table lists the exceedance percentages of the respective heatwave characteristic for CMIP6 multi-model ensemble (MME) SSP5-8.5 for data subdivided by Global Warming Levels (GWLs). The Table shows the region name, dates of the reference event, name of the heatwave characteristic ('Parameter_Name'), the reference value of the respective characteristic ('Parameter'), the respective reference data set ('Reanalysis'), the total number of heatwaves in the respective sample ('total_HW_num'), the number of heatwaves in the sample exceeding the reference value ('Parameter_Exceedance_Number'), and the exceedance percentage ('Parameter_Exceedance_Percentage'), the data subset type, the CMIP generation, and the repsective experiment. Data based on ERA5

Supplementary Data 413: Heatwave Characteristic Exceedange Percentages (Tx97.5p) - Table lists the exceedance percentages of the respective heatwave characteristic for CMIP6 multi-model ensemble (MME) SSP5-8.5 for data subdivided by Global Warming Levels (GWLs). The Table shows the region name, dates of the reference event, name of the heatwave characteristic ('Parameter_Name'), the reference value of the respective characteristic ('Parameter'), the respective reference data set ('Reanalysis'), the total number of heatwaves in the respective sample ('total_HW_num'), the number of heatwaves in the sample exceeding the reference value ('Parameter_Exceedance_Number'), and the exceedance percentage ('Parameter_Exceedance_Percentage'), the data subset type, the CMIP generation, and the repsective experiment. Data based on ERA5

Supplementary Data 414: Heatwave Characteristic Exceedange Percentages (Tx99.0p) - Table lists the exceedance percentages of the respective heatwave characteristic for CMIP6 multi-model ensemble (MME) SSP5-8.5 for data subdivided by Global Warming Levels (GWLs). The Table shows the region name, dates of the reference event, name of the heatwave characteristic ('Parameter_Name'), the reference value of the respective characteristic ('Parameter'), the respective reference data set ('Reanalysis'), the total number of heatwaves in the respective sample ('total_HW_num'), the number of heatwaves in the sample exceeding the reference value ('Parameter_Exceedance_Number'), and the exceedance percentage ('Parameter_Exceedance_Percentage'), the data subset type, the CMIP generation, and the repsective experiment. Data based on ERA5

Supplementary Data 415: Heatwave Characteristic Exceedange Percentages (Tx95.0p) - Table lists the exceedance percentages of the respective heatwave characteristic for CMIP6 multi-model ensemble (MME) SSP5-8.5 for data subdivided by Global Warming Levels (GWLs). The Table shows the region name, dates of the reference event, name of the heatwave characteristic ('Parameter_Name'), the reference value of the respective characteristic ('Parameter'), the respective reference data set ('Reanalysis'), the total number of heatwaves in the respective sample ('total_HW_num'), the number of heatwaves in the sample exceeding the reference value ('Parameter_Exceedance_Number'), and the exceedance percentage ('Parameter_Exceedance_Percentage'), the data subset type, the CMIP generation, and the repsective experiment. Data based on ERA5

Supplementary Data 416: Heatwave Characteristic Exceedange Percentages (Tx97.5p) - Table lists the exceedance percentages of the respective heatwave characteristic for CMIP6 multi-model ensemble (MME) SSP5-8.5 for data subdivided by Global Warming Levels (GWLs). The Table shows the region name, dates of the reference event, name of the heatwave characteristic ('Parameter_Name'), the reference value of the respective characteristic ('Parameter'), the respective reference data set ('Reanalysis'), the total number of heatwaves in the respective sample ('total_HW_num'), the number of heatwaves in the sample exceeding the reference value ('Parameter_Exceedance_Number'), and the exceedance percentage ('Parameter_Exceedance_Percentage'), the data subset type, the CMIP generation, and the repsective experiment. Data based on ERA5

Supplementary Data 417: Heatwave Characteristic Exceedange Percentages (Tx99.0p) - Table lists the exceedance percentages of the respective heatwave characteristic for CMIP6 multi-model ensemble (MME) SSP5-8.5 for data subdivided by Global Warming Levels (GWLs). The Table shows the region name, dates of the reference event, name of the heatwave characteristic ('Parameter_Name'), the reference value of the respective characteristic ('Parameter'), the respective reference data set ('Reanalysis'), the total number of heatwaves in the respective sample ('total_HW_num'), the number of heatwaves in the sample exceeding the reference value ('Parameter_Exceedance_Number'), and the exceedance percentage ('Parameter_Exceedance_Percentage'), the data subset type, the CMIP generation, and the repsective experiment. Data based on ERA5

Supplementary Data 418: Heatwave Characteristic Exceedange Percentages (Tx95.0p) - Table lists the exceedance percentages of the respective heatwave characteristic for CMIP6 multi-model ensemble (MME) SSP5-8.5 for data subdivided by Global Warming Levels (GWLs). The Table shows the region name, dates of the reference event, name of the heatwave characteristic ('Parameter_Name'), the reference value of the

respective characteristic ('Parameter'), the respective reference data set ('Reanalysis'), the total number of heatwaves in the respective sample ('total_HW_num'), the number of heatwaves in the sample exceeding the reference value ('Parameter_Exceedance_Number'), and the exceedance percentage ('Parameter_Exceedance_Percentage'), the data subset type, the CMIP generation, and the repsective experiment. Data based on ERA5

Supplementary Data 419: Heatwave Characteristic Exceedange Percentages (Tx97.5p) - Table lists the exceedance percentages of the respective heatwave characteristic for CMIP6 multi-model ensemble (MME) SSP5-8.5 for data subdivided by Global Warming Levels (GWLs). The Table shows the region name, dates of the reference event, name of the heatwave characteristic ('Parameter_Name'), the reference value of the respective characteristic ('Parameter'), the respective reference data set ('Reanalysis'), the total number of heatwaves in the respective sample ('total_HW_num'), the number of heatwaves in the sample exceeding the reference value ('Parameter_Exceedance_Number'), and the exceedance percentage ('Parameter_Exceedance_Percentage'), the data subset type, the CMIP generation, and the repsective experiment. Data based on ERA5

Supplementary Data 420: Heatwave Characteristic Exceedange Percentages (Tx99.0p) - Table lists the exceedance percentages of the respective heatwave characteristic for CMIP6 multi-model ensemble (MME) SSP5-8.5 for data subdivided by Global Warming Levels (GWLs). The Table shows the region name, dates of the reference event, name of the heatwave characteristic ('Parameter_Name'), the reference value of the respective characteristic ('Parameter'), the respective reference data set ('Reanalysis'), the total number of heatwaves in the respective sample ('total_HW_num'), the number of heatwaves in the sample exceeding the reference value ('Parameter_Exceedance_Number'), and the exceedance percentage ('Parameter_Exceedance_Percentage'), the data subset type, the CMIP generation, and the repsective experiment. Data based on ERA5

Supplementary Data 421: Heatwave Characteristic Exceedange Percentages (Tx95.0p) - Table lists the exceedance percentages of the respective heatwave characteristic for CMIP6 multi-model ensemble (MME) SSP1-2.6 for entire dataset from 2020 to 2100. The Table shows the region name, dates of the reference event, name of the heatwave characteristic ('Parameter_Name'), the reference value of the respective characteristic ('Parameter'), the respective reference data set ('Reanalysis'), the total number of heatwaves in the respective sample ('total_HW_num'), the number of heatwaves in the sample exceeding the reference value ('Parameter_Exceedance_Number'), and the exceedance percentage ('Parameter_Exceedance_Percentage'), the data subset type, the CMIP generation, and the repsective experiment. Data based on ERA5

Supplementary Data 422: Heatwave Characteristic Exceedange Percentages (Tx97.5p) - Table lists the exceedance percentages of the respective heatwave characteristic for CMIP6 multi-model ensemble (MME) SSP1-2.6 for entire dataset from 2020 to 2100. The Table shows the region name, dates of the reference event, name of the heatwave characteristic ('Parameter_Name'), the reference value of the respective characteristic ('Parameter'), the respective reference data set ('Reanalysis'), the total number of heatwaves in the respective sample ('total_HW_num'), the number of heatwaves in the sample exceeding the reference value ('Parameter_Exceedance_Number'), and the exceedance percentage ('Parameter_Exceedance_Percentage'), the data subset type, the CMIP generation, and the repsective experiment. Data based on ERA5

Supplementary Data 423: Heatwave Characteristic Exceedange Percentages (Tx99.0p) - Table lists the exceedance percentages of the respective heatwave characteristic for CMIP6 multi-model ensemble (MME) SSP1-2.6 for entire dataset from 2020 to 2100. The Table shows the region name, dates of the reference event, name of the heatwave characteristic ('Parameter_Name'), the reference value of the respective characteristic ('Parameter'), the respective reference data set ('Reanalysis'), the total number of heatwaves in the respective sample ('total_HW_num'), the number of heatwaves in the sample exceeding the reference value ('Parameter_Exceedance_Number'), and the exceedance percentage ('Parameter_Exceedance_Percentage'), the data subset type, the CMIP generation, and the repsective experiment. Data based on ERA5

Supplementary Data 424: Heatwave Characteristic Exceedange Percentages (Tx95.0p) - Table lists the exceedance percentages of the respective heatwave characteristic for CMIP6 multi-model ensemble (MME) SSP1-2.6 for entire dataset from 2020 to 2100. The Table shows the region name, dates of the reference event, name of the heatwave characteristic ('Parameter_Name'), the reference value of the respective characteristic ('Parameter'), the respective reference data set ('Reanalysis'), the total number of heatwaves in the respective sample ('total_HW_num'), the number of heatwaves in the sample exceeding the reference value ('Parameter_Exceedance_Number'), and the exceedance percentage ('Parameter_Exceedance_Percentage'), the data subset type, the CMIP generation, and the repsective experiment. Data based on ERA5

Supplementary Data 425: Heatwave Characteristic Exceedange Percentages (Tx97.5p) - Table lists the exceedance percentages of the respective heatwave characteristic for CMIP6 multi-model ensemble (MME) SSP1-2.6 for entire dataset from 2020 to 2100. The Table shows the region name, dates of the reference event, name of the heatwave characteristic ('Parameter_Name'), the reference value of the respective characteristic ('Parameter'), the respective reference data set ('Reanalysis'), the total number of heatwaves in the respective sample ('total_HW_num'), the number of heatwaves in the sample exceeding the reference value ('Parameter_Exceedance_Number'), and the exceedance percentage ('Parameter_Exceedance_Percentage'), the data subset type, the CMIP generation, and the repsective experiment. Data based on ERA5

Supplementary Data 426: Heatwave Characteristic Exceedange Percentages (Tx99.0p) - Table lists the exceedance percentages of the respective heatwave characteristic for CMIP6 multi-model ensemble (MME) SSP1-2.6 for entire dataset from 2020 to 2100. The Table shows the region name, dates of the reference event, name of the heatwave characteristic ('Parameter_Name'), the reference value of the respective characteristic ('Parameter'), the respective reference data set ('Reanalysis'), the total number of heatwaves in the respective sample ('total_HW_num'), the number of heatwaves in the sample exceeding the reference value ('Parameter_Exceedance_Number'), and the exceedance percentage ('Parameter_Exceedance_Percentage'), the data subset type, the CMIP generation, and the repsective experiment. Data based on ERA5

Supplementary Data 427: Heatwave Characteristic Exceedange Percentages (Tx95.0p) - Table lists the exceedance percentages of the respective heatwave characteristic for CMIP6 multi-model ensemble (MME) SSP1-2.6 for entire dataset from 2020 to 2100. The Table shows the region name, dates of the reference event, name of the heatwave characteristic ('Parameter_Name'), the reference value of the respective characteristic ('Parameter'), the respective reference data set ('Reanalysis'), the total number of heatwaves in the respective sample ('total_HW_num'), the number of heatwaves in the sample exceeding the reference value ('Parameter_Exceedance_Number'), and the exceedance percentage ('Parameter_Exceedance_Percentage'), the data subset type, the CMIP generation, and the repsective experiment. Data based on ERA5

Supplementary Data 428: Heatwave Characteristic Exceedange Percentages (Tx97.5p) - Table lists the exceedance percentages of the respective heatwave characteristic for CMIP6 multi-model ensemble (MME) SSP1-2.6 for entire dataset from 2020 to 2100. The Table shows the region name, dates of the reference event, name of the heatwave characteristic ('Parameter_Name'), the reference value of the respective characteristic ('Parameter'), the respective reference data set ('Reanalysis'), the total number of heatwaves in the respective sample ('total_HW_num'), the number of heatwaves in the sample exceeding the reference value ('Parameter_Exceedance_Number'), and the exceedance percentage ('Parameter_Exceedance_Percentage'), the data subset type, the CMIP generation, and the repsective experiment. Data based on ERA5

Supplementary Data 429: Heatwave Characteristic Exceedange Percentages (Tx99.0p) - Table lists the exceedance percentages of the respective heatwave characteristic for CMIP6 multi-model ensemble (MME) SSP1-2.6 for entire dataset from 2020 to 2100. The Table shows the region name, dates of the reference event, name of the heatwave characteristic ('Parameter_Name'), the reference value of the respective characteristic ('Parameter'), the respective reference data set ('Reanalysis'), the total number of heatwaves in the respective sample ('total_HW_num'), the number of heatwaves in the sample exceeding the reference value ('Parameter_Exceedance_Number'), and the exceedance percentage ('Parameter_Exceedance_Percentage'), the data subset type, the CMIP generation, and the repsective experiment. Data based on ERA5

Supplementary Data 430: Heatwave Characteristic Exceedange Percentages (Tx95.0p) - Table lists the exceedance percentages of the respective heatwave characteristic for CMIP6 multi-model ensemble (MME) SSP1-2.6 for entire dataset from 2020 to 2100. The Table shows the region name, dates of the reference event, name of the heatwave characteristic ('Parameter_Name'), the reference value of the respective characteristic ('Parameter'), the respective reference data set ('Reanalysis'), the total number of heatwaves in the respective sample ('total_HW_num'), the number of heatwaves in the sample exceeding the reference value ('Parameter_Exceedance_Number'), and the exceedance percentage ('Parameter_Exceedance_Percentage'), the data subset type, the CMIP generation, and the repsective experiment. Data based on ERA5

Supplementary Data 431: Heatwave Characteristic Exceedange Percentages (Tx97.5p) - Table lists the exceedance percentages of the respective heatwave characteristic for CMIP6 multi-model ensemble (MME) SSP1-2.6 for entire dataset from 2020 to 2100. The Table shows the region name, dates of the reference event, name of the heatwave characteristic ('Parameter_Name'), the reference value of the respective characteristic ('Parameter'), the respective reference data set ('Reanalysis'), the total number of heatwaves in the respective sample ('total_HW_num'), the number of heatwaves in the sample exceeding the reference value ('Parameter_Exceedance_Number'), and the exceedance percentage ('Parameter_Exceedance_Percentage'), the data subset type, the CMIP generation, and the repsective experiment. Data based on ERA5

Supplementary Data 432: Heatwave Characteristic Exceedange Percentages (Tx99.0p) - Table lists the exceedance percentages of the respective heatwave characteristic for CMIP6 multi-model ensemble (MME) SSP1-2.6 for entire dataset from 2020 to 2100. The Table shows the region name, dates of the reference event, name of the heatwave characteristic ('Parameter_Name'), the reference value of the respective characteristic ('Parameter'), the respective reference data set ('Reanalysis'), the total number of heatwaves in the respective sample ('total_HW_num'), the number of heatwaves in the sample exceeding the reference value

('Parameter_Exceedance_Number'), and the exceedance percentage ('Parameter_Exceedance_Percentage'), the data subset type, the CMIP generation, and the repsective experiment. Data based on ERA5

Supplementary Data 433: Heatwave Characteristic Exceedange Percentages (Tx95.0p) - Table lists the exceedance percentages of the respective heatwave characteristic for CMIP6 multi-model ensemble (MME) SSP1-2.6 for entire dataset from 2020 to 2100. The Table shows the region name, dates of the reference event, name of the heatwave characteristic ('Parameter_Name'), the reference value of the respective characteristic ('Parameter'), the respective reference data set ('Reanalysis'), the total number of heatwaves in the respective sample ('total_HW_num'), the number of heatwaves in the sample exceeding the reference value ('Parameter_Exceedance_Number'), and the exceedance percentage ('Parameter_Exceedance_Percentage'), the data subset type, the CMIP generation, and the repsective experiment. Data based on ERA5

Supplementary Data 434: Heatwave Characteristic Exceedange Percentages (Tx97.5p) - Table lists the exceedance percentages of the respective heatwave characteristic for CMIP6 multi-model ensemble (MME) SSP1-2.6 for entire dataset from 2020 to 2100. The Table shows the region name, dates of the reference event, name of the heatwave characteristic ('Parameter_Name'), the reference value of the respective characteristic ('Parameter'), the respective reference data set ('Reanalysis'), the total number of heatwaves in the respective sample ('total_HW_num'), the number of heatwaves in the sample exceeding the reference value ('Parameter_Exceedance_Number'), and the exceedance percentage ('Parameter_Exceedance_Percentage'), the data subset type, the CMIP generation, and the repsective experiment. Data based on ERA5

Supplementary Data 435: Heatwave Characteristic Exceedange Percentages (Tx99.0p) - Table lists the exceedance percentages of the respective heatwave characteristic for CMIP6 multi-model ensemble (MME) SSP1-2.6 for entire dataset from 2020 to 2100. The Table shows the region name, dates of the reference event, name of the heatwave characteristic ('Parameter_Name'), the reference value of the respective characteristic ('Parameter'), the respective reference data set ('Reanalysis'), the total number of heatwaves in the respective sample ('total_HW_num'), the number of heatwaves in the sample exceeding the reference value ('Parameter_Exceedance_Number'), and the exceedance percentage ('Parameter_Exceedance_Percentage'), the data subset type, the CMIP generation, and the repsective experiment. Data based on ERA5

Supplementary Data 436: Heatwave Characteristic Exceedange Percentages (Tx95.0p) - Table lists the exceedance percentages of the respective heatwave characteristic for CMIP6 multi-model ensemble (MME) SSP1-2.6 for entire dataset from 2020 to 2100. The Table shows the region name, dates of the reference event, name of the heatwave characteristic ('Parameter_Name'), the reference value of the respective characteristic ('Parameter'), the respective reference data set ('Reanalysis'), the total number of heatwaves in the respective sample ('total_HW_num'), the number of heatwaves in the sample exceeding the reference value ('Parameter_Exceedance_Number'), and the exceedance percentage ('Parameter_Exceedance_Percentage'), the data subset type, the CMIP generation, and the repsective experiment. Data based on ERA5

Supplementary Data 437: Heatwave Characteristic Exceedange Percentages (Tx97.5p) - Table lists the exceedance percentages of the respective heatwave characteristic for CMIP6 multi-model ensemble (MME) SSP1-2.6 for entire dataset from 2020 to 2100. The Table shows the region name, dates of the reference event, name of the heatwave characteristic ('Parameter_Name'), the reference value of the respective characteristic

('Parameter'), the respective reference data set ('Reanalysis'), the total number of heatwaves in the respective sample ('total_HW_num'), the number of heatwaves in the sample exceeding the reference value ('Parameter_Exceedance_Number'), and the exceedance percentage ('Parameter_Exceedance_Percentage'), the data subset type, the CMIP generation, and the repsective experiment. Data based on ERA5

Supplementary Data 438: Heatwave Characteristic Exceedange Percentages (Tx99.0p) - Table lists the exceedance percentages of the respective heatwave characteristic for CMIP6 multi-model ensemble (MME) SSP1-2.6 for entire dataset from 2020 to 2100. The Table shows the region name, dates of the reference event, name of the heatwave characteristic ('Parameter_Name'), the reference value of the respective characteristic ('Parameter'), the respective reference data set ('Reanalysis'), the total number of heatwaves in the respective sample ('total_HW_num'), the number of heatwaves in the sample exceeding the reference value ('Parameter_Exceedance_Number'), and the exceedance percentage ('Parameter_Exceedance_Percentage'), the data subset type, the CMIP generation, and the repsective experiment. Data based on ERA5

Supplementary Data 439: Heatwave Characteristic Exceedange Percentages (Tx95.0p) - Table lists the exceedance percentages of the respective heatwave characteristic for CMIP6 multi-model ensemble (MME) SSP2-4.5 for entire dataset from 2020 to 2100. The Table shows the region name, dates of the reference event, name of the heatwave characteristic ('Parameter_Name'), the reference value of the respective characteristic ('Parameter'), the respective reference data set ('Reanalysis'), the total number of heatwaves in the respective sample ('total_HW_num'), the number of heatwaves in the sample exceeding the reference value ('Parameter_Exceedance_Number'), and the exceedance percentage ('Parameter_Exceedance_Percentage'), the data subset type, the CMIP generation, and the repsective experiment. Data based on ERA5

Supplementary Data 440: Heatwave Characteristic Exceedange Percentages (Tx97.5p) - Table lists the exceedance percentages of the respective heatwave characteristic for CMIP6 multi-model ensemble (MME) SSP2-4.5 for entire dataset from 2020 to 2100. The Table shows the region name, dates of the reference event, name of the heatwave characteristic ('Parameter_Name'), the reference value of the respective characteristic ('Parameter'), the respective reference data set ('Reanalysis'), the total number of heatwaves in the respective sample ('total_HW_num'), the number of heatwaves in the sample exceeding the reference value ('Parameter_Exceedance_Number'), and the exceedance percentage ('Parameter_Exceedance_Percentage'), the data subset type, the CMIP generation, and the repsective experiment. Data based on ERA5

Supplementary Data 441: Heatwave Characteristic Exceedange Percentages (Tx99.0p) - Table lists the exceedance percentages of the respective heatwave characteristic for CMIP6 multi-model ensemble (MME) SSP2-4.5 for entire dataset from 2020 to 2100. The Table shows the region name, dates of the reference event, name of the heatwave characteristic ('Parameter_Name'), the reference value of the respective characteristic ('Parameter'), the respective reference data set ('Reanalysis'), the total number of heatwaves in the respective sample ('total_HW_num'), the number of heatwaves in the sample exceeding the reference value ('Parameter_Exceedance_Number'), and the exceedance percentage ('Parameter_Exceedance_Percentage'), the data subset type, the CMIP generation, and the repsective experiment. Data based on ERA5

Supplementary Data 442: Heatwave Characteristic Exceedange Percentages (Tx95.0p) - Table lists the exceedance percentages of the respective heatwave characteristic for CMIP6 multi-model ensemble (MME)

SSP2-4.5 for entire dataset from 2020 to 2100. The Table shows the region name, dates of the reference event, name of the heatwave characteristic ('Parameter_Name'), the reference value of the respective characteristic ('Parameter'), the respective reference data set ('Reanalysis'), the total number of heatwaves in the respective sample ('total_HW_num'), the number of heatwaves in the sample exceeding the reference value ('Parameter_Exceedance_Number'), and the exceedance percentage ('Parameter_Exceedance_Percentage'), the data subset type, the CMIP generation, and the repsective experiment. Data based on ERA5

Supplementary Data 443: Heatwave Characteristic Exceedange Percentages (Tx97.5p) - Table lists the exceedance percentages of the respective heatwave characteristic for CMIP6 multi-model ensemble (MME) SSP2-4.5 for entire dataset from 2020 to 2100. The Table shows the region name, dates of the reference event, name of the heatwave characteristic ('Parameter_Name'), the reference value of the respective characteristic ('Parameter'), the respective reference data set ('Reanalysis'), the total number of heatwaves in the respective sample ('total_HW_num'), the number of heatwaves in the sample exceeding the reference value ('Parameter_Exceedance_Number'), and the exceedance percentage ('Parameter_Exceedance_Percentage'), the data subset type, the CMIP generation, and the repsective experiment. Data based on ERA5

Supplementary Data 444: Heatwave Characteristic Exceedange Percentages (Tx99.0p) - Table lists the exceedance percentages of the respective heatwave characteristic for CMIP6 multi-model ensemble (MME) SSP2-4.5 for entire dataset from 2020 to 2100. The Table shows the region name, dates of the reference event, name of the heatwave characteristic ('Parameter_Name'), the reference value of the respective characteristic ('Parameter'), the respective reference data set ('Reanalysis'), the total number of heatwaves in the respective sample ('total_HW_num'), the number of heatwaves in the sample exceeding the reference value ('Parameter_Exceedance_Number'), and the exceedance percentage ('Parameter_Exceedance_Percentage'), the data subset type, the CMIP generation, and the repsective experiment. Data based on ERA5

Supplementary Data 445: Heatwave Characteristic Exceedange Percentages (Tx95.0p) - Table lists the exceedance percentages of the respective heatwave characteristic for CMIP6 multi-model ensemble (MME) SSP2-4.5 for entire dataset from 2020 to 2100. The Table shows the region name, dates of the reference event, name of the heatwave characteristic ('Parameter_Name'), the reference value of the respective characteristic ('Parameter'), the respective reference data set ('Reanalysis'), the total number of heatwaves in the respective sample ('total_HW_num'), the number of heatwaves in the sample exceeding the reference value ('Parameter_Exceedance_Number'), and the exceedance percentage ('Parameter_Exceedance_Percentage'), the data subset type, the CMIP generation, and the repsective experiment. Data based on ERA5

Supplementary Data 446: Heatwave Characteristic Exceedange Percentages (Tx97.5p) - Table lists the exceedance percentages of the respective heatwave characteristic for CMIP6 multi-model ensemble (MME) SSP2-4.5 for entire dataset from 2020 to 2100. The Table shows the region name, dates of the reference event, name of the heatwave characteristic ('Parameter_Name'), the reference value of the respective characteristic ('Parameter'), the respective reference data set ('Reanalysis'), the total number of heatwaves in the respective sample ('total_HW_num'), the number of heatwaves in the sample exceeding the reference value ('Parameter_Exceedance_Number'), and the exceedance percentage ('Parameter_Exceedance_Percentage'), the data subset type, the CMIP generation, and the repsective experiment. Data based on ERA5

Supplementary Data 447: Heatwave Characteristic Exceedange Percentages (Tx99.0p) - Table lists the exceedance percentages of the respective heatwave characteristic for CMIP6 multi-model ensemble (MME) SSP2-4.5 for entire dataset from 2020 to 2100. The Table shows the region name, dates of the reference event, name of the heatwave characteristic ('Parameter_Name'), the reference value of the respective characteristic ('Parameter'), the respective reference data set ('Reanalysis'), the total number of heatwaves in the respective sample ('total_HW_num'), the number of heatwaves in the sample exceeding the reference value ('Parameter_Exceedance_Number'), and the exceedance percentage ('Parameter_Exceedance_Percentage'), the data subset type, the CMIP generation, and the repsective experiment. Data based on ERA5

Supplementary Data 448: Heatwave Characteristic Exceedange Percentages (Tx95.0p) - Table lists the exceedance percentages of the respective heatwave characteristic for CMIP6 multi-model ensemble (MME) SSP2-4.5 for entire dataset from 2020 to 2100. The Table shows the region name, dates of the reference event, name of the heatwave characteristic ('Parameter_Name'), the reference value of the respective characteristic ('Parameter'), the respective reference data set ('Reanalysis'), the total number of heatwaves in the respective sample ('total_HW_num'), the number of heatwaves in the sample exceeding the reference value ('Parameter_Exceedance_Number'), and the exceedance percentage ('Parameter_Exceedance_Percentage'), the data subset type, the CMIP generation, and the repsective experiment. Data based on ERA5

Supplementary Data 449: Heatwave Characteristic Exceedange Percentages (Tx97.5p) - Table lists the exceedance percentages of the respective heatwave characteristic for CMIP6 multi-model ensemble (MME) SSP2-4.5 for entire dataset from 2020 to 2100. The Table shows the region name, dates of the reference event, name of the heatwave characteristic ('Parameter_Name'), the reference value of the respective characteristic ('Parameter'), the respective reference data set ('Reanalysis'), the total number of heatwaves in the respective sample ('total_HW_num'), the number of heatwaves in the sample exceeding the reference value ('Parameter_Exceedance_Number'), and the exceedance percentage ('Parameter_Exceedance_Percentage'), the data subset type, the CMIP generation, and the repsective experiment. Data based on ERA5

Supplementary Data 450: Heatwave Characteristic Exceedange Percentages (Tx99.0p) - Table lists the exceedance percentages of the respective heatwave characteristic for CMIP6 multi-model ensemble (MME) SSP2-4.5 for entire dataset from 2020 to 2100. The Table shows the region name, dates of the reference event, name of the heatwave characteristic ('Parameter_Name'), the reference value of the respective characteristic ('Parameter'), the respective reference data set ('Reanalysis'), the total number of heatwaves in the respective sample ('total_HW_num'), the number of heatwaves in the sample exceeding the reference value ('Parameter_Exceedance_Number'), and the exceedance percentage ('Parameter_Exceedance_Percentage'), the data subset type, the CMIP generation, and the repsective experiment. Data based on ERA5

Supplementary Data 451: Heatwave Characteristic Exceedange Percentages (Tx95.0p) - Table lists the exceedance percentages of the respective heatwave characteristic for CMIP6 multi-model ensemble (MME) SSP2-4.5 for entire dataset from 2020 to 2100. The Table shows the region name, dates of the reference event, name of the heatwave characteristic ('Parameter_Name'), the reference value of the respective characteristic ('Parameter'), the respective reference data set ('Reanalysis'), the total number of heatwaves in the respective sample ('total_HW_num'), the number of heatwaves in the sample exceeding the reference value ('Parameter_Exceedance_Number'), and the exceedance percentage ('Parameter_Exceedance_Percentage'), the data subset type, the CMIP generation, and the repsective experiment. Data based on ERA5

Supplementary Data 452: Heatwave Characteristic Exceedange Percentages (Tx97.5p) - Table lists the exceedance percentages of the respective heatwave characteristic for CMIP6 multi-model ensemble (MME) SSP2-4.5 for entire dataset from 2020 to 2100. The Table shows the region name, dates of the reference event, name of the heatwave characteristic ('Parameter_Name'), the reference value of the respective characteristic ('Parameter'), the respective reference data set ('Reanalysis'), the total number of heatwaves in the respective sample ('total_HW_num'), the number of heatwaves in the sample exceeding the reference value ('Parameter_Exceedance_Number'), and the exceedance percentage ('Parameter_Exceedance_Percentage'), the data subset type, the CMIP generation, and the repsective experiment. Data based on ERA5

Supplementary Data 453: Heatwave Characteristic Exceedange Percentages (Tx99.0p) - Table lists the exceedance percentages of the respective heatwave characteristic for CMIP6 multi-model ensemble (MME) SSP2-4.5 for entire dataset from 2020 to 2100. The Table shows the region name, dates of the reference event, name of the heatwave characteristic ('Parameter_Name'), the reference value of the respective characteristic ('Parameter'), the respective reference data set ('Reanalysis'), the total number of heatwaves in the respective sample ('total_HW_num'), the number of heatwaves in the sample exceeding the reference value ('Parameter_Exceedance_Number'), and the exceedance percentage ('Parameter_Exceedance_Percentage'), the data subset type, the CMIP generation, and the repsective experiment. Data based on ERA5

Supplementary Data 454: Heatwave Characteristic Exceedange Percentages (Tx95.0p) - Table lists the exceedance percentages of the respective heatwave characteristic for CMIP6 multi-model ensemble (MME) SSP2-4.5 for entire dataset from 2020 to 2100. The Table shows the region name, dates of the reference event, name of the heatwave characteristic ('Parameter_Name'), the reference value of the respective characteristic ('Parameter'), the respective reference data set ('Reanalysis'), the total number of heatwaves in the respective sample ('total_HW_num'), the number of heatwaves in the sample exceeding the reference value ('Parameter_Exceedance_Number'), and the exceedance percentage ('Parameter_Exceedance_Percentage'), the data subset type, the CMIP generation, and the repsective experiment. Data based on ERA5

Supplementary Data 455: Heatwave Characteristic Exceedange Percentages (Tx97.5p) - Table lists the exceedance percentages of the respective heatwave characteristic for CMIP6 multi-model ensemble (MME) SSP2-4.5 for entire dataset from 2020 to 2100. The Table shows the region name, dates of the reference event, name of the heatwave characteristic ('Parameter_Name'), the reference value of the respective characteristic ('Parameter'), the respective reference data set ('Reanalysis'), the total number of heatwaves in the respective sample ('total_HW_num'), the number of heatwaves in the sample exceeding the reference value ('Parameter_Exceedance_Number'), and the exceedance percentage ('Parameter_Exceedance_Percentage'), the data subset type, the CMIP generation, and the repsective experiment. Data based on ERA5

Supplementary Data 456: Heatwave Characteristic Exceedange Percentages (Tx99.0p) - Table lists the exceedance percentages of the respective heatwave characteristic for CMIP6 multi-model ensemble (MME) SSP2-4.5 for entire dataset from 2020 to 2100. The Table shows the region name, dates of the reference event, name of the heatwave characteristic ('Parameter_Name'), the reference value of the respective characteristic ('Parameter'), the respective reference data set ('Reanalysis'), the total number of heatwaves in the respective sample ('total_HW_num'), the number of heatwaves in the sample exceeding the reference value

('Parameter_Exceedance_Number'), and the exceedance percentage ('Parameter_Exceedance_Percentage'), the data subset type, the CMIP generation, and the repsective experiment. Data based on ERA5

Supplementary Data 457: Heatwave Characteristic Exceedange Percentages (Tx95.0p) - Table lists the exceedance percentages of the respective heatwave characteristic for CMIP6 multi-model ensemble (MME) SSP3-7.0 for entire dataset from 2020 to 2100. The Table shows the region name, dates of the reference event, name of the heatwave characteristic ('Parameter_Name'), the reference value of the respective characteristic ('Parameter'), the respective reference data set ('Reanalysis'), the total number of heatwaves in the respective sample ('total_HW_num'), the number of heatwaves in the sample exceeding the reference value ('Parameter_Exceedance_Number'), and the exceedance percentage ('Parameter_Exceedance_Percentage'), the data subset type, the CMIP generation, and the repsective experiment. Data based on ERA5

Supplementary Data 458: Heatwave Characteristic Exceedange Percentages (Tx97.5p) - Table lists the exceedance percentages of the respective heatwave characteristic for CMIP6 multi-model ensemble (MME) SSP3-7.0 for entire dataset from 2020 to 2100. The Table shows the region name, dates of the reference event, name of the heatwave characteristic ('Parameter_Name'), the reference value of the respective characteristic ('Parameter'), the respective reference data set ('Reanalysis'), the total number of heatwaves in the respective sample ('total_HW_num'), the number of heatwaves in the sample exceeding the reference value ('Parameter_Exceedance_Number'), and the exceedance percentage ('Parameter_Exceedance_Percentage'), the data subset type, the CMIP generation, and the repsective experiment. Data based on ERA5

Supplementary Data 459: Heatwave Characteristic Exceedange Percentages (Tx99.0p) - Table lists the exceedance percentages of the respective heatwave characteristic for CMIP6 multi-model ensemble (MME) SSP3-7.0 for entire dataset from 2020 to 2100. The Table shows the region name, dates of the reference event, name of the heatwave characteristic ('Parameter_Name'), the reference value of the respective characteristic ('Parameter'), the respective reference data set ('Reanalysis'), the total number of heatwaves in the respective sample ('total_HW_num'), the number of heatwaves in the sample exceeding the reference value ('Parameter_Exceedance_Number'), and the exceedance percentage ('Parameter_Exceedance_Percentage'), the data subset type, the CMIP generation, and the repsective experiment. Data based on ERA5

Supplementary Data 460: Heatwave Characteristic Exceedange Percentages (Tx95.0p) - Table lists the exceedance percentages of the respective heatwave characteristic for CMIP6 multi-model ensemble (MME) SSP3-7.0 for entire dataset from 2020 to 2100. The Table shows the region name, dates of the reference event, name of the heatwave characteristic ('Parameter_Name'), the reference value of the respective characteristic ('Parameter'), the respective reference data set ('Reanalysis'), the total number of heatwaves in the respective sample ('total_HW_num'), the number of heatwaves in the sample exceeding the reference value ('Parameter_Exceedance_Number'), and the exceedance percentage ('Parameter_Exceedance_Percentage'), the data subset type, the CMIP generation, and the repsective experiment. Data based on ERA5

Supplementary Data 461: Heatwave Characteristic Exceedange Percentages (Tx97.5p) - Table lists the exceedance percentages of the respective heatwave characteristic for CMIP6 multi-model ensemble (MME) SSP3-7.0 for entire dataset from 2020 to 2100. The Table shows the region name, dates of the reference event, name of the heatwave characteristic ('Parameter_Name'), the reference value of the respective characteristic

('Parameter'), the respective reference data set ('Reanalysis'), the total number of heatwaves in the respective sample ('total_HW_num'), the number of heatwaves in the sample exceeding the reference value ('Parameter_Exceedance_Number'), and the exceedance percentage ('Parameter_Exceedance_Percentage'), the data subset type, the CMIP generation, and the repsective experiment. Data based on ERA5

Supplementary Data 462: Heatwave Characteristic Exceedange Percentages (Tx99.0p) - Table lists the exceedance percentages of the respective heatwave characteristic for CMIP6 multi-model ensemble (MME) SSP3-7.0 for entire dataset from 2020 to 2100. The Table shows the region name, dates of the reference event, name of the heatwave characteristic ('Parameter_Name'), the reference value of the respective characteristic ('Parameter'), the respective reference data set ('Reanalysis'), the total number of heatwaves in the respective sample ('total_HW_num'), the number of heatwaves in the sample exceeding the reference value ('Parameter_Exceedance_Number'), and the exceedance percentage ('Parameter_Exceedance_Percentage'), the data subset type, the CMIP generation, and the repsective experiment. Data based on ERA5

Supplementary Data 463: Heatwave Characteristic Exceedange Percentages (Tx95.0p) - Table lists the exceedance percentages of the respective heatwave characteristic for CMIP6 multi-model ensemble (MME) SSP3-7.0 for entire dataset from 2020 to 2100. The Table shows the region name, dates of the reference event, name of the heatwave characteristic ('Parameter_Name'), the reference value of the respective characteristic ('Parameter'), the respective reference data set ('Reanalysis'), the total number of heatwaves in the respective sample ('total_HW_num'), the number of heatwaves in the sample exceeding the reference value ('Parameter_Exceedance_Number'), and the exceedance percentage ('Parameter_Exceedance_Percentage'), the data subset type, the CMIP generation, and the repsective experiment. Data based on ERA5

Supplementary Data 464: Heatwave Characteristic Exceedange Percentages (Tx97.5p) - Table lists the exceedance percentages of the respective heatwave characteristic for CMIP6 multi-model ensemble (MME) SSP3-7.0 for entire dataset from 2020 to 2100. The Table shows the region name, dates of the reference event, name of the heatwave characteristic ('Parameter_Name'), the reference value of the respective characteristic ('Parameter'), the respective reference data set ('Reanalysis'), the total number of heatwaves in the respective sample ('total_HW_num'), the number of heatwaves in the sample exceeding the reference value ('Parameter_Exceedance_Number'), and the exceedance percentage ('Parameter_Exceedance_Percentage'), the data subset type, the CMIP generation, and the repsective experiment. Data based on ERA5

Supplementary Data 465: Heatwave Characteristic Exceedange Percentages (Tx99.0p) - Table lists the exceedance percentages of the respective heatwave characteristic for CMIP6 multi-model ensemble (MME) SSP3-7.0 for entire dataset from 2020 to 2100. The Table shows the region name, dates of the reference event, name of the heatwave characteristic ('Parameter_Name'), the reference value of the respective characteristic ('Parameter'), the respective reference data set ('Reanalysis'), the total number of heatwaves in the respective sample ('total_HW_num'), the number of heatwaves in the sample exceeding the reference value ('Parameter_Exceedance_Number'), and the exceedance percentage ('Parameter_Exceedance_Percentage'), the data subset type, the CMIP generation, and the repsective experiment. Data based on ERA5

Supplementary Data 466: Heatwave Characteristic Exceedange Percentages (Tx95.0p) - Table lists the exceedance percentages of the respective heatwave characteristic for CMIP6 multi-model ensemble (MME)

SSP3-7.0 for entire dataset from 2020 to 2100. The Table shows the region name, dates of the reference event, name of the heatwave characteristic ('Parameter_Name'), the reference value of the respective characteristic ('Parameter'), the respective reference data set ('Reanalysis'), the total number of heatwaves in the respective sample ('total_HW_num'), the number of heatwaves in the sample exceeding the reference value ('Parameter_Exceedance_Number'), and the exceedance percentage ('Parameter_Exceedance_Percentage'), the data subset type, the CMIP generation, and the repsective experiment. Data based on ERA5

Supplementary Data 467: Heatwave Characteristic Exceedange Percentages (Tx97.5p) - Table lists the exceedance percentages of the respective heatwave characteristic for CMIP6 multi-model ensemble (MME) SSP3-7.0 for entire dataset from 2020 to 2100. The Table shows the region name, dates of the reference event, name of the heatwave characteristic ('Parameter_Name'), the reference value of the respective characteristic ('Parameter'), the respective reference data set ('Reanalysis'), the total number of heatwaves in the respective sample ('total_HW_num'), the number of heatwaves in the sample exceeding the reference value ('Parameter_Exceedance_Number'), and the exceedance percentage ('Parameter_Exceedance_Percentage'), the data subset type, the CMIP generation, and the repsective experiment. Data based on ERA5

Supplementary Data 468: Heatwave Characteristic Exceedange Percentages (Tx99.0p) - Table lists the exceedance percentages of the respective heatwave characteristic for CMIP6 multi-model ensemble (MME) SSP3-7.0 for entire dataset from 2020 to 2100. The Table shows the region name, dates of the reference event, name of the heatwave characteristic ('Parameter_Name'), the reference value of the respective characteristic ('Parameter'), the respective reference data set ('Reanalysis'), the total number of heatwaves in the respective sample ('total_HW_num'), the number of heatwaves in the sample exceeding the reference value ('Parameter_Exceedance_Number'), and the exceedance percentage ('Parameter_Exceedance_Percentage'), the data subset type, the CMIP generation, and the repsective experiment. Data based on ERA5

Supplementary Data 469: Heatwave Characteristic Exceedange Percentages (Tx95.0p) - Table lists the exceedance percentages of the respective heatwave characteristic for CMIP6 multi-model ensemble (MME) SSP3-7.0 for entire dataset from 2020 to 2100. The Table shows the region name, dates of the reference event, name of the heatwave characteristic ('Parameter_Name'), the reference value of the respective characteristic ('Parameter'), the respective reference data set ('Reanalysis'), the total number of heatwaves in the respective sample ('total_HW_num'), the number of heatwaves in the sample exceeding the reference value ('Parameter_Exceedance_Number'), and the exceedance percentage ('Parameter_Exceedance_Percentage'), the data subset type, the CMIP generation, and the repsective experiment. Data based on ERA5

Supplementary Data 470: Heatwave Characteristic Exceedange Percentages (Tx97.5p) - Table lists the exceedance percentages of the respective heatwave characteristic for CMIP6 multi-model ensemble (MME) SSP3-7.0 for entire dataset from 2020 to 2100. The Table shows the region name, dates of the reference event, name of the heatwave characteristic ('Parameter_Name'), the reference value of the respective characteristic ('Parameter'), the respective reference data set ('Reanalysis'), the total number of heatwaves in the respective sample ('total_HW_num'), the number of heatwaves in the sample exceeding the reference value ('Parameter_Exceedance_Number'), and the exceedance percentage ('Parameter_Exceedance_Percentage'), the data subset type, the CMIP generation, and the repsective experiment. Data based on ERA5

Supplementary Data 471: Heatwave Characteristic Exceedange Percentages (Tx99.0p) - Table lists the exceedance percentages of the respective heatwave characteristic for CMIP6 multi-model ensemble (MME) SSP3-7.0 for entire dataset from 2020 to 2100. The Table shows the region name, dates of the reference event, name of the heatwave characteristic ('Parameter_Name'), the reference value of the respective characteristic ('Parameter'), the respective reference data set ('Reanalysis'), the total number of heatwaves in the respective sample ('total_HW_num'), the number of heatwaves in the sample exceeding the reference value ('Parameter_Exceedance_Number'), and the exceedance percentage ('Parameter_Exceedance_Percentage'), the data subset type, the CMIP generation, and the repsective experiment. Data based on ERA5

Supplementary Data 472: Heatwave Characteristic Exceedange Percentages (Tx95.0p) - Table lists the exceedance percentages of the respective heatwave characteristic for CMIP6 multi-model ensemble (MME) SSP3-7.0 for entire dataset from 2020 to 2100. The Table shows the region name, dates of the reference event, name of the heatwave characteristic ('Parameter_Name'), the reference value of the respective characteristic ('Parameter'), the respective reference data set ('Reanalysis'), the total number of heatwaves in the respective sample ('total_HW_num'), the number of heatwaves in the sample exceeding the reference value ('Parameter_Exceedance_Number'), and the exceedance percentage ('Parameter_Exceedance_Percentage'), the data subset type, the CMIP generation, and the repsective experiment. Data based on ERA5

Supplementary Data 473: Heatwave Characteristic Exceedange Percentages (Tx97.5p) - Table lists the exceedance percentages of the respective heatwave characteristic for CMIP6 multi-model ensemble (MME) SSP3-7.0 for entire dataset from 2020 to 2100. The Table shows the region name, dates of the reference event, name of the heatwave characteristic ('Parameter_Name'), the reference value of the respective characteristic ('Parameter'), the respective reference data set ('Reanalysis'), the total number of heatwaves in the respective sample ('total_HW_num'), the number of heatwaves in the sample exceeding the reference value ('Parameter_Exceedance_Number'), and the exceedance percentage ('Parameter_Exceedance_Percentage'), the data subset type, the CMIP generation, and the repsective experiment. Data based on ERA5

Supplementary Data 474: Heatwave Characteristic Exceedange Percentages (Tx99.0p) - Table lists the exceedance percentages of the respective heatwave characteristic for CMIP6 multi-model ensemble (MME) SSP3-7.0 for entire dataset from 2020 to 2100. The Table shows the region name, dates of the reference event, name of the heatwave characteristic ('Parameter_Name'), the reference value of the respective characteristic ('Parameter'), the respective reference data set ('Reanalysis'), the total number of heatwaves in the respective sample ('total_HW_num'), the number of heatwaves in the sample exceeding the reference value ('Parameter_Exceedance_Number'), and the exceedance percentage ('Parameter_Exceedance_Percentage'), the data subset type, the CMIP generation, and the repsective experiment. Data based on ERA5

Supplementary Data 475: Heatwave Characteristic Exceedange Percentages (Tx95.0p) - Table lists the exceedance percentages of the respective heatwave characteristic for CMIP6 multi-model ensemble (MME) SSP5-8.5 for entire dataset from 2020 to 2100. The Table shows the region name, dates of the reference event, name of the heatwave characteristic ('Parameter_Name'), the reference value of the respective characteristic ('Parameter'), the respective reference data set ('Reanalysis'), the total number of heatwaves in the respective sample ('total_HW_num'), the number of heatwaves in the sample exceeding the reference value ('Parameter_Exceedance_Number'), and the exceedance percentage ('Parameter_Exceedance_Percentage'), the data subset type, the CMIP generation, and the repsective experiment. Data based on ERA5

Supplementary Data 476: Heatwave Characteristic Exceedange Percentages (Tx97.5p) - Table lists the exceedance percentages of the respective heatwave characteristic for CMIP6 multi-model ensemble (MME) SSP5-8.5 for entire dataset from 2020 to 2100. The Table shows the region name, dates of the reference event, name of the heatwave characteristic ('Parameter_Name'), the reference value of the respective characteristic ('Parameter'), the respective reference data set ('Reanalysis'), the total number of heatwaves in the respective sample ('total_HW_num'), the number of heatwaves in the sample exceeding the reference value ('Parameter_Exceedance_Number'), and the exceedance percentage ('Parameter_Exceedance_Percentage'), the data subset type, the CMIP generation, and the repsective experiment. Data based on ERA5

Supplementary Data 477: Heatwave Characteristic Exceedange Percentages (Tx99.0p) - Table lists the exceedance percentages of the respective heatwave characteristic for CMIP6 multi-model ensemble (MME) SSP5-8.5 for entire dataset from 2020 to 2100. The Table shows the region name, dates of the reference event, name of the heatwave characteristic ('Parameter_Name'), the reference value of the respective characteristic ('Parameter'), the respective reference data set ('Reanalysis'), the total number of heatwaves in the respective sample ('total_HW_num'), the number of heatwaves in the sample exceeding the reference value ('Parameter_Exceedance_Number'), and the exceedance percentage ('Parameter_Exceedance_Percentage'), the data subset type, the CMIP generation, and the repsective experiment. Data based on ERA5

Supplementary Data 478: Heatwave Characteristic Exceedange Percentages (Tx95.0p) - Table lists the exceedance percentages of the respective heatwave characteristic for CMIP6 multi-model ensemble (MME) SSP5-8.5 for entire dataset from 2020 to 2100. The Table shows the region name, dates of the reference event, name of the heatwave characteristic ('Parameter_Name'), the reference value of the respective characteristic ('Parameter'), the respective reference data set ('Reanalysis'), the total number of heatwaves in the respective sample ('total_HW_num'), the number of heatwaves in the sample exceeding the reference value ('Parameter_Exceedance_Number'), and the exceedance percentage ('Parameter_Exceedance_Percentage'), the data subset type, the CMIP generation, and the repsective experiment. Data based on ERA5

Supplementary Data 479: Heatwave Characteristic Exceedange Percentages (Tx97.5p) - Table lists the exceedance percentages of the respective heatwave characteristic for CMIP6 multi-model ensemble (MME) SSP5-8.5 for entire dataset from 2020 to 2100. The Table shows the region name, dates of the reference event, name of the heatwave characteristic ('Parameter_Name'), the reference value of the respective characteristic ('Parameter'), the respective reference data set ('Reanalysis'), the total number of heatwaves in the respective sample ('total_HW_num'), the number of heatwaves in the sample exceeding the reference value ('Parameter_Exceedance_Number'), and the exceedance percentage ('Parameter_Exceedance_Percentage'), the data subset type, the CMIP generation, and the repsective experiment. Data based on ERA5

Supplementary Data 480: Heatwave Characteristic Exceedange Percentages (Tx99.0p) - Table lists the exceedance percentages of the respective heatwave characteristic for CMIP6 multi-model ensemble (MME) SSP5-8.5 for entire dataset from 2020 to 2100. The Table shows the region name, dates of the reference event, name of the heatwave characteristic ('Parameter_Name'), the reference value of the respective characteristic ('Parameter'), the respective reference data set ('Reanalysis'), the total number of heatwaves in the respective sample ('total_HW_num'), the number of heatwaves in the sample exceeding the reference value

('Parameter_Exceedance_Number'), and the exceedance percentage ('Parameter_Exceedance_Percentage'), the data subset type, the CMIP generation, and the repsective experiment. Data based on ERA5

Supplementary Data 481: Heatwave Characteristic Exceedange Percentages (Tx95.0p) - Table lists the exceedance percentages of the respective heatwave characteristic for CMIP6 multi-model ensemble (MME) SSP5-8.5 for entire dataset from 2020 to 2100. The Table shows the region name, dates of the reference event, name of the heatwave characteristic ('Parameter_Name'), the reference value of the respective characteristic ('Parameter'), the respective reference data set ('Reanalysis'), the total number of heatwaves in the respective sample ('total_HW_num'), the number of heatwaves in the sample exceeding the reference value ('Parameter_Exceedance_Number'), and the exceedance percentage ('Parameter_Exceedance_Percentage'), the data subset type, the CMIP generation, and the repsective experiment. Data based on ERA5

Supplementary Data 482: Heatwave Characteristic Exceedange Percentages (Tx97.5p) - Table lists the exceedance percentages of the respective heatwave characteristic for CMIP6 multi-model ensemble (MME) SSP5-8.5 for entire dataset from 2020 to 2100. The Table shows the region name, dates of the reference event, name of the heatwave characteristic ('Parameter_Name'), the reference value of the respective characteristic ('Parameter'), the respective reference data set ('Reanalysis'), the total number of heatwaves in the respective sample ('total_HW_num'), the number of heatwaves in the sample exceeding the reference value ('Parameter_Exceedance_Number'), and the exceedance percentage ('Parameter_Exceedance_Percentage'), the data subset type, the CMIP generation, and the repsective experiment. Data based on ERA5

Supplementary Data 483: Heatwave Characteristic Exceedange Percentages (Tx99.0p) - Table lists the exceedance percentages of the respective heatwave characteristic for CMIP6 multi-model ensemble (MME) SSP5-8.5 for entire dataset from 2020 to 2100. The Table shows the region name, dates of the reference event, name of the heatwave characteristic ('Parameter_Name'), the reference value of the respective characteristic ('Parameter'), the respective reference data set ('Reanalysis'), the total number of heatwaves in the respective sample ('total_HW_num'), the number of heatwaves in the sample exceeding the reference value ('Parameter_Exceedance_Number'), and the exceedance percentage ('Parameter_Exceedance_Percentage'), the data subset type, the CMIP generation, and the repsective experiment. Data based on ERA5

Supplementary Data 484: Heatwave Characteristic Exceedange Percentages (Tx95.0p) - Table lists the exceedance percentages of the respective heatwave characteristic for CMIP6 multi-model ensemble (MME) SSP5-8.5 for entire dataset from 2020 to 2100. The Table shows the region name, dates of the reference event, name of the heatwave characteristic ('Parameter_Name'), the reference value of the respective characteristic ('Parameter'), the respective reference data set ('Reanalysis'), the total number of heatwaves in the respective sample ('total_HW_num'), the number of heatwaves in the sample exceeding the reference value ('Parameter_Exceedance_Number'), and the exceedance percentage ('Parameter_Exceedance_Percentage'), the data subset type, the CMIP generation, and the repsective experiment. Data based on ERA5

Supplementary Data 485: Heatwave Characteristic Exceedange Percentages (Tx97.5p) - Table lists the exceedance percentages of the respective heatwave characteristic for CMIP6 multi-model ensemble (MME) SSP5-8.5 for entire dataset from 2020 to 2100. The Table shows the region name, dates of the reference event, name of the heatwave characteristic ('Parameter_Name'), the reference value of the respective characteristic

('Parameter'), the respective reference data set ('Reanalysis'), the total number of heatwaves in the respective sample ('total_HW_num'), the number of heatwaves in the sample exceeding the reference value ('Parameter_Exceedance_Number'), and the exceedance percentage ('Parameter_Exceedance_Percentage'), the data subset type, the CMIP generation, and the repsective experiment. Data based on ERA5

Supplementary Data 486: Heatwave Characteristic Exceedange Percentages (Tx99.0p) - Table lists the exceedance percentages of the respective heatwave characteristic for CMIP6 multi-model ensemble (MME) SSP5-8.5 for entire dataset from 2020 to 2100. The Table shows the region name, dates of the reference event, name of the heatwave characteristic ('Parameter_Name'), the reference value of the respective characteristic ('Parameter'), the respective reference data set ('Reanalysis'), the total number of heatwaves in the respective sample ('total_HW_num'), the number of heatwaves in the sample exceeding the reference value ('Parameter_Exceedance_Number'), and the exceedance percentage ('Parameter_Exceedance_Percentage'), the data subset type, the CMIP generation, and the repsective experiment. Data based on ERA5

Supplementary Data 487: Heatwave Characteristic Exceedange Percentages (Tx95.0p) - Table lists the exceedance percentages of the respective heatwave characteristic for CMIP6 multi-model ensemble (MME) SSP5-8.5 for entire dataset from 2020 to 2100. The Table shows the region name, dates of the reference event, name of the heatwave characteristic ('Parameter_Name'), the reference value of the respective characteristic ('Parameter'), the respective reference data set ('Reanalysis'), the total number of heatwaves in the respective sample ('total_HW_num'), the number of heatwaves in the sample exceeding the reference value ('Parameter_Exceedance_Number'), and the exceedance percentage ('Parameter_Exceedance_Percentage'), the data subset type, the CMIP generation, and the repsective experiment. Data based on ERA5

Supplementary Data 488: Heatwave Characteristic Exceedange Percentages (Tx97.5p) - Table lists the exceedance percentages of the respective heatwave characteristic for CMIP6 multi-model ensemble (MME) SSP5-8.5 for entire dataset from 2020 to 2100. The Table shows the region name, dates of the reference event, name of the heatwave characteristic ('Parameter_Name'), the reference value of the respective characteristic ('Parameter'), the respective reference data set ('Reanalysis'), the total number of heatwaves in the respective sample ('total_HW_num'), the number of heatwaves in the sample exceeding the reference value ('Parameter_Exceedance_Number'), and the exceedance percentage ('Parameter_Exceedance_Percentage'), the data subset type, the CMIP generation, and the repsective experiment. Data based on ERA5

Supplementary Data 489: Heatwave Characteristic Exceedange Percentages (Tx99.0p) - Table lists the exceedance percentages of the respective heatwave characteristic for CMIP6 multi-model ensemble (MME) SSP5-8.5 for entire dataset from 2020 to 2100. The Table shows the region name, dates of the reference event, name of the heatwave characteristic ('Parameter_Name'), the reference value of the respective characteristic ('Parameter'), the respective reference data set ('Reanalysis'), the total number of heatwaves in the respective sample ('total_HW_num'), the number of heatwaves in the sample exceeding the reference value ('Parameter_Exceedance_Number'), and the exceedance percentage ('Parameter_Exceedance_Percentage'), the data subset type, the CMIP generation, and the repsective experiment. Data based on ERA5

Supplementary Data 490: Heatwave Characteristic Exceedange Percentages (Tx95.0p) - Table lists the exceedance percentages of the respective heatwave characteristic for CMIP6 multi-model ensemble (MME)

SSP5-8.5 for entire dataset from 2020 to 2100. The Table shows the region name, dates of the reference event, name of the heatwave characteristic ('Parameter_Name'), the reference value of the respective characteristic ('Parameter'), the respective reference data set ('Reanalysis'), the total number of heatwaves in the respective sample ('total_HW_num'), the number of heatwaves in the sample exceeding the reference value ('Parameter_Exceedance_Number'), and the exceedance percentage ('Parameter_Exceedance_Percentage'), the data subset type, the CMIP generation, and the repsective experiment. Data based on ERA5

Supplementary Data 491: Heatwave Characteristic Exceedange Percentages (Tx97.5p) - Table lists the exceedance percentages of the respective heatwave characteristic for CMIP6 multi-model ensemble (MME) SSP5-8.5 for entire dataset from 2020 to 2100. The Table shows the region name, dates of the reference event, name of the heatwave characteristic ('Parameter_Name'), the reference value of the respective characteristic ('Parameter'), the respective reference data set ('Reanalysis'), the total number of heatwaves in the respective sample ('total_HW_num'), the number of heatwaves in the sample exceeding the reference value ('Parameter_Exceedance_Number'), and the exceedance percentage ('Parameter_Exceedance_Percentage'), the data subset type, the CMIP generation, and the repsective experiment. Data based on ERA5

Supplementary Data 492: Heatwave Characteristic Exceedange Percentages (Tx99.0p) - Table lists the exceedance percentages of the respective heatwave characteristic for CMIP6 multi-model ensemble (MME) SSP5-8.5 for entire dataset from 2020 to 2100. The Table shows the region name, dates of the reference event, name of the heatwave characteristic ('Parameter_Name'), the reference value of the respective characteristic ('Parameter'), the respective reference data set ('Reanalysis'), the total number of heatwaves in the respective sample ('total_HW_num'), the number of heatwaves in the sample exceeding the reference value ('Parameter_Exceedance_Number'), and the exceedance percentage ('Parameter_Exceedance_Percentage'), the data subset type, the CMIP generation, and the repsective experiment. Data based on ERA5
