# Supplementary material for: Recent extreme heatwaves are no match for future events
Source: Nat Commun. 2026 Nov 5;17:11456. doi: 10.1038/s41467-026-76809-5 (PMC13740290; doi:10.1038/s41467-026-76809-5)
Supplement: Supplementary file 3 [file 41467_2026_76809_MOESM3_ESM.pdf]

nature portfolio

Peer Review File

Recent extreme heatwaves are no match for future events

Corresponding Author: Mr Philipp Aglas-Leitner

This file contains all reviewer reports in order by version, followed by all author rebuttals in order by version.

Attachments originally included by the reviewers as part of their assessment can be found at the end of this file.

Version 0:

Reviewer comments:

Reviewer #1

(Remarks to the Author)
Understanding how heat waves will evolve under future climate scenarios is crucial for anticipating impacts on human health, ecosystems, and socio-economic systems. Over the past decades, the frequency, intensity, and duration of heat waves have increased in many regions, consistent with observed global warming trends. The manuscript under review contributes to this effort by comparing observed heat wave characteristics with those projected by climate models for future periods. While the study presents an interesting and relevant framework, I have several questions regarding the methods, as well as a few suggestions that may help to strengthen the analysis and enhance the clarity of the manuscript.

Major comments:
I suggest further contextualizing the heat waves selected as reference events by comparing them with other heat waves observed during the historical period using ERA5 and Berkeley Earth datasets. This could be achieved by presenting graphs similar to those shown in Figure 1, including the different attributes of the heat waves. This comparison would help place the selected heat waves in a broader climatological context and facilitate a clearer interpretation of the results when comparing them with future projections.

Page 8, lines 124-146 and lines 254-255: The paragraph is potentially misleading because it compares growth factors without clearly acknowledging that the European and Pacific Northwest reference heatwaves already cover a relatively modest fraction of land area, whereas the Australian, Chinese, and Southern African reference events are much more spatially extensive. The interpretation of the projected changes in spatial extent would benefit from a clearer discussion of the baseline size of the reference events in each region. In Europe and the Pacific Northwest, the reference heatwaves cover around 30% of the regional land area, so there is substantial room for future events to become larger. In contrast, the reference events in Australia, China, and southern Africa already exceed about 60% of the respective regional land areas, which inherently limits the possible relative growth in spatial extent.

Figure 3: I am somewhat surprised that the heat waves in India and Pakistan, as well as those in the Pacific Northwest, Canada, and the USA, do not appear as particularly severe in the current climate, given that several studies report record-breaking temperatures associated with these events (e.g., White et al., 2023). Consistently, Figure 5 shows very short return periods for these events, whereas observational analyses suggest return periods exceeding 100 years for the Western Canada heat wave (e.g., Malinina and Gillett, 2024). Taken together, these results raise the question of whether the models adequately represent key heat-wave characteristics (e.g., intensity, duration, and spatial extent) in the present-day climate. Could the authors clarify whether such an evaluation was performed?

White, R.H., Anderson, S., Booth, J.F. et al. The unprecedented Pacific Northwest heatwave of June 2021. Nat Commun 14, 727 (2023). https://doi.org/10.1038/s41467-023-36289-3
Malinina, E., and Gillet, N. (2024). The 2021 heatwave was less rare in Western Canada than previously thought. Weather and Climate Extremes, 43, Article 100642. https://doi.org/10.1016/j.wace.2024.100642

Lines 488-491: The authors apply a targeted bias correction by adjusting the mean temperature bias above the 80th percentile between the model and reference data, which simplifies the correction relative to full quantile mapping approaches. While this may be appropriate if the bias is approximately constant in the warm tail, it would strengthen the manuscript to justify this choice further—e.g., by showing how the bias varies across higher quantiles (P90, P95) or

comparing the corrected tail distribution to observations. Additionally, a brief comparison with standard quantile mapping results (even for a test case) could demonstrate that the simpler method sufficiently captures the relevant features for heatwave projections.

Lines 556-560: Since the grid resolution varies across models, could the authors clarify how this might affect the estimated heat-wave areas? For example, could differences in grid size lead to apparent variations in spatial extent that are due to resolution rather than actual climatic differences? Would a sensitivity test using a common regridded resolution help assess the robustness of the results?

Lines 591-594: The empirical return period estimation by counting events exceeding reference values is simple and transparent, but for rare heatwaves it may lack precision due to limited historical occurrences. Have the authors considered fitting an appropriate statistical distribution (e.g., GEV) to the data to estimate return periods? Fitting a distribution could provide more robust estimates, especially for rare or extreme events, and would allow for interpolation or extrapolation beyond the observed sample.

Supplementary Information: There are too many figures and tables, which also do not have captions. Is there any way to summarize the information?

Minor comments:
Lines 457-458: What complications can arise from using a predefined region?

(Remarks on code availability)
The provided code allows for the identification of heat waves and the calculation of their severity, and it is generally well commented. However, I did not find code related to the bias-correction procedure or the estimation of return periods.

Reviewer #2

(Remarks to the Author)
This manuscript investigates future heatwave projections using climate model simulations under multiple emissions scenarios. Its main contribution is the event-based approach that embeds observed high-impact heatwaves into future projections, enabling a direct comparison between past extremes and future conditions. The analysis considers multiple heatwave metrics and combines multi-model ensembles with large initial-condition ensembles, providing a comprehensive assessment of uncertainties. Although the general conclusion that future heatwaves will intensify under continued warming is not unexpected, the results offer useful quantitative evidence that is highly relevant for heatwave mitigation and adaptation. The manuscript is clearly written and methodologically sound. I recommend minor revision, mainly to improve clarity and presentation.

1. Lines 97–122. Please clarify how the proportions reported in this section (e.g., the 12–22% values in Line 97) are calculated. In addition, four heatwave metrics are considered in the study. Are these proportions derived from heatwave severity only, or are similar increases found consistently across the other metrics as well?

2. Line 170. This statement is unclear in its current form. Please consider rephrasing it for clarity or removing it if it is not essential to the main argument.

3. Overall, the manuscript contains too many long and complex sentences. Revising these for conciseness would improve readability and accessibility. For example, Lines 178–183 are hard to follow, and the sentence beginning with “The in …” in Line 191 appears incomplete or unclear. Shortening or restructuring these sentences would substantially improve clarity.

4. Line 206. The discussion shifts from SSP emission scenarios to Global Warming Levels (GWLs) somewhat abruptly. A brief transitional explanation would help clarify the relationship between the SSP-based analysis and the GWL framework, including how the GWLs are defined and applied in the analysis.

5. Lines 214–215. Please specify which Global Warming Level is being referred to here.

6. Regional definition. The term “Pacific Northwestern USA” is used, but it is unclear whether all regions analyzed in the study are explicitly defined or outlined in a map. If not, please clarify the regional definitions and indicate them consistently.

(Remarks on code availability)

Reviewer #3

(Remarks to the Author)
Reviewer Comments

I have read this manuscript very carefully and thoroughly. Given the scope of the study, the very large number of

supplementary figures (>180) and tables (>30), it is evident that the authors have invested substantial effort and care into this work. Overall, I highly appreciate the ambition, originality, and general conceptual framework proposed in this paper.

General assessment and strengths

The overarching research logic proposed by the authors is novel, well motivated, and compelling. By placing several major, recently observed global heatwave events into a future-oriented statistical framework, the study provides an intuitive and powerful way to contextualize the integrated intensity of recent extreme heat events relative to what climate models project for the coming decades. This framing substantially improves interpretability and helps readers recognize that some of the most socially and economically impactful heatwaves observed in recent years may, from a future-climate perspective, no longer represent the upper tail of extremes. Instead, the results suggest that even more frequent and more severe events should be expected in the coming decades.

This conceptual approach is innovative and valuable, and I fully agree that such a framework can significantly advance our understanding of major heatwave hazards under climate change. Given that this overall logic is sound, my comments below focus primarily on technical and methodological aspects, particularly those related to heatwave definition and event identification.

Scope of expertise and perspective

Before detailing specific concerns, I would like to acknowledge the limitations of my own expertise. I am a researcher from China's national meteorological research system, and my professional experience over the past decade has focused primarily on observed weather hazards in China and their impacts on socio-economic systems. My understanding of heatwave events in other regions is largely informed by the scientific literature and media reports. Consequently, the concerns raised below are especially grounded in the Chinese observational and operational experience, though I believe they have broader relevance.

Conceptual distinction: extreme events vs. weather hazards vs. meteorological disasters

A key issue concerns the conceptual distinction between "extreme events", "weather hazards", and "meteorological disasters", which are often used interchangeably but are fundamentally different.

• Extreme events are defined statistically, referring to events with low occurrence probability in one or more relevant dimensions, such as severity, timing, spatial extent, or other characteristics.

• Weather (natural) hazards are events that produce direct or indirect adverse impacts on socio-economic systems, at least within one sector (for example, extreme heat restricting outdoor labor and reducing productivity, or increasing electricity demand and leading to grid overload and higher household energy expenditures).

• Meteorological disasters represent cases where severe weather causes direct socio-economic losses, such as loss of life, damage to housing, or destruction of public infrastructure, emphasizing direct losses.

From this perspective, the 22 regional heatwave events selected in this study clearly fall into the category of weather hazards, and many of them can reasonably be classified as disaster-level events, given their documented societal and economic impacts.

However, the heatwave definition adopted in the manuscript is purely statistical, based on the calendar-day 90th percentile of daily maximum temperature during the warmest six months over the 1961–1990 baseline. While this is a classical and well-established approach for identifying statistical extremes, it does not necessarily correspond to the threshold at which heat events begin to generate meaningful socio-economic impacts.

Concern 1: Heatwave threshold may be too low from a hazard perspective

From a Chinese operational and observational standpoint, the chosen threshold appears too low to represent impactful heatwave hazards.

Using the authors' definition implies that, on average, each grid point experiences approximately 18 heatwave days per year during the 1961–1990 period. This is difficult to reconcile with empirical experience. For comparison, within China's national meteorological hazard monitoring and warning system:

• The lowest-level heat warning is issued only when daily maximum temperature reaches 35 °C for at least three consecutive days.

• The highest-level heat warning requires daily maximum temperature to exceed 40 °C.

In practice, heatwave events that cause substantial socio-economic impacts in China almost always exceed 35 °C, and typically exceed 40 °C.

To illustrate this point quantitatively, I analyzed historical observations from Beijing and Shanghai, China's two largest metropolitan areas, using the authors' exact methodology (15-day moving window percentile estimation followed by 31-day smoothing) over the warmest six months (April–September) during 1961–1990 (see attached figures):

• Beijing:

o Calendar-day 90th-percentile thresholds range from approximately 22 °C to 35 °C (Fig. 1a).

o Only 9 calendar days have 90th-percentile thresholds exceeding 35 °C.

o The mean 90th-percentile threshold is approximately 31.2 °C.

o Corresponding calendar-day 99th-percentile thresholds are shown for reference (Fig. 1b).

• Shanghai:

o Calendar-day 90th-percentile thresholds exceed 35 °C on only 16 calendar days (Fig. 2a).

o The mean 90th-percentile threshold is approximately 30.2 °C.

o Corresponding calendar-day 99th-percentile thresholds are shown for reference (Fig. 2b).

Under this definition, many identified "heatwave days" would not produce noticeable impacts on socio-economic systems, leading to an over-identification of heatwave events. This likely explains why the manuscript reports an unusually large number of heatwave events, including cases outside China where heatwave durations exceed 180 days, which I found conceptually puzzling and which motivated my closer examination of the methodology.

Concern 2: Mismatch between statistical extremes and impactful events
In summary, the manuscript applies a statistical extreme-event framework (calendar-day 90th percentile relative to 1961–1990) to characterize events that are, by construction, major weather hazards with documented socio-economic impacts. This creates a clear conceptual mismatch:
• The statistical framework assumes frequent "extreme" heatwave days in the historical baseline; in practice, this algorithm identifies a large number of statistically defined "heatwave" events that cannot reasonably be considered hazards, for example cases where daily maximum temperature may not even reach 30 °C, but is flagged solely because it occurs at an unusual time of year.
• The events being analyzed and contextualized are rare, high-impact hazards that caused widespread socio-economic impacts.
From the Chinese perspective, truly impactful heatwave hazards during 1961–1990 were relatively rare. The assumption of ~18 heatwave days per year therefore conflicts with both operational practice and lived experience.
I therefore strongly encourage the authors to reconsider the heatwave threshold definition, particularly if the objective is to assess and contextualize hazard-level heatwave events rather than purely statistical extremes.

Concern 3: Choice of reference dataset for spatial extent
Regarding observational datasets, the manuscript relies primarily on Berkeley Earth (1° resolution), with ERA5 used as a secondary reference. While Berkeley Earth has advantages in handling sparse and discontinuous observations, I am more inclined to trust ERA5 for defining the spatial extent and geographical boundaries of heatwave events, due to its substantially higher spatial resolution.
At a 1° grid resolution, it is difficult to accurately capture true heatwave boundaries, especially in regions with strong topographic gradients. For example, among the 22 global events, two involve China. Event "g)" in Table S1 spans approximately 89.5°E–122.5°E and 20.5°N–40.5°N, covering both eastern lowland China and the Tibetan Plateau. Treating high-elevation regions (>3000 m) and eastern plains as a single homogeneous heatwave-affected domain is problematic. In practice, heatwaves capable of causing major socio-economic impacts are extremely unlikely over the Tibetan Plateau, even if statistically extreme temperatures occur there.
I therefore recommend using ERA5 as the primary dataset for defining spatial extent, with Berkeley Earth retained as a complementary reference.

Concern 4: Event splitting and alternative identification criteria
The authors note that, depending on the dataset, some events may be split when regional-mean temperature briefly falls below the threshold. While this behavior is expected under a strict spatial-mean definition, it may reduce robustness.
As an alternative (offered here as a reference rather than a prescription), China's national weather service identifies regional heatwave events using area-based criteria, for example:
1. At least 20% of the region must experience heatwave conditions on the event initiation day;
2. Persistence for at least two consecutive days;
3. During at least one day of the event, ≥50% of the region must be affected.
Under this framework, spatial continuity is not required, and short-lived dips in regional-mean temperature do not necessarily split a physically coherent heatwave event. Such criteria may yield more robust event identification, particularly when comparing across datasets.

Final remarks
Despite these concerns, I would like to reiterate that the conceptual contribution of this study is highly valuable. By characterizing unprecedented recent heatwave events in terms of heatwave severity, cumulative heat, duration, and spatial extent, and integrating these dimensions into an overall measure of integrated intensity, the authors move well beyond traditional attribution approaches that rely solely on the single most extreme temperature within an event.
This multidimensional framework has strong potential to advance how the community understands major meteorological hazards under climate change. The extensive supplementary material further reflects the authors' rigorous and systematic approach, which deserves strong recognition.
I hope the authors find these comments constructive and useful. I would be happy to discuss any of these points further if clarification is helpful, and I wish the authors every success in revising this impressive study.

(Remarks on code availability)
The authors provide extensive supplementary figures and tables (over 180 figures and more than 30 tables) that document the statistical analyses in detail and help readers follow the analysis workflow. No source code is provided, and reproducibility therefore relies on the description in the manuscript and supplementary materials.

Version 1:

Reviewer comments:

Reviewer #1

(Remarks to the Author)
Thank you for the detailed and well-reasoned responses.
Regarding the return period, the rationale for retaining the empirical approach is persuasive, particularly the observation that multiple heatwave events occurring within the same year may violate the block maxima assumption required for GEV analysis. Nevertheless, the authors may wish to consider, at least as a sensitivity analysis, the application of a Generalized

Pareto Distribution (GPD) within a Peaks-Over-Threshold (POT) framework. In contrast to GEV, the GPD is specifically formulated for threshold exceedances and is therefore more closely aligned with the event-selection methodology adopted in the manuscript. Importantly, this approach would retain the full set of exceedance events, thereby avoiding the reduction in sample size associated with annual maxima methods, while also enabling parametric estimation of return periods beyond the observed record. Even a limited comparison between empirical and GPD-based return period estimates for a subset of regions would strengthen the robustness of the analysis and help address potential concerns regarding the stability and precision of tail estimates.

Line 115: To begin the results section, provide a brief introduction to what will be analyzed.

Lines 382-387: improve sentence structure.

(Remarks on code availability)
The authors provide a set of Jupyter Notebook demo scripts accompanied by a README file. The README is concise and reasonably informative: it specifies that no installation is required, describes the expected directory structure, lists approximate runtimes and RAM requirements for each notebook, and identifies the tested module versions (numpy 2.4.3, xarray 2026.1.0).
I did not execute the notebooks, but upon inspection of the code the overall structure appears logical and consistent with the methodology described in the manuscript. The scripts are organized into three thematic blocks: climatology and threshold computation, model evaluation, and return time estimation. The use of standard, widely available Python libraries (numpy, xarray) is appropriate and ensures broad accessibility.

Reviewer #2

(Remarks to the Author)
The authors have carefully addressed my concerns, and I recommend accepting the manuscript in Nature Communications.

(Remarks on code availability)
The link seems not to work. ie., URL: https://ln5.sync.com/dl/9d2fd2dc0#it33i58q-rr3h5qs9-s9t7xuhe-6cdivkp5 (Password: peu9IFQbNGfUmWZ)

Reviewer #3

(Remarks to the Author)
I appreciate the authors' thorough efforts in revising the manuscript and responding to the reviewer comments. The authors have provided extensive explanations, additional references, and supplementary sensitivity analyses to justify their methodological choices.

While I remain somewhat unconvinced by the use of relatively low percentile-based thresholds for defining heat extremes from a practical meteorological perspective, I recognize that the approach adopted in this study is supported by parts of the existing literature and has now been discussed more transparently in the revised manuscript. In particular, some studies tend to incorporate relative discomfort, marginal mortality increases, or vulnerable subgroup responses into broader definitions of heat impacts. My main concern is that, if such relatively low thresholds are generalized too broadly, the concept of "heat extremes" itself may become diluted and lose practical meaning for impact-oriented climate risk assessment and disaster management. The additional analyses using higher percentile thresholds also improve the overall robustness assessment.

Overall, I believe the authors have taken the reviewer comments seriously and have adequately addressed the major concerns raised during the review process. Therefore, I have no further major comments and support publication of the manuscript in its revised form.

(Remarks on code availability)

Version 2:

Reviewer comments:

Reviewer #1

(Remarks to the Author)
Thank the authors for their detailed and comprehensive responses. I appreciate their careful consideration of our comments and the clarifications they have provided. Based on the revisions and the authors' clarifications, I recommend acceptance of the manuscript in its current form.

(Remarks on code availability)

Open Access This Peer Review File is licensed under a Creative Commons Attribution 4.0 International License, which permits use, sharing, adaptation, distribution and reproduction in any medium or format, as long as you give appropriate credit to the original author(s) and the source, provide a link to the Creative Commons license, and indicate if changes were made.
In cases where reviewers are anonymous, credit should be given to 'Anonymous Referee' and the source.
The images or other third party material in this Peer Review File are included in the article's Creative Commons license, unless indicated otherwise in a credit line to the material. If material is not included in the article's Creative Commons license and your intended use is not permitted by statutory regulation or exceeds the permitted use, you will need to obtain permission directly from the copyright holder.
To view a copy of this license, visit https://creativecommons.org/licenses/by/4.0/

Peer Review - Response to Reviewers

General Remarks

We would like to thank all three reviewers for their time and constructive comments, as well as their overall positive feedback. We have responded to each comment below, with the reviewer comments in **bold** and our response in blue, and the appropriate references and figures where applicable.

At first, we would like to mention a few general changes to the manuscript based on the individual reviewer's comments, facilitating interpretation of changes for all reviewers:

1. We have improved the contextualization of the observed reference events, based on comments by Reviewer 1, by e.g. putting the reference values in the context of other heatwaves in the reference data sets, i.e. Berkeley Earth and ERA5 (for more information see Table 2 in manuscript and methods), and by adding two additional heatwave characteristics to the framework, i.e. maximum severity and maximum heatwave area, and setting a focus on these additional characteristics in the GWL-based analysis.
2. Based on comments by Reviewer 2, we have made the text more reader-friendly by shortening the sentences where possible.
3. Based on comments by Reviewer 3, we have calculated heatwaves based on three additional higher percentile thresholds – in addition to the 90th percentile (Tx90p [1]), we calculated heatwaves based on Tx95p, Tx97.5p, and Tx99p.

Reviewer 1

Understanding how heat waves will evolve under future climate scenarios is crucial for anticipating impacts on human health, ecosystems, and socio-economic systems. Over the past decades, the frequency, intensity, and duration of heat waves have increased in many regions, consistent with observed global warming trends. The manuscript under review contributes to this effort by comparing observed heat wave characteristics with those projected by climate models for future periods. While the study presents an interesting and relevant framework, I have several questions regarding the methods, as well as a few suggestions that may help to strengthen the

analysis and enhance the clarity of the manuscript.

We would like to thank you very much for the positive evaluation of our paper and for considering our framework interesting and relevant. Please find our answers to your comments below.

I suggest further contextualizing the heat waves selected as reference events by comparing them with other heat waves observed during the historical period using ERA5 and Berkeley Earth datasets. This could be achieved by presenting graphs similar to those shown in Figure 1, including the different attributes of the heat waves. This comparison would help place the selected heat waves in a broader climatological context and facilitate a clearer interpretation of the results when comparing them with future projections.

Thank you very much for this suggestion. We definitely agree that there is need for further contextualization. Hence, we now invested substantial effort in contextualizing the heatwaves further and improving our framework (see discussion below about reference heatwaves in Pacific Northwestern USA & Canada and India & Pakistan). Therefore, in addition to the suggested visualization similar to Figure 1 in the manuscript, which we present for the historical model runs to cover the entire time window since 1850 (see Figs. S1 – S6), we added three additional means of contextualization, two of which are also relevant for your next comment below regarding the spatial extent of the reference events.

1. As a means of comparing the selected reference heatwaves with other heatwaves in the historical period in Berkeley Earth and ERA5, we ranked the heatwaves (see Table 2 in manuscript) computed from the historical period in the reference data sets. For the sake of consistency with our main analysis in the paper, we start this historical period parallel to our climatological base period (1961-1990) and continue to the end of the available data (2023). As described in the caption of Table 2 of the manuscript, the ranking is based on four categories: (1) heatwave characteristic among the top 3.0% from 1961 to date of reference heatwave, (2) top 3.0% in entire reference dataset from 1961 to 2023, (3) peak value from 1961 to date of reference heatwave, and (4) peak value in entire reference dataset from 1961 to 2023. We think that this provides an additional important means of contextualization not necessarily attained by the aforementioned figures alone. For instance, it provides a more detailed quantitative contextualization, similar to the exceedance percentage discussed in the results section of the manuscript, that is easy to interpret across all six characteristics.
2. We also added ridgeline plots (see Figs. S7 – S18) similar to Figures 3 and 4 in the manuscript for the historical period sub-divided into individual decades.
3. We also provide figures showing the spatio-temporal evolution of the reference events on a geographical map (lines 831–833).

We think that these additions provide a solid basis for contextualization of the selected reference events.

Page 8, lines 124-146 and lines 254-255: The paragraph is potentially misleading because it compares growth factors without clearly acknowledging that the European and Pacific Northwest reference heatwaves already cover a relatively modest fraction of land area, whereas the Australian, Chinese, and Southern African reference events are much more spatially extensive. The interpretation of the projected changes in spatial extent would benefit from a clearer discussion of the baseline size of the reference events in each region. In Europe and the Pacific Northwest, the reference heatwaves cover around 30% of the regional land area, so there is substantial room for future events to become larger. In contrast, the reference events in Australia, China, and southern Africa already exceed about 60% of the respective regional land areas, which inherently limits the possible relative growth in spatial extent.

Thank you very much for highlighting the need for improvement in this regard. In addition to a clearer discussion of the baseline sizes (lines 211–213, 465, 473), we added two additional means of contextualization (event ranking and reference event maps), as mentioned in the answer to your comment above, that have also been used to address this comment and to re-write the highlighted sections (see lines 209–227, 462–488). Even though you only mentioned the discussion of the baseline size of the reference events, we think that this also concerns the remaining heatwave characteristics. Hence, we also used the additional contextualization for the discussion of the baselines for the remaining characteristics. We think that this substantially improves the interpretability of our results.

Figure 3: I am somewhat surprised that the heat waves in India and Pakistan, as well as those in the Pacific Northwest, Canada, and the USA, do not appear as particularly severe in the current climate, given that several studies report record-breaking temperatures associated with these events (e.g., White et al., 2023). Consistently, Figure 5 shows very short return periods for these events, whereas observational analyses suggest return periods exceeding 100 years for the Western Canada heat wave (e.g., Malinina and Gillett, 2024). Taken together, these results raise the question of whether the models adequately represent key heat-wave characteristics (e.g., intensity, duration, and spatial extent) in the present-day climate. Could the authors clarify whether such an evaluation was performed?

White, R.H., Anderson, S., Booth, J.F. et al. The unprecedented Pacific Northwest heatwave of June 2021. Nat Commun 14, 727 (2023). https://doi.org/10.1038/s41467-023-36289-3

Malinina, E., and Gillet, N. (2024). The 2021 heatwave was less rare in Western Canada than previously thought. Weather and Climate Extremes,

43, Article 100642. https://doi.org/10.1016/j.wace.2024.100642

Thank you very much for highlighting the differences to the studies mentioned above. We are sorry that our attempt to address differences to other reports in the discussion of the manuscript was not clear enough. Hence we updated the discussion to make it clearer where the differences to previous studies originate from (lines 657–674). There are three main differences to the aforementioned studies:

1. Unlike White et. al. 2023 [2] (reporting the temperature value of single station locations, e.g. in Lytton, Canada, grid-box level temperature records, and area-averaged absolute temperature covering approximately 8°x5° on the pacific coast), we did not focus our analysis on a relatively small region where the highest absolute temperatures have been observed. Instead, we attempted to capture the reference heatwave in its entire spatio-temporal evolution. This leads to a substantially larger region analyzed in this study (see aforementioned figures displaying the spatio-temporal evolution of the reference heatwaves). Of the studies in your comment, Malinina & Gillet (2024) [3] set a focus on a, compared to the other studies, relatively larger geographical region, e.g. British Columbia. This politically pre-defined region, however, is still considerably smaller than our analyzed region.
2. The next significant difference to the aforementioned studies is that, as a measure of the intensity of the heatwave, we do not use absolute temperatures, but heatwave severity [4]. This metric is, of course, based on the absolute temperature values but represents a relative index where the temperature is normalized by the difference between the temperature threshold and the respective climatological mean, see methods (lines 964–965). Heatwave severity has previously been used in the context of marine heatwaves and we think that it provides a valuable means for inter- and intra-regional, terrestrial heatwave comparison, which is one of the strengths of this study.
3. While White et. al. 2023 [2] present temperature data for individual days and Malinina & Gillet (2024) [3] set a focus on TXx (annual maximum daily maximum temperature), we used median values (severity, spatial extent) for the entire heatwave analyzed. However, Malinina & Gillet (2024) [3] also show in their ERA5-based analysis of the state of Oregon, USA, that the length of the observed time window (TX7x, i.e. the warmest week of the year) can have a relevant influence on the results. Thus, based on your comment and the TX7x-based results in Malinina & Gillet (2024) [3], we see that some reference heatwaves, especially Pacific Northwestern USA & Canada, are insufficiently captured with only the four heatwave characteristics (especially median values) presented in the previous version of the manuscript. Hence, in addition to the median severity and median percentage of locally affected area (spatial extent) per heatwave we now also computed the maximum severity and maximum spatial extent for each heatwave. This should substantially improve the contextualization and interpretability of our results. However, as we do not only focus on the grid boxes with the highest observed absolute temperatures or politically pre-defined regions, as mentioned above, the differences to the aforementioned studies resulting from differences in geographical definition of the reference heatwave still remain.

Thus, to further address your comment, we

1. show in our results section that heatwaves in Pacific Northwestern USA & Canada and India & Pakistan were not only exceptional on the more localized level (as mentioned above) but also on the geographically significantly larger regional level – see, for instance, maximum severity in Pacific Northwestern USA & Canada and heatwave length and cumulative heat in India & Pakistan (lines 233–236, 259–261, 377–387, and Table 2 in manuscript)
2. updated the paragraph in the discussion, highlighting the main differences to previous studies (lines 657–674)
3. extended our analysis framework to not only include median severity and median percentage of locally affected area (spatial extent) per heatwave, but also the respective maxima (see Table 2 of manuscript).
4. included a brief discussion of the cumulative heat results in the first sub-section of the results (lines 259–279)
5. re-wrote the results section concerning the return periods (lines 534–614) to now cover all six analyzed heatwave characteristics and not only median severity. We find that this provides a clearer picture of the observed heatwave characteristics as not every heatwave characteristic is equally exceptional.
6. in addition to our quantile-mapping-based model evaluation (see comment below and methods section), we performed a sensitivity test evaluating if the models are capable of adequately representing area-weighted-mean and grid-box severity, which are the basis of all six heatwave characteristics. See discussion of test results below.

Sensitivity Test:
To get an estimate of whether the models are capable of adequately representing the central metrics, i.e. area-weighted-mean and grid-box severity, which all of our six heatwave characteristics are based on, we analyzed distributions of area-weighted-mean and grid-box severity per individual model, region, and model scenario, and compared them to the same types of distributions of reference data (see Fig. 1 in response letter). Our sensitivity test shows that the distributions of the severity values do not display substantial differences to the same type of distribution of reference data. These results are robust across model scenarios and regions. Inspired by Malinina & Gillet (2024) [3], we used the time series from the start of our baseline period up to 2023 to utilize a large enough sample. Thus, we concatenated the individual model's historical run to the respective future scenario. Based on our sensitivity test, we find that the model sample selected by our quantile-mapping-based model evaluation (see comment below) should provide a solid basis for the calculation of our heatwave characteristics. A more detailed analysis, however, would be beyond the scope of this study.
Considering that our sensitivity test shows no substantial differences between the individual models and the reference data in the distribution of severity values during the analyzed time interval and that Malinina & Gillet (2024) [3] highlight the sensitivity of return period estimates to region choice and also show that the length of the analyzed heat-affected time-window (e.g. TX7x as opposed to TXx [3]) can have

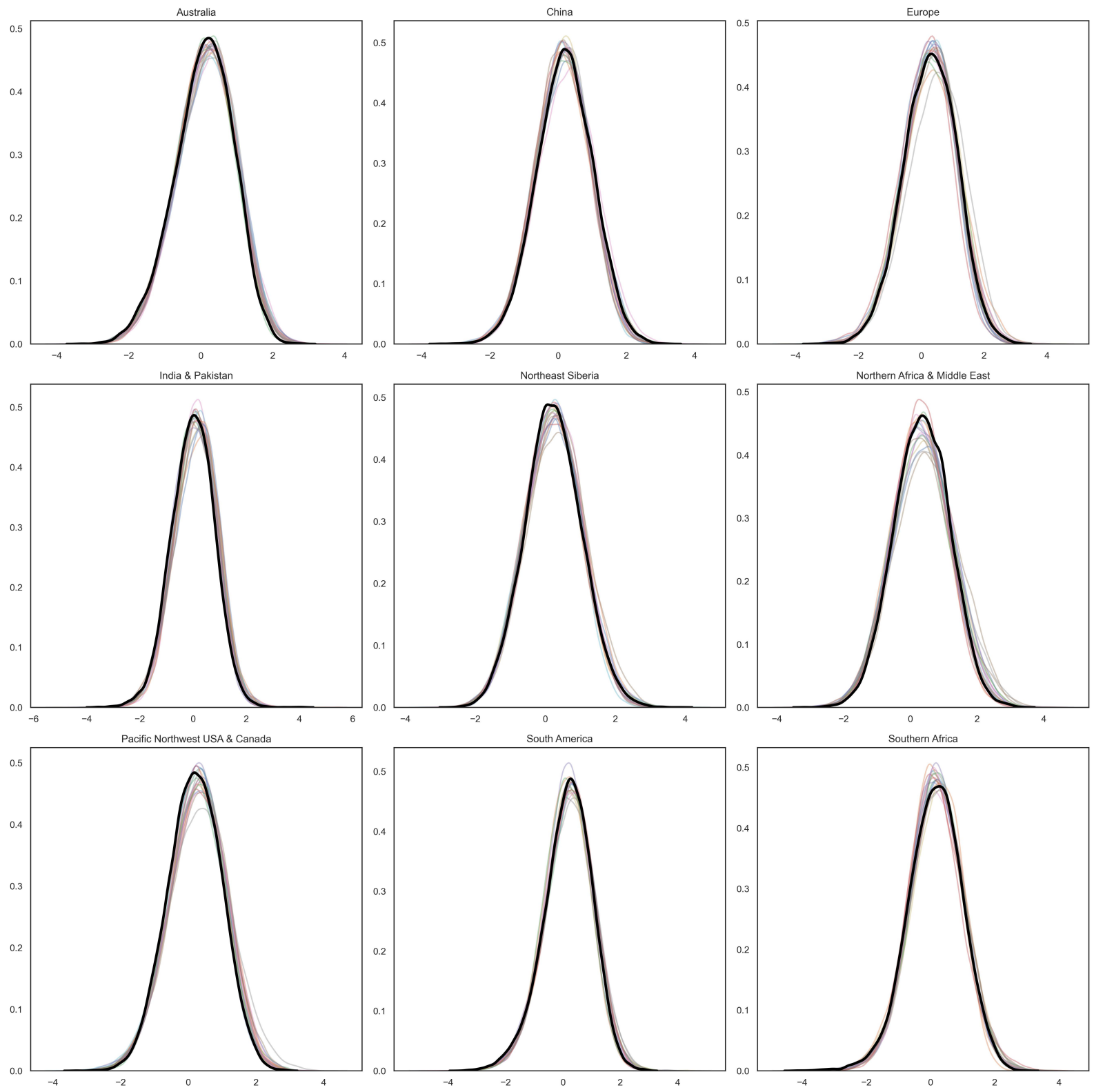

Fig. 1 Severity Sensitivity Test – Figure shows the distribution of area-weighted-mean severity values based on historical runs and SSP1-2.6 in MME-CMIP6. Colored thin lines show distribution of individual models and black line distribution of reference data. Our sensitivity test shows that the distributions of the severity values do not display substantial differences to the same type of distribution of reference data. These results are robust across model scenarios and regions. Inspired by Malinina & Gillet (2024) [3], we used the time series from the start of our baseline period up to 2023 to utilize a large enough sample.

a relevant influence on the return time, we conclude that the differences to their findings mainly result from the application of a different metric (severity index instead of absolute temperature) and a larger geographical region, as discussed above.

We hope that the discussion above clarifies the differences to previous studies and we find that the updates listed above substantially improve the clarity and quality of the study.

Lines 488-491: The authors apply a targeted bias correction by adjusting the mean temperature bias above the 80th percentile between the model and reference data, which simplifies the correction relative to full quantile mapping approaches. While this may be appropriate if the bias is approximately constant in the warm tail, it would strengthen the manuscript to justify this choice further—e.g., by showing how the bias varies across higher quantiles (P90, P95) or comparing the corrected tail

distribution to observations. Additionally, a brief comparison with standard quantile mapping results (even for a test case) could demonstrate that the simpler method sufficiently captures the relevant features for heatwave projections.

We would like to thank you very much for this comment. We think that there might be a misinterpretation of what kind of test we performed for evaluating the climate models. We would like to apologize if the previous description in the methods section was not clear enough. Hence we added a new paragraph (lines 855–908) to provide a more detailed description of how we tested the model performance and included example figures of the model evaluation results below (Fig. 2–5 in response letter). We would also like to note that, as mentioned in the updated description, our model evaluation approach is based on standard quantile-mapping approaches and uses confidence intervals computed from CanESM5-SMILE climate variability to compare the respective tail distributions of model and reference data and rank the models based on this performance test. For more details, please refer to the manuscript. The aforementioned example figures show how the bias varies across all available quantiles in the temperature window above the 80th percentile. As a consequence of this evaluation approach, models with a fairly constant bias in the tail of the temperature curve relative to the reference data (see Fig. 2–4) will reach a high performance index in the aforementioned ranking. Furthermore, climate models with a clearly different tail behavior (see Fig. 5) will perform poorly in our model evaluation. We find that this evaluation approach provides an effective means to reject the models with clearly poor performance in the tail of the temperature distribution. We hope that this updated description and the figures will help answer the questions.

Lines 556-560: Since the grid resolution varies across models, could the authors clarify how this might affect the estimated heat-wave areas? For example, could differences in grid size lead to apparent variations in spatial extent that are due to resolution rather than actual climatic differences? Would a sensitivity test using a common regridded resolution help assess the robustness of the results?

Thank you very much for raising this point. To address your comment, we added a new sub-section in the methods section (lines 1005–1036). We did consider regridding for the purpose of area calculations. Conservative regridding, for example, would have had the advantage that area-weighted-mean results remain the same, compared to using the native grid (at least to numerical precision) while providing a standard grid resolution across all models, with potential advantages when comparing spatial extent of heatwaves across different models. However, regridding can have significant disadvantages compared to using the native grid, like averaging across coastal gridpoints' ocean fraction at high resolution and interpolating to a grid/grid-resolution that does not align with the effective resolved orography and physics of the model. Thus, we designed a sensitivity test to estimate if different resolutions of the models might have a significant effect on the distribution of the computed heatwave areas, i.e.

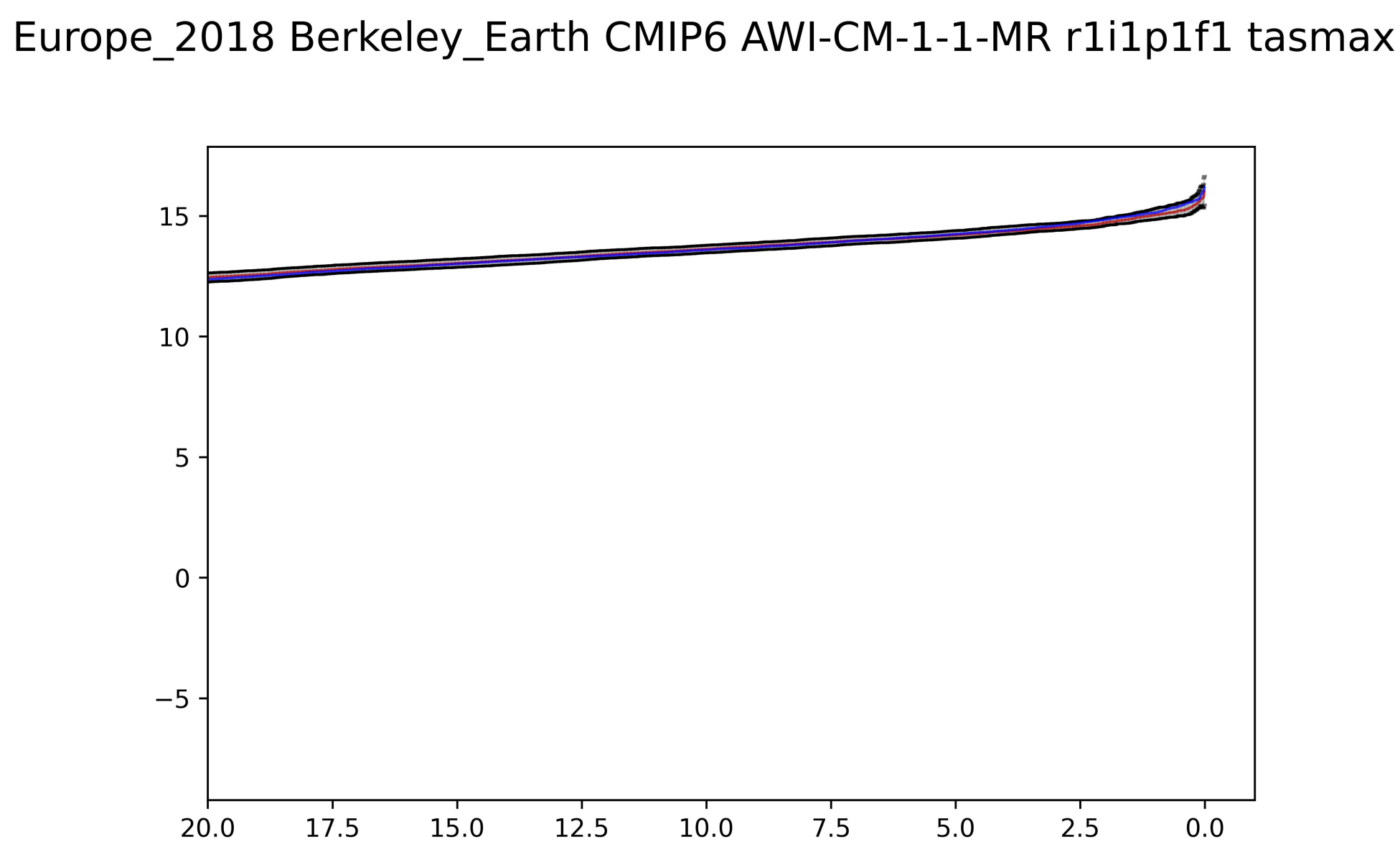

Fig. 2 Model Evaluation – Figure shows the model evaluation of the AWI-CM-1-1-MR model for Europe. Figure shows (1) the applied confidence intervals during the model evaluation. (2) the red line represents the model data and (3) the blue line the vertically shifted Berkeley Earth data. This model passed the model evaluation.

if the apparent differences in the spread in heatwave areas among different models can be explained by the different grid resolutions. In other words we test if the apparent differences are only statistical artifacts rather than differences in model physics. Our sensitivity test works as follows:

1. Selection of three different regular lon-lat grids (r320x160, r256x128, r128x64) available for the Climate Data Operators (CDO) (MPI for Meteorology, Hamburg, Germany).
2. Re-gridding our entire set of CMIP6 models using conservative re-gridding (CDO remapcon) for SSP1-2.6, SSP3-7.0, and historical model runs.
3. Calculation of local severity and, subsequently, heatwave area for each heatwave in the respective sample.
4. Kernel Density Estimates (KDEs) (Figs. 6–9 in response letter) of the entire distribution of heatwave area values per model, region, model run, and grid resolution.

The results of our sensitivity test show that the distributions of the computed heatwave areas for the different grid resolutions do not display substantial differences (Figs. 6–9). Hence, our test indicates that differing grid resolutions, if anything, only have a minor effect on model-specific differences regarding distributions of heatwave areas. As shown in Figures 6–9, these results are robust across model scenarios (SSP1-2.6 and SSP3-7.0). Figures 6 and 7 show results for EC-Earth3-Veg, which

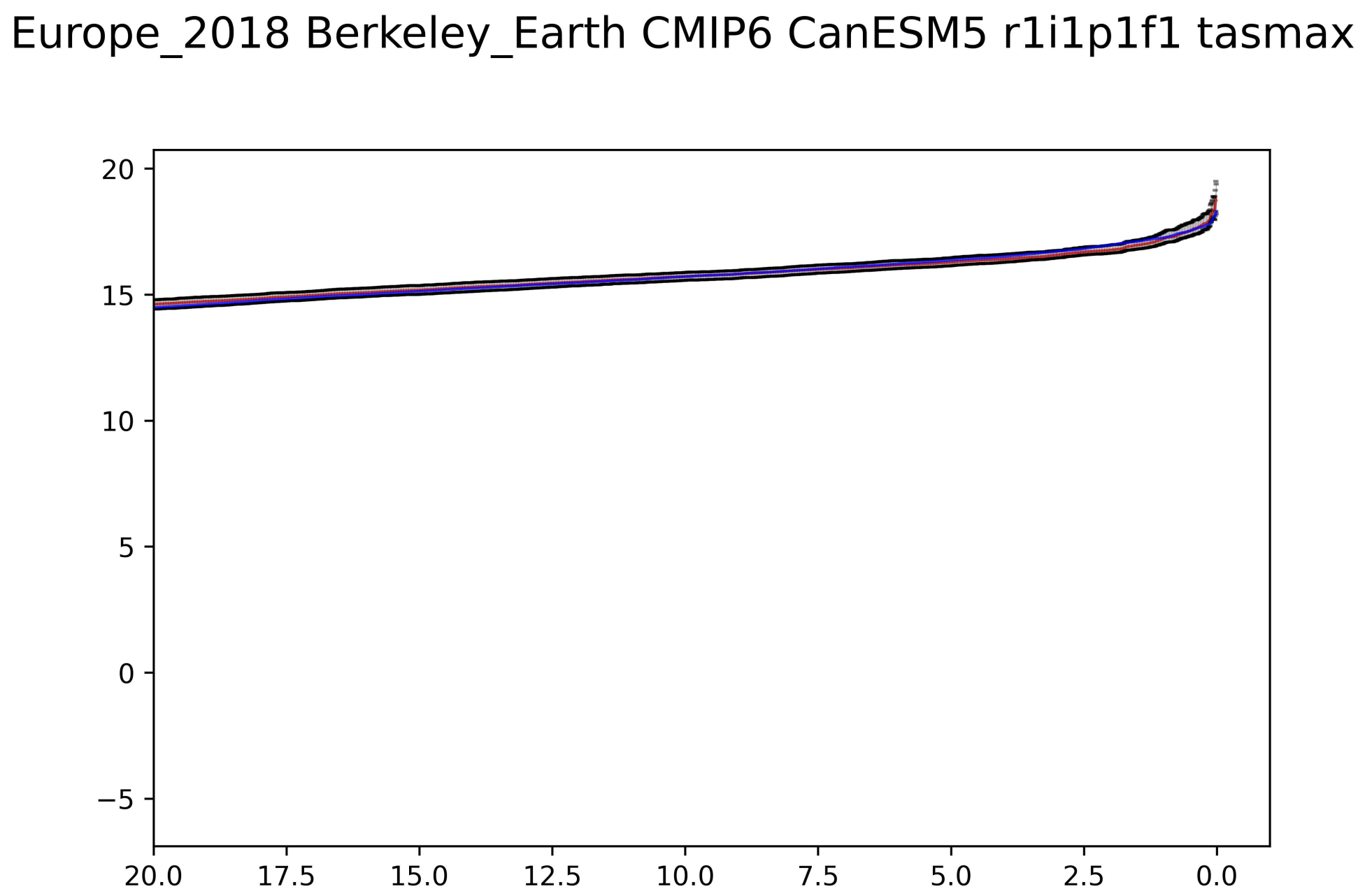

Fig. 3 Model Evaluation – same as Figure 2 but with the CanESM5 model. This model passed the model evaluation.

has a relatively high grid resolution (512x256). Figures 8 and 9 show results for MIROC-ES2L with a relatively low grid resolution (128x64). Thus, Figures 6–9 also show that the results are robust for using both a finer as well as a coarser target grid for regridding.

Given the, if anything, minor effect of different grid resolutions on the overall distribution of heatwave areas (Figures 6–9) and that the differences due to regridding are much smaller than the model differences, we think that, especially considering the disadvantages of regridding, using the models' native grids is the better choice for the purpose of our study.

Lines 591-594: The empirical return period estimation by counting events exceeding reference values is simple and transparent, but for rare heatwaves it may lack precision due to limited historical occurrences. Have the authors considered fitting an appropriate statistical distribution (e.g., GEV) to the data to estimate return periods? Fitting a distribution could provide more robust estimates, especially for rare or extreme events, and would allow for interpolation or extrapolation beyond the observed sample.

Thank you very much for highlighting this important point. Yes, we did consider fitting a GEV distribution to our data for the purpose of calculating the return periods. While acknowledging the caveats of using an empirical return period method,

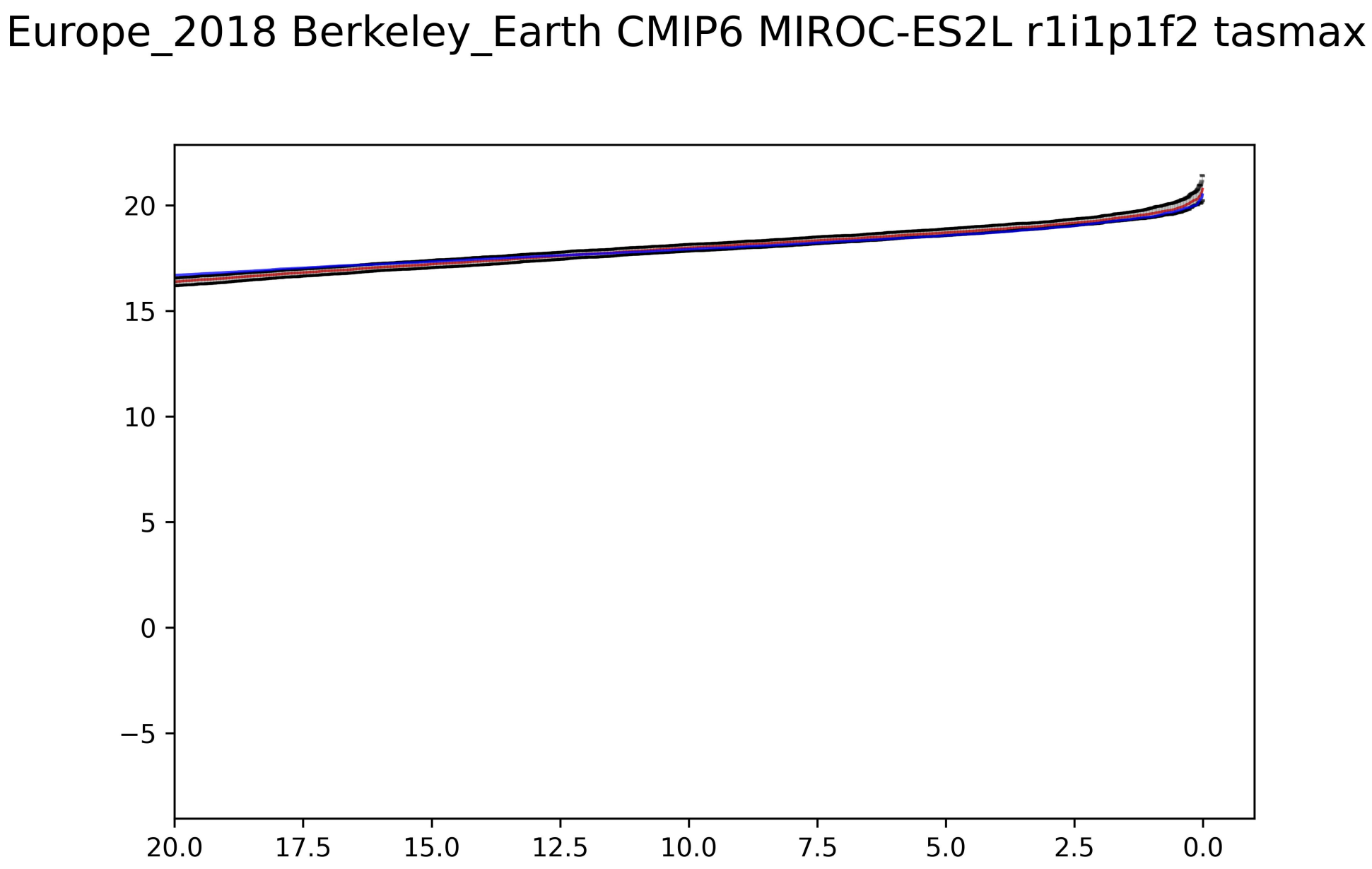

Fig. 4 Model Evaluation – same as Figure 2 but with the MIROC-ES2L model. This particular model shows the worst performance among the models selected. But even in this case the bias is approximately constant in the warm tail.

such as the limitation of the maximum return period to the length of the analyzed time window, we think that, for the purpose of our study, applying an empirical return period approach has significant advantages compared to GEV. In addition to only using minimal assumptions (essentially non-parametric) and, as you mentioned, being transparent and simple to interpret, our empirical return period approach does not only use block maxima, as in the case of fitting a GEV distribution, but uses the entire sample of events exceeding the reference heatwave characteristic. Hence, apart from avoiding the reduction in sample size as a result of fitting a GEV distribution, taking the entire sample of exceeding events is relevant in our case as our analysis shows that there is a considerable number of heatwaves with a heatwave characteristic value greater than those of the observed event but less than the respective block maximum. Hence, by only taking block maxima into account, our analysis would lose a considerable number of relevant projected events. Our analysis indicates that in many of the selected regions, future heatwaves exceeding the respective reference heatwaves are projected to happen more often than once per year. Hence, our empirical return period approach provides a higher precision compared to using block maxima. If anything, our results are conservative in the sense that we compare the return periods in the present-to-near-future and the end-of-century time windows to the historical 55-year period, which shows return periods limited by the length of the time window. However, as indicated in the results section, the relatively large percentage of models not able to simulate the respective heatwave characteristic of the

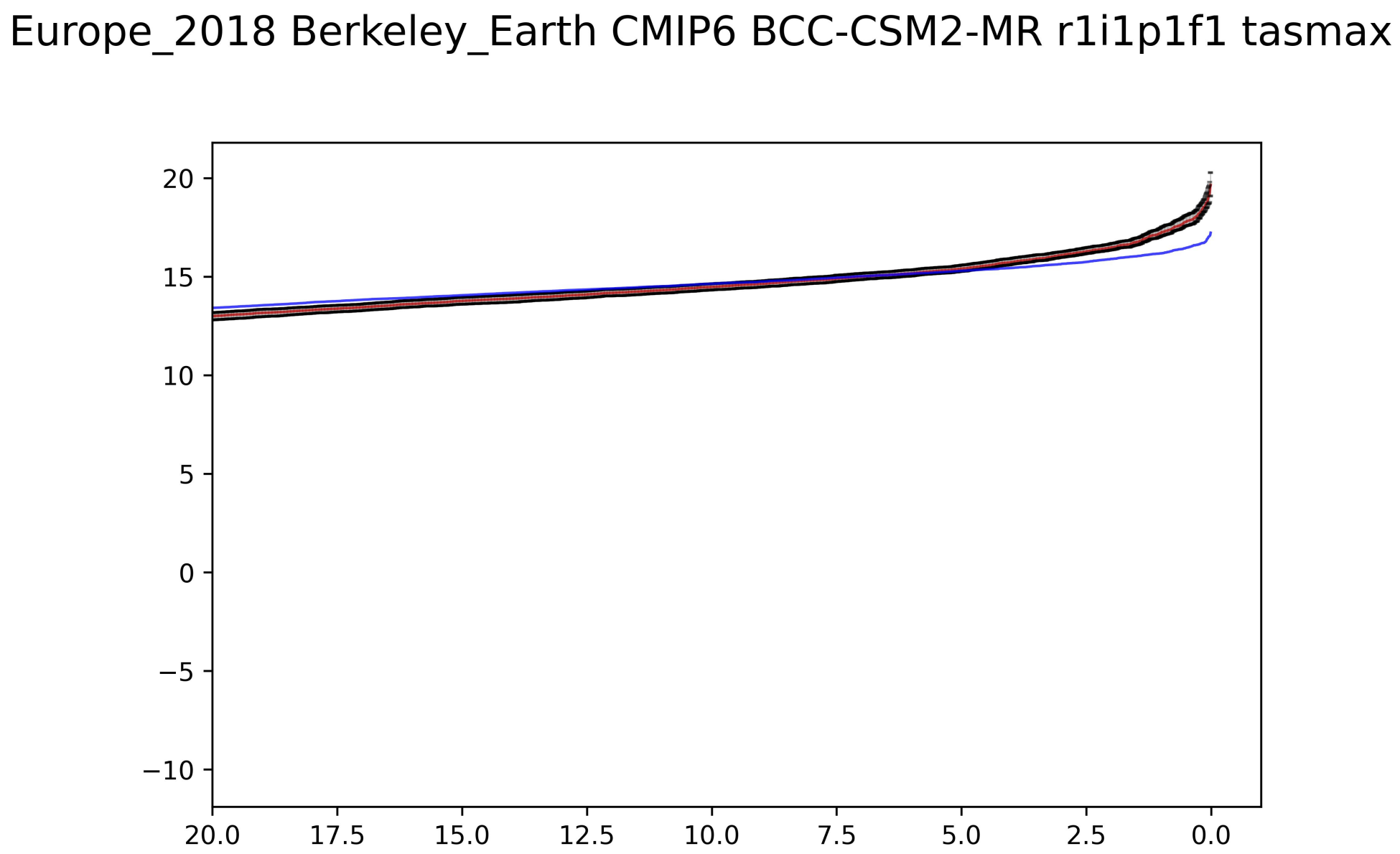

Fig. 5 Model Evaluation – same as Figure 2 but with the BCC-CSM2-MR model. This model failed the evaluation. This case shows that models with a clearly different tail behavior perform poorly in our evaluation.

observed event indicates that the return period would likely be considerably longer than 55-years. Nevertheless, the return times markedly decrease in our analysis in the present-to-near-future and the end-of-century time windows compared to the historical 55-year period. Due to the reasons mentioned above, which we included in lines 1073–1097 of the methods section, we think that the empirical return time approach is the more suitable choice for the purpose of our study.

Supplementary Information: There are too many figures and tables, which also do not have captions. Is there any way to summarize the information?

Thank you very much for your input regarding the supplementary material. We have invested substantial effort to give a better overview of the supplementary material and make it more accessible: We (1) have collected the supplementary figures and tables in a single PDF and a zip file, respectively, containing a table of content and the relevant captions. (2) Specific figures can now be accessed via bookmarks in the PDF that are directly linked to the (3) newly added figure captions discussing the figure on the subsequent PDF-page. (4) Table captions have been provided in a separate document added to the aforementioned zip-file. We very much hope that this improves the usability of the supplementary material. Regarding the number of supplementary materials, we are in contact with the editors to get editorial guidance. However, we think that for the purpose of peer review (and as Reviewer #3 positively

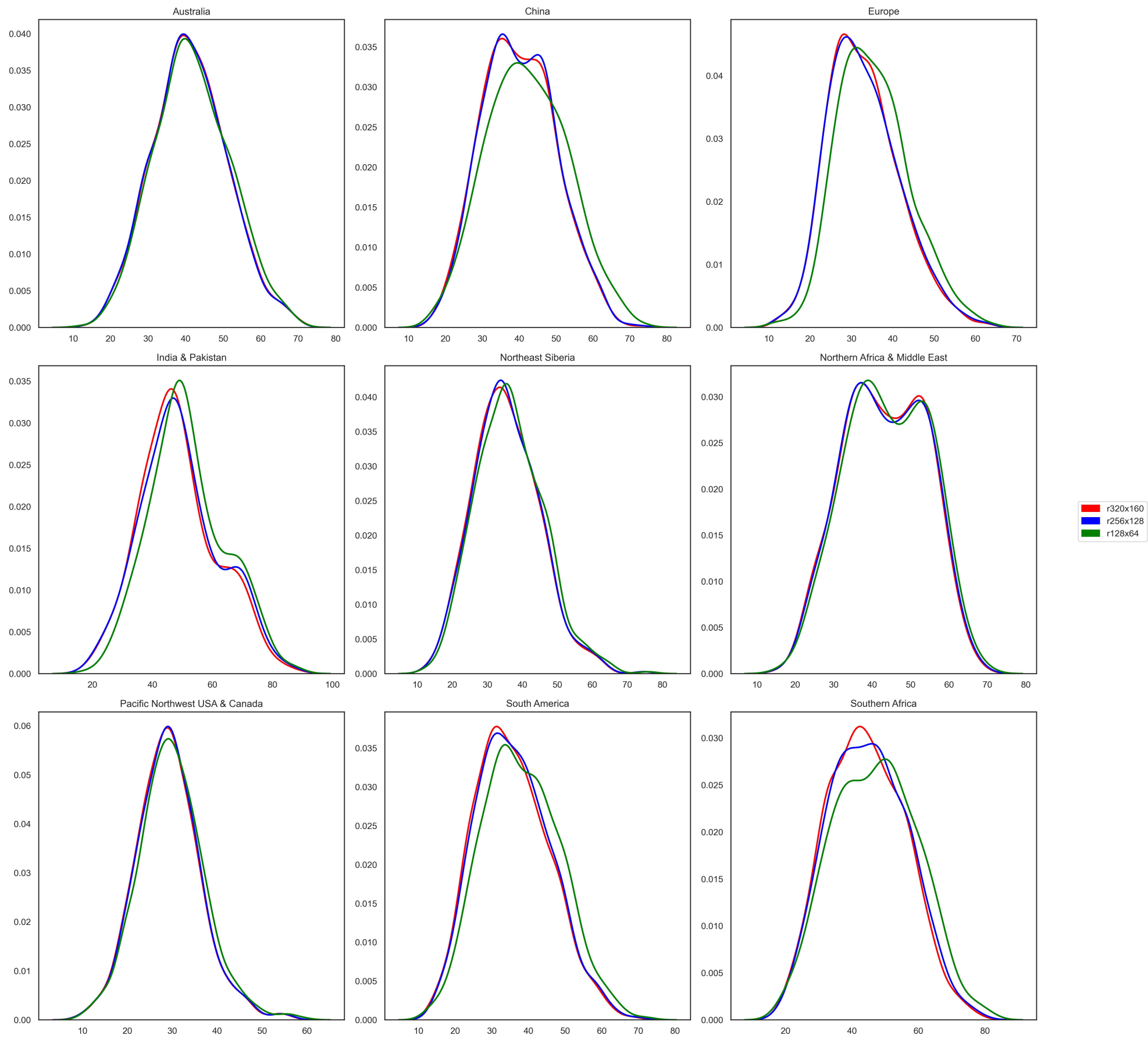

Fig. 6 Grid Resolution Sensitivity Test – Figure shows the heatwave area distributions for each region per grid resolution used for regridding. This figure shows the results for SSP1-2.6 in EC-Earth3-Veg.

highlighted the extent of information we provided in the supplements: "The extensive supplementary material further reflects the authors' rigorous and systematic approach, which deserves strong recognition." "The authors provide extensive supplementary figures and tables (...) that document the statistical analyses in detail and help readers follow the analysis workflow.") it was better to include all potentially relevant materials than accidentally removing relevant information. And, especially for the purpose of peer review, we wanted to avoid accidentally removing supplementary information that might be considered important by Reviewer #3. In addition, to address other reviewers' comments, we had to do additional calculations, e.g. higher temperature thresholds, and, hence, had to add additional supplementary material to back-up the results discussed in the updated manuscript. However, we have already e.g. removed the multi-characteristic-figures per individual model in CMIP5 and the tables referred to in Annex I, and will further adjust the number and selection of

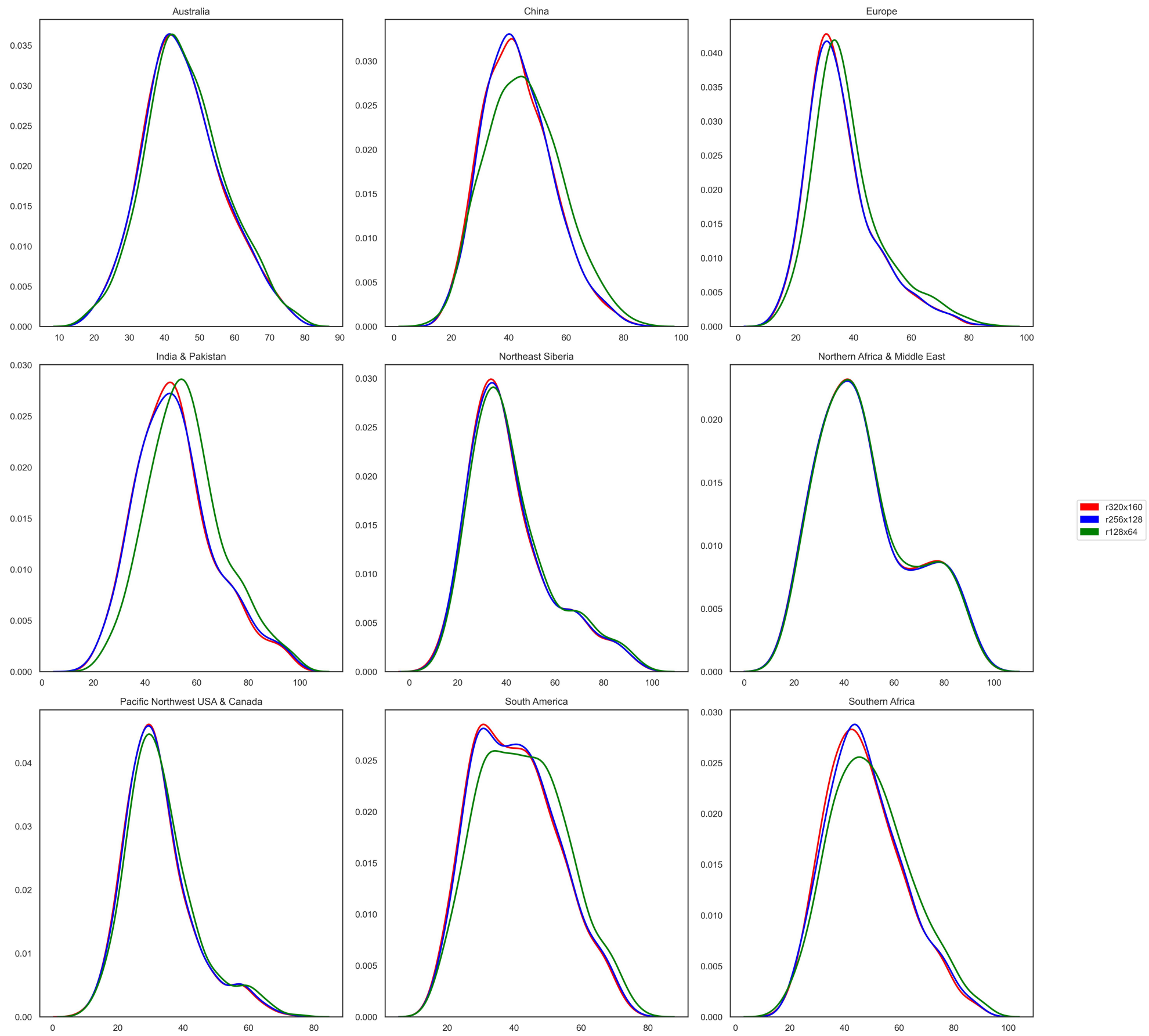

Fig. 7 Grid Resolution Sensitivity Test – Figure shows the heatwave area distributions for each region per grid resolution used for regridding. This figure shows the results for SSP3-7.0 in EC-Earth3-Veg.

supplements in accordance to the aforementioned editorial guidance.

Lines 457-458: What complications can arise from using a predefined region?

As the spatio-temporal evolution of heatwaves does not stop at pre-defined regions, i.e. politically defined regions or regions defined in previous peer-reviewed studies, using such pre-defined regions could lead to neglecting relevant parts of the reference heatwaves. This explanation was added to the methods section of the manuscript (lines 818–822).

The provided code allows for the identification of heat waves and the calculation of their severity, and it is generally well commented. However,

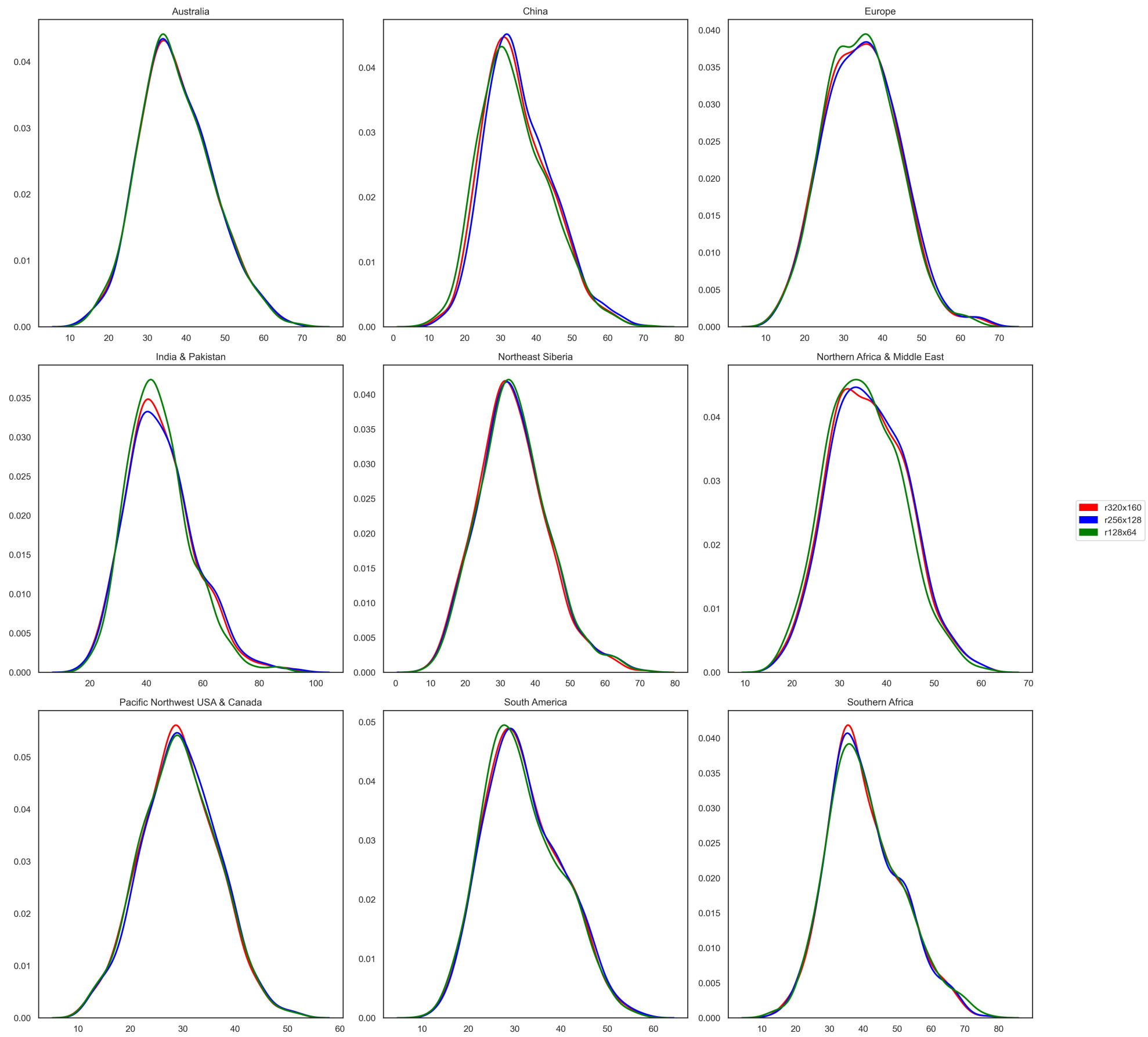

Fig. 8 Grid Resolution Sensitivity Test – Figure shows the heatwave area distributions for each region per grid resolution used for regridding. This figure shows the results for SSP1-2.6 in MIROC-ES2L.

I did not find code related to the bias-correction procedure or the estimation of return periods.

Thank you very much for highlighting the missing scripts for the model evaluation and the return period. We have updated the repository to include the mentioned scripts as workable demo versions for a subset of the data.

Reviewer 2

This manuscript investigates future heatwave projections using climate model simulations under multiple emissions scenarios. Its main contribution is the event-based approach that embeds observed high-impact

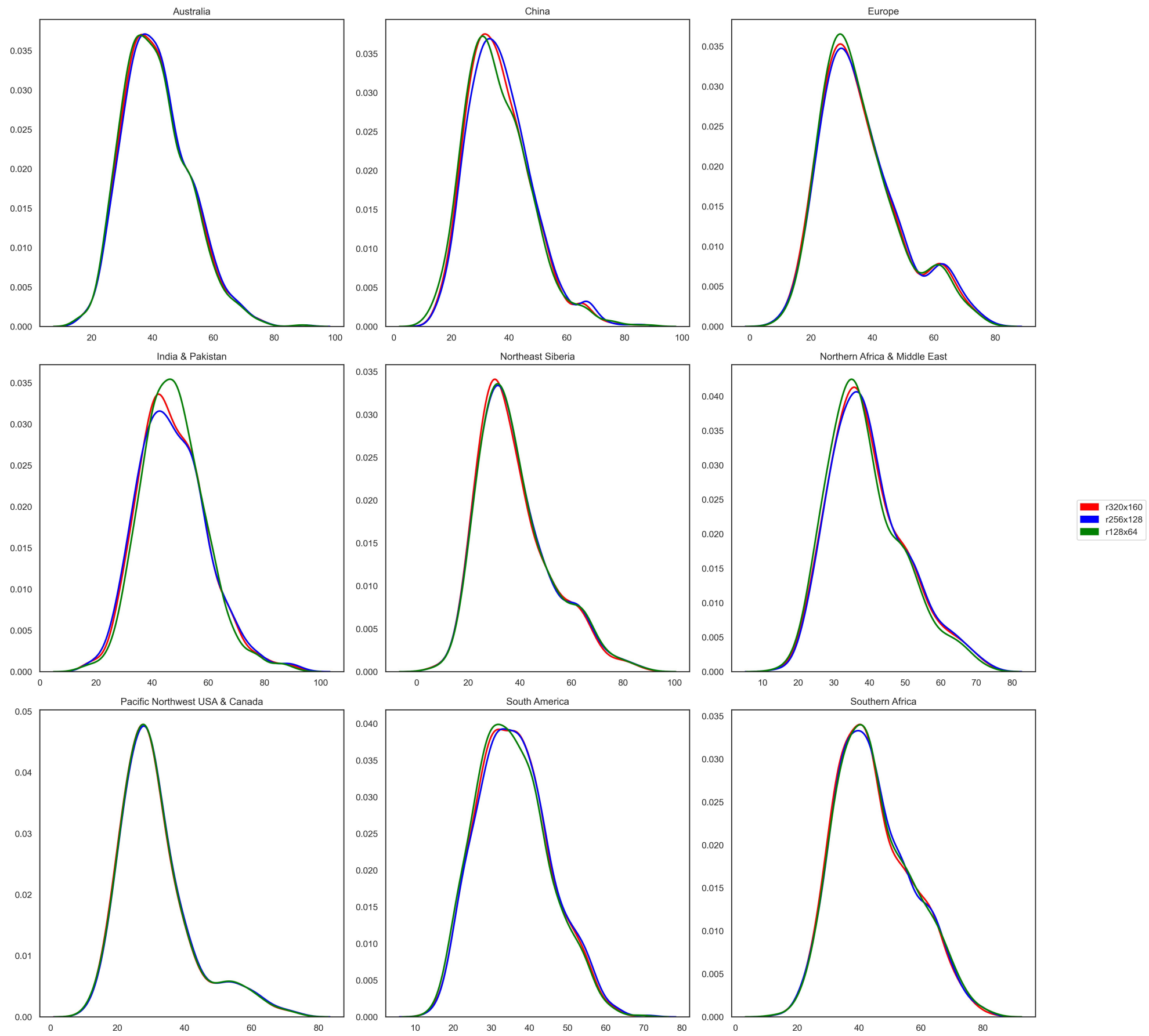

Fig. 9 Grid Resolution Sensitivity Test – Figure shows the heatwave area distributions for each region per grid resolution used for regridding. This figure shows the results for SSP3-7.0 in MIROC-ES2L.

heatwaves into future projections, enabling a direct comparison between past extremes and future conditions. The analysis considers multiple heatwave metrics and combines multi-model ensembles with large initial-condition ensembles, providing a comprehensive assessment of uncertainties. Although the general conclusion that future heatwaves will intensify under continued warming is not unexpected, the results offer useful quantitative evidence that is highly relevant for heatwave mitigation and adaptation. The manuscript is clearly written and methodologically sound. I recommend minor revision, mainly to improve clarity and presentation.

We would like to thank you very much for the positive evaluation of our paper and for considering our work useful, clearly written and methodologically sound. Please

find our answers to your comments below.

Lines 97–122. Please clarify how the proportions reported in this section (e.g., the 12–22% values in Line 97) are calculated. In addition, four heatwave metrics are considered in the study. Are these proportions derived from heatwave severity only, or are similar increases found consistently across the other metrics as well?

Thank you very much for this comment. To clarify the calculation of the proportion, we added a paragraph in the methods section ("Heatwave Characteristics and Exceedance Percentage") explaining the computations and providing an example to enhance readability (lines 995–1003).
Regarding the second question: No, the proportions are not limited to severity alone and the exceedance likelihoods of other heatwave characteristics are not necessarily similar to the ones of heatwave severity. The discussion of the exceedance percentages for different heatwave characteristics is divided into separate paragraphs. The paragraph after the one mentioned in your comment, for instance, discusses the exceedance percentages regarding the spatial extent of heatwaves. To make it clearer and more reader-friendly, we re-phrased a few key parts to highlight which of the heatwave characteristic is being discussed (e.g. see line 209). We are very sorry that this was not clear enough before and hope that the improvements to the text make it easier to follow.
In addition, as the different heatwave characteristics show different exceedance percentages and are not necessarily equally rare in the historical period (e.g. an exceptionally large heatwave is not necessarily exceptionally long), we ranked the heatwaves found in the reference data sets (Berkeley Earth, ERA5) and added rank-related indicators to Table 2 (for more details see table caption and methods section lines 823–833) to improve the interpretability of the increases across the heatwave characteristics.

Line 170. This statement is unclear in its current form. Please consider rephrasing it for clarity or removing it if it is not essential to the main argument.

We have updated the incomplete statement and hope that it's easier to understand this way – see lines 284–287.

Overall, the manuscript contains too many long and complex sentences. Revising these for conciseness would improve readability and accessibility. For example, Lines 178–183 are hard to follow, and the sentence beginning with "The in . . . " in Line 191 appears incomplete or unclear. Shortening or restructuring these sentences would substantially improve clarity.

As the paragraph indicated by "Lines 178-183" in your comment contained many long and complex sentence structures, we re-wrote, re-structured, and shortened the

entire paragraph (lines 309–322). In the newly added text of the updated manuscript, we stayed as concise as possible.

Line 206. The discussion shifts from SSP emission scenarios to Global Warming Levels (GWLs) somewhat abruptly. A brief transitional explanation would help clarify the relationship between the SSP-based analysis and the GWL framework, including how the GWLs are defined and applied in the analysis.

We now added a transitional paragraph at the beginning of the GWL-section (lines 343–360) explaining focus and advantages of each analysis type (SSP- and GWL-based analysis). We hope that this improves the readability of the manuscript and the interpretability of our results. As the definition of GWLs requires a longer description, we discuss the GWL calculations in the methods section (lines 1038–1055).

Lines 214–215. Please specify which Global Warming Level is being referred to here.

Statement has been replaced during manuscript update, and references to which specific GWL is being discussed have been added more often to enhance readability.

Regional definition. The term "Pacific Northwestern USA" is used, but it is unclear whether all regions analyzed in the study are explicitly defined or outlined in a map. If not, please clarify the regional definitions and indicate them consistently.

Thank you very much for highlighting this potential source of misunderstanding. The region titles used in our study are supposed to roughly capture the affected region in an intuitive way (see methods lines 829–833). The title will, of course, not perfectly capture all parts of a specific region. Hence, to address your comment, we provide figures showing the spatio-temporal evolution of the reference events on a geographical map (lines 831 – 833) in addition to the list of regional coordinates in Table 1 of the manuscript. This should give a good idea of the geographical region analyzed and provide additional insight into, and contextualization of the selected reference events. Furthermore a quick note on the region title mentioned in the comment, to avoid potential misunderstandings regarding this particular region: The actual region title is not "Pacific Northwestern USA" but "Pacific Northwestern USA & Canada". Hence, the selected region does not only include regional parts in the USA but also parts of Canada. For more information please see Table 1 of the manuscript and the aforementioned figures.

Reviewer 3

I have read this manuscript very carefully and thoroughly. Given the scope of the study, the very large number of supplementary figures (>180) and tables (>30), it is evident that the authors have invested substantial effort and care into this work. Overall, I highly appreciate the ambition, originality, and general conceptual framework proposed in this paper.

We would like to thank you very much for the positive evaluation of our paper and for appreciating the ambition, originality and the conceptual framework of the paper and the effort and care that went into this work. Furthermore, we would also like to highlight that we very much appreciate your careful and thorough review. You have provided us with a lot of food for thought and have helped improve this paper and future papers by encouraging us to become even more ambitious by, inter alia, presenting results for multiple temperature thresholds (Tx90p, Tx95p, Tx97.5p, and Tx99p).

We have attempted to focus our response on the individual comments. However, as your concerns 1 and 2 as well as the "conceptual distinction" are very inter-related many of our points will also be relevant for the other comments. Hence, it may happen that we repeat or re-iterate a point made in a previous comment. We hope that this will facilitate the interpretability of our response without creating redundancy.

General assessment and strengths

The overarching research logic proposed by the authors is novel, well motivated, and compelling. By placing several major, recently observed global heatwave events into a future-oriented statistical framework, the study provides an intuitive and powerful way to contextualize the integrated intensity of recent extreme heat events relative to what climate models project for the coming decades. This framing substantially improves interpretability and helps readers recognize that some of the most socially and economically impactful heatwaves observed in recent years may, from a future-climate perspective, no longer represent the upper tail of extremes. Instead, the results suggest that even more frequent and more severe events should be expected in the coming decades. This conceptual approach is innovative and valuable, and I fully agree that such a framework can significantly advance our understanding of major heatwave hazards under climate change. Given that this overall logic is sound, my comments below focus primarily on technical and methodological aspects, particularly those related to heatwave definition and event identification.

We would like to thank you very much for highlighting the strengths of our study and for considering our approach as novel and innovative, well-motivated, compelling, and valuable.

Scope of expertise and perspective

Before detailing specific concerns, I would like to acknowledge the limitations of my own expertise. I am a researcher from China's national meteorological research system, and my professional experience over the past decade has focused primarily on observed weather hazards in China and their impacts on socio-economic systems. My understanding of heatwave events in other regions is largely informed by the scientific literature and media reports. Consequently, the concerns raised below are especially grounded in the Chinese observational and operational experience, though I believe they have broader relevance.

We would like to thank you very much for providing us with information on your professional background and expertise. We highly appreciate this information as it has helped a lot in interpreting your comments and working with your feedback to improve the paper.

Conceptual distinction: extreme events vs. weather hazards vs. meteorological disasters

A key issue concerns the conceptual distinction between "extreme events", "weather hazards", and "meteorological disasters", which are often used interchangeably but are fundamentally different.

- **Extreme events are defined statistically, referring to events with low occurrence probability in one or more relevant dimensions, such as severity, timing, spatial extent, or other characteristics.**

- **Weather (natural) hazards are events that produce direct or indirect adverse impacts on socio-economic systems, at least within one sector (for example, extreme heat restricting outdoor labor and reducing productivity, or increasing electricity demand and leading to grid overload and higher household energy expenditures).**

- **Meteorological disasters represent cases where severe weather causes direct socio-economic losses, such as loss of life, damage to housing, or destruction of public infrastructure, emphasizing direct losses.**

From this perspective, the 22 regional heatwave events selected in this study clearly fall into the category of weather hazards, and many of them can reasonably be classified as disaster-level events, given their documented societal and economic impacts. However, the heatwave definition adopted in the manuscript is purely statistical, based on the calendar-day 90th percentile of daily maximum temperature during the warmest six months over the 1961–1990 baseline. While this is a classical and well-established approach for identifying statistical extremes, it does not

necessarily correspond to the threshold at which heat events begin to generate meaningful socio-economic impacts.

First of all, we would like to thank you very much for this detailed summary of the conceptual distinctions. We certainly agree that the observed reference heatwaves fall into the category of weather hazards, and many of them can reasonably be classified as disaster-level events. However, we think that this classification as weather hazards and/or meteorological disasters does not preclude a statistical extreme event analysis, as the selected heatwaves also fall in the category of extreme events. Hence, the observed reference events fall into the intersection of extreme events and (at least) weather hazards.
Furthermore, we think that, to a certain extent, an attempt to contextualize projected heatwaves with recent heatwaves will most likely use weather hazards or meteorological disasters as a reference as these are the events that are being reported on, and as these are also the events that are in recent memory of the affected population. However, we think that this additional characteristic of such heatwaves does not preclude using them as a reference for a statistical analysis. We also agree that the statistical basis of our analysis framework is a well-established approach for identifying statistical extremes, which facilitates comparison to previous studies.
Furthermore, we also agree that a specific statistically defined threshold does not necessarily correspond to a specific impact or a set of specific impacts. However, we argue that the term "meaningful" socio-economic impact is very context-dependent. According to the temperature threshold choice ($\geq 35°C$) presented in concern 1 below, we assume that you defined the term "meaningful" as a situation where temperatures reach levels threatening human survivability. We certainly see the merit in using this particular absolute temperature threshold for a focus on specific impacts. However, as the term "meaningful" is very context-dependent, we argue that depending on the selected sector/impact, "meaningful" impacts can already occur at lower temperatures. Please see more detailed discussion on this and corresponding references under concern 1 below.
In addition, we would like to note that, as mentioned in the updated discussion of our manuscript, even though many of the analyzed reference heatwaves have known reported impacts, we do not quantify and extrapolate specific impacts/impact types into the future - a study focus worthy of future applications of our framework, however, beyond the scope of this study (lines 708–720). Such an extrapolation of impacts would, for instance, require the focus on a specific set of impacts to be extrapolated and, in addition, a significantly sized set of additional assumptions and probably a focus on much smaller geographical scales (sub-regional or grid box level only), as the additional assumptions, depending on the selected impact, might not be valid across large regions (see discussion of literature below). As a result, this could hamper inter-regional comparability, which is one of the strengths of the current study.
Nevertheless, as mentioned in the first part of our response you have provided us with a lot of food for thought and have encouraged us to become more ambitious in this paper and future studies. Hence, in the updated manuscript we do not only present results based on the 90th temperature percentile but also results based on Tx95p,

Tx97.5p, and Tx99p. For more details on this, please see response to the following comment. So, short of a complete re-design/re-conceptualization of the study, we attempted to take up as many of your valuable suggestions within our framework as possible, which, we think, significantly improved and strengthened our study.

Concern 1: Heatwave threshold may be too low from a hazard perspective From a Chinese operational and observational standpoint, the chosen threshold appears too low to represent impactful heatwave hazards. Using the authors' definition implies that, on average, each grid point experiences approximately 18 heatwave days per year during the 1961–1990 period. This is difficult to reconcile with empirical experience. For comparison, within China's national meteorological hazard monitoring and warning system:

- **The lowest-level heat warning is issued only when daily maximum temperature reaches 35 °C for at least three consecutive days.**
- **The highest-level heat warning requires daily maximum temperature to exceed 40 °C.**

In practice, heatwave events that cause substantial socio-economic impacts in China almost always exceed 35 °C, and typically exceed 40 °C.
To illustrate this point quantitatively, I analyzed historical observations from Beijing and Shanghai, China's two largest metropolitan areas, using the authors' exact methodology (15-day moving window percentile estimation followed by 31-day smoothing) over the warmest six months (April–September) during 1961–1990 (see attached figures):

- **Beijing:**
  - **Calendar-day 90th-percentile thresholds range from approximately 22 °C to 35 °C (Fig. 1a).**
  - **Only 9 calendar days have 90th-percentile thresholds exceeding 35 °C.**
  - **The mean 90th-percentile threshold is approximately 31.2 °C.**
  - **Corresponding calendar-day 99th-percentile thresholds are shown for reference (Fig. 1b).**
- **Shanghai:**
  - **Calendar-day 90th-percentile thresholds exceed 35 °C on only 16 calendar days (Fig. 2a).**
  - **The mean 90th-percentile threshold is approximately 30.2 °C.**
  - **Corresponding calendar-day 99th-percentile thresholds are shown for reference (Fig. 2b).**

Under this definition, many identified "heatwave days" would not produce noticeable impacts on socio-economic systems, leading to an over-identification of heatwave events. This likely explains why the manuscript reports an unusually large number of heatwave events, including cases outside China where heatwave durations exceed 180 days, which I found

conceptually puzzling and which motivated my closer examination of the methodology.

Thank you very much for your thorough review of our methodology, including the insights into the Chinese heat warning system, as well as trying to reproduce our results using the methods described in the manuscript. We very much appreciate the effort you put into this review.

Below you find the results of our extensive literature research on the relevance (and potential impact-relevance) of our results. We apologize for the length of the section below. However, as you have done a very thorough review of our paper yourself, we assumed that you would appreciate an equally thorough response to your comments and would be interested in more than just simple references/links to relevant publications. Hence, below you can find a list of relevant publications with an explanation why we think they are relevant to our study and your comments, and the corresponding citations. Note that the figures and tables mentioned in the discussion below refer to the respective publication and do not refer to the material of our analysis. The following discussion of the impact-relevance of lower temperatures is also relevant for you next comment below.

Impact-relevance of lower absolute temperatures, and percentile-based thresholds:

Here we would like to discuss a few points and the results of several studies that emphasize the relevance of lower temperatures and/or location-specific percentile-based heatwave thresholds for specific impacts, and, thus, support the relevance of our methodological choices.

1. Vanos et. al. (2023) published a physiology-based human survivability-model in Nature Communications [5], where they show that healthy females in two age groups (18-40 yrs and over 65 yrs), under certain conditions relating to relative humidity and shade/sun-exposure, can have survivability limits at significantly below 40°C and even slightly under 35°C. This does, of course, not mean that all of the conditions analyzed in their study are equally likely, but it shows that life-threatening conditions can already occur under 40°C and even slightly under 35°C (Figure 2, Table 2) without even taking into account sensitivity to illness or health status, which is even more relevant for the elderly population. Going beyond the question of simple survivability, they also present a liveability-model, where they estimate the limit to safe physical activities represented by metabolic equivalents (i.e. how much energy the body uses during physical activity relative to resting) under a large number of different (environmental) conditions. Their physiology-based estimates show that temperatures below 35°C (or in some cases even below 30°C) can pose significant restrictions on the possible physical activities (from sport to even moderate everyday activities) of healthy individuals (Figure 4). As a quick interpretative sidenote, even though it is beyond the scope of the aforementioned study, it seems reasonable to assume that this might have the potential for different kinds of trickle-down effects from decreased productivity to even mental

health issues (again, this model does not account for sensitivity to illness etc.). Hence, we think that these results emphasize the relevance of temperatures even below 30°C for human health and liveability.

2. Schwarz et. al. (2020) [6] published a study in Environmental Research Letters on "the health burden of fall, winter and spring extreme heat events in Southern California (...)", where they examined the relationship between hospitalizations and extreme heat events. To avoid misunderstandings, please note that the references tables below refer to the tables in Schwarz et. al. (2020) and not our analysis. They analyzed over 1.5 million unscheduled hospitalizations categorized by several ICD-9 codes, including acute renal failure (584), dehydration/volume depletion (276.5), and ischemic heart disease (410-414). Among all the medical diagnoses analyzed, the three mentioned above showed the strongest association with extreme heat. To account for seasonality and month-of-year, they used a relative temperature threshold (99th percentile) for the extreme heat definitions on seasonal and monthly basis. As a result, the seasonal temperature thresholds ranged from 31.68°C in fall to 25.35°C in winter, and the monthly from 32.71°C [Table S1] in September to 22.78°C in December. Despite the relatively low absolute temperature threshold in December, their results show "a particularly strong association with extreme heat events with the monthly definition in December (OR: 1.30, 95% CI: 1.10, 1.54)" for ischemic stroke. Furthermore, "[f]or the diagnoses related to volume depletion (acute renal failure and dehydration), the strongest association was observed during the fall and spring (...)" with limited association in winter for dehydration (OR: 1.16 [1.01, 1.33]). For the latter two diagnoses the results also show a clear association with extreme heat in the month of May (figure S2), which has a temperature threshold of less than 29°C. In addition, their results also show an association (OR: 1.27 [1.06, 1.53]) (see table S5) between extreme heat in the month of March (threshold 26.18°C) and acute myocardial infarction. They state that "[i]t is plausible that the health impacts of extreme heat events that are observed in fall, winter and spring which correspond to moderate temperatures on the absolute scale are related to the population's ability to acclimate to high ambient temperatures. (...) Interestingly, we generally found stronger effects of extreme heat in the spring and hospitalizations (table S5), although the temperature thresholds are lower than for extreme heat events in the fall (table S1). This could indicate that the population is more susceptible in the spring season as they are not acclimated to heat following the winter season. December is the month with the highest association with extreme heat events for hospitalizations for ischemic stroke (figure S3). This specific effect observed could also be related to the lack of acclimation of the population to heat during the winter period; these extreme deviations from the temperature distribution of that month could make it difficult for non-acclimated biological systems to respond."
Even though there are significant methodological differences to our calculations, we think that the results of this study support the relevance of our results and methodological choices (relative threshold etc.), and that, even though some absolute temperatures might be relatively low compared to extreme temperature

values in peak summer season, the time window when extreme temperatures (i.e. temperatures above a specific percentile) occur can have a significant effect on potential health impacts.

3. Guirguis et. al. (2018) [7] published a paper in GeoHealth on "Heat, Disparities, and Health Outcomes in San Diego County's Diverse Climate Zones", where they analyzed hospitalization data for three unique climate regions in San Diego County, California, USA alongside temperature data to assess health impacts of ambient air temperature at varying exceedance thresholds. They report health impacts at lower temperatures at the coastal region of the county (median daily high temperature of 24.6°C and 95th percentile of 30.7°C) compared to inland locations for multiple disease categories. They "used daily maximum temperatures in the range of 65 °F (18.3 °C) to the regional 95th percentile (see Table 1); therefore, the upper bound varies by region. From Figure 3 (left column) a significant health effect is observed for all three regions for the heat illness category. Within this category, the temperature threshold at which a significant impact is observed emerged at cooler temperatures for the Coastal region than the Inland and Desert regions. In the Coastal region, a significant health impact (RR = 1.31; 95% confidence interval [CI; 1.07, 1.61]) emerged at 73 °F (22.7 °C). In the Inland region, we detected an impact (1.37; 1.07, 1.79) at temperatures at or above 79 °F (26 °C). In the Desert region, the initial impact (2.77; 1.09, 6.55) is seen at 90 °F (32.2 °C)." According to their results "[t]he health risk [for the other disease categories] emerged at temperatures in the range of 74–81 °F (23–27 °C), depending on disease category." Even though the specific details of the methodology and the geographical extent of the analyzed region differs from our study, we think that this again highlights the impact-relevance of lower absolute temperatures for human health.

4. Petitti et. al. (2016) [8] published a study in Environmental Health Perspectives on "Multiple Trigger Points for Quantifying Heat-Health Impacts (...)", where they quantified the link between temperature variables, including daily maximum temperature, and ten health (mortality and morbidity) events in Maricopa County, Arizona, USA (including the city of Phoenix), in a time window from May to October (similar to the time window analyzed in our study). Based on these links, they estimated three different "trigger points", including an increasing risk temperature (IRT) and a minimum risk temperature (MRT) defined as "the lowest temperature above which a consistent increase in relative risk was observed (i.e., the slope of the temperature–health event relationship is always positive above the MRT)." For daily maximum temperature, they e.g. found an IRT of 27°C for both heat-related hospitalizations and hospitalizations due to "consequences of heat and dehydration" and an IRT of 29°C for heat-related emergency department visits. Furthermore, they estimated a minimum risk temperature of heat-related mortality of 26°C and MRT for mortality due to consequences of heat and dehydration at 25°C. For heat-related hospitalizations, hospitalizations due to consequences of heat and dehydration, and heat-related emergency department visits the minimum risk temperature is estimated at 22°C.

Even though there are, of course, several methodological differences to our study, including the focus on a relatively small geographical region compared to our study, we think that these results highlight the relevance of relatively low temperatures for human-health impacts and that the respective temperature is strongly dependent on the analyzed impact type.

5. Matzarakis et. al. (2020) [9] published a paper (Atmosphere (MDPI)) on the heat health warning system (HHWS) in Germany. In HHWS, the heat warning thresholds are calculated using "perceived temperature" (PT) in °C, a thermal index allowing "for an assessment of the atmospheric environment in terms of human thermal stress." In addition, this "thermo-physiologically based procedure contains variable thresholds taking into account the short time acclimatization of the people." This way, the HHWS accounts for physiological changes that "occur if humans are consecutively exposed to heat (. . .)." To account for this "short-term heat acclimatization in the German HHWS, the thermal conditions of the last 30 days are used for adjusting (. . .) the thresholds of the heat stress categories accordingly (. . .). Hence, these are variable thresholds[,]" with a fixed upper limit of 34°C PT. In other words, the warning level cannot rise beyond this particular threshold. "In additon, climatological reasons account for regional differences in the thresholds of the thermo-physiciological stress categories. This is reflected by generally lower values in northern Germany compared to the southern parts of the country (. . .)." Furthermore, the HHWS also takes into account the different heat stress on the elderly population in Germany. Hence, the heat warning threshold for e.g. "extreme heat stress" is lower for seniors than for the rest of the population. Even though there are significant methodological differences to our calculations, as PT takes into account several meteorological variables, not only air temperatures - hence, PT is, of course, a more complex metric than the simple air temperature in our results - this paper shows that more variable as well as local-climatology-based and age-dependent temperature thresholds can be relevant, even for heat warning systems.

6. To switch to a not directly human-health-related impact: Vogel et. al. (2019) [10] published a study on "The effects of climate extremes on global agricultural yields" in Environmental Research Letters in which they show that "growing season climate factors - including mean climate as well as climate extremes - explain 20%-49% of the variance of yield anomalies (the range describes the differences between crop types)", where "[e]xtreme event indicators explain 18%-43% of the variance of yield anomalies, contributing more than half of the explained variance in maize, soybeans and rice[.]" Among those extreme event indicators, they use TX90p, i.e. the "[p]ercentage of days during the growing season with daily maximum temperature above the 90th percentile[.]" They also show that "temperature-related predictors are more strongly correlated with crop yield anomalies than precipitation-related predictors across all four crops." Furthermore, their analysis show that TX90p is among the "temperature predictors with the highest predictive capacity." In addition, they "identify hot spot regions that are critical for food production and at the

same time particularly susceptible to fluctuations in climate conditions, including climate extremes (. . .).” Among those hot spot regions showing ”strong impacts of climate extremes” are e.g. ”North America for maize, spring wheat and soybeans production, Asia for rice and maize production and Europe for production of spring wheat (. . .).”

Due to several methodological differences between their study and our analysis, our results are, of course, not directly comparable to their results. However, we think that this study highlights the agricultural impact-relevance of the 90th percentile as a temperature threshold and, thereby, underlines the relevance of the methodological choices in and the results of our study.

We hope you can see that we did extensive research on the relevance (and potential impact-relevance) of our results. We do, of course, see the merit in your proposed threshold of 35°C, especially in the context of your professional experience. However, we think that the presented literature emphasizes the relevance of our results even for temperatures below the aforementioned temperature threshold, which, according to the literature listed above, are capable of producing noticable impacts on e.g. human health and agriculture, including temperatures as low as the lowest 90th percentile threshold in your calculations based on the historical observations in Beijing, i.e. 22°C (see minimum risk temperature in Petitii et. al. [8]) To further address your comment, we added a new paragraph in the discussion (lines 690–706).

Improving strength of study by including additional (higher) temperature thresholds in our study:

Having discussed all of those points and studies above regarding the impact-relevance of relatively low temperatures and the application of Tx90p, we would like to re-iterate that we do, of course, see the merit in, inter alia, using higher temperature thresholds for a heatwave analysis that is aimed at contextualizing projected heatwaves to recent extreme reference heatwaves. Thus, short of a complete re-design of our study based on a specific set of absolute temperature thresholds, we invested substantial effort in the improvement of the quality of our study by including three higher percentile-based temperature thresholds (Tx95p, Tx97.5p, and Tx99p). As discussed in the updated paper, these higher percentiles are applied to all heatwave characteristics in the same way as the 90th percentile. We think that presenting the results based on these higher percentiles alongside Tx90p significantly strengthens our study and increases the relevance of our results. Due to limitations in word count and readability considerations, we decided that it is best to choose selected higher-percentile-based results to present in the main text alongside Tx90p and present the remaining results in the supplementary material. In general, the trends discussed for Tx90p-based results are very similar to the trends for the higher-percentile-based analysis (e.g. lines 123–124, 320–322, 577–578).

Concern 2: Mismatch between statistical extremes and impactful events In summary, the manuscript applies a statistical extreme-event framework (calendar-day 90th percentile relative to 1961–1990) to characterize events

that are, by construction, major weather hazards with documented socio-economic impacts. This creates a clear conceptual mismatch:

- **The statistical framework assumes frequent "extreme" heatwave days in the historical baseline; in practice, this algorithm identifies a large number of statistically defined "heatwave" events that cannot reasonably be considered hazards, for example cases where daily maximum temperature may not even reach 30 °C, but is flagged solely because it occurs at an unusual time of year.**
- **The events being analyzed and contextualized are rare, high-impact hazards that caused widespread socio-economic impacts.**

From the Chinese perspective, truly impactful heatwave hazards during 1961–1990 were relatively rare. The assumption of ∼18 heatwave days per year therefore conflicts with both operational practice and lived experience. I therefore strongly encourage the authors to reconsider the heatwave threshold definition, particularly if the objective is to assess and contextualize hazard-level heatwave events rather than purely statistical extremes.

Regarding the first point in your comment please see the discussion above of impact-relevance of lower absolute temperature/relative "statistical" thresholds for e.g. human health and agriculture. Hence, we would argue that the mentioned mismatch only occurs when focusing on a specific subset of impacts. However, as we do not attempt to extrapolate recorded impacts into the future, we think this is not a major concern for our study. Nevertheless, we added paragraphs discussing these points in lines 690–720.
In addition, as shown, for instance, by Schwarz et. al. (2020) [6], Guirguis et. al. (2018) [7], and Petitti et. al. (2016) [8], extreme yet relatively lower absolute temperatures do show effects on hospitalization rates and hospital treatments. Considering, from a socio-economic viewpoint, that hospitalization and hospital treatments are a scarce and expensive resource, we would argue that this can reasonably be classified as "meaningful" socio-economic impacts. And more importantly, going beyond a simple socio-economic viewpoint, heat-related-effects as described in the aforementioned studies do have significant impacts on the livelihoods of people and their quality of life. The same arguments can be made about losses in agricultural yields, see Vogel et. al. (2019) [10].
Regarding the second point, as mentioned above, we think that the classification as weather hazards and/or meteorological disasters does not preclude a statistical extreme event analysis, as the selected heatwaves also fall into the category of extreme events. Hence, the observed reference events fall into the intersection of extreme events and (at least) weather hazard.

Concern 3: Choice of reference dataset for spatial extent

Regarding observational datasets, the manuscript relies primarily on Berkeley Earth (1° resolution), with ERA5 used as a secondary reference.

While Berkeley Earth has advantages in handling sparse and discontinuous observations, I am more inclined to trust ERA5 for defining the spatial extent and geographical boundaries of heatwave events, due to its substantially higher spatial resolution. At a 1° grid resolution, it is difficult to accurately capture true heatwave boundaries, especially in regions with strong topographic gradients. For example, among the 22 global events, two involve China. Event "g)" in Table S1 spans approximately 89.5°E–122.5°E and 20.5°N–40.5°N, covering both eastern lowland China and the Tibetan Plateau. Treating high-elevation regions (>3000 m) and eastern plains as a single homogeneous heatwave-affected domain is problematic. In practice, heatwaves capable of causing major socio-economic impacts are extremely unlikely over the Tibetan Plateau, even if statistically extreme temperatures occur there.
I therefore recommend using ERA5 as the primary dataset for defining spatial extent, with Berkeley Earth retained as a complementary reference.

Acknowledging that observational uncertainty can be an issue in some regions, as discussed in more detail in the methods section and to compensate for different limitations of certain data products (e.g. grid resolution), we did not only focus on a single reference data set but analyzed and present results relative to two independent data sets, representing different types of data products, i.e. Berkeley Earth land-surface observational and ERA5 reanalysis data. The distinction between primary and secondary reference is, arguably, a bit artificial. Even though we (chronologically) started out with Berkeley Earth as a reference data set, for the purpose of region selection, we double-checked the reasonableness of the selected regional boundaries using ERA5. Considering the similarities in the spatio-temporal evolution of the selected reference heatwaves and the sub-regions affected by them (see Figs. 10–11 in response letter), using ERA5 (chronologically) as the starting point would not have significantly influenced our selection of the regional boundaries. For further clarification, we now also provide figures showing the spatio-temporal evolution of the reference events on a geographical map in the supplementary material (Figs. S449 – S498) and the 2022 reference heatwave in China in the figures below (Figs. 10–11 in response letter). These sets of figures show that the locations affected by local extreme heat (Tx90p) approximately cover the same geographical boundaries. Hence, (chronologically) starting with ERA5 as the reference would not have significantly influenced the selection of the regional boundaries. When comparing the subplots, please note that the ERA5-based heatwave starts 3 days and ends 1 day before the event in the Berkeley Earth dataset. Furthermore, we also used both Berkeley Earth and ERA5 for our model evaluation (see description in methods section). Except for the return time figures, we also present both reference values in figures in the main text. For the return time figures (Fig. 5 and 6 in the manuscript), we focus on one of the two data sets (i.e. Berkeley Earth) to ensure readability. However, as mentioned in the updated return time section (lines 540–541), the results discussed in the main text of this sub-section are relative to both Berkeley Earth and ERA5. The only difference is that for the purpose of readability we decided to present exceedance percentages relative to one of the two reference data

sets in the main paper and present the exceedance percentage relative to the other in the supplementary material. Acknowledging the potential advantages of ERA5, e.g. higher resolution, for the purpose of this study we think that Berkeley Earth is better suited for the results in the main paper as it interpolates weather station thermometer data to any location on Earth, while at the same time maximizing the usage of short and discontinuous temperature records. However, as we do, of course, see the merit of your points we invested substantial effort to address your comments by

1. providing the aforementioned figures showing the spatio-temporal evolution of the reference heatwaves in both Berkeley Earth and ERA5,
2. removing the artificial distinction between primary and secondary reference data,
3. discussing both Berkeley-Earth- and ERA5-based results for the return time calculations,
4. including the ERA5-based reference values in Table 2 of the manuscript,
5. adding a short paragraph regarding the use of two reference data sets in the discussion (lines 684–688), and
6. discussing ERA5-based exceedance percentages where the word limit allowed for the additional information. Nevertheless, we would also like to re-iterate that for the sake of readability and to meet the word limit, we had to focus on one of the data sets for exceedance percentages in the main text.

For our discussion on socio-economic impact-relevance of extreme yet relatively lower absolute temperatures see above.

Concern 4: Event splitting and alternative identification criteria
The authors note that, depending on the dataset, some events may be split when regional-mean temperature briefly falls below the threshold. While this behavior is expected under a strict spatial-mean definition, it may reduce robustness. As an alternative (offered here as a reference rather than a prescription), China's national weather service identifies regional heatwave events using area-based criteria, for example:

1. **At least 20% of the region must experience heatwave conditions on the event initiation day;**
2. **Persistence for at least two consecutive days;**
3. **During at least one day of the event, ≥50% of the region must be affected.**

Under this framework, spatial continuity is not required, and short-lived dips in regional-mean temperature do not necessarily split a physically coherent heatwave event. Such criteria may yield more robust event identification, particularly when comparing across datasets.

First of all, we would like to thank you very much for this suggestion. This approach seems very interesting. Hence, we would very much like to apply it in a future heatwave analysis, and would be very happy to receive a citable reference for future use.

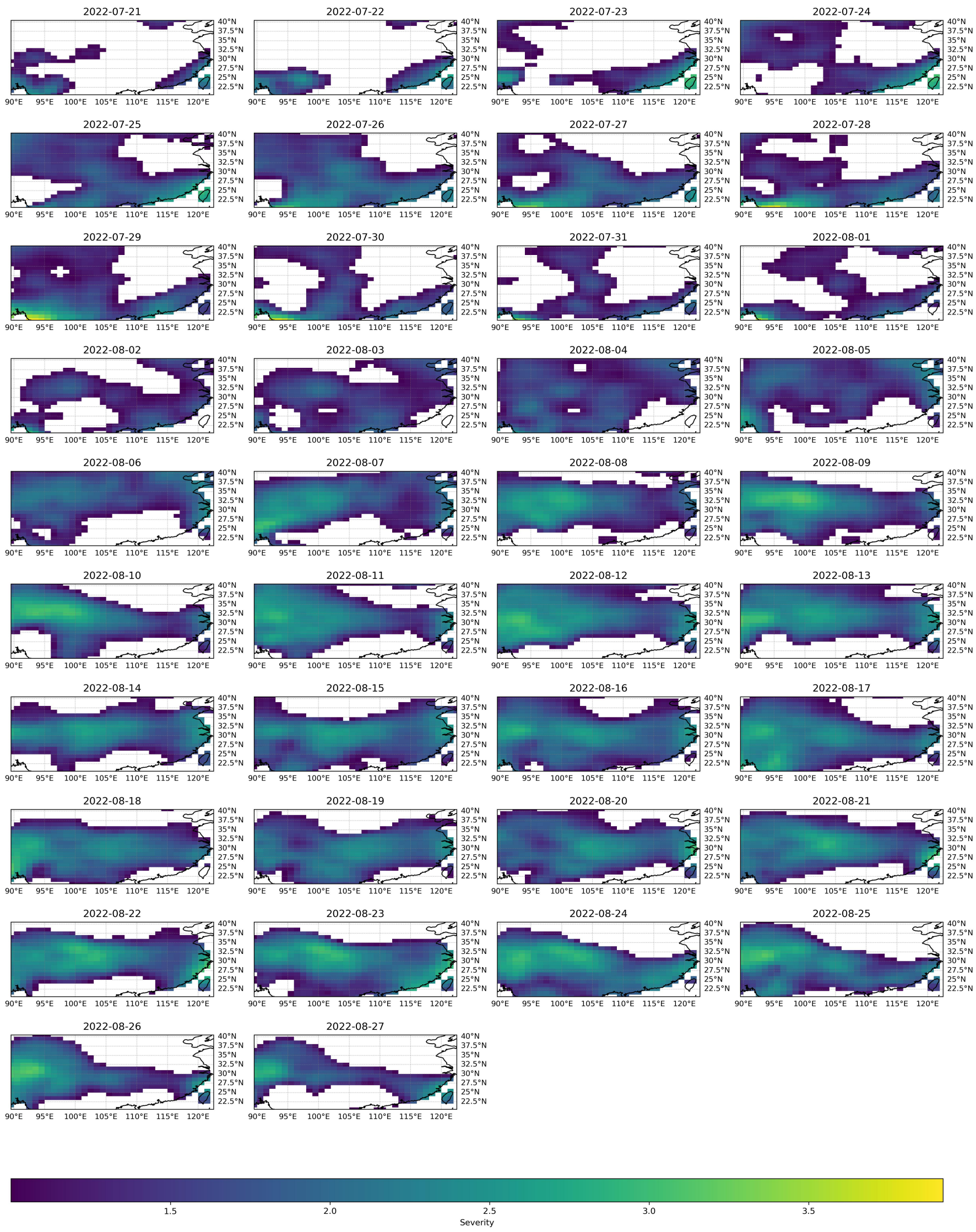

Fig. 10 Reference Heatwave Mapping – Reference heatwave in China 2022 based on Berkeley Earth data.

Apart from that, we would also like to note that, as already indicated in your comment, "event-splitting" is expected under a strict application of any heatwave definition. In other words, there is no guarantee that the (strict) application of a specific heatwave definition will not lead to potential "event-splitting". However, a non-strict application would either result in (1) a quasi-arbitrary definition of heatwaves or (2) effectively the introduction of a second (or multiple) additional heatwave threshold(s), in other words lowering the heatwave threshold temperature.

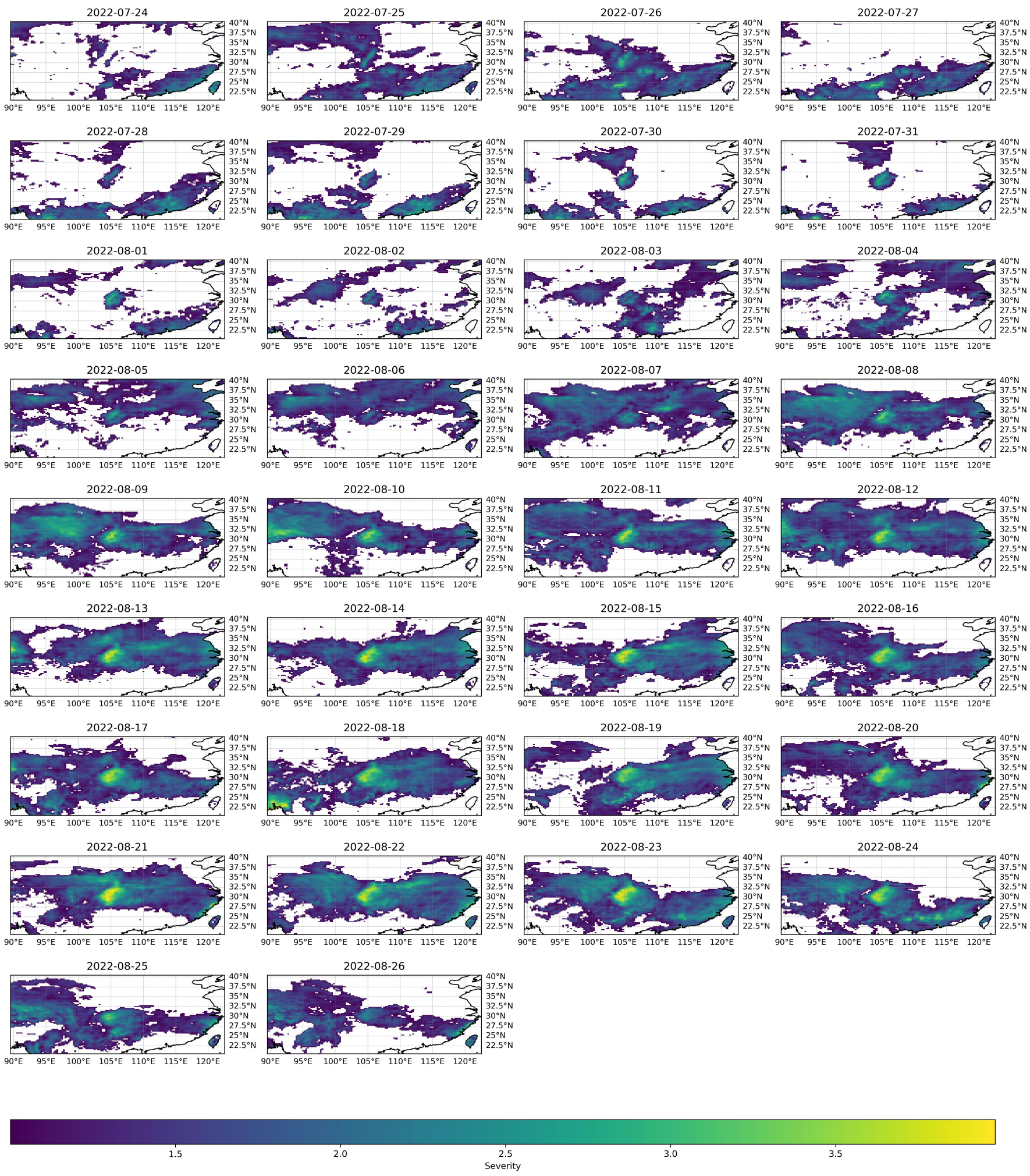

Fig. 11 Reference Heatwave Mapping – Reference heatwave in China 2022 based on ERA5 data.

(3) it would lead to complications concerning the calculations of cumulative heat as considering days with temperatures (slightly) below the temperature threshold would actually lead to adding negative temperature anomalies to the cumulative heat value. In other words, we would subtract the temperature anomaly instead of adding it, which contradicts the definition of cumulative heat [11].
Nevertheless, we would argue a strict application of the heatwave threshold can indeed increase the robustness of the results, as e.g. it highlights that heatwaves are projected to cover very large time windows without a single day of relief.

Final remarks

Despite these concerns, I would like to reiterate that the conceptual contribution of this study is highly valuable. By characterizing unprecedented

recent heatwave events in terms of heatwave severity, cumulative heat, duration, and spatial extent, and integrating these dimensions into an overall measure of integrated intensity, the authors move well beyond traditional attribution approaches that rely solely on the single most extreme temperature within an event.

This multidimensional framework has strong potential to advance how the community understands major meteorological hazards under climate change. The extensive supplementary material further reflects the authors' rigorous and systematic approach, which deserves strong recognition.

I hope the authors find these comments constructive and useful. I would be happy to discuss any of these points further if clarification is helpful, and I wish the authors every success in revising this impressive study.

We would like to thank you very much for considering our conceptual contribution as highly valuable and for appreciating our attempt to move beyond traditional approaches as well as our rigorous and systematic approach.

In addition, we would also like to thank you very much for your thorough review and the food for thought that you have provided us with. Short of a complete re-design/re-conceptualization of the study, we attempted to take up as many of your valuable suggestions as possible within our framework, which, we think, significantly improved and strengthened our study. As mentioned in the updated discussion of the paper (lines 708–720), even though we do not quantify and extrapolate specific impacts/impact types into the future, which might limit the direct applicability of our results for questions about specific impacts, we think that our study still provides valuable impact-relevant information and a useful framework for future impact-specific studies attempting to extrapolate specific recorded impacts into the future.

Remarks on code availability

The authors provide extensive supplementary figures and tables (over 180 figures and more than 30 tables) that document the statistical analyses in detail and help readers follow the analysis workflow. No source code is provided, and reproducibility therefore relies on the description in the manuscript and supplementary materials.

Thank you very much for appreciating our extensive supplementary library. We were indeed hoping that readers may find it valuable in understanding the analysis and following the analysis workflow. Regarding code availability, there might be a misunderstanding. We did indeed provide source code, inter alia, for the very calculations you have attempted to reproduce, in an online repository on Sync.com (link and corresponding password was provided with the MTS submission form). In the manuscript, we do mention that the code will be made available upon reasonable request. This was done as we were thinking about taking up the offer from Research Square for "In-Review" and wanted to make sure that, for now, only Peer Reviewers have access to the source code.

We did have some technical issues, though, in the beginning such that we had to

upload the scripts again a few days after the submission of the manuscript as we realized that the initial upload was not successful. Considering that we were able to fix the issue relatively fast, the source code should have been available during peer review via the link provided. However, just in case you happened to use the link and tried to access the source code in that very time window in the beginning we would like to apologize very much for the unfortunate inconvenience. We have now provided the central calculation scripts as workable demo versions for a subset of the data. The link to the scripts has now been provided in the same document as the link to the supplementary material. Finally, we would like to highlight again that we very much appreciate the effort you put into attempting to reproduce our results based on the description in the method section.

References

[1] Perkins, S. E. & Alexander, L. V. On the measurement of heat waves. *Journal of Climate* **26**, 4500–4517 (2013). URL http://dx.doi.org/10.1175/JCLI-D-12-00383.1.

[2] White, R. H. *et al.* The unprecedented pacific northwest heatwave of june 2021. *Nature communications* **14**, 727 (2023).

[3] Malinina, E. & Gillett, N. P. The 2021 heatwave was less rare in western canada than previously thought. *Weather and Climate Extremes* **43**, 100642 (2024). URL https://www.sciencedirect.com/science/article/pii/S2212094724000033.

[4] Hobday, A. *et al.* Categorizing and naming marine heatwaves. *Oceanography* **31** (2018). URL http://dx.doi.org/10.5670/oceanog.2018.205.

[5] Vanos, J., Guzman-Echavarria, G., Baldwin, J. W. *et al.* A physiological approach for assessing human survivability and liveability to heat in a changing climate. *Nature Communications* **14**, 7653 (2023). URL https://doi.org/10.1038/s41467-023-43121-5.

[6] Schwarz, L. *et al.* The health burden of fall, winter and spring extreme heat events in southern california and contribution of santa ana winds. *Environmental Research Letters* **15**, 054017 (2020). URL https://doi.org/10.1088/1748-9326/ab7f0e.

[7] Guirguis, K. *et al.* Heat, disparities, and health outcomes in san diego county's diverse climate zones. *GeoHealth* **2**, 212–223 (2018). URL https://agupubs.onlinelibrary.wiley.com/doi/abs/10.1029/2017GH000127.

[8] Petitti, D. B., Hondula, D. M., Yang, S., Harlan, S. L. & Chowell, G. Multiple trigger points for quantifying heat-health impacts: new evidence from a hot climate. *Environmental health perspectives* **124**, 176 (2015).

[9] Matzarakis, A., Laschewski, G. & Muthers, S. The heat health warning system in germany—application and warnings for 2005 to 2019. *Atmosphere* **11** (2020). URL https://www.mdpi.com/2073-4433/11/2/170.

[10] Vogel, E. *et al.* The effects of climate extremes on global agricultural yields. *Environmental Research Letters* **14**, 054010 (2019). URL https://doi.org/10.1088/1748-9326/ab154b.

[11] Hirsch, A. L., Ridder, N. N., Perkins-Kirkpatrick, S. E. & Ukkola, A. Cmip6 multimodel evaluation of present-day heatwave attributes. *Geophysical Research Letters* **48** (2021). URL http://dx.doi.org/10.1029/2021GL095161.

Peer Review - Response to Reviewers

General Remarks

We would like to thank all three reviewers for their time and constructive comments and for appreciating our effort in revising the original manuscript. In addition, we would also like to thank Reviewers 2 and 3 very much for supporting the publication of our study in Nature Communications. For the remaining comments by Reviewer 1, we have responded to each comment below, with the reviewer comments in **bold** and our response in blue, and the appropriate references and figures where applicable.

Reviewer 1

Thank you for the detailed and well-reasoned responses. Regarding the return period, the rationale for retaining the empirical approach is persuasive, particularly the observation that multiple heatwave events occurring within the same year may violate the block maxima assumption required for GEV analysis. Nevertheless, the authors may wish to consider, at least as a sensitivity analysis, the application of a Generalized Pareto Distribution (GPD) within a Peaks-Over-Threshold (POT) framework. In contrast to GEV, the GPD is specifically formulated for threshold exceedances and is therefore more closely aligned with the event-selection methodology adopted in the manuscript. Importantly, this approach would retain the full set of exceedance events, thereby avoiding the reduction in sample size associated with annual maxima methods, while also enabling parametric estimation of return periods beyond the observed record. Even a limited comparison between empirical and GPD-based return period estimates for a subset of regions would strengthen the robustness of the analysis and help address potential concerns regarding the stability and precision of tail estimates.

We would like to thank you for this insightful comment and for raising this important methodological question. We agree that the application of a Generalized Pareto Distribution (GPD) within a Peaks-Over-Threshold (POT) framework can be a useful alternative to only selecting block-maxima for the analysis by fitting a GEV. Where applicable, GPD can alleviate some of the issues that arise from fitting a GEV (e.g. substantially reducing the sample size), while still remaining within the

same theoretical framework, i.e. Extreme Value Theory (EVT). In the context of heatwave research and extreme temperatures, the GPD is, generally, well-suited to modeling the distribution of daily maximum temperature [1, 2] during heat events, or the maximum intensity of individual heatwaves. Due to these reasons, we did indeed consider the use of GPD within a POT framework. However, we do not believe that this approach is theoretically appropriate in the specific case of our study, with issues arising that substantially limit the applicability of a GPD for this purpose:

1. The Peaks-Over-Threshold (POT) framework is theoretically motivated by the Pickands-Balkema-de Haan theorem. This theorem states that for a sufficiently high threshold u, $X - u | X > u$ is approximately GPD distributed. This theorem is formulated in terms of a single random variable given that it exceeds the threshold u. In other words, for a sufficiently high threshold, the distribution of individual threshold exceedances is well approximated by a GPD [2, 3]. This leads to one of the main issues for applying GPD/POT in the context of our study: In contrast to individual threshold exceedances, the heatwave characteristic "cumulative heat", as one of the central heatwave characteristics analyzed, is essentially a random sum of exceedances throughout the course of a heatwave event. As such, the distribution of cumulative heat is determined jointly by (1) the tail behavior of the individual daily temperature exceedances, (2) the distribution of event duration and (3) the dependence among exceedances within an individual heatwave. As a consequence, random sums display distinct asymptotic behavior and require separate theoretical treatment [4]. To the best of our knowledge, there is no theoretical basis for assuming that a sum such as cumulative heat inherits the generalized Pareto distribution of its daily threshold exceedances. Hence, applying GPD/POT to cumulative heat in our study for the purpose of estimating the return times of the cumulative heat levels of our reference heatwaves risks fundamental misspecification.

2. The theoretical foundation of GPD/POT leads to other potential issues in the context of our study: The Pickands-Balkema-de Haan theorem states that for a sufficiently high threshold, the distribution of individual threshold exceedances is well approximated by a GPD. In our study, however, two of the six heatwave characteristics for which we estimate the return time are median values, i.e. median severity and median heatwave area. These are summary statistics computed from multiple daily exceedances within each event. While this summary statistic might itself exhibit a right-skewed distribution, there is, to the best of our knowledge, no theoretical basis for assuming that the event median inherits the generalized Pareto distribution of its daily threshold exceedances.

Due to these limitations discussed above, the applicability of GPD/POT would be limited to only half of the heatwave metrics we analyze.
To further address your comment, we performed a sensitivity test on one of the heatwave characteristics to which GDP/POT is theoretically applicable, i.e. maximum severity. This sensitivity test compares empirical and GPD-based return time

estimates. In detail, we

1. calculated GPD-based return time estimates during both the present-to-near-future and end-of-century 40-year periods from 2015–2054 and 2060–2099, respectively (see Figs. 5 and 6 in manuscript), for all nine regions discussed in the manuscript for each model in CMIP6-MME.

2. we computed the differences between the empirical and the GPD-based return time estimates for the sample presented in Figs. 5 and 6 of the manuscript and calculated the median difference in return time between the empirical and GPD-based estimates as well as the 5th and 95th percentile of the respective sample (Figs. 1 and 2 of response letter).

The results show that the median differences in return time estimates of both methodological approaches are small and in most cases negligible and do not affect the overall conclusions of the study. They also show that, apart from a small number of outliers, the error bars are of limited length and the values of the 95th percentile in most samples are in the low single-digits. We acknowledge that a few exceptional cases may warrant further investigation. However, identifying and systematically evaluating the factors responsible for these differences would require additional analyses beyond the objectives and scope of the present work and could constitute a separate study in its own right.
We agree that considering the use of a Generalized Pareto Distribution within in a Peaks-Over-Threshold framework, where applicable, can provide valuable insights. However, as discussed above, there are substantial issues with the application of GPD/POT in the case of large parts of our study. Addressing these issues with scientific rigor would require substantial new analyses and validation that extend beyond the objectives of the current study and would constitute a substantial expansion of the current work or even an entirely new study rather than a revision of the existing manuscript. In addition, we think that the performed sensitivity test indicates the overall robustness of our empirical return time estimates.
As already mentioned in the manuscript (lines 845–847), we acknowledge the shortcomings of an empirical estimation of the return periods, especially the limitation of the maximum return time by the length of the selected time window. However, considering (1) the substantial theoretical issues discussed above, which risk fundamental misspecification, (2) the very limited and, in most cases, negligible differences between the return period results computed in our sensitivity test and (3) to ensure direct comparability of the return times among all six heatwave characteristics due to methodological consistency across all analyzed characteristics, we think that the chosen empirical approach is better suited in the case of our study. We included a brief discussion of the issues related to the applicability of GPD in our study in the manuscript (lines 869–876).

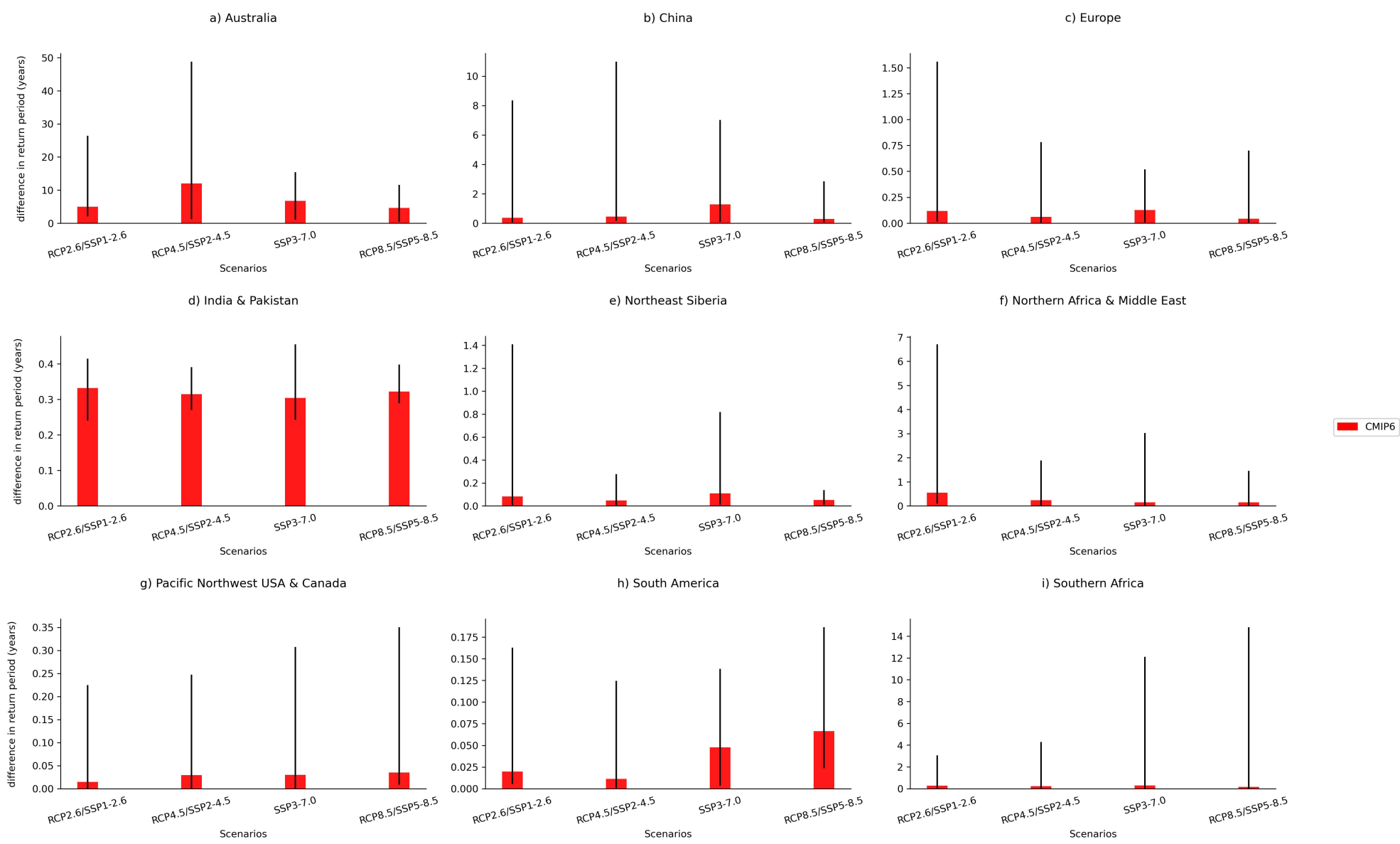

Fig. 1 Differences in return time (in years) between 2015 and 2054 for maximum heatwave severity of the respective observed event. For each region (a-i), values are shown for the different scenarios (increasing in emissions from left to right for CMIP6 ensembles). The error bars indicate the 5th and 95th percentile of the respective sample.

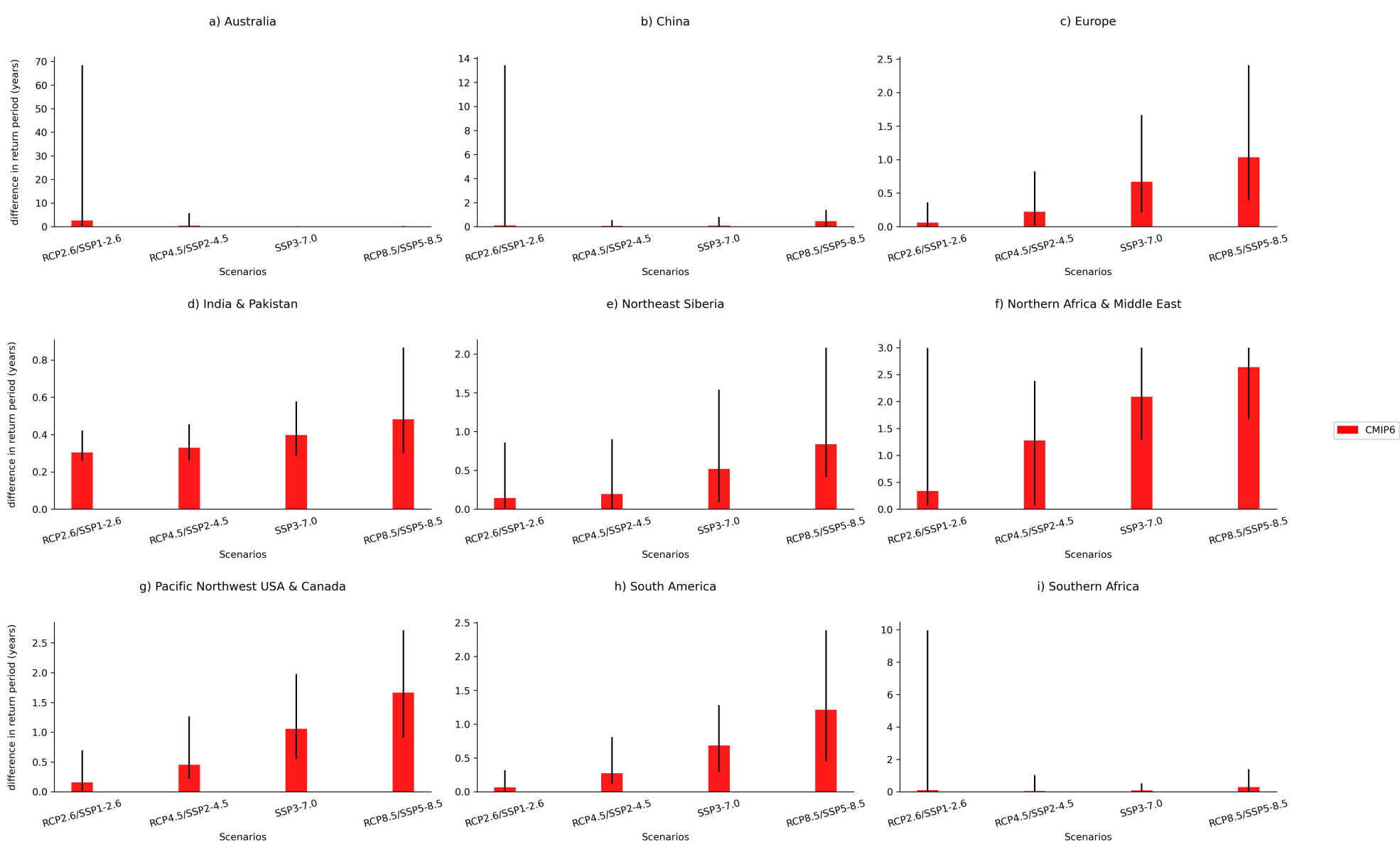

Fig. 2 As Fig. 1, but for 2060–2099 rather than 2015–2054

Line 115: To begin the results section, provide a brief introduction to what will be analyzed.

Thank you very much for this suggestion. We previously included a short description of our analysis in the introduction. However, we agree that it does improve the readability of the manuscript when describing our analysis in the beginning of the results section. Hence, we have moved the aforementioned description to the beginning of the results section and added some more details (lines 95–106).

Lines 382-387: improve sentence structure.

Thank you very much for this suggestion. We have improved the indicated subparagraph by restructuring and shortening the sentences (lines 292–297).

References

[1] Brown, S. J., Caesar, J. & Ferro, C. A. T. Global changes in extreme daily temperature since 1950. *Journal of Geophysical Research: Atmospheres* **113** (2008). URL https://agupubs.onlinelibrary.wiley.com/doi/abs/10.1029/2006JD008091.

[2] Zahid, M., Blender, R., Lucarini, V. & Bramati, M. C. Return levels of temperature extremes in southern pakistan. *Earth System Dynamics* **8**, 1263–1278 (2017). URL https://doi.org/10.5194/esd-8-1263-2017.

[3] Coles, S. *An Introduction to Statistical Modeling of Extreme Values* Springer Series in Statistics (Springer London, London, 2001).

[4] Denisov, D., Foss, S. & Korshunov, D. Asymptotics of randomly stopped sums in the presence of heavy tails. *Bernoulli* **16**, 971–994 (2010).

Reviewer Comments

I have read this manuscript very carefully and thoroughly. Given the scope of the study, the very large number of supplementary figures (>180) and tables (>30), it is evident that the authors have invested substantial effort and care into this work. Overall, I highly appreciate the ambition, originality, and general conceptual framework proposed in this paper.

General assessment and strengths

The overarching research logic proposed by the authors is novel, well motivated, and compelling. By placing several major, recently observed global heatwave events into a future-oriented statistical framework, the study provides an intuitive and powerful way to contextualize the integrated intensity of recent extreme heat events relative to what climate models project for the coming decades. This framing substantially improves interpretability and helps readers recognize that some of the most socially and economically impactful heatwaves observed in recent years may, from a future-climate perspective, no longer represent the upper tail of extremes. Instead, the results suggest that even more frequent and more severe events should be expected in the coming decades.

This conceptual approach is innovative and valuable, and I fully agree that such a framework can significantly advance our understanding of major heatwave hazards under climate change. Given that this overall logic is sound, my comments below focus primarily on technical and methodological aspects, particularly those related to heatwave definition and event identification.

Scope of expertise and perspective

Before detailing specific concerns, I would like to acknowledge the limitations of my own expertise. I am a researcher from China's national meteorological research system, and my professional experience over the past decade has focused primarily on observed weather hazards in China and their impacts on socio-economic systems. My understanding of heatwave events in other regions is largely informed by the scientific literature and media reports. Consequently, the concerns raised below are especially grounded in the Chinese observational and operational experience, though I believe they have broader relevance.

Conceptual distinction: extreme events vs. weather hazards vs. meteorological disasters

A key issue concerns the conceptual distinction between "extreme events", "weather hazards", and "meteorological disasters", which are often used interchangeably but are fundamentally different.

- Extreme events are defined statistically, referring to events with low occurrence probability in one or more relevant dimensions, such as severity, timing, spatial extent, or other characteristics.
- Weather (natural) hazards are events that produce direct or indirect adverse impacts on socio-economic systems, at least within one sector (for example, extreme heat restricting outdoor labor and reducing productivity, or increasing electricity demand and leading to grid overload and higher household energy expenditures).

- Meteorological disasters represent cases where severe weather causes direct socio-economic losses, such as loss of life, damage to housing, or destruction of public infrastructure, emphasizing direct losses.

From this perspective, the 22 regional heatwave events selected in this study clearly fall into the category of weather hazards, and many of them can reasonably be classified as disaster-level events, given their documented societal and economic impacts.

However, the heatwave definition adopted in the manuscript is purely statistical, based on the calendar-day 90th percentile of daily maximum temperature during the warmest six months over the 1961–1990 baseline. While this is a classical and well-established approach for identifying statistical extremes, it does not necessarily correspond to the threshold at which heat events begin to generate meaningful socio-economic impacts.

Concern 1: Heatwave threshold may be too low from a hazard perspective

From a Chinese operational and observational standpoint, the chosen threshold appears too low to represent impactful heatwave hazards.

Using the authors' definition implies that, on average, each grid point experiences approximately 18 heatwave days per year during the 1961–1990 period. This is difficult to reconcile with empirical experience. For comparison, within China's national meteorological hazard monitoring and warning system:

- The lowest-level heat warning is issued only when daily maximum temperature reaches 35 °C for at least three consecutive days.
- The highest-level heat warning requires daily maximum temperature to exceed 40 °C.

In practice, heatwave events that cause substantial socio-economic impacts in China almost always exceed 35 °C, and typically exceed 40 °C.

To illustrate this point quantitatively, I analyzed historical observations from Beijing and Shanghai, China's two largest metropolitan areas, using the authors' exact methodology (15-day moving window percentile estimation followed by 31-day smoothing) over the warmest six months (April–September) during 1961–1990:

- **Beijing**:
  - Calendar-day 90th-percentile thresholds range from approximately 22 °C to 35 °C (Fig. 1a).
  - Only 9 calendar days have 90th-percentile thresholds exceeding 35 °C.
  - The mean 90th-percentile threshold is approximately 31.2 °C.
  - Corresponding calendar-day 99th-percentile thresholds are shown for reference (Fig. 1b).
- **Shanghai**:
  - Calendar-day 90th-percentile thresholds exceed 35 °C on only 16 calendar days (Fig. 2a).
  - The mean 90th-percentile threshold is approximately 30.2 °C.
  - Corresponding calendar-day 99th-percentile thresholds are shown for reference (Fig. 2b).

Under this definition, many identified "heatwave days" would not produce noticeable impacts on socio-economic systems, leading to an over-identification of heatwave events.

This likely explains why the manuscript reports an unusually large number of heatwave events, including cases outside China where heatwave durations exceed 180 days, which I found conceptually puzzling and which motivated my closer examination of the methodology.

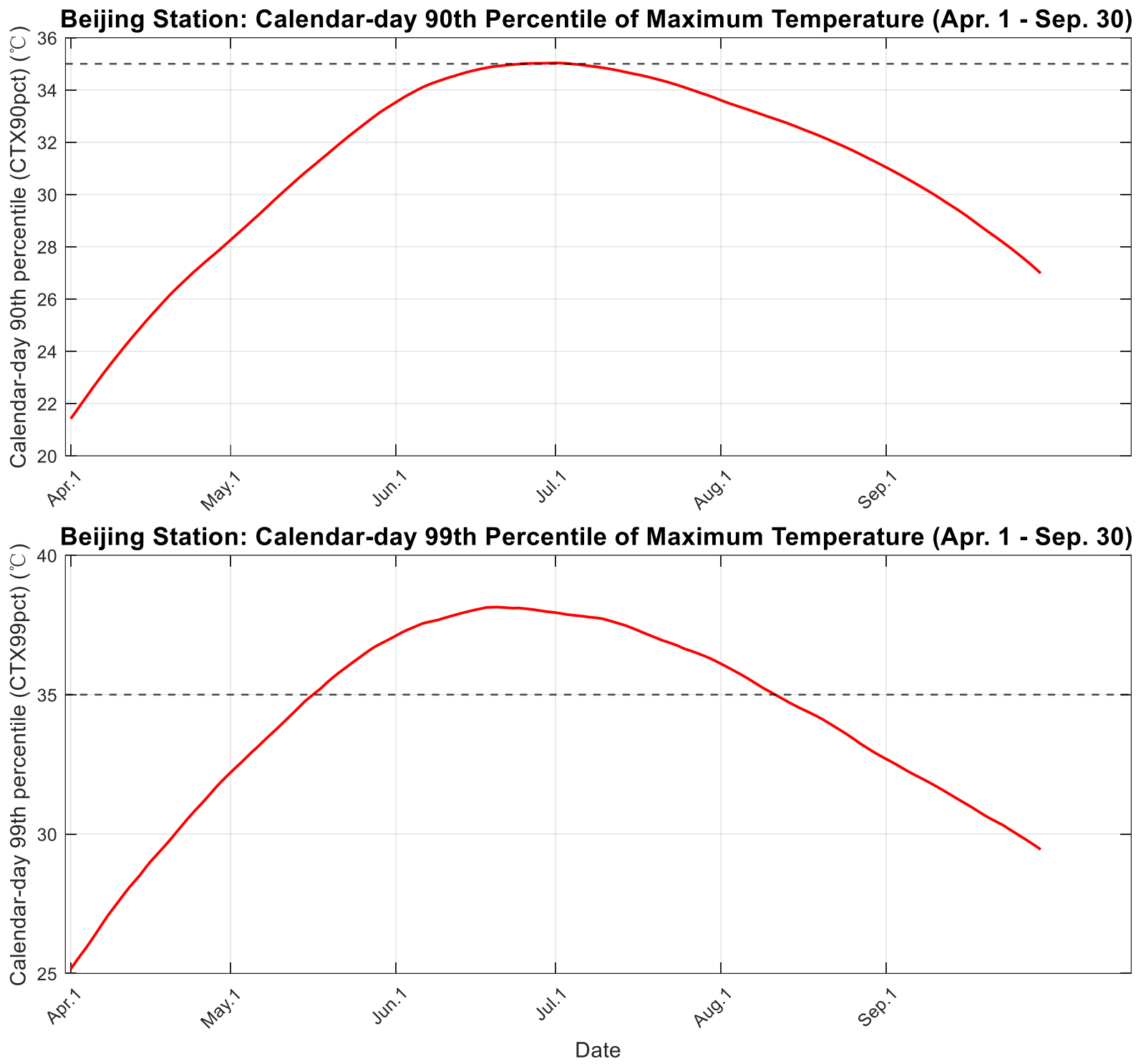

Fig. 1 | Calendar-day 90th & 99th percentile of daily maximum temperature during the warmest six months (1961–1990) at the Beijing meteorological station, China.

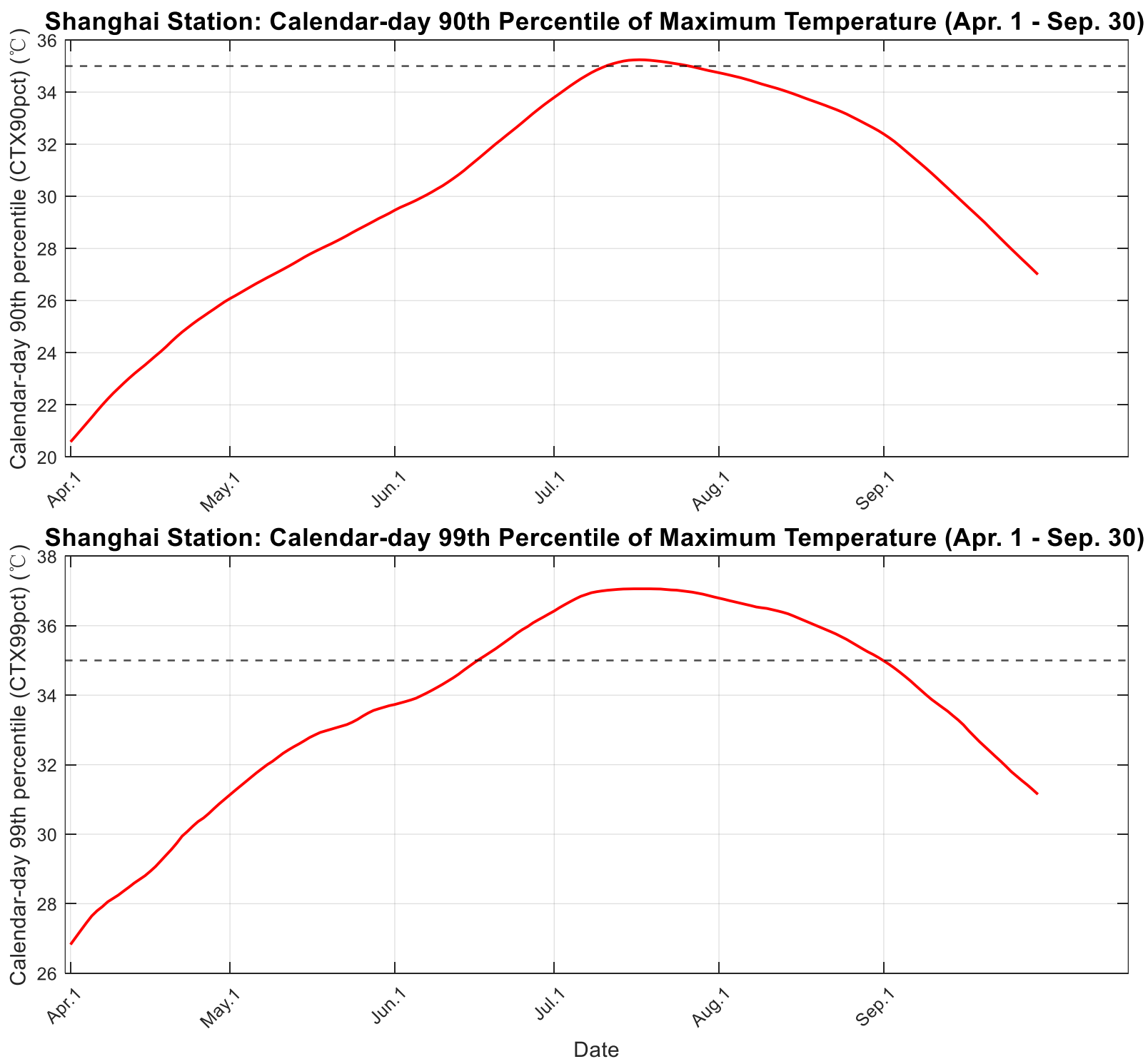

Fig. 2 | Calendar-day 90th & 99th percentile of daily maximum temperature during the warmest six months (1961–1990) at the Shanghai meteorological station, China

Concern 2: Mismatch between statistical extremes and impactful events
In summary, the manuscript applies a statistical extreme-event framework (calendar-day 90th percentile relative to 1961–1990) to characterize events that are, by construction, major weather hazards with documented socio-economic impacts. This creates a clear conceptual mismatch:

- The statistical framework assumes frequent "extreme" heatwave days in the historical baseline; in practice, this algorithm identifies a large number of statistically defined "heatwave" events that cannot reasonably be considered hazards, for example cases where daily maximum temperature may not even reach 30 °C, but is flagged solely because it occurs at an unusual time of year.
- The events being analyzed and contextualized are rare, high-impact hazards that caused widespread socio-economic impacts.

From the Chinese perspective, truly impactful heatwave hazards during 1961–1990 were relatively rare. The assumption of ~18 heatwave days per year therefore conflicts with both operational practice and lived experience.
I therefore strongly encourage the authors to reconsider the heatwave threshold definition, particularly if the objective is to assess and contextualize hazard-level heatwave events rather than purely statistical extremes.

Concern 3: Choice of reference dataset for spatial extent
Regarding observational datasets, the manuscript relies primarily on Berkeley Earth (1° resolution), with ERA5 used as a secondary reference. While Berkeley Earth has advantages in handling sparse and discontinuous observations, I am more inclined to trust ERA5 for defining the spatial extent and geographical boundaries of heatwave events, due to its substantially higher spatial resolution.
At a 1° grid resolution, it is difficult to accurately capture true heatwave boundaries, especially in regions with strong topographic gradients. For example, among the 22 global events, two involve China. Event "g)" in Table S1 spans approximately 89.5°E–122.5°E and 20.5°N–40.5°N, covering both eastern lowland China and the Tibetan Plateau. Treating high-elevation regions (>3000 m) and eastern plains as a single homogeneous heatwave-affected domain is problematic. In practice, heatwaves capable of causing major socio-economic impacts are extremely unlikely over the Tibetan Plateau, even if statistically extreme temperatures occur there.
I therefore recommend using ERA5 as the primary dataset for defining spatial extent, with Berkeley Earth retained as a complementary reference.

Concern 4: Event splitting and alternative identification criteria
The authors note that, depending on the dataset, some events may be split when regional-mean temperature briefly falls below the threshold. While this behavior is expected under a strict spatial-mean definition, it may reduce robustness.
As an alternative (offered here as a reference rather than a prescription), China's national weather service identifies regional heatwave events using area-based criteria, for example:

1. At least 20% of the region must experience heatwave conditions on the event initiation day;

2. Persistence for at least two consecutive days;
3. During at least one day of the event, ≥50% of the region must be affected.

Under this framework, spatial continuity is not required, and short-lived dips in regional-mean temperature do not necessarily split a physically coherent heatwave event. Such criteria may yield more robust event identification, particularly when comparing across datasets.

Final remarks

Despite these concerns, I would like to reiterate that the conceptual contribution of this study is highly valuable. By characterizing unprecedented recent heatwave events in terms of heatwave severity, cumulative heat, duration, and spatial extent, and integrating these dimensions into an overall measure of integrated intensity, the authors move well beyond traditional attribution approaches that rely solely on the single most extreme temperature within an event.

This multidimensional framework has strong potential to advance how the community understands major meteorological hazards under climate change. The extensive supplementary material further reflects the authors' rigorous and systematic approach, which deserves strong recognition.

I hope the authors find these comments constructive and useful. I would be happy to discuss any of these points further if clarification is helpful, and I wish the authors every success in revising this impressive study.
